# Association of a 7.9 kb Endogenous Retrovirus Insertion in Intron 1 of *CD36* with Obesity and Fat Measurements in Sheep

**DOI:** 10.1186/s13100-025-00349-w

**Published:** 2025-03-14

**Authors:** Ahmed A. Saleh, Ali Shoaib Moawad, Naisu Yang, Yao Zheng, Cai Chen, Xiaoyan Wang, Bo Gao, Chengyi Song

**Affiliations:** 1 College of Animal Science & Technology, Yangzhou University, Yangzhou, 225009 Jiangsu China; 2 Animal and Fish Production Department, Faculty of Agriculture (Al-Shatby), Alexandria University, Alexandria City, 11865 Egypt; 3 Department of Animal Production, Faculty of Agriculture, Kafrelsheikh University, Kafrelsheikh, 33516 Egypt

**Keywords:** Sheep Genome, Retrotransposon, Full-length ERV, *CD36* gene, BCS, Fat Composition

## Abstract

**Background:**

Endogenous retroviruses (ERVs) enhance genetic diversity in vertebrates, including sheep. This study investigates the role of Ov-ERV-R13-*CD36* within *CD36* gene and its association with phenotypic traits in sheep. Analyzing 58 sheep genomes revealed that ERVs constitute approximately 6.02% to 10.05% of the genomic content. We identified 31 retroviral insertion polymorphisms (RIPs) from 28 ERV groups. Among these, Ov-ERV-R13-*CD36*, which is specifically classified as a beta retrovirus, was selected for further analysis due to its location in *CD36* gene, known for its role in fat metabolism, obesity (OB), body weight (BW), and body condition score (BCS). We assessed the association of Ov-ERV-R13-*CD36* with OB and BCS across six sheep breeds, utilizing data from 1,355 individuals.

**Results:**

Genomic analyses confirmed that Ov-ERV-R13-*CD36* is located within *CD36* gene on Chromosome 4, with polymorphisms across various sheep genomes. In a subset of 43 genomes, 22 contained the Ov-ERV-R13-*CD36* insertion, while 21 exhibited wild-type variants. The studied animals showed variability in BCS and fat content associated with the Ov-ERV-R13-*CD36* variant. Notably, Rahmani sheep exhibited a significantly higher BCS (4.62), categorized as obese, while Barki sheep displayed the lowest BCS (2.73), classified as thin to average. The association analysis indicated that sheep with the RIP^−/−^ genotype correlated with higher OB and BCS, particularly in Rahmani and Romanov *x* Rahmani breeds.

**Conclusions:**

Findings suggest that Ov-ERV-R13-*CD36* within *CD36* gene correlates with beneficial economic traits associated with OB and BCS, particularly in Rahmani and Romanov *x* Rahmani breeds. This indicates that Ov-ERV-R13-*CD36* could be a valuable genetic marker for breeding programs aimed at enhancing traits like fat deposition and body condition in sheep.

**Supplementary Information:**

The online version contains supplementary material available at 10.1186/s13100-025-00349-w.

## Background

Endogenous retroviruses (ERVs) are a type of long terminal repeat (LTR) retrotransposons that are remnants of ancient retroviral infections in mammalian genomes, making up a significant proportion (8-10%) of these genomes [1–5]. Though often considered genomic parasites, retrotransposons, including ERVs, play crucial roles in genome organization, biological processes, species diversity and evolution [6].

Regions high in retrotransposon density, especially ERVs, are key to identifying genomic interactions and associations [7]. Due to their abundance and activity, they serve as molecular markers, aiding in genetic diversity, phylogenetic studies, and mapping [8]. Retrotransposon insertion polymorphism (RIP) markers have been applied in domestic animal studies like sheep, deer and chicken [9–15].

ERVs are remnants of ancient viral infections, embedded within mammalian genomes, that offer valuable insights into host-virus evolutionary dynamics. In sheep, various ERV families have been categorized, highlighting their genetic diversity and potential biological roles [16]. Notably, the study of endogenous-Jaagsiekte-sheep-retroviruses (enJSRVs) illustrates how genetic mutations can confer viral resistance, demonstrating the complex interplay between viral evolution and host defense mechanisms [17]. Beyond their biological significance, ERVs also play a critical role in tracing genomic evolution and economic traits across species. For instance, Chessa *et al*. [13] employed ERVs to trace the migration and distinguish between primitive and modern sheep breeds, shedding light on domestication processes. Similarly, Elleder *et al*. [14] identified CrERVγ in mule deer, revealing evolutionary links to economically significant livestock such as sheep and pigs.

Also, research has demonstrated the significant role of ERVs in the genetic evolution of different species, highlighting their regulatory functions and impacts on economically important traits [18–20]. Furthermore, studies have identified multiple ERV groups and families, suggesting that they continue to influence evolutionary processes and gene expression, thereby shaping traits of economic significance, particularly in mammals like ruminants and rodents [20, 21].

In a study on bovine genetics, research revealed that an LTR retrotransposon insertion in *APOB* gene affects cholesterol biosynthesis and lipid metabolism, impacting cattle health and productivity [4]. Another study focused on the ERVK [2-1-LTR] clade, identifying loci that influence ERV mobilization rates, which could alter genetic diversity and health traits in the herd [5]. Collectively, these studies underscore the critical role ERVs play in shaping genetic evolution and economic traits in various species.

ERVs can impact gene expression and genetic variation, potentially offering benefits within the host genome [22–24]. Despite beneficial aspects, ERV presence/absence (*Pres/Abs*) within candidate genes can alter economic traits, disease states and health indicators [2, 4, 5, 25–28].

For instance, Wang *et al*. [29] identified five RIPs within porcine *TLR* genes, including a 192 bp ERV insert in *TLR6*'s first intron, enhancing *TLR6* and *TLR1* expression. This ERV insertion acts as an enhancer, significantly boosting expression of *TLR*-related genes, indicating its role in influencing the immune response in pigs. In this aspect, variations in genes like *SUFU*, *SYCP2L* and *GLIPR1L1* have shown correlations with body measurements in goats, useful as molecular markers for body proportions [30]. Moreover, *CD36* gene SNPs were tied to fat weight and skin yellowness, offering insights for chicken breeding [31].

Numerous studies have established *CD36* gene as a candidate gene associated with traits such as obesity (OB), fat profile, fat deposition, body weight (BW), carcass characteristics, disease resistance and health indices in mammals [31–34]. *CD36* gene, also known as fatty acid translocase (FAT), scavenger receptor class B member 3 (SCARB3) and glycoprotein 88 (GP88), is a significant 88-kD class B scavenger receptor glycoprotein found on the surface of platelets. It is expressed in various mammalian cell types, including platelets, monocytes, macrophages, dendritic cells and subsets of T and B cells [35–38]. As a receptor, *CD36* binds multiple ligands, such as thrombospondin-1, collagen, oxidized phospholipids, oxidized low-density lipoprotein, long-chain fatty acids, erythrocytes parasitized by *Plasmodium falciparum* and apoptotic cells [39–41].

*CD36* is part of a family of integral membrane proteins located in many body tissues, playing a crucial role in fatty acyl translocation. It functions as a multi-ligand cell surface receptor for oxidized LDL lipoproteins (ox-LDL), long-chain fatty acids, aged neutrophils, and *Plasmodium falciparum*-parasitized erythrocytes. These interactions are implicated in various diseases, including insulin resistance, diabetes, atherosclerosis and malaria [42–51]. Furthermore, *CD36* is found on the outer mitochondrial membrane of skeletal muscle, where it facilitates long-chain fatty acid transport and regulates fatty acid oxidation within muscle mitochondria [52].

Our previous research on full-length ERVs in sheep indicated their polymorphism and potential to influence economic traits and disease resistance through interactions with candidate genes [19]. In this aspect, several studies confirmed that *CD36* gene is considered as a candidate gene associated with OB/body condition score (BCS), fat profile, fat deposition, carcass traits, disease resistance and health indices in mammals [31–34].

*CD36* gene has been extensively studied in humans. However, *CD36* and its implications for production, health and economic traits in livestock have long been neglected by researchers [31–34, 53].

This study mainly examined the link between the Ov-ERV-R13-*CD36* polymorphism and its effects on related phenotypes, including OB/BCS, meat composition and fat measurements, across different sheep breeds. The objectives were: **a)** to develop a protocol to mine full-length ERVs across 58 sheep genomes, aiming to identify RIP markers for future phylogenetic studies, genetic diversity and QTL mapping, **b)** to investigate correlations between ERVs located in genes linked to key economic traits in sheep, **c)** to explore the association between Ov-ERV-R13-*CD36* located in *CD36* gene, focusing on economic traits, **d)** to examine the differences in *CD36* gene in terms of its location, type, number of transcripts, gene length, predicted amino acid sequences and exon and intron details in sheep and other eight animal species, **e)** to study the *Pres/Abs* of Ov-ERV-R13-*CD36* within *CD36* between reference and 42 non-reference sheep genomes.

## Methods

### The assembled genomes and gene annotation files employed in the investigation

By utilizing a total of 57 assembled non-reference sheep genomes in addition to the reference genome (Additional File 1, Table S1), our study conducted a genome-wide analysis of ERV-RIPs. These genomes were sourced from the National Centre for Biotechnology Information (NCBI) whole-genome sequencing (WGS) database, accessed on 6 January 2024. The gene annotation file was acquired from the NCBI database (https://ftp.ncbi.nlm.nih.gov/genomes/all/annotation_releases; accessed on 12 January 2024). Specifically, we downloaded a Bed format file containing gene features, which included information on the coordinates of long non-coding RNA (lncRNA) genes, protein-coding genes and other gene annotations.

### Genome-wide ERV-RIP screening protocol in sheep

Screening for ERVs across the sheep genome on a genome-wide scale was outlined for mining. This protocol, utilizing 58 sheep genomes, delineated five primary procedural steps, according to Moawad *et al*. [19], Du *et al*. [54] and Chen *et al*. [55], with slight modifications, as follows;

#### Step 1: Identification of Full-length ERV Insertions

To initiate the process, a custom library containing full-length ERV sequences from our previous work [19], which comprises 28 groups/families (Table S2), was utilized to mask both the 57 non-reference genomes and the reference genome using RepeatMasker (-nolow, -lib custom library) [56]. Furthermore, these 28 groups/families, including the studied ERV-RIP (Ov-ERV-R13-*CD36*) were examined for similarity using the Repbase browser (https://www.girinst.org/repbase/update/browse.php).

Subsequently, ERVs encapsulating viral proteins such as Group-specific antigen (Gag), Polymerase (Pol) and Envelope protein (Env), flanked by identifiable LTRs and surpassing a length of 5 kb, were classified as full-length ERVs and retained for further scrutiny. The translation of these identified full-length ERVs was executed using GENSCAN (http://hollywood.mit.edu/GENSCAN.html; accessed on 26 January 2024). Following this, the translated sequences were explored against the Pfam database to analyze protein domains and functions [57]. The database utilized the hmmsearch command within the HMMER tool [58] for protein domain determination, enabling the identification of conserved protein families and aiding in the functional annotation of novel proteins across various species. The extraction of 500 bp flanking sequences, both upstream and downstream, surrounding these insertions was facilitated using the bedtools [59] commands flank and getfasta (Version 2.27.1), Additional File 2, Graph S1.

The evolutionary activity was assessed by estimating the insertion times of individual elements using the calcDivergenceFromAlign.pl tool within the RepeatMasker program [60–62]. This estimation utilized representative sequences for each element.

#### Step 2: Alignment with the Reference Genome

The flanking sequences bordering the identified full-length ERV insertions within the non-reference genomes were aligned with the reference genome using Blat [63] (-minIdentity = 90, -minScore = 450). Subsequently, the alignment outcomes were refined based on a length criterion ranging from 450 ~ 550 bp. Additionally, insertions with flanking sequences mapping to multiple genomic positions were omitted. In cases where insertions could not be aligned to the reference genome via the upstream 500 bp flanking sequence, the downstream 500 bp flanking sequences were similarly aligned, and the results from both sets were consolidated. This process facilitated the acquisition of each insertion's corresponding information within the reference genome from every non-reference genome.

#### Step 3: Detection of Full-length ERV Insertion Polymorphisms

The process of identifying putative full-length ERV insertion polymorphisms between the non-reference and reference genomes involved utilizing bedtools window (-w 50, -v) [59]. Differential insertions, which deviated from the reference genome, were categorized as potential full-length ERV insertion polymorphisms. More specifically, full-length ERV insertions from non-reference genomes that occupied a distinct window (comprising the ERV insertion site and a 400 bp flanking region) compared to the reference genome were regarded as putative full-length ERV insertion polymorphisms (Graph S1).

#### Step 4: Discovery of ERV Insertions through Data Mining and Validation of Polymorphisms via PCR

Initially, sequences containing the 500 bp flanking regions and the ERV sequences of potential full-length ERV insertions were extracted from all genomes using bedtools getfasta [59]. Subsequently, these sequences were consolidated, and any duplicates were eliminated using bedtools merge (-s, -d 10). Then, 28 ERV groups in sheep (*Ovis aries*) were discovered (Additional File 3, Data set 1). The conserved domains within these 28 ERV groups were investigated using the NCBI-Conserved-Domains online tool (https://www.ncbi.nlm.nih.gov/Structure/cdd/wrpsb.cgi; accessed on 17 February 2024). After analyzing the masked 58 genomes, 31 full-length ERV-RIP insertions were identified (Data set 2).

For further analysis, 14 ERVs associated with relevant candidate genes (Table S3 and Data set 3) were selected for validation through PCR amplification, using the primers listed in Table S4. Subsequently, five full-length or active ERVs were selected based on their structural characteristics (Graph S2) and polymorphism (Graph S3).

#### Step 5: Selecting one of the ERV-RIPs for comprehensive exploration

One specific ERV-RIP, designated as Ov-ERV-R13-*CD36*, was selected for detailed investigation due to its unique association with the CD36 gene and the valuable insights obtained from data mining (Table S3).

### Selection of Potential ERV-RIP Intersecting with Candidate Genes

During the comprehensive screening process for ERV-RIP markers, several ERV-RIPs were identified that intersect with candidate genes associated with economically significant traits in sheep. Among these, Ov-ERV-R13-*CD36* was selected for further investigation due to its location in *CD36* gene.

*CD36* is well-documented for its influence on traits such as BCS, BW and fat distribution within the body [31, 39]. As such, the intersection of Ov-ERV-R13-*CD36* with *CD36* gene presents a promising avenue for understanding the genetic mechanisms underlying these important traits. Identifying and analyzing this interaction could provide valuable insights into breeding strategies aimed at improving these economic traits in sheep [64].

### Investigation of *CD36* Gene in Sheep and Eight Other Species

*CD36* gene was analyzed across the reference genomes of nine species: sheep (*Ovis aries*), goats (*Capra hircus*), domestic cattle (*Bos taurus*), water buffalo (*Bubalus bubalis*), pigs (*Sus scrofa*), rabbits (*Oryctolagus cuniculus*), chicken (*Gallus gallus*), zebrafish (*Danio rerio*) and domestic ferrets (*Mustela putorius furo*). This part of the investigation aimed to determine the gene's location, type, number of transcripts, gene length, predicted amino acid sequences, as well as details on exons and introns. Reference genomes were utilized for each species (Table S5). Subsequently, further research focused on the genomic regions surrounding *CD36* gene, including neighbouring genes and exon organization, as detailed in Table S5.

### Analyzing Ov-ERV-R13-*CD36* Intersection Involving *CD36* Gene in the Sheep Reference Genome

The intersect program within bedtools version 2.27.1 [59], was employed to investigate the distribution bias of full-length ERV within sheep genomes and their overlaps with host genes. Through the bedtools intersect functionality, the examination of overlaps between two sets of genomic features was facilitated, offering precise control over the reporting of intersections. Host genes were sourced from the sheep annotation files retrieved from the NCBI database and then compared with the results obtained from RepeatMasker version 4.0.9 [56]. RepeatMasker was employed to annotate all ERVs in the sheep genomes using a custom library of previously identified full-length ERVs.

*CD36* gene information, including its exons and introns, was obtained from the NCBI database (https://www.ncbi.nlm.nih.gov/gene/?term=LOC101115115), Ensembl (https://www.ensembl.org/Sheep/Search/Results?q=LOC101115115;site=ensembl;facet_species=Sheep), and UCSC Genome Browser (https://genome.ucsc.edu). These data were organized for figure preparation using the "IBS" (v1.0.3) Illustrator of Biological-Sequences Software, available at https://www.nuget.org/packages/IBS.Data/1.0.3. Predictions of the structures for *CD36* gene in a reference genome (GCA_016772045.2) were made using Geneious Prime Software (Augustus Tool, Version 7_ 2024), available at https://www.geneious.com. While, the domains for Ov-ERV-R13-*CD36* and *CD36* gene were predicted using the NCBI Conserved domains tool (https://www.ncbi.nlm.nih.gov/Structure/cdd/wrpsb.cgi) according to Wang et al. [65], Lu et al. [66] and Marchler-Bauer et al. [67]. Additionally, the amino acid sequences for *CD36* gene and Ov-ERV-R13-*CD36* were predicted utilizing GENSCAN (http://hollywood.mit.edu/GENSCAN.html).

### Exploring Differentiation of Ov-ERV-R13-*CD36 *within *CD36* Gene Among Reference Genome and 42 Non-reference Genomes in Sheep

In this phase of the study, 43 sheep genomes were collected, including one reference genome and 42 non-reference genomes, as detailed in Table S6. These genomes were sourced from the NCBI database (https://ftp.ncbi.nlm.nih.gov/genomes; accessed on 12 January 2024). The investigation focused on identifying *Pres/Abs* in Ov-ERV-R13-*CD36* located within *CD36* gene across these genomes. Subsequently, the genomes containing Ov-ERV-R13-*CD36* insertion were analyzed for differences in long terminal repeats (LTRs), ERV length and domain structures using Fast-PCR software version 6.8 [68] and the NCBI Conserved Domains tool.

### Test Ov-ERV-R13-*CD36*-*Pres/Abs* in six sheep breeds

The methodology adopted in this part encompasses a comprehensive analysis of six distinct international sheep breeds, aimed at assessing traits such as OB/BCS, meat chemical composition, fat content and genetic polymorphism. The breeds examined in this study included Barki, Rahmani, Rahmani *x* Barki crossbred, Awassi, Ossimi and the Romanov *x* Rahmani crossbred. Barki sheep, primarily found in Egypt, are raised for meat production and are well-adapted to arid environments. Rahmani sheep, native to the Nile Delta region, serve dual purposes, providing both meat and wool. Rahmani *x* Barki crossbred combines traits from both breeds to enhance meat production and environmental adaptability. Awassi sheep, common in the Middle East, are mainly bred for milk production but also provide meat and wool. Ossimi, also found in Egypt, are primarily raised for meat and wool with good climate adaptability. Romanov sheep, originating in Russia, are known for their prolificacy and are typically used to improve reproductive traits and lamb production. Romanov *x* Rahmani crossbreed aims to combine the prolificacy of Romanov sheep with the adaptability of Rahmani sheep, suited for enhanced meat production [69, 70]. Through a structured experimental design and rigorous ethical protocols, data collection encompassed physical and chemical assessments, blood sampling for DNA isolation, and PCR verification to investigate genetic variations. This multi-faceted approach provides a robust framework for understanding the phenotypic and genotypic diversity within these sheep breeds, thereby contributing valuable insights into their suitability and performance in various agricultural and breeding applications.

#### Animals

The present study is part of a large project aimed at evaluating the production traits of six sheep breeds, encompassing a total of 1,355 animals, specifically; Barki (B, *n*=366), Rahmani (R, *n*= 198), Rahmani *x* Barki crossbred (RB, *n*= 426), Awassi (A, *n*= 143), Ossimi (O, *n*= 112) and Romanov *x* Rahmani (V, *n*= 110) as illustrated in Additional File 4, Fig. S1. The parents of these animals were sourced from five distinct geographical regions in the northern part of Egypt, namely, Alexandria governorate (GPS: 31.206208, 29.919704), Matrouh Province, El-Hammam district (GPS: 30.833132, 29.397580), Matrouh Province (GPS:31.353910, 27.235560), Salloum plateau city (GPS: 31.573626, 25.15922) and Sakha city (GPS: 31.090560, 30.943543). Although initially raised in the experimental station in northern Egypt, the experimental animals belonging to the six sheep breeds were adult individuals (average age of 36.50±0.75 months) randomly selected from respective contemporary groups born within a timeframe of 1~3 weeks. These animals were all kept under a semi-intensive system, allowing for free-range activities within spacious concrete barns and maintained under uniform conditions of feeding, climate and overall management.

### Phenotypic traits

#### Obesity (OB)/Body Condition Score (BCS)

Three experienced judges independently assessed the body conditions of the studied sheep breeds using OB/BCS, which applied a scoring scale ranging from 1-5 with increments of 0.5 points, as established by Morand-Fehr and Hervieu [71] and Russel *et al*. [72]. The median BCS recorded was 3, within a range of 1.50 - 5.00. In cases of discrepancies among the judges, a consensus decision was reached to determine the final score. The assessments were conducted at three specific sites: thoracic vertebrae or sterna (BCS^a^), rib cage (BCS^b^) and lumbar vertebrae behind the ribs/loin eye (BCS^c^) [73, 74], as illustrated in Fig. S2A & B. Detailed information on the visual body condition scoring system is provided in Table S7. OB/BCS evaluation was conducted on 1,355 individuals.

### Meat chemical composition, physical assessment and fat measurements

#### Breeds preparation

Six sheep breeds/genotypes (a total *n*=36); Barki (B; *n*=3♂ and 3♀), Rahmani (R, *n*=3♂ and 3), Rahmani *x* Barki crossbred (RB, *n*=3♂ and 3♀), Ossimi (O, *n*=3♂ and 3♀), Awassi (A, *n*=3♂ and 3♀), Romanov *x* Rahmani (V, *n*=3♂ and 3♀), were used to determine the meat chemical composition, assess physical parameters and measure the fat content. These individuals were slaughtered when they reached an age of 38 months.

#### Slaughtering

The animals were weighed, subjected to an 18 hrs fasting period with ad libitum access to water, and then weighed again just before slaughter. Subsequently, the animals were slaughtered and processed following standard commercial procedures. All experimental protocols and procedures were executed in compliance with the guidelines outlined in the Guide for the Use and Care-of-Agricultural-Animals in Research and Teaching by the Federation-of-Animal-Science-Societies (FASS, 2010) and the Guide for the Care and Use of Agricultural Animals in Research and Teaching, 3^rd^ ed (https://www.aaalac.org/about/Ag_Guide_3^rd^_ed.pdf). Moreover, all animal experiments were ethically approved by the ethics committee of Alexandria University, Faculty of Agriculture (*Al-Shatby*), Egypt (No. AU082209203103) and ethics committee of Yangzhou University, College of Animal Science and Technology, China (No. 202403005).

### Measuring physical assessment, meat composition and fat measurements

#### Post-Slaughter Processing

After slaughtering and bleeding, the head was removed at the atlanto-occipital joint, and the feet were detached at the carpal and tarsal joints. Initially, the carcasses were partially skinned while lying on their backs before being suspended by the hind legs for complete skinning. Weights for both carcass and non-carcass components were recorded immediately. Non-carcass components included the lungs, trachea, heart (collectively referred to as the pluck), head, skin, feet, digestive tract, liver, spleen and pancreas. The weight of the digestive contents was calculated by subtracting the weight of the empty digestive tract from that of the full digestive tract, and this was used to determine the empty live weight (ELW).

#### Physical assessment

Specific fat deposits such as kidney, omental, pelvic and mesenteric fats were isolated and weighed. Physical assessments, including fat, muscle and bone percentages were conducted following methodologies described by Sen *et al*. [75] and Santos *et al*. [76]. Carcasses were divided into sections, and the loin eye area was recorded. The trimmed meat, bone and dissected fat were weighed separately, which included initial measurements of bone (kg and %), trimmed meat (kg and %) and dissected fat (kg and %). Ratios such as lean:fat, lean:bone, and carcass:fasted weight (%) were also calculated to provide deeper insights into carcass composition. Composition analysis of chilled cuts was performed manually, calculating the distribution of lean, fat and bone based on the chilled carcass weight.

#### Fat measurements

For detailed fat measurements, the procedures outlined by Ekiz *et al*. [77] and Kirton *et al*. [78] were applied. Weights for specific fat deposits, including heart fat, kidney fat, gut fat, fat tail and gastrointestinal (GI) tract fat, were individually measured. This helped ascertain total fat stores and total body fat, which were calculated by summing all fat depot weights, providing a comprehensive assessment of fat distribution.

#### Determining the Chemical Composition of Meat

The chemical composition, including moisture, crude protein and ash, was determined according to AOAC, [79] and Madruga *et al*. [80] on the rib-eye area (*longissimus dorsi*) (Fig. S3A & B). Specifically, 100 g samples were taken from the left rib-eye area of each lamb. These samples were carefully trimmed to remove connective tissue and external fat. The samples were then freeze-dried, ground to pass through a 1 mm sieve, and stored for later analysis. Ash content was measured by combusting the meat samples at 600 °C for 8 hrs. The nitrogen content was assessed using the Kjeldahl method, with protein content calculated by multiplying the nitrogen value by 6.25. Fat extraction was performed using the Soxhlet apparatus.

### Blood sampling, DNA isolation, manipulation and PCR verification

#### Blood sampling

A venous blood sample of 5 ml was individually collected from each of the 144 randomly selected animals out of a total of 1,355. The blood samples were drawn from the jugular vein using venojects, treated with 0.5 ml of 2.7% EDTA (Pspark, U.K) as an anticoagulant, and immediately transferred in an ice box to the laboratory for further processing.

#### DNA isolation and processing

Genomic DNA was extracted from individual blood samples collected from randomly chosen males and females sheep of the experimental Barki (B; *n*=12♂ and 12♀), Rahmani (R, *n*=12♂ and 12), Rahmani *x* Barki crossbred (RB, *n*=12♂ and 12♀), Ossimi (O, *n*=12♂ and 12♀), Awassi (A, *n*=12♂ and 12♀) and Romanov *x* Rahmani (V, *n*=12♂ and 12♀) groups using a DNA isolation kit (Tiangen Biotech, Beijing, China). The isolated DNA samples were subjected to electrophoresis on a 1.2% agarose gel in 0.5x TBE buffer, as described by Sambrook *et al*. [81], supplemented with 0.5 μg/ml ethidium bromide. Electrophoresis was conducted using an apparatus with a power supply and visualization was carried out under an ultraviolet transilluminator equipped with a Gel documentation system (Chemi.DocTM XRS+ with Image LabTM Software, BIO-RAD, USA). The purity and integrity of the DNA were assessed using a NanoDrop Spectrophotometer (2000/2000c, Thermo Fisher ScientificTM, USA), with the optical density (OD) ratio monitored at 260/280 nm. DNA samples with a high-quality rating, displaying an OD ratio between 1.80 and 2.00 (average 1.82), were utilized for subsequent analyses, while samples of insufficient quality were re-extracted. The average measurements for concentration, volume and mass were determined as 70.47 ng/μL, 29.5 μL and 2.14 ng, respectively.

#### PCR verification on narrow scale

Six sheep breeds; Barki, Rahmani, RB, Ossimi, Awassi, and Romanov *x* Rahmani were chosen for PCR validation of full-length ERV polymorphisms. DNA samples were extracted from blood samples as described previously. Subsequently, ten individual samples from each breed were combined for initial PCR analysis to identify the expected ERV insertion polymorphisms within *CD36* gene. Given the typical length of full-length ERVs exceeding 8 kb, the careful design of double PCR primer pairs was implemented for genotype verification. The primer design strategy is elucidated in Fig. S4A. Primer sets (1 and 2) and (1 and 4) for Ov-ERV-R13-*CD36* were precisely crafted utilizing Oligo-7 software (V.7/2024) (https://www.oligo.net). The primers were synthesized by Vazyme Biotech Co., Ltd, located in Nanjing, China. Subsequently, the following primers were used: P1_13_F: 5'-AATGGTAAGCTCCCAAACTCA-3', P2_13_R: 5'-ACCAAGGGCAAACTTCCTCGATG-3', and P3_13_R: 5'-TTCACAAGCACTGACGGAT-3'.The amplification procedure used Green SuperMix (TaKaRa, Japan), with 10 pM of primers and 100 ng of genomic DNA per sample. The PCR protocol consisted of 40 cycles with denaturation at 95°C for 1 min, annealing at 58-60°C for 1 min, extension at 72°C for 1 min, followed by a final extension step at 72°C for 2 min. The amplification was performed using a T100 Thermal Cycler from BIO RAD, Singapore. Subsequently, 7-10 µL of PCR products and 4 µL of DL5000 molecular weight markers were subjected to electrophoresis on 1.0 % agarose gels in 1× TAE buffer at a constant voltage of 130 V for 30~45 min. The gels were stained with ethidium bromide and visualized under ultraviolet fluorescence. PCR amplification results showing bands within specific size ranges can determine the presence or absence of homozygous ERV insertion. A band of 1,056 bp with primers 1 and 2 indicates the ERV^+/+^ insertion genotype, while the absence of a band of 1,159 bp with primers 3 and 4 confirms this genotype. If a band of 1,159 bp is present without the 1,056 bp band, it suggests the ERV^-/-^ genotype. Observing both shorter and longer bands signifies a heterozygous RIP genotype (ERV^+/−^), as shown in Fig. S4B. This genotyping approach was based on the methodology described by Du et al. [54]'s framework.

#### PCR verification and Genotyping Investigation for Ov-ERV-R13-CD36 on a Large Scale

This part of the genotyping process involved testing 24 randomly selected individuals from each breed, resulting in a total sample size of 144 animals to assess Ov-ERV-R13-*CD36*.

### Statistical analysis

#### Analysis of variance

The study collected and analyzed data on OB/BCS, meat chemical composition, physical assessment, and fat measurements of the observed breeds. The normality of the data was assessed using the Shapiro-Wilk test (SAS, 2009), which indicated that all data followed a normal distribution (Shapiro-Wilk test (W) ≥ 0.90). The effects of Ov-ERV-R13-*CD36* on the target traits were evaluated using the GLM procedure in SAS, based on the model:

$${\mathrm Y}_{\mathrm{ijkl}}=\mathrm\mu+{\mathrm G}_{\mathrm i}+{\mathrm B}_{\mathrm j}+{\mathrm S}_{\mathrm k}+{\mathrm e}_{\mathrm{ijkl}}$$

Where: µ is the overall mean, G_i_ signifies the effect of the i^th^ genotype for Ov-ERV-R13-*CD36* (^+/+^, ^−/−^ and ^+/−^), B_j_ represents the fixed effect of the j^th^ breed, S_k_ indicates the fixed effect of the k^th^ sex and e_ijkl_ denotes the residual error. Variations between means within each weight category were analyzed using the least significant difference (LSD_0.05_).

#### Genetic Indices and Equilibrium Analysis

The chi-squared test (*χ2*) was employed to assess whether the six populations conformed to Hardy-Weinberg equilibrium (*HWE*). HWE analysis was conducted in the context of genetic association studies. Additionally, heterozygosity (*Ho*), expected heterozygosity (*He*), effective allele number (Ne) and polymorphic information content (*PIC*) were determined using Nei's methodology [82, 83].

## Results

### Analysing the Ov-ERV-R13-*CD36* Intersection Involving *CD36* Gene in Sheep Genomes

**Fig. 1:**
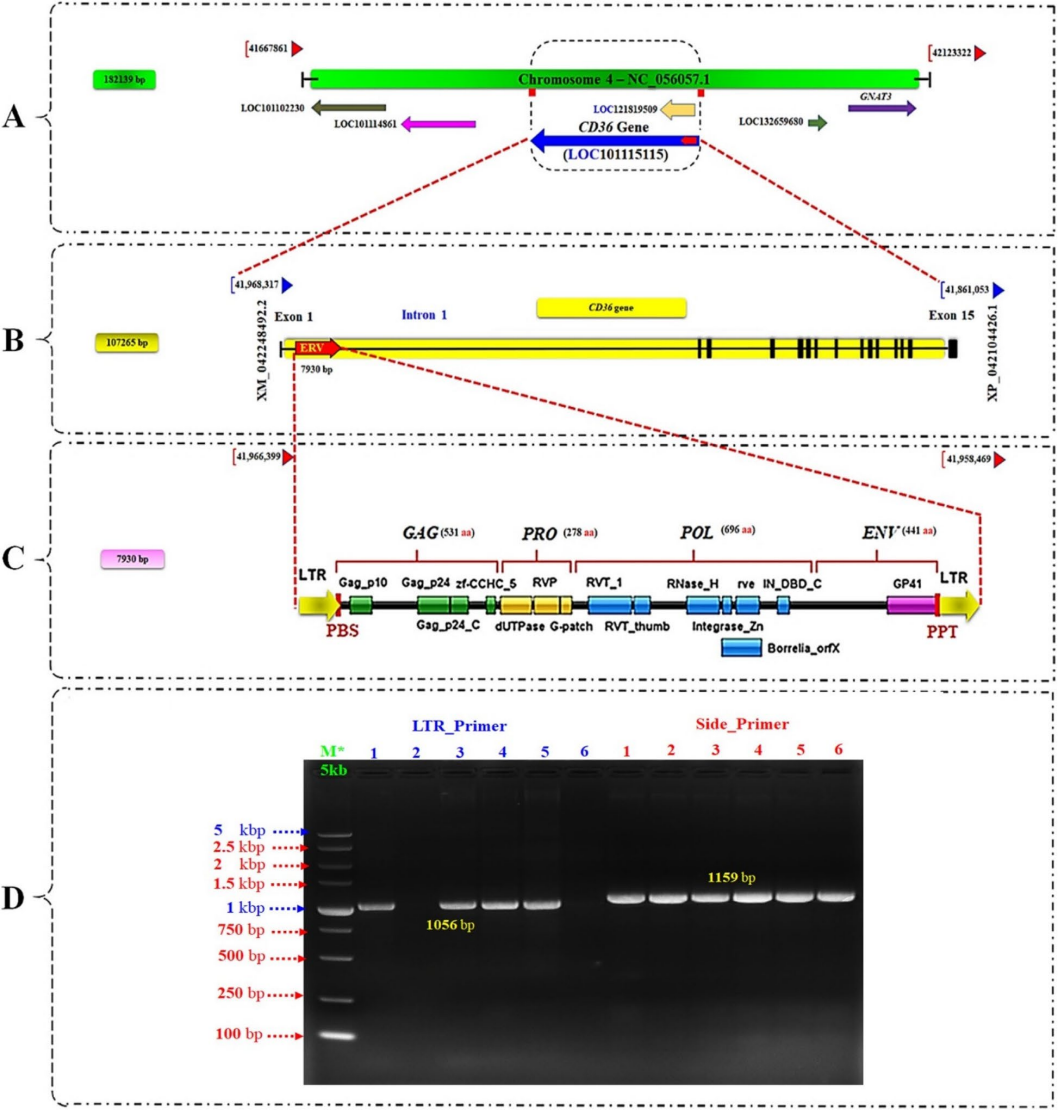
**A** Genetic map of Chromosome 4 in sheep (*Ovis aries*) indicating specific positions and directions for genes, including Cluster of Differentiation 36 (*CD36*) gene. **B** Genomic localization and functional implications of the *CD36* gene with Endogenous Retrovirus (ERV) insertion (Ov-ERV-R13-*CD36*) in sheep. **C** The structure organization of the Ov-ERV-R13-*CD36* is as follows: [Gag_p10: Retroviral GAG p10 protein; - Gag_p24: GAG gene protein p24 (core nucleocapsid protein); - RT_RNaseH: Reverse transcriptase RNaseH domain; -RNaseH: Endonuclease domain of reverse transcriptase;- zf-CCHC_5: GAG polyprotein viral zinc-finger; - trimeric_dUTPase: Trimeric dUTP diphosphatase; - RVT_thumb: Reverse transcriptase thumb domain; - Integrase_Zn: Integrase Zinc binding domain; - rve: Integrase core domain;- PBS: Primer binding site; - PPT: Polypurine tract[. **D** PCR Verification for the selected Ov-ERV-R13-*CD36*. 10 pooled samples per breed (Total; *n*=60); 1: Barki, 2: Rahmani, 3: Rahmani *x* Barki crossbred, 4: Awassi, 5: Ossimi, 6: Romanov *x* Rahmani. M: DNA Ladder 5 kbp.

Previous ERV annotation and full-length ERV insertion prediction [19] side by side with the current investigation, revealed that, in the reference genome, there were originally 218 full-length ERVs identified (Table S8). Using our specific methodology and criteria, which are thoroughly explained in the methods section, we identified 28 distinct groups (Table S2) that are distributed across 31 ERV-RIPs (Data set 2) in 58 sheep genomes. Upon further analysis through PCR testing and data mining, we discovered that 14 out of these 31 ERV-RIPs intersect with candidate genes (Table S3 and Data set 3) and exhibit polymorphic characteristics (Graph S3). Finally, one of these 14 ERVs, which is particularly associated with the traits of interest in our study, was selected for more detailed examination. The full-length ERV insertion, designated Ov-ERV-R13-*CD36*, located on chromosome 4, was selected due to its location within the significant candidate gene *CD36* (Table S3 and Fig. 1). Ov-ERV-R13-*CD36*, belonging to the Cap_ERV_24 family/group, is classified as a Class II (Beta retrovirus). By investigating Repbase data, we found that Cap_ERV_24/Ov-ERV-R13-*CD36* is novel.

Based on the multiple sequence alignment with available genomes, a 7,930 bp full-length ERV insertion on Chromosome 4 within *CD36* gene was revealed (Fig. 1A and Data set 4). Further genomic annotation identified that Ov-ERV-R13-*CD36* is located in intron 1 of the mutant *CD36* gene based on the reference genome (GCA_016772045.2, NC_056057.1:41861053-41968317), specifically within Transcript NCBI: XM_042248492.2, comprising 15 exons and 14 introns (Fig. 1B). Genomic localization and domain analysis of Ov-ERV-R13-*CD36* indicated that each LTR region is approximately 445 bp, with domains covering Group-Specific Antigen (Gag; 531 aa), Protease (Pro; 278 aa), Polymerase (Pol; 696 aa), and Envelope (Env; 441 aa) (Fig. 1C). Subsequently, the Ov-ERV-R13-*CD36* on chromosome 4 was subjected to initial PCR verification by using 10 pooled samples per breed. PCR amplification performed on the examined sheep breeds resulted in the production of a distinctive short fragment of 1,056 bp and a longer fragment of 1,158 bp (Fig. 1D), indicating the occurrence of a *Pres/Abs* polymorphism of full-length ERV in the studied sheep breeds. Additionally, domains for *CD36* and Ov-ERV-R13-*CD36* are presented in Additional File 5.

**Fig. 2:**
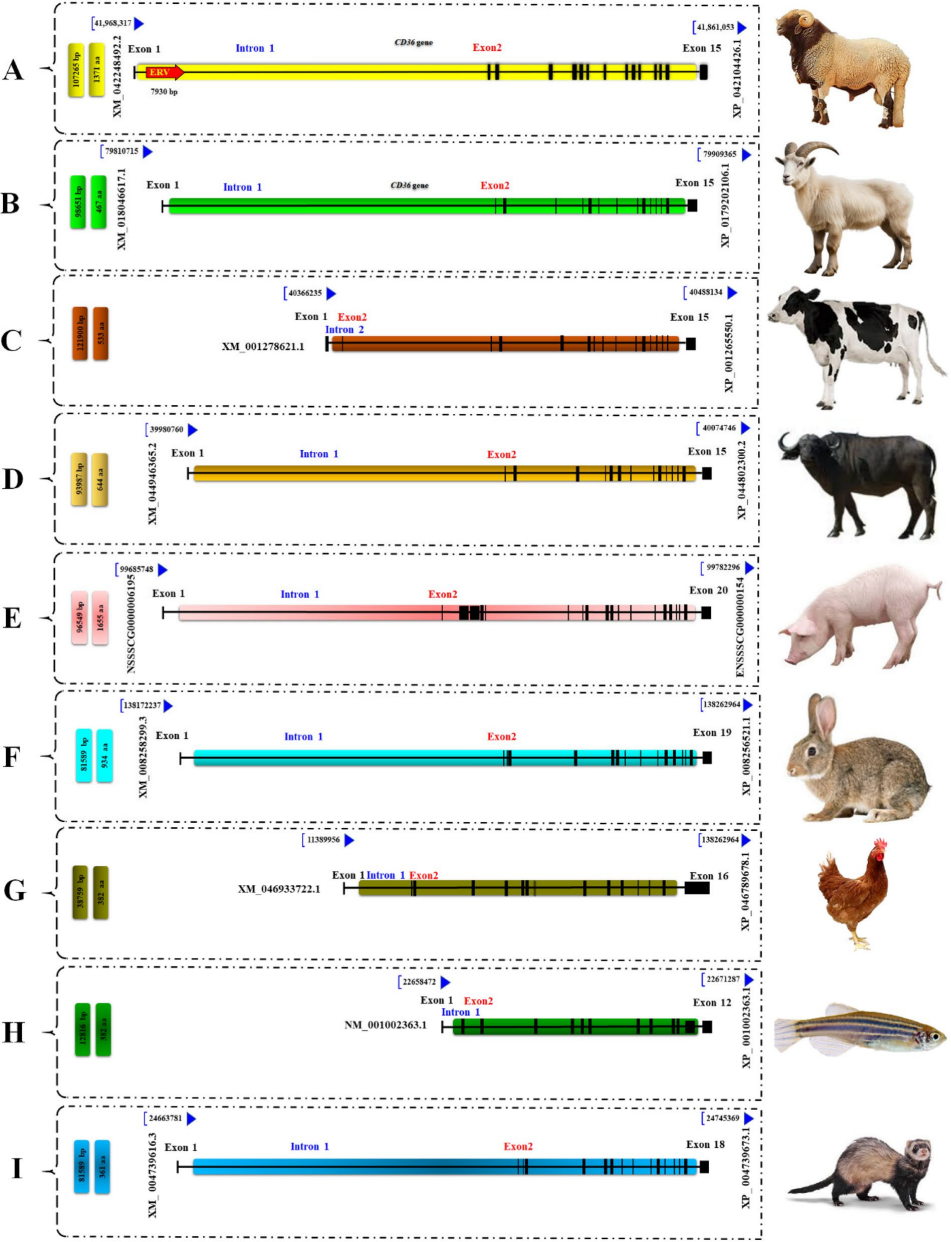
Analysis of *CD36* gene and its exon organization in the reference genomes of sheep (**A**), goats (**B**), cattle (**C**), buffaloes (**D**), pigs (**E**), rabbit (**F**), chicken (**G**), zebrafish (**H**) and domestic ferret (I).

Comparative genomic analysis revealed that *CD36* in several animal genomes (i.e., sheep, goats, cattle, buffaloes, pigs, rabbit, chicken, zebrafish and domestic ferret) has a large first intron, with several nearby genes, including the well-annotated *GNAT3* gene (Fig. 2 and Fig. S5). Fig. 2 provides a comprehensive overview of the structural similarities and differences surrounding *CD36* across diverse species, which may help elucidate how ERV insertions in this region could impact gene regulation and contribute to phenotypic variation. Furthermore, by identifying conserved genomic features, we can better understand the evolutionary pressures shaping *CD36* gene, which may have implications for its functional roles in various traits across different species. Worth mentioning, Ov-ERV-R13-*CD36* was identified in the reference genome of sheep within intron 1 of *CD36* gene, as shown in Fig. 2A, and is absent from the genomes of other species (Fig. 2B-I). Table S9 highlights significant differences in *CD36* gene across various species in terms of gene length, predicted amino acid sequences and transcriptional complexity. Notably, the sheep *CD36* gene spans 107,265 bp and has the highest number of transcripts (35), reflecting greater transcriptional complexity than goats and domestic ferrets, which have only 6 and 4 transcripts, respectively. The pig exhibits the largest predicted amino acid sequence, consisting of 1,655 amino acids, contrasting sharply with the shorter sequences found in zebrafish and chicken, each with only 382 amino acids.

In addition, alignment of the genomic regions of Ov-ERV-R13-*CD36* across 43 sheep genomes revealed that 22 genomes contain ERV insertions (mutant type), while 21 genomes exhibit an absence of ERV (wild type), as outlined in Table S10. The aligned sequences for Ov-ERV-R13-*CD36* in these 43 genomes are presented in Additional File 6. Furthermore, 22 genomes with the Ov-ERV-R13-*CD36* insertion were analyzed for variations in LTRs, ERV length, and domain structures, revealing notable variability in the lengths of the entire ERV, LTR and specific protein-coding domains, as shown in Table 1. The aligned sequences for the Ov-ERV-R13-*CD36* insertion within *CD36* gene in the 22 selected genomes are provided in Additional File 7. The total lengths of ERV insertion were 7,930 bp for 6 genomes, and 7,943 bp for 15 genomes and 7,756 bp for one genome. The length differences in the ERV domains (Gag, Pol and Env) likely result from accumulated mutations. The Gag domain typically ranges from nucleotide (nt) sequences coding for 531 to 616 amino acids (aa), with outliers at nt sequences coding for 193 and 173 aa in genomes such as GCA_002742125.1 and GCA_022416785.1, respectively. The Pro domain generally measures from nt sequences coding for either 278 or 289 aa, though shorter sequences are present in some genomes. The Pol domain is mostly represented by nt sequences coding for 777 aa long, although some, such as GCA_016772045.2, exhibit a shorter length of 696 aa. The Env domain ranges widely from nt sequences coding for 441 to 618 aa, but it is reduced to a length of 328 aa in GCA_002742125.1.

**Table 1:** Analysis of the Ov-ERV-R13-CD36 insertion in CD36 gene in the reference genome and 21 selected non-reference sheep genomes

**Sheep Breeds**	Strand	ERV Length (bp)				LTR-Similarity	Gag (aa)	Pro (aa)	Pol (aa)	Env (aa)	length of left and right LTR
		**Genome and Genome Location)**	**Length (bp)**	**Start position**	**End-Position**						
**Rambouillet**	**-**	**GCA_016772045.2:41862971-41870900**	**7,930**	**1,918**	**9,847**	**100%**	**531**	**278**	**696**	**441**	**444/444**
**East Friesian**	**-**	**GCA_022416915.1:42467070-42475012**	**7,943**	**1,918**	**9,860**	**100%**	**616**	**289**	**777**	**618**	**444/444**
**East Friesian**	**-**	**GCA_022416755.1:41900700-41908642**	**7,943**	**1,918**	**9,860**	**100%**	**531**	**278**	**696**	**441**	**444/444**
**Kermani**	**-**	**GGCA_022416685.1:41862971-41870913**	**7,943**	**1,918**	**9,860**	**100%**	**531**	**289**	**777**	**618**	**444/444**
**Ujimqin**	**-**	**GCA_017524585.1:42000250-42008192**	**7,943**	**1,918**	**9,860**	**100%**	**616**	**289**	**777**	**618**	**444/444**
**Qiaoke**	**-**	**GCA_024222265.1:41862971-41870900**	**7,930**	**1,918**	**9,847**	**100%**	**616**	**278**	**452**	**441**	**444/444**
**Tibetan**	**-**	**GCA_022432845.1:41862971-41870900**	**7,930**	**1,918**	**9,847**	**100%**	**531**	**278**	**696**	**441**	**444/444**
**Kazak**	**-**	**GCA_022416785.1:41862971-41870900**	**7,930**	**1,918**	**9,847**	**100%**	**173**	**278**	**696**	**441**	**444/444**
**Dorper**	**-**	**GCA_022538005.1:42000250-42008192**	**7,943**	**1,918**	**9,860**	**100%**	**616**	**289**	**777**	**661**	**444/444**
**Yunnan**	**-**	**GCA_022416745.1:41904579-41912521**	**7,943**	**1,918**	**9,860**	**100%**	**616**	**289**	**777**	**618**	**444/444**
**Romney**	**-**	**GGC_022416725.1:41862971-41870913**	**7,943**	**1,918**	**9,860**	**100%**	**173**	**278**	**696**	**441**	**444/444**
**Suffolk**	**-**	**GCA_022244695.1:41212520-41220462**	**7,943**	**1,918**	**9,860**	**100%**	**616**	**289**	**777**	**618**	**444/444**
**Romanov x White Dorper**	**-**	**GCA_022432825.1:41704158-41712100**	**7,943**	**1,918**	**9,860**	**100%**	**616**	**289**	**777**	**618**	**444/444**
**Romanov x White Dorper**	**-**	**GCA_022416775.1:41431105-41439047**	**7,930**	**1,918**	**9,860**	**100%**	**616**	**289**	**777**	**618**	**444/444**
**Chinese Merino**	**-**	**GCA_024222175.1:41862971-41870900**	**7,930**	**1,918**	**9,847**	**100%**	**466**	**278**	**696**	**441**	**444/444**
**East Friesian**	**-**	**GCA_021328995.1:41983611-41991553**	**7,943**	**1,918**	**9,860**	**100%**	**616**	**289**	**777**	**618**	**444/444**
**Kermani**	**+**	**GCA_021327315.1:30862869-30870811**	**7,943**	**1,918**	**9,860**	**100%**	**616**	**289**	**777**	**618**	**444/444**
**Ujimqin**	**-**	**GCA_021327145.1:46826-54768**	**7,943**	**1,918**	**9,860**	**100%**	**616**	**289**	**777**	**618**	**444/444**
**Romney**	**-**	**GCA_021325855.1:35456-43398**	**7,943**	**1,918**	**9,860**	**100%**	**616**	**289**	**777**	**618**	**444/444**
**Chinese Merino**	**-**	**GCA_021327395.1:7447602-7455544**	**7,943**	**1,918**	**9,860**	**100%**	**616**	**289**	**777**	**618**	**444/444**
**Texel**	**+**	**GCA_021325875.1:2480428-2488370**	**7,943**	**1,918**	**9,860**	**100%**	**616**	**289**	**777**	**618**	**444/444**
**White Dorper**	**-**	**GCA_002742125.1:45499852-45507607**	**7,756**	**1,919**	**9,674**	**99%**	**193**	**203**	**279**	**328**	**257/257**

### Population genetic analysis of Ov-ERV-R13-*CD36* by PCR

**Fig. 3:**
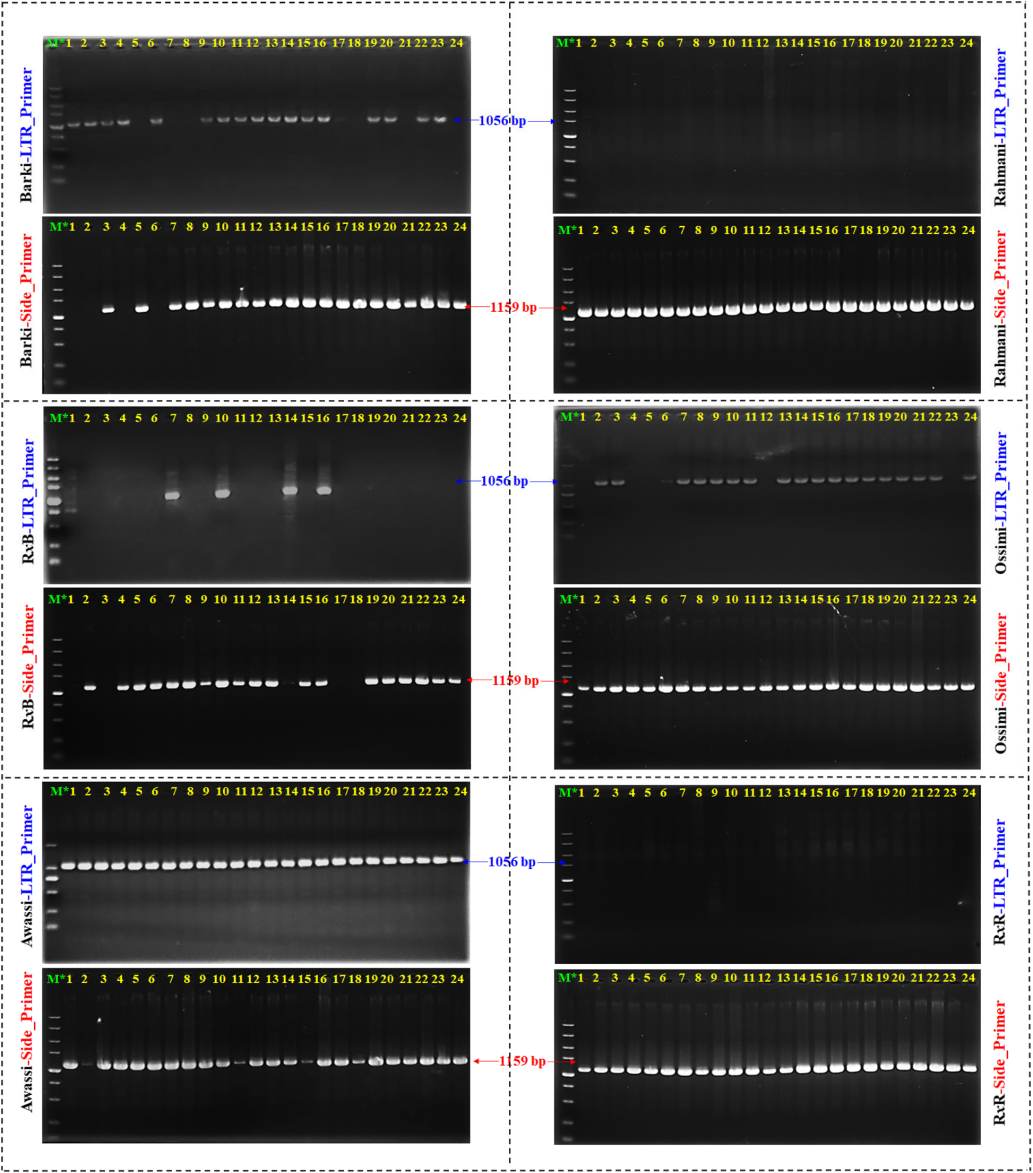
PCR Verification for the selected Ov-ERV-R13-*CD36*. 24 individuals (*n*=12♂ and 12♀) per breed, (Totally *n*=144); R*x*B: Rahmani *x* Barki crossbred and R*x*R: Romanov *x* Rahmani. M: DNA Ladder 5 kbp.

Furthermore, to validate the findings on a population level across a broader spectrum, a total of 144 individuals were randomly selected from six sheep breeds, with 24 individuals from each breed, comprising both male and female populations. This selection aimed to assess the occurrence of *Pres/Abs* in the genomes of the studied sheep breeds for this Ov-ERV-R13-*CD36*. This RIP is polymorphic in diverse breeds, and the representative PCR detection results are illustrated in Fig. 3. The analysis of Ov-ERV-R13-*CD36* genotyping in the studied sheep breeds revealed notable variations. Among the examined breeds, Barki displayed a diverse distribution with RIP^+/+^ at 16.67%, RIP^−/−^ at 29.17% and RIP^+/−^ at 54.16%. Rahmani and Romanov *x* Rahmani exhibited a distinct pattern with 100% RIP^−/−^ genotype, RB took the second place for RIP^−/−^ genotype at 83.33 after Rahmani and Romanov *x* Rahmani, while Awassi showed 100% RIP^+/−^ (Table 2 and Fig. S6). These results underscore the genetic diversity present among the sheep populations, as reflected in *H*_*o*_, *PIC* and *Ne*, emphasizing the genetic complexity within these sheep breeds (Table S11).

**Table 2:** Ov-ERV-R13-*CD36* genotypes and their association with Obesity (OB)/ Body Condition Score (BCS) in the studied sheep breeds.

**Trait/ genotype**	**ONH-RSA**^**1**^	**RSA-GI**^**2**^	**SLA**^**3**^	**Ov-ERV-R13-*CD36***			**OB/ BCS**		
**Breeds**	**No.**	**No.**	**No.**	**RIP**^**+/+**^** (*Pres)***	**RIP**^**−/−**^** (*Abs*)**	**RIP**^**+/−**^** (*Pres/Abs*)**	**Mean**	**Condition**	***p*-values**
**Barki**	366	24	6	16.67%	29.17%	54.16%	2.73±0.25^**c**^	Thin ~ Average	< 0.001
**Rahmani**	198	24	6	0.00%	100.00%	0.00%	4.62±0.48^**a**^	Obese	< 0.001
**Rahmani *x* Barki**	426	24	6	4.17%	83.33%	12.50%	3.96±0.33^**b**^	Fat	0.259
**Awassi**	143	24	6	0.00%	0.00%	100.00%	3.15±0.75^**d**^	Average	0.088
**Ossimi**	112	24	6	0.00%	16.66%	83.34%	3.25±0.26^**d**^	Average	0.479
**Romanov *x* Rahmani**	110	24	6	0.00%	100.00%	0.00%	4.51±0.38^**a**^	Obese	< 0.001
**Total**	1,355	144	36	6	75	63	----	----	----

^1^ONH-RSA: The number of individuals per herd remains consistent under identical feeding and weather conditions were randomly selected at the same age to measure Obesity (OB) and Body Condition Score (BCS)

^2^RSA-GI: Randomly selected Animals for Genetic Investigation

^3^SLA: Slaughtered Animals.

The observed absence in Ov-ERV-R13-*CD36* could potentially impact *CD36* gene`s function in regulating OB/BCS and fat storage within the body. Absence in critical regions of *CD36* gene (Figs. 1 & 3) may disrupt its normal physiological activities related to fat metabolism and storage, especially for Rahmani, Romanov *x* Rahmani and RB breeds (Tables 2 and 3). Understanding the implications of these absences on *CD36* function is essential for elucidating their potential role in obesity development and fat accumulation processes.

### Genotypes of Ov-ERV-R13-*CD36* and their association with economic traits in sheep

The current study identified potential associations between Ov-ERV-R13-*CD36* genotypes and both BCS and OB measurements (Table 2), as well as fat measurements (Table 3) in the studied sheep breeds. The analysis of Ov-ERV-R13-*CD36* genotypes across various breeds revealed noteworthy correlations with OB/BCS. Among the studied breeds, Rahmani and Romanov *x* Rahmani exhibited the highest OB/BCS (*p* < 0.001), both displaying a 100% RIP^−/−^ genotype. In contrast, Barki breed had the highest frequency of the RIP^+/+^ genotype (16.67%) and the lowest OB/BCS (*p* < 0.001), indicating a range in body condition from thin to average. Also, RB breed demonstrated a significant presence of the RIP^−/−^ genotype, accounting for 83.33% of the population, and achieved the second-highest OB/BCS after Rahmani and Romanov *x* Rahmani breeds (Table 2).

**Table 3:** The physical assessment, chemical composition and fat measurements of the studied sheep breeds

**No.**	Category	Measurements	Mean ± S.D*							*p-values*
		**Breeds**	**Barki**	**Rahmani**	**RB**^**1**^	**Ossimi**	**Awassi**	**Romanov *x* Rahmani**		
		**RIP**^**−/−**^** (*Aps*)**	29.17%	100.00%	83.33%	0.00%	16.66%		100.00%	
**1**	**A-Physical assessment**	**Final live body weight**	49.042±2.633^**c**^	59.641±3.23^**a**^	53.790±2.783^**b**^	52.220±1.750^**b**^	54.866±2.360^**b**^		57.538±2.258^**a**^	< 0.001
**2**		**Bone (Kg)**	3.574±0.378^**c**^	3.706±0.365^**b**^	4.199±0.538^**a**^	3.603±0.174^**bc**^	3.690±0.276^**b**^		3.898±0.641^**b**^	0.003
**3**		**Bone (%)**	14.337±1.655^**b**^	14.581±1.662^**b**^	13.131±1.419^**c**^	14.524±0.875^**b**^	13.100±1.098^**c**^		15.529±2.881^**a**^	0.001
**4**		**Trimmed meat (Kg)**	17.781±1.231^**c**^	18.248±0.946^**b**^	23.83±0.252^**a**^	15.625±0.782^**d**^	17.475±1.597^**c**^		17.787±1.637^**c**^	< 0.001
**5**		**Trimmed meat (%)**	71.261±2.165^**b**^	71.605±2.001^**b**^	74.689±1.541^**a**^	62.889±1.886^**c**^	61.782±1.433^**c**^		70.382±4.225^**b**^	< 0.001
**6**		**Dissected fat (Kg)**	2.257±0.181^**c**^	3.682±0.13^**a**^	2.837±0.231^**bc**^	2.397±0.203^**c**^	2.853±0.139^**bc**^		2.876±0.180^**b**^	0.002
**7**		**Dissected fat (%)**	4.625±0.371^**c**^	6.193±0.406^**a**^	5.189±0.554^**b**^	4.597±0.424^**c**^	4.634±0.029^**c**^		4.997±0.190^**b**^	0.041
**8**		**Lean: Fat ratio**	47.937±2.32^**c**^	55.052±3.118^**b**^	52.274±2.347^**b**^	48.038±1.716^c^	58.327±2.915^**a**^		57.003±2.108^**a**^	< 0.001
**9**		**Lean: Bone ratio**	47.298±2.498^**d**^	55.025±3.166^**b**^	51.73±2.196^**c**^	47.499±1.759^**d**^	57.811±3.152^**a**^		56.542±2.137^**a**^	< 0.001
**10**		**Carcass: fasted weight **^**(**^**%)**	52.29±4.099^**b**^	53.636±1.622^**b**^	59.461±2.053^**a**^	48.69±2.132^**c**^	47.079±4.635^**c**^		52.652±0.403^**b**^	0.001
**11**		**The net meat ratio (%)**	44.77±3.299^**b**^	45.793±0.845^**b**^	51.647±1.827^**a**^	41.616±1.796^**c**^	40.912±4.104^**c**^		44.464±1.181^**b**^	0.002
**12**	**B-Chemical composition**	**Moisture content (%)**	70.276±1.942^**c**^	71.578±0.795^**b**^	73.309±1.588^**a**^	72.959±1.839^**a**^	74.401±1.662^**a**^		70.276±2.03^**c**^	0.004
**13**		**Crude protein (%)**	19.684±0.406^**b**^	19.828±0.579^**b**^	22.221±0.338^**a**^	18.671±0.263^**c**^	18.342±0.401^**c**^		19.424±0.368^**b**^	0.001
**14**		**Ash content (%)**	1.006±0.058^**a**^	1.007±0.041^**a**^	0.835±0.069^**c**^	0.867±0.045^**c**^	0.774±0.041^**d**^		0.971±0.075^**b**^	0.001
**15**		**Collagen (%)**	2.756±0.334^**b**^	2.801±0.15^**b**^	2.289±0.052^**c**^	2.508±0.201^**bc**^	3.03±0.364^**a**^		2.65±0.256^**bc**^	< 0.001
**16**	**C-Fat measurements**	**Total Fat Stores (Kg)**	2.257±0.275^**c**^	3.539±0.328^**a**^	2.826±0.247^**b**^	2.370±0.180^**c**^	2.770±0.214^**b**^		2.850±0.187^**ab**^	0.024
**17**		**Heart Fat Weight (g)**	88.076±2.853^**c**^	74.094±2.293^**d**^	85.629±5.218^**c**^	90.310±2.720^**b**^	100.790±2.500^**a**^		100.590±1.640^**a**^	0.046
**18**		**Kidney Fat Weight (g)**	229.324±3.610^**b**^	243.28±2.770^**ab**^	200.176±6.977^**c**^	225.46±5.960^**b**^	204.505±10.415^**c**^		330.80±1.320^**a**^	0.035
**19**		**Gut Fat Weight (g)**	479.355±1.469^**b**^	206.677±4.487^**c**^	192.376±8.939^**c**^	482.20±3.590^**b**^	505.18±3.590^**b**^		812.81±1.590^**a**^	0.001
**20**		**Fat Tail Weight (kg)**	1.329±0.058^**c**^	2.600±0.072^**b**^	3.272±0.100^**a**^	1.570±0.040^**d**^	2.600±0.040^**b**^		0.726±0.120^**e**^	0.056
**21**		**GI Tract Fat (g)**^**2**^	862.690±31.023^**b**^	913.183±23.654^**a**^	939.82±34.60^**a**^	780.50±66.75^**c**^	851.98±66.75^**b**^		939.76±66.560^**a**^	0.004
**22**		**Total Fat in the body (kg)**	3.586±0.255^**c**^	6.216±0.458^**a**^	6.098±0.479^**a**^	5.363±0.141^**b**^	5.363±0.141^**b**^		5.496±0.258^**ab**^	< 0.001

^*^SD Stander deviation

^1^RB: Rahmani *x* Barki crossbred

^2^GI Tract Fat: gastrointestinal tract fat.

Additionally, the analysis of dissected fat in various sheep breeds uncovered potential genetic associations with Ov-ERV-R13-*CD36* genotypes. Rahmani sheep, with a 100% RIP^−/−^ genotype, exhibited the highest dissected fat weight (3.682 ± 0.13 kg, *p* = 0.002), suggesting a predisposition to fat accumulation. Conversely, Barki sheep had the lowest fat weight (2.257 ± 0.181 kg), indicating leaner body composition. Also, Romanov *x* Rahmani sheep showed relatively high fat weight (2.876 ± 0.18 kg, *p* = 0.002), closely followed by BR breed, which exhibited a fat weight of 2.837 ± 0.231 kg. These findings may emphasize the complex relationship between genotype and fat deposition in sheep (Table 3).

The variations in fat levels and genetics among Rahmani, Romanov *x* Rahmani, and BR sheep breeds may correspond to their Ov-ERV-R13-*CD36* genotypes. The introduction of hyper-breed genetics may enhance total fat content in RB crosses [70], with the exception of Rahmani and Romanov x Rahmani breeds. This potential enhancement is likely associated with an increase in the frequency of ERV^−/−^ individuals, which may positively influence genetic traits related to fat content.

## Discussion

ERVs exhibit a significant antisense bias in gene introns, indicating strong selection against elements aligned with gene transcription. This suggests that such biases might influence economic traits by potentially affecting gene regulation [53, 84, 85]. The findings from Klymiuk *et al*. [16] and Cumer *et al*. [17] provide an overview of the ERVs present in the ovine genome and their evolutionary implications. Klymiuk *et al*. [16] identified multiple OERV families and discovered novel open-reading frames, suggesting that these sequences could have active biological roles or implications in sheep biology. On the other hand, Cumer *et al*. [17] challenged previous notions regarding the domestication-associated fixation of protective enJSRV mutations by proposing an alternative model based on their evolutionary timeline. This model posits that such protective mutations, which arose after key evolutionary divergences in the Ovis lineage, may have provided a selective advantage due to natural selection, rather than domestication alone.

In this aspect, the integration of ERVs can have a significant impact on gene activity and ultimately influence phenotype by modulating regulatory elements such as introns, exons, promoters and enhancers of genes [28, 86–90]. Phenotypic variations resulting from ERV-*Pres/Abs* have been documented in diverse domesticated species, including dogs [91], cats [92], sheep[19] and pigs [54]. For instance, a study on pigs found a correlation between ERV-D14-RIP within *STAB2L* target gene and traits related to BW and growth. This correlation affects variations in BW and growth rate, particularly in Large White pigs [54]. The identification of polymorphisms and differentiations arising from ERV-*Pres/Abs* in domesticated animals may significantly contribute to revealing underlying structural variations responsible for phenotypic diversity and could potentially impact breeding practices [10].

In the present study, the analysis of ERV distribution in 58 sheep genomes, including one reference genome (ARS-UI_Ramb_v2.0), revealed varying genomic coverage of ERVs, ranging from approximately 6.02% to 10.05% (Table S8). Using a specialized dataset of 28 ERV groups (Data set 1 and Table S2) across 58 sheep genomes, bioinformatic analysis identified 31 ERV-RIPs (Data set 2). Table S2 provides a detailed classification of the analyzed ERV families and highlights their relevance to Ov-ERV-R13-*CD36*. Our investigation of Repbase data revealed that 22 out of the 28 families, including the tested family (Cap_ERV_24/Ov-ERV-R13-*CD36*), are novel, while the remaining 6 have been previously identified, including Cap_ERV_1 (OviAri-5.324_LTR), Cap_ERV_4 (OviAri-5.2557_int), Cap_ERV_10 (OviAri-1.272_LTR), Cap_ERV_12 (OviAri-6.2056), Cap_ERV_13 (OviAri-1.306), and Cap_ERV_14 (OviAri-3.284_LTR). Further validation revealed that out of the 31 full-length ERV-RIPs, 14 ERVs (45.16%) were located within candidate genes (Table S3) and subsequently confirmed through PCR detection (Data set 3 and Graph 3). Moreover, the selected ERV insertion fragments analyzed were over 7 kb in length, indicating a predominance of truncated copies within the sheep genome, specifically the selected RIP named Ov-ERV-R13-*CD36* spanning approximately 7.93 kb. In this regard, Du *et al*. [54] reported that the presence of full-length ERV insertion polymorphisms, characterized by substantial structural changes exceeding 5 kb, pronounced genetic influence. This suggests a potential impact for some ERVs on gene expression and function due to significant overlap with coding and non-coding regions [85, 93–97].

In-depth investigation of the selected Ov-ERV-R13-*CD36* revealed significant structural variations in *CD36* gene, attributed to the absence of ERV insertions, particularly in Rahmani and Romanov *x* Rahmani breeds (Fig. 1D). PCR verification on a large scale further validated the occurrence of *Pres/Abs* in the genomes of the studied sheep breeds for this specific Ov-ERV-R13-*CD36* (Fig. 3). The 100% Ov-ERV-R13-*CD36* ^−/−^ genotype (absence /wild type) observed in Rahmani and Romanov *x* Rahmani breeds, verified using PCR, aligns with our investigation of 43 sheep genomes (Additional File 6). Among these, 21 were found to have absences (Table S10) across several international sheep breeds: Rambouillet, East Friesian, Polled Dorset, Waggir, Charollais, Texel, Romanov, White Dorper, Hu, Yunnan, Kermani, Ujimqin, Romney, Suffolk and Charollais.

The associations between Ov-ERV-R13-*CD36* genotypes and economic traits, including OB/BCS (Table 2), and meat chemical composition and fat measurements (Table 3), displayed notable variations among different sheep breeds. Rahmani and Romanov *x* Rahmani breeds, with a unique 100% RIP^−/−^ genotype, followed by RB breed (83.33%) demonstrated the highest mean for OB/BCS and fat content among all the studied breeds. These findings imply that these particular breeds may have lost the protective effect of *CD36* gene against OB/BCS due to the absence of Ov-ERV-R13-*CD36* within *CD36* gene. In contrast, Barki breed, with the highest distribution of the RIP^+/+^ genotype (16.67%) among different breeds, and comprising 54.16% RIP^−/+^ and 29.17% RIP^−/−^ genotypes, displayed the lowest OB/BCS and mean dissected fat weight. This finding indicates that Barki breed may have a protective effect due to the function of *CD36* gene against OB/BCS through insertion. The varying distribution of RIP genotypes within the studied breed highlights the genetic diversity present in this population and its potential impact on fat metabolism.

The current results align closely with Bokor *et al*. [98] research findings in humans indicating that SNPs in *CD36* were significantly associated with higher body mass index and body fat. Also, the current result agrees with Clop *et al*. [99] who identified three *CD36* variants associated with growth and fat deposition by analyzing the genome of pigs. In this aspect, Zhao *et al*. [31] found 11 SNPs in *CD36* gene in chicken, with specific mutations detected at different locations; 2 SNPs in the 5′ flanking regions, 8 SNPs in the intron region and 1 SNP in the exon region, which belonged to a synonymous mutation. These mutations were associated with specific traits, such as abdominal fat weight, full-bore weight rate and skin yellowness. Haplotypes of these SNPs were correlated with heart weight, stomach weight, wing weight, leg skin yellowness and shin skin yellowness before slaughter. In birds, the SNP (g. 476593 T > C) of *CD36* gene was significantly associated with total cholesterol and lipoprotein cholesterol levels, the result demonstrated that *CD36* might be an important genetic marker for the selection of lipid metabolism and meat quality traits in ducks [100]. Additionally, Rać *et al*. [101] reported that *CD36* expression in skeletal muscles increases in response to elevated levels of triacyl glycerides (TG) and fatty acids (FA) in the plasma. This regulatory mechanism is influenced by the energy demands of the tissue.

*CD36* not only transports fatty acids into the cell for lipid synthesis and metabolism but also affects cholesterol uptake and inflammatory responses [102, 103]. In *CD36* knock-down mice, *CD36* deficiency suppressed fat deposition in the viscera, subcutis and gonads caused by high-fat feeding and decreased BW [104]. Researchers injected *CD36* knockout precursor adipocytes into the back of mice and then fed them at high-fat levels, then found that the weight of new fat and the size and number of adipocytes in the experimental group were lower than those in the control group compared to the mice injected with no knockout cells [105]. These results suggest that *CD36* plays a prominent role in the differentiation and development of adipose tissue [106]. Long-chain fatty acids (LCFAs) are an important substrate for ATP production within the skeletal muscle. As a fatty acid transporter protein, *CD36* locates in skeletal muscle cell membranes and mitochondrial membranes, and plays a major role in the uptake and transport of LCFA, regulating energy sources and lipid metabolism in skeletal muscle [107]. Also, it serves as a ligand-receptor of thrombospondin, long-chain fatty acids, oxidized low-density lipoproteins (LDLs), fatty acid profile and oxidized lipid uptake [51].

The current findings align with the research conducted by Fagerberg *et al*. [108], which revealed a ubiquitous expression of *CD36* in various organs, with the most pronounced expression observed in fat tissues. The authors proposed that *CD36* protein might function intracellularly to influence adipose tissues and organ size, thereby regulating adipogenesis and other related processes. Furthermore, *CD36*-RNA is found in several tissues. The researchers confirmed that *CD36* gene expression was positively correlated with other genes influencing obesity, BCS, fat measurements and body size, as determined through quantitative transcriptomics analysis (such as RNA-Seq) which measured normalized mRNA abundance and verified using statistical correlation methods like Spearman correlation coefficient. The current findings suggest that the homozygous Ov-ERV-R13-*CD36*^-/-^ variant in the Rahmani, Romanov *x* Rahmani, and RB breeds may be associated with a larger body size and extreme OB/BCS.

Therefore, exploring the molecular mechanisms through which *CD36* affects OB/BCS or fat measurements in various domesticated animals, notably sheep, could offer valuable insights into potential therapeutic targets for OB and metabolic disorders, especially with the newly generated and organized data concerning *CD36* gene across various species.

While this study provides valuable insights into the role of Ov-ERV-R13-*CD36* in economic traits associated with *CD36* in sheep, there are limitations that warrant consideration. Future research should explore how environmental factors may influence the expression and effects of ERV insertions. Additionally, employing advanced techniques, such as quantitative real-time PCR (qRT-PCR) and RNA sequencing, could offer a more detailed understanding of *CD36* gene expression and its regulatory mechanisms. Future investigations will show whether Ov-ERV-R13-*CD36* represents a causal variant statistically associated with the observed trait, or simply a tag variant in linkage disequilibrium with a causal variant

## Conclusions

In conclusion, our extensive examination of Ov-ERV-R13-*CD36* within *CD36* gene across multiple sheep breeds has revealed potential genetic correlations with OB and BCS, as well as fat deposition. Through detailed genomic investigations, including PCR analysis and data mining, we initially identified 31 ERV-RIPs among 58 sheep genomes. From these, 14 displayed polymorphic characteristics that intersect with candidate genes, with Ov-ERV-R13-*CD36* being particularly associated with economically beneficial traits. Our findings emphasize Ov-ERV-R13-*CD36* as a potential genetic marker, specifically its full-length 7,930 bp insertion within intron 1 of the mutant *CD36* gene on chromosome 4. This presence, validated by PCR, revealed distinct variation in ERV-RIP^-/-^&^+/+^ genotypes across different breeds. Notably, Rahmani and Romanov *x* Rahmani exhibited a uniform RIP^−/−^ genotype that correlated with significantly higher (*p* < 0.001) OB/BCS and increased fat accumulation. While these insights offer a basis for strategically targeting breeding programs aimed at enhancing these traits, it is essential to acknowledge the limitations of our study. A more comprehensive understanding of how the ERV insertion affects *CD36* expression and its causal relationship with OB is needed. Future research could benefit from broader and more diverse sample populations, exploring environmental factors and further elucidating the function of *CD36* variations in fat metabolism. Additionally, it would be valuable to investigate the potential impacts of Ov-ERV-R13-*CD36* absences on *CD36* functionality and their role in OB and fat accumulation processes more deeply. Employing qRT-PCR in future studies would enhance our understanding of gene expression dynamics related to these traits. Overall, this research lays a foundational step towards comprehending the genetic basis of these economically significant traits in sheep, opening promising avenues for subsequent breeding programs and genetic research.

## Supplementary Information

Additional File 1. Supplementary Tables: Table S1. The tested 57 assembled non-reference sheep genomes in addition to one reference genome. Table S2. The library included 28 ERV families sourced from LTR retrotransposons used in the current investigation. Table S3. The anticipated variations in the insertion of full-length ERVs in the genomes of sheep. Table S4. Primers utilized in PCR-Based activation tests for 31 Endogenous Retroviruses (ERVs). Table S5. Investigated Genomes for CD36 gene in sheep and eight other species. Table S6. Reference genome and 42 non-reference genomes in Sheep.Table S7. Body Condition Score (BCS). Table S8. Analysis of Endogenous Retroviruses (ERVs) in 58 sheep Genomes: Distribution and Characterization of Full-Length Sequences. Table S9. Investigating CD36 gene in sheep and eight other species. Table S9. Exploring the differentiation in Ov-ERV-R13-CD36 and CD36 gene for Reference genome and 42 non-reference genomes in Sheep. Table S11. Ov-ERV-R13-CD36 genotyping in the studied sheep breeds and population genetic parameters

Additional File 2. Mining Protocols: Graphs

Additional File 3. Data Sets: Data Set 1. 28 Endogenous Retrovirus (ERV) groups were identified through a comparison of genomic coordinates. Data Set 2. 31 ERV-RIP insertion polymorphisms were predicted through a comparison of genomic coordinates using 28 ERV groups. Data Set 3. 14 out of the 31 full-length Endogenous Retrovirus (ERV) insertion polymorphisms were chosen following a comparison of genomic coordinates. Data Set 4. Sequences for Chromosome 4 in sheep (Ovis aries), CD36 gene and Ov-ERV-R13-CD36

Additional File 4 Supplementary Figures: Fig. S1. Sheep breeds scrutinized in the present study; Barki (B), Rahmani (R), Rahmani x Barki cross (RB), Awassi (A), Ossimi (O) and Romanov x Rahmani (V). Fig. S2. A) Points of consideration; 1. the sterna (sternum region), 2. the ribs (the rib cage) and 3. the lumbar vertebrae region which involves the spinous and transverse processes of spine ''behind the ribs'' (a: spinous process, b: flank and c: transverse process) are palpated to determine BCS. B) Diagram ''cross-section'' for assigning BCS in small ruminants. Fig. S3. A) The Rib-eye area (longissimus dorsi) in sheep. B) The muscle longissimus thoracis et lumborum (LL) in sheep. Fig. S4. A) Design principles for full-length LTR polymorphic primers. 1&2) LTR-primer produces a shorter band 1,056 bp. 3&4) Side-primer produces a band of 1,159 bp. B) After PCR, a band of 1,056 bp with Primer (1 and 2) and no band of 1,159 bp with Primer (1 and 3) indicates ERV+/+. While a band of 1159 bp without the 1,056 bp band suggests ERV-/-, while both bands mean ERV+/-. Fig. S5. Analysis of CD36 gene and its genomic regions and nearby genes in the reference genomes of sheep (A), goats (B), cattle (C), buffaloes (D), pigs (E), rabbit (F), chicken(G), zebrafish (H) and domestic ferret (I). Fig. S6. PCR Verification for the selected Ov-ERV-R13-CD36 in 24 individuals (n=12♂ and 12♀) of Rahmani x Barki crossbred Breed, M: DNA Ladder 5 kbp

Additional File 5. Domains for CD36 and Ov-ERV-R13-CD36.

Additional File 6. The alignment of CD36 gene for one reference and 42 non-reference genomes.

Additional File 7. The insertion Ov-ERV-R13-CD36 in CD36 gene for Reference genome and selected 21 non-reference genomes in Sheep.

## Data Availability

All data generated or analyzed during this study are included in this manuscript, its information files, and additional files; A. Additional File 1. B. Additional File 2. C. Additional File 3. D. Additional File 4. F. Additional File 6. G. Additional File 7.

## Abbreviations

LTRs: Long Terminal Repeats
ERVs: Endogenous Retroviruses
RIPs: Retroviral Insertion Polymorphisms
LINEs: Long Interspersed Nuclear Elements
SINEs: Short Interspersed Nuclear Elements
*CD36*: Cluster of differentiation 36
NCBI: National Centre for Biotechnology Information
PCR: Polymerase Chain Reaction
QTL: Quantitative Trait Locus
WGS: Whole-Genome Sequencing
lncRNA: Long Non-Coding RNA Genes
Gag: Group-Specific Antigen
Pol: Polymerase
Pro: Protease
Env: Envelope
*HWE*: Hardy-Weinberg Equilibrium
*Ho*: Heterozygosity
*He*: expected heterozygosity
*Ne*: Effective Allele Number
*PIC*: Polymorphic Information Content
