# Supplementary material for: Association of a 7.9 kb Endogenous Retrovirus Insertion in Intron 1 of CD36 with Obesity and Fat Measurements in Sheep
Source: Mob DNA. 2025 Mar 14;16:12. doi: 10.1186/s13100-025-00349-w (PMC11908002; doi:10.1186/s13100-025-00349-w)
Supplement: Supplementary file 2 — Additional File 2. Mining Protocols: Graphs [file 13100_2025_349_MOESM2_ESM.pdf]

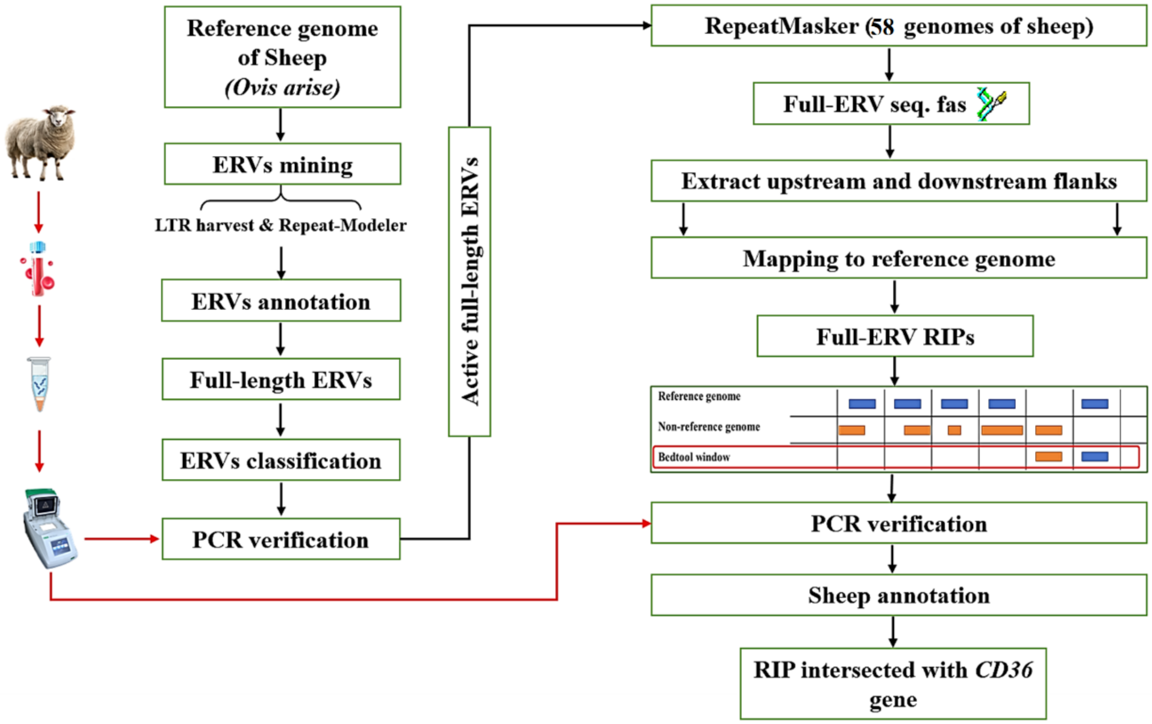

Graph S1. Steps for Mining Protocol.

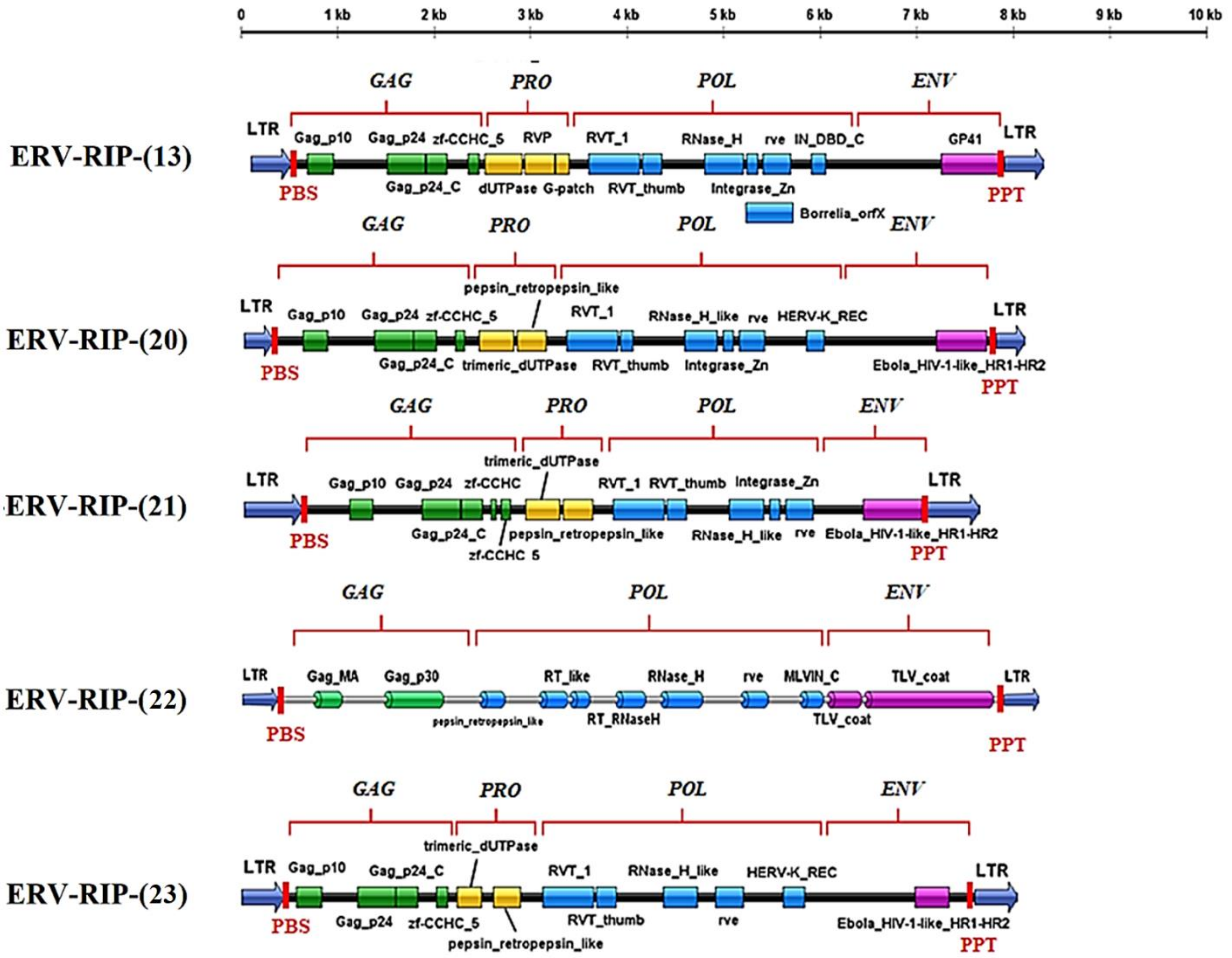

Graph S2. The Structure organization of the Ovis-ERV: 13, 20, 21, 22 and 23, is as follows [**Gag_p10:** Retroviral GAG p10 protein; **- Gag_p24:** GAG gene protein p24 (core nucleocapsid protein); **- RT_RNaseH:** Reverse transcriptase RNaseH domain; **-RNaseH:** Endonuclease domain of reverse transcriptase; **-MLVIN_C:** Murine leukemia virus-like integrase C-terminal domain; **-TLV_coat:** Sheep betaretrovirus capsid protein; **- zf-CCHC_5:** GAG polyprotein viral zinc-finger; **- trimeric_dUTPase:** Trimeric dUTP diphosphatase; **- pepsin_retropepsin_like:** Cellular and retroviral pepsin-like aspartate proteases; **- RT_like:** Reverse transcriptase (RT, RNA-dependent DNA polymerase) like family; **- RVT_thumb:** Reverse transcriptase thumb domain; **- Integrase_Zn:** Integrase Zinc binding domain; **- rve:** Integrase core domain; **- HERV-K_REC:** Rec (regulator of expression encoded by corf) of HERV-K-113; **- Ebola_HIV-1-like_HR1-HR2:** Heptad repeat 1-heptad repeat 2 region (ectodomain) of the transmembrane subunit of various endogenous retroviruses (ERVs) and infectious retroviruses, including Ebola virus and human immunodeficiency virus type 1 (HIV-1); **- PBS:** Primer binding site; **- PPT:** Polypurine tract] .

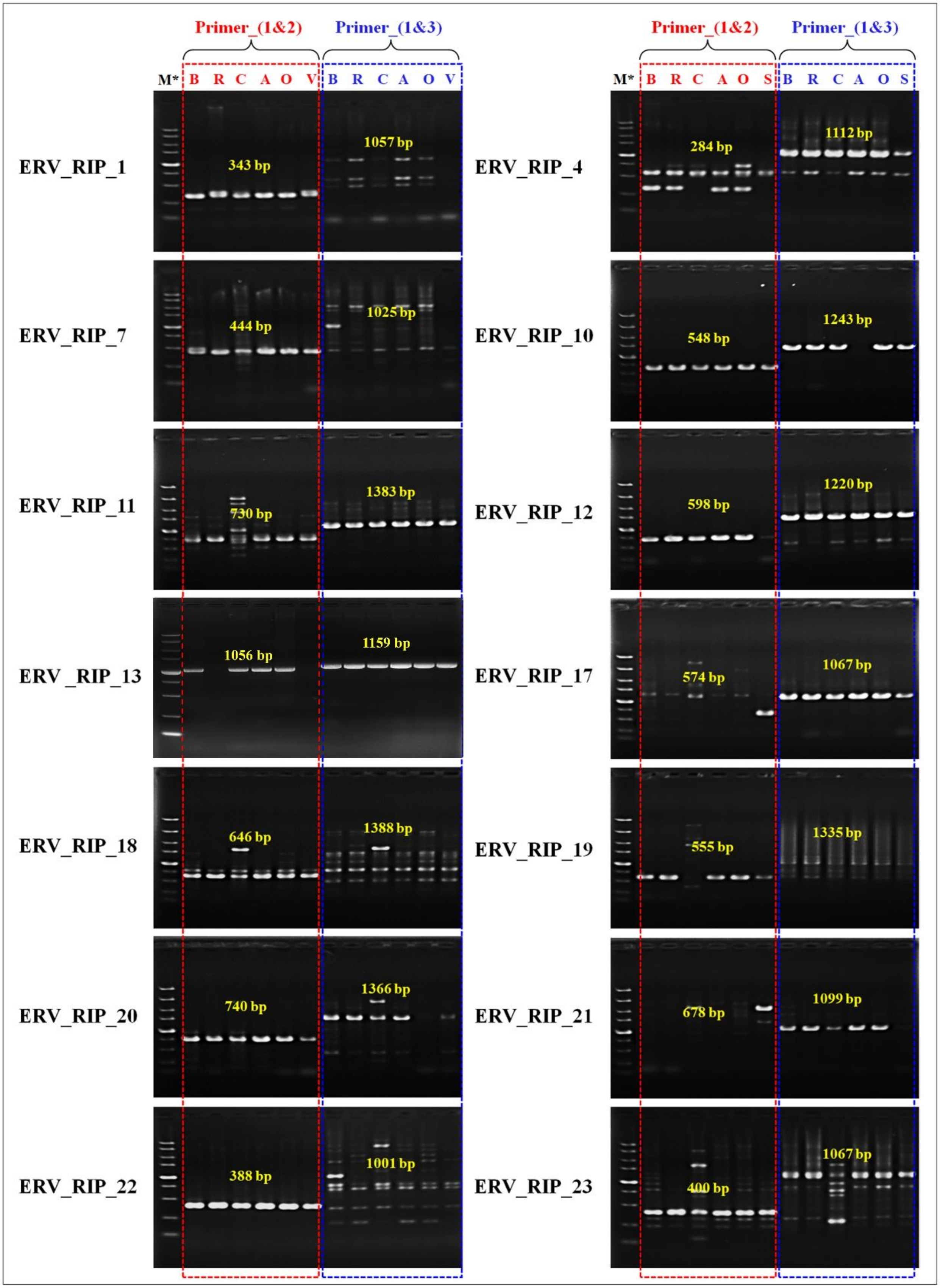

Graph S3. PCR Confirmation of Active ERVs among ERV-RIPs. This figure provides the PCR results evaluating the presence of 14 purportedly active endogenous retroviruses (ERVs) among the 31 identified ERV-Related Insertion Polymorphisms (ERV-RIPs).
