# Supplementary material for: Association of a 7.9 kb Endogenous Retrovirus Insertion in Intron 1 of CD36 with Obesity and Fat Measurements in Sheep
Source: Mob DNA. 2025 Mar 14;16:12. doi: 10.1186/s13100-025-00349-w (PMC11908002; doi:10.1186/s13100-025-00349-w)
Supplement: Supplementary file 3 — Additional File 3. Data Sets: Data Set 1. 28 Endogenous Retrovirus (ERV) groups were identified through a comparison of genomic coordinates. Data Set 2. 31 ERV-RIP insertion polymorphisms were predicted through a comparison of genomic coordinates using 28 ERV groups. Data Set 3. 14 out of the 31 full-length Endogenous Retrovirus (ERV) insertion polymorphisms were chosen following a comparison of genomic coordinates. Data Set 4. Sequences for Chromosome 4 in sheep (Ovis aries), CD36 gene and Ov-ERV-R13-CD36 [file 13100_2025_349_MOESM3_ESM.pdf]

Data Set 1

Data Set 1. 28 Endogenous Retrovirus (ERV) groups were identified through a comparison of genomic coordinates.

ERV_1

ACACTGATTGAAACCGCCCACCCTGGCCAGGCACCATAGTAACCATTTGCATGAGTT
GTTTTATGACAGGAGATCCTGGTAAGGAATACAGAACTAATAAGCCATCACCAACT
GGAAGAGTCCAGGAAAGGTCGAAAGGAGATACTGCGTGTCTGTCCACTTCCCAGAA
TCCCTCTTGCTAGCATCCATCTTGGCTGAGCGATGTTTGCGCCACCAGGAAAGACTC
TGAATTAGAATGATTGGCCAAAGACCACCCGAAACTAATCCCATCACCATAAAACC
TAACATTGTGAGCCATGTGGCAGAGCAGTTCTCCTGGGTTCCCTTACCCAACTGCTC
TCCACCCGGGTGCCCTTTCCCAATAAAATCTCTCGCTTTGTCAGCACGTGTCTCCTCA
GACAATTCTTTTCCTAGTGTTAGACAAGTGCCTGCTCTTGGGGCCCTGTAGCGGGGC
CTCCCTCTTCCTACAACAACTGGCGACCACGAAGGGACACCCCCATTTTTACTGGGG
CTGACATCCAGTCTGCTCGGGGTACTCCAGGACCAGCTCACCTGCCGATGGATCTGA
CCCAGCGGCCGCAACTGGGACTCGTTTGTCCCTGGTCTCCTCCTGACGCAGACAACT
GGCCAGGGTGCCCTGACCAGGTAAGGAACAAGAGATTTTGTTGACCTCCTTTATCTC
ACCCCTCCTTTCAACCTATCCTATCCTATTTTTCTAGTCCCCCAGTCCTGGATACAGA
AATCTTGTCGAAGGGCCTCAGCCTGAGCTGAGGATTGGAGACTGATCACCTCCTCTT
GGCAGAGAACTCGAACTCTGATTCTGATTCCAGTCCTCCCTTCTCTGGAGGCCCAGG
GAAAAGTCCCTTAACGCCTGGGTATCTGTAGGTGGCAGAAGACGTCTGTAAGGCCA
CCCTTTTTTTCTTTTTCCTCTTTCTCTCCCCGACTCTTAGATCTTTTGTCTCTTCCCACT
CCTTCTCTCTCAACCTGGCCTCTCCTCCTCTCTTTAAAAACCCAAGTTATTTCCATTC
ACTCTGTCGATACTTGGACCTGAAGTTCTGGGTCTTTCCAAGAGATTTTCTGAGAGG
CTACGCTCTCTGCTCCCCATCAATTGTGCTTTCTATTCTTGGGGAGACAATCGGGCGT
TAGAAACCCTCCATCGGGTGCCCTGTTTCTATAAATTCAGTCATTTAATTGAGCATTT
GGCTGAGTGCTTCCACGTGGAAGTGATTGATGGGATTCAGTATAGTCACACCTGGTC
TTGGGTGAAAATTAACATCCTAGAAACATCCTTAGGGGTTAATTCACCTTTCAGGAC
GAGTCCTTCAGCCTCGTTGATTGTGCTTACTACTGAACATTTCCTTTGGTGCATATAC
AATCAGGCAATTTGCGACCAGGAGAGAGCTTACAATCTCTAGAGTCAGATATAGGG
GCACCCACCCCAATACTGGTCCCCTGGGAATGAGATATAAACTATACCATCACTAAA
ATGGGAAATAAGCCTAGCTTCCCTTTAGACACCCTGCTGGGTTGTCTTTTGGCCCACT
AGGAGGGATACTAACTGGAAGGGCTAAAAAAGAAAAAACTTATCAAATACTGTACT
CAATACTGGCCTACTTACTCCCTGGGAGGAGGAGAAAATGGCCCCAGTTGGGATCA
CTTGGATACAACACCATTTTACAGCTCAGTCTATACTGCAAACGCGAGGGTAAATAC
AAGGAGGGTCCCCATGTCCAGGCCTTTGTGGCCCTCTACCAGGATTCCGGGAAAAA
GGGAAAATATAAGCTTGAGGACTTTGAGAAATGCTCTTCCAAAATCCTTTTAGCAAG
GTCTGAAATAGAAGATCCCCTTGACCTTCCTTGACCTCCTCTGCTTCAGCAGAAAGG
TCACGGGGAAAGGAGGGTAAGACACACCCGGAAATTACGAGCTCGGACCTAGAAGT
CAGGAGGCTGGGTCCAGAAATCATGGGATCTGAAGGGAAGTCACTCACCATCACCT
CGGTTCCTCCTCCATATGCCCCCTCGCCCCATGGGTTTATGATCAGGAAGTAAGCTTT
GTCCTCCACAGAACGAAAGGTCTTCCTCAGACAAAATCTGCCCCCTAAGAGAGGTA
CCAGACAGGGAGGGGGGAACAATCCACATTCATGCTCCATTCTCTATGCAAGACATT

ACCCAATGCAAGGAACAACTGGGCTCATATTCCGAGAACCCCCCCAACTTTAAGGA
CGAATTTGAATGTCTTAGCCTTAACTTCTCCCTCACCTGGATGGATATAATGGTCATC
CTCACCCAGTGTTGTAATGATGAGGAAAGAGCTCGAATCCTAGATCAGGCTAGAAA
AGTGGCTGATGAGAGACAGCGGGCAGATGTCAGTCTGGCCCCTGCCGAAGAGGCAA
TCCCCTCTACAGAGCCAGACTGGGACCCTAATACAAGGGCAGGAGAGGAATCCGTA
AGACACCTCATTACTTGCCTACTACAGGGAATGACCCAGGGTGTCCGAAAGGGTGTT
AATTATAATAAGATAAAAGAGGTTACACCGGAAGAGAATGAGAACCCAGCTCTTTT
TTTGGGCAGACTCACAGAAGCCTTTAAAAACTTTACCAAGACAGATCTAAAAATCAT
GGAAGGAAGAGTCCTGTTGGGCCATTCCTTCATAACCCAGGCAGCCCCAGACATAA
GGAGAAAATTACAAAAACTAGGAAAGGGACCAGAGACTCCAATTTCCGATTTGGTG
GAGGAGGCAAATAGGGTGTTTTTGAACAGGGACCAGGAGGAGGAAGCAAGAAGGG
AGCAAAAGGAAGCCTGAAAAGATAGGAGAATGGAGAGGCAGACCCAGGCCTTGGC
CCAACAACAGGCTAAAATCCTGGCTTTGATTCATCCTGCCACTATGCGAGAGTCTGG
GGTAGAGCAAGGAAGGAAAAAGGATCTTAACAATCCACCGTGTCTGAGACACACCC
AGTGTGCCTACTGCAAAGAGGATGGGCACTGGAAAAGGGAGTGCCCATATTACCCT
AAGGGAAGGCGTATGGAGACCCCTAAACCTGTTCCCTCTGTATTAGTCCTGGGCGAC
CAGGACTGACAGTACTCAGTGGCTCAAGAAATCCCCGTGAATGGTGAGGTCCAGAT
CACTCCTCTCGACCCTTGGGTGACTCTACAAATAAAAGGTTAAGGATATGAACTTTC
TTTTAGACACGGAGGCTGCCTTCTCTGTTCTTCCTTTCTGATTAGGACTTTTAGACTC
CCAGATTAAAATGGTTATAGGGGTAGATGGAAAACCCCAGAGCCGAAATTTTACTA
AGCCCCTCTGCTGTAAGGTGGGCAATTGGCAAGGAACCCACCCCTCCTTGTACATCC
CTGGTTGCCCCACTCCCCTACTGGGGAGAGACCTTCTTTGCTGCCTCCAGACCAGAG
TGACTTGGGGAAAGCTAGAAGTCAATAATTTCCTTTGCCTAGTGTCTGAATGTTTAC
AGTTAGATGTAGAAAAGGAGGAGTCTGTCCCTTTCTTAGATGAGGTCAATCCCCAGG
AGTGGGATATCTCCAGCCCTGGTCTAGCCATAAATGTTCCACTGGTAAAGATTCTTC
TGAGGCCAAATGCTCCCTACCCCTGGAAAAGACAATATCCCTTAAAACCAGAAGTT
CTTGAAGGTCTTGGACCATCAGTTAACAAATACCAAGATACTGGAATTCTAATCACC
TGTGAATCTCCCTGCAATACTCCAATCTTGCCTGTTAAAAAACCAGATGGATCTTAT
CGATTCATCCAGGATCTAAGTGCTGTTAATGAAGCTGTGGTTCCCATCCACCCCATA
GTCCCAAATCCGTATACACGCCTATCTCAAGTCCCAGGAAATGCTAAGTACTTCTCA
GTCTTGGATCTTAAAGATGCCTTCTTTTGTATTATTCTCCATCCTGAATCCCCAAAAT
TCTTTGCTTTTGAGTGGCGGGACTTGGAAACAAGGGAGGCCATATAACTCTGCTGGA
CTCGACTACCACAAGGGTTTAGGGACAGCCCTCATATTTCTGGAACTTCCTTGGGGA
GAGAACTAAGGGAATTAACTTCAACCAACAGTAATCTAATACAATATGTGGATGAC
TTGCTCATAGCTAGTCCAAATTTTACCAGCTCTCAAATGGACACTATTAAAACTCTA
AATTTCCTGTATGAAAAGGGTTATCGAGTATCCTCCAGGAAAGCTCAGATTAGCTTA
ACCCAAGTAAAATACCTTGGATTTATAATTATGGAAGGAAAAAGAATGCTCGACCC
CCAGAGGAAATCCCTCATCTTAAATACCCTATACCCCCAAACAAAAAAACAGCTTA
GGGGATTTTTAGGAATGACTGGGTTTTGCAGGATCTGGATTCCTAATTATGGCAATC
TGGCCCAACCCCTATATGAAAAGTTAAGAGGGAAAGAGGAAGAGCCACTCGACCAG
GATGAGACCTGCAAGGTGGCCTTTAATGCCCTAAAAGAGTCTGTCACTACAGCCCCA
GCTTTAGGCCTCCCAAACCTGGAAAAGCCTTTGAGGCTTTATGTTTCTGAAAGGATA
GGAACTGTTCTTGGGATGTTAGGACAAATGATGGGGCCTGTATTACAACCCATGGCT

TATCTCTCAAAACAACTAGATGAGGTGGCCAGAGGGTGGCCCACTTGCCTCTGGGCA
GTAGCGGCCACCACTCTTATGGTTAAGGAGGCATCTAAGCTGACCCTGGGTCAGCCC
ACCACAGTGTATATGCCTCACCAGGTGCAAGCAGTCTTGGAAACTAAAGAGGATAG
GTGGATGACAGGGAGAAGGATCACACAATACCAAGCCCTCCTCCTTGACACTCCAG
AAATAAAGTTGAGGGTCTGTCAGACTTTAAATCCAGCCACCTTACTGCCAGATCCCC
CTACTTCCCCTGTGGATCATCAATGCATTCAAATCATAGACGAGTTATACTTTTCTCA
CCCAGACCTATCAGAGACACCTTTATGTGACCCAGAGGAAAAATAGTACACAGATA
GCAGTAGTTTTGTAGAAAAGGAAGAGAGGAAAGCAGGATATGCTGCAGTGAGCCTA
GAAGAAACTAGGAAAAGTGGGATTCTTCTCCCAGAACCTCAGCCCAGAAAGCTGAA
CTTTTTGCCCTGCCAAGAGCTTTGGAATTAGGGGAAGGAAAGAGGATTAATATGGA
CTGTAAGTATGCTTTCCTCATCCTGCATGCCCATGCAGCAATTTGGAAAGAAAGAGG
GATGCTCAGTGCTCGAAGCTCTCCTATTAAACACAAGGAGCTCATTCTCAGGCTCCT
GGAGGCAGTCAGACTTCCTGCTAAATTAGCTGTCATCCACTGTAAGGGTCACCAAAA
GGGGCAAGAAGAGGAGGCCCAGGGAAATAGAAAAGCTGACCAGGAGGCAAAACGA
GCTGCTAGCTGCTAGATAGCTGTCACTGCTATCTGCCCCCTTTTCCCCAAGGAAACC
CTGACTCCAGATTATACCCCAGAGAAACATTCCCGATAAGCTGAGCGGGGCTGGGA
GATCGGGTCACATGGGTGGTTTCAGACTGATCAGGCCCAAGTAATACTCCCTGACTC
TCAGGTTTGGAAAATTATTAACTCCTTACATAAAAGTGCCCATTTTGGAAGAGATAA
TCTGGAAATCTTGCTCAAGCCTATTCTCTACCATCCCCAGTTGGCTAAGGTTGTTAGG
TCACACAGAACTGTGATACTTGTCTAAGAAATAATCCAAAGACCAGACCCTGGGCA
CCTCCTCTAATTAAACCTGTCCAACTTCGGGGATCATACCCAGAAGAGGACTGGCAG
GACTTACAGCCATGCCAAAGACCCAAGGGTTTTCTTATTTGCTAGTCTTTATTGACAC
GTTCACAGGATGGATTGAAGTGTTCCCTACTAAGACAGAGAGAGCCACAGAAATCT
GTAAAGCTCTGCTGAAGGATACAGTACCTATGTTTGGGTTGCTTCGATCACTTCAGA
GTGACAATGGGCCCTCATTTACAGCCACGATATTGCAGGACCTGGCCGCATGCTCAG
GCATCAAATACCGCCTCCACTCACCCTGGAGGCCTCAAGCTTTGGGAAAGGTAGAG
AGAGCCAACCAGACTCTTAAAAGGGCTCTGGCCAAGCTATGTCAAGAGACACATAA
TAAGTGGATCCATGTGTTACCCATAGCCCTCATGAGGGTACAGACAGCCCCTAAATG
GCCACTATTACTAAGCCCTTAGGAACTGATGTATGGATGTCCATTCTTAACTTCTGAT
TTGTTTTTTGATGAGGACAGCAATACTCTCTTAAAACATATAATTGACCTGGGAAGG
TTTCAACAAGAACTCCAACGATATGGAGAACAGATCCTCCCTAGACCACAGGAAAA
CTTGAAAAATCCCCAGGTAGAGCCGGGGGACCGAGTATTAGTAACGACCTGGCAAG
AAAAAGGGAGTCAAAGCCAGCTGTCCGAGAAATGGACAAGACCATATCAGGTTGTC
CCAGTAACTTCAACTGCTGTAAAAGTGAAGGGTCTATCTGCCTGGGTCCACAATTCT
AGAATAAAACCCTATGGCCTACAGGAAGGGGAGACAGGGAATGAGACTCCAAAAC
CAGAGGACAACTATTCCTGTGAACCTGTTGAAGATCTCAGACTCTTGTTCAGGTGGA
ACCCAATCATCTCTCCCTCAGATAAGTAAAAATGCATCATATGATGTTCTTCCCCTTT
CTAATCATGTCTCCTTGTATGGCTATTAATCTCTCCCTAAACGAGGCATATACGCTTG
CAAACCACACTGCTTCTCTACTCAACCTAACCAGCCCCTGCTGGATCTGCATTAAAG
GCAGGAGTTTGGGGTATGTCAACCTGTCCCAGGGTATCAGTGGCCTACCCTTCCAGC
CGAGCTACAAGCTGTTACCCAGCACACCCCATCTGGTCACAGTATGACCTTTTGGGA
AAAATGGGAAAAAAACTTCATGCCCTGGTATCAGCAACAACTGTCTATGTTTACCCC
CTCGATAATTACAGACCCACCTGAGCCCTTATTTTCTCTTTTGACCAACATTCCCCAA

GTGACCTTCCCTACCTGTCTAAAAAGTGACTCCCTGACAGGGGTGTCAGTGAGAAAT
CTAGGCAACTCACAATATGCGGTGACCTTCACTGTTGAAACTGATGGGAAAAACTG
GGACCAAAAATTTCACTAGGGTATTACCATTTAGGCCAAAGTCTGGGAACTAGAGA
TTCTGGCAGAACTGACATACTATATCTATCCCTGAACATAAGGCTCTCTTCAGATGC
TATTAACCAGTCTCAAAAATCCATGTCCTGTTCTGGACGACCAAAGAACCACACCCA
TTCATCCTGGTTTCTAAATCGGGCAGGCAACTCTGACCCCTGTTATTCCCTTGTAGGT
GTACCCACCCCTGAAAACCTAACCCTATGGTGGGAGCCTAAACAAAATACTCTTTAC
CTTGAGAACAAAACCCAGGATGCCTCACAACACTTAATCCCTTCCTAAATGCCCTCA
CTGCTGCCGGTTTTGCAGCAAGTGGAGTGATAGTCTCCTCCCAGCTACTAAGAGACT
GCCCCTGTTCCATTATGCCACGTTCCAGTTGTAAGGCAGATGGAAATCCGGGTTTGC
TGCCCAGGTTTAAACCCAGTCATGGGGTCTATTTCATATGTGAGGATAAAGCTTATT
TATCCCTACCACCTTTTTGGTCAGAAGCTTGCTCCCTAGGCTATGTTACACCCCACAT
AGATCTGGCCTGGAGTAATACCTCCTTGCCTTTACCAGTATATACCAACCAGAGGAT
ACATCGTGCAGTAATACTTACCTCTCCTTTCCTGGCATTAGGACTGACTGCTGGTTTG
ACAGGAGCTGCCATGGGGGGAACTTCTTTACATAAATTTCAGCAGCTTTCTACCAAC
ACTGCTGTATCCATAGAAAAAACAGCGAGGACCCTCCAATGCCTACAATCCCAGCT
AGACTCCCTAGCCGCAATGGTCCTACAAAATCATCGGGCCCTAGATTTACTGACTGC
AGGACGGGGGGCACTTATCTCTATCTAAAGGAAGAATGCTGGTTTTATTACAACCAA
TCTGGGCAGTTCCAAGAAGATATCAAGGGGCGTTTGGAACAGGCCACAAAGATCCA
GGATTTGAGTTCAACAAGTGTATGGAGTTCTCTTTCTTTCCCTTCTTGGTTCCTACCA
TTCATGGGTCCAGTGATAACTATCTTATTAGGCCTCCTATTTGGCCCCTTATACTCCA
ACTCCTTACAAAGTTTATTTCCTGCAGACTCCAGAAGTTCCAGGCCAAATTGATATT
ACTACAAGGGTAGAAGCTGGTTTCAGATGTGGAACACCCCAACTTAGATCAGGTAG
AGAGAGAGACTTCTGCCCTGCTAGGCAGGCCTACACCCATGCACAGCAGGAAGAAG
TTACAGAAGAAAGAGACCTCCGCCCCAATTCCCAAGAAATATCTTGAATATGAAGT
CTCTTGGGGGAATTGTCAGGAGAAGCAACACTGATTGAAACCGCCCACCCTGGCCA
GGCACCATAGTAACCATTTGCATGAGTTGTTTTATGATAGGAGATCCTGATAGGAAT
AAGGAACTAATAAGCTACCACCAACCGGAAGAGTCTGGGAAAGGTCGAAAGGAGA
CACTGTATGTCCGTCCACTTCCCAGAATCCCTCTCACTAGCATCCATCTTGGCTGAGC
AATGTGTGCACCACCAGGAAAGACTCTGAATTAGAATGATTGGCCAAAGACCACCC
GGAA

ERV_2

TGTTGAGGCCTGATAGAGGCCATGACTAAAATGGGCTGGAGCAAGCATTCCGGAAA
CAGCAGAGACACTGCCCAGGTGGGATCAAAATTAATTACCCCTGACGGGAGGACTC
AGCCTGTCAGCATGCTCCCTGTAGCCTGGTCTGTGCCAGCCGGGAGGAGGATAGATA
TCTCCTAAGAAAGCCCGCATGCGCGAAAATCTAGCCAATTAGCAGATGCAAAGAAA
CCCTTGTAACCAATCCAACCTTGCCAATTCCCTGTCTTTGTCCTAAACCTATAAATAC
TACTGTAATATAGGGCTCTGGGCTCTGGCTCTATTCCACTGTGTTGGATGCAGCCAG
GGCCCTGGCTCAAGCTAGCAATAAATTCCCTTTTTTGCGTTTGCGTTGCCGTGGATGT
CTTGTTCTCTCAGTTCTGGGGATCGGGACCTTGGGCATAACATTTGGGGGCTCGTCC
AGGATCCCTTGACTGAGAAGAACAACACCTTCCAGGCATAAGGGAAGCCTCACTAG
GAGGAAATATATCTGGACGCTGGCATCAGGTCAGGGGGCAAAAGAGTCGCCCGGCC

AGCATAAGTCCGAGGCCCAGCATTTGAGCTGGAAGGCAGCTGGTTCTGTGAGCCGTT
CTGTAAAGGAATGGGGAAAAACATTTCTCCCGATCCTGAGTCTGGTGGGCAGTGGA
TGCCCTTCGGTAAGGTAACTTTGCGGAACAGGACAACAGCAACCCAGGATAAACCT
AGTTCAGTGTTTTCGGCTGATATTTGTTCGTCTACTGTCTGTCGCTTGTCTTTGTGTGC
CAGTGGCGTGCTTGCTTTTGTGCGTTTATTGTGAGTGTTGCCGTCATGGGTCAAAGTT
ATTCTACACCGCTATCTGTAATGACTGACCATTTTTCCGATTTCAAGTCCAAAGCTCA
GAATCTATCAATGCTAGTAAAGAAGAGCAAGCTAAAAACCCTATGCTCATCTGAGT
GGCCGACATTTCAGGTCGGCTGGCCACCAGAGGGAACTTTCAGCCTGCCGGTCATCC
AGGCTGTTAAAGAAAACATAACGGCTCCTGACCCTCTGGGCCATCTGGACCAGGCT
CCCTACATCCTGGTCTGGGAAAATCTAGTAGAAGACCCTCCCGTCTGGCTGAAACCC
TTTATTCACAACCCCCCCCCAGTGCTTCTGTTCCACAGGCCCTGGTCCTGGAGGCCTC
TAAGGAAGAAGACCGGGAGGCACGGGAGAGCCGGAAGAAACCAATACTCCAGGAA
TCTTCTCTATACCCTAGTCTGATTGATTTAGACACTGAAATCTCGCCTCCCCCATATA
TCCCGCCACCCTTCCTCCCGAAGGTACCTCAGGTGTCGTCCGGAGAACAAAGGGGG
AACTCAGAACCCTCGGCTCCACAGCAGGAGGGGGGCCAGCCCAGGAAACTCGCGG
AAGGACCCGAGGGGGTGGAGACTCGTCTGACTACGGGAGCCTAGAGGCCCCTTCGT
CCACTGTTCGAGCGCTTCCTGTCCAGGTCGGGCGGCAAATCCAGACGGGGAACGGA
ATTATCAATATTGGCCTTTCTCTACCAGTGACTTATATAACTGGAAAGCTCAAAACC
CCTCTTTTTCTGAAAAAACCCAAGGCCTTATTGATCTTTTAGACACTATCCTGTTTAC
TCACAATCCTACTTGGGATGATTGTCAGCAGCTGTTACAGGTGCTTTTTACCACGGA
GGAATGGGAGCAAATTCTGTCAGAGGCACGGAAACATGTTCCTGGAGTGGATGGGA
GATCAACCATGCAGCCTTACCTAGTGGAAGAAGAGTTCCCTTCCATGCGGCCGAACT
GAGACTTTGAACAGGCAGAAGGTAGGGAGCGTCTCCAAGCGTATCGCCAGACTCTC
ATGGCCAGCCTTAGGGCAGCCACCAGGAAACCCACGAATTTATCTAAGGTGAATCT
GATAAGACAGGAGCCCGGCAGCCTTCTTAGAGAGGTTAATGGAAGCCTTTAGGCAG
TATACGCCCATGGACCCCCAGGCTGATGTGTCGCGCGCAGCAGTTCTGTTAGCTTTT
GTGAACCAGGCAGCTCCAGATATCAGGAGAAAGTTACAGAAGATAGAAGGGCTGG
GGGAATTGTCAATACAGGATCTGGTGAGGGCAGCTGAGAAAGTGTTTAATAACAGA
GAGACCCCTGAGGAGAGGGAGGAACAGATCAGACGGGAGGAAAGGGAATATAGGG
CTGAAGAAAACTGGAGAAATCAGAAAGAGCTGGCTCAGATCCTTTTGGCGGGGACA
AGAACGGGGCCTGAAGCCCCGAAAACTAAAGACACCCGGTCGGGAGGAAAGGAAA
AACCAGCTAGACCTGCTCTAAAGAGAGACCAGCGCGTGTACTGCAAAGAGCAGGGA
CACTGGGAAAATGAGTGCCCCAAAAGAGAGCTGAAGAGAAAGACTGTGAGAAAGG
AGGAACCTTCCCGGGGACCCACGTCTTATATGCAGGGGAAGAAAAGAGACCTGAAG
AGAAAGACTGTGAGAAAGGAGGAATCTTCCCAGGGACCCACATCTTATATGCAGGG
GAAGAAAAGAGATCTGAAGAGAAAGACTGTGAGAAAGGAGGAACCTTCCCCAGGG
ACCCACGTCTTATATGCAGGGGAAGATAGTGATTAGGGGGGTCAGCGCCTGGCACC
TCTCCCCTAGCCCTGGGTAACTATTAATGTGGAGGGGAAACCGATTGGCTTCATGGT
GGATACGGGAGCCCAATACTCAGTTCTCAATCAAAAATTTGGGCCGATGTCCAAAA
AGACTAGCTTGGTCCAGGGAGGCACCAGGACAGAGATATTACTGGACTACTAAATG
AAAAGTGAACTTGGGAGCCCAATGGGTGTCCCACTCATTTCTGGTGATCCCAGAATG
TCAGGCCCCTTTATTAGGAAGAGACCTATTGGCCAAAGTCAATGCGCAAATTCACTT
TGACTCTGGGGGAATATCAGTCACAGACGGGCTTGGACAACCAATTCATGTTTTATC

CCTGGCGCTGAGAGATGAATACAGACTACATTCACCAAAGCCCCCTGCAGCTGTGG
ATCCTGCTATGCAACAGTGGATTCAGAAATACCCTCTGGCCTGGGCAGAGATAGTGG
GAGTAGGACTGGCTAAACAAAGACTTCCCATTGTTGTTGAATTAAAAGCAAATGCTA
CTCCTATGAGGGTGAAACAGTATCCCATGAGCCAGGAGCCTCGGCAAGGAGTAATG
CCACACATCCAGCGCCTCCTAAAGGCAAGAATTCTCAAAAAGTGCTGGTCCCCATG
GAACACTCCCCTGTTGCCTGTGAAAAAGCCCGGGGGAACAGACTTTAGACCAGTCC
AAGATCTTCGTGAAGTCAACAAACGGGTGAGTGACATTCATCCCACTGTCCCTAACC
CCTACACCCTCCTGAGCAGCTTGCCACCAGACTATGTCTGGTATACAATTTTAGACTT
GAAAGATGCCTTTTTCAGCTTGCCTTTGGCCCCCCAGAGCCAGGAAATGTTCGCATT
TGAATGGGCTGACGAGGACGGCCAAACTGTGGGCAGCTGACCTGGACTCGCCTCCC
ACAGGGGTTCAAAAACTCGCCGACGTATTTCAATGAGGCTCTGGGTGAAGATCCCTG
TGAGTACCGAACCAGCCACCCCGAAGTCGTTCTGTTGCAGTATGTAGATGACCTCAT
GCTGGCCGCTACTACTAAAGAGGTATGCCTAAAGGCCACAGGCGATCTCCTCCAGA
CTCTGGGGACATTGGGGTACCGGGCAAGTACAAAGAAGGCCCAAATTGCTAGACAG
GAAGTCATTTATTTGGGATATAAAATAAAACAGGGGCAGAGATGGTTGACTCAGGC
TATGAAAGAGACTATTCTACGGATCCCCGAGCCAACGACTCCCCGGCAGGTGAGGG
AGTTTTTAGGGATGGTTGGGTACTGCAGGCTATGGATCATGGGGTTTGCTGAAAAGG
CCCGACCTATATATGAAGGAAGCAGAGAAAATAGAGACTGGACTTGGACTGAGCCA
ATGAGGCGGGCATTTCAGGAACTTCGACAGGCGTTACTGGAAGCGCCAGCCCTTGCT
CTTCCGGACCCGGCTAAGACGTTTCAACTGTTTGTGGATGAAAAGCAGGGAGTCGG
GAAGGGAGCCCTGACGCAGCAATGGGGACCGTGGAGACGGCCTGTGGCATATCTCT
CTAAACGACTGGACCCAGTGGCTGCGGGATGGCCACCCTGTCTCTGCATCATTGTGG
CTACTGCTCTCCTTGTCCATGATGCTGACAAGCTGACTTATGGACAGAGCCTCCTGG
TCTACACTCCTCATGCAATAGAGGGGATCCTCAAACAGCCACCGGGTAAGTGGATTT
CTAATGTTTGCTTAACCCGCTACCAGGCCCTGCTGCTGGATGCTCCCCGAATATCCTT
TCAGACACCTTGTTTTCTAAACCCTGCCACTTTGCTGCCGATCCTGGAGGAGGACGG
GCCCCTCCATGACTGTGTTGAAGTGTTAGCTGAGGTAACCGCCATACGAAAAGACCT
CAGCAACTTGCCATTAAAACACAGTGAGCTGATATGGTTCACGGACAGGAGCAGTT
ATATAAAGGATGGACAGAGAAAAGCGGGGGCAGCCATAGTAGATGACACTGGGAG
GGTCATCCGGGCTGAGGCCTTGTCCCCCTGGAACATCCACCCAAAAAGCAGAACTG
ATAGCTGTGATACAAGCACTAGAGAGGGCAAAAGGAAAAAGAGTCACTATTTACAC
TGAGAGCCGATATGCATACGGCACTGTGCACATTCAAGGCCCTATATATAAAGAGC
GGGGGCTTCTGATGGCAGAGGGGAAAGAGATTAAAAATTTGCCTGAGATTCGCAGA
CTCCTGGCAGCAGTCCACTTGCCCCGGGCGGTATCCATAGTACATGTCCCGGGACAC
CAGAAAGGAGAGGATGCCTGGGCCCAAGGGAACCATGCTGCCGATACAGCAGCCTG
TGAGGCAGCTGCTGGAGACTACAGGACCCGTGTACTGACTGTGGGATTGCCACCGC
CAGGGATGGGAACACTTCCCCCCAGCCCCATATACTCCCCCTCTGATTTACGCTGGG
TGCAAGATAATACCACCCATCCTGTTGGCAAAAACGGATGGTATCGGGACCAAGAT
GACAACCTGTTGCTCCCTGCTGACCTGGGCAAACATCTCAGTACCCATTTACATCAA
ACCACCTATTTGGGAGAGAAAAAGACTTTGACACTCCTACAAACGGCACAGCTGCG
GTTTCCCCAACAGAAGAAAACCATCCAGGATATAGTCCACACCTGCAAGGCCTGTC
AGATGATGAGATCGGGAAAAGGACAGCATGCAGGTGTAAGATATCGGGGGGAAAG
GCCAGGACATCATTGGGAGATAGATTTCACTGAGGTAAGACCAGGCATGTATGGGT

ATCATTACCTGCTAGTTTTGGTTGATATGTTTTCCGGGTGGGTAGAAGCTTACCCCAC
TAAAAAGGAGACAGTGGTAGAGGTGGCTAAGAGACTCCTAGAAGAGATTATACCTA
GGTTTGGGCTGCCGGTATCTATCGGCTCTGACAATAGACCTGCATTTGTGAGTAAAA
CTGAGCAGGGACTGGCCTCAGCCCTGGGGACCAAATGGAAGTTACATTGCGAGTAC
AGTCCCCAGAGCTCAGGACAGGTAGAAAGAGTGAATCGGACCCTAAAAGAAACTTT
AACAAAACTGGCAATTGAGACTGGCAGGGACTGGGTGACCCTCCTTCCCTTTGTGCT
TTTTCGGGCGTGCAATATCCCCTATAAACTAGGTCTGACTCCCTTTGAAATTGTATAT
GGAAGTCCACCTCCCATTTGTCCGGTGTCTGAAGGAAAGAGTAAACCACCTCTTTCG
TTACGTGCCTTCCAACAGGAGATGCTAGCTTTGAGTAAGGTGCATAAACATATCTGG
TCACTGATACGGAAAATTTATGAGAGCCAGAATGAAGGGACGATCCCGTCCCATGA
TATAGGACTGGGGAATTGGGTTTGGGTCAAGAGACATAAGTCAAAGACCTTGGAAC
CAAGATGGAAGGGCCCTTATGTTGTTCTCCTTACCACCCCTACTGCCCTGAAGGTGG
ACGGAATTGGACCCTGGATACACTGTAGCCACGCATGGTGGGCCACCCCCGAAGAA
CAAGAGAGGGCCCAAAGAGAATGGAAGCCGATACTGCATCCCTCCAATCCCTTAAA
ACTGAAACTCGCCCAGCAGCTGGTCTCGGACGGATCAACCTGACTCTTCTGATAATA
GCCACCTTCCTGGACCCTGGGACCGTCAGCGCTAACCCTCATCAACCAATGAACCTG
ACTTGGATGATTCTGAGCACAACTACGGGAGAAGTAATTAACTCCACCTCGGCCATT
CATCCCCAGAACACCTGGTGGCCGGACTTGGAGTTCGACCTCTGTTTTTTGGCCATG
GGATCATGGGACATCGGCGACTGGGAGGTAAAGACGCCCGGTAAGCCTGAATGTGG
GGCTGGCATTAATAGGTGTAATACTCGACCCCCCACTAACTCAGGGCCTGGGTGCAG
CCACTTCATCCAATGAGCAAGCTTGCGGAAAACACCCTTCTATGTGTGCCCCGGAGG
AAGGCGGGACCAGGCGACCGTCAATAAATGCGGGGGTGCTAGTGAGTTTTATTGTG
CCAATTGGGGATGCGAGTCAATGGGACAGTCCATTGGGAGCCCCCTATTCGTGGGG
ACTTGATTACCTTGGGTCACATTCCGGGGCCTGCTGGGCTAACTGGGGGACATAGAA
AGGGGTCCCTCATAACTGGGCCTTGTATGAATTTTCTTTGCAACCCGGTAAGGCTCA
AATTCTTAACTGAAGGGAAGAGGTTCCCCAGTTGGGAAGCTGGACAATCCTGGGGC
CTTCAGTTATATCAGTCAGGATATAACAGTGGATTGCTATTCACTGTCAGGTTAAAA
GTAGGACCCATACAGATCGGTCCAACTCGGGGCATAGGGCCCAATTCAGTCCTAGT
GCCACGAGAAGCTACCCACGCTCCCATTCGGCCTGCACACACCCCCGCCCTAGCACC
CACCCAAACGCCTCCCGGGCCCTACATTACTAATACAAGCAACTCCCCTCCAGTTAG
CCGTGCGGGACCTAGCATTAAAGATGAGCAGGACCCATTATTTAATATGATAATCAA
CTCCTATCAGGTTCTAAATTCCACACGCCCGGATCTGACTAGCAGTTGCTGGTTATGT
TATGACATCAAACCCCCTTATTATGAAGGTATAGCTGTCCCAGGAAGTTACAGTCCA
ACCCAAAACCACGCAGCTTGCCGATGGCAGCAGAAAGGAAACGACAGATTAACCCT
CCAACAAGTAACCGGGCGGGGCCTTTGCATAGAAAATGTCCCCCAAACCTATCAAC
ACCTCTGCAAGTCTATAAATTCTACAGTGACAAACAGGTATCTGGAGCCTCCCCAGG
ACAACTGGTGGGCCTGCTCCACAGGTTTAGCCCCTTGTTTTCATGGGTGGGTGTTAA
ATAATTCCAAAGATTTCTGTGTATTGGTACTATTAGTCCCCCGACTGTTCTATCACTC
CAATAATAAAAATCCTCTCAAAACTAGAAGCTTCACACCGATCTAAGAGAGAGCCA
GTCTCAGCCTTAACCCTCACAGTCCTACTGGGGTCAGGGGCAGCCGGGGCCGGAAC
TAGGATATCCTTTTTAATAATGCAGAATCAACACTATTCTAGCCTAAGAGCAACCAT
TGATCCTGATATTGAAAGATTAGAAAGCTCAATCAGCCACCTTGAGAAGTCCCTTAC
TTCACTATCAGAAGTGGTTTTGCAGAACAGGAGGGGCCTAGATTTGATCTTCCTGCA

ACAAGGCGGATTATGTGCGGCCTTAGGAGAGGAATGTTGCTTCTATGCAGACCACA
CTGGGGTGGTGAGAGAGTCTATGGCAAAAGTGAGAGAAGGATTAGCACTGCCCAAG
AGAGAGCGAGAAGCCCAGCAGGGATGGCTCGAGTCCTGGTTCCATCAATCTCCTTG
GCTAACGACACTAATTTCTACCTTGCTCGGACTGCTCATTATACTCCTACTCATTCTT
ACCTTCGGCCCACGTATTTCAAACAGATTAATAACCTTTGCAAGAGAACGTGTCAGT
ACAGTTCAGGTAATGGTATTAAGACAACATTACAGGCAGTGAATGGAGAGGAGGAT
TCCTCTCCATGATCAAAGGACAAGGGGGAAATGTTAAGGCCTGATAGAGGCCATGA
CTAAAATAGGCTGGAGCAAGCATCCCGGAAACAGCTGAGACACTGCCTGGGCAGAA
TCAAAATTAATTACCCATGACGGGAGGACTCAGCCTGTCAGCATGCTCCCCGTAGCC
TGGTCTATGGCAGTCGGGACGAGGATAGATATCTCCTAAGAAAGCCCGCGCGCGTG
AAAATCTAGCCAATTAGTAGATGCAAAGAAACATTTGTAACCAATCCAACCTTGCCA
ATTCCCTGTCTTTGTCCTAAACCTATAAATACTACTGTAATCCAGGGCTCGGGGCTCT
GGCTCTATTCCACTGTGTTGGATGCAGCCAGGGCTCTGGCTCGAGCTAGCAATAAAT
TCCCTTTTTTGCGTTTGCATTGCCGTGGATGTCTTGTTCTCTGAGTTCTGGGGATCCG
GACCTTGGGCATAACA

ERV_3

CCCCGGAAAAGTGCCGCAGACAAGTCCCGGAGGCGGGCCGACAACCAAGCAGTCC
AATCAGGTGCCGACACAGAGCCCTTGGACACCAACCACCGCTGTAGCCCGAGCTTT
CTACCTTATATGGGAGCCTGGCCCTGGCTATAAAACCTTTCCCCACCCCTCATACCTC
GCAGACTCCCTTTGCTTCCTTGCTCACCCACCCCCGGGAGTTCTGCCCGAGAGCGAC
CGCCCAATAAAGGCTCTGATCAACGGTCCATAGAGGTGGCTCTTTCTTCCCGCGGCG
TTTCTTACATCTGGCACCCAAAGTGGGGCTCAAGGTGAGGGCCCCCGGCACTAGCTG
TTGAGGCCCCCTCGAGCTCCACCGCTGCAGTAGCCCCGGACCCAGGCGGCTGACCA
ACCCCCCGGACAGCAGGATACAGGGTAAGTCCCTTCGGCTTTGGGGCCCGCCCTCCC
TGGCGACCACCTCCTAGGGCCCGGTAACGGGTGCGCACTTACTGGGCCCTCCCGGTC
ACCAGCTTGTAGGGGAGACGTCCCCAGTGGCTGAGTAGACCTCCGGCTTCGGCCCTT
CCTGGTCCCCAGCTCGTGAGGAAGACGTCCCGAACAGCTGCACGGACCTCCGGCTTC
CCTTGGCACGTCCGAAGATGTTCGGGCAGCGGGGGTTGCCTCCACGCTCTGTGGTCG
CCATGGGATCGACGGCCTCCAAACCCAATCCCCAATACTCCACCCCCTTAGAGTGCC
TGCTGGCTAACCTGCGGACCCTAAGACTAAAGGGATATATCCGCCCCAAGCAACTC
ACTTTCTGTGTTCACAAGCCTGGCCTCAGTATCCCCTAGATAATGGCTCACAGTGGC
CAACCACAGGGACAATGGACTTTGACGTCCTCCATGATTTAGATAATTACTGCCGAA
GAATGGGAAAATGGTCTGAGGTCCCCTATGTTCAGGCCTTTTGGGCGTTGCGCTCTC
GCCCCACCCTGTGCACCACGTGCGCTCCCAGCCAAATCCTACTTATCATGGCTCCCC
CGATTCCACCCTCCCGGGCCAAACCAGCTCCTCCCGTCTCTGAGTCCTCTGCTTTCTC
TGTTCCCCCGGAAGATCTGGTGGCCCCTCCTCCCTATACCTCTCCCATGGTTCCCACC
CCTTCCACTCTGGTTCCTGCCCCCTCCCCCTCCACTCCGGCCCCTCCTACCTCCGCTT
CCGCCCCCGAGACTCCACCACTGGCCCCGGATCCTCCAGCCCTTAACCCCATCCTGT
ATCCTCCTCTTCCCCCTGTCACTCCCTCCCCTTCTCCGGTTAGCTCCCACACTCGCTCC
CACAGCAACCCTCCGGGAGCCTCCCCTCCCCCTCCCCCAGCCCCGCTACTCCCTCTC
CGACAAGTAGCCGGAGCTGAAGGTCTAGCCCAAGTCCACGTCCCCTTCTCCCTCCAA
GACTTAGCACAGATTGAGGCCAAACTGGGTTCCTTCTCCTCCAACCCCACTCAGTAC

ATTAAGCAGTTTACTGGTCTGACCCGCGCCTACGCCTTGACATGGCAGGACATATAT
GTCATCCTGGGGTCTACCACCACCCCCGAAGAGAGGCAGGCCATCTGGACGGCAGC
CAAGGCTCAGGCCGACCAGCAGCACTTTGCCAACCCCTCCCCTGAGTGCCCCCCGGG
GGCCCAGGCGGTTCCTGACACTGACCCTGATTGGAACTACCAGGAAGGGGGTGGCG
GCCAGCTGCGAGTACGCTATATGATAAAGTGTATCCTCAATGGGATGGAAACGTCCT
CTCATAAGGTTGTAAACCTCCTCAAACTAGATGAGGTGACCCAGGGGCCCGACGAA
AACCCAGCCATATTCCTTAATCGGCTGACCGAGGCCCTCGTCCAATACACCAGGCTG
TCCCGAGTCCCCCATTGGGGCGGCCACCTTGGCCAATCGTTTTATCTCCCAGTCTGCC
CCCGATATCCGAAAAAAGTTGGCCAAGGCAGAGGACGGCCCTCAGACCCCTATCCG
AGACCTGGTAAAAATGGCTCTTAAGGTCTACAATGCCCGCGAAGAAACTGCTGAGG
CCAGCCGAAAGGCAAGGCTCAAGCAAAAGGCCGAATTTCAGGCAAGCCTCCTCAAC
TAGCAAACCCAGGCCTTGGCAGCGGCCCTGCGGCCGGCGGCGGGCTCGGGGCCCCA
AAACCCCCCTCCGGGGGCCTGCTTCAAGTGCGGCCAAGAAGGGCACTGGGCCAAGA
TGTGCCCCAATCCGCGGCCTCCTTCCAAGCCGTGCCCGTTGTGCAAACAACGAGGAC
ACTGGGCTAGTGACTGTCCCCAGGCCTCTCGGGCCTCGACCTCTAGGGGCCAGGGAC
CAGAGTGCCCCAGGGAGACCTCCTGCCCTCCTCCGGCTTTGGAGCTGCTGAGCTTCG
ACGGTGACTGACACCGCCCAGACTCGGGGACCCCAATAACCCAAGCCTAGCCCAGG
GTAACACTCCAGGTAGCGGGTAAGTCCATCAATTTTTTGGTTGATACGGGGGCTACC
TATTCAGTCCTTCCCTCTTTCGGGGGCACTCTACGTCTTTCCCAGGTTTCGGTTATGG
GCATTGACAGCCAACCCTCGTGTCCGCTCCAAACCCAACCATTATCCTGTCAATTAG
ATTCCTGTCTATTTACCCACTCCTTTTTGGTCATCCCCTCCTGCCCTACTCCTCTCTTG
AGAAGAGATATACTTGCTAAGCTGAAGGCTACTCTTCAGCTAGCTCCAGGATCAGCT
CCCACGTCAGGGGCCTTCCTAATGCTACTTGTTGACCCTCCAACCTCTTCTGTTAATC
CTGAGGTCTGGGACACCCGAGTCCCGGTGGTGGCTCAGCACCACCCTCCAGTCCTCA
TCCGGCTAAGGGACCCCACCTGTATCCCAGCCCGGTCCCAGTTTCCTTTGTCTACTCG
CAACCTCAGGGGGCTAAAGCCCATCATCGACCGTCTCATGGGACAGGGTCTCCTGAT
CCCCAAGACCTCCCCCTGTAACACGCCCATCCTCCCTGTTCAAAAGGCTTCAGGAGA
CTACCGGCTAGTGCAGGATCTCCGCCTGATCAACGCGGCGGTGATCCCTGCCCATCC
ACTTGTCCCTAACCCCTACACCCTCCTTTCTTCCATACCTCCTCAGACCTCTCATTTC
ACCGTTACTGACCTCAAGGATGCCTTTTTACCATCCCACTCCACCCGGACTGCCAA
TTCCTCTTTGCTTTCACCTGGACTGACCCCGACACCCAGCTGACCACACAACTCACA
TGGACCGTACTCCCTCAGGGGTTCAGAGACAGTCCCCACTACTTCGGGCAGGCTCTG
TCCCGGGACCTGGCCAGATGCTCGCTTTGCCCTAGTACCCTCCTCCAATACGTAGAC
GACTTGCTCCTTTGCAGCCCCTCAGAAGAGACCTCCCGACAACATACTACAACTCTC
CTCAATTTCCTTGGTTCCCAGGGTTACAGAGCCTCACAATCCAAAGCCCAACTGACT
CAGACTTCTGTCGTCTATCTGGGCCTCCAAATCACTCCGACCACTAAAGCCCTGACA
GCAGAGCGGTGTAGCCTCCTCCGGTCCATCTGCCCTCCGGCCGATGGAGACCAGATA
TTGTCCTTCTTAGGACTGACGGGATTCTTCTGACACTGGGTCCCAAATTATGCTACCC
TGGCCAAACCTCTATATGCTGCAGTTAAAGAGACTCCCACAGGGCCGCTGTCTTCCC
CCACCGAAGTGACTCAGGCCTTCCACGCTTTACGCTCAACCCTATTGGCTGCACCCC
CTCTCTTTCTCCCAAATCCCAACTATCCACACCATCTATACACTGATGAAAAGGGAG
GGATAGCCTTTGGAGCCTTGGTGCAACCGATTGGCCCCGAATTGCTGCCTATTGCTT
ACATATCCAAACAACTTGACCCCACAGCCAGGGGATGGCTCCCCCGCCTGCGGGCA

CTAGCGGCGGCAACAACGTTGTACGCCGATGCAAAAAAGCTGATTCATGGCCAACC
CCTGACCGTCTTCTCGCCCCACCGTCTTGGTGACCTTTTAGCCTCTAGATTTCTTTCTG
AACTCAGCAAGTCCAGGCTCCAACAATTTCACCTGGTATTCCTGGACAATCCTCAGG
TTTCTGTGGGCTGCTCCCCACAATTAAACCTGCTTTCCTCGCTACCCTCACTCCCTGT
CTCCTCAGAACCCCCAGCCCACTCATGCTTGGAGGTCCTTGAGTCCCTTATGCAACC
ACCCCACAACCTGTTCTCCAAACCTCTACCAAATCCAGAGATAACTTTGTTCATCGA
TGGGAGCTCAAAGCGAGACCCCAATGGAAACCGAAGGGAGGCTTATGCTGTGGTAA
CCACCCGGGAAGTCCTAGAAGCTCAGCCCTTGCCACCCGGGACGACTTCTCAGAAG
GCTGAACTAACAGCCCTAACTAGAGCCTTACACCTAGCAGAAGGAAAGAGGGCCAA
TATTTACACAGACTCCAAGTATGCCTTCTTGATTGCCCATTCTCACGCGGCAATCTGG
AAAGAACGGGGCTTCCTGACCACTAAAGGATCCCCCATCTGTAATGCCCCCCACATT
ACTCGACTGTTGGATGCCTTATCCCTGCCCAAAGAGGTCGCTATCATACACTGCAAG
GGCCACCAAAATACCCATGACACTGTCACTCTGGGTAATAATATGGCAGATCAAGT
GGCTCAACAAATTACCTTACAACAAGCTCCAGAGCCCCTGCTGACTTTGAGATCCAC
CCTCAACCCAGATTATTCAAGCAAGGAAGCCAGAGCCTTGCTGGCGCAAGAAGGGG
CTCAGAGGCACCCGTCAGGATGGATACTACTCCATAATAAACCGGTGCTTCCTGAGT
GCCAGACTAAGGCCATAATATCCCAAATCCACAATACCCTCCACATCGGACCAAGG
GCTCTTTTTTCCTTTCTCAGCCCTGTCTTTTCTCCCCTACATCTCAGACAGACCATATA
TGAGGTCCACCGCTCCTGCCTAACATGCACCTCCACCTCTCCTCAAGGAGAACTCCA
ACCCCCACAGGAGACTCACCAGTTGAGGGGACATATGCCCGGGCAGGATTGGCAGA
TAGACTTCACCTACATGCCCAGACATCGAACCTACCGCTGTCTGCTGGTCTTGGTGG
ACACCTTTTCAGGATGGGTCGAGGCCTTCCCCACCGCCCGGGAGATGGCAGCGGCG
GTGGCGGATGTCTTGACAGCCCATCTCATTCCCAGATTTGGATTGCCAAACTCTCTCC
AGTCTGACAATGGGCCTGCATTTATCTCACAGATCTCTCAGCAGGTGGCTGTGGCCC
TGGGTATCGACTGGCATCTCCACATCCCCTATCGGCCCCAATCGTCAGGCAAGGTAG
AGCGGGTGAACGGCATCATCAAGACACATCTGACCAAGCTTGCCTCAGAACTGCGG
CTGTCTTGGGTTGACCTCCTTCCTCTGGCGCTCACTCGCATTCGCACCACACCACACT
CCAAAACAGGTTTGACCCCTTTTGAACAGCTTGACGGCAGGCCCTATCTCCTGACTC
ACCTCCCAGAGGGAGAGGCTCCCCCACTCGCGGGGTACCTCCCCCTCTTCTCCCTCC
TATGATCCTTGCTGAGGGAACACGCAGACCGGGTCCGCCACAACCGACAGACGACG
AGAGGCCCACCCAGCCTCTGGCCCCAGGAGATCAGGTACTGCTGAAAACCCTGAGT
CCCCGCCCACTACAGCCTCGCTGGACGGGACCCCATACTGTAATCCTCACCACTCCT
ACAGCAGCCAAACTCCTGGGACACGAGCGCTGGTACCACCTGACCCAACTCAAACG
AGCTCCCCCCGGTCTCCCAGAGACAGGGGTGGCCCCGGCCCCGACCCCTTCTCCCAA
TCACTCTTACTGCTCCACCCTCACGGGGCCGACCAAACTGACCATCACCCGGACCCC
CCTGTCGTCGATTCCTGAATGACTGAGAGACCACAGTCCAATTCGATCTGATGCTGG
CCTGGCATCACCCGCTGTGGCTGCTGACAATTACAGCTCTGACCATACTCTTTGCCA
TTGGATTAGCGGCCACGGCCCCCCGCGATTGGTCCCTCACTCTTCGACTGTCCCTGG
CCCTCACATACCTAGCCACCTGCTTCCTACTCCTGGGGTGCTATTCCCTTGGGACACC
CTGACCTGGTTACCAGCTCCATCCTTACGGACCCCCGAGGCTGGTTGTCCCCCTTTC
ACCCCAACCTCGGAACGATGCGGGGCTCTCTACTCTGGGTTACTCTGGGGGTCCTGG
AAAAAGGGGGACACACCAACCAAAACCCCCACCAGTCCTTTCAGATCACTTGGACG
CTCCGGAATGGACTGACTCGCGAGGTACTCAATTTGACCACCGGAATTCACCCCCCT

AATACGTGGTGGCCAGATCTGTATTTCAACCTCAAAGACCTTATAAAGACTACTTGG
ACAGCGGCCCAGACCAGAAACTTTGGATTCTGGGCATGTCCCGGACACCTAAAGAC
ACATAATTGGGAGACTTGTGGGGGGCTGCAACATTATTTCTGTTGGTCCTGGAGCTG
TGTGACCTCCAATGATGGGAGATGGAAATGGGAGGTCGGGAACCTGGACCTAGTCA
ATTTCTCGTTTGTCCAGCCCTATCACGGGCCCTGGGAGCAAGATTACCAAAATCTGT
TCGGAGATTATGTCAGGCTGAGTTGGAAGCAGCATTGGAGCCCAACTGCTCAGGTG
AAAGTAATGTTCGACCAGAAAGCGGCTAAACTGGAGAGGTCTTGGGTATCCGGGCT
ATCATGGGGCCTCCAACTCTACGCTGAGTGGTTTGAAGCAAAGCCAGGGGGAATTCT
GGTTGTTAGTCAGACAATAGAACCTGTTCAGGCCCATGCTGTGGGGCCCAACCAGGT
TATTGCACCTCCCCGCCCCTTACCACGGGGGACAAACGCCACAAATGTCGTGACCTC
GCCTGCCCTCACCCCCTCCCTTCAGCTGACCCGGACTGACCCACCAGAGACCGAGGA
ACCCCTGTGGGCCTTGGTCAAAGAAACCTATGGGGCCCTCAACCACTCCAATCCTAA
CGCGACCCAATCCTGCTGACTCTGCTACACCCTACATCCACCTTACTACAAGGCCTC
GGGCTTAAATGCTACCTACAACTTATCCATTCTCTCCAACCCACCGCAGTGCTCTTG
GGGAGACCGCAAGGTGGGCCTTACCATGAAACAAGTATGGGGTTCGGGACCTGCTT
AGGCACGGTCCCTACAGATAAACAAACCTTGTGCACCCAGACTGGCGATGATACCA
ATTTCACCAATAAGACCTACGTCATACCCGAAACCGGGGGGTGGTGGGTGTGCTCAC
AGACTGGGCTAACGCCCTGTCTCCACTTGGCTGTCTTCAACCAGAGCAGGGAATTCT
GTGTCATGGTAGTGGTGGTGCCCAAGATTACATACCATCCAGAAGAGGTCCTCTACA
ACTTTTGGGACTGAGACACCCCAGCTCCTAGACATAAGAGGGAGCCCGTTACAGCT
ATTACTCTGGCAACACTGTTCGCCCTGGGAGCAGCTGGGATGGGCACAGGCATTGCT
TCCCTGACCACACAACATCAGGGCCTGATCACCCTGAGGGCCGCAACTGACGAAGA
CATCGCCCGCATAGAGAAGTCCATGATGGCCCTAGAAATGTCCCTGACTTCCCTTTC
CGAAGTGGTATTACAAAACCGCAGAGGACTAGACCTGGTCTTTTTGCAGCAGGGGG
GCCTCTGTGCAGCCCTCAAAGAAGAATGCTGTTTCTACGCTGATCATACTGGGGTGG
TTCGAGAGTCCATGGCTAAAATAAGAGAGGGATTAGAACGCAGGAAGAGAGAGAG
GGAGGCCTAGCAGGGCTGGTTTGAGTCCTGGTTCCAGAGCTCCCCTTGGCTGACAAG
TTTGCTATCCTCCCTAACGGGACCACTCATCATTCTCCTGCTGCTCCTGACCTTTGGG
CCCTGTATCCTAAATAAGCTTTTGGCCTTTATCAAACAACGGCTCAACATGGTCCAG
CTGATGGTGCTGCGAAAGCAGTACCAAGGGCTTCCAACAAACACTCTGTGACAAAA
TTAACGGCAGACACCCCGTCATCCCAGCCACACCCTACCCCTCGCCACCCCCGTTCA
GCAGGAAGTAGCCAGAGAGGACTGGCGCCCCTTGTCCTATATCAAAAAGGCCGGGA
TGAAAGGTCGGGCATGTTGAGGCATGCCCGGGCGTGTTAAGGCATGCCCCCGGAAA
AGTGCCGCAGACAAGTCCCAGAGGCGGGCCGACAACCAAGCAGTCCAATCAGGTGC
CGACACGGAGCCCTTGGACACCAACCACCGCTGTAGCCCGCGCTTTCTACCTTATAT
GGGAGCCCGGCCCTGGCTATAAAACCTTTCCCCACCCCTCATACCTCGCAGACTCCC
TTTGCTTCCTTGCTCACCCGCCCCCGGGAGTTCTGCCCGAGAGCGACCGCCCAATAA
AGGCTCTGATCAACGGTCCATAGAGGTGGCTCTTTCTTCCCGCAGCGTTTCTCACA

ERV_4

TGTTAGGGACCAAACAACGGTCTCTAGGACCTGAGTCATGTTTACCAGAAAGAGAC
AGGATATGCGCTCATCCTGCCTGGCCAATCATGTAACGCCAGCTACTCCTGTAACGG
AGACAAAGAACTGCCTGTATATAAGCTGCCATACTCCTTTGTTCACGGCTCTTGTCA

GATTCCCTTGTGTGGGATGAGACTTGGGCCCTAGCGCGCTAGGAATAAACAAACTCC
CTTCTTGCGTTTGCAATACTGTGGTGGACTTGCTCTCTCGGTCGGTTTGGAGATACGG
GCTCGGAGCATAACATCTGGGGGCTTGTCCGGGATCTCCGTCCCGCCAGGGAAGAC
AACTCTCCTGGTAGAGGGAGTAACCTCGTTAGGAGATAAGAGCTCTGAGCACCGGT
GCTAATGTTGCAGAAACCCAGATTAAGTCCGCGGCCTGGTATCGTACTAGGGAGGC
ATCTGGCTGTAAAGTGCAGATGCAAGTCAGCGTAAGGCCGGGGCCTGGTTTCGTGCT
GGGTAGGCAGCTGGCTCTGGTTACTGGATTAAGTCAGCATAAGGCTGGGGCCTGGTT
TCGTGCTGGGGAGGCAGCTGGCTCTGTGAACATCCTGCAGGTAAGATTGAGTGCATT
GTCGGTGGCCACCTTGTGTTTGTTATCTGTTTGTCTATTTGTGGTGTCTGCGCTGCTCT
TTGTGTGCTCTGTTGGCTCCCAGTGTACTTTTCTGTGATCATGGGACAAACAACTTCT
ACTCCTTTATCTCTTATGATTAACCACTTCTCTGATTTCAAGTCTAGAGCTCAGAATC
TTTCGTTATTGGTAAAGAAGAGCAAACTGGTGAGTTTCTGTTCTGTTGAGTGGCCCA
CCTTTGACGTCGGATGGCCGCAAGAGGGAACCTTCAATCCCCAAATTATCCAGGCA
GTTAAAGAGAGGGTGCTTACTCCTAGTCCTGCCGGGCACCCAGATCAGATGCCCTAC
ATTCTGGTCTGGCAGGATCTAGTGAGGAACCCGCTGGAATGGCTTAAACCCTTTGTT
CTCGCTCCTCCTAAACCTCCCCGTCCCTCTTCCCCAACTCCAACCTCGCCAAGCCCAC
AGGTGCTAGTAATGAAGGCTTCCAAAGAAAAAGAAGAAAAAAAGGATGAAAATCG
ACCAAAGCTGGTATTCCAGGAATCTTCTCTGTATCCTAATCTGATAGATATGGAGAC
CGAATTGTTCCCACCCCCGTATGCAGATCCACATCTGCCCTTGCTTCCACAGGTTTCA
TTGGGAGAAGCCAGGAGGAGAACCGAGCCTTCAGCTCATCCCAGAGAAGGGGGCCC
CGCCCAGGGAACTCAGGGAAAAAACAAGAGAAATGGCCAACGTAGCGGAAGAAGA
CCCAGAAGTCCCCTCCTCCACTGTTCACGCATTTCCGGTCCAGGCGGGACCAGCCAG
AGAGGGTGGAGAGCGGACATATCAGTATTGGCCCTTTTCCACTAGTGATTTGTACAA
CTGGAAAACCCAGACTCCCTCCTTCTCTGAAAAACCACAGGGTCTTATTGATCTTTT
AGAGTCTATCCTTTTTACTCATAATCCCACTTGGGATGATTGTCAGCAACTGTTACAG
GTGCTTTTCACTACAGAAGAGCACGAGCGGATCCTGTCAGAAGCTCGAAAGAGTGT
GCCAGGGTTGTATGGGAGGCCCACAATACAGCCTAACCTCATTGAGGAGGGATTCC
CCTTAGTGCGACCCAACTGGGACTTTGAATGCGCTGAAGGTAGGGAGCGTCTCCGG
GTATACCGTCAGACTCTTATGGCCGGCCTTAGAGCTGCCGCCAGAAAGCCAACTAAT
TTGGCCAAAATAAATTCAGTGAGGCAGGAACCAAATGAGAGCCCAGCAGCCTTCCT
TGAAAGGATAATGGAAGCTTTTAGACAGTATACCCCTATGGACCCACAGGCAGATG
AGTCACGAGTGGCAGTTATGTTAGCATTTGTAAATCAAGCAGCCCCTGATATTAGAA
AATTACAAAAGATAGAGAGGTTAAATGAACAATCCTTGCAAGATCTAGTGAGGGCA
GCCGAGAGAGTTTTTAATCATAGAGAGACCCCAGAAGAGAGAGAGGACCGCATTAG
AAGAAAAGAAAGAGAATTTAGAGCTGAAGAAAACCGTAAAAATCAAAAAGAGCCA
GCCCAGATATTTTTTGCTGGGGTTGAAAACAAAAACAGGTTCCAGAAAGGAAAAAA
ATTGGACTCAAAAACTGAAGAAAAAATGACAAGGCGTAAGCTTGAGAAAAACCAAT
GTGCGTTTTGCAAAGAGTTTGGACATTGGAAAGATAAATGCCCCAAGAAAAATCTA
AAAGAGGGGCCCAAGAACCCCAAGAACGAGACTCCCTCTCCAGACAGTCATATCCT
CTACGCGGGTGAGGATAGCGACTAGGGGGGTCTGGGCTCAAAGCCCCTCCCCGAGT
CCTGGGTAACTATAAATCTGGAGGGGAAACCGGTTGGCTTCATGGTGGACACGGGA
GCCCAATACTCAGTCTTAAACCAAAAAGATGGACCCATGTCTAAGAAAAGTAGCTG
GGTGCAGGGAGCAACCGGGACTAAACGATATAGATGGACTACAAAACGGCATGTGA

ACTTGGGGGCCCACCAGGTAACCCATTCTTTTCTGGTGATACCTGAGTGTCCAGCGC
CCTTGTTGGGAAGGGATTTACTGTCTAAAGTAAATGCCCAAATTCATTTCGACCACG
GACAAGTGTCAGTTTTAGATGGGACCGGGCATCCTCTTCAGGTCTTGTCTCTGGCAT
TAAAAGATGAATACAGACTCTACTTGCCAGAGGCCCCAGCGACAATAAGCCCCGAA
GTACAACCATGGGTTCAAAGATACCCTCAGGCCTGGGCTGAAACTGCAGGAATGGG
ACTAGCCAAACAGAGGCCCCCTATCATTGTGGAACTGAAAGCCAGTGCTTCCCCGGT
GAGGGTACGACAGTATCCCATGAGTCAAGAGGCTCGACAAGGAATTACTCCGCATA
TACAACACCTCATAGATGCTGGGGTCCTAAAAAGATGCCGGTCCCCATGGAACACT
CCCCTGTTGCCTGTGAAAAAGCCTGGGGGAACTGATTTTAGACCAGTTCAAGATCTA
CGAGAAGTCAACAAATGGGTGAATGATATTCATCCTATGGTTCCTAACCCTTATACA
TCGCTAAGCAACTTGCCTCCAAACTACATTTGGTACACTGTTTTAGATTTAAAAGAT
GCCTTTTTCAGTTTGCCTCTTGCCCCCGCAAGCCAAGAGATCTTTGCCTTCGAATGGC
AGGAAGACGGTGGTCAGTCCCCTGTGCAGCTGACATGGACTCGCTTACCACAGGGTT
TCAAAAACTCGCCCACGTTATTTAATGAGGCCCTGGACGAAGACCTCCGTGATTATC
GGGGTTGAACACCCTACCATTGTTTTATTACAATATGTTGATGACCTTATGCTGGCA
GCGGCTACAGAGAAAGAGTGCCAAGAGGCAACAGGTGACCTTCTCCAAACCTTGGG
GACTTTAGGTTACCGGGCTAGTGCCAAAAAGGCCCAGATTGCCAAGCAAGAGGTTA
CATACCTCGGTTATAAGATAAAACAGGGCCAGAGGTGGCTAACACAGGCTATGAAA
GAAACCATCCTCCAGATCCCTGAGCCAGCTAACCCTAGACAAGTGAGAGAATTTCT
GGGAATGGTGGGATATTGCTGGTTATGGATCTTGGGGTTTGCAGAAAAGGCCAGGC
CCCTATATGAGGGGACCAAAGAAAACAAGGACTGGAAACGGACTGAGCCAATGAA
AGAGGCCTTCCAAGAGCTCAGGCGAGCCTTGCTAGAAGCTCCTGCCCTTGCCCTCCC
TGATCCGTCTAAGCCTTTCCAATTATTTGTAGATGAAAAGCGGGGGATAGGAAAAG
GGGTACTAACACAGAGATGGGGACCATGGAAGCGACCTGTAGCTTACCTTTCCAAG
AGACTGGACCCAGTGGCAGCCGGATGGCCACCTTGCCTCCGTATCATCGCGGCCACC
ACGCTCTTAGTCCACGATGCTGATAAACTGACTTATGGACAGAGACTCTTGGTCTAC
ACTCCTCATGCCATAGAGAGAGTTTTAAAGCAGCCCCCAGGTAAATGGATTTCTAAT
GCCCGCTTGACGCACTACCAGGCCTTGCTACTTGACACCCCATGGATTCATTTCCAA
ATGCCCTGCACTCTAAATCCGGCCACTCTTTTGCCCAATCCGGGGGAAAATAGCCCC
CTCCATGATTGTGATGAGATACTGGCCGGGGTAACAGCAATGCGAAAGGACTTAAC
CGATACTCCACTGGATAACAGTGAGCTGAAATGGTTCACAGACGGCAGCAGTTATG
TAAAAGATGGACAGAGACGGGTCGGAGCCGCAGTAGTAGATGACTCTGGACGGACG
ATATGGGCAGAGGCCCTTCCCCCGGATACCTCAGCACAAAAGGCAGAGTTAATTGC
CCTGATTCAAGCATTAGAGAGAGCCAAAGGAAAAAGAATAACTCTTTTCACTGACA
GTCGCTATGCTTTTGGCATGGTACACATCCAGGGCCTGATATATCGGGAACGAGGGT
TTTTGACAGCTAAAGGAAAAAAAATTAAAAACTTGCCTGAAATCCGTAGACTTTTAG
AGGCTGTACAGATGCCTCGGGCTGTGTCAATAGTACACGTACCTGGACATCAGAAG
GGTGACAGCCCCACGGCACAAGGGAATCGTGCCGCAGACCTGGCAGCTCGAAAAGT
AGCTGATAAAGATTTCATCACCCCTGTGTTGGTGATCGGACTTCCACCTCCAGGTAT
GGGAACTCTGCCCCCAACCCCTGTGTATTCATCCACAGACTTTGCTTGGATCCAAAA
ACGCACCAACCTTCAAAAAGATAAAGATGGATGGTACCGAGACTCAGACGGCTACT
TGATACTCCCTGCTCAGTTGGGACGGCAACTATGTGAGCATTTGCACTTGTCTACTC
ATCTGGGAGAAAAGAAGACTCTGATGCTCTTTCAAACTGCGCGCCTGCGATTTCCCC

GGCACCAGACAACTGTAAAGAACATAGTGCATGCTTGTAAGGCATGTCAACAGATG
AGGCCAGGAAAAGGACAACATGCAGGACTGAGGTATCGGGGAGAAGGACCAGGGC
AACACTGGGAAATAGATTTCACCGAGGTAAGGCCAGGCAAGTATGGTTACCGGTAC
TTGTTAGTGTTGGTGGATACCTTCTCAGGGTGGGTGGAGGCTTTTCCTACAAAGGGA
GAAACCGCGATGATAGTGGCAAAAAGATTTTAGAAGAAATAGTTCCCAGGTTTGGC
CTGCCAGTGACCATCGGCTCTGATAATGGACCTGCTGTTGTGAGTCAAATAGTTCAG
AGCCTTGCCCTAGCCCTGGGGACTAAATGGAAGTTACATTGTGAATACAGTCCACAG
AGCTCAGGGCAAGTAAAAAAAAATGAATCAGACTCTAAAAGAAACTTTAACTAAATT
GGCTATAGAGACTGGCGGGGACTGGGTGACCCTCCTTCCCTTCGCCCTCTTCCGTGC
GCGTAATACTCCTTATCAACTTAATCTGACCCCATTTGAGATTCTGTATGGGAGACCT
CCCCCTGTATGTCCAATATTTGAAGGGAAGAAACTACCGCCTCCCACGTTGGGGCAA
CTCCAAGAGGCCTTGATGGCCTTAAGCAAGGTGCACTCTCGTGTCTGGAAACTGCTC
CAGGAAATACATGTGGGTCAAAATAAGGGAACTATTCCCTCACATGACATTGGCCC
AGGAGACTGGGTATGGGTCAAAAGGCACCAAACCAATGCACTAGAACCCAAATGGA
AGGGTCCTTATGTTGTTCTTCTTACCACCCCAACTGCCCTAAAGGTTGACGGTATCGG
GCCTTGGGTGCATTGCAACCACGTACGCCCAGCTGCTTCAGCAGAGCAAGAAGACG
CTAAGAAAAAATGGGAAGCATCTCTGCACCCATCCAACCCCTTGAGGCTAAAGCTT
CGAAGGCGCCAATAGGACCAGGACAACTCGGCTGGGCCCTCTTGTGGATGACCCAG
TTACTCTGCTCCGGAGTTGCCAGCGTGAACCCGCATCAACCCATCAAAATCACCTGG
AAGCTGCAAAATGGACTAACACGAGAGGTGCTAAACTCCGCTACTGCAATACATCC
ACCAAACACATGGTGGCCAGACTTGTACTTTGACCTTAAGCCGATGGTAAATGTGCC
TTGGGCTAGGGGTTATCTCCGAGAACAAGGGTTCTGGGCATGCCCAGGCGCACCCA
GGCATGACTGGAAGACCTGTGGGGGGGCACAAGACTCTTATTGTAAAACTTGGGAT
TGTGTTACTTCTAATGAGGGACCTCGGCACTGGGAAGTAGGAAATCGAGATTTACTT
AATTTTTCATTCGCCAAGCCCCTCCCTAGGGTCCTCGGAGATCCAACATTTAGCTGTG
AAAGTTGCAATTATGCACAAGTCAGAATAAGGTTCAATCCAGAAAAAAGCAAAAAA
GAGGGGACCTGGATCTCTGGCCTATCTTGGGGGCTACAGACGAGGGAATCAGGATG
GTTTGGGGTTGATGGGAAAGGGATTATAGTAGTGAGCCAGGTCTTAGAGCCAATTCT
TGTACACAGTATCGGGCCCAACAAAGTAAAAAAAGCTGGCTGTAACCCCCCGCCTA
ACGACTTCCATGCCAACATCTTTGGCAGTGAGTCCCGAAGCAGAAGCACTCGCTGA
AATAGATCCCCTATGGAAGCTGATTAGGGCAGCTTATGCCACCCTGAATCAGACCCA
TCCTGAGGCAACTAAATCTTGTTGGTTATGTTCCAACTTAATTCCCCCTTATTACGAA
GCAGTAGGCCTCAATGCCTCTTATGACTTGGCCAACAGCACCGATCCTCCTCAGTGT
CACTGGGGGGACCGAAAGGTGGGTCTGACGATGAAAGAGGTATGGGGAAAGGGCT
TATGCATGGGCACGGTGTTACCAGCAAAGCCTCCACTTTGTGTGCATGTCGTCGAGC
CTGATGATTTGCCTGTAGCCAAATGGTTAATACCTCAAATGGGGAGATGTTGGGTCT
GTTCACACACGGGGCTAACTCCATGTCTGCATAGCTCAATCTTTGACCCCAAAGAAG
AATTCTGTGTTATGGTGGCTGTCATGCCAAAGATTCTGTACCGACCAAAAAAAGCAA
TATATGATTATTGGGCCCAGAAATTAACTCTAATTCCTCCAAAAAGAACTTACAAAG
TTAAGAGGGAACCTCTTACCACCATAACTATAGCAACTATGTTCGGTCTTGGAATAG
CCGGGGCTGGAACCGGAATAACAGCTCTGTCCCTGCAAGGCCAAGGATTTAACTCC
CTGAGAGCGGCCATAGATGAAGACATTACCTGTATAGAGCAATCTATTAGTCATTTA
GAATCGTCTCTAACTTCTCTATCTGAAGTAGTTCTGCAAAACAGGAGGGGATTAGAT

CTGCTTTTTCTGCAACAAGGGGGACTCTGTGCTGCCCTAGGAGAAGAGTGCTGTTTC
TTTGTGGATCATACAGGAATAGTTAGAAAATCTATGGCCAAAGTGAGAGAAGGACT
AGCCCAACGTAAACGAGAACGTGAGGCTCAACAGGGATGGTTTGAATCTTGGTTTC
AACAATCCCCTTGGCTGACTACCTTAATCTCCACCTTGTTAGGACCCCTGCTAGTACT
ATTACTAATGCTTACCGTCGGCCCATGTATTATCAATAGACTTGTAGCCTTTGTAAAG
GAACGCATAAATACAGTACAGCTGTTTGTGCTTCGACAACAATATCAAACTGTGTCT
CAGGACCGAGAGGAAGATTCCTCTATATGATCTAAGGACAGGGGTGGAATGTTAGG
TACCAAACGACGGTCTCTAGGACCTGAGTCATGTTTACCAGAAAGAGACAGGATAT
GTGCTCATCCTGCCTGGCCAATCATGTAACGCCAGCTACTCCTGTAACGGAGACAAA
GAACTGCCTGTATATAAGCCGCCATACTCCTTTGTTCATGGCTCTTGTCAGATTCCCT
TGTGTGGGATGAGACTTGGGCCCTAGCGCGCTAGAAATAAACAAACTCCCTTCTTGC
GTTTGCAATACTGTGGTGGACTTGCTCTC

ERV_5

TGTTAGGGCCAGCGCCAGGACAGGCTCATCAGGCCACACTAGGCCTAAAACCTGGG
TCATGATTCCAGGAACTACACACCCGAGCCAATCAAGAGGGCCCCCGTCTGAGCCA
TAGTTGAGCCAATCCGGATAGAGATGCAGGATTCAAAATTCGTGGGTGCGCGTACG
GCTCAGCCAATCACCACATGCCAACCACACTATTGCTGAGACAAAGGACCGCCTGT
ACATACGCAGCTATGATTCAGAGCTCGGGGCTCTCATCCAGACTCCACTGCGCTGGA
TGAGACTTGAGCCCTAGCTCGAGCTAGCAATAAACCCCTTTATGCTTTTGCATTGCT
GTGGATGTCTTATTCTCTCAGTTTTGGAGACTCGGACACTGGGTATAACATTTGGGG
GCTCGTCTGGGATGCCTTTTGACTGAGGAGAAAAACACCCTCCTGGCAGAAGGGGTT
CCTTAGCTCAAAGGGGACTAAACGTAGCCAGTATAAGGCCGAGGCCCTGCATTGTG
CTGGGAAGGTGGTTGGCTCTGTAAGGGGACTAAACATAGCCGGCGTAAGACCGAGT
CCCGGCATCGTGCTGGGAAGGCGGCTGGCTTTGTGGACGCCTTGTTGGTAAGATTAC
CTCGAGGGAAAGAGAGTTTCAGGGGGGCCCAAGAAGACCTAGTGCTGTGTTCTTGG
CCAATGTGTATTTGTTATCTGTTTGTAGTGTCTGTGTGAGTGCCGGCATAGTCTCTGT
TCTTTATGTGTTCATTTTGTCTCCTGTGTTCTCTGTAATCGTGGGGCAAGCGACTTCT
ACTCCTTTGTCCCTTATGACTGACCACTTTTCTGATTTTAAGTCTAGAGCACAGAATC
TATCCGTGTTGGTAAAGAAGAACAAACTAATGACTTTTTGTTCCGCCGAGTGGCCTG
CCTTTAATGTCGGCTGGCCACGAGAGGGAACCTTCTGCCTACCAATTATTCGAGCAG
TTACAGAGAACATGTTCGCTCCTGGCCCTTCTAGACACCCAGACCAAACTCCCTATA
TTTTGGTCTGGCAAGATTTGGTGGGAAATCCGCCAGCCTGGCTGAAACATTTTATTC
CCCAGCCCTTTACTTCCCCTTCCCCCACTTCGTCCATCCCACAGGTACTTGTTGTGGA
AGCATCCAAAGAGGAAGAGCACAAACAGTGCAACGGCTGGGTGAAGCCAGTATTCC
AGGAATCCTCACTCTGTCCTAACCTAATTGACCTGGAAACCGATCTCTCCCCACCCC
CCTATGCGCATCCACCTTTGCCCCCTCAGGTACCTCAGATTTCATCCAGAGGAACAC
GAAAGGATACCGAACCCTCGGCCCCAACTCAGGAGGGGGGCCCCACCCAGGGAACT
CGAGGAAGAACTAGGGAACCCTAAATGTGGCGGACGAGGACAGCCCAGAAGCCCC
ACCTCCACAGTCCGGGCACTTCCTGTTTGGGCGGGGCCAGCCAGCCCGGTAGGGGA
GCAAACTTACCAATACTGGCCCTTTTCCACTAGTGATCTATATAATTGGAAAACTCA
AACTCCTTCATTCTCGGAAAAGCCTCAGGGCCTCATTGACCTTTTAGAATCCATTCTC
TTCACCCATAACCCCACTTGGGATGATTGTCAGCAGCTGTTACAAGTACTCTTCACC

ACAGAGGAACGTGAACGGATATTGTCAGAAGCACGGAAAAATGTTCCAGGGGTGGA
TGGGAGACCTACCACGCAACCGAATCTGATTGATGAGGGATTCCCCTTGACGAGGC
CTGGATGGGACTTTGAGCATGCTGAAGGTAGGGAGTATCTCCGAATGTACCGCCAG
ACTCTTATGGCTGGTCTTTGAGCAGCCGCCAGGAGACCAACGAATTTGGCTAAGGTA
AACCTGGTTAGACAGGAGCTGAATGAGAGCCCAGCAGCCTTCCTTGAGAGATTAAT
GGAAGCTTTTAGGCAATATACCCCCATGGACCCACAGGCCGACGAGTCACGTGTGG
CAGTTATGCTAGCATTTGTGAATCAAGCAGCCCCAGATATCAGGAGGAAACTGCAG
AAAATAGGTTAGGGGAACAATCCATACAAGACCTGGTGAGGGCAGCAGAGAGGGTT
TTTAACCATAGGGAAACTCCAAAAGAAAGGGAAGAACGTGTTAGATGGGAAGAGA
GAGAGTTCAAGGCCTAGGAAAACCACAGAAATCAGAAAGAATTAGCCCAGATATCC
TTTGCAGGGGTGAGAAAGGGAGCTGATTCTCAAGGGACTAATGAAGTTAGGCTGAG
AGACGAAGAAAAGCTAGTCGGACAGAGACTAAACAAAGACCAATGTGCACATTGCC
AGGAGTGAGGACACTGGAGAAGAGAATGCCCTAAAAGAAAGTTGGGGGGAAAGCC
CACCAGGAAAGAAGCACCTTCCCAGGGGGCCCACGTCTTATATGCAGGAAACGATA
GCGATTAGGGGGGGTCAAGGCCCGGCCCCCCTCCCCGAGTCCTGGGTAACTACATG
TGGAGGGGAAACCATTCTGCTTCATGGTGGACATGGGAGCCCAATACTCAGTCCTTA
ACCGAAAAGACGGACCGATGTCCAAAAAGACCAGCTGGGTACAGGGGGCCACCAG
AACTAAACGATATGGATGGACTACTAAGCGTCAAGTGGACTTGGGGGCCCAACAAG
TGTCCCACTCATTTCTCGTGATACCAGAATGCCCAGCTCCCTTACTAGGAAGGGATT
TATTATCTAAGGTCAATGTTCAGATTCACTTTGACCACGGAGGGATATCAGTTATGG
ATGGGACCGGATACCCTATACAAGTTTTGTCCTTGGCACTGAGGGATGAATATAGAC
TATACCAGCCGGGGCCTCCCATGGCTATTGACCCTAATGTGCAACCTTGGGTCCAAA
AATACCCCCTGGCCTGGGCAAAAACAGCGGGGGTAGGGCTAGCCAAGCAGAGACCC
CCCATCTTTGTTGAACTGAAAGCAGATGCCACCCCTATACAGGTAAAACAGTACCCC
CTGAGTCTGGAGGCCCAATGAGGAATCATGCCACATATCCAGCGGCTCTTGAAGGT
AGGGATTCTCAAAAGGTATCGATCTCCGTGGAACAATCCCCTGTTACCAGTGAAAAA
GCCTGGCGGAATGGACTTTAGACCAGTCCAGGATCTTCGTGAAGTCAACGAATGGG
TGAATGACATTCATCCCACCGTCCCTAACCTGTACACCCTCTTGAATGGCCTGCCAC
CAGACTATGTCTGGTATACAGTCCTAGACTTGAAGGATGCTTTTTTTCAGTTTGCCGC
TGGCCCATTCGAGCCAAGAGATCTTCGCTTTCGAATGGACCAAGGAGGGCAGCCAG
ACTACAGGACAACTAACCTGGACTCGCCTCCCTCAAGGCTTCAAAACTCACCAATGC
TATTTAATGAGGCTCTGGGTGAAGACCTCCGTGAGTACTGGGCTAACCACCCCAATG
TTGTCTTGTTTCAGTATGTGGATGATCTTATATTAGCCGCTGCTACTGAGGAGGCATG
CCTAGAGGCGACAGGCGACCTCCTCCAAACTTTGGGGACATTGGGCTACCAGGCTA
GTGCAAAGAAGGCCCAAACTGCTAAACAGGAGGTCACCTACTTAGGATATAAGATA
AAGCAGGGGTGGAGATGGCTGACACAGGCTATGAAAGAGACCATTTTGCAGATCCC
TGAGCCAGCAACTCCTCATCAAGTGAGAGAATTTCTTGGGACTGTTGGGTATTGCAG
GCTATGGATTTTGGGGTTTGCGGAAAAGGCCCGGCCATTGTATGAAGGGAGTAGGG
AGAGTAAAAATGGGACTTGGACTGAGCCAATGAGACAGGCATTTCAAGAACTTCGG
CAGGTGTTGCTGAAAGCCCCGGCCCTTGCCCTCCCTAACCCATCTAAGCCCTTTCAA
CTGTTTATGGATTAAAAAACAGGGAGTAGGAAAGGGAATCTTGATGTAGCAATGGG
GGCCTTGGAGGCAACCTGTAGCCTACCTCTCTAAAAGACTGGACCCGGTGGCCACA
GGGTGGCCACCCTGCCTCCGAATCATTGCCACCTCTGCTCTCCTGGTCCATGATGCT

GACAAGTTAACATATGGACAACAGCTCCTGGTCTACACTCCCCATGCCATTGAAGGG
ATTCTCAAACAGCCACCAGATAAATGGATCTCCAATGCCTGCTTAACCCATTACCAG
GCCTTACTGCAGGATACCCCCCAGATACACTTTCAGACGCCCTGCTTTCTGAACCCG
GCCACTCTCCTGCCTGTCCCGGAGAAAGACAGCCCCCTCCATGATTATGGTGAGATA
TTGGCTGATGTGATGGCCATACGAAAAGACCTAAAAGATGTGCCCTTAAAAGACAA
TGAACTGGTATGGTTTACAGACGGAAGTAGTTTTGTAAAAGATGGACAAAGAAGGG
CAGGGGCAACCATTGTGGATGACTCTGGAAGGGTCATCTGGGCTGAAGCTCTGCCCC
CTGGGACATCCGCCCAAAAAGCAGAATTAATAGCCTTGACACAGGCACTAGAGAGG
GCAGAAGGAAAAAGGATCGCCATTTATACCGACAGCTGGTATGCATTTGGAACAGT
GCATATTCAGGGCCCAATTTATAGAGAGCAGGGGTTTACAACAGCAGAGGGAAAAG
AAGTTAAAAACCTACTCGAGATCCTCAGACTCCTAGCAGAAGTGCACCGGCCCCGA
GCAATGTCCATAGTACATGCCCCAGGACACCAAAAAGGAGAAGATATCAAAGCTCG
GGCAACGAGGCTGCCGATGTGGCGGCCTGGGAAGCAGCCCTTGGAGACTGCAAAAC
CCCCATATTGACTGTGGGATTGCCACCCCTGGGAAAGGGAACCCTGCCCCTGACCCC
CAAGTATTCCTCTTCCGATCTAAGTTGGATTCAAGAGAATGCTAACTGTCCAGAGGG
CAAAGACGGATGGTATCGAGACCAAAATGGCAACTTGTTGCTTCCGGCTAACTTGG
GTCGACACCTTTGCACGCACCTATATCAGACCATCCATCTGGGAGAAAAAAAAGAC
TCTAGCACTCTTACAGACAGCGCATCTGCAGTTTCCCCGACAAAAGGCAACTATACA
AGACATAGCCCGTGCCTGTAAGGCATGCCAGATAATGAGACCAGGAAAAAGACAAC
ATATGGGTATAAGGTACTGGGGAGAAAGGCCAAGACAACACTGGGAGATAGATTTT
ATAGAGGTAAGGCCAGGCAAGTATGGGTACCGTTACCTGTTAGTTCTGGTCGATACT
TTCTCTGGGTGGGTGGAAGCATATCCCACTAAGAGGGAAACAGCAACAATGGTAGC
CAAAAGGCTCCTAGAAGAGATAGTGCCTAGGTTTGGGCTGCCAACAACCATCGGCT
CCAATAATGGACCTGCTTTTGTGAATCAGATTGTTCAGGGACTGGCCTTAGCTCTGG
GGACCAAATGGGAGCTACATTGCAAATACAATCCCCAGAGCTCAGGACAGGTTGAA
AGAATGAATCGGACTCTAAAAGAAACTTTGGCAAAACTGGCAATAGAGACTGGCGG
AGACTGGGTAACTCTCCTTCCCTTCGCTCTCTTCCGAGCGCATTATACCCCCTACAAG
TTGAACCTAATCCCCTTTGAAATTGTTTATGGGGGGTCCCCTTCAATGTGTCCTATCC
TTGAGGGAAGAATCCAGCCACCTCCTTCATTGTGGCAATTCCAGGAAGCCCTAATGG
CCTTAAGCAAGGTGCATGTGCACATCTGGACTTCAGTCAAAGAAATCCATGAAGGC
CAAGAAAAGGGGACAATTCCTTCACACAACATTGGTCCGGGGGACTGGGTTTGGGT
CAAACGGCATCAATTCAAGACATTAGAACCTAGATGGAAGGGCCCTTATGTTGTTCT
TACTACCCCTACTGCCCTGAAGGTCGATGGCATTGGACCTTGGGTGCACTGTAACCA
CGTGTGCCATGCCACTACGGAAGAACAGGAAAAGGCCCAAAGAGAATGGAATGTG
ACACCACACCCCTCCAACCCTTTAAGGATGAAGCTCATCCATTGACAGGACCCAGAC
GAATCACCCTGACCTTCCTGTTGGTGACCATCCTCCTCGACCCGGGAGCCACCAGTG
GAAACCCACACCAGCCAGCCAAAATCACCTGGAAACTTCAAAATGGACTAATGCAA
GAGGTGCTTAACTCAACCTCAGGAATACATCCCCCAAACACCTGGTGGCCAGATCTG
TACTTTGACCTTAAAGAGGTAGTAGGCGTACCCTGGGCGAAAGGTCTCCTCCGACAT
TATGGGTTCTGGGCATGTCCTGGCCACCAAAGGAGTAACTGGAAGACTTGTGGAGG
TCTACAGGACTACTTCTGTAAGTCCTGGACTTATGTTACTTCTAATGATGGGAATTGG
CATTGGGAAGTAGGAAACCGAGACCTACTCAACTTCTCGTTCACAAGGCCCTCGGCC
CAAGCCCTCGGAGACCCATACTATAACTGTGACGATTATGACGATGCACAGGTTAA

AATAAGGTTCAATCCAGAGGCTGCGAAAAAAGAGAGGTCTCGGGTTTCCGGTCTGT
CCTAGGGAATACAAACAAGTATCAAATGGACCCACGGGTATTTTGGAGGAATAATA
ATTGTCAGCCAGATCATAGAGCCAACACAGGTGCATAGTATAGGACCCAACCCAGT
CGAAAAGACTGATTCACTAGAAATAGCTGAGACATATCTGACACCGAGTCTTGCTAC
ACTAGATTCTACATCCTCCAGTTCCCTCGGTCCCCCAGTCCCTACCCCCAACATCGG
GAAATCAGACCCATTATGGAAGCTTGTTAAGACAGCCTATACAACACTAAACCAAA
CCAACCCAGAAGCAACTGAATCCTGTGGGCTCTGCTACACTCTATACCCTCCTTATT
ATGAAGCAATAGGCTTAAATGCTTCTTACAGCCTGACCACTAGCATAGATCCACCTA
AATGCCACTGCAGAGAATGAAAAGTGAGCCTCACAATGAGAGAAGTATGGGGAAA
AGGACTTTGTGTAGGTAAAGTCCCACCAGAGAAATCCCCTTTGTGTGCTCGGTCAGC
CAAGCTCACAGAGATAGATGAAACAAAACGGATTATACCAGAAGCAGGGGGTTGGT
GGGTCTGCTCTCATACCAGACTGACTCCATGCTTACATGTCTCAGTTTTTAATCAAAA
TAGGGAGTTTTGCGTATTGATGGCTGTTGTGCCCAAAAATCTTATACCACCCCGAAG
AAGTTATATATAGCTATTGGACTGAAAAAATAACAAATCAGCTGGCAGGGAACAGA
GTTACAAGAGAGCCCATTACAGCCATCACATTAGCAACCATGTTCAACCTTGGGGTT
GTCGGAGCAGGAACCGGAATAATGGCCTTGTCCCTCTAAGGCCAAGGGTTTACTTCA
TTGCAGGCAGCTATAGATGAAGACATCACTCACCTAGAAGAGTCAATTAGTCACCTA
GAAAAATCATTGACTTCCCTGTCCGAGGTAATTTTACAAAACCAGAGGGGGTTAAAT
CTGGTTTTCTTGCAGCAAGGAGGACTCTGCGCTACTCTGGGAGAAGAATGTTGTTTT
TACGCAGATCATACCAGGGTAATGAGAGAATCCATGGCCACAATAAGGGAAGGATT
AGCCCAACGAAAAAGAGCGAGGCCCAGCAGGGATGGTTTGAGTCTTCGTTCCAACA
ATCTCCATGGCTAACAACACTGATCTCCACCCTGGTGGGGTCTCTTATGGTGCTATTA
TTAATACTCACCTTTGGCCCATGTATCCTAAACAAACTTGTAATTTTTGTGAAAGAAC
GTATTAATACAGTTCAACTACTCGTACTCAGGCAACAATACCAAGCCTTGCCCCAGA
ACAAAGAGGAAGATTCCTGTATATGATCAAGAGACAGGGGGGAAATGTTAGGGCCA
GTGCCAGGACAGGCTCAGCAGGCCACACTAGGCCTAAAACCTGGGTCATGATTCCA
GGAACTACACACTCGAGCCAATCAAGAGGGCCCCCGTCTGAGCCATAGTTGAGCCA
ATCTGGATAGAGATGCAGAATTCAAAATTTGCGGGTGCGCGTACAGCTCAGCCAAT
CACCACATGCCAACCACACTACTGCTGAGACAAAGGACTGCCTGTATATACGCAGC
TATGATTCAGAGCTCGGGGCTCTCATCCAGACTCCACTGCGCTGGATGAGACTTGAG
CCCTAGCTCGAGCTAGCAATAAACCCCTTTATGCTTTTGCATTGCTGTGGATGTCTTA
TTCTCTCAGTTTTGGGGACTCGGACACTGGGCATAACA

ERV_6

GGACAGTAGCTCCTGCGTTAAACATTCCATAACCAAATAAGGAGGTCAGTAGCTCCT
GCGCTAAACATTCCATAACCAAATAAGGAGGTCAGTAGCTCCTGCGCTAAACATTCC
ATAACCAAATAAGGAGGTCAGTAGCTCCTGCGCTAAACATTCCATACCAAATAAGA
AAAATCTATAGGGAAGCAGCATCAGGGGAATGTATGAGCTCAGCTTCGCATGTAAC
CAATCAGGTAGTGCCAACTATGCCTGTTGCTGGACTAAGGGACAAACTGTATATAAA
CCGCCATACCTCTTTGTTCGGGGTCCAGCCGCATTCTGCTGTGTCGGAGAGGCTAGG
ACCCTGGCGCGCCAGAAATAAACTCCCTTTATGCCTTTTGCATTACTTTGGTGGACTT
GTTCATTCGGTCGGGTAGGGGACACGGACAAGGAGCATAACATTTGGGGGCTCGTC
CGGGATCTCCGCCCCACCGGAGGAATAATTCCCCTGGTAAGAGGGAGAAGCCCCGC

TGACAGGCAAAGAGCTCTGAACTCCGAAGCACCGGTGCTAATAGTACAGGCCTAGA
TTAAGTCCGGGGCCCAGTATCGTACTGGGGAGGCATCTAGGTGTGAGAGACAAGGC
AAGCCCAGCGTAAGGCCGAGTCCCCGGTAGCGTACCGGGAAGGCGGCTGGCACTGA
GGACGTCCTGACGGTAAGACATTTGCAAATAGAAAAGGGGTCTCTAGTGCTAAAAG
AGACTGAATTGGAATCTGTTTGTTGTGTCATTTTTGTGACTCTGTGTGCCGGCACTGT
CTGTGTGAGTGTCTTGTTGTCACTGTCTGTGTTCTCTGTGCCCATGGGGCAATCTACT
TCTACTCCGCTGTCCCTGATGACTGACCATTTTTCTGATTTTAAGTCTAGAGCTCGGA
ATCTTTCGTTACTAGTAAAGAAGAGCAAACTGGTGACGTTCTGTTCCGCCGAATGGC
CCACCTTTGATGTCGGATGGCCACAAGAGGGAACCTTCAATCCCCAGATCATCCAGG
CAGTTAAAGAGAGGGTGCTTACTCCTGGTCCTGCTGGGCACCCAGACCAGACTCCCT
ACATTCTGGTCTGGCAGGATCTAGTAAAGAACCCACCGGAATGGCTTAAACCCTTCG
TTCTTGCTTCTCCTAAACCTCCCCGTCCCTCTTCCCCGGCTCCAACCTTACTAAACCC
ACAGGTGCTAGTCATGAAAGCGTCTGAAAAAACGAAAGAAAACCAGGACGAAAAA
CGACCCAAGCCGGTGTTCCAGGAATCTTCCTCTCTGTACCCTAATTTGATTGACTTGG
AAACCGAACTGTCCCCACCCCCGTATGCGGATCCAAATCCACCTTTGCTTCCACAGG
TTCCTCAAGTCTCGTCGGGAGAAGCCCAGAGGAGGGCCGAGCCCTCAGCTCCCCCT
AGGGGAGGAGGCCCCGCCCAGGGAACTCGGGAAAGGGCAAGGGAGATGGCTAGCG
CAGCGGAGGAGGAAGGCCCGGAATTGCCCTCCTCCACTGTTTACGTGTTTCCGGTCT
GGGCGGGGCCAGCCAGAGAGGGTGGGGAACGCACATATCAGTACTGGCCTTTTGCT
ACTAGTGATTTGTACAATTGGAAAACCCAAACCCCCTCTTTCTCTGAGAAACCTCAA
GGTCTTATTGATCTGTTAGAATCTATCCTCTTTACTCACAACCCCGCTTGGGATGATT
GCCAACAACTGCTACAAGTACTTTTCACTACAGAAGAGCGTGAACGGATCCTGTCAG
AAGCCCGGAAAAATGTGCCGGGGGCAGATGGGAGGCCCACAATACAGCCTCACCTC
ATTGAAGAGGGGGTCCCCTTGGTGCGACCCAACTGGGACTTCGAACGCGTTGAAGG
TAGGGAGCGTCTCCGAGTATACCGTCAGATCCTCATGGCTGGCCTTAGAGCGGCCGC
CAGAAAACCAACTAATTTGGCCAAGGTAAATTCGGTGAGGCAAGAGCCCAATGAGA
GCCCGGAAGCCTTCCTTGAAAGGATAATGGAAGCTTTTAGACAGTATACCCCCATGG
ACCCCCAGGCAGATGAGTCTAGGGCAGCAGTCATGCTAGCATTTGTAAATCAGGCA
GCCCCCGATATTAGGAGAAAGTTACAAAAGATAGAGAGGCTCGGTGAACAGTCCCT
ACAAGATTTAGTGAGGGCAGCAGAGAGGGTTTTCAATCATAGAGAGACCCCGGAAG
AAAGAGAGGACCGCATTAGAAGGGAAGACAGAGAATTTAGGGCTGAAGAAAACCG
TAAAAATCAAAAGGAGCTGGCTCAGATATTTTTCGCTGGGATTGAAAACAAAAATA
GATCCCAAAAAGGGAAAAGGCCAGATTCAAAAACTGAGGAGAAACCTGCAAGGCG
CAAGCTTGAAAAGAACCAATGTGCATTTTGTAAAGAGTTTGGACATTGGAAAGATA
AATGCCCCAAGAAAAACCTAAAAGAGGGGCCAAAAAATTCCAAGAATGAGACCCC
CCCTCCTGACAGTCATATCCTCTATGCAGGAGAGGATAGTGACTAGGGGGGTCAGG
GCTCGACGCCCCTCCCCGAGTCCTGGATAACTATAAATGTGGAGGGGAAACCGGTT
GGCTTTATGGTAGACACGGGAGCTCAATACTCAGTCTTAAACCAAAGAGATGGGCC
TATGTCTAAGAAAAGTAGCTGGGTACAGGGAGCAACCGGGACTAAGCGATATGGAT
GGACTACAAAACGGCATGTGAATTTGGGGACCCACCAAGTAACTCATTCCTTTCTGG
TGATACCCGAATGCCCAGCGCCCTTGCTGGGAAGAGATTTACTGTCTAAAGTGAATG
CCCAAATTCATTTTGGCCATGGACAAGTGTCAGTTTTAGATGGAACCGGGCACCCTC
TACAGGTTCTGTCTCTGGCATTGAAGGATGAATACAGACTGTACTTGCCAGAGGCCC

CAGCGACAGTAAGTCCTGAAGTACAGCCATGGGTTCAAAGATACCCTCAGGCCTGG
GCTGAAACAGCAGGAATGGGATTGGCCAGACAGAGACCCCCTATTGTTGTGGAGTT
AAAAGCGGGTGCCACACCAGTGAGGGTACGGCAATATCCCATGAGTCAAGAAGCCC
GGCGAGGAATCACTCCTCACATACAACGCCTCACAGACGCTGGGGTCTTAAAGAGA
TGCCGGTCCCCATGGAACACCCCCCTGCTTCCCATAAAGAAGCCTGGGGGAACTGAT
TTTAGACCAGTTCAAGACCTGCGAGAGGTCAACAAGCGGGTGAGTGACATCCATCC
TACGGTCCCTAACCCTTATACATTGCTAAGCAGTTTGCCACCAAGCTACGTATGGTA
TACTGTTTTAGATTTAAAAGATGCCTTTTTCAGTCTGCCTCTCGCCCTGGCGTGCCAA
GACATCTTCGCCTTCAGATGGCTAGAAGACGGTGGACAGACTCCTGTGCAGCTGACG
TGGACTCGCCTACCACAGGGGTTTAAGAACTCACCCACGTTGTTTAACGAGGCCTTA
GATGAAGACCTCCGTGAGTACCGGGTTGAACACCCTACCATTGTTTTATTACAATAT
GTTGATGACCTTATGCTGGCGGCGACTACAGAAAAAGAGTGCCAGGAGGCAACAGG
TGACCTTCTCCAAACCCTTGGGACTTTAGGTTTCAGGGCTAGCGCCAAAAAGGCCCA
GATCGTCAAGCAAGAGGTTACATACCTTGGTTACAAGATAAAACAGGGCCAGAGGT
GGCTAACACAGGCTATGAAAGACACCATCCTTCAGATCCCTGAACCTACCACTCCTA
GGCAAGTGAGAGAGTTTCTGGGAACTGTAGGATATTGCCGATTGTGGATCTTGGGGT
TTGCAGAAAAAGCCAGGCCCTTATATGAAGGGACCAAAGAAAACAAAGACTGGAA
GTGGACTGAATCAATGAAAACGGCTTTTCAGGAGCTCAGGCGTGCCTTGCTGGAAG
CTCCTGCCCTGGCCCTTCCTGACCCATCTAAGCCGTTCCAATTATTTGTGGATGAAAA
GCGAGGGATAGGAAAAGGGGTACTAACACAGAAATGGGGACCTTGGAAGCGTCCC
ATAGCCTACCTTTCAAAGAAATTAGACCCAGTGGCAGCCAGGTGGCCACCTTGCCTC
CGAATTATTGCAGCCACCGCGCTTCTAGTCCATGATGCTGATAAGCTGACTTATGGC
CAGAAACTCTTGGTTTACACTCCTCATGCTATAGAGAGAGTCCTGAAACAACCTCCG
GGCAAATGGATTTCCAATGCCCGCTTAACACACTACCAGGCCTTGCTGCTCGACACC
TCCCGGATTCATTTTCAAACACCCTGCACTCTAAACCCAGCTACTCTTTTGCCCAATC
CAGAAATAGATAGCCCCCTCCACGATTGTGACGAGATACTAGCCGGAGTAACAGCA
GTACGAAAGGACTTAACAGACACGCCACTGGATAACAGTGACCTAATATGGTTCAC
AGATGGAAGCAGTTATGTTAGAGATGGACAGAGACGGGCGGGAGCCGCAATAGTA
GATGACTCTGGACAGACGATATGGGCAGAGACTCTTTCCCCAGACACCTCAGCCCA
AAGAGCAGAGTTAATTGCCCTGATTCAGGCATTAGAGAGAGCTAAAGGTAAAAGAA
TAACTATTTTCACTGACAGTCGCTATGCTTTTGGCACGGTACACATTCAGGGCCCGA
TTTATCGGGAACGCGGGTTTTTAACAGCTGAAGGAAAAGAGATCAAAAACTTACCA
GAAATCCGTCGACTTCTGGAAGCTGTGCAGTTGCCTCGAGCTGTATCAATAGTACAT
GTACCTGGACATCAAAAGGGGGACAGCCTCACAGCCCGAGGAAATCGTGCTGCTGA
TTTGGCGGCTCGGAAGGCGGCTGACAAAGAGTACACCGCTCCAGTGCTGGCAATCG
GACTTCTGCCCCCAGGTATGGGAACTTTGCCCCCAACCCCTGAGTATTCGTCCACAG
ACCTGGCCTGGATCCAGGAATATCCCAACCTCCAACAAGGAGAGGATAAATGGTAC
CGGGACTCCGATGGCTACTTGATACTTCCTGCTCAGTTGGGACGACAACTGTGTGAA
CATCTGCACTCATCTACTCATCTGGGAGAGAAAAAGACTCTGCTGCTTTTTCAAACC
GCACGCCTATGATTTCCCCGGCACCAAACAACCGTAAAAAACATAGTACAAGCTTG
TAAGACATGCCAACAGATGAAGCCAGGAAAGAGGCAACACGCAGGACTGAGGTAT
CGAGGGGAAGGCCCAGGACAGCACTGGGAGATAGATTTTACTGAGGTAAGGCCAGG
CAAGTATGGTTACCGCTACTTGCTAGTGTTGGTAGATACCTTCTCAGGGTGGGTAGA

GGCTTTTCCTACTAAGGGAGAAACTGCAATGGTAGTGGCTAAAAAGATTTTAGAAG
AGATAGTACCCAGGTTTGGCCTGCCGGTGACCGTTGGCTCTGATAATGGCCCTGCTT
TTGTGAGCCAAATAATACAGAACCTTGCCCGGGCTCTAGGAACTAAGTGGAAATTA
CATTGTGAATACAGCCCACAGAGCTCGGGGCAAGTTGAAAGAATGAATCGGACCCT
AAAAGAAACTTTAACTAAATTGGTTATGGAGACTGGCGGGGACTGGGTGACTCTCCT
TCCCTTCGCCCTTTTTCGCGTGCGTAATACTCCTTATCAGCTCAATTTGACCCTATTTG
AAATTCTGTATGGGCGCCACCCTCCCGTATGTCCAAAATTTGAAGGGAAAAAGTTTC
CACTTCCCACCTTGGGACAATTCCAGGAGGCTTTGATGGCTTTGGGCAAAGTACACT
CTTGTGTCTGGAAACTACTCCGAGAGGTACATGAGGGTCAAGGTGAGGGAATTAGC
CCCTCACATAACATTGGCCCCGGGGACTGGGTGTGGGTCAAAAGGCACCACGCCAG
GGCACTGGAACCCAGATGGAAAGGTCCTTATGTTGTTCTTCTTACCACCCCAACTGC
CTTGAAGGTCGACGGTATCGGGCCCTGGGTGCATTGCAATCACGTACGCCCAGCTAC
TTCAGCCGAACAAGAAGACGCGAAAAGACAATGGGAAGCGTCTCTACACCCGTCCA
ACCCTTTAAAATTGAAGATCCAGCGCCGGCCGCAGGACCGACGAGACTCATCTGGA
CCGTCATCTGGATGACTGTGTTGCTTTGTCCTGTGACTGCCAGTGTGAACCCCCACCA
ACCTGTTAAGATCACCTGGACGCTCTGGAACGGACTGACTCGAGAGGTACTCAGTTT
GACCACCGGAATTCACCCCCCTAATACGTGGTGGCCGGATCTGTATTTCAACCTCAA
AGACCTTATAAAGACTACTTGGACAGCGGCCCAGACCAGAAACTTTGGATTCTGGG
CATGTCCCGGACACCTGAAGAGACATAATTGGGAGACTTGTGGGGGGCTGCAACAT
TATTTCTGTTGGTCCTGGAGCTGTGTGACCTCCAATGATGGGAGATGGAAATGGGAG
GTCGGGAACCGGGACTTAGTCAATTTTAAAACCTCCCTATGTGTCAATGTCACAAGA
TCTGTAAACGGGCCTCTGGCTAGCTGGATGATTCCCCAGATGGGAGGATGGTGGGTA
TGTTCCAGAACCGGGTTGACCCCTTGTGTACATGAGTCAATTTTTGATCCCAAAGAA
GAATTTTGTGTCATGGTAGCAGTAGTACCTAAAATAATATACAGGTCGGAAGAAACT
GTGTACGACTATTGGGCCCATAGGTCAACCCTCAATCAGCAAGAAAGAGCATATAG
AATTAAAAGAGAACCCCTTACTGCAATTACTATAGCCACTATGTTTGGCTTAGGGAT
AGCCGGAGCTGGAACTGGAATTACAGCTTTGTCTATGCAAAGTCAAGGATTTAACTC
TTTAAGGGCAGCGATAGATGAAGATATCACCCGACTAGAACAGTCTATAAGTCATTT
AGAGTCTTCTTTAACTTCCTTGTCTGAAGTAGTTTTGCAAAATAGAAGAGGGCTAGA
TTTGCTATTTCTACAACAGGGGGGACTTTGTGCCGCCCTGGGAGAGGAATGTTGTTT
TTACGCAGACCACATGGGTATAGTTAGAGAATCTATGGCCAAAGTGAGAGAGGGGC
TAGCCCAGCGTAAACGAGAGAGGGAGGCCCAACAAGGATGGTTTGAGTCTTGGTTT
CAACAATCCCCTTGGCTGACTACTTTAATCTCAACCTTACTAGGACCCTTGATAATCC
TTCTAATAATGCTCACGTTCGGTCCCTGCATCATCAACAGACTTGTAGCCTTTGTAAA
AGAACGTATTAACACAGTGCAGTTGTTTGTGCTACGGCAACAATATCAAACTATATA
ACTGGGAACAGAGGAAGATTCCTCTGTATGATCAAAGAACAGGGGGGAATGTTAGG
TTATCGGAAGACTTAGTGAGGTGTGTAACTACATCAGGGACCTGGGACAGTAGCTCC
TGCGTTAAACATTCCATAACCAAATAAGGAGGTCAGTAGCTCCTGCGCTAAACATTC
CATAACCAAATAAGGAGGTCAGTAGCTCCTGCGCTAAACATTCCATAACCAAATAA
GGAGGTCAGTAGCTCCTGCGCTAAACATTCCATAACCAAATAAGGAGGTCAGTAGC
TCCTGCGCTAAACATTCCATAACCAAATAAGGAGGTCAGTAGCTCCTGCGCTAAACA
TTCCATAACCAAATAAGGAGGTCAGTAGCTCCTGCCCTAAACATTCCATAACCAAAT
AAGGAGGTCAGTAGCTCCTGCGCTAAACATTCCATAACCAAATAAGGAGGTCAGTA

GCTCCTGCGCTAAACATTCCATAACCAAATAAGGAGGTCAGTAGCTCCTGCGCTAAA
CATTCCATAACCAAATAAGGAGGTCAGTAGCTCCTGCGCTAAACATTCCATACCAAA
TAAGAAAAATCTATAGGGAAGCAGCATCAGGGGAATGTATGAGCTCAGCTTCGCAT
GTAACCAATCAGGTAGTGCCAACTATGCCTGTTGCTGGACTAAGGGACAAACTGTAC
ATAAACCGCCATACCTCTTTGTTCGGGGTCCAGCCGCATTCTGCTGTGTCGGAGAGG
CTAGGACCCTGGCGCGCCAGAAATAAACTCCCTTTATGCCTTTTGCATTACTTTGGTG
GACTTGTTCATTCGGTCGGGTAGGGGACACGGACAAGGAGCATAACATT

ERV_7

TGTTAGGGACCAAACGACAGTCTCTAGGACCTGAGTCATGTTTACCAGAAAGAGAC
AGGATATGTGCTCATCCTGCCTGGCCAATCATGTAACGCCAGCTACTCCTGTAATTG
AGACAAAGAACTGCCTGTATATAAGCCGCCATACTCCTTTGTTCGGGGCTCTTGTCA
GATTCCCTTGTGTGGGATGAGACTTGGGCCCTAGCGCGCTAGGAATAAACAAACTCC
CTTCTTGCGTTTGCAATACTGTGGTGGACTTGCTCTCTCGGTCGGTTCAGAGATACGG
GCTCCGAGCATAACATCTGGGGGCTCGTCCGGGATCTCCGTCCCGCCGGGGAGGAC
AACTCTCCTGGTAGAAGGGAGTAACCTCGTTAGGAGATAAGGGCTCTGAGCACCGG
TGCTAACGCTGCAGAAGCCCAGATTAAGTCCAAGGCCCGGTATCATACTGGGGAGG
CATCTGGCTCTGGTTACTGGATTAAGTCAGCGTAAGGCCGGGGCCTGGTTTCGTGCT
GGGGAGGCAGCTGGCTCTGGTTACTGGATTAAGTCAGCGTAAGGCCGGGGCCTGGC
TTCGTGCTGGGGAGGCAGCTGGCTCTGGTTACTGGATTAAGTCCAGCATAAGGCCGG
GGCCCAGTTTCGTGCTGGGGAGGCATCTGGGTGTAAAGTGCAGAGGTAAGCCCAGC
GTAAGGCCAGGGCCCAGTTTCGTGCTGGGGAGGCGGCTGGCTCTGGTTACTGGATTA
AGCCCAGCGTAAGGCCAGGGCCCAGTTTCGTGCTGGGGAGGCGGCTGGCTCTGGTT
ACTGGATTAAGTCCAGCGTAAGGCTGGGGCCCGGTTTTGTGCTGGGGAGGTGGCTG
GCTCTGTGAACATCCTGCAAGTAAGACTGAGTGCATTGTCGGTGGCCACCTTGCGTT
TGTTATCTGTTTGTCTATTCGTGGTGTCTGCGTTGCTCTTTGTGTGCTCTGTTGGCTCC
CAGTGTACTTTTCTGTGATCATGGGACAAACAACTTCTACTCCTTTATCTCTTATGAT
TAACCACTTCTCTGATTTCAAGTCTAGAGCACAGAATCTATCATTGCTGGTGAAAAA
AAAAGCAAGTTAGTAACTTTTTGTTCTGCCGAGTGGCCTGCTTTTGATGTCAGCTGG
CCACAAGAAGGCACCTTCAGCCTGCCTACTATTCAAGTGGTCAGAGAGAAGGTGCT
CACCCCCTACCCTTCAGGACACCCAGCCAGACCAAACTCCATACATTTTGGTCTGAC
AGAACCTGGTGGAAAACCCCCTGGCCTGGCTAAAACCTTTTGTTTTTCAGTCCCTCA
CTTCCCTTCCCTCTTCCCCTCCCTTGCTTCCACAGGTTCCACAGGTTTCATCAAGAGA
AGCCAAAAAGAGAACCAAGCCTTCAGCTCCTCCCAGAAAAAGGGGCCCCGCCTAGG
GAACTCGGAGAAAAGGCAAAAAAAAAAAAAAAAAAAAAAAAAAAAAAAAATGGCCG
GCGTAGCAGAAGAAGACCCGGAGGTTCCTTCCTCCACCATTCATGTGTTTCCGGTCC
GGGCAGGACCAGCCAGAGAGGGTGGAGAACGGACATATCAGTATTGGCCCTTCTCC
ACTAGTGATTTGTACAATTAAAAAACCCAGACTCCCTCCTTCTCTGAAAAACCACAG
GGTCTTATTGATCTTTTTTAGAGTCTATCCTGTTTACTCACAATCCCACTTGGTATGA
TTGTCAGCAACTGTTACAGGTACTTTTTACTACAGAAGAGCACGAACGGATCCCTGT
CAGAAGCCCTGAAAAATGTGCCAGGGGTAGATGGAGGCCCACAATACAGCCTAAC
CTCATTGAGGAGGGGTTCCCCTTGGTGCGACCCAACTGGGACTTCAAACGCGCTAAA
GGTAAGGAGCCTCTCCGAGTGTACCGTCAGACTCTCATGGCTGGCCTTAGAGAGGCC

ACCAAAAAGCCAACTAATTTGGCCAAAATAAATCCAGTGAGACAACAGCCAAATGA
GAGCCCAGCAGCCTTCCTGGAAAGGATAATGGAAGCTTTTAGACAGTATACCCCTAT
GGACCCACAGGCAGATGAGTCACGAGTGGCAGTTATGTTAGCATTTGTAAATCAAG
CAGCCCCCCATATTAAAAAAAAGTTACAAAAGATAGAGAGGTTAAATGAACAATCC
TTGCAAGATCTAGTGAGGGCAGCCGAGAGTTTTTAATCATAGAGAGACCCCAGAAG
AGAGAGAGGACCACATTAAAAGAAAAAAAAAGAAAATTTAGAGCTGAAGAAAACC
GTAAAAATCAAAAAGAGCTGGCCCAGATATTTTTTGCTGGGGTTAAAAACAAAAAC
AGGTTCCAAAAAGGGAAAAAATTGGACTCAAATACTGAAGAAAAAATGACAAGGC
GTAAGCTTGAGAAAAACCAATGTGCATTTTGTAAAGAGTTTGGACATTGGAAAGAT
AAATGCCCCAAGAAAAATCTAAAAAAGGGGCCCAAGAACCCCAAGAACAAGACTC
CCTCTCCAGACAGTCATATCCTCTATGCGGGTGAGGATAGCGACTGGGGGGGTCAG
GGCTCAAAGCCCCTCCCCGAGTCCTGGGTAACTATAAATGTGGAGGGGAAACCGGT
TGGCTTCATGGTGGACACGGGAGCCCAATACTCAGTCTTAAACCAAAAAGATGGAC
CCATGTCTAAAAAAAGTAGCTGGGTGCAGGGAGCAACTGGGACTAAATGATATGGA
TGGACTACAAAATGGCATGTGAACTTGGGGGCCCACCAGGTAACCCATTCTTTTCTG
GTGATATCTGAGTGTCCAGCACCCTTGTTTGGAAGAGATTTACTGTCTAAAGTAAAT
GCCCAAATTCATTTCGACCACGGACAAGTGTCAGTTTTAGATGGGACCGGGCATCCT
CTTCAGGTCCTGTGTCTGGCATTAAAAGATGAATACAGACTCTACTTGCCAGAGGCC
CCAGCGACAATAAGCCCCGAAGTACAACCATGGGTTCAAAGATACCCTCAGGCCTG
GGCTGAAACAGCAGAAATGGGACTGGCCAAACAGAGGCCCCCTATCATTGTGGAAC
TAAAAGCCAGTGCTTCCCCGGTGAGGGTACGACAGTATCCCATGAGTCAGGAGGCT
CGACAAGGAATTACTCCTCATATACAATGCCTCATAGATGCTGGGGTCCTGAAAAGG
TGCCGGTCCCCATGGAACACTCCCCTGTTGCCTGTGAAAAAGCCTGGGGGAACTGAT
TTTAGACCGGTTCAAGATCTACGAAAAGTCAACAAACGGGTAAATGATATTCATCCT
ATGGTTCCTAACCCTTATACATTGCTAAGCAACTTGCCTCCAAACTACATTTGGTACA
CTGTTTTAGATTTAAAAGATGCCTTTTTCAGTTTGCCTCTTGCCCCCGCAAGCCAAGA
GATCTTTGCCTTCGAATGGCAGGAAGACGATAGTCAGACCCCTGTGCAGCTGACATG
GACTCGCTTACCACAGGGTTTCAAAAACTCGCCCACGTTATTTAATGAGGCCCTGGA
CGAAGACCTCCGTGAGTATCGGGTTGAACACCCTACCATTGTTTTATTACAATATGT
TGATGACATTATGCTGGCAGCGGCTACAGAGAAAGAGTGCCAAGAGGCAACAGGTG
ACCTTCTCCAAACCTTGGGGACTTTAGGTTACAGAGCCAGTGCCAAAAAGGCTCAGA
TTGCCAAGCAAGAGGTTACATACCTCGGTTATAAGATAAAACAGGGCCAGAGGTGG
CTAACACAAGCTATAAAAAAACCATCCTCCAGATCCCTGAGCCGGCTAACCCTAGA
CAAGAGAGAGAATTTCTGGGAACTGTGGGATATTGCCGGTTATGGATCTTGGGGTTT
GCAGAAAAGGCCAGGCCCCTATATGAAGGGACCAAAGAAAACAAGGACTGGAAAT
GGACTGAGCCAATAAAAGAGGCCTTCCAAGAGCTCAGGCGAGCCTTGCTAGAAGCT
CCTGCCCTTTCCCTCCCTGATCCATCTAAGCCTTTCCAATTATTTGTAGATAAAAAGC
AGGGGATAAAAAAAAGGGGTACTAACACAGAGATGGGGACCATGGAAGCGACCTG
TAGCTTACCTTTCCAAGAGACTGGACCCAGTGGCAGCCGGATGGCCACCTTGCCTCC
GTATCATCGCGGCCACTGCACTCTTAGTCCATGATGCTGATAAACTGACTTATGGAC
AGAGACTCTTGGTCTACACTCCTCATGCCATAGAGAGAGTTTTAAAGCAACCCCCAG
ATAAATGGATTTCTAATGCCCGCTTGACGCACTACCAGGCCTTGCTACTTGACACCC
CACTGATTCATTTCCAAATGCCCTGCACTCTAAATCCGGCCACTCTTTTGCCCAATCC

AGAAAAAAAATAGCCCCCTCCATGATTGTGATGAGATACTGGCCAGGGTAACAGCA
ATGCGAAAAGACTTAACCGATACTCCACTGGATAACAGTGAGCTAAAATGGTTCAC
AGACGGCAGCAGTTATATAAAAGATGGACAGAGACGGCTGGGAGCCGCAGTAGTA
GATGACTCTGGACAGACGATATGGGCAGAGGCCCTTCCCCCGGATACCTCAGCACA
AAAGGCAGAGTTAATTGCCCTGATTCAAGCATTAGAGAGAGCCAAAGAAAAAAAT
AACTATTTTCACTGACAGTCGCTATGCTTTTGGCATGGTACACATCCAGGGCCCAAT
ATATCCGAAACGGGGGTTTTTGACAGCTGAAAAAAAAAAAAAATTAAAAACTTGCC
TAAAATCCGTAGACTTTTAGAGGCTGTACAGATGCCTCAGGCTGTGTCAATAGTACA
CGTACCTGGACATCAGAAGGGTGACAGCCCCACGGCATGAGGGAATCATGCCGCAG
ACCTGGCAGCTCGAAAAGTAGCTGATGAAGATTTCATCACCCCTGTGTTGGCGATCA
GACTTCCACCTCCAGGTATGGGAACTCTGCCCCCAACCCCTGAGTATTCATCCACAG
ACTTTGCTTGGATCCAAAAACACACCAACCTTCAGAAAGATAAAGATGGATGGTAC
CGAGACTCAGACGGCTACTTAATACTCCCTGCTCAGTTGGGACGGCAACTATGTGAG
CATTTACACTCGTCTACTCATCTGAGAAAAAAAAGAAGACTCTGATGCTCTTTCAAA
CTGCACGCCTGAGATTTCCCCAGCACCAGACAACTGTAAAGAACATAGTGCATGCTT
CTAAGGCGTGTCAACAGATGAGGCCAGGAAAAGGACAACATGCAGGACTGAGGTAT
CGGGGAGAAGGACCAGGGCATCACTGGGAAATAGATTTCACCGAGGTAAGGCCAG
GCAAGTATGGTTACCGGTACTTGTTAGTGTTGGTGGATACCTTCTCAGGGTGGGTGG
AGGCTTTTCCTACAAAGGGAGAAACCGCGATGATAGTGGCAAAAAAGATTTAAAAT
ATATATATATATAGTTCCCAGGTTTGGCCTGCCAGTGACCATCGGCTCTGATAATAG
ACCTGCTTTTGTGAGTCAAATAGTTCAGAGCCTTGCCCTAGCCCTGGGGACTAAATA
AAAGTTACATTATAAATACAGTCCACAGAGCTCAGGGCAAGTAAAAAAAAATAAAT
CGGACTCTAAAAAAACTTTAACTAAATTGGCTATAGAGACTGGCGGGGACTGGGTG
ACCCTCCTTCCCTTCGCCCTCTTCCGTGCGCGTAATACTCCTTATCAACTTAATCTGA
CCCCATTTAAAATTCTGTATGGGAGACTTCCCCCTGTATGTCCAATATTTAAAAGAA
AGAAACTACCGCCTCCCACGTTGGGGCAATTCCAAGAGGACTTGATGGCCTTAAGC
AAGGTGCACTCTCGTGTCTGGAAACTGCTCCAGAAAATACATGTGGGTCAAAATAA
GTGTTCCCTCACATGACATTGGCCCAGGAGACTGGGTATGGGTCAAAAGGCACCAA
ACCAAGGCACTAAAACCCAAATGGAAGTGTCCTTATGTTGTTCTTCTTACCACCCCA
ACTGCCCTAAAGGTCGACGGTATCGGGCCTTGGGTGCATTGCAACCACATACGCCCA
GCTGCTTCAGCAGAGCAGGAAGACACTAAAAAAAGAATGGAAAGCATCTCTGCACC
CGTCCAACCCCCTGAGGCTAAAGCTTCGAAGGCGCCAACAGGACCAGGACAACTCG
GCTGGGCCGTCTTGTGGATGACCCAGTTATTCTGCTCCAGAGTTGCCAGCGTGAACC
CGCATCAACCCGTCAAAATCACCTGGAAGCTGCAAAATGGACTAACACGAGAGGTG
CTAAACTCCACTACCGCCATACATCCACCAAACACATGGTGGCCAGACTTGTACTTT
GACCTTAAGCCGATGGTAAATGTGTCTTGGGCTAGGGGTTATCTCCGAGAACAAGG
GTTCTGGGCATGCCCAGGCGCACCCAGACATGACTGGAAGACCTGTGGGGGGGCAC
AAGACTCATTGTAAAACTTGGGATTGTGTTACTTCTAATGATGGACCTCGGCGCTGA
GAAGTAAAAAATCGAGATTCATTTAATTTTTCATTCGCCAAGCCCCTCCCTAGGGTC
CTCGGAGATCCAACTTTTAGCTGTAAAAGTTGCAATTATGCACAAGTCAGAATAAGG
TTTAATCCAGAAAAAAGCAAACAAGAGGGGATTAGATCTGCTCTTTCTGCAACAAG
GGGGACTCTGTGCTGCCCTAGGAGAAGAGTGCTGTTTCTATGGGGATCATACAGAA
ATAGTTAAAAAATCTATGGCCAAAGTGAAAAAAGGACTAGCCCAACGTAAACGAAA

ACGTGAGGCTCAACAGGGATGGTTTGAATCTTGGTTTCAACAATCCCCTTGGCTGAC
TACCTTAATCTCCATCTTGCTAGGACCCCTGCTAGTACTTTTACTAATGCTTACCTTC
AGCTCATGCATTATCAATAGACTTGTAGCCTTTGTAAAGGAACGCATAAATACAGTA
CAGCTGTTTGTGCTTCGACAACAAAATCAAACTGTGTCTCAGGACCGAGAGGAAGA
TTCCTCTATATGATCTAAGGACAGGGGGAAATGTTAGGGACCAAACAACGGTCTCTA
GGACCTGAGTCATGTTTACCAGAAAGAGACAGGATATGCACTCATCCTGCCTGGCCA
ATCGTGTAACGCCAGCTACTCCTGTAACTGAGACAAAGAACTGCTTGTATATAAGCC
GCCATACTCCTTTGTTCAGGGCTCTTGTCAGATTCCCTTGTGTGGGATGAGACTTGGG
CCCTAGTGCGCTAGGAATAAACAAACTCCCTTCTTGCGTTTGCAATACTGTGGTGGA
CTTGCTCTCTCGGTCGGTT

ERV_8

GTTACAGCCTCACCAGAGCCATGACAGGCTCAGAGAGGTGCACTAGGCCAAAAATA
ATCCCTGAGTCATGCTTCTGGGGCTTCTGGGGATGATAAATCTGGCCAATCAGAGAG
ATGATAACTCTGGCCAACCAGAGGGATGATAACTGGCCAATCAGTAAATACCAGGA
AACCCTTGCAGCCAATCAACCCTTGCCAACTCCCCGTCTTTGCTCTAAACTTATAAAT
ACTGCTGTAAATCTGGGCTCAGGGCTCTTGCTCCACTCCACTCCGTTGGATGTGGTG
GGAGGCCCTGGCTCGAGCTAACAATAAACCCCTTTATGCTTTTGCATTGCTGTGGAT
GTCTTATTCTCTCAGTTTTGGGGACTCGGACACTGGGCAAAACATTTGGGGGCTCAT
CCGGGATCCTTTAACTGGGAAGAATAACATTCTCCCGGTAGAAGGGGTTTCCTCACT
AGGAGGAGAGATATCTGAACACCGGTGTTAGTTTTGGATTAAGTCCAACGTAAGGC
CAAGGCCCAGCATCGAGCTGGTGGGGTGGGGGTGGGGGGGCAGCTGGCTCTGTGAA
CATCCTGCAGGTAAGACTGAGTGCATTGTTGGTGGCCACCTTGCGTTTGTTATCTGTT
TGTCTACTTGTGGTGTCTGCATTGGTCTTTGTGTGCTCTGTTGGCTCCCAGTGTACTTT
TCTGTGATCATGGGACAAACAACTTCTACTCCTTTATATCTTATGACTAACCACTTCT
CTGATTTTAAGTCTAGAGCACAGAATCTATCATTGCTGCTGAAGAAAAGCAAGTTAG
TAACTTTTTGTTCTGCCGAGTGGCCTGCTTTTGATGTTGGCTGGCCACAAGAAGGTAC
CTTCAATCTGCCTGCTATTCGAGTGGTCAGAGAGAAGGTGCTCACCCCTTACCCTTT
GGGGCACCCAGCCCAAACTCCATACATTCTGGTCTGGCAGGACCTGGTGGAAAACA
CCCCCGGCCTGGCTAAAACCTTTTGTTTTTCAGCCCCTCACTTCCCTTCCCTCTTCCCT
GTCCTCGGCTTCATTATGTCCACAGGTACTAGTTGTGGAAGCATCCAAAGAGAAAGA
AGACAAAAAGCACAGCAACCTGGTGAAACTGGTATTCCAGGAATCCTCACTATACC
CCAATTTACTCGACCTGGAGACAGAACTCTCCCCGCCCCGCTATGGGGATCCACTTT
TGCCCCCGCAGGTTCCTCAGGTCTCTTCTGGAGGGATACAAAGGGACACCGAGCCTT
CAGCCCCAGCCTGGGAAGGAGGCCCCGCCCAAGGGACTCGGGGAAGAACCAGGGG
CATCACCAATATGGCGGAAGAAAACAACCCAGAGGCCCCCTCCTCCACAGTCCGGG
CATTTCCAGTTCGGGCGGGGCCTGCCTGAGCAGACAGAGAACAGACATACCAGTAC
GGGCCCTTTTCCACCAGTGATCTTTACAACTGGAAAACTCAGACCCCTTCATTCTCTG
AAAAAACTCAGGGCCTCATTGACCTTTTAGAATCTATCCTTTTCACCCATAACCCCA
CCTGGGATGACTGTCAACAGTTGTTACAAGTGCTCTTTACTACAGAGGAACGCAAAC
GGATCTTGTCAGAAGCACGAAAGAATGTGCCAGGGGTAGATGGGAGACCCACTACA
CAGCCTAACATGATTGATGAGGGATTTCCTCTGACGCGGCCCTGCTGGGACTTCGAG
AGCACTGAAGGTAGGGAGCATCTCCGAGTGTACAGCCTGACTCTCATGGCCGGCCT

CCGAGTGGCTGCCAGGAGGCCAACAAATTTGGCTAAAGTAAATCTTTTTAGACAAG
AGCCAAATGAGAGCCCGGAAGCTTTCCTTGAGAGATTAATGGAAGCTTTTAGGCAG
TATACCCCCACGGACCCACAAGCCGATGAGTCGCACGCAGCAGTCATGCTAGCATT
CATGAATCGGGCAGCCCCGGATATCAGGAGAAAACTACAAAAAATAGAGAGGTTGG
GAGAGCAATCCCTGCAAGACCTGGTGAGAGCAGCAGAGAGGGTTTTTAATCATAGA
GAGACTCCAGAGGAAAGGGAGGAATGTGTTAGGCTAGAGGAAAGAGAATTCAGGG
CCAAAGAAAACTGTAAGAATCAGAAAGAATTAGCCCAGATATTCTTTGCAGGGGTG
GAACAGGGAGCTAGTTCTCAGAGAACAAGGGAAGTCCATTCGAAGGGTGAAGGGA
AGCCAGCGAGGCAAGGACTTAAAAAGGACCAGTGTGCTTTTTGCAAGGAGATAGGA
CACTGGAAGAGTAAATGCCCCAAGAGAAACCTAAGGGAGAGACCCACCAAGCAGG
AGGTGTCCTCCTCCTCCACAGGGGCCCACATCCTATATGCAGGAGAGGATAGTGATT
AGGGGGGTCAGGGCCCGGCACCCCTCCCTGAGTCCTGGGTAACTATACATGTGGAG
GGGAAACCGGTTGGCTTCATGGTGGACACAGGAGCTCAATACTCGGTCCTTAACCA
GAAAGACAGACCAATGTCTAAAAAGACCAGCTGGGTACAAGGGGCCACCGGGACT
AAATGATATGGACGGACTACTAAACATCAAGTGAACTTGGGGGCCCAGCAGGTGAC
CCACTCTTTTCTTGTGATACCAGAATGCCCAGCACCCTTGCTGGGAAGAGACTTACT
ATTTAAACTCAATGCCCACATTCACTTTGACCACAGAGAGATATCAGTTCTAGATGG
GACCGGACATCCCATACACGTTTTGTCTCTGGCATTGAGAGACGAATACAGACTGTA
TCAGCCAAAGCCGCTCATGGCCATTGACCCCAATGTACAACCTTGGGTCCAAAAATA
CCCTCTAGTCTGGGCAGAAAATGCGGGGGTAGGACTAGCCAAACAGAGGCCTCCCA
TCATTGTCAAACTGAAGTCAGACGCCACCCCTATCCAGGTAAAACAATACCCTTTGA
GCCTAGAAGCCCGGTGAGGAATCACACCACATATACAATGGCTTCTGGAGGCAAAA
ATTCTTAAAAGGTGCCGATCTCCATGGAATACTCCTTTGTTACCTGTGAGAAAGCCA
GGGGGAATGGACTTTAGGCCTGTGCAGGATCTTTGTGAAGTCAACAAACGGGTGAA
TGATATACATCCAACCGTCCCTAACCCGTACACCCTTTTGAGTGGCTTTCCTCCAGAC
TACGTCTGGTATACTGTTCTGGACTTGAAAGATGCTTTTTTCAGTTTACCCTTGGCCC
CCTTGAGCCAAGAGATCTTCGCATTCGAATGGATAGAAGAAGGCAGCCAGACCTCA
GGACAGCTAACTTGGACTCGACTTCCGCAAGGCTTCAAGAATTCACCAACGCTATTC
AATGAGGCTCTGGGTGAAGACCTCCATGAGTACCAGGTCGATCACCCCAACATTGTT
CTATTGCAGTATGTTGATGATTTATGCTAGCCGCAACCACTGAGGAGGCATGCCTAG
AAGCAACAGGCAACCTCCTTCAAACTTTGGGGACCTTGGGGTACCGGGCTAGTGCA
AAGAAGGCTCAAATTGCTAAGCAGCAAGTCATATACCTAGGGTATAAAATAAAACA
AGGACGGAGATGGCTGATGCAGGCCATGAAAGAGGCCATATTGCAGATCCCTGATC
CGGCAACTCCTCAACAAGTGAGAGAATTTCTTGGGGCTATTGGGTATTGCTGGCTAT
GGATCTTGGGATTTGCTGAAAAGGCCCGGCCACGATATGAAGGAAGTAGAGAAAAT
AAAAACTGGACTTGGACTGAACCAATGAAACGGAATTTTCAAGAACTCAGACAGGC
TCTGCTGGAAGCCCCAGCCCTTGCTCTCCCTGACCCGTCCAAGCCCTTCCAATTGTTT
GTAGATGAAAAACGGGGAATGGGAAAAGGAGTCTTGATGGGAAAGGTCGCCTTGG
AGGCGACCAGTGGCCTACCTATCCAAACGACCGGAGAAAGGCCAGGGCAACACTGG
GAGATAGATTTTACAGAGGTAAGACCAGGCAAGTATGGGTACCGTTATCTGTTAGTT
TTGGTGGATAATTTTTCTGGGTGGGTGGAAGCATTCTCTACTAAGGGGGAAACAGCA
ATGATAGTAGCTAAAAAGATCCTAGAAGAAATAGAACCTAGGTTTGGGCTGCCGGT
GACTATTGGCTCTGATAACAGGCCTGCCTTTGTGAGTCAGATTGTACAGGGACTGGC

CTTAGCTCTGGGGACCAAATGGAAGCTGCATTTTGAATACAATCCCCAGAGCTCAGG
ACAGGTTGAGAGGATGAATCAGACTCTAAAAGAAACTTTGGCAAAATTGGCAATAG
AGACTGGCGGGGACTAGGTGACTCTCCTTCCCTTCACTCTCTTCCGTGCGTGTAATAC
CCCCTACAAGCTGAACCTAACCCCTTTTGAAATTATGTATGGGAGGCTCCCTCCCAT
ATGCCCTATCTTTGAGGGAAGAAAACAACCACCCCCTACTTTAGGACAATTCCAGGA
AGCCCTGATGGCATTAGGCAAGGTGCATATGCATGTCTGGAAATTGATCAGGGAAA
TCCACGAGGGTCAGAACAAGGGGACCATCCCCTCACATAATGTTGGTCCTGGAGATT
GGGTCTGGGTCAAGCGACATCAACCCAAGACATTAGAACCTAGATGGAAAGCTCCT
TATGTTGTTCTTCTTACCACCTCCACTGCTTTAAAGGTCGACGGTATCGGACCTTGGG
TGCACTGCAACCATGTACGCCAAGCCACTCCAGAAGAGCAAGAAAAGGCCCAAAAA
GAATGGGAGGTAACGCCACACCCTTCAAACCCCTTCAAGATAAAGCTCATCTGCCA
ACAGACAAGTCATCCTGACTGTCCTATTGGTGACCATCCTTCTCGACCCTGGAGCCA
CCCATAACAACCTGTACCAGCCGGCCAAAATCACCTGGAAGCTTCAAGACGGACTA
ACACGAGACATGCTCAACTTAACCACAGAAATACATCCTCCAAACACCTGGTAGCC
AGACCTATATTTTGACCTAAAGCTGGTGGTGGGTGTACCTTGGGCAAGGGGTTTCCT
CCGGATGCAGGGGTTCTGGGCATGCCCGGGCTACCAGAGGACCAAATGGAAGACTT
GTGGGGGGGCACAAGATTATTTTTGTAAATCATGGGATTGTATTACTTCTGATGATG
GGCCTCGGGGCTGGGAAGTAGGAAATCGGGACCTACTTAACATCTCGTTCGCAAAG
TCCACCCCCAGAGCCCCTGGGGACCCAACTTTTGAATGTGGAGAGAGTTGTAACTAT
GCACAGGTTAAGACAAGGTTCAATCAGAAAAAAGCACAAAAAAGAGAGGGCTTGG
ATTTCTGGCTTATCCTGGGGAATACAAACAAGAACGGTGGCAGGCGCGCAAATTTA
CGGAGGAATTATAATTATAAGCCAGATTCTAGAACCAACACAGATACACAGTATAG
GTCCCAACCCAGTGGAGAAAATTGACGTAACTCCCAGCCCGACCACAGCGGTTTCC
ACATCCTTAGTCTCACTTGATCCTCTGGGTCCCATCCGCAACCTCAAAAACTTAGAC
CCATTATGAAAATTAGTTAAGGCAGCCTATATAACCTTAAATCAAACTAACCCGGAA
GCAACTAAATCTTGCTGGCTTTGCTATAATCTATACCCCCCCCTCCTTACTATGAAGC
AGTGTGTCTTAACGCCTCTTATGGCCTCACTGATAGTATAGATCCTCCTCAATGTCGC
TGGAGAGAATGGAAAATGGGCCTCACAATGAAAAAGGTTTGGGGAAAGGGACTCTG
TGTGGGCAGAGTTCCACCTGAAAATTCCCCCTTATGTGCCTACACAACCAAACCCAC
AGAGCTAACTGAGATTAAATGGATTATCCCAGAGGTGGGGGGATGGTGGGTCTGCT
CACGTACTGGTCTAACCCCTTGTTTACACATCTCAGTATTCAATCCTAAAGAAGAGT
TCTGCATCTTGGTGGCAGTTGTGCCAAAAATCCTGTACCACCCTGAAAAGATAACGT
ACGACTACTGGGCCCAAAAAACAAACCCCCAGTCAAATGATACAAGGCAGAACCAA
GAGAGAACCTATTACAGCCATCACCATAGCAACCATGTTCGGCTTTGGGATCGCTGG
GGCAGGGACTGGGATAGCAGCCTTGTCCCTCCAAGGCCAGGGATTTACTTCATTGCA
GCCATAGATGAAGACATTACCCACCTAGAAGAGTCAATTAGTCACCTAGAAAAATC
GCTGACCTCCCTATCCGAGATGGTCTTGCAAAACCGAAGAGGGCTAGATCTAAGTTT
TCTGCAGCAAGAGGGGCTCTGCGCGGCCCTGGGAGAAGAATGTTGTTTCTAAGCAG
ACCACACCGGGATAATAAGAGAATCTATGGCAAAGGTGAGAGGAGGACTAGCCCA
GCGAAAGAGCGAGAAGCCCAGCAGGGATGGTTTGAATCTTGGTTTCAACAATCTCC
GTGGCTGATGACATTAATTTCCACCCTGGTGGGACCTCTTATGGTGCTTTTACTAATA
CTCACCTTTGGCCCATGCATCCTCAACAGGCTCATTACGTTTATAAAAGAGCATATT
AATACAGTTCAACTATTTGTGCTTAGACAGCAATATCAAACTGTACCCCAGAACAAA

GAGGAAGATTCCTCTATATGATCTAAAGACAAGGGGGGAACGTTACAACCTCACCA
GAGCCATGACAGGCTCAGAGAGGTGCACTAGGCCAAAAATAATCCCTGAGTCATGC
TTCTGGGGCTTCCGGGGATGATAACTCTGGCCAGTCAGAGAGATGATAACTCTGGCC
AATCAAAGGGATGATAACTGGCCAATCAGTAAATACCAGGAAACCCCTGCAGCCGG
TCAACCTTGCCAACTCCCCATCTTTGTTCAAAACTTATAAATACTGCTGTAAATCTGG
GCTCGGGGCTGTTGCTCCACTCCACTGTGTTGGATGTGGCGGGGAGCCCTGGCTCGA
GCTAGCAATAAACCCTTTTATGCTTTTGCATTGCTGTGGGTGTCTTATTCTCTCAGTT
TTGGGGACTCGGACACTGGGCAAAACATTT

ERV_9

GCTCCATGACAGGCTTCAGGGGGCCAAGGTAGGCCTAAGTCTCCTTCACAGAGGGG
CTGAGTTATTGCCAAGGAGGAGCTGAGAGCCTCCTCTGCCAGTGCCAGGACTCGAC
CAATCAGTGTACTCTCTGTAGCCTGGTTCAAGCTTATCAGGACGAGGATGAAAACCC
CCTGAGAAAGCCCGCGCACGCGAAAATCTAGCCAATTAGTAGATGCTAAGGAACCC
TTGCAACCAATCCACCCCTGCCAACTCCCTGTCTTTGTCTTAACCCTTTAAATACTGC
TGTAATTCAGGGCTCGGGGCCCTTGTTCCACTCCACTGCGTTGGATGAAACTTGGGC
CCTGGCTCGAGCTAGCAATAAATTCCCTTTTTGCATTTGCATTGCTGTGGATGTCTTG
TTCTCTCAGTTCTGGGGGTTGGGACCTTGGGCATAACATTTGGGGGCTCGTCCGGGA
TCCCCTGACTGAGAAGAACAACACCTTCCAGGCATAAGGAAAGCCTCACTAGGAGG
AAGTATATCTGGACGCCAGCATCAGGTCAGGGGGCGAAAGAGTCACCCGGCCAGCA
TAAGTCTGGGGCCCAGCATTCGAGCTGGAAGGCAGCTGGTTCTGTGAGCCATTCTGT
AAAGGAACAGGGGAAAACGTTTCTCCCGATCCTGAGTCTGGTGGGCAGTGGACGCC
CTTTGGTAAAGTAACTTTGGGGAACAGGACAACAGCAACCCAGGATAAACCTAGTT
CAGTGTTTTCAGCCGATATTTGTTCGTCTACTGTCTGTCGCTTGTCTTTGTGTGCCGGT
GGTGCGCTTGCTGTTTGTGCGTTTATTGTGGGTGTTGTCGCCATGGGTCAAAGTTATT
CTACACCACTATCTGTAATGACTGACCATTTTCCCGATTTTAAGTCCAAGGCTCAGA
ATCTATCCGTGCTAGTAAAGAAGAGCAAGCTGAAAACCCTATGCTCATCTGAGTGGC
CGACATTTCAGGTTGGCTGGCCGCCGGAGGGAACTTTCGGCCTGCCGGCCGGTCATC
CAGGCTGTTAAAGAAAAGATAATGGCTCCTGACCCTCGGAGCCATCCGGACCAGGC
TCCTTACATCCTGGTCTGGGAAAATCTAGCAGAAGACCCTCCCGTCTGGCTAAAACC
CTTTGTTCACAACCCCCCAGTGCTTCTGTTCCACAGGTCCTGGTCATGGAGACCTCTA
AGGAAGAAAACCGGGAGGCACGGGAGAGCCGGAAGAAACCGATACTCCAAGAATC
TTCCCTATACCCTAGTCTGATTGATTTAGACACTGAAATCTCACTTCCCCCATATATC
CCGCCACCCTTGCTCCCGCAGATACCTCAGTTGTCATCCAGAGAACAAAGGGGGAA
CTTAGAACCCTCAGCTCTGCAGCAGGAAGGGGGGCCAGCCCAGGGAACTTGCGGAA
GGACCCAAGGGGGCAGAGACTCGTCTGACTATGGGAGCCGAGAGGCCCCTTTGTCT
ACCGTTCGAGCGCTTCCTGTCCGGGTCCGGCCGGCAAATCCTGACGGAGAATGGAA
TTATCAATATTGGCCGTTCTCTACCAGCGACTTACATAACTGGAAAGCTCAAAACCC
CTCTTTTTCTGAAAAGCCCCAAGGCCTTATTGATCTTTTAGACACTATCCTGTTTACT
CACAATCCTACTTGGGATGATTGTCAGCAGCTGTTACAGGTGCTTTTCACCACAGAG
GAACGGGAGCGAATTCTGTCGGAAGTGCAGAAACACGTTCCCGGAGCGGATGGGGA
GACCGAGAATGCAGCCCCACCTAGTGGAGAAAGGGTTCCCTTCTATGCGGCCGAAC
TGGGACTTTGAGCATGCGGAAGGTAGGGAGCATCTTTGGGTCTACCGCCAGACTCTA

ATGGCCAGCCTCCGGGCAGCCACCAGGAAACCGACAAATTTATCTAAGGTGAATCT
GGTAAGACAGGAGCCAAACGAGAGCCCAGCAGCCTTCTTAGAGAGGTTAATGAAAG
CCTTTAGGCAGTATACGCCCATGGACCCCCAGGCTGATGAGTCACGCGCAGCAGTTC
TGTTAGCTTTCGTGAACCAGGCAGCTCCAGATATCAGGAGAAAGTTACAGAAGATA
GAAGGTCTGGGGGAACTGTCAATACAGGATCTGGTGAGGGCAGCTGAGAAAGTGTT
CAATAACAGAGAGACCCCTGAGGAGAGGGAGGAACGGATTAGACGGGAAAAAAGA
GAATATGAGAAATGGATTAGATGGGAGGAAAGGGAATATAGGGCCGAAGAAAACT
GGAAAAATCAGAAAGAGCTGGCTCAGATCCTTTTTGCGGGGATGAGAAAGGGGCCC
AAAGCCCTGAGGACTAAAGACACCCGGTCGGGAGGAAAGGAAAATCCAGCTAGAC
CTGCCCTAAAGAGAGATCAATGCGCTTACTGTAAAGAGCAGGGACACTGGAAAAAA
TGAGTGCCCCAAGAGAGATCTGAAAAAAAAGACTGTAAGAAAGGAAGACTCTTCCT
CGGGGACCCACGTCTTATATGCAGGGGAAGATAGTGATTAGGGGGGTCAGGGCCCG
GCACCTCTCCCCAAGTCCTGGGTAGCTATCAATGTGGAGGGGAAACCGGTCGGCTTC
ATGGTGGATATGGGAGCCCAATACTCAGTCCTCAACCAAAAATTTGGGCCGATGTCC
AAAAAGACTAGCTTGGTCCAGGGAGCCACCGGGACAAAACTAAACGAAAAGTGGA
CTTGGGAGCCCAACGGGTGTCCCACTCATTTCTGGTGATCCCGGAATGTCCAGCCCC
TTTATTAGGAAGAGACCTATTGGCCAAAGTCAATGCACAAATTCACTTTGACTCTGG
GGGAATATCAGTCACAGATGGGCTTGGACAACCAATTCATGTTTTATTCCTGGCACT
GAGAAATGAATACAGACTATATTCACCAAAACCCCCTGCAGCTGTGGATCCTGCTAT
GCAACAGTGGATTCAGAAGTACCCTCTGGCCTGGGCAGAGATAGCAAGAATGGGAC
TGGCTAAACAAAGACCTCCCATTGTTGTTGAATTAAAAGCAAATGCTATTCCTGTGA
GGGTGAAACAGTATCCCATGAGTCAGGAGGCCCGGCAAGGAATTATGCCACACATC
CAGCGCCTCCTAAAGGCAGGAATTCTCAAAAAGTGCCGGTCCCCATGGAATACTCC
CCTGTTGCCTGTGAAAAAGCCCAGGGGAGCAGACTTTAGACCAGTCCAAGATCTTCG
TGAAGTCAACAAACGGGTGAGTGACATTCATCCCACTGTCCATAACCCATACACCCT
CCTGAGCAGCTTGCCACCAGACTATGTCTGGTATACAATTTTAGACTTGAAAGATGC
CTTTTTCAGCTTGCCTTTGGCCCCCCAGAGCCAGGAAATCTTCGCCTTTGAATGGGCT
GACGAGGACGGCCAAACTGTGGGGCAGCTGACCTGGACTCGCCTCCCACAGGGGTT
CAAAAACTCACCGACATTGTTCAGTGAGGCTCTAGGCGAAGATCTCTGTGAGTATCG
AACCAGCCACCCCGAAGTCGTTCTGTTGCAGTATGTAGATGACCTAATGCTGGCCGC
TACAACTAAGGAGGTATGCCTAAAGGCCACAGGTGATCTCCTCCAGACTTTGGGGA
CATTGGGGTACTGGGCAAGTGCAAAGAAGGCCCAAATTGCTAGACAGGAAGTCATT
TATTTGGGATATAAAATAAAACAGGGGCAGAGATGGTTGACTCAGGCTATGAAAGA
GACTATTCTACGGATCCCCAAGCCAACAACTCCCCAGCAGGTGAGGGAGTTTTTCGG
GACGGTTGGGTACTGCAGGGTATGGATTATGGGGTTTGCTGAAAAGGCCCGACCTCT
ATATGAAAGAAGCAGAGAAAACAGAGACTGGACTTGGACTGAGCCAATGAGGCGG
GCATTTCAGAAACTTCGACAGGCGTTACTGGAAGCCCCAGCCCTTGCTCTTCTGGAC
CCGGCTAAGCCGTTTCAACTGTTTGTGGATGAAAAGCAGGGAGTAGGGAAGGGAGT
CCTGACGCAGCAATGGGGACCATGGAGACAGCCTGTGGCATATTTCTCTAAACGACT
GGATCCAGTAGCTGCGGGATGGCCACCCTGTCACCGTATCATCGCGGCCACTGCTCT
CCTTGTCCATGATGCTGACAAGCTGACTTATGGACAGCGTCTCCTGGTCTACACGCC
ACACGCCATAGAGAAAATCCTCAAACAGCCACCTAAAGAAAACCATACAGGATACA
ACCTGCACCTGCAAGGCCTGTCAGATGATGAGACCAGGAAAAGGACAGCACACAGG

TGTAAAATATCGGGGGGAAAGGCCAGGACATCATTGGGAGATAGATTTTACAGAGG
TAGACAGAATTGGACCCTGGATACATTGCAACCATGTGCGACAGGCCACTCCAGAA
GAACAAGAGAAAAAAAAAAAGAGGAAAAAAAAAAAAGAACAAGAGAGGGCTCA
AAGAAAATGGAAGTCAACACTGTATCCCTCCAATCCCTTAAAACTGAAGCTCGTCCG
ACAGCAGATCTCAGACGGATCATTCTGATTCTCCTGATGATGGCTGTCCTCCTCGAC
CCCGGAACTGCCAGCACTGACCCACATCAACCTGTCAAAATCACCTGGAGACTTCA
AAATGGGCTGATGTGAGAGGTACTCAATACGACCACTGAGATACATCCTCCAAACA
CCTGGTGGCTGGACTTATATTTCAACCTCAAGGACTCAGTGGACACCCCTTGGTCAG
TGCCTTTGATCTGACTTAGAGGTTTTTGGGCGTGTCCTGGTCATAAAAGTGACAGGG
AAACTTGTGGTGGTATGCAACATTATTTCTGTAGGTCATGGAGCTGTGTGACTTCAA
ATGATGGGGTAAGGAGATGAAAAACTAGCAACCGAGATTTAGTCAACTTCTCCTTC
ACTCAGCCCTGTGCCAAGAGATAAATTCTATAAATTCTATGATCAAGAAGATGAGAT
ACCTAAAGATGAGATTGCTCAGGTAAAATTAACGTTTAACCAGAAGTTGGCTATGCA
GGAAAAGTCTTGGGTGTCTGGATTATCTTGGGGCTTCCAGCTACAGGCTGATTGGTT
CAGTGTAAAACCTGGGGGAATCCTGATTATGAGTCAGATCATAGAACCTATCCAAA
CCCAAAGTGTGGGCCCTAATCAGATTGAAAATCTGGGCCCACAAAGGGTGTTTAAG
GCAGATCCGACTCAGAGGGCTACAGTTCTGAGCCCTACACCCTCCGTTTCCCGACCA
GGAGACCAGCCTAATATAGCAGAGACACTAGACCCCTTGTCCCCCACCTCCAACCTA
GAAATGTCAGACCCGTTGGTGGGAACTAGCTAAAGTAGCCTTTAAAGCCCTAAACC
ATTCTAACCCCAGAGCAACTCAATCCTGTTGGCTCTGCTACACTCTTCACCCTCCCTT
TTATGAAGCAATAGGCTTAGATGTTTCTTACAACCTGTCCATCAGCTCAGGTCCACA
CCAATATCACTGGAAAAAATATAGGGTCGGTCTCACTATGAGGAAAGTTTGGGGAA
GAAGACTTTGCACAGGTAAAGTTCCACTTGAAAAGACTCCCTTGTTCGCCCGAACAG
TCAATCTCACAGAACTAGACCAAGTAAAATGGATCGTACCAGCGGTAGGGGGATGG
TGGGTCTGCTCACATACCGGGCTGACTCCGTGTCTAGATGCATCGGTTTTTAACCTG
AATAGAGAGTTTTGTGTATTAGTGACTGTAATGCCCCAAATCTTCTATCACCCTGAA
AAAGTTATGTATGACTGTTGGGCCAAGGACACTATAGATCAGTTAGGGAAAAGCAG
AGTTAAGAGAGAGCCTATCACAACCGTCATGCTAGCGAACATGTTCGGTCTCAGGAT
TGCTGGGCCAGGGACAAAAATAACATCCCTGACTATGCAAAATCAGGGATTCACTT
CCCTACGAGCGGCAATAGATAAAGACATTATCGGTATAGAACAGTCAATCAGCCAC
CTTGAAGAATCCCTAACTTCACTGTCAGAAATGGTTTTGCAAAACAGAAGGGGCCTA
GACTTGATCTCCCTACACCAAGTCGGATTATGTGCGGCCTTGGGAGAAGAATGTTGC
TTTTATGCCGACCACAATGGGGTGGAGAGAGAATCCATGGCAAAAGTGAGAAAAAG
ATTAGCACAGCAAAAGAGAGAACCGGAAGCCCAGCAAGTGTGGTTCGAGTCCTGGT
TCCATCAATCTCCTTGGCTAACAACACTAATTTCTACCCTACTTGGACGACTCATTAT
ACTCCTACTCATTCTAACTTTTGGCCCATGTATTTTAAATAAATTAATAACCTTTGTA
AAAGAACAAATCAGTACCGTTCAGGTAATGGCACTGAGGCAACAATATCAGGCAGT
GTCTCAGAATGGAGAGGAAGATTCCTCTCAAGGATAAAAAGACAGGGGGGAATGTT
AGGATCAAACAGGCACCATGACAGGCTTCAGGGGGCCAAGGTAGGCCTAAGTCTCC
TTCACAGAGGGGCTGAGTTTTTGCCAAGGAGGAGCTGAGATCCTCCTCTGCCAGTGC
CAGGACTCAACCAATCAGTGTACTCTCTGTAGCCTGGTTCAAGCTTATCAGGACGAG
GATGAAACCCCCCTGAGAAAGCCTGCGCATGTGAAAATCTAGCCAATTAGTAGACG
CTAAGGAACCCTTGCAACCAATCCGCCCCTGCCAACTCCCTGTCTTTGTCTTAACCCT

ATAAATACTGCTGTAATTCAGGGCTCGGGGCCCTTGTTCCACTCCACTGCATTGGAT
GAAACTTGGGCCCTGGCTCGAGCTAGCAATAAATTCCCTTTTTGCGTTTGCATTGCTG
TAGATGTCTTGTTCTCTCAGTTCTGGGGATTGGGACCTTGGGCATAACA

ERV_10

GGACTCTGAACTTTGTACCCTGGTCTATGGTAATGGCATACCAATGGAAAACCAGAC
CCCTGGGATAAAAGAGCCTCAGGACTTGTACTTGGACTCTCCGTTGCCTAAAAGAAT
ATGCTAATTATCTCTGTAACAGAACAAAGTCATTAATTCCATTATGTTCATTGGGAT
ATGACCACAGTCCTATTGATAAATGTCCACTATTTATCTAGTCTTGTGGCACATGAAT
CATGGGCTAACTTTGATCATATCTCTCTTTTACCTTGTCCAGACTAGTTTCAAGGAAT
TTGGGGAGGTGGGTTTGAGCAAGTACACTTAGGGTATATAAGGTTTTCACAAAAACT
GGTCGAGTCCTTAGCTAAGAGGAGACTCTGCCTTGGCCCACCAGTGTAATAAAGTGC
ACTCCGCTATCTGCATTGTCCTTCTGAGTGAGTTTGTTTCCCGGAACACGTGGCTACA
ATGTTTGGTACATGGGCCAGGAATCTCCTCACTTTGAGGAGATAAGTCCCATTTACT
ACTCCGAGGCTTTGTGGCTCAGATCTTCTAGAAGGGGGAAGGCACCTCACCCTTTGG
AAGGATTCTGCTTCTCAACGCCCGGACCTATCATGTTAGCAGGTAGTGGACAGCAGC
AGGGGAACTGAACACTCAGGTGAGGAGGAACCCACCCGGCAGGGTGGAAGAGGGG
GCCTGAGCATCCCCCTGGGAGGGACTAGAAGGGGCACGGACCCACAGGAGACTGG
AATAGGCAGGCGGCGATTGCTTGGTACACAGGTCGATGAGCCTGCTAGGGCTTAGG
AAGTGAATTTGTAAAGGTCATTTAGGAGGTGTGTCCACGCCGACTTAGGGAAAATTA
TTACCAGCGATCGCCAGGAGATTTTTAGAATAGGAAACTGGTCCTTGTATGCCTGTA
TTCTGCCTTTCCCCAGGAGGTGTCCCATCTGCTGTGAAATTCTTGACCCCCTCGGAAT
TGTTAGGCTAGATAGGGGTATACATAAGTGAGTATGAATTGGCTTTTCCGGAGATGG
CCTGGGACATGGGATATTTAACCCATCTGTGTTTTCATCTGCACCTGATCAAGCCCA
CCAAGGTAGAATGAACTTTAAGGGACAAACAATCTTGGACCACGTGGTGTTCTGGTT
CATCTATGCTGACCAGCAGGTGAAGACACACCAATCCCCTTCTCCCTCTGGGACCTG
GCAGGTGAGGCTCTTCTTACCCCAGTTAGGAAGGAGGTAGAATGGCAATTTAAGTGT
CACTGAGTGGCAATACCAGAAGTACACATGGTACTGAGAACTTCATAGACAGAACT
GCTGGGGTCCAGCCCTGGTGGATCCAGGGAATTAGAAGCGGGGACAGCGTTGGCGA
GGAAAACTTATTTATTTATTAATATAAGATTAGATTAGGAAGAAATAGTGTAGTAGG
AAAATTAAGTGGAGAAAGAGGGCTGAATAACTTGGATTACGTGGAAGACCAATAAA
ATTGCAGACAAGGAATCTGCACCATCTACGTTGGGCCACCGGCTCTTGCTTGAATAT
CTAAGGGTGCCTCGCCTTAGGCTCCCTTTTGTGCGGGTTTTAACAGCCAGGGCAAGT
AAGTAGACTTAGCGAGCCTCCGCGCCCCAGATGGGAATTCAGCCTGAAATTTAAGC
AAAGAGGAGAGGGAGGGAAAGGGTAAAAAGGAGAGAGAGACACGGGGAAACCAG
TCCAGCGACCTGCTCCAGAGCCCCTATTGTCTAGAAAGGCCTTTTATACCTTTTTTTG
TACATAGAGATCAATGGGTAACACAAAGTTATGCATCGTTAGCAGTCCAGATTCTTA
TCAAAACGAGGCTTTTCTCTCTGCATACCTAATTGTATACACAAGTCTTAGGTAATTT
ACATCATCTTCTGGCCAAAAGGGCCAATTAACATTTTACAGCCTTTTTTCTGATAAG
GGTTTGTCAACCAGAAGACTTATTTGCGTTGATCCACCCAAAGTCTGGTGCCATTCT
CAGAAAGCACTAAATAAAGTTACATTCTTACATAGCAAGGACACAAGAGGAGTGCA
GTGATATATAACAAAGAAAAAAGTAATTAACTCAAAAGTCTAGTGTTGCTAACATC
AAAACTACTATATATCTTTTTTCAATCCCTTTTACATTGATTAACATCCTCCCAGGTG

CCTAAAAGATAGAGAATATGGAGGTCTGGCAGGCAATCATTAAGTCAACAGTGAAA
ACTCGTCACCAATATGATTTTTAACTCTTTAGAAAAGGCTCTGTTTCTTTAAGATGCT
TTTAAGCTTTGTGCCTCTCACAGTTGGAGGGAGGGGGGGTGTAAACCATTCACAAGC
TGTAAGAGGTCCAGGGAACCTGTTAGGCAAGCTAGAGAGCTATCAGAGGGGGTTTA
ACTGAAACATCCCTTTCAAATGCAGAAGACTAAAGCCCTGAGTTGACTTTTTTCCAG
AAAATATCAGAAGAGTGGAAAAGCAGAGTACAAAAGCCGGCAGATTTTTGTTGTTT
TTTTTGTTGTTGGGTTACATGCTCAGGAAATTCCAGGGGGACCCCTGAAGTCTGATC
ACGACCTTGCGTATGTCAGCTTCCTTCCTCATGACCTTGTCACGGGCGGAATTCCTCA
CACTGGCTTCCCGCACAGAACGAAAAGGAAAAGTAGAAGAAGGCCCGAGAAGGTA
AGCAAGATGAGGGGAAGTGAATCTAGGACAACTGTACTGGAGTGCAGGATTAAAAA
TTTAGAGAAGGGATTTGGAGGAGACTATGGGGTGAAGATGACGCCTAACCGCCTTC
ACATACTCTGTGAGGTGGAATGGCCCCCTATGGGAGTAGGACGGCCGCCAGAGGAC
ACCATGAACTTAAAAATAGAGGAAGCAGTCTATACAGCAGTCACAGGAGAGCCAGG
ACACCCAGATCAATATCCATATAGGGACTCATGGCTGGGTTTAGCTCAAGACCCTCC
TACTTGGACAAGGTTCTGTATCCAGGAGGGAAAGGGAAAAATATCAATGGCACAAA
AATTGACTGATGATAGAAAAGGAAATTCTATGGGATGTGGACGGGGATGAACTGAC
CCCTCCCCCGTACTGGACAATGACGCGCCCACTGGCCCAGTGCTCGACCAGGATCGG
AGGCTGCCTTAATGCCCGATCCAGGGCCAGGTGAAGTCCTGCAGCAGCCGCTGCTCC
TCCGCCAGCTCTCCCCGAGGTCACAGAGCCACCACCTTGGCAGGCTCCGATCCTAGT
GACAGAAGCCTCTGGCCAGCACTTCCAGGATCCGGCTTCAGCCAGACCGCCCAAGC
TATACCCACCTCTCCTGGTGAGTACTGACAAGAAGGGGAGGGAGACATTGGAATTA
AGCAGAGACTGCGCTCTCCCAGAGCACCGGAGGGAACCAAAGAGAACAGACAAGA
GATTTGCAATGCTAGCTATCTATCTCGGGCCCTCCGTTAAACCTCCTTTGCCCCGGGG
ACAGGACAGTCCTTCAACTGGTCCCAACAGAGACCCCAGGCCCAAAACCATTGTGC
CCAGTGCCGAGCTTTTGGCCATTGGAAGAATGAATGCCCTAAGGTAGGGAAAGAAG
AGGAAGCTCCCCCAGTTGTGGGGCTTGCTGACTTGGAAATTAAATAGGGCTGCCGG
GGCTCAGAGATACCAGGTCCCGGAGAGCCCATGGTAACCTTAAAAGCAGGGGACCA
AAACATTGACTTCATGGAGGATACAGGAGCAGAACTGTCAGTAGTAAAACCTGTGG
CACCACTGTCCAAAAAGACTACCGCTGTAACTGGGGTATCGGGAGAAGAGAGGATT
AAATCGTTTTGCCAGCCCAGAAAATGTCAGATGGGGGGGCACCAAGTGATTCATGA
ATTCCTCTACATTCCTGAGTGCCCAGTACCCCTGTTGGGAAGAGACTTGTTCTCCAA
ACTAGGAGCACAAGTGACTTTCTCCCCTGAGGAGAGGCCCACCTTCTGGATGGACA
CTTCGACTTATTTGCTCTCTTTCTCAAGAGCCCCCCAAGATGAGTGGAGGTTGAATG
AGCCTCTGAAGGAAGAACCGGGTGGGCCGGAAGAGCAAGAGATAGAGCTAACTCA
ATTATTCCCCGAGGTCTGGGCTAAAGACAAGCCCCCTGCCCCCTAGTCTGGCTAAAC
ATCAAGCCCCAGTGATAATAGAACACAAACCAGGCACCATCCTGTTTAGAAAGCAC
CAGTACCCGCTACCGATAGACGCATGGGCCGGCATACTGCCCCACATCAATAGACT
GAAACAGGCGGGCATTCTAGTAGAGTGCCAATCGGCTTGGAATACGCCAATCCTGC
CAGTCAGAAAGGAAGGAGGACAGGGCTATAGGCCTGTACAGGATCTCAGGCTAGTC
AACCAGGCTGCTGTGACTTTACACCCCACTGTTCCAAACCCCTGTACCTTACTGAGC
CTCCTCCCACCGAGGACTCAAGTTTATACCCGCCTGGATCTCAAGGATGGCTTCTTC
TGCGTATGCCTCGCCCCAGCGTCACAGCCCATCTTTGCCTTTGAATGGGAAGATCCA
TTGGGGGCACCAAACCACAGCTCACCTGGACTCCCCCACAGGGGTTTAAGAACTCC

CCAGCCATCTTCAGGGAAGCCTTGGCTTCTGGCCTGGACTCATTCCATCTGGAAGAG
TATGGATGTTGGCTCCTACAATATGGGGATGGCCTGCTGCTGGCCGCTGAGACCAAG
GAAAAGTGCTGGGAAGGGACAAAAGCACTGCTCCAGCTGCTGATGGAAGCAGGTTC
CCGGGTGTTGAAGAAGGAGGCACAGATCCGCAGGGAGGAGGTAAGGTCGCTGGGG
TTTGTTTTAAGGAAGGACACAAGGGTCCAGACCCTAGTTGGGTCCTATACACTGATG
GCACCAGCCTGATACAACAAGGACAACAGCCGTCAGGTTAGCCAAAGCGGAAGGG
CCGTCAAGACTGAAAAGGGGTGGTGGGAATTGCCAAGTGGCAAATTATTGGTACCA
AAGGAGCTGGCACACACTCTGGTAGCCAAACACACCAAGCGACCCGCCTAGGCCAG
GCTGCCTGCTCACAGGTTAACGCTGCCTCTCGGCTATTCAGACAAAAACCTCCGGGC
ATTCAGCTGAAAGGCACGCTGCCCTTTGAACACCTGGGAGTGGACTTCACTGAAATG
AAACCTCACCGACACTACTGTTACCTGCTGGTCAAGGTGTGTACGTTCTCAGGATGC
GTAGAAGCTTTTCCTACCTGGACTGAAGGAGCATCAGAAGTAGCCCACTGCCTGCTT
AGGGAAATAGTTCCCAGATTTGGACTTCCTACCAGCATTGGTTCAGACAATGGTCCG
GCTTAAAGTAGCTGATTTAGTACAACAAGTAAACAAAACTTTACACATCAAATGGA
AACCGCACACTGAATATAGGCCCAGAGACGGTGGAATGAACCAACCGGACATTAGA
GAGACTCTCCAAGTGGATCATAGAGACTGACTGCTCCTGGGTGGACTGGCTTCCGAC
GGCTCTGCTCAGACTCAGGATGACCCCACAGTCCCAAGGCTATTCTCCATACGAAAT
TGTGTATGGGAGGCTCCCTCCCATAATAATACAGGTGTCAACAAATTTGCCTCAGGT
AAGGGGGGATAGGATTTCACAGCAGATGGAACTGGGTAAGGTAATAAATCGGGTAA
CTAAGTTTGTACAAGAAAGGGTGCTGTTCCCCCTTGGGGAACAGATTCATGAGTTTA
CGCTTGGTGACCAAGTACGGGTCAAATATTGGACACATGATCTGCTAGCCCCTTGGT
GAAAGGGCCCTCATGTTATTCTAACTACCCCTACTGCAGTTAAAGTGGCAGGTACTG
CCCCTTGGATCCATCATACGAGGGTGAAGAGGACATACCGCGCAGACCCAAAAAAC
GCTGAGTGGACTGCACAAAGGGACCCCGCTGACCCTCGAGAGACTAAGACCATCCT
TAAGAAGAAGGGAAAGAAGACCCTGGACGAGCCCCTTCAGGATAAAGCTGCACAAT
CAGCTCCTGCTGCTTGGCTCATCAACGTGATTTTGATTTTAACTTCCCTTTCAACTCA
GGACAAAGTTTTCATCTCATGGGCACATTCCTACGCAGACTTCCGCAACACTTCCAG
CTGCTGGTATGTGGGGCTATGCCTCTGACAGTGATGGAGGGTCTTCCTTGGTGGGTG
TCACCGCTCTGCCAAGGAGATTTTAAACCACTCTGCTCTTTTCTGGGATGACAAAAA
GAGACTTTCCTCTCTCTTGTCAATCATAACCTCTCCTTGCTCTCTTGGTGTAAGACCT
ACAGTCAGTCAATAGACTCGGGTCATGGGGTTACATTTGACACAAATGCCAGTGTAA
CAAAAGACTAACCTACAACAAGCCCCGGTAAATCTACCTGATTTACATGCTAGGTGG
ACAAGATCTGTGTTTCAATGGTCCCTTATTCGTACCCTCTATCGGGACAACAGATATT
ATGATTAAAGTAGAGGCCTTGACTAATTTTACAAAACAGGCCCTCCTAGATAGAACA
ATAGCCATCCAAGCCTTAAATGAAAGCAAATCCAAAATGAGAAAAGCGGTAATTCA
TAATGGAATGGCTTTGGACATACTCACAGCTGCTCAAGGAGGGACCTGTGCCATAAT
TAAGGTTGAATGTTGTGTATACAGTCCTGACTTATCTGGCAATTTTCGCCTGCTTTAG
ATGACATGAAAAACCAGGTACAAGCAATGTCAAATGAAAACCTTCCTTTCTGGACTT
CGGTCCTATCTTGGGTGAAGGGCGATTGGTGGAAAACTATATTAGCCACTGTTGTAG
TTGCCTTGATAGTTCTGCTTTGTGGACCCTGAATTTTACAATGTATTATGAACTTTGT
AACCCAAAGGTTGATGTCATTCTCCCAAATTGGCGGTCGGAGAGCCAGGGTGCAAT
ATATCCCTATGAGTGATGCTCATAATATGAGTTAAGAGCATCAAGAGGGGGGAATG
AAGGAGGAAACAGACAGAGCTGGACTCTGCCTCAGGCCAGGCTGCGAACATTAGGC

TACACGCATGGTTCCCCTCCACCATAGGACTCTGAACTGTGTGCCAGGCCTAGAGAA
ATGGCATACCAATGGGAAACCAGATCGCCCTGGATAAAAGAGCCTCAGGACTTGTA
CTTAACTCTCCATTGCCTAAAAGAATATGCTAATTATCTCTGTAATAGAACAAAGTC
ATAAATTCCATTATGTTTATCGGATTATGACCACAGTCCTATTGATAAATATCCACTG
TTTATCTAGTCTTGTGACACATGAATCATGGGCTAACTTTGATCAATCTCTCTTTTAC
CTTGTCCAGACTAGTTTCAAGGAATTTGGGGAGGTGGGTTTGAGCGAGTGCACTTAG
GGTATATAAGGTTTTCACAAAAACTGGTCGAGTCCTCGGCTAAGAGGAGACTCTGCC
TGGGCC

ERV_11

CAGTCACCACTTTTACTTCTGTGATCTTGCTTGCTCCTTACATCCATGGGAATTAGGG
CTGCTGCGGAGGGGAGGGATCTGAGCTGTTTGAGTGGGAAGTTTACAGTGTAGGAA
AGATATCCAGAACAAAGCCGGGGCTCCCAGCCTTTGGAGGTGACTCCGCTGGGCCT
GCACCAGTGCTGAATAAACCCTGCTGTTCTGTATCTCTGAGTGATTCTTGTGTCTTTC
CGACACTATGGTTTCTATAATATTTTCTGCTGCTTTGGCCAGGAAGCTGACAACCGA
ATTGGCCCCTTGCACCACCATTGTCAGGGAACCAGAGAAAGCTGTGGCCGCCGACC
TGGTGATAGACGGATAGGCACCACCCGGCCATTTGCTGGAGCCCGATTCTCACTCAA
GTCGCGCTGGCCTTGGCCCATGCTGCTTCCCCAACAGACTCCGAAGGAGAGCAGAA
GCGGGTTCCAGTGCAGGACGTCAGATGGGTGAGTGCCCTGGGGACATCTGCCTGAG
TCAGGACCCACCATATGGCAGAAGAGGCACCTGATCACCTCCCAGTGACCAGAGAG
TCACTCAGAATAGGGTATGTTCCCTCGGGTGGTCTGTCAGTTCTGGTGTGTGTGGGA
GAATGATTGGGCGGAGTGAAAGCCAGCGCTCCACCGTCTTTGGACTATGGAGCCTG
AGAGGGTCCTAACCTGTGGTTCCACAATGAGCTCATTCAGTTCAGGGCAGTGCCAGC
CTCTGGGGGATTTAATCTGTCCTTGCGAGGCTGGTCTGCGTTGGGAGTTTATATTGAC
CTGCCCATGCTAGGTGGTATCTAAATTTCCATATGGGGAGCAGCCAGATGGATGAAA
ATGAGTGTCTTTGTTTTGTTGTGGGACACCACCTCAGGGTGGGAACTTTCACATAAC
GGGCAACTCGATTTCTAAACTCACTATCTTAGAGTGTATGGTTAAAAATTTTAAGAA
AGGATTTTTCTGGGGATCAGGCTTCCCTCATAGCTCAGTTGGTAAAGAATCTGCCTG
CAATGCAGGAGACCCCAATTCTCTGCCTGGGTTGGGAAGATCTGCTGGAGAAGGGA
TAGGCTACCCACTCCAGTATTCTTGGGCTTCCCTTGTGGCTCAGCTGGTGAAGAATCT
GCCTGCAATGCAGGAGACCTGGGTTCATATCCCTGGGTTGGGAAGAGCCCCTGGAG
AAGGGAAAGGCTACCCACTCCAGTAATCTGGCCTGGAGAATTCCATGGACTGTACA
GTCCATGGGGATCGAAAAGAGTCAGACATGACTAAGTGACTTTAACTTTTCCTGGAG
ATTATGGTGTAAAATTGAGTCCTGAAAAACTTTACAGTCTTTGTACCCTGGCGTAGC
CCTCCTTTGGGGTCGGTGACCCCCAGAAGGTGCCTTAGATATACCCACTGTCCGAGT
AGTGTCATAGCGGGAGACTCCAGCCACCTGCACCAATTCCTATACACCAATTAATGG
TTGGAAATTGCCCAGTGAGGGCCCCCTTGGGTTCGATTCTATGTCTCAGGTCAGGGA
TAGTGTAAAGTCCTAGTAGTTCAATCAAAGAAAAGGATCTCGTGCCAAGAGAAGGA
ACCACCTATCCTCCAGGGAGAGGCTGAAGAGCTGCCTCCAGTGTCCCCACCTTACGT
CCCCACGGCTCCTGCACCCAGAGCAGCAGATCCCCATTTACCAGACAGCCCACCGCC
TTTCATTCCTTCTCCTCCTGACGTTCCTGAAGCTGTTGTGCCCCCTCGGCCAGCCATC
CCAGAACCAATGGGTAGGCGCCTCTGGTCGGCTCAGGGAGCAGCTGACCCAGCACT
ACAAATGCCCTTACCAGAGACTCAAGGGCTGCAGCACTTTGCCACCGATAGGATCCT

ACATGAGGGAGGACCCGTTTTTTACTACCAGCCTTTTTCCATGACTGATTCCCTTAGC
TGGAAACATCACACCACCTCTGCTCTGAAAAGCCCCAAGGGCTGGTTGATCTCTTAG
AATCCACCTTCCAGATCCACCACCCTTCCTGAGGAACCCGCCAACAGCTTCTCCTCA
CCCTATTCAATACAGAGGAGCACCGAAGTCACGACAGAGGCTGGGAAGCGGCTCTG
GGCCAATGCCCCAGGGGGCCAGTTAGGCGCAGAGAGCTGGGATCGGGAGAATTTCC
CGGAGGAAGAGCCTTGCTGGGCCCCAATACCGAGGGAGGGGAATGAACCAGTTGGA
GAGGTATCGACAGGCCCTCCTACAGGGACTAAGAGCCAGGGTTAAGAAGCCCACTA
ACATGGCTAAGACCAGTGAGGTGCTACAAAAGCCAGATGAAAGCCCGGCCGATTTC
TATAAAAGGCTATGTGAAGCCTTTCGGGTCTTCACCATTTTGATCCTGAAGCCCCTG
AATATCAACGGTTGATAAATGCAGCCTTTGTAGGACAGGCCCAATATGACATCCAG
AAAAAAGTTACAGAAGTTAGAAGGGTCTGCAGGTAAAAATGCCACAGAACTGCTAG
AAATAGCTAACAAAGTCTTTGTCAATCGCAACCAAAAAGCACGCCGAGAAGCAGAA
AAGAGGATGAAGCAAAAGCGGCACTCTTGGCAGCTGCCCTGTCAAAGCCTTCCCCC
ACCCCACGCCCCTACCTTGGGACCACCCCATAAGGCAAGGGGCCCAGATCCGAAAG
GGAGCGTTCCCCTCAGCCGTGATCACTGTGACTATTGCAAGGAAAAGGGACACTGG
AAAAATGAGTGCCCTAGCACCCTGAGAAGAAAACCAAAGCCCCAGGCTCCCTGAGC
TGATATCAAACCAAGCCACCCAGCGCTAATCTGATTGGGCTAGCTGGAGCAAAATC
CGACTGGGGGGACCGGGCTCTCTCCAACTGCGCCCCCAAGAGCCCATGGTCAGAAT
TCAAGTAGGGGGCCATCCTATGGACTTTATGGAGGACACAAGTGATGAGCATTCAG
CGGTCACCCAGCAGACAGCCCCCTCTCGGGAAAAGAAGCTACCATCCTCGGGGCCG
CAGGTGCCCAGACCCACAGGCCATTCTGCAGTCCCTGACAGTGCCTCCTAGGGGACC
ATGTGATAATGCACGAATTCCTCTACCTGCTCAACTGCCCCGTTCCCTTGTTGGCAAG
AGAGCTGTTTGCAGAGATGGGGGCCCAGATTTCCTTTTCTGCTGATGGATCACCTCA
GCTAAAGCTAGCCCCACCGCCTTCCTCCTTGATTACGACTCTCATTATAAGGAGAGA
AGAAGAATGGCACCTGTATTCCTCCCCTCCAGGAGAAGGGACCATTCCTCCTGAATT
AGAGACTGAGTGCCCGCTAGTTTGGGTGGAAGAAAATCCTCTGGTTCTGGCAAAAC
ACCATGCTCCCATCCTGGTTGATCTGAAACTGGGAGCTCAGCCTGTAAAACTACAAC
AGTATCCAATTTCCCGGGAGGCCTGACTGGGAATCCAGACTTATTCAGACAGACTAC
TCCAACACAGACTTCTGATGAAATGCCAGTCACCCTGGAACACCCTGGAACACCCC
ATTACTACCTGTGAAGAAGCCAGGGACTCATGACTACCATCTAGTTAAGGACTTAAG
AGCTGTGAATGAGGCAGCGATTACTCTGCATCTGGCAGTGCTTAATCTGTACACCCT
ACTAGCACTGATCCCGTCCCCAGCTGAATGGTTTACTTGCTTGGACCTGAAAGATGC
CTTCTTCTGCCTCTGGGTTGCACCTGTCAGCCAATCGCTATTTGCATTTAAATGGGAA
AACCCACACATGGGAGACAAGGAACAGCTCACTTGGACGCGGCTTCCTCAGGGGTT
CAAGAACTCCTCTACCCTCTTCAGCAGAGCTTTGACTGTCAACTTGGCTAACTTTCCT
GGACAGGAGTTAGACTGTGTCCTGCTGCAATATGTCAATGACCTCACGCTGGCCAGG
ACAACACAGGCTTGCTGCCCAGAAGGAACAAAGGCCCTCCTCTCCTTGTTAATAGA
AGCTGGATACCAGGTATCAAAAGAAAAAGGCACTCCTGAGAGACATTGTCCCCAGA
TGTGGAATGCCTCTGACCATAGAGTCAGACAATGGACAGGCATTTGTAGCCGAGAC
AGTACAATAGACGGCAAGGGCTTTGAAGATTCAGCGGAAATTACATACTGCCTACC
TACCCCAGCGCAGAAGGTGATGACACCCCACTCCAGTGCTCTTGCCTGGAAAATCCC
TTGGATGGAGGAGCCTGGAAGGCTGCAGTCCATGGAGTCGCTGAGGGTCGGACACA
ACTGAGCGACTTTACTTTCACTTTTCACTTTCATGCATTGGAGAAGGAAATGGCAAC

CCACTCCAGTGTTCTTGCCTGGAGAATCCCAGGGACAGGGAAGCCTGATGGGCTGC
CATCTATGGGGTCACACAGAGTCGGACATGACTGAAGTGACTTAGCAGCAGTACCT
GCCCCAGAGCTCAGGGAAAGTGGAATGCGAGAATCGAACCCTCAAACAAACCCTAG
CAAAACTATGCCAAGGGAACAGCTTACCTTGGGTAGGCATGTTACCCATGGCCCTCT
TGAAGGTGAGGTATCCACCCTGGGCAGGGATAGGGTTTTCACCATTAGAAATCCTGT
ATGGATGGCCACCCCAGCTAGTCAACCTAAGGGGAGACACCAGAGAACTGGGGAAC
TTAGACGTATCTAGGCAGCTGCAAGGGTTATGACACACAATCTCTCAAATACATACG
TGGAGAGTTGATAGAATACCAACCCTTAGGCATAGCAGTACATCCCCACCAACCAG
GGGACCAAGCATGGGTGAAGGACTGGAAAAAAAGAGCCTTTAGAACCCTTGTGGAA
AGGGCCCTATTCTATAATCTTAACTGCTCACTGGTCTCAAGGTGACAGGAATAGACG
CTTGGATCCATTACTCCAGAGTCAAACCTACAAGTCTGACCAATAGCTGAGGGGAGT
GGGAAGCAGCCCTCAGTCTGGAGGAGCCCCTGGGCTTGATCTTGTGAAGGAGGAAA
CAGCCACGCCGAAGCCCTGCTCGGACCACACTGGAAGCTGGTCAGTCAACGCATGG
CTGAAGCGTGAGGAACCCACTGGACTGACAAAGGCAGATATATTGGGTATTAACTG
GACTGGGGCTCGCCCTGCTCATCTTATGCAGAATAAAGCTTTACTTTTTCTCTCCCCT
GGAGTGGACTTGTATCCAAAAACTCTCCCTCTGTGTTGTCTGCTTCTTCATAGTTCTT
GATTTTTGGAGTTTTCTCCTACTGTGATCAACTTAAATATGAAAATGACGTATTTTTG
CCTCATAGTCCTGTTAAGTTGTAAAATCCCCTCCTCATCTGAGAGTCAGGATGATTGT
TCGGAATGTATAGAATCTTTCTATTATGAGAAAGGGTGGGTCTGCTGTGCCTTTGCTT
ATTATAAGCACCAGTTGACCGCATGTCATAGTTCAGCCACTACACGCACTAGAGAAG
GGAAACATTGTTGGAAGGGAGGAATTAAAGCCCAAAATATAGATGGCCAAAGATGT
TACAGGTTTCCAGGACAAGCAACTTGTTGGAACCTAGGCAAAGAGAAAACAAAAAT
TGACGTCGACCTAGGAAAATGGCCTATTGAGTATATCTGGCTGGACCCCGACCCCTT
AGTATCCACCCCTGGCCCACCGACATTAAGCCCTGGGCCAGGATTCAAGGGACTAT
ATGATCAACTCATTTCCCTAAAAGATGAACCAAAAGTGCCTACTGTTGAAAATAATC
TCTTTATCCATTTAGCCGAGCAGGTTACCTGAGAATTGGGTATCACTAACTGCTGGG
TTTGTGGAGGAGCCCTGATGAGTGAAACATGGCCCTGGAAGGGCACCAGACTGGAC
GCCTTTCTGCTACTGCAATGGGATCAAACAATTAGTAGGCAGGCAGGAGGTCCTATT
TGGGGTTGGCCCTGTCCTCAGAGACGATAGGCAAAAAATGCTTGAGCCGAGAAAGG
ATGTTATACCTGGATCAGAAAAACAAAATGCAGAGGAACCCTGGTCTATAGTGACA
CCAGTAACAGCCTTTTGTGGTGACCAGGGGCCCCAAGCTGGTATTGGGCTCCCCAAA
AAGGAGGGTATAGAAATCACACTTGCATTCCCTTCAACTCCACCACCAGGATTCTAG
TGTAAAACTGTACAGGAAACCAACCTGGTATTAATCCCTTTCTTGCAATCCCAGGCA
TTAGCCCATATTGGAAACATCTTAAATCCACAAACCCAGACTTATGGCAGGCCCCAG
AGGGTCTCCTTTAGATCTGTGGGAGAAGGGCCTACTCATGACTCCCATCCAGATAGG
GAGGGGAGGAACCTGCACCATAGGAATGATTCAGCCAGATTTTTTCCTCCTACTTGG
CTCACAAGGAGACACGCTTGGTGTCCCAATCTATGATGACTTAAAATGTTGAGAAAG
GCGGTCTCTAGAGATAGGAGGCCATCACCCCGAAAATGACTGGCCTCCCCAGTGAA
TACTCGATATTATGGTCCAGCCACAAAGGCACAAGATGGGAGTTGGGGATACAGGA
CACCCATTTATGTGCTAAATCGAATAATCTGACTACAAGTGATGCTACAGATTATAA
CCAATGAAACTGCTGCTGCCTTGGAACTGCTGGCACAGCAACAATCCCAGATGAAA
ACTGCCATCATTCAAGATCACTTGGCACTAGACTACCTCCTAGCAGAGGAAGGAGG
AGTCTGTGGGAAATTCAGTCAAACTGACTGTTACCTCCAAACAGATGATAATGGAA

AAGCTGTGATAGACATAGCTAAACATAGCAGGAAGATTGCCCATGTTCAGTTCAGA
CCTGGAAGGGATGGGATTCAGATGGCCTCTTTGGAGGATGGTTCTCTTGGCACGGGG
GGTTTAAAACAATGATTGGGATGGTCATAGTCATACTTGCTGGAGGTCTGCTTATTC
CCTGCCTCATATCCCTCCTAATCAGAGTTGTAACAGGGTTCATAGAGGCAGTTGTCC
CACGGAGGACGGCCACCCAGTTGCTGCTTTTAAAGGAATACCAGCAAGTGCCAGAA
GATGATGTTCTGTGACAGCTGCAGATGCATCCTAAGCATCAAGATGGGGGGATGAG
GTAGGAACGAAATCAAAACCCCCAATTAAAGTGACAGTTGCTTGCTTTCCCTTCCCT
TATCTTGTTTTTAAACAGCTGCTGCTCATCAATCACCACTTTCACTTCTGTGATCTTG
CTTGCTCCTTACATCCATGGGAATTAGGGCTGCTGGGGAGGGGAGGGATATGAGCT
GTTTGAGTGGGAAGTTTACGGTGTAGGAAAGATACCCAGAGCAAAGCGGGGGCGCC
CAGCCTTGG

ERV_12

CAGTATGTTATACTCCGATATCGTTCCTCTAATCTATGTAAAGGACACTATTTGTATG
GTGCTCTGCCCTTCTTCAAGATTCAAGCTAATCCTTTTATGGCCCAAGATGAACCATT
TGGAGCCAAGATTATCCCAAACTACATCCTATGGATGAGGGGCCTGGTTCCATTCTA
AATTTTGAGACATTTCTTTCTTTCATTAACAGACTGCTGGTGACTATATAACATCCAG
CTGAAGACTAGCAGGGGGGTACTCTTTCTGCCCCCTGCTGATGCCTATGTCAGAAGC
TTTCTCTATCTCTTTTATGCTTTAATAAAACTTTGGAACCCAAAAGCTCTGATCGATC
AAGCCTCGTCTCTGGCCCCGGATTTTATTCTTCTCCTCTGGGGACCAAGAATCCTGGC
GTCGTAATTCAACAACAACCTTTCATCTTGGGGACTCGTCCGGGATCCTTCAGGACA
AGGTAAGGATGCTTGGAGCTCTAGTTCTTTGTTCTCTTAGCAAACACATTTTCTGCTG
TGCTTTACTAACTCTACGGTGTGCTTGTGTGAATGAATGACATGCCCTGCGCGAGGC
AAGTGAGGAGCCCTGCTCTGCGGTTCCACGGTGATCTCATACGGCTTATGGCAGAAA
CCTGTCGGGGGTTATACCGACCTGCCAAGGCCAAGAGGCACCCAGTGTCTCCTTTGG
GAACCGACCAGAAATGGGCAAAGCGTGTGGACCGAACTCTCCTTTCTCGGTCAAAC
TTTCCGGTCTCTTTGACCTTTTCATAACTCCTTGGGAATTAGAAGTACTAACCTAATC
TATCGGATCATAGACTTTCCAGGGACTTGTGATCTATACTGTTACTGTGTACTGTGGC
AAACTTGGATTGGTAGTCAAGAAAGCGCCTAGCCTCGCTAGGAATCACAAGCTCAG
AAGCTAGATGGAGCTCTGGCCCCAAGAACATCTCCGAGGTTGAAGGTTACTCAGATT
GGGACTGCAATGGGTTTTTTTCCTTTGGTAACGCTGGCTCTTAGTGGACCAGAGGAG
GCTCTCATACTGGTGTGGTGATGCTTGCAAAGAACATCCCAGCTTGATGTTCGTATC
GGTCTTATTGTGGTCAGGACTCAGGGTCGTGCACAGGCACTCAGGTGATGAATGTTT
CCCCCAGCGGCCTTAGCCTGGGAGGCATTCCGGAAGGTGACTCTGATTGCACCCCGG
GTGGCATCAGAGGCAAACAAGGTTAAAGGTGAAGAGCTGGACATCAGGTAGAGAT
GCTAGCAGGTCTCCCCCTGGTACATCCCCACCCCGTCTCGGTGGTAGAACCGAGAGG
GTCAAGACGGCACTGCATCGGTAAGGGAGAGACTAAGTCCGACCAGGAAGGAAAA
GCTTTTGGTGTAAAGTCTGTCTACACCCCCATCTAGAGCAGGGAGGGACGCCTCCGG
TAGAAAAATGGCACTGGTCGCTTTTTTCTCTCTTACAGATGGGAGCTAACAATGCCA
GCCTCACTCCTTTGAACTGTATCCTGAAAAACACTGGGATAGATTCGATCCCCAGGC
CTTAAAGAAGACACACCTGGTCTTCCTATGCGATACTGCATGGCCACGGTATCCATT
GGAGGATGGCGAACGGTGGCCAGTTGGAGGGTCTCTTAAGTATAATACTGTTTTACA
ATTAGACCGGTTCTGTAAGGAACAAAGGAAAAGTGATCTTAAAAGATAGATTTATC

ACTCACTTGGCTCCAGATATCCTCCATAAGCTACAAAAATGGGCGAATGGACCAAAT
CAGTCTTTAGATACTCTGTTACAACTGGCTTAGATGGTCTATTACGGTAGGGAATAT
GAGGAAAAGAAAGAAAGGCAAAGAAAGACAAAGGAAAAGGCGGAAGCCTTCGCC
ATGGCTATGGACCCAGGTGAAAAGAGAAGGGCTTGCTATTACTGCGGAAAGGAGGG
GCACCTCAAGCGGGATTGCCCTCAGGCATCTAAGCCGGCCCGGCTCCAAGTCCGGTC
TGCAAAGGACCACACTGGAAGAGAGACTGCCCTCAGAGGCGTAGGTCTCCGGGGTC
GGACTCTCAAGACAATCAGGACTGAAGGTGCCCGGGGGTCCCCACACAAGCTCCCG
TCCTAATTACACCTGAGGAACCCTGGGTATTAATAATTGTGGGGGGCCAATCCGTCG
ATTTCCTTCTAGATACTGGGGCAACTTACTCTGTGCTTACTGAAGCCCCTGGCCCACT
TTCTTCCCGATCCGCTTCCGTAATGGGACTGTCTGGATGAGCCAAAAGGTATTATTTC
AGTTATTCTTTATCTTGCACCTGGGATTCTGTGCTGTTTTCACACAAGTTTCTGATCA
TGCCAGAATCTCCCTCACCCCTTTTGGGGAGGGATATACTGAGCAAGGTCCATGCCT
CTGTTTTCATGAATATGGAGCCCTTTCTTTCTCTACCTTTAATAGAACAAAATGTAAA
TCCTAGAGTCTGGGCTGATGGAAAATCTGTGGGTCGAGCACAAAATGCTATTCCTGT
AGTTGTCAAGCTCAAAGACCCACACTTATTTCCACATAAGAAGCAGTATCCTCTGAA
ACCTGAGGTTAAGGAAGGGTTAAAACCCATCAATGAAAATTTAAAGGAACAGGGAC
TATTAATTCCCTGTAACAGTCCTTGCAACACTCCTATTTTGGGTATAAAGAAATCAA
GTGGTAAATGGAGACTTGTCCAAGATTTACGTATAATAAATGAGGTTGTAGTTCCTT
TACACCCCGTGGTGCCTAATCCTTATACTCTATTGTCTGAAATTCCTGAATGAGCCAA
ATATTTCTCAGTAATTGATTTAAAAGATGTCTTCTATTTAGTGCCTTTGGCAGAGAAA
AGTCAATTTCTATTTGCCTTTGAAGACCCTACGCAGCCAGCTTCTCAGTTAACCTGGA
CAGTTTTGCCCCAGGGATTTCATGACAGTCCTCACTTATTTGGACAAAGTTTGTCACG
GGATCTACAAAACTTTAACAGCTCTGAAGCAGTGGTGTTACAATATGTAGATGATAT
TTTGCTCTGTGCTGAGACAGAGGAAGCTTGTTCGCGAGCCTCAGAAGATTTCTTAAA
CTTTCTGGCAGGCTGTGGTTACAAGGCATCAAGAGAAAAGGCTCAGCTTTGTCAACA
ATCAGTTAGATATCTGGGCCTAATCATATCAGAAGGGACTAGGGCCATAGGCCCTG
AGAGAATTAAGCCTATACTAAATCATCCCCTACCTATGACTTTAAGACAATTGAGAG
GATTTTTGGGAATCACAGGTTACTGTCACATTTGGATTCCGGGTTATGGGGAACTTG
CCCTGCCTTTATATAAACTTATAGCTGAAACTAACTCAGCAGGCCCAAACTGACAAA
CTGGTTTGGTCACCAGAAACTCAAAAGGCTTTTAAGGTTCTTCAGACTGCTCTCCTG
CAAGCTCCTGCTCTGAGCTTGCCCACAGGGTCAGAACTTAATCTGCAGCTGAAAGGA
AAGGTGTGGCCTTGGGAGTTTTGACACAACCCCGAGGGCCTCACCAGCAACCTATTT
CTTATCTAAGCAGAGAATTAGATGTAATTTCACCTGGGTGGCCCCACTGCCTAAGAG
TAATTGGGGCAGCGGCTTTATTAGCACCTGAAGCTTTAAAAATAATTATTGGATGAA
ACCTTACTGTACTGACTTCTCATGATGTGAGTGGAATCTTAAATTCTAAGGTTAATAT
TTGGATGACAGACAGTAGGCTTCTTAAATATCAGTCATTGTTGTTAAAAGGACCAGT
AACTAAGCTTAAAGTTCGTGGAAATTTAAATCCAGCCGCTTTCCTTCCTGAGAAGGA
AAATAAAACACCTGATCACGATTGTTCTCAATTCCTAACTTTAAACTATGCAGCTCG
GGAAGATCTAATGGATACCCCATTAGACAATCCTGACCTGAAAATATTTACAGATGG
CAGTTCTTTTGTTCAAGATGGAAAGCGTAAAGCAGGTTATGCCGTGGCGACTACTGA
ACAGGTTTTGGAAGCAAAATCTCTCCCCCAGGGAACCGGTGCTCAGTTAGCAGAGCT
TGTGGCTCTGACCCGAGCTCTAGAGTTAAGCAAAGGGCAGCAGGTAAATATCTACA
CAGATTCTAAATATGCTTATTTGACTTTACATGCTCATGCTGCAATATGAAAAGAAA

GTTTAAAACAGCAACAGGTGAACCTATTAAGCATTTCAGAGAGATCGATGCTATATA
TTGTCCTAAAGAAGTAGCTGTTATGCATTGCAAAGGGCACAGCAGGGATGGGAGTA
AAGTAGCTGAAGGTAATCAGTTGGCTGACTCTCAAGCCAGAAAAGCGGCACTTTAC
GGAAACCCCTTCACTGCAGACGCCTTTGATCTGGACAGGTTCTGTGGAACAGGAAA
AACCACAATACACTGAGGAAGAATTAGAAAGGTATGAAAAAAGAGGAGCACAGAT
TACTGATAAAGGATGGTTACAGTCTGAGGATGGACGATTAATAATTCCTGAAAATGC
TCAATGGAAAATTCTTAAGGGTTTACATCAGAGTTTTCATTTGGGTCCAGAGTACTT
ACCAAATGGCTTCTCGTTTGTTTGAAGGTAAAAATGTAATGAAAACTTTAAAGAATA
TAATCAAAAGGTGTGAAGTTTGTCAGAAAAATAACCCAAAGACTGAAAAACTAGCA
AAATCTGGATTACAAAGAAGTGGGAAGTATCTTGGAGAGGACTGGGAATCGATTTT
ACTCATATGCCAAAAGCTAATGGATATTCTTGCTTATAAGTTTGGGTAGATACTTTTA
CTGGATGGATTAAAGCTTTTGCCTGTCGCAGTGAACAGGCTAAGGAGGTTATAAAG
ATTTTAATCCATGAAATTATCCCCAGGTTTGGGCTGCCACGGAGCCTTCAGAGTGAC
AATGGCTCTGCCTTTAAAGTTGCTGTAACTCAGGGGGTATCTAAAGCTCTAGGAATA
GAATATCACTTACACTGTTCCTGGAGACCCCAATCCTCAGGAAAGGTTGAAAAAGCT
AATGATACTATCAAAAGACATCTGCGCAAATTAACTCAAGAGACGCAGGACAATTG
GATTAAAGTCCTACCCATAGCTTTAATGAGGGCTTGAACTGCCCCCAGAAAGGAGG
GACTGTCCCCCTTTGAATGTATTTATGGAAGGACTTTCTTATGCACAGACATTGTTAT
AGACCCTGAAGCCTTGGAATTAACTAGTTATGTAACTCAGCTCTCAGCTTTTCAACA
GGCATTAACAGAACTCCGGGAGATGACTCCTGACCCCGCCTCTGAGTCAAGCAAGC
CTCTATTTGAGCCAGGAACTGAGGTCCTCATAAAAACTTTGGGATCTGGGGGCCCAT
CCCTCGAGCCCCTTTGGGAAGGCCCTTACCAGGTTATTCTTTCTTCTCCCACAGCTGT
CAAAGTGCCAGGAATTGATTCGTGGGTACATCACACTCGAGTAAAGAGGTGGCACC
CTGACCAGAACTAAGTGACATCTTTTTATGTCTTTATGTTCTATGCTCTGACTTTGTA
CTTTTCAGATGGGCCTGATAACCTATGTGAGCTTACTTCTGCTGACTCCAAATATCCT
GAGTCTGCCGTTTGATCCTCCAGACAATGCCTTCCTGTCCTGGGCTCACTCCTACGCT
GCATTCCACAATCGGTCTAACTGCTGGGTCTGTGGAACACTCCCCTCTTCATCAGTG
GAAGGCTTCCCGTGGTGGGCATCTCCACTTCAAGGAAAAGACTTTCGCCAAGTGTGT
AAATACCTTCGACAACAATTACATGCGATGCCTCTTCTTCATCTGATGACACCTACC
AACCCTAAAATAGACTGGTGCAGCACTTTGTACTTTAACTATATGGACATAATGTGA
CTTTTAATTTTGATTATACATTGTCTCAGTTCAATGACTATTTTGCTACACATAAGGC
AATTAGGTCTAGATCTAATGGTTTTTTACCTGACGTTTATCAAATATGGGATGAGGTT
ATGTGGCTAACTCCTGAAAGAGGACGTTTAATATCTACTGCCCCTATATGCTGGGAA
CAAACAGAGCCATCCCCAAGAGTTAGCCAACGACTTAATTACAATGATTGGAAACA
ATTGGGATTTTTGCCTCAAGAAAGATGCAATGTAATCATTCCCATGTTTTCCGACCCC
AGTTCAGGTTCTCTTTGTCTGGCCAGGCACTGATCGGGACTGGATATCTCAGTCACG
CTGCCTTGCTCCAAATGGGACTTATTGGATATGTGGCTCTTACCTATGGGCATGGCTT
CCCCCTGGTTGGATAGGGAGATGCACCCTGGGTCTAGCCTTTACTCTCAGCTTTATAT
TTTCAGAGCTCCCAGAAAAGCCTGCTAATTTACCCCACCTTAAAACTCGGTGGGCAA
GGTCCGTATTTCACTGGGATGATTATTTGGCTGCAGTGTTTGTTCCCTCTTTGGGAAC
TACAGATGTTATGCTACAAGTGGATGCTTTGACTAATTTCACTCAACAGGCATTACA
AGATTCTCAAAAGGCTATTTCAGCTCTTAATGCTAAATAAACACAAATTAAAAAGGT
GGTTTTACAAAACAGATTGGCTTTAGATATTCTGACAGCTGCACAAGGGGGAACGTG

TGCTATTATTCATACCCGATGCTGTCCCTATATACCTGATAGGAGCACGAAGGTTAC
TCATTTTACTAAACACATGAACAGGATGACTGGGGCCATGGCTACTCCTGAAGCCTC
AATTGCCTCACTTTGGGAGACATTAAGTAGTTCCCCATGGTGGACAACTATCTTAAT
TACAATAATTCTGATTGTTTTGTTTTTATTGTTTGCTCTCTGCATCTGTAAGTGTATAA
CTGGATTTGTTTCTAGCCTCATGAAAGCTTTCAAGTTACAAATGGTTGCTCAAACTCC
TGCTACTGTTGCAGCTGCCTCCAGCTACTATTTGGGGCCCCTGGATCAGATATCCTCA
ATATAAGGATTAGGAGAATATGTTGCCTCCCCAATTTAGGGACAACACCCCTTCTCA
GCTCCGAAGCAGTTATGGAACGAAAACGGTGCCCATTTTCCCTAGGCAACATAATTC
TCCTGAAAGAAAAGGGGGGAATGAGAGGGTAACAGGCAGGAAGGCCAGGGGTCTC
CAAATGGAGGAAATAGCCTGCAAGTGTCAGACTTTTTTCTCTCTCTTAAGCAGCAGG
AGGAAACAAAGTAGCGATATTTTTTCCTTCTCTATACAAATTTAAAAGGAGGTTTTT
CTCTTAAAATGCTGTGTTGCCACGACATCTGGTTTCACCTGAAGTTAACCAATGCCTT
TTTCTTATGGAAATGTTTATCTTAAGCTATGGTAATGTACTATGCATTTACCCCAAAC
TCTGTCTTCAAGTCGGTTCCGCCTTTTGGCTCAGTATGTTATACTCCGATAATCTATA
CTTCCTCTAATCTATGTAAAGGACACTATTTGTATGGTGCTCTGCCCTTCTTCAAGAT
TCAAGCTAATCCTTTTATGGCCCAAGATGAACCATTTGGAGCCAAGATTATCCCAAA
ATACATCTTATGGGTGAGGGGCCTGGTTCCATTCTAAGTTTTGAGACATTCCTTTCTT
TCATTAACAGACTGCTGGTGTCTATATAACATCCAGCTAAAGACTAGCAGGGGGTAC
TCTTTCTGCCCCCTGCTGATGCCTATGTCAGAAGCTTTCTCTATCTCCTTTATACTTTA
ATAAAACTTTGGTACACAAAAGCTCTGAGTGATCAAGCCTCGTCTCTGGCCCCGGAT
T

ERV_13

TCTCCCCCTCTCCCTCCCCCATGCACTCCTCCACTCTCTTCTCTTCAAGTCTTTGGGTT
GGCATGCCCTCACCCTTCGAGGATGGAGTCTCCTGCTATCTTCTAAATAAAATAGAG
CTGTAACACTGATTTACCTGAGAGCTATAACACAGTTTGTCCAAGACCCGAGAGCTG
TGATGCACCGAGGGCTTTAATGTCTGTCGCTCCAAATCTTTGTTGTGATGAGACAAA
GAACCGAGGAACATACACTTGCGTGACATCTATGGTGCTGTGACTCGGATATAACCT
GGCTGAAACAACCTCCGCGTGGAAGAGGCCAGGCACAGCAGGAGCCCAACTCAGC
GAAGCTCCCGCAGTAGAGGCGGAAGGTGAAGAAAATCCAGCGCAGGGGAAGGCCC
ATCATGCTGGAAACCGGAACAACGGAAAACAAACTCAGCGGAAAGCTCACGTGGCC
CAGTCTCAGATCCCAGAAGACCTCTGGTTAAGGTAAGAGGTCCTCACTGGAGGGAC
ATGCCTAATGAAATCTTAATTCCTTTACAGTCTCTCGTTTTTTGTTCCCGCAAACCTC
CTGCGAACAGGCGGGCGGCAGGGGGCACAACTGAGGGACTCTGGAGAGGCTGCTCC
TCAGCATGTCTCAAAGGCACTATCTGCTGAGCCCCAGTAGCTGTTACACAAGCCAGT
GGGGGTTCTTCTGTCCTTTCTCTTCTCTGTGCCAAGGATCAGACCTATGAAATTGTGA
GCACCGGTCAGATATTTAGCAAATTTTCCAGCGGGCTATGAAGGGGATTCCTTGGCA
CGTTTTTCCCACTGCTTTTTCCTCCTGTCCTTCAGCTCTCTCCCAGGACTCAAGCTCG
GCCAAAACTAATGAAACTCAGGCCCTGATGTCTCATTGCAAAAATTCATTGAGAGAC
AAAGAGATAAGAGGTGGATTTGTTAGGATTCAGAGAGAAGCCACTCTTCAGGGTGT
GAACCATTGCCAAGGGCAAGGGCTGGGGCCACGGATTAGGCCTGGCTAGGTTTTG
TGAAGGGGTGGAATTCATATGCTAATGAGTGGGAGGATCATCCCTACCATTGGGGA
ACCACCCACTCCTCCCTCTTTTGCCTTGGAGCTGTCCTGCCACCTCTGGGTGTGTCTG

TTGGCTTATAGATTGGGGATTAAGTACTTGAATTTCACTTTTCATCTTGGACCCAGTT
GGTTTTAATTGGTTTACATTATCCCCTTGTGCTATGTCATTCTTTCAAATGTTGTGCTC
TGCCCCTTTCCCTCCTGTTTCATGCTCTTTGCCTGAGCCCCATCCAGACCCACAAGGT
TGCCTCTACGATCTTCTGGAGAGACAACCAGAAAATAGATGGCCTTGGGAGCGAAA
TACTGTATAATATCCAACCCCCTAATCCTACCCAGTACTGGTCACACTGGAGATCTT
GGGAACGCCCAACTCCATCAGTCCAGGGGCCCCAGGAGGTCATCACGCCTTGACCT
GCCTAGGATCTGGTCCTCGAAATGTTGTCATGCCTTAACCTGCTCGGGGATCCGCTA
GTCCCAGGGGTCCCAGGAGGTCGTCACGCCTCGACCTGCCCAGGGATTTGATCTCTA
GGAGGCTGTCACGCCTCAGCCTGCCTGGGGATCTGCACCCTGACCTGGGGACGCCTG
GCTCTCAGGTTACAACAGTACTAGGTAGGATGAGCTTACATCATATCTATTCCCATC
CACGGTGGGCAAACTAGCAACGAGTTACAAACCCCTTAATTCTATCCAGCATTGGTC
ATGCTGGAGACCTTGGGGATGCCCAAACTCCAGTCCGGGGGTCCCAGGAGGTCATC
ACGCCTTGACCTGCCTAGGGATCTGGTCCTCGAAAAGTTATCACGCCTCAACCTGCT
CGGGGATCCGCCAGTCCCAGGGGTCCCAGGAGGTCGTCATGCCTCAACCTGCCCAG
GGACCCAATTCTCGGGAGGCCGTCATGCCCTGACCTGCCCGGGGATTCGATCTCTAG
GAGGCTGTCACGCCTCAGCCTGCCTGGGGATCTGCACCCCTGACCTGGGGATGCCTG
GCTCTCAGGTTGCAACAGTTCTGAGTAGGATAATCTTCAGAAAAACTCACCCCTAAG
GAAGTCCACCCATGGAAACAGAAGGAAGCCTATTACTTGTGGGACTGTGCCCAGTC
TCAGCAATAACTCAGGAGCCCGGTGAGAACCCCATTGCCTTTCTGGAAAGGCTGAA
AGAGGCACTCCAAAAGTTTACCAATCTGGACTTAGACTCTTACGAGGGACAGGTGA
TTTTAAAGGAAAAATTCCTGTCCCAATGTGCATCAGATATCAGAATTAAGTTACAAC
AACTACAACAGCAGGACCCTGCTGCCTCTTTAGATGAGATGGTCCAGACAGCCACC
AATACCTTTTATAACAGAGAACAGGAGAAGGAGGCCAAGGCCCAGGAGAAGGAGG
GAAAGAAAGAGACAAGCCATGCCCAGATGCTGGCTGCCCTCCAGAGAAGCCCTATT
GCAAACCCCGAGTCCTTGAAGGACAAGGCATGAGACAAATGCCTGATCTGTAGACA
GGCGGGGCATTGGGCCAAAGTGTCCAAACCATGACAAGTCTCCTAAAACAGCTTGC
CACAAATGGCATCAACTGGGACATTGGGCGGCACTCTGCCCTCGGGACCCAAGAGC
CTCAAGGTCAAGTGCCAAGCCTACCCTCACGATGGTTCAACAAGACTGAAGCGGCC
TGCTCCAGCCAGCCCACCTGTCACAGATAACCATCACGGGGCTGGAGCCAAGGGTG
CAACTGGATGTGGCAGGTAGGTCCGAGAATTTCTTGGTTGACACAGGGGCTGCCTAC
TCTGTCTTGATCTCCTACTCCGGACGCTTCTCCTCCCAAACCTGTACCATTTTGGGTG
CTACAGGAAAAGCAACTACTAAAAGATTCACCCGAGCACTTATTTGTTGCTGGGATG
GACAAATATTTTCCCACCAGTTTCTGGTGGTCCCTGAGTATCCTACTCCCTTATTGGG
AAGATATATATTCACTAAACTGGGGACCACCCTTGTGATGGGAAGTTTTTCAGCCCC
TAGAGCTCTACAGCTCCTGGTTACTACTGAGGAACCCATTACACTTTCAATAGAGAG
GGACCAAAAACCATAGGAAGACAAAATTAACCCCCAGGTGTGGGACCAGGGGATTC
CCAGACGAGCTTACCAAGCCGAAACAGTCATCATTGTCCTCCGAGATCCCACTCGGT
TTCCTAACCAGAAACAATACCCACTCAAAAGAGAGGCTCAGAAGGGACAGCCTTTA
ATAAATAAATTCCTTGCTTGTGGGCTATTGGTCCCCACCAGCTCGCCATGTAACACT
CCAATCCTCTCAGTAAAGAAAAAAGACGGAACCTGGTGAATGGTTCAAGATCTCCA
GATCGTAAATGAAGCTGTAGTCCCCCTCCGTCCCACAGTACCCAATCCCTATGTAAT
CTTGGGAGAAATCCCACCCAGTGCCAAGTGGTTTACAATCTTGGATCTCAAAGATGC
ATTTTTTGCATACCACTGGCTAAACAATCCCAATATCTTTTTGCCTTTGAGTGGGAGG

CCCCAGGAGAAAAACGCCAACAGATGACTTGGACAGTATTACCTTGGGGTTCAGAG
ATAGCACCCACCTGTTTGGACAGGCCCTTAGCCAGGATCTCCTAGATCTGGACCTGG
GACCTAATGGAAAAATATTACAATACGTAGATGACCTACTAATCTGCTCTCCAGATG
AGAAAAGTGCCCAACAACATGCAATTCAGGTTCTAAACTTCTTGGCAGAAAGGGGA
TATAAAGTCTCCCGTGCTAAGGCACAGATGGTCGAGACAAAGGTCATTTACCTGGG
AGTTCAGATTACACACGGGTCCAGGAGGCTGTCCTCTGATCGGGTACAAGGAATCCT
CCAGTTGCTCTCCCCCACGACTTGAAAACAATTGCGAGCTTTCCTGGGACTAACTGG
ATATTATAGAATCTGGATACCCAACTATGGTCTAATTGCCCAGCCCTTATATGAAAG
CTTAAAGGGGTGAGACGATTCAATCCCACTGATGTGGGGAACTCCTCAAAAGAAGG
CAGAGGCTACACTAAAACAGGCCTTAACTCAGGCACCTGCCTTGAGGTTGCCAGAC
CCAGAAAAAGCATTCCAACCTTATGTCCATGAAAGAGAGGGAATAGCTTTGGGAGT
GTTAACTCAAAGGTTGGGATCTGAGCCCCAGCCTGTAGCTTACTTATCCAAAAAGCT
TGATCCAACTACCTGAGGCTGGCCCCCCTGCCTTCAAAATCTTGCAGCTATTGCAAT
CATGATAGAAGATGCTTTAAAACTCTCCTTTGGGGGCAAACTAACTATTTTTACCAG
CCACCAAGTAAAACAACTCTTAAATGGAAGAGGCCATTTATGGATGTCTGGGTCGCT
AAGAGTTGGGCGTGACTGAGCGACTTTACTTTCACTTTTCACTTTCATGCATTGGAG
GAGGAAATGGCAACCCACTCCAGTATTCTTGCCTGGGGAATCCCAGGGACAGAGGA
ACCTAGTGGGCTGCCATCTATGGGGTCGCACAGAGTTGGACACGACTGAAGCGACT
TAGCAGCAGCAGCAGCAGATCAAAGAATCCTCAGATATCAAGTAATGCTGATGGAA
AATCCAGGCCTCACTATATCCCCTTGTGAGGGTCTTAACCCAGCCGCCCTCATGCCT
ACCCCTGAGGGCTCTCTCCCCTTTCACTCATGTCTAGAAACCTTGGACCACTGGACA
AAACCCTGAGAGGGATTGTCAGAAGATCCTCTGACCAATCCTGAGGAAATCTGGTA
CACTGATGGAAGCAGCTTTGTCTTGGATGGAAAAAGAAGAGCCAGGTATGCAGTAG
TCTCCAATTTTGAGACCATAGAGGCTAAGCCTCTGTCACCAGGTACTTCAGCCCAGT
TAGCTAATCTCATAGCCCTGACTCGAGCTTTAGAGCTGGGAAAAGGAAAAAGAATA
GCCATTTACACTGACTCCAAGTATGTCTGCCTGGTGCTACATGCACATGCTGCTATTT
GGAAAGAAAGGGGCCACTTGACCACCCGAGGGTCCCCAATCAAATATGGTGATCAG
ATTCTTTGACTCTTGGAGGCAGTCCATCTGCCCACTGAGGTTTCAGTCTCCCACCGTA
AAGGACATGAGGGAACCAAGCAGCCAATCAGGCAGCTAGGAGAGCAGCATTACAG
AACCATGACCTAACAGGGGTTGCCACCTTAGTTCCACAGACTAATTTGCCAGAAACT
CCTTCCTATACTGAAGGTGAGACTCTCAAAGCTAAGAGCGAGGGCTTCCAAGAAGA
TCATATGAGGTGGCTCCAAAAGGAGGGACTCCTTTTTCTGCCTGGGAACCTCCAATG
GAAGTTGGTTAACTCCTTACATGCCACTACTCATTTAGGAGAAAAGGCCCTCCAAAG
ATTACTAGAAAGGTCCTTCAGAGGAACAGGCTTCCAAACAACTATAAGACAGGTGG
TCTGTTGTCCCACTTGCCAATTAAACAACCCCCAAGGAGCTCGAAGACCCCAGCTGG
CCCAGCCCATCCAACGACATGGGGCCTACCCAGGAGAGGACTGGCAGATGGACTTC
ACCCAGATGCCAGTTTCTCAAGGGTATAAATACCTATTAGTCATGATAGATACATTC
ACAGGATGGATTGAAGCTTTCCCACCCAGACTGAGAAGGCTGAGGAGGTGATATAA
AAACTGCTCCATGAAATCATTCCAAGATTTGGTCTGCCCAGGTCATTACAAAGTGAC
AATGGGACATCATTTACTTCCAAGGTCACCCAAGGGGTCTCAAAAGCATTGGGCATT
ACTTATTATCTCCATTGTGCCTAGCCAATCAATTCTTAAAATCAGCTATAAAAAAGA
TAACCCAGGAGACCTCCCTGGGGTGGAAGGAGGCTTTACCGATAGCTCCTCCGCAC
CCGCATTGCCCCTAAGGAACAGGCTGGTCTTGGTCCTTATGAGATGCTATATGGGAG

ACCTTTTGTTTATGTCAATGACCTCTTCCTAGATCCAGAGGCTCAGACCCTCCAGTCT
TATACCATGGCCATTGGGCAATTCCAACAGGATATACAGTTGTGGGGTATGAACCAG
CACCCAAAAGATTATAAGGAGTCACCACTATATGCTCTGGGGACTCAAGTCCTAATT
AAAGTCTGGAAAGATGGGTCCCCAAAAGCTCAACTCCAGCCCACATGGAAGGGCCC
CTACCCTGTAATACTTTCTACCCCCACAGAAGTCAGGGTACCGGGACATGACTCCTG
GATTCACTACCATGAGTCAAGCCATGGAAGAAGACAGAAGAGGACAGTCAATACAC
CTGTGAGCCCCTCGGAGATCTCAGATACCTATTCAGGACTACCAATGAGTGCCATTC
TAATGAACACCCCCCAAATCTGGTTTCTGGGGATAAGATTTCTCAGGATAACTCTAA
ACAGCCAACATAGCTTGACAGAGATTGTACTCCAAAACAGACAAGAGATAGATCTT
CTGATCCCTGAACAAGGAGGGACTTGAGTCATCCTGGCGATGTGAATTTAAAACTGA
CTTTCATGTTTGGGAATGCATGTAAGAATTAAAGATTTTAAAATTTAAAAAATAATA
ATAAATTTAAAAAAAACTGACTAATGCCCCTACTAGTCCTTGTTACGCTACATTGAT
GATGCTTATGATTATTCCATGAACTGTCAATTGTCTAACCTGTCTTGTCTCTGCCTAG
GTCAACAAGCTACAACATGCAGTGCCAGTTCAACAAAGATATAAAACTACAGCCGA
CCATGAAAAATATCACACCCTTAGATGGACACCGCTATAAGCACTCTGAGGCTTGAG
ACTAACAAGAGGGGGAGGCCCAATACCCCTCACCGCCCCAGTTCAGCAGGAAGTAG
CCAGAAAGACCTCGACGCCCCTATTCCCAAAGAATTGGGCCTCCCATCTCTTGAGGG
GGGAATGTTAGGTAGGTAGAATAGGGAAAAGGAGTCCAAAATGGCGGTGGCTAAA
AGACAAGGAAGGGAAAAGCCCGCGAAAATAGAACAAAAGAAGGTCCGAGGACCAG
AGTGAAGACTTCAGGTAGAACAAACAGCACTCCTGGCTAAGCCCAGTTTGCACAGG
GCAGGCCCAGGTGGAGGAAAACATAAAAGGAGGAGCCAAAGCGCTTTCTCTCTCTC
TTTCTCTCCTGCATGCATGTGCTCTTTCCCTCTCTCTCTCTCCCCCTCTCCCTCCCCCA
TGCACTTCTCCACTCTCTTCTCTTCAAGTCTTTGGGTTGGCATGCCCTCACCCTTCGA
GGATGGATTCTCCTGCTATCTTCTAAATAAAATACAGCTGTAACACTGATGTACCTA
AGAGCTATAACACGGTTTGTCCAAGACCCGAGAGCTGTGATGCACCGAGGGCTTTA
ATGTCCGTCACTCCAAATCTTTGTCATGACGAGACAAAGAACCGAGGAACATACACT
CGGGTGACA

ERV_14

TGAAGAATCTAAGCTACTCCATTTTGTTAACAGAAAAACTCCATTTTGTAAGGTTCC
AGGAAAAGAGCCTTTGGAAATCCCCTGACCTCACCCCACCACCCATGAGCCAATCG
GACCCCAGACCAGAATCTCCTGACAATGTTCAGGAACTTGTGGTCTACCTAGGTAAT
AGGGACCAATCAAAAGCCAACACACAACCCTTCGAAAGAAAGTGACCAATCAGAC
GTCCCCCTACCTAGAAATTCTTTTTTTCATGAACACTCCCTACATAAGCAATGTAATC
CAAAACCCGGGGCTCCTCCCTATAGCCGCTGCGTTGGTAACGGTGGGAGCCCCAGCT
CGAGCTTGGTGATAAAGACTCTCTTGCTTTTGCATTGGATATTGGCTCCCTGGTGGTC
ACTGGGAGATTTCGCGACTTGGGCATAACATTTGGGGGCTCGTCTGGGATCTCCCAC
TGGCTCCATGGGAAGACCAATCCGGAGAGTCATCTATGGCCGGTAAGCTTTTTTTCT
TTCTTTCTTTTCGTTTCTGACAGGGCCTATTTTCTGAGACCAGTATATGACCCAGGTG
GACGCGCTGACTGGTCACCGGTGTGGGTCATTGGGAGATGTCCCCGACCCCCTCGGA
GGGGATGGAGTGCCCCTGTACTCTGTAGACAATAGTGGGTCGCTCCTGCTGAACGGC
TGGAGGCCGTCATCTTACTTTCAGTTTTGCCTCCAGACTCCCGGAGGTCTGTTTCTCC
TGTCTCTCTGTCTGTCTGTGTGGCTGTTTGTATTGTGGTATTGTCTGTGTTAATTTACC

AACATCTGACCGATCCAGGAAAGATGGGGCAACATCAATCTAGCCCTACTCCTCTAT
CTCTGATCACTGACCATTTTAAGGAGGTCAAGATGCACGCTCATGACCTCAGTGTAG
AAATTAAAAGGAATAAATTAATCACTTTTTTCAGCTCAGAATGGCTGACCTTCCATG
TAGGCTGGCCCTCAGAAGGCACCTTTGACTTGGGAACTGTACACCAAGTCCAGGATA
TCACCTTCTGACCACGGATAGGACACCCTGACCAAGTTCCCTATATCATAGTATGGG
AAAATATCATACTGCACCCCCTCCTTAGGTAAAACCTTTTTTGCCTCAACAGGGATTT
TCTACTGCTTAGGGGTTAGTGACACGTGTAGGAAAGAACCTGAAGAATCCCAAGGA
GCCTGAAGCAACGGCACCGCTATACCCCATCTTGCAGGGGGGAACAGAAGAAGAAC
TCCTCTTCCCTCCCCCTTACCAACCCCTTGAACCACCATGGGCCCCTGCCATCCCTCC
ACCCAGGGCCGAAACACAGGAAGGGGGTCCCGCACAAAATACTCGCCATAGGAGA
GCCATGTCCCTGGAGGGGCCAGCAGACTCAACAGTGGCCCTCCTCTTACGAGTGGCC
AGCCCCCCCGATGAGGAAGGAAACCAACCTCATCAATATTGACCCTTCTCTACTAGT
GACCTATATAACTGGAGAGCCCAGAATGCTAAGTTCTCAGACAACCCTAGAGATCT
AACTAACCTTCTAGAGACTGTGCTTTTTATTCATCAGCCCACCTGGGACGACTGCCA
ACAGCTGCTCCAGATTCTCTTCACCACAGGAGAGAGGGAGAGAATACAGAATGAGG
CCCGTAAAAGGGTCCCAGGGGCAAATGGAGAACCCACCACTAATAGAGATGAAATT
AACACCTCTTTTCCTTTATCGCGACCTGACTGGGATTACAATATGGCACAAGGTAAG
GAGAGGCTCCGGGTCTACCGCCAGACTCTTTTGGGGGGGCTTAAGGCAGCTGCACG
GAAACCTACTAACCTAGCTAGAGTAGGAGATGTTCAACAAGGCCCCACTGAGAGTC
CCGCCGCTTATCTGGAAAGGCTGGTGGAGGTCTCTAGACAATACACCCCCATGGATC
CAGAAGCAGAAGGCACTCAGGCTACTTTGATAATGCATTTTGTCAACCAGGCAGCCC
CCAACATTAGGAAGAAGTTGCCAAAACTAGAATGCCTGGGTGAAAAGAGTATCCAG
GATTTAATAACAGTAGCAGAAAGAGTCTAGAACACCCAAGAAACCCCTGAGGAAAA
ACAAGCCAAAGCAGCAGATCGCCAAACCCGTAACATGGAATGCATCCTGCTGGCTG
CAACAGTTCCTGACTCGGGAGAGAGGGAGCGCCAGTTGTGCCGCCTGGCTGCTGAA
GGTAAGAGCTGTCCCCGTATGCGACCAGCATTAGGAAAGAACCAATGTGCCTATTGT
AAAGAGAAGGGACATTGGGCCAAGGACTGCCCTAAGAAAAAGAAGGAACCATGAA
AGACCCCCATCCTGGCCATCGAAGAACTGAGCGATTAGGGAGGGCAGGGTTTGGCG
CCCCTCCCCAAACCCAGGGTAACCCTAAGAGTGGAGGGGACCCCAATTGACTTCCTT
GTTGATACGGGGGCTCAATGTTCGGTTTTGCTAGAGCCCCAGGGAAAACTGGATGG
AAAGACCTCTTGGGTGCAGGGGGCTACTGGAATGAAACAATACCAATGGACTACCC
AAAGATCAGTGGACATGGGTGCAGGCCGGGTATCCAACTCTTTCATGGTCATTTCGG
AATGCCCCTTCCCTCTGTTGGGAAGAGACCTGTTGACCAAAATGGGGGCCCAGATTC
ACTTCCTCCCGGGGGAAACTAAAATACCTGACCAGTTGGGCAGACCGATTCAAGTA
CTGACAATTCAATTAGAAGATGAATATCGGTTGCACCAAAAACTGATGCTCCCCGAA
GCGGACATTCAAAAATGGCTAGACGAATTCCCTGATGCGTGGGCTGAGACAAGGGG
AACCGGTTTGGCTGGACACCGTCCTCCTATATATATAGATCTCAAGCTGGGAGCTTT
TTGCCTTTGAATGGTCTGACCCAGAAGAAGGAATCAACGGCCAGCTGACCTGGACTC
GGCTGCCACAGGGATTTAAGAATTCTCCCACAATCTTCGATGAGGCCCTCCATGAGG
ACCTGGGTGAGTTCAGATAACAACACCCCCAACTAACCTTGTTACAATATGTAGATG
ACCTCTTAATTGCAGCAAAGGACCAACAGACTGGCCTTATGGGCACCCGAGAACTA
TCGTAGACCCTTGGAAAATTAGGGTACCGAGCCTCAGCCAAAAAGGCTCAGATATG
CCAACCAGAGGTCACCTACTTAGGGTATGTATTAAGGGAAGGACAGAGATGGCTGT

CAGAGGCACGAAAGGAGACGGTACTCAAAATTCCTATCCCAGATTCACCTCGAAGG
GTGAGAGAATTTTTGGGGTCTGCTGGTTTCTGCCATCTTTGGATCCCAAATTATACAG
AACTGGCTACACCTTTATATGAAGCCACAAAAAGTACCACCCCTTTCAGTTGGACAG
AGCAGATGGAGACCACTTTCAAGACTATTAAAACAGCCCAGCTGTCAGCCCCCACC
CTAAGGATGCCTGACGTTACAAAACCCTTTCTCCTGTATGTAGATGAAAAACAAGGA
GTGGCAAAGGGGGTACTGGTGCAACACCTGGGACCTTGGAAGTGGCCAGTGGCTTA
CTTGTCTAAACGCCTAGACCCCGTGGCGTCAGGCTGGCCCCCATGCCTCCGCATGAT
TGCAGCAGTGTCCCTAATGGTCAAGGATGCAGATAAACTGACACTGGGGCAAGAGT
TGCAGGTCACCACCCCACACGCCATTGAGGGTGTCCTAAAACAACCCCCAGATAGG
GGGATCAGCAATGCTCGGCTGACCCACTACCAGGGATGACTGCTAAACCCCTCCAG
AGTCATCTTTCTGCAGCCTACTGCTCTCAATCTGGCTACACTGCTTCCTAAGCTGGAT
TTGGAAGCCCCAATCCATGACGGCAGCGACATACTAGTCCAGGTACATGGGACGCA
AGAGGACTTGCAAGATCGCCCTCTAGCGGATGCTGAAGTTACCTGGTATACGGAAG
GAAGCAGTTTCGTCGGAGATGGGCTCAGGTATGCAGGGGCAGCCATAACCACGGAA
ACCTAAATTGTGTGGGTAGAAGCACTACCTCCGGGCACTTCGGCTCAAAGGGCTGA
ACTAATTGCCCTAGCAAAAGCCCTCCAGCTGGGGAAGGACAAAAAACTTAACATCA
TTACTGATAGCCGATACGCCTTTGCTACTGCCCATATACACAGAGCCATCTACAGAG
AGAGAGGACTCCTCACAGCCAAGGGTAAAACGATCAAAAATAAAGAAGAAATAAA
AGCCCTCCTCTCAGCATTATGGCTTCCTAAAAAACTAGCCATAATACACTGCCCGGG
ACACCAAAAGTCAGACACCCTGACCTCAAGGAGAAACAATTTAGCAGACAGAGCAG
CCCGAAATGCAGCCCAGGGCACCATGATAGAAGCCACCCTTCAATTGCCCGACCCC
AGGAGCTCTGTTCTGCCAGCTTTACCCAACTACTCACCAAGAGACTTAGACTGGATA
AAAAGTTTACCTATGACCCAACAACTGGCCGGGTGGTGTCAGGCAGTGGACAGCTC
CCTGATCCTCCCGGAGGAACTAGGAAAGCAGGTGTTACTGAGGATGCATCACGCCA
CTCACCTGGGAACCCGCAAGATGCAGGACCTGATTAGACATGCCAAGATTACCATG
AGAGACATCAGATCGACAATAGAAAATATCGTATCAACCTGCAAGGCCTGCCAGCT
CACCAACACTGCCCGTCATCCAGCCAACCATGGGTCTAGAGAACGCAGGAGTCGGC
CAGGAGCATACTGGGAGGTGGACTTCACTGAGGTAAAACTGGGTAAATATCGATAC
AAATATCTACTGGTATTCATAGATACATTCTCAGGGTGGGTAGAAGCCTTCCCCACC
AAGAGAGAAACAGCACAGGTGGTGGCCAAAAAGCTGATTGAAGACATCTTGCCACG
GTTCGGGTTTCCTGCACAGGTGGGGTCAGACAATGGGCCAGCCTTCGTATCTCAGGT
AAGCCAGGGGGTAGCCCAGGCTTTGGGGGCTAGGTGGAAGTTACATTGTGCTTATA
GGCCCCAGAGCTCAGGACAAGTAGAAAGGATGAATAGGACACTCAAAGAAACATT
AACAAAACTAGTTGCTGAGACTGGCAGGGACTGGGTGGCTCTTCGCTTTACCGGGTG
CGAAACTCCCCCTATCAACTGGGACTTACCTCCTTCGAAATTATGTATGGGATACCT
CCCCCAATAATTCTAACCTAAAAACAGAGGTGCTAAAAGAAATAGATGATCAAAGA
TTACTCTTTTGTCTCCAGTCCTTACAATACTCCTACAGGGACACGTGGAAGAGGCTC
AAAGCATTATATGAATCAGGCCCACCAACCTGAATCCCACCGCTACCGACAAGGAG
ACTGGGTGTACATGCGCTGCCATTGTCAAGAGACCCTAGAGAAGAGCCCAGGTGGA
AAGGGCCCTTCCTAGTTGTCTTGACGACATCCACTGCTCTAAAAGTTGACGGCATAT
CCACTTGGGTTCACTACACCCACGTATGGCCTGCGGACCTGTTCTCCCTAAGGGAAG
AATTCCTTCCCCAATGGAAGACCAAGCTGGACAAAACAAATCCACTCAAACTCAAG
CTACAAAGGCGATAAAATTACTGTTTATGCTGTTGTCCCTACAGCTTACTGCTGCCG

CTAACCCTCATCAACCGATGAACCTGACTTGGATGATTCTAAGTGCAACTACAGGAG
AAGTAATTAAGTCCACCTCGACCATTCATCCCAAAAACACGTGGTGGCCAGACTTGG
AATTCGACCTCTGTCTTCTGGCCGCTGGATCTTGGGACATTAGCGAATGGGAGGTAA
AGACACCCGGCAAGCCTGAATGTGGGGCTGGCACTAATAGGTGCAATACTCGGCCC
CCTACTAACTCAGGACCTGGATGCAGCCACTACATTCAATGGGCAAGCTTACGGGA
AACACCCTTCTATGTGTGGCCTGAAGGGAGATGGAACCGGGCGACCCTCAATAACT
GCGGGGTGCTGACAAGTTTTACTGTGCCACTTGGGGATGCGAGTCAACGGGGATGG
TCGATTGGGAGCCCCCTATTCGTGGTGACTTGATTACCTTGGGTCGCGTTCCGGGGC
CCACTTGGTCAACTGGGGGACATGGGAGGGAGTCCCTGGCCAAGATAAGAGAAGGG
CTGAGTCAGAGAAAAAGAGAAAGAAAAAAGTCTCAAAATTGGTTTGAATCCTGGTT
CAACTCATCCCCCTGGTTCGCAACTCTCATTTCTTCCTTAGTGGGGCCCCTGATTATC
TTGTTACTGCTACTCACCTTCAGGCCATACTTACTAAACAAATTGGTGGCCTTCATTA
AAAGCCGCATCAACACGGTGCAGCTCATGGTACTCAGATCCCAATATACGGCCCTG
CCAATGGTCCCCTTAGTGGGAGACAATATAGAGCTGACCCCAGACCCATGATTGGGT
CTGTCCTGGATCCTAGAGAAGGGGGGAATGAAGAATCTAAGCTACTCCATTTTGTTA
ACAGAAAAACTCCATTTTGTAAGGTTCCAGGAAAAGAGCCTTTGGAAATCCCCTGAC
CTCACCCCAACACCCAAAGCCAATCGGACCCTAGACCAGAATCTCCTGACTTAACGT
TCAGGAACTTGTGGTCTATCTAGGTAATAGGGACCAATCAAAAGCCAATACATAAC
CCTTCGAAAGAAAGTGACCAATAAGACGTCCCCCTACCTAGAAATTCTTTTTTCATG
AACACTCCCTACATAAGCAATGTAATCCAAAACCTGGGACTCCTCCCTATAGCCTCT
GTGTCAGTAACGGTGGGAGCCCCAGCTCGAGCTTGGTAATAAAGACTCTCTTGCTTT
TGCATCGGATATCGGCTCCCTAGTGGTCATTGGGAGATTTCAC

ERV_15

GTCAGGGCAAGGTCAGGATGAAAGGTCAGGGCAAGGTCAGGCATGTCGGGACATGT
CCCGGCAGAGGGCGCTGCAGACAAGCCCCGGAGACAGGCCGGCAACCAAGCCAAC
CAATCAGGAGCTGACACGAAGCCCTCGCCGTCCAATCAGGAACCGACACGGAGCCC
TTGGACACCAATCACCGCTGAGCCCGCGTTTTCTTCCTTATATGGGAGCCCCTGCCC
GGGCTATAAAATCCTTCCCCACCCTCACAGACTCCGCAGACTCCCTTTACCCCGCAG
ACTCCCTTTGCTTCGCAGACCCCCCTCGCTCCCTTGCTTACCCGCCCCGGGAGTTCTG
CCCGAGAGCGACCGCCCAATAAAAGGCCCTGATCAACGGTCTATAGGGGTGGCTCC
TTCTTCCTGCGGCGTTTCTTACATCTGGCGCCCAACGTGGGGCTTGAGGTGAGGGCT
TCCGGCACTCGCCATAGAGGCCCCCTCGAGCTCCACCACCGCGGTAGCCCCCCCGAC
CCAGGCGGCTGACCAACCCCCCAGATGGCAGGATACGGGGTAAGTCCCTTCGGCTT
TGGAGCCCGCCCTCCCTGGCGACCACTTCCTAGGACCCAGTAACGGGTGCGCACTTA
CTGGGCCCTCCTGGTCACCAGCTCGTGAGGGAGACGTCCCGAACGGCTCTGTAGACC
TCCGGCTTCGGCCTCCGGCTTCGGCCTTCCTCGGTCCCCAGCTCAGGAGGGAGACGT
CCCGAACGGCTGTGCGGATCCCCGGCTTCCCTTGGCACCCAAAGACATTCGGGCAGC
GGGGGTTAGTCTCCGTTTGGTGATCGCCATGGGATCGACGGCCTCCAAACCTGATCC
CCAATATTCCACCCCCTTAGAGGGCCTGCTGGCTAACCTGCGGACCCTAAAACTAAA
GGGATACATCCGCCATATTGGCTCACAATGGCCAGCCACAGGGACATTTGACTTTGA
CGTCCTCCGTGATTTAGATAACTACTGCCAGAGAATGGGAAAATGGTCTGAAGCCCC
CTATGTTCCGGCCTTTTGGGCATCTCACCCCACCCTGTGCACCACGTGCACTCTCCGC

CAAATCCTGCTTATCCTGGCTCCCCCTATTCCACCCTCCCGGGCCAAACCAGCTCCTC
CCATCTCTGAGTCCTCTGCTTTCTCTGTTCCCCTGGAAGATCTGGTGGCCCCTCCTCC
CTATACCTCTCCCATGGCCCTTACCCCTTCCACTCCAATTTCTGCTACCTCTCCCTCC
ACTCCGGACTCTCCTACCTCCGCTCCCGCCCCCGAGACTCCACCACCGGTCCCGGAT
CCTCCGGCCCTTAACCCTATCTTGTGTCCTCCTCTTCCCCCCGTCACTCCCTCCCCTTC
CCCGGTTAGCTCCCACACTCGCTCCCACAGCAACCCTCCGGGAGCCTCCCTTCCCCC
TCCCCCAGCCCCGCTACTCCCTCTCCGACAAGTAGCTGGAGCTGAGGGTCTAGCCCA
AGTCCATGTCCCCTTCTCCCTCCAAGACTTAGCACAGATTGAGGCCAAACTGGGTTC
CTTCTCCTCCAACCCCACTCAGTACATCAAGCAGTTTACTGGTCTGACCTGCGCCTAC
ACCTTGACATGGCAGGACATGTATGTCATCCTGGGGTCTACCACCACCCCCGAGGAG
AGGCAGGCCATCTGGACGGCAGCCAAGGCTCAGGCCGACCAGCGGCACTATGCCAA
CCCCTCCCCCAAGCGCCCCCCAGGGGCTCAGGCGGTTCCTGACACTGACCCTGATTG
GAACTACCAGGAGAGGGGTGGCGGCCAGCTGCGAGTATGCTATATGATCGAGTGTA
TCCTCGACGGGATGGAAACGTCCTCTCACAAGGTTGTAAACCTCCTCAAACTAGATG
AGGTGACCCAGGGGCCCGACAAAAACCCAGCCATGTTCCTTAATCGGCTGACCGAG
GCCCTCGTCCAATACACCAGGCTGTCACCTGAGTTCCCTATCGGGGCGGCCACCTTA
GCCAATCGTTTTATTTCCCAGTCTGCCCCCGATAACCGGAAAAAGTTGGCCAAGGCC
AAGGACGGCCCTCAGACCCCTATCCGAGACCTGGTAAAAATGGCTTTTAAGGTCTAC
CATGCCCGCGAGGAAACTGCTGAGGCCAGCCGAAAGGCAAGGCTCAAGCAAAAGG
CCGAATTTCAGGCAAGCCTCCTAAACCAGCAAACCCAGGCCTTGGTAGCGGCCCTG
CGGCCGGCGGCAGGCTCAGGGCCCCAAAACCCCCCTCTGGGGGTCTGCTTCAAGTG
CGGCCAAGAAGGGCACTGGGCCAGGATGTGCCCCAATCTGCGACCTCCTTCCAAGC
CGTGCCCATTGTGCAAACGACGAGGACACTGGGCTAGTGACTGTCCCCAGGCCTCTT
GGGCCTCGACCTCTAGGGGCCGGGGACCAGAGAGCCCCAGGGAGACCTCCTGCCCT
CCTTCGGCTTTGGAGCTGCTGAACTTCGACGGTGACTGACGCCGCCCAGACTCAGGG
ACCCCAATAACCCAAGCCGAGCCCAGGGTAACGCTCCAGGTAGCGGGTAAGTCCAT
CAATTTCCTACTTATTCGGCCCTTTCCTCTTTCGAAGGCATTTTACGTCGGTTTCGGTT
ATGGGCATTGACGGCCAACCCTCGTGTCCGCTCCAAACCCAACCACTATCCTGTCGG
TTAGATTCCTGTCTATTTACCCACTCCTTTCTGGTCATCCCCTCCTGCCCTACTCCTCT
TTTGGGAAGAGATATACTGGCTAAGCTGAAGGCCACTCTTCAGTTAGCTCCAGGGTC
GGCTCCCACGTCAGGGGCCTTCCTAATGCTACTGGTTGACCCTCCAACCCCTTCTGTT
AATCCTGAGGTCTGGGACACCCGAGTCCCGGTGGTGGCTCGGCACCACCCTCCAGTC
CTCATCCAGCTAAGGGACCCCACCTGTTTCCCAGCCCGGTCCCAGTTTCCTTTGTCTA
CTCGCAACCTCAGAGGGCTAAAGCCCATCATCGACCGTCTCATGGGACAGGGTCTCC
TGGTCCCCACGACCTCCCCCTGTAACACGCCCATTCTCCCTGTTCGGAAGGCTTCAG
GAGACTACTGGCTAGTGCAGGATCTCTGCCTGATCAACGCGGCAGTGATCCCTGCCC
ATCCACTTGTCCCTAACCCCTACACCCTCCTTTCTTCCATACCTCCTCAGACCTCCCA
TTTCACAGGGATGCCTTTTTGCCATCCCACTTCACCCACATGCCCAGACATCGAACC
TACAGCTATCTGCTGGTCTTGGTGGACACCTTTTCAGGATGGATCGAGGCCTTCCCC
ACAGCCCGGGAGACGGCAGCGGCAGTGGCAGAGGTCTTGATGGCCCATCTCATTCC
CAGATTTGGGTTGCCAAACTCTCTCCAGTCTGACAATGGGCCTGCATTTATCTCGCA
GATCTCTCAGCAGGTGGCTGCGGCCCTAGGTATTGGCTGGCATCTTCACATCCCCTA
TCGGCCCCAATCATCAGGCAAGGTAGAGCGGGTGAACGGCATCATCAAGACACATC

TGACCAAGACAGCCGCAGTTCTGAGGCAAGCCTGTCTTGGGTCGACTTCCTTCCTCT
GGCGCTCACTCGCATTCACACCACACCACACTCCAAAACAGGTTTGACCCCTTTTGA
ACTGCTCTACGGCAGGCCCTATCTCCTGACTCACCTCCCCGAGGGAGAGGCTCCCCC
ACTCGCAGGATACCTCCCCCTCTTCTCCCTGCTATGATCCTTGCTGAGGGAACATGC
AGACCGGGTCCTGCCGCAACCGACAGACGATGCAGGGCCCACCCGGCCTCTAGCAC
CAGGAGATCAGGTACTGCTGAAAACCCTGAGTCCCCGCCCACTACAGCCTCGCTGG
ACGGGACGCCATACTGTAATCCTCACCACGCCTACAGCCGCCAAACTCCTAGTACAC
GAGCCCTGGTACCACCTGACCCGACTTAGACAAGCTCCCCCGGGTCTCCCGGAAAC
AGGGGTGGCCCCGGGAAGACATTAGGGAGAGCAACCGAGAGAGAGGACAGAAGGA
GTAGGACAAAGAGAAAGGGAGACGAGGGAAGGAGAGAACAAGGGGGAAGAGAGC
AAGAGTAGAGAGGAAGTGAGAGAAAGCAAAAGAAAGTCAGAGAAAGGAAAGAGA
AGAGTTGAAGAGTGAAAAGAAGAGAAGGCAAAAGAAAGTAGGAAAGAGAGATAG
AGTGGTGGAGAAAGGGGAAGGAAGGAAGAAGGATAAAGACTGAAACTGAAAGAGT
GAGAGACGAGAGACAGAAAGGAATTAGAGAGTGTGACAGAAGGGAAAGGAAAGA
GCAAAGAGAGACAGAATCGAAGGAAAAGAGAGAGACGGAGAGGAACCGAAGGAT
TGGTCCCCCACTCTTCGACTGTCCCTAGCCCTCACATACCTGACCACCTGCTTCCTAC
TCCTGGAGTGCTATTCCCTTGGGACCCCCTGACCTGGTTACCAGCTCCATCCTCACG
GGACCGCCAAGGCTGGTTATCCACCGCACCCCTTTTCACCCCAATCTCGGAACAATG
CGGGGCTCTCTACTCTGGGTTGTTCTGGGGGTACTGGGGGTCCTGGAGGAAGGGGG
ACACACCAACCAAAACCCCCACCAGTCCTTTCAGATCACTTGGACGCTCCGGAATGG
ACTGACTCACGAGATACTCAATTTGACCACTGGAATTCACCCCCCTAATACGTGGTG
GCCAGATCTGTATTTCAACCTCAAAGACCTTATAAAGACTTCTTGGTCAGCGACCCG
GACCAGAAACTTTGGGTTCTGGGCATGTCCCGGACACCTAAAGACACATAATTGGG
AGACTTGTGGAGGACTGCAACATTATTTCTGTTGGTCCTGGAGCTGTGTCACCTCCA
ACGATGGGAGATGGAGATGGGAGGTCGGGAACCGGGACCTAGTCAATTTCTCGTTT
GTCCAACCCTATCGCGGGATTGCTCAGGTGAAAGTAATGCTCAACCAGGAAGCGGC
TAAACTGGAGAGGTCTTGGGTATCTGGGCTATCATGGGGCTTCCAACTCTATGCTGA
GTGGTTTGAAGCAAAGCCAGGGGGAATTCTGGTTGTTAGTCAGACAATAGAACCTG
TTCAGGCCCATGCTGTGGGGCCCAACCAGGTTATTGCACCTCCCCGCCCCTCACCAC
GGGGGACAAACGCCACAAATGTTGTGACCTCACCTACCCTCACACCCTCCATTCAGC
TGACCCGGACTGACCCGCTAGAGACCCAGGAACCCCTGTGGGCCTTAGTCAAAGAA
ACTTACAAGGCCCTCAACCACTCCAATCCTAACACAACCCAATCCTGCTGGCTCTGC
TACACCCTACATCCACCTTACTACGAGGCCGTGGGCTTAAGTGCTACCTACAACTTA
TCCATTCTGTCCAACCCACCACAGTGTTCTTGGGGAGACCGCAAGGTGGGCCTTACC
ATGAGAGAAGTATGGGGTTCGGGGACCTGCTTAGGCACGGTCCCTACGGATAAACA
AACCTTGTGCGCCCAGACTGGCGATGATACCAACTTCGCCAATAAGACCTACGTCAT
ACCTGACACTGGGGGATGGTGGGTGTGCTCACAGACTGGGCTAACGCCCTGTCTCCA
CTTGGCTGTCTTCAACCAGAGCAGGGAATTCTGTGTCATAGTAGCGGTAGTGCCCAA
GATTACATACCATCCAGAAGAGGTCCTCTACAACTTTTGGGACCAAAACACCCCAGC
TCCTAGACATAAGAGGGAGCCCATTACAGCTATTACCCTGGCAACGCTGTTCGCCCT
GGGAGCAGCTGGGACAGGCACGGGCATTGCTTCCCTGACCACACAACATCAGGGCT
TCATCACCCTGAGGGCCGCAATTGATGAAGACATCGCCCGCATAGAAAAGTCCATG
ACGGCCCTAGAAAAGTCCCTGACTTCCCTTTCCGAAGTGGTATTACAAAACCGCAGA

GGACTAGACCTGGTTTTTTTGCAGCAGGGGGGCCTCTGTGCAGCCCTCAGAGAAGAA
TGCTGTTTCTACGCTGATCTTACTGGGGTGGTTTGTGAGTCCATGGCTAAAGTAAGA
GAGGGATTAGAGCACAGGAGGAGAGAGAGGGAGGCTCAGCAGGGCTGGTTCGAAT
CCTGGTTCCAGAGCTTCCAGAGCTCCCCTTGACTGACAACTTTGCTATCTTCCCTAGT
GGGACCGATCATCATTCTCCTGCTGCTCCTGACTTTCGGGCCCTGTATCCCAAACAA
GCTTTTGGCCTTCATCAAACAACGGCTCGACACGATCCAGCTGATGGTACACCGACA
ACAGTACCAAGGGCTTCCAACAAGCACTCCGTGGCAAGATTAACGGCAGACGCCCC
CTGTCATCCCGGCTGTACCCTACCCCTCATCGCCCCCGTTCAGCAGGAAGTAGCCAG
AGAGAGTCGGCACCCCTTGTCCTATATCAAAAAGGCCGGCATGAAAGGTCAGGGCA
AGGTCAGGATGAAAGGTCAGGGCGAGGTCAGGATGAAAGGTCAGGGCAAGGTCAG
GCATGTCGGGACAGGTCCCGGCAGAGGGCGCTGCAGACAAGCCCCGGAGACGGGC
CGGCAACCAAGCCAACCAATCAGGAGCCGACACGAAGCCCTCGCCGTCCAATCAGG
AACCGACACGGAGCCCTTGGACACCAATCACCGCTGAGCCTGCGTTTTCTTCCTTAT
ATGGGAGCCCCGGCCCAGGCTATAAAACCCTTCCCCACCCTCACAGACTCCGCAGA
CTCCCTTTACCCC

ERV_16

CAGTGAACTTTGGACTATATGCCTGCTTGCACTTTCAGTGAACTTTGGACTATGTGCC
TAGTAGCCATAGGGATAACATACCTATGGCCTCCTGGACCGATAAAACACCAGATTC
ATACGAAGATTTCCCAGTGCCAAAAAGAATGCAATAATCCCCTATGTAATCACACCT
TTGTAATCTTTATGGCACCCATTGGTGTAGGCTACAACGTATAACCAGCTGACCCTT
CTGATTATGAATCATGGTTGTAACCCGATTGTATGTCCCTTTAACATTTTCCAGGCTA
GGTTTAAGGAATTTGGGGATGTGGGCTTGAGCATGTACACTTAAGGTATATAAGGTT
TTCACAAAAGTCAGCCGAGGACCCTGGGGTCCCTGGCCTGGGTCCCAGACCTGCTG
GTGTAATAAACGACACTCAACTATCCACACTGTCCTTCTGAGTGAATTTGTTTCCCA
AAGGGTTTGGCTATAACATTTGGTGCATTGGCTGGGAAACTCCTCACTTTGAGGAGA
CAAGTCCTGTTTGGGGCCACACCAAGGCCTTGTGGCTCAGATATTCTAGAGGGGGA
AGGTGCCTCGCCCTTCTGGAAAAATTCTCTTTCTTGACGCCTGGACCTTCCAAGTTAA
GTGGCCAGTGGATGACAGCAGGGGAATTGAGCTCTCGGCTAAGGAAGGTTCCCACC
CTGCGGGGTGGAAGAGGGGGCCTGATCAACTCCTGAGAGAGAGCAGGAAATGCAC
AAACTGTAAATGTCAAGGGAGGCAGTGGAGGTAGTGTCCACACGGTCCTGAGTTAA
ATTTGTCAGGTGGTCATGAGAAGGCTTTTCGAAGAAAATTGAATGTCACACCGTCTT
GGGACAAATTATTGCCAGACCATTGTGAGAGGGCTTTGAGGGGAAGAAACTTGGTC
TTTGTTGTGTGCTCATATTCTACTCTCCCCTAGGAGATGTCCCATCTGCCATGGAATT
CTTAATCCCCTCAGAATTGTCAGGCTAGAAGGAGGGGGATACATAAGTGAGTACAA
ATTGGCTTTTCCAGAGATGGCGTGGGACGTGGGATATTTAACCCATCTGTGTTTTCAT
CTGCACCTGATCAACCCCACCAAGGCAAAGTGGACTTTAAAAGTCAAGGGAGAAAC
AGTCTTGGACCACGTGGTGTTCTGGTTTGGCTGTGCTGACCAGCAGATGAAGACATG
CCAATTCCCCTTCTCCCTCTGGGATCTGGCAGGTAAGGCTCTTCTCACCCCAATTAGG
AAGGAGGCAGAATGGCAATTTAAATGTTTCATTGAGTTGAAATATCAGAAGTACAT
AGAGTACCGAGAACTCCGTACACAGAATGGAAAGGAAATGTAGAAGAAGGTCCAA
GAAGGTAAGCAAGATGGGAGGAAATGAATCTCAGACAACAGTACTGGAGTGCATG
AGTAAAAATTTTAAGAAGGGATTTGGAGGAGACTATGGGGTGAAGATAACACCTAA

CCGCCTCCACACACTCTGTCAGGTCAAATGGCCCTCTATGGGAGTAGGGTGGCCACC
AGAAGGCATCATGAACTTAAAAACAGTGGAAGCAATCTATACAATAGCCACAGGAG
AGCCAGGACAGCCAGATCAATTTCTACTTATTGGCTTATGGCTAGGGTTAGCTCAAG
AGCCCCCTCCTTGGATGAGATTCTATATCAAGAAAGGGAAAGGAACAATATTAATG
GCACAAAAATTGACTGATAAAAAAGAAATTCTGCAGGATTTGGACGGGGACAACTT
GCCCCCTTCCCCATACTGGATGATGACGTGCCCATCACCCAATGCTCCACCAGGACT
GGAGGCCACCTTAATGCCCAATCCAAGGCCAGGTAAAGTTCCTGCAGTGGCCACTG
CTCCTACACCGACTGTCCTGGAGATCATAGAGCCACTGTTTCAGCAGCCTCTGATCC
TGGCAATGGAGACCTCTGGTCAACGCCCCCAGGATTCCAGCTCAGCCGGACCTCCTA
GACTGTATCTACCTCTCCCGGTGAGTACTGATGGGAAGGGGAAAGAAAATTAGGAA
CTAGACAGAGGCTGCACTCCACCAAGGAACAGGGGGAAAGAACCCCACTACAGAT
GCCTCTCAGAGAGCTATAACAGCCTCCTGTTCAGGACGCAGGCGGGCACTACCATC
AGCCCTCTGTAGCCTATTATTACCAGCCATTTTCCTCTATGGATATATTAAACTGGCA
GAGACACACTCCACCATACTCGGGGGAGTCACAAGTCATCATTAGGCTAATGGAGA
CTATTTTTCGAACCCAGTGCCTTACATGGGATGCCATAATCCAACTACTAGCATCCC
CTTTCATCACTGAGGAAAGACACAGGATCCTAACTGAGGCCAGAAAATGATTAAGA
ACTGGCGCTTGAGAGTACCAGAATACGATTAAGAAATGATTAAGATGGCACTTGAG
CATACTGCAGCAGTGGGCAGAACTAGCCACCCCTGATAGAGGCCCAACTGGGACTA
TAACACAGAGGAAGGAGGGGGGGCCACCTGGAGAGATGTTGGGTGGCTATTTTACA
AGGCCTCAAGGGGGGGCCCGAAAACCTATGAATATGGCAAAACCCTCCAAAGTGAT
TCAAACCGAATCACCCTCTGGGTTCTATGAAAGACTGTGTGAAGCCTACAGACTTTA
TACACCAATAGATCCAGAGACCTCTGGGTCTCAGATGGTGATAAATGCAGCCTTTGT
GTCTCAAGACTACCCTGATATCAGACATCAACTTCAAAAGTTGGACAGGGTATTGGC
CACGACTAGCTTACAAATAATTGAGATCGCTGATAAGGTATTCAGAAATAGAGACG
TGGAGTCTAAGGGGGAAGCTGAGAAAAGACAGAAGATAATATGAGGGCAGACCAG
AGGATCGTGGTACTGGCCATGGCTTTGGGAAGGTCTCCTCTCTGAGTGGGGGCCAAT
ATCTCGGCAAGCCCTCTACAAGAAGGCCCACAGCCACCCTGCAACAAAACCAATGT
GCTCCATGCTGAGACTTTAGTCACTGGGAAAATGAATGCCCTCAAAATAATGGAGA
AAAAGAATTGGCATCGGCTGTTATAGGGCTGGTTGGACTAGAGGCTGAATAGGGGT
GCCAGGGTTCAAAGACACAAGGTCCCCGAGAGCCCATGGTGAAACTAAAAGTAGGG
GACCAAATTACTGACTTTACGGTGGACATTGGGGCAGAAATGTCAGTGGTGACTGA
ACCGGTAGTACCCCTCTCAATAAAGGCCACTGGCATAGAAGGAGTAACCAGAGAAA
AGATTATCAGATCATTCTGTTTACCCCAGAAATGCCAAATAGGGGGCATCAAGTGAC
TCATGAATTCCTGTATATTCCTGAATGCCCAGTACATCTTTTGGGAAGAGACTTGTCA
TCTAAATTGGGGGCACAAGTGACCTTTCCCCCTCAAGAAAGACCTACTTTCCGGGTG
TGGATATTTACTCTATTATTTAAACTATCTATTTACTCTCCCTCTCAGTAACCCTTCAA
GATGAATTGAGGTTACATGACCCTCTGGAAGGAAATCTGGATGGACTAGACAGCTG
AGAGAGGGAACTAATCCGATGATTCCCCAAGGTTTGGGAGGAAAATTAACTCCCAA
TCCCCACACTGGGCTTGCCATACATCAAGCTCCAGTGGTAATAGAACTCAAACCCGG
TGCCATCCCAGTCAGAAAACATCAGTACCCATTGCCACTAGAGGCTCGAGTTGGCAT
TTTGCCACACATAAGCAGACTGAAACAGGAAAGCATCCTGATTGAATGCCAGTCGG
CCTGGAACACACCAATTCTACCAGTCAAGAAAGAAGGGGGACAGGACTATAGACCT
GTGCAGGACCTCAGACTGGTCAACCAGGCCACTGTGACTCTACATCCCACCGTTCCT

AACCCCTATACTTTAACTAGCCTCCTTCCACCGAGTGCTAATATCTATACTTGTTTAG
ATCTCAAGAATGCCTTCTTCTGTATATGCCTTGCCCCAGTGTCTCAACCCATTTTTGC
TTTTGAATGGGAAGACCTAGCAGGGGGCACCAAACAACAGCTCACCTGGACTCACC
TCCCACAAGGATTTAAGAATTCCCCAACCATCTTTGGGGAAGCCATGGCTTCCAAAC
TGGACTCATTCCAGCTAGAGTTCAGATGCTGGTTGCTACAACATGTGGACGACTTGC
TGTTGGCTGCCAAGAACAGCGAAGAGTGCTGGGAAGGGACCAAGGCTTTACTAGAG
CAGTTGATGGAGTCCGGCTACCAAGTCTAGAAAAAGAAGGCACAGATCTGCAAAGA
GGAGGTAAGATATTTGGGGTTTGTTCTGAGAGGAGGGACAAGGCTGTTAGACCAGT
TCAGAAAAGAGGTCATTTTGAGAATCCCACAACCAAGAACCAGATGATGGGTCCAA
GAGTTCTTGGGAGCCACTGGGTTTTGTAGGATTTGGATTCTGGGCTATTCCAAGACG
GCTCAGACGTAATATGAACTCCTAACGGGATCAGAAGGAGATTCACTAAATTGGAC
TGCTAGACAGCAACAGCCCTTTGAGGAATTAAAGCTGGCAATCACATCAGCACCCG
CCCTGGGCCTGCCAGACCCTAAGCCGTTTACTCTTTATGTGACTGAAAAGGACAAGG
TGGCTATGGGAGTGCTGTCCCAGACTATGGGGACATGGGACAGACCCGTGGCTTCTC
TCTCAAAACGGCTGGACAATGTTGCCACCGGGTGGCCGGGATGCTTATGGGCAGTTG
CTGTGGTTGCCTTACTGGTCCAGGAGGCAACCAAGCTGACTTTGGGCCAAGATTTGA
TCATAAAAGTCCCCCATGAGGTCAACACTCTCCTGTGAGGGGACCCCCATAAATGGC
TGTTGACATTCTGGAATACTCAATACTAGGGACTGTTATGTGAGAACCCTCATGTTA
CTATTGAGCCTCGTCAGGCCCTGAATCCAGCCACTCTCCTTCCTGAGGGAGAAGGTG
GGCCCTCACATGATTGCAAGGAAATCCTAGAAGAAGTTTATGCCAGCAGACCTGAC
TTGAGAGACCTGTCAATCCTGGACCTGCCTTGGGTTCTGTACACCAATGGCACCAGC
CTGATGAAACAAGGACAGTGACTGTCAGGATATGCAGTAGTCACAGAAGAAACCAT
CTTTGAGGCTAGCTCTCTGCCGTCACCGGTCTGCTCAACGGGCCAAACTATATGCTC
TAATCCGGGCCCTCCAGCTGTCAAAAGGCAAGAAGACAAACATTTGCACAGACTCC
AGGTACGCTTCTGGTACTCTGTGTGTACATGGGGATTATATGAAGAGAGACGTCTTC
TGACAGCCCACAGAAAGGACATTAAAAACAAGAAATCTTGACTCTATTAGATGCTG
TATGGGAACCTGAAAAGGTAGCAGTGATACATTGCTGGGGTCACCAAAAGGAAGAC
ACCCACAAGCACAGGGAAACTGACTAGCAGATAAAAGTGCAAAACATGTGGCTGAG
AAATTTGGGGCTGCTGGTGGGGGACCTCAAAAATGCCTGAGTTAATGTTGATTCTCC
CACAGTATACCCTGGCCCAAGATCAGCTGGCTGAAGCAGAAAGGGCCACCATGAAT
GAAAAACGTTGGTGGGAACTGCCAGATGGCAGGTTACTGGTACCCGAGGCATTAGC
CCCCCATACTGGTGTCTCAGGTTCACCAGGCAACCTACTTGGGACATGACAAAATGG
AAGAATTAATTCAAAAATATTTCTTAATTCCATGACTTTCCTCCCTATGTAGACTGTT
CTGCTTGCTCAGATGTCAATGCTGCCCTTCGACATAAACAAAAACTTCCAGGAATAC
AGTTAAAAGGCACTCTACCCTTTGAACATTTAGAAGTGGACTTTACCGAGATGAAAC
CCTGCTGACACTATCACTATTTACTGGTCATGGTACGTACCTTCTCAGGATGGGTAG
AGGCCTTCCCCACTTGAAATGAAGTAAATGAAGTGGCTTGCTGTCTGCTCCGAGAAA
TAACCACCAGATTTGGGTTCCCAACCAGTGTAGGATCAGACAACGGCCCTGCCTTTG
GTAACCGACTTAATTCAACAGGTCTATAAAGGTCTAAATATCAAGTGGAAATTACAT
ATGGTGTATAGGTCCCAGAGTTCCAGAGTGGTGAAAGAACCAACCAAACTCTTAGA
CACTTTCAAAATGAATCATAGAGACTAACTGTTCATGGGTAGACTTGCTTCTGGCAG
CCTTACTCAAATTAAGGGTAACCCCGCATTCCCAAGGTTTTTCTTATGAAACTGTCTA
TGGGAAGCCCTCTCCCATAGTAAGACTGGTTTGGGCGAATCTGCCACAGGTAAGGG

CAGATGGGATTTCACAGCAGATGGAACAGCTGGGTCAGGTAATAAATCAGGTAACT
AAGTTTGTACAAGAAAGATTGCCAGTCCCCCTTGGGGAACAGATTCACGAATTTGTG
CCCAGTGATCAGGTGTGGGTCAAGGACTGGAAACACGACCGACTCCTTGGCCCCTC
ATTGGAAGGGTCCGTATACTGTTGTTCTAACCACCCCTACAGCCTAAGAGCTATTCA
AGCACTAAATACTGAACAAATACAAATGAGAAAAGTGGTAATTCAAAACAGAATGG
CTTTAGACATACTTACAGCCACTCAAGGAGGGACCTGTGCTATAATTAAAGCCAAAT
GTTGTGTATATATTCCTGATTTGTCTGGCAATATATCAGTCGCTCTAGATGACATGAA
GGATCAAGTAAAAGCTATGTATGATGAAAATCTTCCTTTTTGGACTTCTGTTCTATCA
TAGGTAAAGGGTGATTGGTAGAAACCTATATTAACCATTGTTACAGTTGTTCTGTTG
ATTCTGTTACGTGGACCTTGTATTCTCCAATGTGTTGTTAAGTTTGTATCACAGAGGA
TGACTTCGTTTACCCAAATATTTACCAGGAAGCCTAAAATGCAGTACATCTCAGTAA
ATGATGCTTGCACTGGAAGTTGAGAGCATCAAGAGGGGAGAATGAAGGAGGAAAG
CAACAGGCCGAGCTGACTCCATTTTGAAAAAGAAGCAAACTACACCTTACATTCCC
AGTGAACTCTGGACTGTACGCCTGCTTGCACTTTCAGTGAACTTTGGACTATGTGTCT
AGGAGCCATGGGGATAACTACCTACTACGGAACTGGCCTCCCGGACTGATAAACAC
CAGATTCCATACCAAGATTTCCCAGTGCCAAAAAGAATGTAACAATCTCTTATGTAG
TCAATCACCTTTGTAATCTTTATGGCACCCATTGGTGTAGGCTACAGCATATAACCA
GCTGACCCTTCTGATTATGAATTATGGCTGTAACCCAATTGTATCTCCCTTTAACATT
TTCCAGGCTAGGTTTAAGGAATTTGGGGATGTGGGCTTGAGCATGTACACTTAAGGT
ATATAAGGTTTTCACAAAAGTTGGCTGGGGTCCCTGGC

ERV_17

TACACATGGGGTATAAAAGATTTTCACAAATGCTGGTTGAGGTCCTTGGCTAAGAGG
AGACTCTGCCCTGGGCCCGCCGGTGTAATAAATTGCACTCCACTATCTGCATTGTCC
TTCTGAGTGAGTCTGTTTCCTGGAACGCGTGGCTATAACATTTGGTGCTTTGGCTGGG
AAACTCCTCACTTTGAGGAGACAGGTCTCATTTGAGGCCACCCCGAGGCTTTGTGGC
TTCAATCTCCTAGAGGGGGGAAGGCGCCTCGCCCCTCTGGAAGAATTCAGCCTTTCA
ACGCCTGGTTTCTTCGCTTTGGCAGGTAGTGAATGGCAGCAAGGGAACTGAGCGATC
AGATGAGGATGAACCCACCCGGCAGGGTGGAAAAGGGGGCCTGATCACCCCTCTGG
GAGGGACTAGAAGGGGCATGGGCCCACAGGGGCCTAGAATAGGCAGGCAGCAGCG
ATTGCTTGGTACATAGATCAATGAGCATGCTACGGTTTAGGAAGGTAATTTGTGAAG
GTCATTTAGGAGGTATGTCCACGCCGTCTCAGGGAATATTACTACGAGCAATTGCCA
GGGGATTTTTAGGATAGGAAACTCGTCCTTGTACGCTCATATTCTGCCCTTCCCCAG
GAGGTGTCCCATCTGCTGTGAAATTCTTGACCCCCTCGGAATTGTTAGGCTAGAAGG
AAGGGGATACATAAGTGAGTATGAATTGGCTTTCCCAGAGATGGCCTGGGACATGG
TATATTTAACCCATCTGTGTTTTCATCTGCACCTGATCAAGCCCACCAAGGCAGAAC
GGACTTTATGGGAGGAACAGTTTTGGACCACGTGGTGTTCCGATTTGGCCATGCTGA
CCGGCAGGTGAAGGCACACCGATCCCCCTTCTCCCTCTGGGATCTGGCAGGTAAGGC
TCTTCTCACCCCAAATAGGAAGGAGGCAGAATGGCAATTTAAGTGTCATTGAGTGG
AAATATCAGAAGTACAGAGGGTACTGAGAACTTCGTAGACAGAATGAAAAGGAAA
ATTAGAAGAAGGTTCAAGAAGGTAAGCAAGATTGGAGGAAGTGAATCTAAGGCAA
CTGTATTGGAGTGCATGATTAAAAATTTTGAGAAGGGATTAGGAGGAGACTATGGG
GTGAAGATGAAGCCTAACAGCCTCCACATACTCTGTGAGGTCAAATGGCCCCCTATG

GGAGTAGGATGGCCACCAGAGAACACCATGAACTTAAAAATAGTGGAAGCAGTCTA
TACAGTAGTCACAGGAGGGCCAGGGCACCTGGATCAATATCCATATATTGACTCATG
GCTAGGGTTAGCTCAAGACCCTCCTGCTTGGACAAGGTTCTGTATCCAGAAGGGAAA
GGGGAAAACATTAATAGCACAAAAATTGACTGATGATAAAAAAGGAAATTCTACAG
GATTTGGACGGGACGACCTGACCCCTCCCCCATACTGGATAATGACACTCCTGCCTC
CGTGCTCCACCAGGATTGGAGGCGGGCTTAATGCCCAGTCCAGGGTCAGGTGAAGT
CCTGCAGCAGCCGCTGCTCCTCTGCCAGCTCTTCCCAAGGTCGTAGAGCTGCCACCT
CAGCAGGCTCCAATCCCAGTGACAGAGCCTCTGGCCAGCACTTCCAGGATCTGGCTC
CAGCTGAACTGCCCAAGCTATACCCGTCTCTCCCTGTGAGTACTGACAAGAAGGGA
AGGGAGACATTGGAATTAAGCAGAGACTGTGCTCTACCAGAGAGCTGGAGGGAATG
GAAGAGACCAGACAAGAGATTTGCAATGCTAGCTGCTGCTCTGGGAAAGTCTATCT
CGGGCCCTCCGGAAAACCTCCTCTGCCCCAGGGACAGGACAGTCCTTCAGCCGGTCC
CAACGGAGGCCCCGGGCCCCATTACAGCCAAACCAGTGTGCCTGGTGCCAAGCTTTT
AGCCACTGGAAGAATGAATGCCCTAAGGCAAGAAAGGAAGAAGAAGCTCCCGCAG
TTGTGAGGCTTGCTGACTTGGAAATTAAATAGGGCTGCCAGGGCTCAGAGATATCAG
GTCCCCGAGAGCCCATGGTAACCTTAAAAGTGGGGGACCAAAACATTGACTTCATG
GTGGATACAGGAGCAGAACTGTAGGTAGTAACAAAATTTGTGGCACCACTGTCCAA
AAAGACTACCGCTGTAACTGGGGTATGGGGAGAAGACATGATTAAATCGTTTTGCC
AGCCCAGAAAATGTCAGATGGGGGGCACCAAGTGATTCACGAATTCCTCTACACTC
CTGAGTGCCCAGTACCCCTGCTGGGAAGAGACTTGCTCTCCAAACTGGGAGCACAA
GTGACTTTCCCCCTGAGGAGAGGCCCATCTTCTGGATGGACTATGACTTATTTGCTCT
CTCTCTCAATACCACCCCAAGATGAGTGGAGGTTGCATGAGCCTCCGATGGAAGAA
CCAGGTGGGCCGGAAGAGCATGAGAGAGAGCTAACTCAATTATTCCCTGAGGTCTG
AGTGAAAGACAAACCCTGCTCCCCACCCAGGCTGGCTAGACATCAAGCCCCAGTGA
TAACAGAACTCAAACCAGGCACCACCCTGGTTAGAAAGCACCAGTACCTGCTACCG
ATAGAGGCCTGGACCGGCATACTGCCCCATATCAATAGATTGAAACAAGCGGGCAC
TCTAGTAGAGTGCCAGTCGGCTAGGAATACAGCGATCCTGCCAGTCAAAAAGGAAG
GAGGATAGGACTATAGGCCTGTACAGGATCTCAGGCTAGTCAACCAGGCTACTGTG
ACTTTACACCCCACTGTTCCAAACCCCTATACCTTTTAGCCTCCTCCTGCCGAGGACT
AAAGTTTATACTTGCTAGATCTCAAGGATGCCTTCTGCGTACTCCTCGCCCCAGCGTC
ACAGCCCATCTTTGCCTTTGAATGGAGGATCCATTCGGGGGCACCAAGCAACAGCTC
ATCTGGACTCCCCCACAAGGGTTTAAGAACCCCCTAGCCATCTTTGGGGAAGCCTTG
GCTTCTGACCTGAACTCATTCCATCCGGAAGACTACGGATGTTGGCTCCTACGTTAC
GGGGATGACCTGCTGCTGGCCGCCGAGACCAAGGAAAAATGCTGGAAAGGGAAAA
AAGCACTGCTCCAGCTGCTGATGGAAGCAGGTTACTGGCTGTTGAAGAAGAAGGCA
CAGATCTGCAAGGAGGAGGTAAGGTATCTGGGGTTTGTTTTAAAGAAGGACACAAG
GGTCCAGACCCTAGTTGGGTCCTGTATACTGATGGCACTAGCCTGATAAAACAAGGA
CAACGGCTGTCAGGTTAGCCAAAGCGGAAGGGGCCATTAAGACTGAAAAGTGATGG
TGGGAATTGCCAAGTGGCAAATTATTGGTACCAGAGGAGCTGGCACACAATCTGGT
AAGCCAAACACACCTAGCGACCCACCTAGGCCATGCTGCCTGCTCACAGGTTAATG
CTGCCTCTCGGGTATTCAGACAAAAACCCCTGGGTATTTAGCGGAAAGGCACGCTGC
CCTTTGAACACCTGGGAGTGGACTTCACTGAAATGAAACCTCACCGACACTACCGTT
ACCTGCTGATCATGGTATGTACGGTCTCGGGATGGGTAGAAGCTTTTCCTACCTGGA

CTGAAAGAGAATCAGAAGTAGCCTGGTGCCTGCTTAGGGAAATAGTTCCCAAATTT
GGACTTCCTACTAGCATTGGTTCAGACAGTGACCTGGCTTTTGTAGCTGATTTAGTAC
AACAAGTAAGCAAAACTTTAAACATCAAATGGAAACTGCACACTGCATATAGGCCC
AGAGTTCTGAGATGGTGGAATGAACCAACTGGACATTAAAGAGACTCTCCAAGTAG
ACCACAGAGACTGACTGCTCCTGGGTGGACTTGCTTCCTAGGGCTCTGCTCAGACTC
AGGATGACCCCACAGTCCCAAGGCTATTCTCCATACGAAATTGTAGTAGGAGGCCC
CCTCCCATAATAAAACAGGTATCAACCAATTTGCCTCAGGTAAGGGGGAATAGGAT
TTCACAGCAGATGGAACTGGGTAAGGTAATAAATTGGGTAACTAAGTTTGTACAAG
AGAGGGTGCCGTTCCCCCTCGGGAACAGATTCATGAGTTTACGCTTGGTGACCAAGT
ATGGGTCAAAGGTTGGAAACATGATTTACTAGCCCTTTGGTGAAAGTGCCCTTATGT
TATTCTAACTACCCCTAGTGCAGTTAAAGTTGCAGGTATTGTCCCTTGGATCAATCAT
ATGAGGGTGAAGAGAACATACCACGCAGACCCAAAAAACACTGAATGGACTGCAC
AGAGGGACCCCGCTGACCCTCGAGAGACTAAGACCATCCTTAAGAAGAAGGAAAA
GAAGATCCTGGAGGAGCCCCTTCAGGATGCAGCCACACAACCAACTCCTGCTGCTT
GGCCTCACCAACGTGATTTTGAATTTAACTTCCGTTTCAACTCAGGACAATGCTTTCA
TCTCATGGGCACATTCCCACGCAGACTTCCACACTCCCAGCTGCTGGGTGTGTAGGG
CTGTGCCTCTGTCAGTGACGGATGGACTTCCTTGGTGGGTGTCACCGCTCTGCCAAG
GAGATTTTAAGCCACTCTGCTCTTTCTTGGATGACAAAAAGAGACCTTCCTCTCTTTT
GTCAATAACCTCTCCTTGCTCTTTTGATGTAAGACCTACACTCAATAGACTCAGGTCA
TGGGGTTACGTTTGATATAAATGCCAGTGTAACAAAAGCCTAACCTACTTGTAAATC
TACCCCGGTAGCCCTGGTAAATCTACCTTATTTACATGCAAGATGGACAAGATCCAT
GTTTCAATGATATGAGTATATTGCTGCCTTATTCGTATCCTCTATAGGGACAACAGAT
ATCAATGATTAAAGCAGAGGCCTTGACTAACTTCACACAACAGGCCCTCCTAGATAG
AACAAAAGCCATCCAAGCCTTAAATGAAGAGCAAATCCAAATGAGAAAAGCAGTG
ATTCATAATAGCATGGCTTTGGACATACTCACAGCTGCTCAAGGAGGGACCTGTGCC
ATAATTAAGGTTGAATGTTGTGTATACATTCCTGACTTATCTGGCAATGTATTGGCTG
CTTTAGATGACATGAAAAACCAGGAAAAAGCAACGTCAAATGAAAACATTCCTTTC
TGGACTTCGGTTCTATCTTGGGTGAAGGGTGGAAAACTGTATTTACCACTGTTATAG
TTGTCTTGATAGTTCTGCTTTGTGGACCCTGAATTTTACAATGTGTTATGAACTTAGT
AACCCGAAGGTTGATGTCATTCTCCCAAATTGACGGTCGGAGAGCCAGGGTGCAAT
ATATCCCTATGAATGTGCTCATAATGTGAGTTAAGAGCATCAAGACGGGGGAATGA
AGAAGGAATTCATAGGGCCTGGACTCCATCTTAGGCCTGTTCATGCTGATCATGCTC
AGCCACCTTTCCAATGGGCTCTGAACTCTGTGTTTAGTGCCTATGAAAACAACAACA
GAAGGATAAGACCCCCTCCAGACAGGGGAACCTTGAAGATCGTATCTAGGTTACTC
ATCGCCTAAAAGAAAACATACACTAATCACCCTTCCTCCAGACAGGCCATAAATTTT
TCTGTGTCTATCAGAGTGTAACCTCAGATTTATTGATTATTGGCTAATTGTTTGACTG
TTTGAGCACATGAGCACATAGCACGTGAATGATGGGTTATTGGGATTGTATTTTCCT
TGGTTTATGAAAGTCTCAAGGAATTTGGAGTGGTGGATTCAGACATGTACACATGGG
GTATAACAGATTTTCACAAATGCTGGTCGGGGTCCTTGGCTAAAAGGAGACTCTGCC
TTGGGCCCGCCGGTGTAATAAACTGCACTCCACTATCTGCATTGTCCTTCTGAGTGA
GTTTGTTTCCTGGAACGTGTGGCTACAACAATT

ERV_18

AATGGCTCATGATGTTGACCTCGCAAACAAAGAATAAAATCACAGTGACCCTTGAG
ACTGACCAAATTGCCCAAATGGCACACTGTTTTCCCACTGGCACCTACCTTCTCAAA
GCTGCAAGACCACAGGATTCCAGACCTCTGGCTACGAGACCACAGGACTCACCTAC
AGGCTAAAAATTAACTGTCCCTTCAGAGAATTCTTCATCGGCAGATTATAAGAAAGA
CCCCATTGGAAAGGGAGGACACTGGTTCTGCTAAGGGCCAGTCACCTTCTTTTTTCC
CTCTAATAAATTTCCTTGTCTTGCCTGACTGCCCAGCTGGCTCTGTTTTTCTCTGCACT
CATCTTACATTCTGGTGCTGAAACCTGGGACGGAAGGTCCACCGCAGCTGGGTGGTG
GGCTCCTCTCACAAGCACACCTCCGGTGGTCCCTTCTGTTGGACCTGGCCGAGAAAC
TGAGATGAGCTCCGGGATGGGACCATCCACCTGTCTTGCTGGACTGACATCCACCCA
CCAGCCCACATTTCATCCATCTGTTTCAAGGGGGAGTGAAATCCCATTCCCTTGGAT
CTTCTCTCTCTCTCTCTCTCTCTCTCTCTCTCTCTCTCTCGCCTTGTTTCCAAGCCCTAG
CGCTAGGCAGGAGGATCTCCATGGCCCTCGGGCCCTCGGCCTTGCGCTCCATCTGAC
TTTGAAATTGGGGGGCACCCATTTCAAAGCAGACTGTTATTACTCTCTGAGAAGGTC
CTGAGAACTGGGATGCCTTTTCTCTCGGCCCTTTCTCCTTGCTCTCCCTCACCCTTGT
CTACCCTTCCCATTCGGCCACAATGGGAAATTCTCAGTCCCAGCCCTCAAAATCTGC
TCCTCTAGGATGCCTTCTCTGAGACCTAAAAGCCTTGGGCTTCCAAGGGGCGATTGG
ACCAAAAAGACTTATTTACTATTCTAACACTGTCTGACCACAATACACTTGATAATA
GGTCCCAATGGCCAGAAAACAGAATTCTGGATTATAATCCTCTACGGGACATCAGTA
ACTTCTGCCGCCACAACAGCAAGTGGTCAGAGATCCCCCATGTCCAGGCTTTCTTCG
CTTTCCGCTCACGACCCTCCCTCTGTGAATCCTGTTCTACTTCTCAAATACTCTTAGC
CCACTCTGGGCCACATCCTCCTCATACAATGTCTCCTGATCCCTGTTCAGACTTTTCT
TCTTCTTCCTTCGATCCTTCTGACCACAGTCCACCCCCTATCGCTCCCAATCCCCTTG
CAGCTGCTCCAGACCCAGTTTCACAGCGTCCACCTTACGCCCCTTCCTGCCTCCCCCC
TTCTCAAGCACAGGCCTCCACCACCACTCCCAGCCCCACTCCCCTGCCCTTGCCTGA
GATAAGCTCTGAGACCTCAGCCCCACGCTCTACCGCAGGGTCCCAAGGCTTTACCCT
GACCTCCCTAGATCCCCTCCCATATCCAGTCCTGAACAAGCAGGAAACTTTACAGCA
GGACCCCTCGCCTTACCCTGCTCCACTCCTCCCTGTATGGGAAGCAGCTGGAGCAGA
AGGCATTGTTGGGGTTCATGTCCCATTCTCCCTCACCGATCTATCTCAAATCGAAAA
GCAGCTTGGCTCTTTCTCTTTAGACTCTGATAATTATCTGAAAGAAATCAAGTATCTT
ACCCAGTCTTACAACTTAACCTGGCATGATATTTACATCATCCTTTCCTCCACTCTTC
TCCCAGAAGAGGAGGAATGAGGATGGCAAGCCTCTCAGGCACATGCTGATGAGATA
CAGAGGACAGACGACACTAAGCCCACAGGGGCCATGGCTGTCCCCCGAGATGATGC
CAACTGGGGTTTTCAGGCAGGGAGACCTGGAGGAGCAGCCCGTAGTCACATGGTTG
CTTGCCTCATCACGGGCCTTCAAAAGGCAGGACATAAAGCCGTCAACTTTGATAAGC
TCCAGCTAATAACTCAAGGACTAGATGAAAACTGGGCACAATTTCTGGCCAGGTTA
ATGGAAGCCCTACAAAAATATACAAGATTAGACCCCACTTCAACAGAGGGCATCAT
TGTCCTTAACAGCCATTTTATCTCCCAGTCATCCCCAGATATCTGCAAGAAACTAAA
AAAGGCAGAAGGCCCTCAAACCCCTCAATGAGACCTTTTAAATACAATTTTCAATAA
CCAGGAAGAGAAGACAAATCTAGAGAAGGCCCAGGGTGATCAGGTTAAATACCGCC
TATTAGCCACTGCCCTACATGGCTCCACATTTCCACCAATCAACAAAGACAGGAAGC

CCCCTGGGCCCTGCTTCAAATGCAGCAAAGATAGTCACTGGGCCCTCATGCCTAAAC
CAAGGCCCCCTCCAGGTCCGTGTCCCAGCTGTGGCATAAAGGGACATTGGAAGGTC
AACTGCCCAAATCCCCCTCCAGGGATCCGGACATCTCCTCCTGGTCCCGAGCAGGAG
TCCTCCGACCCAGCTCTGCCCAGCCTCCTTGGACTTGCTGCTGAAAACTGAAGGTGC
CCAGGGCCCCGGCTCTCATCACCTCTACGGAGCCCAGGGTAGCTTTCTTAGTGTCAG
TTAAGCCGATCTCTTTTCTCATTGACACGGGTGCCGCTTACTCTGCTGTGCCTGCCTA
CTCTGGAAACCCCAAGGTCTCTCAGGTCTCTGTTACAGGGGTTGATGGTTTAATATC
TACACTACGAACAACCGAGCCTCGACTTTGCACACTTCAGGGTACCCCATTTTCCCA
TTCTTTTCTCATACTCCCAAAATGCCCCACTCCTATTCTTGGAGAGACCTGCTCTCAA
AATCCAAAGCCTCTATTACTGTCCCAGGCCCATCTTCTGATCTAGCCTGGCTACTGCT
CCTCAACCCCACCTTCTCTTCCCCCACCCCATTGCCCTCCTCGTCCATAAACCCCATA
GTTTGGGATACAGACAACCTATCTGTCACCTCTCACCATGCTCAATTCATATCCGCCT
CAAAAATCCCTCTAAATTCCCCCAATCACCCACAATATCCAATCCAAAAACATCAAC
AAGGGTTAAAGCCTATCATCACCAAACTCCTACATCAGGGTCTCTTGTGCCCAAGCT
ACTCTCCCTGTAACTCCCCTATCTTACCCATCAAAAAGCCAAAACGGCTCCTATTGC
CTGGTCCCAGGCCTGAGAGTTATCAGTGTGGCTGTCATCCCCATATACCCAGGAGTT
CCAAATCCCTATACTCTTCTCTCGTTCCCTCTTCCACCACCCACTTCACTGTTCTACA
CCTCAAGGATGCCTTTTTACCATCCCTTTACACCCAGACTCTCAAGGCCTCTTTGCCT
CTATCTGGACCGATCCAGACAATCATCGCTCCCCACAGCTGACAGGGACAGTCCTCC
CACAAGTCTTTCGTGATAGCCCTTATTTCTTTGGCCAAGCTCTAGCATCAGAACTAAC
CTCTCTTGACCTTCCTCTGAGTACTGTCCTCCAATATGTGGATGATCTCCTCCTTTGT
AGCCCTTCACTTACACACTCTCAGCAACACACTGCACAACTCTTCAATTTTCTAGCC
AATCGAGGCTATTGAGTATCTCCTACCAAGGTTCAGCTCTCTCTTCCTAGAGTCACTT
ATCTTAGAGTCCTTTTATTACGAACCAAAAGATGTATTACTACTGATAGAAAGTCCC
TTGTATCTACCCTACCACTCCCTACATCAAAAACAGATCCTGTCCTTCTTGGAGTTAG
CTGGGTATCTACACTTCTGGATTCCTAATTTTGCCCTCTTGGCACAACCCCTGTATCA
AGCCACTCAAGGAGATCTTTTAGAACTAGAGATAAAATCAAATATCCGTTCAGCCTT
TAAAACCCTTAAGCAAGCCATTCTCTCAGCCCCAGCTCTCACACTTCCTGACCTGTCT
TGCCCCTTTATACTCTACTGAAAGACACAAAATTGCCCTAAGAGTTTTGAGACAAAA
TTAGGGCCCCTCCTTCACCCCTGTTACTTATCTGTCAAAGCAATTAGATGCCACTATT
CGAGGACGGCCAGCCTGCCTACATGCTCTGGCAGCAGCTGCGCTCCTCTCTCAGGAA
GGTAAACAACTTCCTTTTGGAGCACCCACGGTCATTGATTCACCACATGACTTTAAG
GACTGTCTCACAAATCCATGACCCTTCTATCTCCTTCACACATTCAGCTAATTCATGT
CACCCTTCTTGAATCTCCCGAGTTTTCCTTTGAACTCTGTCCTACTCTCAACCCTGCC
ACCCTTATCCCGCATTCTTCTGAGCCTCCCACCCATACTTGCAAAGAGGCATTAGAG
GACCTGATGCCCCATTTCTCCCACATTTCCTCAATACCTTTAAATAACCCTGACTTTA
CTTGGTATATTGATGGCACTCCTCTACCACATCAGAAGGAAAAAAGGCAGCTGGAT
GTGCCGTTGTCTCTGACACCGAAATTATTGAACCCCAACTTGGCCTCTCGGGAACCT
CTTCCCAAAAGGCAGTACTTATTGCACTAACTAGAGCGCCTTCCCTCGCAGCTAACA
AGAGAGCAAACATATACACTGATTCTAAGTATGCTTTCCACATCATACACTGACATG
CTGCCATCCAGAAGGAGCGAGGGCTCCTATCTGCCAAAGGTTCTCCCATAACTAACA
CCCTGCTCATACTCCAACTTTTAAAAGCAGCTAATATGCCAACTGAAGTAGGCATCC
TACATTGCCAAGGTCACCAGAGGGCCTCAGACCTCATTTCATGGGGCAACAACACC

GCAGACAGTGAAGCAAAACAAGCCTCACTCCAATCACCAGCTCAACAACTTATAGT
AATCTCCAACATAAAACCCCTTCACCTCCCCGAAGACACACGATTGTTACAAGAGG
AAGCACAGCCACAAGGGGACTGGGTACAAAACCAGGGCTGCCGTGTCCTTCCCCAA
CCTCAGGCCACACCGATTCTTACAGACATTCATCAGGCCCTACAGGTAGGCACTAAA
CCTCTTCATCACCTCTTAAGACCCCTTATGGCGTCACTGACTCGATGACCATGAGTCT
GAGCAAGCTCTGGGAGTTGGTGATGGACAGGGAAGCCTGGCGTGCTGCAGTCCATG
GGGTCACAAAGAGTCGGACACGACTGAGCAGTTGAACTGAACTGAAGACCTCTTAT
CACCTATCCTAACATTCTTTCACTTCTATACGTCACTCACTCTTGCATTATCTGCTCCC
CAGTCTTACCACAGGGGGCCCTAAAGCCTGTTCTCTCACTCTCAACATGTCAGTCTT
GAGGGCATATCCCAGGAGAGGACTGGCCAATTGACTTCACGCACATGCCTCCAACA
CGAAAATTAACACTCATGTTCACTGTCAGAGACACTTTCTCAGGGTGGATTGAGGCT
TTTCCTATGAGATCAGAAACTGCCTCAGAAACTGAGTAACACTTGAGTTTCTCATAT
GAGAAGTCATCCCTTGCTTTCATCTCCCACTCTCTCTCCAATCTGATAATGGGCCAGC
TTTCATTTCCCAAATCCCTCAACAAGTAGCCCAATCTTTTGGCATAACCTGACAACTA
CATATTCCTTACAGACCCTCCCTATCCCACTAATTCATAACCGACCGAAATGGGCAA
TTCAATTCATTCCTCTATAAATAAGTATAGGAATAGCAGCTGGGATTGGAACAGCGA
CTGCAGGACTCACAGCCTCTTTAAATTAGTACCGAAGCCTTTCTAAAGACCTCACTG
AAAGCCTAGAAGAAATGGCTACTAGCCTTATCCCTGTCCAAAACCAGCTAGATTCCC
CGGCAGTCGTGGTCCTCCAAAACAGAAGAGGACTAGACCTTCTGGCGGCAGAAAAC
AGGGGCCCGTGTCTATTTTTGGAGGACGTGCGCTGCTTCTACACCAGCAAATCAGGC
GTTGTAAAGAAAGAAAGTGAAAGTTGCTCAGTTGTGTCCCACGCTTTGCAACCGGAC
TATTCAGTCCATGGAATTCTCCAGGCCAGAATGCTGGAGTGGGTAGCCTTTCCCTTC
TCCAGGGGATCTTCCCAACCCAGGGATCGAACCCAGGCCTCCTGCATTGCAGGCGG
ATTCTGTACCAGCTGAGCCACTGAGTTGTAAAGGAAGCAGCAAGAAATCTGACAGA
AAGAGCCTCTAGAGCATATAAACACCCCAGTCACTCATGGGAAAACTGGCGAAGCA
ATTGGAATTGGCTGCCCTGGGTCCTACCTTTGCTAGGCCTTCTTCTTCTCCTTAGCCT
TATTTTAACTTTTGGCCCATGTTTAATACATCTTTTTTCAAAATTTCTTCAGGACCGCT
AATGAGCATTTACCAACTGAACTACCCATAAGCTACTTCTAGCTCGCTCAAACACTT
TGACCCTACACAAATCCCCTTGACTCACATTCCAGACTTTTCTCATCTCACCCCCATG
TGCCCCCGTCCTGCAGAAACCAGTTAGGTAAAATTGACCTTAGGCTCTTATCCCAAA
TCAAAAAGGCTGAATGTTAGAGTCTATTTGGGGGTTCATGGTAGAAATGGCTCATGA
TGTTGACCTTGCAAACAAAGAATAAAATCACAGTGACCCTTGAGACTGACCGAATT
GCCCAAACAACATTCTGTTTTCCCACTGGCACCTACCTTCTCAAAGCTACAAAACCA
CTGGATTCCAGACCTCTGACTATGTGACCACAGGACTCAACTACAGACTAAAAATTA
CC

ERV_19

TATGGTGATCTGCCCCTCTTCAAGATTCAAGTTAATCATTTTATGGCCCAAGATAAA
CCATTTGGTGCCAAGATTATCCCAAAATGCATCTTATTGGTGAGGGGCCTGGTGCCA
TTCTAAGTTTTGAGACATTCCTTTCTTTCATTAACAGGCTGCTTGTGACTATATAACA
TCCAGCTGAAGACTAGCAGAGGGGTACTCTTTCTACCCCCTTCTGATGCCTACGTCA
GAAACTTTCTCTATCTCCTTTATACTTTAATAAAACTTTATTACATAAAAGCTCTGAG
CGATCAAGCCTCATCTTTCGCCTGGAATTGAATTCTTCTCCTCCAGGAGCCAAGAAT

GCCGGCGTCGTAATTCAACAAAAACCTTTCATCTTGGGGGCTTGTCCGGGATCCTTC
AGGACAAGGTAAGGATGCTTGGAGCTGTAGTTCTTTGTTCTCTTAGTGAACAAGTTT
TCTGCTGCGCTTAAGCAACACTACGAACAAAGCTAATGGAGGTGATGGAATTAAAG
TTGATCTGTTTCAAATCCTGAAAGATGATGCTGTGAAAGTGCTGTACTCAATATGCC
AGCAAATTTGGAAAACTCAGCAGTGGCCACAGGACTGGAAAAGGTCAGTTTTCATT
CCAATCCCAAAGAAAGGCAATGCCAAAGAATGCTCAGACTACAGCACAATTGCACT
CATCTCACATGCTAGTAAATTAATGCTCAAAATTCTCTAAGCCAGGCTTCAGCAATA
CGTGAACTGTGAACTCCCTGATGTTCAAGCTGGTTTCAGAAAAGGCAGAGGAACCA
GAGATCAAATTGCCAACATCTGCTGGATCATGGAAAGAGCAAGAGAGTTCCAGAAA
AACATCTATTTCTGCTTTATTGACTATGACAAAGCCTTTGACTGTGTGATCAGAATAA
ACTGTGGAAAACTCTGAAAGAGATGGGAATACCAGACCACCTGACCTGCCTCTTGA
GAAACCTGTGTGCAGGTCAGGAAGCAACAGTTAGAACTGGACATGGAATAACAGAC
TGGTTCCAAATAGGAAACGGAGTTAGTCAAGGCTGTATATTGTCACCCTGCTTATTT
AGCTTGTATGCAGAGTACATCATGAGAAACGCTGGACTGGAAGGAACACAAGCTGG
AATCAAGATTGCTGGGAGAAATATCAATAACCTCAGATATGCAGATGACACCACCC
TTATGGCAGAAAGTGAAGAAGAACTAAAGAGCCTCTTGATGAAAGTGAAAGAGGA
GAGTGAAAAAGTTGGCTTAAAGCTCAACATTCGGTAAACGAAGATCATGGCATCTG
GTCCCATCACTCCATGGTAAATAGATGGGGAAACAGTAGAAACAGTGTCAGACTTA
TTTTTTGGGGCTACAAAATCACTGCAGATGGTGACTGCAGCCATGAAATTAAAAGAT
GCTTACTCCTTGGAAGAAAAGTTATGACCAACCTAAATAGTATACTCAAAAGCAGA
GACATTACTTTAACGACTAAGGTCAGTCTAGTGAAGGCTATGGTTTTTCCTGTGGTC
ATGTATGGATGTGAGAGTTGGACTGTGAAGAAGGCTGAGCGCCAAAGAATTGATGC
TTTTGAACTGTGGTGTTAGAAAAGACTCTTGAGAGTCCCTTGGACTGCAAGAAGATC
CAACCCATCCATTCTGAAGGAGATCAGCCCTGGGATTTCTTTGGAAGGAATGGTGCT
AAAGCTGAAACTCCAGTACTTTGGCCATGACATGAGAAGAGTTGACTCATTGGAAA
AGACTCTGATGCTGGGAGGGACTGGGGGCAGGAGGAGAAGGGGATGACAAAGGAT
GAGATGGCTGGATGGCATCACGGACTCCATGAACATGCATCTGAGTGAACTCTGGG
AGATGGTGATGGACAGGGAGGTTTGGCGTGCTGCGATTCACGGAGTCGCAAAGAGT
CAGACACGACTGAGCAACTGAACTAAACTGGCCTGAACTAACTAACTCTACGGTGT
GCTTATGTGAATGAATGACATGCCATGCGTGAAACAAGTGAGAAGCCTTGCTCTGTG
GTTCCATGGTGATCTCATACAGTTCATGGCAGAAACCTGTAGGGGGTTTATACCGAC
CTGCCAATGCCAAGAGGCACCCAATGTCTCCTTTGGGAACCAACCAGAAATGGGCA
AAGCGTGTGGACTGAACTCTCCTTTCTCGGTCAAATTTTCCAGTCTCTTTGACCATTT
CATAACCCCTTGGGAATTAGAAGTTCTAACCTAATCTATTGGATCATAGACTTTAAA
GGGACTTGTGATCTATACTGTTACTGTGCACTGTGGCTTAGTTCCCAAACTTGGATTG
GTAGTCAAGAAAGCGCCTAACCTCACTAGGAATCAGAAATTCAGAAGCTAGATGGA
GCTCTAGCCCCAAGAACATCTGTGAGGTTAAAGGTTGCTCAGATTGGGACTGCAATT
TTTTTCCCCCCTTTGGTAATGCTGGCTCTTAGTCGACCAGAGGAGGTTCTTATACTGG
TGTGGTAATGCTTGAAAAAATCATCCCAGCTTTATGTTCGTATCAGTCTTATTATGGT
CAGGAATATACTCAGGGTCGTGCACAGGCACTCAGGTGGTGAATGTTTCCCAACCA
GTGGTCTTAGCTTGGGAGGCATTCCGGAAGTTTACTCTGATTGCACCCCAGGTGGCA
TCAGAGGCAAGCAAGGTTAAAGGTGAAGAGCTGGATATCAGGTAGAGATGCTAGCA
GGTCTATCCCTGGTACATCCCCACCCCATCTCGGTGGTAGAACCGGAAGAGTCGAGT

ACAGCACCTGCATCGGTAAGTGACAGACTAAGTCCGACCAGGAAGGAAAAGCTTTT
GGTGTAAAGTCTGTCTACACCCCATCTACAGCTGGGAAGGACGCTTCCAGTAGAAA
AATGGCACTGGTCGCTTTATTTCTCTCTTACAGATGGGAGCTAACAATTCCATCCTCA
CTCTTTTGAACTGTATCCTGAAAAACTGGGATAGATTTGATCCCCAGAGCTTAAAGA
AGACACACCTGGTCTTCCTATGTGATACTCCATGGCCACAGATTCCATTGGAGAATG
GAGAATGGTGGCCAGTTGGAGGGTCTCTTAAGTATAATACTGTTTTACAATTAGACT
TGTTCTGTAAGGAACAAGGGAAAAATGATCTTAAAGATAGATTTCTCACTCAGTCGG
TTCCTGATATCCGCCGTAAGCTACAAAAATGGGCATATGAACCAAATCAGTCTTTAG
ATACTCTGTTACAACTGGCTCAGACAGTCTATTATGGTAGGGAATATGAGGAAAAG
AAAGAAAGGCAAAGAAACACAAAGGAAAAGGCGGAAGCCTTCGCAATGGCTATGA
AAAGCTTCCTTAAACTGCCGGAGAAAAATGCCCAGAGGGACCCAGGTGAAAAGGTA
TGAGCTTGCTATTAACTGTGGAAAGGAGGGGCACCTCAAGCGGGATTGCCCTCAGG
CATCTAAGCAGGCCCTGGCTCCATGTCCGACCTGCAAAGGACCACAATGGAAGAGA
GACTGCCCTCAGAGGTGTAGGTCTCCGGGTTCGGACTCTCAAGACAATCACGACTGC
AGGTGCCTGGGGGTCCCCACACAAGCTCCTGTCCTAATTACACCTGAGGAACTCTGG
GTATTAATAATTGGGGGGTTCAATTGGTCGATTTCCTTTTAGATACTGGGGCAACTT
AATCTGTGCTTACTGAAGCCCCTGAGCCACAATCTTCCGATCCGCTTCCATAATGGG
ACTGTCTGGATGAGCCAAAAGGTATTATTTCACTTATTCTTTATCTTGCAACTGGGAT
TCTGTCATGCCAGAATCTCCCTCACCCCTTTTGGGGAAGGATATACTGAGCAAGGTC
CATGCCTCTCTTTTTATGAATATGGAGCCCTCCCTTTCTCTCCCCTTAGTTGAACAAA
ATGTAAATCCTGGAGTATGGGCTGATGGAAAATCTGTGGGTCGAGCACAAAATGCT
ATTCCTGTAGTTTCAAGCTCAAAGGCCCACACTTTTTTCCACATAAGAAGCAGTATC
CTCTGAAACCTGAGGTTAAGGAAGGGTTAAAACCCATCATCGAAAATTTAAAAGAA
CAGGGACTATTAACTCCCTTTAACAGTACTTGCAACACTGCTATTTTGTGCATAAAG
AAATCAAATGTTAAATGGAGACTAGTTCAAGATTTACGTATAATAAATGAGGCTGTA
GTTCCTTTACACCCCATGGTGCCTAATCCTTATACTCTTGTCTGAAATTCCTGAACGA
GCCAAATATTTCTCAGTAATTGATTTAAAAGATGCCTTCTATTCAATGCCTTTGGTGG
AGGAAAGTCAATTTCTATTTGCCTTTGAAGACCCTACACAGCCAGCTTCTCAGTTAA
CCTGGACAGTTTTGCCCCAGGGATTTCGTGACAGTCCTCACTTATTTGGACAAAATTT
GTCACGGGATCTACAAAACTTTAATAGCTCTGAAGCGGTGGTGTTACAATATGTAGA
TGATATTTTGCTCTGTGCTGAGACAGAGGAAGCTTGTTTGTGAGCCTCAGAAGATTT
CTTAAACTTTCTGGCAGGCTGTGGTTACAAGGCATCAAGAGAAAGGATCAGCTTTGT
CAACAATCAGTTAGATATCTGGGCCTAATCATATCAGAAGGGACTAGGGCCATAGG
CCCTGAGAGAATTAAACCTATACTAAATAATCCCCTATGTATGACTTTAAGACAATT
GAGAAGATTTTTGGGAATCACAAGTTACTGTCGCATTTGGATTCCAGGTTATGGGGA
ACTTGCCTGGCCTTTATATAAATTTATAGCTGAAACTCAACAGGCCCAAACTGACAA
ACTGGTTTGGTCTCCAGAAACTCAAAAGGCTTTTAAGGTTCTTCAAACTGCTCTCCTG
CAAGCCCCAGCTCTGAGCTTTCCCACATGGTCAGAATTTAATTTGTTTGTCACTGAA
AGGAAAGGTATGGCCTTGGGAGTTTTGACCCAACCCCGAGGGCCTCACCAGCAACC
TACTGCTTATCTAGGCAGAAAATTAGATGTAATTTCACGTGGGTGGCCCCACTGCCT
AAGAGTAATTGGGGCAGTGGCTTTATTAGCACCTGAAGCTTCAAAAATAATTAATGG
ATGAAAACTTACTGTACTGACTTCTCATGATGTGAGTGGAATCTTAAATTCTAAGGT
TAATATTTGGATGACAGACAGTAGGCTTCTTAAATATTAGTCATTGTTGTTAGAAGG

ACCAGTCACTAAGCTTAAAGTTTGTGGAAATTTAAATCCTGCCACTTTCCTTCCTGAG
AAAGAAAATGAAACACCTGATCACGATTGTTCTCAATTCCTAACTTTAAACTATGCA
GCTCGGAAAGATCTAATGGATACCCCATTAGACAATCCTGACATGGAAATATTTACA
GATGGCAGTTCTTTTGCTTGGGATGGAAAGCGTAAAGCAAGTTTTGCCCTGGTGACT
CCTGAACAGGTTTTGGAAGCAAAATCTCTCCCCCAGGGAACCAGTGCTCAGTTAGGG
GAGCTTGTGGCTCTGACCTGAGCTCTAGAGTTAAGCAAAGGGCAGCGGGTAAATAT
CTACACTGATTCTAAGTATGCTTATTTGACTTTACATGTTCATGCTGCAATATGGAAA
GAAAGACAGTTTAAAACAGCAAAAGGAGAACCTATTAAGCATTTCAGAGAGATCAA
GAGACTTTTAACTGCTATATATTGTCCTAAAGAAGTAGCTGTTATGCATTGCAAAGG
GCACAGCAGGGATGGGAGTACAGTAGCTGGAGGTAATCAGCTGGCTGACTGTCAAG
CCAGAAAAGCAGCACTTTACAAAGCCCCTTCACTGCAGATGCCTTTGATCTGGGCAG
GTCCTGTGGAACAGGAAAAACCGTAATATCCTGAGTAAGAATTAGAAAGATATGAA
AAAAGAGGAACAAAGATTACTAATAAAGGATGGTTACAGTCTGAGGATGGACGATT
AAAAATTCCTGAAAATGCTCAATGGAAAATTCTTAAGGGTTTACATCAGAGTTGTCA
TTTGGGTGTGGAGAGTACTTATCATGGCTTCTCGTTTGTTTGAAGGTAAAAATGTAA
TGAAAACTTTAAAAAATATTATCAAAATGTGTGAGGTTTGTCAGAAAAATAACCCA
AAGACTGAAAAGCTAACGAAATCTGGATTACAATGAAGTGGAAAGTATCCTGGAGA
AGACTGGGTAATTGATTTTACTCATATACCAAAACCTAATGGATATCCTTGCTTACA
AGTTTGGGTATATAGTTTTACTGGACGGATTGAGGCTTTTCCCTGTCGTAGTGAACA
GGCTAAGGAGGTTATAAAGATTTTAATCCATGCAATTATCCCCAGGTTTGGGCTGCC
ACGAAGCCTTCAGAGTGACAATGGCTCCGCCTTTAAAGCTGCTGTAACTCAGGGTTA
TCTAAAGCTCTAGGAATAGAATATCACTTACACTGTTCCTGGAGATCCCAGGAAAGG
TTGAAAAAGCTAATGACATTATCAAAAGACATCTGAGCAAATTACCTCAAGAGAGG
CAGGACAATTGAAGTCCTACCCATAGCTCTAATGAGGGCTCGAACTGCCCTCAGAA
AGGAGGAACTGTCCCCCTTTGAATGTATTTATGGAAGGCCTTTCTTACACACAGACA
TTGTTATAGACCCTGAAGCCTTGGAATTAATTAGTTATGTAACTCAGCTCTCAGCTAT
TCAACAGACATTAACAAAACTCCAGGAGACGACTCCTGACCCCGCCTCTGAGTCAA
GCAAGCCTCTATTTGAGCCAGGAACTGAGGTCCTCATAAAAACTTTGGGATCTGGGG
GCCCATCCCTCGAGCGTCTCTGGGAAGGCCATTACCAGATTATTCTTTCTTCTCCCAC
AGCTGTCAAAGTGCCAGGAATAGATTCGTGGGTACATCACACTCAAGTTAAGAGGT
GGCACCCTGACCAAAACTAAGTGACATCTTTTTATGTCTTTATTTTCTATGCTCTGAC
TTTGTACTTTTCAGATGGGCCTGATAACCTATGTGAGCTTACTTCTGCTGACTCCAAA
TATCCTGAGTCTGCCATTGGATCCTCAAGACAATGCTTTCCTGTCCTGGGCTCATTCC
TATGCTGCATTCCACAATCGGTCTAACTGCGGGGTCAGTGGAACAAACCCCTCTTCA
TCAGTGGAAGGCTTCCCATGGTGGACATATCCACTTCAAGGAAAAGATTTTCTCCAA
GTGTGTGAATACCTTCAACAACAGTCACAAGCGATGCCTCTCCTTCATCTGATGACA
TCTACCAAGCCTAAAATGGATGGGTGCAACACTTTGTACTTTTAACTATGGACATAA
AGTGGCTTTTAATTTTATTATACATTGTCTTGGTTCAATGACTTTTGCTACACATAAA
GCAAATAGGTCTAGATCTAATGGTTTTTTACCTGACGTTTTTCACATATGGGATGAG
GTTATATGGCTAACTCCTGAAAAAGGACTTTTAATATCTACTGCCCCTATATGCTGA
GAACAAACAGCTGCTGCTGCTGCTAAGTCGCTTCAGTCGTGTCTGACTCTGTGCGAC
CCCACAGACAGCAGCCTACCAGTCTCCGCCACTGGGATTCTCCAGGCAAGAACACT
GGAGTGGGTTGCCATTTCCTTCTCCAATGTATGAAAGTGAAAAGTGAAAGTGAAGTC

GCTCAGTCGTGTGCGACTCTTTGCGACCCCATGGACTGCAGATTCCAGGCTCATCCG
TCGATGGGATTTTCCAGGCAAGAGTACTGGAGTGGGGTGCCATTGCCTTCTCCGGAG
GACAAACAGAGCCATCCCCAAAAGTTAGCCAACAACTTAATTACAATGATTGGAAA
CAATTGGGATTTTTGCCTCAGAAAATACGCAACATAATCATTCCAATGTTTTCCAAC
CCTGGTTCAAGTTCTCCCTTTGTGTGGCCAGGCACTAATTGGGACTGGATATCTCAGT
CACGCTGGCTTGCTCCAAACGGGACTTAGTGGATATGTGGCTCTTAATATGGGCCCG
GCTTCTCCCTGATTGGATAGGGAGATGCACTCTGGGTCTAGCCTTTACTCACGGCTTT
GTATTTTCAGAGCTTTTAGAAAAGCCTGATAATTTACCCCACCGTAAATCTCCATGG
GCCGGATTGGTATTTCACTGATATGATTATTTGGCTGCAGTGTTTCTTCCCTCTTTGA
AAACTACAGATGTTATGCTACAAGTGGATGCTTTGACTAATTTTACTCCACAGGCAT
CATAAGATTCTCCAAAGGCTATTTAAGCTCTAATGCTGAACAAGCACAAATTAAAAA
GGTGGTTTTACAAAACAGATTGGCTTTAGATATTCTGACAGCTACGCAAGGAGGAAC
GTGTGCTATTACTCATACCCAATGCTGTACATATATATCCAATATGAACACAAATGT
TACTTATTTTACTAAACACATGAACAAGATGATTGGGGCCATGGATACTCCTGAAGC
CTCAATTGCCTCACTTTGGGAGACGTTAACCAGTTCCCCATGGTGGACAACTATCTT
AATTATAATAATTCTGATTGTTTTCTTTTTACTGTTTTCTCCCTGCATCTGTAACTGTA
TAACTAGATTTGTTTCTAGCCACATGAAAGCTTTCAAGTTGCAAATGGTTGCTCAAA
CTTCTGCTACTGCTGCAGCTGCCTCCAACTACTATTTGGGGCCCCTGGATCAGATATC
CTCAATATGAGGATTAAGAAAATATGCTGCCTCACCAATTTAGGGACAACACCCTTC
TCAGCTCAGAAGCAGTTATGGAATGAAAACAACGCCCCTTTTCCCTAGGCAACATAA
TTCTCCTAAAAGAAAAGTGGGGAATGAGAGGATAACAGGCAGGAAAGCAGGGGTC
TCCAAATGTAGGAAATAGCCTGCAAGTGTCAGACATTTTTCTCTCTCTTAAGCAGCA
GGAGGAAACAAACTAGTGATATTTTTTTTCCTTCTCTATACAAATTTAAAAGGAGGT
TTCTCTTAAAATTCTATATTGCCATAATGACACCTGGTTTCACCTGAAGTTAACCAAT
GCCTTTTTCTTATGGAAATGTCTATCCTAAGCTATGCTAATGAACTATGCATTTACCC
CAAACTCTGTCTTCAATTCGGTTCTGCCTCTTGGCTCAGAACCTACTTGATAAACCAG
TGTGTTATACTCTGATATTGTTCCTCTAACCTATATAAATGAAACTATTTATATTGTG
ATCTGCCCTTCTTCAAGATTCAAGTTAATCATTTTATGGCCCAAGATAAACCATTTGG
TGCCAAGATTATCGCAAAATACATCTTATGGGTGATGGGCCTGGTGCCATTCTAAGT
TTTAAGACATTCCTTTCTTTCATTAACAGGCTGCTTGTGACTATATAACATCCAGCTG
AAAACTAGCAGGGGGTACTCTTTCTCCCCCATCTGATGCCTATGTCAGAAACTTTCT
CTATCTCCTTTATACTTTAATAAAACTTTATTACACAAAAGCTCTGAGTGATCAAGCC
TCGTCTTTGGCCCCGGATTGAATTCTTCTCCTCCGGGGGCCAAGAATCCCGGCATCG
TAATTCAACAATAACCTTTCA

ERV_20

TGAAGGGTTAATGCAGCCACAATAGGGAAAGTGGAGAAGTGCCTGCAGACGGGGCT
CTCTGCTCGGGCTGAACGTACTTGCAAGTGAGGCGTTCTGCCAAGGAGTCTGGACAT
AGCCTTGAGTTTGATGGTCCCTTGCAAACGAGGGAACATTCCCTTCTTGTGATAAGA
AGAAGGAGGAGGGCTCTGGACAGACTCTGCAGTAAGCAGAAATTTCAGTCCCTTTT
GCTGTATGATAACATTTATGCACATGCGCTATACTGAAAAGGCTTGGTCATACAGTC
TGGAATTCCGCCCAGGGGGGCTATATAAAAGTGAACCACAAGCTCGCTTGCTTGCGC
AGTTCTTTTTCCTCTGGCCAGAATGGTGTCTGTCTTTTGTGTGTGTTGTCTTTGTGTCA

TTTCACTGGCAATCTCCAACATCTGGAGCCCAACGTGGGGCTCGAGTGAAACCGAA
AGGGTGAGTAACCCCGGGGGGATTTTAAATCCATAGCAGGGGAACTTTTGGGAAAA
TCATGGGGAATTCCTCACCCTAGCAGGGGAACTTTCAGGAAATCATGGGGAATTCCT
CATCATTAAGGACACAGTACATGGAGTTAGTCAAAGGACTTCTCCACTCCATAGGCG
TTAAGGTCTTGACTCGTCGATTGAGTGAGCTCTTTCGCTTGGTGGAGAAATATTGTC
ATTGGTTTCAATATCAAACTAAGTTACAGTTAAATTTGAAGGAATGGAAAATAATTC
AAAAAGAATTGAGAAAGCAACATCAGAAGGGTAATGTGATTCCTTTGAAGTTATGG
ACTTTATGTAATGCTGTAACACAGGCTTTGACTTTGCTATCTACTGATAATGAGAGT
AAATCTAATGCTTCAATGAAGGAAGAGGCAATTTATGAGGATGTGCCAGACGTTGG
TGGGGCTTCTGTGTTGCCTGAAGGCAAGGATACAGGTGAGCCTCCTCCTGTAAATGG
TGAAACATCTGATTGTTCAGAATCAGATTCGGAGGCTTCTTCGGTTTCGTCAGAGGA
GGGTAAAGAGATTAAAGAAATGACCCATCTATTCCAGGAGTGGTGGAAATCCCATA
AGGAGGAGAAGAAATCTGCGCCTTCTGCTCCTCCTTGTGCTTCTCTTTTCCCCACTGC
GGTTGGTCGGCCCGATGTGCGCAGGGAACATTGTCGGTTCTCCTTTCCTTTGTCTATG
CTTCATGATGATGACTCGCCTGCTCCCCCTGGTGGTTTTATCGATCCTCCACAATTAT
TTCCCATCCAGAGACAGCAAGATGACAACGTGATAAATGTTCAATACACTCCTTTGG
AATATAAATTTTTTAAAGATCTTAAAGCTACAGTAGCGCAGTATGGTCCTCAATCTC
CCTTTGTTTTGGCTATGCTGGAATCATTGGGAAAAGGCAAATTAATTATTCCATTAG
ATTGGGAATCTATTGCCCAAGCTGTCTTGGAGGGTTCTCAATGGTTGCAACTTCGTA
GCTGGTGGGAAGAAGAAGCTAGAAAGCAGGCTCGGATTAATGAAGGACAGAATCC
CCCTGGTCCTCTTGAGGATAAGTTAATGGGAGAGGGCTGTTATCGGGCTTTAAGAGA
ACAGGCTCAGTACTCTGATCAGGACTTACAGCAAGTCCGCCAGGTCTTTTTACGAGC
ATGGCATCGTGTGGTGCCTACTGGCCACACCCAGCCCTCCTTTGTTAAAACAATGCA
AGGCCCCAGTGAGCCATATACTGATTTTCTAGCAAGACTGAGGGTAGCTATGGAATG
AGCTGTAGGGAGGGATGAAATTTCAGAGATATTATTACAAACTTTAGCATTTGAAAA
TGCAAATACTGAATGCAAACGTATCCTGGGACCATTAAAAGGACAGGGTGCATCTA
TAGCTGAATATATCAGAGCCTGCTTGGGAGTAGGAGGAACAGAGCATCAGGCTAGT
GTCTTTGCTACAGCCTTGGCCAAAGCTATGAGACCACAAAAGGGAGGTAACTGCTTC
CATTGTGGAAAACCTGGTCACATGAGAAGAGAATGTTGGAAATTAAAAGCTGATCA
AGGTGCAATTCCTAAAGACAGATCTTTTGCTGGGAGGAATAAGACTCCTCCCGGACC
TTGCCGTCGATGCGGGAAGGGGCTTCACTGGACCAATGAGTGCAAATCTAAAACAG
ACAAAATGGGCAACCCGATACCGGGAAACTATCCTGCGGGCCTGAGTCCTTGGGGC
CCAGGAACAATACCGGAGGCTTTTCCTCCTTGCCCTCCTTCCCTCCCAGCAACAGTG
ACCAGTCAATGCCTCATTAAAAGGACCTCAAATGATGATTTCAGACTTACGGTCTGC
TACTTCAGGAAGTGCTGCTGCTGATTTGCCACTAGCTGAGAATGTTCTTTTGTCACCA
GGAGGAGGCATTTACAAATTAAAAACAAATGTATTCGGACCACTGCCTAAAGGCAC
CTTTGGCTTAATATTAGGCCGTAGCAGTGCGGCTTTGAGAGGTCTAACCATAATTCC
TGGGGTAATAGACCCCGACTATGTTGGGGAAATCTTGATTATGGTTTCTACTTCTAC
CACGCTTTCATTATTAGCTGGGGAACGTATTGCTCAAATACTTCTCCTACCTTATCAT
CCCTTTTTGGCTCTTCCTAATGAACGAACAGGAGGATTTGGAAGTACTGGGCGACAT
ATATTTTGGGAAATGCTTATCAAAGATTCTCGCCCTGTTCTCTCCTTAATTATACAAG
GAAAAAACTTTGAGGGACTAGTAGACACAGGGGCAGATGTTTCAGTTATTTCTTCTC
AACAATGGCCCCAAGATTGGAAAAAAGAAAAAAGCCCTCTAATGCTGACGGGACTG

GGCTCTATTGCAGATGTCTGGAAGAGTACCCATCCCTTGCAATGCCAATTCCATAAT
GCAAGATCAGTGTCTGTTACCTTTTATATTGTAAACATACCTATTACTATTTGGGGAA
GAGATCTTCTCTCTCCATTAGGGGCTTTTGTAACCATTCCACCGGAAAACTAGTAGC
CACTGCTCAAATTCTTCGAGCACTCCCATTAAAATGGTTAACTAATACTCCAAAATG
GGTTGAGCAGTGGCCATTACCACAAATGAAGCTCGAGGCGTTAGAACAATTAGTAC
AAGAACAACTCCAACTTGGTCATATAGAGCCCTCTACCTCCCCCTGGAATTCTCCTG
TTTTTGTTATAAAAAAGAAATCTGGAAAATGGAGAATGTTAACCGATTTACGAGAA
GTTAATAAATGTATTGAACCTATGGGAGCATTACAATTGGGACTCCCCTCTCCAGCT
CTTATTCCTCAGAATTGGTCCTTAATGGTGCTAGATCTTAAGGACTGTTTTTTTGCCA
TTCCCCTACAATTACAAGATAGAGATAAATTTGCTTTTACAATTCCTGTTCTTAATCA
TGCTCAGCCTGTTAAGCGTTATCAATGGACAGTCTTACCACAGGGAATGACAAATAG
TCCTACTTTATGTCAAGAATTCATAGCTTGCTCTTTACAATCCCTCCGTCAAGAATAC
CCCAATTATATTCTATATCAGTATATGGATGATCTCCTATTAGCAGCTCCTAGTATTG
TCGAACGTGATGAATTCTTTCTAAAAGTACAGGAGGCTTTAAGACTATACAATTTGC
AAACAGCCCCAGAAAAAATTCAAAAGGACTTTCCTATTTCGTATTTAGGGACAATAT
TGGAACAACATAGAATAATGCCCCAAAAGTTGCAATTCAGAAGAGACCACCTCAAA
ACCTTAAATGATTTTCAAGTTATTGGGAGATATCAATTGGCTACGCCTGGTACTTGG
GATTCCTACTTATCAATTACGACATTTGTTTTCTTCTTTAGCGAGAGATAACAGCTCT
GGATAGCCCCCGGACTTTAACCCCATTGGCTTTACAGGAACTTCAATTTGTTGAGCA
ACGACTAAATGACGGCTTTTTTATTTACTTACATGCGTCTCAACCTATTTTGTTTATA
ATATTTCATACCCCTTATTCTCCATCTGGTGTAATTGCTCAAGAAAAAGGATTAATA
GAATGGATTTTCTTACCTAACAGCTTTTCAAAAAAAATGACTACATATATGGATAAA
TTAGCCTTCCTTATACAGAAAGGTCGCCATCGCATTTTACGATTGTCAGGATGTGAA
CCACACCAGATTGTTACTCGGTTAACAACTGCTCAAATATCTCGATGTTTACAATTTA
ATGAAAACTGGCAAATTTCTCTTGCCTCATTTCCTGGTTCGTTTTCTAATCACTATCC
ATCATCTAAATTGATTGATTTTCTTCGGGCTAACTCTATGATATCTCAATCCCCAATT
TCAGATGTTCCAGTTAAGGGACCCACTATTTTTACAGATGCAAATAAAAATACCACT
GGATATTGGACCCTGGAAAGTTCCAAGGTTCTCCCCCATTCATTTTCTTCTGTACAGC
CCGCTGAATTGGGGGCTATCTATTTAGTTTTACAAGATTTTCCCCAACTTCCTATTAA
CATTGTTTCAGATTCTCGATATGCTGTTCTCTCTTGCTTACAGCTTCCCCATGTCTCCC
TTCCACTGACCCTTAAAACAGCTATTGATAAATTGTTTTACCAAGTACAACAATTGC
TCTTGCAGCGTTCAGAGTTAATTTTCTTTACTCACATTCGTGCACATTCCGCCCTTCC
TGGACCCTTATCATTCGGAAATGCTACAATTGATGCCTTAATTTATCCTATAGAAGC
AGCAAAACAAGAACATCTCTTACAGCATACCAACTCCAAAGGGTTACAAAAATCTC
ATACTATTACTCGAAAACAAGCTCAAAATATTGTTCGCTCTTGTTCCATATGTGCACC
TTTTACTTTGCCATTTACCCCACCAGGTGTCAACGTAAGAGGACAACAAGCAAATCA
GATATGGCAAATGGATGTAATTTACGTACATCATACTATAGACACATGCACACGTTT
TCAATGGACCACTGCATTACATTCTGAAAAGGCTGATGCTGTTATTACTCATTTGTTA
TCTTGTTTTGCAGTTATGGGATTACCAATTGAATTGAAAACTGATAATGCACCTGCCT
ACCAATCCGCGAAATTAGCTCACTTTTTATCCCAATATCATATAACTCATACTTTTGG
TATTCCTTATAATAGTTAAGAGCAAGCTATCATTGAAAGAGCTAATCGTACCTTGCG
TGAATATCTTGAAAAAATAAAAAAAGGGGGAACAGGAGAGATATATGAAACCTAA
AGACATTTTGAATAAAACCTTACTTACCCTAAATTTTTTGAATATTTGGAGCAAGGG

AAATCTATCAGCAGCAGAGTTGCATTTTCAAGGGAAAGAAGAGGATAAGAAGATCT
TGAATATGCCTATTTGGTATAAAGATAAAGAGAAAGGTTGGATCCCAGCATCATTAA
TATATCTGGGACGAGGGTATGCTTTCATTTCTGTTAATAATTACAGGTTTTGGACCCC
AGCGAGATTGATCAAAATCGACAATGGCTGATCCCTTTGTTCAAAAATTCAAAGAGC
TTACTATGCAGAGAAGCTTTACTTTTGGTACAAGGGAAGCAACACCTCCTACATGGG
GTCAAATGAAGAAGTTGACCCAGGAAGCAGAAAAGACGTTAATGAAAGCGGGACA
GCCTCTGAATCCTACCAATCTTTTGCTTGCCATGATGGTGGTGGTAACATGTCAGGT
AATCGGTGTCTCGGCAAGTAATCATACGTATTGGGCATATATACCTAATCCCCCATT
AGTAAGAGTAGTTTCCTGAGGGGAACCAGAAGTGCAGGTATGCACTAATGAGACTG
CCTTCTTTCCCCCACCAGCTTGCGGGGGAACAGAACAACTATCTCATCATAAACAAC
AGTATAATATTAGTAGTTTGACCATTGCAGCGGAAAGTATTCCTTTGTGCATAGGAG
GACACACTTTTGTCTGTCCGCTAAGGAACATTCTCATCATTCCTATAATACATGGGG
GGTAAAATATAATAATTATCATTTTGCTACTTTTACTATGCTTGTTTCCACCAGGGGC
TTTTAACACCTCGATAGAACCGCTAGACATTCATAATGGAATATATATGTCACTATG
TCCTGTTAACTGTTTTGCTCCTTCTCTAGAATCTTTGGAATGGGAATGTTGCCAAGGT
CATCAACCCTTTAAAGTCATGAATTATTCTGGGTCTGTCATTGTAGATTGGAGTCCA
GATCATGGGCAATTCTTAGAAAAATGGTCAAATAAGTCTCTTAGGTGGCATCGTGCA
AATAGCACTTTGATGGGCAATGGTAATGAAACAGTTAAATGGCAGCAGTTTGCACTT
GTCCCTCCTCAATTACAATTGCAGGGATATCCACACATTCAAGGGGATATTTGGAAA
CTATGGGCAGTTTCTGGTAATCTCACTGTCTGGTCAGGAAACTATACTTTGGACAGT
GGTGACTCTTCAGGTCCATTTCATGTTAATTTACACGTTAATAAATCTTATTCCGCAA
TGGCATGTGTAAAATATCCTTTTGCATTGTTATATGGAAATTGGACCTGGAATGATA
CTGTGGGGTCTGTGTCATGTGACTACTGTAATCTAACTCAATGTGTAAATCAGTCTTG
GTGGAAAGAATTTGAAAGACGAGTGTGTAACTCCAATTTTTCGTTAGTAATTGTTAA
AGCTTGGACAGAAGTATGGTTGCCTATAAATCTGACTAGACCATGGTCAGATTCTTT
TGCTGTTTCTCATCTAGTAACCGCTGTACAGACTTTGTTACATCGATCTCAACGTGTG
CTTGGTGTGGTCATTACTTCGATTCTAACAGTCGCGTCAGTAACTGCAACAATGGCG
GTAGCAGGCCTCGCGTTACACCAAGGAATTCAAACAGCTGATTTTGTTCAGGACTGG
CATAAAGACTCTCATTTGTTATGGCAACAACAGCGAGATTTGGATGCACAACTTGCT
ACTGACGTGCTCAATCTTCAACACACCGTTTCCTGGCTTGGAGATCAATTAGCTGTTT
TATCTACACAAAGTGTGTTGAAATGTGATTGGAATTCTTCTCAGTTTTGTATAACACC
TGTACCATTTAACATGAGTGAGGGATGGGAAAGAGTAAAACGATCTTTGACTGGAC
ATCAAAATCTCACTACAGAAATTATGGACCTGGAACGACAAATTTTGTCTACTTTTA
GCGGGACTTTACCTGACATTGTGGGGTCTGGTTTGTTGAAAAGTCTTCAAGAAGGAT
TGAATAACTTAAATCCATTAGGGCATGTATCCACACTAATTGGGACTACCTTTGGGA
ACACTATGTTTATATTACTTTTATGTTGTGTTGCTTTTCTAGTCTTCCAACGATGGCG
GAAAAGGAAACAACTAAAGCGTGAAGCAGAGAAGATCCAGACCATGCCACAATTT
ATTAAAGCAAATAAAAAAGGGGGAGATGAAGGGTTAATGCAGCCACAATAGGGAA
AGTGGAGATGTGCCTGCAAACGGGGCTCTCTGCTCGGGCTGAATGTACTTGCAAGTG
AGGCGTTCTGCCAAGGAGTCTGGACATAGCCTTGAGTTTGATGGTCCCTTGCAAACG
AGGGAACATTCCCTCTTGTGATAAGAAAAAGGAGGAGGGCTCTGGACAGACTCTGC
AGTAAGCAGAAATTTCAGTCCCTTTTGCTGTATGATAACATTTATGCACATGCGCTA
TACTGAAAAGGCTTGGTCATACAGTCTGGAATTCCGCCCAGGGTGG

ERV_21

TGTAGGGAACTAGATTTAAGGAAGGTTGAGAAGTGCTTGCAAACGAGACTCTCAAC
CAGGGCTGGACATTCTTGCAAACGAGATGTTCTGCCCAGTGTAGGGAACTAGATTTA
AGGAAGGTTGAGAAGTGCTTGCAAACGAGACTCTCAACCAGGGCTGGACATTCTTG
CAAACGAGATGTTCTGCCCAGTGTAGGGAACTAGGTTTAAGGAAGATTGAGAAGTG
CTTGCTAACGAGATGCTCAGCTAAAAATCCTGTTTGTTCCCGTGGGAAAAGAACATT
GCATTGCACACAAGGATGTTTCTCTGATTCCTCAATAGGAACAGACCTTGGGACAAA
ACGGATTCTAAGTTGATAAGGAAGTTCCCCAAAACAAAGTCTTAGCTGCAGTAAAT
AAGCCAAGGTAAAGGTTATCTCGCCCTGCGCCTGCGCACTGTATAGTCTCTAAATCA
TAGTGCTTGGCAGCTTGCCCTGTAGGGGGTTGTACAAAGGATATAAAAATTAAGCA
GCTGTAAGAAGCAGGTGTTAAAATATAAAGATTAAAACCACTCTGCAGTGTTGGTGT
GTCATTCCGTCGCCGACATCTGGCGCCCAACGTGGGGCTCGACGGAACCGAAAGGG
TAAGCACCCCGGGGCAAAAGGCTAAAAGGGGGACTTTCGGGAAATAGAGAAAAAG
AGAAAAATAAAAATCCGAAAGGGTAAACACCCCGGAGCAAAAGGCTGATAGGGGG
ACTTTCGAGAAAAAGAGAAAAATAAAAATCCGAAAGGGTAAACACCCCGGAGCAA
AAGGCTGATAGGGGGACTTTCGAGAAAAAGAGAAAAATAAAAATCCGAAAGGGTA
AACACCCCGGAGCAAAAGGCTGATAGGGGGACTTTCGAGAAAAAGAGAAAAATAA
AAATCCGAAAGGGAGAGCACCCCGGAGCAAAACGCTGAAAGGGGGACTTTCGAGA
AAATAAGAAAAATAAGAGAAAAAGAACAGAAAAAAGAAAAAAGAAAAAGAAAAA
ACTATGGGGAATTCCCCGTCTTTAAAGTCACAATATATGGAGCTAGTTAAGGGGCTC
CTGCATTCTATAGGAATTAAAACGTCTACTCGCCGCTTGAGTGAGTTGTTCTGTCTTA
TAGAGCAGCATTGTTATTGGTTTCAATATCAAACAGAAGTACAGCTGAATTTGAAGG
AATGGAAAGTGGTGCAGAAAGAGTTAAGAAGGCAGCATCAAAAAGGAAATGTGAT
TCCTTTAAGGCTGTGGACTCTATGTAGCGCTATAACACAGGCTTTAAAATTAATGGC
TGTTGATAGTGAGGCCGTCTCATGTTCTCTTAAAAAGGAAGATCCCCTATATGAAGA
TGTGCCTATGAGTGAGGAGAAGGAAGATAAAGGTGAAAGGGGGGCTTCATCTCGGC
CTCCGCTCAAACCCCCAGATGATAGTAAAGATTCATCCACTGAGTCAGATAGAGATG
TAAGTTCTGGGGAGGATTCAGATTCTGTAGAGGCAATGACTACTGCCTTTCGGAAGG
TTTTATCAAATAAAAAGCGAACTTCCAAGGCTGTTAAATCCTCTGCACCACTCTACG
CATCCTTATTTCCAGTAGCTGCCGACAAGGCTGAGGTAGGGAGAGAGAATCAGCGA
TTTACATTTCCTGCTACTAAGTTCTATGATGATGATGATCTTTCAGCACCTCCTGGAG
GTTTTGTGGATCCACCTCGCCTCTTTCCCATTATACGTCAGCATGATGCACGGGCAG
GAATGATTAATGTTCAATATATGCCTTTGGAGTATAAATTTTTTAAAGATCTTAAGG
CCGCTGTTTCTCAATATGGTCCCCAATCTCCTTTTGTATTATCAATGTTGGAGAATGT
AAAAACTTCTAAATTAATTCTTCCTCTTGATTGGGAATCAATAGCTCAAGCTGTTTTA
GAAGTATCTCAATGGTTACAGCTTCGTAGTTGGTGGGAAGAAGAAGCCCGAAAACA
AGCTAGGATTAATGAGGGGCAAAATCCACAAGGTCCTTTAGAAGATAAGTTGATGG
GAGAAGGGCAATATCGAGCCTTGAGGGAACAAGCTCAGTACACAGATCAGGAATTG
CAACAGGTGCGTCAGGTTTTCTTAAGGGCGTGGCGTAGAGTGGTACCTACTGGACAG
GCTCAACCGTCTTTTGTGAAAACTATACAAGGTCCTAATGAGCCTTATACAGATTTTT
TGGCCCGCCTTAGGGTGGCTATTGAACGGACAGTTGGTAGAGATGAGATCTCAAAA
ATTTTGCTTGATACATTGGCATATGAAAATGCTAATCCAGAATGTAAAAGGATTTTG
GGACCTTTAAAAGGACAAGGGGCTTCTGTGGCTGAGTTCATAAGAGCATGCTCTGG

AGTTGGAGGAATTGAACATCAAGCCTCTGTTTTTGCAGCAGCTTTAGCAAAGGCTGT
GAAACCTCAGAGGGGAGGAAATTGTTTTAATTGCGGGAAGCCAGGACATTTTCAGA
AAGATTGTAAGAGACGAAGAAATGAGCCAAAAAATGAGCGATTGCCAGAAAAGCG
ACAGCCCTTAGGTCTTTGTAGGAGATGTGGCAAGGGAAAGCATTGGGCTCAGTAGT
GTAAATCTAAAACTGATCGGGAAGGGAATCCCTTAAGAGCCCCTTTGTCGGGAAAC
TTCCCAGTGGGCCTAAGCTCCTGGGGCCCAGGAACAGTACCGGGAGCTCCTTGCCA
GTTTCCTGCACAGGTCAATGCCAATCTCGACAGCCAGTTTCCTCGAATGGTGCTCAA
ATGATGATTTCAGATTTGAAGGCTGCTACATTAGGTAGTGCAGCTGCAGATCTTCCA
TTGGCTGAAAATGTTATTTTATCCCCAGGAGGAGGTATTTATAAATTACGGACTAAT
GTTTTTGGCCCATTACCAAGAGGAACTTTTGGGTTAATTTTGGGACGAAGTAGTGCA
ACATTAAGAGGATTGATTGTATACCCAGGAGTAATTGATTCTGATTATGTTGGGGAA
ATTTTAATTATGGTTTCTACATCACAGACTCTTTCTTTACTAGCAGGGGAACGGATTG
CACGACTTCTCTTGTTACCTTATCACCCGTTTTCTTCATATTCTAATGAGAGAGTCGG
TGGTTTTGGAAGTACGGAAAAAAGTATATTTTGGGAAATGCTGATTAATGACTCTCA
TCCTTTAATGTCACTGATTATTGAAGGTATACAGTTTGAAGGATTAGTGGATACAGG
AGCAGATGTTTCGGTTATTTCTTTCCAGCAGTGGCCTAATGACTGGAAAAAAGAAAA
AATTCCTCTTGTTTTAACAGGCTTAGGATCAATAGCAGATGTGTGGAGAAGCACTCA
ACCTTTGTCATGCCAATTGTCTAATGGAAAGAAAGTATCTATTTCTTTTTACATTGTT
AATATACCTATTAATATCTGGGGAAGAGATCTTTTATTTTCTTTAGGAACAACACTC
ACCATCTCGTCGGAAAACTTGTAGCCACTGCTCAGATTCCTCGAGCTCTCCCATTGA
AATGGTTAACTAATGTCCCTAAATGGGTTGAGCAGTGGCCGCTTCCAAAAGTGAAGC
TCGAGGCTTTAGAACAATTAGTAAAAGAACAGCTTCAGTCTGGCCATATTGAACCTT
CTACGTCTCCTTGGAATTCTCCTGTTTTTGTCATTAAGAAAAAATCTGGTAAATGGAG
AATGTTAACTGATTTAAGGGCAGTTAACAAATGTATAGAACCTATGGGAGCTTTGCA
ATTAGGTCTCCCTTCTCCTGCTTTGATTCCACAGGATTGGTCTTTGATGGTTTTAGAT
TTGAAGGATTGTTTTTTTAATATTCCTTTGCAAATAAAAGATAGAAATAAATTTGCTT
TTACTATTCCAGTGTATAATCATGGGCAGCCCGTAAAACGTTATCAATGGACAGTGT
TGCCTCAAGGAATGATTAATAGCCCTACACTTTGTCAGGAGTTTGTTAATCGTGCTCT
TATTACTGTGAGACAACAATTTTCTAATTGTCTTCTCTATCATTATATGGATGATCTT
CTATTGGCAGCTCCTAGTAAAGAGGAACGAGATACATTTTTTATTCATGTAAAGAAA
GCTTTAAGTGATTTTAATCTTCAAATTGCTCCTGAAAAAATTCAAACTGAATTTCCTA
TTTCATATTTAGGTGCTATTTTGGAACGACAAAGAATAAAACCTCAAAAGGTCCAAA
TTAGACGAGATAATTTGAAAACTCTTAATGATTTTCAGAAGCTTTTGGGTGATATTA
ACTGGTTACGTCCCATGTTAGGGATTCCTACACATCAATTGCGTCATTTATTTTCTAC
TTTAGAGGGTGATACTGCTCTTAACAGTCCTCGATCTCTTACTTCTCAAGCAAAAGA
GGAATTACACTTTGTAGAGCAACGGTTGAATGAAGGGTTTCTTATCTACAACAGGAT
CAACCTATATATTTCATAGTGTTCCATACTCCCTATTCTCCTACTGAAGTTATTGCTC
AATCTGCAGGGTTGATTGAGTGGGTATTTTTACCCAATAACTATACAAAAAAGCTTA
CTACTTATACGGATAAAATTGCTTTCCTTATTTTGAAAGGTCGAGGCCGTATTACTCA
GTTAATTGGAAGTGACCCTCAAACAATTATTACTCAACTTACTTCCACCCAAATTTCT
AATTGCTTACAGTTTAATGAAAATTGGCAGATAGCTCTTGCATCTTATTCAGGGACT
TTTTCTAATCAATATCCTCAATCTAAAATGATAGATTTCCTTCGACATACATCAATGG
TTTGTAAATCTCCTATATCTAATATTCCAGTTGAAGGAAAAACTATTTTTACAGATGC

CAATAAAAATACTGCTGGCTATTGGACTGATACTACTTCTAAAGTTGTTCCACATTC
CTTTTCTTCTGTGCAGCCTGCTGAACTTTGGGCCATTTGTTTGGCATTACAGGATTTT
TTTGATATTCCTATTAATATTGTTTCTGATTCTAAATATGCAGTCTTTTCATGTATTTA
TCTTCCTGAAGCCACTTTACCGGTAACTGTAAAAACTAATATTGATAAATTATTTTTT
CAGGTTCAACAATTATTAATTAGGCGAACTAACCCTGTCTTTTTTACTCATATACGAG
CTCATTCTTCTCTCCCCGGACCATTATCTCAAGGAAATGCAAATATTGATGCCTTACT
TTATCCTTTACAGTCTGCAACACAAGAACATTGTTTACATCATACTAATTCTAAAGG
GTTACAAAAAACTTATTCGTTAACACGAAAACAAGCTCAACGCATTGTTCGTTCTTG
TTCCATATGTGCACCGTTTATTTTGCCCTTTGCCCCTCCTGGAGTAAATCCCAGAGGA
CTACAATCTAACCAAATTTGGCAAATGGATGTTGTTTTTATTTCTTCATTCGGTAAAC
AGAAATATGTTCATCATACTATAGATACTTATTCTCATTTTCAATGGGCTACAGCTTT
AAGTTCAGAAAAGGCAGATTCTGTTATTACTCATTTATTATTTTGTTTTGCTATTATG
GGCATACCTATAGAATTAAAAACTGATAATGCTCCCGCATATCAATCCTCTAAATTA
TCTCAATTTTTAGAACAATATCATATTAAGCACACTTTTGGTATTCCTTATAATAGTC
AAGGACAAGCTATAATTGAAAGAGCTAATCGGACTCTTCAAAAATACATTAAAAAA
TAAGAAAGGGGGAAAAATGTATGATATCACTAAGGGGCCATTTCAAGTTCATTTGC
ATATTAATAAATCTTACTCTGCTATAGTATGTGTAAAATTTCCTTATTCTTTACTGTA
TGGGCAATGGATTTGGAATGAAATTTTGGGAGTTATATCATGTTCTGATTGTAACTT
GACTCAATGCATAAATCGTTCTTGGTGGGAAAATGTTGAAAACAAGATGGTCCATTC
CAATAATTATTCTTTGGTCATTGTGAAAGCACGGACTGAACTTTGGTTGCCTGTTAAT
CTTACTCGACCTTGGTCGGACTCTTTTGCTGTCACTCATCTTGTGAATGCTGTACAAA
CTCTTCTCCATCGATCTCGAAGAATGCTTGGCATTGTCATCGCATCAATTCTCGCCGT
GGCTTCAGTAACAGCAACAGCAACTGTAGCAGGTCTTGCTTTGCATCAAGGCATACA
GACTGCTGATTTTGTCAGGGAATGGCATAAAGATGCTCATTTATTGTGGCAACAACA
GCATGATTTGGACGCTCAATTGGCTACTGATGTATTGAATTTACAACACACAGTTTC
CTGGTTAGGAGATCAAGTGACTGTGTTAGCTACAAAGAGTGTGTTAAAATGTGATTG
GAACTCATCTCACCTATGTGTAACCCCTGTTCCCTTTAATATGAGTGAGGGTTGGGA
AAAAGTGAAAAGGTCTTTAGTGGGACACCAAAATTTAACTGCTGAAATTATAGAAT
TGGAACAAACTATATTGTCAACTTTTAGTAAGACGCTGCCTGATATTCTTGGTTCTGA
TATACTGAAAAGTCTTCAAGAAGGATTAGACAACCTTAATCCTTTAGGGCATGTATC
TACATTGTTAACAACATCCTTTGTGAATACTTTGTTAATTGTTGCTTTATGTTTTATTG
CTTTTATAGTCTACCGGCGCTGGCGAAAAGGGAAACAATTAAAAGAAAAAGCTTTG
CATATTCAAGCGCTTATTCAGCATCTACAAGAAAAGAAAGGGGGAGATGTAGGGAA
CTAGATTTAAGGAAGGTTGAGAAGTGCTTGCAAACGAGACTCTCAACCAGGGCTGG
ACATTCTTGCAAACGAGATGTTCTGCCCAGTGTAGGGAACTAGATTTAAGGAAGGTT
GAGAAGTGCTTGCAAACGAGACTCTCAACCAGGGCTGGACATTCTTGCAAACGAGA
TGTTCTGCCCAGTGTAGGGAACTAGGTTTAAGGAAGATTGAGAAGTGCTTGCTAACG
AGATGCTCAGCTAAAAATCCTGTTTGTTCCCGTGGAAAAGAACATTGCATTGCACA
CAAGGATGTTTCTCTGATTCCTCAATAGGAACAGACCTTGGGACAAAACGGATTCTA
AGTTGATAAGGAAGTTCCCCAAAACAAAGTCTTAGCTGCAGTAAATAAGCCAAGGT
AAAGGTTATCTCGCCCTGCGCCTGCGCACTGTATAGTCTCTAAATCATAGTGCTTGG
CAGCTTGCCCTGTAGGGGGTTGTACAAAGGATATAAAAATTAAGCAGCTGTAAGAA

GCAGGTGTTAAAATATAAAGATTAAAACCACTCTGCAGTGTTGGTGTGTCATTCCGT
CGCCGACA

ERV_22

GGAACTTCTTGAGTTGCAGCAAGGGTGTGAAAGACCCTTTCGAAGTTCAAGAGGGA
AGGTGTGATTAGCCTCGAGACGTCAGTGGAAAAGGGCCTCATCTCGCCTGGAGGAG
AGAACCTCCTTGATTTTCTCGAGTTGCGATAGGTTCCTCTCGAGTTACGACAGGGAC
CTCATGGACCCACTCGTGTGGCCTCAGGAAAGGCCAGTCTCCATATGAGTTGCCAGG
GGACCCTCGGGATTCCTCTCCAGTCTGTGTCGGGACCTAAGTCTTCGTCTGGAGCCG
AGGCCAGAACCTGAAGTTTCCTCTCCAGTGCTGATATTGATCTTTGGGTTCTTCTGGC
GTCTCCACAGGGGAGTCAGGCCTCGTCTTGACTGGAGACATCCACGTCCGCTTTCCT
CCCTAGCTGTAGCAGCAGTGTTCAAATTTCCCCTGCAGTTTACACAGGGATTTGTGT
CTTTCCCTCTAGGCTTTACCCCAGGGTTGTCAAAAGTGGCACCGTGGTGTGTGTCGA
TTTTCGGGATGACAGTCAAGGTAGTACTGGGAAATCAGTTCCTCTGGAGTGGACTGA
GACATTTTGGGGTCTTTTGGAAGGGTGGCACGATCCTTGAAGTTCCTCTCGACTTTCC
TGTTGAGAGCGCCTCCTCTTGAGATGCGACGGGAATGCCGGAATCCTTTCCCGAAGA
AGCAAGGAAAGGTACCCGCATCTCGAGCGGAGGAGGGGAATCGGGGCTCCTCTTGT
GTTGTGGTGGGACCCTCGGTGTTCCTCTCAAGTGAGACGGGTATGTCGGGGAACTTT
TTGAGTTGCAGCAACAGTGTGAAGGACCCTTTTGAGGTTCAAGGTGGAAGGTGTGAT
TTCCCTCGAGACTTGCAGCAAGAAAGGGCGTCATCTCACCTGGAGGGGAGAACTTC
CTAGTTTTTCTTGTGTTCCTGCAGTTTCCTCTTGAGTTACGACGGGGACCTCAAGGAC
CTGCTCCTGTAGCCTCAGGAAAGGCCACTCTCCATGTGAGTTGCTAGGACCTCTCGG
GATTCTGCTCCAGTCGTTTCTGCGTCCTCAGTCCTCGTCTGGAGCTGAGGCCAGAAC
CTGAGGTTTCCTCTCTAGTGCTGACATGTTTCTTGGGATTCCTCTGGAGTCTGCACAA
GGAATCAGGCCTCATCTCTAGTGAAGTTATGCATGTCCAGTTTCCTCCCGATCTGGA
GCAGCAGCTTCAGGCTTCCTGTCAAGTTGACACAGTGATCTGTGGCTTTCCCTCGAG
GCTTTCTCACAGGGCTGTCACACGTGCCACCATGGTGTGAGTTGATAGTCGGTGTGA
CAGTCGAGACAGTGCAGGGGAATCAGGTTCCTCTTGTGGAGGAGCTGGAAGGCTTT
AAGCCAATACTGAAAATGTATTTTCTCCATTTATGATGAAGAGTCAATACGGCCAGC
CTTAAGGCATTTGAAGGCTTCCTTCTGACCACCTGTTTCTGAGAACAAGGACTATTG
TTTATGATAAGACTCCCTTTAATGTTTTGCCAAAGCCATGTTATGTCTTGGGCGATGA
GAACTGTATTTTATGCTTGAATGCTTTAATGTTTATCTGGAATGGCTATGTACATGTC
TGCCTTATGCTCTATTCCCTGAGCATTATAGAACTGTGCAGTTAGATAATAAACTTTG
TCAGACCACTAGAGGCTGTCCCTGAGTGTCCTTTTTAGAGTGTGGTTCTCTGAGCCTT
ACAACTGGCGCCCAACGTGGGGCTCAAAGCAGCAGACTGACTTTGAAAAAGGGCTA
CACTCCTGCAGAAGCGAGGTAAACATGATGAGACATCAGTCAAGTAAAATTCCTTTT
GCTCACCTCATGCATCATTTCTTGAAACGATACAGAGTTAATCTGCTTAAAGAGCAG
TTAACTAGCTGTTACCAGACAGTGGTTAAATATAATCCATGTTTTCCGGAGGAAGGA
ACCTTGGATTTATAAACTTAGACAAAATAAAAGATAATATTTTAAAAGCTTATAGAC
AGGGGGTAAAAATTCCCCCACAATGGTGGGTAACTTGGTCTTTTTTATGGGCTGTTA
TAAAACAATTAGATGGCTCAAGAAGTAATTTAGAGGTTAAAACTGTTGGATCCCTAC
ATAAATACAAAATTTAAAAAAAAGATCTATCAGAGGTTTTAAATCAAAAATATCAT
GCTAGAGCAGATTACAGACAAACAAGAGTCACATATGCTAAAAGCAGTCAAACAAT

CAACTTTGCCAGAGGCCCTGATCTGCCTTTACCTTGCTTTTAGCGAGCTCATATTAAC
AAGCCTCTAACACTGGCCTTTCTGCTCACTGCAACGCTGTCTGATGCTGCTCTTGCTC
CCCTATTTCCTAGTATTCCTCCTCAGGACATGCCTCTTTGTTCGTGGCCTTTTCCGGCC
CAACTTAACAATCCTCAACCTAGACATAATCAATGGCAATCACCTGATTTTGGCTTG
CTCATGAAATTCAAAAAGGCATATACGTAAAAATAAAAATTAGAAAAAAACAGTAT
TGTAAAGTAAAATAAAGCAAAAATAAAAATTAAAAAAGTACTGTAAAGTAAAATAA
AGTAAAAATAAAAATAAAAATAAACAGTATTGTAAAGTAAAATAAAATTAAAATTA
AAATTAGAAAAAAAAAAAAAGGCATATACGTTATATAGACCTACCTCACCCTACTG
TATGAAATTTCTTAGGGGCTGGGCAGATCAATGGCTTCATGCAGATTTTTTTACAGTT
GCTAAAATGGTTATGACTCCACAATAGCTACTACAAAGGCAAATGTGGGTCACAGA
TAAAGCCAAATTAATCTTGCAAGAACAACAGAGCAGGAAAAACCCTACTGGACTAT
ATTTTAAAATTCTCACTGGCACCAAAGCTATGGCTAAAACTGCTGTGCAATTACAAT
TTGTGCAGCCGCCCATGTTATAATAGATTTAAAAAGTAGCTATCAGAACATGGGCTA
AAATCGACAGTTCCACCTATAATAGATCTTTTTTAAAAATATTGCAGGGACCAACTA
AAAAATATACTCAATTTATTGATAAATTAAAAGAGGCCATTGATAACAGTCTTAAAG
ACACATCTTTGCAAAAAATCATTTTAAAACAATTAGCCTTTAATAATGCTAATAAAG
ATTGTCAGGCTATTATCAGACCCATTAGGGGGGAAAAAAGTTATAAAATACTTAAA
AGCCTGCAAAAATGTGGAGATGATTCAACATAAAGCTAAAATAGCCACTTTAAAAA
CCTTATAAATTTCCCCAAAGTCTAAAGTTAAATGTTTTAACTACAGCAAGCCAAGAC
ACATGCAGGAGCAGTGTCACTTGCCTCGACAAACAGGTCTTTCTCCTGACAAAGGAG
GGGCTATTAAAACTAAGCCCCTCAGGCTCTGCCCAAGATATAAAAAGAAAAACCAC
TGGCTGACTAAATGCCGCTCTAGATTTGATAAACTAGGTAATGTTTTACCCATTCAG
ACACTTCCTTCAAAACACTAAAACAGGGGCTCTCCTCTAGCCCCATTAAACAAGGAA
GACAATCAAATATTACTAAATTAACAGCGACTACCAGACATAACACATTTATAGAC
ATTCCTACTCCCCGAGATATAAAATTATTAATGATAAATAATTCTATAAAAATCTTA
GCGAAATATTTTGGCCCTATACCAAAAAATACTATAGGCCTCCTATTAAAACAAAAC
ACCATGCACGAGATAATTGTACATACTAAGATCATTAATAAAGATTACACGAATAA
AATTACAATAATGTTGCATGTGACTCACAACTTGTATTTACAAAAGGGTAACAGATT
TGCTCAGCTATTGCTATTACCTTATGTGCCCCACTTAAAAAAAAAAAAAACAGACACC
AAAACAGGTGGCTTTGAGAGTACCAACATTACTACAGCCCTTTCTACTGTTATAAAA
AAATCAATAAACCCATGTTAAAATTAAAAATTAAAACAAAAACTTTCAAAAAAATG
TTAGACACTGGGGCAAATGTTTCCATCATAAAAACAAAAAAATGGCCTTTGGACTG
GCCTACAATTTTAATCTCACACCAGTTGGTAAAAATAAAAACTACAGATGCAGCTCA
AACTTATGTTAGTTCATCTTACTTACAAGTCCTGGGCAGTAATCAATTAGTCACTTAC
ATTAAACCGTATATCGCCCATTACCATTAAATTTGTGGGAAAGAGACTTTCTACAAC
AAGTTCAAGCAACTATACAATTAAATAAACTTTTTCTTAGGAGTCACTGAGATAAAG
CAACTAAAGTTAAAATAAAAGTCTGATAAACCTATCTAGACAGCTCAGTGGCCCCTA
TCAAAAAAAAATAATAAAGTCCGCTTTACATACTGTGGTGGCTAAACTACTATAACA
AAATAAAATGAAAACTACTCAGGCACCATAAAATTCACCAGTTTTTGTCATTAAAAA
AATCAAATAAATAAAAAATTCTAAAAGATTTAAAAATATAAACACTATAATGATTC
CTATGGGAACATTACAACCAAGACTCCCAAGCCCTGTTATAGTCCCTAAAGACTGGG
CTATTGTGATTATTGATTTACAAGATTGCTTTTTCACTATACCTTTACATGCAAATGA
CAGATAACGCTTTGCTTTCTCAATACCTTATATTAATAATCAATCCCCTGCTCAACGA

TATCAATAAAAGGTCCTGCCCCAAGATATGATAAACTTCCCTAGAGTCTATCAATTT
GTTGTTGATAAAATTTTACATCCTATCAGACAACAATTCCCTGAAGCATATCTCATTC
ATTACATATATGACATTTTATTGGCTTCTCCCTCAAAATCTCAATTAAGTTTAATAGG
TAATGAGGTCATAACTAATTTAACTAATCATGAGCTACTAATAACAAAAGATAAATT
ACAACACCTTTCCCCTTTTAAATATCTTAAATATCTTATGGACCGCTCTACTGTAAAG
CCACAAAAACTTTCTATTAAAAGAGATAATTTACAAACACTTAATGATTTCCAAAAA
CTTCTTGGAGATATTAATTGGCTACAACCTACCTTAAAAATTCCCACATATGCTTTAC
AAAACTTATTCAAATTATTAAAAGATTCCTCTGATTTAAATAGTCCCCGACAACTTA
CCCCGATGCTAAAAAAATTACAATTGTTAAAACAGAAAATTCAACGAACATTTGTTT
ACCATATTAATTACAATTCCCCTTTTCAGATATATGTCTTTGATACTAAGATATCTCC
TACTGCCATTATAAAGCAAGATAATCACCCTATTAAATATGTATATCTCCATTCCAA
ATAGACTAAACACATTGTTTCCTATATAGACTTAATTAAAAAAAAATCATTTTTCTTA
CACGCTCTTACTTGTACACTATAGCTAGATATGACCCTACTCAAATTTACCTACCTTT
GACAAAAACAAAAATTGATAATCTCTCCAGGTGTCCACTACCATTAAGATAGCCCTT
GCTGATTATTCAAGAGAGTTATTGGCCAACCCACCTAAAAAAAATATGAAATTTCTT
ACAAAATACTTCTTTTATCATCAACAATATTATTTCTAAACACCGTCTCATAAATGCA
CCTAATTATTTTATAAATAAAAATAAGGCAAGATGGACAACGATAATAGGTCCCAA
CCTACAAAAAAAATTAAAAATCCTTATCAATCTGTTAAAAAAACAAAATTATTCACA
TTATATTGCTTACTTACTCTAATAAAAACCCCATTAAATGTTTTAACTGATTCTCGCT
ACTTGGCACATCCTTTCCCATCCTTTGTAACAGCTCATTTTATAGCCAATAAAAATGA
TCTTATACATTTGTTCTTATTGATTCAACAAAAAATAAGAGCTAGACTCCATCCTTTC
TTTATTACTCATATTTGTGCTCATTCCCATTTACCAAGACCCCTCAATTTAGGCAATG
ATTTGGCTGATCGCCTCATCGCCCCTATATTTTCTTCCCCCAAACAAAAACATCAGCT
CTTCTATACTAAAGCTAATAGACTACACGTTCAATATAAGATACCATTATAAACAGC
TAAAAAAAACTTGTTCAAGACTGTGCCACATGTGCCCCCTTTCATTTGACCAGTAGT
CCCCAAGAGACAAATCCTAGAGGCTTACAAACAAATAAATTATGACAAGCTGATTT
TACACATTCCAATCTGCCCCCTTTTAAATTGTTATTTGTACTCATAAATACCTTTTCC
AACTTCATTTAAGCAGTTCCTTCCACCACCGAAACTACTAAAGCTGCCGTCACAGCT
CTTCTGCAATATTTTTCAATGATGGAGATCCCTGCTTCCATCAAAACTGACAATGGTC
CTGCTTTTACAGCCAATGCTTTTCGTAATTTCATACATCAATGAGATATTTGCCATCT
TACTGGCATCCCGTACAACCCTTAATGTCAAGCCATCATTAAATGGGCCCACCGTAC
ACTCAAGTTCATTCTTAATAAACAAAACAGGGAAAATAAACCAAGGGACACCTATG
GACCTAAAGCCATTTTGCCTATAGCCCTTCTAACAATCAATTATTTTTAATTTGCCTC
AATCAATTATTTTAATTTGCCTCTACATAGTCAGGAAACACAAACAGAGGGACATTT
TTCTGATTCACCCTCACATATGCCTAAACAAACTGCTGTTTGGGTTAAATGTCTTAAT
CAATGGTGGCCAGGAACACTTAAGTTCCTAGGCAAGGGATATTATCTTGTCATTTTA
GATGATGGAACAGAGCAGTGGGTCCCACTCAAAAGAGTCAGAGGATGGACAGACCT
TGCTCCACACCCCCTGACAGGTCACAGCACTAAAACAAAAATCGCAACAGATGCCT
TCTGAGCCTCCTTATGACAATGTATCTTAAAAAAAAAAAAATTGTCTGTGCCGGGAAT
GGACTGGTCCACCTGTACAAAAAGGTTTGAAAGTTTCTGTTCACACCAACATGACTT
TTGTTGATTGGGGGCCTCATGGACTCTTTGTAAATTGCTCAAAATCACACACACACA
CACACACACACACAAATAATAATAACACCTGCCAATGGTATAATGTTTCAGCACCA
CGTTTTAACAGAACGCAAACAAGACTTTGGCATCAACGCGATGAACTTTTGAACTGA

TACAATGGAGATCTTTCACCTCCCAGACCCCAGATAATCAGTCCAGTCCTGGGGCCT
GAACACTGGAATCTTTAGAAAATTCCTGCTTCTTTGTTTCAGTTTAGGGTCTCATATG
CTTAAGCACATGTTTCCATCCCACAAAATTATACTATTGAGTATAATTATACTGGTTA
TGTTCTGGCTTGTGTAAATGCGCCCTATGTTTTTGCTATTAGACAATTTAAGAATAAT
GGCTCAATTCTCTCCTGTACTGATGGTCATTTGTATACTTGTTTAAATCATAGTGTGC
CAATTAATGTTACTAAAAACAGTGTATTTTTAGTCTGGAAAAGGACTGATTTGTGGG
TTCCAGTTAAGATTTCTGAACCTTGGTCAGATTCCATGTTCTTGTCTTTTGTTCTGAG
AAAATCCCTAAAAAAACCAAGCAAATTTTTTTTTTTTATTGGTTAGACTATAACTACC
ATAGTGGGTATTATTTCAATTGTAACTGTTGGTACAGTTTCTAAAATGGCATTATATA
ATTCTATTCAAAATCATGATTTCATTACTGCTTGTAAAAAGGATTCTTATGATCTCTG
GGCCCAACAAGCTCAGATAGATCAACAAATACAAATCACATTTACCTTCAACAACA
TTTCCCCTGTGTTATATAACACGGACTTCTAAAATATATTATCAGATAGTACTTCCTT
TTTAAATCCAAAATATTGGGTACCCCATAGGATGGCTTTTCATGCGCTGATCTATTTG
TTTGTTATTGTTCTTATAAGATTACAAACATTATGTGCTCAAGCCACCGTCACCCAAA
AAGCGAGAGTAACAATGGCTACAACAGTCCTTACTCTTAAAAAGAAAAATGTGGAA
GAGCTTGACGGATTTAAGTAAATACTGAAAATGTATTTACTCATGACGAAGGGTCAA
TACACCCGGCCTTGAGGCGTTCGAACTTCCTTCTGACCACCTGTATCTGAGAGCAAG
GAATGTGGCTTCCTGATAAGACTCCCTTTAGAGGTTCGCCAAAGCTATGTTATGTCT
GGGGAGATGGGAACTGTATTTTATGCTTGAATGCTTTAATGTTTACCTGGAAAGGCT
ACGTACAAGTCTGCTGTAGCTATATTCTCTGAGAATTATAAAACTGCCCAGTTATAT
AATGAACTTTGTCAGTCCGATAGAGGCTGAGCCTGAGTGTCTTTTTCAGAGTGCAGT
TATACAAGCCTTATACCTCTGGAGTGGACTGAGACATTTGGCAGTCTTTTGGAATGG
TGGCAGGAACCCTGGAGCTCCTCTCGACTTTCCTGTTGAGAGCGCCTCCTCTTGAGA
TGTGACGGGAATGCCAGGAATCCTTTCCCGACGAAGCAGGGAAAGGAATCCTCATC
TCGAGCGGATGAGGGAGAAACGTGGCTCTTCTTAAAGTTGGTGTGACCCTCGGTGTT
CCTCTCGAGTGGAGACGGGTATATCGAGGAACTTCTTGAGTTGCAGCAAGGGTGTCA
AGGACCCTTTCGAGGTTCAACAGGGAAGGTGTGATTTCCCTCGAGATGCCACAGTGG
AAAAGGGCCTCATGTCGCCTGGAGGGGAGAACCTCCTGGTTTTTCTCGAGTTGCGGT
TGATTTCTCTCCAGTTATGACGGGGATCTCAGGGACTCACTCGTGTGGCCTCAGGAA
CGGCCAGTCTCCATGCGAGTTGACCAGGGCCTCTCTGGATTCCTCTCCAGTC

ERV_23

ATCAGGGAATGTTTAACAGGAGGATTCCTACATGCTGTTTCTCATAAATCCTCTGTTC
CTTATCAGTTTCTGTTGCCAGCAACCAGTGGTATGGCATGCCGCTCCCAGGACTGAG
GTCATAGGAGGAGACAGGCTTGAATCTCCTTCAGTAAACATTCTTACAACAAAACTG
CTTAGTCTCGATCCTCTTTCTTGAGGTGTGGATTGTCTCCTGATCTTGTGACCATTATT
AATCCCTGTTCCCTTGGTAACGGTTGCTGTACGTTTGGTTTTCTGATCTTTATCATTGT
CGAAAGAAATTTCTTGTACAACAGCCTATATATACTCACAGAAAGATCATGAAAGC
ACCTTTGCTCCATCAGAGCTTGAGTCTTCGTGTCTTTCTTTCTTTCTTTTTCTCTCTCTC
TCTTTCACTTTCTTATCATCGACTCCAGACTGCCAGGTTCCGGTCCATTAAAGGACCC
CAACAAGTGGCGCCCAAACAGGGACTTGGCATACGTGGCATTTCGGACAGAGTGGC
TCAGGGGCTACTGAGGTTGGGACCTATTGAGGTCGGTAAGTACTAAGACAGGGGTC
AAACGGCCAGAAAGCCCTAACATTTCTCTACTTTACTTCACCATTTGCTTAAAGCTC

AGGAACTTTTGGTTTTGCGCCAATGAATAGAGGCTTGCTTTCAAACAGTGGTTAAAT
GCAGTCCTTGGTTTCCTGATAAAGGCAGTTTTGATTTAGAAACTTGGCACCAGGTCA
AAGAAAATGTTTAATGAGCTGCCAGGAAGGGAAAAAACATTCCAATTGATTTATGG
CCCCTATGGGCCCTCATTAAAGCTGTAATTCTGCCATTTCAAGGTAAATTCTAGCCCT
CCCAATATTCGACAACAGACAGAACACTTATTACATGAATATAAGTTAAATGATAA
AACTTTACAAAAGGTCCAATTAAAATAACGTAAAATATTTCAAAAATTTCGTACTAA
TCCTATCCTGGCTACTCCAAATGCTCCTCCTCTCCCAACAGGCGCAAGTCCAAAAGT
CTCTCCTTTGCTAGAACCTAATAATTCTGATAACGTCTCTTGAGACACCTTTTGACAC
CAAAACCGGCAATGCTTTTCTAGATAATAATGATAAGTCCTTAACACAGACTCATAT
TTGCAAAAAATTGGCACTTACTTGCTCTCCTCCATGACAGAGGCTCTCTCAATTACTG
GCTTTGCAATCACAAATCTCTGAAGCCAATGGTTTCTCTGCCTTTCCAGTTTTAAGAA
ATCCTGATGCTCGAGGGCACATAATACCGCAATATGAATGTATTATTATTATTTTTTT
TACAAGCAACAGATGAAAAAGGCTATAACTATGTATAGTCCACATTCGCCTTTTACT
AAAGAGCTTCTAAATGTTGTGGCATTTTCTATTGGAAATTATTCTCTATGATTGGTGA
GTTTTAATAAAAGCTCTCCTTAAACCAGGAGAATATCTTCAATGGACAATGTGGTTT
CATGATAATAGCCAGAGATCACGCTAACAAGAATGCTTGAGCTGGCACTCCCCCAA
ACCAAATTACTTTTGAAATGTTAACTGGTGCCCGACAATTTGATACCATAGAAGCTC
AAATACAATGCCCTCCCTTGTTGCATGAAAATTAAAAACAGTGGCCCTTGAAGCTTG
GGATCAAATTACTCCTCAAGGAGAGCCTACAGGTAGCTACACTAAAACATTACAAG
GACCTAATGAACTCCGGGAGTTGGTGATGGACAGGGAGGCCTGGCGTGCTGCGATT
CATGGGGTCACAAAGAGTCGGACACGACTGAGCAACTGAACTGAACTGAACTGAAT
GAAAATTAGGCCGATTTTTTTTAGCTAGATTAGAAACTGCTATTTCCCGTACTGTAAT
CGGAGAAGAAACCAAAAAACAGCTAGAGAAATTACTTGCTTATGAGAGTGCAAATC
AGAGATGTCAAAAAGCTATTGCTCCAATTCGTGAGACTGGGACTATTACTGATTGTT
TGAAGGCTTGTTGCAATCTAGGATCAGAAGCTCAAAAGATGCAAATGCTAGCTGAG
ACAATGGCTGCTGCCTTTAGAAAGGGAAATGAAGGAGGAGTTACATGTGGAGATAA
AAACCATTTAAAAAGGGACTGCCCTAAGAAGGCTAATAAAAAACTTCCAAGAACCT
GCCCTCACTGCCATCGATATACCAGCCCTAAATGATTTTTTTCCTTTACCCTCAAGCA
GTCCCTTCTAGAGTACCTACTGGACTTTTTGGACCCCTACCCCCACAAACCTTCGGTC
TTTTACTTGGTCAATCTAGTTTGATTACTAAAGGAATTACTGTTCACCCTGGAATAAT
TGATTCAGATTATAAAGGAGAGATTAAAATTATGATGTCATCTCAGAGTCTATGGCA
ATTCAAAAAGGGGGATAAAATTGCTCAATTGCTTCTTTTGCCTTACATTTCTATTAAC
TCCTCTAATAATGTACAGACAGGTGGATTCAGCAGTACAGATCAAAAACAATCCTTA
TAGACATCATTGGTATCTAATTATGCCCTACCAAATATAAATATCAAAATTAATGGT
AAAAGATTTTCTGCTCTCCTCGACACTGGATCTGATATTACTATTATTTCAAACACTT
ATGGCCCAAATCCTGGCCTATATAAAAGGTCTCTTGCCAGATTGTGGGGATTTCTCA
AACTAAAGTACAATAAGTCTATCAAAGTGTTCAAATATACCCATGTGAGGGACCAG
AAGGCCAACCTGTAACATTAAGACCTTATGTGATAAATGCACCCCTTAATCTAATAG
GAAGGGACTTACTTATGCAATGGCAAACTCAGATATACATTCCACATTTTTCCTAGG
GGCCACTGCTCATTTAACAAACAAAGCAATTATTAAAATAACTTGGAAGAATAACA
AGCCTATTCGGACAGAGCAATGGCCCATTGTGACAGAAAAATTACAGGCTGCTAAA
GAACTTACAGACACACAATTAGAATGAAAACATATTGAGGAATCTTGCTCTCCTTGG
AACTCTCCTATTTTTGTTATATATATATATATAAAATCTAACAAATGGCATCTCTTAA

CAGACCTTAGAAAAGTTAATGCATCTATGAAACCTATGGGTGCATTATAACCAGGG
ATCCCATCACCTACTACTAGTCCTCAAAATTGGTACATTATCATTACTGATTTACAAG
ACTGCTTTTTAAATATACCTTTACACTCTTTAGACCGAGAGAGATTCACTTTCTCTCT
CCCTTTTCCTAATCACATTGGGCCTCATAAAAAATTTCAATAGACTGTGTTACCTCAA
GGTATGCTTAATAGTCCTACTATTTGTCAAAATTTTGTAGCCAAGGCTTTATATCCAA
TGTGACAGCAATTCCCTCATGCTTATGTCATTAATAATACACTGTAAATACACAAAG
GAGAAATGATATTTTTGACACTGAATAGCCATGATATTCTTTAGACTCCAGAGAAAA
CTGATTAAAATAAAATCTGTTTTGAAATTGCAAAACTGGTTCTTCTTACACGTGCAG
TTAGGAAAAGGTTAACTTGTTAAAATACTGTTTTGGAGCTCAAATTCTGCCTGGGAA
ACAAAAACAGGCCTAAGAAAAAACCTAAAGTTAACTGTTATCATGTCCTTGGGATA
CACAGCCCACTCTGTCTGGGAGTCCAGCCATAAACAAATTGGTGGAACTCAGAAAA
AAAAAAAATCAAAAAGATATTTTAAATTTCAAGTGGAAAATGATGTCTGTCTGTCTA
TCTAAAATTATCTATGTCTCAATGTATGTCTTTGTTTTTGGACAATATGAAGTTAATG
AGCTCTATTTAAATTCAAGTTCACGTGAACGGAAAAATATTCAATAATAAATATAAT
GTTTATTTAAATATAAATATAATTTAAATACAATTGTTAATCTAATTAAGACATATCT
TAAATTATCAACATTATATTATACTTTTGTTGTGCCTAGATTTAAGGTAAACTAAGTT
TGTCAACAAAAAAGTAACTCTTTATATAAATAAATATATAAATGAGATGAAAACTTT
TAGATAAACTCTATTAAAAATAATTATATTTTAAAAATGTCTATCTAAAATAATCTCT
CAGAATTGGGGTAACTTAAATTTCTAGAATTGTACTAAACTAAATGATAAAAGTTTA
TTAAATAGCTAGGTCATTTCCAAATAAAATAAGATTTTAAAACATTAATTACTGAAC
ACTAACTTCCTCTTACAAAAAGTTTTTCTTACAGAAAAACTAAAGAGATTTTAGACT
ATTAATAAATATATAAATATAAATATATATTTATATAAAATATAAATATATATTTAT
ATGAAATATGTTAATATAATAAATATATTCACCAATGCTAATATACAAGATACTTCA
TAGTTGCTAAAGAAAAGTAAGATGTATGCTTTTAATAAAAGGATATAAGAAATGGC
AAATAAAATGATGAATACAAAAATATAAAAAAGGTTTATGACAAATGAAATAGAAT
TTTATGGCAAATGAATGTAACTCATTGCCCTGAACTTTCTTTCTCTTCCTTCTTACAT
GTCTCATTCGATACAAATTCTTCTTTTATATGGGCCACACCTCTCCGAGGTGAAGCTA
CATGACATGTTATAACCCACCTGTTAGCTTACTCTGCAATAATGAGAACACCTAATT
CTATAAAAACAGACAATGGCCCTGCCTATATTTCTAGGCAGTTCAAATAATTTTTAC
ATTCATTCTCTATTAAACAGGTTACAGACATTCCTTATAATCCACAAACACAAGACA
TAGTTAAACAAACACAGTACACACTGAAACTACAAATAAAAAATTAAATAAAGGGG
AATAAACAGGAACACTTTTATCTTCCTCATCCAGAAGAGACTTTGCTAGATTCCAAT
GCAATATATTCTTTAAGCCTATAACTATTGTTAATATAGCTTTATTTGTTTTAAATTTT
TAAACTTACCGCAAGGAGAAATTTTGACCAAAGCAGAAAAGCATTTTGAGACACTG
AAGGACACCTCCCTTCTTTTGCCCATTTGGTATCAAGACGGGTTAACTAATCAATGG
AAATCTAGGAAACTAATCTTACAGGGAAAGGGGTATGCTTGTATTTCTCCAGATGGA
TCCAACGAACTCACATGGCTTCCTCTTCGGAAGATTTGACCCAAGGGGGCCCCCAAC
ATTCAAACTAGAGACGAAACAACAAAGACCCCAAGAGGAAGAAATTCCAATACAA
GCCATGGCAGATTTAAAAATTTCCAAAAAACACCGCACTCGACGTCATAGACCTTAT
GATCTCCCTACTTGGGGACAGGTAAAACCCCTTACTAATCAAGCTGAAAATCTGATT
TCTCAACAGGGAATGCCTTGGAATCCTGAAAATATTTTTGTTGCTATGCTTGCTTTGT
TTGCTTTTGCTTCCCCCGCTCAGGCTGACTTGATTAATCACACTTATTGGGCTTATAT
ATCTAACCCCTCCTTTCATTGTAGGTTATAGAATGGACAGATATAAGACCAATCTCA

TCCACTAATGACTCAACACATATGCCCCCTCCTTGGAATTTGGAGGCACCCTCTCAT
CCTGAGGACGAAGGAAGACTAACATTTCTCTAGGCTATGAAATCCTTCCTTTATGCA
TGGGCCCAACAAAATTATGTATTAATGTTAGTCAACAAACATGGGCTTTCATCCTGC
CTCCAGAAAGGAACTTCCACACATTGCTTGGACTGTTTACTGCCCTGTCCTTTTATAA
AAACCATGTCAATACTACATGTCAATACATACAAAAGTTAGAATGTAAGGGGTTTAC
TTATAAAGACTTTAAATATACTCCTGTTTATTGAGATAAATGTCAAGATAAATCAGG
GAAATTAATGTTTATGGCCAATTACACCATTGTTAATTGGGGACCCCATAGTATGTG
TGTATCTAACTGCTTAGATGATATTAACAGCACTATGTGTGACTATGCTACTCAAGT
AGCATAGAAGGTTACTAACACTACAATATAGCATTACCATGACAAAGGACTTCTTGG
GTGGCTTGAAGGTGGAATGGCCCCCCCTTGTCCTCAAATCATCCTCAATAAACAGAT
TGGGCCTGAACAATGGGACATGTGGAAACTTGCTGTGAGCACCAAAGAACTTGGAA
CTTGGACTGGACATTTCACAGGGACCAATCATAGTCATCGGAGAAGGCAATGGCAC
CCCACTCCAGTACTCTTGCCTGGGAAATCCCATGGATGGGGGAGCCTGGTGGGCTGC
AGTCCATGGGGTCACTAAGAGTTGGACACTACTCAGCGACTTCACTCTCACTTTTCA
CTTTCATGCACTGGAGAAGGAATGGCAACCCACTCCAGTATTCTTGCCTGGAGAATA
CCAGGGACAGAGGAGCCTGGTGGGTTTCCATCTATGGGGTCGCACAGAGTCGGACA
CGACTGATGCGACTTAGCAGCTGCAGCAGCAGCAGCAGCAGCAGCAGCAGCAGCAG
CAGCAGCAGCAGCAGTTGTAGCCATAGGAACTATTTCTTTTGTTATAATCAATCATA
TTTCATACAAGCTTGTATTCCCCTTCCTTTTGTTCTAGCTATAGGAAGTTTACAATTA
AATGAGACTTTATGTTCTGTAACTTGTATAAATTGCAAATTATATACTTGTCTTAACT
CTTCTGCTTTCTTAAGAAATGAATCCCTTTAGATTCTCTGATCTTGACGTAGTCTGTG
GTTACCAATAAATCTCCAGCGGCCCTGGGAAGAAGGTCCCATGGCTGGACTTGCTTC
CTGGTTACTTACTAAACTGCTCTGGTGATCCAAACGATTCATTGGATGGCTGATTCTT
GGCATTTTGGGATTAATAGCTGTTTGCACCACTGCTGCTGTCACTGGTGTTGCTTTAC
AAACCTCAATTCAAACACATAATTTTATCCAAAATTGGACTAAAGATGCTCATACTA
TGTGGGCCACTCAGGCTCAGATAGGTGAGGATATTCGAGATGAAATACAGGAACTA
AAAACAGCCATCAAATGGGTTGGAGACCAATTAATAGATGAAAAAACAGGTGATGC
TAAAATGTGATTGGAATTCTACTCACTTTTGTGTTACTCCTGTTCAATTCAATCATAG
TGCCTACAACTGGGAACAAATCAAATTTCATTTACAAAACATACATAATAATGCTTC
TCTGAATGTACAATTATTACAAAAAGAAATCTTTGAAACCTTTTCTAAAAATCTGCC
CTCTTCCACTAATTTGAAAACTTTAGCTGAACAACTAGCTGATCAATTATCTGGGCT
AGACCCATGCAGATGGTTTCAAAGCGTTACTCGCACCATCGGGTCTGGAACTGTAAT
TTTGGTAATTGTCTTGATAATTATATTTGTCATTTACCATTGCCTTCATGCAAAAATT
GTTAAAACTAGACAAACTCAAATGGTCAGAACCCTTTTTACAAATATTATAAATAAA
TAAGGGGGAATTATCAGGGAATGTTTAACAGGAGGATTCCTACATGCTGTTTCTCGT
AAATCCTCTGTTCCTTTTCAGTTTCTGTTGCCAGATACCAGTGGTATGGCACACTGCT
CCCAGGACTGAGGTCATAGGATGAGACAGGCTTGAATCTCCTTCAGTAAACATTCT

ERV_24

TGCGGGGGACTACCCGTGAAGGGTTAAGTCTTGGGAGCTGCTCGGCGTTATGCAGA
GCCCTAGGACATGTGCCTAAGCTCCCTGTCCCGCCACCCTCAAGAATTTTTATAGCC
CTTAAGGCTCCAAGATGTTTGGTTTCGGCAACATGTCATAGAAGATAGATTAGCTTA
TTGTGATCTGTATACAATGGTACGGGTCTGGTGATTGTATCTGGAGATGAAAAACAA

TCTTGTAAAGGTCAGAAATCACGTATTTTATCCTATATATGCTGCAGCATAATAAAG
CAAGGTATCAGCCATTTTGGTCTGATCCTCTCAACCCCATCTTTTGTCTCTCTCTTATC
TTCTTAGCGGGGACGCTCCGTTCTCTCCCTGTGCAGGTGCGACTCTTGCTTGTGCTGG
CCGCGGCAGGTGGCGCCCAACGTGGGGCGGTTCGACAGTTTTCCTCGCCACTACTCT
CATTAATTAAAAAGAGTGAGTATATAAGTATACAAGTGCATTACAATTGAGGAGGA
GTAGTAAGGTATATAGTTGAGAGTATAAATATGGGACAGACGCATAGTCGTCAATT
GTTTGTACATATGCTATCTGTAATGTTGAAACATCGGGGAATTACTGTTTCCAAACCT
AAACTAATCAATTTTCTTTCATTTATTGAGGAGGTTTGCCCTTGGTTCCCCAGAGAAG
GTACAGTAAATTTGGAGACATGGAAGAAGGTAGGGGAACAAATCCGGACTCATTAT
ACTTTACATGGCCCTGAAAAAAATCCCTGTTGAAACTTTATCCTTTTGGACACTAATTC
GTGATTGTCTGGATTTTGATAATGATGAATTAAAACGTTTAGGAAATTTATTAAAAC
AGGAAGAAAATCCTCTCCATGTTCCTGATTCGGAACCCAGGTATGCTGTTCCCGAGG
GAGTTGAAGGTGATCCTCCGCTTCCTAATTTATTGCGTCCTTCGGATAATGATGATTC
ACTTTCCTCCACAGATGAGGCAGAATTAGACGAAGAAGCTGCTAAATACCATCAAG
AAGATTGGGGTTTTTTAGCACAAGAAAAGGGGGCGTCAACATCTAAAGATGAATTG
GTTGAATGTTTAAAAAACCTCACTATTGCTTTACAGAACTCAGGAATCAAGTTTCCT
AGTAACAATGCCAAATCTCCTTCTGCTCCGCCTCTTCCCCCTGCTTATGCTCCTTCTG
TTGTGGCTGGTCTCGATCCCCCTCCAGGGCCCTCTCCACCGTCTGAAAACATGTCTCC
GCTGCAGAAGGCATTGAGACAAGCACAGCGACTTGGTGAGGTTGTCTCTGATTTTTC
TCTTGCTTTTCCTGTCTTTGAAAATAACAACCAGCGTTATTATGAATCACTGCCTTTT
AAGCAACTAAAAGAGTTAAAGATTGCTTGTTCACAATACGGTCCTACCGCTCCATTC
ACCATTGCTATGATAGAAAATTTGGGTACTCAAGCTTTACCCCCAAATGATTGGAAA
CAAACAGCTAGGGCTTGTCTTTCAGGGGGAGATTATTTACTATGGAAATCTGAATTT
TTTGAACAATGTGCTCGTATAGCTGATGTTAACCGACAGCAAAATATACAAACCTCC
TATGAAATGTTGATTGGTGAAGGCCCTTACCAGGCTACTGATACTCAACTTAATTTC
TTACCTGGTGCATATGCACAAATATCAAATGCAGCTCGGCAGGCATGGAAAAAACT
TCCTAGCTCCAGTACTAAGACAGAAGATCTTTCAAAAGTCCGACAGGGACCTGATG
AGCCTTACCAAGACTTCGTGGCACGGCTTTTAGATACTATAGGTAAGATAATGTCAG
ATGAACAGGCTGGGATGTTATTGACAAAACAATTGGCTTTTGAAAACGCTAACTCTG
CCTGTCAAGCTGCCTTAAGACCTTATCGTAAAAAGGGAGATCTGTCTGATTTTATTC
GCATTTGTGCTGATATTGGACCCTCCTACATGCAAGGCATTGCTATGGCAGCAGCAT
TACAAGGAAAAAGCATAAAGGAGGTACTTTTTCAGCAGCAAGCCCGGAATAAGAAA
GGACTTCAAAAGTCAGGTAATTCTGGTTGTTTTGTTTGTGGTCAACCTGGCCATCGG
GCGGCAGTGTGCCCTCAAAAACAACAAAGCCCTGTTAACATTCCTAATTTGTGCCCA
CGATGTAAAAAAGGAAAACATTGGGCACGGGATTGCCGTTCTAAAACGGATATTCA
AGGTAATCCTTTACCCCCGGTTTCGGGAAACTGGGTGAGGGGCCAGCCCCTGGCCCC
GAAACAATGTTATGGGGCAACGCTGCAGGTTCCAAAAGAACCATTGCAGACCTCTG
TCGAGCCACAAGAGGCAGCGCGGGATTGGACCTCTGTGCCACCTCCTACACAGTAC
TAACTCCCGAGATGGGGGTTCAAACCCTTGCCACAGGAGTGTTTGGGCCTTTACCTC
CAGGGACAGCTGGATTGCTCTTGGGGCGCAGCAGTGCGTCTTTAAAAGGAATACTTA
TTCACCCTGGTGTGATTGACTCTGATTATACAGGAGAGATAAAAATATTAGCCTCCG
CTCCTAATAAAATTATTGTAATCAATGCAGGACAACGTATAGCTCAACTCCTTTTAG
TTCCATTAGTTATACAAGGAAAAACAATTAATAGAGACCGTCAAGATAAAGGTTTC

GGGTCATCTGACGCCTATTGGGTGCAAAATGTTACCGAGGCACGACCAGAACTTGA
GCTACGCATTAATGGTAAGCTTTTCCGCGGAGTGCTTGATACAGGGGCCGATATTAG
TGTTATTTCTGATAAATACTGGCCTACTACATGGCCTAAACAGATGGCTATTTCCACT
CTTCAAGGTATTGGCCAAACTACCAATCCAGAACAAAGCTCGTCCCTTCTTACTTGG
ACAGATAAAGACGGCCATACAGGTCAATTTAAGCCTTATATTCTGCCCCATCTTCCA
GTTAATCTATGGGGGCGTGATATATTAAGCAAAATGGGTGTTTATTTATATAGTCCT
TCACCCACCGTAACAGATTTGATGTTAGATCAGGGATTACTTCCAAATCAAGGTTTG
GGTAAACAACATCAAGGCATCGTTTTACCCCTTGATTTAAAACCTAATCAAGAGCGA
AAAGGCTTGGGGTGTTTTCCCTAGGGACCTCTGATTCTCCTGTGACACATGCCGATC
CTATTGATTGGAAATCTGAGGAACCGGTATGGGTCGATCAGTGGCCCCTGACACAG
GAAAAACTTTCTGCCGCACAACAGCTGGTGCAGGAACAGCTGAGGCTTGGACATAT
TGAACCCTCTACCTCTGCTTGGAATTCCCCAATTTTTGTTATTAAAAAGAAGTCTGGG
AAATGGAGATTGCTACAAGACCTTCGTAAGGTAAATGAAACAATGATGCATATGGG
AGCCCTACAACCTGGGTTACCCACTCCTTCTGCTATACCTGATAAATCCTATATCATT
GTTATAGATTTAAAAGATTGTTTTTACACCATTCCTCTTGCACCTCAAGATTGTAAAA
GATTTGCCTTCAGTTTACCCTCTGTTAATTTCAAAGAGCCTATGCAACGTTATCAATG
GAGAGTCCTCCCGCAAGGAATGACTAATAGTCCTACGTTGTGTCAAAAATTTGTTGC
TACAGCATTAGCTCCCGTTCGTCAACGCTTTCCTCAGCTATATTTAGTTCATTATATG
GATGATATATTACTAGCTCATGCTGACGAACAGCTATTGTACCAAGCTTTTTCTATTC
TAAAGAAACACTTAAACCTTAATGGTCTTGTTATTGCTGATGAAAAAATTCAAACTC
ATTTTCCTTATAATTATTTGGGTTTCTCCTTATATCCTCGCGTTTATAATACTCAATTA
GTAAGATTACAGACTGACCATTTAAAAACTCTAAATGATTTTCAAAAACTTCTAGGA
GACATTAACTGGATACGCCCTTATTTAAAACTACCCACTTATACCTTGCAGCCTTTGT
TTGATATCCTTAAAGGTGATTCTGACCCTGCGTCACCCCGAACACTTTCTTCAGAAG
GGCGATCAGCCTTACAATCAATAGAGGAAGCTATTAGACAACAACAGATTACTTATT
GTGATTACCAACGATCATGGGGTTTATATATACTTCCTACCCCCGAGCACCCACAGG
GGTTCTCTATCAAGATAAACCTTTGCGATGGATATATCTGTCTGCTACTCCAACTAA
ACATCTGCTCCCTTACTATGAACTTGTTGCAAAAATTGTAGCAAAGGGACGTCACGA
GGCCATCCAGTATTTTGGAATGGAACCCCCCTTCATTTGTGTTCCTTATGCTTTAGAA
CAACAAGATTGGCTTTTTCAATTTTCAGATAATTGGTCTATAGCTTTTGCAAATTACC
CGGGACGGATTACTCATCATTACCCTTCTGATAAATTGTTACAATTTGCTAGCTCTCA
TGCCTTTATTTTTCCAAAAATAGTTCGCCGACAACCTATTCCCGAAGCGACACTTATA
TTTACAGATGGATCTTCTAATGGTACTGCAGCTTTAATTATTAACCATCAAACTTATT
ACGCACATACCAGCTTTTCTTCTGCTCAGGTTGTGGAATTATTTGCAGTCCACCAAGC
ATTACTAACTGTACCTACTTCCTTTAATTTATTTACAGACAGCTCCTATGTGGTCGGT
GCCTTACAGATGATTGAAACTGTTCCAATTATCGGCACCACCTCTCCTGAAGTTCTTA
ACTTATTTACATTAATTCAACAGGTCCTCCACTGTCGCCAACACCCCTGTTTCTTTGG
GCATATTCGTGCACACTCCACCCTTCCTGGTGCCCTCGTACAAGGCAATCACACTGT
GGACGTTCTTACTAAACAAGTGTTTTTCAATCAGCTATTGATGCAGCTCGAAAATC
CCATAACTTACATCACCAAAATAGTCATTCTCTACGGTTGCAATTTAAAATTTCCCGT
GAAGCTGCACGGCAAATTGTTAAATCTTGCACTACTTGTCCTCAATTCTTTGTTCTCC
CTCAATATGGTGTCAACCCTCGAGGTTTACGCCCTAATCACCTCTGGCAAACAGATG
TTACTCACATTCCTCAATTTGGGCGTCTTAAATATGTTCATGTCTCTATTGACACTTTT

TCCAATTTTCTCATGGCCTCCCTTCACACTGGAGAATCGACACGTCACTGTATTCAAC
ATTTGCTGTTTTGCTTTTCTATTTCAGGAATCCCACAAAACCTTAAAACAGATAATGG
ACCTGGTTATACTAGCCGTTCTTTTCAACGTTTTTGTCTTTCTTTTCAAATTCATCATA
AAACAGGAATTCCATATAACCCACAGGGCCAAGGTATTGTGGAACGAGCTCATCAG
CGTCTCAAACATCAACTATTAAAACAGAAAAAGGGGAATGACTTGTATAGCCCCTC
ACCGCATAATGCCTTGAATCATGCTCTTTATGTTTTAAATTTTTTAACTTTAGACGCA
GAAGGCAATTCAGCAGCCCAGCGTTTTTGGGGAGAACGATCCTCATGCAAAAAACC
ACTTGTACGATGGAAGGATCCACTTACCAATCTGTGGTATGGGCCAGACCCTGTACT
AATATGGGGACGAGGGCATGTTTGTGTCTTTCCACAGGATGCCGAAGCACCGCGTTG
GATACCGGAAAGGCTGGTACGCGCGGCAGAGGAACTCCCTGACACATCAGATGCGT
CGCATGACACTGAGCGAGCCCACGAGTGAGCTGCCTACCCAGAGGCAGATTGAGGC
GTTGATGCGACATGCTTGGAATGAGGCTCATGTACAACCTCCAGTGACACCTATTAA
TATACTGATCATGTTATTATTATTGTTACAGCGGATACAAAACGGGGAGGCTGCGGC
TTTTTGGGCATACATTCCTGATCCGCCCATGATCCAATCCTTAGGATGGGATAAAGA
AACAGTACCTGTATATGTCAATGATACAAGACTTTTAGGAGGAAAATCAGATATTCA
CATTTCTCCTCAGCAAGCCAATATTTCCTTTTATGGTCTTACTACACAATACCCTATG
TGCTTTTCTTATCAATCACAGCATCCTCACTGTATACAGGTGTCAGCTGATATATCCT
ATCCTCGAGTGACTATTTCTGGCATTGATGAAAAAACCGGAAAAAGATCATACCGTA
ACGGAACTGGACCTCTCGACATTCCGTTTTGCGGCAGACGTCTAAGCATCGGCGTAG
GAATAGACATTCCTTGGACTTTATGTCGAGCACGGGTCGCATCAGTGTATAACATCA
ACAATGCCAATGCCATCCTTTTATGGGACTGGGCACCTGGGGGAAAACCTGATTTCC
CCGAATATCGAGGACAGCATCCACCCATTCTCTCTGTAAACACTGCTCCTATATTTC
AAACTGAACTGTGGAAACTTTTGGCTGCTTTTGGTCATGGCAATAGTCTATATTTAC
AACCCAATATTAGTGGGAGTAAATATGGAAATGTAGGAGTTACGGGGTTTTTATATC
CTCGAGCTTGTGTCCCTTACCCGTTTATGTTGATACAAGGCCATATAGAAATAACAC
TGTCATTGAATATTTATCATTTAAATTGTTCTAATTGCATACTTACTAATTGCATAAG
AGGTGTTGCTAAAGGAGAACAAGTTATAATAGTAAAACAACCTGCTTTTGTCATGTT
ACCTGTTGAAATAACTGAAGAATGGTATGATGAGACTGCTTTAGAATTACTACAACG
CATTAACACGGCTCTTAGCCGCAAGGAAAGAAGTGTGAGCCTGATTATTCTGGGTAT
AGTATCTTTAATCACCCTTATAGCAACTGCTGTTACTGCTTCTGTATCCTTAGCACAA
TCCATTCAAGCTGCTCATACTGTAGATTCCTTGTCATATAATGTTACTAAAGTAATGG
GAACTCAAGAAGATATAGATAAAAAGATAGAAGATAGATTATCAGCTTTATATGAT
GTAGTTAGAGTTCTAGGAGACCAAGTTCAGAGTATTAATTTTCGCATGAAAATTCAA
TGCCATGCTAATTATAAATGGATTTGTGTTACAAAAAAGCCTTACAATACATCTGAT
TTTCCGTGGGATAAGGTGAAAAAACATCTACAAGGAATCTGGTTTAATACTAATGTT
TCTTTAGATCTTTTACAATTGCATAATGAAATTCTTGACATCGAAAATGCTCCAAAA
GCTACTTTGAATATAGCTGATACTGTTGATAATTTTTTACAAAATTTATTTTCTAACT
TTCCTAGCCTTCATTCACTGTGGCGAAGTATAATTGCTGTGGGCACGGTTCTGACTGT
TGTGCTTATCATAATTTGTCTAACTCCTTGTCTTATTCGTAGTATTGTTAAAGAATTTC
TACAGATGAGAGTTTTAATACATAAAAACATGTTGCAACACCGACATCTTATGGAGC
TTTTAAAAAATAAAGAGAGGGGAGCTGCGGGGGACTACCCGTGAAGGGTTAAGTCT
TGGGAGCTGCTCGGCGTTATGCAGAGCCCTAGGACATGTGCCTAAGCTCCCTGTCCC
GCCACCCTCAAGAATTTTTATAGCCCTTAAGGCTCCAAGATGTTTGGTTTCGGCAAC

ATGTCATAGAAGATAGATTAGCTTATTGTGATCTGTATACAATGGTACGGGTCTGGT
GATTGTATCTGGAGATGAAAAACAATCTTGTAAAGGTCAGAAATCACGTATTTTATC
CTATATATGCTGCAGCATAATAAAGCAAGGTATCAGCCATTTTGGTCTGATCCTCTC
AACCCCATCTTTTGTCTCTCTCTTATCTTCTTAGCGGGGACGCTCCGTTCTCTCCCTGT
GCAGGTGCGACTCTTGCTTGTGCTGGCCGCGGCAAG

ERV_25

TGAAGGAGCTGGGAGGCTTTAAGCAAATACTGAGAATGTATTTGCTCCACTCGTGAC
GAAGGTTGGAAGCTGGGACGAGCATAACAAAGGGTTATGAGCGTTCACCAAGTGCC
CAAGGCTGGGAACGGATGACGAAGGGTCATACGCCCGGCCTTGAAGCGTTCGGAGG
CTTCCTTCTGACCACCTGTTTCTGGGAGCAAGGACTGTTGTTTCATGATAAGACTCCC
TTTAGAGTTTCGCCAAAGCTATGTTACGGCTTGGGTGGTGGGAACTGTATTTTATGCT
TGAATGCTTTGACGTTTTATCGAGAAAGGTTACGTGCAAGTCTGCTTTATGCTCTGCT
CCCTGAGACCATATATCTGCGAAAGCTGGATAATAAAATTTGTCAGTCCACTAGAGG
CTGTCCCTGAGTGTTCCTTTCAGAGTGTGGTTCTCCGAACCTTACAACTGGTGCCCAG
TGTGGGGCTCAAGCAGCAGACTGATTTTAAACAAGGACTGCATTCCTGCAGAAGCA
AGGTAAGCAGGATGGGACATCAGACAAGTAAAATTCCTTTTGTTCATCTCATGCATC
ATTTCTTGAAACAATACGGGGTTAATCTGCCTGAAGAGCGGTTAACTAGCTGTTACC
AGACAGTGCTTGAATATAATCCATGGTTTCCGGAGGAAGGAACCTTGGATTTACAAA
CTTGGACCAGAGTAAAGAATAATGTTCTAAAAGCTTATACACAGGGGGTAAAAATT
CCCCCACAATGGTGGGTAACTTGGTCTCTTTTACAGGCCATTATGGAAAAATTAGAT
GGTTCAGGAGGAGATTTAGAGGTTGAAACTGTTCGATCCCTGCATGAATGTGAATTA
GAAGAGAAAGATCTATCAGAGGTTTTAAATCAGAAAAATGTCATGCTAGGGCAGAT
TACGGACAAACAAGAGTCACAGGTGCTGAAAGCTGTTAAACAATCAACTTTGCCAG
AGGTCCCTGATCCGCCGCGAGCTCATATTAACAAGCCTCTAACACCGGCCTTTCCGC
CCACTGCCACGCTGTCTGCTGCTGCTTCTGCTCCCCTATTTCCTAGCATTCCTCCTCC
GGACATGCCTCTTTGGGCGTGGGCTTTTCCAGTCCAATTTAATAATCCTCAACCTGG
ACATAATCAGTGGCAATCACCTGATTTCGGTTTGCTCACACAATTCAAAAAGGCATG
TACGCTATATGGTCCTACCTCACCCTACTGTATGGAATTTCTTAGGGGCTAAGCAGA
TCAATGGCTTCATGCAGATTTTTTTACAGTTGCTAAAATGGTTATGACTCCACAACA
GCTACTACAATGGCAAATGTGGGTCACGGATGAAGCCAAATTAATCTTGCAAGAAC
AGCAAAGCAAGGGAAACCCTACTGGACTAAATTTTGAAATTCTCACCGGCACCAAA
GCTATGGCTAAAACTGCTGCGCAATTACAATTTGTGCAGCTGCCCACGTTATACTGG
ATTAAGGAAGCAGCTATCAGAGCATGGGCTAAAATTGACAGTTCCACCTCTGATGG
ATCTTTTGTAAAAATACTGCAGGGACCAACTGAAGAATATGCTCAATTTATTGGTAA
ATTGAAGGAGGCCATTGATCACAGTCTTAAGGATGCATCTTTGTGAGAAATCATTTT
GAAACAACTGGCTTTTGACAGTGCTAATGAAGATTGTCAGGCTATTATCAGACCTAT
TAGGGAGCAAGGAGGAATTATGGAATACTTGAAAGCCTGCAGGAATGTGGGGACGA
TTCAACATAAAGCTAAAATAGCCACTTTAGAAACCTTAAATGTTTCCCAAAAGTCTA
AAGTTATATGTTTTAGCTGCAGCAAGCCGGGACACATGTGGAAGCAGTGTCGCTTGC
CTCGGCAAACAGGTCTTTCTCCTGACAAAGGAGGGGCTATTAAAACTAAGCCCCCC
GGGCTCTACCCAAGATGTAAAAAGGGGAATCATTGGCTGAGTGAATGCTGCTCTAG
ATTTGATAAACAAGGTAATGCTTTACCCATTCAGACACCTCCTTCGGGAAATTAGAA

CAAGGGCTCTCCTCTAGCTCCGGTAAACAAGGAGGACAACCAAATATTACTGAATT
AACAGTGGCTACCAGACATAGCACATGTGTAGACATTCCTGCTCCCCAAGATATGGA
ATTATTAATGGTAAATAATCTTATGAAAATCTTAACTGGATATTTTGGCCCTATACCA
AAAAATACTGTAGGCCTCCTGTTGAGACGAAGCAGCAACACCATGTGCAGGATAAT
TGTACATACTGGGATCACTGATGAAGATTACACGGGTGAAACTGCAGTAATATTACA
TGTGACTTGCAACTTGTATTTACAAAAGGGTGACAGATTTGCTCAGCTATTGCTATT
ACCTTATGTCCCCCCACTTAATAGAAAAGCAGACACCAAAACAGGTGGCTTTGGGA
GTACCAACATTACTGCAGCCCTTTCTAACTGTTATAAAAAAAAATCAATACGCCCAT
GTTAAAATTAAAAATCAGAGGAAGAACTTTGGAAGGAATGTTAGACACTGGGGCAG
ATGTTTCCATCATAAGAACAAAGGAATGGCCTTCAGATTGGCCTGCAGTTTTAGCCT
CACACCAGTTGGTGGGAATAAGAACTGCAGATGCAGCTCAAACTTATGTTAGTTCAT
CTTATTTACAAGCCCTGGGCCCTGATCAATTAGTCGCTTACATTAAATCGTATATTGC
CCCATTACCATTAAATCTGTAGGGAAGAGACTACAACAAACTCAGGCGACTACACA
ATTGAATAAGAACCTTTTTCTTAGGGGTCACTAAGATAAAGCCACTGAAGTTAGAAT
GGAAGTCTGATAAACCTATCTGGACAGCTCAATGGCCCCTATCAGAAGAGAAACTG
TCTGCTTTGCATACTTTGATGGCTGAACTACTACAACAAAATAGAATAGAAACTACT
CAATCACCATGGAACTCACCAGTTTTTGTCATTAAAAAGAAATCAGGTAAATGGAA
AATGCTAACAGATTTAAGGAATATTAACGCTATAATGACTCCTATGGGAGCATTACA
ACCAGGACTCCCAAGCCCTGCTATGGTCCCTAAGGACTGGGCTATTATGATTATTGA
TTTAAAAGATTGATTTTTCACTATACCTTTATATCCCGATGACAGACAACGCTTTGCC
TTCTCAATACCTTCCATTAATAATCAATCTCCTGTTCAACAGTATCAATGGAAGGTCC
TGCCTCAAGGTATGATGAACTCCCCTACGGTCTGTCAATTCGTTGTTGATAAAATTTT
GCAGCCCATCAGACAGCAATTCCCTGAGGCATATCTCATTCATTACATGGATGACAT
TTTATTGGCTTCTCCCTCAGAATCTCAATTAAGTTTATTAGGTAATGAGGTCATAACT
AATTTAACTAATCATGGGCTGCTAATAGCAGAAGATAAATTGCAACACCATTCCCCT
TTTAAATATCCTGGATATCTTATGGACTGCTGCACTGTAAAGCCACAGAAACTTTCT
ATCAGAAGGGATAATTTACAAACACTTAATGACTTCCAGAAACTTCTTGGGGATATT
AATTGGCTACAACCTACCTCGGGAATGCCCACATACGCTTTACAAAACTTATTCAAA
TATTAGAAGGTTCCTCTGATTTGAATAGTCCCCGACAACTTACCCCTGAGGCTGAGA
AAGAATTACAATTGGTAAAACAGAGAATTCAACAAGCATTTGTTTACCGTATTAATT
ACAATTCCCCTTTTCAGATATATGTCTTTGGTACTAAGATATCTCCTACTGCCATTAT
AGTGCAGGATAATTACCCTATTGAATGGGTATATCTCCATTCCAAACAGACTAAACA
CATTGTTTCCTATATAGACTTAACAGGGAAAATCATTTTTCTTGCACACTCTCGCTTA
TGCACTGTAGCTGGATATAACCCTACTAAGATTTACCTACCTTTAGCAAAAACAGAA
ATTCATAATGCTCTTCAGGTGTATACTACCATTCAGATAGCCCTTGCTGATTATTCAG
GGGAATTATTGGCCAACCCACCTAAAAGAAAATTATGGAATTTCTTACAAAACACTT
CTTTTATCATCAACAATATTGTTTCTGAACACCCTCTCATGAATGCACCTAACTATTT
CATAGATGGAAATAAGGCAGGATAGACAGCCATAGTAGGTCCCAACCTGCAAGAGA
AAATTAAAAGTCCTTATCAATCCGTTCAAAAAACCGAATTATTCACATTATATTGTTT
ACCTACTCTAATAAAAACCCCATTGAATGTTTTAACTGATTCTCGCTACGTGGCACA
TCTTTTTCCATCCTTTGTAATGGCTCATTTTATATCCAATGAAAGTAATCTTATACATT
TGTTCTTATTGATTCAGCAAGAAATAAGAGCTAGACTCCATCCCTTCTTCATTACTCA
CATTCGTGCTCATTCCCATTTGCCAGGACCCCTCAGTTTAGGCAATGATTTGGCTGAT

CGCCTCATCGCCTCTATATTTTCTTCCCCCGAACAGGAACATCAGCTCTTTATACTAA
CGCAATAGACTACACATTCAATATAAAATACCATTACAAACGGCTAGAAAAATTGTT
CGGGACTATGCCACATGTGCCCCCTTTCATTTGACCACTAGTCCCCAAGGGACTAAT
CCTAGAGGCTTACAAGCAAATGAATCATGGCAAGCTGATTTTACACATTACAAACTG
CCCCCTTTTAAATTGTTATTTGTAGTCATAGATACATTTTCCAGCTTCATTTGGGCAG
TTCCTTCCACCGCCAAGACTACTAAAGCTGCCGTCACAGCTCTTCTGCAATGTTTTTC
AATGATGGGGATCCCTGCCTCCATCAAAACGGACAATGGTCCTGCTTTTACAGCCAA
TGCTTTTCGTGATTTCATGCATCAGTGGGGTATTTGCCATCTTACTGGCATCCCGTAC
AACCCTCAAGGCCAAGCCATCATTGAACGGGCCCACCGTACACTCAAGCTCGTTCTT
AATAAACAAAACAGGGGGAATAAGCTAAGGGACCCCTATGGACCTAAAGCCATCTT
GCCTATAGCCCTTTTAACAATCATTTGTTTTAATTGGCCTCTACACAGTCAGGAAACA
CGAGCAGAGCGACATTTTTCTGATTCACCCTCACGTATGCCTGAACAAACTGTTGTT
TGTTCTTGATCAATGGCGGCCAGGAACACTTAAGCTCCTAGGCAAGGGATGTTGTCT
TGTCATTTTAGATGATGGAACCGAACAGTGGGTCCCAATCAGAAGAGTCAGAAGAC
GGACAGACCTTGCTCCACACCCCTCGACCGGTAACAGCACTAAAACGGAAATTGCA
ACAGATGACATTGAAAGACAGCAAGAAAATGAAACGTCCCCAATGCAATGTGCCTT
TTCCGACCTGGGCTCAAATGAAAAATCTGTCAAGACGAGCTGAGGATACTCTGTTAA
TGACCAATAGCGAGGTAACACCGGAAAAACTGTTGCTGGCCATGATGGCCGTTTTA
ACCTGTGCTTCTGGGGTAAGTGGTAATTACACCGATTGGGCTTATATCCCCGACCCG
CCTCTTTTACAAGTGGTGGATTGGACAGAATCATCTCCTGTGGTTTTCACAAGTGAT
AGCTTACATTTTCCTGCCCCCGGCTGGATCTAGGACCACGAATAAAAGGAGGAGGA
GGAAAGACAGTTAACTTAACACTTACGTATCCAGTGCTCCCCATCTGTTTTGGTGCC
CTCCCTTTGTGTCTCCACCTATATCCACAATGGTGGGCTTACTCTTTCTCAAATGGCT
CTTTCCGTCTTGGAATGTTCGCTACCATGACCTTTATTCTTAACTGGACAGACTACCT
ACCATGGACAAATAGCCAACTTCACCCACTATCTATTTAGGCCTGCTGAGCCTCCTT
GCGACAATGTATCTTGGAAGAAAAAATTGTCTGTGCTGGGAATGGACTGGTCCACCT
GTAGAGAGTTTGGAAAAGTCTCTGTTCATGCCATCATGACTTGTGTTGATTGGGGGC
ATCATGGACTCTTTGTGAATTGCTCAGAATCACAAGAAAATAATTACACCTGCCCAT
GGTGTAATGTTTCAGCACCACATTTTAACAGAACACAAACAGGACTCTGGCATCAAC
GGGATGAACTTTTGAACTGGTACAACGGAGGTCTTTCACCTCCCAGACCCCAGCTAA
TTAGTCCAGTCCCGGGGCCTGAACACTGGCATCTTTGGAAAATTCCTGCTTGCTTGTC
CCGGTTTAGCGTCTCATATGCTTATGCACATGTTTCCATCCCACAAAATTATACTATT
GAGTATAATTATACCAGTTATGTTCGTTCTTGTGTAAATGCGCCCTATCTTTTTGCTA
TTAGACAGTTTAGGAGTAATGGCTCAATTCCGTCCTGTACTGAGTGTTGTTTACTTGC
TTAAACCATAGTGTGCCAATTAATGTTAGTAAAGACAGTGGTTTTTTAGTCTGGCAA
AGGACTGATTTGTGGGTTCCAGTTAAGATTTCTGAACCCTGGTCAGATTCCATGTTGT
TGTTTTTCGTTCTGAGAGAATCCCTAAAAAGAAGCAAATGCTTTATTGGCTGGATTG
TAGCTGCCATAGTGGGTATTATTTCAGTTGTAACTGTTGGTACAGTTTCTGGAATGGC
ATTGTATAATTCTATTCAAAATCATGATTTCATTACTGCTTGGAAAAAGGATTCTCAT
GAGCTCTGGGCCCGGCAAGCTCAGATAGATCAACAAATACAAACACGCTTAGATGA
CCTACAAGCCGCCCTTATGTATGTGGGGGATGATCTGCATACTTTACAGGTACAATT
GAAGTTGTGGTGTCACTGGAATTTCACTACTTTCTGTTTGACCAATATGCCATACAAT
GCCACTGAATATCCTTGGGAACAAATAAAGTTACATCTTTTAGGTTCGAAATCAAAC

ACTAGTCTAGATATACAGAAACTGAAGCAACAAATCACGTCTACTACCTTCAGCAGC
ATTCCCCCTGTTATATAACACTGACTTCTATAATACACTATCCGCTAGTACTTCCTTT
CTAAATCCAAAAAATTGGGTACCCCATACAATGGCTTCTTACGCGCTGATCTGTTTA
TTGTTTGTTACTGTTCTTATAGGATTCCGAACGTTATGTGCTCAAGCCACCGCTGCCC
AAAAAGCAGGAGTAACCATAGCTGCAGCAGTTCTTGCTCTTGAGGAAAAAGGGAGG
AAATATGGAGGAGCTGGGAGGCTTTAAGCAAATACTGAGAATGTATTTGCTCCACTC
GTGACGAAGGTTGGAAGCTGGGACGAGCATAACAAAGGGTTATGAGCGTTCACCAA
GTGCCCAAGGCTGGGAACGGATGACGAAGGGTCATACGCCCGGCCTTGAAGCATTC
GGAGGCTTCCTTCTGACCACCTGTTTCTGGGAGCAAGGACTGTTGTTTCATGATAAG
ACTCCCTTTAGAGTTTCGCCAAAGCTATGTTACGGCTTGGGTGGTGGGAACTGTATT
TTATGCTTGAATGCTTTGATGTTTTATTGAGAAAGGTTACGTGCAAGTCTGCTTTATG
CTCTGCTCCCTGAGACCATGTATCTGCGAAAACTGGATAATAAAATTTGTCAGTCCA
CTAGAGGCTGTCCCTGAGTGTTCCTTTCAGAGTGTGGTTCTCCGA

ERV_26

TGTAGCGAATATGCTGTGCACAGGCCTGTGCTATAATTAACAGGGCTTCTTTGAAGA
ATAAGAATGACCTTCTCATGACCCCCAGGCAAAGGGCTAGGAGCAGTAAAAAGTAC
CTTGTGGAAAAACATCTTGAAAACAAGATGCTGAAATAGTGATGTGACTGATTGAA
AACCATTAGTGACTATTCTCATGACCAGAGCCTTGAAGGAGTTGCTGAGAAAGGTCA
TCACTAGCTGAAGGGCTAAACATGGTCATGGAAGGCCTGAACGTTTGACATTGGGG
ACTGTGCATTGTATATCGTTATCCCCAATCTGAGATTGTAAAAACAGATGTCTTTAG
AGCACGTACAAGGAAGTAGATGTTAGCAATTTAAAAAAAAAAAAAAAGCCTACAAG
GATCAGGATCCTGGCTTTTGCCTGTGAATGGCATCACCACCCCCGCACCACCCCCCC
CCGACCCTCACCTTATTTCTGAGTCTTATTTTTCTTTATTCTTCTGTGGCACTGGTCCC
TCAGGTTGGTTTGTTGTTGGGCTAGTCCGAACATTTGGCACCCAAACAGGGACTTGA
GGGCATGAGTAGCTTTCGAGGTTTTTTTGACAGTGCCAGAAAATATGGGGAATTCCA
GCTTAGAGATAGAAGGACGGTGGAAGTACTTGGTGAGTACACCCCCATGGATTTGT
CAATCAGGGACACAATGGGGCAATCTCCTTCTAAGCATTCTGACTACACTGTGCTTC
TAAAAACCTTACTACATAGCTCTGGAGTAAAAGTGAAAGAAGAGAATTTGCAGGAA
TTATTTCAGGCAATTCATAAGCACTGTTACTGGTTGGACCCAGAAAAAAGGGAATTT
GCTTCTTAATGGTGGAAAGAAGTGATGCGTTGTTTGTGTACAGCCTATCAGTCTGGG
GAACCCATTCCTATAAGTGTGTGGTCTCTTTGTAACCTTATTTACACTACTTTGGCTC
CTTTACAGTCTGAACAGTCTGACTCTTCTGATTCAGAATCTGATACTCCTGCTCCTCC
TGCATCTCAGGAGTCAACCTGTCTGGCCAGCTCATATTTATGAAAATCTGGATAAAG
AGAAATGTAATTCTTTGGAAGGGGAGAGAGACACTCAATTTTTGAGTCCTAAATCCC
ATGTTAACTATGGTACTGCACAACATCCTAAAGCTTTCCCTTTATCACCAGTCTCACA
GCCTTTGGCTCCTTGACCAGAGCCCCCTATAGTTCAGAAACCCCCTCCACTAGATCC
CGATACACCATGGGATGATGCGGTATTTAGAGCCACAGTGACAATACCAAAGACCT
GCTCTGCTGGTGCAGACCAATATCTGCACCTGCTCTCTCATCTTTTCAGCAAGCTGTA
TGAGCAGCAAGGTGAGAAGGCGACCTTGATACTTTGGTTTTGCCTGTTACCGTGATC
CAGGTGCCCCTTAATGCTCAGCTTCCACAAGGCGGGGCACAGTTTGAACATGCTCTC
TCTTTTAAAACTATTAAGGAGAGTAAACAGGCATACACGCAATATGCAGCTACATCT
CCTTACACACAGGTCTTACTCAGGGGTTGGCTCAGTCTGAAGACTCATTCCATATGA

TTGGGAAATGATTGCTAGAACTTGCTTATCCACTTCGGAATTTTTACAGTTTCAAACA
TGGTGGCAAGGTGAAGACTGTCAACAGGCTCAAACAAATGCAGCTGCTAATCCTCC
TCTTAATATTACTGTGGAACAATTAATGGGGTCTGGGCATATCAAGAGGTTCAGAGA
CAACTACAATTCGATGATCAACTTATAGGGCATGCGAAAAGGTCAGCCCCCAGGAC
AATCTCATCCTTCTTCTGTTAATGTAAAACAATCTAATGGAGAACGTTATACTGATTT
TATCACCCAATTAAGACGAAATCTGGTGAGAACTGTTGTTCAAATTGAATTAAAGGA
TATGTTATTATAGATGCTTGCCTATGATAATACAAATTCAGAATGTCAAAGGTTTTA
CAGCCTCATAAAGCACAAGGAAAACCTCACAAGGATTATATTAAGGCTTGTCATGA
TATTGGGTCAGAACCCTATAAGATGCGATTGTTGGCTCAAGCTATTATGAGCTTGAA
ACAACAAGCAAATCAGCAGGTTAAATGTTTTAGTTGCGGTAAGAGACGACATGTGC
AGAAAAATTGCAAGACTAATAAAAATACACCTAATAAGCCTACTCCCACAAATGAA
AAGAAAACTGGATTATGCCCTTGCTGAAATAAAAGCAATCATTAGGCTAATCAGTGT
AGATCTAAATTTCATAAAAATAGATCCCCTTTGTTAGGAAACTGAAAGAAAGGCCCC
ACTCAGGGCCCATCAAACAACATGAGCTCTTAACAAACCTGGAACGCTGTTCCTTTT
GTGTACCCAGTCAGCGAACAGCAACCTCATCCAGCCCTTCCAAACCATACACCTGGG
TTACCGACCTCCTTCCTGCAACATCCAGGAGCACAACATTAGATGTCCCCGCCATCG
ATAATTTACTTCTTATGCCAGCTATGGGAATTTATAAAATCCCTACTGGAATTAAAG
GTCCAATACCTAAAGGAAGTGTGGGGTTATTATTAGGTTATTATTAGTAGTTTAACA
AGTAAGGGAGTTCAAGTCCTAATGGGGGTAATAGATGAAGATTATGAGGGCAAATT
TTATATTATGATGAAAACAGAGTATCCTTATCAAATAATAAAAGGGGATCGTAAAG
CTCAATTGTTACTATTACCTTATATCACTACAAGTAAATCTCATATTAAAAGAACTG
AAGGATTTGAAAGTGCAGGAAAACAGGTATACTGGCAAACTTTTGTGTCTGATTCTA
GACCCACATTATTTGTATATATCAATAATAAGCAATTTTCAGGACTTGTACATACCG
GAGCTGATATTTCTATAATATCTAAAAAGCATTGGCCAGTTTCTTGGTCCTTAACAG
ATGTCCCTATTATGTTAACTGGAATTGGAACTATGCAAACTATTCAAAAAAGTACTA
ATATTTTGACCTGTTCAGGCCCCAAACAACAACCTGCAACTTTACAACCTTTAGTTG
CAGATATCCCTATTAATTTATGGGGACAAGATCTATTGATGCAATGGAGAGCTTATG
TAACAATTCCTACTATATCTTCTCAAGCTAAACAAATTACGGTAAATATGAGATACA
ATCCTGTTCAAAATACATAAGCTTTTTAGTGGAGGCCACTGCCAAACAACAAAAACA
GCACTTAAGTTGACATGGAAATCTGATGAGCCTATTTGGACAGAGCAGTGGCCCCTA
TCACGTGAAAAGTTACAGGCTCTTGAACAGCTAGTAGAGGAACAATTATCTCTTAAT
CATATTGCCTCAACTACTAGTCCTTGGAATACCCCGTCTTTGTTATTAAGAAGAAATC
TCTGAAGTGGAGAATGTTAACCAATTTAAGAAAAACTAATGCTATAATCGAACCTAC
GGGTGCTTTTCAACCAGGCATTCCATCACCTTCTATGATACCATGAAATTGGAAAAT
AAAAATCATTGATCTTAAAGATTGTTTTTATACTATTCTTCTACAAGAATCTGATGCT
CTCCATTTTGCTTTTACTATATTGTCAATTAATAATAAAAAATCAGTGTCTCAATATT
ACTGGAAGGTATTGCCACAAGGAATGCATAATAGCCTTATATTATGTCAATTATACA
TCTCCCAACCACTTAAATTTATTAGAAAACAATTCCTTCATGCTTCTATTATCCACTA
TATGAATGATATTCTTTTAGCTTCTCCTTCTGCTGAGGAACTTCAATGTATTTTCTTA
AATACAGACGTTAAATTAAAAGAATATGGGTTATATATTGCTGTTGATAAATCATAA
AAGCAGGAACCCACATACCTATTCAGGATATATATTGCAAAGAAATATTAAACCAC
AAAAAAATACGAATTTGAACTGACTCCTTGAAATTTTTAAGTGATTTTCAGAAATTA
TTAGGAGATATAAATTGGTTACAACCATCTTTAGGAATTACCAATCATTCCTTAACA

CACATATTTCAAGTCCTACAAGGAGAATCTGATTTAAACAGACCCCGTGTACTAACA
GAACAGGCTAAACAGAAATTGGATCTTGTAAATCAAGCTATACAACAAAGACAACT
TAAACAAGTAGATCTGACTGTACCAATTTCTTTACTTATTCTTCCTACTTTAGATTCC
CCTACAGGAATATTATGACAACTGCAGAGTATCCTTGAATGGGGAATTTTATCCCAC
ACTCCTAAGAAAATAAGGACTACTTACATAATGCTTATTTCCTTTCTTATTTCAAAAA
TGAGATCCAGATGTATACAACTTTCTGGTTATGATCCGTATGAAATTGTTTTTCCTTT
CACTAATGATCAAATCCATAGATTATTTGTTACCAGTATAGATTGTCAAATTGCCATT
GCTGATTTCATAGGTGAGGTTAATAACCATTTTCCAAAGTCACCTTTGATTACTATTG
CTGCTAAACCAAATGGATAATTCCTATTATTACTAAAAAATTTACTCTTGCTAATACC
CCCATGTATTTTACTGACACAAATAAAACTCTCAAAGCCGGTTATATTGGACCCACA
ACAAAGGTTTATCAATCTACCCTAGCCACTGTTCAACAAGATGAATTAGAGGCTATT
GCTACCTTATTACAGGATGTTTCTAAATATTATATATTATATATAGCTTTAAATATTA
TATCAGATTATAAATATGCTGTACAGATAACACAAATTATTGAAACCATATCTTTAC
CAAAGGCTTCTTCTAAATCGTCTATTATTACAATATTATCCCAATTACAAAATATAGT
AAGGCTTCGTTCTGCCCCTTTTTACATTACACATATCAGATCTCATTCCAAATTGCCT
GGACCATTGGCAAAGGGCCATGATTCCATCAACTCTTTGTTAATTGCTTTTCTAATTC
CCTATGAATTTCACCAACTCACACAAATAAATATACATGGCTTGACAAATAAATTTC
AATTATCCAGACAAGCGGCCCGAAATATTGTTTCTGCCTGCCCTACATATACTCTTA
CTCACCACATTCCTACAACTACAGGGGTTAATCCCAGAGGCCTTTATCCCAGTGATA
TCTGGCAGACTGGTGTTACTCATTTCCTTCTTTTGGACATTTGGGAGCTGTTCATGTT
TCTGTGGACTTTCTCCCTTCGCTTACCTAAATTACCTTATATTCCTTTACATTCAGAAT
TGTGGAAAATATCAGCTGCCTTCCAACCCATAACAGCATGGAAGGGGAAATTTAATT
TGATAGTATCTGGTCATTCCTTACATTTTGATAAAAATTCAACCCATTGGATTAAAGC
ATGTGTTCATCTACCTTTTGTATTTTTGATAGGACCTACTTTTTATAATGCCTCTTCTG
GGTTACTTACTTGTCAAAATTGTTCCTTTTACACTTGTATCAATTCTCTCCAATTTCAA
AATGACTATTCTCTATATATTATAAAAATCAGATCTGGAGTATGGCTTCCTGTAAAA
ATGAAAAAACCATGGCAGGATAGCCCCACAGTCTATATTGTAGAAGAAATCCTTAA
GAAAGTGTTCAAACACACTGAACGGTTTATTGGACTGTTAAGTGCTGCCATTTTAGG
AATTATTGCTATAGCTACTACAGCAGCTGTAGCAGGACGGCATTACTTCAGAGTGTA
CAGACTGTGCACTTTGTGCAAGAATGGCATAAAGACTCTGATGTTCTTTAGTCTACC
CAGATGGAAAATTAGCTGCGTAAATGGCTGATTTACAACAGGTTGTTATACTACTAG
GTGATCAAGTAAATAGTCCGCAAAAACAGATTTTTCTTAAGTGTGATTGGAACATAA
CTTCTTTTCGTGTCACTCCTCACAAATATAATGAATCTTCATTTCATTGAGACAAAGT
TAAACAGCACCTCTTAAATCAAGGCAATATATCTCTGGATATTAATAATTTACAACA
AGAGATTATGGAAATTTTCTCTCAGAAACTACACCTTTTGCAAGGATCTAATCTGCT
GGAAGCAGCAGCAGATGGGATTTCTCAATTAAATCCCGTTCAACACTTGACAACTAT
CAGAGGATCAATGGCTGGTTTTATGTTAATATTTATATGTCTTTTTTTCTGTTTCTACA
TAGTCTGGCAGTGAACCAAGAAAGCCAATGGACAGCAACAGCAATTAGTGACTATC
GCTTTGGCTTTTATGATCAACTATAATCATCAAAAATAAGCTTCTAAAAATAAAAAG
GGGGTGTGTAGGGAATATGCTATGCACAGGCCTGTACTATAATTAACAGGGCTTCTT
TGAAGAATAAGAATGACTTTCTCATGGCCCCCAGGCGAAGGGCTGGGAGCAGTAAA
AAGTACATCGTGGAAAAGCATCTTGAAAACAAGATGC

ERV_27

TCAGGAAGCAACAGTTAGAACTGAACATGGAACAAAAGACTGGTTCCAAATAGCAA
AAGCAGTATTTCATGGCTGTGTATTGTCACCTGCTTATTTAACTTATATGCAGAATAC
ATCATGAGAAACACTGGGCTGGAAGAAGCACAAGCTGGAATCAAGATTGCCGGGAG
AAATATCAGTAACCTCAGATGTGCAGATGACACCACCCATATGGCAGAAACTGAAG
AGGAACTAAAAAGCCTCTTCATGAAAGTGAAAAAGGAGAGTGAAAAAATTGGCTAA
AGCTCAACATTCAGAAAACGAAGATCATGGCATCTGGTCCCATCACTTCATGGGAA
GTAGATGGGGAAACAGTGGAAACAGTGTCAGACTTTATTTTTTGGGGCTCCAAAATC
ACTGCAGATGGTGATTGCAGCCATGAAATTAAAAGACGCTTACTCCTTGAAAGGAA
TGTTATGACCAACCTAAATAGCATATTGAAAAGCAGAGACATTCCTTAGCCAGCAA
ACGTATGTCTAATCAAGGCTATGGTTTTTCCAGTGGTCATGTATGGATGTGAGATTT
GGACTGTGAAGAAAGCTGAGTGTGGAAGAATTGATGCTTTTGAACTGTGGTGTTGG
AGAAGACTCTTGCAAGTCTCTTGGGCTGCAAGTAGATCCAACCAGTCCATTCTAAAA
GAGATCAGTCCTGGGGGTTCTTTGGAAGGAGTGGTGCTAAAGCTGAAACTCTCATAC
TTTGGCCACCTCATGCAAAGTTGCCATATTGGAAAAGACTCTGATTCTGGGAGTGAC
TGGGGCAAGAGGAAAAGGGAGCAACAGAGGATGAAATAGCTGGATGCATCACTGA
CTCGATGGTCGTGAGTTTGAGTGAACTCCGAGAGTTCGTGATGGATAGGGAGTCCTG
GCATGCTTCAATTCATGGGGTCACAAAGAGTCAGACACGACTGAGCAACTGAACTG
AACTGAACAGAAGCAGAAGACATTAGGAAGAGGTGGAACGTATACACAGAAAATC
TGTACAAAAAAGAGCTTCAAGACTCAGATAATCACGATAGTGTCTTCACTTGTGGGG
AGCCTCATTAGGCATTACTGACAAAATGGAGGCATGACCTCAACTTCTTCTCTTTGT
CCCGCAGGCACAGACCCAGGATGAAGGATTTTGGCCTTGTGATTCTGACTTGTTTTT
TCCTTTCCTCAGCTTAGTTGACTGAAAAGAATATTAATGTGCTTATTGTTCTTGAGAG
GAGCATGAGAAGGCACATCCTTCTGCTGTTGTGCTCAGAGAATTATTCATAATCATT
GACATTTGTTCAAGGATCTTTACAAAAAAATTGTCCCAGGATGAGCACGTAGGCCTC
AGCTTGAGGCCATGGGAAGGATTGTTATCTGAAACTTATTTGTAAGGGAAATGTTTA
CGGCAAAAGAGTTTACTGAGTTTAAGGCTTAGGAATAATTAAAAATAGTTAGAAGC
TAAAGATTTAAGTAACATTGTAATGTTAGCATATTTTGCTATAGCTTATAGAGATTA
GGAATTTTAGAGATATTAATAGCTAGAAGCCTTTTTAAGAGGTAGTGAGATCAGGAT
ACTAGGGGCAAACAGATTTAGAAAGATAAGAAATACATTGAGGTTTGTGTTATGCA
GCCCAGATATGAGCATGAGTTACAATGTAATCACAAGTTAAAGTAAACACAATTCT
GAGAGTACTGCTGAAGCAGAAACTCTGCTTGAAGGACAACAGTGATTTATGGAGAC
AATAAATCTGGGTGAGGGGAAATTGAAAATGTCAAACCTCTGACCTAATGCTTTTGT
AAAAGTATAAAAGAGAATCTAAAGCTTGAAATAAGCATACAGTCCAAGAAAACTGA
GAGGCTGCATCATTACTCACCGACACCTCCCATCCTTTCAGGCTGATTCCCTGGCTTC
TGGACTCTGGCAGGTGGTGCCTGAATAGGAATATCAAACATTTGAAGGGTAAGTAA
CTTCGTGATTGTAGTGCAGGGTGGTTAGGATTGTACCTGTGGGTGGCCGGCACCCCT
CTGCAGTTTAGGCAGGGTGAGGAGGGTACAGAAGGAATAAAGAATATTCTCTAGGA
AAGTAGGTCTCTCTGCATCGAAGAATAGACAAATGTTTATTGAAATATTACTTCACA
TGTTAGCCCATAGAGGTGTTAAAGTAATCAAGGGAAGACTAAGTTTCTAATCTTTGT
CCAAGAGCAATGCCCATGGTTTCCTGAGGAAGGTACCGTAAATCTAGAAATGGACT
AAGGTTGGAGATCAGTTACATTTGTTTCATACTGTATACGGCCCAGAAAATGTTCCT
GTAGATGCTTTTGCTCTGTGGATTATGATCAGGGATGTCCTGGATCCTCGGCATGAG
GCTGTTAAACAGCCTATGGGAGAGGCTGGAGCAGTGAATGGTGGGTCTCCACCTTCT

GTGCAGATCATGTTTTCTGCCACTGACGAAGAAAAGGAGGGAAACTGTTCTCAACCT
CCTGAGGAAGGAGAGGGCTTGCAGGACACTGCGTCCAGGGGCTCTAGTGAGCCTCC
TCCCCCTCTATGCAAACCTTTGAAAGTTTATCCACCACTTTCTGATTTAAGGCGATTG
CCACCACCTCCGTTTGTGCCTCCCAGGGCCCAGGAGTTCCCCACTTTGCCCCCAAAG
CCTCCCAGAGCATTGCCTGAGGCTGACGAGGATGAAGACTTTTCTGAAACAAAAGA
GCTTTTGAGGCAGTCTCCTGTACAATATGCAGAGTGTGATCCTTGGAGTAAACCGCC
TAAGGGGGGGGGGGGCTCACCCAGCCTAGAAGGGAGGCAGAACAACAGGGGGAC
TTGGAATTTTCTATGCCCTTTCCAGTAGCTTTTACCAAAACCGAAGGGGATGATAAG
GAAAAAGGAAAACGGGAATCAATCCCATATAAGCTACTGAAAGACCTTAAACAAGC
CTGTCATGATTATGGGTCTACCTCCCCTTACACACTTACTCTGCTAGATGCCTTGGCT
GGGAGATGGATGGCACCATGTAATTGGAGAATGGTTTCAAAGGCTTGCCTTCCTGGA
GGAGAGCACCTCCTGTGGTTGACAGAGTATGATCAGTTGGCTAGACTACAGTCACCA
GAAAACAAAACCTCTAATGATGCACAGCTCTGGGCAGTGGGATCTGCAGCATTAAA
AGGAGATGGAGAATACCAATCTAACATAACCCAAGCAAGGTTATCTAAAGAAGCAC
TTAATCAGATTATGGCCATAGATGTCTTGACATGGAAATCTCTGCCCCCTTCAGATG
GAAAATTATCCACTCTGAATAATATCAAACAGGGACCCGATGAGAAATATGAAGAA
TTTGTGGCCAGATTGAAAACTGCTGTAGAAAGAACTATTAAGAGTACTGACCCAGC
AGAGATAGTTCTTAAACAATTGGCTTATGAGAATGCTAATTCTACCTGTCAGGCTTT
GCTTAGACCTTTCAGAAGTAAAGAAAGTCTTTGGACTTACATACAGACCTGCCAGGA
AGTAGGAACGTCCTTTATGCAGGGGGTTGCCTTAGCCTCAGCTTTAAGGGAAGAAAC
AGCTGCCCGAGTTATTCAGGGAATGAGAAAAAAGATTAATCCTAATGGAAATGATT
TTACAAATAAAAGGTTCTTCTCTTGTGGCCAAATGGGGCATTTTTGCCTGTAATATCC
TGCCAAGCAGGGACAACAAACTGTGCCAACACAGACTAAGACCAATCCCCCAAAGG
CACATTGCCCCCCATGCCACAAAAGGGTATCATTGGGCAAAAGACTGTAGATCTAA
ATTCCATAAGGATGGGACTACGCTCACCCTCCAAGTACAGAGAGTAATTTTTCCCAG
TTTCAAGGAAACGGGCAGCAGGGGCAGCCCTGACCCTGGACAACAATAGGGGCAGC
CACGTTGAATTCCTTTATTCCCTTTGTCCTGTCTCAGAAATCCCCAGAGCAACCCCAG
GTAACGCAGGACTGGACCTCAGTTCCACCACCACAACAATATTAATCCCTGATGTAC
TGGTTACTCCGATTCCCACAGGAGTGGCTGGCCCCTTACCTGAGGGCGTTGTAGGAC
TTGCTCTGGGGTGTAGCTTGCTCTCCCTTCAAGGAATTTTGGTGGTGCCTGCTGTAGC
AGATTCTGATTATACTGGGGAAATTAAAGTTTTGATCTCTCCACCTATCAAAACTGT
GCAAATTAATAAAGGTCAAATAATAGCACAGCTTTTGCTTTTACCTTATCATCAAAC
AGGAAGAACCTTGGCTTCTTAAGCTAGGGGCCCCAGAGTATTTGGATCTAGTGATCT
AGCCTTTTGGGTGCAGGAAATTACAGCTTCAAGGCCTTTGAAAGATCTTTTAATTCA
AGGAAATAAAATGTTAGGGCTGTTAGATACTGGAACAGATATCTCTTGCATTGCTGG
AAAAGATTGGCCCTCATCCTGGCCAACATGCTTGACCAGTGCCGACTTGGTAGGAAT
AGGGTCAGTGCCCTAGGTTGCTAAGAGCTCACAAATTTTGACATGGTCAGATGAGA
AGGGCACAAGGCACCTTTTGTCCATATGTGATTACTTCACAACCTTTTTCTTTATGAG
GGAGGGATATATTATCTCAGATGGGAATGCTTTTATATAGCCCAGATGAAAAGGTTA
CTAATCAAATGCTGCAAATGAGGTATAATTCTGATAAAGGACTTGGTAAAGATCAG
CAGGGAATTGTTTCTCCATTAGAAGCAGTTCCTAACAAGAATAGAGAAGGTCTGGG
ATACTCAAATTTATCCTAAAGGCTGTTGCTCTTGCTGCCAACCCTATTACCTGGAAAT
CTGATGATTCGGTATGCGTGGAGCAATGGCCATTAACAGCTGAAAAGCTGCAGGCA

GCTGAGGATTTAGTTATGGAACAACTGGCAGGCAGGCATATAGAGCCTTCTAATAG
CCCCTGGAATATCTGTATTTTTGTTATTACGAAGAAATCTGGAAAATGGAGATTGTT
ACAAGATTTGAGAGCTATGAATGCAACTATGGAAGATATGGGGGCCCTCCAGCCAG
GCCTCCCTTCCCCAGTGGCTGTGCCCTTTCAATATAATGTGATAGTTATAGATCTACA
GGATTGTTTCGTTACCATCCTCCTGGCTGATCAGGATTGTAAACGGTGCTTTCAGTCT
CCCTTCAGCTAATTTTAAACAGCCCTATAGAAGGTTTCAATGGAAGGTTTTGCCTCA
GGGAATGAAAAATAGCCATACCTTGTGTCAGAATTTGTCAACCAAACTGTGCAAAA
TGTTAGAGGAAATTATAAAGATCTGTATTTGACACATTATATGGATGATATTTTGGC
TGCTCATAGGACAGAGCCTTGTTGCAACAAATATTATCTGAATTGATTGAGGCCTTG
GAAAACTGGGGTTTAAAGATAGTTCCAGATAAGATACGAGCAAATCCTCCTTTTTCT
TATGTAGGGAGGGTATTAAATACCCATACTGTGAGTCATGCTCCTTTGCAGCTGCAG
AGAAGTCCTTTACTAACTTTAAATGATTTCCAGAAACTATTAGGAGATATTGATTAG
ATATGCCCACATTTAAGACTGACTACTGCAGATTTAAAGCCTCTGTTTGATTGCTTAA
AAGGCGATCCTAATCCCAGTTCTAAGAGAGAATTGACTAGTGAGGCAGAGTCAGCT
CTTGTTAGAGTGGATGAGGCTTTAAGTGATCAGTTAATTAGGATTAATATTATCAGA
GGATGGGATTTAATTATTCTCACCACAGAGCATACACCTACAGGATGCTTGTGTCAA
GAAGACCCATTGGAATGGCTCCATGTACAAGTGGCCCTAAGAAAATAGTTTTGTATG
ATCCCAGCTTGGTGGCACAATTAATTATAAAACGTAGCAAAAGAGGTGTGGAACTTT
TGGGGAAAAAAATAGCAAATATTGTGATTCCATTCAATAAGGATCAACTGCAATTTC
TTTTACAAAATAGTGATGATTGGCAAGTTGCCCTGATAGATTCCAGAGATCAAATTT
TATTTCATTTGCCCTCAAACCCCCTTTTGCATTTTTTGAAAACACATCCAGTGATTTTT
CTGAAGAAATTTTCTATTCAACCTTTGGAAAGGACAATTTTACTGTTCACAGATGGT
TCCTCTAATGGTAAAGCAGTCACAATTATTATTGGAAAATCCCATGTTCAAGTAACT
GAAGAGACATCTGCCCAGAGGGCTGAGTCAAGAGCAGTTATTTGGGGTTTTCAACA
TGTAAGAGACTGTACCTTCAATCTTTTGACTGCCTCCCGATATATTGTAGAGTTATTT
CCTCATATTGAGACTGCTAATATTTTGGAAAATAAAGCTATAATCTTCTCCTTGTTAT
CTGATTTACAGAAGGAGATTAAACATAAAGATAAGAAATATTTTGTGGGACATAGT
GGAGCCCATTCCAGCTTGCCCGGCCCTTTGCATGAAGGAAATGCTTTGGCAGACGCC
TTAACTAAAGCAATTGCTTTAAACTTACATAAAAAGATTGACAAGGCCAAAAGTTCT
CTCAAAATTCACCATCAAAATGCAGCTGGTTTAAGGTATGAATTTCATATCTCCAAG
GAAGCAGCTAGACAAATAGTTAAATTGGGTTCAAATTGCCCAATATTTAATACATCT
CTACCATTAAGAGTAAATCCCCGAGGCCTTAGGCCTAATGCTCTCTGGCAAATGGGC
ATAACTCATATCCCAGCCTTTGGAAAACTGTCCTTTCTGCATGTTACCATGGATACCT
TTTCTCATGTTATTATAGCCTCTGCTAGATCAGGTGAAGGAGCAAGAGATGTAATTC
AGCACTTGTTTCAACAGCTTTTCCCAAATAGGAGTCCCCAAACAGATAAAAACAGAT
AATGCCCCAGCTTATACTTCTGCTGTTTTTAAGAGATTTTTGTCAACAATTTTCCATA
GTACATTCAGCAGGAATACCTTATAATCCCCGAGGCCAAGCCATAGTGGAAAGGGC
ACACCAGACTTTAAAAAATCAGATCGATAAATTAACACAGGGAGAATTTAAGTATT
CCTCTCCACGTCATGTTCTACACCATGGTTTATTTGTAATTAATCATTCAAATATGAA
CACACAGGGGCAGACTGCAATGATGAGACACTGGATTCCTGAAGGGGCTATGACTC
AGGTTCTGGTTAAGTGGAAAGACATCCTTATCGGAGAGAGGAAAGGGTCAGATATG
CTGTTAAACTGCAGGAGAGGGTATGCTTGTGTGTTTCCACAGGATTCAACTTCTCCT
GTTTGGATTTCTGACAGACTGATTCGACATGTCCAGTCCCATGAGACCTCCAGGATC

ACAAAGACCCACAAGGTCACTGAAATCTCTGGAGTCCTGGAGTCAGGGACTGAGGA
AAGATGGGGAGACCAGAAGTTCGTCGAGTCCAGACCTGAGGGAGCCAAGGAAAAG
TGAAAGGGCGAGTGAATCTTCACCTGAGGCTTCACTGCTAGTACATCACTTCAGGCA
CCTGGAGCTTCAAGAAGAGACATGCTGATCAAGTCCAACCGTATTGTGCTCCATCTC
GTCACTGTCATTGTACTCAAACAACTACCTCCAGCACGGTACCAACTTGGGGCCTAA
AACATCTCACTCAGCAGGCTGAAGAATTAACAAAAAGGGGGAGACATGAGGCCACT
CCCATGGTAATGTTTGTGCTATGGAGGTGATGGAATACCAGTTGACCTGTTTCAAAT
CCTGAAAGATGATGCTGTGAAAGTGATGCACTCAAAATGCCAGGAAATTTGGAAAA
CTCAGCAGTGGCCACAGGACTGGAAAAGGTCAGTTTTCATTCCAATTCCAAAGAAA
GACAATGCAAATGAATGCTCAAACTACCGCACAATTGCACTCATCTCACATGCTAGT
AAAGTAATGCTCAAAATTCTCCAAGTCAGGCTTCAGCAATAGGTGACCATGAACCCC
CTGATGTTCAAGCTGGTTTTAGAAAAGGCAGAGGAACCAGAGGTCAAATTGCCAAC
ATCCGCTAGATCATCAAAAAAGCAAGAGTGTTTCAGAAAAACATCTATTTCTGCTTC
ATTAACTACGCCAAAGCCTTTGACTGTGTGGATCACAAGAAACTGTGGATAATTCTG
AAAGAGATGGGAATACCAGACCACCTGACCTGCCTCTTGAGAAACCTCTATGCAGG
TCAGGAAGCAACAGTTAGAACTGGACATAGAACAACAGACTGCTTCCAAATAGGAA
AAGGAATACGTCAAAGCTGTATATTGTCACCCTGCTTATGTAACTTATATGCAGAGT
ACATCATGAGAAACATTGGGCAGGAAGAAGCACAAGCTGGAATCAAGATTGCCGGG
AGAAATGCCAATCACCTCAGATATGCAGATGACACCACCCTTATGGCAGAAAGTGA
AGAGGAGCTAAAAAGCCTCTTGATGAAAGTGAAAGAGGAGAG

ERV_28

TGGACTGCAAGGAGATCCAACCAGTCCATTCTGAAGGAGATCAACCCTGGGATTTCT
TTGGAAGGAATGATGCTAAAGCTTAAACTCCAGTAATTTGGCCACCTGATGCGAAG
AGTTGACTCATTGGAAAAGACTCTGATGCTGGGAGGGATTGGGGGCAGGAGGAGAA
GGGGACAACAGAGGATGAGATGGCTGGATTGCAACACTGACTCGGTGGACATGAGT
CTGAGTGAACTCCGGGAGTTGGTGACAGACAGGGAGGCCTGGCATACTGCGATTTG
TGGGGTCGCAAAGAGTCAGACATGACTGAGCAACTGAACTAAACTGAACTGAACGC
CCAATGGCAAATAATACCTCAGTAGGAAGGCATTAATTTTTCTCACATGCAACAAAT
GAAAAAAGCTGTAACTATGTATGGCTGTCATTCACCTTTTACTAAGAAACTTTTAAA
CACTATGGCATCTTCTCCTGGAAATTTTGCAACTTATGATTGGCGACTTTTGATAAGC
GCTCATCTTAAACCGGGAGAATATCTTCAGTGGACCATGTGGCTTCAGGGTATGGCC
CAAGATCGTGCCAATTCTAATGCCCGAGCTGGTACTGCCCAAAAGCAAATTACTTTT
GAAATGTTAACTGGTACAGGGCAATGTAATGCTATAGAAACTCAAATACAAAGCCC
TCCTTGGTGGAAAACTATCTTGACTGTAATAAATGTAGTCATCTGGTTCTTGCTGTTT
GCATTTGTATCTGTAACTATATAATGTACTTTGTATCTAAGTAACTTAGGCATTTACA
TTACAAGTGATTGTTCATGCTCCTGCAGTTTCCGCTACCTCCTCTAGCTATTACTCAG
GGCCACTGGATCAGAGACCCTCAATAGTAGGATGAGGAGAATATGTTGCCTCAACA
ATTTAGGGTCTATGCCCCTTCCCGGCAGGAAGAAGTTACAGAACGATTTCTCCACCC
CTTTCCCTGCCAACACTATTCTCCTAAAAGCAGGAGGAAATGAAAGAGTTAATGCTT
AGGCAGGCAGTCCAAGGCCCTGTAAGGAGGAGACAAACATTCTCACTCATGCTTTC
CTTAAGCCTTGTGCAGCAACATAGCTTGCCAGAGAACTGACCTTTCTTTAGGTCCAG
AACTAATGATTAGACAGCCGAATGTCTTACTCATGGAAATGCTTTTCTTAGGCTCTA

TGGTAATGATTATAATTGTAACAAATCTTGCCTGGGAACCTATATCTCAAGACTTGT
ACCCCTGCTTACACAGCAACAGTATATCTTGCATAGAGACATTGGCCGGAACCTGTT
CTCCCTGGCTAATCTTGTACCAAAGTTATCTCAGGATGTATGTCTTGGGAAAGGGCC
TAATAGACCTCTTACAACGTTGAGGTATTCTTTTTATCTGTTCTCAGCAGCTAGTTGA
GAAGTATAGAAGGTCCCACTTAAACTAGTGAAGTGGATACTCTTTCTGCCCCCACAA
ACTTTCTGCCTTTTACTTGGCCGATCTAGTTTGACTTCTAAAGGAATTAGTGTTCACC
CTGGAATAATTGATTCAGATGATGAGGGAGAAATTCAAATTATGATGTCATCTCAGA
TACTATGGCGTTCAAAAAGGGGGCAAGATGGCCCAACTACTCCCTTCACCTTACATG
CCTATTAGCTCCTCTAATGGCGTCTGCACAGGTGGATTTGGCGGTTCAGATCAAAAA
CAGTCCTTATGGACGTCCATAGTATCTGAATGTGCACGACCGAATATAAATATCAAA
ATTAATGGCAAAAGATTTTCTGGTCTTCTTGATACTGGATCTGATATTACCATTATTT
CCAAATATTTATGGCCCAAATCTTGGCCTATACAGAGAATTTCTTGCCAAACTGCAG
CAGTTTCTCAAACCAAAGCACAAGAGGTTTATCAAAGTGTCCAAATATATCCATGTG
AGGGACCAAAAGGCCAGCCTGCAACATTACAACGTTATGTGATAGGTACACCCCTC
AATTTAATATGAAGAGATTTACTTATGCAACGGCAAACTCAAATATATATTCCACAT
TTTTCCTAGGGGCCACTGCTCACCTAACAATCAATGCAATTATTAAAATTACTTGGA
AAAATGACGAGCCTATTTGGACAGAGCAATGGCCCCTCACAAAAGATAAATTGGAG
GCTACTAAAGAACTTATCAACACAATGGAAATCACAGCATATTGAGGAATCTTATTC
CCCTTGGAATTTTCCCATTTTTGTAATAAATCTGGCAAATGGCATCTCTTAACAGACC
TTAGAAAAATTAAAACATCTATGAAAGTTATGGGTGCATTGCAACCAGGAATTCCAT
CACCTGCCACTATCCCTCAAAACTGGCATATTATTATTATAGGTTTACAAGGTTGCTT
TTTTACTATAGTTTTACACCCTCTAGACCCAGACAGATTTGCTTTCTTTCTTCCTTATT
CTAATCATGCTGCTGCTACTGCTGCTAAGTCACTTCAGTTGTGTCCGACTCTGTGCGA
CCCCATAGAAGGCAGCCCACCAGGCTCCTCTGTCCCTGGGGTTCTCCAGGCAAGAAC
ACTGGAGTGTGTTGCCATTTCGTTCTCCAATGCATGAAAGTGAAAAGTGACAGGGAA
GTCGCTCAGTCGTGTCTGACCCTTAGCATGGACTGCAGCCTACCAGGCTCCTCCGTC
CATGGGATTTTCCAGGAAAGAGTCCTGGAATGGGGTGCCACTGCCTTCTCCGATCCT
AATCATATCGGGCCTAATAAATGATATCAGTGGACTGTATTGCCTCAAGGAATGATG
AATTCTCCCACCGTGTGTCAATATTATGTAGCCAAAGCCCTTGAACCTGTGAGAAAA
CAATTTCCTAACTTTCTTGTTATTCATTATGTGGATGATACATTGTCTTCAGCTCCCTG
TGTTTTAGAAACTGAACAAATGTTTGACATAGCTCAACAGTGCTTGGAAGACTCTGG
ATTAAACATTGCTCCTGAAAATATTCAAACCTCTACTCCTTACCATTACTTAGGCTCT
GTTGTTAATAGACAACGTATTACTCCCCAACTAACAGATTCATGTTAATAAATTATC
AACCTTGAATGATTTTCAGAAACTTTTAGGTTATATAAACTGGATTAGACCCACTTT
AGGCATTGCAAATGATCAACTGACTAATTTATTGAATACTTTAAAAGGAGATCCCTA
TTTAAATAGCCCTTGTTTGGTACAAAATAAATTCCAAAAACAGTTTCTTACTCGTATT
AGACTTGATTTACCTCTCGAGTTGTTTATACTCCCTTCTCTCTATTCCCCGACGGGCC
TTCTTGCCCAACAAGAACACCTGATAGAATGGATCTCTACCCATTTTAGAGGGACGA
GGTCACTTACGCCCTATATTGATTTGATCACTCTAATAATTATCAATGGGAGAAATA
GAACTAAGACACTAATTAGTTCGATCCTCATAGCATTGTAATTAATAAACCTCAATT
TGAGAATGCACCACAAACGTCTACCAATTTCCAAATAGCCTTTCTGGAATATTTTAA
AGATATTTCATTTCATAATCCTTCTAACAAACTCTGAAATTTCTTGAAAAATACTGAA
TTTATTATCTCTTGCATTATCACATTACAACCTATACCTCGAGCTGATGTTTTCTATAT

AGATGGGACTAAAAACACCAAAGCCTCTTTCTGGTCTGTCGAAGAATACAAAGTCTT
TTATACTAAATTTCATTCTGCTCAACAAAACGAATTATATACTCATTCAAGTTATTCA
CCTACACCCTTACCTTATTAATATAGTCTCTGACTCTTTATATTCAGTTTTTATATTAG
GAAATATAGAAACCTCTATTATAAATTCCAACCAACCTATTATTCAACAAGTTTTTCT
CGAACTACAGTCTCTTATTAGGGACTGCACTTCCCCCATTTACATTACCCATATTCGA
ACACATTCCTGCCTTCCTGGCCCTATGACTCATGGTAATGAACAAGCTGATAAACTT
GTTTCTTTTGCTACTCCCGAGGAACAACATGCTCTATTGTACAATAATGCTGGCTCCT
TAAACCAAATTTAGAAAATTCCATACTGGTGGACTGCAAGGAGATCCAATCAGTCC
ATTCTGAAGGGGATCAGCCCTGGGATTTCTTTGGAAGGAATGATGCTAAAGCTGAA
ACTCCAGTACTTTGGCCACCTCATGTGAAGAGTTGACTCATTGGAAAAGACTGATGC
TGGGAGGGATTGGGGGCAGGAGGAGAAGGGGACGACAGAGGATGAGATGGCTGGA
TGGCATCACTGACTCGAAGGGCGTGAGTCTGAGTGAACTCCGGGAGCTGGTGATGG
ACATGGAGGCCTGGCGTGCTGCGACTCATGGGGTCGCAAAGAGTCGGACACGACTG
AGCTACTGAACTGAACTGAACT

Data Set 2

Data Set 2. 31ERV-RIP insertion polymorphisms were predicted through a comparison of genomic coordinates using 28 ERV groups.

>NC_056054.1:102854042-102860826#SHEEP_RIP_01(-)

GTGGAGGAGCTGGAAGGCTTTGAGCAAATACTGAAAATGTATTTGCTCCACTCATGACGAA
GGTTGGAAGCTGGGACGAGCATAACAAAGGGTTATGTGCGTTCACCGAGTGCCCAAGGCTG
GGAACGGATGACAAAGGGTCAATACGCCCGGCCTGGAGGAGTTCAAAGGCTTCCTTCTGAC
CACCTGTTTCTGAGAGCAAGGACTATTTTTTCATGATAAGACTCCCTTTAGAGTTTCGTCAAA
GCTATGTTATGGCTTGGGCGGTAGGAATTGTATTTTATCCTTGAATGTTTTAATGTTTATCTG
GAATGGCTACATGCAAGTCAGCCTTATGCTCTATTCCCTGAAAATTATAAAACTATACAATT
GGATAATAAACTTTGTCAGTCCACTAGAGGCTGTCCCCAAGTGTCCTTTTCAGAGTGCGGTT
CTTGGAGCCTTATGGATGGCGCCCAACGTGGGGCTCGAAGCAGCAGACTGATTTTGAAGAA
GGGCCGCACTCCTGCAGAAGCGAGGTAAGCAGAATGGGACATCAGACAAGTAAAATTCCTT
TAGTTCTTCTCATGCATCATTTCTTGAAACAATATGAGGTTAATCTGCCTGAAGAGCAGTTAA
CTAGCTGTTACTAGACAGTGGTTGAATATAATCCATGGTTCCCTTAGGAAGGAACCTTAGAT
TTACAAATTTAGACCAGAGTGAAAAACAATGTTCTAAAAGCTTATAGACAGGGAATAAAAA
TTCCCCCACAATGGCCTCTTTTACGGGCCATTATGGAATGATTAGATGGCTCTGGAGGAAAT
TTAAAGGTTGAAACTGTTCGATCCCTGCATGAATGTGAATTAGAAGAGAAAGATCTATCAGA
GGTTTTAAATCAGAAAAATGTCACGCTAGAGCAGATTACGGACAAACAAGAGTTACAGGTG
CTAAAAGCAATTAAACAATCAACTTTGCCAGAGGCCCCTGATCCGCCTTTACCTTGCTTTTTG
CGAGCTCATATTAACAAGCCTCTGACACTGGCCTTTCCGCCCGCTGCGATGCTGTCCTCCCCT
ATTTCCTAGCATTCCTCCTCGGGACATGCCTCTTTGTGCGTGGGCTTTTCCGGTCCAATTTAA
TAATTCTCAACTTGGACATAATCAATGGCAGTCACCTGATTTTGGTTTGCTCACGCAATTCAA
TAAGGCATGTACGTTATATGGACCTACCTCACCCTACTGTATGGAATTTCTTAGGGGCCAGG
TGGATCAGTGGCTTCATGCAGATTTTTTTACAGTTGCTAAAATGGTTATGACTCCACAACAGC
TACTACAATGGCAAATGTGGGTCACGGATGAAGCCACATTAATCTTGCAAGAACAGCAAGC
AGGGAAAACCCTACTGGACTAAATTTTAAAATTCTCACCGGCACCAGAGCTATGGCTAAAAC
TGATGCACAATTACAGTTTGTGCAACCCCCCATGTTATACTAGATTAAGGAAGTAGCTATCA
GAGCGTGGGCTAAAATGACAGTTCCACCTCTAATGGATCTTTTGTAAAAATATTGCAGGGAC
CAACTAAAAAATATACTCAATTTATTGATAAATTGAAGGAGGCCATTGATCAGTCTTACGGA
TGCATCTTTGCGAGAAACCATTTTGAAACAATTAGCCTTTGATAATGCTAATGAAGATTGTC
AGGCTATTATCAGACCCCTTAGGGGGCAAAGAGAAGTTCTGAAATACTTGAAAGCCTACAG
GAATGTGGGGACAATTCAACATAAAGCTAAAATAGCCGCTTTAGAAACCTTAAATGTTTCCC
AGAAGTCTGAAGTTAAATGTTTTAACTGCGGCAAGCCGGGACACACGCGGAAGCAATGTCG
CTTGCCTCGGCAAACAGGTCTTTCTCCTGACAAAGGAGGGGCTATTAAAACTAAGCCCCCCG
GGCTCTGCCCAGGATGTAAAAAGGGGAATCACTGGCTGACTGAATGCCACTCTAAATTTGAT
AAACAAGGTAATGCTTTACCCATTCAGACACCTCCTTCGGGAAACTAGAACAGGGGCTCTCC
TCTAGCCCCATTAAACAAGGAGGACAACCAAATATTACTAAATTAACAGCGGCTACCAGAC
ATAACACATGTATAGACATTCCTACTCCCTGAGATATAAAATTATTAATGGTAAATAATCCT
ATGAAAATCTTAACTGGATATTTTGGCCCTATACCAAAAAATACTATAGACCTCCTATTGGA
ACAAAACAACACCATGCGCAAGATAATTGTACATACTGAGATCATTGATAAAGATTACACA
GGTAAAATTGCAATAATGTTACATGTGACTCACAACTTGTATTTACAAAAGGGTGACAGATT
TGCTCAGTTATTGCTATTACCTTATGTGCCCCCACTTAATAGAAAAGCAGACGCCAGAACAG
GTGGCTTTGGGAGTACGAACGTTACTGCAGCCCTTTCTACTGTTATAAAAGAAATCAATAGG
CCCATGTTAAAATTAAAAATCAGAGAAAGAACTTTTGAAAGAATGTTAGACACTGGGGCAA

ATGTTTCCATCATAAGAACAAAAGAATGGCCTTCGGACTGGCCTACAATTTTAACCTCACAC
CAGTTGGTGGGAATAAGCACTGCAGATGCAGCTCAAACTTATGTTAGTTCATCTTACTTACA
AGCCCTGGGCCCTAATCAATTAGTCGCTTACATTAAACCGTACATTGCCCCATTACCATTAA
ATTTGTGGGGAAGAGACTTTCTACAACAAGCTCAAGCGACTATACAATTAAATGAAACTTTT
TCTTAGGGGTCACTGAAATAAAGCCACTAAAGTTAAAATAAAAGTCTAATAAACCTATCTGG
ACAGCTCAATGGCCCCTATCAAAACAAACCAAAAAAAAACTGTCTGCTTTGCATACTTTGGTG
GCTGAACTACTACAACAAAATAAAATAAAAACTACTCAATCACCATGAAATTCACCAGCTTT
TGTCATTAAAAAAAAATCAGGTGAATAGAAAATGCTAACAGATTTTAAAAACATTAACACT
ATAATGATTCCTATGGGAGCATTACTACCAGGACTCCCAAGCCCTGCTATGGTCCCTAAGGA
CTGGGCTATTATGATTTTTGATTTACAAGACTGCTTTTTCACTATACCTTTACATCCAGAGGA
CAGGCAACGCTTTGCCTTCTCAATACCTTCTATTAATAATCAATCCCCTGCTCAACGGTATCA
ATGGAAGGTCCTGCCCCAAGGTATGATGAACGCCCCTGTGGTCTGTCAATTCGTTGTTGATA
AAATTTTGCACCCCATCAGATAGCAATTCCCTGAGGCATATCTCATTCATTACATGGATGAC
ATTTTATTCATTGGCTTCTTCCTCAGAATCTCAATTAAGTTTATTAGGTAATGAGGTCATAAC
TAAACTAATCATGAGCTACTAATAGCAGAAGATAAATTGCAACACCATTCCCCTTTTAAATA
TCTTGGATATCTTATGGACCGCTCCACTGTATAGCCACAGAAGCTTTCTATTAGAAGGGATA
ATTTACAAACACTTAATGATTTCCAAAAACTTCTTGGGGATATTAATTGGCTACGACCTACCT
TGGGAATTCCCTCATATGCTTTACAAAACTTATTCAAATTATTAGAAGGTTCCTCTGATTTAA
ATAGTCCCCGACAACTTACCCCTGATGCTAAGAAAAAATTACAATTGATAGAACAGAAAATT
CAACAAACATTTGTTTACCGTGTTAATTACAATTCCCCTTTTCAGATATGTCTTTGGTACTAA
AATATCTTCTACTGCCATTCTAGTGCAGAATAATCACCTATTGAATGTGTATATCTCCATTCC
AAACAGACTAAACGCATTGTTTCCTATATAGACTTAATAGGGAAAATCATTTTTCTTGCACG
CTCTTGTTTGAGCAGTATAGCTGGATATGACCCTACTCAGATTTACCTACCTTCGACAAAAAC
AGAAATTGATAATGCTCTCCAGGTGTCCACTACCATTCAGATAGCCCTTGCTGATTATTCAG
GGGAGTTATTGGCCAACCCACCTAAGGAAAATTATGGAATTTCTTACAAAATACTTCTTTTA
TCATCAACAATATTATTTCTGAACACCCTCTCATGAATGCACCTAATTATTTTATAGATGGAA
ATAAGGCAAGATGGGCAACCATAATAGGTCCCAACCTACAAAAAAAAAAAAATTAAAAGTCC
TTATCAATCTGTTCAAAAAACAGAATTATTCACATTATATTGTTTACTTACTCTAATAAAAAC
CCCATTGAATGTTTTAACTGATTCTCGCTACGTGGCACAGCTTTTCCCATCTTTTGTAATGGC
TCATTTTATATCCAATGAAAATGATCTTATACATTTGTACTTATTGATTCAGCAGGAAATAAG
AGCAAGACTCCATCCCTTCTTTATTACTCACATCCGTGCTCGTTCCCATTTACCAGGACCCCT
CAATTTAGGCAATGATTTGGCTGATCGCCTCATCGCCCCTATATTTTCTTCCCCCGAACAGGA
ACATCAGCTCTTCCATACTAACGCTAATAGACTACACGTTCAAAATAAGATACCGTTACAAA
CGGTTGGAAAAGTTGTTCGGAACTGTGCCACATGTGCCCCCTTTCATTTGACCACTAGTCCCG
AGGGACTAATCCTAGAGGCTTACAAGCAAATGAATTATTGCAAGCTGATTTTACACATTGCA
AACTGCTCCCTTTTAAATTGTTATTTGTAGTCAGACACCTTTTCCGGCTTCATTTGGGCAGCT
CCTTCCACTGCAGAGACTACTAGAGCTGCCGTCACAGCTCTTCTGCAATGTTTTTCTGTGATG
GGGATCCCTGCCTCCATCAAAACTGACAATGGTCCAATGCTTTTCGTGATTTCATGCATCAGT
GGAGTATTTGCCATCTTACTGGCATCCCATACGGCCCTCAAGGTCAAGCCGTCATTGAATGG
GCCCACCATACACTCAAGCTCATTCTTAATAAACAAAACAGGGGAATAAACTAAGGGGCCC
CTATGGACCTAAAGCCATTTTGCCTATAGCCCTTCTAACAATCAATTATTTTAATTTGCCTCA
ATCAATTATTTTGTCTCTACACAGTCAGGAAACATGAACAGAGCGACATTTTTCTGATTCACC
CTCACGTATGCCTGAACAAATTGCTGTTTGGGTCAAATGTCTTAATCAATGGTGGCCAGGAA
CACTTAAGTTCCTAGGCAAGGGATATTATCTCGTTATTTCAGATGATGGAACTGAGCAGTGG
GTCCCACTCAAAAGAGTCAGAAGACGGACAGACCTTGCTCCACACCCCCTGACCGGTCACA
GCACTAAAACAGAAATTGCAACAGATGCCTTCTGAGCCTCCTTACGACAACGTATCCTGAAA
GAAAAAATCATCTGTGCCAGGGATGGACTGGTCCACCTGTAGAGGAAGGTTAAAGAAGTTT

CTGTTCATGCCAACATGACTTTGTTGATTGGGGGCCTCATGGACTCTGTGAATTGCTCAGAAT
CACAGAAAATAATCACACCTGCCAATGGTATAATGTTTCAGCACCACATTTTAACAGAACAC
AAACAGGACTCTAGCATCAACGCAATGAACTTTTGAACTGGTACAATGATACACCTCCCAGA
CCCCGGATAATCAGTCCAGTCCTGGGGCCTGAACACTGGCATCTTTGGAAAATTCCTGCTTG
CTTGTCCCGGTTTAGAGACTTGTATGCTTATGCACATGTTTCCATTCCACATAATTATACTAT
TGAGTATAATTATACCGGTTATGTTCATGCTTGTGTAAATGCGCCCTGTCTTTTTGCTATTGG
ACAATTTAGGAGTAATGGCTCAATTCTGTCCTGTACTGTCGTTTGTATACTTGTTTAAATCTT
AACGTGCCAATTAATGTTACTAAAGACAGTGGTTTTTTAGTCCGGCAAAGGACTGATTTGTG
GGTTCCAGTTAAGATTTCTGAACCTTGGTCAGATTCCACGTTGTTGTCTTTTGTTCTGAGAGA
ATCCCTGAAAAGAAGCAAACGCTTTATTGGTTGGATTATAGCTACCATAGAGGGTATTATTT
CAATTGTAACTGTTGGTACAGTTTCTGGAATGGCATTATATAATTCTATTCAAAATCATGATT
TCATTACTGCTTGGGAAAAGGCTTCTCATGATCTCTGGGCCCAACAAGCTCAGATAGATCAA
CAAATACAGATCACGTCTACCTTCAGCAGCATTCCCCCTGTGTTATATAACACTGACTTCTAT
AATACACTATCTGCTAGTACTTCCTTTTTAAATCCAAAAAATTGGGTACCCCATACGATGGCT
TCTTATATGCTGATCTGTTTGTTTGTTATTGTTCTTATAGGATTCCGAACGTTATGTGCTCGAG
CCACCGCTGCCCAAAAAGCAAGAGTAAAAATGGCTGCAACAGTCCTTGCTCTTGAGGAAAA
AGGGAGGAAACGTGGAGGAGCTGGAAGGCTTTGAGCAAATACTGAGAATGTATTTGCTCCA
CTCATGACAAAGGTTGGAAGCTGGGATGAGCATAACAGAGGGTTATGAGCGTTCACCGAGT
GCCCAAGTCTGGGAACGGATAATGAAGGGTTATACGCCCGGCCTTGAGGCATTTGAAGGCTT
CCTTCTGACCACCTGTTTCTGAGAGCAAGGACTGTTGTTTCATGATAAGACTCCCTTTAGAGT
TTCGCCAAAGCTATGTTATGGCTTGGGCGGTAGGAACTGTATTTTATCCTTGAATGCTTTAAT
GTTTATC

>NC_056054.1:132248742-132256073#SHEEP_RIP_02(-)

TGAAGGGTTAATAGGGTAGCAGAGATGTGCCTGCAAATGGGCCTCTCTGCTAGGGCTGGAC
GTCCTTGCAAATGAGGCATTCTGCCAAAGAGTCTGGACACAGCCTTGAGTTTGATGGTCCCT
TGCAAACAAAGGAGCATTCCTTTCTTGTGATAAAGAAGAAATAGAGGGCTTTGTACAGACTC
TGCAGTAGACTAGGATTTTACTCCCCTTTGCTATATGATAACATGTATGCACCTGCGCTGTGC
TGAAAAGGCTTATTCATGCAGTCTGGAATTCTGCTTAGGGGGCTTTTATAATAAACGGCAAT
TAATTTTTTGTCCAGTTCTGTTCCTCCGGCCAGAGTGTGCGTGTCGTCTGTCTCTTGTGTGTGT
CTTGTTCTGTGTCATTTCACTCGTAATTTCCAACATCTGGCGCCCAACGTGGGGCTCGAGTGA
AACCAAAAGGGTGAGTAACCCTGGGGGGATTTTAAATCCATAGCAGGGGAACTTTCGGGAA
AATTATGGGGAATTCCTCACCCTAGCAGAGGGGAGCTTTCAAAAAAAATCATGGGAATTCCT
CATCATTATTACGGGCACAATACATGAAGTTAGTCCAAGGACTTCTCCACTCCATAGGCGTT
AAAGCCTCGACTCGTTGGTTGAGTGGGCTCTTTCGCTTGGTGGAGCAATATTGTCATTGGTTT
CAATATCAAACTAAGTTACAGTTAAACTTGAAGGAATGGAAAATAATTCAAAAGGAATTGA
GAAAGCAACATCAGAAGGGTAATGTGATCCCTTTGAAGTTATGGACTTTGTGTAGTGCTCTA
ACACAGGCTTTGACCTTGCTCTCTACTAATAATGAAACTAAATCTAATGCTTCAGGGAGGGG
AGAAATAATTTATGAGGATGTGTCAGATGTTGGTGGGGCTTCTGCATCACCTGAAGGCAGGG
ATACAAATGAGCCTCCTCCTGTAAATGGTGAAACATCTGATAGTTCAGAATCAGATTTGGAG
GCTTCTTCAGTTTTGTCAGAGGAGGGCAAAGAGATTAAAGAAATGACCCATCTATTCCAGGA
ATGGTGGAAATCACGTAAGGAGGAGAAAAAATCTACACCTTCTGCTCCTCCTTGTGCTTCTC
TTTTCCCCACTGTGGTTAATCGGCCCGATGTGGGCAGGGAACATTGTCGGTTCTCCTTTCCTT
TGTCTATGCTTCATGATGATGACTTGTCTGCTCCCCCTGGTGGGTTTATCGATCCTCCACAAT
TGTTTCCCATCCAGAGACAGCAGGATGGCAATGCGATAAATGTTCAATATGCTCCTTTGGAA
TATAAATTTTTTAAAGATCTTAAAGCTGCAGTAGCACAGTACGGTCCTCAATCTCCCTTTGTT
TTGGCTATGCTGGAATCATTGGGAAAAGGCAAATTAATCATTCAGTTAGATTGGGAATCTAT

TGCCCAAGCTGTCTTGGAGGGCTCTCAATGGTTGCAACTTCGTAGCTGGTGGGAAGAAGAAG
CTAGAAAGCAGGCTCGGATTAATGAGGGACAGAATCCCCCTGGTCCTCTTGAGGACAAGCT
AATGGGAGAGGGCCAATATCAGCTTTAAGAGAACAGGCTCAATACTCTGATCAGGACTTAC
AACAAGTCTGCCAGGTCTTTTTATGAGCATGGCGCCGTGTGGTGCCTACTGGCCACGCCCAG
CCCTCCTTCGTTAAAACGATGCAAGGCCCCAATGAGCCGTATACTGATTTTTTTAGCAAGATT
GAGGGTAGCTGTGGAATGGGCTATAGGGAGGGATGAGATTTCAGAGATATTATTACAAACT
TTAGCATTTGAAAATGCAAATCCTGAATGCAAGCATATACTGGGACCTTTAAAGGGACAGG
GTGCACCTATAGCTGAATATATCAGAGCCTGCTCAGGAATAGGAGGGACTGAGCATCAGGC
TAATGTCTTTGCTACAGCCTTGGCCAAAATTATGAGACCACCAAAGGTAGGTAACTGCTTTC
ATTGTGGAAAACCTGGTCGTATGAAAAGAGAGTGTTGGAAATTAAAAGCTGATCAAGGTGT
ATTTCCTAAAGACAGATCTCTTGCTGGGAAGAATAAGACTCCTCCTGGACTTTGCCATTGGT
GAGGGAAGGGGTTTCATTGGACTAATGAATGCAGATGTAAAACAGACAAAATGGGCAACCT
GATACCGGGAAACTATCCTGCGGGCCTAAGTCCTTGGGGCCCAGGAACAATACCGGGGACT
TCTCCTCCTTGCCCTCCTCCCATCCCATCTGCCTCAACCCTATTCCCTTCCAACAACCGTTACG
AGTCGATGCCCCGTTAAAAGGACCTCAGATGATGATTTCAGGCTTACAGTCTGCTACTTCAG
GGAGTGCTGCTGATGATTTGCCACTAGCTGATAATGTTCTTTTGTCACCAGGGGGGAGGCATT
TATAAATTAAAAACAAATGTATTTTGACCACTGCTTAAAGGCACTTTTGGCGTGATATTAGT
CCTTAGCAGCGCGGCTTTGAGAGATTTAACCATAATTCCTGGGGTAATAGACTCTTATTATGT
TGGGGAAATTTTAATTATGGTCTCTACTTCTACCACACTTTCATTGTTAGCTGGGGAATGTAT
TGCTCAAATACTTCTCCTACCTTATCACCCCTTTTTGGCTCTTCCTAATGAACGAACAGGAGG
ATTTGGAAGTACTGGGCGACATATATTTTGGGAAATGCTTATCAAAGATTCCCACCCTGTTCT
CTCCTTGATTATACAGGGAAACAACTTTGAAGGACTAGTAGACACAGGGGCGGATGTTTCAG
TCATTTCTTCTCAACAATGGCCCTGAGATTGGGAAAAAGGAAAAAGCCCTTTAATGCTGATG
GGATTGGGCTCCATTGCAGATGTCTGGAAGAGTACCCATCCCTTACAATGTCAATTCCATAA
TGGAAGATCAGTGTTTGTTACCTTTTATATTGTAAATATACCTATTAATATATGGGGAAGAG
ATCTTCTCTCTCCTTTGGGGGCTTCTGTAACCATTCCATCGGAAAACTAGTAGCCACTGCTCA
AATTCCTCAAGCACTCCCATTAAAATGGTTAACTAATACTCCAAAATGGGTTGAGCAGTGGC
CATTACCACAAATGAAGCTCGAGGCATTAGAACAATTAGTACAAGAACAACTCCAACTTGG
TCATATAGAGCCCTCTACCTCACCCTGGAATTCTCCTGTTTTTGTTATAAAAAGAAATCTGGA
AAATGGAGAATGTTAACTGATTTACAAGGAGTTAATAAATGTATTGAACCTATGGGAGCCTT
GCAGTTTGGGACTCCCCTCTCCAGCTCTTATTCCTCAGAATTGGTCCTTAATGGTGTTAGATC
TTAAAGACTGGTTTGTTTGTTTTGTTTTGTTTCGTTTTTTTTACCATTCCCCTACAATTGCAAG
ATAGAGATAAATTTGCTTTTACAGTTCCTGTTCTTAATCATGCTCAGCCTGTTAAGTATTATC
AATGGACAGTCTTACCACAAGGAATGATAAATAGTCCTACCTTATGCCAAGAGTTTGTAGCT
TGCTCTTTACAATCCCTCCGTCAAGAATACTCCAATTATATTCTATATCATTATATGGATGAT
CTCCTATTGGCAGCTCCTAGTATTGCTGAACGTGACGAATTCTTTTTAAAAGTACAGGAGGC
TTTAAGACTATACAATTTGCAAATAGCCCTGGAAAAAAATCAAAAGGACTTTCTTATTTCGT
ATTTAGGGACAATATTGGAACAACATAGAATTAGGCCCCAAAAGTTGCAAATTAGAAGAGA
CCATCTCAAAATCTTAAGTGATTTTCAAAAGTTATTGGGAGATATTAATTGGCTATGCCCGGT
ACTTGGGATTCCTACTTATCAATTGTGACATTTGTTTTCTACTTTAGAAGGAGATATAGCTCT
GGATAGCCCCCAGACCTTAACCCCATTGGCTTTACAGGAACTTCAATTTGTTGAGCAATGAC
TAAATGACGGCTTTTTGACTGACTTACATGCATCTCAACTTATTTCTTTTATAATATTTCATAC
CCTTTATTTCCCATCTGGTGTAATTGCTCAAGAAAGAGGATTAATAGAATGGGTTTTCTTACC
TAACAGTTTTTCCAAAAAATTGACTATATATATGGATAAATTAGTCTTCCTTATACAGAAGG
GTCACCATCGTATTTTACAATTATCAGGATGTGAACCACAGAAGATTGTTACTCAGTTAACA
ACTGCTCAAATATCTCGATGTTTACAATTTAATGAAAACTGGCAAATTTCTCTTGCCTCATAT
CCTGGTTCGCTTTCTAATCATTATCCATCATCTAAATTGATTGATTTTCTCCGGACTAACACT

ATGATATCTCATTCCCCAATTTCAGATGTTCCAGCTAAGGGAACCACTATTTTTACAGATCCA
AATAAAAATACTGCTGGATATTGGACCCCCCAAAATTCCAAGGTTCTCCCCCACTCATTTTCT
TCTGTACAGCCTGCTGAATTGTGGGCTATCTATTTAGTTTTGCAAGATTTTCCCCAACTTCCT
ATTAACATTTTTTCAGATTCTTGATATGCTGTTCTTTCTTGCCTACAGCTTCCCCGTGTCTCCC
TTCCATTGACTCTTAAAACAGCTTTTGATAAATTGTTTTACCAAGTACAACAATTGCTCTTGC
AGCGTTCAGAGTTAATTTTCTTTACTCACATCCGTGCACATTCTGCCCTTCCTGGACCCTTAT
CATCCGGAAATGCTACAATTGATGCCTTACTTTATCCTATAGGAGCAGCAAAACAAGAACAT
CTCTTACAACATACTAACTCCAAAGGGTTACAAAACTCTCATGCTATGACTTGAAAACAAGC
TCAAAATATTGTTCATTCTTGTTCCATATGTGCACCCTTTGCTTTGCCATTTACCCCACCAGGT
GTCAACATAAGAGGACTACAAGCAAATCAGATATGGCAAACGGATGTAATTTACATTTCTTC
CTTCGGACAACAAAAATGTGTGCATCATACTATAGATACTTGCACACATTTTCAATGGGCCA
CTGCATTACATTCTGAAAAGGCTGACGCTGTTATTACTCATTTGTTATCTTGTTTTGCAGTTAT
GGGATTACCAATTGAATTGAAAACTGATAATGCACCTGCTTACCAATCCGCAAAATTAGCTC
ACTTTTTATCTCAATACCATATAACTCATATTTTTGGTATTCCTTATAATAGTCAAGGGCAAG
CTATCATTGAAAGAGCTAATTGTACCTTGCATGATTATCTTGAAAAAATAAAAAAGGGAGAA
CAAGAGAGATTTATGGAATTTAGGGAATAGAGCAACTGTCTCATCATAAACCACAATATAAT
ATTAGTAATTTGACCATTGCAGTGGAAGGTATTCCTTTATGTAAAGGGGGACACCCCTTTTGT
CTGTCCACCAAGGAACATTCTCATCATTCTTACAATACATGGGGGGTAAAATATAATAATTA
CCATTTTGCTACTTTTACTGTGCTTGTTTCCACCAGGGGGCTTAGCACCTCGACAGAACTGAT
AGATATTCATAATGGAAAACACATGTCACTATGTCCTGTTAGATTTTTTGTTCCTTGTCTAGA
ATATTTGGAGTGGGAACGTTGCCGAGGTCATCGACCCTTTAAAGTCATGAATTATTCTGGGG
CCATCATTGTAGATTGGAGTCCAGATCATGGGCAATTCTTAGAAAAATGGTCAAGTAAATCT
TTTAGGTGGCATTGTGCAAATAGCACTTTGAATGGCAATGGTAATGAAACAGTTAAATGGCA
GCAATTTGCACTTGTCCCTCCTCAATTACAATTGCAAGGATATCCGCACATTCAAGGAGATA
TTTGGAAACTATGCGCAGTTTCTGGTAATCTCACTATCTGGTCAGGAAACTATGCTTTGGACA
GTGGTGACTCTTCGGGTCCATTCCATGTTAATTCACATGTTAATAAATCTTATTCTGCAATAG
CATGTGTAAAATATCCTTTTGCATTGTTATATGGGAATTGGACCTGGAAGGATACTGTGGGG
TCTGTGTCATGTGACTATTATAATCTAACTCAATGTGTAAATCAGTCTTGGTGGGAAGAATTT
GAAAGACAAGCCTATAATTCCAATTTCTCGCTAGTAATTGTTAAGGCTTGGACAGAAGTATG
GTTACCTATAAATGTGACTCAGCCGTGCTCAGATTCTTTTGCTGTTTCTCATCTAGTAACCGC
TGTACAGACTTTGCTACACCGATCTCGACGTATGCTTGGTGCGGTCATTGCTTCGATTCTAGC
AGTTGCGTCAGTAACTGCAACAGCAGCAGTGGCAGGTCTTGCATTACACCAAGGAATTCAA
ACAGCTGATTTTATTTGGGACTGGCATAAAGACTCTCATTTGTTATGGCGACAACAGCGAGA
TTTGGATGCCCAACCTGCTACCAACATGCTCAATCTTCAACACACCGTTTTCTGGCTTGGAGA
TCAATTGGCTGTTTTATCTACACGAAGTGTGTTGAAATGTGATTGGAATTCTTCTCAGTTTTG
TATAACACCTGTACCATTTAACATGAGTGAAGGATGGGATAAAGTAAAACGATCCTTGACTG
GGCATCAAAATCTCACTACGGAGATTATGGACCTGGAACGACAAATTTTGTCTACTTTTAGC
AGGACTTTACCTGACATTACAGAGTCTGATTTGCTGAAAAGTCTTCAAGAGGGAATGAATAA
CTTAAATCCATTAGGGCATGTATCCTCACTAATTGGGACTACTTTTGGGAACACTGTGTTTAT
ATTACTTTTATGTTGTGTTGCTTTTCTAGTCTTCCGGCGATGGCGGAAAGGGAAACAACTAAA
GCACGAAGCAGAGAAGATCCAGACCATGCTACAATTTATAAAAGCAAATGAAAAAGGGGG
AGATGAAGGGTTAATAGGGTAGCAGAGATGTGCCTGCAAACGGGCCTCTCTGCTAGGGCTG
GACGTCCTTGCAAACGAGGCGTTCTGCCAAAGAGTCTGGACACAGCCTTGAGTTTGATGGTC
CCTTGCAAACAAGGGAGCATTCCCTTCTTGCGATAAAGAAGGAATAGAGGGCTTTGTACAG
ACTCTGCAGTAGACTAGGATTTTACTCCCCTTTACTATACGATAATATGTATGCACCTGCACT
GTGCTGAAAAGGCTTATTCATGCAGTCTGGAATTCTGCTTAGAGGGCTTTTATAATAAACAG

CAATTAGTTTTTTGTCCAGTTCTGTTCCTCTGGCCAGAGTGTGCGTGTCGTCTGTCTCTTGTGT
GTGTCTTGTTCTGTGTCATTTCACTCGTAATCTCCAACA

>NC_056054.1:236947557-236954938#SHEEP_RIP_03(+)

AGGTGAAGGGTTAATGCGGCCACAATAGGGAAAGTGGAGATGTGCTGCTTACAAACAAGAT
GTTCTGCCAAGGAGACGGACACAGCCTTGAGACTAATGGTCCCTTGCAAACGAGGGAGCAT
TTCCTTCTTGTGATAAGGGCTCTGGACGCACTCTGCAGTAGGCCGGAATTTCACTCCCTCTTG
CTGTACGATAACATGTATGCACCTGCACTGTACTGAAAAGGCTTACTCATGCAGTCTGGAAT
TCTGCCTATAAAAGCGCAAATAGTTTGTGCAGTTCTGTCCCTCCAGCTGGAGTGTGTGTATCG
TCTGTCTCTTGTGTGTGTGTGTCTTGTTTTGTGTCATTTCACTTGTAATCTCCAACATCTGGCG
CCCAACGTCGGGCTCGAGTGAAATCGAAAGAGTGAGTAACCCTGGGGGGATTTTAAATCCA
TAGCAGGGGAACTTTCAGGAAAATCATGGGGAATTCCTCACCCTAGCAGGGGAACTTTCAG
AAAATCATGGGGAATTCATCATCATCATTACGGGCACAATACATGGAGTTAGTCAAAGGACT
TCTCCACTCCATAGGCGTTAAAGCCTCGACTCTTTGATTGAGTGAGCTTTTTCACTTGGTGGA
GCAATATTGTCATTGGTTTCAATATCAAACTAAGTTACAGTTAGACTTGAAGGAATGGAAAA
TAATTCAAAAGGAATTGAGAAAGCAACATCAGAAGGGTAATGTGATCCCTTTGAAGTTATG
GACTTTGTGTAGTGCTATAACACAGGCTTTGACCTTGCTCTCTACTGATAATGAAACTAAATC
TAATGCTTCAAGGAGGGGAAAAATAATTTATGGGAATGTGTCAGACATTGGTGATGCTTCTG
CATCGCCTGAAGGCAAGGATATAAATAAGCCTCCTCCTGTAAATGGTGAAACATCTGATAGT
TCAGAATCAGAATCGGAGGCTTCTTTGGTTTCGTCAGAGGAGGGCAAAGAGATTAAAGAAA
TGACCCATCCATTCCAGGAATGGTGGAAATCCTGTAAGGAGGAAAAAAAATCTACCCCTTCT
GCTCCTCCTTGTGCTTCTCTTTTCCCCACTGAGGTTAATCGGCCCAATGTGGGCAGGGAACAT
TGTCGGTTCTCCTTTCCTTTGTCTGTGCTTCATGATGATGACTTGCTGCTCCCCCTGGTGGGTT
TATCGATCCTCCACAATTATTTCCCATCCAGAGACAGCAGGATGGCAATGTGATAAATGTTC
AATATGCTTCTTTGGAATATAAATTTTTTAAAGATCTTAAAGCTGCAGTAGCGCAATACTATC
CTCAATCTCCCTTTATTTTGGCTATGCTGGATTCATTGGGGAAAGGCAAATTAGGGGAAAGA
AAAATTCAGAGATATTATTACAAACTTTAGCATTTGAAAATGCAAATACTGAATGCAAACGT
ATACTGGGGCCATTAAAAGGACAGGGTACATCTATAGCTGAATATATCAGAGCCTGCTCGG
GAGTAAGAGGAACTGAGCATCAAGCTAATGTCTCTGCTATAGCCTTGGCCAAAACTATGAG
ACCACCAAAGGGAGGTAACTGCTTCCATTGTGGAAAACCTGGTCATATGAAAAGAGAGTGT
TGGAAATTAAAAGCTGATCAAGGTTCAATTCCTAAAGACAGATCTTTTGCTGGGAGGGATAA
GACTCCTCCTGGACCTTGCTGTCGATGCGGGAAGGGGCTTCATTGGACTAATGAGTGCAAAT
CTAAAACAGACAAAATGGGCAACCCAATACCGGGAAACTATCCTGCGGGCCTAAGTTCTTG
GGGCCCAGGAACAATACCAGGGACTTCTCCTCCTTGCCCTCCTGCCATCCCACCTGCCCCAA
CCCTGTTCCCTCCCAGCAACCATTACAAGTCAATGCCCCATTAAAAGGACCTCAGATGATGA
TTTCGGACTTATGGTCTGCTACTTCAGGGAGTGCTACTGCTGATTTGCCACTAGCTGACAATG
TTCTTTTGTCACCAGGGAGAGGCATTTATAACTTAAAAACAAATGTATTTGGACCACTGCCT
AAAGGCACTTTTGGCTTGATATTAGGCCATAGCAATGCGGCTTTGAGAGGTTTAACCATAAT
TCCTGGGTTAATAGACTCTGACTATGTTGGGGAAATTTTAATCATGGTCTCTACTTCTACCAC
ACTTTCATTGTTAGCTGGGGAACATATTGCTCAAATACTTCTCCTACCTTATCACCCCTTTTTG
GCTCTTCCTAATGAACAAACAGGAGGATTTAGAAGTACTGGGCAATATATATTTTGGGAAAT
GCTTATCAAAGATTCTCACCCTGTTCTCTCCTTGATTATACAAGGAAACAACTTTGAGGGACT
AATAGACACAGGGGCAGATGTTTCAGTCATTTCTTCTCAACAATGGCCCCAAGATTGGGAAA
AAGAAAAAAAGCCCTTTAATGCTGATGGGATTGGGCTCCATTGCAGACGTCTGGAAGAGTA

CCCATCCCTTGCAATGTCAATTCCATAATGGAAGATCAGTGTTTGTTACCTTTTATATTGTAA
ATATACCTATTAATATATGGGGGAGAGATCTCCTCTCTCCTTTGGGGGCTTCTGTAACCATTC
CATCGGAAAACTAGTGGCCACTGCTCAAATTCCTCGAGCACTCCCATTAAAATGGTTAACTA
ATACTCCAAAATGGGTTGAGCAGTGGCCATTACCACAAATGTAGCTCGAGGCGTTAGAACA
GTTGGTACAAGAATAACTCCAACTTGGTCATATAGAGCCCTCTACCTCACCCTGGAATTCTC
CTGTTTTTGTTATAAATAAGAAATCTGTAAAATGGAGAATGTTAACTGATTTACGAGAAGTT
AATAAACGTATTGAACCTATGGGAGCATTGCAATTGGGACTCCCCTCTCCAGCTCTTATTCCT
CAGCATTGGTCCTTAATGGTGCTAGATCTTAAAGACTGGTTTTTTACCATTCCCCTACAATTG
CAAGATAGAGATAAATTTGCTTTTACAGTTCCTGTTCTTAATCATGCTCAGCCTGTTAAGCAT
TATCAATGGACAGTCTTACCACAAAGAATGATAAATAGTCCTACCTTATGCCAAGAATTCGT
AGCTCGCTCTTTACAATCCCTCCGTCGAGAATACCCCAATTATATTCTATATCATCATATGGA
TGATCTCCTATTAGCAGCTCCTAGTATTGCTGAACGTGATGAATTCTTTTTAAAAGTACAGGA
GGCTTTGAGACTATACAATTTGCAAATAGCCCAGGAAAAAATTCAAAAGGACTTTCCTATTT
CATATTTAGGGACAATATTGGAACAACATAGAATTAGGCCCCAAAAGTTGCAAATTAGAAG
AGATCATCTCAAAATCTTAAATGATTTTCAAAAGTTATTGGAAGATATTAATTGGCTACGCC
CAGTACTTGGGATTCCTACTTATCAATTACGACATTTGTTTTCTACTTTAGAAGGAGATACAG
CTCTGGATAGCCCCCAGACCTTAACCCCATTGGCTTTACAGGAACTTCAATTTGTTGAGCAA
CGACTAAATGACGGCTTTTTGACTTACTGACATGCGTCTCAACCTATTTCTTTTATAATATTT
CATACCCCTTATTCTCCATCTGGTGTAGTTGCTCAAGAAAAAGGACTAATAGAATGGGTTTT
CTTACCTAACAGTTTTTCCAAAAAATTGACTATATATATGGATAAATTAGCCTTCCTTATACA
GAAGGGTCGCCATTGTATTTTACAATTGTCAGAATGTGAACCACACCAGATTGTTACTCAGT
TAACAACTGCTCAAATATCTCGATGTTTACAATTTAATGAAAACTGGCAAATTTCTCTTGCCT
CATATCCTGGTTCATTTTCTAATCATAATCCATCATCTAAATTGATTGATTTTCTCTGGACTAA
CAATATGATATCTCATTCCCCAATTTCAGATGTTCCAGTTAAGGGACCCACTATTTTTACAGA
TGCAAATAGAAATACTGCTGGATATTGGACCCTGGAAAGTCCCAAGGTTCTCCCCCACTCAT
TTTCTTCTATACAGCCTGCTGAATTATGGGCTATCTATTTAGTTTTGCAAGATTTCCCCCAATT
TCCTATTAACATCGTTTCAAATTCTCAATATGCTGTTCTTTCTTGCCTACAGCTTCCCCATGTC
TCCCTTCCACTGACCCTTAAAAGAGCTATTGATAAATTGTTTTACCAAGTACAACAATTGCTC
TTGCAGCGTTCAGAGTTAATTTTCTTTACTCACATCTGTGCACATTCTGCCCTTGCTGGACCC
TTATCATTTGGAAATGCTACAATTGATGCCTTACTTTATCCTATAGAAGCAACAAAACAAGA
ACATCTCTTACAACATACCAACTCCAAAGGGTTACAAAAATCTCATGCTATTACTCAAAAAC
AAGCTCAAAATATTGTTCGTTCTTGTTCCATAGGTGCACCCTTTGCTTTGCCATTTACCCCAC
CAGGTGTCAACATGAGAGGACAACAAGCAAATCAGATATGGCAAAAGGATGTAATTTTCAT
TTCTTCCTTCGGACAACAAAAATGTGTGCATCATACTATAGATACTTGCACACATTTTCAATG
GGCCACTGCATTACATTCTGAAAAGGCTGATGCTATTATTACTCATTTGTTATCTTGTTTTGC
AGTTATGGGATTACCAATTGAATTGAAAACTGATAATGCACCTGCCTACCAATCCGCAAAAT
TAGCTCACTTTTTATCTCAATACCATATAACTCATACTTTTGGTATTCCTTATAATAGTCAAG
GGCAAGCTATCATTGAAAGAGCTAATCGTACCTTGTCTGATTATCTTGAAAAAATAAAAAAG
GGGGAACAAGAGAGATTTATGAAACCTAAAGATTTATGAAACCTAAAGACATTCTGAATAA
GACCTTACTTACCCTAAATTTTTTGAATGTTTGGAGCAAGGGAAATCTATCAGCAGCAGAGT
TGCATTTTCAAGGGAAAGAAGAGGATAAGAAGATCTTGAATATGCCTATTTGGTATAAAGAT
AAAGAGAAAGGTTGGATCCCAGCATCATTAATATATTTGGGATGAGGGTATGCTTTCATTTC
TGTTGATAATTACAGGTTTTGGACCCCAGCAAAATTGATCAAAATCAGCAATGGCTGATCCC
CTTGTTCAAAAAATTCGAAGAGCTTACTATGCAGAGAAGCCTTACTTCCCATACAAGGGATG
CAACACCTCCTACATGGGGTCAATTGAAGAGGTTGACCCAGGAAGCAAAGAAAATGTTAAT
GAAGGTGGGGCAACCTCTGAATCCTGCCAATCTTTTGCTTGCCATGATGGCGGTGGTGACAT
GTCAGATAATCGGTGTATCGGCAAGTAATCATACATACTGGGCATATATACCTAATCCCCCA

TTAGTAAGAGCAGTTTCCTGGGGGGAGGCAGAAGTGCAGGTATGCACTAATGAGACTGCCT
TCTTTCCCCCGCCAGCTTGCAGGGGAATAGAACAACTATCTCATCATAAACAGCAATATAAT
ATTAGTAATTTGACCATTGCAGTGGAAGATATTCCTTTGTGTATAGGAGGACACCCCTTTTGT
CTGTCCACCAAGGAACATTCTCATCATTCTTATAATACATGGGGGGTAAAGTATAATAATTA
CCATTTTGCTACTTTTACTGTGCTTGTTTCCACCAGGGGATTTAACACCTTGACAGAACCGAT
AGACATTCATAATGGAAAACACATGTCACTATGTCCTGTTAACTTTTTTGTTCCTTCTCTAGA
ATTTTTGGAGTGGGAACATTGCCGAGGTCATCGACCCTTTAAAGTCATGAATTATTCTGGGG
CCATCATTGTAGTCCAGATCATGGGCAATTCTTAGAAAAATGGTCAAATAAATCTTTTAGGT
GGCATCATGCAAATATCACTTTGATGGGCAATGGTAATGAAACAGTTAAATGGCAGCAATTT
GCACTTGTCCCTCCTCAATTACAATCACACGGATATCCGCACATTCAAGGAGATATTTGGAA
ACTATGGGCGGTTTCTGGTAATCTCACTATCTGGTCAGGAAACTATACTTTGGACAGCAGTG
ACTCTTCAAGTCCATTCCATGTTAACTTACATGTTAATAAATCTTATTCCACAATGGCATGTG
TAAAATATCCTTTTGCATTGTTATATGGGAATTGGACCTGGAATGATACTGTGGGGTCTGTGT
CATGTGACTATTGTAATCTAACTCAATGTGTAAATCAGTCTTGGTGGGAAGAATTTGAAAGA
TGAACCTATAACTCCAATTTCTCGTTAGTAATTATTAAGGCTCGGACAGAAGTATGGTTACCT
ATAAATCTAACTCGGCTGTGGTCAGATTCTTTTGCTGTTTCTCATCTAGTAACTGCTGTACAG
ACTTTGCTACACTGATCTTGACGTATGCTTGGTGTGGTCATTGCTTCGATTCTAGCAGTCACG
TCAGTAACTGCAACAGCAGCTGTAGCAGGTCTTGTGTTACACCAAGAAATTCAAACAGCTGA
TTTTATTCGGGACTGGCATAAAGACTCTCATTTGTTATGGCAACAACAGTGAGATTTGGATG
CCCAACTTGCTACCGACGTGCTCAATCTTCAATACACTGTTTCCTGGCTTGGAGATCAACTGG
CTGTTTTATCTACACGAAGTGTGTTGAAATGTGATTGGAATGCTTCTCAGTTTTGTATAACAT
CTGTACCATTTAACATGAGCCCTTCAAGAGATTAACCAAGATATATAAATAAAGAGTTCTAG
ATGCCCAACATCTAGAAAGATTCCACCTAGCCCCCAAGAGTTGGCTGGATAGCCAATGACG
GGTAAGACCCTCAGAGGAGGTCAACCTAAGACAGGCACAGCCACGAGAGGGGCTGGCGAG
GCTGGACGTTGGCCCCCTACAGCTTTACGCCTTGCTCTACAGAACATCAATACAAATGTCTC
GAGGGCTTAAATAAAATAAAAAAGGGGGAGATGAAGGGTTAATGTGGCCACAATAGGGAA
AGTGGAGATATGCTGCTTACAAACAAGATATTCTGCCAAGGAGATGGACACAGCCTTGAGA
CTAATGGTCCCTTGCAAACGAGGGAACATTTCCTTCTTGTGAGGGCTCTGGACAGACTCTGC
AGGAGGCCGGAATTTCATTCCCTCTTGCTGTACGATAACATGTATGCACCTGCGCTGTACTG
AAAAGGCTTATTCATGCAGTCTGGAATTCTGCCTAGGGGGGCTTTATAATAATAAAGCGCAA
ATAGTTTGTGCAGTTCTGTTCCTCTGGCCGGAGTGTGTGTATCGTCTGTCTGGTGTGTGTGTG
TGTCTTGTTTTGTGTCATTTCACTCGTAATCTCCAACA

>NC_056054.1:272298309-272306235#SHEEP_RIP_04(-)

CTGCGGGGGACGACCCGTGAAGGGTTAAGTCTTGGGAGCTCCCTGGCAGGTATGCCGGGCC
CTAGGACACGTGCCTAAGCTCCCTGTCCCGCCACCCTCAAGAACTTTTGTAACCCTTAAGGC
TCCAAGATGTTTGGTTTCGGCAACATTTCATAGAAGATAGATTATCTTATTGTGTATACTTCA
TAGAAGATAGATATTCTGATTGTGTTCTATATACAATGGTAAGGGTCTGGTGATTGTATCCTG
AGATTAAAAACAACCTTGTGAGTGCCTTAAGTCACGTACTTTACCCTATATATACCGCAGCA
CAATAAAGCAAGGTATCAGCCATTTTGTTCTGATCCTCTCAACCCCATATTTTGTCTATCTCT
TATTTTCTTAGCGGGGACGCTCCGTTCTCTCCCCTGTGCAGGTGCGACTCCTGCTTGTGCTGG
CCGCGGCAGGTGGCGCCCAACGTGGGGCCGTTCGACAGCTTTCCTCGCCACTACTCTTATTA
ATTGAAAAGAGTGAGTATATGAGTACACAAGTGAATTAATTTGAGGAGGAGTAGTAAGGTA
TATAGTTGAGAGTATAAATATGGGACAGACGCATAGTCGCCAGTTGTTTGTGCATATGTTAT
CTGTAATGTTAAAACATAGGGGAATTACTGTTTCTAAACCTAAATTAATCAATTTTCTTTCAT
TCATCGAGGAAGTTTGCCCTTGGTTCCCAGAGAAGGTACAGTAAATTTAGAGACATGGAAG
AAGGTAGGGGAACAAATTCGGACTCATTATACGTTACATGGCCCTGAAAAAATCCCTGTCGA

AACTTTATCCTTTTGGACACTAATTCGTGACTGCCTGGACTTTGATAATGATGAATTAAAATG
TTTAGGAAATTTATTAAAACAGGAAGAAGATCCTCTCCATGTTCCTGATTCGGAACCCAGGT
ATGCTGTTCCCGAGGGGGTTGAAAGCGACCCTCCGTTTTCTAACTTATTGCGTCCTTCGGATA
ATGATGATTTACTTTCATCCACAGATGAGGCAGAATTAGACGAAGAAGCTGCTAAATACCAT
CAAGAAGATTGGGGTTTTTTAGCACAAGAAAAAGGGGCTTTAACATCTAAAGATGAATTGG
TTGAATGTTTTAAAAACCTCACTATTGCTTTACAGAACGCAGGAATCAAGCTTCCTAGTAAC
AATGCCAAATCTCCTTCTGCTCCGCCTCTTCCCCCTGCTTATGCTCCTTCTGTTGTGGCTGGTC
TCGATCCCCCTCCAGGGCCCCCTCCACCGTCTGAGAGCATGTCTCCGCTGCAAAAGGCATTG
AGACAGGCACAGCGACTTGGTGAGGTTGTCTCTGATTTTTCTCTTGCTTTTCCTGTCTTTGAA
AATAACAACCAGCGTTATTATGAATCACTGCCTTTTAAACAACTGAAAGAGTTAAAGATTGC
TTGCTCACAATACGGTCCTACCGCTCCATTCACCATTGCTATGATAGAAAGTTTGGGTACTCA
AAATCTACCCCCAAATGATTGGAAACAAATAGCTAGCGCATGTCTCTCAGGGGGAGATTATT
TATTATGGAAATCTGAATTTTTTGAACAATGTGCTCGTATAGCCGATGTTAATCGACAGCAA
GGTATACAGACCTCCTATGAAATGTTGATTGGTGAAGGCCCTTACCAGGCTACTGATACTCA
ACTTAATTTCTTACCTGGTGCATATGCACAAATATCAAATGCAGCTCGGCAGGCATGGAAAA
AACTTCCTAGCTCCAGTACTAAGACAGAGGATCTTTCAAAAGTCCGGCAGGGACCTGATGAG
CCTTACCAGGACTTTGTGGCACGGCTCTTAGATACTATAGGTAAGATAATGTCAGATGAACA
GGCTGGGATGTTATTGGCAAAACAATTGGCTTTTGAAAACGCTAACTCTGCTTGTCAAGCTG
CTTTAAGACCTTATCGAAAAAAGGGAGATCTGTCTGATTTTATTCGCATTTGTGCTGACATTG
GACCCTCCTACATGCAAGGCATTGCTATGGCAGCAGCATTACAAGGAAAAAGCATAAAAGA
GGTACTTTTTCAGCAGCAAGCCCGGAACAAGAAAGGACTTCAAAAGTCAGGTAATTCGGGT
TGCTTTGTTTGTGGTCAGCCTGGCCATCGGGCTGCAGTGTGCCCTCAAAAACAACAAAGCCC
TGTTAACACTCCTAATTTGTGCCCACGCTGTAAAAAAGGAAAGCATTGGGCGCGGGATTGCC
GTTCCAAAACGGATGTTCAAGGTAATCCTTGCCCCCGGTTTCGGGAAACTGGGTGAGGGCCA
GCCCTGGCCCCGAAACAATGTTATGGGGCAACACTGCAGGTTCCAAAAGAACCATTGCAGA
CCTCTGTCGAGCCACAAGAGGCAGCGCGGGATTGGACCTCTGTGCCACCTCCTACACAGTAT
TAACTCCTGAGATGGGGGTCCAAACCCTTGCCACAGGAGTGTTTGGGCCTTTACCTCCAGGG
ACAGCTGGACTGCTTTTAGGGCGCAGCAGTGCGTCTTTAAAAGGAATACTTATTCATCCTGG
TGTGATTGACTCTGATTATACAGGAGAGATAAAAATATTAGCCTCCGCTCCTAACAAAATTA
TTGTAATCAATGCAGGACAGCGTATAGCTCAACTTCTTTTAGTTCCATTAGTCATACAAGGA
AAACAATTAACCGAGACCGTCAAGATAAAGGTTTCGGGTCCTCTGACGCCTTTTGGGTGCAA
AATGTTACCGAGGCACGACCAGAACTTGAGCTACGCATTAATGGTAAGCTTTTCCGCGGAGT
GCTTGATACAGGGGCCGATATTAGTGTTATTTCTGATAAATATTGGCCTACTACATGGCCAA
AACAGATGGCTATTTCCACTCTCCAGCGTATTGGCCAAACTACCAATCCAGAACAGAGTTCA
TCCCTTCTTACTTGGAAGGATAAAGATGGACATACAGGCCAATTTAAACCTTATATTCTGCC
CTATCTTCCAGTTAATCTATGGGGCGTGATATATTAAGCAAAATGGGTGTTTATTTATATAGT
CCTTCACCCACTGTGACAGATTTGATGTTAGATCAGGGCTTACTTCCAAATCAAGGTTTAGGT
AAACAACATCAAGGCATCATTTTGCCCTTGATTTAAAAACCTAATCAAGATCGAAAAGGCTT
GGGGTGTTTTCCCTAGGGACCTCTGATTCTCCTGTGACACATGCCGATCCTATTGATTGGAAA
TCGGAGGAACCGGTATGGGTCGATCAGTGGCCCCTAACACAGGAAAAACTTTCTGCCGCAC
AACAGCTGGTGCAGGAACAGCTGAGACTTGGGCATATTGAACCCTCTACCTCTGCGTGGAAT
TCCCCAATTTTTGTTATTAAAAAGAAGTCTGGGAAATGGAGATTGCTACAAGATCTTCGTAA
GGTAAATGAAACAATGATGCATATGGGAGCCCTACAACCTGGGTTGCCCACTCCTTCTGCTA
TACCTGATAAATCCTATATCATTGTTATAGATTTAAAAGATTGTTTTTACACTATTCCTCTTGC
ACCTCAAGATTGCAAAAGATTTGCTTTCAGTTTACCCTCTGTTAATTTTAAAGAGCCTATGCA
ACGCTATCAATGGAGAGTTCTCCCGCAAGGAATGACTAATAGCCCTACGCTGTGCCAAAAAT
TTGTTGCTACAGCAATAGCTCCGGTTCGTCAACGTTTTCCTCAGCTATATTTGGTTCATTATA

TGGATGATATATTACTAGCTCATGCTGACGAACATCTATTGTATCAAGCTTTTTCGATTCTAA
AACAACATTTAAGCCTTAATGGTCTTGTTATTGCTGATGAAAAAATTCAAACTCATTTTCCTT
ATAATTATTTGGGTTTCTCCTTATACCCTCGCGTTTATAATACCCAATTAGTAAAACTGCAGA
CTGACCATTTGAAAACTCTAAATGACTTTCAAAAACTTTTAGGAGACATTAATTGGATACGT
CCTTATTTAAAATTATCCACTTATACCTTGCAGCCATTATTTGACATCCTTAAAGGTGACTCT
GATCCTGCGTCACCCCGAACACTTTCTTTAGAAGGACGAACAGCTTTACAATCAATAGAAGA
AGCTATTAGACAACAACAGATTACTTATTGTGATTACCAACGATCATGGGGTTTGTATATAC
TTCCTACCCCCCGAACACCCACAGGGGTTCTCTATCAAGATAAACCTTTGCAATGGATATAT
TTGTCTGCTACTCCAACTAAACATCTGCTCCCTTACTATGAACTTGTTGCAAAAATTGTAGCA
AAGGGACGTCACGAGGCCATCCAATATTTTGGTATGGAACCCCCTTCATTTGTGTTCCTTATG
CTTTAGAACAACAAGATTGGCTTTTTCAATTTTCAGATAATTGGTCTATAGCTTTTGCAAATT
ACCCGGGACGGATTACTCATCATTACCCTTCTGATAAATTGTTACAATTTGCTAGCTCTCATG
CCTTTATTTTTCCAAAAATAGTTCGCCGACAACCTATTCCCGAAGCGACACTTATATTTACAG
ATGGATCTTCTAATGGAACTGCAGCTTTAATCATTAACCATCAAACCTATTACGCACAAACC
AGTTTTTCTTCTGCTCAAGTTGTGGAATTATTTGCAGTCCACCAAGCGTTGCTAACTGTACCT
ACTTCCTTCAATTTATTTACAGACAGCTCCTATGTGGTCGGTGCCTTACAGATGATTGAAACT
GTTCCAATTATTGGCACCACCTCTCCTGAAGTTCTTAACTTATTTACATTGATTCAACAGGTT
CTCCATTGCCGCCAACACCCCTGTTTCTTTGGACATATTCGTGCACACTCCACCCTTCCTGGT
GCCCTGGTACAAGGCAATCACACTGCGGACGTTTTTACTAAACAAGTGTTTTTTCAATCAGC
TATTGATGCAGCCCGAAAGTCCCATGATTTACATCACCAAAATAGTCATTCTTTACGCTTGCA
ATTTAAAATTTCCCGTGAAGCTGCACGACAAATTGTTAAATCTTGCTCTACCTGTCCTCAATT
CTTTGTTCTCCCTCAATATGGTGTCAACCCTCGAGGTTTACGCCCTAATCACCTCTGGCAAAC
AGATGTTACTCACATTCCTCAATTTGGGCATCTTAAATATGTTCATGTCTCTATTGACACTTTT
TCCAATTTTCTCATGGCCTCCCTTCACACTGGAGAATCAACACGTCACTGTATTCAACATTTG
CTGTTTTGCTTTTCTACTTCAGGAATCCCACAAACCCTTAAAACAGATAATGGACCTGGTTAT
ACTAGCCGTTCTTTTCAATGTTTTTGTCTTTCTTTCCAAATTCATCATAAAACAGGAATTCCTT
ATAATCCACAGGGACAAGGTATTGTGGAACGAGCCCATCAACGCCTTAAACATCAATTATTA
AAACAAAAAGGGGAATGAACTGTATAGCCCCTCACCGCATAACGCCTTAAACCATGCTCTTT
ATGTTTTAAATTTTTTAACTTTAGACGCAGAAGGCAATTCAGCAGCCCAGCGTTTTGGGGAG
AACGATCCTCATGCAAAAAACCACTTGTACGATGGAAGGATCCACTTACCAATCTGTGGTAT
GGGCCAGACCCTGTACTAATATGGGGACGAGGGCATGTTTGTGTTTTTCCACAGGATGCCGA
AGCGCCGCGCTGGATTCCGGAAAGGCTGGTACGCGCGGCAGAGGAACTCCCTGACACATCA
AATGCAACGCATGACACTGAGCGAGCCCACGAGTGAGCTGCCTACCCAGAGGCAAATTGAG
GCGCTGATGCGATATGCTTGGAATGAGGCTCATGTACAACCTCCAGTGACACCTACTAATAT
ACTGATCATGTTATTATTATTGTTACAGCGGATACAAAACGGGGCGGCTGCGGCTTTTTGGG
CATACATTCCTGATCCGCCTATGATTCAATCCTTAGGATGGGATAAAGAAACAGTACCTGTA
TATGTTAATGATACAAGTCTTTTAGGAGGAAAATCAGATATTCACATTTCTCCTCAGCAAGC
CAATATCTCCTTTTATGGTCTTACTACTCAATACCCTATGTGCTTTTCTTATCAATCACAGCAT
CCTCATTGTATACAGGTGTCAGCTGATATATCCTATCCTCGAGTGACTATTTCAGGCATTGAT
GAAAAAACCGGAAAGAGATCGTACCGTGACGGAACCGGACCCTCGACATTCCGTTTTGTGG
CAAACATTTAAGCATCGGCATAGGAATAGACACTCCTTGGACTTTATGTCGAGCACGAATTG
CATCGGTGTATAACATCAACAATGCCAATACCACCCTTTTATGGGACTGGGCACCTGGAGGA
ACACCTGATTTCCCGAATATCGAGGACAGCATCCACCCATTCTTTCTGTAAACACTGCTCCTA
TATATCAGACAGAACTGTGGAAACTTTTGGCTGCTTTTGGTCATGGTAATAGCCTATATTTAC
AACCCAATATTAGTGGGAGTAAATATGGTGATGTGGGAGTTACAGGATTTTTATATCCCCGA
GCTTGTGTTCCTTATCCATTCATGTTGATACAAGGCCATATGGAAATAACACTGTCATTGAAT
ATTTATCATTTAAATTGTTCTAATTGCATACTTACTAATTGCATTAGAGGTGTAGCCAAAGGA

GAACAAGTTATAATAGTAAAACAACCTCCTTTTGTAATGTTACCTGTTGAAATAACTGAAGA
ATGGTATGATGAGACTGCTTTAGAATTGTTACAACGCATTAATACGGCTCTTAGCCATCCTA
AAAGAGGTCTGAGCCTGATTATTCTGGGTATAGTGTCTTTAATCACCCTTATAGCAACTGCTG
TTACCGCTTCTGTATCTTTAGCACAATCCATTCAAGCTGCTCATACTGTAGATTCCTTGTCAT
ATAATGTTACTAAAGTAATGGGAACTCAAGAAGATATAGATAAAAAATAGAAGATAGATTA
TCAGCTTTACATGATGTAGTTAGAGTTCTAGGAGAACAAGTTCAGAGCATTAATTTTCGCAT
GAAAATTCAATGCCATGCTAATTATAAATGGATTTGTGTTACAAAAAAGCCTTACAATACTT
CTGACTTTCCGTGGGATAAGGTGAAAAAACATCTGCAAGGAAGTTGGTTTAATACTACTGTT
TCTTTAGATCTTTTACAATTGCACAATGAAATTCTTGACATCGAAAATTCTCCAAAAGCTACT
TTGAATATAGCTGATACCGTCGATAATTTTTTACAAAATTTATTTTCTAACTTTCCTAGCCTTC
ATTCACTGTGGCGAAGTATAATTGCTATGGGCGCGGTTCTGACTGTTGTGCTTATCGTAATTT
GTTTAGCTCCTTGCCTTATTCGTAGCATTGTTAAAGAATTTTTACATATGAGAGTTTTAATAC
ATAAAAACATGTTGCAACACCAACATCTTATGGAGCTTTTAAAAAATAAAGAGAGGGGAGC
TGCGGGGGACGACCCGTGAAGGGTTAAGTCTTGGGAGCTCCCTGGCAGGTATGCCGGGCCC
TAGGACACGTGCCTAAGCTCCCTGTCCCGCCACCCTCAAGAACTTTTGTAACCCTTAAGGCT
CCAAGATGTTTGGTTTCGGCAACATTTCATAGAAGATAGATTATCTTATTGTGTATACTTCAT
AGAAGATAGATATTCTGATTGTGTTCTATATACAATGGTAAGGGTCTGGTGATTGTATCCTG
AGATTAAAAACAACCTTGTGAGTGCCTTAAGTTACGTACTTTACCCTATATATACCCCAGCA
CAATAAAGCAAGGTATCAGCCATTTTGGTCTGATCCTCTCAACCCCATCTTTTGTCTATCTCT
TATTTTCTTAGCGGGGACGCTCCGTTCTCTCCCCTGTGCAGGTGCGACTCTTGTTTGTGCTGG
CCGCGGCA

>NC_056054.1:86894211-86902143#SHEEP_RIP_05(+)

CTGCGGGGGACGACCCGTGAAGGGTTAAGTCTTGGGAGCTCCCTGGCAGGTATGCCGGGCC
CTAGGACACGTGCCTAAGCTCCCTGTCCCGCCACCCTCAAGAACTTTTATAACCCTTAAGGC
TCCAAGATGTTTGGTTTCGGCAACATTTCATAGAAGATAGATTATCTTATTGTGTATATTTCA
TAGAAGATAGATATTCTGATTGTGTTCTGTATACAATGGTAAGGGTCTGGTGATTGTATCCTG
AGATTAAAAAACAACCTTGTGAGTGCCTTAAGTCACGTACTTTACCCTATATATACCGCAGC
ACAATAAAGCAAGGTATCAGCCATTTTGGTCTGATCCTCTCAACCCCATCTTTTGTCTCTCTC
TTATTTTCTTAGCGGGGACGCTCCGTTCTCTCCCTGTGCAGGTGTGACTCTTGCTTGTGCTGG
CCGCGGCAGGTGGCGCCCAACGTGGGGCTCGAGCTCGACAGTTCTCCTCGCCACTACTCTTA
TTAATTGAAAAGAGTGAGTATATGAGTACACAAGTGAATTAAATTGAGGAGGAGTAGTAAG
GTATATAGTTGAGAGTATAAATATGGGACAGACACATAGTCGTCAGTTGTTTGTGCATATGT
TATCTGTAATGTTAAAACATAGGGGAATTACTGTTTCTAAACCTAAATTAATCAATTTTCTTT
CATTCATCGAGGAAGTTTGCCCTTGGTTCCCCAGAGAAGGTACAGTAAATTTAGAGACATGG
AAGAAGGTAGGGGAACAAATTCGGACTCATTATACTTTACATGGCCCTGAAAAAATCCCTGT
CGAAACTTTATCCTTTTGGACACTAATTCGTGACTGCCTGGACTTTGATAATGATGAATTAAA
ATGTTTAGGAAATTTATTAAAACAGGAAGAAGATCCTCTCCATGTTCCTGATTCGGAACCCA
GATATGCTGTTCCCGAGTGGGTTAAAAGCGACCCTCCGTTTTCTAACTTATTGCATCCTTCAG
ATAATGATGATTTACTTTCATCCACAGATGAGGCAGAATTAGACGAAGAAGCTGCTAAATAC
CATCAAGAAGATTGGGGTTTTCTAGCACAAGAAAAAGGGGCGTTAACATCTAAAGATGAAT
TGGTTGAATGCTTTAAAAACCTCACTATTGCTTTACAGAACGCAGGAATCAAGCTTCCTAGT
AACAATGCCAAATCTCCTTCTGCTCCGCCTCTTCCCCCTGCTTATGCTCCTTCTGTTGTGGCTG
GTCTCGATCCCCCTCCAGGGCCCCCTCCACCGTCTGAGAACATGTCTCCGCTGCAAAAGGCA
TTGAGACAGGCACAGCGACTTGGTGAGGTTGTCTCTGATTTTTCTCTTGCTTTTCCTGTCTTTG
AAAATAACAACCAGCGTTATTATGAATCACTGCCTTTTAAACAACTGAAAGAGTTAAAGATT
GCTTGCTCACAATACGGTCCTACCGCTCCATTCACCATTGCTATGATAGAAAATTTGGGTACT

CAAGCTTTACCTCCAAATGATTGGAAGCAGACAGCTAGGGCATGTCTCTCAGGGGGAGATTA
TTTATTATGGAAATCTGAATTTTTTGAACAATGTGCTCGTATAGCTGATGTTAACCGACAGCA
AGGTATACAGACCTCCTATGAAATGTTGATTGGTGAAGGCCCTTACCAAGCTACTGACACTC
AACTTAATTTCTTACCTGGTGCATATGCACAAATATCAAATGCGGCTCGGCAGGCATGGAAA
AAACTTCCTAGCTCCAGTACTAAGACAGAGGATCTTTCAAAAGTCCGGCAGGGACCTGATGA
GCCTTACCAGGACTTCGTGGCACGACTTTTAGATACTATAGGTAAGATAATGTCAGATGAAA
AGGCTGGGATGGTATTGGCAAAACAATTGGCTTTTGAAAACGCTAACTCTGCTTGTCAAGCT
GCTTTAAGACCTTATCGAAAAAAGGGAGATCTGTCTGATTTTATTCGCATTTGTGCTGACATT
GGACCCTCCTACATGCAAGGCATTGCTGTGGCAGCAGCATTACAAGGAAAAAGCATAAAAG
AGGTACTTTTCCAGCAGCAAGCCCGGAACAAGAAAGGACTTCAAAAGTCAGGTAATTTGGG
TTGCTTTGTTTGTGGTCAGCCTGGCCATCGGGCTGCAGTGTGCCCTCAAAAACAACAAAGCC
CTGTTAACACTCCTAATTTGTGCCCACGCTGTAAAAAAAGGAAAGCTTTGGGCGCGGGACTG
CCGTTCCAAAACGGATGTTCAAGGTAATCCTTGCCCCCGGTTTCGGGAGAACTGGGTGAGGG
CCAGCCCCTGGCCCCGAAACAATGTTATGGGGCAACACTGCAGGTTCCAAAAGGACCATTG
CAGACCTCTGTCGAGCCACAAGAGGCAGCGCGGGATTGGACCTCTGTGCCACCTCCTACACA
GTATTAACTCCCGAGATGGGGGTCCAAACCCTTGCCACAGGAGTGTTTGGGCCTTTACCTCC
AGGGACAGCTGGACTGCTTTTAGGGCGCAGCAGTGCGTCTTTAAAAGGAATACTTATTCATC
CTGGTGTGATTGACTCTGATTATACAGGAGAGATAAAAATATTAGCCTCCGCTCCTAACAAA
ATTATTGTGATCAATGCAGGACAGCGTATAGCTCAACTTCTTTTAGTTCCATTAGTCATACAG
GGAAAAACAATTAACCGAGACCGTCAAGATAAAGGTTTCGGGTCCTCTGACGCCTATTGGGT
GCAAAATGTTACCGAGGCACGACCAGAACTTGAGCTACGCATTAATGGTAAGCTTTTCCGCG
GAGTGCTTGATACAGGGGCCGATATTAGTGTTATTTCTGATAAATATTGGCCTACTACATGG
CCAAAACAGATGGCTATTTCCACTCTCCAGGGTATTGGCCAAACTACCAATCCAGAACAGAG
TTCATCCCTTCTTACTTGGAAGGATAAAGATGGACATACAGGCCAATTTAAACCTTATATTCT
GCCCTATCTTCCAGTTAATCTATGGGGGCGTGATATATTAAGCAAAATGGGTGTTTATTTATA
TAGTCCTTCACCCACTGTGACAGATTTGATGTTAGATCAGGGCTTACTTCCAAATCAAGGTGT
AGGTAAACAACATCAAGGCATCGTTTTGCCCCTTGATTTAAAATCTAATCAAGATCGAAAAG
GCTTGGGGTGTTTTCCCTAGGGACCTCTGATTCTCCTGTGACACATGCCGATCCTATTGATTG
GAAATCTGAGGAACCGGTATGGGTCGATCAGTGGCCCCTAACACAGGAAAAACTTTCTGCC
GCACAACAGCTGGTGCAGGAACAGCTGAGACTTGGGCATATTGAACCCTCTACCTCTGCTTG
GAATTCCCCAATTTTTGTTATTAAAAAGAAGTCTGGGAAATGGAGATTGCTACAAGATCTTC
GTAAGGTAAATGAAACAATGATGCATATGGGAGCCCTACAACCTGGGTTGCCCACTCCTTCT
GCTATACCTGATAAATCCTATATCATTGTTATAGATTTAAAAGATTGTTTTTACACTATTCCT
CTTGCACCTCAAGATTGCAAAAGATTTGCTTTCAGTTTACCCTCTGTTAATTTTAAAGAGCCT
ATGCAACGCTATCAATGGAGAGTTCTCCCGCAAGGAATGACTAATAGCCCTACGCTGTGCCA
AAAATTTGTTGCTACAGCAATAGCTCCGGTTCGTCAACGTTTTCCTCAGCTATATTTGGTTCA
TTATATGGATGATATATTACTAGCTCATGCTGACGAACATCTATTGTATCAAGCTTTTTCGAT
TCTAAAACAACATTTAAGCCTTAATGGTCTTGTTATTGCTGATGAAAAAATTCAGACTCATTT
TCCTTATAATTATTTGGGTTTCTCCTTATATCCTCGTGTTTATAATACCCAATTAGTACAATTA
CAGACTGACCATTTAAAGACTCTAAATGACTTTCAAAAACTTTTAGGAGACATTAATTGGAT
ACGTCCTTATTTAAAATTACCCACTTATACCTTGCAGCCATTATTTGACATCCTTAAAGGTGA
CTCTGATCCTGCGTCACCCCGAACACTTTCTTTAGAAGGACGAACTGCTTTACAATCAATAG
AAGAAGCTATTAGACAACAACAGATTACTTATTGTGATTACCAACGATCATGGGGTTTGTAT
ATACTTCCTACCCCCCGAGCACCCACAGGGGTTCTCTATCAAGATAAACCTTTGCGATGGAT
ATATTTGTCTGCTACTCCAACTAAACATCTGCTCCCTTACTATGAACTTGTTGCAAAATTGTA
GCAAAGGGACGTCACGAGGCCATCCAATATTTTGGTATGGAACCCCCTTCATTTGTGTTCCTT
ATGCTTTAGAACAACAAGATTGGCTTTTTCAATTTTCAGATAATTGGTCTATAGCTTTGCAAA

TTACCCGGGACGGATTACTCATCATTACCCTTCTGATAAATTGTTACAATTTGCTAGCTCTCA
TGCCTTTATTTTTCCAAAAATAGTTCGCCGACAACCTATTCCCGAAGCGACACTTATATTTAC
AGATGGATCTTCTAATGGAACTGCAGCTTTAATCATTAACCATCAAACCTATTACGCACAAA
CCAGTTTTTCTTCTGCTCAAGTTGTGGAATTATTTGCAGTCCACCAAGCGTTGCTAACTGTAC
CTACTTCCTTCAGTTTATTTACAGACAGCTCCTATGTGGTCGGTGCCTTACAGATGATTGAAA
CTGTTCCAATTATCGGCACCACCTCTCCTGAAGTTCTTAACTTATTTACATTGATTCAACAGG
TTCTCCATTGCCGCCAACACCCCTGTTTCTTTGGACATATTCGTGCACACTCCACCCTTCCTG
GTGCCCTGGTACAAGGCAATCACACTGCGGACGTTCTTACTAAACAAGTGTTTTTCCAATCA
GCTATTGATGCAGCCCGAAAATCCCATGATTTACATCACCAAAATAGTCATTCTTTACGCTTG
CAATTTAAAATTTCCCGTGAAGCTGCACGGCAAATTGTTAAATCTTGCTCTACTTGTCCTCAA
TTCTTTGTTCTCCCTCAATATGGTGTCAACCCTCGAGGTTTACGCCCTAATCACCTCTGGCAA
ACAGATGTTACTCACATTCCTCAATTTGGGCGTCTTAAATATGTTCATGTCTCTATTGACACT
TTTTCCAATTTTCTCATGGCTTCCCTTCACACTGGAGAATCAACACGTCACTGTATTCAACAT
TTGCTGTTTTGCTTTTCTACTTCAGGAATCCCACAAACCCTTAAAACAGATAATGGACCTGGT
TATACTAGCCGTTCTTTTCAACGTTTTTGTCTTTCTTTCCAAATTCATCATAAAACAGGAATTC
CTTATAATCCACAGGGACAAGGTATTGTGGAACGAGCCCATCAACGCCTTAAACATCAATTA
TTAAAACAAAAAAAGGGGAATGAACTGTATAGCCCCTCACCGCATAACGCCTTATGCTCTTT
ATGTTTTAAATTTTTTAACTTTAGACGCAGAAGGCAATTCAGCAGCCCAGCGTTTTTGGGGA
GAACGATCCTCATGCAAAAAACCACTTGTACGATGGAAGGATCCACTTACCAATCTGTGGTA
TGGGCCAGACCCTGTACTAATATGGGGATGAGGGCATGTTTGTGTTTTTCCACAGGATGCCG
AAGCGCCGCGCTGGATTCCGGAAAGGCTGGTACGCGCGGCAGAGGAACTCCCTGACACATC
AAATGCAACGCATGACACTGAGTGAGCCCACGAGTGAGCTGCCTACCCAGAGGCAAATTGA
GGCGCTGATGCGTTATGCTTGGAATGAGGCTCATGTACAACCTCCAGTGACACCTACTAATA
TACTGATCATGTTATTATTATTGTTACAGCGGATACAAAACGGGGCAGCTGCGGCTTTTTGG
GCATACATTCCTGATCCGCCTATGATTCAATCCTTAGGATGGGATAAAGAAACAGTACCTGT
ATATGTTAATGATACAAGTCTTTTAGGAGGAAAATCAGATATTCACATTTCTCCTCAGCAAG
CCAATATCTCCTTTTATGGTCTTACTACTCAATACCCTATGTGCTTTTCTTATCAATCACAGCA
TCCTCATTGTATACAGGTGTCAGCTGATATATCCTATCCTCGAGTGACTATTTCAGGCATTGA
TGAAAAAACCGGAAAGAGATCGTACCGTGACGGAACCGGACCCCTCGACATTCCGTTTTGT
GACAAACATTTAAGCATCGGCATAGGAATAGACACTCCTTGGACTTTATGTCGAGCACGAAT
TGCATCGGTGTATAACATCAACAATGCCAATACCACCCTTTTATGGGACTGGGCACCTGGAG
GAACACCTGATTTCCCGAATATCGAGGACAGCATCCACCCATTCTCTCTGTAAACACTGCTC
CTATATTTCAAACTGAACTGTGGAAACTTTTGGCTGCTTTTGGTCATGGCAATAGTCTATATT
TACAGCCCAATATTAGTGGGAGCAAATATGGTGATGTGGGAGTTACAGGATTTTTATATCCC
CGAGCTTGTGTTCCTTACCCATTCATGTTGATACAAGGCCATATGGAAATAACGCTGTCATTG
AATATTTATCATTTAAATTGTTCTAATTGCATACTTACTAATTGCATTAGAGGTGTAGCCAAA
GGAGAACAAGTTATAATAGTAAAACAACCTGCTTTTGTAATGTTACCTGTTGAAATAACTGA
AGAATGGTATGATGAAACTGCTTTAGAATTGTTACAACGCATTAATACGGCTCTTAGCCGTC
CTAAAAGAGGTCTGAGCCTGATTATTCTGGGTATAGTGTCTTTAATCACCCTTATAGCAACTG
CTGTTACTGCTTCTGTATCTTTAGCACAATCCATTCAAGCTGCTCATACTGTAGATTCCTTGTC
ATATAATGTTACTAAAGTAATGGGAACTCAAGAAGATATAGATAAAAAAATAGAAGATAGA
TTATCAGCTTTATATGATGTAGTTAGAGTTTTAGGAGAACAAGTTCAGAGCATTAATTTTCGC
ATGAAAATTCAATGCCATGCTAATTATAAATGGATTTGTGTTACAAAAAGCCTTACAATACT
TCTGACTTTCCGTGGGATAAGGTGAAAAAACATCTGCAAGGAATTTGGTTTAATACTAATGT
TTCTTTAGATCTTTTACAATTGCACAATGAAATTCTTGACATCGAAAATTCTCCAAAAGCTAC
TTTGAATATAGCTGATACCGTCGATAATTTTTTACAAAATTTATTTTCTAACTTTCCTAGCCTT
CATTCACTGTGGCGAAGTATAATTGCTATGGGCGCGGTTCTGACTGTTGTGCTTATCATAATT

TGTCTAGCTCCTTGCCTTATTCGTAGCATTGTTAAAGAATTTCTACATATGAGAGTTTTAATA
CATAAAAACATGTTGCAACACCAACATCTTATGGAGCTTTTAAAAAATAAAGAGAGGGGAG
CTGCGGGGGACGACCCGTGAAGGGTTAAGTCTTGGGAGCTCCCTGGCAGGTATGCCGGGCC
CTAGGACACGTGCCTAAGCTCCCTGTCCCGCCACCCTCAAGAACTTTTATAACCCTTAAGGC
TCCAAGATGTTTGGTTTCGGCAACATTTCATAGAAGATAGATTATCTTATTGTGTATATTTCA
TAGAAGATAGATATTCTGATTGTGTTCTGTATACAATGGTAAGGGTCTGGTGATTGTATCCTG
AGATTAAAAAACAACCTTGTGAGTGCCTTAAGTCACGTACTTTACCCTATATATACCGCAGC
ACAATAAAGCAAGGTATCAGCCATTTTGGTCTGATCCTCTCAACCCCATCTTTTGTCTATCTC
TTATTTTCTTAGTGGGGACGCTCCGTTCTCTCCCTGTGCAGGTGTGACTCTTGCTTGTGCTGG
CCGCGGCA

>NC_056054.1:91835546-91843550#SHEEP_RIP_06(+)

ACATGTATGCACCTGCGTTGTACTGAAAAGGCTTATTCACGCAGTCTGTAATTCTGCCTAGG
GGGCTTCTATAATAATAAACAGCAATTAGTTTTGCGCAGTTCTGTTCCTCCGGCCGGAGTGT
GTATTGTCTGTCTCTTGTGTGTCTCGTGTTTTGTCTTTGTGTCATTTCGCTCGCAACATCTGGC
ACCCAACGTGGGGCACGAGTGAAACCGAAAGGGTGAGTAACCCCAGGGGAATTTTAAATCC
ATAGCAGGGGAACTTCCGGGAAAATCATGGGGAATTCCTCACCCTAGCAGGGAACTTTCAG
AAAATCATGGGGAAGATGGCAATGACGACCCTGTATGCAAGACAGGGAAAGAGACACAGA
TGTGTATAACGGACTTTTGGACTCAGAGGGAGAGGGAGAGGGTGGGATGATTTGGGAGAAT
GACATTCTAACATGTATACTATCATGTAAGAATTGAATCGCCAGTCTATGTCTGACGCAGGA
TGCAGCATGCTTGGGGGCTGGTGCATGGGGATGACCCAGAGAGATGTTATGGGGAGGGAGG
TGGGAGGGGGTTCATGTTTGGGAACGCATGTAAGAATTGAAGATTTTAAAATTTAAAAAATA
AAAAACTGAAGAAAAAAAAAAAAAAAAGAAAAGAAAATCATGGGGAATTCCTCATCATCAT
TATGGACACAATACATGGAGTTAGTCCAAGGACTTCTCCACTCCATAGGTGTTGAAGCCTCG
ACTCGTCGATTGAGTGAGCTCTTTCGCTTGGTGGAGCAATATTGTCATTGGTTTCAATATCAA
ACTAAGTTACAGTTAAACTTGAAGGAATGGAAAATAATTCAAAAGGAATTGAGAAAGCAAC
ATCAGAAGGGTAATGTGATCCCTCTGAAGTTATGGACTTTGTGTAGTGCTATAACACAAGCT
TTGACCTTGCTCTCTACTGATAATGAAACTAAATCGAATGCTTCAAGGAGGGGAGAAATAAT
TTATGAGGATGTGTCAGACATTGGTGGGGCTTCTGCATCGCCTGAAGGCAAGGATACAAATG
AGCCTCCTCCTGTAAATGGTGAAACATCTGATAGTTCAGAATCAGATTCGGAGGCTTCTTCA
GTTTCGTCAGAGGAGGGCAGAGAGATTAAAGAAATGACCCATCTATTCTGGGAATGGTGGA
AATCCCATAAGGAGGAGAAAAAATCTACACCCTCTGCTCCTCCTTGTGCTTCTCTTTTCCCCA
CTGCGGTACATCGGCCTGATGTGGGAAGGGAACATTGTCGGTTCTCCTTTCCTTTGTCTGTGC
TTCATGATGATGACTTGGCTGCTCCCCCTGGTGGGTTTATCGATCCTCCACAATTGTTTCCAA
TCCAGAGACAGCAAAATGACAATGTGATAAATGTTCAATACACTCCTTTGGAATACAAATTT
TTTTTAAGATTTTAAAGCTGCAGTAGCGCAGTGCGGTCCTCAATCTCCCTTTGTTTTGGCTAT
GCTGGAATCATTGGGAAAAGGCAAATTGATCATTCCATTAGATTGGGAATCTATTGCCCAAG
CTGTGTTGGAGGGTTCTCAATGGTTTCAACTTCGTAGCAGGTGAGAAGAAGCTAGAAAGCAG
GCTTGGATTAATGAGGGACAGAATCCCCCTGGTCCTCTCGAGGACAAGCTAATGGGAGAGG
GCCCTTATTGGGCTTTAAGAGAACAGGCTCAGTACTCTGATCAGGACTTACAACAAGTCCGC
CAGGTCTTTTTACGAGCATGGCGCCGTATGGTGCCTACTGGCCACGCCCGGCCCTCCTTTGTT
AAAACAATGCAAGGCCCCAATGAGCCATATACTGATTTTCTAGCAAGACTGAGGGTAGCTGT
GGAATGGGCTGTAGGGAGGGATGAGATTTCAGAGATATTATTACAAACTTTAGCATTTGAAA
ATGCAAATCCTGAATGCAAGCGTATACTGGGACCTTTAAAGGGACAGGGTGCATCTATAGCT
GAATATATCAGAGCCTGCTCAGGAGGAGGAGGAGCTGAGCATCAGGCTAATGTCTTTGCTA
CAGCCTTGGGCAAAGCTATGAGACCACAAAAGGGAGGTAACTGCTTCCATTGTGGAAAACC
TGGTCATATGAAAAGAGAGTGTCGGAAATTAAAAGCTGATCAAGGTGCAATTCCTAAAGAC

AGATCTCTTGCTGGGATGAATAAGACTCCTCCTGGACTTTGCCGTTGGTTCGGGAAGGGGTT
TCATTGGACTAACGAATGCAGATCTAAAACAGACAAAATGGGCAACCCGATACCAGGAAAC
TATTCTGTGGGCCTGAGTCCTTGGGGCCCAGGAACAATACCGAGGACTTCTCCTCCTTGCCCT
CTTCCCCATCCCATCTGCCCCAACCCTATTCCCTCCCAACAACCGTTAGGAGTCGATGCCCCG
TTAAAAGGACCTCAGATGATGATTTCCGTCTTACGGTCTGCTACTTCAGGGAGCGCTGCTGC
TGATTTGCCACTAGCTGATAATGTTCTTTTGTCACCAGGGGGAGGCATTTATAAATTAAAAA
CAAATGTATTTGGACCACTGCCTAAGGGCACTTTTGGCTTGATATTAGGCCATAGCAGCGCA
GCTTTGAGAGATTTAACCATAATTCCTGGGGTAACAGACTCTGACTATGTTGGGGAAATTTT
AATTATGGTCTCTACTTCTACCACGCTTTCATTGTTAGCTGGGGAACGTACTGCTCAAATACT
TCTCCTACCTTATCACCCCTTTTTGGCTCTTCCTAATGAACGAACAGGAGGATTTGGAAGTAC
TGGGCGACATATATTTTGGGAAATGCTTATCAAAGATTCTTGCCCTGTTCTCTCCTTGATTAT
ACAAGGAGACAACTTTGAGGGACTAGTAGACACAGGAGCGGATGTTTCAGTCATTTCTTCTC
AACAATGGCCCCAAGGTTGGGAAAAAGAAAAAAGCCCTCTAATGCTGACAGGATTGGGCTC
CATTGCAGATGTCTGGAAGAGTACCCATCCCTTGCAACGTCAATTCCATAATGGAAGATCAG
AGTTTGTTACCTTTTATATTGTAAATATACCTATTAATATATGGGGAAGAGATCTTCTCTCTC
CTTTGGGGGCTTCTGTAACCATTCCATCAGAAAACCAGTAGCCGCTGCTCAAATTCCTCGAG
CACTTCCATTAAAATGGTTAACTAATACTCCAAAATGGGTTGAGCAGTGGCCATTACCACAA
ATGAAGCTCGAGGCGTTAGAACAATTAGTACAAGAACAACTCCAATGTGGTCATATAGAGC
ACTCTACCCTCCCCTGGAATTCTCCTGTTTTTGTTATAAAAAAGAAATATGAAAAATGGAGA
ATATTAACCGATTTACAAGAAGTTAATAAATATATTGAACCTATGGGAGCATTACAATTGGG
ACTCCCCTCTCCAGCTCTTATTCCTCAGAATTGGTCCTTAATGGTGCTAGATCTTAAAGACTG
TTTTTTACCATCCCCCTACAATTGCAAGATAGAGATAAATTTGCTTTCACAGTTCCTGTTCTT
AATCATGCTCAGCCTGTTAAGCATTATCAATGGACAGTCTTACCACAAGGAATAATAAATAG
TCCTACCTTATGCCAAGAATTCGTAGCTCGCTCTTTACAATCCCTCCGTCAAGAATACCCCAA
TTATATTCTATATCATTATATGGATGATCTCCTATTAGCAGCTCCTAGTATTGCTGAACGTGA
TGAATTCTTTTTAAAAGTACAGGAGGCTTTAAGACTATACAATTTGCAAATAGCCCCAGAAA
AAATTCAAAAGGACTTTCCTATTTCATATTTAGGGACAATATTGGAACAACATAGAATAAGG
CCCCAAAAGTTGCAAATCAGAAGAGACCACCTCAAAACCTTAAATGATTTTCAAAAGTTATT
GGGAGATATCAATTGGCTACGCCCGGTACTTGGGATTCCTACTTATCAATTACGACATTTGTT
TTCTAATTTAGAAGGAGATACAGCTCTGGATAGCCCCCAGAACTTAACCCCATTGGCTTTAC
AGGAACTTCAATTTGTTGAGCAACGACTAAATGACAGCTTTTTGACTTACTTACATGCGTCTC
AACCTATTTCGTTTATAATATTTCATACCCCTTATACTCCATCTGGTGTAATTGCTCAAGAAA
AAGGATTAACAGAATGGGTTTTCTTACCTAACAGTTTTTCCAAAAAATTGACTACATATATG
GATAAATTAGCCTTCCTTATACAAAAAGGTCGCCATCGTATTTTAAAACTATCAGGATGTGA
ACCACACCAGACTGTTACTCAGTTAACAACTGCTCAAATATCTCGATGTTTACAATTTAATG
AAAACTGGCAAATTTCTCTTGCCTCATATCCTGGTTCGTTTTCTAATCATTATCCATCATCTA
AATTGATTGATTTTCTCTGGACTAATGCTATGATATCTCATTCCCCAATTTCAGATGTTCCAG
TTAAGGGACCCACTATTTTTACGGATGCAAATAAAAACACTGCTGGACATTGGACCCTGGAA
AGTTCCAAGGTTCTCCCCCACTCATTGTCTTCTGTACAGCCCACTGAATTGTGGGCTATCTAT
TTAGTTTTGCAAGATTTTCCCCAACTTCCTATTAACACTGTTTCAGATTCTCGATATGCTGTTC
TCTCTTGCCTCAGCTTCCCCATGTCTCCCTTCCATTGACCCTTAAAACAGCTATTGATAAATT
GTTTTACCAAGTACAACAATTGCTCTTGCAGCATTCAGAGTTAATTTTGTTTACTCACATCCG
TACACATTCTGCCCTTCCTGGACCCTTATCATTCAGAAATGCTACAACTGATGCCTTACTTTA
TCCTATAGAAGCAGCAAAACAAGAACATCTCTTAGAGCATACCAACTCCAAAGGGTTACAA
AAATCTCATGCTATTACTCGAAAACAAGCCCAAAATATTGTTCGTTCTTGTTCCATATGTGTA
CCCTTTGCTTTGCCATTTACCCCACCAGGTGTCAACATGAGAGGACAACAAGCAAATCAGAT
ATGGCAAATGGATGCAATTTACATTTCTTCCTTTGGACAACAAAGATGTGTGCATCGTACTA

TAGACACTTGCACACATTTTCAGTGGGCCACTGCATTACATTCTGAAAAGGCTGACGCTGTT
ATTGCTCATTTGTTCTCTTGTTTTGCAGTTATGGGATTACCAATTGAATTGAAAACTGATAAT
GCACCTGCTTACCAATCCGCAAAATTAGCTCACTTTTTATCTCAATACCATATAACTCATACT
TTTGGTATTCCTTCTAACAGTCAAGGGCAAGCTATCATTGAAAGAGCTAATCGTACCTTGCG
TGATTATCTTGAAAAAATAAAAAGGGGTAACAAGAGAGATTTATGAAACCTAAAGACATTC
TGAATAAAACCTTACTTACCCTAAATTTTTTGAATGTTTGGAGAAAGGGAAATCTATCAGCA
GCAGAGTTGCATTTTCAAGGGAAAGAAGAGGATAAGAAGATTTTGAATACGTCTATCTGGT
ATAAAGATAAAGAGAAAGGTTGGATCCCAGCATCACTAATATATTTGGGACAAGGGTATGC
TTTCATTTCTGTTAATAATTACAGGTTTTGGACCCCAGCAAGATTGATCAAAATCAACAATG
GCTGATCCCTTTGTTCAAAAATTCGAAGAGCTTACTATGCAGAGAGGCTTTACTTTCCGTACA
AGGGAAGCAACACCTCCTACGTGGGGTCAAATGAAGAGGTTGACCCAGGAAGCAGAGAAG
ACATTAATGTAGGCGGGGGCCACCTCTGAATCCTACCAATCTTTTGCTTGCCATGATGGTGG
TGGTGACATGTCAGGTAATCAGTGTATTGGCAAGTAATCATACATACTGGGCATATATACCT
AATCCCCCCAGTAGTAAGAGCAGTAGTAAGTTCCTGGGGGGAACCAGAAGTGCAGGTATGT
ACTAATGAGACTGCCTTCTTTCCCCCACCAGCTTGCGGGGGAATAGAACAACTACCTCATCA
TAAACAACAATATAATATTAGTAATTTGACCATTGCAGTGGAAGGTATTCCTTTGTGTAGAG
GAGGACACCCCTTTTGTCTGTCCACCAAGAAACATCCTCATCATTCCTATAATACATGGGGG
GTAAAGTATAATAATTACCATTTTGCTACTTTTACTGTGCTTGTTTCCACCAGGGGATTTAAC
ACGACAGAACCGACAGGCATTCATAATGGAAAACACATGTCGCTATGTCCTGTTAACTTTTT
TGCTCCTTCTCTAGAATCTTTGGAGTGGGAACATTGCCGAGGTCATCGACCCTTTAAGGTCAT
GAATTATTCTGGGGCCATCATTGTAGATTGGAGTCCAGATCATGGGCAATTCTTGGAAAAAT
GGTCAAATAAATCTCTTAGGTGGCATCGTGCAAATAGCACTTTGATGGGTAAAGGTAATGGA
ACAGTTAAATGGCAGCAATTTGCACTTGTCCCTCCTCAATTACAATTGCAAAGATATCCGCA
CATTCAAGGGGATATTTGGAAACTCTGGGCAGTTTCTGGTAATCTCACTGTCTGGTCAGGAA
ACTATACTTTGGATAGTGGTGACTCTTCAGTTCCTTTCCATGTTGATTTACATGTTAATAAAC
CTTATTCCACAATGGCATGTGTAAAATATCCTTTTGCATTGTTATTTGGGAATTGGACCTGGA
ATGACACTGTGGGGTCTGTGTCATGTGATTACTGTAATCTAACTCAATGTGTAAATCAGTCTT
GGTGGGAAGAATTTGAAAGACGAGCCTATAACTCCAATTTCTTGTTAGTAATTGTTAAGGCT
CAGACAGAAGTATGGTTACCTATAAATCTGACTCGGCCATGGTCAGATTCTTTTGCTGTTTCT
CATCTAGTAACCGCTGTACAGACTTTGCTACACCGATCTCAACGTATGCTTGGTGTGGTAATT
GCTTCGATTCTAGCAGTCGCATCAGTAACTGCAACAGCAGCGATAGCAGGCCTTGCGTTACA
CCAAGGAATTCAAACAGCTGATTTTGTTCGGGACTGGCATAAAGACTCTCATTTGTTATGGC
AACAACAGCAAGATCTGGATGCACAACTTGCTACTGACGTGCTCAATCTTCAACACACCGTT
TCCTGGCTTGGAGATCAATTGGCTGTTTTATCTACACGAAGCGTGTTGAAATGTGATTGGAA
TTCTTCTCAGTTTTGTATAACACCTGTACCATTTAACATGAGTGAAGGATGGGATAAAGTAA
AACGATCCTTGACTGGGCATCAAAATCTCACTACGGAGATTATGGACCTGGAACAACAAATT
TTGTCTACTTTTAGCAGGACTTTACCTGACATTACAGGGTCTGATTTGCTGAAAAGTCTTCAA
GAGGGAATGAATAACTTAAATCCATTAGGGCATGTATCCTCACTAATTGGGACTACCTTTGG
GAACACTGTGTTTCTATTACTTTTATGTTGTGTTGCTTTTCTAGTCTTCCGGCGGTGGCGGAA
AGGGAAACAACTAAAGCGCGAAGCAGAGAAGATCCAGACCATGCTACAATTTATAAAAGCA
AATAAAAAAGGGGGAGATGAAGGGTTAATAGGGTAGCAGAGATGTGCCTGCAAACGGGCC
TCTCTGCTCGGGCTGGACGTCCTTACAAACAAGGCGTTCTGCCAAAGAGTCTGGACACAGCC
TTGAGTTTAATGGTCCCTTGCAAATGAGGGAGCATTCCCTTCTTGTGATAAGAAGGAAAAGA
GGGCTTTGGACAGACTCTGCAGTAGACAGGGATTTCACTCCCCTTTGCTGTACGATAACATG
TATGCACCTGCGCTGTACTGAAAAGGCTTATTCACGCAGTCTGGAATTCTGCCTAGGGGGCT
TCTATAATAATAAACAGCAATTAGTTTTGCGCAGTTCTGTTCCTCCGGCCGGAGTGTGTATTG
TCTGTCTCTTGTGTGTCTCATGCTTTGTCTTTGTGTCATTTCGCTCGCAACA

>NC_056054.1:98025573-98033196#SHEEP_RIP_07(+)

TGAAGGGTTAATAGGGTAGCAGAGATGTGCCTGCAAACGGGTCTCTCTGCTCATGCTGAGCG
TCCTTGCATACGAGGCTTTCTGCCAAAGAGTCTGGATACAGCCTTGAGTTTAATAGTCCCTTG
CAAACGAGGGAGCATTCCCTTCTTGAGATAAGAGGGAGATGAGGGCTTTGTGCAGACTCTG
CAGTAGACAGAGATTTCACTCCCCTTTGCTGTACGATAACATGTATGCACCTGCACTGTGCT
GAAAAGGCTTATTCTTACAGTCTGGAATTCTGCCTAAGGAGGGCTTTATAATAATAAACGGC
AATTAGTTTGCCCAGTTCTGTTCCTCTGGCCAGAGTGGTGTCCTGTCTGTCTCTTGTGTGTCTT
GTATGTTCTGTGTCATTTCACTCGTAGTAACCAACATCTGGCGCCCAACGTGGGGCTCAAGT
GAAACCGAAAGAGGTAAGAAACCCCGGGGGGGTTTTAGATCCATAGCACGGGAGCTTTCGG
AAACATGGGAATTCCTCACCCTAGCGGAAAAAATTGCTAGGTCAATTCTTGTGCATAATCAT
GGGGAATTCTTCACCCTAGCAGATGGGAACTTTCAAAAGTTCATGGTTCAAAAAACCATGGG
GAAATCCTGCTAGGAAGAAATAAATGTTCATTACGGGCACAATATGTGAATTTATATATTGA
ATCATGTTATAGAATTTATGTAATGCTATAACACAGGCTTTGACTTTGCTATCTACTGATAAT
GAGACTAAATCTAATGCTTTAATGAAGGGAGAGGCAATTTATGAGGATATGCCAAATGTTG
GTGGAATTTCTGCCTTGCCTGAAAGTAAGGATACAAATAAGCCTCCTTCTGGAAATGGTGAA
ACATCTGATAGTTCAGAATCAGATTCGGAGGCTTCTTCGGTTTCGTCAGAGGAGGGCAAAGA
GATTAAAGAAATGACCCATCTATTCCGGGAATAGTGGAAATCCCGTAAGGAGGAGAAAAAA
TCTACACCTTCTGCTCCTGCTTGTGCTTCTCTTCTCCCCACTGCAGTTGATCGGCCCGATGTGG
GCAGGGGACATTGTCCGTTCTCCTTTCCTTTGTCTATGCTTCACGATGATGACTTGTCTGCTC
CCCCTGGTGGGTTTATTGATCTTCCACAATTGTTTCCCATCCAGAGACAGCAGGATGGCAAT
GTGATGAATGTTCAATATGCTCCTTTGGAATATAAATTTTTTAAAGATCTTAAAGCTGCAATA
GCGCAATATGGTCCTCAATCTCCCTTTGTTTTGGCTATGCTGGAATCATTGGGAAAAGGCAA
ACTAATCATTCCATTAGATTGGGAATCCATTGCCCAAGCTGTCTTGGAGGGTTCTCAATGGTT
GCAACTTCGTAGCTGGTGGGAGGAAGAAGCTAGGAAGCAAGCTTGGATTAATGAGGGACAA
AATCCCCCTGGTCCTCTCGAGGACAAATTAATGGGAGAGGGCCAATATCAGGCTTTAAGAG
AACAGGCTCAATACTCTGACCAGGACTTACAACAAGTTCACCAGGTCTTTTTACGAGCATGG
TGCCGTGTGGTGCCTACTGGCCAAGCCCAGCCCTCCTTTGTTAAAACAATGCAAGGCCCTAA
TGAGCCATATACTGATTTTCTAGCAAGATTGAGGGTAACTGTGGAACGGGCTGTAGGGAGG
GATGAAATCTCAGGGATATTATTACAAACTTTGGCATTTGAAAATACAAATCCTGAATGCAA
GCGTATACTGGGATTTTAAAGGGACAGGGTGCCTCTATAGCTGAATATATCAGAGCCTGCTC
TGGAGTAGGAAGAACTGAGCACCAGGCTAATGTCTTTGCTACGGCCTTGGCCAAGGTTATAA
GACCACCAAAGGGAGGTAACTGCTTTCATTGTGGAAAACCTGGTCATATGAAAAAAGAGTG
TCAGAAATTAAAAGCTGATAAAGACAGATCTCTTGCTAGGAAGAATAAGGCTCCTCCTGGA
CTTTGCCGTCGGTGAAGGAGGGGATTTCATTGGACTAATAAATGCAAATCTAAAACAGACA
AAATGGGCAACCCGATACCGGGAAACTATCCTTCGGGCCTGAGTCCTTGGGGCCCAGGAAC
AATACCGGGGACTTCTCCTCCTTGCCCTCCTCCCATTCCATCTGCCCCGTTAAAAAGACCTCA
GATGATGATTTCGGACTTACGGTCTGCTACTTCAGGGAGTGCTGCTGCTGATTTGTCACTAGC
TGATAATGTTCTTTTGTCACCGAGGGGAGGCATTTATAAATTAAAAACAAATGTATTTGTAC
CACTGCCTAAAGGCACTTTTGGCTTAATATTAGGCCGTAGCAGCGCGGCTTTGAGAGGTTTA
ACCATAATTCCTGGGGTAATAGACTCTGATTATGTTGGGGAAATTTTAATTATGGTCTCTACT
TCTACCACACTTTCATTGTTAGCTAGAGAATGTATTGCCCAAATACTTCTCCTACCTTATCAC
CCCTTTTTGGCTCTTCCTAATAAACAAACAGGGGGATTTGGAAGTACTGGGCGAAATATATT
TGGGGAAATGCTTATCAGAGATTCTCACCCTGTTCTCCCCTTGATTATACAAGGAAACAACT
TTGAGGGACTAGTAGATACAGGGGCAGATGTTTCAATCATTTCTTCTCAGCAATGGCCCCAA
GGTTGGGAAAAGGAAAAAAGCCCTTTAATGCTGACGGGATTGGGCTCCATTGCAGATATTTG
GAAGAGTACCCATCCCTTGCAATGTCAATTCCATAATGGAAGATCAGTGTTTGTTACCTTTTA

TATTGTAAATATACCTATTAATATATGGGAGAGAGATCTCCTCTCTCCTTTGGGGGCTTCTGT
AACCATTCCATTGGAAAACTAGTGGCCACTGCTCAAATTCCTTGAGCACTCCCATTAAAATG
GTTAACTAATACTCCTAAATGGGTTGAGCAGTGGCCATTACCACAAATGAAGCTCGAGGCAT
TAGAACAAGTAGTACAAGAACAACTCCAACTTGGTCATATAGAGCCCTCTACCTCACCCTAA
AATTCTCCTGTTTTTGTTATTTAAAAAAATCTAAAAAATAAAAAATGTTCACTGATTCACAAG
AAGTTAATAAATGTATTGAACCTATGGGAGCATTGCAGTTGGGACTCCCCTCTCCAGCTCTT
ATTCCTCAAAATTGGTCCTTAATGGTGCTAGATCTTAAAGACTGTGTTTTTTTACCATTCCCC
TACAATTTCAAGATAGAAATAAATTTGCTTTTACAGTTCCTGTTCTTAATCATGCTCAACCTG
TTAAGCGTTATCAATGGACAGTCCTACCGCAAGGAATGATAAATAGTCCTACCTTATGTCAA
GAATTTGTAGCTCACTCTTTGCAATCTCTCCGCCAAGAATACCCTAATTATATTCTATATCAT
TATATGGATGATCTCCTATTGGCAGCTCCTAGTATTGCTGAACGTGACGAATTCTTTTTAAAA
GTACAAGAGGCTTTAAGACTACACAATTTGCAAATAGCCCCAGAAAAAATTCAAAAGGACT
TTCCTATTTCATATTTAGGGACAATATTGGAACAACATAGAATTAAGCCCCAAAAGTCACAA
ATTAGAAGAGACCATCTCAGAACCTTAAATGATTTTCAAAAATTATTGGGAGATATTAATTG
GCTACGCCCGGTACTTGGGATTCCTACTTATCAGTTATGACATTTGTTTTCTACTTTAGAAGG
AGATACAGCTTTCGATAGCCCCCGGACCTTAATCCCATTGGCTTTACAGGAACTTCAACTTGT
TGAACAACGACTGAATGACAGCTTTTTGACTGACTTACATGCATCTCAACCTATTTCTTTTAT
CATATTTCATACCCCTTATTCCCCATCTGGTGTAATTGCTCAAGAAGAAGGATTAATAGAAT
GCATTTTCTTACCTAACAGTTTTTCCAAAAAATTGACTATATATATGGATAAATTAGCCTTCC
TTATACAGAGAAGTCACCATCATATTTTACAATTATCAGGATGTGAACCACACCAGATTGTT
ACTCAGTTAACAACTGCTCAAATATCTCGATGTTTACAATTTAATGAAAACTGGCAAATTTCT
CTTGCTTCATATCCTGGTTCGTTTTCTAATCATTATCCATCATCTAAATTGATTGATTTTCTCC
AGACTAACACTATGATATCTCATTCCCCAATTTCAGATGTTCCAGTTAAAGGACCTACTATTT
TTACAGATGCAAATAAAAATACTGCTAGATATTGGACCCCAGAAAATTCCAAGGTTCTCCCC
CACTCATTTTCTTCTGTACAGCCTGCTGAATTGTGGGCTATCTAGTTTTACAAGATTTTCCCC
AACTTCCTATTAACATTGTTTCAGATTCTCGATATGCTGTTCTCTCTTGCCTACAGCTTCCTCA
TGTCTCCCTTCCACTGACTCTTAAAACAGCTATTGATAAATTGTTTTACCAAGTACAACAATT
ACTCTTGCAACTTTCAGAGTTAATTTTCTTTACTCACATCCATGCACATTCTGCCCTTCCTGGA
CCCTTATCATTTGGAAATGCTACAATTGATGCCTTACTTAATCCTATAGAAGCAGCAAAGCA
AAAACATCTCTTACAACATACCAACTCCAAAGGGTTAGAAAAATCTCACACTATTACTCAAA
AACAAGCTCAAAATATTGTTCGTTCTTGTTCCATATGTGCACCCTTTGCTTTGCCATTTACCTC
ACCAGGTGTCAACATGAGAGGACAACAAGAAAATCAGATATGGCAAATGAATGTAATTTAC
ATTTCTTCCTTCGGACAACAAAAATGTGTGGGCAACTGCATTACATTCTAAAAAGGCTGACG
CTGAGCTGTTATTACTCATTTGTTATCTTGTTTTGCAGTTATGGGATTACCAATTGAATTGAA
AACTGATAATGCACCTGCTTACCAATCCGCAAAATTAGCTCACTTTTTATCTCAATACCATAT
AACTCATACTTTTGGTAATCCTTATAATAGTCAAGGGCAAGCTATTATTGAAAGAGCTAATC
ATACCTTGCATGATTATCTTGAAAAAATAAAAAAGGGGGAACAAGAGAGATTTATGAAACC
TAAAGACATTCTGAATAAAACCTTACTTACCCTAAATTTTTTGAATGTTTGGAGCAGGGGAA
ATCTATCAGCAGCAGAGTTGCACTTTCAAGGGAAAGAAGAGGATAAGAAGATCTTGAATAC
ACCTATTTGGTATAAAGATAAAGAGAACGGCTGGATCCCAGCATCATTAATATATTTGGGGC
AAGGGTATGCTTTCATTTCTGTTGATAATTACAGGTTTTGGACCCCAGCAAGATTGATCAAA
ATCAGCAATGGTTAATCCTCTTGTTCAGAAATTTGAAGAGCTTACTATGCAGAGAAGCCTTA
CCTCCCATACAAGGGAAGCAACACCTCCTACGTGGGGTCAAATGAAGAGGTTGACCCAGGA
AGCAGAGAAGACATTAATGAAGGTGGGGCAACCTCTGAATCCTACCAATCTTTTGCTTGCCA
CGATGGTGGTGGTGACATGTCAGGTAATCGGCGTATCAGCAAGTAATTATACATATTGGGCA
TATATACCTAATCCCCCATTAGTAAGAGCAGTTTCCTGGGGGGAACCAGAAGTGCAGGTATG
TACTAATGAGACTGCCTTCTTTCCCCTGCCAGCTTGCAGGGGAATAGAACAACTATATCATC

ATAAACAACAATATAATATTAGTAATTTGACCACTGCGGTGGAAGGTATTCCTTTATGTATA
GGGGGACACCCCTTTTGTCTGTCCACCAAGGAACATTCTCATCATTCTTATAATACATGGGG
GGTAAAGTATAATAATTACCATTTTGCTACTTTTACTGTGCTTGTTTCCACCAGGGGATTTAG
TACCTCGACAGAACCGATAGACATTCATAATGGAAAACACATGTCACTATGTCCTGTTAACC
TTTTCGTTCCTTCTCTAGAATCTTTGGAGTGGGAACGTTGCCGAGGCCATCCACCCTTTAAGG
TCATGAATTATTCTGGGGCCATCATTGTAGATTGGAGTCCAGATCATGGACAATTCTTAGAA
AAATGGTCAAATAAACCTTTTAGGTGGCATCGCGCAAATACCACTTTGATGGGCAATGGTAA
CGAAACAGTTAAATGGCAGCAATTTGCACTTGTCCCTCCTCAATTACAATTGCAAGGATATC
CTCACATTCAAGGAGATATTTGGAAACTATGGGCGGTTTCTGGTAATCTCACTATCTGGTCA
GGAAATTATACTTTGGACAGTGGTGACTCTTCGGGTTCATTCCATGTTAATTTACATGTTAAT
AAATCTTATTCCACAATGGCATGTGTAAAATATCCTTTTGCATTGTTATATAGGAATTGGACC
TGGAATGATACTCTGGAGTCTGTGTCAAGTAACTATTGTAATTTAACTCAATGTATAAATGTC
TTGGTGGGAAGAATTTGAAAGACAAGCCTTTAACTCCAATTTCTCGCTAGTAATTGTTAAAG
CTCAGACAGAATTATGGTTGTCTATAAATCTGACTCAGCCGTGGTCAGATCCTTTTGCTGCTT
CTCATCTAGTAACCGCTGTACAGACTTTGCTACACCGATCTCGACGTATGCTTGCTGTGGTCA
TTGCTTCGATTCTCGCAGTCGCATCAGTAACTGAAACAGCAGCTGTAGCAGGCCTTGCGTTA
CACTAAGGAATTCAAACAGCTGATTTTGTTCGGGACTGGCAAAAGGACTCTCATTTGTTATG
GCAACAACAGCAAGATTTGGATGCACAACTTGCTACCGACGTGCTTAATCTTCAAACACACC
GTTTCCTGGCTTGGAGATCAATTGGCTGTTTTATCTACATGAAGTGTGTTGAAATGTGATTGG
AATTCTTCTGTTTTGTATAACACCTGTACCATTTAACATGAGCCCTTCAAGAGATTAACCGAG
ACATATAAATAAAAAGAGTTCTAGATGCCCATCATCTAGAGAGATTCCGCCTAGCCCCTAAG
AGGTGGCTGGATAGCCAATGACGGGTAAGACCCTCAGAGGAGGGCAATCTAAGACAGGCAC
AGCCGCAAGAGGGGCTGGCGAGGCTGGAGGTTGGCCGCCTACAGCTTTATGCCTTGCTCTAC
AAAACATCAATACAAATGTCTCGAGGGCTTGAATACAATAAAAAAGGGGGAGATGAAGGGT
TAATAGGGTAGCAGAGATGTGCCTGCAAATGGGTCTCTCTGCTCATGCTGAGCGTCCTTGCA
TACGAGGCGTTCTGCCAAAGAGTCTGGATACAGCCTTGAGTTTAATAGTCCCTTGCAAACGA
GGGAGCATTCCCTTCTTGAGATAAGAGGGAGATGAGGGCTTTGTGCAGACTCTGCAGTAGAC
AGAGATTTCACTCCCCTTTGCTGTACGATAACATGTATGCACCTGCACTGTACTGAAAAGGC
TTATTCTTACAGTCTGGAACTCTGCCTAAGGAGGGCTTTATAATAATAAACGGCAATTAGTTT
GCCCAGTTCTGTTCCTCTGGCCAGAGTGGTGTCCTGTCTGTCTCTTGTGTGTCTTGTATGTTCT
GTGTCATTTCACTCGTAGTAACCAACA

>NC_056055.1:114718350-114726241#SHEEP_RIP_08(-)

GAAGGGTTAATAGAGTAGCAGAGATGTGCCTGCAAATGGGCCTCTCTGCTAGGGTTGGACG
TCCTTGCAAACGAGGTGTTCTGCCAAAGAGTCTGGACACAGCCTTGAGTTTAATGGTCCCTT
GCAAACGAGGTAGCTTTCCCTTCTTGTGATAAAGAGGGAATAGAGGGCTTTGTACAGACTCT
GCAGTAGACTAGTATTTTACTCCCCTTTGCTATACGATAACATATATGCACCTGCGCTGTGCT
GAAAAGGCTTATTCATGCAGTCTGGAATTCTGCCTAGGGGGCTTTTATAATAAATGGCAATT
AGTTTTTTGCCCAGTTCTGCTCCTCCAGCTGGAGTGTGCGTGTTGTCTGTCTCTTGTGTGTATC
TTGTTCTGTGTCATTTCACTCGTAATTTCCAACATCTGGCGCCCAACGTGGGGCTTGAGTGAA
ACCGAAAGGGTGAGTAACCCCGGGGGGATTTAAATCCATAGCAGGGGAACTTTCGGGAAA
ATTACGGGGAATTCCTCACCCTAGCAGTCGGGAGCTTTCAAAAAAATCATGGGGAATTCCTC
ATCATCATTACAGGCACAATACATGGAGTTAGTCCAAGGACTTCTCCACTCCATAGGCGTTA
AAGCCTCAACTCGTCGGTTGAGTGAGCTCTTTCACTTGGTGGAGCAATATTGTCATTGGTTTC
AATATCAAACTAAGTTACAGTTAAACTTGAAGGAATGGAAAATAATTCAAAAGGAATTGAG
AAAGCAACATCAGAAGGGTAATGTGATCCCTTTGAAGTTGTGGACTTTGTGTAGTGCTATAA
CACAGGCTTTGACCTTGCTTTCTACTGAGAGTGAAACTAAATCTAATGCTTCAGGGAGGGGA

GAAATAATTTATGAGGATGTGGCAGACGTTGGTGGGGCTTCTGCATCGCCTGAAGGCAAGG
ATACAAATGAGCCTCCTCCTGTAAATGGTGAAACATCTGATAGTTCAGAATCAGATTCGGAG
GCTTCTTCAGTTTTGTCAGAGGAGAGCAAAGAGATTAAAGAAATGACCCATCTATTCCAGGA
ATGGTGGAAATCCCGTAAGAAGGAGAAAAAATCTACACCTTCTGCTCCTCCTTGTGCTTCTC
TTTTCCCCACTGCAGTTAATCGGCCCGATGTGGGCAGGGAACATTGTCGGTTCTCCTTTCCTT
TGTCTATGCCTCATGATGATGACTTGTCTGCTCCGCCTGGTGGGTATATCGATCCTCCACAAT
TGTTTCCCATCCAGAGACAGCAGGATGGCAATGTGATGTTCAATATGCTCCTTTGGAATATA
AATTATTTAAAGATCTTAAAACTGCAGTAGCGCAGTACGGTACTCAATCTTCCTTTGTTTTGG
CTATGCTGGAATCATTGGGAAAAGGCAAATTAATCATTCCATTAGACTGGGAATCTATTGCC
CAAGCTGTCTTGGAGGGTTCTCAGTGGTTGCAACTTTGTAGCTAGTGGGAAGAAGAAGCTAG
AAAGCAGGCTCGGATTAATGAGGGACAGAATCCCCCTGGTCCTCTCGAGGACAAGGTAATG
GGAGAGGGCCAATATTGGGCTTTAAGAGAACAGGCTCAATACTCTGATCAGGACTTACAAC
AAGTCCACCAGGTCTTTTTACGAGCATGGCGCCGGGAGGTGCCTACTGGCCACGCCCAGCCC
TCCTTTGTTAAAACAATGCAAGGCCCCAATGAACCATATATTGATTTTCTAGCAAGATTGAG
GGTAGCTATGGAACAGGCTGTAGGGAGGGATGAGATTTCAGAGATATTATTACAAACTTTA
GCATTTGAAAATGCAAATCCTGAATGCAAGCGTATACTGGGAACTTTAAAGGGACAGGGTA
CATCTATAGCTGAATATATCAGAGCCTGCTCGGGAATAGGAGGGACTGAGCATCAGGCTAA
TATCTTTGCTCCAGCCTTGGCCAAACTTATGAGACCACCAAACGGAGGTAACTGCTTTCATT
GTGGAAAACCCGGTCATATGAAAAGAGAGTGTCAGAAATTAAAAGCTGATCAAGGTGCAAT
TCCTAAAGACAGATCTCTTGCTGGGAAGAATAAGACTCCTCCTGGACTTTGCCATCGGTGCG
GGAAGGGGTTTATTGGACTAATGAATGCAGATCTAAAACAGACAAAATGGGCAACCCGAT
ACCGGGAAACTATCCTGCGGGCCTAAGTCCTTGGGGCCCAGGAACAATACCGGGGACTTCTC
CTTGCCCTCCCACTTCTCCTCCCATCCCATCTGCCCCAGCCCTATTCCCTCCCAACAACCGTT
ACGAGTCGATGCCCCGTTAAAAGGGCCTCAGATGATGATTTCGGACTTACGGTTTACTACTT
CAGGGAGTGCTGCTGCTGATTTGCCACTAGCTGATAATGTTCGTCACCAGGGGGAGGCATTT
ATAAATTAAAAACAAATGTATTTGGACCACTGCCTAAAGGCACTTTTGGCTTGATATTAGGC
CGTAGCAGTGTGGCTTTGAGAGGTCTAACCATAATTCCTGGGGTAATAGACTCCGACTATGT
TGGGGAAATTTTGATTATGGTCTCTACTTCTACCACACTTTCACTGTTAGCTGGGGAACGTAT
TGCTCAAATACTTCTCCTACCTTATCACCCCTTTTTGGCTCTTCCTAATGAACGAACAGGAGG
ATTTGGAAGTACTGGGCGACATATATTTTGGGAAATGCTTATCAAAGATTCCCACCCTGTTCT
CTCCTTGATTATACAGGGAAACAACTTTGAAGGACTAGTAGACACAGGGGCGGATGTTTCAG
TCATTTCTTCTCAACAATGGCCCCAAGATTGGGAAAAAGGAAAAAGCCCTTTAATGCTGACA
GGATTAGGCTTCATTGCAGATGTCTGGAAGAGTACCCATCCCTTGCAATGTCAATTCCATAA
TAGAAGGTCAGTGTTTGTTACCTTTAATATTGCAAATATACCTATTAATATATGGGAAAGAG
CTCTTCTCTCTCCTTTGGGGGCTTCTGCAACCATTCCACTGGAAAACTAGTAGCCACTGCTCA
AATTCCGCGAGCACTCCCATTAAAATGGTTAACTAATACTCCAAAATCGGTTGAGCAGTGGC
CATTACCACAAATGAAGCTCGAGGCATTAGAACAATTAGTACAAGAACAACTCCAACTTGG
TCATATAGAGCCCTCTACCTCTCCCTGGAATTCTCCTGTTTTTGTTATAAAAAAGAAATCTGG
AAAATGGAGAATGTTAACTGATTTACGAGAAATTAATAAATGTATTGAACCTATGGGAGCCT
TGCAACTGGGACTCCCCTCTCCAGCTCTTATTCCTCAGAATGGGTCCTTAATGGTGTTAGATC
TTAAAGACTGTTTTTTTTTTTTTTTTACCATTCCCCTACAATTGCAAGATAGAGATAAATTTGC
TTTTACAGTTCCTGTTCTTAATCATGCTCAGCCTGTTTAAGCGTTATCAATGGACAGTCTTAC
CACAAGGAATGATAAATAGTCCTACCTTATGCCAAGAATTCGTAGCTCGCTCTTTACAATCC
CTCCGTCAAGAATACCCCAATTATATTCTATATCATTATATGGATGATCTCCTACTGGCAGCT
CCTAGTATTGCTGAATGTGATGAATTCTTTTTAAAAGTACAGCAGGCTTTAAGACTATACAA
TTTTCAAATAGCCCCGGAAAAAATTCAAAAGGACTTTCCTATTTCATATTTAGGGACAATAT
TGGAACAACATAGAATTAGGCCCCAAAATTTGCAAATTAGAAGAGACCATCTCAAAACCTT

AAATGATTTTCAAAAGTTATTTGGAGATATTAATTGGCTACGCCCAGCACTTGGGATTCCTA
CTTATCAATTACGACATTTGTTTTCTACTTTAGAAGGAGATACAGCTCTGGATAGCCCCCGGA
CCTTAACCCCATTGGCTTTACAGGAACTTCAATTTGTTGAGCAATGACTAAATGACAGCTTTT
TGACTTACTTACATGCATTTCAAACTATTTCGTTTATAATATTTCATACCTCTTATTCCCCATC
TGGTGTAATTGCTCAAGAAAAAGGATTAATAGAATGGGTTTTCTTACCTAACAGTTTTACCA
AAAAATTGACTATATATATGGATAAATTAGCCTTCCTTATACAGAAGGGTTGCCATCGTATT
TTACAATTATCAGGATCTGAACCACACCAGATTGTTACTCAGCTAACAACTGCTCAAATATC
TCGATGTTTACAATTTAATGAAAACTGGCAAATTTCTCTTGCCTCATATCCTGGTTCGTTTTCT
AATCATTATCCATCATCTAAATTGATTGATTTTCTCCAGACTAACACTATGATATCTCATTCC
CCAATTTCAGATGTTCCAGTTAAGGGATCCACTATTTTTACAGATGAAAATAAAAATACTGC
TGGATATTGGACCCCGGAAAATTCCAAGGTTCTCCCCCACTCATTTTCTTCTGTACAGCCCGC
TGAATTGTGGGCTATCTATTTAGTTTTGCAAGATTTTCCCCAACTTCCTATTAACATTGTTTCA
GATTCTCGATATGCTGTTCTCTCTTGCCTACAGCTTCCCCATGTCTCTCTTCCATTGACTCTCA
AAACAGTTATTGATAAATTGTTTTACCAAGTACAACAATTGCTCTTGCAGCGTTTCTTTACTC
ACATCCGTGCACATTCTGCCCTTCCTGGACCCTTATCATTCGGAAATGCTACAATTGATGCCT
TACTTTATCCTATAGAAGCAGCAAAACAAGAACATCTCTTACAACATACCAACTCCAAAGGG
TTACAAAAATCTCATGCTATTACTCGAAAACAAGCTCAAAATATTGTTCGTTCTTGTTCCATA
TGTGCACCCTTTGCTTTGCCATTTACCCCACCAGGTGTCAACATAAGAGGACTACAAGCAAA
TCAGATACGGCAAATGGATGTAATTTACATTTCTTCCTTCGGACAACAAAAATGTGTGCATC
ATACTATAGATACTTGCACACATTTTCAATGGGCCACTGCATTACATTCTGAAAAGGCTGAC
GCTGTTATTACTCATTTGTTATCTTGTTTTGCAGTTATGGGATTACCAATTGAATTGAAAACT
GATAATGCACCTGCTTACCAATCCGCAAAATTAGCTCACTTGTTATCTCAATACCATATAACT
CATACTTTTGGTATTCCTTATAATAGTCAAGAGCAAGCTATCATTGAAAGAGCTAATCGTAC
CTTGCATGATTATCTTGAAAAAATACAAAAGGGGGAACAGAGATTTATGAAACCTAAAGAC
ATTCTGAATAAAACCTTACTTACCCTAATTTTTTTTAATGTTTGGAGCAAGGGAAATCTATCA
GCAGCAGAGTTGCATTTTCAAGGGAAAGAAGAGGATAAGAAGATCTTGAATACGCCTATTT
GGTATAAAGATAAAGAGAAGGGTTGGATCCCAGCATCATTAATATATTTGGGACGAGGGTA
TGCTTTCATTTCTGTTGATAATTACAGGTTTTGGACCCCAACAAGATTGATCAAAATCAACAA
TGGCTGATCCCCTTGTTCAAAAATTCGAAGAGCTTACTATACCGAGAAGCCTTATTTCCCGTA
CAAGGGAAGCAACACCTCCTATATGGGGTCAAATGAAGAGGTTGACCCAGGAAGCAGAGAA
GACGTTAATGAGGGCGGGGCAACCTCTGAATCCTACCAATCTTTTGCTTGCCATGATGGCGG
TGGTGACATGTCAGGTAATCAGTGTATCGGCAAGTAATCATACATATTGGGCATATATACCT
AATCCCCCATTAGTAAGAGCAGTTTCCTGTGGGGAACCAGAAGTGCAGGTATGTACTAATGA
GACTGCCTTCCTTCCCCCACCAGCTTGCAGGGGAATAGAACAACTATCTCATCATAAACAAC
AATATAATATTAGTAATTTGACCATTGCAGTGGAAGGTATTCCTTTATGTATAGGGGGACAC
CCCTTTTGTCTGTCCACCAAGGAACATTCTCATCATTCTTAAAATACATGGGGGGTAAAGTAT
AATAATTACCATTTTGCTACTTTTACTGTGCTTGTTTCCACCAGGGGATTTAGCACCTCGACA
AAACTGATAGATATTCATAATGGAAAACACATGTCACTATGTCCTGTTAACTTTTTTGTTCCT
TCTCTAGAATCTTTGGAGTGGGAATGTTGCTGAGGTCATCGACCCTTTAAAGTCATGAATTAT
TCTGGGGCCATCATTGTAGATTGGAGTCCAGATCATGGGCAATTCTTAGAAAAATGGTCAAA
TAAATCTTTTAGGTGGCATCGTGCAAGTAGCACTTTGATGGGCAATGGAAATGAAACAGTTA
AATGGCAGCAATTTGCACTTGTCCCTCCTCAATTACAATTGCAAGGATATCCGCACATTTAA
GGGGATATTTGGAAACTATGGGCAGTTTCTGGTAATCTCACTGTCTGGTCAGGAAACTATAC
TTTGGACAGTGGTAACTCTTCGGGTCCATTCCATGTTAATTTACATGTTAATAAATCTTATTC
TGCAATGGCATGTGTAAAATATCCTTTTGCATTGTTATATGGGAATTGGACCTGGAATGATA
CTGTGGGGTCTGTGTCATGTGACTATTGTAATCTAACTCAATGTGTAAATCAGTCTTGGTGGG
AAGAATTTGAAAGACGAGCCTATAATTCCAATTTCTCGCTAGTAATTGTTAAGGCTCGGACA

GAAGTATGGTTACCTATAAATCTGACTCGGCCGTGGTCAGATTCTTTTGCTATTTCTCATCTA
GTAACCGCTGTACAGACTTTGCTACATCGATCTCGACGTATGCTTGGTGTGGTCATTGCTTCG
ATTCTAGCAGTCACGTCAGTAACTGCAACAGCAGCGGTAGCAGGTCTTGCGTTACACCAAGG
AATTCAAACAGCTGATTTTATTCAGGACTGGCATAAAGACTCTCATTTGTTATGGCAAAAAC
AGCGAGATTTGGATGCCCAACTTGCTACTGATGTGCTCGATCTTCAACACACCATTTCCTGGC
TTGGAGATCAATTGGCTGTTTTATCTACACGAAGTGTGTTGAAATGTGATTGGAATTCTTCTC
AGTTTTGTATAACACCTGTACCATTTAACATGAGTGAAGGATGGGATAAAGTAAAACGATCC
TTGACTGGGCCTCAAAATCTCACTATGGAGATTATGGACCTGGAACGACAAATTCTGTCTAC
TTTTAGCAGGACTTTACTTGATATTACGGGGTCTGATTTGCTGTAAAGTCTTCAAGAGGGAGT
GAATAACTTAAATCCATTAGGACATGTATCCTCACTAATTGGGACTACTTTTGAGAACACTG
TGTTTATATTACTTTTATGTTGTGTTGCTTTTCTAGTCTTCCGGTGATGGCGGAAAGGGAAAC
AACTAAAGCACGAAGCAGAGAAGATCCAGACCATGCTACAATTTATAAAAGCAAATAAAAA
AGGGGGAGAAGAAGGGTTAATAGGGTAGCAGAGATGTGCCTGCAAATGGGCCTCTCTGCTA
GGGTTGGACGTCCTTGCAAACGAGGCGTTCTGCCAAAGAGTCTGGACACAGCCTTGAGTTTA
ATGGTCCCTTGCAAACGAGGGAGCATTCCCTTCTTGTGATAAAGAGGGAATAGAGGGCTTTG
TACAGACTCTGCAGTAGAGTAGTATTTTACTCCCCTTTGCTATACGATAACATATATGCACCT
GCGCTGCGCTGAAAAGGCTTATTCATGCAGTCTGGAATTCTGCCTAGGGGGCTTTTATAATA
AACGGCAATTAGTTTTTTGCCCAGTTCTGTTCCTCCGGCTGGAGTGTGCGTGTTGTCTGTCTC
TTGTGTGTGTCTTGTTCTGTGTCATTTCACTCGTAATCTCCAACA

>NC_056055.1:203103042-203110970#SHEEP_RIP_09(+)

TGCGGGGGACTGCCCGTGAAGGGTTAAGTCTTGGGAGCTCCCTGGCAGGTATGCCAGGCCCT
AGGACACGTGCCTAAGCTCCCTGTCCCGCCACCCTCAAGAGTTTTTATAACCCTTAAGGCTC
CAAGATGTTTGGTTTCGGCAACATTTCATAGAAGATAGATTATCTTATTGTGTATATTTCATA
GAAGATAGATATTCTGATTGTGTTCTGTATACAATGGTAAGGGTCTGGTGATTGTATCCTGA
GATTAAAAAACAACCTTGTGAGTGCCTTAAGTCACGTACTTTACCCTATATATACTGCAGCA
CAATAAAGCAAGGTATCAGCCATTTTGGGCTGATCCTCTCAACCCCATCTTTTGTCTATCTCT
TATTTTCTTAGCGGGGACGCTCCGTTCTCTCCCTGTGCAGGTGTGACTCTTGCTTGTGCTGGC
CGCGGCAGGTGGCGCCCAACGTGGGGCTCGAGCTCGACAGTTTTCCTCGCCACTACTCTTAT
TAATTGAAAAGAGTGAGTATATGAGTAAACAAGTGAATTAAATTGAGGAGGAGTAGTAAGG
TATATAGTTGAGAGTATAAATATGGGACAGACGCATAGTCGCCAGTTGTTTGTGCATATGTT
ATCTGTAATGTTAAAACATAGGGGAATTACTGTTTCTAAACCTAAATTAATCAATTTTCTTTC
ATTCATCGAGGAAGTTTGCCCTTGGTTCCCCAGAGAAGGTACAGTAAATTTGGAGACATGGA
AGAAGGTAGGGGAACAAATTCGGACTCATTATACTTTACATGGCCCTGAAAAAATCCCTGTC
GAAACTTTATCCTTTTGGACACTAATTCGTGACTGCCTGGACTTTGATAATGATGAATTAAAA
CGTTTAGGAAATTTATTAAAACAGGAAGAAGATCCTCTCCATGTTCCTGATTCGGAACCCAG
GTATGCTGTTCCCGAGGGGGTTGAAAGCGACCCTCCGTTTTCTAACTTATTACGTCCTTCAGA
TAATGATGATTTACTTTCATCCACAGATGAGGCAGAATTAGACGAAGAAGCTGCTAAATACC
ATCAAGAAGATTGGGGTTTTTAGCACAAGAAAAGGGGCGTTAACATCTAAAGATGAATTGG
TTGAATGCTTTAAAAACCTCACTATTGCTTTACAGAACGCAGGAATCAAGCTTCCTAGTAAC
AATGCCAAATCTCCTTCTGCTCCGCCTCTTCCCCCTGCTTATGCTCCTTCTGTTGTGGCTGGTC
TCGATCCCCTCCAGGGCCCCCTCCACCGTCTGAGAACATGTCTCCGCTGCAAAAGGCATTGA
GACAGGCACAGCGACTTGGTGAGGTTGTCTCTGATTTTTCTCTTGCTTTTCCTGTCTTTGAAA
ATAACAACCAGCGTTATTATGAATCACTGCCTTTTAAACAACTGAAAGAGTTAAAGATTGCT
TGCTCACAATACGGTCCTACCGCTCCATTCACCATTGCTATGATAGAAAATTTGGGTACTCA
AGCTTTACCTCCAAATGATTGGAAGCAGACAGCTAGGGCATGTCTCTCAGGGGAGATTATTT
ATTATGGAAATCTGAATTTTTGAACAATGTGCTCGTATAGCTGATGTTAACCGACAGCAAGG

TATACAGACCTCCTATGAAATGTTGATTGGTGAAGGCCCTTACCAGGCTACTGATACTCAAC
TTAATTTCTTACCTGGTGCATATGCACAAATATCAAATGCGGCTCGGCAGGCATGGAAAAAA
CTTCCTAGCTCCAGTACTAAGACAGAGGATCTTTCAAAAGTCCGGCAGGGACCTGATGAGCC
TTACCAGGACTTCGTGGCACGACTTTTAGATACTATAGGTAAGATAATGTCAGATGAAAAGG
CTGGGATGGTATTGGCAAAACAATTGGCTTTTGAAAACGCTAACTCTGCTTGTCAAGCTGCT
TTAAGACCTTATCGAAAAAAGGGAGATCTGTCTGATTTTATTCGCATTTGTGCTGACATTGG
ACCCTCCTACATGCAAGGCATTGCTATGGCAGCAGCATTACAAGGAAAAAGCATAAAGAGG
TACTTTTCCAGCAGCAAGCCCGGAACAAGAAAGGACTTCAAAAGTCAGGTAATTCGGGTTG
CTTTGTTTGTGGTCAGCCTGGCCATCGGGCTGCAGTGTGCCCTCAAAAACAACAAAGCCCTG
TTAACACTCCTAATTTGTGCCCACGCTGTAAAAAAGGAAAGCATTGGGCGCGGGATTGCCAT
TCCAAAACGGATGTTCAAGGTAATCCTTTGCCCCCGGTTTCGGGAAACTGGGTGAGGGGCCA
GCCCCTGGCCCCGAAACAATGTTATGGGGCAACACTGCAGGTTCCAAAAGGACCATTGCAG
ACCTCTATCGAGCCACAAGAGGCAGCGCGGGATTGGACCTCTGTGCCACCTCCTACACAGTA
TTAACTCCCGAGATGGGGGTCCAAACCCTTGCCACAGGAGTGTTTGGGCCTTTACCTCCAGG
GACAGCTGGACTGCTTTTAGGGCGCAGCAGTGCGTCTTTAAAAGGAATACTTATTCATCCTG
GTGTGATTGACTCTGATTATACAGGAGAGATAAAAATATTAGCCTCCGCTCCTAACAAAATT
ATTGTAATCAATGCAGGACAGCGTATAGCTCAACTTCTTTTAGTTCCATTAGTCATACAGGG
AAAACAATTAACCGAGACCGTCAAGATAAAGGTTTCGGGTCCTCTGACGCCTATTGGGTGCA
AAATGTTACCGAGGCACGACCAGAACTTGAGCTACGCATTAATGGTAAGCTTTTCCGTGGAG
TGCTTGATACAGGGGCCGATATTAGTGTTATTTCTGATAAATATTGGCCTACTACATGGCCA
AAACAGATGGCTATTTCCACTCTCCAGGGTATTGGCCAAACTACCAATCCAGAACAGAGTTC
ATCCCTTCTTACTTGGAAGGATAAAGATGGACATACAGGCCAATTTAAACCTTATATTCTGC
CCTATCTTCCAGTTAATCTATGGGGGCGTGATATATTGAGCAAAATGGGTGTTTATTTATATA
GTCCTTCACCCACTGTGACAGATTTGATGTTAGATCAGGGCTTACTTCCAAATCAAGGTTTAG
GTAAACAACATCAAGGCATCATTTTGCCCCTTGATTTAAAAACCTAATCAAGATCGAAAAGG
CTTGGGGTGTTTTCCTAGGGACCTCTGATTCTCCTGTGACGCATGCCGATCCTATTGATTGGA
AATCTGAGGAACCGGTATGGGTCGATCAGTGGCCCCTAACACAGGAAAAACTTTCTGCCGC
ACAACAGCTGGTGCAGGAACAGCTGAGACTTGGGCATATTGAACCCTCTACCTCTGCTTGGA
ATTCCCAATTTTTGTTATTAAAAAGAAGTCTGGGAAATGGAGATTGCTACAAGATCTTCGTA
AGGTAAATGAAACAATGATGCATATGGGAGCCCTACAACCTGGGTTGCCCACTCCTTCTGCT
ATACCTGATAAATCCTATATCATTGTTATAGATTTAAAAGATTGTTTTTACACTATTCCTCTT
GCACCTCAAGATTGCAAAAGATTTGCTTTCAGTTTACCCTCTGTTAATTTTAAAGAGCCTATG
CAACGCTATCAATGGAGAGTTCTCCCGCAAGGAATGACTAATAGCCCTACGCTGTGCCAAAA
TTTGTTGCTACAGCAATAGCTCCGGTTCGTCAACGTTTTCCTCAGCTATATTTGGTTCATTAT
ATGGATGATATATTACTAGCTCATGCTGACGAACATCTATTGTATCAAGCTTTTTCGATTCTA
AAACAACATTTAAGCCTTAATGGTCTTGTTATTGCTGATGAAAAATTCAGACTCATTTTCCTT
ATAATTATTTGGGTTTCTCCTTATATCCTCGTGTTTATAATACCCAATTGGTAAAACTGCAGA
CTGACCATTTAAAAACTCTAAATGACTTTCAAAAACTTTTAGGAGACATTAATTGGATACGT
CCTTATTTAAAATTACCCACTTATACCTTGCAGCCATTATTTGACATCCTTAAAGGTGACTCT
GATCCTGCGTCACCCCGAACACTTTCTTTAGAAGGACGAACTGCTTTACAATCAATAGAAGA
AGCTATTAGACAACAACAGATTACTTATTGTGATTACCAACGATCATGGGGTTTGTATATAC
TTCCTACCCCCGAGCACCCACAGGGGTTCTCTATCAAGATAAACCTTTGCGATGGATATATTT
GTCTGCTACTCCAACTAAACATCTGCTCCCTTACTATGAACTTGTTGCAAAAATTATAGCAAA
GGGACGTCACGAGGCCATCCAATATTTTGGTATGGAACCCCCTTCATTTGTGTTCCTTATGCT
TTAGAACAACAAGATTGGCTTTTTCAATTTTCAGATAATTGGTCTATAGCTTTTGCAAATTAC
CCCGGACGGATTACTCATCATTACCCTTCTGATAAATTGTTACAATTTGCTAGCTCTCATGCC
TTTATTTTTCCAAAAATAGTTCGCCGACAACCTATTCCCGAAGCGACACTTATATTTACAGAT

GGATCTTCTAATGGAACTGCAGCTTTAATCATTAACCATCAAACCTATTACGCACAAACCAG
TTTTTCTTCTGCTCAAGTTGTGGAATTATTTGCAGTCCACCAAGCGTTGCTAACTGTACCTAC
TTCCTTCAATTTATTTACAGACAGCTCCTATGTGGTCGGTGCCTTACAGATGATTGAAACTGT
TCCAATTATCGGCACCACCTCTCCTGAAGTTCTTAACTTATTTACATTGATTCAACAGGTTCT
CCATTGCCGCCAACACCCCTGTTTCTTTGGACATATTCGTGCACACTCCACCCTTCCTGGTGC
CCCTCGTACAAGGCAATCACACTGCGGACGTTCTTACTAAACAAGTGTTTTTCCAATCAGCT
ATTGATGCAGCCCGAAAATCCCATGATTTACATCACCAAAATAGTCATTCTTTACGCTTGCA
ATTTAAAATTTCCCGTGAAGCTGCACGGCAAATTGTTAAATCTTGCTCTACTTGTCCTCAATT
CTTTGTTCTCCCTCAATATGGTGTCAACCCTCGAGGTTTACGCCCTAATCACCTCTGGCAAAC
AGATGTTACTCACATTCCTCAATTTGGGCGTCTTAAATATGTTCATGTTTCTATTGACACTTTT
CCAATTTTCTCATGGCTTCCCTTCACACTGGAGAATCAACACGTCACTGTATTCAACATTTGC
TGTTTTGCTTTTCTACTTCAGGAATCCCACAAACCCTTAAAACAGATAATGGACCTGGTTATA
CTAGCCGTTCTTTTCAACGTTTTTGTCTTTCTTTCCAAATTCATCATAAAACAGGAATTCCTTA
TAATCCACAGGGACAAGGTATTGTGGAACGAGCCCATCAACGCCTTAAACATCAATTATTAA
AACAAAAAAGGGGAATGAACTGTATAGCCCCTCACCGCATAACGCCTTAAACCATGCTCTTT
ATGTTTTAAATTTTTTAACTTTAGACGCAGAAGGCAATTCAGCAGCCCAGCGTTTTTGGGGA
GAACGATCCTCATGCAAAAAACCACTTGTGCGATGGAAGGATCCACTTACCAATCTGTGGTA
TGGGCCAGACCCTGTACTAATATGGGGACGAGGGCATGTTTGTGTTTTTCCACAGGATGCCG
AAGCGCCGCGCTGGATTCCGGAAAGGCTGGTACGCGCAGCAGAGGAACTCCCTGACACATC
AAATGCAATGCATGACACTGAGTGAGCCCACGAGTGAGCTGCCTACCCAGAGGCAAATTGA
GGCGCTGATGCGTTATGCTTGGAATGAGGCTCATGTACAACCTCCAGTGACACCTACTAATA
TACTGATCATGTTATTATTATTGTTACAGCGGATACAAAACGGGGCAGCTGCGGCTTTTTGG
GCATACATTCCTGATCCGCCTATGATTCAATCCTTAGGATGGGATAAAGAAACAGTACCTGT
ATATGTTAATGATACAAGTCTTTTAGGAGGAAAATCAGATATTCACATTTCTCCTCAGCAAG
CCAATATCTCCTTTTATGGCCTTACTACTCAATACCCTATGTGCTTTTCTTATCAATCACAGCA
TCCTCATTGTATACAGGTGTCAGCTGATATATCCTATCCTCGAGTGACTATTTCAGGCATTGA
TGAAAAAACCGGAAAGAGATCGTACCGTGACGGAACCGGACCCCTCGACATTCCGTTTTGT
GACAAAAATTTAAGCATCGGCATAGGAATAGACACTCCTTGGACTTTATGTCGAGCACGAAT
TGCATCGGTGTATAACATCAACAATGACAATACCACCCTTTTATGGGACTGGGCACCTGGAG
GAACACCTGATTTCCCCGAATATCGAGGACAGCATCCACCCATTCTCTCTGTAAACACTGCT
CCTATATTTCAAACTGAACTGTGGAAACTTTTGGCTGCTTTTGGTCATGGCAATAGTCTATAT
TTACAGCCCAATATTAGTGGGAGCAAATATGGTGATGTGGGAGTTACAGGATTTTTATATCC
CCGAGCTTGTGTTCCTTACCCATTCATGTTGATACAAGGCCATATGGAAATAACGCTGTCATT
GAATATTTATCATTTAAATTGTTCTAATTGCATACTTACTAATTGCATTAGAGGTGTAGCCAA
AGGAGAACAAGTTATAATAGTAAAACAACCTGCTTTTGTAATGTTACCTGTTGAAATAACTG
AAGAATGGTATGATGAAACTGCTTTAGAATTGTTACAACGCATTAATACGGCTCTTAGCCGT
CCTAAAAGAGGTCTGAGCCTGATTATTCTGGGTATAGTATCTTTAATCACCCTTATAGCAACT
GCTGTTACTGCTTCTGTATCTTTAGCACAATCCATTCAAGCTGCTCATACTGTAGATTCCTTG
TCATATAATGTTACTAAAGTAATGGGAACTCAAGAAGATATAGATAAAAAAATAGAAGATA
GATTATCCGCTTTATATGATGTAGTTAGAGTTCTAGGAGAACAAGTTCAGAGCATTAATTTTC
GCATGAAAATTCAATGCCATGCTAATTATAAATGGATTTGTGTTACAAAAAAGCCTTACAAT
ACTTCTGACTTTCCGTGGGATAAGGTGAAAAAACATCTGCAAGGAATTTGGTTTAATACTAA
TGTTTCTTTAGATCTTTTACAATTGCACAATGAAATTCTTGACATCGAAAATTCTCCAAAAGC
TACTTTGAATATAGCTGATACCGTCAATAATTTTTTACAAAATTTATTTTCTAACTTTCCTAGC
CTTCATTCACTGTGGCGAAGTATAATTGCTATGGGCGCGGTTCTGACTGTTGTGCTTATCATA
ATTTGTCTAGCTCCTTGTCTTATTCGTAGCATTGTTAAAGAATTTCTACATATGAGAGTTTTA
ATACATAAAAACATGTTGCAACACCAACATCTTATGGAGCTTTTAAAAAATAAAGAGAGGG

GAGCTGCGGGGGACTGCCCGTGAAGGGTTAAGTCTTGGGAGCTCCCTGGCAGGTATGCCAG
GCCCTAGGACACGTGCCTAAGCTCCCTGTCCCGCCACCCTCAAGAGTTTTTATAACCCTTAA
GGCTCCAAGATGTTTGGTTTCGGCAACATTTCATAGAAGATAGATTATCTTATTGTGTATATT
TCATAGAAGATAGATATTCTGATTGTGTTCTGTATACAATGGTAAGGGTCTGGTGATTGTATC
CTGAGATTAAAAAACAACCTTGTGAGTGCCTTAAGTCACGTACTTTACCCTATATATACTGC
AGCACAATAAAGCAAGGTATCAGCCATTTTGGGCTGATCCTCTCAACCCCATCTTTTGTCTAT
CTCTTATTTTCTTAGCGGGGACGCTCCGTTCTCTCCCTGTGCAGGTGTGACTCTTGCTTGTGCT
GGCCGCGGCA

>NC_056056.1:39411628-39419559#SHEEP_RIP_10(+)

TGCAGGGGACGACCCGTGAAGGGTTAAGTCTTGGGAGCTCCCTGGCAGGTATGCCGGGCCC
TAGGACACGTGCCTAAGCTCCCTGTCCCGCCACCCTCAAGAGTTTTTATAACCCTTAAGGCTC
CAAGATGTTTGGTTTCGGCAACATTTCATAGAAGATAGATTATCTTATTGTGTATATTTCATA
GAAGATAGATATTCTGATTGTGTTCTGTATACAATGGTAAGGGTCTGGTGATTGTATCCTGA
GATTAAAAAACAACCTTGTGAGTGCCTTAAGTCACGTACTTTACCCTATATATACCGCAGCA
CAATAAAGCAAGGTATCAGCCATTTGGGGCTGATCCTCTCAACCCCATCTTTTGTCTATCTCT
TATTTTCTTAGCGGGGACGCTCCGTTCTCTCCCTGTGCAGGTGTGACTCTTGCTTGTGCTGGC
CGCGGCAGGTGGCGCCCAACGTGGGGCTCGAGCTCGACAGTTTTCCTCGCCACTACTCTTAT
TAATTGAAAAGAGTGAGTATATGAGTAAACAAGTGAATTAATTTGAGGAGGAGTAGTAAGG
TATATAGTTGAGAGTATAAATATGGGACAGACGCATAGTCGCCAGTTGTTTGTGCATATGTT
ATCTGTAATGTTAAAACATAGGGGAATTACTGTTTCTAAACCTAAATTAATCAATTTTCTTTC
ATTCATCGAGGAAGTTTGCCCTTGGTTCCCCGGAGAAGGTACAGTAAATTTAGAGACATGGA
AGAAGGTAGGGGAACAAATTCGGACTCATTATACTTTACATGGCCCTGAAAAAATCCCTGTC
GAAACTTTATCCTTTTGGACACTAATTCGTGACTGCCTGGACTTTGATAATGATGAATTAAAA
CGTTTAGGAAATTTATTAAAACAGGAAGAAGATCCTCTCCATGTTCCTGATTCGGAACCCAG
ATATGCTGTTCCCGAGGGGGTTAAAAGCGACCCTCCGTTTTCTAACTTATTGCATCCTTCAGA
TAATGATGATTTACTTTCATCCACAGATGAGGCAGAATTAGACGAAGAAGCTGCTAAATACC
ATCAAGAAGATTGGGGTTTTTAGCACAAGAAAAGGGGCGTTAACATCTAAAGATGAATTGG
TTGAATGCTTTAAAAACCTCACTATTGCTTTACAGAACGCAGGAATCAAGCTTCCTAGTAAC
AATGCCAAATCTCCTTCTGCTCCGCCTCTTCCCCCTGCTTATGCTCCTTCTGTTGTGGCTGGTC
TCGATCCCCCTCCAGGGCCCCCTCCACCGTCTGAGAACATGTCTCCGCTGCAAAAGGCATTG
AGACAGGCACAGCGACTTGGTGAGGTTGTCTCTGATTTTTCTCTTGCTTTTCCTGTCTTTGAA
AATAACAACCAGCGTTATTATGAATCACTGCCTTTTAAACAACTGAAAGAGTTAAAGATTGC
TTGCTCACAATACGGTCCTACCGCTCCATTCACCATTGCTATGATAGAAAATTTGGGTACTCA
AGCTTTACCTCCAAATGATTGGAAGCAGACAGCTAGGGCATGTCTCTCAGGGGAGATTATTT
ATTATGGAAATCTGAATTTTTTGAACAATGTGCTCGTATAGCTGATGTTAACCGACAGCAAG
GTATACAGACCTCCTATGAAATGTTGATTGGTGAAGGCCCTTACCAGGCTACTGACACTCAA
CTTAATTTCTTACCTGGTGCATATGCACAAATATCAAATGCGGCTCGGCAGGCATGGAAAAA
CTTCCTAGCTCCAGTACTAAGACAGAGGATCTTTCAAAAGTCCGGCAGGGACCTGATGAGCC
TTACCAGGACTTCGTGGCACGACTTTTAGATACTATAGGTAAGATAATGTCAGATGAAAAGG
CTGGGATGGTATTGGCAAAACAATTGGCTTTTGAAAACGCTAACTCTGCTTGTCAAGCTGCT
TTAAGACCTTATCGAAAAAAGGGAGATCTGTCTGATTTTATTCGCATTTGTGCTGACATTGG
ACCCTCCTACATGCAAGGCATTGCTATGGCAGCAGCATTACAAGGAAAAAGCATAAAGAGG
TACTTTTCCAGCAGCAAGCCCGGAACAAGAAAGGACTTCAAAAGTCAGGTAATTCGGGTTG
CTTTGTTTGTGGTCAGCCTGGCCATCGGGCTGCAGTGTGCCCTCAAAAACAACAAAGCCCTG
TTAACACTCCTAATTTGTGCCCACGCTGTAAAAAAGGAAAGCATTGGGCGCGGGACTGCCGT
TCCAAAACGGATGTTCAAGGTAATCCTTTGCCCCCGGTTTCGGGAAACTGGGTGAGGGCCAG

CCCTGGCCCCGAAACAATGTTATGGGGCAACACTGCAGGTTCCAAAAGGACCATTGCAGAC
CTCTGTCGAGCCACAAGAGGCAGCGCGGGATTGGACCTCTGTGCCACCTCCTACACAGTATT
AACTCCCGAGATGGGGGTCCAAACCCTTGCCACAGGAGTGTTTGGGCCTTTACCTCCAGGGA
CAGCTGGACTGCTTTTAGGGCGCAGCAGTGCGTCTTTAAAAGGAATACTTATTCATCCTGGT
GTGATTGACTCTGATTATACAGGAGAGATAAAAATATTAGCCTCCGCTCCTAACAAAATTAT
TGTGATCAATGCAGGACAGCGTATAGCTCAACTTCTTTTAGTTCCATTAGTCATACAAGGAA
AAACAATTAACCGAGACCGTCAAGATAAAGGTTTCGGGTCCTCTGACGCCTTTTGGGTGCAA
AATGTTACCGAGGCACGACCAGAACTTGAGCTACGCATTAATGGTAAGCTTTTCCGCGGAGT
GCTTGATACAGGGGCCGATATTAGTGTTATTTCTGATAAATATTGGCCTACTACATGGCCAA
AACAGATGGCTATTTCCACTCTCCAGGGTATTGGCCAAACTACCAATCCAGAACAGAGTTCA
TCCCTTCTTACTTGGAAGGATAAAGATGGACATACAGGCCAATTTAAACCTTATATTCTGCC
CTATCTTCCAGTTAATCTATGGGGGCGTGATATATTAAGCAAAATGGGTGTTTATTTATATAG
TCCTTCACCCACTGTGACAGATTTGATGTTAGATCAGGGCTTACTTCCAAATCAAGGTTTAGG
TAAACAACATCAAGGCATCATTTTGCCCCTTGATTTAAAACCTAATCAAGATCGAAAAGGCT
TGGGGTGTTTTCCTAGGGACCTCTGATTCTCCTGTGACGCATGCCGATCCTATTGATTGGAAA
TCTGAGGAACCGGTATGGGTCGATCAGTGGCCCCTAACACAGGAAAAACTTTCTGCCGCACA
ACAGCTGGTGCAGGAACAGCTGAGACTTGGGCATATTGAACCCTCTACCTCTGCTTGGAATT
CCCCAATTTTTGTTATTAAAAAGAAGTCTGGGAAATGGAGATTGCTACAAGATCTTCGTAAG
GTAAATGAAACAATGATGCATATGGGAGCCCTACAACCTGGGTTGCCCACTCCTTCTGCTAT
ACCTGATAAATCCTATATCATTGTTATAGATTTAAAAGATTGTTTTTACACTATTCCTCTTGC
ACCTCACGATTGCAAAAGATTTGCTTTCAGTTTACCCTCTGTTAATTTTAAAGAGCCTATGCA
ACGCTATCAATGGAGAGTTCTCCCGCAAGGAATGACTAATAGCCCTACGCTGTGCCAAAAAT
TTGTTGCTACAGCAATAGCTCCCGTTCGTCAACGTTTTCCTCAGCTATATTTGGTTCCTTATAT
GGATGATATATTACTAGCTCATGCTGATGAACATCTATTGTATCAAGCTTTTTCGATTCTAAA
ACAACATTTAAGCCTTAATGGTCTTGTTATTGCTGATGAAAAAATTCAGACTCATTTTCCTTA
TAATTATTTGGGTTTCTCCTTATATCCTCGTGTTTATAATACCCAATTAGTAAAATTACAGAC
TGACCATTTAAAAACTCTAAATGACTTTCAAAAACTTTTAGGAGACATTAATTGGATACGTC
CTTATTTAAAATTACCCACTTATACCTTGCAGCCATTATTTGACATCCTTAAAGGTGACTCTG
ATCCTGCGTCACCCCGAACACTTTCTTTAGAAGGACGAACTGCTTTACAATCAATAGAAGAA
GCTATTAGACAACAACAGATTACTTATTGTGATTACCAACGATCATGGGGTTTGTATATACTT
CCTACCCCCCGAACACCCACAGGGGTTCTCTATCAAGATAAACCTTTGCGATGGATATATTT
GTCTGCTACTCCAACTAAACATCTGCTCCCTTACTATGAACTTGTTGCAAAAATTGTAGCAAA
GGGACGTCACGAGGCCATCCAATATTTTGGTATGGAACCCCCCTTCATTTGTGTTCCTTATGC
TTTAGAACAACAAGATTGGCTTTTTCAATTTTCAGATAATTGGTCTATAGCTTTTGCAAATTA
CCCAGGACGGATTACTCATCATTACCCTTCTGATAAATTGTTACAATTTGCTAGCTCTCATGC
CTTTATTTTTCCAAAAATAGTTCGCCGACAACCTATTCCCGAAGCGACACTTATATTTACAGA
TGGATCTTCTAATGGAACTGCAGCTTTAATCATTAACCATCAAACCTATTACGCACAAACCA
GTTTTTCTTCTGCTCAAGTTGTGGAATTATTTGCAGTCCACCAAGCGTTGCTAACTGTACCTA
CTTCCTTCAATTTATTTACAGACAGCTCCTATGTGGTCGGTGCCTTACAGATGATTGAAACTG
TTCCAATTATCGGCACCACCTCTCCTGAAGTTCTTAACTTATTTACATTGATTCAACAGGTTC
TCCATTGCCGCCAACACCCCTGTTTCTTTGGACATATTCGTGCACACTCCACCCTTCCTGGTG
CCCTGGTACAAGGCAATCACACTGCGGACGTTCTTACTAAACAAGTGTTTTTCCAATCAGCT
ATTGATGCAGCCCGAAAATCCCATGATTTACATCACCAAAATAGTCATTCTTTACGCTTGCA
ATTTAAAATTTCCCGTGAAGCTGCACGGCAAATTGTTAAATCTTGCTCTACTTGTCCTCAATT
CTTTGTTCTCCCTCAATATGGTGTCAACCCTCGAGGTTTACGCCCTAATCACCTCTGGCAAAC
AGATGTTACTCACATTCCTCAATTTGGGCGTCTTAAATATGTTCATGTCTCTATTGACACTTTT
TCCAATTTTCTCATGGCCTCCCTTCACACTGGAGAATCGACACGTCACTGTATTCAACATTTG

CTGTTTTGCTTTTCTACTTCAGGAATCCCACAAACCCTTAAAACAGATAATGGACCTGGTTAT
ACTAGCCGTTCTTTTCAACGTTTTTGTCTTTCTTTCCAAATTCATCATAAAACAGGAATTCCTT
ATAATCCACAGGGACAAGGTATTGTGGAACGAGCCTATCAACGCCTTAAACATCAATTATTA
AAACAAAAAAAGGGGAATGAACTGTATAGCCCCTCACCGCATAACGCCTTAAACCATGCTC
TCTATGTTTTAAATTTTTTAGCTTTAGACGCAGAAGGCAATTCAGCAGCCCAGCGTTTTTGGG
GAGAACGATCCTCATGCAAAAAACCACTTGTACGATGGAAGGATCCACTTACCAATCTGTGG
TATGGGCCAGACCCTGTACTAATATGGGGACGAGGGCATGTTTGTGTTTTTCCACAGGATGC
CGAAGCGCCGCGCTGGATTCCGGAAAGGCTGGTACGCGCGGCAGAGGAACTCCCTGACACA
TCAAATGCAACGCATGACACTGAGCGAGCCCACGAGTGAGCTGCCTACTCAGAGGCAAATT
GAGGCGCTGATGCGTTATGCTTGGAATGAGGCTCATGTACAACCTCCAGTGACACCTACTAA
TATACTGATCATGTTATTATTATTGTTACAGCGGATACAAACGGGGCAGCTGCGGCTTTTGG
GCATACATTCCTGATCCGCCTATGATTCAATCCTTAGGATGGGATAAAGAAACAGTACCTGT
ATATGTTAATGATACAAGTCTTTTAGGAGGAAAATCAGATATTCACATTTCTCCTCAGCAAG
CCAATATCTCCTTTTATGGTCTTACTACTCAATACCCTATGTGCTTTTCTTATCAATCACAGCA
TCCTCATTGTATACAGGTGTCAGCTGATATATCCTATCCTCGAGTGATTATTTCAGGCATTGA
TGAAAAAACCGGAAAGAGATCGTACCGTGACGGAACCGGACCCCTCGACATTCCGTTTTGT
GACAAACATTTAAGCATCGGCATAGGAATAGACACTCCTTGGACTTTATGTCGAGCACGAAT
TGCATCGGTGTATAACATCAACAATGCCAATACCACCCTTTTATGGGACTGGGCACCTGGAG
GAACACCTGATTTCCCCCGAATATCGAGGACAGCATCCACCCATTCTCTCTGTAAACACTGC
TCCTATATTTCAAACTGAACTGTGGAAACTTTTGGCTGCTTTTGGTCATGGCAATAGTCTATA
TTTACAGCCCAATATTAGTGGGAGCAAATATGGTGATGTGGGAGTTACAGGATTTTTATATC
CCCGAGCTTGTGTTCCTTACCCATTCATGTTGATACAAGGCCATATGGAAATAACGCTGTCAT
TGAATATTTATCATTTAAATTGTTCTAATTGCATACTTACTAATTGCATTAGAGGTGTAGCCA
AAGGAGAACAAGTTATAATAGTAAAACAACCTGCTTTTGTAATGTTACCTGTTGAAATAACT
GAAGAATGGTATGATGAAACTGCTTTAGAATTGTTACAACGCATTAATACGGCTCTTAGCCG
TCCTAAAAGAGGTCTGAGCCTGATTATTCTGGGTATAGTGTCTTTAATCACCCTTATAGCAAC
TGCTGTTACTGCTTCTGTATCTTTAGCACAATCCATTCAAGCTGCTCATACTGTAGATTCCTT
GTCATATAATGTTACTAAAGTAATGGGAACTCAAGAAGATATAGATAAAAAAATAGAAGAT
AGATTATCAGCTTTATATGATGTAGTTAGAGTTTTAGGAGAACAAGTTCAGAGCATTAATTT
TCGCATGAAAATTCAATGCCATGCTAATTATAAATGGATTTGTGTTACAAAAAGCCTTACAA
TACTTCTGACTTTCCGTGGGATAAGGTGAAAAAACATCTGCAAGGAATTTGGTTTAATACTA
ATGTTTCTTTAGATCTTTTACAATTGCACAATGAAATTCTTGACATCGAAAATTCTCCAAAAG
CTACTTTGAATATAGCTGATACCGTCGATAATTTTTTACAAAATTTATTTTCTAACTTTCCTAG
CCTTCATTCACTGTGGCGAAGTATAATTGCTATGGGCGCGGTTCTGACTGTTGTGCTTATCAT
AATTTGTCTAGCTCCTTGTCTTATTCGTAGCATTGTTAAAGAATTTTTACATATGAGAGTTTT
AATACATAAAAACATGTTGCAACACCAACATCTTATGGAGCTTTTAAAAAATAAAGAGAGG
GGAGCTGCAGGGGACGACCCGTGAAGGGTTAAGTCTTGGGAGCTCCCTGGCAGGTATGCCG
GGCCCTAGGACACGTGCCTAAGCTCCCTGTCCCGCCACCCTCAAGAGTTTTTATAACCCTTA
AGGCTCCAAGATGTTTGGTTTCGGCAACATTTCATAGAAGATAGATTATCTTATTGTGTATAT
TTCATAGAAGATAGATATTCTGATTGTGTTCTGTATACAATGGTAAGGGTCTGGTGATTGTAT
CCTGAGATTAAAAAACAACCTTGTGAGTGCCTTAAGTCACGTACTTTACCCTATATATACCG
CAGCACAATAAAGCAAGGTATCAGCCATTTGGGGCTGATCCTCTCAACCCCATCTTTTGTCT
ATCTCTTATTTTCTTAGCGGGGACGCTCCGTTCTCTCCCTGTGCAGGTGTGACTCTTGCTTGTG
CTGGCCGCGGCA

>NC_056057.1:33404754-33412479#SHEEP_RIP_11(+)

ACTCTGCAGTAGACAGAGATTTCACTCCCCTTGCTGTATGATAACATGTATGCACCTGCGCT
GTACTGAAAAGGCTTATTCATGCAGTCTGGAATTCTGCCTAGGGGGCTTTATAAAAATAAAC
CACAATTAGTTTTGTCCAGTTTTTTTCCTCCGGCCGGAGTGGTGTATTGTCTGTCTCTTGTGTG
TCTCGTGTGTTTTGTCTTTGTGTCATTTCGCTCACGACATCTGTCGCCCAACGTGGGGCTTGA
GTGAAACCGAAAGGGTGAGTAACCCCGGGGGGATTTTAAATCCATAGCAGGGGAGCTTTCG
GGAAAATCATGGGGAATTCCTCACCCTAGCAGGGTAACTTTCGGAAAATCATGGGGAATTCC
TCATCATCATTTCAGACACAATACAGGGAGTTAGTCAAAGGACTTCTCCACTCCATAGGCGT
TAAGGTCTCGACTCATCGATTGAGTGAGCTCTTTCGCTTGGTGGAGCAATGTTGTCATTGGTT
TCAATATCAAACTAAGTTACAGTTAAATTTGAAGGAGTGGAAAATAATTCAAAAGGAATTG
AGAAAGCAACATCAGAAGGGTAATGTGATCCCTTTGAAGTTATGGACTATGTGTAGTGCTAT
AACACAGGCTTTGACCTTGCTTTCTACTGACAGTGAAACTAAATCTAATGCTTCAAGGAGGG
GAGAAATAATTTATGAGGATGTGTCAGACGTTGGTGGGGCTTCTGCATCGCCTGAAGGCAAG
GATACAGGTGAGTCTCCTCCTGTAAATGGTGAAACATCTAATAGTTCAGAATCAGATTCAGA
GGCTTCTTCGGTTTCGTCAGAGGAGGGCAAAGAGATGAAAGAAATGACCCATCTATTCCGG
GAATGGTGGAAATCCCGTAAGGAGGAGAAAAAATCTACACCTTCTGCTCCTCCTTGTGCTTC
TCTTTTCCCCACTGCGGTTGATCGGCGCGATGTGGGCAGGGAACATTGTCAGTTCTCCTTTTC
TTTGTCAATGCTTCATGATGATGACTTGCCTGCTCCCCCTGGTGGGTTTATCGATCCTCCACA
ATTATTTCCCATCCAGAGACAGCAAAATGACAACGTGATAAATATTCAATACACTCCTTTGG
AATATAAATTTTTTAAAGATCTTAAAGCTGCAGTAGCACAATACAGTCCTCAATCTCCCTTTG
TTTTGGCTATGCTGGAATCACTGGGAAAAGGCAAATTAATCATTCCGTTAGATTGGGAATCT
ATTGCCCAAGCTGTCTTGGAGGGCTCTCAATGGTTGCAACTTCGTAGCTGGTAGGAAGAAGC
TAGAAAGCAGGTTCAGATTAATGAGGGACAGAATCCCCCCGGTCCTCTCGAGGACAAGCTA
ATGGGAGAGGGCCATTATCGGGCTTTAAGAGAACAGGCTCAATACTCTGATCAGGACTTAC
AACAAGTCCACCAGGTCTTTTTAAGAGCATGGCGCCGTGTGGTGCCTACTGGCCACGCCCAG
CCCTCCTTTGTTAAAACAATGCAAGGCCCCAATGAGCCTTATACTGATTTTCTAGCAAGGTTG
AGGGTAGCTGTGGAATGGGCTGTAGGGAGGGATGAGATTTCAGAGATATTATTACAAACTTT
AGCATTTGAAAATGCAAATCCTGAATGCAAGTGTATACTGGCACCATTAAAGGGACAGGGT
GCACCTATAGCTGAATATATCAGAGCCTGCTCAGGAGTAGGAGGAACTGAGCATCAGGCTA
ATGTCTTTGCTATAGCCTAGGCCAAAGCTATGAGACCACAAAAGGGAGGTAACTGCTTCCAC
TGTGGAAAACCTGGTCATATGAAAAGAGTGTGTCAGAAATTAAAAGCTGATCAAGGTGCAA
TTCCTAAAGACAGATCTCTTGCTGGGAAGAATAAGACTCCTCCTGGACTTTGCCGTCGGTGC
GGGAAGGGGCTTCGTTGGACTAATGAATGCAGATCTAAAACAGACAAAATGGGCAACCTGA
TACTGGGAAACTATCCTGCGGGCCTAAGTCCTTGGGGCCCAGGAACATTACTGGGGACTTCT
CCTCCTTGCCCTCCTCCCATCCCATCTGCCCCAACCCTATTCCCTCCCAACAACCGTTACGAG
TTGATGCCCCATTATAAGGACCTCAGATGATGATTTCGGACTTACGGTCTGCTACTTCAGGG
AGTGCTGCTGCTGATTTGCCACTAGCTGATAATGTTCTTTTGTCACCAGGGGGAGGTATTTAC
AAATTAAAAACAAATGTATTTGGACCACTGCCTAAAGGCACTTTTGGCTTGATATTAGGCTG
TAGCAGCGTGGCTTTGAGAGGTTTAACCATAATTCCTGGGGTAATAGACTCTGACTATGTTA
GGGAAATTTTAATTATGGTCTCTGCTTCTACCAAGCTTTCATTGTTAGCTGGGGAGCGTATTG
CTCAAATACTTCTCCTATCTTATCACCCCTTTTTGGCTCTTCCTAATGAACGAACAGGAGGAT
TTGGAAGTACTGGGTGGCATATATTTTGGAAAATGCTTATCGAAGATTCTTGCCCTGTTCTCT
CCTTGATTATACAAGGAAACAACTTTGAGGGACTAGTAGACACAGGGGCAGATGTTTCAGTC
ATTTCTTCTCAACAATGGCCCCAAGATTGGAAAAAAGAAAAAAGCCCTCTAATGCTGACGG
GACTGGGCTCCATTGCAGACGTCTGGAAGAGTACCCATCCCTTGCAATGCCAATTCCATAAT
GGAAGATCAGTGTTTGTTACCTTTTATATTGTACACATACCTATCAATATATGCGGAAGAGA
TCTTCTCTCTCCTTTGGGGGCTTCTGTAACCATTCCATCGGAAAACTAGTAGCCACTGCTCAA
ATTCCTCGAGCACTCCCATTAAAATGGTTAACTAATACTCCAAAATGGGTTGAGCAGTGGCC

ATTACCACAAGTGAAGCTAAGAGGCATTAAAACAATTAATACAAGAACAACTTCAACTTGG
TCATATAGAGCCCTCTACCTCCCCCTGGAATTCTCCTGTTTTTGTTATAAAAAAGAAATCTGG
AAAATGGAGAATGTTAACTGATCTACGAGAAGTTAATAAATGTATTGAACCTATGGGAGCA
TTGCAATTGGGACTCCCCTCTCCAGCTCTTATTCCTCAGAATTGGTCCTTAATGGTGCTAGAT
CTTAAAGACTGTTTTTTTTTACCATTCCCCTACAATTGCAAGATAGAGATAAATTTGCTTTTA
CAGTTCCTGTTCTTAATCATGCTCAGCCTGTTAAGCGTTATCAGTGGACAGTCTCACCACAAG
GAATGATAAATAGTCCTACCTTATGCCAAGAATTCGTAGCTCGCTCTTTACAATCCCTCCATT
GAGAATACCCCAATTATATTCTATATCATTATATGGATGATCTCCTATTAGCAGCTCCTAATA
TTGCTGAACGTGATGAATTCTTTTTAAAAGTACAGGAGGCTTTAAGACTATACAATTTGCAA
GTAGCCCCAGAAAAAATTCAAAAGGACTTTCCTATTTCGTATTTAGGGACAATATTGGAACA
ACATAGAATAAGGCCCCAAAAGTTGCAAATTAGAAGAGACCATCTCAAAACCTTAAATGAT
TTTCAAAAGTTATTGGGAGATATTAATTGGCTACGCCCGGTACTTGGGATTCCTACTTATCAA
TTACGACATTTGTTTTCTGCTTTAGAAGGAGATACAGCTCTGGATAGCCCCCGGACCTTAACC
CCATTGGCTTTACAGGAACTTCAATTTGTTGAGCAACGACTAAATGACAGCTTTTTTACTTAC
TTACATGTGTCTCAATCTATTTCGTTTATAATATTTCATACCCCTTATTCTCCATCTGGTGTAA
TTGCTCAAGAAAAAGGATTAACAGAATGGGTTTTCTTACCTAACAGTTTTTCCAAAAAATTG
ACTACATATATGGATAAATTAGCCTTCCTTATACAGAAAGGTCGCCGTCGTATTTCACAATT
ATCAGGATGTGAACCACACCAGATTGTTACTCAGTTAACAACTGCTCAAATATCTCGATGTT
TACAATTTAATGAAAACTGGCAAATTTCTCTTACCTCATATCCTGGTTCATTTTCTAATCATT
ATCCGTCATCTAAATTGATTGATTTTCTTCGGACTAACACTATGATATCTCATTTCCCAATTTC
AGATGTTCCAGCTAAGGGGCCCACTATTTTTACAGATGCAAATAAAAATACTGCTGGATATT
GGACCCCGGAAAGTTCCAGGGTTCTCCCCCACTCATTTTCTTCTGTACAGCCCACTGAATTGT
GGGCTATCTATTTAGTTTTGCAAGATTTTCCCCAACTTCCTATTAACACTGTTTCAGATTCTTG
ATATGCTGTTCTCTCTTGCCTACAGCTTCCCCATGTCTCCCTTCCACTGACCCTTAAAACAGC
TATTGATGAATTGTTTTACCAAGTACAACAATTGCTCTTGCGATGTTCAGAGTTAATTTTCTT
TACTCACATCCATGCACATTCTGCCCTTCCTGGACCCTTATCATTCGGAAATGCTACAATTGA
TGCCTTACTTTATCCTATAGAAGCAGCAAAACAAGAACATCTCTTACAGCATACCAACTCCA
AAGGGTTACAAAAATCTCATGCTATTACTTGAAAACAAGCTCAAAATATTGTTCATTCTTGTT
CCATATGTGCACCCTTTGCTTTGCCATTTACCTCACCAGGTGTCAACATGAGATGACAACAA
GCAAATCAGATATGGCAAATGGATGTAATTTACATTTCTTCTTTTGGACAACAAAAAAGTGT
GCATCATATTATAGATGCTTGCATACATTTTCAATGGGCCACTGCATTACATTCTGAAAAGG
CTGACGCTGTTATTACTCATTTGTTATCTTGTTTTGCAGTTATGGGATTACCAATTGAATTGA
AAACTGATAATGCACCTGCCTACCAATCTGCAAAATTAGCTCACTTTTTATCTCAATACCATA
TAACTCATACTTTTGGTATTCCTTATAATAGTCAAGGGCAAGCTATCATTGAAAGAGCTAAT
CGTACCTTGTGTGATTATCTTGAAAAAATAAAAAAGGGGGAACAAGAGAGATTTATGAAAC
CTAAAGACATTCTGAATAAAACCTTACTTACCCTAAATTTTTTGAATGTTTGGAGTAAGGGA
AATCTATCAGCAGCAGAGTTGAATTTTCAAGGGAAAGAAGAGGATAAGCAGATCTTGAATA
TGCCTATTTGGTATAAAGATAAAGATAAAGGTTGGATCCCAGCATCATTAATATATTTGGGA
CGAGGGTATGCTTTCATTTCTGTTAATAATTACAGGTTTTGGACCCCAGCAAGACTGATCAA
AATCAACAATGGCTGATCCCTTTGTTCAAAAATTCAAAGAGCTTACTATGCAGAGAAGCTTT
ACTTTCTGTACAAGGGAAGCAACACCTCCTACATGGGGTCAAATGAAGAGGTTGACCCAGG
AAGCAGAGAAGACATTAATGAAGGCGGGGCAACCTCTGAATCCTACCAATCTTTTGCTTGCC
ATGATGGCGTTGGTGACATGTCAGGTAATCGGTGTATCGGCAAGTAATCATACATATTGGGC
ATATATACCTAATCCCCCATTAGTAAGAGCAGTTTCTTGAGGGGAACCAGAAGTGCAGGTAT
GTACTAATGAGACTGCCTTTTTTCCCCCACCAGCTTGAGGGGGAATAGAACAACTATCTCAT
CATAAACAGCAATATAATATTAGTAATTTGACCATTGCAGTGGAAGGTATTCCTTTATGTAT
AGGAGGACACCCCTTTTGTCTGTCCACCAAGGAACATTCTCATCATTCTTACAATACATGGG

GGGTAAAATATAATAATTACCATTTTGCTACTTTTACTGTGCTTGTTTCCACCAGGGGATTTA
GCATCTCGACAGAACCGGTAGATATTCATAATGAAATACATATGTCACTATGTCCTGTTAAC
TTTTTTGCTTCTTCTCTAGAATCTTTGGAATGGGAATGTTGCCGAGGTCATCGACCCTTTAAA
GTCATGAATTATTCTGGGTCTATCATTGTAGATTGGAGTCCAGATCATGGGCAATTCTTAGA
AAAATGGTCAAATAAATCTTTTAGGTGGCATTGTGCAAATAGCACTTTGATCGGCAATGGTA
ATGAAACAGTTAAATGGCAGCAATTTGCACTTGTCCCTCCTCAATTACAATTGCAAGGGTAT
CTGCACATTCAAGGGGATATTTGGAAACTATGGGCAGTTTCTGGTAATCTCACTATCTGGTC
AGCAAACTATACTTTGGATAGTGGTGACTCTTCGGGTCCATTCCATGTTAATTTACATGTTAA
TAAATCTTATTCTGCAATGGCATGTGTAAAATATCCTTTTGCATTGTTATATGGGAATTGGAC
CTGGAATGATACTGTGAGATCTGTGTCATGTGACTACTGTAATCTAACTCAATGTGTAAATC
AGTCTTGGTGGGAAGAATTTGAAAGACGAACCTATAACTCCAATTTCTCGTTAGTAATTGTT
AAGGCTCGGACAGAAGTATGGTTACCTATAAATCTGACTCGGCCATGGTCAGACTCTTTTGC
TGTTTCTCATCTAGTAACCGCTGTACAGACTTTGCTACATCGATCTCGACGTATGCTTGGTGT
GGTCATTGCTTCGATTCTAGCAGTCGCGTCAGTAACTGCAACAGTGGCAGTGGCAGGACTTG
CATTACACCAAGGAATTCAAGCAGCTGATTTTATTCGGGACTGGCATAAAGACTCTCATTTG
TTATGGCAACAACAGCGAGATTTGGATGCACAACTTGCTACCGACGTGCTCAATCTTCAACA
CATCGTTTCCTGGCTTGGAGATCAATTAGCTGTTTTATCTACACGAAGTGTGTTGAAATGTGA
TTGGAATTCTTCTCAGTTTTGTATAACACCTTTACCATTTAGCATGAGTGAAAGATGGGATAA
AGTAAAATGATCCTTGACTGGGCATCCAAATCTCACTATGGAGATTATGGACCTGGAATGAC
AAATTTTGTGTACTTTTAGCAGGACTTTACCTGGCATTACGGGGTCCTGATTTGCTGAAAAGT
CTTCAAGAGAGAATGAATAATTTAAGTCCATTAGGGCGTGTATCCTCACTAATTGGGACTAC
CTTTGGGAACACTGTGTTTATATTACTTTTATGTTGTGTTGCTTTTCTAGTTTTCCAGCGATGG
CGGAAAGGGAAACAACTAAAGCGCGAAGCAGAGAAGATTCAGTCCATGCTACAATTTATAA
AAGCAAATAAAAAAGGGGGAGATGAAGGGTCATTGCAGCCATAATAGGATAGCGGAGATG
TGCCTGCAAACGGGTGTCTCTGCTTGGGCTGAACATGCTTGCAAACGAGGCATTCCGCCAAG
GAGTCTGGACACAGCCTTGAGTTTAATGGTCCCTTGCAAACGAGGGAACATTCCCTTCTTGT
GATAAGAAGAAAGAAAGGGCTCTGGACAGAACTCTGCAGTAGACAGAGATTTCACTCCGCT
TGCTGTACGATAACATGTATGCACCTGCGCTGTACTGAAAAGGCTTATTCATGCAGTCTGGA
ATTCTGCCTAGGGGGCTTTATAAAAATAAATCACAATTAGTTTTGCGCAGCTTTTTTCCTCCG
GCCGGAGTGGTGTATTGTCTGTCTCTTGTGTGTCTCGTGTGTTTTGTCTTTGTGTCATTTCACT
CACAACA

>NC_056057.1:395192-403082#SHEEP_RIP_12(-)

AGGTGAAGGGTTAATAGGGTAGCAGAGATGTGCCTGCAAACGGGCCTCTTTGCTAGGGCTG
GATGTCCTTGCAAACGAGGAGTTCTGCCAAAGAGTCTGGACACAGCCTTGAGTTTAACGGTC
CCTTGCAAACGAGGGAGCATTCCCTTCTTGTGATAAAGAAGGAATAGAGGGCTTTGTACAGT
CTCTGCAGTAGACCAGGATTTTACTCCCCTTTGCTATACGATAACATGTATGCACCTGCACTG
TGCTGAAAAGGCTTATTCATGCAGTCTGGAATTCTGCCTAGGGGGCTTTTATAATAAACGGT
AATTAGTTTTTTGCCCACTTCTATTCCTCTGGCCAGAGTGTGCATGTTGTCTGTCTTTTGTGTG
TCTTGTTCTGTGTCATTTCACTTGTAATCTCCAACATCTGGCACCCAACGTGGGGCTCGAGTG
AAATCGAAAGGGTGAGTAACCCCGGGGGGGTTTTAAATCCATAGCAGGGGAACTTTCGGGA
AAATTATGGGGAATTCCTCACCCTAGCAGAGGGGAGCTTTCAAAAAAAAATCATGGGGAAT
TCCTCATCATCATTATGGGCACAATACATGGAGTTAGTCCAAGGACTTCTCCACTCCATAGG
CATTAAAGCCTCGACTTGTCGGTTGAGTGAGCTCTTTCGCTTAGTGGAGCAATATTGTCATTG
GTTTCAATATCAAACTAAGTTACAGTTAAACTTGAAGGAATGGAAAATAATTCAAAAGGAA
TTGAGAAAGCAACATCAGAAGGGTTATGTGATCCCTTTGAAGTTGTGGACTTTGTGTAGTGC
TATAACACAGGCTTTGACCTTGCTCTCTACTGATAATGAAACTAAATCTAATGCTTCAAGGA

GGGGAAAAATTTTATGAGGATGTGTCAGACGTTGGTGGGGCTTCTGCATCGCCTGAAGGCAA
GGATACAAATGAGCCTTCTCCTGTAAATGGTGAAACATCTGATAGTTCAGAATCAGATTTGG
AGGCTTCTTCGGTTTTGTCAGAGGAGGGCAAAGAGATTAAAGAAATGACCTATCTATTCCAG
GAATGGTGGAAATCCCATATGGAGGAGAAAAAATCTACACCTTCTGCTCCTCCTTGTGCTTC
TCTTTTCCCCTCTGTGGTTAATCAGCCCAATGTGGGCAGGGAACATTGTCGGTTCTCCTTTCC
TTTGTCTATGCTTCATGATGATGACTCGCCTGCTCCCCCTGGTGGGTTTATCGATCTTCCACA
ATTGTTTCCCATCCAGAGACAGCAGGATGGCAATGTGATAAATGTTCAATATGCTCCTTTGG
AATATAAATTTTTTAAAGATCTTAAAACTGCAGTAGTGCAGTACGGTCCTCAATCTCCCTTTG
TTTTGGCTATGCTGGAATCATTGGGAAAAGGCAAATTAATCATTTCATTAGATTGGGAATCT
ATTGTCCAAGCTGTCTTGGAGGGCTCTCAATGGTTGCAACTTCATAGCTGGTGGGAAGAAGA
AGCTAGAAAGCAGGTTCGGATTAATGAGAGACAGAATCCCCCTGATCCTCTTGAGGACAAG
CTAATGGGAGAGGGCCAATATCGGGCTTTAAGAGAACAGACTCAATACTCTGACCAAGACT
TACAACAAGTCCGCCAGGTCTTTTTACGAGCATGGCACCTACTGGCCACACCCAGCCCTCCT
TTGTTAAAACAATGCAAGGCCCCAATGAGCCATATACTGATTTTCTAGCAAGATTGAGGGTA
GCTGTGGAACAGGCTGTAGGGAGGAATGAGATTTCAGAGATATTATTACAAACTTTAGCATT
TGAAAATGCAAATCCTGAACTCAAGCATATACTGGGACCTTTAAAGGGACAGGGTGCATCG
ATAGCTGAATATATCAGAGCCTGCTCAGGAATAGGAGGGACTGAGCATTAGGCTAATGTCTT
TGCTACAGCCTTGGCCAAAGTTATGAGACCACCAAAGGGAGGTAACTGCTTTCATTGTGGAA
AACCTGGTCATATGAAAAGAGAGTGTCGGAAATTAAAAGTTGATCAAGGTGTAATTCCTAA
AGACAGATCTCTTGCTGGGAAGAATAAGACTCCTCCTGGACTTTGCCATCAGTGCAGGAAGG
GGTTTCATTGGGCTAATGAATGCAGATCTAAAACAGACAAAATGGCCAACCCAATACTGGG
AAACTATCCTGCAGGCCTAAGTCCTTGGGGCCCAGGAACAATACCGGGGACTTATCCTCCTT
GCCCTCCTCCCATCCCATCTGTCCCAACCCTATTCCCTCCCAACAACCGTTACGAGTCAATGC
CCTGTTAAAAGGACCTCAGATGATGATTTTGAACTTACGGTCTGCTACTTCAGGGAGTGCTG
CTGCTGATTTGCCACTAGCTGATAATGTTCTTTTGTCACCAGGGGAAGGCATTTATAAATTAA
AAACAAATGTATTTGGACCACTGCCTAAAGGCACTTTTGGCTTGATATTAGGCTGTAGCAGC
ACGGCTTTGAGAGGTTTAACCATATTTTCTGGGGTAATAGACTCTGATTATGTTGGGGAAAT
TTTAATTATGGTCTCTATTTCTATCACACTTTCATTGTTAGCTGGGGAACATATTGCTCAAAT
ACTTCTCCTACCTTATCACCCCTTTTTGGCTCTTCCTAATGAACAAACAGGAGGATTTGGAAG
TACTGGGCGACATATATTTTGGGAAATGCTTGTCAAAGATTCCCGCCCTGTTCTCTCCTTGAA
TATACAGGGAAACAACTTTGAAGGACTAGTAGACACAGGGGTGGATGTTTCAGCCATTTCTT
CTCAACAATGGCCCCAAGATTGGGAAAAAGAAAAAAGCCCTTTAATGCTGACGGGATTGGG
CTCCATTGCAGATGTCTGGAAGAGTACCCATCCCTTACAATGTCAATTCCATAATGGAAGAT
CAGTGTTTGTTACCTTTTATATTGTAAATATACCTATTAATATATGGGGAAGAGATCTTCTCT
CTCCTTTGGGGGCTTCTGTAACCATTCTATAGGAAAACTAGGAGCCACTGCTCAAATTCCTC
GAGCACTCCCATTAAAATGGTTAACTAATACTCCAAAATGGGTTGAGCAGTGGCCATTACCA
CAAATGAAGCTCGAGGCATTAGAATAATTAGTACAAGAACAACTCCAACTTGGTCATATAG
AGCCCTCTACCTCCCCCTGGAATTCTCCTGTTTTTGTTTTAAAAAATAAATCTAGAAAATGGA
GAATGTTAACTGATTTACGAAAAGTTAATAAATGTATTGAACCTATGGGAGCCTTGCAATTG
GTACTCCCCTCTCCAGCTCTTATTCCTCAGAATTGGTCCTTAATGGTGTTAGATCTTAAAGAC
TGTTTTTTTTTTTTTTACCATTCCCCTCCAATTGAAAGATAGAGATAAATTTGCTTTTACAGTT
CCTGTTCTTAATCATGCTCAGCCTGTTAAGCATTATCAATGGACAGTCTTACCACAAGGAAT
GATAAATAGTCCTACCTTATGCCAAGAATTTGTAGCTTGCTCTTTACAATCCCTCCATCAAGA
ATACGCCAATTATATTCTATATGATTATATGGATGATCTCCTATTGGCAGCTCCTAGTATTGC
TGAACGTGATGAATTCTTTTTAAAAGTACAGGAGGCTTTAAGACTATACAATTTGCAAGTAG
CCCCGTAAAAAATTCAAAAGGACTTTCCTATTTCATATTTAGGGACAATATTGGAACAACAT
AGAATTAGGCCCCAAAAGTTGCAAATTAGAAGAGACCATCTCAAAACCTTAAATGATTTTCA

AAAGTTATTGGGAGATATTAATTGGCTACACCTGGTACTTGGGATTCCTACTTATCAATTACG
ACATTTGTTTTCTACTTTAGAAGGAGACACAGCTCTGGATAGCCCCCCGGACCTTAACCCCA
TTGGCTTTACAGGAATATCAATTTGTTGAGCGACGACTAAATGACGGCTTTTTGACTTACTTA
CATGCATCTCAACCTATTTCTTTTGTAATATTTCATACCCCTTACTCCCTATCTGGTATAATTG
CTCAAGAAAAAGGATTAATAGAATGGGTTTTCTTACCTAACTGTTTTTCCAAAAAATTGACT
GTATATATGGACAAATTAGCCTTCCTTATACAGAAGGGTCACCATCATATTTTACAATTATCA
GGATGTGAACCACACCAGATTGTTACTCAGTTAATAACTGCTCAAATATCTCGATGTTTACA
ATTTAATGAAAACTGGCAAATTTCTCTTGCCTCATATCCTGGTTCATTTTCTAATCATTATCC
ATAATCTAAATTAATTGATTTTCTCTGGACTAACACTATGATATCTCATTCCCCGATTTCAGA
TGTTCCAGTAAAGGGACCCGCTATTTTTACAGATGCAAATAAAAATACTGCTGGATATTGGA
ACCTGGAAAATTCCAAGGTTCTCCCCCACTCATTTTCTTCTGTACAGCCCACTGAATTGTGGG
CTATCTATTTAGTTTTGCAAGATTTTCCCTAACTTCCTATTAACATTGTTTCAGATTCTCAATA
TGCTGTTCTCTCTTGCCTACAGCTTCCCCATGTCTCCCTTCCATTGACTCTTAAAACAGTTATT
GATAAATTGTTTTACCAAGTACAACAGTTGCTCTTGCAGCGTTTAGAGTTAATTTTCTTTAGT
CACATCCATGCACATTCTGCCCTTCCTGGACCCTTATCATTCAGAAATGCTACAATTGATGTC
TTACTTTATCCTATAGAAGCAGCAAAACAAGAACATCTCTGACAACATACCAACTCCAAAGG
GTTACAAAAATCCCATGCTATTACTCGAAAACAAGCTCAAAATATTGTTCATTCTTGTTCTAT
ATGTGCACCTTTTGTTTTGTCATTCACCCCACCAGGTGTCAACATAAGAGGACTACAAGCAA
ATCAGATATGGCAAATGGATGTAATTTACATTTCTTCCTTCGGACAACAAAAATGTGTGCAT
CATACTGTAGATAATTGCACACATTTTCAATGGGCCACTGCATTACATTCTGAAAAGGCTGA
TGCTGTTATTACTCATTTGTTGTCTTGTTTTGCAGTTATGGGATTACCAATTGAATTGAAAAC
TGCACCTGCTTACCAATCCGCAAAATTAGCTCACTTTTTATCTCAATACCATATAACTCATAC
TTTTTGTATTCCTTATAATAGTCAAGGGCAAGCTATCATGGAAAGAGCTAATCATAGCTTGC
ATGATTATCTTGAAAAAATAAAAAAGGGGGAACAAGAGAGATTTATGAAACTTAAAGACAT
TTTGAATAAAACCTTACTTACCCTACATTTTTGAATGTTTGGAGCAAGGGAAATCTATCAGC
AGCAGAGTTGCATTTTCTAGGGAAAGAAGAGGATAAAAAGATCTTGAATATGCCTATTTGGT
ATAAAGATAAAGAGAAAGGTTGGATCCCAGCATCATTAATATATTTGGGACGAGGGTATGC
TTTCATTTCTGTTGATAATTACAGGTTTTGGACCCCAGCAAGATTGATCTAAATCAACAATGG
CTGATCCCCTTGTTCAAAAATTCAAAGAGCTTACTATACAGAGAAGCCTTACTTCCCGTACA
AGGGATGCAACACCTCCTACATGGGGTCAAATGAAGAGGTTGACCCAGGAAGCAGAGAAGA
CGTTAATGAAGGCGGGGCAACCTCTGAATCCTACCAATCTTTTGCTTGCCATGATGGCAGTG
GTGACATGTCAGGTAATCGGTGTATCGGCAAGTAATCATACGTATTGGGCATATATACCTAA
TCCCCCATTAGTAAGAGCAGTTTCCTGGGGGGAACCAGAAGTGCAGGTATGTACTAATGAG
ACTGCCTTCTTTCCTCCGCTAGCTTGTGGGGGAATAGAACAACTATCTCATCATGAACAACA
ATATAATATTAGTAATTTGACCATTGCAGTAGAAGGTATTCCTTTATGTATAGGGGGACACC
CCTTTTGTCTGTCCACCAAGGAACATTCTCATCATTCTTATAAATACATACGGGGTGAAATAT
AATAATTACCATTTTGCTACTTTTACTGTGCTTGTTTCCACCAGGGGATTTAGCACCTCGACA
GAACTGATAGATATTCATAATGGGAAACACATGTCGCTATGTCCTGTTAACTTTTTTGTTCCT
TCTCTAGAATCTTTGGAGTGGGAACATTGCCGAGGTCATCGACCCTTTAAAGTCATGAATTA
TTCTGGGGCCATCATTGTAGATTGGAGTCCAGATCATGGGCAATTCTTAGAAAAATGGTCAA
ATAAATCTTTTAGGTGGCATCGTGCAAATAGCACTTTGATGGGCAATGGTAATGAAACAGTT
AAATGGCAGCAATTTGCACTTGTCCCTCCTCAATTACAATTGCAAGGATATCCGCACATTCA
AGGAGATATTTGGAAACTATGGGTGGTTTCTGGTAACCTCACTATCTGGTCAGGAAACTATG
CTTTGGACAGTGGTGACTCTTCAGGTCCATTCCATGTTAATTTACATGTTAATAAATCTTATT
CTGCAATGGCATGTGTAAAATATCCTTTTGCATTGTTATATGGGAATTGGACCTGGAATGAT
ACTGTGGTGTCTGTGTCATGTGACTATTGTAATCTAACTTAATGTGTAAATCAGTCTTGGTGG
GAAGAATTTGAAAGACCAACCTATAATTCCAACTTCTCGCTAGTAATTGTTAAGGCTCGGAC

AGAAGTATGGTTACCTATAAATCTGACTCTGCCGTGGTCAGATTCTTTTGCTGTTTCTCATCT
AGTAACCACTGTACAGACTTTGCTACACTGATCTCGAGGTATGCTTGCTGTGGTCATTGCTTC
GATTTTAGCAGTTGTGCCAGTAACTGCAACAGCAGCAGTAGCAGGTCTTGTGTTACACCAAG
GAATTCAAACAGCTGATTTTATTTGGGACTGGCATAAAGACTCTCATTAGTTATGGCAACAA
CAGTGAGATTTGGATGCCCAACTTGCTATCGATGTGCTCAATCTTGAACACACCATTTCCTGG
CTTGGAGATCAATCGGCTGTTTTATCTACACGAAGTGTGTTGAAATGTGATTGAAATTCTTCT
CAGTTTTGTATAACACCTGTACCATTTAACATGAGTGAAGGATGGGATAAAGTAAAACGATC
CTTGACTGGGCATCAAAATCTCACTATGGAGATTATGGACCTGGAAAGACAAATTTTGTCTA
CTTTTAGCAGGACTTTATCTGACATTATGGGGTCTGATTTGCTGAAAAGTCTTCAAGAGGGA
ATGAATAACTTAAATCCATTAGGGCACGTATCCTCACTAATTGGGACTACTTTTGGGAACAC
TGTGTTTATATTACTTTTATGTTGTGTTGCTTTTCTAGTCTTCCAGCGATGGCGGAAAGGGAA
ACAACTAAAGAGCAAAGCAGAGAAGATCCGGACCATGCTACAGTTTGTAAAAGCAAATAAA
AAAGGGGGAGATGAAGGGTTATTAAGGTAGCAGAGATGTGCCTGCAAACGGGCCTCTTTGC
TAGGACTGGATGTCCTTGCAAACGAGGCATTCTGCCAAAGAGTCTGGACACAGCCTTGAGTT
TAATGGTCCCATGCAAACGAGGGAGCATTCCCTTCTTGTGATAAAGAAGGAATAGAGGGTTT
TGTACAGACTCTGCAGTAGACTAGGATTTTACTCCCCTTTGTTATACGATAACATGTATGCAC
CTGCGCTGTGCTGAAAAGGCTTATTCATGCAGTCTGGAATTCTGCGTAGGGGGCTTTTATAA
TCAACGGCAATCAGTTTTTTGCCCAGTTCTGTCCCTCCGGCCGGAGTGTGCGTGTTGTCTGTC
TTTTTTATGTCTTGTTCTGTGTCATTTCACTTGTAATCTCCAACA

>NC_056057.1:41962471-41970401#SHEEP_RIP_13(-)

TGCGGGGGACGACCCGTGAAGGGTTAAGTCTTGGGAGCTCCCTGGCAGGTATGCCAGGCCC
TAGGACACGTGCCTAAGCTCCCTGTCCCGCCACCCTCAAGAGTTTTTATAACCCTTAAGGCTC
CAAGATGTTTGGTTTCGGCAACATTTCATAGAAGATAGATTATCTTATTGTGTATATTTCATA
GAAGATAGATATTCTGATTGTGTTCTGTATACAATGGTAAGGGTCTGGTGATTGTATCCTGA
GATTAAAAAACAACCTTGTGAGTGCCTTAAGTCACGTACTTTACCCTATATATACTGCAGCA
CAATAAAGCAAGGTATCAGCCATTTTGGGGCTGATCCTCTCAACCCCATCTTTTGTCTATCTC
TTATTTTCTTAGCGGGGACGCTCCGTTCTCTCCCTGTGCAGGTGCGACTCTTGCTTGTGCTGG
CCGCGGCAGGTGGCGCCCAACGTGGGGCTCGAGCTCGACAGTTTTCCTCGCCACTACTCTTA
TTAATTGAAAAGAGTGAGTATATGAGTAAACAAGTGAATTAAATTGAGGAGGAGTAGTAAG
GTATATAGTTGAGAGTATAAATATGGGACAGACGCATAGTCGCCAGTTGTTTGTGCATATGT
TATCTGTAATGTTAAAACATAGGGGAATTACTGTTTCTAAACCTAAATTAATCAATTTTCTTT
CATTCATCGAGGAAGTTTGCCCTTGGTTCCCCAGAGAAGGTACAGTAAATTTGGAGACATGG
AAGAAGGTAGGGGAACAAATTCGGACTCATTATACTTTACATGGCCCTAAAAAAATCCCTGT
CAAAACTTTATCCTTTTGGACACTAATTCGTGACTGCCTGGACTTTGATAATGATGAATTAAA
ACGTTTAGGAAATTTATTAAAACAGGAAGAAGATCCTCTCCATGTTCCTGATTCGGAACCCA
GATATGCTGTTCCCGAGGGGGTTAAAAGCGACCCTCCGTTTTCTAACTTATTGCATCCTTCAG
ATAATGATGATTTACTTTCATCCACAGATGAGGCAAAATTAGACGAAGAAGCTGCTAAATAC
CATCAAGAAGATTGGGGTTTTTTAGCACAAGAAAAGGGGCGTTAACATCTAAAGATGAATT
GGTTGAATGCTTTAAAAACCTCACTATTGCTTTACAGAACGCAGGAATCAAGCTTCCTAGTA
ACAATGCCAAATCTCCTTCTGCTCCGCCTCTTCCCCCTGCTTATGCTCCTTCTGTTGTGGCTGG
TCTCGATCCCCCTCCAGGGCCCCCTCCACTGTCTGAGAACATGTCTCCGCTGCAAAAGGCAT
TGAGACAGGCACAGCGACTTGGTGAGGTTGTCTCTGATTTTTCTCTTGCTTTTCCTGTCTTTG
AAAATAACAACCAGCGTTATTATGAATCACTGCCTTTTAAACAACTGAAAGAGTTAAAGATT
GCTTGCTCACAATACGGTCCTACCGCTCCATTCACCATTGCTATGATAGAAAATTTGGGTACT
CAAGCTTTACCTCCAAATGATTGGAAGCAGACAGCTAGGGCATGTCTCTCAGGGGGAGATTA
TTTATTATGGAAATCTGAATTTTTTGAACAATGTGCTCGTATAGCTGATGTTAACCGACAGCA

AGGTATACAGACCTCCTATGAAATGTTGATTGGTGAAGGCCCTTACCAGGCTACTGATACTC
AACTTAATTTCTTACCTGGTGCATATGCACAAATATCAAATGCGGCTCGGCAGGCATGGAAA
AACTTCCTAGCTCCAGTACTAAGACAGAGGATCTTTCAAAAGTCCGGCAGGGACCTGATGAG
CCTTACCAGGACTTCGTGGCACGACTTTTAGATACTATAGGTAAGATAATGTCAGATGAAAA
GGCTGGGATGGTACTGGCAAAACAATTGGCTTTTGAAAACGCTAACTCTGCTTGTCAAGCTG
CTTTAAGACCTTATCGAAAAAAGGGAGATCTGTCTGATTTTATTCGCATTTGTGCTGACATTG
GACCCTCCTACATGCAAGGCATTGCTATGGCAGCAGCATTACAAGGAAAAAGCATAAAAGA
GGTACTTTTCCAGCAGCAAGCCCGGAACAAGAAAGGACTTCAAAAGTCAGGTAATTTGGGT
TGCTTTGTTTGTGGTCAGCCTGGCCATCGGGCTGCAGTGTGCCCTCAAAAACAACAAAGCCC
TGTTAACACTCCTAATTTGTGCCCACGCTGTAAAAAAAGGAAAGCATTGGGCGCGGGATTGC
CGTTCCAAAACGGATGTTCAAGGTAATCCTTTGCCCCGGTTTCGGGAAACTGGGTGAGGGCC
AGCCCTGGCCCCGAAACAATGTTATGGGGCAACACTGCAGGTTCCAAAAGGACCATTGCAG
ACCTCTGTCGAGCCACAAGAGGCAGCGCGGGATTGGACCTCTGTGCCACCTCCTACACAGTA
TTAACTCCCGAGATGGGGGTCCAAACCCTTGCCACAGGAGTGTTTGGGCCTTTACCTCCAGG
GACAGCTGGACTGCTTTTAGGGCGCAGCAGTGCGTCTTTAAAAGGAATACTTATTCATCCTG
GTGTGATTGACTCTGATTATACAGGAGAGATAAAAATATTAGCCTCCGCTCCTAACAAAATT
ATTGTAATCAATGCAGGACAGCGTATAGCTCAACTTCTTTTAGTTCCATTAGTCATACAGGG
AAAAACAATTAACCGAGACCGTCAAGATAAAGGTTTCGGGTCCTCTGACGCCTATTGGGTGC
AAAATGTTACCGAGGCACGACCAGAACTTGAGCTACGCATTAATGGTAAGCTTTTCCGAGGA
GTGCTTGATACAGGGGCCGATATTAGTGTTATTTCTGATAAATATTGGCCTACTACATGGCC
AAAACAGATGGCTATTTCCACTCTCCAGGGTATTGGCCAAACTACCAATCCAGAACAGAGTT
CATCCCTTCTTACTTGGAAAGATAAAGATGGACATACAGGCCAATTTAAACCTTATATTCTG
CCCTATCTTCCAGTTAATCTATGGGGGCGTGATATATTGAGCAAAATGGGTGTTTATTTATAT
AGTCCTTCACCCACTGTGACAGATTTGATGTTAGATCAGGGCTTACTTCCAAATCAAGGTTTA
GGTAAACAACATCAAGGCATCATTTTGCCCCTTGATTTAAAAATCTAATCAAGATCGAAAAG
GCTTGGGGTGTTTTCCTAGGGACCTCTGATTCTCCTGTGACACATGCCGATCCTATTGATTGG
AAATCTGAGGAACCGGTATGGGTCGATCAGTGGCCCCTAACACAGGAAAAACTTTCTGCCG
CACAACAGCTGGTGCAGGAACAGCTGAGACTTGGGCATATTGAACCCTCTACCTCTGCTTGG
AATTCCCAATTTTTGTTATTAAAAAGAAGTCTGGGAAATGGAGATTGCTACAAGATCTTCGT
AAGGTAAATGAAACAATGATGCATATGGGAGCCCTACAACCTGGGTTGCCCACTCCTTCTGC
TATACCTGATAAATCCTATATCATTGTTATAGATTTAAAAGATTGTTTTTACACTATTCCTCTT
GCACCTCAAGATTGCAAAAGATTTGCTTTCAGTTTACCCTCTGTTAATTTTAAAGAGCCTATG
CAACGCTATCAATGGAGAGTTCTCCCGCAAGGAATGACTAATAGCCCTACGCTGTGCCAAAA
ATTTGTTGCTACAGCAATAGCTCCGGTTCGTCAACGTTTTCCTCAGCTATATTTGGTTCATTA
TATGGATGATATATTACTAGCTCATGCTGACGAACATCTATTGTATCAAGCTTTTCGATTCTA
AAACAACATTTAAGCCTTAATGGTCTTGTTATTGCTGATGAAAAATTCAGACTCATTTTCCTT
ATAATTATTTGGGTTTCTCCTTATATCCTCGTGTTTATAATACCCAATTAGTACAATTACAGA
CTGACCATTTAAAAACTCTAAATGACTTTCAAAAACTTTTAGGAGACATTAATTGGATACGT
CCTTATTTAAAATTACCCACTTATACCTTGCAGCCATTATTTGACATCCTTAAAGGTGACTCT
GATCCTGCGTCACCCCGAACACTTTCTTTAGAAGGACGAACTGCTTTACAATCAATAGAAGA
AGCTATTAGACAACAACAGATTACTTATTGTGATTACCAACGATCATGGGGTTTGTATATAC
TTCCTACCCCCCGAGCACCCACAGGGGTTCTCTATCAAGATAAACCTTTGCGATGGATATAT
TTGTCTGCTACTCCAACTAAACATCTGCTCCCTTACTATGAACTTGTTGCAAAATTGTAGCAA
AGGGACGTCACGAGGCCATCCAATATTTTGGTATGGAACCCCCTTCATTTGTGTTCCTTATGC
TTTAGAACAACAAGATTGGCTTTTTCAATTTTCAGATAATTGGTCTATAGCTTTTGCAAATTA
CCCGGGACGGATTACTCATCATTACCCTTCTGATAAATTGTTACAATTTGCTAGCTCTCATGC
CTTTATTTTTCCAAAAATAGTTCGCCGACAACCTATTCCCGAAGCGACACTTATATTTACAGA

TGGATCTTCTAATGGAACTGCAGCTTTAATCATTAACCATCAAACCTATTACGCACAAACCA
GTTTTTCTTCTGCTCAAGTTGTGGAATTATTTGCAGTCCACCAAGCGTTGCTAACTGTACCTA
CTTCCTTCAATTTATTTACAGACAGCTCCTATGTGGTCGGTGCCTTACAGATGATTGAAACTG
TTCCAATTATCGGCACCACCTCTCCTGAAGTTCTTAACTTATTTACATTGATTCAACAGGTTC
TCCATTGCCGCCAACACCCCTGTTTCTTTGGACATATTCGTGCACACTCCACCCTTCCTGGTG
CCCTCGTACAAGGCAATCACACTGCGGACGTTCTTACTAAACAAGTGTTTTTCCAATCAGCT
ATTGATGCAGCCCGAAAATCCCATGATTTACATCACCAAAATAGTCAGTCTTTACGCTTGCA
ATTTAAAATTTCCCGTGAAGCTGCACGGCAAATTGTTAAATCTTGCTCTACTTGTCCTCAATT
CTTTGTTCTCCCTCAATATGGTGTCAACCTCGAGGTTTACGCCCTAATCACCTCTGGCAAACA
GATGTTACTCACATTCCTCAATTTGGGCGTCTTAAATATGTTCATGTTTCTATTGACACTTTTT
CCAATTTTCTCATGGCTTCCCTTCACACTGGAGAATCAACACGTCACTGTATTCAACATTTGC
TGTTTTGCTTTTCTACTTCAGGAATCCCACAAACCCTTAAAACAGATAATGGACCTGGTTATA
CTAGCCGTTCTTTTCAACGTTTTTGTCTTTCTTTCCAAATTCATCATAAAACAGGAATTCCTTA
TAATCCACAGGGACAAGGTATTGTGGAACGAGCCCATCAACGCCTTAAACATCAATTATTAA
AACAAAAAAAGGGGAATGAACTGTATAGCCCCTCACCGCATAACGCCTTAAACCATGCTCTT
TATGTTTTAAATTTTTTAACTTTAGACGCAGAAGGCAATTCAGCAGCCCAGCGTTTTTGGGGA
GAACGATCCTCATGCAAAAAACCACTTGTGCGATGGAAGGATCCACTTACCAATCTGTGGTA
TGGGCCAGACCCTGTACTAATATGGGGACGAGGGCATGTTTGTGTTTTTCCACAGGATGCCG
AAGCGCCGCGCTGGATTCCGGAAAGGCTGGTACGCGCAGCAGAGGAACTCCCTGACACATC
AAATGCAATGCATGACACTGAGTGAGCCCACGAGTGAGCTGCCTACCCAGAGGCAAATTGA
GGCGCTGATGCGTTATGCTTGGAATGAGGCTCATGTACAACCTCCAGTGACACCTACTAATA
TACTGATCATGTTATTATTATTGTTACAGCGGATACAAAACGGGGCAGCTGCGGCTTTTTGG
GCATACATTCCTGATCCGCCTATGATTCAATCCTTAGGATGGGATAAAGAAACAGTACCTGT
ATATGTTAATGATACAAGTCTTTTAGGAGGAAAATCAGATATTCACATTTCTCCTCAGCAAG
CCAATATCTCCTTTTATGGTCTTACTACTCAATACCCTATGTGCTTTTCTTATCAATCACAGCA
TCCTCATTGTATACAGGTGTCAGCTGATATATCCTATCCTCGAGTGACTATTTCAGGCATTGA
TGAAAAAACCGGAAAGAGATCGTACCGTGACGGAACCGGACCCCTCGACATTCCGTTTTGT
GACAAACATTTAAGCATCGGCATAGGAATAGACACTCCTTGGACTTTATGTCGAGCACGAAT
TGCATCGGTGTATAACATCAACAATGCCAATACCACCCTTTTATGGGACTGGGCACCTGGAG
GAACACCTGATTTCTCCGAATATCGAGGACAGCATCCACCCATTCTTTCTGTAAACACTGCTC
CTATATTTCAAACTGAACTGTGGAAACTTTTGGCTGCTTTTGGTCATGGTAATAGCCTATATT
TACAGCCCAATATTAGTGGGAGTAAATATGGTGATGTGGGAGTTACAGGATTTTTATATCCC
CGAGCTTGTGTTCCTTACCCATTCATGTTGATACAAGGCCATATGGAAATAACACTGTCATTG
AATATTTATCATTTAAATTGTTCTAATTGCATACTTACTAATTGCATTAGAGGTGTAGCCAAA
GGAGAACAAGTTATAATAGTAAAACAACCTGCTTTTGTAATGTTACCTGTTGAAATAACTGA
AGAATGGTATGATGAAACTGCTTTAGAATTGTTACAACGCATTAATACGGCTCTTAGCCGTC
CTAAAAGAGGTCTGAGCCTGATTATTCTGGGTATAGTGTCTTTAATCACCCTTATAGCAACTG
CTGTTACTGCTTCTGTATCTTTAGCACAATCCATTCAAGCTGCTCATACTGTAGATTCCTTGTC
ATATAATGTTACTAAAGTAATGGGAACTCAAGAAGATATAGATAAAAAATAGAAGATAGAT
TATCAGCTTTATATGATGTAGTTAGAGTTCTAGGAGAACAAGTTCAGAGCATTAATTTTCGC
ATGAAAATTCAATGCCATGCTAATTATAAATGGATTTGTGTTACAAAAAGCCTTACAATACT
TCTGACTTTCCGTGGGATAAGGTGAAAAACATCTGCAAGGAATTTGGTTTAATACTAATGTT
TCTTTAGATCTTTTACAATTGCATAATGAAATTCTTGACATCGAAAATTCTCCAAAAGCTACT
TTGAATATAGCTGATACCGTCGATAATTTTTTACAAAATTTATTTTCTAACTTTCCTAGCCTTC
ATTCACTGTGGCGAAGTATAATTGCTATGGGCGCGGTTCTGACTGTTGTGCTTATCATAATTT
GTTTAGCTCCTTGCCTTATTCGTAGCATTGTTAAAGAATTTCTACATATGAGAGTTTTAATAC
ATAAAAACATGTTGCAACACCAACATCTTATGGAGCTTTTAAAAAATAAAGAGAGGGGAGC

TGCGGGGGACGACCCGTGAAGGGTTAAGTCTTGGGAGCTCCCTGGCAGGTATGCCAGGCCC
TAGGACACGTGCCTAAGCTCCCTGTCCCGCCACCCTCAAGAGTTTTTATAACCCTTAAGGCTC
CAAGATGTTTGGTTTCGGCAACATTTCATAGAAGATAGATTATCTTATTGTGTATATTTCATA
GAAGATAGATATTCTGATTGTGTTCTGTATACAATGGTAAGGGTCTGGTGATTGTATCCTGA
GATTAAAAAACAACCTTGTGAGTGCCTTAAGTCACGTACTTTACCCTATATATACTGCAGCA
CAATAAAGCAAGGTATCAGCCATTTTGGGGCTGATCCTCTCAACCCCATCTTTTGTCTATCTC
TTATTTTCTTAGCGGGGACGCTCCGTTCTCTCCCTGTGCAGGTGCGACTCTTGCTTGTGCTGG
CCGCGGCA

>NC_056058.1:32045606-32053675#SHEEP_RIP_14(+)

ATAACATTTATGCACATGCGCTGTACTGAGAAGGCTTAGTCATGAAGTCTGGAATTCTGCCA
AGGGGGCTTTTTATAAAAATAAACTGCAGCTTGTTTGCGCAGTTCTTTTCCTCCAGCTGGAGT
TGTGTCTTTGTCTGTTTCTTGTGTGTGTGTCTTGTCTTTGTGTTATTTCGCTCGCAACATCTGG
CGCCCAACGTGGGGCTCGAGTGAAATGAAAGGGTGAGTAACCCCGGGAGGATTTTATATCC
ATAGCAGGGGAACTTTCGGGAAAATCATGGGGAATTCCTCATCATTACGGACACAATACAT
GGAATTGGTCAAAGGACTTCTCCCCTCCATAGGCGTTAAGGCCTCGACTCGCCGATTGAGTG
AGCTCTTTCGCTTGGTGGAGCAATATCGTCATTGGTTTCAATATCAAACTAAGTTACAGTTAA
ATTTGAAGCAGTGGAAAATAATCCAAAAAAAAAAAAAAAATGAGAAAGCAACATCAGAAG
GGTAATGTGATCCCTTTAAAGTTATGAACTTTATGTAGTGCTATAACACAGGCTTTGACTTTG
CTCTCTACTGATAGTGAAACTAAATCTAATATTTCAATGAAGAGAGAGGCAATTTATGAGGA
TGTGCCAGACGTTGGTGGGGCTTCTGCACCGCCTGAAGGTAAGGATACAGGTGAGCCTCCTC
CTGTAAATGGTGAGACATCTGATAGTTCAAAATCAGATTCGGAGGCTTCTTCGGTTTCATCA
GAGGAGGGCAAAGAGATTAAAAAAATACCCATCTATTCCAGGAGTGGTAGAAATCCCATTA
AAGAGCAGAAGAAATTTACACCTCCTGCTCCTCCTTATGCTTCTCTTTTCCCCACTGTGGTTG
ATCGGCCGGATGTAGGCAGGGAACATTGTCAGTTCTCCTTTCCTTTGCCTACGCTTCATGACG
ATGACTTACCTGCTCCCCCTGGTGGATTTATCGATCCTCCGTAATTATTTCCTATTCAGAGAC
AGCAAAATGCCAATGAGATAAATGTTCAATATACTCCTTTAAAATATAAATTTTTAAAAGAT
CTTAAAGCTGCAGTAGCACAATATGGTCCTCAATCCCCCTTTGTTTTGGCTATGTTAAAATCA
TTGGAAAAAGGCAAATTAATTATTCTGTTAGATTGAGAATCCATTGCCCAAGCTGTCTTGGA
GGGTTCTCAATGGTTGCAACTTCGTAGTTGGTGAGAAGAAAAAGCTAAAAAGCAAGCTCAG
ATTAATAAAAGACAAAATCCCCCTGGTCCTCTCAAGGACAAGCTAATGGGAGATGGCCATT
ATCGAGCTTTAAGAGAACAGGCTCAATACTCTGACCAGGACTTACAACAAGTTCGCCAGGTC
TTTTTACCAGCATGGCGCCATGTGGTGCCTACTGGCCACGCCCAGCCTTCCTTTGTCAAAACC
ATACAGGGCCCCAGTGAGCCATATACTGATTTTCTAGCAAGATTGAGGGTAGCTATGAAATG
AGCTGTAGGGAGGGATGAAATTTCAGAGATATTATTACAAACCTTAGCATTTGAAAATACAA
ATCCTAAATGTAAGCGTATACTGGGGCCATTAAAGGGACAGGGTACATCTGTGGCTAAATAT
ATCAGAGCCTGCTCGGGAATAGGAGGAAGAGAGCATCAGGCTAATGTCTTTGCTACAGCTTT
GGCCAAAGCTCTGAGACCACAAAAGGGAGGTAACTGCTTCCATTGTGGAAAACCTGGTCAT
ATGAAAAGAGAATGTCAAAAATTAAAAGCTGATCAAGATGCAATTCCTAAAGACAGATCTC
TTGCTGGAAAAAATAAGACTCCTCCTAGACCTTGCCATCGGTGCGGGAAGGGGCTTCACTGG
ACTAACGAGTGCAAATCTAAAACAGACAAAATGGGCAACCCGATACCGGAAAACTATCCTG
TGGGCCTAAGTCCTTGGGGCCCAAAAACAATACCAGGGACTTCTCCTCCCTGCCCTCCTGCT
GTCCCACCTACCCCAACCCTGTTCCCCTCCAACAACCATTACGAGTCAATGCCCCATTAAAA
GGACCTCAAATGATGATTTCAGATTTGCGGTCTGCTACTTCAAGGAGTGCTGCTGCTGATTTG
CCACTAGCTGAAAATGTTCTTTTGTCACCAGGAGGAAACATTTATAAGTTAAAAAAAAAATGT
ATTTGGACCACTGCCTAAGGGCACTTTTGGCTTGATATTAGGCCATAGCGGTGTGGCTTTGA
GAGGTCTAACCATAATTCCTGGGGTAATAGACTCTGACTATGTTGGAAACATTTTAATTATG

GTTTTTACTTCTACCACGCTTTCATTGTTGGCTGGGGAGCATATTGCTCAAATACTTCTCCTA
CCTTATCATCCTTTTTTGGCTCTTCCTAATAAACGAACAAGAGGCTTTAAAAGTACTGGGTGA
CATATATTTTGGAAAATGCTTATCAAGGATTCTCACCCTGTTCTCTCCCTGATTATACAACAA
CAACAACAAAAAATCTTTGAGGGACTAGTAGACACAGGGGCGGACATTTCAGTCATTTCTTC
TCAACAATGGCCCCAAGGTTAAAAAAAAAAAAAAAAAAAAAAAAAAAAGCCCTCTAATGC
TGACGGGACTGGGCTCAATTGCAGATGTCTGGAAGAGTACCCATCCCTTGCAATGCCAATTC
CATAATGGAAGATCAGTGTTTGTTACCTTTTATATTGTAAACATACCTGTCAATATATGAGGA
AGAGATCTTCTCTCTCCTTTGGGGGCTTCTGTAGCCATCCCATCGGAAAACTAGTAGCCACTG
CTCAAATTCCTTGAACACTCCCATTAAAATGGTTAACCAATACTCCAAAATGGGTTGAGCAG
TGGCCATTACCACAAATGAAGCTCGAGGCATTAAAACAATTAGTACAAAAACAACTCCAAC
TTGGTCATATAAAACCCTCTACCTCCCCTTAAAATTCTCCTGTTTTTGTTATTTAAAAAAAAT
ATATAAAAAATAAAATATGTTAACCAATTTAAGAGAGGTTAATAAATGTATTAAACCTATGA
GAGCATTACAATTGGGACTCCCCTCTCCAGCTCTTATTCCTCAAAATTGGTCCTTAATGGTGA
TAGATCCTAAAGACTTGTTTGTTTTTGTTTTTTTTTAACCATTCCTCTACAATTGCAAGATAGA
GATAAATTTGCTTTTACAGTTCCTGTTCTTAATCATGCTCAGGCTGTTAAGCGTTATCAATGG
ACAGTCTTACCACAGGAAATGATAAATAGTCCTACCTCATGCCAAAAATTCGTAGCTCATTC
TTTACGACCTGTCTGTCAAAAATATCCCAATTATATTTTATATCACTATATGGATGATCTCCT
ATTAGCAGCTCCTAGTATTGCTAAACGTAATGAATTCTTTCTAAAAGTACAGGAGGCTTTAA
GACAACACAATTTGCAAATAGCTCTGGAAAAAATTCAAAAAGACTTTCCTATTTCGTATTTA
TGGACGGTATTAGAACAACATAAAATAAGGCCCCAAAAGTTGCAAATTCAAAGAGACACAT
CAAAATCTTAAATGATTTTCAAAAGTTATTGAGAGATATCAATTGGCTACACCTGGTACTTG
GGATTCCTACTTATCAATTACGGCATTTGTTTTCTACTTTAGAAGGAGATACAGCTCTGGATA
GCCCCCGGACATTTACCCCATTGGCTTTACACGAACTTCAATTTGTTGAGCAACAACTAAAT
GAAGGCTTTTTGACTTACTTACATGTGTCTCAACCTATTTTGTTTGTAATATTTCATACCCCTT
ATTCTCCATCTGGTGTAATCACTCAAAAAAAAAAAAAAAGGATTAATAAAATGGGTTTTTCT
TACCTAACAGGTTTTCTAAAAAATTGACTACATATATGGATAAATTAGCATTCCTTACACAG
AAAGGTCGCCATCGTATTCTACAATTGTCAGGATGTGAACCACACCAGATTGTTACAAAATT
AACAACTGCTCAAATATCTCGATGTTTACAATTTAACGAAAACTGGCAGATTTCTCTTGCCTC
ATTTCCTGGTTCGTTTTCTAATCACTATCCATCATCTAAATTGATTGATTTTCTTCGAACTAAC
TCTATGATATCTCAATCCCCAATTTCAGATGTTCCCATTAAGGGACCCACTATTTTTACTGAT
GCAAATAAAAATACTGCTGGATATTGGACCCCAGAAAGTTCTAAGGTTCTCCCCCACTCATT
TTCTTCTGTACAGCCCACTGAGTTGTGGGCTATCTATTTAGTCTTGCAAGATTTTCCCCGACT
TCCTATTAACATTGTTTCAGACTCTTGATATGCTGTTCTCTCTTGCCTACAGCTTCCCCATGTC
TCCCTTCTGCTGACTCTTAAAACAGCTATTGATAAATTGTTTACCAAGCACAACAATTGCTCT
TGCAGCATTCAGAGTTAATTTTCTTTACTCACATCCGTGCACATTCTGCCCTTCCTGGACCCT
TATCATTCGGAAATGCTACAATTGATGCCTTACTTTATCCTATAGAAGCAGCAAAACAAGAA
CATCTCTTACAGCATACCAATTCCAAAGGGTTACAAAAATCTCATGCTATCACTCGACGACA
AGCTCAAAATATTGTTCGTTCTTGTTCCATATGTGCACCCTTTGCTTTACCATTTACCCCACCA
GGTGTCAATGTGAGAGGACAACAAGCAAATCAGATATGGCAAATGGATGTAATTTACATTT
CTTCTTTTGAACAACAAAAATGTGTGCATCATACTATAGATACTTGCACACATTTTCAATGGG
CCACTGCATTACATTCTGAGAAGGCTGATGCTGTTATTGCTCATTTGTTATCTTGTTTTGCAG
TTATAGGACTACCAATTGAACTGAAAACTGATAATGCACCTGCCTAACAATCCGCAAAATTA
GCTCACTTTTTATCCCAATACCATATAACTCATACTTTTGGTATTCCTTACAATAGTCAAGGG
CAAGCTATCATTGAAAGGGCTAATCGTACCTTGCATGAATATCTTGAAAAAATAAAAAAGG
GGGAACAGGAGAGATTTATGAAACCAAAAGACATTCTGAATAAAACCTTACTTACCCTAAA
TTTTTTGAATATTTGGAGCAAGGGAAATCTACCAGCAGCAGAGTTGCATTTTCAAGGGGAAG
AAGAGGATAAGAAGATCTTGAATATGCCTATTTGGTATAAAGATAAAGATAAAGGTTGGAT

CCCAGCATCATTAATATATCTGGGATGAGGGTATGCTTTCATTTCTGTTAATAATTACAGGTT
TTGGACCCCAGCGAGACTGATCAAAATCAACAATGGCTGATCCCTTTGTTCAAAAATTTGAA
GAGCTTACTATGCAAAAAAGCTTTACTTTCTTTACAAGGGAAGCAACACCTCCTACGTGGGG
TCAAATGAAAAAGTTGACCCAGGAAGCAGAGAAGACGTTAATGAAGGCGGGACAACCTCTG
AATCCTACCAACCTTTTGCTTGCCATGATGTCGGTGGTAACATGTCAGGTAATTGGTGTATCA
GCAAGTAATTATACATATTGGGCATATGTACCTAATCCCCCATTAGTAAGAGCAATTTCCTG
GGGGGAACCAGAAGTGCAGGTATGCACTAATGAGACTGCCTTCTTTCCCCCGCCAGCTTGCG
GGGGAATAGAACAACTATCTCATCATAAACAACAATATAATATTAGTAATTTGACCACTGCA
GTGGAAGGTATTCCTTTGTGTATAGGAGGACATCCCTTTTGTCTGTCCACCAAGGAACATTCT
CATCATTCCTGTAATACATGGGGGGTAAAGTATAATAATTATCATTTTGCTACTTTTACTGTG
CTTGTTTCCACCAGGGGATTTAACACCTCGACAGAACCGATAGACATTCATAATGGAATACA
TATGTCACTATGTCCTGTTAACTTTTTTGCCCCTTCTTTATTTTTTTTTTCTTCTCAAACAGTTT
AATAGCAAATAAACAAAGGAAGGTACCACACTTGGCAGATACAAAATGACTTTTTGTCCGT
GTGTCTGTGCAATTAAAACAGATTTTAGGTTCCCCATTGGGACAAAAGGAAAAACACACAA
AAAAGGAAGATTTCCTTTTAAACACATACTCCCTTGCTCCAAACTTGTAACAATTTTTTTTTT
CTTTTTTAATCCACTACATAAACTCTGTGATCAGGTCAGAAAAGCTACAGGGGCTCTTCTTGC
CCTTTTTTTTTTTTTTTTAAACCATTAAAGTAAAATCCATAATTTTCTACATAGTACAACACAA
GTTCACACAAAAAAGACATTTTCTTTTGCAAATCAAAACAGGTTTAGAATCTTTGGAATGGG
AACATTGCTGAGGTCATCGACCCTTTAAAGTCATTAATTATTCTGGGTCTATCATTGTAGATT
GGAGTCCAGATCATGGGCAATTCTTAGAAAAATGGTCAAATAAATCTCTTAGGTGGCATCGT
GCAAATAGCACTTTGATGGGCGATGGTAACGAAACAGTTAAATGGCAGCAATTTACACTTGT
CCCTCCTCAATTACAATTGCAGGGATATCCGCACATTCAAGGGGATATTTGGAAACTATGGG
CAGTTTCTGGTAATCTCACTATATGGTCAGGAAACAATACTTTGGATAGTGGCGACTCCTCG
GGTCCATTTCATGTTAATGTACATGTTAATAAATCTTATTCTGCAATGGCATGTGTAAAATAT
CCTTTTACATTGTTATATGGGAATTGGACTTGGAATGATACTGTGGGGTCTGTATCATGTGAT
TATTGTAATCTAACTCAATGTGTAAATCAGTCTTGGTGGGAAGAATTTGAAAGACAAGCTAA
TAACTCCAATTTTTCATTAGTAATTGTTAAAACTCGGACAGAAGTATGGTTACCTATAAATCT
GACTCGGCCGTGGTCAGATTCTTTTGCTGTTTCTCATCTAGTAACCGCTGTACAGACTTTGCT
ACATCGATCTCGACGTATGCTTGGTGTGGTCATTGCTTCAATTCTAGCAGTTGTGTCAATAAC
TGCAACAGCAGGAGTGGCAGGCCTTGCATTACACCAAGGAATTCAAATAGCTGATTTTGTTC
GGGACTGGTATAAAGACTCTCATTTGTTATGGCAATAACAGCGAGATTTGGATGCACAACTT
GCTACCGACATGCTCAATCTTCAATACACCGTTTCCTGGTTTGGGGATCAATTAACTGTTTTA
TCTACCCTAAGTGTACTGAAATGTGATTGAAATTCTTCTCAGTTTTGTACAATACCTGTACCA
TTTAACATGAGTGAGGGATGGGAAAGAGTAAAACGATCTTTGACTGGGCATCAAAATCTCA
CTACAGAGATTATGGACCTGGAATGACAAATTTTGTCTACTTTTAGCAGGACTTTACCTGAT
ATTACGGGGTCTGATTTGTTGAAAAGTCTTCAAGAGGGAATGAATAACTTAAATCCATTAGG
GCATGTATCCACACTAATTGGGACCACATTTGGGAACACTGTGTTTATGTTACTTTTATGTTG
TATTGCTTTTCTAGTCTTCCAGCGATGGCGGAAAGGGAAACAACTAAAGCACAAAGCAGAG
AAGATCCAGACCATGCTGCAATTTATTCAGGCAAATAAAAAAGGGGGAGATGAAGGAAGTT
AATATGGCCACATAGGGAAGTGGAGATGTGCCTGCAAACGGGACTCTCTGCTCGGGCGGAA
CATGCTTGCAAACAAGATGTTCTGCCAAGGAGTCTGGACACAGCCTTGAGTTTAATGGTCCC
TTGCAAACGAGGGAACATTCCCTTCATGTGATAAGGAGGAAGAAAGGGCTCTGGACAGACT
CTGCAGTAAACAGGAATTTCACTCCCTTTTGCTGTACGATAACATTTATGCACATGCGCTGTA
CTGAGAAGGCTTAGTCATACAGTCTGGAATTCTGCCAAGGGGGCTTTTTATAAAAATAAACC
GCAGCTTGTTTGTGCAGTTCTTTTCCTCCGGCCAGAGTTGTGCCTTTGTCTGTTTCTTGTGTGT
GTGTCTTGTCTTTGTGTCATTTCGCTCGCAACATTT

>NC_056059.1:14160260-14168178#SHEEP_RIP_15(+)

TGCGGGGGACGACCCGTGAAGGGTTAAGTCTTGGGAGCTCCCTGGCAGGTATGCCAGGCCC
TAGGACACGTGCCTAAGCTCCCTGTCCCGCCACCCTCAAGAGTTTTTATAACCCTTAAGGCTC
CAAGATGTTTGGTTTCGGCAACATTTCATAGAAGATAGATTATCTTATTGTGTATATTTCATA
GAAGATAGATATTCTGATTGTGTTCTGTATACAATGGTAAGGGTCTGGTGATTGTATCCTGA
GATTAAAAAACAACCTTGTGAGTGCCTTAAGTCACGTACTTTACCCTATATATACTGCAGCA
CAATAAAGCAAGGTATCAGCCATTTTGGGGCTGATCCTCTCAACCCCATCTTTTGTCTCTCTC
TTATTTTCTTAGCGGGGACGCTCCGTTCTCTCCCTGTGCAGGTGCGACTCTTGCTTGTGCTGG
CCGCGGCAGGTGGCGCCCAACGTGGGGCTCGAGCTCGACAGTTTTCCTCGCCACTACTCTTA
TTAATTGAAAAGAGTGAGTATATGAGTAAACAAGTGAATTAAATTGAGGAGGAGTAGTAAG
GTATATAGTTGAGAGTATAAATATGGGACAGACGCATAGTCGCCAGTTGTTTGTGCATATGT
TATCTGTAATGTTAAAACATAGGGGAATTACTGTTTCTAAACCTAAATTAATCAATTTTCTTT
CATTCATCGAGGAAGTTTGCCCTTGGTTCCCCAGAGAAGGTACAGTAAATTTAGAGACATGG
AAGAAGGTAGGGGAACAAATTCGGACTCATTATACTTTACATGGCCCTGAAAAAATCCCTGT
CGAAACTTTATCCTTTTGGACACTAATTCGTGACTGCCTGGACTTTGATAATGATGAATTAAA
ACGTTTAGGAAATTTATTAAAACAGGAAGAAGATCCTCTCCATGTTCCTGATTCGGAACCCA
GATATGCTGTTCCCGAGGGGGTTAAAAGCGACCCTCCGTTTTCTAACTTATTGCATCCTTCAG
ATAATGATGATTTACTTTCATCCACAGATGAGGCAGAATTAGACGAAGAAGCTGCTAAATAC
CATCAAGAAGATTGGGGTTTTTAGCACAAGAAAAGGGGCGTTAACATCTAAAGATGAATTG
GTTGAATGCTTTAAAAACCTCACTATTGCTTTACAGAACGCAGGAATCAAGCTTCCTAGTAA
CAATGCCAAATCTCCTTCTGCTCCGCCTCTTCCCCCTGCTTATGCTCCTTCTGTTGTGGCTGGT
CTCGATCCCCTCCAGGGCCCCCTCCACCGTCTGAGAACATGTCTCCGCTGCAAAAGGCATTG
AGACAGGCACAGCGACTTGGTGAGGTTGTCTCTGATTTTTCTCTTGCTTTTCCTGTCTTTGAA
AATAACAACCAGCGTTATTATGAATCACTGCCTTTTAAACAACTGAAAGAGTTAAAGATTGC
TTGCTCACAATACGGTCCTACCGCTCCATTCACCATTGCTATGATAGAAAACTTGGGTACTCA
AGCTTTACCTCCAAATGATTGGAAGCAGACAGCTAGGGCATGTCTCTCAGGGGGAGATTATT
TATTATGGAAATCTGAATTTTTGAACAATGTGCTCGTATAGCTGATGTTAACCGACAGCAAG
GTATACAGACCTCCTATGAAATGTTGATTGGTGAAGGCCCTTACCAGGCTACTGACACTCAA
CTTAATTTCTTACCTGGTGCATATGCACAAATATCAAATGCGGCTCGGCAGGCATGGAAAAA
CTTCCTAGCTCCAGTACTAAGACAGAGGATCTTTCAAAAGTCCGGCAGGGACCTGATGAGCC
TTACCAGGACTTCGTGGCACGACTTTTAGATACTATAGGTAAGATAATGTCAGATGAAAAGG
CTGGGATGGTATTGGCAAAACAATTGGCTTTTGAAAACGCTAACTCTGCTTGTCAAGCTGCT
TTAAGACCTTATCGAAAAAGGGAGATCTGTCTGATTTTATTCGCATTTGTGCTGACATTGGAC
CCTCCTACATGCAAGGCATTGCTATGGCAGCAGCATTACAAGGAAAAAGCATAAAGAGGTA
CTTTTCCAGCAGCAAGCCCGGAACAAGAAAGGACTTCAAAAGTCAGGTAATTCGGGTTGCTT
TGTTTGTGGTCAGCCTGGCCATCGGGCTGCAGTGTGCCCTCAAAAACAACAAAGCCCTGTTA
ACACTCCTAATTTGTGCCCACGCTGTAAAAAAGGAAAGCATTGGGCGCGGGATTGCCGTTCC
AAAACGGATGTTCAAGGTAATCCTTGCCCCCGGTTTCGGGAAACTGGGTGAGGGCCAGCCCT
GGCCCCGAAACAATGTTATGGGGCAACACTGCAGGTTCCAAAGGACCATTGCAGACCTCTAT
CGAGCCACAAGAGGCAGCGCGGGATTGGACCTCTGTGCCACCTCCTACACAGTATTAACTCC
CGAGATGGGGGTCCAAACCCTTGCCACAGGAGTGTTTGGGCCTTTACCTCCAGGGACAGCTG
GACTGCTTTTAGGGCGCAGCAGTGCGTCTTTAAAAGGAATACTTATTCATCCTGGTGTGATT
GACTCTGATTATACAGGAGAGATAAAATATTAGCCTCCGCTCCTAACAAAATTATTGTAATC
AATGCAGGACAGCGTATAGCTCAACTTCTTTTAGTTCCATTAGTCATACAAGGAAAAACAAT
TAACCGAGACCGTCAAGATAAAGGTTTCGGGTCCTCTGACGCCTATTGGGTGCAAAATGTTA
CCGAGGCACGACCAGAACTTGAGCTACGCATTAATGGTAAGCTTTTCCGCGGAGTGCTTGAT

ACAGGGGCCGATATTAGTGTTATTTCTGATAAATATTGGCCTACTACATGGCCAAAACAGAT
GGCTATTTCCACTCTCCAGGGTATTGGCCAAACTACCAATCCAGAACAGAGTTCATCCCTTCT
TACTTGGAAGGATAAAGATGGACATACAGGCCAATTTAAACCTTATATTCTGCCCTATCTTC
CAGTTAATCTATGGGGGCGTGATATATTAAGCAAAATGGGTGTTTATTTATATAGTCCTTCAC
CCACTGTGACAGATTTGATGTTAGATCAGGGCTTACTTCCAAATCAAGGTTTAGGTAAACAA
CATCAAGGCATCATTTTGCCCCTTGATTTAAAACCTAATCAAGATCGAAAAGGCTTGGGGTG
TTTTCCTAGGGACCTCTGATTCTCCTGTGACGCATGCCGATCCTATTGATTGGAAATCTGAGG
AACCGGTATGGGTCGATCAGTGGCCCCTAACACAGGAAAAACTTTCTGCCGCACAACAGCT
GGTGCAGGAACAGCTGAGACTTGGGCATATTGAACCCTCTACCTCTGCTTGGAATTCCCCAA
TTTTTGTTATTAAAAAGAAGTCTGGGAAATGGAGATTGCTACAAGATCTTCGTAAGGTAAAT
GAAACAATGATGCATATGGGAGCCCTACAACCTGGGTTGCCCACTCCTTCTGCTATACCTGA
TAAATCCTATATCATTGTTATAGATTTAAAAGATTGTTTTTACACTATTCCTCTTGCACCTCA
AGATTGCAAAAGATTTGCTTTCAGTTTACCCTCTGTTAATTTTAAAAAGCCTATGCAACGCTA
TCAATGGAGAGTTCTCCCGCAAGGAATGACTAATAGCCCTACGCTGTGCCAAAAATTTGTTG
CTACAGCAATAGCTCCGGTTCGTCAACGTTTTCCTCAGCTATATTTGGTTCATTATATGGATG
ATATATTACTAGCTCATGCTGACGAACATCTATTGTATCAAGCTTTTTCGATTCTAAAACAAC
ATTTAAGCCTTAATGGTCTTGTTATTGCTGATGAAAAATTCAGACTCATTTTCCTTATAATTA
TTTGGGTTTCTCCTTATATCCTCGTGTTTATAATACCCAATTAGTAAAACTGCAGACTGACCA
TTTAAAAACTCTAAATGACTTTCAAAAACTTTTAGGAGACATTAATTGGATACGTCCTTATTT
AAAATTACCCACTTATACCTTGCAGCCATTATTTGACATCCTTAAAGGTGACTCTGATCCTGC
GTCACCCCGAACACTTTCTTTAGAAGGACGAACTGCTTTACAATCAATAGAAGAAGCTATTA
GACAACAACAGATTACTTATTGTGATTACCAACGATCATGGGGTTTGTATATACTTCCTACCC
CCCGAGCACCCACAGGGGTTCTCTATCAAGATAAACCTTTGCGATGGATATATTTGTCTGCT
ACTCCAACTAAACATCTGCTCCCTTACTATGAACTTGTTGCAAAATTATAGCAAGGACGTC
ACGAGGCCATCCAATATTTTGGTATGGAACCCCCTTCATTTGTGTTCCTTATGCTTTAGAACA
ACAAGATTGGCTTTTTCAATTTTCAGATAATTGGTCTATAGCTTTTGCAAATTACCCCGGACG
GATTACTCATCATTACCCTTCTGATAAATTGTTACAATTTGCTAGCTCTCATGCCTTTATTTTT
CCAAAAATAGTTCGCCGACAACCTATTCCCGAAGCGACACTTATATTTACAGATGGATCTTC
TAATGGAACTGCAGCTTTAATCATTAACCATCAAACCTATTACGCACAAACCAGTTTTTCTTC
TGCTCAAGTTGTGGAATTATTTGCAGTCCACCAAGCGTTGCTAACTGTACCTACTTCCTTCAA
TTTATTTACAGACAGCTCCTATGTGGTCGGTGCCTTACAGATGATTGAAACTGTTCCAATTAT
CGGCACCACCTCTCCTGAAGTTCTTAACTTATTTACATTGATTCAACAGGTTCTCCATTGCCG
CCAACACCCCTGTTTCTTTGGACATATTCGTGCACACTCCACCCTTCCTGGTGCCCTCGTACA
AGGCAATCACACTGCGGACGTTCTTACTAAACAAGTGTTTTTCCAATCAGCTATTGATGCAG
CCCGAAAATCCCATGATTTACATCACCAAAATAGTCATTCTTTACGCTTGCAATTTAAAATTT
CCCGTGAAGCTGCACGGCAAATTGTTAAATCTTGCTCTACTTGTCCTCAATTCTTTGTTCTCC
TCAATATGGTGTCAACCTCGAGGTTTACGCCCTAATCACCTCTGGCAAACAGATGTTACTCA
CATTCCTCAATTTGGACGTCTTAAATATGTTCATGTTTCTATTGACACTTTTTCCAATTTTCTC
ATGGCCTCCTTCACACTGGAGAATCAACACGTCACTGTATTCAACATTTGCTGTTTTGCTTTT
CTACTTCAGGAATCCCACAAACCCTTAAAACAGATAATGGACCTGGTTATACTAGCCGTTCT
TTTCAACGTTTTTGTCTTTCTTTCCAAATTCATCATAAAACAGGAATTCCTTATAATCCACAG
GGACAAGGTATTGTGGAACGAGCCCATCAACGCCTTAAACATCAATTATTAAAACAAAAAA
GGGGAATGAACTGTATAGCCCCTCACCGCATAACGCCTTAAACCATGCTCTTTATGTTTTAA
ATTTTTTAACTTTAGACGCAGAAGGCAATTCAGCAGCCCAGCGTTTTTGGGGAGAACGATCC
TCATGCAAAAAACCACTTGTGCGATGGAAGGATCCACTTACCAATCTGTGGTATGGGCCAGA
CCCTGTACTAATATGGGGACGAGGGCATGTTTGTGTTTTCCACAGGATGCCGAAGCGCCGCG
CTGGATTCCGGAAAGGCTGGTACGCGCGGCAGAGGAACTCCTGACACATCAAATGCAACGC

ATGACACTGAGTGAGCCCACGAGTGAGCTGCCTACCCAGAGGCAAATTGAGGCGCTGATGC
GTTATGCTTGGAATGAGGCTCATGTACAACCTCCAGTGACACCTACTAATATACTGATCATG
TTATTATTATTGTTACAGCGGATACAAAACGGGGCAGCTGCGGCTTTTTGGGCATACATTCCT
GATCCGCCTATGATTCAATCCTTAGGATGGGATAAAGAAACAGTACCTGTATATGTTAATGA
TACAAGTCTTTTAGGAGGAAAATCAGATATTCACATTTCTCCTCAGCAAGCCAATATCTCCTT
TTATGGCCTTACTACTCAATACCCTATGTGCTTTTCTTATCAATCACAGCATCCTCATTGTATA
CAGGTGTCAGCTGATATATCCTATCCTCGAGTGACTATTTCAGGCATTGATGAAAAAACCGG
AAAGAGATCGTACCGTGACGGAACCGGACCCCTGGACATTCCGTTTTGTGACAAACATTTAA
GCATCGGCATAGGAATAGACACTCCTTGGACTTTATGTCGAGCACGAATTGCATCGGTGTAT
AACATCAACAATGCCAATACCACCCTTTTATGGGACTGGGCACCTGGAGGAACACCTGATTT
CCCCGAATATCGAGGACAGCATCCACCCATTCTCTCTGTAAACACTGCTCCTATATTTCAAAC
TGAACTGTGGAAACTTTTGGCTGCTTTTGGTCATGGCAATAGTCTATATCTACAGCCCAATAT
TAGTGGGAGCAAATATGGTGATGTGGGAGTTACAGGATTTTTATATCCCCGAGCTTGTGTTC
CTTACCCATTCATGTTGATACAAGGCCATATGGAAATAACACTGTCATTGAATATTTATCATT
TAAATTGTTCTAATTGCATACTTACTAATTGCATTAGAGGTGTAGCCAAAGGAGAACAAGTT
ATAATAGTAAAACAACCTGCTTTTGTAATGTTACCTGTTGAAATAACTGAAGAATGGTATGA
TGAAACTGCTTTAGAATTGTTACAACGCATTAATACGGCTCTTAGCCGTCCTAAAAGAGGTC
TGAGCCTGATTATTCTGGGTATAGTGTCTTTAATCACCCTTATAGCAACTGCTGTTACTGCTT
CTGTATCTTTAGCACAATCCATTCAAGCTGCTCATACTGTAGATTCCTTGTCATATAATGTTA
CTAAAGTAATGGGAACTCAAGAAGATATAGATAAAAAATAGAAGATAGATTATCAGCTTTA
TATGATGTAGTTAGAGTTTTAGGAGAACAAGTTCAGAGCATTAATTTTCGCATGAAAATTCA
ATGCCATGCTAATTATAAATGGATTTGTGTTACAAAAAGCCTTACAATACTTCTGACTTTCCG
TGGGATAAGGTGAAAAAACATCTACAAGGAATTTGGTTTAATACTAATGTTTCTTTAGATCT
TTTACAATTGCACAATGAAATTCTTGACATCGAAAATTCTCCAAAAGCTACTTTGAATATAG
CTGATACCGTCGATAATTTTTTACAAAATTTATTTTCTAACTTTCCTAGCCTTCATTCACTGTG
GCGAAGTATAATTGCTATGGGCGCGGTTCTGACTGTTGTGCTTATCATAATTTGTCTAGCTCC
TTGCCTTATTCGTAGCATTGTTAAAGAATTTCTACATATGAGAGTTTTAATACATAAAAACAT
GTTGCAACACCAACATCTTATGGAGCTTTTAAAAAATAAAGAGAGGGGAGCTGCGGGGGAC
GACCCGTGAAGGGTTAAGTCTTGGGAGCTCCCTGGCAGGTATGCCAGGCCCTAGGACACGT
GCCTAAGCTCCCTGTCCCGCCACCCTCAAGAGTTTTTATAACCCTTAAGGCTCCAAGATGTTT
GGTTTCGGCAACATTTCATAGAAGATAGATTATCTTATTGTGTATATTTCATAGAAGATAGAT
ATTCTGATTGTGTTCTGTATACAATGGTAAGGGTCTGGTGATTGTATCCTGAGATTAAAAAA
CAACCTTGTGAGTGCCTTAAGTCACGTACTTTACCCTATATATACTGCAGCACAATAAAGCA
AGGTATCAGCCATTTTGGGGCTGATCCTCTCAACCCCATCTTTTGTCTCTCTCTTATTTTCTTA
GCGGGGACGCTCCGTTCTCTCCCTGTGCAGGTGCGACTCTTGCTTGTGCTGGCCGCGGCA

>NC_056059.1:81401343-81409128#SHEEP_RIP_16(+)

TGAAGGGTTAATGTGGCCACAATAGGGAAAGCAGAAATGTGCCTGCAAATGGGACTCTGCC
TGGGCCGAACGTGCTTGCAAACAAGATGTTCTGCCAAGGAGACTGGACACAGCCTTGAGTTT
AATGGTCCCTTGCAAACGAGGGAACATTCCCTTACTGTGATAGGAAGGAAGAAAGGGCTCT
GGACAGACTCTGCAGTAAGCCGGAATTTCACTCCCTTCTGCTGTACAATAACATTTATGCAC
ATGCGCTATACTGAAAAGGCTTAGTCATACAGTCTGGAATCCTGCCAAGGGGGCTATATAAA
AGTAAACCGCAAGCTTGCTTCCTTGTGCAGTTCTTTTCCTCTGGCCAGAATTGTGTCTGTCTC
TTGTGTGTCTTGTCTTTGTGTCATTTCACTCGCAATTTCCAACATCTGGCGCCCAACATGGGG
CTCGAGTGAAACCGAAAGGGTGAGTAACCCCGGGGGGATTTTAAATCCATAGCAGGGGAAC
TTTCAATGGGAGAATGACATTCTAACATGTATACTATCATGTGAATTGAATCGCCAGTCTAT
GTCTGACGCAGGATGCAGCATGCTTGGGGCTGGTGCATGGGGATGACCCAGAAAGATGTTA

TGGGGAGGGAGGTGGGAGGGGGGTTCATGTTTGGGAATGCATGTAAGAATTAAAGATTTTA
AAATTTAAAAAATAAAAAACTAAAAAAAAAAAAAAAAAAAGAAAATCATGGGGAATTCCTC
ATCATCATTACGGACACAATACATGGAGTTGGTCAAAGGACTTCTCCACTCCATAGGCGTTA
AGGTCTCAACTCATTGATAGAGTGAGCTCTTTCGCTTGGTGGAGCAATATTGTCATTGGTTTC
AATATCAAACTAAGTTACAGTTAAATTTAAAGGAATGGAAAATAATTTAAAAAAAAAAACTG
AAAAAGCAACATCAGAAGGGTAATGTGATCCCTTTGAAGTTATGGACTTTATGTAATGCTAT
AACACAGGCTTTGACTTTGCTATCTACTGATAATAAAACTAAATCTAATGCTTCAATGAAGG
GAGAGGCAATTTATGAACATGTGCCAAACGTTGGTGGGGTTTCTGCATTTCCTAAAGGTAAG
GATACAGGTGAGCCTCCTTCTGGAAATGGTAAAACATCTGTTAGTTCAAAATCAGATTTGGA
GGCTTCTTCAGTTTTGTCAGAGGAGGGCAAAGAGATTAAAAAAAATGACCCATCTATTCCAG
AAATGGTGAAAATCCCATAGGGGAAAAAAAAAAAAAAATCTGCACCTTCTGCTCCTCCTTAT
ACTTCTCTTTTCCCCACTGCAGTTGATCCCCCGATGTGGGCAGGGAACATTGTAAGTTCTCCT
TTCCTTTGTCTATGCTTCATGATAATGACTTACCTACTCCCCCTAGTGGTTTTATTGATCCTCC
ACAATTGTTTCCCATCCAGAGACAGCAAGATGACAACGTGATAAATATTCAATACACTCCTT
TAAAATATAAATTTTTTAAAGATCTTAAAGCTGCAGTAGCGCAAAATGGTCCTTAATCTCCC
TTTGTTTTGGCTATGCTGAAATCATTAGGGAAAAGGCAAACTAATCATTCTGTTAAATTGAA
AATCTATTGCCCAAACTGTCTTGGAGGGTTCTCAATGGTTACAACTTCGTAGCTGGTGAAAA
AAAAAGAGCTAAAAAGCAGGCCCAGATTAATAAAAGACAAAATCCTCCTGGTCCTCTCAAA
GACAAGTTAATGGGAGAGGGCCATTATCGGGCTTTAAAAAAGCAGGCTCAATACTCTGATC
AAGACTTACAACAAGTCCGCCAGGTCTTTTTACGAGCACTGTGTGGTGCCTACTGGCCACAC
CCAGCCCTCCTTTGTTAAAACAATGCAAGGTCCCAATGAGCCATATACTAATTTTCTAGCAA
GATTGAGGGTAGCTGTGGAACAGGCTATAAAAAAAGATAAAATTTCAAAAATATTATTACA
AAGGTTAGCATTTAAAAATGCAAATCCTAAATGCAAGCGTATACTGAGACCATTAAAGAGA
CAGGGTACATCTATAGCTAAATATATAAGAGCCTGCTCAGGAGTAGGAGGAACTGAGCATC
AGGCTAATGTCTTTGCTACAGCCTTGGCCAAAGCTATAAAACCACAAAAGGGAGGTAACTG
CTTCCATTGTGAAAAACCTGGTCATATTAAAAAAAAAAAAAAAAAAAAGTCAAAAATTAAAA
GCTGATCAAGGTGCAATTCCTAAAGACAGATCTTCTGCTGGGAGGAATAAGACTCCTCCTAG
ACCTTGCTGTCAATGCAAGAAGGGGCTTCATTAGACTAATGAGTGCAAATCTAAAACAGAC
AAAATGGGCAACCTGATTCCGAAAAACTATCCTGCAGGCCTAAGTCCTTGGGGCCCAAAAG
CAATACCGGGGACTTTTCCTCCCTGTCCTCCTGCTGTCCTGCCTACCCCAACCCTGTTCCCTC
CCAACAACAGTTACCAGTCAATGCCTCATTAAAAAGACCTCAAATGATGATTTCAGACTTAC
AGTCTGCTACTTCAGAGAGTGCTGCTGCTGATTTGCCACTAGCTAAAAATGTTCTTTTGTCAC
CAAGAAAAAGCATTTACAAATTAAAAACAAATATATTCGGACCACTGCCTAAAGGCAGCTT
TAGCTTAATATTAGGCCATAGCAGTGCAGCTTTGAGACCATAATTCCTGGGATAATAGACTC
CGACTATGTTAAAAAATTTTTGATTATGGTTTCTACTTCTACCATGCTTTCATTGTTAGCTGG
AAAACGTATTGCTCATATACTTCTCCTACCTTATCATCCCTTTTTGGCTCTTCCTAATAAACA
AACAAAAAGATTTAAAAGTACTGGGCGACATATATTTTAAAAAATGCTTATCAAAGATTCTC
GCCCTGTTCTCTCCTTAATTATACAAAAAAAAAAAAAAAAAAAAAACAACTTTGAGAGACTA
GTAGACACAGGGGCAGATGTTTCAGTCATTTCTTCTCAACAATGGCCCCAAGATTAAAAAAA
AAAAAAAAGTCCTCTAATGCTGACAAGACTGGGCTCCATTGCAGATGTCTGAAAAAATACC
CATCCCTTGCAATGCCAATTCCATAATAAAAGATCAGTGTCTGTTACCTTTTATATTATAAAC
ATACCTATTAATATTTAAAAAAAAAATCTTCTCTCTCCTTTGGGGGTTTTTGTAACCATTCCAC
CAAAACACTAGTAGCCACTGCTCAAATTCCTCAAACACTCCCATTAAAATGGTTAACTAATA
CTCCAAAATGGGTTGAGCAATGGCCATTACCACAAACAAAGCTCGAGGCGTTAAAACAATT
AGTACAAAAACAACTCCAATTTGGTCATATAAAGCCCTCTATCTCCTCCTAAAATTCTCCTGT
TTTTGTTATGGAAAAAAAAAAATCTGAAAAATAAAAAATGTTAACCGATTTACAAAAAGTTA
ATAAATGTATTAAACCTATGAAAACATTACAATTGAGACTCCCCTCTCCAGCTCTTATTCCTC

AAAATTGGTCCTTAATGGTGCTAGATCTTAAAGACTGTTTTTTTGCCATTCCCCTACAATTGC
AAGATAGAGATAAATTTGCTTTTACAGTTCCTGTTCTTAATCATGTTCAGCCTGTTAAGCGTT
ATCAATAAATAGTCTTACCACAAAAAATGACAAATAGTCCTACTTTATGCCAAAAATTCATA
ACTCGCTCTTTACAATCCCTCCGTCAAAAATACCCCAATTATATTCTATATCATTATATAGAT
GATCTCCTATTAGCAGCTCCTAATATTGCTAAACGTGATAAATTCTTTCTAAAAACACAAGA
GGCTTTAAGACTATACAATTTACAAATAGCCCCCAAAAAAATTCAAAAAGACTTTCCTATTT
CATATTTAGAGACGATATTAAAACAACATAAAATAAGGCCCCAAAAGTTACAATCAAAAGA
GACCACCTCAAAACCTTAAATGATTTTCAAAAGTTATTGAGAGATATCAATTGGCTACACCC
AGTACTTGAGATTCCTACTTATCAGTTACGACATTTGTTTTCTATTTTAAAAAAATACAACTC
TAGATAGCCCCCAAACTTTAACCCCATTGGCTTTACAAAAACTTCAATTTGTTAAACAACGA
CTAAATGATGGTTTTTTGACTTACTTACATGTGTTTCAACCTGTTTCATTTATAATATTTCATA
CCCCTTATTCTCCATCTAGTATAACTGCTCAAAAAAATTTAATAAAATAGGTTTTCTTACCTA
ACAGCTTTTCCAAAAAATTGACTGCATATATAGATAAATTAGCCTTCCTTATACAAAAAGGT
CACCATCGTATTTTACAATTGTCAATATATAAACCACACCAGATTGTTACTCAATTAACAACT
GCTCAAATATCTCCATGTTTACAATCTAATAAAAACTGGCAAATTTCTCTTGCCTCATTTCCT
GGTTCGTTTTCTAATCACTATCCATCATATAAATTGATTGATTTTCTCCAGACTAACTCTATG
ATATCTCAATCCCCAATTTCAGATGTTCCAGTTAAGAGACCCACTATTTTTACAGATACAAAT
AAAAATACTGCTAGATATTAGACCCCAAAAAGTTCTAAGGTTCTCCCCGACTCATTTTCTTCT
ATATAGCCTGCTGAATTGTGGGCTATCTATTTAGTTTCACAAGATTTTCCCCAAATTCCTACT
AACATTGTTTCAGATTCTCGATATGCTGTTCTCTCTTGCCTACAGCTTCCCCATGTCTCCCTTC
CACTGACCCTTAAAACAGCTATTAATAAATTGTTTTACCAAATACAACAATTGCTCTTGCAG
CATTCAAAATTAATTTTCTTTACTCACATCCTTACACATTCTGCCCTTCCTAGACCCTTATCAT
TCAAAAATGCTACAATTGATGCCTTACTTTATCCTATAAAAACAACAAAACAAAAACATCTC
TTACAGCATACCAACTCCAAAGGCTTACAAAAATCTCATGCTATTACTCAAAAACAAGCTCA
AAATATTGTTCATTCTTGTTCCATATGTACACCCTTTGCTTTACTGTTTACCTCACCAGGTGTC
AACAAAGAAAGACAACAAACAAATCAAATCTAGCAAATGGATATAATTTACATTTCTTCTTT
CAAACAACAAAAATATGTACATCATACTATAGATACGTGCACACATTTTCAATGGGCCACTG
CATTACATTCTAAATAGGCTGACACTGTTATTACTCATTTGTTATCTTGTTTTACAGTTATGA
GATTACCAATTAAATTAAAAACCGATAATACACCTGCCTACCAATCCACAAAATTAGCTCAC
TTTTTATCCCAATATCATATAACTCATACTTTTGGTATTCCTTATAATAGTCAAAGGCAAACT
ATCATTAAAAGAGCTAATCATACCTTGCATAAATATCTTAAAAAAATAAAAAAAGAAAAAC
AGGAGAGATTTATGAAACCTAATGACATTTTGAGTAAAACCTTACTTACCCTAAATTTTTCA
AATATTTGGAGCAAGGGAAATCTATCAGCAGCAGAGTTGCATTTTCAAGGGAAAGAAGAAG
ACAAAAAGATCTTGAATACGCCTATTTGGTATAAAGAGAAAGAGAAAGGTTGGATCCCAGC
ATCATTAATATATCTGGGACGAGGGTACGCTTTCATTTCTGTTAATAATTACAGGTTTTGGAC
CCCAGCAAGATTGACCAAAATCAACAATGGCTGATCCTTTTGTTCAAAAATTCAAAGAGCTT
ACTATGCAGAAAAGCTTTACTTTCCATACAAGAGAAGCAACACCTCCTACGTGGGGTCAAAT
GAAGAGGTTGACCCAGGAAGCAGAGAAGACATTAATGAAGGCGGGGCAACCTCTGAATCTT
ACCAATCTTTTGCTTGCCATGATGTCAGGTAATCAGTGTATCGGCAAGTAATCATACATATTG
GGCATATATACCTAATCCCCCATTAGTAAGAGCAGTTTTCTGGGGGGAACCAGAAGTGCAGG
TATGCACTAATGAGACTGCCTTCTTTCCCCCACCAGCTTGCGGGGGAATAGAACAACTATCT
CATCATAAACAACAATATAATATAATATTAGTAATTTGAACATTGCAGTGGAAGATATTCCT
TTGTGTATAGGAGGACACCGCTTTTGTCTGTCCACCAAGAAACGTTCTCATCATTCCTATAAT
ACATGGGGGGTAAAGTACAATAATTATCATTTTGCTACTTTTATTGTGCTTGTTTCCACCAGG
GGCTTTAACAGCTCGACAGAACCGATAGACATTCATAATGGAATACATATGTCACTATGTCC
TGTTAACTTTTTTGCTCCTCCTCTATAATCTTTGGAATGGAAACGTTGCCGAGGTCATCGACC
CTTTAAAGTCATGAATTATTCTGGGTCTATCATTGTAGATTGGAGTCCAGATCATGGGCAATT

CTTAGAAAAATGGTCAAATAAATCTCTTAGGTGGCATCATGCAAATAGCACTTTGATGGACA
ATGGTAATGGAACAGTTAAATGGCAGCAATTTGCACTTGTCCTTCCTCAATTACAATTGCAA
GGATATCCGCACATTCAAAAAATATTTAGAAACTATGGGCAGGTTCTGGTAATCTCACTATC
TGGTCAGAAAACTAGACTTTGGATAGTGGTGACTCTTCGGGTCCATTTCATATTAATTTACAC
TTTAATAAATCTTATTCCATAATGGCATGTGTAAAATATCCTTTTCCATTGTTATATGGAAAT
GGGACCTAAAATGATACTGTGGGGTCTGTGTCATGCGATTATTGTAATCTAACTCAATTTCAT
TAGTAATTGTTAAAGCTCGGACAGAAGTATGGTTGCCTATAAATCTGACTCGGCGGTGGTCA
GATTCTTTTGCTGTTTCTCAACTAGTAACTGCTGTACAGACCTTGCTACATCAATCTTGACGT
ATGCTTGGTGTGGTCATTGCTTCGATTTTAGCAGTTGCGTCAGTAACTGCAACAGCGGCGGT
AACAGGCCTCGCGTTACACCAAGATATTCAAACAGCTGATTTTGTTCGGGACTGGCATAAAG
ACTCTCATTTGTTATGACAACAACAGCGAGATTTAGATGCACAACTTGCTATTGATGTGCTC
AATCTTCAACACACCGTTTCCTGGCTTGGAGATCAATTAGCTGTTTTATCTACACGAAGTGTG
TTAAAATGTGATTAAAATTCTTCTCAGTTTTGTATAACACCTGTACCATTTAACATGAGTAAA
GGATGGGATAAAGTAAAACGATCCTTGACTGGGCATCAAAATCTCACTACGGAGATTATGG
ACCTAAAACGACAAATTTTGTCTACTTTTAACAAGACTTTACCTGACATTATGGGGTCTGATT
TGCTAAAAAGTCTTCAAGAGGAAATAGATAACTTAAATCCATTAGGGCGTGTATCCTTACTA
ATTGAGACTACCTTTAAAAACACTGTGTTTATATTACTTTTATGTTGTGTTGCTTTTCTAGTCT
TCCAGTGATGGCAAAAAGGAAAACAACTAAAGCGCAAAGCAGAGAAGATCCAGACCATGC
TACAATTTATAAAAGCAGATAAGAAAGGGGGAGATGAAGGGTTAATGCGGCCACAATAGGG
AAAGTGGAGATGTGCCTGCAAACAGGACTCTCTGCCTGGGCCAAACCTGCTTGCAAAAAAG
ATGTTCTGCCAAGGAGACTGGACACAGCCTTGAGTTTGATGGTCCCTTGCAAACGAGGGAAC
ATTCCCTTCCTGTGATA

>NC_056060.1:17005264-17013194#SHEEP_RIP_17(+)

CTGCGGGGGACGACCCGTGAAGGGTTAAGTCTTGGGAGCTCCCTGGCAGGTATGCCAGGCC
CTAGGACACGTGCCTAAGCTCCCTGTCCCGCCACCCTCAAGAGTTTTTGTAACCCTTAAGGCT
CCAAGATGTTTGGTTTCGGCAACATTTCATAGAAGATAGATTATCTTATTGTGTATATTTCAT
AGAAGATAGATATTCTGATTGTGTTCTGTATACAATGGTAAGGGTCTGGTGATTGTATCCTG
AGATTAAAAAACAACCTTGTGAGTGCCTTAAGTCACGTACTTTACCCTATATATACCGCAGC
ACAATAAAGCAAGGTATCAGCCATTTTGGTCTGATCCTCTCAACCCCATCTTTTGTCTATCTC
TTATTTTCTTAGCGGGGACGCTCCGTTCTCTCCCTGTGCAGGTGCGACTCTTGCTTGTGCTGG
CCGCGGCAGGTGGCGCCCAACGTGGGGCTCGAGCTCGACAGTTTTCCTCGCCACTACTCTTA
TTAATTGAAAAGAGTGAGTATATGAGTAAACAAGTGAATTAAATTGAGGAGGAGTAGTAAG
GTATATAGTTGAGAGTATAAATATGGGACAGACGCATAGTCGCCAGTTGTTTGTGCATATGT
TATCTGTAATGTTAAAACATAGGGGAATTACTGTTTCTAAACCTAAATTAATCAATTTTCTTT
CATTCATCGAGGAAGTTTGCCCTTGGTTCCCCAGAGAAGGTACAGTAAATTTAGAGACATGG
AAGAAGGTAGGGAACAAATTCGGACTCATTATACTTTACATGGCCCTGAAAAAATCCCTGTC
GAAACTTTATCCTTTTGGACACTAATTCGTGACTGCCTGGACTTTGATAATGATGAATTAAAA
CGTTTAGGAAATTTATTAAAACAGGAAGAAGATCCTCTCCATGTTCCTGATTCGGAACCCAG
ATATGCTGTTCCCGAGGGGGTTAAAAGCGACCCTCCGTTTTCTAACTTATTGCATCCTTCAGA
TAATGATGATTTACTTTCATCCACAGATGAGGCAGAATTAGACGAAGAAGCTGCTAAATACC
ATCAAGAAGATTGGGGTTTTTAGCACAAGAAAAGGGGCGTTAACATCTAAAGATGAATTGG
TTGAATGCTTTAAAAACCTCACTATTGCTTTACAGAACGCAGGAATCAAGCTTCCTAGTAAC
AATGCCAAATCTCCTTCTGCTCCGCCTCTTCCCCTGCTTATGCTCCTTCTGTTGTGGCTGGTCT
CGATCCCCTCCAGGGCCCCCTCCACCGTCTGAGAACATGTCTCCGCTGCAAAAGGCATTGAG
ACAGGCACAGCGACTTGGTGAGGTTGTCTCTGATTTTTCTCTTGCTTTTCCTGTCTTTGAAAA
TAACAACCAGCGTTATTATGAATCACTGCCTTTTAAACAACTGAAAGAGTTAAAGATTGCTT

GCTCACAATACGGTCCTACCGCTCCATTCACCATTGCTATGATAGAAAATTTGGGTACTCAA
GCTTTACCTCCAAATGATTGGAAGCAGACAGCTAGGGCATGTCTCTCAGGGGAGATTATTTA
TTATGGAAATCTGAATTTTTTGAACAATGTGCTCGTATAGCTGATGTTAACCGACAGCAAGG
TATACAGACCTCCTATGAAATGTTGATTGGTGAAGGCCCTTACCAGGCTACTGATACTCAAC
TTAATTTCTTACCTGGTGCATATGCACAAATATCAAATGCGGCTCGGCAGGCATGGAAAAAA
CTTCCTAGCTCCAGTACTAAGACAGAGGATCTTTCAAAAGTCCGGCAGGGACCTGATGAGCC
TTACCAGGACTTCGTGGCACGACTTTTAGATACTATAGGTAAGATAATGTCAGATGAAAAGG
CTGGGATGGTATTGGCAAAACAATTGGCTTTTGAAAACGCTAACTCTGCTTGTCAAGCTGCT
TTAAGACCTTATCGAAAAAAGGGAGATCTGTCTGATTTTATTCGCATTTGTGCTGACATTGG
ACCCTCCTACATGCAAGGCATTGCTATGGCAGCAGCATTACAAGGAAAAAGCATAAAAGAG
GTACTTTTCCAGCAGCAAGCCCGGAACAAGAAAGGACTTCAAAAGTCAGGTAATTTGGGTT
GCTTTGTTTGTGGTCAGCCTGGCCATCGGGCTGCAGTGTGCCCTCAAAAACAACAAAGCCCT
GTTAACACTCCTAATTTGTGCCCACGCTATAAAAAAGGAAAGCATTGGGCGCGGGATTGCCG
TTCCAAAACGGATGTTCAAGGTAATCCTTGCCCCCGGTTTCGGGAGAACTGGGTGAGGGCCA
GCCCCTGGCCCCGAAACAATGTTATGGGGCAACACTGCAGGTTCCAAAAGGACCATTGCAG
ACCTCTGTCGAGCCACAAGAGGCAGCGCGGATTGGACCTCTGTGCCACCTCCTACACAGTAT
TAACTCCCGAGATGGGGGTCCAAACCCTTGCCACAGGAGTGTTTGGGCCTTTACCTCCAGGG
ACAGCTGGACTGCTTTTAGGGCGCAGCAGTGCATCTTTAAAAGGAATACTTATTCATCCTGG
TGTGATTGACTCTGATTATACAGGAGAGATAAAAATATTAGCCTCCGCTCCTAACAAAATTA
TTGTGATCAATGCAGGACAGCGTATAGCTCAACTTCTTTTAGTTCCATTAGTCATACAAGGA
AAAACAATTAACCGAGACCGTCAAGATAAAGGTTTCGGGTCCTCTGACGCCTATTGGGTGCA
AAATGTTACCGAGGCACGACCAGAACTTGAGCTACGCATTAATGGTAAGCTTTTCCGCGGAG
TGCTTGATACAGGGGCCGATATTAGTGTTATTTCTGATAAATATTGGCCTACTACATGGCCA
AAACAGATGGCTATTTCCACTCTCCAGGGTATTGGCCAAACTACCAATCCAGAACAGAGTTC
ATCCCTTCTTACTTGGAAGGATAAAGATGGACATACAGGCCAATTTAAACCTTATATTCTGC
CCTATCTTCCAGTTAATCTATGGGGGCGTGATATATTAAGCAAAATGGGTGTTTATTTATATA
GTCCTTCACCCACTGTGACAGATTTGATGTTAGATCAGGGCTTACTTCCAAATCAAGGTTTAG
GTAAACAACATCAAGGCATCATTTTGCCCCTTGATTTAAAAATCTAATCAAGATCGAAAAGG
CTTGGGGTGTTTTCCTAGGGACCTCTGATTCTCCTGTGACACATGCCGATCCTATTGATTGGA
AATCTGAGGAACCGGTATGGGTCGATCAGTGGCCCCTAACACAGGAAAAACTTTCTGCCGC
ACAACAGCTGGTGCAGGAACAGCTGAGACTTGGGCATATTGAACCCTCTACCTCTGCTTGGA
ATTCCCCAATTTTTGTTATTAAAAAGAAGTCTGGGAAATGGAGATTGCTACAAGATCTTCGT
AAGGTAAATGAAACAATGATGCATATGGGAGCCCTACAACCTGGGTTGCCCACTCCTTCTGC
TATACCTGATAAATCCTATATCATTGTTATAGATTTAAAAGATTGTTTTTACACTATTCCTCTT
GCACCTCAAGATTGCAAAAGATTTGCTTTCAGTTTACCCTCTGTTAATTTTAAAGAGCCTATG
CAACGCTATCAATGGAGAGTTCTCCCGCAAGGAATGACTAATAGCCCTACGCTGTGCCAAAA
ATTTGTTGCTACAGCAATAGCTCCGGTTCGTCAACGTTTTCCTCAGCTATATTTGGTTCATTA
TATGGATGATATATTACTAGCTCATGCTGACGAACATCTATTGTATCAAGCTTTTTCGATTCT
AAAACAACATTTAAGCCTTAATGGTCTTGTTATTGCTGATGAAAAAATTCAGACTCATTTTCC
TTATAATTATTTGGGTTTCTCCTTATATCCTCGTGTTTATAGTACCCAATTAGTAAAACTGCA
GACTGACCATTTGAAAACTCTAAATGACTTTCAAAAACTTTTAGGAGACATTAATTGGATAC
GTCCTTATTTAAAATTACCCACTTATACCTTGCAGCCATTATTTGACATCCTTAAAGGTGACT
CTGATCCTGCGTCACCCCGAACACTTTCTTTAGAAGGACGAACTGCTTTACAATCAATAGAA
GAAGCTATTAGACAACAACAGATTACTTATTGTGATTACCAACGATCATGGGGTTTGTATAT
ACTTCCTACCCCCCGAGCACCCACAGGGGTTCTCTATCAAGATAAACCTTTGCGATGGATAT
ATTTGTCTGCTACTCCAACTAAACATCTGCTTCCTTACTATGAACTTGTTGCAAAAATTGTAG
CAAAGGGACGTCACGAGGCCATCCAATATTTTGGTATGGAACCCCCCTTCATTTGTGTTCCTT

ATGCTTTAGAACAACAAGATTGGCTTTTTCAATTTTCAGATAATTGGTCTATAGCTTTTGCAA
ATTACCCCGGACGGATTACTCATCATTACCCTTCTGATAAATTGTTACAATTTGCTAGCTCTC
ATGCCTTTATTTTTCCAAAAATAGTTCGCCGACAACCTATTCCCGAAGCGACACTTATATTTA
CAGATGGATCTTCTAATGGAACTGCAGCTTTAATCATTAACCATCAAACCTATTACGCACAA
ACCAGTTTTTCTTCTGCTCAAGTTGTGGAATTATTTGCAGTCCACCAAGCGTTGCTAACTGTA
CCTACTTCCTTCAATTTATTTACAGACAGCTCCTATGTGGTCGGTGCCTTACAGATGATTGAA
ACTGTTCCAATTATCGGCACCACCTCTCCTGAAGTTCTTAACTTATTTACATTGATTCAACAG
GTTCTCCATTGCCGCCAACACCCCTGTTTCTTTGGACATATTCGTGCACATTCCACCCTTCCT
GGTGCCCTGGTACAAGGCAATCACACTGCGGACGTTCTTACTAAACAAGTGTTTTTCCAATC
AGCTATTGATGCAGCCCGAAAATCCCATGATTTACATCACCAAAATAGTCATTCTTTACGGT
TGCAATTTAAAATTTCCCGTGAAGCTGCACGGCAAATTGTTAAATCTTGCTCTACTTGTCCTC
AATTCTTTGTTCTCCCTCAATATGGTGTCAACCCTCGAGGTTTACGCCCTAATCACCTCTGGC
AAACAGACGTTACTCACATTCCTCAATTTGGGCGTCTTAAATATGTTCATGTCTCTATTGACA
CTTTTTCCAATTTTCTCATGGCTTCCCTTCACACTGGAGAATCAACACGTCACTGTATTCAAC
ACTTGCTGTTTTGCTTTTCTACTTCAGGAATCCCACAAACCCTTAAAACAGATAATGGACCTG
GTTATACTAGCCGTTCTTTTCAACGTTTTTGTCTTTCTTTCCAAATTCATCATAAAACAGGAAT
TCCTTATAATCCACAGGGACAAGGTATTGTGGAACGAGCCCATCAACGCCTTAAACATCAAT
TATTAAAACAAAAAAAGGGGAATGAACTGTATAGCCCCTCACCGCATAACGCCTTAAACCA
TGCTCTCTATGTTTTAAATTTTTTAACTTTAGACGCAGAAGGCAATTCAGCAGCCCAGCGTTT
TTGGGGAGAACGATCCTCATGCAAAAAACCACTTGTACGATGGAAGGATCCACTTACCAATC
TGTGGTATGGGCCAGACCCTGTACTATATGGGGACGAGGGCATGTTTGTGTTTTTCCACAGG
ATGCCGAAGCGCCGCGCTGGATTCCGGAAAGGCTGGTACGCGCGGCAGAGGAACTCCCTGA
CACATCAAATGCAACGCATGACACTGAGTGAGCCCACGAGTGAGCTGCCTACCCAGAGGCA
AATTGAGGCGCTGATGCGTTATGCTTGGAATGAGGCTCATGTACAACCTCCAGTGACACCTA
CTAATATACTGATCATGTTATTATTATTGTTACAGCGGATACAAAACGGGGCAGCTGCGGCT
TTTTGGGCATACATTCCTGATCCGCCTATGATTCAATCCTTAGGATGGGATAAAGAAACAGT
ACCTGTATATGTTAATGATACAAGTCTTTTAGGAGGAAAATCAGATATTCACATTTCTCCTCA
GCAAGCCAATATCTCCTTTTATGGTCTTACTACTCAATACCCTATGTGCTTTTCTTATCAATCA
CAGCATCCTCATTGTATACAGGTGTCAGCTGATATATCCTATCCTCGAGTGACTATTTCAGGC
ATTGATGAAAAAACCGGAAAGAGATCGTACCGTGACGGAACCGGACCTCTCGACATTCCGT
TTTGTGACAAACATTTAAGCATCGGCATAGGAATAGACACTCCTTGGACTTTATGTCGAGCA
CGAATTGCATCGGTGTATAACATCAACAATGCCAATACCACCCTTTTATGGGACTGGGCACC
TGGAGGAACACCTGATTTCCCCGAATATCGAGGACAGCATCCACCCATTCTTTCTGTAAACA
CTGCTCCTATATTTCAAACTGAACTGTGGAAACTTTTGGCTGCTTTTGGTCATGGCAATAGCC
TATATTTACAGCCCAATATTAGTGGGAGCAAATATGGTGATGTGGGAGTTACAGGATTTTTA
TATCCCCGAGCTTGTGTCCCTTATCCATTCATGTTGATACAAGGCCATATGGAAATAACACTG
TCATTGAATATTTATCATTTAAATTGTTCTAATTGCATACTTACTAATTGCATTAGAGGTGTA
GCCAAAGGAGAACAAGTTGTAATAGTAAAACAACCTGCTTTTGTAATGTTACCTGTTGAAAT
AACTGAAGAATGGTATGATGAAACTGCTTTAGAATTGTTACAACGCATTAATACGGCTCTTA
GCCGTCCTAAAAGAGGTCTGAGCCTGATTATTCTGGGTATAGTGTCTTTAATCACCCTCATAG
CAACTGCTGTTACTGCTTCTGTATCTTTAGCACAATCCATTCAAGCTGCTCATACTGTAGATT
CCTTGTCATATAATGTTACTAAAGTAATGGGAACTCAAGAAGATATAGATAAAAAATAGAA
GATAGATTATCAGCTTTATATGATGTAGTTAGAGTTTTAGGAGAACAAGTTCAGAGCATTAA
TTTTCGCATGAAAATTCAATGCCATGCTAATTATAAATGGATTTGTGTTACAAAAAGCCTTAC
AATACTTCTGACTTTCCGTGGGATAAGGTGAAAAACATCTGCAAGGAATTTGGTTTAATACT
AATGTTTCTTTAGATCTTTTACAATTGCACAATGAAATTCTTGACATCGAAAATTCTCCAAAA
GCTACTTTGAATATAGCTGATACCGTCGATAATTTTTTACAAAATTTATTTTCTAACTTTCCTA

GCCTTCATTCGCTGTGGCGAAGTATAATTGCTATGGGCGCGGTTCTGACTGTTGTGCTTATCA
TAATTTGTCTAGCTCCTTGTCTTATTCGTAGCATTGTTAAAGAATTTCTACATATGAGAGTTTT
AATACATAAAAACATGTTGCAACACCAACATCTTATGGAGCTTTTAAAAAATAAAGAGAGG
GGAGCTGCGGGGGACGACCCGTGAAGGGTTAAGTCTTGGGAGCTCCCTGGCAGGTATGCCA
GGCCCTAGGACACGTGCCTAAGCTCCCTGTCCCGCCACCCTCAAGAGTTTTTGTAACCCTTA
AGGCTCCAAGATGTTTGGTTTCGGCAACATTTCATAGAAGATAGATTATCTTATTGTGTATAT
TTCATAGAAGATAGATATTCTGATTGTGTTCTGTATACAATGGTAAGGGTCTGGTGATTGTAT
CCTGAGATTAAAAAACAACCTTGTGAGTGCCTTAAGTCACGTACTTTACCCTATATATACCG
CAGCACAATAAAGCAAGGTATCAGCCATTTTGGTCTGATCCTCTCAACCCCATCTTTTGTCTA
TCTCTTATTTTCTTAGCGGGGACGCTCCGTTCTCTCCCTGTGCAGGTGCGACTCTTGCTTGTGC
TGGCCGCGGCA

>NC_056061.1:52960381-52967871#SHEEP_RIP_18(+)

CATAACAAAGGGTTATGAGCGTTCACTGAGTGCCCAAGGCTGGGAACGGATAATGAAGGGT
TATATGCCCGGCCTTGAGGCGTTCAAAGTCTTCCTTCTGACCTCCTGTTTCTGGGAGCAAGGA
CTGTTGTTTCATGATAAGACTCCTTTCAGAGTTTCGCCAAAGCTATGTTATGGCTTGGGTGGT
GGGAACTGTATTTTATACTTGAATGCTTTGATGTTTTATCGAGAAAGGCTACATGCAAGTCTG
CTTTATGCTCTGCTCCCTGAGACCATATATCTGCAAAAAGATGGATAATAAAATTTGTCAGT
CCACTAGAGGCTGTCCCTGAGTGTTCTTTTCAGAGTGCAGTTCTCCAAGCCTTAGAACTGGC
ACCCGATGTGGGGCTTGAAGCAGCAGACTGATTTTGAAAAAGGGCCGCACTCCTGCAGAAG
CGAGGTAAGCAGAATGGGACATCAGACAAGTAAAATTCCTTTTGTTCATCTCATGCATCATT
TCTTGAAACAATAAGGGGTTAATCTGCTTGAAGAGCAGTTAACTAGCTGTTACCAGACAGTG
GTTGAATATAATCCATGGTTTCTGGAGGAAGGAACCTTGGATTTACAAACCTGGACCAGAGT
AAAGAACAATGTTTTAAAAGCTTATAGACAGGGGGTAAAAATTCCCCCACAATGGTGGGTA
ACTTGGTCTCTTTCACGGGCTGTTATGGAACAATTAGATGGCTCAGGAGGAGATTTAGAGGT
TGAAACTGTTCAATCCCTGCATGAATGTGAATTAGAAGAGAAAGATCTATCAGAGGTTTTAA
ATCAGAAAAATGTCATGCTAGAGCAGGTTACGGACAAACAAGAGTCACAGATGCTGAAAGC
AGTTAAACAATCAACTTTGCCAGAGGCCCCTGATCCGCCTTTACCTTGCTTTTCGCAAGCTCA
TATTAACAAGCCTCCAACACCGGCCTTTGCCTTTCCACCGGCTGCAACGCTGTCTGCTGCTGC
TACTGCTCCCCTATTTCCTAGCAGTCCTCCTCGGGACATGCCTCTGTGCGTGGGCTTTTCCGG
TCCAATTTAATAATCCTCAACCTGGACATAATCAATGGCAATCACCTGATTTTGGTTTGCTCA
CGCAATTCAAAAAGGCATGTACGTTATATGGACCTACCTCACCCTACTGTATGGAATTTCTT
AGGGGCTGGGCTGATCAGTAGCTTCATGTGGATTTTTTTACAGTCGCTAAAATGGTTATGAC
TCCACAACAGCTACTACAATGGCAAATGTGGGTCACGGATGAAGTCAAATTAATCTTGCAAG
AACAGCAAAGCGGGGAAAACCCTGCTGGAGTAAATTTTGAAATTCTCACCGGCACCGGAGC
TATGGCTGAAACTGCTGCGCAATTACAATTAGTGCAGCCGCCCATGTTATACTGGATTAAGG
AAGCAGCTATCAGAGCATGGGCTAAAATCGACAGTTCCACCTCTGATGGATCTTTTGTAAAA
ATATTGCAGGGACCAACTGAAGAATATGCTCAATTTATTGGTAAATTGAAAGAGGCCATTGA
TCACAGTCTTAAGGATGAATCTTTGTGAGAAATCATTTTGAAACAACTGGCTTTTGATAATG
CTAATGAAGATTGTCTATAATAGGCTATTATCAGACCTATTAGGGAGCAAGGACGAGTTATA
GAATACTTGAAAGCCTGCAGGAAGGTGGAGACGATTCAACATAAAGCTAAAATAGCTGCTT
TAGAAACCTTAAATGTTTCCCAAAAGTCTAAAGTTAAATGTTTTAACTGCGGCAAGCCGGGA
CACAGGCGGAAGCAGTGTCACTTGCCTTGGCAAACAGGTCTTTCTCCTGACAGAGGAGGGG
CTATTAAAACTAAGCACCCCCGGGCTCTGCCCAAGATGTAAAAAGGGGAATGATTGGCTGA
GTGAATGCCACTCTAGATTTGATAAACAAGGTAGTGCTTTACCCATTCAGACACCTCCTTCG
GGAAACTAGAACAGGGACTCTCCTCTAGCCCCGTTAAACAAGGAGGACAACCAAATATTAC
TGAATTAACAGCGGCTACCAGACATAGCGCATGTGTAGACATTCTGCTCCCCGAGATATGGA

ATTATTAATGGTAAATAATCCTATGAAAATCTTAGCTGGATATTTTGGCCCTATACCAAAAA
ATACTGTAGGCCTCCTGTTGGGAGGAAGCAGCAGCACCATGCGTGGGATAATTGTACATACT
GGGATCATTGATGAAGATTACATGGGTGAAATTGCAATAATGTTACATGTGACTCGTAGCTT
GTATTTACAAAAGGGTGACAGATTTGCTTAGCTATTGCTATTACCTTATGTGCCCCCACTTAA
TAGAAAAGCAAACACCAGAACAGGTGGCTTTGGGAGTACCAACGTTACTGCAGCCCTCTGT
ACTGTTATAAAAGAAATCAATAGGCCCATGTTAAAATTAAAAATCAGAGGAAGAACTTTTG
AAGGAATGTTAGACACTGGGGCAGATATTTCCATCATAAGAACAGAGGAATGGCCTTCAGA
TTGGCCTGCAGTTTTAGCCTCACACCAGTTGGTGGGAGTAGGAACTGCAGATGCAGCTCAAA
CTTATGTTAGCTCATCTTACTTAAAAGCCCTGGCCCTGATCAATTAGTCGGTTACATTAAAC
CGTACATTGCTCCATTACCGTTAAATTTGCGGGGAAGAGACTTTCTACAAGCTCAAGTGACT
ATACAATTGAATGAAACTTTTTCTTAGGGGTCACTGAGATAAAGCCACTGAAGTTAGAATGG
AAGTCTGATAAACCTATCTGGACAGCTCAGTGGCCCCTATCAAAAGAGAAACTGTCCGCTTT
GCATACTTTGGTGGCTGAACTACTACAACAAAATAGAATAGAAAGTACTTAATCACCATGGA
ATTCACCAATTTTTGTCATTAAAAAGAAATCAGGTAAATGGAGAATGCTAACAGGAATATTA
ACACTATAATGATTCCTATGGGAGCATTATAACCAGGACTCCCAAGCCTTGCTATGGTCCCT
AAGGACTGGGCTGTTATGATTATAGATTTACAAGATTGCTTTTTCACTATACCTTTACATCCA
GATGACAGGCAACATTTTACCTTCTCAATACCTTCCATTAATAATCAAACCCCTGTTCAATGG
TATCAATGGAAGGTCCTGCCCTAAGGTATGATGAACTCTGTTATGGTCTGTCAATTCGTTGTT
GATAAAATTTTGCAGCCCATCAGACAGCAATTCCCTGAGGCATATCTCATTCATTACATGGA
TGACATTTTATTGGCTTCTCCCTCAGAATCTCAATTAAGTTTATTATGTAATGAGGTCAGAAC
TAATTCAACTAATCATGGGCTGCTAATAGCAGAAGATAAATTGCAACATCATTCCCCTATTA
AATATCTTGGATATCTTATGGACCGCTCCACTGTAAAGCCGCAGAAACTTTCTATTAGAAGG
GATAATTTACATACACTTAATGATTTCCAGAAACTTCTTGGGGATATTAATTGGCTACGACCC
ACCTTGGGAATTCCCACATATGCTTTACAAAACTTATTCAAATTATTAGACGGTTCCTCTGAT
TTGAATAGTTCCCAACAACTTACCCCTGAAGCTGAGGAAGAATTACAATTGGTAGAACAGA
GAATTCAACAGGCATTTGTTTACTGTATTAATTATAATTCCCCTTTTCAGATATATGTCTTTG
GCACTAAGATATCGCCTACCGCCATTATAGTGCAGGATAATCACCCTATTGAATGGGTATAT
CTCCATTCCAAACAGACTAAACACATTGTTTCCTATATAGACTTAATAGGGAAAATCATTTTT
CTTGCACGCTCTCACTTGTGCGCTATAGCTGGATATGACCCTACTCAGATTTACCTACCTTTG
ACAAAAATAGAAATTGATAATGCTCTCCAGGTGTCCACTACCATTCAGATAGCCCTTGCTGA
TTATTCAGGGGAGTTATTGGCCAACCCACCTGAAGGAAAATTATGGAATTTCTTACAAAATA
CTTCTTTTATCATTAACAATATTGTTTCTGAACACCCTCTCATGAATGCACCTAATTATTTTAT
AGATGGAAATAAGGCAGGATGGGCAGCCATAATAGGTCCCAACCTGCAAAAGAAAATTAAA
AGTCCTTATCAATCCATTCAAAAAACAGAATTATTTGCATTATATTGTTTACTTACTCTAATA
AAAACCCCATTAAATGTTTTAACTGATTCTCGCTACGTGGCACATCTTTTCCCATCTTTGTAA
CGGCTCATTTTATATCCAATGAAAATGATCTTATACATTTGTTCTTATTGATTCAACAAGAAA
TAAAAGCTAGACTCCATCCCTTCTTTATTACTCACATTCGTGCTCATTCCCGTTTACCAGGAC
CCCTCAGTTTAGGCAATGATTTGGCTGATCGCCTCATCGCCCCTATATTTTCTTTCCCCGAAC
AGGAACATCAGCTCTTCCATACTAACGCTAATAGACTACACGTTCAGTATAAGATACCGTTA
CAAATGGCTAGAAAAATTGTTCAGGACTGTGCCGCATGTGCCCCCTTTCATTTGACCACTAG
TCCCCAAGGGACTAATCCTAGAGGCTTACACGCAATGGCAAGCTGATTTTACACATTACAAA
CTGCCCCCTTTTAAATCATTATTTATAGTCATAGACACCTTTTATGGCTTCATTTGGGCAGTTC
CTTCCACCGCTGAGACTACTAAAGCTGCCGTCACAGCTTTTCTGCAATGTTTTTCAGTGATGG
GGATCCCTGCCTCCATCAAAACAGACAATGGTCCTGCTTTTACAGCCAATGCTTTTTGTGATT
TCATGCATCAGTGGGGGTATTTGCCATCTTACTGGCATCCCGTACAACCCTCAAGGTCAAGC
CATCATTGAATGGGCCCACTGTACACTCAAGCTCGTTCTTAATAAACAAAACAGGGGGAATA
GACTAAGGGACCCCTATGGATCTAAAGCCATTTTGCCTATAGCCCTTTTAACAATCAATTATT

TTAATTTGCCTCTACACAGTCAGGAAACACGAGCAGAGTGACATTTTTCTGATTCACCCTCA
CGTATGCCTGAACAAACTGCTCTTTGGGTTAAATGTCTTGATCAATGGCGGCCAGGAACACT
TAAGTTCCTAGGCAAGGAATATTGTCTTGTCATTTTAGATGATGGAACCGAATAGTGGGTCC
CACTCAGAAGAGTCAGAAGACGGGCAGGCCTTACTCCACACCCCCCGACCGGTAACTAAAA
CAAAAATTGCGACAGATGACACTGAAAGACAGCAAGAAAATGAAACGTCCCTGACGCACTG
CGCCTTTTCCGACCTGGGCACAAATGAAAAATCTGTCCAGACGAGCTGAGGATACTCTGTTG
ATGACCAACAGTGAGGTAACACCGGAAAAACTGTTGCTGGCCATGATGGCCGTTTTAACCTG
TGCTTCTGGGGTAAGTGGTAATTACACCTATTGGGCTTATATCCCCAACCTGCCTCTTTTACA
AGTGGTGGATTGGACAGAATCATGATAGCTTACATTTTCCTGCCCCCTGGTCGGATCTAGGA
CCACGAATAAAAGAAGAAGAAGGAAAGACAGTTCGGTGCCCTCCCTTTGTGTCTCCGCCTAT
ATCCACAATGGTGGGCTTACTCTTTCTCAAATGGCTCTTTCCTTCTTGGAATGTTCGCTACCA
TAACCTTTGTTTTTAACTGGACAGAGACTACCTACCATGGACAAATAGCCAACTTCAACCAC
TATCTATTTAATTCTTCTGAGCCTCCTTGTGACAACGTTATCTTGGAAGAAAAAATCATCTGT
GCCAGGAATGGACTGGTCCACCTGTAGAGGAAGGTTTGGAAAAGTTTCTGTTCATGCCAACA
TGACTTTTGTTGATTGGGGGCCTCATGGACTCTGTGAATTGCCCAGAATCACAAGAAAATAA
TAACACCTGCCAATGGTATAATGTTTCAGCACCACATTTTAACAGAACACAAACAGGACTCT
GGCATCAATGCGATGAACTTTTGAACTGGTACAATGGAGGTCTTTCACCTCCCAGACCCTGG
ATAATCAGTCCAGTCTTGGGGCCCGAACACTGGCATCTTTGGAAAATTCCTGCTTGTTTGTCT
CGGTTTAGTGTCTCATATGCTTATCACATGTTTCCATCCCACAAAATTATACTATTGAGTATA
ATTATACTGGTTATGTTCGTGCTTGTGTAAATGCAGCCTATCTTTTTGCTATTGGGCAATTTA
GGAGTAATGGCTCAATTCTGTCCTGTACTGAGTGTCATTTGTATACTTGTTTAAATCATAGTG
TGCCAATTAATGTTACTAAAGACAGTGTTTTTTAAGTCTGGCAAAGGACTGATTTGTGGGTTC
CAGTTAAGATTTCTGAACCTTGGTCAGATTCCACGTTGTTGTCTTTTGTTCTGAGAGAATCCC
TAAAAAGAAGCAAATGCTTTATTGGCTGGATTATAGCTGCCATAGTGGGTATTCTTTCAGTT
GTAACTGTTGGTACAGTTTCTGGAAAGGCATTGTGTAATTCTATTCAAAATCATGATTTCATT
AATGCTTGGAAAAAGGATTCTCATGATCTCTGGGCCCGGCAAGCTCAGATAGATCAACAAAT
ACAAACACGCTTAGATGACCTACAAGCCGCCCTTATGTATGTGGGGGATGATCTGCATGCTT
TACAGATCCAGTTGAAGTTGCGGTGTCACTGGAATTTCACTACTTTCTGTTTGACCAATATGC
CATACAATGCCACTGAATATCCTTGAGAACAAATAAAGTTACACCTTTTAGGTTCGAAATCA
AACACTAGTCTAGATATAGAGAAACTGAAGCAACAAATCATGCCTACCTTTAGCAGCATGCC
CCCTGTGTTATATAACACTGACTTCTGTAATACACTATCCGCTAGTACTTCCTTTTTAAATCC
AAAAAAATGGGTACCCCATACGATGGCTTCTTATGCGCTGATCTGTTTATTGTTTGTTATTGT
TCTTATAGGATTCCGAACGTTATGTGCTTGAGCCACTGCCGCCCAAAAAGCAGGAGTAACCA
TGGCTGCAGCAGTCCTTGCTCTTGAGGAAAAAGGGAGGAAATGCGGAGGAGCTGGAAGGCT
CCTAAGCATACACTGAGAATGTATTTGCTCCACTCATGACGAAGGTTGGAAGCTGGAATGAG
CATAACAAAGGGTTATGAGCGTTCACTGAGTGCCCAAGGCTGGGAATGGATAATCAAGGGT
TATATGCCTGGCCTTGAGGCATTTGAAGGCTTCCTTCTGACCACCTGTTTCTGGGAGCAAGG
ACTATTGTTTCATGATAAGACTCCTTTTAGAGTTTTGCCAAAGCTATGTTATGGCTTGGGTGG
TGGGAACTGTATTTTATGCTTGAATGCTTTGATGTTTTATCGAGAAAGGCTACATGCAAGTCT
ACTTTATGCTCTGCTCCCTGAGA

>NC_056063.1:18713439-18721364#SHEEP_RIP_19(+)

TGCGGGGGACGACCCGTGAAGGGTTAAGTCTTGGGAGCTCCCTGGCAGGTATGCCGGGCCC
TAGGACATGTGCCTAAGCTCCCTGTCCCGCCACCCTCAAGAACTTTTGTAACCCTTAAGGCTC
CAAGATGTTTGGTTTCGGCAACATTTCATAGAAGATAGATTATCTTATTGTGTATACTTCATA
GAAGATAGATATTCTGATTGTGTTCTATATACAATGGTAAGGGTCTGGTGATTGTATCCTGA
GATTAAAAAACAACCTTGTGAGTGCCTTAAGTTACGTACTTTACCCTATATATACCGCAGCA

CAATAAAGCAAGGTATCAGCCATTTTGGTCTGATCCTCTCAACCCCATCTTTTGTCTATCTCT
TATTTTCTTAGCGGGGACGCTCCGTTCTCTCCCTGTGCAGGTGTGACTCTTGCTTGTGCTGGC
CGCGGCAGGTGGCGCCCAACGTGGGGCTCGAGCTCGACAGTTTTCCTCGCCACTACTCTTAT
TAATTGAAAAGAGTGAGTATATGAGTAAACAAGTGAATTAATTTGAGGAGGAGTAGTAAGG
TATATAGTTGAGAGTATAAATATGGGACAGACGCATAGTCGTCAGTTGTTTGTGCATATGTT
ATCTGTAATGTTAAAACATAGGGGAATTACTGTTTCTAAACCTAAATTAATCAATTTTCTTTC
ATTCATCGAGGAAGTTTGCCCTTGGTTCCCCAGAGAAGGTACAGTAAATTTAGAGACATGGA
AGAAGGTAGGGGAACAAATTCAGACTCATTATACTTTACATGGCCCTGAAAAAATCCCTGTC
GAAACTTTATCCTTTTGGACACTAATTCGTGACTGCCTGGACTTTGATAATGATGAATTAAAA
CGTTTAGGAAATTTATTAAAACAGGAAGACGATCCTCTCCATGTTCCTGATTCGGAACCCAG
GTATGCTGTTCCCGAGGGGGTTGAAAGCAACCCTCCGTTTTCTAACTTATTGCGTCCTTCGGA
TAATGATGATTTACTTTCATCCACAGATGAGGCAGAATTAGACGAAGAAGCTGCTAAATACC
ATCAAGAAGATTGGGGTTTTTTAGCACAAGAAAAAGGGGCGTTAACATCTAAAGATGAATT
GGTTAAATGTTTTAAAAACCTCACTATTGCTTTACAGAACGCAGGAATCAAGCTTCCTAGTA
ACAATGCCAAATCTCCTTCTGCTCCGCCTCTTCCCCCTGCTTATGCTCCTTCTGTTGTGGCTGG
TCTCGATCCCCGTCCAGGGCCCCCTCCACCGTCTGAGAACATGTCTCCGCTGCAAAAGGCAT
TGAGACAGGCACAGCGACTTGGTGAGGTTGTCTCTGATTTTTCTCTTGCTTTTCCTGTCTTTG
AAAATAACAACCAGCGTTATTATGAATCACTGCCTTTTAAACAACTGAAAGAGTTAAAGATT
GCTTGCTCACAATATGGTCCTACCGCTCCATTCACCATTGCTATGATAGAAAATTTGGGTACT
CAAGCTTTACCTCCAAATGATTGGAAGCAGACAGCTAGGGCATGTCTCTCAGGGGAGATTAT
TTATTATGGAAATCTGAATTTTTGAACAATGTGCTCGTATAGCTGATGTTAACCGACAGCAA
GGTATACAGACCTCCTATGAAATGTTGATTGGTGAAGGCCCTTACCAGGCTACTGATACTCA
ACTTAATTTCTTACCTGGTGCATATGCACAAATATCAAATGCGGCTCGGCAGGCATGGAAAA
AACTTCCTAGCTCCAGTACTAAGACAGAGGATCTTTCAAAAGTCCGGCAGGGACCTGATGAG
CCTTACCAGGACTTCGTGGCACGACTTTTAGATACTATAGGTAAGATAATGTCAGATGAACA
GGCTGGGATGTTATTGGCAAAACAATTGGCTTTTGAAAACGCTAACTCTGCTTGTCAAGCTG
CTTTAAGACCTTATCGAAAAAGGAGATCTGTCTGATTTTATTCGCATTTGTGCTGACATTGGA
CCCTCCTACATGCAAGGCATTGCTATGGCAGCAGCATTACAAGGAAAAAGCATAAAGAGGT
ACTTTTCCAGCAGCAAGCCCGGAACAAGAAAGGACTTCAAAAGTCAGGTAATTCGGGTTGC
TTTGTTTGTGGTCAGCCTGGCCATCGGGCTGCAGTGTGCCCTCAAAACAACAAAGCCCTGTT
AACACTCCTAATTTGTGCCCACGCTGTAAAAAAGGAAAGCATTGGGCGCGGGATTGCCGTTC
CAAAACGGATGTTCAAGGTAATCCTTTGCCCCGGTTTCGGGAAACTGGGTGAGGGCCAGCCC
TGGCCCCGAAACAATGTTATGGGGCAACACTGCAGGTTCCAAAGGACCATTGCAGACCTCTG
TCGAGCCACAAGAGGCAGCGCGGGATTGGACCTCTGTGCCACCTCCTACACAGTATTAACTC
CCGAGATGGGGGTCCAAACCCTTACCACAGGAGTGTTTGGGCCTTTACCTCCAGGGACAGCT
GGACTGCTTTTAGGGCGCAGCAGTGCGTCTTTAAAAGGAATACTTATTCATCCTGGTGTGAT
TGACTCTGATTATACAGGAGAGATAAAAATATTAGCCTCCGCTCCTAACAAAATTATTGTGA
TCAATGCAGGACAGCGTATAGCTCAACTTCTTTTAGTTCCATTAGTCATACAAGGAAAAACA
ATTAACCGAGACCGTCAAGATAAAGGTTTCGGGTCCTCTGACGCCTTTTGGGTGCAAAATGT
TACCAAGGCACGACCAGAACTTGAGCTACGCATTAATGGTAAGCTTTTCCGCGGAGTGCTTG
ATACAGGGGCCGATATTAGTGTTATTTCTGATAAATATTGGCCTACTACATGGCCAAAACAG
ATGGCTATTTCCACTCTCCAGGGTATTGGCCAAACTACCAATCCAGAACAGAGTTCATCCCT
TCTTACTTGGAAGGATAAAGATGGACATACAGGCCAATTTAAACCTTATATTCTGCCCTATC
TTCCAGTTAATCTATGGGGGCGTGATATATTAAGCAAAATGGGTGTTTATTTATATAGTCCTT
CACCCACTGTGACAGATTTGATGTTAGATCAGGGCTTACTTCCAAATCAAGGTTTAGGTAAA
CAACATCAAGGCATCATTTTGCCCCTTGATTTAAAACCTAATCAAGATCGAAAAGGCTTGGG
GTGTTTTCCTAGGGACCTCTGATTCTCCTGTGACACATGCCGATCCTATTGATTGGAAATCTG

AGGAACCGGTATGGGTCGATCAGTAACCCCTAACACAGGAAAAACTTTCTGCCGCACAACA
GCTGGTGCAGGAACAGCTGAGACTTGGGCATATTGAACCCTCTACCTCTGCTTGGAATTCCC
AATTTTGTTATTAAAAGAAGTCTGGGAAATGGAGATTGCTACAAGACCTTCGTAAGGTAAAT
GAAACAATGATGCATATGGGAGCCCTACAACCTGGGTTGCCCACTCCTTCTGCTATACCTGA
TAAATCCTATATCATTGTTATAGATTTAAAAGATTGTTTTTACACTATTCCTCTTGCACCTCA
AGATTGCAAAAGATTTGCTTTCAGTTTACCCTCTGTTAATTTTAAAGAGCCTATGCAACGCTA
TCAATGGAGAGTTCTCCCGCAAGGAATGACTAATAGCCCTACGCTGTGCCAAAAATTTGTTG
CTACAGCAATAGCTCCCGTTCGTCAACATTTTCCTCAGCTATATTTGGTTCATTATATGGATG
ATATATTACTAGCTCATGCTGACGAACATCTATTGTATCAAGCTTTTTCGATTCTAAAACAAC
ATTTAAGCCTTAATGGTCTTGTTATTGCTGATGAAAAAATTCAGACTCATTTTCCTTATAATT
ATTTGGGTTTCTCCTTATATCCTCGTGTTTATAATACCCAATTAGTACAATTACAGACTGACC
ATTTAAAAACTCTAAATGACTTTCAAAAACTTTTAGGAGACATTAATTGGATACATCCTTATT
TAAAATTACCCACTTATACCTTGCAGCCATTATTTGACATCCTTAAAGGTGACTCTGATCCTG
CGTCACCCCGAACACTTTCTTTAGAAGGACGAACTGCTTTACAATCAATAGAAGAAGCTATT
AGACAACAACAGATTACTTATTGTGACTACGAACGATCATGGGGTTTGTATATACTTCCTAC
CCCCCGAGCACCCACAGGGGTTCTCTATCAAGATAAACCTTTGCGATGGATATATTTGTCTG
CTACTCCAACTAAACATCTGCTCCCTTACTATGAACTTGTTGCAAAATTGTAGCAAAGGGAC
GTCACGAGGCCATCCAATATTTTGGTATGGAACCCCCTTCATTTGTGTTCCTTATGCTTTAGA
ACAACAAGATTGGCTTTTTCAATTTTCAGATAATTGGTCTATAGCTTTTGCAAATTACCCGGG
ACGGATTACTCATCATTACCCTTCTGATAAATTGTTACAATTTGCTAGCTCTCATGCCTTTATT
TTTCCAAAAATAGTTCGCCAACAACCTATTCCCGAAGCGACACTTATATTTACAGATGGATC
TTCTAATGGAACTGCAGCTTTAATCATTAACCATCAAACCTATTACGCACAAACCAGTTTTTC
TTCTGCTCAAGTTGTGGAATTATTTGCAGTCCACCAAGAGTTGCTAACTGTACCTACTTCCTT
CAATTTATTTACGGACAGCTCCTATGTGGTCGGTGCCTTACAGATGATTGAAACTGTTCCAAT
TATCGGCACCACCTCTCCTGAAGTTCTTAACTTATTTACATTGATTCAACAGGTTCTCCATTG
CCGCCAACACCCCTGTTTCTTTGGACATATTCGTGCACATTCCACCCTTCCTGGTGCCCTGGT
ACAAGGCAATCACACTGCGGACGTTCTTACTAAACAAGTGTTTTTCCAATCAGCTATTGATG
CAGCCCGAAAATCCCATGATTTACATCACCAAAATAGTCATTCTTTACGGTTGCAATTTAAA
ATTTCCCGTGAAGCTGCACGGCAAATTGTTAAATCTTGCTCTACTTGTCCTCAATTCTTTGTT
CTCCCTCAATATGGTGTCAACCCTCGAGGTTTACGCCCTAATCACCTCTGGCAAACAGATGTT
ACTCACATTCCTCAATTTGGACGTCTTAAATATGTTCATGTCTCTATTGACACTTTTTCCAATT
TTCTCATGGCTTCCCTTCACACTGGAGAATCAACACGTCACTGTATTCAACATTTGCTGTTTT
GCTTTTCTACTTCAGGAATCCCACAAACCCTTAAAACAGATAATGGACCTGGTTATACTAGC
CGTTCTTTTCAACGTTTTGTCTTTCTTTCCAAATTCATCATAAAACAGGAATTCCTTATAATCC
ACAGGGACAAGGTATTGTGGAACGAGCCCATCAACGCCTTAAACATCAATTATTAAAACAA
AAAAAGGGGAATGAACTGTATAGCCCCTCACCGCATAACGCCTTAAACCATGCTCTTTATGT
TTTAAATTTTTTAACTTTAGACGCAGAAGGCAATTCAGCAGCCCAGAGTTTTTGGGGAGAAC
GATCCTCATGCAAAAAACCACTTGTACGATGGAAGGATCCACTTACCAATCTGTGGTATGGG
CCAGACCCTGTACTAATATGGGGACGAGGGCATGTTTGTGTTTTTCCACAGGATGCCGAAGC
GCCGCGCTGGATTCCGGAAAGGCTGGTACGCGCGGCAGAGAACTCCCTGACACATCAAATG
CAACGCATGACACTGAGCGAGCCCACGAGTGAGCTGCCTACCCAGAGGCAGATTGAGGCGC
TGATGCGATATGCTTGGAATGAGGCTCATGTACAACCTCCAGTGACGCCTGCAAAAATACTG
ATCATGTTATTATTATTGTTACAGCGGATACAAAACGGGGCAGCTGCGGCTTTTTGGGCATA
CATTCCTGATCCGCCTATGATTCAATCCTTAGGATGGGATAAAGAAACAGTACCTGTATATG
TTAATGATAGAAGTCTTTTAGGAGGAAAATCAGATATTCACATTTCTCCTCAGCAAGCCAAT
ATCTCCTTTTATGGTCTTACTACTCAATACCCTATGTGCTTTTCTTATCAATCACAGCATCCTC
ATTGTATACAGGTGTCAGCTGATATATCCTATCCTCGAGTGACTATTTCAGGCATTGATGAA

AAAACCGGAAAGAGATCGTACGGTGACGGAACCGGACCCCCTCGACATTCCGTTTTGTGAC
AAACATTTAAGCATCGGCATAGGAATAGACACTCCTTGGACTTTATGTCGAGCACGAATTGC
ATCGGTGTATAACATCAACAATGCCAATACCACCCTTTTATGGGACTGGGCACCTGGAGGAA
CACCTGATTTCCCCGAATATCGAGGACAGCATCCACCCATTCTTTCTGTAAACACTGCTCCTA
TATATCAGACAGAACTATGGAAACTTTTGGCTGCTTTTGGTCATGGCAATAGCCTATATTTAC
AGCCCAATATTAGTGGAAGTAAATATGATGATGTGGGAGTTACAGGATTTTTATATCCCCGA
GCTTGTGTCCCTTATCCATTCATGTTGATACAAGGCCATATGGAAATAACACTGTCATTGAAT
ATTTATCATTTAAATTGTTCTAATTGCATACTTACTAATTGCATTAGAGGTGTAGCCAAAGGA
GAACAAGTTATAATAGTAAAACAACCTGCTTTTGTAATGTTACCTGTTGAAATAACTGAAGA
ATGGTATGATGAAACTGCTTTAGAATTGTTACAACGCATTAATACGGCTCTTAGCCGTCCTA
AAAGAGGTCTGAGCCTGATTATTCTGGGTATAGTGTCTTTAATCACCCTTATAGCAACTGCTG
TTACTGCTTCTGTATCTTTAGCACAATCCATTCAAGCTGCTCATACTGTAGATTCCTTGTCAT
ATAATGTTACTAAAGTAATGGGAACTCAAGAAGATATAGATAAAAAAATAGAAGATAGATT
ATCCGCTTTATATGATGTAGTTAGAGTTCTAGGAGAACAAGTTCAGAGCATTAATTTTCGCA
TGAAAATTCAATGCCATGCTAATTATAAATGGATTTGTGTTACAAAAAAGCCTTACAATACT
TCTGACTTTCCGTGGGATAAGGTGAAAAAACATCTGCAAGGAATTTGGTTTAATACTAATGT
TTCTTTAGATCTTTTACAATTGCGCAATGAAATTCTTGACATCGAAAATTCTCCAAAAGCTAC
TTTGAATATAGCTGATACGGTCGATAATTTTTTTACAAAATTTATTTTCTAACTTTCCTAGCCT
TCATTCACTGTGGCGAAGTATAATTGCTATGGGCGAGGTTCTGACTGTTGTGCTTATCATAAT
TTGTCTAGCTCCTTGCCTTATTCGTAGTATTGTTAAAGAATTTCTACATATGAGAGTTTTAAT
ACATAAAAACATGTTGCAACACCAACATCTTATGGAGCTTTTAAAAAATAAAGAGAGGGGA
GCTGCGGGGGACGACCCGTGAAGGGTTAAGTCTTGGGAGCTCCCTGGCAGGTATGCCGGGC
CCTAGGACATGTGCCTAAGCTCCCTGTCCCGCCACCCTCAAGAACTTTTGTAACCCTTAAGG
CTCCAAGATGTTTGGTTTCGGCAACATTTCATAGAAGATAGATTATCTTATTGTGTATACTTC
ATAGAAGATAGATATTCTGATTGTGTTCTATATACAATGGTAAGGGTCTGGTGATTGTATCCT
GAGATTAAAAAACAACCTTGTGAGTGCCTTAAGTCACGTACTTTACCCTATATATACCGCAG
CACAATAAAGCAAGGTATCAGCCATTTTGGTCTGATCCTCTCAACCCCATCTTTTGTCTATCT
CTTATTTTCTTAGCGGGGACGCTCCGTTCTCTCCCTGTGCAGGTGTGACTCTTGCTTGTGCTG
GCCGCGGCA

>NC_056063.1:24352091-24359775#SHEEP_RIP_20(-)

TGAAGGGTTAATAGGGTAGCAGAGATGTGCCTGCAAATGGGCCTCTCTGCTAGGGCTGGAC
ATCCTTGCAAATGAGGTGTTCTGCCAAAGAGTCTGGACACAGCCTTGAGTTTAATGGTCCCT
TGCAAACAGGGAGCATTCCCTTCTTGTGATAAAGAAGGAATAGAGGGCTTTGTACAGACTCT
GCAGTAGACTAGGATTTTACTCCCCTTTGCTATATGATAACATATATGCACCTGCGCTGTGCT
GAAAAGGCTCATTCATGCAGTCTGGAATTCTGCCTAGGGGGCTTTTATAATAAACAGCAATT
AGTTTTTTGCCCAGTTCTGTTCCTCTGGCCGGAGTGTGCGTGTCATCTGTCTCTTGTGTGTCTT
GTTCTGTGTCATTTCACTCGTAATCTCCAACATCTGGCACCCAACGTGGGGTTCGAGTGAAA
CCGAAAGGGTGAGTAACCCCGGGGGGATTTTAAATCCATAGCAGGGGAACTTTTGGGAAAA
TTGTGAGGAATTCCTCACCCTAGCAGAGGGGAGCTTTCAAAAAAATCGTGGGGAATTCCTCA
TCATTACGGGCACAATACATGGAGTTAGTCCAAAGGCTTCTCCACTCCATAGGCATTAAAGC
CTCAACTCGTCAGTTGAGTGAGCTCTTTCGCTTGGTGGAGCAATATTGTCATTGGTTTCAATA
TCAAACTAAGTTACAGTTAAACTTGAAGGAATGGAAAATAATTCAAAAGGAATTGAGAAAG
CAACATCAGAAGGGTAATGTAATCCCTTTGAAGTTATGGACTTCGTGTAGTGCTATAACACA
GGGTTTGACCTTGCTCTCTACTGATAATGAAACGAAATCTAATGCTTCAAGGAGGGGAGAAA
TAATTTATGGGGATGTGTCAGACGTTGGTGGGGCTTCTGCATCGCCTGAAGGCAAGGATACA
AATGAGCCTCCTCCTGTAAATGGTGAAACATTGGATAGTTCAGAATCAGATTTGGAGGCTTC

TTTGGTTTTGTCAGAGGAGGGCAAAGAGATTAAAGAAATGACCCATCTATTCCAGGAATGGT
GGAAATCCCTTAAGGAGGAGAAAAAATCTACACCTTCTGCTCCTGCTTGTGCTTCTCTTCTCC
CCACTGCGGTTAATCGGCCCGATGTGGGCAGGGAACATTGTCGGTTCTCCTTTCCTTTGTCTG
TGCTTCATGATGATGACTTGCCTGCTCCCCACAATTGTTTCCCATCCAGAGACAGCAGGATG
GCAATGTGATAAATGTACAATATAATACGCTCCTTTGGAATATAAATTTTTTAAAGATCTTA
AGGCTGCAGTAGCGCAGTACGGTCCTCAGCTCCCTTTGTTTTGGCTATGCTGGAATCATTGG
GAAAAGGCAAATTAATCATTCCGTTAGATTGGGAATCTATTGCCCAAGCTGTCTTGGAGGGG
TCTCAATGGTTGCAACTTCGTAGCTGGTGGGAAGAAGCTAGAAAGCAGGCTCAGATTAATG
AGGGACAGAATCCCCCTGGTCCTCTTGAGGACAAGCTAATGGGAGAGGGCCAATATCGGGC
TTTAAGAGAACAGACTCAATACTCTGATCAGGACTTACAACAAGTCCGCCAGGTCTTTTTAC
GAGCATGGCGCCGTGTGGTGCCTACTGGCCACGCCCAGCCCTCCTTTGTTAAAACAATGCAA
GGCCCCAATGAGCCATATACTGATTTTCTAGCAAGATTGAGGGTAGCTGTGGAACGGGCTAT
AGGGAGGGATGAGATTTCAGAGATATTATTACAAACTTTAGCATTTGAAAATGCAAATCCTG
AATGCAAGCGTATACTGGGACCTTTAAAGGGACAGGGTGCATCTATAGCTGAATATATCAG
AGCCTGCTCAGGAGTAGGAGGAACTGAGCATCAGGCTAATGTCTTTGCTACAGCCTTGGCCA
AAGCTATGAGACCACAAAAGGGAGGTAACTGCTTCCATTGTGGAAAACCTGGTCATATGAG
AAGAGAATGTCAGAAATTAAAAGATGATCAAGGTGCAATTCCTAAAGACAGATATCTTGCT
GGGAAGAATAAGACTTCTCCTGGACTTTGCCGTCAGTGTGGGAAGGGGTTTCATTGGACTAA
TGAATGCAGATCTAAAACAGACAAAATAGGCAACCCGATACCGGGAAACTATCCTGCGGGC
CTAAGTCCTTGGGGCCCAGGAACAATACCGGGGACTTCTCCTCCTTGCCCTCTTCCCCATCCC
ATCTGCCCCAACCCTATTCCCTCCCAACAACCATTACGAGTCGATGCCCCATTAAAAGGACC
TCAGATGATGATTTCGGACTTAACGGTCTGCTACTTCAGGGAGTGCTGCTGCTGATTTGCCAC
TAGCTGATAATGTTCTTTTGTCACCAGGGGAAGGCATTTATAAATTAAAAACAAATGTATTT
GGACCACTGCCTAAAGGCACTTTTGACTTGATATTAGGCCATAGCAGCGCGGCTTTGAGAGG
TTTAACCATAATTCCTGGGGTAATAGACTCTGATTAAGTTGGGGAAATTTTAATTATGGTCTC
TACTTCTACCACACTTTCATTGTTAGCTGGGGAACGTATTGCTCAAATACTTCTCCTATCTTA
TCATCCCTTTTTGGCTCTTCCTAATGAATGAACAGGAGGATTTGGAAGTACTGGGCGACATA
TATTTTGGGAAATGCTTATCAAAGATTCCCGCCCTGTTCTCGCTTTGATTATACAGGGAAACA
ACTCTGAAGGACTAGTAGACACAGGGGCGGATGTTTCAGTCATTTCTTCTCAACAATGGCCC
CAAGATTGGGAAAAAGAAAAAAGCCCTTTAATGCTGACGGGATTGGGCTCCATTGCAGATG
TCTGGAAGAGTACCCATCCCTTGCGATGTCAATTCCATAATGGAAGATCAGTGTTTGTTACCT
TTTATACTGTAAATATACCTATTAATATATGGGGAAGAGATCTTCTCTCTCCTTTGGGGGCTT
CTGTAACCATTCCATTGGAAAACTAGTAGCCACTGCTCAAATTCCTCGAGCACTCCCATTAA
AATGGTTACCTAATACTCCAAAATGGGTTGAGCAGTGGCCATTACCACAAATGAAGCTCGAG
GCATTAGAACAATTAGTACAAGAACAACTCCAACTTGGTCATATAGAGCCCTCTACCTCACC
CTGGAATTCTCCTGTTTTGGTTATAAAAAAGAAATCTGGAAAATGGAGAATGTTAACTGATT
TACGAGAAGTTAATAAATGTATTGAACCTATGGGAGCATTACAATTGGGACTCCCCTCTCCA
GCTCTTATTCCTCAGAATTGGTCCTTAATGGTGTTAGATCTTAAAGACTGTCTTTTTTACCATT
CCCCTACAATTGCAAGATAGAGATAAATTTGCTTTTACAGTTCCTGTTCTTAATCATGCTCAG
CCTGTTAAGCGTTATCAATGGACAGTCTTACCACAAGGAATGATAAATTGTCCTACCTTATG
CCAAGAATTCGTAGCTCGCTCTTTACAATCCCTCCATCAAGAATACCCCAATTATAGTCTATA
TCATTATATGGATGATCTCCTCTTGGCAGCTCCTAGTATTGCTGAACGTGATGAATTCTTTTT
AAAAGTACAGGAGGCTTTAAGACTATACAATTTGCAAATAGCCCCAGAAAAAATTCAAAAG
GACTTTCCTATTTCATATTTAGGGACAATATTGGAACAACATAGAATTAGGCCCCAAAAGTT
GCAAATTAGAAGAGACCATCTCAAAACCTTAAATGATTTTCAAAAGTTATTGGGAGATATTA
ATTGGCTACGCCTGGTACTTGGGATTCCTACTTATCAATTATGACATTTGTTTTCTACTTTAG
AAGGAGATACAGCTCTGGATAGCCCCCGGACCTTAACCCCATTGGCTTTACGGGAACTTCAA

TTTGTTGAGCAATGACTAAATGATGGCTTTTTGACTTACTTACATGCATCTCAACCTATTTCT
TTTATAATATTTCATACCTCTTATTCCCCATCTGGTGTAATTGCTCAAGAAAAAGGATTAATA
GAATGGGTTTTCTTACCTAACAGTTTTTCCAAAAAATTGACTATATATATGGATAAATTAGCC
TTCCTTATACAGAAGGGTCACCATCGTATTTTACAATTATCAGGATGTGAACCACACCAGAT
TGTTACTCAGTTAACAACTGCTCAAATATCTCGATGTTTACAATTTAATGAAAACTGGAAATT
TTCTCTTGCCTCATATCCTGATTTGTTTTCTAATCATTATCCATCATCTAAATTGATTGATTTT
CTCTGGATTAACAGAGAAAATATATCTCATTCCCCAATTTCAGATGTTCCAGTTAAGGGACC
CACTATTTTTACAGGTGCAAATAAAAATACTGCTGGATATTGGACCCTGGAAAATTCCAAGG
TTCTCCCTCACTCATTTTCTTCTGTACAGCCCACTGAATTATGGGCTGTCTATTTAGTTTTGCA
AGATTTTCCCCAACTTCCTATTAACATTGTTTCAGATTCTCGATATGCTGTTCTCTCTTGCCTA
CAGCTTCTCCATGTCTCCCTTCCATTGACTCTTAAAACAGCTATTGATAAATTGTTTTACCAA
GTACAACAATTGCTCTTGCAGCGTTCAGAGTTAATTTTCTTTACTCACATCCGTGCACATTCT
GCCCTTCCTGGACCCTTATCATCCAGAAATGCTACAATTGATGCCTTACTTTATCCTATAGAA
GCAGCAAAACAAGAACATCTCTTACAACATACCAACTCCAAAGGGTTACAAAAATCTCATG
CTATTACTTGAAAACAAGCTCAAAATATTGTTCGTTCTTGTTCCATATGTGCACCCTTTGCTT
TGCCATTTACCCCACCAGGTGTCAACATGAGAGGACTACAAGCAAATCAGATATGGCAAAT
GGATGTAATTTACATTTCTTCCTTTGGACAACAAAAATGTGCGCATCATACTATAGATACTTG
CACACATTTTCAATGGGCCACTGCATTACATTCTGAAAAGGCTGACGCTGTTATTACTCATTT
GTTCTCTTGTTTTGCAGTTATGGGATTACCAATTGAATTGAAAACTGATAATGCACCTGCTTA
CCAATCTGCAAAATTAGCTCACTTTTTATCTCAATACCACATAACTCATACTTTTGGTATTCC
TTATAATAGTCAAGGGCAAGCTATCATTGAAAGAGCTAATCGTACCTTGCTTGATTATCTTG
AAAAAATAAAAAAGGGGGAACAAGAGAGATTTATGAAACCTAAAGACATTCTGAATAAAA
CCTTACTTACCCTAAATTTTTTGAATGTTTGGAGCAAGGGAAATCTATCAGCAGCAGAGTTG
CATTTTCAAGGGAAAGAAGAGGATAAGAAGATCTTGAATACGCCTATTTGGTATAAAGATA
AAGAGAAAGGTTGGATCCCAACATCATTAATATATTTGGGACGAGGGTATGCTTTCATTTCT
GTTGATAATTACAGGTTTTGGACCCCAGCAAGAGTGATCAAAATCAACAATGGCTGATCCCT
TTGTTCAAAAATTAGAAGAGCTTACTATACAGAGAAGCCTTACTTCTCGTACAAGGGAAGCA
ACACCTCCTACATGGGGTCAAATGAAGAGGTTGACGCAGGAAGCAGAGAAGACGTTAATGA
AGGCGGGGCAACCTCTGAATCCTACCAATCTTTTGCTTGCCACGATGGCGGTGGTGACATGT
CAGGTAATCGGTGTATCGGCAAGTAATCATACATATTGGGCACATATACCTAATCCCCCATT
AGTAAGAGCAGTTTTCTGGGGGGAACCAGAAGTGCAGGTATGTACTAATGAGATTGCCTTCT
TTCCCCCGCCAGCTTGCGGGGGAATAGAACAACTATCTCATCATAAACAACAATATAATATT
AGTAATTTGACCATTGCAGTGGAAGGTATTCCTTTATGTATGGGAGGACACCCCTTTTGTCTG
TCCACCAAGGAACATTCTCATCATTCTTATAATACATGGGGGGTAAAATATAATAGTTACCA
TTTTGCTGCTTTTACTGTGCTTGTTTCCACCAGGGGATTTAGCACCTCGACAGAACCGATAGA
CATTCATAATGGAAAACACATGTCACTATGTCCTGTTAACTTTTTTGTTCCTTCTCTAGAATC
TTTGGAGTGGGAACGTTGCCAAGGTCATCGACCCTTTAAAGTCATGAATTATTCTGGGGCCA
TCACTATAGATTGGAATCCAGATCATGGGCAATTCTGAGAAAAATGGTCAAATAAATCTCTT
AGGTGGCATCGTGCAAATAGCACTTTGATGGGCAATGGTAATGAAACAGTTAAATGGCAGC
AATTTGCACTTGTCCCTCCTCAATGACAATTGCAAGGATATCCGCACATTCAAGGAGATATT
TGGAAACTATGGGCGGTTTCTGGTAATCTCGCTATCTGGTCAGGAAACTATACTTTGGACAG
TGGTGACTCTTCCGGTCCATTCCATGTTAATTTACATGTTAATAAATCTTATTCTGCAATGGC
ATGTGTAAAATATCCTTTTGCATTGTTATATGGAAATTGGACCTAGAATGATACTGCGGGGT
CTGTGTCATGTGACTATTGTAATCTAACTCAATGTGTAAATCAGTCTTGGTGGGAAGAATTTG
AAAGATGAGCCTATAATTCCAATTTCTCGCTAGTAATTGTTAAGGCTCAGACAGAAGTATGG
TTACCTATAAATCTGACTCGGCCGTGGTCGGATTCTTTTGCTGTTTCTCACCTAATAACCGCT
GTACAGACTTTGCTATACCGATCTCAACGTATGCTTGGTGTGGTCAATGCTTCGATTCTAGCA

GTCGCGTCAGTAACTGCAACAGCAGCAGTGGCAGGTCTTGCATTACACCAAGGAATTCAAA
CAGCTGATTTTATTCAGGACTGGCATAAAGACTCATTTGTTATGGCAACAACAGCGAGATTT
GGATGCCCAACTTGCTACCGACACGCTCAATCTTCAACACACCGTTTCCTGGCTTGGAGATC
AACTGGCTGTTTTATCTACACAAAGTGTGTTGAAATGTGATTGGAATTCTTCTCAGTTTTGTA
TAACACCTGCACCATTTAACATGAGTGAAGGATGGGATAAAGTAAAACGACCCTTGACTGG
GCATCAAAATCTCACTACGGAGATTATGGACCTGGAACGACAAATTTTGTCTACTTTTAGCA
GGACTTTACCTGACATTATGGGGTCTGATTTGCTAAAATGTCTTCAAGAAGGAATGAATAAC
TTAAATCCATTAGGGCATGTATCCTCACTAATTGGGACTACTTTTGGGAACACTGTGTTTATA
TTACTTTTATGTCGTGTTGCTTTTCCAGTCTTCCGGCGATGGCGGAAAGGGAAACAACTAAA
GCACGAAGCAGAGAAGATCCAGACCATGTTACAATTTATAAAAGCAAATAAAAAAGGGGG
AGATGAAGGGTTAATAGGGTAGCAGAGATGTGCCTGCAAATGGGCCTCTCTGCTAGGGCTG
GACGTCCTTGCAAACGAGGCTTTCTGCCAAAGAGTCTGGACACAGCCTTGAGTTTAATGGTC
CCTTGCAAATGAGGGAGCATTCCCTTCTTATGATAAAGAAGGAATAGAGGGCTTTGTACAGA
CTCTGCAGTAGACTAGGATTTTACTCCCCTTTGCT

>NC_056066.1:34288887-34296816#SHEEP_RIP_21(-)

CTGCGGGGGACGACCCGTGAAGGGTTAAGTCTTGGGAGCTCCCTGGCAGGTATGCCAGGCC
CTAGGACACGTGCCTAAGCTCCCTGTCCCGCCACCCTCAAGAGTTTTTATAACCCTTAAGGCT
CCAAGATGTTTGGTTTCGGCAACATTTCATAGAAGATAGATTATCTTATTGTGTATATTTCAT
AGAAGATAGATATTCTGATTGTGTTCTGTATACAATGGTAAGGGTCTGGTGATTGTATCCTG
AGATTAAAAAACAACCTTGTGAGTGCCTTAAGTCACGTACTTTACCCTATATATACCGCAGC
ACAATAAAGCAAGGTATCAGCCATTTGGGGCTGATCCTCTCAACCCCATCTTTTGTCTATCTC
TTATTTTCTTAGCGGGGACGCTCCGTTCTCTCCCTGTGCAGGTGCGACTCTTGCTTGTGCTGG
CCGCGGCAGGTGGCGCCCAACGTGGGGCTCGAGCTCGACAGTTTTCCTCGCCACTACTCTTA
TTAATTGAAAAGAGTGAGTATATGAGTAAACAAGTGAATTAAATTGAGGAGGAGTAGTAAG
GTATATAGTTGAGAGTATAAATATGGGACAGACGCATAGTCGTCAGTTGTTTGTGCATATGT
TATCTGTAATGTTAAAACATAGGGGAATTACTGTTTCTAAACCTAAATTAATCAATTTTCTTT
CATTCATCGAGGAAGTTTGCCCTTGGTTCCCCAGAGAAGGTACAGTAAATTTAGAGACATGG
AAGAAGGTAGGGAACAAATTCGGACTCATTATACTTTACATGGCCCTGAAAAATCCCTGTCG
AAACTTTATCCTTTTGGACACTAATTCGTGACTGCCTGGACTTTGATAATGATGAATTAAAAC
GTTTAGGAAATTTATTAAAACAGGAAGAAGATCCTCTCCATGTTCCTGATTCGGAACCCAGA
TATGCTGTTCCCGAGGGGGTTAAAAGCGACCCTCCGTTTTCTAACTTATTGCATCCTTCAGAT
AATGATGATTTACTTTCATCCACAGATGAGGCAGAATTAGACGAAGAAGCTGCTAAATACCA
TCAAGAAGATTGGGGTTTTTAGCACAAGAAAAGGGGCGTTAACATCTAAAGATAAATTGGT
TGAATGCTTTAAAAACCTCACTATTGCTTTACAGAACGCAGGAATCAAGCTTCCTAGTAACA
ATGCCAAATCTCCTTCTGCTCCGCCTCTTCCCCCTGCTTATGCTCCTTCTGTTGTGGCTGGTCT
CGATCCCCTCCAGGGCCCCTCCACCGTCTGAGAACATGTCTCCGCTGCAAAAGGCATTGAGA
CAGGCACAGCGACTTGGTGAGGTTGTCTCTGATTTTTCTCTTGCTTTTCCTGTCTTTGAAAAT
AACAACCAGCGTTATTATGAATCACTGCCTTTTAAACAACTGAAAGAGTTAAAGATTGCTTG
CTCACAATACGGTCCTACCGCTCCATTCACCATTGCTATGATAGAAAATTTGGGTACTCAAG
CTTTACCTCCAAATGATTGGAAGCAGACAGCTAGGGCATGTCTCTCAGGGGGAGATTATTTA
TTATGGAAATCTGAATTTTTTGAACAATGTGCTCGTATAGCTGATGTTAACCGACAGCAAGG
TATACAGACCTCCTATGAAATGTTGATTGGTGAAGGCCCTTACCAGGCTACTGATACTCAAC
TTAATTTCTTACCTGGTGCATATGCACAAATATCAAATGCGGCTCGGCAGGCATGGAAAAAC
TTCCTAGCTCCAGTACTAAGACAGAGGATCTTTCAAAAGTCCGGCAGGGACCTGATGAGCCT
TACCAAGACTTCGTGACACGACTTTTAGATACTATAGGTAAGATAATGTCAGATGAAAAGGC
TGGGATGGTACTGGCAAAACAATTGGCTTTTAAAAACGCTAACTCTGCTTGTCAAGCTGCTT

TAAGACCTTATCGAAAAAAGGGAGATCTGTCTGATTTTATTCGCATTTGTGCTGACATTGGA
CCCTCCTACATGCAAGGCATTGCTATGGCAGCAGCATTACAAGGAAAAAGCATAAAAGAGG
TACTTTTCCAGCAGCAAGCCCGGAACAAGAAAGGACTTCAAAAGTCAGGTAATTTGGGTTGC
TTTGTTTGTGGTCAGCCTGGCCATCGGGCTGCAGTGTGCCCTCAAAAACAACAAAGCCCTGT
TAACACTCCTAATTTGTGCCCACGCTGTAAAAAAGGAAAGCATTGGGCGCGGGATTGCCGTT
CCAAAACGGATGTTCAAGGTAATCCTTGCCCCCCGGTTTCGGGAAACTGGGTGAGGGCCAGC
CCTGGCCCCGAAACAATGTTATGGGGCAACACTGCAGGTTCCAAAAGGACCATTGCAGACC
TCTGTCGAGCCACAAGAGGCAGCGCGGGATTGGACCTCTGTGCCACCTCCTACACAGTATTA
ACTCCCGAGATGGGGGTCCAAACCCTTGCCACAGGAGTGTTTGGGCCTTTACCTCCAGGGAC
AGCTGGACTGCTTTTAGGGCGCAGCAGTGCGTCTTTAAAAGGAATACTTATTCATCCTGGTG
TGATTGACTCTGATTATACAGGAGAGATAAAAATATTAGCCTCCGCTCCTAACAAAATTATT
GTAATCAATGCAGGACAGCGTATAGCTCAACTTCTTTTAGTTCCATTAGTCATACAAGGAAA
AACAATTAACCGAGACCGTCAAGATAAAGGTTTCGGGTCCTCTGACGCCTATTGGGTGCAAA
ATGTTACCGAGGCACGACCAGAACTTGAGCTACGCATTAATGGTAAGCTTTTCCGCGGAGTG
CTTGATACAGGGGCCGATATTAGTGTTATTTCTGATAAATATTGGCCTACTACATGGCCAAA
ACAGATGGCTATTTCCACTCTCCAGGGTATTGGCCAAACTACCAATCCAGAACAGAGTTCAT
CCCTTCTTACTTGAAAGGATAAAGATGGACATACAGGCCAATTTAAACCTTATATTCTGCCC
CATCTTCCAGTTAATCTATGGGGGCGTGATATATTAAGCAAAATGGGTGTTTATTTATATAGT
CCTTCACCCACTGTGACAGATTTGATGTTAGATCAGGGCTTACTTCCAAATCAAGGTTTAGGT
AAACAACATCAAGGCATCATTTTGCCCCTTGATTTAAAATCTAATCAAGATCGAAAAGGCTT
GGGGTGTTTTCCCTAGGGACCTCTGATTCTCCTGTGACGCATGCCGATCCTATTGATTGGAAA
TCTGAAAAACCGGTATGGGTCGATCAGTGGCCCCTAACACAGGAAAAACTTTCTGCCGCACA
ACAGCTGGTGCAAGAACAGCTGAGACTTGGGCATATTGAACCCTCTACCTCTGCTTGGAATT
CCCCAATTTTTGTTATTAAAAAGAAGTCTGGGAAATGGAGATTGCTACAAGATCTTCGTAAG
GTAAATGAAACAATGATGCATATGGGAGCCCTACAACCTGGGTTGCCCACTCCTTCTGCTAT
ACCTGATAAATCCTATATCATTGTTATAGATTTAAAAGATTGTTTTTACACTATTCCTCTTGC
ACCTCAAGATTGCAAAAGATTTGCTTTCAGTTTACCCTCTGTTAATTTTAAAGAGCCTATGCA
ACGCTATCAATGGAGAGTTCTCCCGCAAGGAATGACTAATAGCCCTACGCTGTGCCAAAAAT
TTGTTGCTACAGCAATAGCTCCGGTTCGTCAACGTTTTCCTCAGCTATATTTGGTTCATTATA
TGGATGATATATTACTAGCTCATGCTGACGAACATCTATTGTATCAAGCTTTTTCTATTCTAA
AACAACATTTAAGCCTTAATGGTCTTGTTATTGCTGATGAAAAATTCAGACTCATTTTCCTTA
TAATTATTTGGGTTTCTCCTTATATCCTCGTGTTTATAATACCCAATTAGTAAAACTGCAGAC
TGACCATTTAAAAACTCTAAATGACTTTCAAAAACTTTTAGGAGACATTAATTGGATACGTC
CTTATTTAAAATTACCCACTTATACCTTGCAGCCATTATTTGACATCCTTAAAGGTGACTCTG
ATCCTGCGTCACCCCGAACACTTTCTTTAGAAGGACGAACTGCTTTACAATCAATAGAAGAA
GCTATTAGACAACAACAGATTACTTATTGTGATTACCAACGATCATGGGGTTTGTATATACTT
CCTACCCCCCGAGCACCCACAGGGGTTCTCTATCAAGATAAACCTTTGCGATGGATATATTT
GTCTGCTACTCCAACTAAACATCTGCTCCCTTACTATGAACTTGTTGCAAAAATTATAGCAAA
GGGACGTCACGAGGCCATCCAATATTTTGGTATGGAACCCCCTTCATTTGTGTTCCTTATGCT
TTAGAACAACAAGATTGGCTTTTTCAATTTTCAGATAATTGGTCTATAGCTTTTGCAAATTAC
CCCGGACGGATTACTCATCATTACCCTTCTGATAAATTGTTACAATTTGCTAGCTCTCATGCC
TTTATTTTTCCAAAAATAGTTCGCCGACAACCTATTCCCGAAGCGACACTTATATTTACAGAT
GGATCTTCTAATGGAACTGCAGCTTTAATCATTAACCATCAAACCTATTACGCACAAACCAG
TTTTTCTTCTGCTCAAGTTGTGGAATTATTTGCAGTCCACCAAGCGTTGCTAACTGTACCTAC
TTCCTTCAATTTATTTACAGACAGCTCCTATGTGGTCGGTGCCTTACAGATGATTGAAACTGT
TCCAATTATCGGCACCACCTCTCCTGAAGTTCTTAACTTATTTACATTGATTCAACAGGTTCT
CCATTGCCGCCAACACCCCTGTTTCTTTGGACATATTCGTGCACACTCCACCCTTCCTGGTGC

CCTCGTACAAGGCAATCACACTGCGGACGTTCTTACTAAACAAGTGTTTTTTCAATCAGCTAT
TGATGCAGCCCGAAAGTCCCATGATTTACATCACCAAAATAGTCATTCTTTACGCTTGCAATT
TAAAATTTCCCGTGAAGCTGCACGGCAAATTGTTAAATCTTGCTCTACTTGTCCTCAATTCTT
TGTTCTTCCTCAATATGGTGTCAACCCTCGAGGTTTACGCCCTAATCACCTCTGGCAAACAGA
CGTTACTCACATTCCTCAATTTGGGCGTCTTAAATATGTTCATGTTTCTATTGACACTTTTTCC
AATTTTCTCATGGCCTCCCTTCACACTGGAAAATCAACACGTCACTGTATTCAACATTTGCTG
TTTTGCTTTTCTACTTCAGGAATCCCACAAACCCTTAAAACAGATAATAGACCTGGTTATACT
AGCCGTTCTTTTCAACGTTTTTGCCTTTCTTTCCAAATTCATCATAAAACAAGAATTCCTTATA
ATCCACAGGGACAAGATATTATAGAACGAGCCCATCAACGCCTTAAACATCAATTATTAAA
ACAAAAAAAGGGGAATGAACTGTATAGCCCCTCACCGCATAACGCCTTAAACCATGCTCTTT
ATGTTTTAAATTTTTTAACTTTAGACGCAGAAGGCAATTCAGCAGCCCAGCGTTTTTGGGGA
GAACGATCCTCATGCAAAAAACCACTTGTACGATGGAAGGATCCACTTACCAATCTGTGGTA
TGGGCCAGACCCTGTACTAATATGGGGACGAGGGCATGTTTGTGTTTTTCCACAGGATGCCG
AAGCGCCGCGCTGGATTCCGGAAAGGCTGGTACGCGCGGCAGAGGAACTCCCTGACACATC
AAATGCATCGCATGACACTGAGTGAGCCCACGAGTGAGCTGCCTACCCAGAGGCAAATTGA
GGCGCTGATGCGTTATGCTTGGAATGAGGCTCATGTACAACCTCCAGTGACACCTACTAAAA
TACTGATCATGTTATTATTATTGTTACAGCGGATACAAAACGGGGCAGCTTCGGCTTTTTGGG
CATACATTCCTGATCCGCCTATGATTCAATCCTTAGGATGGGATAAAGAAACAGTACCTGTA
TATGTTAATGATACAAGTCTTTTAGGAGGAAAATCAGATATTCACATTTCTCCTCAGCAAGC
CAATATCTCCTTTTATGGTCTTACTACTCAATACCCTATGTGCTTTTCTTATCAATCACAGCAT
CCTCATTGTATACAGGTGTCAGCTGATATATCCTATCCTCGAGTGACTATTTCAGGCATTGAT
GAAAAAACCGGAAAGAGATCGTACCGTGACGGAACCGGACCCCTCGACATTCCGTTTTGTG
ACAAAAATTTAAGCATCGGCATAGGAATAGACACTCCTTGGACTTTATGTCGAGCACGAATT
GCATCGGTGTATAACATCAACAATGCCAATACCACCCTTTTATGGGACTGGGCACCTGGAGG
AACACCTGATTTCCCCGAATATCGAGGACAGCATCCACCCATTCTCTCTGTAAACACTGCTC
CTATATTTCAAACTGAATTGTGGAAACTTTTGGCTGCTTTTGGTCATGGCAATAGTCTATATT
TACAGCCCAATATTAGTGGGAGCAAATATGGTGATGTGGGAGTTACAGGATTTTTATATCCC
CGAGCTTGTGTCCCTTATCCATTCATGTTGATACAAGGCCATATGGAAATAACACTGTCATTG
AATATTTATCATTTAAATTGTTCTAATTGCATACTTACTAATTGCATTAGAGGTGTAGCCAAA
GGAGAACAAGTTATAATAGTAAAACAACCTGCTTTTGTAATGTTACCTGTTGAAATAACTGA
AGAATGGTATGATGAAACTGCTTTAGAATTGTTACAACGCATTAATACGGCTCTTAGCCGTC
CTAAAAGAGGTCTGAGCCTGATTATTCTGGGTATAGTGTCTTTAATCACCCTTATAGCAACTG
CTGTTACTGCTTCTGTATCTTTAGCACAATCCATTCAAGCTGCTCATACTGTAGATTCCTTGTC
ATATAATGTTACTAAAGTAATGGGAACTCAAGAAGATATAGATAAAAAAATAGAAGATAGA
TTATCAGCTTTATATGATGTAGTTAGAGTTCTAGGAGAACAAGTTCAGAGCATTAATTTTCGC
ATGAAAATTCAATGCCATGCTAATTATAAATGGATTTGTGTTACAAAAAGCCTTACAATACT
TCTGACTTTCCGTGGGATAAGGTGAAAAAACATCTACAAGGAATTTGGTTTAATACTAATGT
TTCTTTAGATCTTTTACAATTGCACAATGAAATTCTTGACATCGAAAATTCTCCAAAAGCTAC
TTTGAATATAGCTGATACCGTCGATAATTTTTTACAAAATTTATTTTCTAACTTTCCTAGCCTT
CATTCACTGTGGCGAAGTATAATTGCTATGGGCGCGGTTCTGACTGTTGTGCTTATCATAATT
TGTTTAGCTCCTTGCCTTATTCGTAGCATTGTTAAAGAATTTCTACATATGAGAGTTTTAATA
CATAAAAACATGTTGCAACACCAACATCTTATGGAGCTTTTAAAAATAAAGAGAGGGGAGC
TGCGGGGGACGACCCGTGAAGGGTTAAGTCTTGGGAGCTCCCTGGCAGGTATGCCAGGCCC
TAGGACACGTGCCTAAGCTCCCTGTCCCGCCACCCTCAAGAGTTTTTATAACCCTTAAGGCTC
CAAGATGTTTGGTTTCGGCAACATTTCATAGAAGATAGATTATCTTATTGTGTATATTTCATA
GAAGATAGATATTCTGATTGTGTTCTGTATACAATGGTAAGGGTCTGGTGATTGTATCCTGA
GATTAAAAAACAACCTTGTGAGTGCCTTAAGTCACGTACTTTACCCTATATATACCGCAGCA

CAATAAAGCAAGGTATCAGCCATTTGGGGCTGATCCTCTCAACCCCATCTTTTGTCTATCTCT
TATTTTCTTAGCGGGGACGCTCCGTTCTCTCCCTGTGCAGGTGCGACTCTTGCTTGTGCTGGC
CGCGGCA

>NC_056067.1:15049560-15057133#SHEEP_RIP_22(+)

TGCGGGGGACGACCCGTGAAGGGTTAAGTCTTGGGAGCTGCTCAGCAGGTATGCAGAGCCC
TAGGACATGTTCCTAAGCTCCCTGTCCCGCCACCCTCAAGAATTTTTATAGCCCTTAAGGCTC
CAAGATGTTTGGTTTCGGCAACATTTCATAGAAGATAGATTATCTTATTGTGTATACTTCATA
GAAGATAGATTATCTGATTGTGTTCTGTATACAATGGTAAGGGTCTAGTGATTGTATCTTGA
GATTAAAAACAACCTTGTGAATGTCATAAGTCACGTACTTTACCCTATATATACTGCAGCAC
AATAAAGCAAGGTATCAGCCATTTTGGGCTGATCCTCTCAACCCCATCTTTTGTCTCTCTCTT
ATTTTTCTTAGCGGGGACGCTCCGTTCTCTCCCTGTGCAGGTGCGACTCTTGCTTGTGCTGGC
CACGGCAGGTGGCGCCCAACGTGGGGCTCGACTTCGACAGTTTTCCTCGCCACTACTCTTAT
TAATTGAAAAGAGTGAGTATATGAGTATACAAGTGAATTAAATTGAGGAGGAGTAGTAAGG
TATATAGTTGAGAGTATAAATATGGGACAGATGCATAGTCGTCAATTGTTTGTACATATGTT
ATCTGTAATGTTAAAACATCGGGGAATTACTGTTTCCAAACCTAAATTAATCAATTTTCTTTC
ATTTATTGAGGAAGTTTGCCCTTGGTTCCCCAGAGAAGGTACAGTAAATTTAGAAACATGGA
AGAAGGTAGGGGAACAAATTCGGACTCATTATACTTTACATGGCCCTGAAAAAATCCCTGTC
GAAACTTTATCCTTTTGGACACTAATTCGTGATTGCCTGGACTTTGATAATGATGAATTAAAA
CGTTTAGGAAATTTATTAAAACAGGAAGAAGATCCTCTCCATGTTCCTGATTCGGAACCCAG
GTATGCTGTTCCCGAGGGAGTTGAAGGCGACCCTCCGTTTTCTAACTTATTGCGTCCTTCGGA
TAATGATGATTTACTTTCATCCACAGATGAGGCGGAATTAGATGAAGAAGCTGCTAAATACC
ATCAAGAAGATTGGGGTTTTTTAGCACAAGAAAAGGGGGCGTCAACATCTAAAGATGAATT
GGTTGAATGTTTAAAAAACCTCACTATTGCTTTACAGAACTCAGGAATCAAGCTTCCTAGTA
ACAATGCTAAATCTCCTTCTGCTCCGCCTCTTCCCCCTGCCTATGCTCCTTCCGTTGTGGCTGG
TCTCGATCCCCCTCCAGGGCCTCCTCCACCGTCTGAGATCATGTCTCCGCTGCAGAGGGCATT
GAGACAGGCACAGCGACTTGGTGAGGTTGTCTCTGATTTTTCTCTTGCTTTCCCTGTCTTTGA
AAATAACAACCAGCGTTTTTATGAATCACTGCCTTTTAAACAACTGAAAGAGTTAAAGATTG
CTTGCTCGCAATACGGTCCTACCGCTCCATTCACTATTGCTATGATAGAAAGTTTGGGTACTC
AAAATCTACCCCCAAATGATTGGAAACAAATAGCTAGGGCCTGTCTTTCGGGGGGAGATTAT
TTACTATGGAAATCTGAATATTTTGAACAGTGTGCTCGTATAGCCAATGTTAATCGACAGCA
AGGTATACAGACCTCCTATGAAATGTTGATTGGTGAAGGCCCTTACCAGGCTACCGATACTC
AACTTAACTTCTTACCTGGTGCGTACGCACAAATATCAAATGCGGCTCGGCAGGCATGGAAA
AAACTTCCTAGCTCCAGTACTAAGACAGAAGACCTTTCAAAAGTCCGACAGGGACCTGATG
AGCCTTATCAAGACTTCGTGGCACGGCTCTTAGATACTATAGGTAAGATAATGTCAGATGAA
AAGGCTGGGATGGTATTAGCAAAACAATTGGCTTTTGAAAACGCTAACTCTGCCTGTCAAGC
TGCTTTAAGACCTTATCGAAAAAAGGGAGATCTGTCTGATTTTATTCGTATTTGTGCTGACAT
TGGACCCTCCTATATGCAAGGCATTGCTATGGCAGCAGCATTACAAGGAAAAGGCATTAAA
GAGGTACTTTTTCAGCAGCAAGCCAGGAACAAGAAAGGACTTCAAAAGTCAGGTAATTCGG
GTTGCTTTGTTTGTGGTCAACCTGGCCATCGGGCAGCAGTGTGCCCCCAAAAGCAACAAACC
TCTGTTAACACTCCTAATTTATGCCCACGATGTAAAAAAGGGAAGCATTGGGCCCAAGATTG
TCGTTCTAAAACGGATGTTCAAGGTAATCCTTTGCCCCCGGTTTCGGGAAACTGGGTGAGGG
GCCAGCCCCTGGCCCCAAAACAATGTTATGGGGCAACACTGCAGGTTCCAAAAGAACCACT
GCAGACCTCTGTCGAGCCACAAGAGGCAGCGCGGGATTGGACCTCTGTGCCACCTCCTACAC
AGTATTAACACCCGAGATGGGGGTTCAAACCCTTGCCACAGGAGTGTTTGGGCCTTTACCTC
CAGGTACAGCTGGACTGCTTTTGGGGCGCAGCAGCGCGTCTTTAAAGGGAATACTTATCCAT
CCTGGTGTGATTGACTCTGATTATACAGGAGAGATAAAAATATTAGCCTCCGCTCCTAACAA

AATTATTGTAATCAATGCAGGACAACATATAGCTCAACTCCTTTTAGTTCCATTAGTCATACA
AGGAAAAACAATTAACCGAGACCGTCAAGATAAAGGTTTCGGGTCCTCTGACGCCTATTGG
GTGCAAAATGTTACCGAGGCACGACCAGAACTTGAGCTACGCATTAATGGTAAGCTTTTCCG
CGGAGTGCTTGATACAGGGGCCGATATTAGCGTTATTTCTGAAAAATACTGGCCTACTACAT
GGCCTAAACAAACAGCTATTTCCACTCTTCAGGGTATTGGCCAAACTACCAATCCAGAACAA
AGTTCGTCCCTTCTTACTTGGAGGGATAAAGATGGCCATACAGGCCAATTTAAACCTTATAT
TCTGCCCCATCTTCCAGTTAATCTATGGGGGCGTGATATATTAAGCAAAATGGGTGTTTATTT
ATATAGTCCTTCACCCACCGTAACAGATTTGATGTTAGATCAGGGCTTACTTCCAAACCAAG
GTTTAGGTAAACAACATCAAGGCATCGTTTTACCCCTTGATTTAAAATCTAATCAAGATCAA
AAAGGCTTGGGGTGTTTTTCCTAGGGACCTCTGATTCTCCTGTGACACATACCGATCCTATTG
ATTGGAAATCTGAGGAACCGGTATGGGTCGATCAGTGGCTCCTGACACAAGAAAAACTTTCT
GCCGCACAACAGCTGGTGCAGGAACAGCTGAGGCTTGGGCATATTGAACCCTCTACCTCTGC
GTGGAATTCCCCAATTTTTGTTATTAAAAAGAAGTCTGGAAAATGGAGATTGCTACAAGACC
TTCGTAAGGTAAATGAAACAATGATGCATATGGGAGCCCTACAACCTGGGTCGCCCACTCCT
TCCGCTATACCTGATAAATCCTATATTATCATTATAGATTTAAAAGATTGTTTTTACACTATT
CCTCTTGCACCTCAAGATTGTAAAAGATTTGCCTTTAGTTTGCCCTCTGTTAATTTTAAAGAG
CCTATGCAACGCTATCAGTGGAGAGTCCTCCCACAAGGAATGACTAATAGTCCTACGTTATG
TCAAAAATTTGTTGCTACAGCATTAGCTCCCGTTCGTCAGCGTTTTCCTCAGTTATATTTAGT
TCATTATATGGATGATGTATTACTAGCTCATGCTGACGAACATCTATTGTATCAAGCTTTTTC
TATTCTAAAAAATCATTTAAGCCTTAATGGTCTTGTCATTGCTGATGAAAAAATTCAAACTCA
CTTTCCCTATAATTATTTGGGTTTCTCCTTATACCCTCGTGTTTATAACACCCAATTGGTACAA
TTACAGACTGACCATTTAAAAACTCTAAATGACTTTCAAAAACTTCTAGGAGACATTAATTG
GATACGCCCTTATTTAAAACTACCCACTTATACCTTGCAGCCATTATCTGACATCCTTAAAGG
TGACCCTGACCCTGCGTCACCCCGAACACTTTCTCTAGAAGGACGATCAGCCTTACAATCAA
TAGAAGAAGCTATTAGACAACAACAGATTACTTATTGTGATTACCAACGATCATGGGGTTTG
TATATACTTCCTACCCCTCGAGCACCCACAGGGGTTCTTTATCAAGATAAACCTTTGCGATGG
ATATATCTATCTGCTACTCCAACTAAACATCTGCTCCCTTACTATGAGCTTGTTGCAAAAATT
GTAGCAAAAGGACGTCATGAGGCCATCCAATATTTTGGTATGGAACCCCCTTTCATTTGTGT
TCCTTATGCTTTAGAACAACAAGATTGGCTTTTTCAATTTTCAGATAATTGGTCCATAGCTTT
CGCAAATTACCTGGGACGGATTACTCATCATTATCCTTCTGATAAATTGTTACAATTTGCTAG
CTCTCATGCCTTTATTTTTCCAAAAATAGTTCGCTGACAACCCATTCCCGAAGCGACACTTAT
ATTTACAGATGGATCTTCTAATGGAACTGCAGCTTTAATTATTAACCATCAAACCTATTACGC
ACATACCAGTTTTTCTTCTGCTCAGGTTGTTGAATTATTTGCAGTCCATCAAGCATTGCTAAC
TGTACCCACTTCCTTCAATTTATTTACAGACAGCTCCTATGTGGTCGGTGCCTTACAGATGCT
TGAAACTGTTCCAATTATCGGCACAACCTCTCCTGAAGTTCTTAACTTATTTACATTGATTCA
ACAGGTTCTTCACCGTCGCCAACACCCGTGTTTCTTTGGGCATATTCGTGCACATTCCACCCT
TCCTGGTGCCCTCGTACAAGGCAATCACACTGCGGACGTTCTTACTAATCGAGTGTTTTTTCA
ATCAGTTATCGATGCAGCCCGAAAATCTCATAACTTACATCACCAAAATAGTCATTCTTTAC
GGTTACAATTTAAGATTTCCCATGAAGCTGCACGGCAAATTGTTAAATCTTGCTCTACTTGTC
CTCAATTCTTTGTTCTTCCTCAATATGGTGTCAACCCTCGAGGTTTACGCCCTAATCATCTCTG
GCAAACAGATGTCACTCACATTCCCCAATTTGGGCGTCTTAAATATGTTCATGTCTCTATCGA
CACCTTTTCCCATTTTCTCATGGCCTCCCTTCACACCGGAGAATCAACTCGTCACTGTATTCA
ACATTTGCTGTTTTGCTTTTCTATTTCAAGAATCCCACACACCCTTAAAACAGATAATGGACC
TGGTTATACTAGCCGTTCTTTTCAACGTTTTTGTCTTTCTTTTCAAATTCATCATAAAACAGGA
ATTCCTTATAATCCACAAGGACAAGGTATTGTGGAACGAGCCCATCAACGCCTTAAACATCA
ATTATTAAAACAAAAAAAGGGGAATGAACTGTATAGCCCCTCACCGCGTAACGCCTTAAAT
CATGCTCTCTATGTTTTAAATTTTTTAACTTTAGATGCAGAAGGCAATTCAGCAGCCCAGCGT

TTTTGGGGAGAATGATCCTCATGCAAAAAACCACTTGTACGATGGAAGGATCCACTTACCAA
TCTGTGGTATGGGCCAGACCCTGTATTAATATGGGGACGGGGGCATGTTTGTGTTTTTCCAC
AGGATGCCGAAGCGCCGCGCTGGATTCCGGAAAGGCTGGTACGCGCGGCGGAGGAACTCCC
TGACATATCAAATGCATCGCATGACACTGAGCGAGCCCACGAGTGAGCTGCCTACCCAGAG
GCAGATTGAGGCGTTGATGCGACATGCTTGGAATGAGGCTCATGTACAACCTCCGGTGACGC
CTACAAATATACTGATCATGTTATTATTATTGTTACAGCGGATACAAAATGGGGCGGCTGCG
GCTTTTTGGGCATACATTCCCGATCCGCATATGATTCAAAGAAATAGTACCTGTATATGTCA
ATGATACAAGTCTTTTAGGAGGAAAATCAGATATTCACATTTCTCCTCAGCAAGCCAATATC
TCCTTTTATGGTCTTACTACGCAATATCCTATGTGCTTTTCTTATCAATCACAGCATCCTCACT
GTATACAGGTGTCAGCTGATACATCCTACCCTCGAGTGACTATTTCTGGCATTGATGAAAAA
ACCGGAAAAAGATCTTACCGTGACGGAGCCGGACCCCTCGACATTCCGTTTTGCGACAAACA
TTTAAGCATCGGCATAGGAATAGATACTCCTTGGACTTTATGTCGAGCACGGGTCGCATCGG
TGTACAACATCAACAATGCCAATGCCACCCTTTTATGGGACTGGGCACCTGGGGGAACACCT
GATTTCCCCGAATATCGAGGACAGCATCCACCCATTCTCTCTGTAAACACTGCTCCTATATAT
CAGACAGAACTGTGGAAACTTTTGGCTGCTTTTGGTCATGGTAATAGCCTATATTTACAACC
CAATATTAGTGGGAGTAAATATAGTAATGTAGGAGTTACGGGGTTTTTATATCCCCGAGCTT
GTGTCCCTTACCCATTCATGTTGATACAAGGCCATGTGGAAATAACGCTGTCATTGAATATTT
ATCATTTAAATTGTTCTAATTACATACTTACTAATTGCATTAGAGGTGTTGCCAAAGGAGAA
CAAGTTATAATAGTAAAACATTCGCATGAAAATTCAATGCCATGCTAATTATAAATGGATTT
GTGTTACAAAAAAGCCTTACAATACATCTGATTTTCCGTGGGATAAGGTGAAAAAACATCTA
CAAGGAATTTGGTTTAATACTAATGTTTCTCTAGATCTTTTACAATTGCATAATGAAATTCTT
AACATTGAAAATTCTCCAAAAGCTACTTTGAATATAGCTGATACTGTCGATAATTTTTTACAA
AATTTATTTTCTAACTTTCCTAGCCTTCATTCACTGTGGCGAAGTATAATTGCTGTGGGCGCG
GTTCTGACTGTTGTGCTTATCATAATTTGTCTAGCTCCTTGTCTTATTCGCAGTATTGTTAAGG
AATTTCTACATATGAGAGTTTTAATACATAAAAACATGTTGCAACACCAACATCTTATGGAG
CTTTTAAAAAATAAAGAGAGGGGAGCTGCGGGGGACGACCCGTGAAGGGTTAAGTCTTGGG
AGCTGCTCAGCAGGTATGCAGAGCCCTAGGACATGTTCCTAAGCTCCCTGTCCCGCCACCCT
CAAGAATTTTTATAGCCCTTAAGGCTCCAAGATGTTTGGTTTCGGCAACATTTCATAGAAGA
TAGATTATCTTATTATGTATACTTCATAGAAGATAGATTATCTGATTGTGTTCTGTATACAAT
GGTAAGGGTCTAGTGATTGTATCTTGAGATTAAAAACAACCTTGTGAATGTCATAAGTCACG
TACTTTACCCTATATATACTGCAGCACAATAAAGCAAGGTATCAGCCATTTTGGTCTGATCCT
CTCAACCCCATCTTTTGTCTCTCTCTTATTTTTCTTAGCGGGGACGCTCCGTTCTCTCCCTGTG
CAGGTGCGACTCTTGCTTGTGCTGGC

>NC_056067.1:57031180-57039117#SHEEP_RIP_23(-)

TGCGGGGGACGACCCGTGAAGGGTTAAGTCTTGGGAGCTCCCTGGCAGGTATGCCGGGCCC
TAGGACATGTGCCTAAGCTCCCTGTCCCGCCACCCTCAAGAATTTTTGTAACCCTTAAGGCTC
CAAGATGTTTGGTTTCGGCAACATTTCATAGAAGATAGATTATCTTATTGTGTATACTTCATA
GAAGATAGATATTCTGATTGTGTTCTATATACAATGGTAAGGGTCTGGTGATTGTATCCTGA
GATTAAAAAACAACCTTGTGAGTGCCTTAAGTTACGTACTTTACCCTATATATACCGCAGCA
CAATAAAGCAAGGTATCAGCTATTTTGGTCTGATCCTCTCAACCCCATCTTTTGTCTCTCTCT
TATTTTCTTAGCGGGGACGCTCCGTTCTCTCCCTGTGCAGGTGCGACTCTTGCTTGTGCTGGC
CGCGGCAGGTGGCGCCCAACGTGGGGCTCGAGCTCGACAGTTCTCCTCGCCACTACTCTTAT
TAATTGAAAAGAGTGAGTATATGAGTAAACAAGTGAATTAATTTGAGGAGGAGTAGTAAGG
TATATAGTTGAGAGTATAAATATGGGACAGACGCATAGTCGTCAGTTGTTTGTGCATATGTT
ATCTGTAATGTTAAAACATAGGGAATTACTGTTTCTAAACCTAAATTAATCAATTTTCTTTCA
TTCATCGAGGAAGTTTGCCCTTGGTTCCCCAGAGAAGGTACAGTAAATTTGGAGACATGGAA

GAAGGTAGGGGAACAAATTCGGACTCATTATACTTTACATGGCCCTGAAAAAATCCCTGTCG
AAACTTTATCCTTTTGGACACTAATTCGTGACTGCCTGGACTTTGATAATGATGAATTAAAAC
GTTTAGGAAATTTATTAAAACAGGAAGAAGATCCTCTCCATGTTCCTGATTCGGAACCCAGG
TATGCTGTTCCCGAGGGGGTTAAAAGCGACCCTCCGTTTTCTAACTTATTGCGTCCTTCGGAT
AATGATGATTTACTTTCATCCACAGATGAGGCAGAATTAGACGAAGAAGCTGCTAAATACCA
TCAAGAAGATTGGGGTTTTTTAGCACAAGAAAAGGGGCGTTAACATCTAGAGATGAATTGG
TTGAATGTTTTAAAAACCTCACTATTGCTTTACAGAACGCAGGAATCAAGCTTCCTAGTAAC
AATGCCAAATCTCCTTCTGCTCCGCCTCTTCCCCCTGCTTATGCTCCTTCTGTTGTGGCTGGTC
TCGATCCCCCTCCAGGGCCCCCTCCACCGTCTGAGAACATGTCTCCGCTGCAAAAGCATTGA
GACAGGCACAGCGACTTGGTGAGGTTGTCTCTGATTTTTCTCTTGCTTTTCCTGTCTTTGAAA
ATAACAACCAGCGTTATTATGAATCACTGCCTTTTAAACAACTGAAAGAGTTAAAGATTGCT
TGCTCACAATACGGTCCTACCGCTCCATTCACCATTGCTATGATAGAAAATTTGGGTACTCA
AGCTTTACCTCCAAATGATTGGAAGCAGACAGCTAGGGCATGTCTCTCAGGGGGAGATTATT
TATTATGGAAATCTGAATTTTTTGAACAATGTGCTCGTATAGCTGATGTTAACCGACAGCAA
GGTATACAGACCTCCTATGAAATGTTGATTGGTGAAGGCCCTTATCAGGCTACTGATACTCA
ACTTAATTTCTTACCTGGTGCATATGCACAAATATCAAATGCGGCTCGGCAGGCATGGAAAA
AACTTCCTAGCTCCAGTACTAAGACAGAGGATCTTTCAAAAGTCCGGCAGGGACCTGATGAG
CCTTACCAGGACTTCGTGGCACGACTCTTAGATACTATAGGTAAGATAATGTCAGATGAACA
GGCTGGGATGTTATTGGCAAAACAATTGGCTTTTGAAAACGCTAACTCTGCTTGTCAAGCTG
CTTTAAGACCTTATCGAAAAAAAGGAGATCTGTCTGATTTTATTCGCATTTGTGCTGACATCG
GACCCTCCTACATGCAAGGCATTGCTATGGCAGCAGCATTACAAGGAAAAAGCATAAAGGA
GGTACTTTTCCAGCAGCAAGCCCGGAACAAGAAAGGACTTCAAAAGTCAGGTAATTTGGGT
TGCTTTGTTTGTGGTCAGCCTGGCCATCGGGCTGCAGTGTGCCCTCAAAAACAACAAAGCCC
TGTTAACACTCCTAATTTGTGCCCACGCTGTAAAAAAGGAAAGCATTGGGCACGGGATTGCC
GTTCCAAAACGGATGTTCAAGGTAATCCTTTGCCCCCGGTTTCGGGAAACTGGGTGAGGGCC
AGCCCTGGCCCCGAAACAATGTTATGGGGCAACACTGCAGGTTCCAAAAGGACCATTGCAG
ACCTCTGTCGAGCCACAAGAGGCAGCGTGGGATTGGACCTCTGTGCCACCTCCTACACAGTA
TTAACTCCCGAGATGGGGGTCCAAACCCTTGCCACAGGAGTGTTTGGGCCTTTACCTCCAGG
GACAGCTGGACTGCTTTTAGGGCGCAGCAGTGCGTCTTTAAAAGGAATACTTATTCATCCTG
GTGTGATTGACTCTGATTATACAGGAGAGATAAAAATATTAGCCTCCGCTCCTAACAAAATT
ATTGTGATCAATGCAGGACAGCGTATAGCTCAACTTCTTTTAGTTCCATTAGTCATACAAGG
AAAAACAATTAACCGAGACCGTCAAGATAAAGGTTTCGGGTCCTCTGACGCCTTTTGGGTGC
AAAATGTTACCGAGGCACGACCAGAACTTGAGCTACGCATTAATGGTAAGCTTTTCCGCGGA
GTGCTTGATACAGGGGCCGATATTAGTGTTATTTCTGATAAATATTGGCCTACTACATGGCC
AAAACAGATGGCTATTTCCACTCTCCAGGGTATTGGCCAAACTACCAATCCAGAACAAAGTT
CGTCCCTTCTTACTTGGACGGATAAGGACGGTCATACAGGCCAATTTAAACCTTATATTCTGC
CCTATCTTCCAGTTAATCTATGGGGGCGTGATATATTGAGCACAATGGGTGTTTATTTATATA
GTCCTTCACCCACTGTGACAGATTTGATATTAGATCAGGGCTTACTTCCAAATCAAGGTTTAG
GTAAACAACATCAAGGCATCATTTTGCCCCTTGATTTAAAACCTAATCAAAACCGAAAAGGC
TTGGGGTGTTTTCCCTAGGGACCTCTGATTCTCCCGTGACACATGCCGATCCTATTGATTGGA
AATCTGAGGAACCGGTATGGGTCGATCAGTGGCCCCTAACACAGGAAAAACTTTCTGCCGC
ACAACAGCTGGTGCAGGAACAGCTGAGACTTGGGCATATTGAACCCTCTACCTCTGCTTGGA
ATTCCCCAATTTTTGTTATTAAAAAGAAGTCTGGGAAATGGAGATTGCTACAAGATCTTCGT
AAGGTAAATGAAACAATGATGCATATGGGAGCCCTACAACCTGGGTTGCCCACTCCTTCTGC
TATACCTGATAAATCCTATATCATTGTCATAGATTTAAAAGATTGTTTTTACACTATTCCTCTT
GCACCTCAAGATTGCAAAAGATTTGCTTTCAGTTTACCCTCTGTTAATTTTAAAGAGCCTATG
CAACGCTATCAATGGAGAGTTCTCCCGCAAGGAATGACTAATAGCCCTACGCTGTGCCAAAA

ATTTGTTGCTACAGCAATAGCTCCCGTTCGTCAACGTTTTCCTCAGCTATATTTGGTTCATTAT
ATGGATGATATATTACTAGCTCATGCTGACGAACATCTATTGTATCAAGCTTTTCGATTTTAA
AACAACATTTAAGCCTTAATGGTCTTGTTATTGCTGATGAAAAATTCAGACTCATTTTCCTTA
TAATTATTTGGGTTTCTCCTTATATCCTCGTGTTTATAATACCCAATTAGTAAAACTGCAGAC
TGACCATTTGAAAACTCTAAATGACTTTCAAAAACTTTTAGGAGACATTAATTGGATACGTC
CTTATTTAAAATTACCCACTTATACCTTGCAGCCATTATTTGACATCCTTAAAGGTGACTCTG
ATCCTGCGTCACCCCGAACACTTTCTTTAGAAGGACGAACTGCTTTACAATCAATAGAAGAA
GCTATTAGACAACAACAGATTACTTATTGTGATTACCAACGATCATGGGGTTTGTATATACTT
CCTACCCCCCGAGCACCCACAGGGGTTCTCTATCAAGATAAACCTTTGCGATGGATATATTT
GTCTGCTACTCCAACTAAACATCTGCTCCCTTACTATGAACTTGTTGCAAAATTGTAGCAAAG
GGAGGTCACGAGGCCATCCAATATTTTGGTATGGAACCCCCTTCATTTGTGTTCCTTATGCTT
TAGAACAACAAGATTGGCTTTTTCAATTTTCAGATAATTGGTCTATAGCTTTTGCAAATTACC
CGGGACGGATTACTCATCATTACCCTTCTGATAAATTGTTACAATTTGCTAGCTCTCATGCCT
TTATTTTTCCAAAAATAGTTCGCCGACAACCTATTCCCGAAGCAACACTTATATTTACAGATG
GATCTTCTAATGGAACTGCAGCTTTAATCATTAACCATCAAACCTATTACGCACAAACCAGT
TTTTCTTCTGCTCAAGTTGTGGAATTATTTGCAGTCCACCAAGCGTTGCTAACTGTACCTACT
TCCTTCAATTTATTTACAGACAGCTCCTATGTGGTCGGTGCCTTACAGATGATTGAAACTGTT
CCAATTATCGGCACCACCTCTCCTGAAGTTCTTAACTTATTTACATTGATTCAACAGGTTCGC
CATTGCCGCCAACACCCCTGTTTCTTTGGACATATTCGTGCACACTCCACCCTTCCTGGTGCC
CTCGTACAAGGCAATCACACTGCGGACGTTCTTACTAAACAAGTGTTTTTCCAATCAGCTATT
GATGCAGCCCGAAAATCCCATGACTTACATCACCAAAATAGTCATTCTTTACGCTTGCAATT
TAAAATTTCCCGTGAAGCTGCACGGCAAATTGTTAAATCTTGCTCTACTTGTCCTCAATTCTT
TGTTCTCCCTCAATATGGTGTCAACCCTCGAGGTTTACGCCGTAATCACCTCTGGCAAACAG
ATGTTACTCACATTCCTCAATTTGGGCGCCTTAAATATGTTCATGTTTCTATTGACACTTTTTC
CAATTTTCTCATGGCTTCCCTTCACACTGGAGAATCAACACGTCACTGTATTCAACATTTGCT
GTTTTGCTTTTCTACTTCAGGAATCCCACAAACCCTTAAAACAGATAATGGACCTGGTTATAC
TAGCCGTTCTTTTCAACGTTTTTGTCTTTCTTTCCAAATTCATCATAAAACAGGAATTCCTTAT
AATCCACAGGGACAAGGTATTGTGGAACGAGCCCATCAATGCCTTAAACATCAATTATTAAA
ACAAAAAAAGGGAAATGAACTGTATAGCCCCTCACCGCATAACGCCTTAAACCATGCTCTTT
ATGTTTTAAATTTTTTAACTTTAGACGCAGAAGGCAATTCAGCAGCCCAGCGTTTTGGGGGA
GAACGATCCTCATGCAAAAAACCACTTGTACGATGGAAGGATCCACTTACCAATCTGTGGTA
TGGGCCAGACCCTGTACTAATATGGGGATGAGGGCATGTTTGTGTTTTTCCACAGGATGCCG
AAGCGCCGCGCTGGATTCCGGAAAGGCTGGTACGCGCGGCAGAGGAACTCCCTGACACATC
AAATGCAACGCATGACACTGAGCGAGCCCACGAGTGAGCTGCCTACCCAGAGGCAAATTGA
GGCGCTGATGCGTTATGCTTGGAATGAGGCTCATGTACAACCTCCAGTGACACCTACTAATA
TACTGATCATGTTATTATTATTGTTACAGCGGATACAAAACGGGGCGGCTGCGGCTTTTTGG
GCATACATTCCTGATCCGCCTATGATTCAATCCTTAGGATGGGATAAAGAAACAGTACCTGT
ATATGTTAATGACACAAGTCTTTTAGGAGGAAAATCAGATATTCACATTTCTCCTCAGCAAG
CCAATATCTCCTTTTATGGTCTTACTACTCAATACCCTATGTGCTTTTCTTATCAATCACAGCG
TCCTCATTGTATACAGGTGTCAGCTGATATATCCTATCCTCGAGTGACTATTTCAGGCATTGA
TGAAAAAACCGGAAAAAGATCGTACCGTGACGGAACCGGACCTCTCGACATTCCGTTTTGTG
ACAAACATTTAAGCATCGGCATAGGAATAGACACTCCTTGGACTTTATGTCGAGCACAAATT
GCATCGGTGTATAACATCAACAATGCCAATACCACCCTTTTATGGGACTGGGCACCTGGAGG
AACACCTGATTTCCCCAAATATCGAGGACAGCATCCACCCATTTTTTCTGTAAACACTGCTCC
TATATATCAGAAAGAACTATGGAAACTTTTGGCTGCTTTTGGTCATGGCAATAGTCTATATTT
ACAGCCCAATATTAGTGGGAGCAAATATGGTAATGTAGGAGCTACGGGGTTTCTATATCCCT
GAGCTTGTGTTCCTTACCCATTCATGTTGATACAAGGCCATATGGAAATAACACTGTCATTG

AATATTTATCATTTAAATTGTTCTAATTGCATACTTACTAATTGCATTAGAGGTGTAGCCAAA
GGAGAACAAGTTATAATAGTAAAACAACCTGCTTTTGTAATGTCACCTGTTGAAATAACTGA
AGAATGGTATGATGAGACTGCTTTAGAATTGTTACAACGCATTAATACGGCTCTTAGCCGTC
CTAAAAGAGGTCTGAGCCTGATTATTCTGGGTATAGTATCTTTAATCACCCTTATAGCAACTG
CTGTTACTGCTTCTGTATCTTTAGCACAATCCATTCAAGCTGCTCATACTGTAGATTCCTTGTC
ATATAATGTTACTAAAGTAATGGGAACTCAAGAAGATATAGATAAAAAAATAGAAGATAGA
TTATCAGCTTTATATGATGTAGTTAGAGTTCTAGGAGAACAAGTTCAGAGCATTAATTTTCGC
ATGAAAATTCAATGCCATGCTAATTATAAATGGATTTGTGTTACAAAAAAGCCTTACAATAC
TTCTGACTTTCCGTGGGATAAGGTGAAAAAACATCTGCAAGGAATTTGGTTTAATACTAATG
TTTCTCTAGATCTTTTACAATTGCACAATGAAATTCTTGACATCGAAAATTCTCCAAAAGCTA
CTTTGAATATAGCTGATACCGTCGATAATTTTTTACAAAATTTGTTTTCTAACTTTCCTAGCCT
TCATTCACTGTGGCGAAGTATAATTGCTATGGGCGCGGTTCTGACAGTTGTGCTTATCATAAT
TTGTTTAGCTCCTTGCCTTATTCGTAGCATTGTTAAAGAATTTCTACATATGAGAGTTTTAAT
ACATAAAAACATGTTGCAACACCAACATCTTATGGAGCTTTTAAAAAATAAAGAGAGGGGA
GCTGCGGGGGACGACCCGTGAAGGGTTAAGTCTTGGGAGCTCCCTGGCAGGTATGCCGGGC
CCTAGGACACGTGCCTAAGCTCCCTGTCCCGCCACCCTCAAGAGTTTTTGTAACCCTTAAGG
CTCCAAGATGTTTGGTTTCGGCAACATTTCATAGAAGATAGATTATCTTATTGTGTATACTTC
ATAGAAGATAGATATTCTGATTGTGTTCTATATACAATGGTAAGGGTCTGGTGATTGTATCCT
GAGATTAAAAAACAACCTTGTGAGTGCCTTAAGTTACGTACTTTACCCTATATATACCGCAG
CACAATAAAGCAAGGTATCAGCCATTTTGGTCTGATCCTCTCAACCCCATCTTTTGTCTATCT
CTTATTTTCTTAGCGGGGACGCTCCGTTCTCTCCCTGTGCAGGTGCGACTCTTGCTTGTGCTG
GCCGCGGCAGGTGG

>NC_056067.1:57279356-57287291#SHEEP_RIP_24(-)

TGCGGGGGACGACCCGTGAAGGGTTAAGTCCTGGGAGCTGCTCAGCAGGTATGCAGAGCCT
AGGACATGTGCCTAAGCTCCCTGTCCCGCCACCCTCAAGAATTTTTATAGCCCTTAAGGCTCC
AAGATGTTTGGTTTTGGCAACATTTCATAGAAGATAGATTATCTTATTGTGTATACTTCATAG
AAGATAGATTATCTGATTGTGTTCTATATACAATGGTAAGGGTCTGGTGATTGTATCCTGAG
ATTAAAAACAATCTTGTAAGTGCCTTAAGTCACGTACTTTACCCTATATATACTGCAGCACA
ATAAAGCAAGGTATCAGCCATTTTGGTCTGATCCTCTCAACCCCATCTTTTGTCTATCTCTGA
TTTTCTTAGCGGGGTCGCTCCGTTCTCTCCCTGTGCAGGTGCGACTCTTGTTTGTGCTGGCCA
CGGCAGGTGGCGCCCAACGTGGGGCTCGAGCTCGACAGTTCTCCTCGCCACTACTCTTATTA
ATTGAAAAGAGTGAGTATATGAGTAAACAAGTGAATTAAATTGAGGAGGAGTAGTAAGGTA
TATAGTTGAGAGTATAATTATGGGACAGACGCATAGTCGTCAGTTGTTTGTGCATATGTTAT
CTGTAATGTTAAAACATCGGGGAATTACTGTTTCTAAACCTAAATTAATCAATTTTCTTTCAT
TTATTGAGGAAGTTTGCCCTTGGTTTCCCAGAGAAGGTACGGTAAATCTAGAGACATGGAAG
AAAGTAGGGGAACAAATCCGGACTCATTATACTTTACATGGCCCTGAAAAAATCCCTGTCGA
AACTTTATCCTTTTGGACACTAATTCGTGACTGCCTGGACTTTGATAATGATGAATTAAAACG
TTTAGGAAATTTATTAAAACAGGAAGAAGATCCTCTCCATGATCCTGATTCGGAACCCAGGC
ATGCTGTTCCCGAGGGAGTTGAAGGTGACCCTCCATTTTCTAACTTATTGCGTCCTTCGGATA
ATGATGATTTACTTTCATCCACAGATGAGGCAGAATTAGACGAAGAAGCTGCTAAATACCAT
CAAGAAGATTGGGGTTTTTTAGCACAAGAAAAGGGGGCATCAATATCTAAAGATGAATTGG
TTGAATGTTTTAAAAACCTTACTATTGCTTTACAGAACGCAGGAATCAAGCTTCCTAGTAAC
AATGCCAAATCTCCTTCTGGTCCGCCTCGTCCCCCTGCTTATGCTCCTTCTGTTGTGGCTGGTC
TCAATCCCCCTCCAGGGCCCCCTCCACCGTCTGAGAACATGTCTCCGCTGCAGAAGGCATTG
AGACAAGCACAGCGACTTGGTGAGGTTGTCTCTGATTTTTCTCTTGCTTTTCCTGTCTTTGAA
AATAACAACCAGCGTTATTATGAAACACTGCCTTTTAAACAACTGAAAGAGTTAAAGATTGC

TTGCTCACAATACGGTCCTACTGCTCCATTCACCATTGCTATGATAGAAAATTTGGGTACTCA
AGCTTTACCCCCAAATGATTGGAAGCAGACAGCTAGGGCATGTCTCTCAGGGGGAGATTATT
TACTATCGAAATCTGAATTTTTTGAACAATGTGCTCGTATAGCCGATGTTAACCGACAGCGA
GGTATACAGACCTCCTATGAAATGTTGATTGGTGAAGGCCCTTATCAGGCTACTGATACTCA
ACTTAATTTCTTACCTGGTGCATATGCACAAATATCAAATGCGGCTCGGCAGGCGTGGGAAA
AACTTCCTAGCTCCAGTACTAAGACAGAGGATCTTTCAAAAGTCCGGCAGGGACCTGATGAG
TCTTACCAGGACTTCGTGGCACGGCTCTTAGATACTATATGTAAGATAATGTCAGATGAAAA
GGCTGGGATGGTATTAGCAAAACAATTGGCTTTTGAAAACGCTAACTCTGCTTGTCAAGCTG
CCTTAAGACCTTATCGAAAAAAGGGAGATCTGTCTGATTTTATTCGCATTTGTGCTGACATTG
GACCCTCCTACATGCAAGGCATTGCTATGGCAGCAGCATTACAAGGAAAAAGCATAAAAGA
GGTACTTTTTCAGCAGCAAGCCCGGAACAAGAAAGGACTTCAAAAGTCAGGTAATTCGGGT
TGCTTTGTTTGTGATCAACCTGGCCATCGGGCTGCAGTGTGCCCTCAAAAACAACAAAGCCC
TGTTAACACTCCTAATTTGTTCCCACGATGTAAAAAAGGAAGACATTGGGCGCGGGATTGCC
ATTCTAAAACGGATGTTCAAGGTAATCCTTTGCCCCCGGTTTCGGGAAACTGGGTGAGGGGC
CAGCCCCTGGCCCCGAAACAATGTTATGGGGCAACACTGCAGGTTCCAAAAGGACCATTGC
AGACCTCTGTCGAGCCACAAGAGGCAGCGCAGGATTGGACCTCTGTGCCACCTCCTACACAG
TATTAACTCCCGAGATGGGGGTTCAAACCCTTGCCACAGGAGTGTTTGGGCCTTTACCTCCA
GGGACAGCTGGACTGCTTTTAGGGCACAGTAGTGCGTCTTTAAAGGGAATACTTATCCATCC
TGGTGTGATTGACTCTGATTATACAGGAGAGATAAAAATATTAGCCTCCGCTCCTAACAAAA
TTATTGTGATCAATGCAGGACAGCGTATAGCTCAACTTCTTTTAGTTCCATTAGTCATACAGG
GAAAAACAATCAATAAAGACCGTCAGGATAAAGGTTTCGGGTCCTCCGACGCCTATTGGGT
GCAAAATGTTACCGAGGCATGACCAGAACTTGAGCTACGCATTAATGGTAAGCTTTTCCGCG
GAGTGCTTGATACAGGGGCCGATATTAGCGTCATTTCTGATAAATACTGGCCTACTACATGG
CCTAAACAGATGGCTATTTCCACTCTTCAGGGTATTGGCCAAACTACTAATCCAGAACAAAG
TTCGTCCCTTCTTACTTGGACGGATAAAGACGGTCATACAGGCCAATTTAAACCTTATATTCT
GCCCCATCTTCCAGTTAATCTATGGGGGCGTGATATATTGAGCAAAATGGGTGTTTATTTATA
TAGTCCTTCACCCACTGTAACAGATTTGATGTTAGATCAGGGCTTACTTCCAAATCAAGGTTT
AGGTAAACAACATCAAGGCATCATTTTGCCCCTTGATCTAAAACCTAATCAAGATCGAAAAG
GCTTGGGGTGTTTTCCCTAGGGACCTCTGATTCTCCCGTGACACATGCCGATCCTATTGATTG
GAAATCTGAGGAACCGGTATGGGTCGATCAGTGGCCCCTAACACAGGAAAAACTTTCTGCT
GCACAACAGCTGGTGCAGGAACAGCTGAGACTTGGGCATATTGAACCCTCTACCTCTGCTTG
GAATTCCCCAATTTTTGTTATTAAAAAGAAGTCTGGGAAATGGAGATTGCTACAAGACCTTC
GTAAGGTAAATGAAACAATGATGCATATGGGAGCCCTACAACCTGGGTTGCCCACTCCTTCT
GCTATACCTGATAAATCCTATATCATTGTTATAGATTTAAAAGATTGTTTTTACACTATTCCT
CTTGTACCTCAAGATTGTAAGAGATTTGCCTTCAGTTTACCCTCTTAATTTTAAAGAGCCTAT
GCAACGCTATCAATGGAGAGTCCTCCCACAAGGAATGACTAATAGCCCTACGCTGTGCCAA
AAATTTGTTGCTACAGCATTAGCTCCCGTTCGTCAACGTTTTCCTCAGCTATATTTAGTTCATT
ATATGGATGATATATTACTAGCTCATGCTGACGAACATCTATTGTATCAAGCTTTTTCGATTT
TAAAACAACATTTAAGCCTTAATGGTCTTGTTATTGCTGATGAAAAAATTCAAACTCATTTTC
CTTATAATTATTTGGGTTTCTCCTTATATCCTCGCGTTTATAATACCCAATTAGTAAAATTAC
AGACTGACCACTTAAAAACTTTAAACGACTTTCAAAAACTTTTAGGAGACATTAATTGGATA
CGTCCTTATTTAAAATTACCCACTTATACCTTGCAGCCATTATTTGACATCCTTAAAGGTGAC
TCTGACCCTGCGTCACCCCGAACACTTTCTTTAGAAGGACGAACTGCCTTACAATCAATAGA
AGAAGCTATTAGACAACAACAGATTATTTATTGTGATTACCAACGATCATGGGGTTTGTATA
TACTTCCTACCCCCCGAGCACCCACAGGGGTTCTCTATCAAGATAAACCTTTGCGATGGATA
TATTTGTCTGCTACTCCAACTAAACATCTGCTCCCTTACTATGAACTTGTTGCAAAAATTGTA
GCAAAGGGACGTCACGAAGCTATCCAGTATTTTGGCATGGAACCCCCCTTTATTTGTGTTCCT

TATGCTTCAGAACAACAAGATTGGCTTTTTCAATTTTCAGATAATTGGTCTATAGCTTTTGCA
AATTACCCGGGACGGATTACTCATCATTACCCTTCTGATAAATTGTTACAATTTGCTAGCTCT
CATGCCTTTATTTTTCCAAAAATAGTTCGCCGACAACCTATTCCTGAAGCGACTCTTATATTT
ACAGATGGATCGTCTAATGGTACTGCAGCTTTACTTATTAACCATCAAACCTATTATGCACAT
ACCAGTTTCTCTTCTGCTCAGGTTGTGGAATTATTTGCAGTCCACCAAGCATTGCTAACTGTA
CCTACTTCCTTCAATTTATTTGCAGACAGCTCCTATGTGGTCGGTGCCTTACAGATGATTGAA
ACTGTTCCAATTATCGGCACCACCTCTCCTGAAGTTCTTAACTTATTTACATTGATTCAACAG
GTTCTCCACTGTCGCCAACACCCCTGTTTCTTTGGGCATATTCGTGCACACTCCACCCTTCCT
GGTGCCCTCGTACAAGGCAATCACACTGCGGACGTTCTTACTAAACAAGTGTTTTTCAATC
AGCTATTGATGCAGCCCGAAAATCTCATAACTTACATCACCAAAATAGTCATTCTTTACGGT
TGCAATTTAAAATTTCCCGTGAAGCTGCATGACAAATTGTTAAATCTTGCTCTACCTGTCCTC
AATTCTTTGTTCTTCCTCAATATGGTGTCAACCCTCGAGGCTTACGCCCTAATCATCTCTGGC
AAACAGATGTCACTCACATTCCTCAATTTGGGCGTCTGAAATATGTTCATGTCTCTATTGACA
CTTTTTCCAATTTTCTCATGGCCTCCCTTCACACTGGAGAATCGACACGTCACTGTATTCAAC
ATTTGCTATTTTGCTTTTCTATTTCAGGAATCCCACAAACCCTTAAAACAGATAATGGACCTG
GTTATACTAGCCGTTCTTTTCAACGTTTTTGTCTTTCTTTCCAAATTCATCATAAAACAGGAAT
TCCATATAATCCACAGGGACAAGGTATTGTGGAACGAGCCCATCAACGCCTTAAACATCAAT
TATTAAAACAAAAAAAGGGGAATGAACTGTATAGCCCCTCACCGCATAATGCCTTAAACCA
TGCTCTTTATGTTTTAAATTTTTTAACTTTAGACGCAGAAGGCAATTCAGCAGCCCAGCGTTT
TTGGGGAGAATGGTCCTTATGCAAAAAACCACTTGTACGATGGAAGGATCCACTTACCAATC
TGTGGTATGGGCCAGACCCTGTACTAATATGGGGACGAAGGCATGTTTGTGTTTTTCCACAG
GATGCCGAAGCGCCGCGTTGGATACCGGAAAGGCTGGTACGCGCGGCGGAGGAACTCCCTG
ACATATCAGATGCATCGCATGACACTGAGCGAGCCCACGAGTGAGCTGCCTACCCAGAGGC
AGATTGAGGCGTTGATGCGATATGCTTGGAAAGAGGCTCATGTACAACCTCCAGTGACACCT
ACTAATATACTGATCATGTTATTATTATTGTTACAGCGGATACAAAACGGGGCGGCTGCGGC
TTTTTGGGCATACATTCCCGATCCGCCTAAGATTCAATCTTTAGGATGGGATAAAGAAATAG
TACCTGTATATGTCAATGATACAAGTCTTTTAGGAGGAAAATCAGATATTCACATTTCTCCTC
AGCAAGCCAATATCTCCTTTTATGGTCTTACTACTCAATATCCTATGTGCTTTTCTTATCAATC
ACAGCATCCTCACTGTATACAGGTGTCAGCTGATATATCCTATCCTCGAGTGACTATTTCAGG
CATTGATGAAAAAACCGGAAAGAGATCGTACCGTGACGGAACCGGACCCCTCGACATTCCG
TTTTGTGACAAACATCTAAGCATCGGCATAGGAATAGACACTCCTTGGACTTTATGTCGAGC
ACGGGTCGCATCGGTGTATAACATCAACAATGCCGATACCACCCTTTTATGGGACTGGGCAC
CTGGGGGAACACCTGATTTCACCGAATATCGAGGACAGCATCCACCCATTCTCTCTGTAAAC
ACTGCTCCTATATTTCAAACTGAACTGTGTAAACTTTTGGCTGCTTTTGGTCATGGCAATAGC
CTATATTTACAACCCAATATTAGTGGGAGTAAATATGGTGATGTGGGAGTTACAGGATTTTT
ATATCCCCGAGCTTGTGTCCCTTACCCATTCATGTTGATACAAGGCCATATGGAAATAACGC
TGTCATTGAATATTTATCATTTAAATTGTTCCAATTGCATACTTACTAATTGCATTAGAGGTG
TAGCCAAAGGAGAACAAGTTATAATAGTAAAACAACCTGCTTTTGTAATGTTACCTGTTGAA
ATAACTGAAGAATGGTATGATGAGACCGCTTTAGAATTGTTACAACGCATTAACACGGCTCT
TAGCCGCACTCCAAGAAGTGTAAGCCTGATTATTCTGGGTATAGTATCTTTAATCACCCTTAT
AGCAACTGCTGTTACTGCTTCTGTATCTTTAGCACAATCCATTCAAGCTGCTCATACTGTAGA
TTCCTTGTCATATGATGTTACTAGAGTAATGGGAACTCAAGAAGATATAGATAAAAAAAATA
GAAGATAGATTATCAGCTTTATATGATGTAGTTAGAGTTCTAGGAAAACAAGTTCAGAGCAT
TAATTTTCGTATAAAAATTCAATGCCATGCTAATTATAAATGGATTTGTGTTACAAAAAAGC
CTTACAATACATCTGATTTTCCGTGGGATAAGGTGAAAAAACATCTACAAGGAATTTGGTTT
AATACTAATGTTCTTTAGATCTTTTACAATTGCACAATGAAATTCTTGACATTGAAAATTCTC
CAAAAGCTACGTTGAATATAGCTGATACTGTCGATATCTTTTTACAAAATTTATTTTCTAACT

TTCCAAGCCTTCATTCACTGTGGCGAAGTATAATTGCTATGGGCGCGGTTCTGACTGTTGTGC
TTATCATAATTTGTCTAGCTCCGTGTCTTATTCGTAGTATTGTTAAAGAATTTTTACACATGA
GAGTTTTAATACATAAAAACATGTTGCAACACCGACATCTCATGGAGCTTTTAAAAAATAAA
GAGAGGGGAGCTGCGGGGGATGACCCGTGAAGGGTTAAGTCCTGGGAGCTGCTCAGCAGGT
ATGCAGAGCCTAGGACATGCGCCTAAGCTCCCTGTCCCGCCACCCTCAAGAATTTTTATAGC
CCTTAAGGCTCCAAGTTGTTTGGTTTCGGCAACATTTCATAGAAGATAGATTATCTTATTGTG
TATACTTCATAGAAGATAGATTATCTGATTGTGTTCTATATACAATGGTAAGGGTCTGGTGAT
TGTATCCTGAGATTAAAAACAATCTTGTAAGTGCCTTAAGTCACGTACTTTACCCTATATATA
CTGCAGCACAATAAAGCAAGGTATCAGCCATTTTGGTCTGATCCTCTCAACCCCATCTTTTGT
CTCTCTCTTATTTCTTAGCAGGGACGCTCCGTTCTCTCCCTGTGCAGGTGCGACTCTTGTTTG
TGCTGGCCGCGGCA

>NC_056067.1:60740851-60748706#SHEEP_RIP_25(-)

TGCGGGGGACTGCCCGTGAAGGGTTAAGTCTTGGGAGCTGCTCAGCAGGTATGCAGAGCCTT
AGGCATGTTCCTAAGCTCCCTGTCCCGCCCCCCTGAAGAATTTTTATAGCCCTTAAGGCTCCA
GGATGTCTAGTTTCTGCAACACTTCATAGGAAAAGGTATTATGTTCTGTACACAATGGCAAG
GGTCTGGTGATTGTATCTTGAGATTAAACACAATCTTGTAAATGTCACAAGTCACGTACTTTA
TCCTATATATACTGCTGCACAATAAAGAAAGGCATCAGCCATTTTGGTCTGATCCTCTCAAC
CCCATCTTTTGTCTCTCTTACTTTTCTTAGCGGGGATGCTCCATTCTCTCCCTGTGCAGGTGCG
ACTCTTGTTTGTGCTGGCCGTGGCAGGTGGCACCCAACGTGGGGCTGTTCGACAGTTTTCCTC
ACCACTACTCTTATTAGCCGAAAAGAGTGAGTATATAAGTATACAAGTGATTTAAATTAAGG
AGGAGTAGTAAGGTATATAGTTGAGAGTATAAATATGGGACAGATGCATAGTCGTCAATTG
TTTGTACATATGTTATCTGTAATGTTAAAACATCGGGGAATTACTGTTTCCAAACCTAAATTA
ATCAATTTTCTTTCATTTATTGAGGAAGTTTGCCCTTGGTTCCCCAGAGAAGGCACAGTAAAT
TTAGAGACATGGAAGAAGGTAGGGGAACAAATTCGGACTCATTATACTCTACATGGCCCTG
AAAAAGTCCCTGTTGAAACTTTATCCTTTTGGACACTAATTCATGATTGTCTGGACTTTGATA
ATGATGAATTAAAACGCTTAGGAAATTTATTAAAACAGGAAGAAAATCCTCTCCATGTTCCT
GATTCGGAACCCAGGTATGCTGTTCCTGAGGGAGTTGAAGGCGACCCTCTGTTTTTTAAATT
ATCGCGTCCTTCGGATAATGATGATTCACTTTCATCCACAGATGAGGCGGAATTAGACGAAG
AAGCTGCTAAATACCATCAAGAGGATTGGGGTTTTTTGGCACAAGAAAAGGGGGCGTCAAC
ATCTAAAGATGATTTGGTTGAATGTTTAAAAAACCTCACTGTTGCTTTACAGAACTCAGGAA
TTAAGCTTCCTAGTAACAATTCTAAACCTCCTTCTGCCCCGCCTCTTCCCCCTGCCTATGCTCC
TTCCGTTGTGGCTGGTCTCGATCCCCCTCCAGGGCCTCCTCCTCCATCTGAGATTGTGTCTCC
GCTGCAGAAGGCATTGAGGCAAGCACAACGACTTGGTGAGGTTGTCTCCGATTTTTCTCTTG
CTTTTCCTGTCTTTGAAAATAACAACCAGCGTTTTTATGAAGCGCTGCCTTTCAAACAACTAA
AAGAGTTAAAGATTGCTTGCTCACAATACGGTCCTACCGCTCCATTCACTATTGCTATGATA
GAAAATTTGGGTACTCAAAATTTACCCCCAAATGATTGGAAACAAATAGCTAGGGCCTGTCT
CTCGGGGGGAGATTATTTACTATGGAAATCTGAATATGTTGAACAGTGTGCTCGTATAGCCG
ATGTTAATCGTATAGCCGATGTTAATCGGCAGCAAGGTATACAGACCTCTTACGAAATGTTG
ACTGGTGAAGGCGCTTTCCAGGCTACTAATACTCAACTTAATTTCTTACCTGGTGCATACGCA
CAAATATCAAATGCGGCTCGGCAGGCATGGAAAAAACTTCCTAGCTCCAGTACTAAGACAG
AAGATCTTTCAAAAGTCCGACAGGGACCTGATGAGCCTTATCAGGACTTCGTGGCACGGCTC
TTAGATACTATAGGTAAGATAATGTCAGATGAAAAGGCTGGGATGGTATTAGCAAAACAAT
TGGCTTTTGAAAACGCTAACTCTGCCTGTCAAGCTGCTTTACGACCTTATCGAAAAAAGGGA
GATCTGTCTGACTTTATTCGCATTTGTGCTGACATTGGACCCTCCTACATGCAAGGCATTGCT
ATGGCAGCAGCATTACAAGGAAAAAGCATTAAAGAGGTACTTTTTCAGCAGCAAGCCAGGA
ACAAGAAAGGATATCAAAAGTCAGGTAATTCGGGTTGCTTTGTTTGTGGTCAGCCTGGCCAT

CGGGCTGCAGTGTGCCCCCCAAAACAACAAATCCCTGTTAACACTCCTAATTTGTGCCCACG
ATGCAAAAAGGGGACGCATTGGGCCCGGGATTGTCGTTCTAAAACGGATGTTCAAGGTAAC
CCTTTACCCCCGGTTTCGGGAAACTGGGTGAGGGGCCAGCCCCTGGCCCCGAAACAATGTTA
TGGGGCAACACTGCAGGTTCCCAAAGAACCATTGCAGACCTCTGTCGAGCAACAAGAGGCA
GCGCGGGATTGGACCTCTGTGTCACCTCCTACACAGTATTAACACCCGAGATGGGGGTTCAA
ACCCTTGCCACAGGAGTGTTTGGGCCTTTACCTCCAGGAACAGCTGGACTGCTTTTGGGGCG
CAGCAGTGCGTCTTTAAAGGGAATACTTATTCATCCTGGTGTAATTGACTCTGATTATACAG
GAGAGATAAAAATATTAGCCTCCGCTCCTAACAAAATTATTATAATCAATGCAGGACAACGT
ATAGCTCAACTCCTTTTAGTTCCATTAGTTATACAAGGAAGAACAATTAACCGAGACCGTCA
AGATAAAGGTTTCGGGTCCTCTGACGCCTATTGGGTGCAAAATGTTACCGAGGCATGACCAG
AACTTGAGCTACGCATTAATGGTAAGCTTTTCCGCGGAGTGCTTGATACAGGGGCCGATATT
AGCGTTATTTCTGAAAAATACTGGCCTACTACATGGCCTAAACAAATAGCTATTTCCACTCTT
CAGGGTATTGGCCAAACTACCAATCCAGAACAAAGTTCGTCCCTTCTTACTTGGAGAGATAA
AGATGGCCATACAGGTCAATTTAAACCTTATATTCTGCCCCATCTTCCAGTTAATCTATGGGG
GCGTGATATATTTTTATTTATATAGTCCTTCATCCACCGTAACAGATTTGATGTTAGATCAGG
GTTTACTCCCAAACCAAGGTTTAGGCAAACAACATCAAGGTATCATTTTACCCCTTGATTTA
AAATCTAATCAAAGTCGAAAGGGCTTGGGGTGCTTTCCCTAGGGACCTCTGATTCTCCTGTG
ACACATGCTGATCCTATTGATTGGAAATCTGAGGAACCGGTATGGGTCGATCAGTGGCCCCT
AACACAAGAGAAACTTTTTGCCACACAACAGCTGGTGCAGGAACAGCTGAGACTTGGGCAT
ATTGAACCCTCTACCTCTGCGTGGAATTCCCCAATTTTTGTTATTAAAAAGAAATCTGGAAA
ATGGAGATTGCTACAAGACCTTCGTAAGGTAAATGAAACGATGATGCATATGGGAGCCCTA
CAACCTGGATTGCCCACTCCTTCCGCTATACCTGACAAATCCTATATCATTATTATAGATTTA
AAAGATTGTTTTTACACTATTCCTCTTGCACCCCAAGATTGTAAAAGATTTGCCTTTAGTTTG
CCCTCTGTTAATTTTAAAGAGCCTATGCAACGCTATCAATGGAGAGTCCTCCCACAAGGAAT
GACTAATAGCCCTACGTTGTGTCAAAAATTTGTTGCTACAGCATTAGCTCCTGTTCGTCAACT
CTTTCCTCAATTATATTTAGTTCATTATATGGATGATATATTATTAGCTCATGCTGACGAACA
TCTATTGTATCAAGCTTTTTCTATTCTAAAAAAACACTTAAGTCTTAATGGTCTTGTTATTGCT
GATGAAAAAATTCAAACTCACTTTCCCTATAATTATTTGGGTTTCTCCTTATATCCTCGCGTT
TATAATACCCAATTAGTAAAATTACAGACTGACCATTTAAAAACTCTAAATGATTTTCAAAA
ACTTCTAGGAGACATTAGTTGGATACGCCCTTATTTAAAATTACCCACCTACACCTTGCAACC
ATTATTTGACATCCTTAAAGGTGACTCTGACCCTGCGTCACCCCGAACACTTTCTTTAGAAGG
ACGATCAGCCTTACAATCAATAGAAGAAGCTATTAGACAACAACAGATTACTTATTGTGATT
ACCAACGATCATGGGGTTTGTATATACTTCCTACCCCTCGAGCACCCACAGGGGTACTTTAT
CAAGATAAACCTTTGCGATGGATATATCTATCTGCTACTCCAACTAAACATCTGCTCCCTTAC
TATGAGCTTGTTGCAAAAATTGTAGCAAAAGGACGCCATGAGGCCATCCAATTTTTTGGTAT
GGAACCCCCTTTCATTTGTATTCCTTATGCTTTAGAACAACAAGATTGGCTTTTTCAATTTTC
AGATAATTGGTCTATAGCTTTTGCAAATTACCCGGGACGGATTACTCATCATTATCCTTCTGA
TAAATTGTTACAATTTGCTAGCTTTCATGCCTTTATTTTTCCAAAAATAGTTCACCGACAACC
CATTCCCGAAGCGACACTTATATTTACAGATGGATCTTCTAATGGTACTGCAGCTTTAATTAT
TAACCATCAAACCTATTATGCACATACCAGTTTCTCTTCTGCTCAGATTGTGGAATTATTTGC
AGTTCATCAAGCATTGCTGACTGTACCCACTTCCTTCAATTTATTTACAGACAGCTCCTATGT
GGTCGGTGCCTTACAAATGCTTGAAACTGTTCCAGTTATCGGCACAACCTCTCCTGAAGTTCT
TAACTTATTTACATTAATTCAACAGGCCCTTCACCGTCACCAATACCCGTGTTTCTTTGGGCA
TATTCGTGCACATTCCACCCTTCCTGGTGCCCTGGTACAAGGCAACCACACTGCGGACGTTCT
TACTAAACAGGTGTTTTTTCAATCAGCTATCGATGCAGCCCGAAAATCTCATAACTTACATC
ACCAAAATAGTCATTCTTTACGGTTACAATTTAAGATTTCCCGTGAAGCTGCACGACAAATC
GTTAAATCTTGCTCTACCTGTCCTCAATTCTTTGTTCTCCCTCAATATGGTATCAACCCTCGAG

GTCTACGCCCTAATCATCTCTGGCAAACAGATGTCACTCACATTCCTCAATTTGGGCGTCTTA
AATATGTTCATGTCTCCATCGACACCTTTTCCCATTTTCTCATGGCCTCCCTTCATACTGGAG
AATCAACTCGTCACTGTATTCAACATTTGCTTTTTTGCTTTTCTATTTCAGGAATCCCACACAC
CCTTAAAACAGATAATGGACCTGGTTATACTAGCCGTTCTTTCCAACGTTTTTGTCTTTCTTTC
CAAATTCATCATAAAACAGGAATTCCATATAATCCACAGGGACAAGGTATTGTAGAACGAG
CCCATCAACGCCTTAAACATCAATTATTAAAACTAAAAAAGGGGAATGAACTGTATAGTCTC
TCACCGCATAACGCCTTAAATCATGCTCTTTTTGTTCTAAACTTTTTAACTTTAGACGCAGAA
GGCAATTCAGCAGCCCAGCGTTTTTGGGGAGAACGGTCCTCATGCAAAAAACCACTTGTACG
ATGGAAAGATCCACTTACCAATCTGTGGTATGGGCCAGACCCTGTATTAATATGGGGACGGG
GACATGTTTGTATTTTTCCACAGGATGCCGAAGTGCCGCGCTGGATCCCGGAGAGGCTGGTA
CGCGAGACAGAGGAACTCCCTGACATATCAAATGCATCGCATGACATTGGGCGAGCCCACG
AGTGAGCTGCCTACCCAAAGGCAGATTGAGGCGTTGATGCAATATGCTTGGAATGAGGCTC
ATGTACAACCTCCAGTGACACCTACAAACATACTGATCATGTTATTATTGTTACAGCGGATA
CAAAACGGGGCGGCTGCGGCTTTTTGGGCGTACATTCCTGATCCGCCTATGATTCAATCATT
AGGATGGGATAGAGAGATAGTACCTGTTTATGTCAATGATACAAGTCTTTTAGGAGGAAAAT
CGGACATTCACATTTCTCCTCAGCAAGCCAGTATCTCTTTTTATGGTCTTACTACACAATATC
CTATGTGCTTTTCTTATCAATCACAACATCCTCACTGCATACAGGTGTCAGCTGAGATATCCT
ACCCTCGAGTAACTATTTCTGGCATTGATGAAAAAACCGGAAAAAGATCGTACCGTGACGG
AACCGGACCTCTCGACATTCCGTTTTGTGACAAACATTTAAGCATCGGCATAGGAATAGACA
CTCCTTGGACTTTATGTCGAGCCCGGGTTGCATCAGTGTACAACATCAACAATGCCAATGCC
ACCTTTTTATGGGACTGGGCACCTGAGGGAACACCTGATTTCCCCGAATATCGAGGACAGCA
TCCACCCATTCTCTCTGTAAACACTGCTCAAGTATATCAAACAGAACTGTGGAAACTTTTGG
CTGCTTTTGGTCATGGCAATAGCCTATATTTACAACCCAATATTAGTGGGAGTAAATATGGT
AATGTAGGAGTTACAGGGTTTTTATATCCCCGAGCTTGTGTCCCTTACCCATTCATGTTGATA
CAAGGCAATGTGGAAATAACACTGTCATTGAATATTTATCATTTAAATTGTTCTAATTGCATA
CTTACCAATTGCATTAGAGGTGTTGCCAAAGGAGAACAAGTTATAATAGTAAAACAACCTGC
TTTTGTAATGTTACCTGTTGAAATAGCTGAAGCTTGGTATGACGAGACTGCTTTAGAATTGCT
ACAACGCATTAACACGGCTCTTAGCCGCACAAAAAGAAGTTTGAGCCTGATTGTTCTGGGTA
TAGTATCTTTAATCACCCTTATAGCAACTGCTGTTACCGCTTCTGTATCTTTAGCACAATCCA
TTCAAGCTGCTCATACTGTAGACTCCTTGTCATATAATGTTACTAAAGTAATGGGAACATAA
GAAGACATAGATAAAAAAATGGAAGATAGATTATCAGCCTTATATGATGTGGTTAGAGTTCT
AGGAGAACAAGTTCAGAGCATTAGTTTTCGCATGAAAATTCAATGCCATGCTAATTATAATT
GGATTTGTGTTACAAAAAAGGCTTACAATGCATCTGATTTTCCGTGGGATAAGGTGAAAAAA
CATCTACAAGGAATTTGGTTTAATACTAATGTTTCTCTAGATCTTTTGCAATTGCATAATGAA
ATTCTTAATATTGAAAATTCTCCAAAAGCTACTCTGAATATAGCTGATACTGTCAACAATTTT
TTACAAAATTTATTTTCTAACTTCCCTAGCCTTCATTCACTGTGGCGAAGCATAATTGCTGTG
GGCGCGGTTCTGACTGTTGTGCTTATCATAATTTGTTTAGCTCCTTGTCTTATTCGTAGCATTG
TTAAAGAATTTTTACATATGAGAGTTTTGATACATAAAAACATGTTGCAACCCCGACGTCTT
ATGGAGCTTTTAAAAAATAAAGAGAGGGGAGCTGCGGGTGACTGCCCGTGAAGGGTTAAGT
CTTGGGAGCTGCTCAGCAGGTATGCAGAGCCTTAGGCATGTTCCTAAGCTCCCTGTCCCGCC
CCCCTGAAGAATTTTTATAGCCCTTAAGGCTCCAGGATGTCTAGTTTCTGCAACACTTCATAG
GAAAAGGTATTATGTTCTGTACACAATGGTAAGGGTCTGGTGATTGTATCTTGAGATTAAAC
ACAATCTTGTAAATGTCACAAGTCACGTACATTATCCTATATATACTGCTGCACAATAAAGA
AAGGCATCAGCCATTTTGGTCTGATCCTCTCAACCCCATCTTTTGTCTCTCTTACTTTTCTTAG
CGGGGACGCTCCGTTCTCTCCCTGTGCAGGTGCGACTCTTGTTTGTGCTGGCCGCGGCAGG

>NC_056071.1:25126897-25134729#SHEEP_RIP_26(+)

TGTAGGGAACAAGGTATAAGGAAGGTTGAGAAGTGCTTGCAAACGAGACTCTCAACCAGGG
CTGAATATTCTTGCAAACGAGATGTTCAGCTAAGAATCCTCTGTTTGTTCCCGTGGGAAAAG
AACATTTCTTGCACACAAGGATGTTTCTCTGATTCCTCAAAAGGAACAGACCCAGGGACAGA
ATGGATTCTAAGTTGATAAGGAAGTTCCCCAAAACAAAGTCTTAGCTGCATGCAGTAAATAA
GTCAAGGTAAAGCTTATCTCGCCCTGTGCCTGCGCACTGTATAGTCTCTGAATCATAGTGCTT
GGCAACTTGCCCTGTAGGTTGTTGTGCAAGGGATATAAAATAAAGCAGCTGTAAGAAGCAA
GTGTTAAAATACAAAGATTCAAACCACTCTGCTGTGTTGGTGTTTCATTCCGTCGCCAGCATC
TGGCGCCCAGCCCGGGGCACGACGGAACCGAAAGGGTAAGCACCCCGGGGCAAAACGCTG
AAAGGGGGACTTTCAGGAAATAGAGTAAAAGAGAAAAGAAAAAAATCTGAAAGGGTGCGC
ACCCCGGGGCTAAATGCTGAAAGGGGGACTTTTGAGAAAAAGAGAAAAGAAAAAAAAATC
CGGAAGGGTGAGCACCCTGGAGCGAAACGCTGAAAGGGGGACTTTCAAGAAAAAGAAAAA
ACTATGGGGAATTCCCTGTCTTTAAAGTCACAATATATGGAGCTGGTTAAGGGGCTCCTGCA
TTCTATAGGAATTAAAACGTCTACTCGCCGCTTGAGTGAGTTGTTCTGTCTTATAGAGCAGCA
TTGCTATTGGTTTCAATATCAAACAGAAGTACAACTGAATTTGAAGAAATGGAAAGTGGTGC
AGAAAGAGTTAAGAAGGCAGCATCAAAAAGGAAATGTGATTCCTTTAAGGCTGTGGACTCT
ATGTAGCGCTATAACACAGGCTTTAAAATTAATGGCTGCTGATAGTGAGGCTGTCTCATGTT
CTCTTAAGAAGGAAGATCCCCTGTATGAGGATGTGCCTATGAGTGAGAAGGAAGATGCAGG
TGAAAGGGGGGGCTTCATCTCGGCCTCCGCTCAAACCCGCAGATGATAGTAAAGATTCATCC
ACTGAGCCAGATAGAGATGTGAGTTCTGGGGAGGATTCAGATTTTGTAGAGGCAATGACTA
CTGCCTTTCGGAAGGTTTTATCAAATAAAAAGCGAACTTCCAAGGCTGTTAAATCTTCTGCA
CCGCTCTACGCATCCCTATTTCCAGCAGCTGCCGATAAGGCTGAGGTAGGGAGAGAGAATCA
GCTATTTACATTTCCTGCTACTGAGTTCCATGATGATGATGATCTTTCAGCGCCCCCTGGAGG
TTTTGTGGATCCACCTCGCCTCTTTCCCATTATACGTCAGCATGATGCACGGGCGGGAATGAT
TAATGTTCAATATATGCCTTTGGAGTATAAATTTTTTAAAGATCTTAAGGCCGCTGTTTCTCA
ATATGGCCCCCAATCTCCTTTTGTGTTATCAATGTTAGAGAATGTGGGAACTTCTAAATTAAT
TCTTCCTCTTGATTGGGAATCTATAGCTCAAGCTGTTTTAGAAGGATCTCAATGGTTACAGCT
TCATAGTTGGTGGGAAGAAGAAGCCCGAAAACAACCTAGGATTAATGAGGGACAAAATCCA
CAAGGTCCTTTAGAAGATAAGTTGATGGGAGCAGGGCAGTATCGAGCTTTGAGGGAACAAG
CTCAGTACACAGATCAGGAATTACAAAAGGTGCGTCAGGTTTTCTTAAGGGCGTGGCGTAGA
GTGGTACCTACTGGACAGGCTCAACCGTCTTTTGTGAAAACTATACAAGGTCCTAATGAGCC
TTATACAGATTTTTTGGCCCGCCTTCGGGTGGCTATTGAACGGACAGTCGGTCAAGATGAGA
TCTCAAAAATTTTACTTGATACATTGGCATATGAAAATGCTAATCCAGAATGTAAAAGGATT
TTGGGACCTTTAAAAGGACAAGGGGCTTCTGTGGCTGAGTTCATAACAGCATGCTCTGGAGT
TGGAAGAGCTAAACATCAAGCCTCTGTTTTTGCAGCAGCTTTGGCAAAGGTTGTGAAACCTC
AGAGGGGAAGAAACTGTTTTAATTGCGGGAAGCCAGGACATTTTCGGAAAGATTGCAAGAG
ACAAAAAGATGAGCCAAAAAATGAACGATTGCCAGAAAAGTGACAGCCCTCAGGTCTTTGT
AGGAGATGTGGCAAGGGGAAGCACTGGACTCAGGATTGTAAATCCAAAACTGATCGGGAAG
GGAATCCCTTAAGTGCCCCTTTGTTGGGAAACTTCCCAGTGGGCCTGAGCTCCTGGGGCCCA
GGAACAGTACCTGGAGATCCTTGCCAGTTTCCTGCACAGGTCAATGCCAATCTCGACAGCCA
GTTTCCTCGAATGGTGCTCAAATGATGATTTCAGATTTGAAGGCTGCTACATTAGGTAGTGC
AGCTGCAGATCTTCCATTAGCTGAAAATGTTATTTTATCCCCAGGAGGAGGTGTTTATAAATT
ACGGACTAATGTTTTTGGCCCATTACCAAGAGGAACTTTTGGGTTAATTTTGGGACGAAGTA
GTGCAACATTAAGAGGATTGATTGTATACCCAGGAGTAATTGATTCTGACTATGTCAGGGAA
ATTTTAATTATGGTTTCTACATCACAAACTCTTTCTTTACTAGCAGGGGAACGGATTGCACAA
CTTCTCTTGTTACCTTATCATCCGTTTTCTTCATATTCAAATGAGAGAGTCGGTGGTTTTGGA
AGTACGGGAAAAAGTATACTTTGGGAAATGCTCATTAATGACTCTCATCCTTTAATGTCACT
GATTATTGAAGGTAAACAGTTTGAAGGATTAGTGGATACAGGAGCAGATGTTTCGGTTATTT

CTCTCCAGCAGTGGCCTAATGACTGGAAAAAGGAAAAAATTCCTCTTGTTTTAACAGGCTTA
GGATCAATAGCAGATGTGTGGAGAAGCACTCAACCTTTGTCATGCCAATTGTCTAATGGAAA
GAAAGTATTTATTTATTTTTACATTGTTAATATACCTATTAATATCTGGGGAAGGGATCTTTT
ATTTTCTTTTGGAACAATGCTCACCATCTTGTCAGAAAACTTGTAGCCACTGCTCAGATTCCT
CAAGCTCTCCCATTGAAATGGTTAACTGATGTCCCTAAATGGGTTGAGCAGCGGCCGCTTCC
AAAAGTGAAGCTCGAGGCTTTAGAACAATTAGTAGAACAGCTTCAATCTGGCCATATCAAA
CCTTCCACGTCTCCTTGGAATTCCCCTGTTTTTGTCATTAAGAAAAAATCTGGTAAATGGAGA
ATGTTGACTGATTTAAGGGAAGTTGACAAATGTATAGAACCTATGGGAGCTTTGCAATTAGG
TCTCCCTTCTCCTGCTTTGATTCCACAAAATTGGTCTTTGATGGTTTTAGATTTGAAGGATTGT
TTTTTTTAATATTCCTTTGCAAATAAAAGATAGAAATAAATTTGCTTTTACTATTCCAGTGTA
TAATCATGGGCAGCCCGTAAAACGTTATCAATGGACAGTGTTGCCTCAAAGAATGATTAATA
GCCCTACACTTTGTCAGGAGTTTGTTGATCGTGCTCTTATTACTGTGAGGCAACAGTTTTCTA
ATTGTCTTCTCTATCATTATATGGATGATCTTCTATTGGCAGCTCCTAGTAAAGAGGAACGAG
ATAAATTTTTTATTCATGTGAAGAAAGCTTTAAGTGATTTGAATCTTCAAATTGCTCCTGAAA
AAATTCAAACTGAATTTCCTATTTCATATTTAGGTGCTATTTTGGAACGACAAAGAATAAAA
CCTCAAAAGGTCCAAATTAGACGAGATAATTTGAAAACTCTTCATGATTTTCAGAAGCTTTT
GGGTGATATTAACTGGTTATGTCCCATATTAGGGATCCCTACACATTAACTGCGTCATTTATT
TTCTACTTTGGAGGGTGATGCTGCTCTTAACAGTCCTCGATCTCTTACTTCTCAAGCAAAAGA
GGAATTATCCTTTGTAGAGCAACGGTTGAATGAAGGGTTTCTTACTTATCTACAACAGGATC
AACCTATATATTTCATAGTGTTCCATACTCCCTATTCTCCTACTGGAGTTATTGCTCAATCTGC
AGGGTTGATTGAGTGGGTATTTTTACCCAATAACTATACAAAAAAGCTTACTACTTATATGG
ATAGAATTGCTTTCCTTATTTCGAAAGGTCGAGGCCGTATTACTCAGTTAATTGGAAGTGAC
CCTCAAACAATTATTACTCAACTTACTTCCACCCAAATTTCTAATTGCTTACAGTTTAATGAA
AATTGGCAGATAGCTCTTGCATCTTATTCAGGGACTTTTTCTAATCAATATCCTCAATCTAAA
ATGATAGATTTTCTTTGACATACATCAATGGTTTGTAAATCTCCTATATCTAATACTCCAGTT
GAAGGAAAAACTATTTTTACAGATGCCAATAAAAATACTGCTGGCTATTGGACTGATACTAT
TTCTAAAGTCGTTCCACATTCCTTTTCTTCCTTGCAGCCTGCTGAACTTTGGGCCATTTGTTTG
GCATTACAGGATTTTTTTTGATATTCCTATTAATATTGTTTCTGATTCTAAATATGCAGTCTTT
TCATGTATTTATCTTCCTGAAGCCACTCTACCGGTAACTTTAAAAACTAATATTGATAACTTA
TTTTTTCAGGTTCAACAATTATTAATTAGGCGAACTAACCCTGTCTTTTTTACTCATATTTGAG
CTCATTCTTCTCTCCCCAGCCCATTATCTCAAGGAAATGCAAATATAGATGCCTTACTTTATC
TTTTACAGTCTGCAACACAAGAACATTGCTTACATCATACTAATTCTAAAGGGTTACAAAAA
ACTTATTCGTTAACACGAAAACAAGCTCAACGCATTGTTCGTTCTTGTTCCATATGTGCACCA
TTTATTTTGCCCTTTGCCCCTCCTGGAGTAAATCCCAGAGGATTACAATCTAACCAAATCTGG
CAAATGGATGTTGTTTTTATTTCTTTATTCGGTAAACAGAAATATGTTCATCATACTATAGAT
ACTTATTCTCATTTTCAATGGGCTACAGCTTTAAGTTCAGAAAAGGCAGATTCTGTTATTACT
CATTTACTATTTTGTTTTGCTATTATGGGCATACCTATAGAATTAAAAACTGATAATGCTCCT
GCATATCAATCATCTAAATTATCTCAATTTTTAGAACAATATCATATTAAGCACACTTTTGGT
ATTCCTTATAATAGTCAAGGACAAGCTATAATTGAAAGAGCTAATCGGACTCTTCGAGAATA
CATTGAAAAAATAAGAAAGGAGGAAAAGGGTGTGATATCACCAAGGGATGTATTAAATAAA
ACACTATTAACTCTTAACTTTTTGAATGTCTGGGGAAAAAGTAAGATGACTCCAGCAGAACA
GCATTTTGGCAATTCAGAAAAGAAGACAAAAAAGCTTTGAACATTCCCAGGTATGGTATAA
GGATGAGATAAAGGGATGGATTCCGGCAGTACTTCACTTCTTGGGGCGAGGGTATGCTTTTA
TTTCCGTGAATAATTCCAGGATCTGGGTTCCATTGAGATTGGTGAAGTTGCAGGATGAGCGA
CCGGTTCGTGCGCCAGTTTCAGGAACTGTCAATGCAGAGGACTTTCACCTTTGTGACAAGAG
AGGCAGGTCCACTGACATGGGGACAGTTGAAGAAACTGACTCAAGAAGCTGAGAAAACATT
GCAGAAAGCAGGACAACCATTGAATCCTACTAATCTTTTGTTGGCCATGATGGCGGTGGTAA

CTTGTCAGGTAATGTATGTGGAAGCCACAAACTATACCTATTGGGCCTATATCCCTAATCCTC
CTTTGGTGCGGGCGGTGTCCTGGGGTGAAGTAGAAGTCCAAGTTTGTACTAACGAAACTAAT
TTTTTCCCTCCACCTGATTGTGGGGGGATTGAGCAATTATCCCAGCATGTACAGAATTATAAC
TTTACTAATCTTACTATAGCAGCTGAAGGTATTCCTTTATGTATAGGAGGGCATTCTTTTTGT
CTGTCTACAAAAACACACTCACATCATTCTTGTAATACTTGGGGAGTTAATTATAGGGGACA
ACACTTCGCAGTTTTTACTGTGCTCATTTCTTCTCGTGGCTTTAATACTTCACGGTCTTATTTA
AATAAGAGTAAGAGGATACATATGTCCCTCTGCCCTGCTTCATTTTTTGAAGCATCTTTATCA
CGTCTGGAGTGGGAACATTGCAGGGATCATGACCCTCGTCTAGTTATGAATTTTTCAAATCA
TGTAATTGTAGATTGGAGTCCTACTCATGGAGAATTTTTAGAAAAATGGTCTAATCGATCTTT
AAAATGGCATCGCATAAATGGTACCTTGAATGGTCAAGGAAATGAAACTGTAAAATGGCAA
CATTTTGCATTGGTTCCACCTCCATTACAAATGGATGGGCACCCTCAATTACAAGGAGACAT
ACGGAAATTATGGGCAGTGTCAGGTGACCTTACTATATGGACGGGAAACTTTACTTTAAATA
TTAATGATCCTAAGGGGCCATTTCAAGTTCATTTGATATTAATAAATCTTACTCTGCTATAGC
ATGTGTAAAATTTCCTTATTCTTTACTGTATGGGCAGTGGATTTGGAATGAAATTTTGGGAGT
TATATCATGTTCTGATTGTAACTTGACTCAATGTATAAATCATTCCTGGTGGGAAAATGTTGA
AAACAAGATGGTCCATTCCAGTAAATTACTCTTTGGTCATTGTGAAAGCACGGACTGAACTT
TGGTTGCCTGCTAATCTTACTCGACCTTGGTCGGACTCTTTTGCTGTCACTCATCTTGTGAAT
GCTGTACAAACTCTTCTCCATCGATCTCGAAGAATGCTTGGCATTGTCATTGCATCAATTCTC
GCCGTGGCTTCAGTAACAGCAACAGCAACTGTAGCAGGTCTTGCTTTGCATCAGGGCATCCA
GACTGCTGATTTTGTCAGGGAGTGGCATAAGGATGCTCATTTATTGTGGCAACAACAGCATG
ATTTGGACGCTCAATTGGCTACTGATGTATTGAATTTACAACTCACTGTTTCCTGGTTAGGAG
ATCAAGTGACTGTGTTAGCTACAAAGAGTGTGTTAAAATGTGATTGGAACTCATCTCACCTA
TGTGTAACCCCTGTTCCCTTTAATATGAGTGAGGGTTAAGAAAAGGTGAAAAGGTCTTTAGT
GGGACACCAGAATTTAACTGCTTAAATTATAGAATTGAAACAAACTATATTGTCAACTTTTA
GTAAGACGCTGCCTGATATTCTTGGTTCTGATATACTGAAGAGTCTTCAAGAAGGATTAGAT
AACCTTAATCCTTTACAGCATGTATCTACATTGTTAACAACATCCTTTGTAAATACTTTGTTA
ATTGTTGCTTTATGTTTTATTGCTTTTATAGTCTACCTGCGCTGGCGAAAAGGGAAACAATTG
AAAGAAGAAGCTTTGCGTATTCAAGTGCATATTCAGCACCTGCAAGAAAAGAAAGGGGGAG
ATGTAGGGAACAAGGTATAAGGAAGGTTGAGAAGTGCTTGCAAACGAGACTCTCAACCAGG
GCTGAACATTCTTGCAAACGAGATGTTCAGCTAAGAATCCTCTGTTTGTTCCCGTGGGAAAA
GAACATTTCTTGCACACAAGGATGTTTCTCTGATTCCTCAAAAGGAACAGACCCAGGGACAA
AACGGATTCTAAGTTGATAAGGAAGTTCCCCAAAACAAAGTCTTAGCTGCA

>NC_056072.1:14957480-14965080#SHEEP_RIP_27(+)

TGAAGGGTTAATGCAGCCACAATAGGGAAAGTGGAGAAGTGCCTGCAGACGGGGCTCTCTG
CTCGGGCTGAACGTACTTGCAAGTGAGGCGTTCTGCCAAGGAGTCTGGACATAGCCTTGAGT
TTGATGGTCCCTTGCAAACGAGGGAACATTCCCTTCTTGTGATAAGAAGAAGGAGGAGGGCT
CTGGACAGACTCTGCAGTAAGCAGAAATTTCAGTCCCTTTTGCTGTATGATAACATTTATGC
ACATGCGCTATACTGAAAAGGCTTGGTCATACAGTCTGGAATTCCGCCCAGGGGGGCTATAT
AAAAGTGAACCACAAGCTCGCTTGCTTGCGCAGTTCTTTTTCCTCTGGCCAGAATGGTGTCTG
TCTTTTGTGTGTGTTGTCTTTGTGTCATTTCACTGGCAATCTCCAACATCTGGAGCCCAACGT
GGGGCTCGAGTGAAACCGAAAGGGTGAGTAACCCCGGGGGGATTTTAAATCCATAGCAGGG
GAACTTTTGGGAAAATCATGGGGAATTCCTCACCCTAGCAGGGGAACTTTCAGGAAATCATG
GGGAATTCCTCATCATTAAGGACACAGTACATGGAGTTAGTCAAAGGACTTCTCCACTCCAT
AGGCGTTAAGGTCTTGACTCGTCGATTGAGTGAGCTCTTTCGCTTGGTGGAGAAATATTGTC
ATTGGTTTCAATATCAAACTAAGTTACAGTTAAATTTGAAGGAATGGAAAATAATTCAAAAA
GAATTGAGAAAGCAACATCAGAAGGGTAATGTGATTCCTTTGAAGTTATGGACTTTATGTAA

TGCTGTAACACAGGCTTTGACTTTGCTATCTACTGATAATGAGAGTAAATCTAATGCTTCAAT
GAAGGAAGAGGCAATTTATGAGGATGTGCCAGACGTTGGTGGGGCTTCTGTGTTGCCTGAA
GGCAAGGATACAGGTGAGCCTCCTCCTGTAAATGGTGAAACATCTGATTGTTCAGAATCAGA
TTCGGAGGCTTCTTCGGTTTCGTCAGAGGAGGGTAAAGAGATTAAAGAAATGACCCATCTAT
TCCAGGAGTGGTGGAAATCCCATAAGGAGGAGAAGAAATCTGCGCCTTCTGCTCCTCCTTGT
GCTTCTCTTTTCCCCACTGCGGTTGGTCGGCCCGATGTGCGCAGGGAACATTGTCGGTTCTCC
TTTCCTTTGTCTATGCTTCATGATGATGACTCGCCTGCTCCCCCTGGTGGTTTTATCGATCCTC
CACAATTATTTCCCATCCAGAGACAGCAAGATGACAACGTGATAAATGTTCAATACACTCCT
TTGGAATATAAATTTTTTAAAGATCTTAAAGCTACAGTAGCGCAGTATGGTCCTCAATCTCCC
TTTGTTTTGGCTATGCTGGAATCATTGGGAAAAGGCAAATTAATTATTCCATTAGATTGGGA
ATCTATTGCCCAAGCTGTCTTGGAGGGTTCTCAATGGTTGCAACTTCGTAGCTGGTGGGAAG
AAGAAGCTAGAAAGCAGGCTCGGATTAATGAAGGACAGAATCCCCCTGGTCCTCTTGAGGA
TAAGTTAATGGGAGAGGGCTGTTATCGGGCTTTAAGAGAACAGGCTCAGTACTCTGATCAGG
ACTTACAGCAAGTCCGCCAGGTCTTTTTACGAGCATGGCATCGTGTGGTGCCTACTGGCCAC
ACCCAGCCCTCCTTTGTTAAAACAATGCAAGGCCCCAGTGAGCCATATACTGATTTTCTAGC
AAGACTGAGGGTAGCTATGGAATGAGCTGTAGGGAGGGATGAAATTTCAGAGATATTATTA
CAAACTTTAGCATTTGAAAATGCAAATACTGAATGCAAACGTATCCTGGGACCATTAAAAGG
ACAGGGTGCATCTATAGCTGAATATATCAGAGCCTGCTTGGGAGTAGGAGGAACAGAGCAT
CAGGCTAGTGTCTTTGCTACAGCCTTGGCCAAAGCTATGAGACCACAAAAGGGAGGTAACT
GCTTCCATTGTGGAAAACCTGGTCACATGAGAAGAGAATGTTGGAAATTAAAAGCTGATCA
AGGTGCAATTCCTAAAGACAGATCTTTTGCTGGGAGGAATAAGACTCCTCCCGGACCTTGCC
GTCGATGCGGGAAGGGGCTTCACTGGACCAATGAGTGCAAATCTAAAACAGACAAAATGGG
CAACCCGATACCGGGAAACTATCCTGCGGGCCTGAGTCCTTGGGGCCCAGGAACAATACCG
GAGGCTTTTCCTCCTTGCCCTCCTTCCCTCCCAGCAACAGTGACCAGTCAATGCCTCATTAAA
AGGACCTCAAATGATGATTTCAGACTTACGGTCTGCTACTTCAGGAAGTGCTGCTGCTGATT
TGCCACTAGCTGAGAATGTTCTTTTGTCACCAGGAGGAGGCATTTACAAATTAAAAACAAAT
GTATTCGGACCACTGCCTAAAGGCACCTTTGGCTTAATATTAGGCCGTAGCAGTGCGGCTTT
GAGAGGTCTAACCATAATTCCTGGGGTAATAGACCCCGACTATGTTGGGGAAATCTTGATTA
TGGTTTCTACTTCTACCACGCTTTCATTATTAGCTGGGGAACGTATTGCTCAAATACTTCTCC
TACCTTATCATCCCTTTTTGGCTCTTCCTAATGAACGAACAGGAGGATTTGGAAGTACTGGGC
GACATATATTTTGGGAAATGCTTATCAAAGATTCTCGCCCTGTTCTCTCCTTAATTATACAAG
GAAAAAACTTTGAGGGACTAGTAGACACAGGGGCAGATGTTTCAGTTATTTCTTCTCAACAA
TGGCCCCAAGATTGGAAAAAAGAAAAAAGCCCTCTAATGCTGACGGGACTGGGCTCTATTG
CAGATGTCTGGAAGAGTACCCATCCCTTGCAATGCCAATTCCATAATGCAAGATCAGTGTCT
GTTACCTTTTATATTGTAAACATACCTATTACTATTTGGGGAAGAGATCTTCTCTCTCCATTA
GGGGCTTTTGTAACCATTCCACCGGAAAACTAGTAGCCACTGCTCAAATTCTTCGAGCACTC
CCATTAAAATGGTTAACTAATACTCCAAAATGGGTTGAGCAGTGGCCATTACCACAAATGAA
GCTCGAGGCGTTAGAACAATTAGTACAAGAACAACTCCAACTTGGTCATATAGAGCCCTCTA
CCTCCCCCTGGAATTCTCCTGTTTTTGTTATAAAAAAGAAATCTGGAAAATGGAGAATGTTA
ACCGATTTACGAGAAGTTAATAAATGTATTGAACCTATGGGAGCATTACAATTGGGACTCCC
CTCTCCAGCTCTTATTCCTCAGAATTGGTCCTTAATGGTGCTAGATCTTAAGGACTGTTTTTTT
GCCATTCCCCTACAATTACAAGATAGAGATAAATTTGCTTTTACAATTCCTGTTCTTAATCAT
GCTCAGCCTGTTAAGCGTTATCAATGGACAGTCTTACCACAGGGAATGACAAATAGTCCTAC
TTTATGTCAAGAATTCATAGCTTGCTCTTTACAATCCCTCCGTCAAGAATACCCCAATTATAT
TCTATATCAGTATATGGATGATCTCCTATTAGCAGCTCCTAGTATTGTCGAACGTGATGAATT
CTTTCTAAAAGTACAGGAGGCTTTAAGACTATACAATTTGCAAACAGCCCCAGAAAAAATTC
AAAAGGACTTTCCTATTTCGTATTTAGGGACAATATTGGAACAACATAGAATAATGCCCCAA

AAGTTGCAATTCAGAAGAGACCACCTCAAAACCTTAAATGATTTTCAAGTTATTGGGAGATA
TCAATTGGCTACGCCTGGTACTTGGGATTCCTACTTATCAATTACGACATTTGTTTTCTTCTTT
AGCGAGAGATAACAGCTCTGGATAGCCCCCGGACTTTAACCCCATTGGCTTTACAGGAACTT
CAATTTGTTGAGCAACGACTAAATGACGGCTTTTTTATTTACTTACATGCGTCTCAACCTATT
TTGTTTATAATATTTCATACCCCTTATTCTCCATCTGGTGTAATTGCTCAAGAAAAAGGATTA
ATAGAATGGATTTTCTTACCTAACAGCTTTTCAAAAAAAATGACTACATATATGGATAAATT
AGCCTTCCTTATACAGAAAGGTCGCCATCGCATTTTACGATTGTCAGGATGTGAACCACACC
AGATTGTTACTCGGTTAACAACTGCTCAAATATCTCGATGTTTACAATTTAATGAAAACTGG
CAAATTTCTCTTGCCTCATTTCCTGGTTCGTTTTCTAATCACTATCCATCATCTAAATTGATTG
ATTTTCTTCGGGCTAACTCTATGATATCTCAATCCCCAATTTCAGATGTTCCAGTTAAGGGAC
CCACTATTTTTACAGATGCAAATAAAAATACCACTGGATATTGGACCCTGGAAAGTTCCAAG
GTTCTCCCCCATTCATTTTCTTCTGTACAGCCCGCTGAATTGGGGGCTATCTATTTAGTTTTAC
AAGATTTTCCCCAACTTCCTATTAACATTGTTTCAGATTCTCGATATGCTGTTCTCTCTTGCTT
ACAGCTTCCCCATGTCTCCCTTCCACTGACCCTTAAAACAGCTATTGATAAATTGTTTTACCA
AGTACAACAATTGCTCTTGCAGCGTTCAGAGTTAATTTTCTTTACTCACATTCGTGCACATTC
CGCCCTTCCTGGACCCTTATCATTCGGAAATGCTACAATTGATGCCTTAATTTATCCTATAGA
AGCAGCAAAACAAGAACATCTCTTACAGCATACCAACTCCAAAGGGTTACAAAAATCTCAT
ACTATTACTCGAAAACAAGCTCAAAATATTGTTCGCTCTTGTTCCATATGTGCACCTTTTACT
TTGCCATTTACCCCACCAGGTGTCAACGTAAGAGGACAACAAGCAAATCAGATATGGCAAA
TGGATGTAATTTACGTACATCATACTATAGACACATGCACACGTTTTCAATGGACCACTGCA
TTACATTCTGAAAAGGCTGATGCTGTTATTACTCATTTGTTATCTTGTTTTGCAGTTATGGGA
TTACCAATTGAATTGAAAACTGATAATGCACCTGCCTACCAATCCGCGAAATTAGCTCACTT
TTTATCCCAATATCATATAACTCATACTTTTGGTATTCCTTATAATAGTTAAGAGCAAGCTAT
CATTGAAAGAGCTAATCGTACCTTGCGTGAATATCTTGAAAAAATAAAAAAAGGGGGAACA
GGAGAGATATATGAAACCTAAAGACATTTTGAATAAAACCTTACTTACCCTAAATTTTTTGA
ATATTTGGAGCAAGGGAAATCTATCAGCAGCAGAGTTGCATTTTCAAGGGAAAGAAGAGGA
TAAGAAGATCTTGAATATGCCTATTTGGTATAAAGATAAAGAGAAAGGTTGGATCCCAGCAT
CATTAATATATCTGGGACGAGGGTATGCTTTCATTTCTGTTAATAATTACAGGTTTTGGACCC
CAGCGAGATTGATCAAAATCGACAATGGCTGATCCCTTTGTTCAAAAATTCAAAGAGCTTAC
TATGCAGAGAAGCTTTACTTTTGGTACAAGGGAAGCAACACCTCCTACATGGGGTCAAATGA
AGAAGTTGACCCAGGAAGCAGAAAAGACGTTAATGAAAGCGGGACAGCCTCTGAATCCTAC
CAATCTTTTGCTTGCCATGATGGTGGTGGTAACATGTCAGGTAATCGGTGTCTCGGCAAGTA
ATCATACGTATTGGGCATATATACCTAATCCCCCATTAGTAAGAGTAGTTTCCTGAGGGGAA
CCAGAAGTGCAGGTATGCACTAATGAGACTGCCTTCTTTCCCCCACCAGCTTGCGGGGGAAC
AGAACAACTATCTCATCATAAACAACAGTATAATATTAGTAGTTTGACCATTGCAGCGGAAA
GTATTCCTTTGTGCATAGGAGGACACACTTTTGTCTGTCCGCTAAGGAACATTCTCATCATTC
CTATAATACATGGGGGGTAAAATATAATAATTATCATTTTGCTACTTTTACTATGCTTGTTTC
CACCAGGGGCTTTTAACACCTCGATAGAACCGCTAGACATTCATAATGGAATATATATGTCA
CTATGTCCTGTTAACTGTTTTGCTCCTTCTCTAGAATCTTTGGAATGGGAATGTTGCCAAGGT
CATCAACCCTTTAAAGTCATGAATTATTCTGGGTCTGTCATTGTAGATTGGAGTCCAGATCAT
GGGCAATTCTTAGAAAAATGGTCAAATAAGTCTCTTAGGTGGCATCGTGCAAATAGCACTTT
GATGGGCAATGGTAATGAAACAGTTAAATGGCAGCAGTTTGCACTTGTCCCTCCTCAATTAC
AATTGCAGGGATATCCACACATTCAAGGGGATATTTGGAAACTATGGGCAGTTTCTGGTAAT
CTCACTGTCTGGTCAGGAAACTATACTTTGGACAGTGGTGACTCTTCAGGTCCATTTCATGTT
AATTTACACGTTAATAAATCTTATTCCGCAATGGCATGTGTAAAATATCCTTTTGCATTGTTA
TATGGAAATTGGACCTGGAATGATACTGTGGGGTCTGTGTCATGTGACTACTGTAATCTAAC
TCAATGTGTAAATCAGTCTTGGTGGAAAGAATTTGAAAGACGAGTGTGTAACTCCAATTTTT

CGTTAGTAATTGTTAAAGCTTGGACAGAAGTATGGTTGCCTATAAATCTGACTAGACCATGG
TCAGATTCTTTTGCTGTTTCTCATCTAGTAACCGCTGTACAGACTTTGTTACATCGATCTCAA
CGTGTGCTTGGTGTGGTCATTACTTCGATTCTAACAGTCGCGTCAGTAACTGCAACAATGGC
GGTAGCAGGCCTCGCGTTACACCAAGGAATTCAAACAGCTGATTTTGTTCAGGACTGGCATA
AAGACTCTCATTTGTTATGGCAACAACAGCGAGATTTGGATGCACAACTTGCTACTGACGTG
CTCAATCTTCAACACACCGTTTCCTGGCTTGGAGATCAATTAGCTGTTTTATCTACACAAAGT
GTGTTGAAATGTGATTGGAATTCTTCTCAGTTTTGTATAACACCTGTACCATTTAACATGAGT
GAGGGATGGGAAAGAGTAAAACGATCTTTGACTGGACATCAAAATCTCACTACAGAAATTA
TGGACCTGGAACGACAAATTTTGTCTACTTTTAGCGGACTTTACCTGACATTGTGGGGTCTG
GTTTGTTGAAAAGTCTTCAAGAAGGATTGAATAACTTAAATCCATTAGGGCATGTATCCACA
CTAATTGGGACTACCTTTGGGAACACTATGTTTATATTACTTTTATGTTGTGTTGCTTTTCTAG
TCTTCCAACGATGGCGGAAAAGGAAACAACTAAAGCGTGAAGCAGAGAAGATCCAGACCAT
GCCACAATTTATTAAAGCAAATAAAAAAGGGGGAGATGAAGGGTTAATGCAGCCACAATAG
GGAAAGTGGAGATGTGCCTGCAAACGGGGCTCTCTGCTCGGGCTGAATGTACTTGCAAGTG
AGGCGTTCTGCCAAGGAGTCTGGACATAGCCTTGAGTTTGATGGTCCCTTGCAAACGAGGGA
ACATTCCCT

>NC_056074.1:24477179-24484832#SHEEP_RIP_28(-)

TGAAGGGTTAATAGGGTAGCAGAGATGTGCCTGCAAACGGGTCTCTCTGCTCAGGCTGAGG
GTCCTTGCATACGAGGCGTTCTGCCAAAGAGTCTGGATACAGCCTTGAGTTTAATGGTCCCT
TGCAAACGAGGGAGCATTCCCTTCTTGAGATAAGAGGGAGATGAGGGCTTTGTGCAGACTCT
GCAGTAGACAGAGATTTCACTCCCCTTTGCTGTACGATAACATGTATGCACCTGCACTGTAC
TGAAAAGGCTTATTCTTACAGGCTGGAATTCTGCCTAAGGGGGGCTTTATAATAATAAATGG
CAATTAGTTTGCCCAGTTCTGTTCCTCTGGCCAGAGTGGTGTCCTGTCTGTCTCTTGTGTGTCT
TGTATGTTCTGTGTCATTTCACTCGTAGTAACCAACATCTGGCGCCCAACGGATGGGGCTCG
AGTGAAACCGAAAGAGGTAAGAAACCCCGGGGGGATTTTAGATCAATAGCAGGGGAGCTTT
CGGAAACATGGGAATTCCTCACCCTAGCGGAAAAAATTGCTAGGTGAATTCTTGTACATAAT
CATGGGGAACTCTTCACCCTAGCAGATGGGAACTTTCGAAAGTTCATGGTTCAAAAAACCAT
GGGGAAATCCTGCTAGGAAGAAATAAATGTTCATCATGGGCACAATATGTGAATTTATATAT
TGAATCATGTTATATGTGAATTTATATATTGAATCATGTTATAGAATTTATGTAATGCTATAA
CACAGGCTTTGACTTTGCTATCTACTGATAATGAGACTAAATCTAATGCTTTAATGAAGGGA
GAGGCAATTTATGAGGATATGCCAAATGTTGGTGGAATTTCTGCCTTGCCTGAAAGTAAGGA
TACAAATAAGCCTCCTTCTGGAAATGGTGAAACATCTGATAGTTCAGAATCAGATTCGGAGG
CTTCTTCGGTTTCGTCAGAGGAGGGCAAAGAGATTAAAGAAATGACCCATCTATTCTGGGAA
TAGTGGAAATCCCGTAAGGAGGAGAAAAAATCTACACCTTCTGCTCCTGCTTGTGCTTCTCT
TCTCCCCACTGCAGTTGATCGGCCCGATGTGGGCAGGGGACATTGTCCGTTCTCCTTTCCTTT
GTCTATGCTTCATGATGATGACTTGTCTGCTCCCCCTGGTGGGTTTATTGATCCTCCACAATT
GTTTCCCATCCAGAGACAGCAGGAAGGCAATGTGATAAATGTTCAATATGCTCCTTTGGAAT
ATAAATTTTTTTAAGATCTTAAAGCTGCAATAGCGCAATACGGTCCTCAATCTCCCTTTGTTT
TTGCTATGCTGGAATCATTGGGAAAAGGCAAACTAATCATTCCATTAGATTGGGAATCCATT
GCCCAAGCTGTCTTGGAGGGTTCTCAATGGTTGCAACTTTGTAGCTGGTGGGAGGAAAAAGC
TAGGAAGCAAGCTCGGATTAATGAGGGACAAAATCCCCCTGGTCCTCTCGAGGACAAATTA
ATGGGAGAGGGCCAATATTGGGCTTTAAGAGAACAGGCTCAATACTCTGACCAGGACTTAC
AACAAGTTTGCCAGGTCTTTTTACGAGCATGGCGCCGTGTGGTGCCTACTGGCCAAGCCCAG
CCCTCCTTTGTTAAAACAATGCAAGGCCCTAATGAGCCATATACTGATTTTCTAGCAAGATT
GAGGGTAGCTGTGGAACAGGCTGTAGGGAGGGATGAAATCTCAGGGATATTATTACAAACT
TTGGCATTTGAAAATGCAAATCCTGAATGCAAGCGTATACTGGGACCTTTAAAGGGACAGG

GTGCCTCTATAGCTGAATATATCAGAGCCTGCTCGGGAGTAGGAGGAACTGAGCACCAGGC
TAATGTCTTTGCTACGGCCTTGGCCAAGGTTATGAGACCACCAAAGGGAGGTTACTGCTTTC
ATTGTGGAAAACCTGGTCATATAAAAAAAGAGTGTCAGAAATTAAAAGCTGATAAAGACAG
ATCTCTTGCTAGGAAGAATAAGGCTCCTCCTGGACTTTGCCGTCGGTGCAGGAGGGGATTTC
ACTGGACTAATGAATGCAAATCTAAAACAGACAAAATGGGCAACCCAACACCAGGAAACTG
TCCTGCGGGCCTGAGTCCTTGGGGCCCAGGAACAATACCGGGGGCTTCTCCTCCTTGCCCTC
CTCCCATTCCATCTGCCCCGTTAAAAAGACCTCAGACGATGATTTCGGACTTATGGTCTGCTA
CTTCAGGGAGTGCTGCTGCTGATTTGCCGCTAGCTGATAATGTTCTTTTGTCACCAGGGGGA
GGCATTTATAAATTAAAAACAAATGTATTTGTACCACTGCCTAAAGGCACTTTTGGCTTAAT
ATTAAGCCGTAGCAGCGCGGCTTTGAGAGGTCTAACCATAATTCCTGGGATAATAGACTAAT
TATGTTGGGGAAATTTTAATTATGGTCTCTACTTCTACCACACTTTCATTGTTAGCCAGAGAA
CATATTGCCCAAATACTTCTCCTACCTTATCACCCCCTTTTTGGCTCTTCCTAATAAACAAAC
AGGGGGATTTGGAAGTACTGGGTGAAATATATTTTGGGAAATGCTTATCAGAGATTCTCACC
CTGTTCTCCCCTTGATTATACAAGGAAACAACTTTGAGGGACTAGTAGATACAGGGGCAGAT
GTTTCAATCATTTCTTCTCAGCAATGGCCCCAAGGTTGGGAAAAGGAAAAAAGAGCCCTTTA
ATGCTGATGGGATTGGGCTCCATTGCAGATATTTGGAAGAGTACCCATCCCTTGCAATGTCA
ATTCCATAATGGAAGATCAGTGTTTGTTACCTTTTATATTGTAAATATACCTATTAATATATG
GGGGAGAGATCTCCTCTCTTCTTTGGGGGCTTCTGTAACCATTCCATCGGAATACTAGTGGCC
ACTGCTCAAATTCCTTGAGCACTCCCATTAAAATGGTTAACTAATACTCCTAAATGGGTTGA
GCAGTGGCCATTACCACAAATGAAGCTCGAGGCATTAGAACAAGTAGTACAAGAACAACTC
CAACTTGGTCATATAGAGCCCTCTACCTCACCCTAAAATTATCTTGTTTTTGTTATTTTAAAA
AATCTAAAAAATAAAAAATGTTAACTGATTTACAAGAAGTTAATAAATGTATTGAACCTATG
GGAGCATTGCAATTGGGACTCCCCTCTCCAGCTCTTATTCCTCAAAATTGGTCCTTAATGGTG
CTAGATCTTAAAGACTGTTTTTTTACCATTCCCCTACAATTTCAAGATAGAAATAAATTTGCT
TTTACAGTTCCTGTTCTTAATCATGCTCAACCTGTTAAGCGTTATCAATGGACAGTCCTACTG
CAAGAAATGATAAATAGTCCTACCTTATGTCAAGAATTTGTAGCTCACTCTTTGCAATCTCTC
CGTCAATAATACCCTAATTATATTCTATATCATTATATGGATGATCTCCTATTGGCAGCTCCT
AGTATTGCTGAACGTGACTAATTCTTTTTAAAAGTACAAGAGGCTTTAAGACTACACAATTT
GCAAATAGCCCCAGAAAAAATTCAAAAGGACTTTCCTATTTCATATTTAAGGACAATATTGG
AACAACACAAAATTAAGCCCCAAAAGTCACAAATTAAAAGAGACCATCTCAGAACCTTAAA
TGATTTTCAAAAATTATTGAGAGATATTAATTGGCTACACCCGGTACTTGGGATTCCTACTTA
TCAGTTACGACATTTGTTTTCTACTTTAAAAGGAGATACAGCTTTGGATAGCCCCCGGACCTT
AATCCCATTGGCTTTACAGGAACTTCAACTTGTTGAACAACGACTGAATGACGGCTTTTTGA
CTTACTTACATGCATCTCAACCTATTTCTTTTATCATATTTCATACCCCTTATTCCCCTTCTGG
TGTAATTGCTCAAGAAAAAGGATTAATAAAATGGGTTTTCTTACCTAACAGTTTTTCCAAAA
AATTGACAATATATATGGATAAATTAGCCTTCCTTATACAAAAAATCACCATCATATTTTAC
AATTATCAAGAATGTGAACCACACCAGATTGTTACTCAGTTAACAACTGCTCAAATATCTCG
ATGTTTACAATTTAATGAAAACTGGCAAATTTCTCTTGCTTCATATCCTGGTTCGTTTTCTAAT
CATTATCCATCATCTAAATTGATTGATTTTCTCCAGACTAACACTATGATATCTCATTCCCCA
ATTTCAGATGTTCCAGTTAAAGGACCCACTATTTTTACAGATGCAAATAAAACTACTGCTGG
ATATTGGACCCCAGAAAATTCCAAGGTTCTCCCCCACTCATTTTCTTCTGTACAACCTGCTGA
ATTGTGGGCTATCTATTTAGTTTTACAAGATTTTCCCCAACTTCCCATTAACATTGTTTCAGAT
TCTCGATATGCTGTTCTCTCTTGCCTACAGCTTCCTCATGTCTCCCTTCCACTGACTCTTAAAA
CAGCTATTGATAAATTGTTTTACCAAGTACAACAATTACTCTTGCAACTTTCAGAGTTAATTT
TCTTTACTCACATCTGTTCACATTCTGCCCTTCCTGGACCATTATCATTCGGAATGCTACAATT
GATGCCTTACTTAATCCTATACAAGCAGCAAAGCAAGAACATCTCTTACAACATACCAACTC
CAAAGCGTTAGAAAAATCTCACGCTATTACTCGAAAACAAGCTCAAAATATTGTTCGTTCTT

GTTCCATATGTGCACCCTTTGCTTTGCCATTTACCTCACCAGGTGTCAACATGAGAGGACAAC
AAGCAAATCAGATATGGCAAATGGATGTAATTTACATTTCTTCCTTCAGACAACAAAAATGT
GTGGGCCACTGCATTACATTCTAAAAAGGCTGACGCTGTTATTACTCATTTGTTATCTTGTTT
TGCAGTTATGGGATTACCAATTGAATTAAAAACTGATAATGCACCTGCTTACCAATCCACGA
AATTAGCTCACTTTTTATCCCAATACCATATAACTCATACTTTTGGTATTCCTTATAATAGTC
AAGGGCAAGCTATTATTGAAAGAGCTAATCGTACATTGCATGATTATCTTAAAAAAATAAAA
AAGGGGGAACAAGGGAGATTTATGAAACCTAAAGACATTCTGAATAAAACCTTACTTACCC
TAAATTTTTTGAACGTTTGGAGCAGGGGAAATCTATCAGCAGCAAAGTTGCACTTTCAAGGG
AAAGAAGAGGATAAAAAGATCTTGAATACGCCCATTTGGTATAAAGATAAAGAGAAAGGCT
GGATCCCAGCATCATTAATATATTTGGGGCAAGGGTATGCTTTCATTTCTGTTGATAATTACA
GGTTTTAGACCCCAACAAGATTGATCAAAATCAGCAATGGTTAATCAATCCTCTTGTTCAAA
AATTTGAAGAGCTTACTATGCAGAGAAGCCTTACCTCCCGTACAAGGGAAGCAACACCTCCT
ACGTGGGGTCAAATGAAGAGGTTGACCCAGGAAGCAGAAAAGACATTAATGAAGGTGGGG
CAACCTCTAAATCCTACCAATCTTTTGCTTGCCACGATGGCGGTGGTGACATGTCAGGTAAT
CAGCGTATCGGCAAGTAATCATACATATTGGGCATATATACCTAATCCCCCATTAGTAAGAG
CAGTTTCCTGGGGGGAGCCAGAAGTGCAGGTATGTACTATTGAGACTGCCTTCTTTCCCCTG
CCAGCTTGCAGGGGAATAGAACAACTATCTCATCATAAACAATAATATAATATTAATAATTT
GACCACTGCAATGGAAGATATTCCTTTATATATAGGGGGACAACCCCTTTTGTCTGTCCACC
AAGGAATATTCTCATCATTCTTATAATACATGGGGGGTAAAGTATAATAATTACCATTTTGCT
ACTTTTACTGTGCTTGTTTCCACCAGGATTTAGTACCTGGACAGAACCGATGGATACTCATAA
TGGAAAACACATGTCACTATGTCCTATTAACCTTTTTGTTCCTTCTCTAGAGTCTTTGGAGTG
GGAACGTTGCCGAGGCCATCGACCCTTTAAGGTCATGAATTATTCTGGGGCCATCATTGTAG
ATTGGAGTCCAGATCATGGACAATTCTTATAAAAATGGTCAAATAAACCTTTTAGGTGGCAT
CGCGCAAATACCACTTTGATGGGCAACGGTAACGAAACAGTTAAATGGCAGCAATTTGCAC
TTGTCCCTCCTCAATTACAATTGCAAGGATATCCGCACATTCAAGGAGATATTTGGAAACTA
TGGGCGGTTTCTGGTAATCACACTATCTGGTCAGGAAATTATACTTTGGACAGTGGTGACTC
TTTGGGTCCATTCCATGTTAATTTACATGTTAATAAATCTTATTCCGCAATTGCATGTGTAAA
ATATCCTTTTACATTGTTATATGGGAATTGGACCTAAAATGATACTGTGGGGTCTATGTCAAG
TAACTATTGTAATTTAACTCAATGTATAAATGTCTTGGTGGGAAAAATTTAAAAGACAAGCC
TTTAACTCCAATTTCTCGCTAGTAATTTTTAAAGCTCGGACAGAAGTATGGTTGTCTATAAAT
CTGACTCAGCCGTGGTCAGATCCTTTTGCTGCTTCTCATCTAGTAACCGCTGTACAGACTTTG
CTACACCGATCTCGACGTATGCTTGCTGTGGTCATTGCTTCGATTCTCACAGTCGCATCAGTA
ACTGCAACAGCAGCTGTAGCAGGCCTTGCGTTACACCAAGGAATTCAAACAGCTGATTTTGT
TTGGGACTGGCAAAAGGACTCTCGTTTGTTATGGCAACAACAGCGAGATTTGGATGCACAAC
TTGCTACCGACATGCTTAATCTTCAAACACACCGTTTCCTGGCATGGAGATCAATTGGCTGTT
TTATCTACACGAAGTGTGTTAAAATGTGATTGGAATTCTTCTGTTTTGTATAACATCTGTACC
ATTTAACATGAGCCCTTCAAGAGATTAACCGAGACATATAAATAAAGAGTTCTAGATGCCCA
TCATCTAGAGAGATTCCGCCTAGCCCCTAAGAGGTGGCTGGATAGCCAATGACGGGTAAGA
CCCTCAGAGGAGAGCAACCTAAGACAGGCACAGCCGCAAGAGGGGCTAGCGAGGCTGGAG
GTTGGCTGCCTACAGCTTTATGACTTGCTCTACAAAATATCAATACAAATGTCTCGAGGACTT
GAATAAAATAAAAAAGGGGGAGATGAAGGGTTAATAGGGTTGCAGAGATGTGCCTGCAAA
CGGGTCTCTCTGCTCAGGCTGAGCGTCCTTGCATACGAGGCGTTCTGCCAAAGAGTCTGGAT
ACAGCCTTGAGTTTAATGGTCCCTTGCAAACGAGGGAGCATTCCCTTCTTGAGATAAGAGGG
AGATGAGGGCTTTGTGCAGACTCTGCAGTAGACAGAGATTTCACTCCCCTTTGCTGTACGAT
AACATGTATGCACCTGCACTGTACTGAAAAGGCTTATTCTTACAGTCTGGAATTCTGCCTAA
GGAGGGCTTTATAATAATAAATGGCAATTAGTTTGCCCAGTTCTGTTCCTCTGGCCAGAGTG
GTGTCCTGTCTGTCTCTTGTGTGTCTTGTATGTTCTGTGTCATTTCACTCGTAGTAACCAACA

>NC_056074.1:26563070-26570826#SHEEP_RIP_29(+)

CTTGAGATAAGAAGGAGATGAGGGCTTTGTGCAGACTCTGCAGTAGACAGAGATTTCACTCC
CCTTTGCTGTACGATATCATGTATGCACCTGCACTGTACTGAAAAGGCTTATTCTTACAGTCT
GGAATTCTGCCTAAGGAGGGCTTTATAATAATAAACGGCAATTAGTTTGCCCAGTCCTGTTC
CTCTGGCCAGAGTGGTGTCCTGTCTGTCTCTCGTGCGTCTTGTATGTTCTGTGTCATCTCACTC
GTAATCACCAGTGATCTACATCTGGCGCCCAACGCGGGGCTCGAGTGAAACCGAAAGGGTG
AGTCCCCCCGGGGGGATTTTAAATCCATAGCAGGGGAACTTTCGGAAAATTATGGGGAATTC
CTCACCCTAGTGGAGGGAAACTTTTAAAAAAATCATGGGGAATTCCTCATCATCGTTATGGG
CACAATACATGGAGTTAGTCCAAGGACTTCTCCACTCCATAGGCGTTAAGGCCTCAGCTCGT
CGGTTGAGTGAGCTCTTTCGCTTGGTGGAGAAATACTGTCATTGGTTTCAATATCAAACTAA
GTTACAGTTAAACTTGAAGGAATGGAAAATAATTCAAAAAGAATTGAGAAAGCAACATCAG
AAGGGTAATGTGATCCCTTTAAAACTGTGGACTTTATGTAGTGCTATAACACAGGCTTTGAC
TTTACTCTCTACTGATAATAAAACTAAATCGAATGCTTCAAGGAGGGGAGAAATAGTTTATG
AGGATGTGTCAGACATTAATGGGGCTTCTGCATTGCCTGAGGGCAAGGATACAAATGAGCCT
CCTCCTGTAAGTGGTGAAACATCGGATAGTTCAAAATCAGATTCGGAGGCTTCTTCGGTTTT
GTCAGAGGAGGGCAAAGAGATTGAAGAAATGACCCATCTATTTCAGGAGTGGTGGAAATCC
CGTAAAGAGGAAAAAAATCTACACCTTCTGCTCCTGCTTGTGCTTCTCTTCTCCCCACTGCGG
TTGATCGCCCGATGTGGGCAGGGGACATTGTCCCTTCTCCTTTCCTTTGTCTATGCTTCATGA
TGATGACTTGTCTGCTCCCCCTGGTGGGTTTATTGATCCTCCACAATTGTTTCCCATCCAGAG
ACAGCAAGATGGCAATGTGATAAATGTTCAATATGTTCCTTTGGAATATAAATTTTTTAAAG
ATCTTAAAGCTGCAGTAGCGCAATACGGTCCTCAATCTCCCTTTGTTTTGGCTATGCTAAAAT
CATTGGGAAAAGGCAAACTAATCCTTCCGTTAGATTGGGAATCCATTGCCCAAGCTATCTTG
GCGGGTTCTCTATGGTTGCAACTTCGTAGCTGGTGGGAGAAAGAAGCTAGAAAGCAAGCTC
AGATTAATGAGGGACAAAATCCCCCTGGTCCTCTTGAAGACAAATTAATGGGAGAGCGCCA
ATATTGGGCTTTAAGAGAACAGGCTCAATACTCTGACCAGGACTTACAACAAGTCCGCCAAG
TCTTTCTACGAGCATGGCGCCGTGTGGTGCCTACTGGCCAAGCCCAGCCCTCCTTTGTTAAAA
CAATGCAAGGCCCTAATGAGCCATATACTGATTTTCTACAAGATTGAGGGTAGCTATAGAAC
GGGCTGTAGAGAGGGATGAAATCTCAGGGATATTATTACAAACTTTGGCATTTAAAAATGCA
AATCTTGAATGCAAGCGTATACTGGGACCTTTAAAGGGACAGGGTGCCTCTATAGCTGAATA
TATTAGAGCCTGCTCGGGAGTAGGAGGAACTGAACACCAGGCTAATGTCTTTGCTACGGCCT
TGGCCAAGGCTATGAGACCACCAAAGGGAGGTAACTGCTTTCATTGTGGAAAACCTGGTCAT
ATGAAAAAAGAGTGTCAAAAATTAAAAGCTGATAAAGACAGATCCCTTGCTAAAAAGAATA
AGGCTCCTCCTGGACTTTGCCGTCGGTGCAGGAGGGGATTTCATTGGACTAATGAATGCAAA
TCTAAAACAGACAAAATGGGCAACCCGACACCGGGAAACTGTCCTGCGGACCTGAGTCCTT
GGGGCCCAGGAACAATACCAGGGACTTCTCCTCCTTGCCCTCCTCCCATTCCATGTGCCCCA
ACCCTGTTCCCTCCCAGCAACCTTTACGAGTCGATGCCCCGTTAAAAGGACCTCAGACGATG
ATTTCGGACTTGCGGTCTGCTACTTCAGGGAGTGCTGCTGCTGATTTGCCGCTAGCTGATAAT
GTTCTTTTGTCACCAGGGGGAGGCATTTACAAATTAAAAACAAATGTATTTGGACCACTGCC
TAAAGGCACTTTTGGCTTGATATTAGGCCGTAGCAGCGCGGCTTTGAGAGGTCTAACCATAA
TTCCTGGGGTAATAGACTCTGACTATGTTGGAAAAATTTTAATTATGGTCTCTACTTCTACCA
CACTTTCATTGATAGCTGGGGAACGTATTGCTCAAATACTTCTCCTACCTATCACCCCTTTTT
GGCTCTTCCTAATGAACGAACAGGAGGATCTGGAAGTACTGGGCGACATATATTTTGGAAA
ATGCTTATTAAAGATTCTCGCCCTGTTCTCTCTTTGATTATACAAAAACACAACTTTGAGGGA
CTAGTAGATACAGGGGCAGATGTTTCAATCATTTCTTCTCAGCAATAGCCCCAAGATTGGAA
AAAGGAAAAAAGCCCTTTAATGCTGACAGGATTGGGCTCCATTGCAGATGTTTAAAAGAGT
ACCCATCCCTTGCAATGTCAATTCCATAATGAAAGATCAGTGTTTGTTACCTTTTATATTATA

AATATACCTATTAATATATGGGGGAGAGATCTCCTCTCTCCTTTGGGGGCTTCTGTAACCATT
CCATCGAAAAACTAGTGGCCACTGCTCAAATTCCTCGGGCACTCCCATTAAAATGGTTAACT
AATACTCCTAAATGGGTTGAGCAGTGGCCATTACCACAAATGAAGCTCGAGGCATTAGAAC
AAGTAGTACAAAAACAACTCCAACTTGGTCATATAGAGCCCTCTACCTCACCCTGGAATTCT
CCTGTTTTTGTTATAAAAAAAAAATCTGGAAAATGGAAAATGTTAACTGATTTACAAAAAGT
TAATAAATGTATTAAACCTATGGAAGCATTGCAATTGGGACTCCCCTCTCCAGCTCTTATTCC
TCAAAATTGGTCCTTAACGGTGTTAGATCTTAAAGACTGTTTTTTACCATTCCCCTACAATT
GCAAGATAAAAATAAATTTGCTTTTACAGTTCCTGTTCTTAATCATGCTCAACCTGTTAAGCG
TTATCAATGGACAGTCCTACCACAAGAAATGATAAATAGTCCTACCTTATATCAAAAATTTG
TGGCTCGCTCTTTACAATCTCTCCGTCAAAAATACCCTAATTATATTCTATATCATTATATAG
ATGATCTCTTATTGGCAGCTCCTAGTATTGCTGAACGTGACGAATTCTTTTTAAAAGTGCAAG
AGGCTTTAAGACTATACAATTTGCAAATAGCCCCCCCAAAAATTCAAAAGGACTTTCCTATT
TCATATTTAGAGACAATATTAAAACAACATAGAGTTAAGCCCCAAAAGTTACAAATTAGAA
GAGACCAGCTCAAAACCTTAAATGATTTTCAAAAATTATTGAGAGATATTAATTGGCTACGC
CGGTACTTGAGATTCCTACTTATCAGTTACGACATTTGTTTTCTACTTTAAAGGAGATACAG
CTTTAGATAGCCCCCGGACCTTAACCCCATTGGCTTTACAGGAACTTCAATTTGTTAAATAAC
GACTAAATGACGGTTTTTTGACTTACTTACATGCATCTCAACCTATTTTTTTTATCATATTTCA
TACCCCTTATTCCCCATCTGGTGTAATTGCTCAAAAAAAAAAAAAGATTAATAAAATGGGTTT
TTTTACCCAACAGTCTTTCCAAAAAAATTAACTATATATAGAGATAAATTAGCCTTCCTTATAC
AAAAAGGTCGCTATCGTATTTTACAGTTATCAAGAAGTAAACCACACCAGATTGTTACTCAG
TTAACAACTGCTCAAATATCTCGATGTTTACAATTTAATGAAAGCTGGCAAATTTCTCTTGCT
TCATATCCTGGTTCGTTTTCTAATCATTATCCATCATCTAAATTGATTGGTTTTCTCCGAACTA
ACACTATGATATCTCATTCCCCAATTTCAGATGTTCCAGTTAAAGGACCCACTATTTTTACAG
ATGCAAATAAAAATACTGCTGGATATTGGACCCCCCAAAATTCCAAGGTTCTGCCCCACTCA
TTTTCTTCTGTACAGCCTGCTAAATTGTGGGCTATCTATTTAGTTTTACAAGATTTTCCCCAAA
TTCCTATTAACATTGTTTCAGATTCTCGATATGCTGTTCTCTCTTGCCTACAGCTTCCTCATGT
CTCCCTTCCACTGACACCTAAAACAGCTATTGATAAATTGTTTTACCAAGTACAATTACTCTT
ACAACGTTCAGAGTTAATTTTCTTTACTTACATCTGTGCACATTCTGCCCTTCCTAGACCCTT
ATCACTCGAAAATGCTACAATTGATGCTTTACTTTATCCTATAAAAGCAGCAAAACAAAAAC
ATCTATTACAACATACCAACTCCAAAGGGTTACAAAAATCTCATGCTATTACTCAAAAACAA
GCTCAAAATATTGTTCGTTTTTGTTCCATATGTGCACCTTTTGCTTTACCATTTACCCCACCAG
GTGTCAACATAAAAAGACTGCAAGCAAATCAGATATGGCAAATAGATGTAATTTACATTTCT
TCCTTCGGACAACAAAAATGTGTGCATCATACTATAGATACTTGCACACATTTTCAATGGGC
CACCGCATTACATTCTAAAAAGGCTGACGCTATAATTACTCATTTGTTATCTTGTTTTGCAGT
TATGAGATTACCAATTAAATTAAAAACTGATAATGCACCTGCTTACCAATCATCAAAATTAG
CTCACTTTTTATCTCAATACCATATAACTCATACTTTTGGTATTCCTTATAATAGTCAAGGGC
AAGCTATTATTAAAAGAGCTAATCACACCTTGCGTGACTATCTTAAAAAAATAAAAAAGGG
GGAACAAGAGAGATTTATGAAACCTAAAGACATTCTGAATAAAACTCTACTTACCCTAAATT
TTTTGAATGTTTGGAGCAGGGGAAATCTATCAGCAGCAGAGTTGCACTTCCAAGGGAAAGA
AGAAGAAGAAGAAGATAAGAAGATCTTGAATACGCCCATTTGGTATAAAGATAAAGAGAA
AGGCTGGATCCCAGCATTATTAATATATTTGGGACGAGGGTATGCTTTCATTTCTGTTAATAA
TTACAGGTTTTGGACCCCAGCAAGATTGATCAAAATCAACAATGGCTGATCCCCTTGTTCAG
AAATTTGAAGAGCTTACTATGCAGAGAAGCCTTACCTCCCGTACAAGAGAAGCAACACCTCC
TACGTGGGGTCAAATGAAGAGGTTGACCCAGGAAGCAGAGAAGACATTAATGAAGGCGGG
GCAACCTCTGAATCCTACCAATCTTTTGCTTGCCACGATGGCGGTGGTGACATGTCAGGTAA
TCGGCATATTGGCAAGTAATCATACATATTGGGCATATATACCTAATCCCCCATTAGTAAGA
GCAGTTTCCTGGGGGAGCCAGAAGTGCAGGTATGTACTAGTGAGACTGCCTTCTTTCCCCCG

CCAGCTTGCGGGGGAATAGAACAACTATCTCATCGTAAACAACAATATAATATTAGTAATTT
GACCATTGCCGTGGAAAGTATTCCTTTATGTATAGGGGGACACCCCTTTTGTCTGTCCACCAA
GGAACATTCTCATCATTCTTATAATACATGGGGGGTAAAGTATAATAATTACCATTTTGCTGC
TTTTACTGTGCTTGTTTCCACCAGGGGATTTAGTACCTCGACAGAACCGCTGGATACTCATAA
TGGAAAACACATGTCGCTATGTCCCGTTAACTTTTTTGTTCCTTCTCTAGAATCTTTGGAATG
GGAACGTTGCCGAGGTCATCAACCCTTTAAGGTCATGAATTATTCTGGGGCCATCATTGTAG
ATTGGAGTCCAGATCATGGGCAATTCTTAGAAAAATGGTCAGATAAATCTCTTAGGTGGCAT
CGTGCAAATGGCACTTTAATGGGCAATGGTAATGAAACAGTTAAATGGCAGCAATTTGCACT
TGTCCCTCCTCAATTACAATTGCAAGGATATCCGCACATTCAAGGAGATATTTGGAAACTAT
GGGTGGTTTCTGGTAATCTCACTATCTGGTCAGGAAACTATACTTTAGACAGTGGTGACTCTT
CGGGTCCATTCCATGTTAATTTACATGTTAATAAATCTTATTCTGCAATGGCATGTGTAAAAT
ATCCTTTTGCATTGTTATATGGGAATTGGATCTGGAATGATACTATGGGGTCTATATCATGTG
ACTATTGTAATTTAATTCAATGTGTAAATCAGTCTTGGTGGGAAAAATTTGAAAGACGAGCC
TTTAATTCCAATTTCTTGCTAGTAATTGTTAAGGCTCAGATAGAAGTATGGTTACCTATAAAT
CTGACTCGGCCGTGGTCAGATCCTTTTGCTGTTTCTCATCTAGTAACCGCTGTACAGACTTTG
CTACACTGGTCTCGACGTATGTTTGGTGTAGTCATTGCTTCGATTCTCGCAGTCGAGTCAGTA
ACTGCAACAGCAGCAGTAGCAGGTCTTGCGTTACACCAAGGAATTCAAACAGCTGATTTTAT
TTGGGACTGGCATAAGGACTCTCATTTGTTATGGCAACAACAGCGAGATTTGGATGCACAAC
TTGCTACCGACGTGCTCAATCTTCAACACACCGTTTCCTGGCTTGGAGATCAATTGGCTGTTT
TATCTACACGGAGTGTGTTGAAATGTGATTGGAATTCTTCTCAGTTTTGTATAACACCTGTAC
CATTTAACATGAGTGAAGGTTGGGACAAAGTAAAACGATCCTTAACTGGGCATCAAAATCTC
ACTACAGAGATTATAAACTTGAAACAACAAGTTTTATCCTCTTTTAGCAGGACTTTACCTGA
CATTACAGGATCTGATTTGCTAAAAGGTCTTCAAGAGAGAATGAATAATTTGAATCCATTAG
GGCATGTATCCTCACTAGTTGGGACTACTTTTGGAAACACTATGCTTATATTACTTTTATGTT
GTGTTGCTTTTCTAGTCTTCTGGCGATGGCAGAAAGGGAAACAACAAAAGCATGAAGCAGA
AAAGATCCAGACCATGCTACAATTTATAAAAGCAAATAAAAAAGGGGGAGATGAAGGGTTA
ATAGGGTTGCAGAGATGTGCCTGCAAACGGGTCTCTCTGCTCAGGCTGAGCGTCCTTGCATA
CGAGGCGTTCTGCCAAAGAGTCTGGACACAGCCTTGAGTTTAATGGTCCCTTGCAAACGAGG
GAGCATTCCCTTGAGATAAGAAAGAGATGAGGGCTTTGTGCAGACTCTGCAGTAGACAGAG
ATTTCACTCCCCTTTGCTGTACGATATCATGTATGCACCTGCACTGTACTGAAAAGGCTTATT
CTTACAGTCTGGAATTCTGCCTAAGGGGGGCTTTATAATAATAAATGGCAATTAGTTTGCCC
AGTCCTGTTCCTCTGGCCAGAGTGGTGTCCTGTCTGTCTCTCGTGTGTCTTGTATGTTCTGTGT
CATTTCACTCGTAATCACCAGTGATCAACACCT

>NC_056080.1:58034375-58042187#SHEEP_RIP_30(-)

AGGCATTCTGCCAAAGAGTCTGGACACAGCCTTGAGTCTAATGGTCCCTTGCAAACGAGGGA
GCATCCCCTTCTTGTGATAAGAAGGAAAAGAGGGCTTTGGACAGACTCTGCGGTAGACAGA
GATTTCACTCCCCTTTGCTGTACGATAACATGTATGCACCTGCGCTGTACTGAAAAGGCTTAT
TCATGCAGTCTGGAATTCTGCCTAGGGGGCTTTTATAATAATAAAACCGCAATTAGTTTGCG
CAGTTCTGTTCCTCCGGCCGGAGTGTGTATTGTCTGTCTCTTGTGTGTCTCGTGTTTTGTCTTT
GTGTCATTTCACTCGCAACATCTGGCGCCCAACATGGGGCTCGAGTGAAACTGAAAGGGTGA
ATAACCCCAGGGGATTTTAAATCCATAGCAGGGGAACTTTCAGGCAAATCATGGGGAATTCC
TCACCCTAGCAGGGGAACTTTCAGAAAATCATGGGGAATTCCTCATCATCATTACGAACATA
ATACATGGAGTTAGTCAAAGGACTTCTCCACTCCATAGGTGTTAAAGCCTCGACTCATCAAT
TGAGTGAGCTCTTTCGCTTGGTAGAGCAATATTGTCATTGGTTTCAATATCAAACTAAGTTAC
AGTTAAACTTGAAGGAATGGAAAATAATTCAAAAGGAATTGAGAAAGCAACATCAGAAGG
GTAATGTGATCCCATTGAAGTTATGGACTTTGTGTAGTGCTATAACACAGGCTTTGACCTTGC

TCTCTATTGATAATGTAACTAAATCTAATGCTTCAAGGAGGGGAGAAATAATTTATGAGGAT
GTGTCAGACCTTGGTGGGGCTTCTGCATTGCCTGAAGGCAAGGATACAAGTGAGCCTCCTCC
TGTAAATGGTGAAACATCTGATAGTTCAGAATCAGATTTGGAGGCTTCTTCGGTTTCATCAG
AGGAGGGCAAAGAGATTAAAGAAATGACCCATCTATTCCAGGAATGGTGGAAATCCCGTAA
GGAGGAGAAAAAATCTACACCTTCTGCTCCTCCTTGTGCTTCTCTTTTCCCCACTGCAGTTAA
TCGGCTCGATGTGGGCAGGGAACATTGTCGGTTCTCCTTTCCTTTGTCTATGCTTCATCATGA
TGACTTGCCTGCTCCCCCTGGTGGGTTTATCGATCCTCCACAATTATTTCCCATCCAGAGACA
GCAAAATGACAATGTGATAAATGTTCAATACACTCCTTTGGAATATAAATTTTTTTTAAAGA
TCTTAAAGCTGCAGTAGTGCAGTACGGTCCTCAATCTCCCTTTGTTTTGGCTATGCTGGAATC
ACTGGGAAAAGGCAAATTGATCATTCCGTTAGATTGGGAATCTATTGCCCAAGCTGTCTTGG
AGGGTACTCAATGGTTGCAACTTCGTAGCTGGTGGGAAGAAGAAGCTAGAAAGCAGGCTCG
GATTAATGAAAGACAGAATCCCCCTGGTCCTCTCGAGGACAAGCTAATGGGAGAGGGCCCT
TATCAGGCTTTAAGAGAATAGGCTCAGTACTCTGATCAGGACTTACAACAAGTCCACCAGGT
CTTTTTACTAGCATGGCACCGTGTGGTGCCTACTAGCCACACCCAGCCCTCCTTTGTTAAAAC
AATGCAAGGCCCCAATGAGCCATATACTGATTTTCTAGCAAGATTGAGGGTAGCTGTGGAAT
CGGCTTTAGGGACGGATGAGATTTCAGAGATAGTATTACAAACTTTAGCATTTGAAAATGCA
AATCCTGAATGCAAGCATATACTGGGATCTTTAAAGGGACAGGGTGCATCTATAGCTGAATA
TATCAGAGCCTGCTCGGGAGTAGGAGGAGCTGAGCATCAGGCTAATGTCTTTGCTACAGCCT
TGGCCAAAGCTATGAGACCACAAAAGGGAGGTAACTGCTTCCATTGTGGAAAACCTGGTCA
TATGAAAAGAGACTGTCAGAAATTAAAAGCTGATCAAGGTGCAATTCCTAAAGACAGATCT
CTTGCTGGGAAGAATAAGACTCCTCCTGGACTTTGCCATCGATGCGGGAAGGGGTTTCATTG
GACTAATGAATGTAGATCTAAAACAGACAAAATGGGCAACCCGATACCGGGAAACTATCCT
GCGGGCCTAAGTCCTTGGGGCCCAGGAACAATACCGGGGACTTCTCCTCCTTGCCCTCTGCC
ATCCCACCTGCCCCAACCCTCTTCCCTCCCAGCAACCGTTACAAGTCGATGCCCCATTAAAA
GGACCTCAAATGATGATTTCAGACTTTCGGTCTGCTACTTCAGGGAGTGCTGCTGCTGATTTG
CCACTAGCTGATAATGTTCTTTTGTCACCAGGGGGAGGCATTTATAAATTAAAAACAAATGT
ATTTGGACCACTGCCTAAGGGCACTTTTGGCTTGATATTAGGCCGTAGCAGCGTGGCTTTGA
GAGGTTTAACCATAATTCCTGGGGTAATAGACTCTGACTATGTTGGGGAAATTTTAATTATG
GTCTCTACTTCTACCACGCTTTCACTGTTAGCTGGTGAACGTATCGCTCAAATACTTCTCCTA
CTTTATCACCCCCTTTTGGCTCTTCCTAATGAATGAACAGGAGGATTTGGAAGTACTGGGCTG
CATATATTTTGGGAAATGCTTATCAAAGATTCTCGCCCTGTTCTCTCCTTGATTATACAAGGA
AACAACTTTGAGGGACTAGTCGACACAGGGGCGGATGTTTCAGTCATTTCTTCTCAACAATG
GCCCCAAGATTGGGAAAAAGAAAAAAAGCCATTTAATGCTGACGGGATTGGGCTCCATTGC
AGATGTCTGGAAGAGTACCCATCCCTTGCAATGTCAATTCCATAATGGAAGATCAGTGTTTG
TTACCTTTTATATTGTAAATATACCTATTAATATTTGGGGAAGAGATCTTCTCTCTACTTTGG
GGGCTTCTGTAACCATTCCATCGGAAAACTAGTAGCCACTGCTCAAATTCCTCAAGCACTCC
CATTAAAATGGTTAACTAATACTCCAAAACGGGTTGAGCAGTGGCCATTACCACAAATGAA
GCTCGAGGCCTTAGAACAATTAGTAAAAGAACAGCTCCAACTTGGTCATATAGAGCCCTCTA
CCTCCCCCTGGAATTCTCCTGTTTTTGTTATAAAAAAGAAATCTGGAAAATGGAGAATGGTA
ACTGATTTACGAGAAGTTAATAAATGTATTGAACCTATGGGAGCATTACAATTGGGACTCCC
CTCTCCAGCTCTTATTCCTCAGAATTGGTCCTTAATGGTGCTAGATCTTAAAGAATGTTTTTTT
TACCATCCCCCTACAATTGCAAGATAGAGATAAATTTGCTTTTACAGTTCCTGTTCTTAATCA
TGCTCAGCCTGTTAAGCATTATCAATGGACAGTCTTACCACAAGGAATGATAAATAGTCCTA
CCTTATGCCAAGAATTCGTAGCTCACTCTTTACAATCCCTCCATCAAGTATACCCCAATTATA
TTCTATATCATTATATGGATGATCTCCTATTAGCAGCTCCTAGTATTGCTGAACGTGATGAAT
TCTTTTTAAAAGTACAGGAGGCTTTAAGACTATACAATTTGCAAATAGCCCCAGAAAAAATT
CAAAAGGACTTTCCTATTTCATATTTAGGGACAATATTGGAACAACATAGAATAAGGCCCCA

AAAGTTGCAAATTAGAAGAGACCATCTCAAAACCTTAAATGATTTTCAAAAGTTATTGGGAG
ATGTTAATTGGCTACACCCAGTACTTGGGATTCCTACTTATCAATTACGACATTTATTTTCTA
CTTTAGAAGGAGATACAGCTCTAGATAGCCCCCGGACCTTAACCCCATTGGCTTTACAGGAA
CTTCAATTTGTTGAGCAACGACTAAACGACAGCTTTTTTACTTACTTACATGCGTCTCAACTT
ATTCCGTTTATAATATTTCATACCCCTTATTCTCCATCTGGTGTAATTGCTCAAGAAATAGGA
TTAATAGAATGGGTTTTCTTACCTAACAGTTTTTCCAAAAAATTGACTACATATATGGATAAA
TTAGCCTTCCTTATACAAAAAGGTCGCCATCGTATTTTACAACTGTCAGGATGTGAACCACA
CCAGATTGTTACTCAGTTAACAACTGCTCAAATATCTCGATGTTTACAGTTTAATGAAAACTG
GCAAATTTCTCTTGCCTCATATCCTGGTTCATTTTCTAATCATTATCCATCATCTAAATTGATT
GATTTTCTCCAGACTAACGCTACGATATCTCATTCCCCAATTTCAGATGTTCCAGTTAAGGGA
CCCACTATTTTTACAGATGCAGATAAAAATACTGCTGGATATTGGACCCCGGAAAGTTCCAA
GGTTCTCCCCCACTCATTTTCTTCTGTACAGCCCGCTGAATTGTGGGCTATCTATTTAGTTTTG
CAAGATTTTCCCCAACTTCCTATTAACATTGTTTCAGATTCTCAATAAGCTGTTCTCTCTTGCC
TCAGCTTCCCCATGTCTCCCTTCCACTGACCCTTAAAACAGCTATTGATAAATTGTTTTACCA
AGTACAACAATTTCTCTTGCAGTGTTCAGAGTTAATTTTATCCGTGCACATTCTGCCCTTCCT
GGACCTTTATCATTCGGAAATGCTACAATTGATGCCTTACTTTATCCTACAGAAGCAGCAAA
ACAAGAACATCTCTTACAGCATACCAACTCCAAAGGGTTACAAAAATCTCATGCTATTACTC
AAAAACAAGCTCAAAATATTGTTCATTCTTGTTCCATATGTGCACCCTTTGCTTTACCATTTA
CCCCACCAGGTGTCAACATGAGAGGACAACAAGCAAATCAGATATGGCAAATGGATGTAAT
TTACATTTCTTCTTTCGGACAACAAAAATGTGTGCGTCATACTATAGATACTTGCACACATTT
TCAATGGGCCACTGCATTACATTCTGAAAAGGCTGATGCTGTTATTACTCATTTGTTATCTTG
TTTTGCAGTTAAGGGATTACCAATTGAATTGAAAACTGATAATGTACCTGCCTACCAATCCG
CAAAATTAGCTCACTTTTTATCTCAATACCATATAACTCATACTTTTGGTATTCCTTATAATA
GTCAAGGGCAAGCTATCATTGAAAGAGCTAATCGTACCTTGCGTGATTATCTTGAAAAAATA
AAAAAGGGGGAACAAGAGAGATTTATGAAACCTAAAGACATTCTGAATAAAACCTTACTTA
CCCTAAATTTTTTGAATGTTTGGAGCAAGGGAAATCTATCAGCAGCAGAGGTGCATTTTCAA
GGGAAAGAAGAGGATAAGAAGATCTTGAATACGCCTATTTGGTATAAAGATAAAGAGAAAG
GTTGGATCCCAGCATCATTAATATATTTGGGACGAGGGTATGCTTTCATTTCTGTTAATAATT
ACAGGTTTTGGACCCCAGCAAGATTGATCAAAATCAACAATGGCTAATCCCTTTGTTCAAAA
ATTCGAAGAGCTTACTATGCAGAGAAGCTTTACTTTCCCTACAAGGGAAGCAACACCTCCTA
CGTGGGGTCAAATGAAGAGGTTGACCCAGGAAGTAGAGAAGATGTTAATGAAGGCAGGGA
AACCTCTGAATCCTACCAATCTTTTGCTTGCCATGATGGCAGTGGTGACATGTCAGGTAATC
GGTGTATCGGCAAGTAATCATACATATTGGGCATATTTACCTAATCCCCCATTAGTAAGAGC
AGTTTCCTGGGGGGGACCAGAAGTGCAGGTATGTACTAATGAGACTGCCTTCTTTCCCCACC
AGCTTTCAGGGGAATAGAACAACTATCTCATCATAAACAACAATATAATATTAGTAATTTGA
CCATTGCAGTGGAAGGTATTCCTTTGTGCATAGGAGGACACCCCTTTTGTCTGTCCACCAAG
GAACATTCTCATCATTCTTATAATACATGGGGGGGGTAAAGTATCATAATTACCATTTTGCTA
CCTTTACTGTGCTTGTTTCCACCAGGGGATTTAACACCTCGACAGAACCGATAGACATTCAT
AATGGAAAACACATGTCGCTATGTCCTGTTAACTTTTTTGCTCCTTCTCTAGAATCTTTGGAG
TGGGAACGTTGCCGAGGTCATCAACCCTTTAAAGTCATGAATTATTCTGGGGCCATCATTGT
AGATTGGAGTCCAGATCGATCATGGGCAATTCTTAGAAAAATGGTCAAATAAATCTCTTAGG
TGGCATCATGCAAATAGCACTTTGATGGGTAATGGTAATGAAACAGTTAAATGGCAGCAATT
TACACTTGTCCCTCCTCAATTACAATTGCAAGGATATCCGCACATTCAAGGAGATATTTGGA
AACTATGGGCAGTTTCTGGTAATCTCACTATCTGGTCAGGAAACTATACTTTGGACAGTGGT
GACTCTTCAGGTCCATTCCATGTTAATTTACATGTTAATAAATCTTATTCCACAATGGCATGT
GTAAAATATCCTTTTGCATTGTTATATGGGAATTGGACTTAGAATGCTACTGTGGGGTCTGTG
TCATGTGACTATTGTAATCTAACTCAATGTGTAAATCAGTCTTGGTGGGAAGAATTTGAAAG

ACAAGCCTATAACTCCAATTTCTCATTAGTAATTGTTAAGGCTCGGACAGAAGTATGGTTAC
CTATAAATCTGACTTGGCCATGGTCAGATTCTTTTGCTGTTTCTCATCTAGTAACCGCTGTAC
AGACTTTGCTACACCGATCTCAACGCATGCTTGGTGTGGTCATTGCTTCGATTCTAGCAGTCG
CGTCAATAACTGCAACAGCGGCGGTAGCAGGTCTTGCGTTACACCAAGGAATTCAAACAGC
TGATTTTATTCGGGACTGGCATAAAGACTCTCATTTGTTATGGCAACAACAGCGAGATTTGG
ATGCACAACTTGCTACCGACGTGCTCAATCTTCAACACACCGTTTCCTGGCTTGGAGATCAA
TTGGCTGTTTTATCTACACGAAGTGTGTTGAAATGTGATTGGAATTCTTCTCAGTTTTGTATA
ACACCTGTACCATTTAACATGAGTGAAAAGTGGGATAAAGTAAAATGATCCTTGACTGGGC
ATCAAAATCTCACTAAGGAGATTATGGACCTGGAACGACAAATTTTGTCTACTTTTAGCAGG
ACGTTACCTGACATTATGGGGTCTGATTTGCTGAAAAGTCTTCAAGAGGAAATGAATAACTT
AAATCCATTAGGGCATGTATCCTCACTAATTGGGACTACCTTTGGGAACACTGTGTTTATATT
ACTTTTATGTTGTGTTGCTTTTCTAGTCTTCCAGCGGTGGTGGAAAGGGAAACAACTAAAGC
GTGAAGCAGAGAAGATCCAGACCATGCTACAATTTATAAAAGCAAATAAAAAAGGGGGAG
ATGAAGGGTTAACAGGGTAGCAGAGATGTGCCTGCAAACCTGTCTCTCTGCTCGGGCTGAAC
GTCCTTGCAAACAAGGCATTCTGCCAAAGAGTCTGGACACAGCCTTGAGTCTAATGGTCCCT
TGCAAACGAGGGAGCATTCCCTTCTTGTGATAAGAAGGAAAAGAGGGCTTTGGACAGACTC
TGCAGTAGACAGAGATTTCACTCCCCTTTGCTGTACGATAACATGTATGCACCTACGCTGTA
CTGAAAAGGCTTATTCATGCAGTCTGGAATTCTGCCTAGGGGGCTTTTATAATAATAAAACC
ACAGTTAGTTTGCGCAGTTCTGTTCCTCCGGCCGGAGTGTGTATTGTCTGTCTCTTGTGTGTC
TCGTGTTTTGTCTTTGTGTCATTTCGCTCGCAACA

>NC_056080.1:66434922-66442861#SHEEP_RIP_31(-)

TGCGGGGGACGACCCGTGAAGGGTTAAGTCTTGGGAGCTCCTCGGCAGGTATGCCGAGCCC
TAGGACATGTTCCTAAGCTCCCTGTCCCGCCACCCTCAAGAATTTTTATAGCCCTTAAGGCTT
CAAGATGTCCAGTTCCTGCAACCTGTCCTAGAAGATAGATTATCTTATTACGTATACTTCATA
GAAGATAGATTATCTGATTGTGTTCTGTATACAATGGTAAGGGTCTAGTGATTGTATCTTGA
GATTAAAAACAACCTTGTGAATGTCATAAGTCATGTACTTTACCCTATATATACTGCAGCAC
AATAAAGCAAGGTATCAGCCATTTTGGGCTGATCCTCTCAACCCCATCTTTTGTCTCTCTCTT
ATTTTTCTTAGCGGGGACACTCCGTTCTCTCCCTGTGCAGGTGCGACTCTTGCTTGTGCTGGC
CGCGGCAGGTGGCGCCCAACGTGGGGCCGTTCGACAGTTTTCCTCGCCACTACTCTTATTAA
TTGAAAAGAGTGAGTATATGAGTATACAAGTGAATTAAATTGAGGAGGAGTAGTAAGGTAT
ATAGTTGAGAGTATAAATATGGGACAGACGCATAGTCGTCAATTGTTTGTACATATGTTATC
TGTAATGTTAAAACAAGGGGGAATTACTGTTTCCAAACCTAAATTAATCAATTTTCTTTCATT
TATTGAGGAAGTTTGCCCTTGGTTCCCCAGAGAAGGTACAGTAAATTTAGAAACATGGAAGA
AGGTAGGGGAACAAATTCGGACTCATTATACTTTACATGGCCCTGAAAAAATCCCTGTCGAA
ACTTTATCCTTTTGGACACTAATTCATGATTGCCTGGACTTTGATAATGATGAATTAAAACGT
TTAGGAAATTCATTAAAACAGGAAGAAGATCCTCTCCATGTTCCTGATTCGGAACCCAGGTA
TGCTGTTCCCGAGGGAGTTGAAGGCGATCCTCCATTTTCTAACTTATTGTGTCCTTCGGATAA
TGATGATTTACTTTCATCCACAGATGAGGCGGAATTAGACGAAGACGCTGCTAAATACCATC
AAGAAGATTGGGGTTTTTTAGCACAAGAAAAGGGGGCGTCAACATCTAAAGATGAATTGGT
TGAATGTTTAAAAAACCTCACTATTGCTTTGCAGAACTCAGGAATCAAGCTTCCTAGTAACA
ATGCTAAATCTCCTTCTGCTCCGCCTCTTCCCCCTGCCTATGCTCCTTCCGTTGTAGCAGGTCT
CGATCCTCCTCTGGGGCCTCCTCCACCGTCTGAGATCATGTCTCCGCTGCAGAAGGCATTGA
GACAAGCACAACGACTTGGTGAGGTTGTCTCTGATTTTTCTCTTGCTTTCCCTGTCTTTGAAC
ATAACAACCAGCGTTTTTATGAATCACTGCCTTTTAAACAACTGAAAGAGTTAAAGATTGCT
TGTTCACAATACGGTCCTACCGCTCCATTCACTATTGCTATGATAGAAAGTTTGGGTACTCAA
AATCTACCCCCAAATGATTGGAAACAAATAGCTAGGGCCTGTTTGTCGGGGGGAGATTACTT

GCTATGGAAATCTGAATATTTTGAACAGTGTGCTCGTATAGCCGATGTTAACCGACAGCAAG
GTATACAGACCTCCTACGAAATGTTGATTGGTGAAGGCCCTTACCAGGCTACTGATACTCAA
CTTAATTTCTTACCTGGTGCATATGCACAAATATCAAATGCGGCTCGGCAGGCTTGGAAAAA
ACTTCCTAGCTCCAGTACTAAGACAGAAGATCTTTCAAAAGTCCGACAGGGACCTGATGAGC
CTTATCAAGACTTCGTGGCACGGCTCTTAGATACTATAGGTAAGATAATGTCAGATGAAAAG
GCTGGGATGGTATTAGCAAAACAATTGGCTTTTGAAAACGCTAACTCTGCCTGTCAAGCTGC
TTTAAGACCTTATCGAAAAAAGGGAGATCTGTCTGATTTTATTCGCATTTGTGCTGACATTGG
ACCCTCCTATATGCAAGGCATTGCTATGGCAGCAGCATTACAAGGAAAAGGCATTAAACAG
GTACTTTTTCAGCAGCAAGCCCGGAACAAGAAAGGACTTCAAAAGTCAGGTAATTCGGGTT
GCTTTGTTTGTGGTCAACCTGGCCATCGGGCTACAGTGTGCCCCCAAAAGCAACAAACCTCT
GTTAACACTCCTAATTTATGCCCACGATGTAAAAAAGGGAAGCATTGGGCCCAAGATTGTCG
TTCTAAAACGGATGTTCAAGGTAATCCTTTGCCCCCGGTTTCAGGAAACTGGGTGAGGGGCC
AGCCCCTGGCCCCGAAACAATGTTATGGGGCAACACTGCAGGTTCCAAAAGAACCATTGCA
GACCTCTGTCGAGCCACAAGAGGCAGTGCGGGATTGGACCTCTGTGCCACCTCCTACACAGT
ATTAACACCCGAGATGGGGGTTCAAACCCTTGCCACAGGAGTGTTTGGGCCTTTACCTCCGG
GAACCGCTGGACTGCTTTTGGGGCACAGCAGTGCGTCTTTAAAGGGAATACTTATTCATCCT
GGTGTGATTGACTCTGATTATACAGGAGAGATAAAAATATTAGCCTCCGCTCCTAACAAAAT
TATTGTAATTAATGCAGGACAACGTATAGCTCAACTCCTTTTAGTTCCATTAGTTATACAGGG
AAGAACAATTAATAGAGACCATCAAGATAAAGGTTTCGGGTCCTCTGACGCCTATTGGGTGC
AAAATGTTACCGAGGCACGACCAGAACTTGAGCTACGCATTAATGGTAAGCTTTTCCGCGGA
GTGCTTGATACAGGGGCCGATATTAGCGTTATTTCTGATAAATACTGGCCTACTACATGGCC
TAAACAAACAGCTATTTCCACTCTTCAGGGTATTGGCCAAACTACCAATCCAGAACAAAGTT
CATCCCTTCTTACTTGGAAGGATAAAGACGGCCATACAGGCCAATTTAAACCTTATATTCTG
TCCCATCTTCCAGTTAATCTATGGGGGCATGATATTTTAAGTAAAATGGGTGTTTATTTATAT
AGTCCTTCATCCACCGTAACAGATTTGATGTTAGATCAGGGCTTACTTCCAAACCAAGGTTT
AGGTAAACAACATCAAGGCATCGTTTTACCCCTTGATTTAAAATCTAATCAAGATCGAAAAG
GCTTGGGGTGTTTTTTCTAGGGACCTCTGATTCTCCTGTGACACATGCCGATCCTATTGATTG
GAAATCTGAGGAACCGGTATGGGTCGATCAGTGGCCCCTGACACAAGAAAAACTTTCTGCC
GCACAACAGCTGGTGCAGGAACAGCTGAGGCTTGGGCATATTGAACCCTCTACCTCTGCTTG
GAATTCCCCAATTTTTGTTATTAAAAAGAAGTCTGGAAAATGGAGATTGTTACAAGATCTTC
GCAAGGTAAATGAAACAATGATGCATATGGGAGCCCTACAACCTGGGTTGCCCACTCCTTTC
GCTATACCTGATAAATCCTATATCATTATTATAGATTTAAAAGATTGTTTTTACACTATTCCT
CTTGCACCTCAAGATTGTAAAAGATTTGCCTTTAGTTTACCCTCTGTTAATTTTAAAGAGCCT
ATGCAACGTTATCAATGGAGAGTCCTTCCACAAGGAATGACTAATAGCCCTACGCTGTGTCA
AAAATTTGTTGCTACAGCATTAGCTCCCGTTCATCAACGTTTTCCTCAGTTATATTTAGTTCA
TTATATGGATGATATATTACTAGCTCATGCTGAAGAACATCTATTGTATCAAGCTTTTTCTAT
TCTAAAAAAACATTTAAGCCTTAATGGTCTTGTCATTGCTGATGAAAAACTTCAAACTCACTT
TCCCTATAATTATTTGGGTTTCTCCTTATACCCTCGTGTTTATAACACCCAATTGGTACAATTA
CAGACTGACCATTTAAAAACTCTAAATGACTTTCAAAAACTTCTAGGAGACATTAATTGGAT
ACGCCCTTATTTAAAACTACCCACTTATACCTTGCAGCCATTATTTGACATCCTTAAAGGTGA
CTCTGACCCTGCGTCACCCCGAACACTTTCTTTAGAAGGACGATCAGCCTTACAATCAATAG
AATAAGTTATTAGACAACAACAGATTACTTATTGTGATTACCAACGATCATGGGGTTTGTAT
ATACTTCCTACCCCTCGAGCACCCACAGGGGTTCTTTATCAAGATAAACCTTTGCGATGGAT
ATATCTATCTGCTACTCCAACTAAACATCTGCTCCCTTACTATGAGCTTGTTGCAAAAAGTGT
AGCAAAAGGACGTCATGAGGCTATCCAATATTTCGGCATAGAACCTCCCTTCATTTGTGTTC
CTTATGCTTTAGAACAACAAGATTGGCTTTTTCAGTTTTCAGATAATTGGTCTATAGCTTTCG
CAAATTGCCCAGGATGGATTACTCATCATTATCCTTCTGATAAATTGTTACAATTTGCTAGCT

CTCATGCCTTTATTTTTCCAAAAATAGTTCGCCGACAACCTATTCCTGAAGCAACACTTATAT
TTACAGATGGATCGTCTAATGGTACTGCAGCTTTAATTATTAACCACCAAACCTATTACGCA
CATACCAGTTTTTCTTCTGCTCAGGTTGTTGAATTATTTGCAGTCCACCAAGCATTGCTAACT
GTACCCACTTCCTTCAATTTATTTACAGACAGCTCCTATGTGGTCGGTGCCTTACAAATGCTT
GAAACTGTTCCAATTATCGGCACAACCTCTCCTGAAGTTCTTAACTTATTTACATTGATTCAA
CAGGTTCTCCACTGTCGCCAACACCCGTGTTTCTTTGGGCATATTCGCGCACATTCCACCCTT
CCTGGTGCCCTGGTACAAGGCAATCACACTGCGGACGTTCTTATTAAACAAGTGTTTTTCA
ATCAGCTATTGATGCAGCCCGAAAATCCCATGACTTACATCACCAAAATAGTCATTCTTTAC
GGTTACAATTTAAGATTTCCCGTGAAGCTGCACGACAAATCGTTAAATCTTGTTCTACCTGTC
CTCAATTCTTTGTTCTCCCTCAATATGGTATCAACCCTCGAGGTCTATGCCCTAATCATCTCT
GGCAAACAGATGTCACTCACATTCCTCAATTTGGGCGTCTTAAATATGTTCATGTCTCTATCG
ACACTTTTTCCAATTTTCTCATGGCCTCCCTTCACACCGGAGAATCAACTCGTCACTGTATTC
AACATTTACTTTTTTGTTTTTCTGTTTCAGGAGTTCCAAATACCCTCAAAACAGATAATGGAC
CCGGTTATACTAGCCGTTCTTTTCAACGTTTTTGTCTTTCTTTCCAAATTCATCATAAAACAGG
AATTCCTTATAATCCACAGGGGCAAGGTATTGTGGAACGAGCCCATCAACACCTTAAACATC
AATTATTAAAACAAAAAAAGGGGAATGAACTGTATAGCCCCTCACCGCATAACGCCTTAAA
CCATGCTCTTTATGTTTTAAATTTTTTAACGTTAGACGCAGAAGGCAACTCAGCAGCCCAGC
GTTTTTGGGGAGAACGGTCCTCATGCAAAAAACCACTTGTACGATGGAAGGATCCACTTACC
AATCTGTGGTATGGGCCAGACCCTGTATTGATATGGGGACGGGGGCATGTTTGTGTTTTTCC
ACAGGATGCCGAAGCGCCGCGCTGGATTCCGGAAAGGCTGGTACGCGCGGCGGAGGAACTC
CCTGACATACCAAATGCATCGCATGACACTGAGCGAGCCCACGAGTGAGCTGCCTACCCAG
AGGCAGATTGAGGCGTTGATGCGATATGCTTGGAATGAGGCTCATGTACAACCTCCAGCGAC
GCCTGCAAAAATACTGATCATGTTATTATTATTGTTACAGCGGATACAAAACGGGGCGGCTG
CGGCTTTTTGGGCATACATTCCTGATCCGCCTATGATTCAATCCTTAGGATGGGATAAAGAA
ACAGTACCTGTATATGTCAATGATACAAGTCTTTTAGGAGGAAAATCAGACATTCACATTTC
TCCTCAGCAAGCCAATATCTCCTTTTATGGTCTTACTACGCAATATCCTATGTGCTTTTCTTAT
CAATTACAGCATCCTCACTGTATACAGGTGTCAGCTGATATATCCTACCCTCGAGTGACTATT
TCAGGCATTGATGAAAAAACCGGAAAAAGATCGTACCGTGACGGAACCGGACCTCTCGACA
TTCCGTTTTGTGACAAACATTTAAGCATCGCCATAGGAATAGACACTCCTTGGACTTTATGTC
GAGCACGAATTGCATCGGTGTATAACATCAACAATGCCAATACCACCCTTTTATGGGACTGG
GCACCTGGAGGAACACCTGATTTCCCCGAATATCGAGGACAGCATCCACCCATTCTCTCTGT
AAACACTGCTCATACATACCAAACAGAACTGTGGAAACTTTTGGCTGCTTTTGGTCATGGCA
ATAGTCTATATTTACAACCCAATGTTAGTGGGAGTAAATATGGTAATGTAGGAGTTACGGGG
TTTTTATATCCCCGAGCTTGTGTTCCTTACCCATTCATGTTGTTACAAGGCCATGCAGAAATA
ACATTGTCAATGAATATTTATCATTTAAATTGTTCTAATTGCATACTTACTAATTGCATTAGG
GGTGTTGGAAAAGGAGAACAAGTTATAACTGTAAAACAACCTGCTTTTGTAATGTTACCTGT
TGAAATAACTGAAGATTGGTATGATGAGACTGCTTTAGAATTGTTACAGCGCATTAACACGG
CTCTTAGCCATACTAAAAAAAGTGTGAGCCTGATTGTTCTGGGTATAGTATCTTTAATCACCC
TTATAGCAACTGCTGTTACCGCTTCTGTATCTTTAGCACAATCCATTCAAGCTGCTCATACTG
TAGATTCCTTGTCATATAATGTTACTAAAGTAATGGGAACACAAGAAGATATAGATTAAAAA
ATGGAAGATAGATTATCAGCTTTATATGATGTGGTTAGAGTTTTAGGAGAACAAGTTCAGAG
CATTAGTTTTCGCATGAAAATTCAATGTCATGCTAATTATAAATGGATTTGTGTTACAAAAA
AGGCTTATAATGCATCTGACTTTCCGTGGGATAAGGTGAAAAAACATCTACAAGGAATTTGG
TTTAATACTAATGTCTCTCTAGATCTTTTGCAATTGCATAATGAAATTCTTGATATTGAAAAT
TCTCCAAAAGCTACACTGAATATAGCTGATACTGTCAACAATTTTTTACAAAATTTATTTTCT
AACTTCCCTAGCCTTCATTCACTGTGGCGAAGCATAATTGCTGTGGGCGCGGTTCTGACTGTT
GTGCTTATCATAATTTGTTTAGCTCCTTGTCTTATTCACAGTATTGTTAAGGAATTTCTACATA

TGAGAGTTTTAATACATAAAAACATGTTGCAGCACCGGCATCTTATGGAGCTTTTAAAAAAA
TAAAGAGAGGGGAGTTGCGGGGGACGACCCGTGAAGGGTTAAGTCTTGGGAGCTCCTCGGC
AGGTATGCCGAGCCCTAGGACATGTTCCTAAGCTCCCTGTCCCGCCACCCTCAAGAATTTTT
ATAGCCCTTAAGGCTTCAAGATGTCCAGTTCCTGCAACCTGTCCTAGAAGATAGATTATCTT
ATTATGTATACTTCATAGAAGATAGATTATCTGATTGTGTTCTGTATACAATGGTAAGGGTCT
AGTGATTGTATCTTGAGATTAAAAACAACCTTGTGAATGTCATAAGTCATGTACTTTACCCTA
TATATACTGCAGCACAATAAAGCAAGGTATCAGCCATTTTGGGCTGATCCTCTCAACCCCAT
CTTTTGTCTCTCTCTTATTTTTCTTAGCGGGGACGCTCCGTTCTCTCCCTGTGCAGGTGCAACT
CTTGCTTGTGCTGGCCGCGGCA

Data Set 3

Data Set 3. 14 out of the 31 full-length Endogenous Retrovirus (ERV) insertion polymorphisms were chosen following a comparison of genomic coordinates.

>NC_056054.1:102854042-102860826#SHEEP_RIP_01(-)

GTGGAGGAGCTGGAAGGCTTTGAGCAAATACTGAAAATGTATTTGCTCCACTCATGACGAA
GGTTGGAAGCTGGGACGAGCATAACAAAGGGTTATGTGCGTTCACCGAGTGCCCAAGGCTG
GGAACGGATGACAAAGGGTCAATACGCCCGGCCTGGAGGAGTTCAAAGGCTTCCTTCTGAC
CACCTGTTTCTGAGAGCAAGGACTATTTTTTCATGATAAGACTCCCTTTAGAGTTTCGTCAAA
GCTATGTTATGGCTTGGGCGGTAGGAATTGTATTTTATCCTTGAATGTTTTAATGTTTATCTG
GAATGGCTACATGCAAGTCAGCCTTATGCTCTATTCCCTGAAAATTATAAAACTATACAATT
GGATAATAAACTTTGTCAGTCCACTAGAGGCTGTCCCCAAGTGTCCTTTTCAGAGTGCGGTT
CTTGGAGCCTTATGGATGGCGCCCAACGTGGGGCTCGAAGCAGCAGACTGATTTTGAAGAA
GGGCCGCACTCCTGCAGAAGCGAGGTAAGCAGAATGGGACATCAGACAAGTAAAATTCCTT
TAGTTCTTCTCATGCATCATTTCTTGAAACAATATGAGGTTAATCTGCCTGAAGAGCAGTTAA
CTAGCTGTTACTAGACAGTGGTTGAATATAATCCATGGTTCCCTTAGGAAGGAACCTTAGAT
TTACAAATTTAGACCAGAGTGAAAAACAATGTTCTAAAAGCTTATAGACAGGGAATAAAAA
TTCCCCCACAATGGCCTCTTTTACGGGCCATTATGGAATGATTAGATGGCTCTGGAGGAAAT
TTAAAGGTTGAAACTGTTCGATCCCTGCATGAATGTGAATTAGAAGAGAAAGATCTATCAGA
GGTTTTAAATCAGAAAAATGTCACGCTAGAGCAGATTACGGACAAACAAGAGTTACAGGTG
CTAAAAGCAATTAAACAATCAACTTTGCCAGAGGCCCCTGATCCGCCTTTACCTTGCTTTTTG
CGAGCTCATATTAACAAGCCTCTGACACTGGCCTTTCCGCCCGCTGCGATGCTGTCCTCCCCT
ATTTCCTAGCATTCCTCCTCGGGACATGCCTCTTTGTGCGTGGGCTTTTCCGGTCCAATTTAA
TAATTCTCAACTTGGACATAATCAATGGCAGTCACCTGATTTTGGTTTGCTCACGCAATTCAA
TAAGGCATGTACGTTATATGGACCTACCTCACCCTACTGTATGGAATTTCTTAGGGGCCAGG
TGGATCAGTGGCTTCATGCAGATTTTTTTACAGTTGCTAAAATGGTTATGACTCCACAACAGC
TACTACAATGGCAAATGTGGGTCACGGATGAAGCCACATTAATCTTGCAAGAACAGCAAGC
AGGGAAAACCCTACTGGACTAAATTTTAAAATTCTCACCGGCACCAGAGCTATGGCTAAAAC
TGATGCACAATTACAGTTTGTGCAACCCCCCATGTTATACTAGATTAAGGAAGTAGCTATCA
GAGCGTGGGCTAAAATGACAGTTCCACCTCTAATGGATCTTTTGTAAAAATATTGCAGGGAC
CAACTAAAAAATATACTCAATTTATTGATAAATTGAAGGAGGCCATTGATCAGTCTTACGGA
TGCATCTTTGCGAGAAACCATTTTGAAACAATTAGCCTTTGATAATGCTAATGAAGATTGTC
AGGCTATTATCAGACCCCTTAGGGGGCAAAGAGAAGTTCTGAAATACTTGAAAGCCTACAG
GAATGTGGGGACAATTCAACATAAAGCTAAAATAGCCGCTTTAGAAACCTTAAATGTTTCCC
AGAAGTCTGAAGTTAAATGTTTTAACTGCGGCAAGCCGGGACACACGCGGAAGCAATGTCG
CTTGCCTCGGCAAACAGGTCTTTCTCCTGACAAAGGAGGGGCTATTAAAACTAAGCCCCCCG
GGCTCTGCCCAGGATGTAAAAAGGGGAATCACTGGCTGACTGAATGCCACTCTAAATTTGAT
AAACAAGGTAATGCTTTACCCATTCAGACACCTCCTTCGGGAAACTAGAACAGGGGCTCTCC
TCTAGCCCCATTAAACAAGGAGGACAACCAAATATTACTAAATTAACAGCGGCTACCAGAC
ATAACACATGTATAGACATTCCTACTCCCTGAGATATAAAATTATTAATGGTAAATAATCCT
ATGAAAATCTTAACTGGATATTTTGGCCCTATACCAAAAAATACTATAGACCTCCTATTGGA
ACAAAACAACACCATGCGCAAGATAATTGTACATACTGAGATCATTGATAAAGATTACACA
GGTAAAATTGCAATAATGTTACATGTGACTCACAACTTGTATTTACAAAAGGGTGACAGATT
TGCTCAGTTATTGCTATTACCTTATGTGCCCCCACTTAATAGAAAAGCAGACGCCAGAACAG
GTGGCTTTGGGAGTACGAACGTTACTGCAGCCCTTTCTACTGTTATAAAAGAAATCAATAGG
CCCATGTTAAAATTAAAAATCAGAGAAAGAACTTTTGAAAGAATGTTAGACACTGGGGCAA
ATGTTTCCATCATAAGAACAAAAGAATGGCCTTCGGACTGGCCTACAATTTTAACCTCACAC
CAGTTGGTGGGAATAAGCACTGCAGATGCAGCTCAAACTTATGTTAGTTCATCTTACTTACA

AGCCCTGGGCCCTAATCAATTAGTCGCTTACATTAAACCGTACATTGCCCCATTACCATTAA
ATTTGTGGGGAAGAGACTTTCTACAACAAGCTCAAGCGACTATACAATTAAATGAAACTTTT
TCTTAGGGGTCACTGAAATAAAGCCACTAAAGTTAAAATAAAAGTCTAATAAACCTATCTGG
ACAGCTCAATGGCCCCTATCAAAACAAACCAAAAAAAACTGTCTGCTTTGCATACTTTGGTG
GCTGAACTACTACAACAAAATAAAATAAAAACTACTCAATCACCATGAAATTCACCAGCTTT
TGTCATTAAAAAAAAATCAGGTGAATAGAAAATGCTAACAGATTTTAAAAACATTAACACT
ATAATGATTCCTATGGGAGCATTACTACCAGGACTCCCAAGCCCTGCTATGGTCCCTAAGGA
CTGGGCTATTATGATTTTTGATTTACAAGACTGCTTTTTCACTATACCTTTACATCCAGAGGA
CAGGCAACGCTTTGCCTTCTCAATACCTTCTATTAATAATCAATCCCCTGCTCAACGGTATCA
ATGGAAGGTCCTGCCCCAAGGTATGATGAACGCCCCTGTGGTCTGTCAATTCGTTGTTGATA
AAATTTTGCACCCCATCAGATAGCAATTCCCTGAGGCATATCTCATTCATTACATGGATGAC
ATTTTATTCATTGGCTTCTTCCTCAGAATCTCAATTAAGTTTATTAGGTAATGAGGTCATAAC
TAAACTAATCATGAGCTACTAATAGCAGAAGATAAATTGCAACACCATTCCCCTTTTAAATA
TCTTGGATATCTTATGGACCGCTCCACTGTATAGCCACAGAAGCTTTCTATTAGAAGGGATA
ATTTACAAACACTTAATGATTTCCAAAAACTTCTTGGGGATATTAATTGGCTACGACCTACCT
TGGGAATTCCCTCATATGCTTTACAAAACTTATTCAAATTATTAGAAGGTTCCTCTGATTTAA
ATAGTCCCCGACAACTTACCCCTGATGCTAAGAAAAAATTACAATTGATAGAACAGAAAATT
CAACAAACATTTGTTTACCGTGTTAATTACAATTCCCCTTTTCAGATATGTCTTTGGTACTAA
AATATCTTCTACTGCCATTCTAGTGCAGAATAATCACCTATTGAATGTGTATATCTCCATTCC
AAACAGACTAAACGCATTGTTTCCTATATAGACTTAATAGGGAAAATCATTTTTCTTGCACG
CTCTTGTTTGAGCAGTATAGCTGGATATGACCCTACTCAGATTTACCTACCTTCGACAAAAAC
AGAAATTGATAATGCTCTCCAGGTGTCCACTACCATTCAGATAGCCCTTGCTGATTATTCAG
GGGAGTTATTGGCCAACCCACCTAAGGAAAATTATGGAATTTCTTACAAAATACTTCTTTTA
TCATCAACAATATTATTTCTGAACACCCTCTCATGAATGCACCTAATTATTTTATAGATGGAA
ATAAGGCAAGATGGGCAACCATAATAGGTCCCAACCTACAAAAAAAAAAAAATTAAAAGTCC
TTATCAATCTGTTCAAAAAACAGAATTATTCACATTATATTGTTTACTTACTCTAATAAAAAC
CCCATTGAATGTTTTAACTGATTCTCGCTACGTGGCACAGCTTTTCCCATCTTTTGTAATGGC
TCATTTTATATCCAATGAAAATGATCTTATACATTTGTACTTATTGATTCAGCAGGAAATAAG
AGCAAGACTCCATCCCTTCTTTATTACTCACATCCGTGCTCGTTCCCATTTACCAGGACCCCT
CAATTTAGGCAATGATTTGGCTGATCGCCTCATCGCCCCTATATTTTCTTCCCCCGAACAGGA
ACATCAGCTCTTCCATACTAACGCTAATAGACTACACGTTCAAAATAAGATACCGTTACAAA
CGGTTGGAAAAGTTGTTCGGAACTGTGCCACATGTGCCCCCTTTCATTTGACCACTAGTCCCG
AGGGACTAATCCTAGAGGCTTACAAGCAAATGAATTATTGCAAGCTGATTTTACACATTGCA
AACTGCTCCCTTTTAAATTGTTATTTGTAGTCAGACACCTTTTCCGGCTTCATTTGGGCAGCT
CCTTCCACTGCAGAGACTACTAGAGCTGCCGTCACAGCTCTTCTGCAATGTTTTTCTGTGATG
GGGATCCCTGCCTCCATCAAAACTGACAATGGTCCAATGCTTTTCGTGATTTCATGCATCAGT
GGAGTATTTGCCATCTTACTGGCATCCCATACGGCCCTCAAGGTCAAGCCGTCATTGAATGG
GCCCACCATACACTCAAGCTCATTCTTAATAAACAAAACAGGGGAATAAACTAAGGGGCCC
CTATGGACCTAAAGCCATTTTGCCTATAGCCCTTCTAACAATCAATTATTTTAATTTGCCTCA
ATCAATTATTTTGTCTCTACACAGTCAGGAAACATGAACAGAGCGACATTTTTCTGATTCACC
CTCACGTATGCCTGAACAAATTGCTGTTTGGGTCAAATGTCTTAATCAATGGTGGCCAGGAA
CACTTAAGTTCCTAGGCAAGGGATATTATCTCGTTATTTCAGATGATGGAACTGAGCAGTGG
GTCCCACTCAAAAGAGTCAGAAGACGGACAGACCTTGCTCCACACCCCCTGACCGGTCACA
GCACTAAAACAGAAATTGCAACAGATGCCTTCTGAGCCTCCTTACGACAACGTATCCTGAAA
GAAAAAATCATCTGTGCCAGGGATGGACTGGTCCACCTGTAGAGGAAGGTTAAAGAAGTTT
CTGTTCATGCCAACATGACTTTGTTGATTGGGGGCCTCATGGACTCTGTGAATTGCTCAGAAT
CACAGAAAATAATCACACCTGCCAATGGTATAATGTTTCAGCACCACATTTTAACAGAACAC

AAACAGGACTCTAGCATCAACGCAATGAACTTTTGAACTGGTACAATGATACACCTCCCAGA
CCCCGGATAATCAGTCCAGTCCTGGGGCCTGAACACTGGCATCTTTGGAAAATTCCTGCTTG
CTTGTCCCGGTTTAGAGACTTGTATGCTTATGCACATGTTTCCATTCCACATAATTATACTAT
TGAGTATAATTATACCGGTTATGTTCATGCTTGTGTAAATGCGCCCTGTCTTTTTGCTATTGG
ACAATTTAGGAGTAATGGCTCAATTCTGTCCTGTACTGTCGTTTGTATACTTGTTTAAATCTT
AACGTGCCAATTAATGTTACTAAAGACAGTGGTTTTTTAGTCCGGCAAAGGACTGATTTGTG
GGTTCCAGTTAAGATTTCTGAACCTTGGTCAGATTCCACGTTGTTGTCTTTTGTTCTGAGAGA
ATCCCTGAAAAGAAGCAAACGCTTTATTGGTTGGATTATAGCTACCATAGAGGGTATTATTT
CAATTGTAACTGTTGGTACAGTTTCTGGAATGGCATTATATAATTCTATTCAAAATCATGATT
TCATTACTGCTTGGGAAAAGGCTTCTCATGATCTCTGGGCCCAACAAGCTCAGATAGATCAA
CAAATACAGATCACGTCTACCTTCAGCAGCATTCCCCCTGTGTTATATAACACTGACTTCTAT
AATACACTATCTGCTAGTACTTCCTTTTTAAATCCAAAAAATTGGGTACCCCATACGATGGCT
TCTTATATGCTGATCTGTTTGTTTGTTATTGTTCTTATAGGATTCCGAACGTTATGTGCTCGAG
CCACCGCTGCCCAAAAAGCAAGAGTAAAAATGGCTGCAACAGTCCTTGCTCTTGAGGAAAA
AGGGAGGAAACGTGGAGGAGCTGGAAGGCTTTGAGCAAATACTGAGAATGTATTTGCTCCA
CTCATGACAAAGGTTGGAAGCTGGGATGAGCATAACAGAGGGTTATGAGCGTTCACCGAGT
GCCCAAGTCTGGGAACGGATAATGAAGGGTTATACGCCCGGCCTTGAGGCATTTGAAGGCTT
CCTTCTGACCACCTGTTTCTGAGAGCAAGGACTGTTGTTTCATGATAAGACTCCCTTTAGAGT
TTCGCCAAAGCTATGTTATGGCTTGGGCGGTAGGAACTGTATTTTATCCTTGAATGCTTTAAT
GTTTATC

>NC_056054.1:272298309-272306235#SHEEP_RIP_04(-)

CTGCGGGGGACGACCCGTGAAGGGTTAAGTCTTGGGAGCTCCCTGGCAGGTATGCCGGGCC
CTAGGACACGTGCCTAAGCTCCCTGTCCCGCCACCCTCAAGAACTTTTGTAACCCTTAAGGC
TCCAAGATGTTTGGTTTCGGCAACATTTCATAGAAGATAGATTATCTTATTGTGTATACTTCA
TAGAAGATAGATATTCTGATTGTGTTCTATATACAATGGTAAGGGTCTGGTGATTGTATCCTG
AGATTAAAAACAACCTTGTGAGTGCCTTAAGTCACGTACTTTACCCTATATATACCGCAGCA
CAATAAAGCAAGGTATCAGCCATTTTGTTCTGATCCTCTCAACCCCATATTTTGTCTATCTCT
TATTTTCTTAGCGGGGACGCTCCGTTCTCTCCCCTGTGCAGGTGCGACTCCTGCTTGTGCTGG
CCGCGGCAGGTGGCGCCCAACGTGGGGCCGTTCGACAGCTTTCCTCGCCACTACTCTTATTA
ATTGAAAAGAGTGAGTATATGAGTACACAAGTGAATTAATTTGAGGAGGAGTAGTAAGGTA
TATAGTTGAGAGTATAAATATGGGACAGACGCATAGTCGCCAGTTGTTTGTGCATATGTTAT
CTGTAATGTTAAAACATAGGGGAATTACTGTTTCTAAACCTAAATTAATCAATTTTCTTTCAT
TCATCGAGGAAGTTTGCCCTTGGTTCCCAGAGAAGGTACAGTAAATTTAGAGACATGGAAG
AAGGTAGGGGAACAAATTCGGACTCATTATACGTTACATGGCCCTGAAAAAATCCCTGTCGA
AACTTTATCCTTTTGGACACTAATTCGTGACTGCCTGGACTTTGATAATGATGAATTAAAATG
TTTAGGAAATTTATTAAAACAGGAAGAAGATCCTCTCCATGTTCCTGATTCGGAACCCAGGT
ATGCTGTTCCCGAGGGGGTTGAAAGCGACCCTCCGTTTTCTAACTTATTGCGTCCTTCGGATA
ATGATGATTTACTTTCATCCACAGATGAGGCAGAATTAGACGAAGAAGCTGCTAAATACCAT
CAAGAAGATTGGGGTTTTTTAGCACAAGAAAAAGGGGCTTTAACATCTAAAGATGAATTGG
TTGAATGTTTTAAAAACCTCACTATTGCTTTACAGAACGCAGGAATCAAGCTTCCTAGTAAC
AATGCCAAATCTCCTTCTGCTCCGCCTCTTCCCCCTGCTTATGCTCCTTCTGTTGTGGCTGGTC
TCGATCCCCCTCCAGGGCCCCCTCCACCGTCTGAGAGCATGTCTCCGCTGCAAAAGGCATTG
AGACAGGCACAGCGACTTGGTGAGGTTGTCTCTGATTTTTCTCTTGCTTTTCCTGTCTTTGAA
AATAACAACCAGCGTTATTATGAATCACTGCCTTTTAAACAACTGAAAGAGTTAAAGATTGC
TTGCTCACAATACGGTCCTACCGCTCCATTCACCATTGCTATGATAGAAAGTTTGGGTACTCA
AAATCTACCCCCAAATGATTGGAAACAAATAGCTAGCGCATGTCTCTCAGGGGGAGATTATT

TATTATGGAAATCTGAATTTTTTGAACAATGTGCTCGTATAGCCGATGTTAATCGACAGCAA
GGTATACAGACCTCCTATGAAATGTTGATTGGTGAAGGCCCTTACCAGGCTACTGATACTCA
ACTTAATTTCTTACCTGGTGCATATGCACAAATATCAAATGCAGCTCGGCAGGCATGGAAAA
AACTTCCTAGCTCCAGTACTAAGACAGAGGATCTTTCAAAAGTCCGGCAGGGACCTGATGAG
CCTTACCAGGACTTTGTGGCACGGCTCTTAGATACTATAGGTAAGATAATGTCAGATGAACA
GGCTGGGATGTTATTGGCAAAACAATTGGCTTTTGAAAACGCTAACTCTGCTTGTCAAGCTG
CTTTAAGACCTTATCGAAAAAAGGGAGATCTGTCTGATTTTATTCGCATTTGTGCTGACATTG
GACCCTCCTACATGCAAGGCATTGCTATGGCAGCAGCATTACAAGGAAAAAGCATAAAAGA
GGTACTTTTTCAGCAGCAAGCCCGGAACAAGAAAGGACTTCAAAAGTCAGGTAATTCGGGT
TGCTTTGTTTGTGGTCAGCCTGGCCATCGGGCTGCAGTGTGCCCTCAAAAACAACAAAGCCC
TGTTAACACTCCTAATTTGTGCCCACGCTGTAAAAAAGGAAAGCATTGGGCGCGGGATTGCC
GTTCCAAAACGGATGTTCAAGGTAATCCTTGCCCCCGGTTTCGGGAAACTGGGTGAGGGCCA
GCCCTGGCCCCGAAACAATGTTATGGGGCAACACTGCAGGTTCCAAAAGAACCATTGCAGA
CCTCTGTCGAGCCACAAGAGGCAGCGCGGGATTGGACCTCTGTGCCACCTCCTACACAGTAT
TAACTCCTGAGATGGGGGTCCAAACCCTTGCCACAGGAGTGTTTGGGCCTTTACCTCCAGGG
ACAGCTGGACTGCTTTTAGGGCGCAGCAGTGCGTCTTTAAAAGGAATACTTATTCATCCTGG
TGTGATTGACTCTGATTATACAGGAGAGATAAAAATATTAGCCTCCGCTCCTAACAAAATTA
TTGTAATCAATGCAGGACAGCGTATAGCTCAACTTCTTTTAGTTCCATTAGTCATACAAGGA
AAACAATTAACCGAGACCGTCAAGATAAAGGTTTCGGGTCCTCTGACGCCTTTTGGGTGCAA
AATGTTACCGAGGCACGACCAGAACTTGAGCTACGCATTAATGGTAAGCTTTTCCGCGGAGT
GCTTGATACAGGGGCCGATATTAGTGTTATTTCTGATAAATATTGGCCTACTACATGGCCAA
AACAGATGGCTATTTCCACTCTCCAGCGTATTGGCCAAACTACCAATCCAGAACAGAGTTCA
TCCCTTCTTACTTGGAAGGATAAAGATGGACATACAGGCCAATTTAAACCTTATATTCTGCC
CTATCTTCCAGTTAATCTATGGGGCGTGATATATTAAGCAAAATGGGTGTTTATTTATATAGT
CCTTCACCCACTGTGACAGATTTGATGTTAGATCAGGGCTTACTTCCAAATCAAGGTTTAGGT
AAACAACATCAAGGCATCATTTTGCCCTTGATTTAAAAACCTAATCAAGATCGAAAAGGCTT
GGGGTGTTTTCCCTAGGGACCTCTGATTCTCCTGTGACACATGCCGATCCTATTGATTGGAAA
TCGGAGGAACCGGTATGGGTCGATCAGTGGCCCCTAACACAGGAAAAACTTTCTGCCGCAC
AACAGCTGGTGCAGGAACAGCTGAGACTTGGGCATATTGAACCCTCTACCTCTGCGTGGAAT
TCCCCAATTTTTGTTATTAAAAAGAAGTCTGGGAAATGGAGATTGCTACAAGATCTTCGTAA
GGTAAATGAAACAATGATGCATATGGGAGCCCTACAACCTGGGTTGCCCACTCCTTCTGCTA
TACCTGATAAATCCTATATCATTGTTATAGATTTAAAAGATTGTTTTTACACTATTCCTCTTGC
ACCTCAAGATTGCAAAAGATTTGCTTTCAGTTTACCCTCTGTTAATTTTAAAGAGCCTATGCA
ACGCTATCAATGGAGAGTTCTCCCGCAAGGAATGACTAATAGCCCTACGCTGTGCCAAAAAT
TTGTTGCTACAGCAATAGCTCCGGTTCGTCAACGTTTTCCTCAGCTATATTTGGTTCATTATA
TGGATGATATATTACTAGCTCATGCTGACGAACATCTATTGTATCAAGCTTTTTCGATTCTAA
AACAACATTTAAGCCTTAATGGTCTTGTTATTGCTGATGAAAAAATTCAAACTCATTTTCCTT
ATAATTATTTGGGTTTCTCCTTATACCCTCGCGTTTATAATACCCAATTAGTAAAACTGCAGA
CTGACCATTTGAAAACTCTAAATGACTTTCAAAAACTTTTAGGAGACATTAATTGGATACGT
CCTTATTTAAAATTATCCACTTATACCTTGCAGCCATTATTTGACATCCTTAAAGGTGACTCT
GATCCTGCGTCACCCCGAACACTTTCTTTAGAAGGACGAACAGCTTTACAATCAATAGAAGA
AGCTATTAGACAACAACAGATTACTTATTGTGATTACCAACGATCATGGGGTTTGTATATAC
TTCCTACCCCCCGAACACCCACAGGGGTTCTCTATCAAGATAAACCTTTGCAATGGATATAT
TTGTCTGCTACTCCAACTAAACATCTGCTCCCTTACTATGAACTTGTTGCAAAAATTGTAGCA
AAGGGACGTCACGAGGCCATCCAATATTTTGGTATGGAACCCCCTTCATTTGTGTTCCTTATG
CTTTAGAACAACAAGATTGGCTTTTTCAATTTTCAGATAATTGGTCTATAGCTTTTGCAAATT
ACCCGGGACGGATTACTCATCATTACCCTTCTGATAAATTGTTACAATTTGCTAGCTCTCATG

CCTTTATTTTTCCAAAAATAGTTCGCCGACAACCTATTCCCGAAGCGACACTTATATTTACAG
ATGGATCTTCTAATGGAACTGCAGCTTTAATCATTAACCATCAAACCTATTACGCACAAACC
AGTTTTTCTTCTGCTCAAGTTGTGGAATTATTTGCAGTCCACCAAGCGTTGCTAACTGTACCT
ACTTCCTTCAATTTATTTACAGACAGCTCCTATGTGGTCGGTGCCTTACAGATGATTGAAACT
GTTCCAATTATTGGCACCACCTCTCCTGAAGTTCTTAACTTATTTACATTGATTCAACAGGTT
CTCCATTGCCGCCAACACCCCTGTTTCTTTGGACATATTCGTGCACACTCCACCCTTCCTGGT
GCCCTGGTACAAGGCAATCACACTGCGGACGTTTTTACTAAACAAGTGTTTTTTCAATCAGC
TATTGATGCAGCCCGAAAGTCCCATGATTTACATCACCAAAATAGTCATTCTTTACGCTTGCA
ATTTAAAATTTCCCGTGAAGCTGCACGACAAATTGTTAAATCTTGCTCTACCTGTCCTCAATT
CTTTGTTCTCCCTCAATATGGTGTCAACCCTCGAGGTTTACGCCCTAATCACCTCTGGCAAAC
AGATGTTACTCACATTCCTCAATTTGGGCATCTTAAATATGTTCATGTCTCTATTGACACTTTT
TCCAATTTTCTCATGGCCTCCCTTCACACTGGAGAATCAACACGTCACTGTATTCAACATTTG
CTGTTTTGCTTTTCTACTTCAGGAATCCCACAAACCCTTAAAACAGATAATGGACCTGGTTAT
ACTAGCCGTTCTTTTCAATGTTTTTGTCTTTCTTTCCAAATTCATCATAAAACAGGAATTCCTT
ATAATCCACAGGGACAAGGTATTGTGGAACGAGCCCATCAACGCCTTAAACATCAATTATTA
AAACAAAAAGGGGAATGAACTGTATAGCCCCTCACCGCATAACGCCTTAAACCATGCTCTTT
ATGTTTTAAATTTTTTAACTTTAGACGCAGAAGGCAATTCAGCAGCCCAGCGTTTTGGGGAG
AACGATCCTCATGCAAAAAACCACTTGTACGATGGAAGGATCCACTTACCAATCTGTGGTAT
GGGCCAGACCCTGTACTAATATGGGGACGAGGGCATGTTTGTGTTTTTCCACAGGATGCCGA
AGCGCCGCGCTGGATTCCGGAAAGGCTGGTACGCGCGGCAGAGGAACTCCCTGACACATCA
AATGCAACGCATGACACTGAGCGAGCCCACGAGTGAGCTGCCTACCCAGAGGCAAATTGAG
GCGCTGATGCGATATGCTTGGAATGAGGCTCATGTACAACCTCCAGTGACACCTACTAATAT
ACTGATCATGTTATTATTATTGTTACAGCGGATACAAAACGGGGCGGCTGCGGCTTTTTGGG
CATACATTCCTGATCCGCCTATGATTCAATCCTTAGGATGGGATAAAGAAACAGTACCTGTA
TATGTTAATGATACAAGTCTTTTAGGAGGAAAATCAGATATTCACATTTCTCCTCAGCAAGC
CAATATCTCCTTTTATGGTCTTACTACTCAATACCCTATGTGCTTTTCTTATCAATCACAGCAT
CCTCATTGTATACAGGTGTCAGCTGATATATCCTATCCTCGAGTGACTATTTCAGGCATTGAT
GAAAAAACCGGAAAGAGATCGTACCGTGACGGAACCGGACCCTCGACATTCCGTTTTGTGG
CAAACATTTAAGCATCGGCATAGGAATAGACACTCCTTGGACTTTATGTCGAGCACGAATTG
CATCGGTGTATAACATCAACAATGCCAATACCACCCTTTTATGGGACTGGGCACCTGGAGGA
ACACCTGATTTCCCGAATATCGAGGACAGCATCCACCCATTCTTTCTGTAAACACTGCTCCTA
TATATCAGACAGAACTGTGGAAACTTTTGGCTGCTTTTGGTCATGGTAATAGCCTATATTTAC
AACCCAATATTAGTGGGAGTAAATATGGTGATGTGGGAGTTACAGGATTTTTATATCCCCGA
GCTTGTGTTCCTTATCCATTCATGTTGATACAAGGCCATATGGAAATAACACTGTCATTGAAT
ATTTATCATTTAAATTGTTCTAATTGCATACTTACTAATTGCATTAGAGGTGTAGCCAAAGGA
GAACAAGTTATAATAGTAAAACAACCTCCTTTTGTAATGTTACCTGTTGAAATAACTGAAGA
ATGGTATGATGAGACTGCTTTAGAATTGTTACAACGCATTAATACGGCTCTTAGCCATCCTA
AAAGAGGTCTGAGCCTGATTATTCTGGGTATAGTGTCTTTAATCACCCTTATAGCAACTGCTG
TTACCGCTTCTGTATCTTTAGCACAATCCATTCAAGCTGCTCATACTGTAGATTCCTTGTCAT
ATAATGTTACTAAAGTAATGGGAACTCAAGAAGATATAGATAAAAAAATAGAAGATAGATTA
TCAGCTTTACATGATGTAGTTAGAGTTCTAGGAGAACAAGTTCAGAGCATTAATTTTCGCAT
GAAAATTCAATGCCATGCTAATTATAAATGGATTTGTGTTACAAAAAAGCCTTACAATACTT
CTGACTTTCCGTGGGATAAGGTGAAAAAACATCTGCAAGGAAGTTGGTTTAATACTACTGTT
TCTTTAGATCTTTTACAATTGCACAATGAAATTCTTGACATCGAAAATTCTCCAAAAGCTACT
TTGAATATAGCTGATACCGTCGATAATTTTTTACAAAATTTATTTTCTAACTTTCCTAGCCTTC
ATTCACTGTGGCGAAGTATAATTGCTATGGGCGCGGTTCTGACTGTTGTGCTTATCGTAATTT
GTTTAGCTCCTTGCCTTATTCGTAGCATTGTTAAAGAATTTTTACATATGAGAGTTTTAATAC

ATAAAAACATGTTGCAACACCAACATCTTATGGAGCTTTTAAAAAATAAAGAGAGGGGAGC
TGCGGGGGACGACCCGTGAAGGGTTAAGTCTTGGGAGCTCCCTGGCAGGTATGCCGGGCCC
TAGGACACGTGCCTAAGCTCCCTGTCCCGCCACCCTCAAGAACTTTTGTAACCCTTAAGGCT
CCAAGATGTTTGGTTTCGGCAACATTTCATAGAAGATAGATTATCTTATTGTGTATACTTCAT
AGAAGATAGATATTCTGATTGTGTTCTATATACAATGGTAAGGGTCTGGTGATTGTATCCTG
AGATTAAAAACAACCTTGTGAGTGCCTTAAGTTACGTACTTTACCCTATATATACCCCAGCA
CAATAAAGCAAGGTATCAGCCATTTTGGTCTGATCCTCTCAACCCCATCTTTTGTCTATCTCT
TATTTTCTTAGCGGGGACGCTCCGTTCTCTCCCCTGTGCAGGTGCGACTCTTGTTTGTGCTGG
CCGCGGCA

>NC_056054.1:98025573-98033196#SHEEP_RIP_07(+)

TGAAGGGTTAATAGGGTAGCAGAGATGTGCCTGCAAACGGGTCTCTCTGCTCATGCTGAGCG
TCCTTGCATACGAGGCTTTCTGCCAAAGAGTCTGGATACAGCCTTGAGTTTAATAGTCCCTTG
CAAACGAGGGAGCATTCCCTTCTTGAGATAAGAGGGAGATGAGGGCTTTGTGCAGACTCTG
CAGTAGACAGAGATTTCACTCCCCTTTGCTGTACGATAACATGTATGCACCTGCACTGTGCT
GAAAAGGCTTATTCTTACAGTCTGGAATTCTGCCTAAGGAGGGCTTTATAATAATAAACGGC
AATTAGTTTGCCCAGTTCTGTTCCTCTGGCCAGAGTGGTGTCCTGTCTGTCTCTTGTGTGTCTT
GTATGTTCTGTGTCATTTCACTCGTAGTAACCAACATCTGGCGCCCAACGTGGGGCTCAAGT
GAAACCGAAAGAGGTAAGAAACCCCGGGGGGGTTTTAGATCCATAGCACGGGAGCTTTCGG
AAACATGGGAATTCCTCACCCTAGCGGAAAAAATTGCTAGGTCAATTCTTGTGCATAATCAT
GGGGAATTCTTCACCCTAGCAGATGGGAACTTTCAAAAGTTCATGGTTCAAAAAACCATGGG
GAAATCCTGCTAGGAAGAAATAAATGTTCATTACGGGCACAATATGTGAATTTATATATTGA
ATCATGTTATAGAATTTATGTAATGCTATAACACAGGCTTTGACTTTGCTATCTACTGATAAT
GAGACTAAATCTAATGCTTTAATGAAGGGAGAGGCAATTTATGAGGATATGCCAAATGTTG
GTGGAATTTCTGCCTTGCCTGAAAGTAAGGATACAAATAAGCCTCCTTCTGGAAATGGTGAA
ACATCTGATAGTTCAGAATCAGATTCGGAGGCTTCTTCGGTTTCGTCAGAGGAGGGCAAAGA
GATTAAAGAAATGACCCATCTATTCCGGGAATAGTGGAAATCCCGTAAGGAGGAGAAAAAA
TCTACACCTTCTGCTCCTGCTTGTGCTTCTCTTCTCCCCACTGCAGTTGATCGGCCCGATGTGG
GCAGGGGACATTGTCCGTTCTCCTTTCCTTTGTCTATGCTTCACGATGATGACTTGTCTGCTC
CCCCTGGTGGGTTTATTGATCTTCCACAATTGTTTCCCATCCAGAGACAGCAGGATGGCAAT
GTGATGAATGTTCAATATGCTCCTTTGGAATATAAATTTTTTAAAGATCTTAAAGCTGCAATA
GCGCAATATGGTCCTCAATCTCCCTTTGTTTTGGCTATGCTGGAATCATTGGGAAAAGGCAA
ACTAATCATTCCATTAGATTGGGAATCCATTGCCCAAGCTGTCTTGGAGGGTTCTCAATGGTT
GCAACTTCGTAGCTGGTGGGAGGAAGAAGCTAGGAAGCAAGCTTGGATTAATGAGGGACAA
AATCCCCCTGGTCCTCTCGAGGACAAATTAATGGGAGAGGGCCAATATCAGGCTTTAAGAG
AACAGGCTCAATACTCTGACCAGGACTTACAACAAGTTCACCAGGTCTTTTTACGAGCATGG
TGCCGTGTGGTGCCTACTGGCCAAGCCCAGCCCTCCTTTGTTAAAACAATGCAAGGCCCTAA
TGAGCCATATACTGATTTTCTAGCAAGATTGAGGGTAACTGTGGAACGGGCTGTAGGGAGG
GATGAAATCTCAGGGATATTATTACAAACTTTGGCATTTGAAAATACAAATCCTGAATGCAA
GCGTATACTGGGATTTTAAAGGGACAGGGTGCCTCTATAGCTGAATATATCAGAGCCTGCTC
TGGAGTAGGAAGAACTGAGCACCAGGCTAATGTCTTTGCTACGGCCTTGGCCAAGGTTATAA
GACCACCAAAGGGAGGTAACTGCTTTCATTGTGGAAAACCTGGTCATATGAAAAAAGAGTG
TCAGAAATTAAAAGCTGATAAAGACAGATCTCTTGCTAGGAAGAATAAGGCTCCTCCTGGA
CTTTGCCGTCGGTGAAGGAGGGGATTTCATTGGACTAATAAATGCAAATCTAAAACAGACA
AAATGGGCAACCCGATACCGGGAAACTATCCTTCGGGCCTGAGTCCTTGGGGCCCAGGAAC
AATACCGGGGACTTCTCCTCCTTGCCCTCCTCCCATTCCATCTGCCCCGTTAAAAAGACCTCA
GATGATGATTTCGGACTTACGGTCTGCTACTTCAGGGAGTGCTGCTGCTGATTTGTCACTAGC

TGATAATGTTCTTTTGTCACCGAGGGGAGGCATTTATAAATTAAAAACAAATGTATTTGTAC
CACTGCCTAAAGGCACTTTTGGCTTAATATTAGGCCGTAGCAGCGGCTTTGAGAGGTTTA
ACCATAATTCCTGGGGTAATAGACTCTGATTATGTTGGGGAAATTTTAATTATGGTCTCTACT
TCTACCACACTTTCATTGTTAGCTAGAGAATGTATTGCCCAAATACTTCTCCTACCTTATCAC
CCCTTTTTGGCTCTTCCTAATAAACAAACAGGGGGATTTGGAAGTACTGGGCGAAATATATT
TGGGGAAATGCTTATCAGAGATTCTCACCCTGTTCTCCCCTTGATTATACAAGGAAACAACT
TTGAGGGACTAGTAGATACAGGGGCAGATGTTTCAATCATTTCTTCTCAGCAATGGCCCCAA
GGTTGGGAAAAGGAAAAAAGCCCTTTAATGCTGACGGGATTGGGCTCCATTGCAGATATTTG
GAAGAGTACCCATCCCTTGCAATGTCAATTCCATAATGGAAGATCAGTGTTTGTTACCTTTTA
TATTGTAAATATACCTATTAATATATGGGAGAGAGATCTCCTCTCTCCTTTGGGGGCTTCTGT
AACCATTCCATTGGAAAACTAGTGGCCACTGCTCAAATTCCTTGAGCACTCCCATTAAAATG
GTTAACTAATACTCCTAAATGGGTTGAGCAGTGGCCATTACCACAAATGAAGCTCGAGGCAT
TAGAACAAGTAGTACAAGAACAACTCCAACTTGGTCATATAGAGCCCTCTACCTCACCCTAA
AATTCTCCTGTTTTTGTTATTTAAAAAAATCTAAAAAATAAAAAATGTTCACTGATTCACAAG
AAGTTAATAAATGTATTGAACCTATGGGAGCATTGCAGTTGGGACTCCCCTCTCCAGCTCTT
ATTCCTCAAAATTGGTCCTTAATGGTGCTAGATCTTAAAGACTGTGTTTTTTTACCATTCCCC
TACAATTTCAAGATAGAAATAAATTTGCTTTTACAGTTCCTGTTCTTAATCATGCTCAACCTG
TTAAGCGTTATCAATGGACAGTCCTACCGCAAGGAATGATAAATAGTCCTACCTTATGTCAA
GAATTTGTAGCTCACTCTTTGCAATCTCTCCGCCAAGAATACCCTAATTATATTCTATATCAT
TATATGGATGATCTCCTATTGGCAGCTCCTAGTATTGCTGAACGTGACGAATTCTTTTTAAAA
GTACAAGAGGCTTTAAGACTACACAATTTGCAAATAGCCCCAGAAAAAATTCAAAAGGACT
TTCCTATTTCATATTTAGGGACAATATTGGAACAACATAGAATTAAGCCCCAAAAGTCACAA
ATTAGAAGAGACCATCTCAGAACCTTAAATGATTTTCAAAAATTATTGGGAGATATTAATTG
GCTACGCCCGGTACTTGGGATTCCTACTTATCAGTTATGACATTTGTTTTCTACTTTAGAAGG
AGATACAGCTTTCGATAGCCCCCGGACCTTAATCCCATTGGCTTTACAGGAACTTCAACTTGT
TGAACAACGACTGAATGACAGCTTTTTGACTGACTTACATGCATCTCAACCTATTTCTTTTAT
CATATTTCATACCCCTTATTCCCCATCTGGTGTAATTGCTCAAGAAGAAGGATTAATAGAAT
GCATTTTCTTACCTAACAGTTTTTCCAAAAAATTGACTATATATATGGATAAATTAGCCTTCC
TTATACAGAGAAGTCACCATCATATTTTACAATTATCAGGATGTGAACCACACCAGATTGTT
ACTCAGTTAACAACTGCTCAAATATCTCGATGTTTACAATTTAATGAAAACTGGCAAATTTCT
CTTGCTTCATATCCTGGTTCGTTTTCTAATCATTATCCATCATCTAAATTGATTGATTTTCTCC
AGACTAACACTATGATATCTCATTCCCCAATTTCAGATGTTCCAGTTAAAGGACCTACTATTT
TTACAGATGCAAATAAAAATACTGCTAGATATTGGACCCCAGAAAATTCCAAGGTTCTCCCC
CACTCATTTTCTTCTGTACAGCCTGCTGAATTGTGGGCTATCTAGTTTTACAAGATTTTCCCC
AACTTCCTATTAACATTGTTTCAGATTCTCGATATGCTGTTCTCTCTTGCCTACAGCTTCCTCA
TGTCTCCCTTCCACTGACTCTTAAAACAGCTATTGATAAATTGTTTTACCAAGTACAACAATT
ACTCTTGCAACTTTCAGAGTTAATTTTCTTTACTCACATCCATGCACATTCTGCCCTTCCTGGA
CCCTTATCATTTGGAAATGCTACAATTGATGCCTTACTTAATCCTATAGAAGCAGCAAAGCA
AAAACATCTCTTACAACATACCAACTCCAAAGGGTTAGAAAAATCTCACACTATTACTCAAA
AACAAGCTCAAAATATTGTTCGTTCTTGTTCCATATGTGCACCCTTTGCTTTGCCATTTACCTC
ACCAGGTGTCAACATGAGAGGACAACAAGAAAATCAGATATGGCAAATGAATGTAATTTAC
ATTTCTTCCTTCGGACAACAAAAATGTGTGGGCAACTGCATTACATTCTAAAAAGGCTGACG
CTGAGCTGTTATTACTCATTTGTTATCTTGTTTTGCAGTTATGGGATTACCAATTGAATTGAA
AACTGATAATGCACCTGCTTACCAATCCGCAAAATTAGCTCACTTTTTATCTCAATACCATAT
AACTCATACTTTTGGTAATCCTTATAATAGTCAAGGGCAAGCTATTATTGAAAGAGCTAATC
ATACCTTGCATGATTATCTTGAAAAAATAAAAAAGGGGGAACAAGAGAGATTTATGAAACC
TAAAGACATTCTGAATAAAACCTTACTTACCCTAAATTTTTTGAATGTTTGGAGCAGGGGAA

ATCTATCAGCAGCAGAGTTGCACTTTCAAGGGAAAGAAGAGGATAAGAAGATCTTGAATAC
ACCTATTTGGTATAAAGATAAAGAGAACGGCTGGATCCCAGCATCATTAATATATTTGGGGC
AAGGGTATGCTTTCATTTCTGTTGATAATTACAGGTTTTGGACCCCAGCAAGATTGATCAAA
ATCAGCAATGGTTAATCCTCTTGTTCAGAAATTTGAAGAGCTTACTATGCAGAGAAGCCTTA
CCTCCCATACAAGGGAAGCAACACCTCCTACGTGGGGTCAAATGAAGAGGTTGACCCAGGA
AGCAGAGAAGACATTAATGAAGGTGGGGCAACCTCTGAATCCTACCAATCTTTTGCTTGCCA
CGATGGTGGTGGTGACATGTCAGGTAATCGGCGTATCAGCAAGTAATTATACATATTGGGCA
TATATACCTAATCCCCCATTAGTAAGAGCAGTTTCCTGGGGGGAACCAGAAGTGCAGGTATG
TACTAATGAGACTGCCTTCTTTCCCCTGCCAGCTTGCAGGGGAATAGAACAACTATATCATC
ATAAACAACAATATAATATTAGTAATTTGACCACTGCGGTGGAAGGTATTCCTTTATGTATA
GGGGGACACCCCTTTTGTCTGTCCACCAAGGAACATTCTCATCATTCTTATAATACATGGGG
GGTAAAGTATAATAATTACCATTTTGCTACTTTTACTGTGCTTGTTTCCACCAGGGGATTTAG
TACCTCGACAGAACCGATAGACATTCATAATGGAAAACACATGTCACTATGTCCTGTTAACC
TTTTCGTTCCTTCTCTAGAATCTTTGGAGTGGGAACGTTGCCGAGGCCATCCACCCTTTAAGG
TCATGAATTATTCTGGGGCCATCATTGTAGATTGGAGTCCAGATCATGGACAATTCTTAGAA
AAATGGTCAAATAAACCTTTTAGGTGGCATCGCGCAAATACCACTTTGATGGGCAATGGTAA
CGAAACAGTTAAATGGCAGCAATTTGCACTTGTCCCTCCTCAATTACAATTGCAAGGATATC
CTCACATTCAAGGAGATATTTGGAAACTATGGGCGGTTTCTGGTAATCTCACTATCTGGTCA
GGAAATTATACTTTGGACAGTGGTGACTCTTCGGGTTCATTCCATGTTAATTTACATGTTAAT
AAATCTTATTCCACAATGGCATGTGTAAAATATCCTTTTGCATTGTTATATAGGAATTGGACC
TGGAATGATACTCTGGAGTCTGTGTCAAGTAACTATTGTAATTTAACTCAATGTATAAATGTC
TTGGTGGGAAGAATTTGAAAGACAAGCCTTTAACTCCAATTTCTCGCTAGTAATTGTTAAAG
CTCAGACAGAATTATGGTTGTCTATAAATCTGACTCAGCCGTGGTCAGATCCTTTTGCTGCTT
CTCATCTAGTAACCGCTGTACAGACTTTGCTACACCGATCTCGACGTATGCTTGCTGTGGTCA
TTGCTTCGATTCTCGCAGTCGCATCAGTAACTGAAACAGCAGCTGTAGCAGGCCTTGCGTTA
CACTAAGGAATTCAAACAGCTGATTTTGTTCGGGACTGGCAAAAGGACTCTCATTTGTTATG
GCAACAACAGCAAGATTTGGATGCACAACTTGCTACCGACGTGCTTAATCTTCAAACACACC
GTTTCCTGGCTTGGAGATCAATTGGCTGTTTTATCTACATGAAGTGTGTTGAAATGTGATTGG
AATTCTTCTGTTTTGTATAACACCTGTACCATTTAACATGAGCCCTTCAAGAGATTAACCGAG
ACATATAAATAAAAAGAGTTCTAGATGCCCATCATCTAGAGAGATTCCGCCTAGCCCCTAAG
AGGTGGCTGGATAGCCAATGACGGGTAAGACCCTCAGAGGAGGGCAATCTAAGACAGGCAC
AGCCGCAAGAGGGGCTGGCGAGGCTGGAGGTTGGCCGCCTACAGCTTTATGCCTTGCTCTAC
AAAACATCAATACAAATGTCTCGAGGGCTTGAATACAATAAAAAAGGGGGAGATGAAGGGT
TAATAGGGTAGCAGAGATGTGCCTGCAAATGGGTCTCTCTGCTCATGCTGAGCGTCCTTGCA
TACGAGGCGTTCTGCCAAAGAGTCTGGATACAGCCTTGAGTTTAATAGTCCCTTGCAAACGA
GGGAGCATTCCCTTCTTGAGATAAGAGGGAGATGAGGGCTTTGTGCAGACTCTGCAGTAGAC
AGAGATTTCACTCCCCTTTGCTGTACGATAACATGTATGCACCTGCACTGTACTGAAAAGGC
TTATTCTTACAGTCTGGAACTCTGCCTAAGGAGGGCTTTATAATAATAAACGGCAATTAGTTT
GCCCAGTTCTGTTCCTCTGGCCAGAGTGGTGTCCTGTCTGTCTCTTGTGTGTCTTGTATGTTCT
GTGTCATTTCACTCGTAGTAACCAACA

>NC_056056.1:39411628-39419559#SHEEP_RIP_10(+)

TGCAGGGGACGACCCGTGAAGGGTTAAGTCTTGGGAGCTCCCTGGCAGGTATGCCGGGCCC
TAGGACACGTGCCTAAGCTCCCTGTCCCGCCACCCTCAAGAGTTTTTATAACCCTTAAGGCTC
CAAGATGTTTGGTTTCGGCAACATTTCATAGAAGATAGATTATCTTATTGTGTATATTTCATA
GAAGATAGATATTCTGATTGTGTTCTGTATACAATGGTAAGGGTCTGGTGATTGTATCCTGA
GATTAAAAAACAACCTTGTGAGTGCCTTAAGTCACGTACTTTACCCTATATATACCGCAGCA

CAATAAAGCAAGGTATCAGCCATTTGGGGCTGATCCTCTCAACCCCATCTTTTGTCTATCTCT
TATTTTCTTAGCGGGGACGCTCCGTTCTCTCCCTGTGCAGGTGTGACTCTTGCTTGTGCTGGC
CGCGGCAGGTGGCGCCCAACGTGGGGCTCGAGCTCGACAGTTTTCCTCGCCACTACTCTTAT
TAATTGAAAAGAGTGAGTATATGAGTAAACAAGTGAATTAATTTGAGGAGGAGTAGTAAGG
TATATAGTTGAGAGTATAAATATGGGACAGACGCATAGTCGCCAGTTGTTTGTGCATATGTT
ATCTGTAATGTTAAAACATAGGGGAATTACTGTTTCTAAACCTAAATTAATCAATTTTCTTTC
ATTCATCGAGGAAGTTTGCCCTTGGTTCCCCGGAGAAGGTACAGTAAATTTAGAGACATGGA
AGAAGGTAGGGGAACAAATTCGGACTCATTATACTTTACATGGCCCTGAAAAAATCCCTGTC
GAAACTTTATCCTTTTGGACACTAATTCGTGACTGCCTGGACTTTGATAATGATGAATTAAAA
CGTTTAGGAAATTTATTAAAACAGGAAGAAGATCCTCTCCATGTTCCTGATTCGGAACCCAG
ATATGCTGTTCCCGAGGGGGTTAAAAGCGACCCTCCGTTTTCTAACTTATTGCATCCTTCAGA
TAATGATGATTTACTTTCATCCACAGATGAGGCAGAATTAGACGAAGAAGCTGCTAAATACC
ATCAAGAAGATTGGGGTTTTTAGCACAAGAAAAGGGGCGTTAACATCTAAAGATGAATTGG
TTGAATGCTTTAAAAACCTCACTATTGCTTTACAGAACGCAGGAATCAAGCTTCCTAGTAAC
AATGCCAAATCTCCTTCTGCTCCGCCTCTTCCCCCTGCTTATGCTCCTTCTGTTGTGGCTGGTC
TCGATCCCCCTCCAGGGCCCCCTCCACCGTCTGAGAACATGTCTCCGCTGCAAAAGGCATTG
AGACAGGCACAGCGACTTGGTGAGGTTGTCTCTGATTTTTCTCTTGCTTTTCCTGTCTTTGAA
AATAACAACCAGCGTTATTATGAATCACTGCCTTTTAAACAACTGAAAGAGTTAAAGATTGC
TTGCTCACAATACGGTCCTACCGCTCCATTCACCATTGCTATGATAGAAAATTTGGGTACTCA
AGCTTTACCTCCAAATGATTGGAAGCAGACAGCTAGGGCATGTCTCTCAGGGGAGATTATTT
ATTATGGAAATCTGAATTTTTTGAACAATGTGCTCGTATAGCTGATGTTAACCGACAGCAAG
GTATACAGACCTCCTATGAAATGTTGATTGGTGAAGGCCCTTACCAGGCTACTGACACTCAA
CTTAATTTCTTACCTGGTGCATATGCACAAATATCAAATGCGGCTCGGCAGGCATGGAAAAA
CTTCCTAGCTCCAGTACTAAGACAGAGGATCTTTCAAAAGTCCGGCAGGGACCTGATGAGCC
TTACCAGGACTTCGTGGCACGACTTTTAGATACTATAGGTAAGATAATGTCAGATGAAAAGG
CTGGGATGGTATTGGCAAAACAATTGGCTTTTGAAAACGCTAACTCTGCTTGTCAAGCTGCT
TTAAGACCTTATCGAAAAAAGGGAGATCTGTCTGATTTTATTCGCATTTGTGCTGACATTGG
ACCCTCCTACATGCAAGGCATTGCTATGGCAGCAGCATTACAAGGAAAAAGCATAAAGAGG
TACTTTTCCAGCAGCAAGCCCGGAACAAGAAAGGACTTCAAAAGTCAGGTAATTCGGGTTG
CTTTGTTTGTGGTCAGCCTGGCCATCGGGCTGCAGTGTGCCCTCAAAAACAACAAAGCCCTG
TTAACACTCCTAATTTGTGCCCACGCTGTAAAAAAGGAAAGCATTGGGCGCGGGACTGCCGT
TCCAAAACGGATGTTCAAGGTAATCCTTTGCCCCCGGTTTCGGGAAACTGGGTGAGGGCCAG
CCCTGGCCCCGAAACAATGTTATGGGGCAACACTGCAGGTTCCAAAAGGACCATTGCAGAC
CTCTGTCGAGCCACAAGAGGCAGCGCGGGATTGGACCTCTGTGCCACCTCCTACACAGTATT
AACTCCCGAGATGGGGGTCCAAACCCTTGCCACAGGAGTGTTTGGGCCTTTACCTCCAGGGA
CAGCTGGACTGCTTTTAGGGCGCAGCAGTGCGTCTTTAAAAGGAATACTTATTCATCCTGGT
GTGATTGACTCTGATTATACAGGAGAGATAAAAATATTAGCCTCCGCTCCTAACAAAATTAT
TGTGATCAATGCAGGACAGCGTATAGCTCAACTTCTTTTAGTTCCATTAGTCATACAAGGAA
AAACAATTAACCGAGACCGTCAAGATAAAGGTTTCGGGTCCTCTGACGCCTTTTGGGTGCAA
AATGTTACCGAGGCACGACCAGAACTTGAGCTACGCATTAATGGTAAGCTTTTCCGCGGAGT
GCTTGATACAGGGGCCGATATTAGTGTTATTTCTGATAAATATTGGCCTACTACATGGCCAA
AACAGATGGCTATTTCCACTCTCCAGGGTATTGGCCAAACTACCAATCCAGAACAGAGTTCA
TCCCTTCTTACTTGGAAGGATAAAGATGGACATACAGGCCAATTTAAACCTTATATTCTGCC
CTATCTTCCAGTTAATCTATGGGGGCGTGATATATTAAGCAAAATGGGTGTTTATTTATATAG
TCCTTCACCCACTGTGACAGATTTGATGTTAGATCAGGGCTTACTTCCAAATCAAGGTTTAGG
TAAACAACATCAAGGCATCATTTTGCCCCTTGATTTAAAACCTAATCAAGATCGAAAAGGCT
TGGGGTGTTTTCCTAGGGACCTCTGATTCTCCTGTGACGCATGCCGATCCTATTGATTGGAAA

TCTGAGGAACCGGTATGGGTCGATCAGTGGCCCCTAACACAGGAAAAACTTTCTGCCGCACA
ACAGCTGGTGCAGGAACAGCTGAGACTTGGGCATATTGAACCCTCTACCTCTGCTTGGAATT
CCCCAATTTTTGTTATTAAAAAGAAGTCTGGGAAATGGAGATTGCTACAAGATCTTCGTAAG
GTAAATGAAACAATGATGCATATGGGAGCCCTACAACCTGGGTTGCCCACTCCTTCTGCTAT
ACCTGATAAATCCTATATCATTGTTATAGATTTAAAAGATTGTTTTTACACTATTCCTCTTGC
ACCTCACGATTGCAAAAGATTTGCTTTCAGTTTACCCTCTGTTAATTTTAAAGAGCCTATGCA
ACGCTATCAATGGAGAGTTCTCCCGCAAGGAATGACTAATAGCCCTACGCTGTGCCAAAAAT
TTGTTGCTACAGCAATAGCTCCCGTTCGTCAACGTTTTCCTCAGCTATATTTGGTTCCTTATAT
GGATGATATATTACTAGCTCATGCTGATGAACATCTATTGTATCAAGCTTTTTCGATTCTAAA
ACAACATTTAAGCCTTAATGGTCTTGTTATTGCTGATGAAAAAATTCAGACTCATTTTCCTTA
TAATTATTTGGGTTTCTCCTTATATCCTCGTGTTTATAATACCCAATTAGTAAAATTACAGAC
TGACCATTTAAAAACTCTAAATGACTTTCAAAAACTTTTAGGAGACATTAATTGGATACGTC
CTTATTTAAAATTACCCACTTATACCTTGCAGCCATTATTTGACATCCTTAAAGGTGACTCTG
ATCCTGCGTCACCCCGAACACTTTCTTTAGAAGGACGAACTGCTTTACAATCAATAGAAGAA
GCTATTAGACAACAACAGATTACTTATTGTGATTACCAACGATCATGGGGTTTGTATATACTT
CCTACCCCCCGAACACCCACAGGGGTTCTCTATCAAGATAAACCTTTGCGATGGATATATTT
GTCTGCTACTCCAACTAAACATCTGCTCCCTTACTATGAACTTGTTGCAAAAATTGTAGCAAA
GGGACGTCACGAGGCCATCCAATATTTTGGTATGGAACCCCCCTTCATTTGTGTTCCTTATGC
TTTAGAACAACAAGATTGGCTTTTTCAATTTTCAGATAATTGGTCTATAGCTTTTGCAAATTA
CCCAGGACGGATTACTCATCATTACCCTTCTGATAAATTGTTACAATTTGCTAGCTCTCATGC
CTTTATTTTTCCAAAAATAGTTCGCCGACAACCTATTCCCGAAGCGACACTTATATTTACAGA
TGGATCTTCTAATGGAACTGCAGCTTTAATCATTAACCATCAAACCTATTACGCACAAACCA
GTTTTTCTTCTGCTCAAGTTGTGGAATTATTTGCAGTCCACCAAGCGTTGCTAACTGTACCTA
CTTCCTTCAATTTATTTACAGACAGCTCCTATGTGGTCGGTGCCTTACAGATGATTGAAACTG
TTCCAATTATCGGCACCACCTCTCCTGAAGTTCTTAACTTATTTACATTGATTCAACAGGTTC
TCCATTGCCGCCAACACCCCTGTTTCTTTGGACATATTCGTGCACACTCCACCCTTCCTGGTG
CCCTGGTACAAGGCAATCACACTGCGGACGTTCTTACTAAACAAGTGTTTTTCCAATCAGCT
ATTGATGCAGCCCGAAAATCCCATGATTTACATCACCAAAATAGTCATTCTTTACGCTTGCA
ATTTAAAATTTCCCGTGAAGCTGCACGGCAAATTGTTAAATCTTGCTCTACTTGTCCTCAATT
CTTTGTTCTCCCTCAATATGGTGTCAACCCTCGAGGTTTACGCCCTAATCACCTCTGGCAAAC
AGATGTTACTCACATTCCTCAATTTGGGCGTCTTAAATATGTTCATGTCTCTATTGACACTTTT
TCCAATTTTCTCATGGCCTCCCTTCACACTGGAGAATCGACACGTCACTGTATTCAACATTTG
CTGTTTTGCTTTTCTACTTCAGGAATCCCACAAACCCTTAAAACAGATAATGGACCTGGTTAT
ACTAGCCGTTCTTTTCAACGTTTTTGTCTTTCTTTCCAAATTCATCATAAAACAGGAATTCCTT
ATAATCCACAGGGACAAGGTATTGTGGAACGAGCCTATCAACGCCTTAAACATCAATTATTA
AAACAAAAAAAGGGGAATGAACTGTATAGCCCCTCACCGCATAACGCCTTAAACCATGCTC
TCTATGTTTTAAATTTTTTAGCTTTAGACGCAGAAGGCAATTCAGCAGCCCAGCGTTTTTGGG
GAGAACGATCCTCATGCAAAAAACCACTTGTACGATGGAAGGATCCACTTACCAATCTGTGG
TATGGGCCAGACCCTGTACTAATATGGGGACGAGGGCATGTTTGTGTTTTTCCACAGGATGC
CGAAGCGCCGCGCTGGATTCCGGAAAGGCTGGTACGCGCGGCAGAGGAACTCCCTGACACA
TCAAATGCAACGCATGACACTGAGCGAGCCCACGAGTGAGCTGCCTACTCAGAGGCAAATT
GAGGCGCTGATGCGTTATGCTTGGAATGAGGCTCATGTACAACCTCCAGTGACACCTACTAA
TATACTGATCATGTTATTATTATTGTTACAGCGGATACAAACGGGGCAGCTGCGGCTTTTGG
GCATACATTCCTGATCCGCCTATGATTCAATCCTTAGGATGGGATAAAGAAACAGTACCTGT
ATATGTTAATGATACAAGTCTTTTAGGAGGAAAATCAGATATTCACATTTCTCCTCAGCAAG
CCAATATCTCCTTTTATGGTCTTACTACTCAATACCCTATGTGCTTTTCTTATCAATCACAGCA
TCCTCATTGTATACAGGTGTCAGCTGATATATCCTATCCTCGAGTGATTATTTCAGGCATTGA

TGAAAAAACCGGAAAGAGATCGTACCGTGACGGAACCGGACCCCTCGACATTCCGTTTTGT
GACAAACATTTAAGCATCGGCATAGGAATAGACACTCCTTGGACTTTATGTCGAGCACGAAT
TGCATCGGTGTATAACATCAACAATGCCAATACCACCCTTTTATGGGACTGGGCACCTGGAG
GAACACCTGATTTCCCCCGAATATCGAGGACAGCATCCACCCATTCTCTCTGTAAACACTGC
TCCTATATTTCAAACTGAACTGTGGAAACTTTTGGCTGCTTTTGGTCATGGCAATAGTCTATA
TTTACAGCCCAATATTAGTGGGAGCAAATATGGTGATGTGGGAGTTACAGGATTTTTATATC
CCCGAGCTTGTGTTCCTTACCCATTCATGTTGATACAAGGCCATATGGAAATAACGCTGTCAT
TGAATATTTATCATTTAAATTGTTCTAATTGCATACTTACTAATTGCATTAGAGGTGTAGCCA
AAGGAGAACAAGTTATAATAGTAAAACAACCTGCTTTTGTAATGTTACCTGTTGAAATAACT
GAAGAATGGTATGATGAAACTGCTTTAGAATTGTTACAACGCATTAATACGGCTCTTAGCCG
TCCTAAAAGAGGTCTGAGCCTGATTATTCTGGGTATAGTGTCTTTAATCACCCTTATAGCAAC
TGCTGTTACTGCTTCTGTATCTTTAGCACAATCCATTCAAGCTGCTCATACTGTAGATTCCTT
GTCATATAATGTTACTAAAGTAATGGGAACTCAAGAAGATATAGATAAAAAAATAGAAGAT
AGATTATCAGCTTTATATGATGTAGTTAGAGTTTTAGGAGAACAAGTTCAGAGCATTAATTT
TCGCATGAAAATTCAATGCCATGCTAATTATAAATGGATTTGTGTTACAAAAAGCCTTACAA
TACTTCTGACTTTCCGTGGGATAAGGTGAAAAAACATCTGCAAGGAATTTGGTTTAATACTA
ATGTTTCTTTAGATCTTTTACAATTGCACAATGAAATTCTTGACATCGAAAATTCTCCAAAAG
CTACTTTGAATATAGCTGATACCGTCGATAATTTTTTACAAAATTTATTTTCTAACTTTCCTAG
CCTTCATTCACTGTGGCGAAGTATAATTGCTATGGGCGCGGTTCTGACTGTTGTGCTTATCAT
AATTTGTCTAGCTCCTTGTCTTATTCGTAGCATTGTTAAAGAATTTTTACATATGAGAGTTTT
AATACATAAAAACATGTTGCAACACCAACATCTTATGGAGCTTTTAAAAAATAAAGAGAGG
GGAGCTGCAGGGGACGACCCGTGAAGGGTTAAGTCTTGGGAGCTCCCTGGCAGGTATGCCG
GGCCCTAGGACACGTGCCTAAGCTCCCTGTCCCGCCACCCTCAAGAGTTTTTATAACCCTTA
AGGCTCCAAGATGTTTGGTTTCGGCAACATTTCATAGAAGATAGATTATCTTATTGTGTATAT
TTCATAGAAGATAGATATTCTGATTGTGTTCTGTATACAATGGTAAGGGTCTGGTGATTGTAT
CCTGAGATTAAAAAACAACCTTGTGAGTGCCTTAAGTCACGTACTTTACCCTATATATACCG
CAGCACAATAAAGCAAGGTATCAGCCATTTGGGGCTGATCCTCTCAACCCCATCTTTTGTCT
ATCTCTTATTTTCTTAGCGGGGACGCTCCGTTCTCTCCCTGTGCAGGTGTGACTCTTGCTTGTG
CTGGCCGCGGCA

>NC_056057.1:33404754-33412479#SHEEP_RIP_11(+)

ACTCTGCAGTAGACAGAGATTTCACTCCCCTTGCTGTATGATAACATGTATGCACCTGCGCT
GTACTGAAAAGGCTTATTCATGCAGTCTGGAATTCTGCCTAGGGGGCTTTATAAAAATAAAC
CACAATTAGTTTTGTCCAGTTTTTTTCCTCCGGCCGGAGTGGTGTATTGTCTGTCTCTTGTGTG
TCTCGTGTGTTTTGTCTTTGTGTCATTTCGCTCACGACATCTGTCGCCCAACGTGGGGCTTGA
GTGAAACCGAAAGGGTGAGTAACCCCGGGGGGATTTTAAATCCATAGCAGGGGAGCTTTCG
GGAAAATCATGGGGAATTCCTCACCCTAGCAGGGTAACTTTCGGAAAATCATGGGGAATTCC
TCATCATCATTTCAGACACAATACAGGGAGTTAGTCAAAGGACTTCTCCACTCCATAGGCGT
TAAGGTCTCGACTCATCGATTGAGTGAGCTCTTTCGCTTGGTGGAGCAATGTTGTCATTGGTT
TCAATATCAAACTAAGTTACAGTTAAATTTGAAGGAGTGGAAAATAATTCAAAAGGAATTG
AGAAAGCAACATCAGAAGGGTAATGTGATCCCTTTGAAGTTATGGACTATGTGTAGTGCTAT
AACACAGGCTTTGACCTTGCTTTCTACTGACAGTGAAACTAAATCTAATGCTTCAAGGAGGG
GAGAAATAATTTATGAGGATGTGTCAGACGTTGGTGGGGCTTCTGCATCGCCTGAAGGCAAG
GATACAGGTGAGTCTCCTCCTGTAAATGGTGAAACATCTAATAGTTCAGAATCAGATTCAGA
GGCTTCTTCGGTTTCGTCAGAGGAGGGCAAAGAGATGAAAGAAATGACCCATCTATTCCGG
GAATGGTGGAAATCCCGTAAGGAGGAGAAAAAATCTACACCTTCTGCTCCTCCTTGTGCTTC
TCTTTTCCCCACTGCGGTTGATCGGCGCGATGTGGGCAGGGAACATTGTCAGTTCTCCTTTTC

TTTGTCAATGCTTCATGATGATGACTTGCCTGCTCCCCCTGGTGGGTTTATCGATCCTCCACA
ATTATTTCCCATCCAGAGACAGCAAAATGACAACGTGATAAATATTCAATACACTCCTTTGG
AATATAAATTTTTTAAAGATCTTAAAGCTGCAGTAGCACAATACAGTCCTCAATCTCCCTTTG
TTTTGGCTATGCTGGAATCACTGGGAAAAGGCAAATTAATCATTCCGTTAGATTGGGAATCT
ATTGCCCAAGCTGTCTTGGAGGGCTCTCAATGGTTGCAACTTCGTAGCTGGTAGGAAGAAGC
TAGAAAGCAGGTTCAGATTAATGAGGGACAGAATCCCCCCGGTCCTCTCGAGGACAAGCTA
ATGGGAGAGGGCCATTATCGGGCTTTAAGAGAACAGGCTCAATACTCTGATCAGGACTTAC
AACAAGTCCACCAGGTCTTTTTAAGAGCATGGCGCCGTGTGGTGCCTACTGGCCACGCCCAG
CCCTCCTTTGTTAAAACAATGCAAGGCCCCAATGAGCCTTATACTGATTTTCTAGCAAGGTTG
AGGGTAGCTGTGGAATGGGCTGTAGGGAGGGATGAGATTTCAGAGATATTATTACAAACTTT
AGCATTTGAAAATGCAAATCCTGAATGCAAGTGTATACTGGCACCATTAAAGGGACAGGGT
GCACCTATAGCTGAATATATCAGAGCCTGCTCAGGAGTAGGAGGAACTGAGCATCAGGCTA
ATGTCTTTGCTATAGCCTAGGCCAAAGCTATGAGACCACAAAAGGGAGGTAACTGCTTCCAC
TGTGGAAAACCTGGTCATATGAAAAGAGTGTGTCAGAAATTAAAAGCTGATCAAGGTGCAA
TTCCTAAAGACAGATCTCTTGCTGGGAAGAATAAGACTCCTCCTGGACTTTGCCGTCGGTGC
GGGAAGGGGCTTCGTTGGACTAATGAATGCAGATCTAAAACAGACAAAATGGGCAACCTGA
TACTGGGAAACTATCCTGCGGGCCTAAGTCCTTGGGGCCCAGGAACATTACTGGGGACTTCT
CCTCCTTGCCCTCCTCCCATCCCATCTGCCCCAACCCTATTCCCTCCCAACAACCGTTACGAG
TTGATGCCCCATTATAAGGACCTCAGATGATGATTTCGGACTTACGGTCTGCTACTTCAGGG
AGTGCTGCTGCTGATTTGCCACTAGCTGATAATGTTCTTTTGTCACCAGGGGGAGGTATTTAC
AAATTAAAAACAAATGTATTTGGACCACTGCCTAAAGGCACTTTTGGCTTGATATTAGGCTG
TAGCAGCGTGGCTTTGAGAGGTTTAACCATAATTCCTGGGGTAATAGACTCTGACTATGTTA
GGGAAATTTTAATTATGGTCTCTGCTTCTACCAAGCTTTCATTGTTAGCTGGGGAGCGTATTG
CTCAAATACTTCTCCTATCTTATCACCCCTTTTTGGCTCTTCCTAATGAACGAACAGGAGGAT
TTGGAAGTACTGGGTGGCATATATTTTGGAAAATGCTTATCGAAGATTCTTGCCCTGTTCTCT
CCTTGATTATACAAGGAAACAACTTTGAGGGACTAGTAGACACAGGGGCAGATGTTTCAGTC
ATTTCTTCTCAACAATGGCCCCAAGATTGGAAAAAAGAAAAAAGCCCTCTAATGCTGACGG
GACTGGGCTCCATTGCAGACGTCTGGAAGAGTACCCATCCCTTGCAATGCCAATTCCATAAT
GGAAGATCAGTGTTTGTTACCTTTTATATTGTACACATACCTATCAATATATGCGGAAGAGA
TCTTCTCTCTCCTTTGGGGGCTTCTGTAACCATTCCATCGGAAAACTAGTAGCCACTGCTCAA
ATTCCTCGAGCACTCCCATTAAAATGGTTAACTAATACTCCAAAATGGGTTGAGCAGTGGCC
ATTACCACAAGTGAAGCTAAGAGGCATTAAAACAATTAATACAAGAACAACTTCAACTTGG
TCATATAGAGCCCTCTACCTCCCCCTGGAATTCTCCTGTTTTTGTTATAAAAAAGAAATCTGG
AAAATGGAGAATGTTAACTGATCTACGAGAAGTTAATAAATGTATTGAACCTATGGGAGCA
TTGCAATTGGGACTCCCCTCTCCAGCTCTTATTCCTCAGAATTGGTCCTTAATGGTGCTAGAT
CTTAAAGACTGTTTTTTTTTACCATTCCCCTACAATTGCAAGATAGAGATAAATTTGCTTTTA
CAGTTCCTGTTCTTAATCATGCTCAGCCTGTTAAGCGTTATCAGTGGACAGTCTCACCACAAG
GAATGATAAATAGTCCTACCTTATGCCAAGAATTCGTAGCTCGCTCTTTACAATCCCTCCATT
GAGAATACCCCAATTATATTCTATATCATTATATGGATGATCTCCTATTAGCAGCTCCTAATA
TTGCTGAACGTGATGAATTCTTTTTAAAAGTACAGGAGGCTTTAAGACTATACAATTTGCAA
GTAGCCCCAGAAAAAATTCAAAAGGACTTTCCTATTTCGTATTTAGGGACAATATTGGAACA
ACATAGAATAAGGCCCCAAAAGTTGCAAATTAGAAGAGACCATCTCAAAACCTTAAATGAT
TTTCAAAAGTTATTGGGAGATATTAATTGGCTACGCCCGGTACTTGGGATTCCTACTTATCAA
TTACGACATTTGTTTTCTGCTTTAGAAGGAGATACAGCTCTGGATAGCCCCCGGACCTTAACC
CCATTGGCTTTACAGGAACTTCAATTTGTTGAGCAACGACTAAATGACAGCTTTTTTACTTAC
TTACATGTGTCTCAATCTATTTCGTTTATAATATTTCATACCCCTTATTCTCCATCTGGTGTAA
TTGCTCAAGAAAAAGGATTAACAGAATGGGTTTTCTTACCTAACAGTTTTTCCAAAAAATTG

ACTACATATATGGATAAATTAGCCTTCCTTATACAGAAAGGTCGCCGTCGTATTTCACAATT
ATCAGGATGTGAACCACACCAGATTGTTACTCAGTTAACAACTGCTCAAATATCTCGATGTT
TACAATTTAATGAAAACTGGCAAATTTCTCTTACCTCATATCCTGGTTCATTTTCTAATCATT
ATCCGTCATCTAAATTGATTGATTTTCTTCGGACTAACACTATGATATCTCATTTCCCAATTTC
AGATGTTCCAGCTAAGGGGCCCACTATTTTTACAGATGCAAATAAAAATACTGCTGGATATT
GGACCCCGGAAAGTTCCAGGGTTCTCCCCCACTCATTTTCTTCTGTACAGCCCACTGAATTGT
GGGCTATCTATTTAGTTTTGCAAGATTTTCCCCAACTTCCTATTAACACTGTTTCAGATTCTTG
ATATGCTGTTCTCTCTTGCCTACAGCTTCCCCATGTCTCCCTTCCACTGACCCTTAAAACAGC
TATTGATGAATTGTTTTACCAAGTACAACAATTGCTCTTGCGATGTTCAGAGTTAATTTTCTT
TACTCACATCCATGCACATTCTGCCCTTCCTGGACCCTTATCATTCGGAAATGCTACAATTGA
TGCCTTACTTTATCCTATAGAAGCAGCAAAACAAGAACATCTCTTACAGCATACCAACTCCA
AAGGGTTACAAAAATCTCATGCTATTACTTGAAAACAAGCTCAAAATATTGTTCATTCTTGTT
CCATATGTGCACCCTTTGCTTTGCCATTTACCTCACCAGGTGTCAACATGAGATGACAACAA
GCAAATCAGATATGGCAAATGGATGTAATTTACATTTCTTCTTTTGGACAACAAAAAAGTGT
GCATCATATTATAGATGCTTGCATACATTTTCAATGGGCCACTGCATTACATTCTGAAAAGG
CTGACGCTGTTATTACTCATTTGTTATCTTGTTTTGCAGTTATGGGATTACCAATTGAATTGA
AAACTGATAATGCACCTGCCTACCAATCTGCAAAATTAGCTCACTTTTTATCTCAATACCATA
TAACTCATACTTTTGGTATTCCTTATAATAGTCAAGGGCAAGCTATCATTGAAAGAGCTAAT
CGTACCTTGTGTGATTATCTTGAAAAAATAAAAAAGGGGGAACAAGAGAGATTTATGAAAC
CTAAAGACATTCTGAATAAAACCTTACTTACCCTAAATTTTTTGAATGTTTGGAGTAAGGGA
AATCTATCAGCAGCAGAGTTGAATTTTCAAGGGAAAGAAGAGGATAAGCAGATCTTGAATA
TGCCTATTTGGTATAAAGATAAAGATAAAGGTTGGATCCCAGCATCATTAATATATTTGGGA
CGAGGGTATGCTTTCATTTCTGTTAATAATTACAGGTTTTGGACCCCAGCAAGACTGATCAA
AATCAACAATGGCTGATCCCTTTGTTCAAAAATTCAAAGAGCTTACTATGCAGAGAAGCTTT
ACTTTCTGTACAAGGGAAGCAACACCTCCTACATGGGGTCAAATGAAGAGGTTGACCCAGG
AAGCAGAGAAGACATTAATGAAGGCGGGGCAACCTCTGAATCCTACCAATCTTTTGCTTGCC
ATGATGGCGTTGGTGACATGTCAGGTAATCGGTGTATCGGCAAGTAATCATACATATTGGGC
ATATATACCTAATCCCCCATTAGTAAGAGCAGTTTCTTGAGGGGAACCAGAAGTGCAGGTAT
GTACTAATGAGACTGCCTTTTTTCCCCCACCAGCTTGAGGGGGAATAGAACAACTATCTCAT
CATAAACAGCAATATAATATTAGTAATTTGACCATTGCAGTGGAAGGTATTCCTTTATGTAT
AGGAGGACACCCCTTTTGTCTGTCCACCAAGGAACATTCTCATCATTCTTACAATACATGGG
GGGTAAAATATAATAATTACCATTTTGCTACTTTTACTGTGCTTGTTTCCACCAGGGGATTTA
GCATCTCGACAGAACCGGTAGATATTCATAATGAAATACATATGTCACTATGTCCTGTTAAC
TTTTTTGCTTCTTCTCTAGAATCTTTGGAATGGGAATGTTGCCGAGGTCATCGACCCTTTAAA
GTCATGAATTATTCTGGGTCTATCATTGTAGATTGGAGTCCAGATCATGGGCAATTCTTAGA
AAAATGGTCAAATAAATCTTTTAGGTGGCATTGTGCAAATAGCACTTTGATCGGCAATGGTA
ATGAAACAGTTAAATGGCAGCAATTTGCACTTGTCCCTCCTCAATTACAATTGCAAGGGTAT
CTGCACATTCAAGGGGATATTTGGAAACTATGGGCAGTTTCTGGTAATCTCACTATCTGGTC
AGCAAACTATACTTTGGATAGTGGTGACTCTTCGGGTCCATTCCATGTTAATTTACATGTTAA
TAAATCTTATTCTGCAATGGCATGTGTAAAATATCCTTTTGCATTGTTATATGGGAATTGGAC
CTGGAATGATACTGTGAGATCTGTGTCATGTGACTACTGTAATCTAACTCAATGTGTAAATC
AGTCTTGGTGGGAAGAATTTGAAAGACGAACCTATAACTCCAATTTCTCGTTAGTAATTGTT
AAGGCTCGGACAGAAGTATGGTTACCTATAAATCTGACTCGGCCATGGTCAGACTCTTTTGC
TGTTTCTCATCTAGTAACCGCTGTACAGACTTTGCTACATCGATCTCGACGTATGCTTGGTGT
GGTCATTGCTTCGATTCTAGCAGTCGCGTCAGTAACTGCAACAGTGGCAGTGGCAGGACTTG
CATTACACCAAGGAATTCAAGCAGCTGATTTTATTCGGGACTGGCATAAAGACTCTCATTTG
TTATGGCAACAACAGCGAGATTTGGATGCACAACTTGCTACCGACGTGCTCAATCTTCAACA

CATCGTTTCCTGGCTTGGAGATCAATTAGCTGTTTTATCTACACGAAGTGTGTTGAAATGTGA
TTGGAATTCTTCTCAGTTTTGTATAACACCTTTACCATTTAGCATGAGTGAAAGATGGGATAA
AGTAAAATGATCCTTGACTGGGCATCCAAATCTCACTATGGAGATTATGGACCTGGAATGAC
AAATTTTGTGTACTTTTAGCAGGACTTTACCTGGCATTACGGGGTCCTGATTTGCTGAAAAGT
CTTCAAGAGAGAATGAATAATTTAAGTCCATTAGGGCGTGTATCCTCACTAATTGGGACTAC
CTTTGGGAACACTGTGTTTATATTACTTTTATGTTGTGTTGCTTTTCTAGTTTTCCAGCGATGG
CGGAAAGGGAAACAACTAAAGCGCGAAGCAGAGAAGATTCAGTCCATGCTACAATTTATAA
AAGCAAATAAAAAAGGGGGAGATGAAGGGTCATTGCAGCCATAATAGGATAGCGGAGATG
TGCCTGCAAACGGGTGTCTCTGCTTGGGCTGAACATGCTTGCAAACGAGGCATTCCGCCAAG
GAGTCTGGACACAGCCTTGAGTTTAATGGTCCCTTGCAAACGAGGGAACATTCCCTTCTTGT
GATAAGAAGAAAGAAAGGGCTCTGGACAGAACTCTGCAGTAGACAGAGATTTCACTCCGCT
TGCTGTACGATAACATGTATGCACCTGCGCTGTACTGAAAAGGCTTATTCATGCAGTCTGGA
ATTCTGCCTAGGGGGCTTTATAAAAATAAATCACAATTAGTTTTGCGCAGCTTTTTTCCTCCG
GCCGGAGTGGTGTATTGTCTGTCTCTTGTGTGTCTCGTGTGTTTTGTCTTTGTGTCATTTCACT
CACAACA

>NC_056057.1:395192-403082#SHEEP_RIP_12(-)

AGGTGAAGGGTTAATAGGGTAGCAGAGATGTGCCTGCAAACGGGCCTCTTTGCTAGGGCTG
GATGTCCTTGCAAACGAGGAGTTCTGCCAAAGAGTCTGGACACAGCCTTGAGTTTAACGGTC
CCTTGCAAACGAGGGAGCATTCCCTTCTTGTGATAAAGAAGGAATAGAGGGCTTTGTACAGT
CTCTGCAGTAGACCAGGATTTTACTCCCCTTTGCTATACGATAACATGTATGCACCTGCACTG
TGCTGAAAAGGCTTATTCATGCAGTCTGGAATTCTGCCTAGGGGGCTTTTATAATAAACGGT
AATTAGTTTTTTGCCCACTTCTATTCCTCTGGCCAGAGTGTGCATGTTGTCTGTCTTTTGTGTG
TCTTGTTCTGTGTCATTTCACTTGTAATCTCCAACATCTGGCACCCAACGTGGGGCTCGAGTG
AAATCGAAAGGGTGAGTAACCCCGGGGGGGTTTTAAATCCATAGCAGGGGAACTTTCGGGA
AAATTATGGGGAATTCCTCACCCTAGCAGAGGGGAGCTTTCAAAAAAAAATCATGGGGAAT
TCCTCATCATCATTATGGGCACAATACATGGAGTTAGTCCAAGGACTTCTCCACTCCATAGG
CATTAAAGCCTCGACTTGTCGGTTGAGTGAGCTCTTTCGCTTAGTGGAGCAATATTGTCATTG
GTTTCAATATCAAACTAAGTTACAGTTAAACTTGAAGGAATGGAAAATAATTCAAAAGGAA
TTGAGAAAGCAACATCAGAAGGGTTATGTGATCCCTTTGAAGTTGTGGACTTTGTGTAGTGC
TATAACACAGGCTTTGACCTTGCTCTCTACTGATAATGAAACTAAATCTAATGCTTCAAGGA
GGGGAAAAATTTTATGAGGATGTGTCAGACGTTGGTGGGGCTTCTGCATCGCCTGAAGGCAA
GGATACAAATGAGCCTTCTCCTGTAAATGGTGAAACATCTGATAGTTCAGAATCAGATTTGG
AGGCTTCTTCGGTTTTGTCAGAGGAGGGCAAAGAGATTAAAGAAATGACCTATCTATTCCAG
GAATGGTGGAAATCCCATATGGAGGAGAAAAAATCTACACCTTCTGCTCCTCCTTGTGCTTC
TCTTTTCCCCTCTGTGGTTAATCAGCCCAATGTGGGCAGGGAACATTGTCGGTTCTCCTTTCC
TTTGTCTATGCTTCATGATGATGACTCGCCTGCTCCCCCTGGTGGGTTTATCGATCTTCCACA
ATTGTTTCCCATCCAGAGACAGCAGGATGGCAATGTGATAAATGTTCAATATGCTCCTTTGG
AATATAAATTTTTTAAAGATCTTAAAACTGCAGTAGTGCAGTACGGTCCTCAATCTCCCTTTG
TTTTGGCTATGCTGGAATCATTGGGAAAAGGCAAATTAATCATTTCATTAGATTGGGAATCT
ATTGTCCAAGCTGTCTTGGAGGGCTCTCAATGGTTGCAACTTCATAGCTGGTGGGAAGAAGA
AGCTAGAAAGCAGGTTCGGATTAATGAGAGACAGAATCCCCCTGATCCTCTTGAGGACAAG
CTAATGGGAGAGGGCCAATATCGGGCTTTAAGAGAACAGACTCAATACTCTGACCAAGACT
TACAACAAGTCCGCCAGGTCTTTTTACGAGCATGGCACCTACTGGCCACACCCAGCCCTCCT
TTGTTAAAACAATGCAAGGCCCCAATGAGCCATATACTGATTTTCTAGCAAGATTGAGGGTA
GCTGTGGAACAGGCTGTAGGGAGGAATGAGATTTCAGAGATATTATTACAAACTTTAGCATT
TGAAAATGCAAATCCTGAACTCAAGCATATACTGGGACCTTTAAAGGGACAGGGTGCATCG

ATAGCTGAATATATCAGAGCCTGCTCAGGAATAGGAGGGACTGAGCATTAGGCTAATGTCTT
TGCTACAGCCTTGGCCAAAGTTATGAGACCACCAAAGGGAGGTAACTGCTTTCATTGTGGAA
AACCTGGTCATATGAAAAGAGAGTGTCGGAAATTAAAAGTTGATCAAGGTGTAATTCCTAA
AGACAGATCTCTTGCTGGGAAGAATAAGACTCCTCCTGGACTTTGCCATCAGTGCAGGAAGG
GGTTTCATTGGGCTAATGAATGCAGATCTAAAACAGACAAAATGGCCAACCCAATACTGGG
AAACTATCCTGCAGGCCTAAGTCCTTGGGGCCCAGGAACAATACCGGGGACTTATCCTCCTT
GCCCTCCTCCCATCCCATCTGTCCCAACCCTATTCCCTCCCAACAACCGTTACGAGTCAATGC
CCTGTTAAAAGGACCTCAGATGATGATTTTGAACTTACGGTCTGCTACTTCAGGGAGTGCTG
CTGCTGATTTGCCACTAGCTGATAATGTTCTTTTGTCACCAGGGGAAGGCATTTATAAATTAA
AAACAAATGTATTTGGACCACTGCCTAAAGGCACTTTTGGCTTGATATTAGGCTGTAGCAGC
ACGGCTTTGAGAGGTTTAACCATATTTTCTGGGGTAATAGACTCTGATTATGTTGGGGAAAT
TTTAATTATGGTCTCTATTTCTATCACACTTTCATTGTTAGCTGGGGAACATATTGCTCAAAT
ACTTCTCCTACCTTATCACCCCTTTTTGGCTCTTCCTAATGAACAAACAGGAGGATTTGGAAG
TACTGGGCGACATATATTTTGGGAAATGCTTGTCAAAGATTCCCGCCCTGTTCTCTCCTTGAA
TATACAGGGAAACAACTTTGAAGGACTAGTAGACACAGGGGTGGATGTTTCAGCCATTTCTT
CTCAACAATGGCCCCAAGATTGGGAAAAAGAAAAAAGCCCTTTAATGCTGACGGGATTGGG
CTCCATTGCAGATGTCTGGAAGAGTACCCATCCCTTACAATGTCAATTCCATAATGGAAGAT
CAGTGTTTGTTACCTTTTATATTGTAAATATACCTATTAATATATGGGGAAGAGATCTTCTCT
CTCCTTTGGGGGCTTCTGTAACCATTCTATAGGAAAACTAGGAGCCACTGCTCAAATTCCTC
GAGCACTCCCATTAAAATGGTTAACTAATACTCCAAAATGGGTTGAGCAGTGGCCATTACCA
CAAATGAAGCTCGAGGCATTAGAATAATTAGTACAAGAACAACTCCAACTTGGTCATATAG
AGCCCTCTACCTCCCCCTGGAATTCTCCTGTTTTTGTTTTAAAAAATAAATCTAGAAAATGGA
GAATGTTAACTGATTTACGAAAAGTTAATAAATGTATTGAACCTATGGGAGCCTTGCAATTG
GTACTCCCCTCTCCAGCTCTTATTCCTCAGAATTGGTCCTTAATGGTGTTAGATCTTAAAGAC
TGTTTTTTTTTTTTTTTACCATTCCCCTCCAATTGAAAGATAGAGATAAATTTGCTTTTACAGTT
CCTGTTCTTAATCATGCTCAGCCTGTTAAGCATTATCAATGGACAGTCTTACCACAAGGAAT
GATAAATAGTCCTACCTTATGCCAAGAATTTGTAGCTTGCTCTTTACAATCCCTCCATCAAGA
ATACGCCAATTATATTCTATATGATTATATGGATGATCTCCTATTGGCAGCTCCTAGTATTGC
TGAACGTGATGAATTCTTTTTAAAAGTACAGGAGGCTTTAAGACTATACAATTTGCAAGTAG
CCCCGTAAAAAATTCAAAAGGACTTTCCTATTTCATATTTAGGGACAATATTGGAACAACAT
AGAATTAGGCCCCAAAAGTTGCAAATTAGAAGAGACCATCTCAAAACCTTAAATGATTTTCA
AAAGTTATTGGGAGATATTAATTGGCTACACCTGGTACTTGGGATTCCTACTTATCAATTACG
ACATTTGTTTTCTACTTTAGAAGGAGACACAGCTCTGGATAGCCCCCCGGACCTTAACCCCA
TTGGCTTTACAGGAATATCAATTTGTTGAGCGACGACTAAATGACGGCTTTTTGACTTACTTA
CATGCATCTCAACCTATTTCTTTTGTAATATTTCATACCCCTTACTCCCTATCTGGTATAATTG
CTCAAGAAAAAGGATTAATAGAATGGGTTTTCTTACCTAACTGTTTTTCCAAAAAATTGACT
GTATATATGGACAAATTAGCCTTCCTTATACAGAAGGGTCACCATCATATTTTACAATTATCA
GGATGTGAACCACACCAGATTGTTACTCAGTTAATAACTGCTCAAATATCTCGATGTTTACA
ATTTAATGAAAACTGGCAAATTTCTCTTGCCTCATATCCTGGTTCATTTTCTAATCATTATCC
ATAATCTAAATTAATTGATTTTCTCTGGACTAACACTATGATATCTCATTCCCCGATTTCAGA
TGTTCCAGTAAAGGGACCCGCTATTTTTACAGATGCAAATAAAAATACTGCTGGATATTGGA
ACCTGGAAAATTCCAAGGTTCTCCCCCACTCATTTTCTTCTGTACAGCCCACTGAATTGTGGG
CTATCTATTTAGTTTTGCAAGATTTTCCCTAACTTCCTATTAACATTGTTTCAGATTCTCAATA
TGCTGTTCTCTCTTGCCTACAGCTTCCCCATGTCTCCCTTCCATTGACTCTTAAAACAGTTATT
GATAAATTGTTTTACCAAGTACAACAGTTGCTCTTGCAGCGTTTAGAGTTAATTTTCTTTAGT
CACATCCATGCACATTCTGCCCTTCCTGGACCCTTATCATTCAGAAATGCTACAATTGATGTC
TTACTTTATCCTATAGAAGCAGCAAAACAAGAACATCTCTGACAACATACCAACTCCAAAGG

GTTACAAAAATCCCATGCTATTACTCGAAAACAAGCTCAAAATATTGTTCATTCTTGTTCTAT
ATGTGCACCTTTTGTTTTGTCATTCACCCCACCAGGTGTCAACATAAGAGGACTACAAGCAA
ATCAGATATGGCAAATGGATGTAATTTACATTTCTTCCTTCGGACAACAAAAATGTGTGCAT
CATACTGTAGATAATTGCACACATTTTCAATGGGCCACTGCATTACATTCTGAAAAGGCTGA
TGCTGTTATTACTCATTTGTTGTCTTGTTTTGCAGTTATGGGATTACCAATTGAATTGAAAAC
TGCACCTGCTTACCAATCCGCAAAATTAGCTCACTTTTTATCTCAATACCATATAACTCATAC
TTTTTGTATTCCTTATAATAGTCAAGGGCAAGCTATCATGGAAAGAGCTAATCATAGCTTGC
ATGATTATCTTGAAAAAATAAAAAAGGGGGAACAAGAGAGATTTATGAAACTTAAAGACAT
TTTGAATAAAACCTTACTTACCCTACATTTTTGAATGTTTGGAGCAAGGGAAATCTATCAGC
AGCAGAGTTGCATTTTCTAGGGAAAGAAGAGGATAAAAAGATCTTGAATATGCCTATTTGGT
ATAAAGATAAAGAGAAAGGTTGGATCCCAGCATCATTAATATATTTGGGACGAGGGTATGC
TTTCATTTCTGTTGATAATTACAGGTTTTGGACCCCAGCAAGATTGATCTAAATCAACAATGG
CTGATCCCCTTGTTCAAAAATTCAAAGAGCTTACTATACAGAGAAGCCTTACTTCCCGTACA
AGGGATGCAACACCTCCTACATGGGGTCAAATGAAGAGGTTGACCCAGGAAGCAGAGAAGA
CGTTAATGAAGGCGGGGCAACCTCTGAATCCTACCAATCTTTTGCTTGCCATGATGGCAGTG
GTGACATGTCAGGTAATCGGTGTATCGGCAAGTAATCATACGTATTGGGCATATATACCTAA
TCCCCCATTAGTAAGAGCAGTTTCCTGGGGGGAACCAGAAGTGCAGGTATGTACTAATGAG
ACTGCCTTCTTTCCTCCGCTAGCTTGTGGGGGAATAGAACAACTATCTCATCATGAACAACA
ATATAATATTAGTAATTTGACCATTGCAGTAGAAGGTATTCCTTTATGTATAGGGGGACACC
CCTTTTGTCTGTCCACCAAGGAACATTCTCATCATTCTTATAAATACATACGGGGTGAAATAT
AATAATTACCATTTTGCTACTTTTACTGTGCTTGTTTCCACCAGGGGATTTAGCACCTCGACA
GAACTGATAGATATTCATAATGGGAAACACATGTCGCTATGTCCTGTTAACTTTTTTGTTCCT
TCTCTAGAATCTTTGGAGTGGGAACATTGCCGAGGTCATCGACCCTTTAAAGTCATGAATTA
TTCTGGGGCCATCATTGTAGATTGGAGTCCAGATCATGGGCAATTCTTAGAAAAATGGTCAA
ATAAATCTTTTAGGTGGCATCGTGCAAATAGCACTTTGATGGGCAATGGTAATGAAACAGTT
AAATGGCAGCAATTTGCACTTGTCCCTCCTCAATTACAATTGCAAGGATATCCGCACATTCA
AGGAGATATTTGGAAACTATGGGTGGTTTCTGGTAACCTCACTATCTGGTCAGGAAACTATG
CTTTGGACAGTGGTGACTCTTCAGGTCCATTCCATGTTAATTTACATGTTAATAAATCTTATT
CTGCAATGGCATGTGTAAAATATCCTTTTGCATTGTTATATGGGAATTGGACCTGGAATGAT
ACTGTGGTGTCTGTGTCATGTGACTATTGTAATCTAACTTAATGTGTAAATCAGTCTTGGTGG
GAAGAATTTGAAAGACCAACCTATAATTCCAACTTCTCGCTAGTAATTGTTAAGGCTCGGAC
AGAAGTATGGTTACCTATAAATCTGACTCTGCCGTGGTCAGATTCTTTTGCTGTTTCTCATCT
AGTAACCACTGTACAGACTTTGCTACACTGATCTCGAGGTATGCTTGCTGTGGTCATTGCTTC
GATTTTAGCAGTTGTGCCAGTAACTGCAACAGCAGCAGTAGCAGGTCTTGTGTTACACCAAG
GAATTCAAACAGCTGATTTTATTTGGGACTGGCATAAAGACTCTCATTAGTTATGGCAACAA
CAGTGAGATTTGGATGCCCAACTTGCTATCGATGTGCTCAATCTTGAACACACCATTTCCTGG
CTTGGAGATCAATCGGCTGTTTTATCTACACGAAGTGTGTTGAAATGTGATTGAAATTCTTCT
CAGTTTTGTATAACACCTGTACCATTTAACATGAGTGAAGGATGGGATAAAGTAAAACGATC
CTTGACTGGGCATCAAAATCTCACTATGGAGATTATGGACCTGGAAAGACAAATTTTGTCTA
CTTTTAGCAGGACTTTATCTGACATTATGGGGTCTGATTTGCTGAAAAGTCTTCAAGAGGGA
ATGAATAACTTAAATCCATTAGGGCACGTATCCTCACTAATTGGGACTACTTTTGGGAACAC
TGTGTTTATATTACTTTTATGTTGTGTTGCTTTTCTAGTCTTCCAGCGATGGCGGAAAGGGAA
ACAACTAAAGAGCAAAGCAGAGAAGATCCGGACCATGCTACAGTTTGTAAAAGCAAATAAA
AAAGGGGGAGATGAAGGGTTATTAAGGTAGCAGAGATGTGCCTGCAAACGGGCCTCTTTGC
TAGGACTGGATGTCCTTGCAAACGAGGCATTCTGCCAAAGAGTCTGGACACAGCCTTGAGTT
TAATGGTCCCATGCAAACGAGGGAGCATTCCCTTCTTGTGATAAAGAAGGAATAGAGGGTTT
TGTACAGACTCTGCAGTAGACTAGGATTTTACTCCCCTTTGTTATACGATAACATGTATGCAC

CTGCGCTGTGCTGAAAAGGCTTATTCATGCAGTCTGGAATTCTGCGTAGGGGGCTTTTATAA
TCAACGGCAATCAGTTTTTTGCCCAGTTCTGTCCCTCCGGCCGGAGTGTGCGTGTTGTCTGTC
TTTTTTATGTCTTGTTCTGTGTCATTTCACTTGTAATCTCCAACA

>NC_056057.1:41962471-41970401#SHEEP_RIP_13(-)

TGCGGGGGACGACCCGTGAAGGGTTAAGTCTTGGGAGCTCCCTGGCAGGTATGCCAGGCCC
TAGGACACGTGCCTAAGCTCCCTGTCCCGCCACCCTCAAGAGTTTTTATAACCCTTAAGGCTC
CAAGATGTTTGGTTTCGGCAACATTTCATAGAAGATAGATTATCTTATTGTGTATATTTCATA
GAAGATAGATATTCTGATTGTGTTCTGTATACAATGGTAAGGGTCTGGTGATTGTATCCTGA
GATTAAAAAACAACCTTGTGAGTGCCTTAAGTCACGTACTTTACCCTATATATACTGCAGCA
CAATAAAGCAAGGTATCAGCCATTTTGGGGCTGATCCTCTCAACCCCATCTTTTGTCTATCTC
TTATTTTCTTAGCGGGGACGCTCCGTTCTCTCCCTGTGCAGGTGCGACTCTTGCTTGTGCTGG
CCGCGGCAGGTGGCGCCCAACGTGGGGCTCGAGCTCGACAGTTTTCCTCGCCACTACTCTTA
TTAATTGAAAAGAGTGAGTATATGAGTAAACAAGTGAATTAAATTGAGGAGGAGTAGTAAG
GTATATAGTTGAGAGTATAAATATGGGACAGACGCATAGTCGCCAGTTGTTTGTGCATATGT
TATCTGTAATGTTAAAACATAGGGGAATTACTGTTTCTAAACCTAAATTAATCAATTTTCTTT
CATTCATCGAGGAAGTTTGCCCTTGGTTCCCCAGAGAAGGTACAGTAAATTTGGAGACATGG
AAGAAGGTAGGGGAACAAATTCGGACTCATTATACTTTACATGGCCCTAAAAAAATCCCTGT
CAAAACTTTATCCTTTTGGACACTAATTCGTGACTGCCTGGACTTTGATAATGATGAATTAAA
ACGTTTAGGAAATTTATTAAAACAGGAAGAAGATCCTCTCCATGTTCCTGATTCGGAACCCA
GATATGCTGTTCCCGAGGGGGTTAAAAGCGACCCTCCGTTTTCTAACTTATTGCATCCTTCAG
ATAATGATGATTTACTTTCATCCACAGATGAGGCAAAATTAGACGAAGAAGCTGCTAAATAC
CATCAAGAAGATTGGGGTTTTTTAGCACAAGAAAAGGGGCGTTAACATCTAAAGATGAATT
GGTTGAATGCTTTAAAAACCTCACTATTGCTTTACAGAACGCAGGAATCAAGCTTCCTAGTA
ACAATGCCAAATCTCCTTCTGCTCCGCCTCTTCCCCCTGCTTATGCTCCTTCTGTTGTGGCTGG
TCTCGATCCCCCTCCAGGGCCCCCTCCACTGTCTGAGAACATGTCTCCGCTGCAAAAGGCAT
TGAGACAGGCACAGCGACTTGGTGAGGTTGTCTCTGATTTTTCTCTTGCTTTTCCTGTCTTTG
AAAATAACAACCAGCGTTATTATGAATCACTGCCTTTTAAACAACTGAAAGAGTTAAAGATT
GCTTGCTCACAATACGGTCCTACCGCTCCATTCACCATTGCTATGATAGAAAATTTGGGTACT
CAAGCTTTACCTCCAAATGATTGGAAGCAGACAGCTAGGGCATGTCTCTCAGGGGGAGATTA
TTTATTATGGAAATCTGAATTTTTTGAACAATGTGCTCGTATAGCTGATGTTAACCGACAGCA
AGGTATACAGACCTCCTATGAAATGTTGATTGGTGAAGGCCCTTACCAGGCTACTGATACTC
AACTTAATTTCTTACCTGGTGCATATGCACAAATATCAAATGCGGCTCGGCAGGCATGGAAA
AACTTCCTAGCTCCAGTACTAAGACAGAGGATCTTTCAAAAGTCCGGCAGGGACCTGATGAG
CCTTACCAGGACTTCGTGGCACGACTTTTAGATACTATAGGTAAGATAATGTCAGATGAAAA
GGCTGGGATGGTACTGGCAAAACAATTGGCTTTTGAAAACGCTAACTCTGCTTGTCAAGCTG
CTTTAAGACCTTATCGAAAAAAGGGAGATCTGTCTGATTTTATTCGCATTTGTGCTGACATTG
GACCCTCCTACATGCAAGGCATTGCTATGGCAGCAGCATTACAAGGAAAAAGCATAAAAGA
GGTACTTTTCCAGCAGCAAGCCCGGAACAAGAAAGGACTTCAAAAGTCAGGTAATTTGGGT
TGCTTTGTTTGTGGTCAGCCTGGCCATCGGGCTGCAGTGTGCCCTCAAAAACAACAAAGCCC
TGTTAACACTCCTAATTTGTGCCCACGCTGTAAAAAAAGGAAAGCATTGGGCGCGGGATTGC
CGTTCCAAAACGGATGTTCAAGGTAATCCTTTGCCCCGGTTTCGGGAAACTGGGTGAGGGCC
AGCCCTGGCCCCGAAACAATGTTATGGGGCAACACTGCAGGTTCCAAAAGGACCATTGCAG
ACCTCTGTCGAGCCACAAGAGGCAGCGCGGGATTGGACCTCTGTGCCACCTCCTACACAGTA
TTAACTCCCGAGATGGGGGTCCAAACCCTTGCCACAGGAGTGTTTGGGCCTTTACCTCCAGG
GACAGCTGGACTGCTTTTAGGGCGCAGCAGTGCGTCTTTAAAAGGAATACTTATTCATCCTG
GTGTGATTGACTCTGATTATACAGGAGAGATAAAAATATTAGCCTCCGCTCCTAACAAAATT

ATTGTAATCAATGCAGGACAGCGTATAGCTCAACTTCTTTTAGTTCCATTAGTCATACAGGG
AAAAACAATTAACCGAGACCGTCAAGATAAAGGTTTCGGGTCCTCTGACGCCTATTGGGTGC
AAAATGTTACCGAGGCACGACCAGAACTTGAGCTACGCATTAATGGTAAGCTTTTCCGAGGA
GTGCTTGATACAGGGGCCGATATTAGTGTTATTTCTGATAAATATTGGCCTACTACATGGCC
AAAACAGATGGCTATTTCCACTCTCCAGGGTATTGGCCAAACTACCAATCCAGAACAGAGTT
CATCCCTTCTTACTTGGAAAGATAAAGATGGACATACAGGCCAATTTAAACCTTATATTCTG
CCCTATCTTCCAGTTAATCTATGGGGGCGTGATATATTGAGCAAAATGGGTGTTTATTTATAT
AGTCCTTCACCCACTGTGACAGATTTGATGTTAGATCAGGGCTTACTTCCAAATCAAGGTTTA
GGTAAACAACATCAAGGCATCATTTTGCCCCTTGATTTAAAAATCTAATCAAGATCGAAAAG
GCTTGGGGTGTTTTCCTAGGGACCTCTGATTCTCCTGTGACACATGCCGATCCTATTGATTGG
AAATCTGAGGAACCGGTATGGGTCGATCAGTGGCCCCTAACACAGGAAAAACTTTCTGCCG
CACAACAGCTGGTGCAGGAACAGCTGAGACTTGGGCATATTGAACCCTCTACCTCTGCTTGG
AATTCCCAATTTTTGTTATTAAAAAGAAGTCTGGGAAATGGAGATTGCTACAAGATCTTCGT
AAGGTAAATGAAACAATGATGCATATGGGAGCCCTACAACCTGGGTTGCCCACTCCTTCTGC
TATACCTGATAAATCCTATATCATTGTTATAGATTTAAAAGATTGTTTTTACACTATTCCTCTT
GCACCTCAAGATTGCAAAAGATTTGCTTTCAGTTTACCCTCTGTTAATTTTAAAGAGCCTATG
CAACGCTATCAATGGAGAGTTCTCCCGCAAGGAATGACTAATAGCCCTACGCTGTGCCAAAA
ATTTGTTGCTACAGCAATAGCTCCGGTTCGTCAACGTTTTCCTCAGCTATATTTGGTTCATTA
TATGGATGATATATTACTAGCTCATGCTGACGAACATCTATTGTATCAAGCTTTTCGATTCTA
AAACAACATTTAAGCCTTAATGGTCTTGTTATTGCTGATGAAAAATTCAGACTCATTTTCCTT
ATAATTATTTGGGTTTCTCCTTATATCCTCGTGTTTATAATACCCAATTAGTACAATTACAGA
CTGACCATTTAAAAACTCTAAATGACTTTCAAAAACTTTTAGGAGACATTAATTGGATACGT
CCTTATTTAAAATTACCCACTTATACCTTGCAGCCATTATTTGACATCCTTAAAGGTGACTCT
GATCCTGCGTCACCCCGAACACTTTCTTTAGAAGGACGAACTGCTTTACAATCAATAGAAGA
AGCTATTAGACAACAACAGATTACTTATTGTGATTACCAACGATCATGGGGTTTGTATATAC
TTCCTACCCCCCGAGCACCCACAGGGGTTCTCTATCAAGATAAACCTTTGCGATGGATATAT
TTGTCTGCTACTCCAACTAAACATCTGCTCCCTTACTATGAACTTGTTGCAAAATTGTAGCAA
AGGGACGTCACGAGGCCATCCAATATTTTGGTATGGAACCCCCTTCATTTGTGTTCCTTATGC
TTTAGAACAACAAGATTGGCTTTTTCAATTTTCAGATAATTGGTCTATAGCTTTTGCAAATTA
CCCGGGACGGATTACTCATCATTACCCTTCTGATAAATTGTTACAATTTGCTAGCTCTCATGC
CTTTATTTTTCCAAAAATAGTTCGCCGACAACCTATTCCCGAAGCGACACTTATATTTACAGA
TGGATCTTCTAATGGAACTGCAGCTTTAATCATTAACCATCAAACCTATTACGCACAAACCA
GTTTTTCTTCTGCTCAAGTTGTGGAATTATTTGCAGTCCACCAAGCGTTGCTAACTGTACCTA
CTTCCTTCAATTTATTTACAGACAGCTCCTATGTGGTCGGTGCCTTACAGATGATTGAAACTG
TTCCAATTATCGGCACCACCTCTCCTGAAGTTCTTAACTTATTTACATTGATTCAACAGGTTC
TCCATTGCCGCCAACACCCCTGTTTCTTTGGACATATTCGTGCACACTCCACCCTTCCTGGTG
CCCTCGTACAAGGCAATCACACTGCGGACGTTCTTACTAAACAAGTGTTTTTCCAATCAGCT
ATTGATGCAGCCCGAAAATCCCATGATTTACATCACCAAAATAGTCAGTCTTTACGCTTGCA
ATTTAAAATTTCCCGTGAAGCTGCACGGCAAATTGTTAAATCTTGCTCTACTTGTCCTCAATT
CTTTGTTCTCCCTCAATATGGTGTCAACCTCGAGGTTTACGCCCTAATCACCTCTGGCAAACA
GATGTTACTCACATTCCTCAATTTGGGCGTCTTAAATATGTTCATGTTTCTATTGACACTTTTT
CCAATTTTCTCATGGCTTCCCTTCACACTGGAGAATCAACACGTCACTGTATTCAACATTTGC
TGTTTTGCTTTTCTACTTCAGGAATCCCACAAACCCTTAAAACAGATAATGGACCTGGTTATA
CTAGCCGTTCTTTTCAACGTTTTTGTCTTTCTTTCCAAATTCATCATAAAACAGGAATTCCTTA
TAATCCACAGGGACAAGGTATTGTGGAACGAGCCCATCAACGCCTTAAACATCAATTATTAA
AACAAAAAAAGGGGAATGAACTGTATAGCCCCTCACCGCATAACGCCTTAAACCATGCTCTT
TATGTTTTAAATTTTTTAACTTTAGACGCAGAAGGCAATTCAGCAGCCCAGCGTTTTTGGGGA

GAACGATCCTCATGCAAAAAACCACTTGTGCGATGGAAGGATCCACTTACCAATCTGTGGTA
TGGGCCAGACCCTGTACTAATATGGGGACGAGGGCATGTTTGTGTTTTTCCACAGGATGCCG
AAGCGCCGCGCTGGATTCCGGAAAGGCTGGTACGCGCAGCAGAGGAACTCCCTGACACATC
AAATGCAATGCATGACACTGAGTGAGCCCACGAGTGAGCTGCCTACCCAGAGGCAAATTGA
GGCGCTGATGCGTTATGCTTGGAATGAGGCTCATGTACAACCTCCAGTGACACCTACTAATA
TACTGATCATGTTATTATTATTGTTACAGCGGATACAAAACGGGGCAGCTGCGGCTTTTTGG
GCATACATTCCTGATCCGCCTATGATTCAATCCTTAGGATGGGATAAAGAAACAGTACCTGT
ATATGTTAATGATACAAGTCTTTTAGGAGGAAAATCAGATATTCACATTTCTCCTCAGCAAG
CCAATATCTCCTTTTATGGTCTTACTACTCAATACCCTATGTGCTTTTCTTATCAATCACAGCA
TCCTCATTGTATACAGGTGTCAGCTGATATATCCTATCCTCGAGTGACTATTTCAGGCATTGA
TGAAAAAACCGGAAAGAGATCGTACCGTGACGGAACCGGACCCCTCGACATTCCGTTTTGT
GACAAACATTTAAGCATCGGCATAGGAATAGACACTCCTTGGACTTTATGTCGAGCACGAAT
TGCATCGGTGTATAACATCAACAATGCCAATACCACCCTTTTATGGGACTGGGCACCTGGAG
GAACACCTGATTTCTCCGAATATCGAGGACAGCATCCACCCATTCTTTCTGTAAACACTGCTC
CTATATTTCAAACTGAACTGTGGAAACTTTTGGCTGCTTTTGGTCATGGTAATAGCCTATATT
TACAGCCCAATATTAGTGGGAGTAAATATGGTGATGTGGGAGTTACAGGATTTTTATATCCC
CGAGCTTGTGTTCCTTACCCATTCATGTTGATACAAGGCCATATGGAAATAACACTGTCATTG
AATATTTATCATTTAAATTGTTCTAATTGCATACTTACTAATTGCATTAGAGGTGTAGCCAAA
GGAGAACAAGTTATAATAGTAAAACAACCTGCTTTTGTAATGTTACCTGTTGAAATAACTGA
AGAATGGTATGATGAAACTGCTTTAGAATTGTTACAACGCATTAATACGGCTCTTAGCCGTC
CTAAAAGAGGTCTGAGCCTGATTATTCTGGGTATAGTGTCTTTAATCACCCTTATAGCAACTG
CTGTTACTGCTTCTGTATCTTTAGCACAATCCATTCAAGCTGCTCATACTGTAGATTCCTTGTC
ATATAATGTTACTAAAGTAATGGGAACTCAAGAAGATATAGATAAAAAATAGAAGATAGAT
TATCAGCTTTATATGATGTAGTTAGAGTTCTAGGAGAACAAGTTCAGAGCATTAATTTTCGC
ATGAAAATTCAATGCCATGCTAATTATAAATGGATTTGTGTTACAAAAAGCCTTACAATACT
TCTGACTTTCCGTGGGATAAGGTGAAAAACATCTGCAAGGAATTTGGTTTAATACTAATGTT
TCTTTAGATCTTTTACAATTGCATAATGAAATTCTTGACATCGAAAATTCTCCAAAAGCTACT
TTGAATATAGCTGATACCGTCGATAATTTTTTACAAAATTTATTTTCTAACTTTCCTAGCCTTC
ATTCACTGTGGCGAAGTATAATTGCTATGGGCGCGGTTCTGACTGTTGTGCTTATCATAATTT
GTTTAGCTCCTTGCCTTATTCGTAGCATTGTTAAAGAATTTCTACATATGAGAGTTTTAATAC
ATAAAAACATGTTGCAACACCAACATCTTATGGAGCTTTTAAAAAATAAAGAGAGGGGAGC
TGCGGGGGACGACCCGTGAAGGGTTAAGTCTTGGGAGCTCCCTGGCAGGTATGCCAGGCCC
TAGGACACGTGCCTAAGCTCCCTGTCCCGCCACCCTCAAGAGTTTTTATAACCCTTAAGGCTC
CAAGATGTTTGGTTTCGGCAACATTTCATAGAAGATAGATTATCTTATTGTGTATATTTCATA
GAAGATAGATATTCTGATTGTGTTCTGTATACAATGGTAAGGGTCTGGTGATTGTATCCTGA
GATTAAAAAACAACCTTGTGAGTGCCTTAAGTCACGTACTTTACCCTATATATACTGCAGCA
CAATAAAGCAAGGTATCAGCCATTTTGGGGCTGATCCTCTCAACCCCATCTTTTGTCTATCTC
TTATTTTCTTAGCGGGGACGCTCCGTTCTCTCCCTGTGCAGGTGCGACTCTTGCTTGTGCTGG
CCGCGGCA

>NC_056060.1:17005264-17013194#SHEEP_RIP_17(+)

CTGCGGGGGACGACCCGTGAAGGGTTAAGTCTTGGGAGCTCCCTGGCAGGTATGCCAGGCC
CTAGGACACGTGCCTAAGCTCCCTGTCCCGCCACCCTCAAGAGTTTTTGTAACCCTTAAGGCT
CCAAGATGTTTGGTTTCGGCAACATTTCATAGAAGATAGATTATCTTATTGTGTATATTTCAT
AGAAGATAGATATTCTGATTGTGTTCTGTATACAATGGTAAGGGTCTGGTGATTGTATCCTG
AGATTAAAAAACAACCTTGTGAGTGCCTTAAGTCACGTACTTTACCCTATATATACCGCAGC
ACAATAAAGCAAGGTATCAGCCATTTTGGTCTGATCCTCTCAACCCCATCTTTTGTCTATCTC

TTATTTTCTTAGCGGGGACGCTCCGTTCTCTCCCTGTGCAGGTGCGACTCTTGCTTGTGCTGG
CCGCGGCAGGTGGCGCCCAACGTGGGGCTCGAGCTCGACAGTTTTCCTCGCCACTACTCTTA
TTAATTGAAAAGAGTGAGTATATGAGTAAACAAGTGAATTAAATTGAGGAGGAGTAGTAAG
GTATATAGTTGAGAGTATAAATATGGGACAGACGCATAGTCGCCAGTTGTTTGTGCATATGT
TATCTGTAATGTTAAAACATAGGGGAATTACTGTTTCTAAACCTAAATTAATCAATTTTCTTT
CATTCATCGAGGAAGTTTGCCCTTGGTTCCCCAGAGAAGGTACAGTAAATTTAGAGACATGG
AAGAAGGTAGGGAACAAATTCGGACTCATTATACTTTACATGGCCCTGAAAAAATCCCTGTC
GAAACTTTATCCTTTTGGACACTAATTCGTGACTGCCTGGACTTTGATAATGATGAATTAAAA
CGTTTAGGAAATTTATTAAAACAGGAAGAAGATCCTCTCCATGTTCCTGATTCGGAACCCAG
ATATGCTGTTCCCGAGGGGGTTAAAAGCGACCCTCCGTTTTCTAACTTATTGCATCCTTCAGA
TAATGATGATTTACTTTCATCCACAGATGAGGCAGAATTAGACGAAGAAGCTGCTAAATACC
ATCAAGAAGATTGGGGTTTTTAGCACAAGAAAAGGGGCGTTAACATCTAAAGATGAATTGG
TTGAATGCTTTAAAAACCTCACTATTGCTTTACAGAACGCAGGAATCAAGCTTCCTAGTAAC
AATGCCAAATCTCCTTCTGCTCCGCCTCTTCCCCTGCTTATGCTCCTTCTGTTGTGGCTGGTCT
CGATCCCCTCCAGGGCCCCCTCCACCGTCTGAGAACATGTCTCCGCTGCAAAAGGCATTGAG
ACAGGCACAGCGACTTGGTGAGGTTGTCTCTGATTTTTCTCTTGCTTTTCCTGTCTTTGAAAA
TAACAACCAGCGTTATTATGAATCACTGCCTTTTAAACAACTGAAAGAGTTAAAGATTGCTT
GCTCACAATACGGTCCTACCGCTCCATTCACCATTGCTATGATAGAAAATTTGGGTACTCAA
GCTTTACCTCCAAATGATTGGAAGCAGACAGCTAGGGCATGTCTCTCAGGGGAGATTATTTA
TTATGGAAATCTGAATTTTTTGAACAATGTGCTCGTATAGCTGATGTTAACCGACAGCAAGG
TATACAGACCTCCTATGAAATGTTGATTGGTGAAGGCCCTTACCAGGCTACTGATACTCAAC
TTAATTTCTTACCTGGTGCATATGCACAAATATCAAATGCGGCTCGGCAGGCATGGAAAAAA
CTTCCTAGCTCCAGTACTAAGACAGAGGATCTTTCAAAAGTCCGGCAGGGACCTGATGAGCC
TTACCAGGACTTCGTGGCACGACTTTTAGATACTATAGGTAAGATAATGTCAGATGAAAAGG
CTGGGATGGTATTGGCAAAACAATTGGCTTTTGAAAACGCTAACTCTGCTTGTCAAGCTGCT
TTAAGACCTTATCGAAAAAAGGGAGATCTGTCTGATTTTATTCGCATTTGTGCTGACATTGG
ACCCTCCTACATGCAAGGCATTGCTATGGCAGCAGCATTACAAGGAAAAAGCATAAAAGAG
GTACTTTTCCAGCAGCAAGCCCGGAACAAGAAAGGACTTCAAAAGTCAGGTAATTTGGGTT
GCTTTGTTTGTGGTCAGCCTGGCCATCGGGCTGCAGTGTGCCCTCAAAAACAACAAAGCCCT
GTTAACACTCCTAATTTGTGCCCACGCTATAAAAAAGGAAAGCATTGGGCGCGGGATTGCCG
TTCCAAAACGGATGTTCAAGGTAATCCTTGCCCCCGGTTTCGGGAGAACTGGGTGAGGGCCA
GCCCCTGGCCCCGAAACAATGTTATGGGGCAACACTGCAGGTTCCAAAAGGACCATTGCAG
ACCTCTGTCGAGCCACAAGAGGCAGCGCGGATTGGACCTCTGTGCCACCTCCTACACAGTAT
TAACTCCCGAGATGGGGGTCCAAACCCTTGCCACAGGAGTGTTTGGGCCTTTACCTCCAGGG
ACAGCTGGACTGCTTTTAGGGCGCAGCAGTGCATCTTTAAAAGGAATACTTATTCATCCTGG
TGTGATTGACTCTGATTATACAGGAGAGATAAAAATATTAGCCTCCGCTCCTAACAAAATTA
TTGTGATCAATGCAGGACAGCGTATAGCTCAACTTCTTTTAGTTCCATTAGTCATACAAGGA
AAAACAATTAACCGAGACCGTCAAGATAAAGGTTTCGGGTCCTCTGACGCCTATTGGGTGCA
AAATGTTACCGAGGCACGACCAGAACTTGAGCTACGCATTAATGGTAAGCTTTTCCGCGGAG
TGCTTGATACAGGGGCCGATATTAGTGTTATTTCTGATAAATATTGGCCTACTACATGGCCA
AAACAGATGGCTATTTCCACTCTCCAGGGTATTGGCCAAACTACCAATCCAGAACAGAGTTC
ATCCCTTCTTACTTGGAAGGATAAAGATGGACATACAGGCCAATTTAAACCTTATATTCTGC
CCTATCTTCCAGTTAATCTATGGGGGCGTGATATATTAAGCAAAATGGGTGTTTATTTATATA
GTCCTTCACCCACTGTGACAGATTTGATGTTAGATCAGGGCTTACTTCCAAATCAAGGTTTAG
GTAAACAACATCAAGGCATCATTTTGCCCCTTGATTTAAAAATCTAATCAAGATCGAAAAGG
CTTGGGGTGTTTTCCTAGGGACCTCTGATTCTCCTGTGACACATGCCGATCCTATTGATTGGA
AATCTGAGGAACCGGTATGGGTCGATCAGTGGCCCCTAACACAGGAAAAACTTTCTGCCGC

ACAACAGCTGGTGCAGGAACAGCTGAGACTTGGGCATATTGAACCCTCTACCTCTGCTTGGA
ATTCCCCAATTTTTGTTATTAAAAAGAAGTCTGGGAAATGGAGATTGCTACAAGATCTTCGT
AAGGTAAATGAAACAATGATGCATATGGGAGCCCTACAACCTGGGTTGCCCACTCCTTCTGC
TATACCTGATAAATCCTATATCATTGTTATAGATTTAAAAGATTGTTTTTACACTATTCCTCTT
GCACCTCAAGATTGCAAAAGATTTGCTTTCAGTTTACCCTCTGTTAATTTTAAAGAGCCTATG
CAACGCTATCAATGGAGAGTTCTCCCGCAAGGAATGACTAATAGCCCTACGCTGTGCCAAAA
ATTTGTTGCTACAGCAATAGCTCCGGTTCGTCAACGTTTTCCTCAGCTATATTTGGTTCATTA
TATGGATGATATATTACTAGCTCATGCTGACGAACATCTATTGTATCAAGCTTTTTCGATTCT
AAAACAACATTTAAGCCTTAATGGTCTTGTTATTGCTGATGAAAAAATTCAGACTCATTTTCC
TTATAATTATTTGGGTTTCTCCTTATATCCTCGTGTTTATAGTACCCAATTAGTAAAACTGCA
GACTGACCATTTGAAAACTCTAAATGACTTTCAAAAACTTTTAGGAGACATTAATTGGATAC
GTCCTTATTTAAAATTACCCACTTATACCTTGCAGCCATTATTTGACATCCTTAAAGGTGACT
CTGATCCTGCGTCACCCCGAACACTTTCTTTAGAAGGACGAACTGCTTTACAATCAATAGAA
GAAGCTATTAGACAACAACAGATTACTTATTGTGATTACCAACGATCATGGGGTTTGTATAT
ACTTCCTACCCCCCGAGCACCCACAGGGGTTCTCTATCAAGATAAACCTTTGCGATGGATAT
ATTTGTCTGCTACTCCAACTAAACATCTGCTTCCTTACTATGAACTTGTTGCAAAAATTGTAG
CAAAGGGACGTCACGAGGCCATCCAATATTTTGGTATGGAACCCCCCTTCATTTGTGTTCCTT
ATGCTTTAGAACAACAAGATTGGCTTTTTCAATTTTCAGATAATTGGTCTATAGCTTTTGCAA
ATTACCCCGGACGGATTACTCATCATTACCCTTCTGATAAATTGTTACAATTTGCTAGCTCTC
ATGCCTTTATTTTTCCAAAAATAGTTCGCCGACAACCTATTCCCGAAGCGACACTTATATTTA
CAGATGGATCTTCTAATGGAACTGCAGCTTTAATCATTAACCATCAAACCTATTACGCACAA
ACCAGTTTTTCTTCTGCTCAAGTTGTGGAATTATTTGCAGTCCACCAAGCGTTGCTAACTGTA
CCTACTTCCTTCAATTTATTTACAGACAGCTCCTATGTGGTCGGTGCCTTACAGATGATTGAA
ACTGTTCCAATTATCGGCACCACCTCTCCTGAAGTTCTTAACTTATTTACATTGATTCAACAG
GTTCTCCATTGCCGCCAACACCCCTGTTTCTTTGGACATATTCGTGCACATTCCACCCTTCCT
GGTGCCCTGGTACAAGGCAATCACACTGCGGACGTTCTTACTAAACAAGTGTTTTTCCAATC
AGCTATTGATGCAGCCCGAAAATCCCATGATTTACATCACCAAAATAGTCATTCTTTACGGT
TGCAATTTAAAATTTCCCGTGAAGCTGCACGGCAAATTGTTAAATCTTGCTCTACTTGTCCTC
AATTCTTTGTTCTCCCTCAATATGGTGTCAACCCTCGAGGTTTACGCCCTAATCACCTCTGGC
AAACAGACGTTACTCACATTCCTCAATTTGGGCGTCTTAAATATGTTCATGTCTCTATTGACA
CTTTTTCCAATTTTCTCATGGCTTCCCTTCACACTGGAGAATCAACACGTCACTGTATTCAAC
ACTTGCTGTTTTGCTTTTCTACTTCAGGAATCCCACAAACCCTTAAAACAGATAATGGACCTG
GTTATACTAGCCGTTCTTTTCAACGTTTTTGTCTTTCTTTCCAAATTCATCATAAAACAGGAAT
TCCTTATAATCCACAGGGACAAGGTATTGTGGAACGAGCCCATCAACGCCTTAAACATCAAT
TATTAAAACAAAAAAAGGGGAATGAACTGTATAGCCCCTCACCGCATAACGCCTTAAACCA
TGCTCTCTATGTTTTAAATTTTTTAACTTTAGACGCAGAAGGCAATTCAGCAGCCCAGCGTTT
TTGGGGAGAACGATCCTCATGCAAAAAACCACTTGTACGATGGAAGGATCCACTTACCAATC
TGTGGTATGGGCCAGACCCTGTACTATATGGGGACGAGGGCATGTTTGTGTTTTTCCACAGG
ATGCCGAAGCGCCGCGCTGGATTCCGGAAAGGCTGGTACGCGCGGCAGAGGAACTCCCTGA
CACATCAAATGCAACGCATGACACTGAGTGAGCCCACGAGTGAGCTGCCTACCCAGAGGCA
AATTGAGGCGCTGATGCGTTATGCTTGGAATGAGGCTCATGTACAACCTCCAGTGACACCTA
CTAATATACTGATCATGTTATTATTATTGTTACAGCGGATACAAAACGGGGCAGCTGCGGCT
TTTTGGGCATACATTCCTGATCCGCCTATGATTCAATCCTTAGGATGGGATAAAGAAACAGT
ACCTGTATATGTTAATGATACAAGTCTTTTAGGAGGAAAATCAGATATTCACATTTCTCCTCA
GCAAGCCAATATCTCCTTTTATGGTCTTACTACTCAATACCCTATGTGCTTTTCTTATCAATCA
CAGCATCCTCATTGTATACAGGTGTCAGCTGATATATCCTATCCTCGAGTGACTATTTCAGGC
ATTGATGAAAAAACCGGAAAGAGATCGTACCGTGACGGAACCGGACCTCTCGACATTCCGT

TTTGTGACAAACATTTAAGCATCGGCATAGGAATAGACACTCCTTGGACTTTATGTCGAGCA
CGAATTGCATCGGTGTATAACATCAACAATGCCAATACCACCCTTTTATGGGACTGGGCACC
TGGAGGAACACCTGATTTCCCCGAATATCGAGGACAGCATCCACCCATTCTTTCTGTAAACA
CTGCTCCTATATTTCAAACTGAACTGTGGAAACTTTTGGCTGCTTTTGGTCATGGCAATAGCC
TATATTTACAGCCCAATATTAGTGGGAGCAAATATGGTGATGTGGGAGTTACAGGATTTTTA
TATCCCCGAGCTTGTGTCCCTTATCCATTCATGTTGATACAAGGCCATATGGAAATAACACTG
TCATTGAATATTTATCATTTAAATTGTTCTAATTGCATACTTACTAATTGCATTAGAGGTGTA
GCCAAAGGAGAACAAGTTGTAATAGTAAAACAACCTGCTTTTGTAATGTTACCTGTTGAAAT
AACTGAAGAATGGTATGATGAAACTGCTTTAGAATTGTTACAACGCATTAATACGGCTCTTA
GCCGTCCTAAAAGAGGTCTGAGCCTGATTATTCTGGGTATAGTGTCTTTAATCACCCTCATAG
CAACTGCTGTTACTGCTTCTGTATCTTTAGCACAATCCATTCAAGCTGCTCATACTGTAGATT
CCTTGTCATATAATGTTACTAAAGTAATGGAACTCAAGAAGATATAGATAAAAAATAGAA
GATAGATTATCAGCTTTATATGATGTAGTTAGAGTTTTAGGAGAACAAGTTCAGAGCATTAA
TTTTCGCATGAAAATTCAATGCCATGCTAATTATAAATGGATTTGTGTTACAAAAAGCCTTAC
AATACTTCTGACTTTCCGTGGGATAAGGTGAAAAACATCTGCAAGGAATTTGGTTTAATACT
AATGTTTCTTTAGATCTTTTACAATTGCACAATGAAATTCTTGACATCGAAAATTCTCCAAAA
GCTACTTTGAATATAGCTGATACCGTCGATAATTTTTTACAAAATTTATTTTCTAACTTTCCTA
GCCTTCATTCGCTGTGGCGAAGTATAATTGCTATGGGCGCGGTTCTGACTGTTGTGCTTATCA
TAATTTGTCTAGCTCCTTGTCTTATTCGTAGCATTGTTAAAGAATTTCTACATATGAGAGTTTT
AATACATAAAAACATGTTGCAACACCAACATCTTATGGAGCTTTTAAAAAATAAAGAGAGG
GGAGCTGCGGGGGACGACCCGTGAAGGGTTAAGTCTTGGGAGCTCCCTGGCAGGTATGCCA
GGCCCTAGGACACGTGCCTAAGCTCCCTGTCCCGCCACCCTCAAGAGTTTTTGTAACCCTTA
AGGCTCCAAGATGTTTGGTTTCGGCAACATTTCATAGAAGATAGATTATCTTATTGTGTATAT
TTCATAGAAGATAGATATTCTGATTGTGTTCTGTATACAATGGTAAGGGTCTGGTGATTGTAT
CCTGAGATTAAAAAACAACCTTGTGAGTGCCTTAAGTCACGTACTTTACCCTATATATACCG
CAGCACAATAAAGCAAGGTATCAGCCATTTTGGTCTGATCCTCTCAACCCCATCTTTTGTCTA
TCTCTTATTTTCTTAGCGGGGACGCTCCGTTCTCTCCCTGTGCAGGTGCGACTCTTGCTTGTGC
TGGCCGCGGCA

>NC_056061.1:52960381-52967871#SHEEP_RIP_18(+)

CATAACAAAGGGTTATGAGCGTTCACTGAGTGCCCAAGGCTGGGAACGGATAATGAAGGGT
TATATGCCCGGCCTTGAGGCGTTCAAAGTCTTCCTTCTGACCTCCTGTTTCTGGGAGCAAGGA
CTGTTGTTTCATGATAAGACTCCTTTCAGAGTTTCGCCAAAGCTATGTTATGGCTTGGGTGGT
GGGAACTGTATTTTATACTTGAATGCTTTGATGTTTTATCGAGAAAGGCTACATGCAAGTCTG
CTTTATGCTCTGCTCCCTGAGACCATATATCTGCAAAAAGATGGATAATAAAATTTGTCAGT
CCACTAGAGGCTGTCCCTGAGTGTTCTTTTCAGAGTGCAGTTCTCCAAGCCTTAGAACTGGC
ACCCGATGTGGGGCTTGAAGCAGCAGACTGATTTTGAAAAAGGGCCGCACTCCTGCAGAAG
CGAGGTAAGCAGAATGGGACATCAGACAAGTAAAATTCCTTTTGTTCATCTCATGCATCATT
TCTTGAAACAATAAGGGGTTAATCTGCTTGAAGAGCAGTTAACTAGCTGTTACCAGACAGTG
GTTGAATATAATCCATGGTTTCTGGAGGAAGGAACCTTGGATTTACAAACCTGGACCAGAGT
AAAGAACAATGTTTTAAAAGCTTATAGACAGGGGGTAAAAATTCCCCCACAATGGTGGGTA
ACTTGGTCTCTTTCACGGGCTGTTATGGAACAATTAGATGGCTCAGGAGGAGATTTAGAGGT
TGAAACTGTTCAATCCCTGCATGAATGTGAATTAGAAGAGAAAGATCTATCAGAGGTTTTAA
ATCAGAAAAATGTCATGCTAGAGCAGGTTACGGACAAACAAGAGTCACAGATGCTGAAAGC
AGTTAAACAATCAACTTTGCCAGAGGCCCCTGATCCGCCTTTACCTTGCTTTTCGCAAGCTCA
TATTAACAAGCCTCCAACACCGGCCTTTGCCTTTCCACCGGCTGCAACGCTGTCTGCTGCTGC
TACTGCTCCCCTATTTCCTAGCAGTCCTCCTCGGGACATGCCTCTGTGCGTGGGCTTTTCCGG

TCCAATTTAATAATCCTCAACCTGGACATAATCAATGGCAATCACCTGATTTTGGTTTGCTCA
CGCAATTCAAAAAGGCATGTACGTTATATGGACCTACCTCACCCTACTGTATGGAATTTCTT
AGGGGCTGGGCTGATCAGTAGCTTCATGTGGATTTTTTTACAGTCGCTAAAATGGTTATGAC
TCCACAACAGCTACTACAATGGCAAATGTGGGTCACGGATGAAGTCAAATTAATCTTGCAAG
AACAGCAAAGCGGGGAAAACCCTGCTGGAGTAAATTTTGAAATTCTCACCGGCACCGGAGC
TATGGCTGAAACTGCTGCGCAATTACAATTAGTGCAGCCGCCCATGTTATACTGGATTAAGG
AAGCAGCTATCAGAGCATGGGCTAAAATCGACAGTTCCACCTCTGATGGATCTTTTGTAAAA
ATATTGCAGGGACCAACTGAAGAATATGCTCAATTTATTGGTAAATTGAAAGAGGCCATTGA
TCACAGTCTTAAGGATGAATCTTTGTGAGAAATCATTTTGAAACAACTGGCTTTTGATAATG
CTAATGAAGATTGTCTATAATAGGCTATTATCAGACCTATTAGGGAGCAAGGACGAGTTATA
GAATACTTGAAAGCCTGCAGGAAGGTGGAGACGATTCAACATAAAGCTAAAATAGCTGCTT
TAGAAACCTTAAATGTTTCCCAAAAGTCTAAAGTTAAATGTTTTAACTGCGGCAAGCCGGGA
CACAGGCGGAAGCAGTGTCACTTGCCTTGGCAAACAGGTCTTTCTCCTGACAGAGGAGGGG
CTATTAAAACTAAGCACCCCCGGGCTCTGCCCAAGATGTAAAAAGGGGAATGATTGGCTGA
GTGAATGCCACTCTAGATTTGATAAACAAGGTAGTGCTTTACCCATTCAGACACCTCCTTCG
GGAAACTAGAACAGGGACTCTCCTCTAGCCCCGTTAAACAAGGAGGACAACCAAATATTAC
TGAATTAACAGCGGCTACCAGACATAGCGCATGTGTAGACATTCTGCTCCCCGAGATATGGA
ATTATTAATGGTAAATAATCCTATGAAAATCTTAGCTGGATATTTTGGCCCTATACCAAAAA
ATACTGTAGGCCTCCTGTTGGGAGGAAGCAGCAGCACCATGCGTGGGATAATTGTACATACT
GGGATCATTGATGAAGATTACATGGGTGAAATTGCAATAATGTTACATGTGACTCGTAGCTT
GTATTTACAAAAGGGTGACAGATTTGCTTAGCTATTGCTATTACCTTATGTGCCCCCACTTAA
TAGAAAAGCAAACACCAGAACAGGTGGCTTTGGGAGTACCAACGTTACTGCAGCCCTCTGT
ACTGTTATAAAAGAAATCAATAGGCCCATGTTAAAATTAAAAATCAGAGGAAGAACTTTTG
AAGGAATGTTAGACACTGGGGCAGATATTTCCATCATAAGAACAGAGGAATGGCCTTCAGA
TTGGCCTGCAGTTTTAGCCTCACACCAGTTGGTGGGAGTAGGAACTGCAGATGCAGCTCAAA
CTTATGTTAGCTCATCTTACTTAAAAGCCCTGGGCCCTGATCAATTAGTCGGTTACATTAAAC
CGTACATTGCTCCATTACCGTTAAATTTGCGGGGAAGAGACTTTCTACAAGCTCAAGTGACT
ATACAATTGAATGAAACTTTTTCTTAGGGGTCACTGAGATAAAGCCACTGAAGTTAGAATGG
AAGTCTGATAAACCTATCTGGACAGCTCAGTGGCCCCTATCAAAAGAGAAACTGTCCGCTTT
GCATACTTTGGTGGCTGAACTACTACAACAAAATAGAATAGAAAGTACTTAATCACCATGGA
ATTCACCAATTTTTGTCATTAAAAAGAAATCAGGTAAATGGAGAATGCTAACAGGAATATTA
ACACTATAATGATTCCTATGGGAGCATTATAACCAGGACTCCCAAGCCTTGCTATGGTCCCT
AAGGACTGGGCTGTTATGATTATAGATTTACAAGATTGCTTTTTCACTATACCTTTACATCCA
GATGACAGGCAACATTTTACCTTCTCAATACCTTCCATTAATAATCAAACCCCTGTTCAATGG
TATCAATGGAAGGTCCTGCCCTAAGGTATGATGAACTCTGTTATGGTCTGTCAATTCGTTGTT
GATAAAATTTTGCAGCCCATCAGACAGCAATTCCCTGAGGCATATCTCATTCATTACATGGA
TGACATTTTATTGGCTTCTCCCTCAGAATCTCAATTAAGTTTATTATGTAATGAGGTCAGAAC
TAATTCAACTAATCATGGGCTGCTAATAGCAGAAGATAAATTGCAACATCATTCCCCTATTA
AATATCTTGGATATCTTATGGACCGCTCCACTGTAAAGCCGCAGAAACTTTCTATTAGAAGG
GATAATTTACATACACTTAATGATTTCCAGAAACTTCTTGGGGATATTAATTGGCTACGACCC
ACCTTGGGAATTCCCACATATGCTTTACAAAACTTATTCAAATTATTAGACGGTTCCTCTGAT
TTGAATAGTTCCCAACAACTTACCCCTGAAGCTGAGGAAGAATTACAATTGGTAGAACAGA
GAATTCAACAGGCATTTGTTTACTGTATTAATTATAATTCCCCTTTTCAGATATATGTCTTTG
GCACTAAGATATCGCCTACCGCCATTATAGTGCAGGATAATCACCCTATTGAATGGGTATAT
CTCCATTCCAAACAGACTAAACACATTGTTTCCTATATAGACTTAATAGGGAAAATCATTTTT
CTTGCACGCTCTCACTTGTGCGCTATAGCTGGATATGACCCTACTCAGATTTACCTACCTTTG
ACAAAAATAGAAATTGATAATGCTCTCCAGGTGTCCACTACCATTCAGATAGCCCTTGCTGA

TTATTCAGGGGAGTTATTGGCCAACCCACCTGAAGGAAAATTATGGAATTTCTTACAAAATA
CTTCTTTTATCATTAACAATATTGTTTCTGAACACCCTCTCATGAATGCACCTAATTATTTTAT
AGATGGAAATAAGGCAGGATGGGCAGCCATAATAGGTCCCAACCTGCAAAAGAAAATTAAA
AGTCCTTATCAATCCATTCAAAAAACAGAATTATTTGCATTATATTGTTTACTTACTCTAATA
AAAACCCCATTAAATGTTTTAACTGATTCTCGCTACGTGGCACATCTTTTCCCATCTTTGTAA
CGGCTCATTTTATATCCAATGAAAATGATCTTATACATTTGTTCTTATTGATTCAACAAGAAA
TAAAAGCTAGACTCCATCCCTTCTTTATTACTCACATTCGTGCTCATTCCCGTTTACCAGGAC
CCCTCAGTTTAGGCAATGATTTGGCTGATCGCCTCATCGCCCCTATATTTTCTTTCCCCGAAC
AGGAACATCAGCTCTTCCATACTAACGCTAATAGACTACACGTTCAGTATAAGATACCGTTA
CAAATGGCTAGAAAAATTGTTCAGGACTGTGCCGCATGTGCCCCCTTTCATTTGACCACTAG
TCCCCAAGGGACTAATCCTAGAGGCTTACACGCAATGGCAAGCTGATTTTACACATTACAAA
CTGCCCCCTTTTAAATCATTATTTATAGTCATAGACACCTTTTATGGCTTCATTTGGGCAGTTC
CTTCCACCGCTGAGACTACTAAAGCTGCCGTCACAGCTTTTCTGCAATGTTTTTCAGTGATGG
GGATCCCTGCCTCCATCAAAACAGACAATGGTCCTGCTTTTACAGCCAATGCTTTTTGTGATT
TCATGCATCAGTGGGGGTATTTGCCATCTTACTGGCATCCCGTACAACCCTCAAGGTCAAGC
CATCATTGAATGGGCCCACTGTACACTCAAGCTCGTTCTTAATAAACAAAACAGGGGGAATA
GACTAAGGGACCCCTATGGATCTAAAGCCATTTTGCCTATAGCCCTTTTAACAATCAATTATT
TTAATTTGCCTCTACACAGTCAGGAAACACGAGCAGAGTGACATTTTTCTGATTCACCCTCA
CGTATGCCTGAACAAACTGCTCTTTGGGTTAAATGTCTTGATCAATGGCGGCCAGGAACACT
TAAGTTCCTAGGCAAGGAATATTGTCTTGTCATTTTAGATGATGGAACCGAATAGTGGGTCC
CACTCAGAAGAGTCAGAAGACGGGCAGGCCTTACTCCACACCCCCCGACCGGTAACTAAAA
CAAAAATTGCGACAGATGACACTGAAAGACAGCAAGAAAATGAAACGTCCCTGACGCACTG
CGCCTTTTCCGACCTGGGCACAAATGAAAAATCTGTCCAGACGAGCTGAGGATACTCTGTTG
ATGACCAACAGTGAGGTAACACCGGAAAAACTGTTGCTGGCCATGATGGCCGTTTTAACCTG
TGCTTCTGGGGTAAGTGGTAATTACACCTATTGGGCTTATATCCCCAACCTGCCTCTTTTACA
AGTGGTGGATTGGACAGAATCATGATAGCTTACATTTTCCTGCCCCCTGGTCGGATCTAGGA
CCACGAATAAAAGAAGAAGAAGGAAAGACAGTTCGGTGCCCTCCCTTTGTGTCTCCGCCTAT
ATCCACAATGGTGGGCTTACTCTTTCTCAAATGGCTCTTTCCTTCTTGGAATGTTCGCTACCA
TAACCTTTGTTTTTAACTGGACAGAGACTACCTACCATGGACAAATAGCCAACTTCAACCAC
TATCTATTTAATTCTTCTGAGCCTCCTTGTGACAACGTTATCTTGGAAGAAAAAATCATCTGT
GCCAGGAATGGACTGGTCCACCTGTAGAGGAAGGTTTGGAAAAGTTTCTGTTCATGCCAACA
TGACTTTTGTTGATTGGGGGCCTCATGGACTCTGTGAATTGCCCAGAATCACAAGAAAATAA
TAACACCTGCCAATGGTATAATGTTTCAGCACCACATTTTAACAGAACACAAACAGGACTCT
GGCATCAATGCGATGAACTTTTGAACTGGTACAATGGAGGTCTTTCACCTCCCAGACCCTGG
ATAATCAGTCCAGTCTTGGGGCCCGAACACTGGCATCTTTGGAAAATTCCTGCTTGTTTGTCT
CGGTTTAGTGTCTCATATGCTTATCACATGTTTCCATCCCACAAAATTATACTATTGAGTATA
ATTATACTGGTTATGTTCGTGCTTGTGTAAATGCAGCCTATCTTTTTGCTATTGGGCAATTTA
GGAGTAATGGCTCAATTCTGTCCTGTACTGAGTGTCATTTGTATACTTGTTTAAATCATAGTG
TGCCAATTAATGTTACTAAAGACAGTGTTTTTTAAGTCTGGCAAAGGACTGATTTGTGGGTTC
CAGTTAAGATTTCTGAACCTTGGTCAGATTCCACGTTGTTGTCTTTTGTTCTGAGAGAATCCC
TAAAAAGAAGCAAATGCTTTATTGGCTGGATTATAGCTGCCATAGTGGGTATTCTTTCAGTT
GTAACTGTTGGTACAGTTTCTGGAAAGGCATTGTGTAATTCTATTCAAAATCATGATTTCATT
AATGCTTGGAAAAAGGATTCTCATGATCTCTGGGCCCGGCAAGCTCAGATAGATCAACAAAT
ACAAACACGCTTAGATGACCTACAAGCCGCCCTTATGTATGTGGGGGATGATCTGCATGCTT
TACAGATCCAGTTGAAGTTGCGGTGTCACTGGAATTTCACTACTTTCTGTTTGACCAATATGC
CATACAATGCCACTGAATATCCTTGAGAACAAATAAAGTTACACCTTTTAGGTTCGAAATCA
AACACTAGTCTAGATATAGAGAAACTGAAGCAACAAATCATGCCTACCTTTAGCAGCATGCC

CCCTGTGTTATATAACACTGACTTCTGTAATACACTATCCGCTAGTACTTCCTTTTTAAATCC
AAAAAAATGGGTACCCCATACGATGGCTTCTTATGCGCTGATCTGTTTATTGTTTGTTATTGT
TCTTATAGGATTCCGAACGTTATGTGCTTGAGCCACTGCCGCCCAAAAAGCAGGAGTAACCA
TGGCTGCAGCAGTCCTTGCTCTTGAGGAAAAAGGGAGGAAATGCGGAGGAGCTGGAAGGCT
CCTAAGCATACACTGAGAATGTATTTGCTCCACTCATGACGAAGGTTGGAAGCTGGAATGAG
CATAACAAAGGGTTATGAGCGTTCACTGAGTGCCCAAGGCTGGGAATGGATAATCAAGGGT
TATATGCCTGGCCTTGAGGCATTTGAAGGCTTCCTTCTGACCACCTGTTTCTGGGAGCAAGG
ACTATTGTTTCATGATAAGACTCCTTTTAGAGTTTTGCCAAAGCTATGTTATGGCTTGGGTGG
TGGGAACTGTATTTTATGCTTGAATGCTTTGATGTTTTATCGAGAAAGGCTACATGCAAGTCT
ACTTTATGCTCTGCTCCCTGAGA

>NC_056063.1:18713439-18721364#SHEEP_RIP_19(+)

TGCGGGGGACGACCCGTGAAGGGTTAAGTCTTGGGAGCTCCCTGGCAGGTATGCCGGGCCC
TAGGACATGTGCCTAAGCTCCCTGTCCCGCCACCCTCAAGAACTTTTGTAACCCTTAAGGCTC
CAAGATGTTTGGTTTCGGCAACATTTCATAGAAGATAGATTATCTTATTGTGTATACTTCATA
GAAGATAGATATTCTGATTGTGTTCTATATACAATGGTAAGGGTCTGGTGATTGTATCCTGA
GATTAAAAAACAACCTTGTGAGTGCCTTAAGTTACGTACTTTACCCTATATATACCGCAGCA
CAATAAAGCAAGGTATCAGCCATTTTGGTCTGATCCTCTCAACCCCATCTTTTGTCTATCTCT
TATTTTCTTAGCGGGGACGCTCCGTTCTCTCCCTGTGCAGGTGTGACTCTTGCTTGTGCTGGC
CGCGGCAGGTGGCGCCCAACGTGGGGCTCGAGCTCGACAGTTTTCCTCGCCACTACTCTTAT
TAATTGAAAAGAGTGAGTATATGAGTAAACAAGTGAATTAATTTGAGGAGGAGTAGTAAGG
TATATAGTTGAGAGTATAAATATGGGACAGACGCATAGTCGTCAGTTGTTTGTGCATATGTT
ATCTGTAATGTTAAAACATAGGGGAATTACTGTTTCTAAACCTAAATTAATCAATTTTCTTTC
ATTCATCGAGGAAGTTTGCCCTTGGTTCCCCAGAGAAGGTACAGTAAATTTAGAGACATGGA
AGAAGGTAGGGGAACAAATTCAGACTCATTATACTTTACATGGCCCTGAAAAAATCCCTGTC
GAAACTTTATCCTTTTGGACACTAATTCGTGACTGCCTGGACTTTGATAATGATGAATTAAAA
CGTTTAGGAAATTTATTAAAACAGGAAGACGATCCTCTCCATGTTCCTGATTCGGAACCCAG
GTATGCTGTTCCCGAGGGGGTTGAAAGCAACCCTCCGTTTTCTAACTTATTGCGTCCTTCGGA
TAATGATGATTTACTTTCATCCACAGATGAGGCAGAATTAGACGAAGAAGCTGCTAAATACC
ATCAAGAAGATTGGGGTTTTTTAGCACAAGAAAAAGGGGCGTTAACATCTAAAGATGAATT
GGTTAAATGTTTTAAAAACCTCACTATTGCTTTACAGAACGCAGGAATCAAGCTTCCTAGTA
ACAATGCCAAATCTCCTTCTGCTCCGCCTCTTCCCCCTGCTTATGCTCCTTCTGTTGTGGCTGG
TCTCGATCCCCGTCCAGGGCCCCCTCCACCGTCTGAGAACATGTCTCCGCTGCAAAAGGCAT
TGAGACAGGCACAGCGACTTGGTGAGGTTGTCTCTGATTTTTCTCTTGCTTTTCCTGTCTTTG
AAAATAACAACCAGCGTTATTATGAATCACTGCCTTTTAAACAACTGAAAGAGTTAAAGATT
GCTTGCTCACAATATGGTCCTACCGCTCCATTCACCATTGCTATGATAGAAAATTTGGGTACT
CAAGCTTTACCTCCAAATGATTGGAAGCAGACAGCTAGGGCATGTCTCTCAGGGGAGATTAT
TTATTATGGAAATCTGAATTTTTGAACAATGTGCTCGTATAGCTGATGTTAACCGACAGCAA
GGTATACAGACCTCCTATGAAATGTTGATTGGTGAAGGCCCTTACCAGGCTACTGATACTCA
ACTTAATTTCTTACCTGGTGCATATGCACAAATATCAAATGCGGCTCGGCAGGCATGGAAAA
AACTTCCTAGCTCCAGTACTAAGACAGAGGATCTTTCAAAAGTCCGGCAGGGACCTGATGAG
CCTTACCAGGACTTCGTGGCACGACTTTTAGATACTATAGGTAAGATAATGTCAGATGAACA
GGCTGGGATGTTATTGGCAAAACAATTGGCTTTTGAAAACGCTAACTCTGCTTGTCAAGCTG
CTTTAAGACCTTATCGAAAAAGGAGATCTGTCTGATTTTATTCGCATTTGTGCTGACATTGGA
CCCTCCTACATGCAAGGCATTGCTATGGCAGCAGCATTACAAGGAAAAAGCATAAAGAGGT
ACTTTTCCAGCAGCAAGCCCGGAACAAGAAAGGACTTCAAAAGTCAGGTAATTCGGGTTGC
TTTGTTTGTGGTCAGCCTGGCCATCGGGCTGCAGTGTGCCCTCAAAACAACAAAGCCCTGTT

AACACTCCTAATTTGTGCCCACGCTGTAAAAAAGGAAAGCATTGGGCGCGGGATTGCCGTTC
CAAAACGGATGTTCAAGGTAATCCTTTGCCCCGGTTTCGGGAAACTGGGTGAGGGCCAGCCC
TGGCCCCGAAACAATGTTATGGGGCAACACTGCAGGTTCCAAAGGACCATTGCAGACCTCTG
TCGAGCCACAAGAGGCAGCGCGGGATTGGACCTCTGTGCCACCTCCTACACAGTATTAACTC
CCGAGATGGGGGTCCAAACCCTTACCACAGGAGTGTTTGGGCCTTTACCTCCAGGGACAGCT
GGACTGCTTTTAGGGCGCAGCAGTGCGTCTTTAAAAGGAATACTTATTCATCCTGGTGTGAT
TGACTCTGATTATACAGGAGAGATAAAAATATTAGCCTCCGCTCCTAACAAAATTATTGTGA
TCAATGCAGGACAGCGTATAGCTCAACTTCTTTTAGTTCCATTAGTCATACAAGGAAAAACA
ATTAACCGAGACCGTCAAGATAAAGGTTTCGGGTCCTCTGACGCCTTTTGGGTGCAAAATGT
TACCAAGGCACGACCAGAACTTGAGCTACGCATTAATGGTAAGCTTTTCCGCGGAGTGCTTG
ATACAGGGGCCGATATTAGTGTTATTTCTGATAAATATTGGCCTACTACATGGCCAAAACAG
ATGGCTATTTCCACTCTCCAGGGTATTGGCCAAACTACCAATCCAGAACAGAGTTCATCCCT
TCTTACTTGGAAGGATAAAGATGGACATACAGGCCAATTTAAACCTTATATTCTGCCCTATC
TTCCAGTTAATCTATGGGGGCGTGATATATTAAGCAAAATGGGTGTTTATTTATATAGTCCTT
CACCCACTGTGACAGATTTGATGTTAGATCAGGGCTTACTTCCAAATCAAGGTTTAGGTAAA
CAACATCAAGGCATCATTTTGCCCCTTGATTTAAAACCTAATCAAGATCGAAAAGGCTTGGG
GTGTTTTCCTAGGGACCTCTGATTCTCCTGTGACACATGCCGATCCTATTGATTGGAAATCTG
AGGAACCGGTATGGGTCGATCAGTAACCCCTAACACAGGAAAAACTTTCTGCCGCACAACA
GCTGGTGCAGGAACAGCTGAGACTTGGGCATATTGAACCCTCTACCTCTGCTTGGAATTCCC
AATTTTGTTATTAAAAGAAGTCTGGGAAATGGAGATTGCTACAAGACCTTCGTAAGGTAAAT
GAAACAATGATGCATATGGGAGCCCTACAACCTGGGTTGCCCACTCCTTCTGCTATACCTGA
TAAATCCTATATCATTGTTATAGATTTAAAAGATTGTTTTTACACTATTCCTCTTGCACCTCA
AGATTGCAAAAGATTTGCTTTCAGTTTACCCTCTGTTAATTTTAAAGAGCCTATGCAACGCTA
TCAATGGAGAGTTCTCCCGCAAGGAATGACTAATAGCCCTACGCTGTGCCAAAAATTTGTTG
CTACAGCAATAGCTCCCGTTCGTCAACATTTTCCTCAGCTATATTTGGTTCATTATATGGATG
ATATATTACTAGCTCATGCTGACGAACATCTATTGTATCAAGCTTTTTCGATTCTAAAACAAC
ATTTAAGCCTTAATGGTCTTGTTATTGCTGATGAAAAAATTCAGACTCATTTTCCTTATAATT
ATTTGGGTTTCTCCTTATATCCTCGTGTTTATAATACCCAATTAGTACAATTACAGACTGACC
ATTTAAAAACTCTAAATGACTTTCAAAAACTTTTAGGAGACATTAATTGGATACATCCTTATT
TAAAATTACCCACTTATACCTTGCAGCCATTATTTGACATCCTTAAAGGTGACTCTGATCCTG
CGTCACCCCGAACACTTTCTTTAGAAGGACGAACTGCTTTACAATCAATAGAAGAAGCTATT
AGACAACAACAGATTACTTATTGTGACTACGAACGATCATGGGGTTTGTATATACTTCCTAC
CCCCCGAGCACCCACAGGGGTTCTCTATCAAGATAAACCTTTGCGATGGATATATTTGTCTG
CTACTCCAACTAAACATCTGCTCCCTTACTATGAACTTGTTGCAAAATTGTAGCAAAGGGAC
GTCACGAGGCCATCCAATATTTTGGTATGGAACCCCCTTCATTTGTGTTCCTTATGCTTTAGA
ACAACAAGATTGGCTTTTTCAATTTTCAGATAATTGGTCTATAGCTTTTGCAAATTACCCGGG
ACGGATTACTCATCATTACCCTTCTGATAAATTGTTACAATTTGCTAGCTCTCATGCCTTTATT
TTTCCAAAAATAGTTCGCCAACAACCTATTCCCGAAGCGACACTTATATTTACAGATGGATC
TTCTAATGGAACTGCAGCTTTAATCATTAACCATCAAACCTATTACGCACAAACCAGTTTTTC
TTCTGCTCAAGTTGTGGAATTATTTGCAGTCCACCAAGAGTTGCTAACTGTACCTACTTCCTT
CAATTTATTTACGGACAGCTCCTATGTGGTCGGTGCCTTACAGATGATTGAAACTGTTCCAAT
TATCGGCACCACCTCTCCTGAAGTTCTTAACTTATTTACATTGATTCAACAGGTTCTCCATTG
CCGCCAACACCCCTGTTTCTTTGGACATATTCGTGCACATTCCACCCTTCCTGGTGCCCTGGT
ACAAGGCAATCACACTGCGGACGTTCTTACTAAACAAGTGTTTTTCCAATCAGCTATTGATG
CAGCCCGAAAATCCCATGATTTACATCACCAAAATAGTCATTCTTTACGGTTGCAATTTAAA
ATTTCCCGTGAAGCTGCACGGCAAATTGTTAAATCTTGCTCTACTTGTCCTCAATTCTTTGTT
CTCCCTCAATATGGTGTCAACCCTCGAGGTTTACGCCCTAATCACCTCTGGCAAACAGATGTT

ACTCACATTCCTCAATTTGGACGTCTTAAATATGTTCATGTCTCTATTGACACTTTTTCCAATT
TTCTCATGGCTTCCCTTCACACTGGAGAATCAACACGTCACTGTATTCAACATTTGCTGTTTT
GCTTTTCTACTTCAGGAATCCCACAAACCCTTAAAACAGATAATGGACCTGGTTATACTAGC
CGTTCTTTTCAACGTTTTGTCTTTCTTTCCAAATTCATCATAAAACAGGAATTCCTTATAATCC
ACAGGGACAAGGTATTGTGGAACGAGCCCATCAACGCCTTAAACATCAATTATTAAAACAA
AAAAAGGGGAATGAACTGTATAGCCCCTCACCGCATAACGCCTTAAACCATGCTCTTTATGT
TTTAAATTTTTTAACTTTAGACGCAGAAGGCAATTCAGCAGCCCAGAGTTTTTGGGGAGAAC
GATCCTCATGCAAAAAACCACTTGTACGATGGAAGGATCCACTTACCAATCTGTGGTATGGG
CCAGACCCTGTACTAATATGGGGACGAGGGCATGTTTGTGTTTTTCCACAGGATGCCGAAGC
GCCGCGCTGGATTCCGGAAAGGCTGGTACGCGCGGCAGAGAACTCCCTGACACATCAAATG
CAACGCATGACACTGAGCGAGCCCACGAGTGAGCTGCCTACCCAGAGGCAGATTGAGGCGC
TGATGCGATATGCTTGGAATGAGGCTCATGTACAACCTCCAGTGACGCCTGCAAAAATACTG
ATCATGTTATTATTATTGTTACAGCGGATACAAAACGGGGCAGCTGCGGCTTTTTGGGCATA
CATTCCTGATCCGCCTATGATTCAATCCTTAGGATGGGATAAAGAAACAGTACCTGTATATG
TTAATGATAGAAGTCTTTTAGGAGGAAAATCAGATATTCACATTTCTCCTCAGCAAGCCAAT
ATCTCCTTTTATGGTCTTACTACTCAATACCCTATGTGCTTTTCTTATCAATCACAGCATCCTC
ATTGTATACAGGTGTCAGCTGATATATCCTATCCTCGAGTGACTATTTCAGGCATTGATGAA
AAAACCGGAAAGAGATCGTACGGTGACGGAACCGGACCCCCTCGACATTCCGTTTTGTGAC
AAACATTTAAGCATCGGCATAGGAATAGACACTCCTTGGACTTTATGTCGAGCACGAATTGC
ATCGGTGTATAACATCAACAATGCCAATACCACCCTTTTATGGGACTGGGCACCTGGAGGAA
CACCTGATTTCCCCGAATATCGAGGACAGCATCCACCCATTCTTTCTGTAAACACTGCTCCTA
TATATCAGACAGAACTATGGAAACTTTTGGCTGCTTTTGGTCATGGCAATAGCCTATATTTAC
AGCCCAATATTAGTGGAAGTAAATATGATGATGTGGGAGTTACAGGATTTTTATATCCCCGA
GCTTGTGTCCCTTATCCATTCATGTTGATACAAGGCCATATGGAAATAACACTGTCATTGAAT
ATTTATCATTTAAATTGTTCTAATTGCATACTTACTAATTGCATTAGAGGTGTAGCCAAAGGA
GAACAAGTTATAATAGTAAAACAACCTGCTTTTGTAATGTTACCTGTTGAAATAACTGAAGA
ATGGTATGATGAAACTGCTTTAGAATTGTTACAACGCATTAATACGGCTCTTAGCCGTCCTA
AAAGAGGTCTGAGCCTGATTATTCTGGGTATAGTGTCTTTAATCACCCTTATAGCAACTGCTG
TTACTGCTTCTGTATCTTTAGCACAATCCATTCAAGCTGCTCATACTGTAGATTCCTTGTCAT
ATAATGTTACTAAAGTAATGGGAACTCAAGAAGATATAGATAAAAAAATAGAAGATAGATT
ATCCGCTTTATATGATGTAGTTAGAGTTCTAGGAGAACAAGTTCAGAGCATTAATTTTCGCA
TGAAAATTCAATGCCATGCTAATTATAAATGGATTTGTGTTACAAAAAAGCCTTACAATACT
TCTGACTTTCCGTGGGATAAGGTGAAAAAACATCTGCAAGGAATTTGGTTTAATACTAATGT
TTCTTTAGATCTTTTACAATTGCGCAATGAAATTCTTGACATCGAAAATTCTCCAAAAGCTAC
TTTGAATATAGCTGATACGGTCGATAATTTTTTTACAAAATTTATTTTCTAACTTTCCTAGCCT
TCATTCACTGTGGCGAAGTATAATTGCTATGGGCGAGGTTCTGACTGTTGTGCTTATCATAAT
TTGTCTAGCTCCTTGCCTTATTCGTAGTATTGTTAAAGAATTTCTACATATGAGAGTTTTAAT
ACATAAAAACATGTTGCAACACCAACATCTTATGGAGCTTTTAAAAAATAAAGAGAGGGGA
GCTGCGGGGGACGACCCGTGAAGGGTTAAGTCTTGGGAGCTCCCTGGCAGGTATGCCGGGC
CCTAGGACATGTGCCTAAGCTCCCTGTCCCGCCACCCTCAAGAACTTTTGTAACCCTTAAGG
CTCCAAGATGTTTGGTTTCGGCAACATTTCATAGAAGATAGATTATCTTATTGTGTATACTTC
ATAGAAGATAGATATTCTGATTGTGTTCTATATACAATGGTAAGGGTCTGGTGATTGTATCCT
GAGATTAAAAAACAACCTTGTGAGTGCCTTAAGTCACGTACTTTACCCTATATATACCGCAG
CACAATAAAGCAAGGTATCAGCCATTTTGGTCTGATCCTCTCAACCCCATCTTTTGTCTATCT
CTTATTTTCTTAGCGGGGACGCTCCGTTCTCTCCCTGTGCAGGTGTGACTCTTGCTTGTGCTG
GCCGCGGCA

>NC_056063.1:24352091-24359775#SHEEP_RIP_20(-)

TGAAGGGTTAATAGGGTAGCAGAGATGTGCCTGCAAATGGGCCTCTCTGCTAGGGCTGGAC
ATCCTTGCAAATGAGGTGTTCTGCCAAAGAGTCTGGACACAGCCTTGAGTTTAATGGTCCCT
TGCAAACAGGGAGCATTCCCTTCTTGTGATAAAGAAGGAATAGAGGGCTTTGTACAGACTCT
GCAGTAGACTAGGATTTTACTCCCCTTTGCTATATGATAACATATATGCACCTGCGCTGTGCT
GAAAAGGCTCATTCATGCAGTCTGGAATTCTGCCTAGGGGGCTTTTATAATAAACAGCAATT
AGTTTTTTGCCCAGTTCTGTTCCTCTGGCCGGAGTGTGCGTGTCATCTGTCTCTTGTGTGTCTT
GTTCTGTGTCATTTCACTCGTAATCTCCAACATCTGGCACCCAACGTGGGGTTCGAGTGAAA
CCGAAAGGGTGAGTAACCCCGGGGGGATTTTAAATCCATAGCAGGGGAACTTTTGGGAAAA
TTGTGAGGAATTCCTCACCCTAGCAGAGGGGAGCTTTCAAAAAAATCGTGGGGAATTCCTCA
TCATTACGGGCACAATACATGGAGTTAGTCCAAAGGCTTCTCCACTCCATAGGCATTAAAGC
CTCAACTCGTCAGTTGAGTGAGCTCTTTCGCTTGGTGGAGCAATATTGTCATTGGTTTCAATA
TCAAACTAAGTTACAGTTAAACTTGAAGGAATGGAAAATAATTCAAAAGGAATTGAGAAAG
CAACATCAGAAGGGTAATGTAATCCCTTTGAAGTTATGGACTTCGTGTAGTGCTATAACACA
GGGTTTGACCTTGCTCTCTACTGATAATGAAACGAAATCTAATGCTTCAAGGAGGGGAGAAA
TAATTTATGGGGATGTGTCAGACGTTGGTGGGGCTTCTGCATCGCCTGAAGGCAAGGATACA
AATGAGCCTCCTCCTGTAAATGGTGAAACATTGGATAGTTCAGAATCAGATTTGGAGGCTTC
TTTGGTTTTGTCAGAGGAGGGCAAAGAGATTAAAGAAATGACCCATCTATTCCAGGAATGGT
GGAAATCCCTTAAGGAGGAGAAAAAATCTACACCTTCTGCTCCTGCTTGTGCTTCTCTTCTCC
CCACTGCGGTTAATCGGCCCGATGTGGGCAGGGAACATTGTCGGTTCTCCTTTCCTTTGTCTG
TGCTTCATGATGATGACTTGCCTGCTCCCCACAATTGTTTCCCATCCAGAGACAGCAGGATG
GCAATGTGATAAATGTACAATATAATACGCTCCTTTGGAATATAAATTTTTTAAAGATCTTA
AGGCTGCAGTAGCGCAGTACGGTCCTCAGCTCCCTTTGTTTTGGCTATGCTGGAATCATTGG
GAAAAGGCAAATTAATCATTCCGTTAGATTGGGAATCTATTGCCCAAGCTGTCTTGGAGGGG
TCTCAATGGTTGCAACTTCGTAGCTGGTGGGAAGAAGCTAGAAAGCAGGCTCAGATTAATG
AGGGACAGAATCCCCCTGGTCCTCTTGAGGACAAGCTAATGGGAGAGGGCCAATATCGGGC
TTTAAGAGAACAGACTCAATACTCTGATCAGGACTTACAACAAGTCCGCCAGGTCTTTTTAC
GAGCATGGCGCCGTGTGGTGCCTACTGGCCACGCCCAGCCCTCCTTTGTTAAAACAATGCAA
GGCCCCAATGAGCCATATACTGATTTTCTAGCAAGATTGAGGGTAGCTGTGGAACGGGCTAT
AGGGAGGGATGAGATTTCAGAGATATTATTACAAACTTTAGCATTTGAAAATGCAAATCCTG
AATGCAAGCGTATACTGGGACCTTTAAAGGGACAGGGTGCATCTATAGCTGAATATATCAG
AGCCTGCTCAGGAGTAGGAGGAACTGAGCATCAGGCTAATGTCTTTGCTACAGCCTTGGCCA
AAGCTATGAGACCACAAAAGGGAGGTAACTGCTTCCATTGTGGAAAACCTGGTCATATGAG
AAGAGAATGTCAGAAATTAAAAGATGATCAAGGTGCAATTCCTAAAGACAGATATCTTGCT
GGGAAGAATAAGACTTCTCCTGGACTTTGCCGTCAGTGTGGGAAGGGGTTTCATTGGACTAA
TGAATGCAGATCTAAAACAGACAAAATAGGCAACCCGATACCGGGAAACTATCCTGCGGGC
CTAAGTCCTTGGGGCCCAGGAACAATACCGGGGACTTCTCCTCCTTGCCCTCTTCCCCATCCC
ATCTGCCCCAACCCTATTCCCTCCCAACAACCATTACGAGTCGATGCCCCATTAAAAGGACC
TCAGATGATGATTTCGGACTTAACGGTCTGCTACTTCAGGGAGTGCTGCTGCTGATTTGCCAC
TAGCTGATAATGTTCTTTTGTCACCAGGGGAAGGCATTTATAAATTAAAAACAAATGTATTT
GGACCACTGCCTAAAGGCACTTTTGACTTGATATTAGGCCATAGCAGCGCGGCTTTGAGAGG
TTTAACCATAATTCCTGGGGTAATAGACTCTGATTAAGTTGGGGAAATTTTAATTATGGTCTC
TACTTCTACCACACTTTCATTGTTAGCTGGGGAACGTATTGCTCAAATACTTCTCCTATCTTA
TCATCCCTTTTTGGCTCTTCCTAATGAATGAACAGGAGGATTTGGAAGTACTGGGCGACATA
TATTTTGGGAAATGCTTATCAAAGATTCCCGCCCTGTTCTCGCTTTGATTATACAGGGAAACA
ACTCTGAAGGACTAGTAGACACAGGGGCGGATGTTTCAGTCATTTCTTCTCAACAATGGCCC

CAAGATTGGGAAAAAGAAAAAAGCCCTTTAATGCTGACGGGATTGGGCTCCATTGCAGATG
TCTGGAAGAGTACCCATCCCTTGCGATGTCAATTCCATAATGGAAGATCAGTGTTTGTTACCT
TTTATACTGTAAATATACCTATTAATATATGGGGAAGAGATCTTCTCTCTCCTTTGGGGGCTT
CTGTAACCATTCCATTGGAAAACTAGTAGCCACTGCTCAAATTCCTCGAGCACTCCCATTAA
AATGGTTACCTAATACTCCAAAATGGGTTGAGCAGTGGCCATTACCACAAATGAAGCTCGAG
GCATTAGAACAATTAGTACAAGAACAACTCCAACTTGGTCATATAGAGCCCTCTACCTCACC
CTGGAATTCTCCTGTTTTGGTTATAAAAAAGAAATCTGGAAAATGGAGAATGTTAACTGATT
TACGAGAAGTTAATAAATGTATTGAACCTATGGGAGCATTACAATTGGGACTCCCCTCTCCA
GCTCTTATTCCTCAGAATTGGTCCTTAATGGTGTTAGATCTTAAAGACTGTCTTTTTTACCATT
CCCCTACAATTGCAAGATAGAGATAAATTTGCTTTTACAGTTCCTGTTCTTAATCATGCTCAG
CCTGTTAAGCGTTATCAATGGACAGTCTTACCACAAGGAATGATAAATTGTCCTACCTTATG
CCAAGAATTCGTAGCTCGCTCTTTACAATCCCTCCATCAAGAATACCCCAATTATAGTCTATA
TCATTATATGGATGATCTCCTCTTGGCAGCTCCTAGTATTGCTGAACGTGATGAATTCTTTTT
AAAAGTACAGGAGGCTTTAAGACTATACAATTTGCAAATAGCCCCAGAAAAAATTCAAAAG
GACTTTCCTATTTCATATTTAGGGACAATATTGGAACAACATAGAATTAGGCCCCAAAAGTT
GCAAATTAGAAGAGACCATCTCAAAACCTTAAATGATTTTCAAAAGTTATTGGGAGATATTA
ATTGGCTACGCCTGGTACTTGGGATTCCTACTTATCAATTATGACATTTGTTTTCTACTTTAG
AAGGAGATACAGCTCTGGATAGCCCCCGGACCTTAACCCCATTGGCTTTACGGGAACTTCAA
TTTGTTGAGCAATGACTAAATGATGGCTTTTTGACTTACTTACATGCATCTCAACCTATTTCT
TTTATAATATTTCATACCTCTTATTCCCCATCTGGTGTAATTGCTCAAGAAAAAGGATTAATA
GAATGGGTTTTCTTACCTAACAGTTTTTCCAAAAAATTGACTATATATATGGATAAATTAGCC
TTCCTTATACAGAAGGGTCACCATCGTATTTTACAATTATCAGGATGTGAACCACACCAGAT
TGTTACTCAGTTAACAACTGCTCAAATATCTCGATGTTTACAATTTAATGAAAACTGGAAATT
TTCTCTTGCCTCATATCCTGATTTGTTTTCTAATCATTATCCATCATCTAAATTGATTGATTTT
CTCTGGATTAACAGAGAAAATATATCTCATTCCCCAATTTCAGATGTTCCAGTTAAGGGACC
CACTATTTTTACAGGTGCAAATAAAAATACTGCTGGATATTGGACCCTGGAAAATTCCAAGG
TTCTCCCTCACTCATTTTCTTCTGTACAGCCCACTGAATTATGGGCTGTCTATTTAGTTTTGCA
AGATTTTCCCCAACTTCCTATTAACATTGTTTCAGATTCTCGATATGCTGTTCTCTCTTGCCTA
CAGCTTCTCCATGTCTCCCTTCCATTGACTCTTAAAACAGCTATTGATAAATTGTTTTACCAA
GTACAACAATTGCTCTTGCAGCGTTCAGAGTTAATTTTCTTTACTCACATCCGTGCACATTCT
GCCCTTCCTGGACCCTTATCATCCAGAAATGCTACAATTGATGCCTTACTTTATCCTATAGAA
GCAGCAAAACAAGAACATCTCTTACAACATACCAACTCCAAAGGGTTACAAAAATCTCATG
CTATTACTTGAAAACAAGCTCAAAATATTGTTCGTTCTTGTTCCATATGTGCACCCTTTGCTT
TGCCATTTACCCCACCAGGTGTCAACATGAGAGGACTACAAGCAAATCAGATATGGCAAAT
GGATGTAATTTACATTTCTTCCTTTGGACAACAAAAATGTGCGCATCATACTATAGATACTTG
CACACATTTTCAATGGGCCACTGCATTACATTCTGAAAAGGCTGACGCTGTTATTACTCATTT
GTTCTCTTGTTTTGCAGTTATGGGATTACCAATTGAATTGAAAACTGATAATGCACCTGCTTA
CCAATCTGCAAAATTAGCTCACTTTTTATCTCAATACCACATAACTCATACTTTTGGTATTCC
TTATAATAGTCAAGGGCAAGCTATCATTGAAAGAGCTAATCGTACCTTGCTTGATTATCTTG
AAAAAATAAAAAAGGGGGAACAAGAGAGATTTATGAAACCTAAAGACATTCTGAATAAAA
CCTTACTTACCCTAAATTTTTTGAATGTTTGGAGCAAGGGAAATCTATCAGCAGCAGAGTTG
CATTTTCAAGGGAAAGAAGAGGATAAGAAGATCTTGAATACGCCTATTTGGTATAAAGATA
AAGAGAAAGGTTGGATCCCAACATCATTAATATATTTGGGACGAGGGTATGCTTTCATTTCT
GTTGATAATTACAGGTTTTGGACCCCAGCAAGAGTGATCAAAATCAACAATGGCTGATCCCT
TTGTTCAAAAATTAGAAGAGCTTACTATACAGAGAAGCCTTACTTCTCGTACAAGGGAAGCA
ACACCTCCTACATGGGGTCAAATGAAGAGGTTGACGCAGGAAGCAGAGAAGACGTTAATGA
AGGCGGGGCAACCTCTGAATCCTACCAATCTTTTGCTTGCCACGATGGCGGTGGTGACATGT

CAGGTAATCGGTGTATCGGCAAGTAATCATACATATTGGGCACATATACCTAATCCCCCATT
AGTAAGAGCAGTTTTCTGGGGGGAACCAGAAGTGCAGGTATGTACTAATGAGATTGCCTTCT
TTCCCCCGCCAGCTTGCGGGGGAATAGAACAACTATCTCATCATAAACAACAATATAATATT
AGTAATTTGACCATTGCAGTGGAAGGTATTCCTTTATGTATGGGAGGACACCCCTTTTGTCTG
TCCACCAAGGAACATTCTCATCATTCTTATAATACATGGGGGGTAAAATATAATAGTTACCA
TTTTGCTGCTTTTACTGTGCTTGTTTCCACCAGGGGATTTAGCACCTCGACAGAACCGATAGA
CATTCATAATGGAAAACACATGTCACTATGTCCTGTTAACTTTTTTGTTCCTTCTCTAGAATC
TTTGGAGTGGGAACGTTGCCAAGGTCATCGACCCTTTAAAGTCATGAATTATTCTGGGGCCA
TCACTATAGATTGGAATCCAGATCATGGGCAATTCTGAGAAAAATGGTCAAATAAATCTCTT
AGGTGGCATCGTGCAAATAGCACTTTGATGGGCAATGGTAATGAAACAGTTAAATGGCAGC
AATTTGCACTTGTCCCTCCTCAATGACAATTGCAAGGATATCCGCACATTCAAGGAGATATT
TGGAAACTATGGGCGGTTTCTGGTAATCTCGCTATCTGGTCAGGAAACTATACTTTGGACAG
TGGTGACTCTTCCGGTCCATTCCATGTTAATTTACATGTTAATAAATCTTATTCTGCAATGGC
ATGTGTAAAATATCCTTTTGCATTGTTATATGGAAATTGGACCTAGAATGATACTGCGGGGT
CTGTGTCATGTGACTATTGTAATCTAACTCAATGTGTAAATCAGTCTTGGTGGGAAGAATTTG
AAAGATGAGCCTATAATTCCAATTTCTCGCTAGTAATTGTTAAGGCTCAGACAGAAGTATGG
TTACCTATAAATCTGACTCGGCCGTGGTCGGATTCTTTTGCTGTTTCTCACCTAATAACCGCT
GTACAGACTTTGCTATACCGATCTCAACGTATGCTTGGTGTGGTCAATGCTTCGATTCTAGCA
GTCGCGTCAGTAACTGCAACAGCAGCAGTGGCAGGTCTTGCATTACACCAAGGAATTCAAA
CAGCTGATTTTATTCAGGACTGGCATAAAGACTCATTTGTTATGGCAACAACAGCGAGATTT
GGATGCCCAACTTGCTACCGACACGCTCAATCTTCAACACACCGTTTCCTGGCTTGGAGATC
AACTGGCTGTTTTATCTACACAAAGTGTGTTGAAATGTGATTGGAATTCTTCTCAGTTTTGTA
TAACACCTGCACCATTTAACATGAGTGAAGGATGGGATAAAGTAAAACGACCCTTGACTGG
GCATCAAAATCTCACTACGGAGATTATGGACCTGGAACGACAAATTTTGTCTACTTTTAGCA
GGACTTTACCTGACATTATGGGGTCTGATTTGCTAAAATGTCTTCAAGAAGGAATGAATAAC
TTAAATCCATTAGGGCATGTATCCTCACTAATTGGGACTACTTTTGGGAACACTGTGTTTATA
TTACTTTTATGTCGTGTTGCTTTTCCAGTCTTCCGGCGATGGCGGAAAGGGAAACAACTAAA
GCACGAAGCAGAGAAGATCCAGACCATGTTACAATTTATAAAAGCAAATAAAAAAGGGGG
AGATGAAGGGTTAATAGGGTAGCAGAGATGTGCCTGCAAATGGGCCTCTCTGCTAGGGCTG
GACGTCCTTGCAAACGAGGCTTTCTGCCAAAGAGTCTGGACACAGCCTTGAGTTTAATGGTC
CCTTGCAAATGAGGGAGCATTCCCTTCTTATGATAAAGAAGGAATAGAGGGCTTTGTACAGA
CTCTGCAGTAGACTAGGATTTTACTCCCCTTTGCT

>NC_056066.1:34288887-34296816#SHEEP_RIP_21(-)

CTGCGGGGGACGACCCGTGAAGGGTTAAGTCTTGGGAGCTCCCTGGCAGGTATGCCAGGCC
CTAGGACACGTGCCTAAGCTCCCTGTCCCGCCACCCTCAAGAGTTTTTATAACCCTTAAGGCT
CCAAGATGTTTGGTTTCGGCAACATTTCATAGAAGATAGATTATCTTATTGTGTATATTTCAT
AGAAGATAGATATTCTGATTGTGTTCTGTATACAATGGTAAGGGTCTGGTGATTGTATCCTG
AGATTAAAAAACAACCTTGTGAGTGCCTTAAGTCACGTACTTTACCCTATATATACCGCAGC
ACAATAAAGCAAGGTATCAGCCATTTGGGGCTGATCCTCTCAACCCCATCTTTTGTCTATCTC
TTATTTTCTTAGCGGGGACGCTCCGTTCTCTCCCTGTGCAGGTGCGACTCTTGCTTGTGCTGG
CCGCGGCAGGTGGCGCCCAACGTGGGGCTCGAGCTCGACAGTTTTCCTCGCCACTACTCTTA
TTAATTGAAAAGAGTGAGTATATGAGTAAACAAGTGAATTAAATTGAGGAGGAGTAGTAAG
GTATATAGTTGAGAGTATAAATATGGGACAGACGCATAGTCGTCAGTTGTTTGTGCATATGT
TATCTGTAATGTTAAAACATAGGGGAATTACTGTTTCTAAACCTAAATTAATCAATTTTCTTT
CATTCATCGAGGAAGTTTGCCCTTGGTTCCCCAGAGAAGGTACAGTAAATTTAGAGACATGG
AAGAAGGTAGGGAACAAATTCGGACTCATTATACTTTACATGGCCCTGAAAAATCCCTGTCG

AAACTTTATCCTTTTGGACACTAATTCGTGACTGCCTGGACTTTGATAATGATGAATTAAAAC
GTTTAGGAAATTTATTAAAACAGGAAGAAGATCCTCTCCATGTTCCTGATTCGGAACCCAGA
TATGCTGTTCCCGAGGGGGTTAAAAGCGACCCTCCGTTTTCTAACTTATTGCATCCTTCAGAT
AATGATGATTTACTTTCATCCACAGATGAGGCAGAATTAGACGAAGAAGCTGCTAAATACCA
TCAAGAAGATTGGGGTTTTTAGCACAAGAAAAGGGGCGTTAACATCTAAAGATAAATTGGT
TGAATGCTTTAAAAACCTCACTATTGCTTTACAGAACGCAGGAATCAAGCTTCCTAGTAACA
ATGCCAAATCTCCTTCTGCTCCGCCTCTTCCCCCTGCTTATGCTCCTTCTGTTGTGGCTGGTCT
CGATCCCCTCCAGGGCCCCTCCACCGTCTGAGAACATGTCTCCGCTGCAAAAGGCATTGAGA
CAGGCACAGCGACTTGGTGAGGTTGTCTCTGATTTTTCTCTTGCTTTTCCTGTCTTTGAAAAT
AACAACCAGCGTTATTATGAATCACTGCCTTTTAAACAACTGAAAGAGTTAAAGATTGCTTG
CTCACAATACGGTCCTACCGCTCCATTCACCATTGCTATGATAGAAAATTTGGGTACTCAAG
CTTTACCTCCAAATGATTGGAAGCAGACAGCTAGGGCATGTCTCTCAGGGGGAGATTATTTA
TTATGGAAATCTGAATTTTTTGAACAATGTGCTCGTATAGCTGATGTTAACCGACAGCAAGG
TATACAGACCTCCTATGAAATGTTGATTGGTGAAGGCCCTTACCAGGCTACTGATACTCAAC
TTAATTTCTTACCTGGTGCATATGCACAAATATCAAATGCGGCTCGGCAGGCATGGAAAAAC
TTCCTAGCTCCAGTACTAAGACAGAGGATCTTTCAAAAGTCCGGCAGGGACCTGATGAGCCT
TACCAAGACTTCGTGACACGACTTTTAGATACTATAGGTAAGATAATGTCAGATGAAAAGGC
TGGGATGGTACTGGCAAAACAATTGGCTTTTAAAAACGCTAACTCTGCTTGTCAAGCTGCTT
TAAGACCTTATCGAAAAAAGGGAGATCTGTCTGATTTTATTCGCATTTGTGCTGACATTGGA
CCCTCCTACATGCAAGGCATTGCTATGGCAGCAGCATTACAAGGAAAAAGCATAAAAGAGG
TACTTTTCCAGCAGCAAGCCCGGAACAAGAAAGGACTTCAAAAGTCAGGTAATTTGGGTTGC
TTTGTTTGTGGTCAGCCTGGCCATCGGGCTGCAGTGTGCCCTCAAAAACAACAAAGCCCTGT
TAACACTCCTAATTTGTGCCCACGCTGTAAAAAAGGAAAGCATTGGGCGCGGGATTGCCGTT
CCAAAACGGATGTTCAAGGTAATCCTTGCCCCCCGGTTTCGGGAAACTGGGTGAGGGCCAGC
CCTGGCCCCGAAACAATGTTATGGGGCAACACTGCAGGTTCCAAAAGGACCATTGCAGACC
TCTGTCGAGCCACAAGAGGCAGCGCGGGATTGGACCTCTGTGCCACCTCCTACACAGTATTA
ACTCCCGAGATGGGGGTCCAAACCCTTGCCACAGGAGTGTTTGGGCCTTTACCTCCAGGGAC
AGCTGGACTGCTTTTAGGGCGCAGCAGTGCGTCTTTAAAAGGAATACTTATTCATCCTGGTG
TGATTGACTCTGATTATACAGGAGAGATAAAAATATTAGCCTCCGCTCCTAACAAAATTATT
GTAATCAATGCAGGACAGCGTATAGCTCAACTTCTTTTAGTTCCATTAGTCATACAAGGAAA
AACAATTAACCGAGACCGTCAAGATAAAGGTTTCGGGTCCTCTGACGCCTATTGGGTGCAAA
ATGTTACCGAGGCACGACCAGAACTTGAGCTACGCATTAATGGTAAGCTTTTCCGCGGAGTG
CTTGATACAGGGGCCGATATTAGTGTTATTTCTGATAAATATTGGCCTACTACATGGCCAAA
ACAGATGGCTATTTCCACTCTCCAGGGTATTGGCCAAACTACCAATCCAGAACAGAGTTCAT
CCCTTCTTACTTGAAAGGATAAAGATGGACATACAGGCCAATTTAAACCTTATATTCTGCCC
CATCTTCCAGTTAATCTATGGGGGCGTGATATATTAAGCAAAATGGGTGTTTATTTATATAGT
CCTTCACCCACTGTGACAGATTTGATGTTAGATCAGGGCTTACTTCCAAATCAAGGTTTAGGT
AAACAACATCAAGGCATCATTTTGCCCCTTGATTTAAAATCTAATCAAGATCGAAAAGGCTT
GGGGTGTTTTCCCTAGGGACCTCTGATTCTCCTGTGACGCATGCCGATCCTATTGATTGGAAA
TCTGAAAAACCGGTATGGGTCGATCAGTGGCCCCTAACACAGGAAAAACTTTCTGCCGCACA
ACAGCTGGTGCAAGAACAGCTGAGACTTGGGCATATTGAACCCTCTACCTCTGCTTGGAATT
CCCCAATTTTTGTTATTAAAAAGAAGTCTGGGAAATGGAGATTGCTACAAGATCTTCGTAAG
GTAAATGAAACAATGATGCATATGGGAGCCCTACAACCTGGGTTGCCCACTCCTTCTGCTAT
ACCTGATAAATCCTATATCATTGTTATAGATTTAAAAGATTGTTTTTACACTATTCCTCTTGC
ACCTCAAGATTGCAAAAGATTTGCTTTCAGTTTACCCTCTGTTAATTTTAAAGAGCCTATGCA
ACGCTATCAATGGAGAGTTCTCCCGCAAGGAATGACTAATAGCCCTACGCTGTGCCAAAAAT
TTGTTGCTACAGCAATAGCTCCGGTTCGTCAACGTTTTCCTCAGCTATATTTGGTTCATTATA

TGGATGATATATTACTAGCTCATGCTGACGAACATCTATTGTATCAAGCTTTTTCTATTCTAA
AACAACATTTAAGCCTTAATGGTCTTGTTATTGCTGATGAAAAATTCAGACTCATTTTCCTTA
TAATTATTTGGGTTTCTCCTTATATCCTCGTGTTTATAATACCCAATTAGTAAAACTGCAGAC
TGACCATTTAAAAACTCTAAATGACTTTCAAAAACTTTTAGGAGACATTAATTGGATACGTC
CTTATTTAAAATTACCCACTTATACCTTGCAGCCATTATTTGACATCCTTAAAGGTGACTCTG
ATCCTGCGTCACCCCGAACACTTTCTTTAGAAGGACGAACTGCTTTACAATCAATAGAAGAA
GCTATTAGACAACAACAGATTACTTATTGTGATTACCAACGATCATGGGGTTTGTATATACTT
CCTACCCCCCGAGCACCCACAGGGGTTCTCTATCAAGATAAACCTTTGCGATGGATATATTT
GTCTGCTACTCCAACTAAACATCTGCTCCCTTACTATGAACTTGTTGCAAAAATTATAGCAAA
GGGACGTCACGAGGCCATCCAATATTTGGTATGGAACCCCCTTCATTTGTGTTCCTTATGCT
TTAGAACAACAAGATTGGCTTTTTCAATTTTCAGATAATTGGTCTATAGCTTTTGCAAATTAC
CCCGGACGGATTACTCATCATTACCCTTCTGATAAATTGTTACAATTTGCTAGCTCTCATGCC
TTTATTTTTCCAAAAATAGTTCGCCGACAACCTATTCCCGAAGCGACACTTATATTTACAGAT
GGATCTTCTAATGGAACTGCAGCTTTAATCATTAACCATCAAACCTATTACGCACAAACCAG
TTTTTCTTCTGCTCAAGTTGTGGAATTATTTGCAGTCCACCAAGCGTTGCTAACTGTACCTAC
TTCCTTCAATTTATTTACAGACAGCTCCTATGTGGTCGGTGCCTTACAGATGATTGAAACTGT
TCCAATTATCGGCACCACCTCTCCTGAAGTTCTTAACTTATTTACATTGATTCAACAGGTTCT
CCATTGCCGCCAACACCCCTGTTTCTTTGGACATATTCGTGCACACTCCACCCTTCCTGGTGC
CCTCGTACAAGGCAATCACACTGCGGACGTTCTTACTAAACAAGTGTTTTTTCAATCAGCTAT
TGATGCAGCCCGAAAGTCCCATGATTTACATCACCAAAATAGTCATTCTTTACGCTTGCAATT
TAAAATTTCCCGTGAAGCTGCACGGCAAATTGTTAAATCTTGCTCTACTTGTCCTCAATTCTT
TGTTCTTCCTCAATATGGTGTCAACCCTCGAGGTTTACGCCCTAATCACCTCTGGCAAACAGA
CGTTACTCACATTCCTCAATTTGGGCGTCTTAAATATGTTCATGTTTCTATTGACACTTTTTCC
AATTTTCTCATGGCCTCCCTTCACACTGGAAAATCAACACGTCACTGTATTCAACATTTGCTG
TTTTGCTTTTCTACTTCAGGAATCCCACAAACCCTTAAAACAGATAATAGACCTGGTTATACT
AGCCGTTCTTTTCAACGTTTTTGCCTTTCTTTCCAAATTCATCATAAAACAAGAATTCCTTATA
ATCCACAGGGACAAGATATTATAGAACGAGCCCATCAACGCCTTAAACATCAATTATTAAA
ACAAAAAAAGGGGAATGAACTGTATAGCCCCTCACCGCATAACGCCTTAAACCATGCTCTTT
ATGTTTTAAATTTTTTAACTTTAGACGCAGAAGGCAATTCAGCAGCCCAGCGTTTTTGGGGA
GAACGATCCTCATGCAAAAAACCACTTGTACGATGGAAGGATCCACTTACCAATCTGTGGTA
TGGGCCAGACCCTGTACTAATATGGGGACGAGGGCATGTTTGTGTTTTTCCACAGGATGCCG
AAGCGCCGCGCTGGATTCCGGAAAGGCTGGTACGCGCGGCAGAGGAACTCCCTGACACATC
AAATGCATCGCATGACACTGAGTGAGCCCACGAGTGAGCTGCCTACCCAGAGGCAAATTGA
GGCGCTGATGCGTTATGCTTGGAATGAGGCTCATGTACAACCTCCAGTGACACCTACTAAAA
TACTGATCATGTTATTATTATTGTTACAGCGGATACAAAACGGGGCAGCTTCGGCTTTTTGGG
CATACATTCCTGATCCGCCTATGATTCAATCCTTAGGATGGGATAAAGAAACAGTACCTGTA
TATGTTAATGATACAAGTCTTTTAGGAGGAAAATCAGATATTCACATTTCTCCTCAGCAAGC
CAATATCTCCTTTTATGGTCTTACTACTCAATACCCTATGTGCTTTTCTTATCAATCACAGCAT
CCTCATTGTATACAGGTGTCAGCTGATATATCCTATCCTCGAGTGACTATTTCAGGCATTGAT
GAAAAAACCGGAAAGAGATCGTACCGTGACGGAACCGGACCCCTCGACATTCCGTTTTGTG
ACAAAAATTTAAGCATCGGCATAGGAATAGACACTCCTTGGACTTTATGTCGAGCACGAATT
GCATCGGTGTATAACATCAACAATGCCAATACCACCCTTTTATGGGACTGGGCACCTGGAGG
AACACCTGATTTCCCCGAATATCGAGGACAGCATCCACCCATTCTCTCTGTAAACACTGCTC
CTATATTTCAAACTGAATTGTGGAAACTTTTGGCTGCTTTTGGTCATGGCAATAGTCTATATT
TACAGCCCAATATTAGTGGGAGCAAATATGGTGATGTGGGAGTTACAGGATTTTTATATCCC
CGAGCTTGTGTCCCTTATCCATTCATGTTGATACAAGGCCATATGGAAATAACACTGTCATTG
AATATTTATCATTTAAATTGTTCTAATTGCATACTTACTAATTGCATTAGAGGTGTAGCCAAA

GGAGAACAAGTTATAATAGTAAAACAACCTGCTTTTGTAATGTTACCTGTTGAAATAACTGA
AGAATGGTATGATGAAACTGCTTTAGAATTGTTACAACGCATTAATACGGCTCTTAGCCGTC
CTAAAAGAGGTCTGAGCCTGATTATTCTGGGTATAGTGTCTTTAATCACCCTTATAGCAACTG
CTGTTACTGCTTCTGTATCTTTAGCACAATCCATTCAAGCTGCTCATACTGTAGATTCCTTGTC
ATATAATGTTACTAAAGTAATGGGAACTCAAGAAGATATAGATAAAAAAATAGAAGATAGA
TTATCAGCTTTATATGATGTAGTTAGAGTTCTAGGAGAACAAGTTCAGAGCATTAATTTTCGC
ATGAAAATTCAATGCCATGCTAATTATAAATGGATTTGTGTTACAAAAAGCCTTACAATACT
TCTGACTTTCCGTGGGATAAGGTGAAAAAACATCTACAAGGAATTTGGTTTAATACTAATGT
TTCTTTAGATCTTTTACAATTGCACAATGAAATTCTTGACATCGAAAATTCTCCAAAAGCTAC
TTTGAATATAGCTGATACCGTCGATAATTTTTTACAAAATTTATTTTCTAACTTTCCTAGCCTT
CATTCACTGTGGCGAAGTATAATTGCTATGGGCGCGGTTCTGACTGTTGTGCTTATCATAATT
TGTTTAGCTCCTTGCCTTATTCGTAGCATTGTTAAAGAATTTCTACATATGAGAGTTTTAATA
CATAAAAACATGTTGCAACACCAACATCTTATGGAGCTTTTAAAAATAAAGAGAGGGGAGC
TGCGGGGGACGACCCGTGAAGGGTTAAGTCTTGGGAGCTCCCTGGCAGGTATGCCAGGCCC
TAGGACACGTGCCTAAGCTCCCTGTCCCGCCACCCTCAAGAGTTTTTATAACCCTTAAGGCTC
CAAGATGTTTGGTTTCGGCAACATTTCATAGAAGATAGATTATCTTATTGTGTATATTTCATA
GAAGATAGATATTCTGATTGTGTTCTGTATACAATGGTAAGGGTCTGGTGATTGTATCCTGA
GATTAAAAAACAACCTTGTGAGTGCCTTAAGTCACGTACTTTACCCTATATATACCGCAGCA
CAATAAAGCAAGGTATCAGCCATTTGGGGCTGATCCTCTCAACCCCATCTTTTGTCTATCTCT
TATTTTCTTAGCGGGGACGCTCCGTTCTCTCCCTGTGCAGGTGCGACTCTTGCTTGTGCTGGC
CGCGGCA

>NC_056067.1:15049560-15057133#SHEEP_RIP_22(+)

TGCGGGGGACGACCCGTGAAGGGTTAAGTCTTGGGAGCTGCTCAGCAGGTATGCAGAGCCC
TAGGACATGTTCCTAAGCTCCCTGTCCCGCCACCCTCAAGAATTTTTATAGCCCTTAAGGCTC
CAAGATGTTTGGTTTCGGCAACATTTCATAGAAGATAGATTATCTTATTGTGTATACTTCATA
GAAGATAGATTATCTGATTGTGTTCTGTATACAATGGTAAGGGTCTAGTGATTGTATCTTGA
GATTAAAAACAACCTTGTGAATGTCATAAGTCACGTACTTTACCCTATATATACTGCAGCAC
AATAAAGCAAGGTATCAGCCATTTTGGGCTGATCCTCTCAACCCCATCTTTTGTCTCTCTCTT
ATTTTTCTTAGCGGGGACGCTCCGTTCTCTCCCTGTGCAGGTGCGACTCTTGCTTGTGCTGGC
CACGGCAGGTGGCGCCCAACGTGGGGCTCGACTTCGACAGTTTTCCTCGCCACTACTCTTAT
TAATTGAAAAGAGTGAGTATATGAGTATACAAGTGAATTAAATTGAGGAGGAGTAGTAAGG
TATATAGTTGAGAGTATAAATATGGGACAGATGCATAGTCGTCAATTGTTTGTACATATGTT
ATCTGTAATGTTAAAACATCGGGGAATTACTGTTTCCAAACCTAAATTAATCAATTTTCTTTC
ATTTATTGAGGAAGTTTGCCCTTGGTTCCCCAGAGAAGGTACAGTAAATTTAGAAACATGGA
AGAAGGTAGGGGAACAAATTCGGACTCATTATACTTTACATGGCCCTGAAAAAATCCCTGTC
GAAACTTTATCCTTTTGGACACTAATTCGTGATTGCCTGGACTTTGATAATGATGAATTAAAA
CGTTTAGGAAATTTATTAAAACAGGAAGAAGATCCTCTCCATGTTCCTGATTCGGAACCCAG
GTATGCTGTTCCCGAGGGAGTTGAAGGCGACCCTCCGTTTTCTAACTTATTGCGTCCTTCGGA
TAATGATGATTTACTTTCATCCACAGATGAGGCGGAATTAGATGAAGAAGCTGCTAAATACC
ATCAAGAAGATTGGGGTTTTTTAGCACAAGAAAAGGGGGCGTCAACATCTAAAGATGAATT
GGTTGAATGTTTAAAAAACCTCACTATTGCTTTACAGAACTCAGGAATCAAGCTTCCTAGTA
ACAATGCTAAATCTCCTTCTGCTCCGCCTCTTCCCCCTGCCTATGCTCCTTCCGTTGTGGCTGG
TCTCGATCCCCCTCCAGGGCCTCCTCCACCGTCTGAGATCATGTCTCCGCTGCAGAGGGCATT
GAGACAGGCACAGCGACTTGGTGAGGTTGTCTCTGATTTTTCTCTTGCTTTCCCTGTCTTTGA
AAATAACAACCAGCGTTTTTATGAATCACTGCCTTTTAAACAACTGAAAGAGTTAAAGATTG
CTTGCTCGCAATACGGTCCTACCGCTCCATTCACTATTGCTATGATAGAAAGTTTGGGTACTC

AAAATCTACCCCCAAATGATTGGAAACAAATAGCTAGGGCCTGTCTTTCGGGGGGAGATTAT
TTACTATGGAAATCTGAATATTTTGAACAGTGTGCTCGTATAGCCAATGTTAATCGACAGCA
AGGTATACAGACCTCCTATGAAATGTTGATTGGTGAAGGCCCTTACCAGGCTACCGATACTC
AACTTAACTTCTTACCTGGTGCGTACGCACAAATATCAAATGCGGCTCGGCAGGCATGGAAA
AAACTTCCTAGCTCCAGTACTAAGACAGAAGACCTTTCAAAAGTCCGACAGGGACCTGATG
AGCCTTATCAAGACTTCGTGGCACGGCTCTTAGATACTATAGGTAAGATAATGTCAGATGAA
AAGGCTGGGATGGTATTAGCAAAACAATTGGCTTTTGAAAACGCTAACTCTGCCTGTCAAGC
TGCTTTAAGACCTTATCGAAAAAAGGGAGATCTGTCTGATTTTATTCGTATTTGTGCTGACAT
TGGACCCTCCTATATGCAAGGCATTGCTATGGCAGCAGCATTACAAGGAAAAGGCATTAAA
GAGGTACTTTTTCAGCAGCAAGCCAGGAACAAGAAAGGACTTCAAAAGTCAGGTAATTCGG
GTTGCTTTGTTTGTGGTCAACCTGGCCATCGGGCAGCAGTGTGCCCCCAAAAGCAACAAACC
TCTGTTAACACTCCTAATTTATGCCCACGATGTAAAAAAGGGAAGCATTGGGCCCAAGATTG
TCGTTCTAAAACGGATGTTCAAGGTAATCCTTTGCCCCCGGTTTCGGGAAACTGGGTGAGGG
GCCAGCCCCTGGCCCCAAAACAATGTTATGGGGCAACACTGCAGGTTCCAAAAGAACCACT
GCAGACCTCTGTCGAGCCACAAGAGGCAGCGCGGGATTGGACCTCTGTGCCACCTCCTACAC
AGTATTAACACCCGAGATGGGGGTTCAAACCCTTGCCACAGGAGTGTTTGGGCCTTTACCTC
CAGGTACAGCTGGACTGCTTTTGGGGCGCAGCAGCGCGTCTTTAAAGGGAATACTTATCCAT
CCTGGTGTGATTGACTCTGATTATACAGGAGAGATAAAAATATTAGCCTCCGCTCCTAACAA
AATTATTGTAATCAATGCAGGACAACATATAGCTCAACTCCTTTTAGTTCCATTAGTCATACA
AGGAAAAACAATTAACCGAGACCGTCAAGATAAAGGTTTCGGGTCCTCTGACGCCTATTGG
GTGCAAAATGTTACCGAGGCACGACCAGAACTTGAGCTACGCATTAATGGTAAGCTTTTCCG
CGGAGTGCTTGATACAGGGGCCGATATTAGCGTTATTTCTGAAAAATACTGGCCTACTACAT
GGCCTAAACAAACAGCTATTTCCACTCTTCAGGGTATTGGCCAAACTACCAATCCAGAACAA
AGTTCGTCCCTTCTTACTTGGAGGGATAAAGATGGCCATACAGGCCAATTTAAACCTTATAT
TCTGCCCCATCTTCCAGTTAATCTATGGGGGCGTGATATATTAAGCAAAATGGGTGTTTATTT
ATATAGTCCTTCACCCACCGTAACAGATTTGATGTTAGATCAGGGCTTACTTCCAAACCAAG
GTTTAGGTAAACAACATCAAGGCATCGTTTTACCCCTTGATTTAAAATCTAATCAAGATCAA
AAAGGCTTGGGGTGTTTTTCCTAGGGACCTCTGATTCTCCTGTGACACATACCGATCCTATTG
ATTGGAAATCTGAGGAACCGGTATGGGTCGATCAGTGGCTCCTGACACAAGAAAAACTTTCT
GCCGCACAACAGCTGGTGCAGGAACAGCTGAGGCTTGGGCATATTGAACCCTCTACCTCTGC
GTGGAATTCCCCAATTTTTGTTATTAAAAAGAAGTCTGGAAAATGGAGATTGCTACAAGACC
TTCGTAAGGTAAATGAAACAATGATGCATATGGGAGCCCTACAACCTGGGTCGCCCACTCCT
TCCGCTATACCTGATAAATCCTATATTATCATTATAGATTTAAAAGATTGTTTTTACACTATT
CCTCTTGCACCTCAAGATTGTAAAAGATTTGCCTTTAGTTTGCCCTCTGTTAATTTTAAAGAG
CCTATGCAACGCTATCAGTGGAGAGTCCTCCCACAAGGAATGACTAATAGTCCTACGTTATG
TCAAAAATTTGTTGCTACAGCATTAGCTCCCGTTCGTCAGCGTTTTCCTCAGTTATATTTAGT
TCATTATATGGATGATGTATTACTAGCTCATGCTGACGAACATCTATTGTATCAAGCTTTTTC
TATTCTAAAAAATCATTTAAGCCTTAATGGTCTTGTCATTGCTGATGAAAAAATTCAAACTCA
CTTTCCCTATAATTATTTGGGTTTCTCCTTATACCCTCGTGTTTATAACACCCAATTGGTACAA
TTACAGACTGACCATTTAAAAACTCTAAATGACTTTCAAAAACTTCTAGGAGACATTAATTG
GATACGCCCTTATTTAAAACTACCCACTTATACCTTGCAGCCATTATCTGACATCCTTAAAGG
TGACCCTGACCCTGCGTCACCCCGAACACTTTCTCTAGAAGGACGATCAGCCTTACAATCAA
TAGAAGAAGCTATTAGACAACAACAGATTACTTATTGTGATTACCAACGATCATGGGGTTTG
TATATACTTCCTACCCCTCGAGCACCCACAGGGGTTCTTTATCAAGATAAACCTTTGCGATGG
ATATATCTATCTGCTACTCCAACTAAACATCTGCTCCCTTACTATGAGCTTGTTGCAAAAATT
GTAGCAAAAGGACGTCATGAGGCCATCCAATATTTTGGTATGGAACCCCCTTTCATTTGTGT
TCCTTATGCTTTAGAACAACAAGATTGGCTTTTTCAATTTTCAGATAATTGGTCCATAGCTTT

CGCAAATTACCTGGGACGGATTACTCATCATTATCCTTCTGATAAATTGTTACAATTTGCTAG
CTCTCATGCCTTTATTTTTCCAAAAATAGTTCGCTGACAACCCATTCCCGAAGCGACACTTAT
ATTTACAGATGGATCTTCTAATGGAACTGCAGCTTTAATTATTAACCATCAAACCTATTACGC
ACATACCAGTTTTTCTTCTGCTCAGGTTGTTGAATTATTTGCAGTCCATCAAGCATTGCTAAC
TGTACCCACTTCCTTCAATTTATTTACAGACAGCTCCTATGTGGTCGGTGCCTTACAGATGCT
TGAAACTGTTCCAATTATCGGCACAACCTCTCCTGAAGTTCTTAACTTATTTACATTGATTCA
ACAGGTTCTTCACCGTCGCCAACACCCGTGTTTCTTTGGGCATATTCGTGCACATTCCACCCT
TCCTGGTGCCCTCGTACAAGGCAATCACACTGCGGACGTTCTTACTAATCGAGTGTTTTTTCA
ATCAGTTATCGATGCAGCCCGAAAATCTCATAACTTACATCACCAAAATAGTCATTCTTTAC
GGTTACAATTTAAGATTTCCCATGAAGCTGCACGGCAAATTGTTAAATCTTGCTCTACTTGTC
CTCAATTCTTTGTTCTTCCTCAATATGGTGTCAACCCTCGAGGTTTACGCCCTAATCATCTCTG
GCAAACAGATGTCACTCACATTCCCCAATTTGGGCGTCTTAAATATGTTCATGTCTCTATCGA
CACCTTTTCCCATTTTCTCATGGCCTCCCTTCACACCGGAGAATCAACTCGTCACTGTATTCA
ACATTTGCTGTTTTGCTTTTCTATTTCAAGAATCCCACACACCCTTAAAACAGATAATGGACC
TGGTTATACTAGCCGTTCTTTTCAACGTTTTTGTCTTTCTTTTCAAATTCATCATAAAACAGGA
ATTCCTTATAATCCACAAGGACAAGGTATTGTGGAACGAGCCCATCAACGCCTTAAACATCA
ATTATTAAAACAAAAAAAGGGGAATGAACTGTATAGCCCCTCACCGCGTAACGCCTTAAAT
CATGCTCTCTATGTTTTAAATTTTTTAACTTTAGATGCAGAAGGCAATTCAGCAGCCCAGCGT
TTTTGGGGAGAATGATCCTCATGCAAAAAACCACTTGTACGATGGAAGGATCCACTTACCAA
TCTGTGGTATGGGCCAGACCCTGTATTAATATGGGGACGGGGGCATGTTTGTGTTTTTCCAC
AGGATGCCGAAGCGCCGCGCTGGATTCCGGAAAGGCTGGTACGCGCGGCGGAGGAACTCCC
TGACATATCAAATGCATCGCATGACACTGAGCGAGCCCACGAGTGAGCTGCCTACCCAGAG
GCAGATTGAGGCGTTGATGCGACATGCTTGGAATGAGGCTCATGTACAACCTCCGGTGACGC
CTACAAATATACTGATCATGTTATTATTATTGTTACAGCGGATACAAAATGGGGCGGCTGCG
GCTTTTTGGGCATACATTCCCGATCCGCATATGATTCAAAGAAATAGTACCTGTATATGTCA
ATGATACAAGTCTTTTAGGAGGAAAATCAGATATTCACATTTCTCCTCAGCAAGCCAATATC
TCCTTTTATGGTCTTACTACGCAATATCCTATGTGCTTTTCTTATCAATCACAGCATCCTCACT
GTATACAGGTGTCAGCTGATACATCCTACCCTCGAGTGACTATTTCTGGCATTGATGAAAAA
ACCGGAAAAAGATCTTACCGTGACGGAGCCGGACCCCTCGACATTCCGTTTTGCGACAAACA
TTTAAGCATCGGCATAGGAATAGATACTCCTTGGACTTTATGTCGAGCACGGGTCGCATCGG
TGTACAACATCAACAATGCCAATGCCACCCTTTTATGGGACTGGGCACCTGGGGGAACACCT
GATTTCCCCGAATATCGAGGACAGCATCCACCCATTCTCTCTGTAAACACTGCTCCTATATAT
CAGACAGAACTGTGGAAACTTTTGGCTGCTTTTGGTCATGGTAATAGCCTATATTTACAACC
CAATATTAGTGGGAGTAAATATAGTAATGTAGGAGTTACGGGGTTTTTATATCCCCGAGCTT
GTGTCCCTTACCCATTCATGTTGATACAAGGCCATGTGGAAATAACGCTGTCATTGAATATTT
ATCATTTAAATTGTTCTAATTACATACTTACTAATTGCATTAGAGGTGTTGCCAAAGGAGAA
CAAGTTATAATAGTAAAACATTCGCATGAAAATTCAATGCCATGCTAATTATAAATGGATTT
GTGTTACAAAAAAGCCTTACAATACATCTGATTTTCCGTGGGATAAGGTGAAAAAACATCTA
CAAGGAATTTGGTTTAATACTAATGTTTCTCTAGATCTTTTACAATTGCATAATGAAATTCTT
AACATTGAAAATTCTCCAAAAGCTACTTTGAATATAGCTGATACTGTCGATAATTTTTTACAA
AATTTATTTTCTAACTTTCCTAGCCTTCATTCACTGTGGCGAAGTATAATTGCTGTGGGCGCG
GTTCTGACTGTTGTGCTTATCATAATTTGTCTAGCTCCTTGTCTTATTCGCAGTATTGTTAAGG
AATTTCTACATATGAGAGTTTTAATACATAAAAACATGTTGCAACACCAACATCTTATGGAG
CTTTTAAAAAATAAAGAGAGGGGAGCTGCGGGGGACGACCCGTGAAGGGTTAAGTCTTGGG
AGCTGCTCAGCAGGTATGCAGAGCCCTAGGACATGTTCCTAAGCTCCCTGTCCCGCCACCCT
CAAGAATTTTTATAGCCCTTAAGGCTCCAAGATGTTTGGTTTCGGCAACATTTCATAGAAGA
TAGATTATCTTATTATGTATACTTCATAGAAGATAGATTATCTGATTGTGTTCTGTATACAAT

GGTAAGGGTCTAGTGATTGTATCTTGAGATTAAAAACAACCTTGTGAATGTCATAAGTCACG
TACTTTACCCTATATATACTGCAGCACAATAAAGCAAGGTATCAGCCATTTTGGTCTGATCCT
CTCAACCCCATCTTTTGTCTCTCTCTTATTTTTCTTAGCGGGGACGCTCCGTTCTCTCCCTGTG
CAGGTGCGACTCTTGCTTGTGCTGGC

>NC_056067.1:57031180-57039117#SHEEP_RIP_23(-)

TGCGGGGGACGACCCGTGAAGGGTTAAGTCTTGGGAGCTCCCTGGCAGGTATGCCGGGCCC
TAGGACATGTGCCTAAGCTCCCTGTCCCGCCACCCTCAAGAATTTTTGTAACCCTTAAGGCTC
CAAGATGTTTGGTTTCGGCAACATTTCATAGAAGATAGATTATCTTATTGTGTATACTTCATA
GAAGATAGATATTCTGATTGTGTTCTATATACAATGGTAAGGGTCTGGTGATTGTATCCTGA
GATTAAAAAACAACCTTGTGAGTGCCTTAAGTTACGTACTTTACCCTATATATACCGCAGCA
CAATAAAGCAAGGTATCAGCTATTTTGGTCTGATCCTCTCAACCCCATCTTTTGTCTCTCTCT
TATTTTCTTAGCGGGGACGCTCCGTTCTCTCCCTGTGCAGGTGCGACTCTTGCTTGTGCTGGC
CGCGGCAGGTGGCGCCCAACGTGGGGCTCGAGCTCGACAGTTCTCCTCGCCACTACTCTTAT
TAATTGAAAAGAGTGAGTATATGAGTAAACAAGTGAATTAATTTGAGGAGGAGTAGTAAGG
TATATAGTTGAGAGTATAAATATGGGACAGACGCATAGTCGTCAGTTGTTTGTGCATATGTT
ATCTGTAATGTTAAAACATAGGGAATTACTGTTTCTAAACCTAAATTAATCAATTTTCTTTCA
TTCATCGAGGAAGTTTGCCCTTGGTTCCCCAGAGAAGGTACAGTAAATTTGGAGACATGGAA
GAAGGTAGGGGAACAAATTCGGACTCATTATACTTTACATGGCCCTGAAAAAATCCCTGTCG
AAACTTTATCCTTTTGGACACTAATTCGTGACTGCCTGGACTTTGATAATGATGAATTAAAAC
GTTTAGGAAATTTATTAAAACAGGAAGAAGATCCTCTCCATGTTCCTGATTCGGAACCCAGG
TATGCTGTTCCCGAGGGGGTTAAAAGCGACCCTCCGTTTTCTAACTTATTGCGTCCTTCGGAT
AATGATGATTTACTTTCATCCACAGATGAGGCAGAATTAGACGAAGAAGCTGCTAAATACCA
TCAAGAAGATTGGGGTTTTTTAGCACAAGAAAAGGGGCGTTAACATCTAGAGATGAATTGG
TTGAATGTTTTAAAAACCTCACTATTGCTTTACAGAACGCAGGAATCAAGCTTCCTAGTAAC
AATGCCAAATCTCCTTCTGCTCCGCCTCTTCCCCCTGCTTATGCTCCTTCTGTTGTGGCTGGTC
TCGATCCCCCTCCAGGGCCCCCTCCACCGTCTGAGAACATGTCTCCGCTGCAAAAGCATTGA
GACAGGCACAGCGACTTGGTGAGGTTGTCTCTGATTTTTCTCTTGCTTTTCCTGTCTTTGAAA
ATAACAACCAGCGTTATTATGAATCACTGCCTTTTAAACAACTGAAAGAGTTAAAGATTGCT
TGCTCACAATACGGTCCTACCGCTCCATTCACCATTGCTATGATAGAAAATTTGGGTACTCA
AGCTTTACCTCCAAATGATTGGAAGCAGACAGCTAGGGCATGTCTCTCAGGGGGAGATTATT
TATTATGGAAATCTGAATTTTTTGAACAATGTGCTCGTATAGCTGATGTTAACCGACAGCAA
GGTATACAGACCTCCTATGAAATGTTGATTGGTGAAGGCCCTTATCAGGCTACTGATACTCA
ACTTAATTTCTTACCTGGTGCATATGCACAAATATCAAATGCGGCTCGGCAGGCATGGAAAA
AACTTCCTAGCTCCAGTACTAAGACAGAGGATCTTTCAAAAGTCCGGCAGGGACCTGATGAG
CCTTACCAGGACTTCGTGGCACGACTCTTAGATACTATAGGTAAGATAATGTCAGATGAACA
GGCTGGGATGTTATTGGCAAAACAATTGGCTTTTGAAAACGCTAACTCTGCTTGTCAAGCTG
CTTTAAGACCTTATCGAAAAAAAGGAGATCTGTCTGATTTTATTCGCATTTGTGCTGACATCG
GACCCTCCTACATGCAAGGCATTGCTATGGCAGCAGCATTACAAGGAAAAAGCATAAAGGA
GGTACTTTTCCAGCAGCAAGCCCGGAACAAGAAAGGACTTCAAAAGTCAGGTAATTTGGGT
TGCTTTGTTTGTGGTCAGCCTGGCCATCGGGCTGCAGTGTGCCCTCAAAAACAACAAAGCCC
TGTTAACACTCCTAATTTGTGCCCACGCTGTAAAAAAGGAAAGCATTGGGCACGGGATTGCC
GTTCCAAAACGGATGTTCAAGGTAATCCTTTGCCCCCGGTTTCGGGAAACTGGGTGAGGGCC
AGCCCTGGCCCCGAAACAATGTTATGGGGCAACACTGCAGGTTCCAAAAGGACCATTGCAG
ACCTCTGTCGAGCCACAAGAGGCAGCGTGGGATTGGACCTCTGTGCCACCTCCTACACAGTA
TTAACTCCCGAGATGGGGGTCCAAACCCTTGCCACAGGAGTGTTTGGGCCTTTACCTCCAGG
GACAGCTGGACTGCTTTTAGGGCGCAGCAGTGCGTCTTTAAAAGGAATACTTATTCATCCTG

GTGTGATTGACTCTGATTATACAGGAGAGATAAAAATATTAGCCTCCGCTCCTAACAAAATT
ATTGTGATCAATGCAGGACAGCGTATAGCTCAACTTCTTTTAGTTCCATTAGTCATACAAGG
AAAAACAATTAACCGAGACCGTCAAGATAAAGGTTTCGGGTCCTCTGACGCCTTTTGGGTGC
AAAATGTTACCGAGGCACGACCAGAACTTGAGCTACGCATTAATGGTAAGCTTTTCCGCGGA
GTGCTTGATACAGGGGCCGATATTAGTGTTATTTCTGATAAATATTGGCCTACTACATGGCC
AAAACAGATGGCTATTTCCACTCTCCAGGGTATTGGCCAAACTACCAATCCAGAACAAAGTT
CGTCCCTTCTTACTTGGACGGATAAGGACGGTCATACAGGCCAATTTAAACCTTATATTCTGC
CCTATCTTCCAGTTAATCTATGGGGGCGTGATATATTGAGCACAATGGGTGTTTATTTATATA
GTCCTTCACCCACTGTGACAGATTTGATATTAGATCAGGGCTTACTTCCAAATCAAGGTTTAG
GTAAACAACATCAAGGCATCATTTTGCCCCTTGATTTAAAACCTAATCAAAACCGAAAAGGC
TTGGGGTGTTTTCCCTAGGGACCTCTGATTCTCCCGTGACACATGCCGATCCTATTGATTGGA
AATCTGAGGAACCGGTATGGGTCGATCAGTGGCCCCTAACACAGGAAAAACTTTCTGCCGC
ACAACAGCTGGTGCAGGAACAGCTGAGACTTGGGCATATTGAACCCTCTACCTCTGCTTGGA
ATTCCCCAATTTTTGTTATTAAAAAGAAGTCTGGGAAATGGAGATTGCTACAAGATCTTCGT
AAGGTAAATGAAACAATGATGCATATGGGAGCCCTACAACCTGGGTTGCCCACTCCTTCTGC
TATACCTGATAAATCCTATATCATTGTCATAGATTTAAAAGATTGTTTTTACACTATTCCTCTT
GCACCTCAAGATTGCAAAAGATTTGCTTTCAGTTTACCCTCTGTTAATTTTAAAGAGCCTATG
CAACGCTATCAATGGAGAGTTCTCCCGCAAGGAATGACTAATAGCCCTACGCTGTGCCAAAA
ATTTGTTGCTACAGCAATAGCTCCCGTTCGTCAACGTTTTCCTCAGCTATATTTGGTTCATTAT
ATGGATGATATATTACTAGCTCATGCTGACGAACATCTATTGTATCAAGCTTTTCGATTTTAA
AACAACATTTAAGCCTTAATGGTCTTGTTATTGCTGATGAAAAATTCAGACTCATTTTCCTTA
TAATTATTTGGGTTTCTCCTTATATCCTCGTGTTTATAATACCCAATTAGTAAAACTGCAGAC
TGACCATTTGAAAACTCTAAATGACTTTCAAAAACTTTTAGGAGACATTAATTGGATACGTC
CTTATTTAAAATTACCCACTTATACCTTGCAGCCATTATTTGACATCCTTAAAGGTGACTCTG
ATCCTGCGTCACCCCGAACACTTTCTTTAGAAGGACGAACTGCTTTACAATCAATAGAAGAA
GCTATTAGACAACAACAGATTACTTATTGTGATTACCAACGATCATGGGGTTTGTATATACTT
CCTACCCCCCGAGCACCCACAGGGGTTCTCTATCAAGATAAACCTTTGCGATGGATATATTT
GTCTGCTACTCCAACTAAACATCTGCTCCCTTACTATGAACTTGTTGCAAAATTGTAGCAAAG
GGAGGTCACGAGGCCATCCAATATTTTGGTATGGAACCCCCTTCATTTGTGTTCCTTATGCTT
TAGAACAACAAGATTGGCTTTTTCAATTTTCAGATAATTGGTCTATAGCTTTTGCAAATTACC
CGGGACGGATTACTCATCATTACCCTTCTGATAAATTGTTACAATTTGCTAGCTCTCATGCCT
TTATTTTTCCAAAAATAGTTCGCCGACAACCTATTCCCGAAGCAACACTTATATTTACAGATG
GATCTTCTAATGGAACTGCAGCTTTAATCATTAACCATCAAACCTATTACGCACAAACCAGT
TTTTCTTCTGCTCAAGTTGTGGAATTATTTGCAGTCCACCAAGCGTTGCTAACTGTACCTACT
TCCTTCAATTTATTTACAGACAGCTCCTATGTGGTCGGTGCCTTACAGATGATTGAAACTGTT
CCAATTATCGGCACCACCTCTCCTGAAGTTCTTAACTTATTTACATTGATTCAACAGGTTCGC
CATTGCCGCCAACACCCCTGTTTCTTTGGACATATTCGTGCACACTCCACCCTTCCTGGTGCC
CTCGTACAAGGCAATCACACTGCGGACGTTCTTACTAAACAAGTGTTTTTCCAATCAGCTATT
GATGCAGCCCGAAAATCCCATGACTTACATCACCAAAATAGTCATTCTTTACGCTTGCAATT
TAAAATTTCCCGTGAAGCTGCACGGCAAATTGTTAAATCTTGCTCTACTTGTCCTCAATTCTT
TGTTCTCCCTCAATATGGTGTCAACCCTCGAGGTTTACGCCGTAATCACCTCTGGCAAACAG
ATGTTACTCACATTCCTCAATTTGGGCGCCTTAAATATGTTCATGTTTCTATTGACACTTTTTC
CAATTTTCTCATGGCTTCCCTTCACACTGGAGAATCAACACGTCACTGTATTCAACATTTGCT
GTTTTGCTTTTCTACTTCAGGAATCCCACAAACCCTTAAAACAGATAATGGACCTGGTTATAC
TAGCCGTTCTTTTCAACGTTTTTGTCTTTCTTTCCAAATTCATCATAAAACAGGAATTCCTTAT
AATCCACAGGGACAAGGTATTGTGGAACGAGCCCATCAATGCCTTAAACATCAATTATTAAA
ACAAAAAAAGGGAAATGAACTGTATAGCCCCTCACCGCATAACGCCTTAAACCATGCTCTTT

ATGTTTTAAATTTTTTAACTTTAGACGCAGAAGGCAATTCAGCAGCCCAGCGTTTTGGGGGA
GAACGATCCTCATGCAAAAAACCACTTGTACGATGGAAGGATCCACTTACCAATCTGTGGTA
TGGGCCAGACCCTGTACTAATATGGGGATGAGGGCATGTTTGTGTTTTTCCACAGGATGCCG
AAGCGCCGCGCTGGATTCCGGAAAGGCTGGTACGCGCGGCAGAGGAACTCCCTGACACATC
AAATGCAACGCATGACACTGAGCGAGCCCACGAGTGAGCTGCCTACCCAGAGGCAAATTGA
GGCGCTGATGCGTTATGCTTGGAATGAGGCTCATGTACAACCTCCAGTGACACCTACTAATA
TACTGATCATGTTATTATTATTGTTACAGCGGATACAAAACGGGGCGGCTGCGGCTTTTTGG
GCATACATTCCTGATCCGCCTATGATTCAATCCTTAGGATGGGATAAAGAAACAGTACCTGT
ATATGTTAATGACACAAGTCTTTTAGGAGGAAAATCAGATATTCACATTTCTCCTCAGCAAG
CCAATATCTCCTTTTATGGTCTTACTACTCAATACCCTATGTGCTTTTCTTATCAATCACAGCG
TCCTCATTGTATACAGGTGTCAGCTGATATATCCTATCCTCGAGTGACTATTTCAGGCATTGA
TGAAAAAACCGGAAAAAGATCGTACCGTGACGGAACCGGACCTCTCGACATTCCGTTTTGTG
ACAAACATTTAAGCATCGGCATAGGAATAGACACTCCTTGGACTTTATGTCGAGCACAAATT
GCATCGGTGTATAACATCAACAATGCCAATACCACCCTTTTATGGGACTGGGCACCTGGAGG
AACACCTGATTTCCCCAAATATCGAGGACAGCATCCACCCATTTTTTCTGTAAACACTGCTCC
TATATATCAGAAAGAACTATGGAAACTTTTGGCTGCTTTTGGTCATGGCAATAGTCTATATTT
ACAGCCCAATATTAGTGGGAGCAAATATGGTAATGTAGGAGCTACGGGGTTTCTATATCCCT
GAGCTTGTGTTCCTTACCCATTCATGTTGATACAAGGCCATATGGAAATAACACTGTCATTG
AATATTTATCATTTAAATTGTTCTAATTGCATACTTACTAATTGCATTAGAGGTGTAGCCAAA
GGAGAACAAGTTATAATAGTAAAACAACCTGCTTTTGTAATGTCACCTGTTGAAATAACTGA
AGAATGGTATGATGAGACTGCTTTAGAATTGTTACAACGCATTAATACGGCTCTTAGCCGTC
CTAAAAGAGGTCTGAGCCTGATTATTCTGGGTATAGTATCTTTAATCACCCTTATAGCAACTG
CTGTTACTGCTTCTGTATCTTTAGCACAATCCATTCAAGCTGCTCATACTGTAGATTCCTTGTC
ATATAATGTTACTAAAGTAATGGGAACTCAAGAAGATATAGATAAAAAAATAGAAGATAGA
TTATCAGCTTTATATGATGTAGTTAGAGTTCTAGGAGAACAAGTTCAGAGCATTAATTTTCGC
ATGAAAATTCAATGCCATGCTAATTATAAATGGATTTGTGTTACAAAAAAGCCTTACAATAC
TTCTGACTTTCCGTGGGATAAGGTGAAAAAACATCTGCAAGGAATTTGGTTTAATACTAATG
TTTCTCTAGATCTTTTACAATTGCACAATGAAATTCTTGACATCGAAAATTCTCCAAAAGCTA
CTTTGAATATAGCTGATACCGTCGATAATTTTTTACAAAATTTGTTTTCTAACTTTCCTAGCCT
TCATTCACTGTGGCGAAGTATAATTGCTATGGGCGCGGTTCTGACAGTTGTGCTTATCATAAT
TTGTTTAGCTCCTTGCCTTATTCGTAGCATTGTTAAAGAATTTCTACATATGAGAGTTTTAAT
ACATAAAAACATGTTGCAACACCAACATCTTATGGAGCTTTTAAAAAATAAAGAGAGGGGA
GCTGCGGGGGACGACCCGTGAAGGGTTAAGTCTTGGGAGCTCCCTGGCAGGTATGCCGGGC
CCTAGGACACGTGCCTAAGCTCCCTGTCCCGCCACCCTCAAGAGTTTTTGTAACCCTTAAGG
CTCCAAGATGTTTGGTTTCGGCAACATTTCATAGAAGATAGATTATCTTATTGTGTATACTTC
ATAGAAGATAGATATTCTGATTGTGTTCTATATACAATGGTAAGGGTCTGGTGATTGTATCCT
GAGATTAAAAAACAACCTTGTGAGTGCCTTAAGTTACGTACTTTACCCTATATATACCGCAG
CACAATAAAGCAAGGTATCAGCCATTTTGGTCTGATCCTCTCAACCCCATCTTTTGTCTATCT
CTTATTTTCTTAGCGGGGACGCTCCGTTCTCTCCCTGTGCAGGTGCGACTCTTGCTTGTGCTG
GCCGCGGCAGGTGG

Data Set 4

Data Set 4. Sequences for Chromosome 4 in sheep (*Ovis aries*), *CD36* gene and Ov-ERV-R13-*CD36*.

>NC_056057.1:c42043163-41861025 *Ovis aries* chromosome 4

TATAGCCAGTTGATGGGTGGTATCATTGGTTCAGCTATGTCCTTATTGATTTTCTGCCTGCTC
GATCTATTTTTTATAGAGGGGTGTTGAAATCTGCAACTATGATAGTGGATTCATCTATTTGTT
CTTACAGTTTTATCAGTGCCCTATCCCCGCCATTTCACTGTTGGGTAAATACCACTTAGGGAT
TATTGTCTTTTGGGAGAGCTGATGTCTTTATTATCATGTAATGTCTCTTTATTATTATTTTCCT
TTCAATTTTGCATGATAATTCCTCTGAGAACAGAATTCTAGGTTGGTGGATTTTTTTTTTTTTT
CTCTCTCTCTTAGTAGGTAAAGAGTTTTATTCGCTTCTGGCTTTTTTTCAGGATCTACCCTTTA
TCTCTGATTTTCTGGACTTTGAATACAGTATTTTTAGGGTTTGTTTTGGTTTGATTTGTTTTGT
TCTATTTGTATTTATCCTGCGTGGTACTCTCCAAGCTTTCTGGATCTATGGCTTGGCATCAGA
CATTGACTGGGGGAAATTCTCAGTCATTATTTTCTCAAATATTGCTTCTGTTCTTTTCTTCTCC
TTCTGGCACTCCACCACACACATATTATGCCTTTTGCACTTGTCCCACAGTTCTGGAATATTC
TGTTCTGCTTTTATTCATTGTTTTTCTCCTTTTTCAGTTTTTTAAAATTTCTGTTTCGATATCTT
CAAGCTCAGAAATGTTTTCCTCAGCTCTGTATAGTCTACTAAGTCATCAAAGACACTCCTCAT
TTCTGTTACAGTATTTTTGTCCTCTGGCACTTCTTTTTGATTCTTTCTTGGAATTTATATATCTC
TGTATGTGTTACCCATCTGTTTTTGCATGCTTTCTACTTCATTACCTCAGGATCTTAATCACGG
CTGCTTTCAGTTCCCAGTATGATAACATTCTAGCTATACTTGAGTCTGGTTTCCGGTGCTTGC
TCTATCTCTTCAAACTGGGCTTTTTGCCTCTAAGTATGCCTTGTAATTTTACCAAATAAAAGG
AACTGCTGTAATGGGCTTTTACTAATGTGATGATAAGGTGTTGTGGGGAAAGAGAAGCATTC
TGCATTCTTGCAGGTAGGTCCCAGTTTAGTGAGTCTGTGCTTCTGGACTGGACTTCACAAGTG
CTTCTCAGTCCTCTCTCCTCCACCAGGTTGGAGCAGGATGTCTCTAGCGGGCTGGAGTTGGGT
ATTTTCTCTTCTCCCGCTTTAGTTAGGCTCTGATATATTCCCAGCAGATTAGGCTGTGGCTAA
ATGGTTTCTGGTAAGGACTTTCCTGGCAGTCCTCTGGTTAAGATTCTGGGCTTTCACTGCAGG
GCCAAGGGCTCCATCACTGGTCAGGGGACCCCCATAAGTTGTGCAGCCTAGCAAAAAAAAA
AAAAAAAAGAGTTTTCTGCTGAGGGCAGACCCTGTTATGAAGGATAGAGAACTCTGGCATG
TTTCAAAATAATTCCTCTAAGCAAATTTTTTGCTGATCTTCACTGTGATAACCTGCTTGAACT
CCAGGAGATAAATGTTGTTGAAGTATGGAGCCCACCTCTGATCAAGTCCCCTTGGAATTTTA
AACACTCAGACTTGTCCACATTGAGCCTCTGGCAATTTGTCACTTACAGTTCAGGATTTCTAC
CCTGGCTCTGGTTCCCACAGAGGTTTTTGCTCTGGTAAGTTGTGATTCTTTGTATTTATCTTTA
CGTCTCTACAATTTGGTGATGAGATAGCAATGTTCTCTGTGACCTTCCTTCTCTTAGGGTTCT
AAAAAGAGTTGTTGATTTTACAGTTTGTTCAGCTTTTTACTTGTTAGGATTCAGTGGTGACTT
CCAAGCTCCCTACATTCGAACAAGAAATTGAAAGTTGAATTTGCATTTTTAACTGAAATATC
TCACAAACTTGTGACTCCTTTTCATTCATATTTCCTCTGCTTTTGTTTCTTATCTATTCTGTGAC
TTACTGTAATAGCACAGTAATTGGTGCCATATTTTTCTCATGATTCTTCTTCAGTGCATTTTAA
ATGTATTAGTATAGATCCATGTACAGAGTTTTAATATTCCATGAATTTAATATTCCATAAATT
TTCCTAGGTCCCACAAGGACACACTTGTGACTTATAATAATTTTGAAGACAGAAGATTCTTA
GATTGTTGTTCAAAGGGTCTTTCACAAAATAAAATTATCCAGAAATATAAAAATTTTAAATG
ACTTTAGCTAGATTTTTTGAAGGATGATTATATTACCATAAAACCAAAGTTATTTAAGTTGGT
ATTACTCTTTCTGCTTTGCAGGGAAATGAGCTGAAGATTGTTTCTTAAACCAGATCTTGCTAC
AAATTAGGGTAGAAGGTCATGAGGACTTGGATGTATAGGATGGGTCTGGAAATGATGACAG
CAAAAGGGAGAAATTGGACAGAATAAATAAACTCGAAGCCCAAATGACAACAGTCAATTTC
CAGTTTAGTGAGAAGTGGACTTCCTAAGTTCATCTGTTCCTAAACATAATTGACCTTATTATC
ACTCTTTTTATCATCTTCCTTCACCTGTAACATTCTGAGTATTTTTTGAGAACAATAAATACAT
GATTTTGCCTGGAATAACAATTATTCTAAGAAAATCATAGGTGGGAAATAAATTAACTGTAA
CAGGTATAACATATATTACTGTAGGGAAGAGGAACACACTCACTCACTCATTCACTTGCTCT
TTCACTTAGGCTGCCAGATAAACAGCCTGCTTGACTTGGCTACTCAGCTACACATGCAGCAG

CCAGTCTTCCGCAGTATACAGGACTCTCCTTGACCTCCCAGGCACTTTTCTTGTTATCACACC
ACACCCCTAGTCATACTAAGCATCTTAACCTCCACAGATGTGACCTTCTTTCTCTGGACTTCA
GAGATTTTGCACAAATTTCTTCCTCTATCTTGAATTTTCTTACTGCTTTAAACTTCTCCAATTT
GGATAGCTCCTCTAATCCTTCAAGTGTTAAATTTAATATCTTATCCTCTTAAAAGTGAAAACC
AAATACTTTCTATTCTAGTGCACGAGGCCAAGTATTCTTCCTGCCACTCTTGGCTTATCTAGC
TCAAAGTATTCAACAACACATGAAACTTAATCATTCCATTTTTAATTCCTCAAATGCAGTTCC
AGAATAACTAGGGGAGATGATAAGTATTACTTAACTGTTCTAGTCTCAGATCTTGACATACT
ACCTGATTGGTGGTAGGCATTCAAATTTTGTTGACAGAATGAGTGATTTCTGTCTTAGAATG
ACCATTCTTTTGTCTAACTTGATAGAATCGAATTGTTCTGAATGTGGTTAGGCATAGTTCTAA
GCTATGGATATTTGGTGGGAAATTTTTTTCTCTTAAAGATATTGATATAGTATTGTCTCAGCA
TTAATCAAACTAAAACAAGATATGTTGCATGAACAAACAATGCCAAGATCTCAGAAACTTG
GAATAACAATAATTTCATATAGTGTTTATACTACAGTGAGAAAAATTATATGGGGATGCTAT
TCATGGAATGAGGAAACCAGAATTCTAAGGACAGAGTACTGTGGAATTCGGTTCAATCACTC
AGTTGTGTCCGACTGTTTGTGACCCCATGAACCGCAGCATGCCAGGCCTCCCTGTCTATCACC
AACTCCCGGAGTCCAGCCAAACCCATGTCCATTGAGTTGGTGATGCCATCCAACCATCTCAT
CCTCTGTCGTTCCCTTCTCCTCCTGCCCTCAATCTTTCCCAGCATCAGAGTCTTTTCAAATAAG
TCAGCTCTTTGCATCAGGTAGCCAAAGTATTGGAGTTTCAGCTTCAACATCAGTCCTACCAAT
GAACACCCAGGACTGATCTCATTTAGGATGGACTGGTTGGATCTCCTGAAAATCCAAGGGAC
TCTCAAGAGTCTTCTCCAACACCACATTTCAAAAGCATCAATTCTTTGGCACTCAGCTTTCTT
TATAGTCCAACATCTATACATGACTACTGGAAAACCATAGCCTTAACTAGATGGACCTTTGT
TGACAAAGTAATGTCTCTGCTTTTTAATATGCTCTCTAGGTTGGTCATAACTTTCCTTCCAAG
GAGTAAGTGTCCTTTAATTTCATGGCTGCATTCACCACCTGCAGTGATTTTGGAGCCCATAAA
AATAAAGTAAGTCACTGTCTCCACTGTTTCTCCATCTATTTGCCATGAAGTGATGGGACTGG
ATGCCATGATCTTAGTGTTCTGAATGAGGAGCTTTAAGCCAACTTTTTCACTCTCCTCTTTCA
CTTTCATCAAGAGGCTCTTAATTCTTCTTCACTTTCTGCCATAAGGGTGGTGTCATCTGCATA
TCTGAGGTGATTGATATTTTTTCTGGCAATCTTGATTACAGCTTGTGATTCCTCCAGCCCAGC
GTTTCTCATGATGGACTCTGCATAGAAGTTAAATAAGCAGGGTGACAATATCCAGTCTTGAC
GTACTCCTTTTCCTATTTGGAACCAGTCTGTTGTTCCATGTACAGTTCTAACTGTTGCTTCCTG
ACCTGCATTCAGGTTTTTCAAGAGGCAGGTCAGGTGGTCTGGTATGCCCATCTCTTGAAGAA
TTTTCCACAGTTTATTGTGATCCACACAGTCAAAGGCTTTGGCATAGTCAATAAAGCAGAAA
TAGATGTTTTTCTGGAACTGTCTTGCTTTTTCCATAATCCAGCAGATGTCGGCAATTTGATCT
CTGGTTCCTCTGCCTTTTCTAAAAGGCAAGTTCATGGTTCATGTATTGCTGAAGCCTGGCCTG
GAGAATTTTAAGCATTACTTTACTAGCATGTGAGATGAGTGCAATTGTGCGGTAGTTTGAGA
ATTCTTTGGCATTGCCTTTCTTTGGCATTGGAATGAAAACTGACTTTCCAGTCCTGTGGCCAC
TGCTGAGTTATCCAAATTTGCTGGCATATTGAGTGCAGCACTTTCACAGCATCATCTTTCGGG
ATTTGAAATAGCTCAGTGGAATTCCATCACCTCCACTAGCTTTGTTTGTAGTGATGCTTCCTA
AGGCCCACTTGACTTCACATTCCAGGATGTCTGGCTCTAGGTGAATGATCGCACCATCATGA
TTATCTGGGTTGTGAAGATCTTTTGTACAATTCTGTGTCTTCTCTCCACCTCTTCTTAATATGT
TCTGCTTCTGTTAGGTCCCTACCATTTCTGTCCTTTATTGAGCCCATTTTTGCATGAAATGTTC
CCTTGGTATCTCTAATTTTCTTGAGATCTCTAGTCATTCCCATTCTATTGTTTTCCTCTATTTCT
TTGCATTGATCACTGAGGAAGGCTTTCTTATCTCTCCTTGCTATTCTTTGGAACTTGCATTCA
GATGCTTATATCGTTCCTTTTCTCCTTTGCTTTTCACTTCTTTTCTTTTCACAGCGTTTTGTAAG
GCCTCCTCAGACAGCCATGTTGCTTTTTTGCATTTCTTTTCCATGGGGATGGTCTTGATCCCTG
TCTCCTGTACAATGTCACGAACCCCAGTCCATAATTCATCAGGCACTCTGTCTATCAGATCTA
GTCCCTTAAATCTATTTCTCACTTCCACTGTATAGTCATAAGGGATTTGATTTAGGTCATACC
TGAATGGTCTAGTGGTTTTCTCCACTTTCTTCAATTTCAGTCTGCATTTGCCAATAACGAGTT
CATGATCTGAGCCACAGTCCGCTCCCGGTCTTGTTTTTGCTGACTGTATAGAGCTTCTCCATC

TTTGGCTGCAAAGAATATCATCAGTCTGATTTTGGTGTTGACCATCTGGTGATGTCCACGTTT
AGAGTCTTCTTTTGTGTTGTTAGAAGAGGGTGTTTGCTATGACCAGTGCGTTCTCTTGGCAGA
ACTCTATTAGCCTTTGCTCTGCTTCATTCATTTGCCTTGTACTCCAGGTGTTTCTTGACTTCCT
ACTTTCGCGTTCGAGTCCCCTATAATGGAAAGGACATCTTTTTGGGGTGTTAGTCCTAGAAG
GTCTTGTAGGTCTTCATAGAACTTTTCAGCTTCTTTAGAGTTACTGGTCGGGACATAGACCAG
TGGAATTATTCTACAGTAAAATAAAGAAGAAAATTTTTTGCATTTAAGCTTTATTACACGAA
AATAATTATTTCTATTGTATCAGTTTCTACTGCATTTATGCATACCTATGAGAGGAAAAATAA
AGTTTATAGTTGAGTCATAACATTTAAATATCCAAGCTAGCCAATTCACTAAATTTTCTTACA
AAAGTGAGGCATTTTTAATTTGAATATCACTATTTTTCTCTAAAAAAGTAATTAGTTTCTTTA
AAGTTTTCCAATAATATTCCAGAATAAATACACATGAAAGGGTATTTCCATTAGATACATGA
ATTAGCAAGGATGAAAAACTTTCTCACAAAAGACCTTAATGACAATTTGTGACTTGCTTGCA
TGTTACTATATTTTTCCAGATTTTATAATTTGGATTAGTTCTAATCAACTTCCAAAACCTTATT
ATGTTTATGATTTCTTTGAAATACTTGGTTTTTAGTAATTATTCCAGTTGTTTGGGGAATTTTT
TTTTTTTCTATTGAGGTGCTAGCATTCTCTAGCTTATTTCCCACAGATAGAATGTCTTACTTTA
AGAATTTTATATTTTATGTCTTTTGCTACTGAGAGAACAGATTATGACACCTTGTTAAATGAT
AATGCATATCACCTCCTACTGTTGCCCAGTTTCTTCTAGCTTATCGCTTCCTTCTGTAGGCAAT
ATTTTCAAGAAAGGACCAACTCTGTCCAAGTGTTGACAAGGTAGATTGATATATGTATGATC
TAAGTTGAGAGGTTTCAAATTCCCAGCAAATCAGGTCCAGTGTCGTGCATTCCCACACTGGC
CAGGCTTTTGAAGAGTAGCTATTCTGCTTCATAAACAATGGCAACTTAAACTGGTGTATTAA
CTCTTCTGTCATTTCCTGTTTTCACATTTTACCTCTTGGAGTTTTAAAGAGCAATCTTAAATGA
TGAGTTATTTTAGAAATGTAAATAATAAGGAAGGTAAGAAGGAGACTTCATTCACAGGCCA
GATGTGATCAGCACAGCTCAAAGCAATGATTTCCTTTGTTGTTCCATAAGAACTATTAAGAG
GAGATCAGAAAGGGTTAAGGAAAACTATTTGTCTCCAAGAGTTAGGAATACACTTTTTCTTC
TGTGAAGGAGAATATGTAAATATATTATATATAAATATAAATATATGTATTTCTTAATTTTTA
TTTCACTTATTTCAAAATCTTCCAATACTTCCTCTAAGTTGAAAGTATTTCACCTTATTTGGAA
GCCACCAAAATTCTGTTTTATTCCAGTCTTGCTTTGGTAATTATATGTCCTCTGAAGTGGCTT
GAGGACCTCCTGGGGAGAGTGACTCCATTTTTCCTTTTAATTTCAATAGTACCTGAGGTACCT
AGACCTTCAATTAATCTCATTAGAATAATAAGTGAATAAGTGAACTGATGGATGTACTTCAA
AACATTATTCAATTATTGAAGAGATTTGTGGAATAAAAGTCTAGAAAGTCTATTCTTTAGAG
TAGTTTTAAAGTAGATTTAAAATTGATTTTCCAGTAAGCAGATTGCTCAAAATACTCAGGTG
AATTCTCAAGATGGATGTGAGTAATAAATAGTGTGGGTCAAGTATCCTGCTGTGAAAGACAC
AATGTGCTTAACAAATGAGTTCACTTAAAAAAAAAAGTTATTTTATTTGTTTATTTGTTTTTG
TGCATCAAACTTTACTCGATACTGTTTGTAGGTAGACAAATACTACAGGCAGGCATGATCCA
AATGTGTTTGGAAGGAAATTAAATGTATTCTAAATGACTTCACCTTTTAAAAAAACATGTTT
CCGAAGGAGCTTTTATTAAGTAAGCCACAATGTACCAACCATTAAGGACATGTAATCTGCTG
TCCAGTTTCATCAAGCAAACCATATATCATTCCTTCAGTTCAATTCAGTGGCTCAGTCATGTC
CAACGTTTTGTGACCCCATGGACTGCAGCATACCAGGCCTCTCTGTCCATCACCAGCTCCTG
GAGTTTACTCAAACTCATATGTCTATTGAGTCAGTAACACCATCCAACCATCTCATCCTCTGT
CGTCCACTTCTCCTGCCTTCAATCTTTCCCAGCATCAGGGTCTTTCAAATAAGTCAGTTCTTC
ACATAAGGTGGCCAAAGTATTGGAGTTTTAGCTTCAACATCAGTCGTTCCAGTGAATATTCA
GGACTGATTTCCTGTAGGATGGACTGGTTGGATCTCCTTGCAGTCCAATGGATTCTCAAGAG
TCTTCTCTAACACCACAGTTCAAAGGCATCAATTCTTTGGTGCTCAGCTTTCTTTATGTTCCA
ATTCTCACATCCATACATGACTACTGGAAAAACTATAGCTTTGACTAGATGGACCGTTGTTG
GCAAAGTAATGTCTCTGCTTTCTAATATGTTGTCTAGGTTGGTCATAACTTTTCTTCCAAGGA
GTAAGCATCTTTTAATTTCATGGCTGCAGTCACCATCTGCAGTGATTTTGGAGCCCCCCAAAA
TAAAGTCTGTCTCTGTTTCCACTGTTTCCGCATCTATTTGCCATGAAGAGATGGGACCAGATG
CCATGATCTTAGTTTTCTGAATGTTGAGCTTTAAGCCAACTTTTTCACTCTTGTCTTTCACTTT

CATCAAGAGGCTCTTTATTTCTTCTTCTCTTTCTGCCATAAATGTGGCGTCATCTGCATAGGT
AATGATTGGAGTAGACAATATCTCATTGAGATTAACTGCACTATGTATGGAATACTGTTTAT
TATTATAAGGTTAATTTGGTGCTTCATTTTGAAGAGAATCATAGTAGCCTGAGAATCTCATG
ACATTTTCACTGAATGCACCTGGAATTTTTTGTTATTTAATTTGTGTCTGCCTCTATTTTGTTT
AAGTATGACTTTATGTAGTATTAATTATACTGGGCTAATCAACCTTGGGGCTCTTAAACAAG
GATGAGATTGTCTTTGTAAACACAGAATATTTATATACATACTTTTTAGTCTTTCTTCAAAAG
GATAAAAAAAGACATTTTTATGAAAACTGGTATGAAAACTAAAAATTCCTTACTATGCAGAC
TAGTATCAGGCCTGTCTGAGAGAAACCAAAGGAATCCCAGGAAAGAATATTAAATGTTCAG
AATTCAGATCTAGGACATCAAAAGAATCCAAAGCTAACACAAATTTACCTTCAGTCAGTTGT
TCTCTCATTATTACATGACTTGTCATGGACAAATAAAAGTGACAACATTTCCATTTTTGCTGT
CATGTGTATGTTATGCTGAGTGTGCATGTCAAATCATTTCAGTTGCATCCGACTCTTTGTAAC
CCCATGGACTGTAGCTCAACAGGCTTCTCAATTCATGGAATTCTCCAGGCAAGAATATTAGA
GTTAGTTGCCATGCCCTCTTCCAGGGGATCTTCCCAACCCAGGGATCAAACCTGAGTCTCCT
GCATTGGTAGGCAAGTTCTTTACACTAGCCCCACCTGAGAAGCTCCTTTGCTAGTAACATAA
GGAGTTTAGGAAGTTTGGCAAAGAAATTCTAGATGTTTATTTATATTCTTTAAAATATTCCTA
GAAAGAAATTTTCTTGCACTTTACTCTCTTGGAGGTAATTTAAGTGCAGAGGAGAAGTCTAG
GTGTCACAAGACTAATAAGAATCTCTAAAAGAATTTATCTCCTTGATTACATCTACAACTTG
CCTATAGCCTGGTTTTAAGGCTTTCTACCTATTGACTCATCTTGACAACCAGCCAAAAGGAAT
TTCTAGGCACAACATACAGAGTGAAAAGCCTCATTCAGCTTTTTCTGCACTCCTGAGAAAGT
ATTTCCATGCACAAATGTATTCTTGTTTCTTTTTAGTTAAAAGTCTCATCTATAAAAATATGG
AGGATTTGGTGGATTTTTTAAAAAATTAGGGGATTTTAAATATTTGGAAACATTATGTTGTCT
TGAGGCTCTAATCGTCATCAGTAACTCCCCTGCTGCGCCTCCTCCTCTCTCCCCACAGACACC
AGAAGTTCACAGCTTCCTTCTCAATTTCCTGGAGAACAAGTTGGAAGATCTTTCTGAGCAGG
GAACATATTTTATTTATCCTTTTCATTTTTGTCTCTCCTATTGTCCCTGAGTCTTAAAAGATCC
TTAAAACTTATTTATTGATTTTCAGACTTCTTTTCTGACTAAATGTCTCCATACATGAAAGAA
GTAGCAGCAAATAATCATAGTTATCAATACAGATATTTTTTACCCCTGTCCCCCCACCCCCAT
TCTTCCAAGTTATTTTAAGAGGACAGTCACTGTAATTAGCTTATACTTCATTATAGTTTACAT
TTTAAAATAATTCTTAACAGTGTTTTGATTTATAGCCTACTTACTTGTTAAAGGCCTCAAATT
TAAAAAGAAAAAAACCCCATAAAACTAGTATGCCTGTATAAATATTTGAAACTAACATTGTT
TTCTTTTTCATGTTTTCTCTCCTTTTCTTTGTATTTTGGAATCATTAAGCCCAAAGAAAACACA
GTTCAAGTAAAGGAGGTATATCTCAGGGAACCATAGTCTGCTTTCCTATGGTTCTTTATCCTT
AGGAAAGAATAAAGCCTATAAGGCTTTCAAGAAGTAACATCAAGAATATGTTGTCCTTAAT
GACAATTCGGCAAGCACTTTATTTTGCCTGTTGTCTCATATCAGTTCTATGACTCTGATAAGA
AATTAAAGAAGTGAAAATATACCTGTCAGAAGTACAGTAATAAAAAAATGAAAAAAGTAAT
TAAAAAATGGAATGCTTGCTATAAAGATAGCAAAATCAAAGAGTACTATGAAGTAGTATAT
TAATAAATTGTTGACATAAATAGCTGAGAAAAGCTTGGGAGTTTATGAAATCTGAGTGAAA
ATAGAAACTTGAAATTTAATGGCAAGTGATGTGTAGAGATTATATATTAAAAAGTTTTTGTG
TCATGAATTAGAGACCTGTATATTTTTCTCAAAGAAATTCAGCCAGTTCAGGGCCAGGACAA
AATTAAACTCAGGTTTTGCCGAAGTCTCAAAAAATTTATTCATAATGTTACAGGAAGGGGGA
CCACTTCCAGGGCCTGAAACTGGGCTCTTGTCTGACACTCGGAAATCAATTGTCCGAGGAGA
CACATGTGCTGACAAAGCAAGAGATTTTATTGGGAAAGGGCACCCGGGTGGAGAGCAGTAG
GGTAAGGGAACCAGGAGAACTGCTCTGCCCCGTGGCTCGCAGTCTCGGGTTTTATGGTGATG
GGATTAGTTTCCAGGTGGTCTTTGGCCCATCATTCTAATTCAGAGTCTTTCCTGGTGGCTCAC
GCATCATTCAGCAAAGATGGATGCTAGTGAAAGGGATTCTGGGAAGTGGACGGACACGCAG
TATCTCCTTTCGACCTTTCCCAAACTCTTCTGGTTGGTGGTGGTTTATTAGTTCTGTATTCCTT
ATCAGGATCTCCTGTCATAAAACAACTCATGCGAATAGTTACTATGGTGCCTGGCCAGGGTG
GGGGTTTCAATCAGTGTGCTTCCCCTAACAACTTCCCCCTGAGAGACTTCATACTCAAGATA

CTTCTTGGGAATTGCTGTGGAGGTCTCTTTCTTCTGTAACTTCTTCCTGCTGTGCATGGGCTTA
GGCCTGCCTAGCAGAGCAGAAGTCTCTACCTGATCTAAGTTGGAGTGTTCCACGTCTGAAAC
CAGCTTCCACCCTTGTAGTAACAGCAATTTAGTTTGAAACTGTTGGCCCCTCTCAGAGATGA
AATAGACAAGAGCCAAACAAACAGGAGACCTAACAGGATGGTCATCTCTGGTCCCTGGAAA
CAGAAGGCAACATTTGAGGTGAGGGTTGCAGGGTCTGTGACCCTTTTTGATTGGTTGGTGGT
GAGACAACAGAGTTGTGCTCCAGGAATCTTGTGTTCAGTCTGACGTTACCATCCTCCACCTG
GGTGGGGGCTCAAAGACATTGTTGTGCATATTTCTTTGAGTAGGAACCAGGACACTGGAGGC
TGTACCAACCTTTGATTGTTTCTCCAGTTTCTGTATCCCCTCCCTTCCCTGATTAGCAATTGTT
TGAATCCACCCTTTGGAACTCATGGAAGGTCAAGGAAGTTGAATGAAGCCTATATCCTACAG
ACAAGAAATGGAGAACACAGAACTGATCTCTACTTCAGAGCCCCACAGAGTTCTACTTAGTT
TTAATTTAGGGAACTAGGCAACTATGACTGTGATAACTCCCTGTCAAAATGGAGCATAAGAC
TGCCTCAGCTTGGAGTATAGACACTGAACTGGAAGTATTTCTGAGTTCCAAGTTCTAGATCT
GAGTCTTGTTTGATTAAAAGCTCAATCCCTTGGTCTGCCATTTTGGTATAACAGACCCAATTA
GAAGTAGTTGGAGGAGTGATAACTGGAATTTAGTAGACAAAATAAATCTCATTTCTTTTTTA
GAAGAATAGGCCTGAAACCCAGTCTGGACCTGGCACTTCAGGGAGACTTGTAGCCAGGTAG
CCAGTTGCCTAGTTTCACAAAAAGTAAGGTTGCAGTTACTAGCTTCCAGCACACGTTGTTTTG
AGAATGGTGGCATCCAAGCCTGACACGACTCAGAAAACTCATGTGTTTAGCAATACAAGGT
ATAATCTGAAAGTACACCCATAGCATCACCTAGTTACTTCCCAGGATATCTGAACCATAGCT
ACTCTATTTTGACTTTTAATTGTAAACTTTTCCTTAAATAGCCAAAGCAGATTTCACCATTAA
TGACGTCAGTGTTTCTCTAGGTATGAGAAGATGCAAGAATTGGGACTCACAAAATCTTCTCC
TGAAAAGATTTAACTATCTAAAGGCCTGTTCTGCCAGTTTTTCCCAGGGCACAGAGTGCCTC
ATTCTTGATTTTCACCCTGAACTCCTTTCAGGGGCGTTGAAATTCAGCAGTTGCAGCGGCCAT
GATTTAATCTTTGTAGATATAGATGGCAAGTGTCAATCTTCAGTTGGCAGAGCCCCTTTTTGC
TCATAAACTTGACCATGATTTTGAGTGGGGCATTTCATGACTATTTTATCCCATGGTGCTGAG
AATGTCCATTCTCAGGTTTGGCAAAGATTTTGTTGACAGGCCACTTAATGTGCTGTTACTGGA
CTAGGCCATAAAACAGTATTCAGCATTCTCTGGACCACCTGTCTTACTAGCCTCTTGGTCCAG
GAAAATACTCCCTCTTCTTGCTTCTTTCCATATCTAGAGTCACACTGTTACCAACATGGATCA
CACTGATTGAAACCGCCCACCCTGGCCAGGCACCATAGTAACCATTTGCATGAGTTGTTTTA
TGAAAGGAGATCCTGATAAGGAATACAGAACTAATAAGCCACCACCAACCAGAAGAGTTCA
GGAAAGGTTGAAAGGAGATACCGCGTGTCCGTCCACTTCCCAGGATCCCTCTCGCTAGCATC
CATCTTGGCTGAGTGATGTGTGTGCCACCAGGAAAGACTCTGAATTAGAATGATGGGCCAAA
GACCACCCGGAAACTAATTCCATCACTATAAAACCCGAGACTGCGAGCCACGGGGCAGAGC
AGTTCTCCTGGGTTCCCTTACCCTACTGCTCTCCACCCGGGTGCCCTTTCCCAGTAAAATCTC
TTGCTTTGTCAGCACATGTGTCTCCTCGGACAATTGATTTCCGAGTGTCAGACAAGAGCCCA
GTTTTGGGCCCTGGAATGGGTCCCCCTTCCTGCAACAATAATATAAACTTAATATATATATAT
AAATAATATAAATATATACATTCTTTCTTTAAATAATATTTATTTATATTATTTATATATATTA
AGTTTATATTTATTAAAATAAGTAATATCAGTTCAATTCAGTTCAGTCGCTCAGTCGTGTCCG
ACTCTTTGTGACCCCATGAATTGCAGCACGCCAGGCCTCCCTGTCCATCACCAACTCCCAGA
GTAAACTCAGACTCATATATATAAATATAAACTTAAAAAATTATTCATAATATAAACTCTGA
GAACAAAGAATTTTGCCTTCTTCATTGTGGTGTCCCTAGGGTCAAAAACAATGCCAGTCCCT
ATTGAGCATTCAACTAATATTTGTGGGATACATGAATGTGTACTGGATACACTGATTTATTA
AGATAATTTTTGAAGCTTCTTTGTTTCTAAGCTTCTGTGTGAATAATAAGGTCCAAATATAGA
AGCCCTAAAATATTTGATGTTATTTTCTTAAGTACTCATGAACTTTTGGTAAATGAATTGGGT
TATATGTAACTACTGACTCTGGAACATCTTTCACCTTTAAAAACATCAACTCCATGTTTCCAC
TGAAAAATCACTGATATAAATTTAAAAATGTATCATTCTAAGATGGTGAAAAAATAATAATG
GTATGTAATGCTGGAAAAATAATATATCAACTATGTTGATGATCTCTGATAATAGCAAAAAG
GTTACTTTAATCTGATTAAACTGGATGATTTAGTAAGAATCATCCTGGAAGCTTGTTAAAAT

GTATATTTTTGAATCCCCTACCAGTTGAAATACTAGACAAGAAAAATCAAGTCTAAACTCCT
CGGCCTGGCATTTGAGGCTTTTCGTATCAGGTCGCGGCTTTGGTGTGGTGTTCATTCCACCCC
CTTTACTCTCAGTCACACGTGTATTTGTGTCTGCTCTACCTAAAATGCTGAATCTGTCAAATT
ATGTTTATCAGCCATTTCTTGATAGATTTACTTCATTGTTTGTTATCTGTCTCCCTCAGTAGCC
AGTAAAATCCCCAGTGGCAGGATCATTCACCAGCATTTCCTAAGACTCTGGTAAATTTTTCT
GGTAAATTGAATATGATCAATACATATTTGTAATATTAATAGTTGAATGGCATGTGTACACT
TTCCTATAGTATAAGCATAATGATGTAAATATGAAATACAGCTGTATCTTCTATGCAATGGG
ACTGTTGTGGGTGGGTTTATTATTTCTCCTGGCTCTGGGGAGTAGTTTCAGGAATGGGTTCAT
TAAATTGATGGCAAGTCAGGGTTGCGTTTGATTGAAGGGGTTTTCTCCATAGGCAAGGACTG
ACTGGGAATCTTGGTTCTCTCTCTCTCTTTCTCTAGATCACACACCCTCAAAATGGCACATGT
CATAAGTATTTTTTCATTACTTTTGCACCTGTGGTTTATTACTATCTACTCCTTATCATAGTTT
GAGAACTCATGTTTATCTTTAATTAGTAGTCACGCCTTTTGCTTAACTAGTTTATTTTTATATT
ATGAATATTTTTCATAGTGAATTCCTATTAGTTTTCTGTAATTCTTTTGGTATTTGTGCTCAGT
GCAAAAGGGAGTTGGCTTTTTGGAAGCATTCCAGAAACTTTGACCTATGATTTACAGCATCT
ACTTAATGACACAAAAGAAAACAAACAAAAGAACATTTTTGAATGTCCCCAGAGACTTTCC
CCATTAACATCTAATTTTAGATTGTTTCAGTTCCTGCTATTGGTGGTCTTAGGAGCTGCCAGT
TCATACAATGCCTTCAAATTTACATGAGAAGGCAATGAGAATATATGGGAACAAAGTTCTGT
TTTTCATATTCTCCATTTTAACCTTTCCCTTTAAAATTAAGCCAGTGTGAGAGGAAATAAACA
CTTTTTGTCTCCATTGTATGATGGGAATGATATCCTCCTCCATTTCCCCAATGATAATCAGCT
TCCAGATGGTTGCCTTAACTTGCAAATTTTAATCAAGTTGACTCCAAAGAGTAATTTGGCATT
ATTTTTACCAAGTGGAGGTCAACTGTTTTTAACATGGAGCCAAAGGGCCAGATAGTAATCCA
AAGCTGTTCAAATTTGGAGTAAAATCGCTTGAGTTAAAGGCTTCCCTGGTGGCTCAGTTATTT
TAAAAATCTGCCTGTCAGTGTAGGAGACATGGATTCAATCCCTGGGTTGGGAAGATGCCCTG
GAGAAGGAAATGGCAACTCACTCCAGTATTCTTGCCTGGGAAATCTCATAGAGGAGCCTGGT
GGGCTACAGTCCATGGCGTTGCAAAGAGTCAGACACGACTGAGTGACTAAACAACAAAGGA
ATTGTCGCAAAAGGTCAACACTTAAATGCTGAGAAAATTGAAATTTTGGGGTTTATGTAACT
AACTGGCCCAATTTTATGAAGGAACTAGAGAAGTCAAAGTCTGGAACTCTGAAGTCCAAGTT
TGTAAGCTGCCTCTCCTGAAGCGATCCAGCACTCCTCACCCCACAAGGAGTCTTCCACTGTA
CAGGAATATGAATGAGCGAAGACTGGGCCGTAGCACACGTGTGCAGTTATAGGATGACTAT
CATGCTGACAGAAGAAGCACTTGCCTGAGTTATGCTCTAGGCATGGAATCTTTCATTCCATTT
TGCGTTTACTGGCTGCTACTCCTTGTTGCAAGTCCTATTATTGTCTTAGGTTTGTTGCTTTCTA
GCTCTTCTACCCATTCTCCCTACGGTTCCATCTTTCATCTCCATGCTAAGAGGGGGAAGTCTA
CTCTGGGTTAAAGTAAAGCCACCCAATCTTTCCTAGTGCAAATGAGAAAGTTTTTCCCCTGC
AGCTTCTCAGTAATCCGTAGGCGTGGCACACCACCTGTCAATTTAATTAAGTCCCAAGAGAC
TTTCTTAATTTTAGTTTGCAGTTTTTAAGGCTGTCTAGAAGTACTTCACGGAGAAGGCAATGG
CACCCCACTCCAGTACTCTTGCCTGGAAAATCCCATGGATGCAGGAGCCTGGTAGGCTGCGG
TCCATGGGGTCGCTAAGAGTCGGACACGGCTGAGCAACTTCCCTTTCACTTTTCACTTTCATG
CATTGGAGAAGGAAATGGCAACCCACTCCAGTGTTCTTGCCTGGAGAATCCCAGGGATGGG
GGAGCCTGGTGGGCTGCCGTCTATGGGGTCGGCAGAGTCCCAGAGTCGGACACCACTGAAG
CGACTTAGCAGCAGCAGCAGCAGCAGCAGTACTTCAAATGGCTCTTAAATGGTTAAGATAG
GCTCTCTTAAGCGACTGTGTGAATTTGTATTGAAAAAGTGAAACACCAATGCATTTTTTTTGG
TGAACATGGTGCTAGAATCAAACAACGTGTTTGCTGTTGAGGAAGGAAATCTTTTCTCTCTG
TAAAAAAAAAAGGGGGGGGGGTGGTGGCAGAAAAGAATTTCATTTCACTTTTTTTTTTCAAC
TTATTGTTTAATTGGAGGAAAACTGCTTTATAATGTTGTTGGTTTCTGCTGTAAAACAATGCA
AATCAGCCATAATTATACATATATCCCTTCTCTCCTGAGCCTTCCTCTCCCCTCCATCCCACC
CCTCTAGGTCATCACAGAGCGACAGGCTTGGCTCCCGGTATTATATAGTGACTTCTCAGCAG
CCATCTGTTTCACACATGATAGTGTATACATGTTGATGCTTTGTTCTCCATTCATACCACTCTC

TCCTTCCCCCACTGTGTCTGACATAAAAGAATTTCATTTCACTTCTTAATGTATCGTATGAGC
TTTGTAAAAGTAAAACCAGATGGAAAGAAATAGATATTTTAGGCAAAAATGAATAAATCAG
GAAGAAATTGGGAGAAAAACTACTTTGAGATTTCATTTTCACCTTTGCTATTATACCTTCTTA
TCTAGGTCAGAAGACTGTTGTGCCTCTTTAATTAGTGTCTTATAAACATGTTATTACTGGGAT
AAAAAAAATCAAGAGGAAAAGTCACCCTTTGGCTTCCTGCCTTTCAGTCAGGAATATAGGCA
GAGCTTCCGCTACTGTTCCTGTCTCTGTGATAACCTTCATTCTAATGACCCAGAGGTAACTCT
GCCTGGTAATAAGTTTTTCCAGCAAGAATAAATCAATTTTCATGTCTCCTGCAGAGTTTCTGT
GGAAATTGAGGCTACTTATAACGATATTTCTTGAGGTAGTACTCTAAAATGGTTACTTATGG
GTCTATGCTTTAATGATGCTAAGTAATTTATCAAGACATAGAACCTATATTTATAATTTATAA
CACTCCCAGAAATTCACACTGCTCCATGATAAAGTAAGGATTCTGTTTTTCTTGCTTTTACTA
TCTCTGGGCCATAAATCTTAAATGAAAGTAGCTTATGCAGCGTATCTAACTTGGGAATAACG
GACAGAGTCAAATTATCTATTGACAAGGACCAAACATTTAGTGTGAATTAAGATATTTGAGA
ACTAACTTTGGAATGCTTTACAGGCATACTGAGAAACTGGAATTTTCTAACAACTTTCAAGT
GCTCATCTCTCAGTGATAACATTTATTTATGAGACTAAATATATTCTTATATACTTAGTAAGT
CACTCATTTTTGGATGAATCAGTGTCTCTTCTATTTAGCCAGTTTCCACATCTATAATAAACA
TTTCCTGTTTTGCTCAGTTTATCATATGTTATGACCTTTAATTCTTTTCTTATGACTCAACATC
TTATGACATACACAAGTTTAAATGTGTGATATGTGTGGATATGGAGTGTGAGGATAGATAAG
TGTTGGTTTTACTCGGGTGAAACTCAGGTTCAAGTCTTAATAGATACCTCTTGGACATAAAA
AATGAGCTGGTCGGGAATTTCTTTTCCAAATTTTTCACTAGTTTATCATGTTGAAAATACAAT
GCTTTCTGTTTGCTTTTGTTGTTGTCTATTTTATTTTTCTTCACTAATTTTAGGAAGCTAAAATT
CTACGCACCCAATACATGTACTAGATGGGTATCTCTCTTAAGTCATTTAAAGTATGCCAGAT
ATTAGGTGCTATGAACAGAATGTGTCCTCCCCAAATTTAATATGTTGAAGCATTAATTCCCAT
TGTGATGGTATTTAGCAATGGGGTAATTAGGTTTAGAAGAAGTCATCTGGGCAGAGCCCCAA
GATGGGATTAATACACTTAAAAGAAGACAGAGGAAACAGAGCTTTTTTCTCTGCAAGAAGG
GAGCTTCTATAAACAGGAAGGGAGTCCTCACCAAGAGCCCAACCTTGCTGATCTTGACTTCC
AGCCTCCAGAACCATGAGAAATAACTTGTTTTAACCACTCAGTTTATGGTATTTTTTAATAGA
ATTATCAACTAAGATGTTAGAGTTTATCATCTGAATGTTCTTCTTTGAGTTGTTTATATTGTAT
GCCCAGATGAGTAAGGTGTGCACTAGGCCCACATGGAGCTTACAATCTGGAGCAGAAATCA
CAATACATATACCAGCTGCTGTGATATAAGCTAAAAATTAAGAGACAGTATGAGAGTCACA
TCCCATCTGCCATATAAAGAAGAAAAGAAAAAGAAATAATATTTCCATCTTGAGGAATTGTT
TTTACCATTGAGGAACAAGCTAGATTTCTTTAACAAATTTTCAATGTTTATTATTAAAATTTC
AAAAATATGGAATTGAGAAAGAATAGCATAATAAAACCTAAATACCCAATCCCATAGATTC
AACTATTATTAACATTTTGCCATATTTGCTTTTAAAAATTAGATTTTGATATTGGAAAGGCAA
GATAGACATTTCATTTAAAAGAAACAATAAAGGAGGGCACTTGAGTGAAGGCATTCAGAGA
TGGCTTAGGACAAGGCATAAGAGGATAGAGGGCATCTGACGCAGGATGCAGCATGCTTGGG
GCTGGTGCATGGGGATGACCCAGAAAGATGTTATGGGGAGGGAGGTGGGAGGGGGGTTCAT
GTTTGGGAATGCATGTAAGAATTAAAGATTTTAAAATTTAAAAAATAAAAAAAAAAGAATT
AAAAAAAAAAAAAGAGGATAGAGGGCAAAAGAGAAACCAAGATTGTCATATAACATGCCT
GGATCATATGAACATCAAGCTCAGGGACACGTCTATGTAAAGGGATGATGGGGCTAGAAGG
GCACTATTTTGTGTGTAATGCATTATGTTGTGTGTTCTCGTCTGTATTATTTCATATAAGACGT
TCTGAAGTTTTTCTATAGATAAGTTTTTTTTTTTCCTTCTTCAGAGGTGCTATTTAGAAAGAAT
TCCTAGCATGCTGGGTTGAATGTAATTCAGGGAAGATAGAATGAAATCGTGGAGGTCAGTTT
AGCGGACTAGGTATGAGAGCTACATGAGTGCTAATGGAATAAAGCTGAGCAAATGACAAGG
AGCACATCTGCCAATACAGTTTTAGTTAACTTTCAAAGATACGGCCACTGATTTTTTGTGGAT
CATGAAGGGAAGGAGGAGGGAAATTGAAAAAGGGGATAATAATAATCAGCTAGTATTCAAT
GAAAACTTACTCTGTGGCAAGCATAATGCTAAATAGTTTACTTAAGTTATTTTTCATGTGACT
TTCAAAGTCAAATGCCGTTGGTTTTCCTATTCTTAAAAAGAAGAAGTTGAGGCAGAAAGAAA

TTAGGACACTTTTATGGATAAAAAGAATTTGTGAGTAGAAGACTATCTTGGAAATCCAGACC
TGTAGTTTGATTCTACAGGCTTTCATTTTTCTACTCTGCCTCCCTAAAATGCACTTACAAGGA
GAATTTGAGTTTTAGTGAAGGAGAAAGGGACTATGCTATTAAAAATAACAAGAGGAAGAAC
TAACCATGGCAAATGATAATGAGTTTGGTTTGGATGCTGAAGCAGAAGCTCTGGCTGTCATG
CCTGTTCTCTTCTACAGTTTTCTTCCAGTTTTATCTGGTTACCTGGTTGTCCAGAATAAACAAT
AGTTTCCAGCTTCTCTTGCAAATGGGTACTGAAATGTGACAAAATTCTGACCAGTAAGAAGT
AGGTGAAATGCTAAGTTCCGTTTCCAAGAAATGTCATTAAGGAATAAACTTGTGCTCCTCTT
CCACCCCTTCGCCCTGATAGATGGAAGGAGGGGCCATGGCTGAAGCTTCAGCTGCAATTCTG
AAGGATTTATGGTTCGCATCGTGAGACCGGAACAAACCAAACAATAATGAGATAGAAGTAG
CCTGGATCCCTGCCACTGCAAAAATGAGAGAATAAACTTGTCTGAATTATGATTACTCTAGA
ATTTTTCCCATGTGAAAAGCATAATCTTGTCCTACCAAATACAAACATACTGATTTTTATTTC
TCTTACATTGCCTCCTTCTTTTTTCTCCTACTGTGCTAAAGTAATAAGTACCTTCAATGTATAA
ATGCCTATACTTTTTTTTTTTTAAGTATAATTGTTTCACCAGTGGCTTAGGTGGTAAAGAATT
CACCTGTAATACAGGAGACCCGGATTCAATACCTGGGTCAGGAAGATCCCCTGGAGAAGGT
CATGGCAACCCACTCCAGTATTCTTGCCTGGAGGATTCCAAGAAAGAGGAGCTTGGCAAGCT
GCTGTATATGGGATCACAAAGGGTTGGACACAACTGAGAGAATAATACACACACACACACA
CACACACACACACACACACACAGAGTAGAGGGATTGGTGGGTCATGGTAGTTCTGTTTTGCA
TTTTGCAAGGAAACTTCATCTTGTTTGCCACAGTGGCTGTATCAATTTGCATTCCTACCGACA
GTGCAAGAGGGGGTTCCCTATCTCTACGCTATCTCCAGCATTTGTTGTCTGTAGATTTTTTGA
TGATGGCCATTCTGACCAGTGTGAGGTGATACCTCATTGTAGTTTTGACTTGCATTTCTCTAA
TAATTAGTGATGTTGAGCGCTTTTTCATGTGCCTGTTGGGCACCTGCATGTCTGCTTTGGAGA
AATGTCTGTTTAAGTCTTCCACCAATTTTTGGCTTGGGTTGTTTGTTTTTTTGATATTGAGCTC
CATGTTGTTGTTCAGCTGCTAAGTCCTGTTCAACTCTTTGGGATCTCATGGACTGTAACACAC
CAGGTTTCCTTGTCCTTACTGTCTCCTGGAGTTTGCTAAAATTCATGTCCATTGAGTCGGTGA
TGCTACCTAACTATCAAATCCTCTGCTACCTCCTTCTCCTTCTGTCTTCAATCTTTCCCAGCAT
CAAGGTCTCAATAAGTCAGTGCTTCACATCAAGCGGCCAAAGTATTGGAGTTTCAGCTTCAA
CATCCGTCCTTCCAGTGAACACTCAGGACTATCTCCTTTAGGATGGACTGGTTGGATCTCCTT
GCAGTCCAATGGACCCTCAAGAGTTTTCTCCAGCACCACAATTCAAAAGCATCAGTTCCTTG
GTGTGCAGCCTTTTATGGTCCAACTCTCGCATCTGTACATGACTACTGGTAAAACCATAGCTT
TGACTAAATAGACTTTTGTCAGCAAAGTGTCTCTGCTTTTTTAATATACTGTATAGGGTTATT
TTTTTTTTCTTTTTAATAGCTTTTCTTCCAAGGAGCAAGTGTCATTTAATTTCATGACTTTGGT
CACCCTCTGCAGTGATTTTGAAGCCCAAGAAAATAAAATCTGTCAAAGACCTGGGTTTGATC
CCTGGTTTGGAAAGGTCCCCTGGAGAAGGGAATGGATACCCACTCCAGTATCCTTGCCTGGA
GAGTTACATAGATAGAGAAGCTGGGTGGGCTATAGTCCATGGGATCACAAAGAGCTGACTC
ACACTGAGTGACTAACACTCTACTAGCATGTGAAATGAGCACAATTGTATGGTAGTTCAAAC
ATTCTTTGGCATTTCCTTCTTTGGAATTGGAATGAAAACTAATCTTTTCCAGTACTGTGGCCA
CTGCTGAGTTTTCCAAATTTGCTGACATATTGAGTGCAGCACTTTAACAGCATCATCTTTTAG
GATTTTAAATAGCTCAGATGGAATTCCGTCACTTCCCCTAGCTTTGTTCGTAGTAATGCTTCC
CAAAGCCCAGTTGAATTCACACTCCAGGATGTCTGTCTCTAGGCAAGTGACCACACCATCAT
GGTTATCCAGGTTATTAAGCTTTGTGAGCTCTATAAGCTGTTCATATATTTGGGAGATTCATC
CTTTCCCCATTGCTTCATTTGCAAATATTTTTCTCCCATTCTGAGGGTTGTCATTTTGTCTGCC
TTCTGTTTCCCTTTGCAGTGCAAAAGCTTGCAATATTAAATTCTGTTTATTTTATTTTTATTTT
CATTATTCTGGGAGGTGGGTCAGAAAAAACCTTGCTTTGGTTTATGTTAAAGAGTGTTTTTCC
TATATTTTCCTCTGAGTTGTATAGTGTCCAGTCTTACCTTTGGGTCTTTAATCCACTTTGAATC
TATTTTTGTGCATGCTGTTGTTGTTTTTGCTTAGTGGCTAAGTCCTGTCTGACTCTTTGGGACC
CCATGGATTGCAGCATGCCAGGTTCCCCTGTCCTTCATTATCTCCTGGAGTTTGCTCAAATTC
ATGTCCATGAGTCAGTGATACTATCTAAGCATCTTATCGTCTGCTTCCCTTCTCCTTCTGACTT

TAGTCTTTCCCAGCATCAGTTTCTTTCCGAATGAGTCAGTTCTTTGCATCAAGTAGCCAAAGT
ACTAAAACTTCAGCTTCAGCATCAGTCCTTCCAATGAATATTCAGGGTTGATTTCCTTTAGGA
CTGACCTGTTTGATCTCCTTGCTCCCCAAGGGACTCTCAAAAGTTTTCTCCAGCACCACAGTT
CAGAAGCATCAATTCTTTGGCACTCAGCCCTCTTTATGGTCCAGCTCTCACATTTATACATGA
CTACTGGAAAAACCATAGCTTTGACTATATAACCTTTGTCAGCTAAGTGAAGTCTCTGCTTTT
TAATACACTGTCTAGGTTTGTCATGGCTCTCCTTCCAAGGAAGGAAGCCTTCTTTTAACTTCA
TGGTCTCAGTCGCTGTCTGCAGTGATTTTGGAGACCAAGAAAAGAAAACCTATCAGTGCTTC
CACTTTTTCCTGTTCTATTTGCCATAAAGTTATGGGACTGGATGCCATGATCTTAGTTTTCTTT
CATTTTTTTAAATGTTTAATTTTAACCTAACTTTTTCACTCTCCTCCTTGACCCTCATCAATAG
GCTCTTTATTTCCTTTTCATGCTCCTCTTCACTTTCTGCCATTAGAGTGATATCATCTGCATAT
CTGAAGTTGTTGATATTTTCCCAGCAGTCTTGATTCCAGCTTGTGATTCATCCATCCTATCATT
TTGCATTATGAATTCTGCATGTAAGTAAAATAAGCAGGGTGACAATATACAGCTTTGTCATA
CTCCTTTCCCAGTTTTGAACCAGTCAGGTTTTCCATGTCTGGTTCTCACTGTTGCTTCTTGACT
CACGTACAGGTTTATCAGACCCACCACAGCTTAGGTGGTCTGGTATTCCCATCTCTTTGAGA
ATTTTCCATTGTTTGTTGTGATCCACACAGTCATAGGCTTTAGTGTAGTCAGTGACGCAGATG
TTTTCTATGATCCAATGAATGTTGGTAATTTAATCTCTGGTTTCTCTGCCTTTTCTAAATCCAG
CTTGTACATCTGGAAGTTCTCAGTTCATATATTGCTGAAGCCTAGCTTTTTGAGCATAATTAC
TAGCATATGTGTGCTATTAGATAGTTTTCTTTATATGTTATTCTCATTCACCATGTTTTATAAG
TTGACTTCTTATTTTCATAAGCAGTTCAGTTCAGTTCAGTCGCTCAGTCATGTCAGACTCTTT
ACAACCCCATGAATCGCAGCACGCCAGGCCTCCCTGTCCATCACCAACTCCCAGAGTTCACT
CAGACTCACATCCATCGAGTCCGTGATGCCATCCAGCCATCTCATCCTCTGTCATCCCCTTCT
CCTCCTGCCCCCGATCCCTCCCAGCATCAGAGTCTTTTCCAATGAGTCAACTCTTCGCATGAG
GTGGCCAAAGTACTAGAGTTTCAGCTTTAGCATCATTCCTTCCAAAAGAAATCCCAGGGCTG
ATCTCCTTCAGAATGGACTGGTTGGATCTCCTTGCAGTCCAAGGGACTTTCAAGAGTCTTCTT
CAACACCACAGTTCAAAAGCATCAATTCTTCAGCACTCAGCCTTCTTCACAGTCCAACCCTC
ACATCCATACATGACCACAGGAAAACCATAGCCTTGACTAGATGGACCTTAGTCGGGAAAG
TAATGCCTCTGCTTTTGAATATACTATCTAGGTTGGTCATAACTTTTCTTCCAAGGAGTAAGC
GTCTTTTAATTTCACTGCAGTCACCATCTGCAGTGATTTTGGAGCCCCCCAAAATAAAGTCTG
ACACTGTTTCTACTGTTTCCCCATCTATTTCCCATGAAGTGATGGGACCAGATGCGATGATCT
TTGTTTTCTGAATGTTGAGCTTTAAGCCAAATTTTTCGCTCTCCTCTTTCACTTTCATCAAGAG
GCTTTTAGCTCCTCTTCACTTTCTGCCATAAGGGTGGTGTCATCTGCATATCTGAGGTGATTG
ATATTTCTCCCGGCAATCTTGATTCCAGCTTGTGTTTCTTCCAGTCCAGCGCTTCTCATGATGT
ACTCTGCATATAAGTTAAATAAGCAGGGTGACAATATACAGCCTTGACGTACTCCTTTTCCT
ATTTGGAACCACTCTGTTGTTCCATGTCCAGTTCTAACTGTTGCTTCCTGACCTGCATACAGG
TTTCTCAAGAGGCATGTCAGGTGGTCTGGTATTCCCATCTCTTTCAGAATTTTCCACAGTTTA
TTATGATCCACACAGTCAAAGGCTTTGGCGTAGTCAATAAAGCAGAAATAGATGTTTTTCTG
GAACTCTCTTGCTTTTTCCATGATCCAGCGAATGTTGGCAATTTGATCTCTGGTTCCTCTGCCT
TTTCTAAAACCAGCTTGAACATCAGGGAGTTCATGGTTCATGTATTGCTGAAGCCTGGCTTA
GAGAATTTTGAGCATTACTTTATTAGCATGTGAGATGAGTGCAATTGTGTGGTAGTTTGAGC
ATTCTTTGGCAATGCCTTTCTTTGGAATTAGAATGAAAACTGACCTTTTCCAGTCCTGCAGCC
ACTGTTGAGTTTTCCAAATTTACTGGCATATTGAGTGCAGCACTTTCATTTTCATAATATTAT
TGTTAGTATTCCCTTCTTCTGTGAAAACATTTTGTTTAGCAGGAATGAACTTAGAAGTCAGAT
ATGATTTAGGAGTAAATCTCACCTCTGCCATGTATTAGCTATTGTTATTTAGTCGCTAAGTCA
TCTCCAAGTGTTTTTCAGTCCCATGGACTGTAGCCTGCCAGGCTCGTCGTTCTATGGGATTTT
CCAGGAAAGAATACTGGAGTGGGTTGCCATTTCCTCCTCCAGGGGACCTTCACAAAGCAGG
GACTAACTCACATCTCCTCCATTGGTGGGTGGATCCTTTACTACTGAGCCACCAGGGAAGCC
TAGAGAGTATTTTGGTAATACAGAAGTAATGTGAACATTTGATTTTCATAATACATGCATGC

ATGCTATCTAAGTTGCTTCAGTCATGTCCAAATCAGTGTGACCCCATAGACTGTAGCCCGCC
AAGCTCCTCTGTCCATGGGATTTTCCAGGCAAGAATCCTGGAGTAGGTTGCTCTGTCCTACTC
TGTTTATAACATATATTGTCACTTAATTCAGATTCTTAGTTGAATTCTGGTGAACTCTTCATAT
AAATTTATGATGGTGTATCAAATTTTAGTGGCAGCATTTTGAGGTAAAGATGCTAATATTAG
AATTGTTGATGTAATTATTATCAAGTGTATACTGTTACCAATAGATAAATATGATAATATTTG
GAAAAACTTCAATGAAAAAAAATGTTTATAGAATCAGTCATGATTTTTATATGGAGCATCTT
CTTTGGTTATCTCATGACCATGTTTGGTTTCAAGACAACTTGGGAACCTTAAGAGCATATATG
TCTCTTCAGAATGCACTGTTAAGCATCTGTATAGTTCTTATGGGGCTTTGGCTTGTTTCAAAT
TACTTCCCTAAATAACTGATGTGTATAAGATAAATAACTGAAGAAGGGTCTATGTAAACTGG
AATTCAGGGAGAATTTTGGTGTAAGGAAGAAGAGTTAAAGCACAAAAACAGGAAGGGCAA
TGAATTATTAAGGAATGGTACCTCAGTGCTGGGGATTATGGGTTGGATCTACCTTGATGTTTT
GGGAGATGCACTTGAAGATACAGCTTTATTTATTTGAATTACTTGAGTATGTTCCTTTGAATG
TGGCACTTGGTGGACTTGGAGGTAGCACTCAGCCTGTTATCTGTGTTACATAACGGTTGTAA
TAGAAAAAGGTTTCAGTGCTTATTTCCTAATAAATGTTGTTCTGCTGGTGCTGCTAAGTCGCT
TCAGTCGTGTTTAACTCTGTGCGACTCCATAGACAGCAGCCTACCAGGCTCCCCCGTCCCTCG
GATTTTCCAGGTGAGAACACTGGAGTGGGTTGCCATTCCCTTCTCTGTAAAGAGATAATGGA
GGTATATAAACATTTATATGTCATATGTATGCAAATATAAGATAATATATAATACATGCATT
TATATGTATATATAAGATAAAAACAATAATAGTAATGATAGTTGTAGTTAACATATGTTAAA
CATTTTTTAAACTTTTAATTTTGTATTGAGGTATAGCCAATTAACAATGCTGTGATAGTTGTA
GGTGAACAGCAAAGGGACTCAGCTATACATATACATGTATCCATTTCCCCCAGATTCCCCTC
TCATCCAGGCTGCTACATAACACTGAGCAGAGTTCCATGTGCTATACAGTAGGTCCTTACTG
GTTATCCATTTAAAAATATAGCAGTGTATATATCCATTCCAACTTCCCTAACTTCCTGCAGAA
GAATTGGAGCCTCTCTGTTGACCAACTCTGGCTGTAGGCATTGCAGTTTTTGGTGCATCTATC
AATTTGCTGAGCATACTTCTCAAATGTAATGGTTTTGACAAGATTCAGAAAACTGTAGTGGA
TCAGACGGGCAGCAGACCACCTAGACAGGGACCATGACTTCTTTTTGGTGCAAGTTTAGTTT
GGACAAGTTCTTTGGAGCTTCTTCTCAGTCCAACCATTGAGCTGGCCATTGCTGGTGATTGTA
TAAAATCCACTTTTTGTTGCATGTCACAATCCAACTGAGAAATGATTTGCTGTTTTGAGTAGA
ATAAGAGAATACGTCGCTTCAAAGCAGTGATTTTTTTTAAATTTCGATAAGCTCATGAAGCA
CTCTCTCACTGAGGTTTTCACCTTTTTAACTTGCTTCAAATGCTGAATGACTGTAGAATGGTT
GATGTTGAGTTGTTCGGCAACTTCTTGTGTAGTTTTAAGAGGATCAGTTTTGGTGATTGCTCT
CAATTGGTTGTCAACTTTCAATGACCAACCACTGTACTCCTCATCTTCAAGGCTTTTGTCTCC
TTTGCAAAACTTCTTGAACCATCACCGCACTGTGCGTTCATTAGCAGTTCGGGGCCAAATGT
GTTGTTGATGTTGTGAGTTGTCTGCACTGGCTTATGACCCATTTTGAACTTGAATGAGAAATA
TAAATAAACAGCATGTAATAAGTCATTAGCAAAAAAGCAAAAAAAAAAAAAAAGGCAAGAA
ATGTGCATTAAATTGATATATAACATAACCACATTTATTTAAGAATGTATGTCAGTATCAAA
TAGTGAAGTTCAACAATGCAAAACTACAGCTGCTTTTGTACCAATCTAGTATAGAGAACACA
ATGTGTTATTACATAAATTATGAAATTGGAGTTATTTTTTACCTCTACTCCTTTTATTGGTTTG
AAATCATAGTTTTGCCTCTATATAAGTCTTTAGTCTGCATCCTCTGAGCAGCTTTCTCAGCTG
TTAGCCTGGACATTCTCCAGAGTTCGCTCAAGTCCTAGACTCAATGCTGCCAGTCTTCTGAGT
CACCTGATAAATCTGTTGCAGCAGTATTCTCAAGTGTCACATATGCTGCTTCTAAAACTGAA
ATAGACTAACCAAACTGTTGCACTCCTTTCTTAGAGGCGGCATATACAAGATATAATAACCT
GCTCTTTACCTTAAATGGGGTATCCTAAAAATCACTGGCTCTTAAGACCCTCTCTGGTGATCT
GGGCTATACATGTTGGGAGGTTTATCTTCCACCTTCTAGTTTAAAAGTGTCTGGTGAGGCCAT
CCAGAGTACTTGTGCTTTATACTTATTGGTTCCTATTTGTATTTCAAATTCTCTCTACATAGAA
CCAGAATGACATTGTGCAGAATATGAAGCGTCCTGTATACTATATTATGTCAGAGGGCAAGC
CATTAACAAAGCCTTGGACCATGAATGTAAATGCTTTTTGATTGCCATTGCCATGTAAAGGC
AGACTGATTTTCAAAATTATATTTATTTATAGGGATGGAAAAAATGTTTGAGAGATCAGTAG

ATACCTACTATGTGTTTTATGTTGTGTTGATGTGTTTGAATAAAAATAACACATCTACATTAC
AGCTGTAAATAGAGTCACTTTGTTGAGTCTATGGTATTCCATTGTCATCCATATAGTTTTTAC
AAAATATGCCTATCATGTATGATAGAAAAATATCACATTAACTACATGTTTTTGTTCTGTTTG
AGGAAATATTCCAAAGACACTCTTTAATGCTTATACATTTGTCCTCTTGGTGCTTTATTTCTCT
TTGAAGTGATGGAAACAACAAAACTAAGAGAATTCATGGCCCTGATTATCCTTAGCTTTTTT
TGTCTGGGTAATCCATTTTTTCTATGTTTAGGATACATTGTTTCCAGCTGACGCACAAGATTA
TTTCAGAAGGAAATAATCTGTCCTAAACATAGGTATTCTATTATGTAGCTCAGGGCTTTTAA
AATCTGACTTTGGACACTTTTTTTTTTTTTTTAAGTATCTCTGCTGTCCATTTACCCAAGTAAA
CAGAATTTCCAAGCATTAGTCTAGTCATCTGAACTTCCAAAAAACTCCTTAGCTGATTCTTGT
TCTATTAGGTTGAAAACTGTTAAGAGTTAATTGTTTCTCTTTGTCTTAAACCATCACTTTCAA
AGTTCTATTTTGATAATATTTTCTTGCTTCACTCATACTCATGAGATATATGTTAAGATGATA
GCCTAGGGCCTAAGAAAATAATATAAATCTCAATTAACTAATTCTTCTTATTATTATATAACT
ATAGTCTCTTTTTCTAGAACTCTATGGCCTTCAAACAGTAAGATCATCAGTTTCTGGTGCTGG
TTATTTGTGTAATGAACTTCTTTCTCAATTTTATTCTTAGAATCTAAGTTCATACGGGAAAAG
AAATAAATCTTACAGAGGATTTGGATTGTATCAAGTCTGTGATTTTAAAGATACGGTTCTTTA
GGCTTAAGATTTTCCTTCCTTGTACACTTTTAAATATTATAGGATGGTCATAGAACTGTTTGA
AGTTTGAAAATGTGAGTCTCTTAACGTACAAAGTTGTTGTTAATCTTAGGATCCTAGCATGG
AAACAAAAACAAAGAAAGCTCTGTATGTACTCTGTGTTGGGCCAATCTGAATATATTCACAG
TACTCAATATCATTTCATGCTTTTAGCACAACTGGTTTTGCTTATTTTTTTAACAATTTTGTTT
GTTTACTTATTAGTAATATTTACAAAATTTTAAAAATAGAGAACCTCCAAAAAGAAAAAGTA
CCATCTCTGTCCCATTTCTTTTAACTATTTCTAGTTTTAGTTCTTATAGTTCTTCCACAAATGT
ATGTGCACTCCTGGCTTCACTGCTTATTAGCTTAATGACCTTTGGCTTATTAACCTTTTGTAAC
TCTGTAAAATAGAGATGGAAGTATCAACTTTATAGATTGTAATACATTCAAATATTTTAGAA
TTGTATTTGCCATGTAATATATCTTCTTAATACCTCCCAGTTTCTAAACACTATAGTTAGATC
ACTGTTTTGTGAATCATTAATTTGAAATATTTTCTACTGATGAGATGAAGATCATCTGGACAC
TCCTCTAAAGTCATTTCTGTGTGTATTTTAGGCCACGACTTCCTTGTTTTTATTAGACCTTTCA
GTATTTTTTTTTAATCCCTTTAGCCTTTTCCAGAAATGTGTTGGAACCTCCTGTTCTCTGAAGT
TTCTCCTCCCAGTAATTTTATCAGGTTTATGCGTTTATTATGTGTTTCCTATTATTTGAGTGGC
GTTTTGGAGGGAAGGAAGTAAACAATATGAGCTTAGTTTATCATATGTAAGAATAGACAGCT
TGTTCAATTTAAAATCATACATATGAAGAAATCAAGAGGAAGCTAGAAGAATGCTAAATGA
TACATACTTTCATGGAAACAGGAAAGACAGGGATTTTTGCAAAGTTATTTTGAGTTACCTAA
TATTTCTGATACTTAATTTCCCATTGGATACTTAATTTATCCAATAATGTGGATAAATAAGAT
GACTTCTAAGTTCTTTTCAACTTGAATATTTTATCCTTATATTGATTTTATTTTCCCTACAAAT
CTCTACCTTCCTTTCTACTGAACTATACTGGATTAATCTGTTTCAAAACATAATCCACACATA
CTGCTTTCTCTTTGTATGCCCACTATGTTCATGATGGCACATGAAATTTAAAAGATCTCACAG
AAACATTTATCAGTCTTCAGAAATGATCACCTTTCCCCCAAGGTTAGCAATTATTAATAAAA
GGAAAGAACTTCTAATCCTTTTATCTTAATTTTTATTGAAGATTAGGTTCAGCAGTGATCTTT
TTAGTTAATGTATTTGGGGAATACTTAGTTGTGTATTTGGGAAATACATGCATAATTTGACCT
AATAAAACTGGCAGAATATTACTTTCTTCAGAAATGTTTACTTACTATCCACAGCTGAGTTTG
ATATTCCTTCAAAAGCAAACATCCAGGATTCACCATGATTTTTCCTGTTCCTCCATATTCAAC
ATCCGGTCAATTCATTGGCAAATATGCTTTACTACTAATGTATATTTATCCAGTCTTTTCTTG
ACATCAATACTACCCTCTGTTACTTCTCTACCTCTAGCCTCCTGCCCCAGTCTTCTAACTGGTT
CTGCTGTTTCCACTCTTTTGACCTACATTATACTATTCTGAAATGGTTTCCCAACATACTTAG
AAAGAAATAAAAACTGCTACCGTGACCTATAATACTTTCCTAATCTGGCCTGGTTTCTTTGCA
CCTCATCTTCTATTCATTTCTCTCCCCTTCTTTTGCTCCATTCCCATGATACTGGCTCTGGAGT
TTATCTTATTGTCAAACCGTTTGCATCTTGCGGTCACGGCAGTTGTGGTATCCTCTGACCAGA
ATAGTCTCTCAGATCTTTCTATGGCTTATCTCTTTTCCATTAACTTCTTTGTTCCAATGCATCA

CCTCAGACAGGACTTCCCAAAGAGCCAAGCTTGTTGTTCAGATCTAGGCACTCTGTTCCTTTT
GTCCTAATTTATTCTTTTCATGCATTTAGCATTTTGTGGTAGCACATGAATTATTTATGAGTAT
GTATTCATTTATATTTTACTTCTCTTTATCTCCATCCATCTCTCTCCATCTCTCTCTTCCTCTCC
TTCTCTTCCATAGAAGGGTAAGGCAAAGGTTTTGCCTGCTTCCTGCTCTGATGTATACCTTTT
TCTAATACATGCAATAGTGCTGAAAAAATAATGAGCTTAAAAAAATGTTATTCTTTTGAGAG
GAGGCAGAGTCAAGACGGTTGACTAGGAAGACTCTGGGCTCACATTCTCCTTTGAACATATC
AAACCTACGACTAACTACTGAGAAACTCTCCCTAAGAATAACCTGAACTAGCAGAAGCACT
CTTCTATAAATAAGACTGTAAAGAAAGAATCAAATGGAGTCTAGTAGGAGGTGAGAAGACA
TATGGTAATGTATAGAGAAAATATATAGTAAAGGCAGTGGCTCAATCACTCAAATAAGCTA
GCATGGAGGATAAATGTTAAAAATTGTAAAGTCAACTGTAACTACAGTTAACAGTTAAGGG
ACAGACATGAAGATGTAAAGTATGGCATCAAATACAAAAAAACATGGGGGAGAGATGAGTT
AAAAATGTAAATCTTTTAGAATGTGTTTGAACTTAAATGACTATCAGTTTAAAACACATAGA
TATAATTTTAGGTAAACAAATATGAACTTCATGGTACTACAAATCCAAAATCTACAAGAGAT
ACACAAAAATTGGAAAGAAAGGAACCCAAACATAAAACTAAAGAAAAGCATCTGACCGTA
ACAAGACAAAAAGAAAAAAGACTACAAAAATACCCACAAAACAAGTAACAAAATGTCAGT
GAGTACATAATGATCAATAATCACTTTAAATGTCAATGAACCAAATGCCCCAAACAAAAGA
CTTAGGGTGGGTGATTCAATAAGAAAAAAAGACAGAGCTAAATGTTGCTCACAAGAGACTC
AGCTAAAGACATAGATTGAAACAGAGGCAATAGAAAAAGATAGTCTATGAATCATATGGTT
GTTCTATGTTTAATTAATATTACTCAGCCATAAAAGGAATGAAATTTTACCATTTGCAACGAT
ATGGATGGACCTGGAGGATGTTATGTTCAGTGAAGTAAGTCAGAGAAAGACAAATACTGAA
TTTTTTAAACTTATTTGTGCAATCTAAAATATACAGCAAACAAAAAATCAAATAAAACAACT
CACAGATACAGAGAATAAACTAGTTATCACTGGGGAGGAAGACAGGGGAGGGCAAGATAG
GTGAAAAGGATGAAGAGTTACAAATGATGGGTATAAAATAAATATAAGCATGTAATATACA
GCACAGGGAGTATAGTCAACATTTTATACTTTATATGGAGTACTCTCTACAGAAATACTGAA
TCACTATGTCATACACCTGAAATTAATATTGTAAGTTAACTGTACTTCAAACAAAAAGATTTT
ACATTGAATTCTAACTGCTTGTTTAATTGCTTCAAATCCTCACTAGTCTTTAAGAAATTGCAG
AGAGGAAACATGACCATTTTATCCCCAGTACTGACTTGAGTGGGCTTTCTAGGTGGCACTAG
TGGTAAAGAACCCGCCTGACAATGCAAGAGATATGAGGTATGTGGGTTTGATCTCTGAATCC
AGAAGACCCCATGCAGGATGGTGGGGCAACCTATTAATGCCTGGAGAATCCCATGGACAGA
GGAGCCTGGCGGGTTGTGGTCCACAGTGTTGCAAAGAATTGGACACAGTTGAAGTGACATA
GCACACATGCACTGACGAGAGTACTTGGCAAAACATAATGGTGAGAGAAAAAATAATATTT
CTGAATTCAGTGGTGAATCTTATTTGGAAACTACTCCCTATGACATTTATATGAGCCATTCTA
AAAATCATAATAAAGTATTGGCTTTATGGCATTTCTAGGCAGATGGGTTCTCTGAATAATTTT
TAAAATGATAATAAATGTAAATGTATTCAGATTTTATTTATGATGGTCACTTTGCAAGTATTT
GTGGGTTTTTAATCTGTTCCACAAAGGTTCCTTGAATCTATCAGTGTCCAGCTATGTGACAGC
AATTGGACATACTCTATTAGAAATGAGGACATCTCCAACTCTATTAAATAACTGTGTATTTAT
GCAAGTCACCGAACCCCAGGGTATTCCATTTTCTTTGTCTGTAAGCCGAAAAGAATTATGTA
CATGATCTCCAAAAAGCTGTTGTTCTAGTCCAACCGTATGTTTTATGTCATATCAATAATACT
GGCTTTCCATAAAAAAGTAGATGAGTAATTAACCATACAGTCAAACTTTAGTATTATTGCAA
GATGGGTTTAATTCTGTTGTGCTGTTTGTATGAAAATAAAAATTAAGCCTCTTCAATTAGGCA
AGTTTCTGACCTTGGGTTTATTTGCCTATTAGATTATGATGACTTAAGGCAGATGGTTTTCTT
TGAATTAAAAAAAATACTCTAAAAAATTCTAAAAACCATGTTCAGTGCTTACAGTATTACTT
ACCAATGTTTAAAGGTAAACAAATCTGTTTTTAGATTAAAAATCATATGTTCAGTGACGGTT
TCCACTCAGTTATATCCTCTTATGAACTATCTTCACGCATGTATGCTCAGTCATGTCTGACTC
TTTGTCATTCTTTAGACTGTAGCCCACAAGGCTCATCTGTCCATAGGATTTTTCAGGCAAGAA
TACTGGTGGGTTACCATCTCCTTCTCCAGGGACTCTTCCCAATCCAGGGATCAAACGTGTCTC
CTGTGTCTGCTGAATTGCAGGCAGTTCTTTACCTGCTAAGCCAGCAGGGAACTATATCGGTT

CTGTATTCCAACCTTTTTTTCTTACATCTTTCTGTGTTACTATTCTTCTAGTCATTTTTAGGTCT
TTTTCTTAAACTACTTTATGTTCTTCAAGTTTTACTTAATGTAGACCCAAATTGCAAAGGCAG
GACAACGAATATCGTTAGTAGAGGCCGTAAAGGAGTTCCTTGACAATATATTAATATTCTGC
TCACATGCATTTTAATACCATCTAAAATTTTTTTTTAGAAATAACAAAACATTGGTGGTGTAA
ATCAATGTGACCTCTACCAGGAGTTTATTAGCAGGCAGAAATGGATGTGGGATTGGCTGCAT
ATAATAATTAGGGGCATGTAGTTTGCTTTAAAACAAAATGAGCAAACAAAAAACAAGATGG
GGGGCAAAGCAACAAAAACAAAACCCATGCCACCATTATGTTTATACCTTTCAATTGTTTTG
ATTTTATAGAGGCACCTATTAAGTTGAAAACTTCCTCATGTCACACTGAGTAATAGGCAGTA
AGTACCACAATGTCAAGAAGAGTTGTAGTTCCAAAGTTTAGAAAAGAAGATCGTGTACACT
GTAGGTCAGTATTAAGGAAATCAGCCTGAGTCAAGAGCAAAGCAGTAGAAAACACCAGAGA
AAGTATAGAATTAGTGACCTTTGGAGGTCTAAATCAAGATATAAAAATCCTGGCAGCTGATT
GGATTAGGCAGTCAGAAATTTTAGAAAGACCAGAACAAGGCAGAGATGGAAGAAAACAGT
AGAATTCAGGAAAGGATGAAGAAGAGGGGTGATCTTAGACACATGAAGTTTAGGAGCGAG
GACCCCATCACAAGAGGTAGGGCCAGAGACCAATGTGAGGGGGAGCCTTTATTTCACTAAG
GAAAGGAGCTGAGTTCAGTGGGTAAGAGCACAGAAGTATTTAAGTTATGAGTGACTGGTGA
ATGTTTCATCACTCCCTGGCAAGTTCACTTTTTGAATTATCACCTATCCACCGGGTAGGAATT
AGGGCTATAAGAAATAGGGCTCTGGGTAGTTACCTTCAATATTTGTAAGTATGCTTTATACT
GGATATTGCTTTGTTAGCCACAAACTGAAGAGTGGGAAATATGTCATATACCCTTATTCTTGT
ATTTAAAGTAGAGTCTGAGAACCCAATCAGTTGAGAGAATTTTGTTGTTATTATGGTTCTCTT
TCTTCCTGTCTAAGTCATGGATAAATCCTAGAGGATTAATCTGCATGAGAGCCCCAGAGAGC
TCATGTCGTTTGTTCTCACTGGTTATTACTAAAATGTCCAATGACTAAGTCTTCAGGGTACCC
TGGGTTTCTGGGACTCTGTTGCTTCTGTTTTGTTTTGGACACAAAAAACCTTTATGGAAAGTG
GGAGTCCTGGAGATACAAGGAGGTTCGGTTCAGTTCAGTTCAGTTCAGTCGCTCAGTCGTAT
CCGACTCTTTGCGACCCCTTGAATCGCAGCACGCCAGGCCTCCCTGTCCATCACCATCTCCCG
GAGTTCACTCAGACTCACCTCCATCAGGTCCGTGATGCCATCCAGCCATCTCATCCTCTGTCA
TCCCCTTCTCCTCCTGCCCCCAATCCCTCCCAGCATCAGAGTCTTTTCCAATGAGTCAACTCT
TCGCATGAGGTGGCCAAAGTACTGGAGCTTCAGCTTTAGCATCATTCCTTCCAAAGAAATCC
CAGGGTTGATCTCCTTCAGAATGGACTGGTTGGATCTCCTTGCAGTCCAAGGGACTCTCAAG
AGTCTTCGCCAACACCACAGTTCAAAAGCATCAGTTCTTCAGCGCTCAGCCTTCTTCACAGTC
CAACTCTCACATCCACACAGGAAAAACCATAGCTTTGACTAGGTGCTCCTGACTTTTTCCATC
GTCTCCCAAAGTCACTTCTCCATGATATCAAGCATAATCTCTTCACATGTGTTTGAACATTTT
AAAAAAGTGTAACATGGAGTTAACTTCACATGCCTTCTATTTACTACAGCTTAAAAAAATCT
TTATGGTAAGAACATTCAACTGGCACAATATGCTTGGAAATAAGGGAAGGAAAAGTGGAGG
TCAGTCACATATACGTAACTCAATTATTTGTCGAGCCATTTTCCCCCAGTAGCAAACTTATGT
CCTCTCAAAGAACACTTGTTTCCTATTCCTGTGTGACTGCATACCATTATCATGGTCACCATC
TCTACTGCCTCCAGAAAGCAGAAACATTTATGTTACCTGTTACCCCATATGGAGATGTCACC
CCCACATAGTGTGACATATACCCAAAACAGTGCCTGCTGACATCTCAAGAACTAGGGAGGT
GAGCTTGGATTGGAAGACAATGTTTGATGATCCCTTACAGGAGCTTACTCTTTTCTCCCACCA
GCAACTCCATCCCATCCAGAGTCTCAGTCTCAGAAGGGTTCTAGGGCCGAACTTATTCTCAG
TGGTATTTGTTCTAAATATAAGCACATCTGACCTAAATGCAATTATATAAAATTTACATTTAT
TATTGGCAATTATAGCATTGAATCTTACTCTACAGCAATACCTTGTCATCTTCCTAGTTGTCC
CCTTTCTTCCACCATACCTGTATCATGATCTCCTGATGTTTTCCTGGATTAAACAAGCTTGGC
CTCTTGATCTCACCCATTTGAGCTCCAGGCTTTCCGGGATGAGAGAAGAATGAGAATTTGGG
GAGCATAATTGCTTAGGACTACAATGCTCTCACTCTAGCTTACCAGAAAATGTTAGCTGCTC
CAAACAATATGACTTTATGCCATCAATAGCTGCTGAGTGTTGCTTTGTTGGGTGGGTGTGGTT
CACCCCAAAATGTTCTCTTTTCCAACAAAGAGCTGTGTTATGCTTGCAAAAGCTCCTTGCAGT
TGCTCTTGGGGGTGTTTCCTCTTTGAGAGGGGCAGGAAAGAACAAAATGCTTTGTTTCCTAA

CTAGTGGGAAGTCCAATCACGCAGCAACACCACTTCTCAGGTCTGAAGGATAGTGCAGAGA
GTTTTAAGAGTATCTTTCACACCACTGTGCTTGGTGCCAGAGGACAAATATCAGAAAATCAG
TCAAGGTGTTTTACAAAGCCAAGGTATTAGGTGTGTCTGACTCTTAAAGATTAAACCTAGTG
CTTGAGAGAAAAATAAATAAATAAATTGGAGTTGAGGAACAAAGTCAAATGAGGTTAAAAT
ATGGACAAATAGGGTGGCTGCTTGGATCAGAATAATTCCAAAGATGTCAGTTAATGGTCAA
GTCTAGATACTTAAAAACTTTCTGATTCACCACAACAATCCTGATGAAGATACCAATCCTCA
CTCTTTTTTTTATCATACAAAAGAAGCCCAAGAATAAGAATATTCTAGATATACTTAGTGCTG
ATGAGATAATTTGGAGTTGAAATAGAGAGTATATACCATTCAGATGCTAATGAAGCTCTAAG
TCCCTAGATGGGCCAAGTCAAAAAGGAAAGGAGATCTGACTGGTAAATATCAGTCTCTAGG
CTTCCTGCTCCTTTGTGGGTTTCCCTGGTGACTCAGATGGTAAAGAGTCTGCCTGTAATGTGG
GAGATCCAGGTTCAGTCCCTGGGTTGGGAAGATCCTCTGGAGAAAGGAATGGCAATCCATTC
CAGTATACTTGCCTGGAGAACTCCATGGACAGAGGAGCCTGATGGGCTATAGCTCATGGGGT
TGCAAGGAGTCACACATAACTGAGTGACTACTCTTTCACTTTCAGGCTTGCTGGCTCCTGTGT
GGGTATAACACTGACCCTACCCCACCACAAAAGGCTACAAGAAGTGCTCCTTTGGGCTTAGA
ATGGTATTTGTGGATGATACCAAGTTTTTTAAAGTGGAAAGCACTATGTAAGCACTTTCATG
TCTGGCACAAATAGACAATCAATATACTTATCTTAGGTATAGGAATATATTAGAGGATATCC
ATAGCAGCTGCCTAAGACCCAGTTAGGACACAATAAGTCTGGGGACTTTAGTTCATTCAAAG
TGATTTTTCTGGTTAAAAATCTAATCCCTGGAAGACAAAATTGTGGCTTCAATTATTGAAGA
AGCAGGGAGCAGCCCCTCCCCACCCTCAGTATAAGGGCAACAAGGCCTACTACAAAGCCAG
TGTGATAAGTGATATAAAACTGAGGAAGCTTCCGTGTCAGAGATTCAGGAGCCCAGTCCTGT
GCCTTGAAAGGGGCAAGGTGAGTGGTGAGCTGAGTGGCTCCAAGAACAAGTGTTGGAGGAA
CATGGGGTCTGAGAGCCAAGGGAACTAACATAGACAAAGAGGATAGAGACCATCACTAGA
ACCTCAAAGTGCTAAGATGCCAATTTAAAAACAGATTTGTCTTATACCAAGATCAGTTCAGT
TCAGTCACTCAGTCATCTCCGACTCTGCGAACCCATGAATCTCAGCACGCCAGGCCTCCCTG
TCCATCACCAACTCCTGGAGTTTACCCACACTCATGTCCATTGAGTCAGTGATGCCATCCAAC
CATCTCATCCTCAGTCGTCCCCTTCTCCTCCTACCCCCAATCCATCCAGGCATCAGGGTCTTT
TCCCATGAGTCAACTCTTCGCATGAAGTGGCCAAAATATTGGAGTTTCAGCTTCAGCATCAG
TCCTTCCAATGAACACCCAGGACTGATCTCCTTTAAAATGGACTGGTTGGATCTCCTTGCAGT
CCAAGGGACTCTCAAGAGTCTTCTCCAACACCACAGTTCAAAAGCATCAGTTCTTCAGTGCT
CAGCTTTCTTCACAGTCCAACTCTCACGTCCATACATGACCACTGGAAAAACCATAGCCTTG
ACTAGACAGACCTTTGTTGGCAAAGTAATGTCTCTGCTTTTGAATATGGTATCTAGGTTGGTC
ATAACTTTCCTTCCAAGGAGTAAGCGTCTTTTAATTTCATGGCTGCAATCACCATCTACAGTA
ATTTTGGAGCCCCCCAAAATAAAGTCTGACACTGTTTCCACTGTTTCCCCATCTATTTCCCAT
GAAGTGATGGGACCAGACCATTTCTGTTCTTTATCAAGCCCATCTTTGCAGGAAATGTTCCCT
TGGTATCTCTAATTTCTTGAAGAGATCTCTAGTCTTTCCCATTCTGTTGTTTTCCTCTATTTCTT
TGCATTGATCGCTGAAGAAGGCTTTCTTATCTCTTCTTGCTATTCTCTGGAACTCTGCGTTCA
GATGCTTATATCTTTCCTTTTCTCCTTTGCTTTTCACCTCTCTTCTTTTCACAGCTATTTGTAAG
GCCTCCCCAGACAGCCATTTTGCTTTTTTGCATTTCTTTTCCATGGGGATGGTCTTGATCCCTG
TCTCCTGTACAATGTCACGAACCTCATTCCATAGTTCATCAGGCACTCTATCTATCAGATCTA
GACCCTTAAATCTATTTCTCACTACTACAAGGAGATCCAACGAGTCCATTCTGAAGGAGATC
AACCCTGGGATTTCTTTGGAAGGAATGATGCTAAAGCTGAAACTCCAGTACTTTGGCCACCT
CATGAGAAGAGTTGACTCATTGGAAAAAACTCTGATGCTGGGAGAGATTGAGGGCAGGAGG
AGAAGGGGACAACAGAGGATGAGATGGCTGGATGGCATCACGGACTCGATGGACGTGAGTC
TGAGTGAACTCCTGAAGATAGTGATGGACAGGGAGGCCTGGCGTGCTGCAATTCATGGGGT
CGCAAAGAGTCAGACACGACTGAGCAACTGAACTGAACTGAACTGATGGGACCAGATGCCA
TGATCTTCGTTTTCTGAATGTTGAGCTTTAAGCCAACGTTTTCACTCTCCTCTTTCACTTTCAT
CAAGAGGCTTTTTAGCTCCTCTTCACTTTTGCCATAAAGGTGGTGTCATCTGCATATCTGAGG

TGATCGATATTTCTCCCGGCAATCTTGATTCCAACTTGTGCTTCTTCCAGCCCAGCGCTTCTC
ATGATGTACTCTGCATAGAAGTTAAATAAGCAGGGTGACAATATACAGCCTTGACGTACTCC
TTTTCCTATTTGGAATCAGTCTCTTGTTCCATGTCCAGTTCTAACTATTGCTTCCTGACCTGCA
TATAGGATTCTCAAGAGGCAGGTCAGGTAGTCTGGTATTCCCATCTCTTTCAGAATTTTCCAC
AGTTTATTGTGATCCACACAGTCAAAGGCTTTGGCATAGTCAATAAAGCATAAATAGATGTT
TTTCTGGAACTCTCTTTCTTTTTTGATGATCCAGTGGAATCTAAAAAGGATTGTAATTTTCATT
ACTAATCAGAACAACTTGACACCAACAGAAACATACACACACAAACACCCCCCCCCCACAC
ACACACACACAAAACTGTGGCAGGTCTCCTTCTGATAACAATTCCTTATAAGGTTTTTTGTCC
CTATCTAACAGGAGAGTGAAAAAGTTGGTTTAAAACTTTCAACATTCAGAAAACGAAGATC
ATGGCATCTGGTCCCATCACTTCATGGGAAATAGATGGGAAACAGTGAAACAGTGTCAGAC
TATTTTGTGGGGCTCCAAAATCACTGCAGATGGTGATTGCAGCCATGAAATTAAAAGACGCT
TACTCCTTGGAAGGAAAGCTATGACCAACCTAGATAGCTTATTGAAAAGCAGAGACATTACT
TTGCCAACAAAGGTCTGTCTAGTCAAGGCTATGGTTTTTCCAGTGGTCATGTATGGACGTGA
GAGTTGGACTGTGAAGAAAGCTGAGCGCCAAAGAACTGATGCTTTTGAACTGTGGTGTTGG
AGAAGACTCTTGAGAGTCCCTTGGACTGCAAGGAGATCCAACCAGTCCATTTTAAAGGAGAT
CAGTCCTAGGTGTTCATTGGAAGGAATGATGCTAAAGCTGAAACTCCAGTACTTTGGCCACC
TCATGCGAAGAGTTGACTCATGGGAAAAGACCCTGATGCAGGGAGGGATTGGGGGCAGGAG
GAGAAGGGGATGACAGAGGCTGAGATGGCTGGATGGCATCACCGACTCGATGTATGTGAGT
TTGAGTGAACTCCAGGAGTTGGTGATGGACAAGGAGGCCTGGTGTTCTGAGATTCATGGGGT
TGCAAAGAGTTGAACACGACTGAGTGACTGAACTGAACTGAAATAGGAAGGTGATAACATT
ACTCTATAAAAACAGAACCATCTTGCTGCAACCCACATATTCAAAGAATTAAAAAGCAAAG
CTTCCTATATCTTTATTTTTGTAGTAATTAGGATTTGGAATTTATAAATATAGGCTCACTGTA
ATCTCAAGTGTGGTCTCTTTTGCATCACTTCATTCAGGTCAGATTTTTATAAAATTTGTTATTC
CTGAATTCATGGTGTTTATATGACTGTTATTGAATGTAATTTTCATTGGAAATTTTTATGAAG
AGTTATGATTTGCTTTTATTAAAGGGAGGATTGATCCACTATCCAGAATAGCTTTCTATAAAA
TATGAATTATGGTGAGAATTTTGGACAATGAGATATATAAACTAGCAAATAGGAGATTTAAG
ATTTTTCAGAATGTAGGAAAAAAACACAAAAAACTAACCCTGAAGCCAAGAAGAGGTCCAT
ATAGTGATGTATCAGCAGGTCAGCTCAGTTTCTCAGTGGTGTACAACCCTTTGCAACCTCAT
GGACTGCAGCACACCAGGCCTCCATGTCAATCACCAAATCGTGGACCTTGCTCAAACTCATG
TCCATCGAGTCGGTGATGCCATCCAACTGTCTCATCCTCTGTCATCCCCTTCTCTTCCTGCCCC
CAATCCCTCCCAGCATCAAGGTCTTTTCCAATGAGTCAGTTCTTCACATGAGGTGGCCAAAG
TGCTGGATCTTCAGCTTCAGCATCAGTCCTTCCAATGAATATTCAGAACTGATTTCCTTTAGG
ATTGACTGGTTGGATCTCCTTGCAGTCCAAGAGACTCTCAAAAGCCTTCTCCAACACCACAG
TTCAAAAGCATCAATTCTTCAGCACTCAGATCTTTTTTATAGTCCAACGCTCACATTCATAGA
TGACTACTGGACAAACAATAGCTTTGACTAGATGTACCTTTGTTGGCAAAGTAATGTCTCTA
CTTTTTAATATGCTATCTAGGTTGGTCATAACTTTTCTTCCAAGGAGCAGATATCTTTTAATTT
CATGGCTGTAGTCACCATCTGCAGTGATTTTGGAGCCCCCCCCCCAAATAAAGTCTGAAACT
GTTTCCATGTTTCCCCATCTATTTCCCAGGAAGTGATGGGATCAGATGGCATGATCTTAGTTT
TTTGGATGTTGAATTTTAAGCCAACTTTTTACTCTCCTCTTTCACTTTCATCAGGGGCTCTTTA
GTTCTTCTTCACTTTCTTCCATAAAGGTGGTAGCATCTGCTTATCTGAGGTTATTGATATTTCT
CCTGGCAATCTTGATTCCAGCTTGTGCTTCATCCAGCCCAGCATTTCACATGATGTACTGTGC
GTATAAGTTAAATAACTAGGGTGACAATACACAGCCTTGATGTACTCCTTTCCCAATTTAGA
ACCAGTCTGTTGTTCCATGTTCAGTTATAATTGTTGCTCCTTGACTTGCACACAGATTTCTCA
GGAGGCAGATAAGTTGGTCTGGTATTCTCATCTCTTTAAGAATTTCCACAGTTTGTTGTGATC
CACACAGTCAAAGACTTTGGTATAAACAATAAAGTACAAGTAGACGTTTTTCTGGAACTCTC
GTTTTCAATGATCTAATGGATGTTTGCAATTTAATTCTCTGGTTCCTCTGCCTTTTCTAAATCC
AGCTTGCACATCTGGAAGTTCATGCTCATGTACTGTTGAAACCTGGCTTGGAGAATTTTGAG

CATTAATTTGCTAGCATGTGAGATGAGTGCAAATGTGTGGTAGTTTGAGCATTCTTTGTCATT
GCCTTATTTGGGATTGGAATGAAATCTGACATTTTCCAGTCCTGTGGCCACTACTGAGTTTTT
CAGATTAGCTGGAATATTGAGTGCAGCACTTTTATAGCATTATCTTTTAGGATTTGAAATATC
TAAACTGCAATTCCATCACCTCCACTAGCTTTGCTCATAGTGATGCTTCTTAAGGCTCACTTG
ATTTGGCATTCCAGGATGTCTGGCTCTAGGCCAGTGATCACACCATCGTGGTTACCTGGGTC
ATGAAGATCTTTTTTATATAGTTCTTCTGTTTATTCTTGCCACTTCTTCTTAATATCTTCTGCTT
CCATTAGGTCCATACCATTTCTGTCCTTTACTTGATATGTGAAATACATGATGTATGGTATCA
CATATTTGAAATATGTGATATATTTAAAGGCTAAAAACATGGTAAGATGAAAGAAAGGGCT
AAGATGAGATTTATTAAACAAAGAACTTAAAATCCATAAGGTAGATTTAAGTTACTCTGAAT
GCACTTTATTGATCCTTCTATGTTTCTTTTAATGTCAATTTTTCTATAATTGTATTGGGTATTG
TATATAATTATGACTAAGTTCAAAGAATTGAATTCCAGAGGCCCTCTGTTTTCCCAAAAATA
ATTTATGTCTTAACAGTTCTAATTTGGAAGACTTAAATCATCATCTATTATAGTTTATTGAAT
ATAATGACCAGCAATGAATGTTTGTTGTATGAAGAAACTTGAAATCAATAACATTAAAAATA
TTTTTTGAAAAGAATGCAAAGATGGTTGCTGATTTACTGTTTTCTACCAATAATACTTTAATC
TGTAAAAGTTTTTTAGTTTCCTAGATATAATCCTACTCACCCTGTTAGACTCAACTCAATGTT
ACTTCCTAAAAGAAGCCCTCTTCGCGTCCCCAAACAGTTTATCCTGATCCTTAGAGCACCCTG
TGCACATATCTAACTCTCTACTTCTCATACCATCCAATAGTAATGCTGCCCTATCTCAATTCT
TATCTTCCTTACCAGACTTTGAACTTCTTAAGGGCCTGGGATACATGTATTACATCTTCCATA
TCTGGAATATAGAATGCACTTAATCAATCTGTCTAAAATAAATGTGCCCATTTCTTTTTCACT
TCACTTGCAGTCATGTTTTCCATCTTTCTCTGTCCTGTCCTCTGTTTCCTAAGCACCTTTCTCC
CTTGGCCTTGATATTGAGTATGTTTCATCATGTTTTCAACAGAGCCAGTCAGAGTTTCTCACA
ATCATCTCTGCCATAAGTATGTGGGCTAAGGTGCCAGGGTGTCAGGAGGCGGGCCAGCACA
TTGCATAAGTGCCTGGATCATGTAAGCAGATACTGTGACATGGGACAAAGTGAGCAGGGGA
GCCAAAGAAGTAAGACAAAGTCAAGGGGAAAGGAGAAAACCACAGAGCTGCTGCCGCGGT
GCACTAGAAGCAGGGGAGGCTGTCGTATCCTTCTGCAGCCATGGTGTCAGAGAGTTTACTGA
TAAAGAGAAGTTTAAATAAAATCCGGAGTCTAAGTACGAAAAGAGTTATTGGGCCACTTGA
TTGATGATCAAAGACAAGAAGTAGACATTTTCTTTGCACCAGAGAGCTTGTGGTCTCCCTAG
TAAAGCTGTGGTGGAGGCCTGGTTTGCTCACTAATACATGTTTTTATTTCATTTTTCTCTGAA
ATTTGTTACACGTCTTGTTCTGAGAGATTATAAGAGAAAGGAAAAGTTTTAGAATATATTTT
CAGGCAATCTGTGAAATGTCATTTGTTTTCAAATTTTGAAAGGAAAGGGAATTCACTTCATA
GTGCATTTCCCTATTTGAGGCACTATCATTTTCTGCTTACCCTCAAATGATTGTTTTCTAGTGC
TTTTGCATATTTGGCTAAAAAATAGATAACCAGTACACTCTATATACAGCAAAGATGAGAAA
ATGATTGTGGTAAGGGATGCCTGGGGAAGCCACTATGTCTCTACTTGTCCCTATGCAGGAGC
CTTAAGATGGTATTTTCTCAAATCATCTTGTTTAACCCTTCCTGTCAGAAAATCTAATGTCAC
CATTTATCTGTACCCCAGATTTTCTAAATAGCTTTAACATTACAAAAAGAAACATTGTGGAG
CAGGTATATTAGAAATGTCTGGATGTCAGAAAATTTAGGATCTGAGCTCATTCTGTTTGTTA
ATGGCTTGATTTCTCTTGGGAGGAAATGTCTTCCCTTTTTACTTACAAGATCTCCCAAGTGAA
GATGGTTGTTCTTAAGTCCTTCCGCCTTCTCTTTCAATCAAAGTTCTTGGACTTAAAACTGAT
TTCTCCCTCAAGGGAAATGTATGTACATATGGAAGATGTAAATGATTTTGGAGAAAAGGGTA
CAAGATAAAAGGCTACACAGGCTAAGAAGGCAATAATTAGGCATGCTATTGCCTGTAAAAG
TCCTTAAGGAATCTTCTTTTGCAAGATAAACTTATACTTACTACTTGCTTCACAAGAGAAGTC
ACCACAATGAGAACCCCATACACCATAACTAAGAGTAAGCCCCAGTCACCACAACCACAGG
CAGCAATGAAGACTCAGCACAAAGAAAAATGAAATAAATAAATAAATAGGTTTTTTTTTTTT
TTTAAGAATTGCTTGACATTGGAGAGAATTCTGAGATCCAAGGGGAAAGAGTTTAGTTTGAT
CTTAAAACTAAACTTTAAGATTGCTTAATTGGGAAATAGGAAATTCTTATATATGAAGGAGA
CTCATTGTTCATGCTTCTGATAGAACAGAGGAACAGGATACAATCAAACTATTGATTTATAA
TTGTTTATTCCCTTAAAGTATTGATGAATCTCTGCATTCAAAGATATTATTGTGCACCCACTA

TGTTCTATATGTCAAGAGGCAACTTGTTGGAAAAGACTCTGTTGCTGGGAGAAAGTGAAGGC
AGGAGAAGGGGATGACAGAGGATGAGATGGTTAGATGGTATCACTGACTCAATGGACATGA
GCTTGAGCAAACTCCAGGAGATGGTGAAGGACAGGGAAGCCTGGCATGCTGCAGTCCATGC
AGCTGCTAAGAGTTGGACACAACTGAGCAACTAAAACACAACAAATTTTATAGAACAGTGA
TCAGAATAAGGCCACGATCACATTGTTCCAGAGGCTTATAATTTAGTGGAAAATTATAATTA
ATAACACAACACAGCATTTATTAATTTTCAAATGAGTGTCACTTGACAGGAAATTCTCTAGG
AATTCATCAGAAGAAACAATCTCTGAATAGAGAGACTCATAAATTCTCTCTGCTTCCTGGTG
GTATTTTGTCCAGCTTCATGGGAAAATGAACGTTCAACTTAATGTAATTCTTCCCCAAGGAG
AAGCAGCAGCTGTTTATATAGAATTTCTTTTGGCTTTACTATTACCATTAGCAACGGTTATTT
TTGATCTGTAACATGGCTGGTAGCAGCAACAAACAAATGAAGACATTAGTAATATTCTCTAA
GCATATTTCATTTACATCTTTAAAAAACATACCTTTTAAGCCACAATGCAAAATTACTAGAA
GTTTCTATCGTTGCTGCTCAGTCATGAAGTCATGTCCCACTCTTTGTGACCCCATGGACTGTG
GCATGCCAGGCTCTTCTGTCCTTTACTATCCCCCTGAGTTTGCTCAAATTCATGTCCATTGAG
TCAAAGATGCTATTTAACCATCTCATCCTCTGCTGCCCCCTTCTCCTTTTGCTTTCAATCTTTC
CCAGCATCAGGGTCTTTACCAATGAATCAGCTCTTTGCATCAGATGGCCAAAGTATTGCAGC
TTGAGCATCAGTCCTTCAATGAACATTCAGAGTTGATTTCCTTTAAGGTTGACTGGGTTGATC
TCTTTGAAGTCCAAGGGACTCTCAAGAGTCTTCTCCAGCACCACAGTATGAAAGCATTGGCT
TTCAGCCTGCTTTGGATGCTTACGTTATGGTCCAACTCTCACATCCACACATGACTACTGGAA
AAAACATAGCTTTGACTATATGGGCCTTTGTCAGCAAAGTGATGTCACTGCTTTTTAATACAC
TGTCTAGTTTTGTCATAGCTTTCCTTCCAAGAAGCAAGTATTTTTTGATTTTATGGCTGAAGT
CACCATCTATAAATATTTTTGGAGACCAAGAAAATAAAATCTGTCACTACTTGGAATTTTTTC
CAGTTGCCCTGAAGTGATGGGACTGGATGCTGTGATCCTAGTTTTTTGAATGTTGAGTTTTAA
GCCAGCCTAAAACGCTCCTCTTTCACCCTCATAAAGAGGCTTTTCAGTTCCTCTTCACTTTCT
GTCATTAGAGTAGTATCATCTGCATATCTGAGGTAGTTGATATTTCTCCTAGGAGTCTTGATT
TGAGCTTGTGATTCATCTAGCCTGGCATTTTGCATGATGTACTCTGTACAGAAGTTAAATAAG
CAAGGTGACAATATATAGCCTTGTTGTACTCCTTTCCCAATTTTTAATCAGTCTGGTTCCATG
TCTGGCTCTAAGTGTTGCTTCTTGACCCACATACAGGTTTTTCAGGAGGCAGGTAAGGTGGT
CTGGTATTCCCCTATCTAAGATTTTTCCACAGTTTGTTGTGATCCACACAACATTAAAGGCTT
TAGTGTAGTCAAAGAAACAGAAATAGATGTTTTTCTTGAACTCCTTACTTTTGGCATGACCC
AACAAATGTCAATTTGATCTCTGATTCCTCTGCCTTTTTTCTAAATCCAGCTTGTATATCTGG
AAGTTCCCGGTTCATGTACTGCTGAAACCATACTTGAAGAATTTTAAGCATAACCTTGGTAG
ACTGTGAAATGAGCACAATTTTTCATACCAAATGTACAAAGTTTGAACATCCTTTGGCATTG
CTCTTCTTTGGGACTGGAATGAAAACTGAACTTTTCCAGTGCTGTGGCCACTGCTGAGTTTTC
CAAGTTTGTAGCATGTATAGTGCAGCAATTTAACAGCTTCATCTTTTAGGATTTTAAATAGCT
CAGCTGGAATTCTGTCACTTCCACTAGCTCTGTTTGTAGTAATGGTTCTAAGTCCCACTTGAC
TTCATGTCCCAGGATCTCTAGCTCTAGGTAAGTGATGACACCATCGTGGTTATCTAGATCCTA
AAGACCTTTATTATACAGTTCTTCTGTGTATTTTTTGCCATCTCTTCTTAATCTCTTTCTCCTTT
ATCATTCCCATCCATGCATGATGTGTTTCTTTGATATCTCCAAGTTACTTGAAGAGATCTCTA
GTCTTACCCATTCTACTGTTTTCCTATATTTCTTTGCATTGTTCATTTAAGAAGGTCTTCTTAT
CTCCTTGCTATACTCTGGATCTCTACATTCAGTGGGGTGTATCTTTCCCTTTCTCCTTTGCCTT
TCACTTCTCTTCTTTCTTCAGCTCTTTGTCTGAGAGCCTCCTCAGATAAATACTTTACCTTCTT
GCATTTCTTTTCCTTTGGGATGGTTTTGGTCACTGACTCCTGTACAAAGTTACAAACCTCCAT
CCATAGTTCTTCAGGCACTCTGTCTATCAGATCTGATCCCTTGAATCTGTTTGTCACTTCCACT
GCATAATCATAAAGGATTTGATTTAGGTCATACTTTAATGGCCTAGTGGTTTTCCTTTCTTTA
TTCAATTTAAGTCTGATTTTTGTAATAAAGAGGTCATGATTTGAGCCAAGTCAGCTCCAGGTC
TTGTTTTTACTGACTATGTAGAACTTCTCTCTTTAGCTACAAAGAACTTAGTCTGATTTCAGT
ATTGAGCATTTGGTGCTGTCCATGTATAGAGTTGTCTCTTGGGTTGTGGGGAGAGGGTGTTTG

CTATGACCAGCATGTTCTTTTCACAAAACTGTTAGCCTCTGCCCTGCTTCATTTGTACTCCAA
GGCCAAACTTGCCTGTTACTCCAGGTATCTCTGGACTTCCTACTACTTCATTTTAATCCCCAG
TGATAAAAAGGACATCTTTTTTGGTGTTAATTCTAGAAGGTGTTGTAGGTCTTCACAGAAAC
AGTTTACTTCAGTTTCTTTGGCATCAGTGGTTTGGGCATAGACTTGGATTACTGTGATGTTGA
ATGGTTTGCCTTGGAAACAAACTTAGATCATTCGGTCATTTTTGAGGTTGCACTCAAGTACTG
CATTTCAGACTCTTGTTGACTGTGAGAGGTATTCCAATTCTTCTAAGTGATTTTTTGCCCCCA
CAGTAGTGACATAATGGTCATCTGAATTAAATTCACCTATTCTCATTCACTTTAGTTCTTTGA
TTTCCTAAGATGTTGATATACACTCTTGCCATCTCCAGCTTAATCATGTCCAATTTATCTTGAT
TCATGGACTTTATATTTCAGGTTCCTTGGCAATATTGTTCTTACAGTATCAGATTTACCTTTCA
CTACCAGACACCTCCACAACCGAGTGTGGATTACACTGTGGCCCAGCCACTTCATTCTTTACT
GGAGCTACTAGTAATTGCCCTCTGCTCTTTCACAGTAGCATATTGGACACCTTCTAACCTGGG
GTACTCATCTTGCAGCATCATATTTCTTTGCCTTTTCATACTGTCTATGAGCTTCTCCAGGCAA
TACAGGAGTGGGTTGCCATTCCCTCCTCCAGTGGGCCACGTTTTGTCTGAACTCTTCACTATG
ACCTGTCTGTCTTGGGTGGGCCTGCATGGCTCATAGCTTCATTGAGTTAGACAAGCCCCTGC
AGCACAACAAGGCTGCAATCCATGAAGGTGGAAGTTTCTATTACTCCTCTGTGTAAATGTGA
AAAATTTTCTTAAAAAGGAAACTCTGACATCTCCTAAGAAATATAGAACAACATTAATCCTA
TTGATAAACTAAAACAAGTGAAAGTGTATCTAGGACGTAGGTTTTTGGTGATAGAAAAAAG
TAATTGCTTTGATAATATATCTTGATCATTCTCTTACATTGCAGTTGTACAAACACCTTTAAA
AGTTTTATTAAGCCACCAAAACAGTCCACCTAATTGTCCTTCCTAAGGGAAACCATAATAGA
AAAGCACATATCATGCTGTTTGTAAAGCAGCTATGAAAGAACTGCAAAAGAAAGTTTGTGA
ATTCATAGAATGATCAATGGGAAGTTAGATGATCATGACAATTTTCATTAACTCCATGAAAT
TATTCAAAGGCATACACTGAGCTACCTCACATAATGTAGAGTTGTAAGTATCTTTTCTAATTT
TTATGTACCCTATCTAAATAATGCTGCTTAGCAATTCTAAAGAATATCTAAGTATTGCTTAGT
AATTCGAGAAAAATGACACTAGTGTTGAGACTTTCCTCAGGATATTTTCAAGCTCCTTGCAG
ATTAGGCTTCTGAACAAGTTACTTATTTTCTCTAGCTTTTTCCTATGAATTTAGGAATGAAAT
ACTCAGCGTGGTAAAAGGAGGAAGGGGATCTGGAAATCTGATTTAGTTGTATGGTCTGGAA
AGATTTTCAAAGATGGGGAAGGGAAACAACTGAGTGACTGAACGAACTGAAAAGAAGATG
GAGAGTCACTGGTCACATACACTTTTTGTTCTTAAGGCTTGTTTGGTGCTCATTGGTATTTGG
GAGGATTTCTAATGCCATCTGGTAAGTGCAGCTAGTTCACTCACAGAGCCATGGCTCAAAAA
TGTTATTGGGTGACTTTTGTCCTCGGAGTATGCCCAGCCCTCCTTTTAATGGCTCTTGTCACCT
TGATAGCACACTAATCTCTCAATGGCCTATTTGGTACCCAGGAACATGTTTTCTTACATGGCT
CATTGAATTAATTTGTCCAGTAAATTTTCATAGAAAGCTTTCAAGAGTGAAAGCCTCTGTGCT
AAATGAGGGTAAACTCATTAATGCTGGCAAAAAAACTGAGCCAAACCTCCTTCCCTATAAAT
AGGGAATTTATATTAGAAAGTGACAAACCCCTATTTGATATCAAGGTTATATTATCTCTTTGA
TCCTGCAGATTTATTAACACTGTAACTGCCATACTGTCATGAAATAAGTCTGATCAATTTCTT
CTGACTCGTGGACTGGCGTGAATACAAAACCAACATACAATTCATAACTATTTTTTCCTGAT
GACTCCAGACCTGATCTTTGTGACTTTAATCTAATCTCTGACCTAGCTTCCAGTTCCCAGTAT
TTACATATCTGTTTATTTTATATGTTTCCTCCATGTTTCCCTTTCTTTTCATGAAGGCCTTTTGG
ACTGAAGATCTAAAAGTCATTGCTGTTCAAGTAGCAAAATCTCATAAACACTGAAGCTGGGG
GATGGTCATACAGGGTCATTATACCTTTATACCTTTATTTTCCACTTCTGAAAAGTTCAAGAG
AAGGTCAAAAGACAAAGCCACTTTCCTATATTGTGCATGGGAATCAGATGGGTATGAAATTG
TTTAATTTTACAGGAAGAGAGAAGTTTAGAGAAAGACTCACAGGCCTAACTGTACATCAGA
AGGAAAAAACAAAACAAAGGTTCTCTGCTTTAAGAAGTTTGTGTGCAATTAAGTTTTTATAT
GGAAGACTATCAATCTCTAGTGATTTCTGTTTCCTTGAGGGGGTGATACCATGTAAGTCTTGA
TCAACAGACCTTGAAATCTTACTGATAAATTCACCAATTCCTAGGCTGTGATCATTATTGTTA
ACTTAAATTTAAAATCCCACAGGTCTGGGTATCTTTTTTTAACATATCTCTAACATTAGGTGC
CAGGTGTTTCCTCTAGATCTGGGTAGGGCTTATCTGCTTCCCTTGTTTCTGTTATTTGGTATTA

GTTATTTTTGTTGGTGATGGACAGGGATGCCTGGCATGCTGCAGTCCATGGGGTCACAAAGA
GTCAGACATGAATGAGTGACTGAACTGAACTGTTTCCTTCCACGAGGTTGTTTAGAGACATT
GCAGTATAGAGTCCTTGAGCTGGAAGCTCAAAATAGTGTCCCAGGGACAAGGAAGGAAGCT
CTAAGAGAAAAGTGTGGTAGGAAAGGAAAGGAAAAGAAAAAAAGAAAGAAACAAGCAGA
GTGTATGGCTATAACAGATGAGGCTAAGAAAAAAATAGACAACAGTCTTACAACTTGTTTA
ATTATAATCTTAATGTAGAAGGAAAAAAAAAAATAGGGAAATATTCCAGTTTGTATTCTTGGG
TGAATACAACTTTTAATAACTTTTAATACTTTTTTTAAGGATATGACTATTTCCACAGCTTAA
TATTTAATGAATATATTAAATATATCCTTTATATTTAAATTATATATACTAAAAATATCATTT
AATGGGAAAAAAAAACTATGGACAGTGTCATTCACTATGCTTCTTTGATGTAAATTTTGATA
GATGTGATCAAATATATGTACCATCAAAATGCAGTCAGGATTATATTAGCTGCTATAAAAGC
CAAATTCCTCAGGTCCTATAAGTTATCATCTTTCTGCTGTTTTACAATTGATGAAATTAACGA
TTAGAGAGGGTAAGTGGCCTATACTCAGACATCTTCAAGTGGGAGAGTCACATTCCCATTAT
AAACATGCCCAAACCTCCTGATTCAGAACTGTTTCATTGTCAACGGCTCCCATTACATGGAA
AGCATTTTTGGTTCTTTTGACAATACAATAACATTTAAGAGTATATATTCTGTTCCCACCCGC
ACCCTCCCCCACCAGCTTTTCAAAAGTCTCCTACAGAAGATGTCAATAAATCTCAACAGAGT
TGGCATTTAATTTTCCAAAAGTGTTGAATGCTTGAATCTTATATTTTATCTAAATCTCATTGC
AAGTGGTTACACAATGTTTTTTGTCTAAAAAACTACATTATTTCTTAAGAACTCTCTAGACTT
AGAACCAGGACATTTCCAAAAAAAAAAAAAACTTTTTTTTTTTTTAAAATAGATGCTGTATTT
TAGGGATGAAGGCTAACTTCTTTGAAGTCTAACTGAAACTATGTTAAGGAATCATGTAACTA
GGAAACAGTCATTAAATGTTGTTTGTTATGCATAAATGCCAATTCACAACTCAGAGGAAACT
GATTTATTTGCCATCAAGTAACTTTATAGACATAACACAAGTTGTATGTCTATGCCTAAACCA
GTAACGCTGAAGAACCTGAAGTTGAATGGTTCTATGAAGACCTATAAGACCTTTTAGAACTA
ACACCCAAAAAAGATGTCCTTTTCATTATAGGGGATTGGAATGCAAAAGAACGAAGTCAAG
AAACACCTGGAGTACCAGGCAAATTTGGCCTTGGAATGCAGAATGAAGCAGGGAAAAGACT
AATAGAGTTTTGCCAAGAAAATGCACTGGTCATAGCAAACACCCTCTTCAACAACACAAGA
GATGACTCTACACATGGACATCACCAGATGGTCAACACTGAAATCAGATTGATTATATTCTT
TGCAGCCAAAGATGGAGAAGCTCTATACAGTCAACAAAAGCAAGACCAGGAGCTGACTGTG
GCTCAGATCATGAACTCCTTATTAGCAAATTCAGACTCAAATTGAAGAAAGTAGGGAAAAC
CGCTAGACCATTCAGGTATGACCTAAATCAAATCCCTTATGATTATACAGTGGAAGTGAGAA
ATAGATTTAAGGGACTAGATCTGATAGTTAAGAGTGCCTGAAGAACTATGGAATGAGGTTC
GTGACATTGTACAGGAGACAAGGATCAAGACCATCCGCATGGAAAAGAAATACAAAAAAG
CAAAATGGCTGTCTGGGGAGGCCTTACAAATGGCTGTGACAAGAAGAGAGGCAAAAAGCAA
AGGAGAAAAGGAAAGATATAAGCATCTGAATGCAGAGTTCCAGAGAACAGCAAGAAGAGA
TAAGAAAGCCTTCTTCAGCAATCAATGCAAAGAAATAGAGGAAAAGAACACAATGGGAAA
GACTAGAGATCTCTTCAAGAAAATTAGAGATACCAAGGGAACATTTCATGCAAAGATGGGC
TTGATAAAGGACAGAAATGGTCTGGAACTAAGAGAAGCAGAAGATATTAAGAAGAGGTGG
CAAGAATACACAGAAGAACTCTACAAAAAAGATCTTCATGACCCAGATAATCATGATGATG
TGATCACTCATCTAGAGCCAGACATCCTGGAATGTGAAGTCAAGTGGGTCTTAGAAAGCCTC
ACTACAAACAAAGCTAGTGGAGGTGACGGAATTCCAATTGAGCTCATTCAAATCCTGAAAG
ATGATGCTGTGAAAGTGCTGCACTCAATATGCCAGCAAATTTGGAAAACTCAGCAGTGGCCA
CAGGACTGGAAAAGGTCAGTTTTCATTCCAATGCCAAAGAAAGGCAATGCCAAAGAATGCT
CAAACTACCGCACAATTGCACTCATCTCACATGCTAGTAAAGTAATGCTCAAAATTCTCCAA
GCCAGGCTTCAGCAATACGGGAACTGTGAACTTCCTGATGTTCAAGCTGGTTTTAGAAAAGG
CAGAGGAACCAGAGATCAAATTGCCAACATCCGCTGGATCATGGAAAAGCAAGAGAGTTCC
AGAAAAAACATCTATTTCTGCCTTATTGACTATGCCAAAGCCTTTGACTGTGTGGATCATAAT
AAACTGTGGAAAATTCTGAAAGAGACGGGAATACCAGACCACCTGACCTGCCTCTTGAGAA
ATCTGTATGCAGTTCAGGAAACAGCAGTTAGAACTGGACATGGAACAACAGACTGGTTCCA

AATAGGAAAAGGAGTACGTCAAGGCTGGATATTGTCACCCTGCTTATTTAACTTCTATACAG
AGTACATCTTGAGAAACGCTGGGCTGGAAGAAGCACAAGCTGGAATCAAGATTGCTGGGAG
AAATATCAATCACCTCAGATAAGCAGATGACACCACCCTTATGGCAGAAAGTGAAGAGGAA
CTCAAAAGCCTCTTGATGAAAGTGAAAAGGAGAGTGAAAAAGTTGGCTTAAAGCTCAACAT
TCAGAAAATGAAGATCATGGCATCTGGTCCCATCACTTCATGGGAAATAGATGGGGAAACA
GTGGAAACAGTGTCAGACTTTATTTTTTTGGGATCCCAAATCACTGCAGATGGTGACTGCAG
CCATGAAATTAAATATGCTTACTCCTTGGAGGAAAAGTTATGACCAACCTAGATAGCATATT
CAAAAGCAGAGACATTACTTTGCCGACTAAGGTCTGTCTAGTCAAGGCTATGGTTTTTCCTCT
TGTTATGTATGGATGTCAGAGTTGGACTGTGAAGAAACCTGAGCACTGAAGAACTGATGTTT
TTGAACTGTGGTGTTGGAGAAGACTCTTGAGAGTCCCTTGGACTGCAAGGAGATCCAACCAA
TCCATTCTGAGGTTCAGCCCTGGGATTTCTTTGGAAGGAATGATGCTAAAGCTGAAACTCCA
GTGCTTCAGCCACCCCATGCGAAGAGTTGACTCATTGGAAAAGACTCTGATGCAGGGAGGG
ATTGGGGGCAGGAGGAGAAGGGAACGACAGAGGATGAGATGGGTGGATGGCATCACGGAC
TCGATGGACGTGAGTCTGAGTGAACTCCGGGAGTTGGTGATGGACAGGGAGGCCTGGCATA
CTGCGATTCATGGGGTCGCAGAGTCAGACAGGACTGAGCTACTGTACTGAACACAAGTTGTA
AACAAATTCTTAAAATATTTTTAAATACTTATATCTCTTTTGAAATGCATTTTAATAGGTGAT
AAATGAATTCAGGGATCTATCTAGACTTTATTTTTAAAACCAAACTGTACACAGGCTGATCA
TTTGAAGTAGCATCTGATTGTTAAGGGAGAAGACCAAGAGAAGGGAAAGGCTATCCACTGT
AGAATTCTGGCACAGAGCATTCCTTGGGCTATATAGTCCATGGGGTCATAAAGAATTGTACA
CGATTGAGCGACTTTCACTTAAAGAGCTCACTGAAAGTTTCTCACTCAAAAATCTAGAAAGA
CCTATTTGCTGATGGCATGAGCCTAAGCAAAAATCCTACGAATAAGATGAGCTCAGTATTCC
AGGCAAAACACCTATATGTACACTGCATTGGGTGATGAAGCTTGAGGCTAAAATCAAAGAA
GTTTTCAGAGTGGGATGAGAGAATGTCATGAACACAGATTGGAAATGAGCAAATAGGACTG
AGGTTCACAGACTGTATAGCTGAGCAGCAGAGTTCAGATGCCAGCAATGTAGACTAATTTCT
CACATATTCTAAATTTAAATCTATGCTAGTAATTAGATACAGTGACTTAGCAAAATTCTTTTG
CTTTCTACTGGACTTTTTTTTTTAATTCCATTGGAATAATAACCTATGTCTTCAATGAAGGCCT
CAGACCCTCTAACAACCCTCAACCTCCACCCACACACAAATCAGATTATTTTAAACTGTCAG
TGCAGGAGATGCAAAGACATGGATTTGATCCCTGGGTCGCGAAGATCCCCTGGAGTAGGAA
ATGGCAACCCACTCCAGTATTCTCACCTGGGAAATCCTATGGACAGAGGAGCCTGGCAGACT
ACAGTCCACAGGTCACAAAGAGTCAGACATGACTGAACAAGGTACTTAAAAGGAAAGAAAT
TACATTATTGCATGTTCAGCATTTTCTCAGCAGCTGCTTGCTGGCAGGTTTGTGACACTTACA
CCTGGCAAACCACATAAAGTTTTTAATTTCTGGTGTCTTCAGTCACTGAAAAATCCAGGATA
GGTCCCCACTGGGTGTGTCGCCACTCTCTGCGCTAGCTGTTGAAATACTGAGTGTTAAGGGA
CCAGACCTGAGCCATGACAGGCTCAACAAGCTATGCTAGGCCTAAGATTCAGATTCTTGTAA
AACCTGAGTCATGATTCTGGGAATCCACACCCTGGGAAAGTCCCCGCTGGCCCAAACCTGAG
CCAGTCCGGATAGGGATGCGGGATTTGAAAATCGCGCGCACGCATGCACCCGTCGGGCTCA
GCCAATCATTACCCACCAGCTGTACTAAGAACCGCCTGTATAAAAGCAGCTGTGATTCAGAG
CTCCGGGCTCTCATCAAGACTCCACTGTGCTGGATGAGACGGGAGCCCTAGCTCGAGCTAGC
AATAAAACCCCTTTATGCTTTTGCATTGCTGTGGACATCTTATTCTCTCAGTTTTGGGGACTC
GGACTTTGGGCATAACACTGAGGAGAACATTAAAGAAAAACTATTCTCAAGTTCCAGAGTC
ATGATGAGTCTCAGAACAAAAAGCCACCCAAGGATAGGTTGCAGGGGATCTGGGGTTTAAA
AACATACTATTGATTTACATCAGATGCACAGCTGAATGAGCTAGTGTGGAAAGGATACATTA
ACAAAACTGGTAGGATCACAAAACAAAAGCTTCCCCAGTCCCATGGCACCTGTGGGTGGTTC
CTTTTAGTTTCCATTACTCAAACAGTGACCCAGTTAGTCTTCTATGCTTTGGTTTTATGTTTTA
TCTCTTTGAACTAACAGCCATGTAGTAAATCTACAGCTGTTCCTTAAAATTTCCCATTTGATT
CTGTGTACTTTCAAATATAATTTGACCTGGCCTCCATTCAGGCAGAATGATTACTGCTGCATT
ATGCCATTTTAACAGCATAAACAAGTGTGGAGAGTGGTGTGCTTTGATTACTGTGTGTAATT

TAGTCAATCTACCCATTGTGCTTGAAAAAGCAATAATAAAGAGCATAATTCCTAGGTAAAAA
GTAAAATAAAAAATCATCAGAAATTATTGAAATAAAGAACAGAAAGCTGTTACCAGTGTCA
TCTAGGTAACACATACAAATGAAAAGGCAAAAGTTAAAACTGAAGTTAGCTCACATCTTTCA
GGAATTTATTACTAAAGCACCAAAATTTGATATTAATTATCAGAAAACATACTTTTATAATA
TATGAGATGACAAATGAAAGCAAACAGGTTTTCATTATTGTTTTAATAAAAGAGACTATTGT
GATATGCAATTACTAAACAGTTAATTTGAAAAAGAGTCAGACACGATTGAGCGACAGAACT
GAATTGAACTGAATTTGAACTTTACCTCACACAAAAATATAAAAACATCCTTTTATATAATA
TTGTTATTAAATCTTTAGGCTTTATGACAAAACTGTTTCTCCTTCTCATACACTTGCAATTATG
ACTGATTTCTGGCTACATGCTGAGTCACCAGCTGAATCACGGACAGAATCAGTGATATAATC
TGAACTCAGTAATAAGATGCATGGACTTGATACGGCTATCTCTTATCTTCCTCTAGTAGTAAA
TTGTTATAAGCTATTGACTAAAATAAAAATGCTGTTTACATGTACCTAGGTGATACCAGAAA
ATGAGGTGGCAAAGAATAATCAGGTGATATGCTTAGAGTTTGTTGGCTAAAAATATACACA
ACATGAAAGTTGCAAGTTAAGTTTTATTTGGGGCAAAATGAGGACTGCAACCCAGAAGACA
GCACCTCAGATAGCTCTGATACACTCCTCCAAAGAAGCAGTGGGGAAAGGTCAATATATAA
GATTTTGGTGAAGGGGGAGTTCAATGCAATCAAGCACTCATTTTACAAAAGGTCTTCTGTTA
GTTACAAGAAGCTGATGTCACCAGGAAGGGATTTAGTGCTTTTCTAGATAAAAGGAGATGC
AAGGATTGGGATCATGAAATCAGTTCCTGAAAAGATAAAACTATCTAAAGACCTATTCCACC
AGTTTCCCTGGAGCACAGAGTGTCTCACTCTCAAACCTGAATTTCCTTATGGGAGTGTCAGA
GCAGCTGCAGCACAGGGAATAGCAAATGTCCTTGTTGTTCAGCTGCTGGCAAATGCTATGGG
CAAGTGTCCAATGTAGTTGATGGGCAAGTGCCAGTTTGTAGTTGACAAGTTCTAAGCTAAAA
CAAACACACAAACAAACAGAAATTTGGCAATTGAAGACCAATATACGAATAAGAAAATAAA
AGCTCATGGAGAGTTCAGTTCAGTTCAGTCGCTCAGTCGTGTCCGACTCTGCGACCCCATGA
ATTGCAGCACCAGGCTTCCCTGTCCATCACCACCTCCCAGAGTTCACTCAGACTCATGTCCAT
CGAGTCAGTGATGCCATCCAGCCACCTCATCCTCTGCGGTCCCCTTCTCCTCCTGCCCCCAAT
CCCTCCCTGCATCAGAGTCTTTTCCAGTGAGTCAGCTCTTCCCATGAGATAGCCAAAGCACT
GGAGTTTCAGCTTTAGCATCATTCCTTCCAAAGAAATCCCAGGGCAAATCTCCTTCAGAATG
GATTGGTTGGATCTCCTTGCAGTCCAAGGGACTCTCAAGAGTCTTCTCCAACACCACAGTTC
AAAAGCATCCATTCTTCGGCGCTCAGGCTTCACGGTCCAACTCTTACATCCATACATGACCA
CTGGAAAAACCATAATCTTGACTAGACAGAACTTAGTTGGCAAAATAATGTCTATGCTTTTG
AATATGCTGTCTAGGTTGGTCATAACTTTTCTTCCAAGGAGTAAGCGTCCTTACTTCATGGCT
GCAGTCACCATCTGTAGTGATTTTTGAGCCCCCAAAATAAAGTCTGACACTGTTTCCACTGTT
TTCTCATCTATTTCCCATGAAGTGATGGGACCAGATGCCATGATCTTCATTTTCTGAATGTTG
AGCTTTAAGCCAACTTTTTCACTCTCCTCTTTCACTTTCATCAAGAGGCTTTTGAGTTCCTCTT
CACTTTCTGCCATAAGGGTGGTGTCATCTGCTTATCTGAGGTGATTGATATTTCTCCCAGCAA
TCTTGATTCCAGCTTGTGTTTCTTCCAGTCCAGCATTTCTCATGATGTACACTGCATAGAAGT
TAAATAAGCAGGGTGACAATATACAGCCTTGACGTACTCCTTTTCCTATTTGGAACCAGTCT
GTTGTTCCATGTCCAGTTCTAACTGCTGTTTCCTGAACTGCATACAGATTTCTCAAGAGGCAG
GTCAGGTGGTCTGGTATTCCCGTCTCTTTCAGAATTTTCCACAGTTTATTATGATCCACACAG
TCAAAGGCTTTGGCATAGTCAATAAGGCAGAAATAGATGTTTTTTCTGGAACTCTCTTGCTTT
TTCCATGATCCAGTGGATGTTGGCAATTTGATCTCTGGTTCCTCTGCCTTTTCTAAAACCAGC
TTTAACATCTGGAAGTTCACAGTTCACGTATTGCTGACGCCTGGCTTAGACAATTTTCAGCAT
TACTTTACTAGCATGTGAGATGAGTGCAATTGTGCAGTAGTTTGAGCATTCTTTGGCATTGCC
TTTCTTTGGGATTAGAATGAAAACTGACCTTTTCCAGTTCTGTGGCCACTGCTGAGTTTTCCA
AATTTGCTGGCATATTGAGTGCAGCACTTTCACAGCATCATCTTTCAGGATTTGAAAGAGCT
CAATTGGAATTCCATCACCTCCACTAGCTTTGTTCATAGCGATGCTTTTCAAGGTCCACTTGA
CTTCACATTCCAGGATGTCTGGCTCTAGATTAGTGATCACACCATCATGATTATCTGGGTCAT
GAAGATCTTTTCTGTACAGTTTCTCTGTGTATTCTACCACCTCTTAATATCTTCTGCTTCTGTT

AGGTCCAGACCTTTTCTGTCCTTTATTGAGCCCATCTTTGCATGAAATGTTCCCTTGGTGTCTC
TAATTTTCTTGAAGAGATCTCTAGTCTTTCCCATTGTGTTCTTTTCCTCTATTTCTTTGCATTG
ATTGCTGAAGAAGGCTTTCTTATCTCTTCTTGCTGTTCTCTGGAACTCTGCATTCAGATGCTT
ATATCTTTCCTTTTCTCCTTTGCTTTTCACCTCTCTTCTTGTCACAGCCATTTGTAAGGCCTCCC
CAGACAGCCATTTTGCTTTTTTGTATTTCTTTTCCATGGGGATGGTCTTGATCCCTGTCTCCTA
TACAATGTCATGAACCTCATTCCATAGTTCATCAGGCACTCTATCTATCAGATCTAGTCCCTT
AAATCTATTTCTCACTTCCACTGTATAATCATAAGGGATTTGATTTAGGTCATACCTGAATGG
TCAGAAGTTTTCCCTCCTTTCTTCAATTTGAGTCTGAATTTGCTAACAAGGAGTTGATGATCT
GAGCCACAGTCAGCTCCTTGTCTTGCTTCTGTTGATTGTATAGAGCTTCTCCATCTTTGGCTG
CAAAGAATATAATCAATCTGATTTCGGTGTTGACCATCTGGTGATGTCCATGTGTAGAGTCTT
CTCTTGTGTTGTTGGAAGAGGGTGTTTGCTATGACCAGTGCATTTTCTTGGCAAAACTCTTAT
TAGTCTTTGCCCTGCTTCATTCCGCATTCCAAGGCCAAATTTGCCTGGTACTCCAGGTGTTTC
TTGACTTCATACTTTTGCATTCCAATCCCCTATAATGAAAAAGACATCTTTTGGGGTGTTAGT
TCTATAAGGTCTTGTAGGTCTTCATAGAACCATTCAACTTCAGCTTCTTCAGCATTACTGGTT
GGGGCATAGACTTGGATAACTGTGATACTGAATGGTTTGCCTTGGAGATGAACAGAGATCAT
TTTGTCATTTTTGAGATGGCATCCAAGTACTGCATTTCGGACTCTCTTGTTGACCATGATGGC
TACTCCATTTCTTCTGAGGGATTCCTGCCCACAGTAGTAGATATCATGGTCATCTGAGTTAAA
TTCACCCATTCCAGTCCATTTTAGTTCACTGATTCCTAGTATGTCGACGTTCACTCTTGCCATC
TCCTATTTGACCACTTCCAATTTGCCTTGATTCATGGGCCTGACATTCCAGGTTCCTATGCAA
TATTGCTCTTTACAGCATCGGACCTTGCTTCTATCACCAGTCACATCCACAGCTGGGTATTGT
TTTTGCTTTGGCTCCATCCCTTCATTCTTTCTGGAGTTATTTCTCTGCTGATCTCCAGTAGCAT
ATTGGGCACCTTCTGACCTGGAGAGTTCCTCTTTCATTATCCTATCATTTTGCCTTTTCATACT
GTTCATGGGGTTCTCAAGGCAAGAATACGGAAGTGGTTTGCCATTCCCTTCTCCAGTCGACC
GCATTCTGTCATGGAGAGTAACGGAATGTAAAATTTGGGATAATAATAATAGTAATATCAGG
GCATGTAGTACAGATGTCTTGAATATCATTCTATGTTTTTACTATCCTGAGATACTTAAAATT
TTCAAAGCAAGTAAGGGGTGTTAAAAATAGCAAAGCAAAACAAACAAACAAAAAATGGCA
AAGCAAAGGGAAATTGTGAGACAAGATAAGGCAGAGATTAAATTCTGCTGGCCTATTTCAT
AAATCTTCCATATCACCACAGTCCCTACTGAATGCATATTTTAATCAAGCATTCAACTATTGA
TATTTCTGTGATCAGCAAACATACAGGCTTTCTTTTCTTTCTTCCCCATTTAAAAATTATTTCC
TATTTGCTGAGTTGGCAGAACTTCAGGCACATTGATTGCCTTTCTATTTCTGTACATATCCCA
CCTCTTTAAGGGCCTCAGAATTTTTATTAACATTGTGTGCTCTACCTAGAAGCCCTTTTTTTCT
CTTTTCCACTTTTTCAAATCCTACTCATTTTTCAAAGCTTAATACCTGCTACCTCTGCATTTCC
TCATCTCTCTCTGAGATCTCAAAGCAAATTGTTTATGCATATTTTATTATTTTATAACTAGCC
ATTATTTCTATAAAACAGTTTTTCCAACCACTGTTAAAATATTCAAGGATTAACAACAATAA
AAATAGTAAATATGTGTTTTAATTTCAACTTTAAAAATCAGTTTGAAATAAAACTGCTTAAA
AGAGGAAAAAAGAATTTGAAGTCTTAAAGGCTGTCAAATGATATGCATTCAATTTATCAAA
GATGAACACTGCCAGTTATAGGAAAAGGTTTACTCTATTCAATTGTATACTACCATTATTCTT
ATTTTTAAAACTATTTTATAAAGCATTGACTAAAGATATTGAGAGAATTAAGGGTATTAAAA
ACCATAGGTGAAAGTAGAAGTAAAGAAACTTGTATATATATAAATTTGTACCAACACCATCT
TTATCAGATTTGAAAACTGCATTCAGGTATTTCACATTTCAATAAAGAAGAGAGAGTTGTGT
CTAGAAATTCAAGGTCAGTATCCAAAAAGAATATATAGTGTCACATATATTAAATTTAATCT
TTTGTTTGTTAGGTACAAAAGCAAGTCACATGCAAATAGTGTTATTGATGGAGAAGTGTTTT
TTAAAAGTGGCCTCTAACCTTCTTTCTTCTCCCATCATTAAGAAATTAAACATTATAAATATT
TTTAAAATGCACAGTCTAAAACTGTGAAATGCAAAATTCAAGTAACAGTGCAGGAAAGGAT
TTTTTGTCCATGTTTGCTGATAGAAAAGTCGTTAAATTTCAAAACACTGTTCACCATGTATTT
GGTAATGAAACACAACACATTAAAATTTTAATTTATTCATTAACAAATTAAGCTTCTTACTA
ATTATCCGAAAGTAAAATTTCCTTGTTAGTAGAAAAAAAATCAAATCTATTTTTCTATAGGG

CTATATTAAAGAATACACAATCCTTAATAATTTACTAATATAGTAAGAACTCTGACTCTACCT
CATATTATTAAAAATGAATACAATTTTAACATGACAGGCTGTAGGTATATAATTATTAAAAT
GGGTCACAGGTTTCAAAAGGTTCGGAAATGTTAGTTGATGAAAAACAATTTTACACTGAATA
TAATAAATTAATATTTACATTCACTCAGAAATGTGTTGTATAAATAAATCAGTAGATGAATA
AATACATGAATTGAAAGGAGGAGGGGAGAGCAAAATTTGTTAGAAAAGAAACTAATTGTAA
AATCAGACAGACCTGGTTTGAATTTTTAAAACTGCCACTTATTAGGTATAGGCTCTTAGGAA
AGATGTTCCACCAAAAGTCTTTGAGCACAAGTTAATTATAAAAATAATAGGTGTGACAGTGT
CACTAACATACCTACCTATGCAGCAGTCCTTATGTTCACACCATTCTCTACTTGCTCTGCTTG
AGTGTAGTTTCAGCCTGCATGTAGTATACATGTGGTTTCAACCTGTGTTGAAATGTTCAGGA
GAAAGATGCAAATCTTAGAAGTTAGTATTTCTTCCCAGCTTTCCTCTGTGTAATCCTCATATA
TCCAAATTCAGTTGCATCTTTCACACTGAGTTTGTGAAATGGGATGTCAATATGGTAGGGTTT
TTGGATTCTTTTGGTATATTTTTTTGTTCCATTAATTCACATTGTTTCCTATATCAAAGCAAAT
AACCAGAAAGCTATTTCTTAAAGACTTTTCATTCTCCACAGAAATTCTTTCATGGAACTAAG
GGTTGAATGTTAAATCTTAAAACATTTTCTTGTTGTTTCAGTCACTAAATCGTGTCCAACTCT
TGCAACCTCCATCTATGGACTGTAGCCAGCCAGGCTCCTCTGTTCATAGGATTTCCCAGGCA
AGAATACTGGAACAGGTTGCCATTTCTTTCTCCAGGGGCTCTTCCTCACCATGGATCAAATCT
GCGTCTCCTGCTTGGCAGGTGGATTCTCTACCACTGAGCCACCAGGGAAGCTACCTTAAACA
TTTTAGTTGCGGGTTAATGTCATAAATAAATATATAAAAATAGTGTATTCAGCTAGCCTGCA
AATATTTCTGGCATGCTTTCTGCGTGTTGGGTACTCTTCTAAGCAATGTGGAATACAAAAGTG
AACAGGACAGTGAAAATTCCCTGGCCCCAAGGATTTTACATCTTCATATGGTGTATGTTAAA
GATTTTAAAAATACCAAACAAATATATGATAAAATAAGGTAGCATAATAAATGCAGTAGTG
TGGAGTTACAAGATAGAATATGAAGAGAGATTGTTTCTTAAATACATTGGGCCCTAGAGCAA
ATATTTGAGTAGAGAAGCTGAATAAAGAGGGGAGGAGAGCCTGCACAAAAATACCTGGGA
GAAGAGGGTTTTAGGTCAAGGGAAGGTCCAGAGGTAGGATGTAGTTTGGTGTGTTCTAGGC
GGGCCAGTATGGTATGAACAGAGTGAATGAACTACAACATAGTAAGATTTAAAGATAGAGG
AGAAATTAACTGCTAGCCAGATGAGGTTTTTTAGATTCCCAATATTTGAAGACTATTTTCTAC
TTCTGAACCAGTTGTTGGAACTCTTTGAAGACACTGTCCATTTTAATGTCTATCTCTTTTAAA
GATCCAGTAATAAAGTTATCTTAAGATTTGGTTCATGAAGAAATCTGATGCCTGCTGAAGGA
CTGCTAAGTTTTCATTTATGATGAGTGTTTTAAAACACATCACAGTTTCCTTGTGGTTGGGGC
TGCCAGGAGACACCAAGCTTGGGCTTGGCTTGAGTGAACCAATGTTCCTCAGCTAAGGTTTT
AGGCCAAATTCATCTCCCCTCCTTTATTTTTTCTCTTTGTTTGTTTCACATCTTCACTCATTTTC
TGTTGTGTAATGTTGCTTCTAGAAATAGATACAATGAAATAAATCAATAAAACTACCAAATC
TGTATATCAGAGATCTTTCTAAAACTATGATTCATATGAAATCCATTTATTAAATGATTGGTT
AGTATATAGAAACAGTGGACTAGATTAAATGAATAAGCCCTGAATTCAATCAGAGATTAAA
TACATATACTAAGTTTGATAAAACAAGATTTAAATTTACAGGGAAGTAAATCATATATAATA
ATAGGGGAGGTCAGTTCAGGAATAGGTTTTGTTGGCTAAAGGAGAATTGAATAAACCACAG
AGAGAAGAGAGAAGATTTGTTCATTGAATCTTAAAGGAAGACCACTGCTGAATATGGTGAT
ACTTGTCCACTAAACAATATGGTAATATTTATACAATAAACAATGACAATAAATGTTATCAT
TCACATTGTATATGTGGTAATATTATATATAATGTAGTATAAAATATATGTAGATATATAATG
ACAATTTTCTGGTAATGTACATCAAAGTATTTTGAACAAGCGGTTCCTTTCATTTGTACAAAT
TAACCACATAGACCAATAGTAGCACTAACAGTAATTTTTAAACATGAAAGTATTCATTAATT
TCAATTAAATAGTCTAGGATGAAGCACAAATATGTTATATTTTTTTAGAGTTTTCAATGTACA
CACCATGCTAAGGATCATTTGGAGAGTGGCATTTATATAGATAATTGAAAATTCTAGATAGA
GGAAATATTTGAAGCAGTGTGAAATGCAAGATAAAAATGCAGAGACCATTTCATTGAAGAA
TAGACTTCATGCAGGAACAGAAATAATAGTTAAAAATCTTCAGTGCTGGTCTAAGGAGTTTA
AAGTCCATTCTGTACTGTTAATCGGAGAAGGCGATGGCACCCCACTCCAGTACTCCTGCCTG
GAAAATCCTATGGACAGAGGAGCCTGGTGGGCTGCGGTCCATGGGGTCACGAAGAGTCAGA

CACGACTGAGCAACTTCCCTTTCACTTTTCACTTTCATGTATTGGAGAAGGAAATGGCAACC
CACTCCAGTGTTCTTGTCTGGAGAATCCCAGGGATGGGGGAGCCTGGTGGGCTGCCGTCTAT
GGGGTCACACAGAGTCGGACACAACTGAAGTGACTTAGCAGTAGCAGTACTGTTAATGGAA
ATGGGTACTATTAATGTTTTATTTTAAATAAATAAACCATAAAATCAAATTTGTGTTTTAGAA
AGATTACCCTAAAATTAGTGGGAAGCATAAATGAGAGGGAAGTAATTCTGCATGCAGGAAA
ACCAATTAGGGGACCATAGCAATGGTTAAAGGTAGGTGGTGATGGAAATCTGACTTAAGTA
GAGGCAGTGAAAAGAAAACATGGGAAACAAATGGAAAATCCATGACGAAGTTAGAGCTGT
TAGGAATTGGAAGATGATTCAGTGTAAGGGGACCAGTTTCAGGCTGTGATGGCAGGAGGTA
GAGATGTAGAGATAGGGAAGGGGGGCTGTGTTAAGGCAGCAGGGTAAGAGATGCCACGGA
AGGTTGATGAACAGTTATATCACAGTGACTCAGTCACAGCACGGTGTTTAAGGGCTTAAGGC
CTGGAGTCATCTGAGCCCAAATCTGGCTACTTAGAGCATTGTCACTGTGAGTTAAGTTACTT
AATTTTACTAAACATTCATTTAAACTTGTAAAATGGTAAATGTTAGTCTTATATCCAGAGTTT
TAAAACAAATAAATGAGTTAATATATGTTACTAGTGTATAGAGAATTCTCAAAAATGCCAAG
TAAATTTATCATTAGTGTAGAATTTAGGGCAAAAAATAGATTTGGGGATTAACTATGCAGAA
GTGATAAATTGAGCCCAGACAGTAGCATATATTGCCAAGAAAGAGAAGACATAGGAAGAAA
AAAGAATGGATAGGACAGGAATATAGAAGACTCCTATATTTAGAATGCAGATAGAGCAAGG
GAAACACAAACATGGGCAGGGAAAGGACAATCAAGAACATGGATATTTGATCTTTACATGA
AAAATGTATGCACAGAGGAATCCTATTTGAAGTATGATCTAAAGTCTGGACATTTTGGTTCT
AGAGCTTTGTTAGAACATTTTGATTAGCCTTAGTATCATCCTGCTTAATCTTCCCCTATCCTA
AAGAAAACAGAGCTACAAGACACTAAATACTGTTTTGTACAATCACTCATCAAGAAAGTAC
ATACGAATTGTCTGATTATTAGCTAATTATGTGATAACATCAAAACAAAAATTTATAAACAG
ATAATGCTACAACATGTTAGGTAATAATTATAACAAAGCTGTGTCTGATTTAAAAACCAGTG
AGACATCTGCTTGGCTGCTACAATAATTGGCTACTAGCCTGTTGTTTTGTTGTGCTAGAAGGA
CTTTATGTCCTGTGGATATTATTTGCTCTTGGTCCAGGGGGACTCAAGTTTCCATTAGATTAC
AAGCTTCTATACACAGAGCCAGATGTGTGCAAGACACTTGAACAATTTCTAAGGCACTGAAA
AATTTTTCCTTTTAATACTTTTAAGGTTCATTCATTTTCCTTTGGACCTGTCTCCATGAGAATT
TGTCATTTCCCTGTACTTCTAGTCTCTTAAGGTATCACTTAGTGTGTTGTGACTCTCCTTGCCA
CAGCCTTGCAGAAACTTTGTATGATGACCCAAACAAAAGAAGATACATTGCAATAAGCATTC
GCTCCTATTAACATTCTACCCAATTGCACAGTCACTCATCCATTTATATAATCTTTTATTCAA
GAAATTTCCCAGAACCAGCTGAAATATGTTAATCATTGAGCTAGATGTTTTCTCATATGTTAT
CTGAAGAATAAATGTTCATGTTAGGGGGTAGAATAATTCCTAGATAGCTTCAGTTTAAATAG
TTTCTCAATTCTTCTTCTTCCCTTTTCCTCATATACCACCACCACCAAAATCTGTTGATTTTAC
CTACACATTTTACAAATCCATGTTTTTTCCTCCATTTATCATCCATTTTAGGATCTTATCATTT
AAGTCAGTAATAATTTCTTTGCTGGTTTCCCCACATCTATCCTTACTTCTCTTTAATCCATTAA
TAATGAAACTATCAGAGAGCTTGTTCTTAAATGTAATTCAGATTCACCCATGTGTCTCTCTTG
ATTAAAATTTTTCAAAGGCTTCTCATTGTTCTTAGAATAAATCACTACAAATCCTTGTAACAT
TCCACTCTCCCCCAACCCTTCTGTCTTGTCTCATATTACTCTTCCTTAACCTCTCTCAGCCAGT
CACAGTGGTCTTCCACTGTGCTCCTTCCTGTCTGTGCCACTTCCTTATAGTGGTGGATCCTTCC
CTTTGAATGTTTCTTAATCTTTTGCCATTTAAAAGCAATTCATTTTTAGATATTTGTGTATGTG
CTCTATATTATATGTATATATATACATACATATATGTACATATATATCATATATTTAGGTGTA
GTAGAACTAAATGGTAAATAAAGCACTCTCAAGAATCTTATTGTTTAGTGAAGGCAGACAG
GTACTATTTAAGACATCTCGATAATATACATATTATATAGGGAAGAGTACATACAAAAATTT
TAGGGAAAACTCTCTGACCTCGCACCTTCAGTTCAGTTCAGTTGCTCAGTTGTGTCTGACTCT
TTGCAGCCCCATGAACTGCAGCATGCCAGGCCTCCCTGTCTATCACCAACTCCTGGAATTCA
CCCAAACCCATATCCATCAAGCCGTTGATGCCATCCAGCCATCTCATCCTCTCTCGTCCCCTT
CTCCTCCTGCCCCCAATCCCTCCCAGCATCAGAGTCTTTTCCAATGAGTCAACTCTTCGCATG
AGGTGGCCAAAGTACTGGAGTTTCAGCCTCAGCATCAGTCCTTCCAAAGAACACCCAGGACT

GATCTCCTTCAGAATGGACTGGTTGGATCTCCTTGCAGTCCAAGGGACTCTCAAGAGTCTTCT
CCAACACCACAGTTCTTCGGCGCTCAGCTCTCTTCCCAGTCCAACTCTCACATCCATACATGA
CTACTGGAAAACCATAGCCTTGACTAGACGGACCTTAGTTGGCAAAGTAATGTCTCTGCTTT
TGAATATACTATCTAGGTTGGTCATAACTTTCCTTCCAAGGAGTAAGCGTCTTTTAATTTCAT
GGCTGCATTCACCATCTGCAGTGATTTTGGAGCCCAAAAAGATAAACGTCTGACACTGTTTT
CCACTGTTTCCCCATCTATTTCCCATGAAGTGATGGGACCTCTTACCTTATCACAGTGTAAAT
TCCTTCAAAATTTATCCTCATTGATTGCAGTTTATGACAGTACAAAAATGGGGCTTTCGTGTT
GCCTCAGAAGGTAAAGAATCTGCCTGCAGTGCAGGAGACCCAGTTTCAATCCCTGGGTTGGG
AAGATCCCTAGGAAAAGGCAGTGGCTACCCACTCCAGTATTCTTGCCTGCAGAATTTCAAGG
ACAGACAGGCCTTGCATGGACTGAATTCCATGGACAGAGGGCTCAGTCTTTGGGGTTGCTAA
GAGTCAGATATGACTGAGCGACCAACACTTATATACATAAACAACATCTGCATATATCCATT
TATACCTACATACACAGATAAAGAAATACATACAATATATAGATGTATACCCATATGCATTT
ATATATATACATAAATATGTTAATATAAACAAATACAAACAATGTGTGTGTGTTTGTGTATA
TATGCACACTGTCTATACATTTAAATGTATATGTATATTAGGAAACGTCTCAAACCTTTCAGC
CTAGCTTATCACAATAAAACTTTCCTTCATTATTTATCCTACTTCATTATAATATGATTTATTA
AATAGTGCTTATCTCCCTTCACAAAATAAGACATTAAGAGCACAGCTTTGGCCTTTTATTCAC
CATTTTTCCCACAGGAGTAAGAGGATACCATACGCAATACCAAAACACATTTCAGTGTGTTT
TGCTATTGGATGGGTAAGTGATTAAACAAAAACCCATCAACCTTAAAAGGAAAGAATATTG
CTTTGGAGCTATAATATTTGGTTGCAAATTATCGATAAGGCTTTTACAACTAATGAGGATGA
CTCTGGAAGGCAGAAAGCAAAATAAATACACAGTCGAGCAATAATTTTAGGCTTCCAAGAC
TTAAGAGCAGTGCTCCATTAGATCATTTTTAGTAGTAGAGATTTCCACCAATCCTCATGACTT
TTGATTGACAAACACATAGCCAGCACTCACCTCTGACGTGAACTTTGTGAGGAAATGGTGGT
AAAAGCTTTACTTCAAGCTGATGTTTGGAATAAAGACATTTCACACATGTATAGATTTGAGG
AGAGCAAGTTAAGCAGAGAAGATTAATATTGACTTGTCCCCAGCTTTCCTGTAGGAAAATGC
TCCTTATGAAGAATGTCTGAGTGAAATTGTTTATTCCACACTAACATGAGAAAACAGACAAA
AAAGCCTGGAAAGAAGGGTGTGTTACTGGAATCGATGACAAATTAGTCCCCAAAATGTCTTT
CAAATACTAAATATACACGTTAAGCAAGCTGATAATAATTCAAGGATGTTTTTCAAATGTGC
TTTCCCTAAAAATTTAGTAAGGTTCACATGTTTAAGTATCTTGGTGAATATTCTCATTGTTTTT
AAGTGTGTACAGGGGTTTAAGCCAACATTTCCTGATAAGGCCACAAAGTATTCCTTCCCTGT
TATGTTACAGAGACAAGGATGCAAGAACAGATTACTGTTAACACTTACACAGTGAATAGCA
ATATAAACAAAGGGGCGAATCTTTGATTTAAAATTTGAATTTTTCTGAAGGGGATTTTTGAA
AATGCATCATATTATTAATTGAAAAGGCCTAAACCTATTCAGCAGGACATAGTACTTAAGGG
TGAAGTACTAAGAAGGGTGACTACTTAGTCAATGCTGCTAATAATACTTGCTGAAGCACCTT
GGATGGCGCTGGTCAGATAGAGAAGAGGGATCGGAAAAGATGACCCTCTGAGGGCAGCAG
GAAACCAGAATTTGAGGGGTGGGATAGAGATTGGGAAGGACAGAAATATGAGTGTGACTAC
TGCTGCCACTGTATCCATGACGCTAATGCATACGGACAGATTCATTTGACTGCTGTTCTGAG
AAAAACATTTTCACGTAGAAATTGGACATTTCGCAATGATGTTTTATGAAGAACACGCTACT
AACAGCTGGCAACAGCTGCTGTTAGTGGCTGGGTTGTTGGTTCAACTTTAATTCCTTACAGTT
TATGGCACTTGTGTGACAAAATCCAGGAGAGGTTTTGATTTTGCCTAGTTTAATACTTAAAA
GGAAACAGTTTGGGGTGCAAAAGACCTAGGTGTAAGTAATATCCTGTGATGAAATATTTAAT
TCTGAAGATAACTGCTTAAGAATGAAAATTCTCTGGTTGAAGAGGGTCAGAGGGCTTGTCTG
AGTTACCCCAGAGCCTAATCATTTTGTACCTCAGTCCATCACTATTTTCCCTCTCCCTCAACA
CTCACCCTTCAGAAGTCAGTGTCACCAGGGAGTGAAAAGTTCAATGATAATGCATATATAAT
TCGTAGTAAGGATATTCCACATTCTCTATTCTTATTTATAAGGCCTAATCCATATTCAGAGGT
AATGGGTGCTTAGGGTGTGGGCATTCTCTGATACTCATTGTGATTCATAGAAGACATATGTG
AGACTGAGTTCACTGAATTTGTAGTTTGTCTTCTGACTTCTTTGATAAAACATTTTGTTTAAC
AGATTCCTAGTTCAAGTAAAAGAAAAATGTTTTATACACATAGAACCTTTTTTTCTATAAAG

AAATGTGTCACCTTCTATGACAGATGAGAATCTGACCTTCTCATGCCAGAATCTCTCTGGTAT
TGACCACCTGACCTTGCCAGGTGACTCTGTGTCACCTGGATGTCACGTCTTTGTGGTAGGTAT
CCATCTCAGACGGCCAAGCATGATATTAATGGCTGAGAAGTGGCCATCGGACTGGCCCTCCA
GGCTTTGGTAGGTCAAATTTTGCTTATGCAAACTGACAAGGACAATTCAAAGAAAAGTTAAT
GCAAGAGATGAGAGATATCTCTGGCTAGGGTTGTGCTGTACTTACAGATCGTCTATCTTTTCC
TTGTATTAAGGACAATGCTTCTGGGGAGACTTTCTAGTGTCAGAGCAATACTCCAGAGGGAC
CGCTTCTGTACTTTCCCTAACTTTGTTTGCAATAAATTAAAACAGTTTTGGAATTACTTATAG
GTTGATTACTTTGACACAGAGAATGTGTGGATGAAACATTTTTAACCTGTAACTATATTTTAG
GGTGGGAAAGAAACATGCTTTCAGTCAAAATTGGGTGTTAGACTCTCATATGAACTACTTAT
TAGGCACGTGTCCATTGTCAGTATTTATCCTTTTGAGTCTGGTTTCTTTCCTGTAAATATTTGG
ACCTAAAAATATTAACTTGATTTGAAAGGATGTTTCAGGGTTAACAGAAGAGAATGATATTA
CACGATATGTACTTAGCAAAGTGCCAAGCGTGTAGACAGTGCTCAGTGCACAGCATCTATTA
AATAAAATACTAGATATTAAAATACTAGTTTTGGCCATTTGAAAAAAGTCATTCTCCTGCCT
GGAATGACAGGTAATACAGGTTATAGTTCAGCTTCATTTAGAAAGCGGTAACTCCAAAAGCC
ATTGTTTTTCTCTAGTGCCATTCTGTAAAGACTGTACTAACTGAAAATAATACTGCAAAATCT
CAGGAAAGCAGCATGACTTTTCTAGTTTCTTTATCCACTTTGTTTTGATATAGTATTATAGAT
TCTTAATAAATAATATGAATCCCAGGAAGGGACTTATTGTTAGAAATCTTGCAATTATAGCT
TCAGAGTCATATCTTTAATCAGATTGAGGGTAGATCCTTATTCTTATGCTTAACAACAGTACT
AAGCACAATTCGATTTAAATCTATCATGTGATACAACTATACCAAAAGATTAACTTAAGCCA
AAGTACCTTGGCAGTATGACACTTTCCACATCATTAAATGAGGCAAACCAAAAAGGACCAA
ATTACTACTTAATTTGGAGAAAAACATACTAGAAAGTTTACCAAATACATATTTACATTTCA
ATGACTCACTTGGAAAACTTCCTGACAAAAGTTTGTAAATAAATATTTAATATTCATTACTGT
TTTCTTTCAACTTAAGAGAAATTCAAACCTGTATCTATACTATGACAACAAATGCTAAACAT
AGACAGAAGCAAATTGATTGTATATGATTGATCTGAATTCCTGTGCTTCATAAGCAAAGGAA
TTATCTGTTAAGGCGTAACAGGTTTTAACAGGAAAAGGATTAAGGGTTTTATTTTCCTCAAT
GGAGGGGCGAAATGAGCAAGATTTTTCAAGTTAACATTCACTTCATCTGGCTCAGGTGTCAG
GGATCTATATTTATCTCGGTTTAGGCATCTGAAGTGCTGGAAGTCTCAGGATGTCAATGGCTT
CACATGTCAGGATAACCTTAACGACAGACGAATGGTTAAGACCCTGAGCCTCATTTCTGAGT
TCTCAGCTGTGGAAATCATGTGTTTATTTTCTGCATCTCCTCCTACAACAGTCACTCTGAAGC
CAGTTTTAAGATCCTACATCTGGGCAAGCAAGCTTCTCTGAAAGTTGGTGAGCACAGTTATT
TTTGTTTAGGGGTGTGGCTATAAATGTCTTTGTAATTGATGGACTGTCATCAAGTAAACATTA
ATACTTGTTATTTCAAACTCCTTAAGAAGCTAAATACTAACTGGTGTAATAATTTAGTTTATG
ATATTGTTTAAACAGAGTTATAAGCTTTTAACTAAATGGACAGACTTTTTAAGACAAAGTTTT
CATTTCCACTAGTCTCTCAACTGATCCTATGAAATTTCCATTTATACATTCTCATATCTTTTAT
TTTCAAAGTATCAGAAAACTTAACCCAAGTATTTTTAAAAGTACAATTTTGGTGGAACTTTTT
CTTCCTTAAGATTCTGAAGTTGATCTGTCATGTCCTGTTCTTAAACTGATTTCTTATTTTATTA
ATTTTCGTATGTCTTGTTTTAAAAATGTCACTCTTTTGAAAAGGATGCTCTCAAATTCCGAGC
AGTTCAATTTTCTGCTTACAAGTTGAAAAAACATGTTTTTAATGAGATTAGAAGTTGAATGA
CCTTTGTTCATGGGACCTAAAAATGTTTTTTGGTGTTTTGTCAAGACTGACAAGTATATTTAA
GAAATAATCTAAAAGACTCAATCCTCTTAACAAACTCTTAGAAGAAAAAAATATTTCCATAA
ATAAAATTACTGATCACTAGTGATCACAGAAATTATACTGACTGTTGGCACCAGATATTTGA
TGGAAGGGAATTTTGTTTCATGGCAAAGTTTTTGTTCTATGGTGTGTTTCATACTATGGCTAT
ATAAGACCAGGAAACAACAAAGAATGATAGAGGATGACTTATTCAGACAGGAAACTAAGG
GTATGCTTAGAGACAAATAAGTATATAATGATATACTTCTTTAAAGACAACTGCTGATACCA
AAAAAGCAGTTCCATCTGACTATGCTATGTTACAAAGTCTGAGTATGAAATGAACAGGCTTC
TTTACCTTTAAAAAAAGTCACCTTCCATAATTCTTTGGAGAATGGTCTCTTGGAGCCGTGCTG
AATTCAGGGGAAAGCACTCTGGACTAATACGTTTTAAAGTATTAACTTCTGTTCACTTCCTAT

GCAAGAACTCAGGTAAGATCTGGATCAGATGAAGAGAAACAAGCAATCTGTCCTGTTCTCA
GGTATACCTGACTAAGTAAAGAAGGAGGGTTTGTGGGACAATATATGAGACTCCTGCAATC
CTGGCAAGGGGAAAGGAGAAATACTTTCAGAGGATGTACAATACGAACTGTAATTATTTGG
AATTTGATCCTTTTGTAAAGGACAATGACGACTTTGCAATGAATTATTCTGAGAATTACAGA
AAGAAAGCAAACACTTGAGAATAATAAAAAGATTTTCACCTGGGGAAAAATGGTAAGCTCC
CAAACTCAGAAGAATCATTGTTAGGCAAAAAAGGAAAAACAAAGAAGGAAAAAAGGCTTT
AAAATGCTAACAAACGGGTGGCAAAGACCGTATAAGATGGCAGAGTGGATCAATCAAATGA
AAGAATATGTTTTTCTTGGAATGAAGAAATACATAATAATGTACTGCCCTTGAAGTGATTAG
GACTAATTTTGCTTTTAGCATAGATTGTTCGTTGAGATCACTAGCTAAGGAATTTAATATTCT
TAGTATTTACAAACTAAACTTCACAGTCGAATTTTTTAAGGAAAAATTCACAAAATTTTAAA
ATGAAAAAGAAATGCTTTTGGATTGCGGGGGACGACCCGTGAAGGGTTAAGTCTTGGGAGC
TCCCTGGCAGGTATGCCAGGCCCTAGGACACGTGCCTAAGCTCCCTGTCCCGCCACCCTCAA
GAGTTTTTATAACCCTTAAGGCTCCAAGATGTTTGGTTTCGGCAACATTTCATAGAAGATAG
ATTATCTTATTGTGTATATTTCATAGAAGATAGATATTCTGATTGTGTTCTGTATACAATGGT
AAGGGTCTGGTGATTGTATCCTGAGATTAAAAAACAACCTTGTGAGTGCCTTAAGTCACGTA
CTTTACCCTATATATACTGCAGCACAATAAAGCAAGGTATCAGCCATTTTGGGGCTGATCCT
CTCAACCCCATCTTTTGTCTATCTCTTATTTTCTTAGCGGGGACGCTCCGTTCTCTCCCTGTGC
AGGTGCGACTCTTGCTTGTGCTGGCCGCGGCAGGTGGCGCCCAACGTGGGGCTCGAGCTCGA
CAGTTTTCCTCGCCACTACTCTTATTAATTGAAAAGAGTGAGTATATGAGTAAACAAGTGAA
TTAAATTGAGGAGGAGTAGTAAGGTATATAGTTGAGAGTATAAATATGGGACAGACGCATA
GTCGCCAGTTGTTTGTGCATATGTTATCTGTAATGTTAAAACATAGGGGAATTACTGTTTCTA
AACCTAAATTAATCAATTTTCTTTCATTCATCGAGGAAGTTTGCCCTTGGTTCCCCAGAGAAG
GTACAGTAAATTTGGAGACATGGAAGAAGGTAGGGGAACAAATTCGGACTCATTATACTTT
ACATGGCCCTAAAAAAATCCCTGTCAAAACTTTATCCTTTTGGACACTAATTCGTGACTGCCT
GGACTTTGATAATGATGAATTAAAACGTTTAGGAAATTTATTAAAACAGGAAGAAGATCCTC
TCCATGTTCCTGATTCGGAACCCAGATATGCTGTTCCCGAGGGGGTTAAAAGCGACCCTCCG
TTTTCTAACTTATTGCATCCTTCAGATAATGATGATTTACTTTCATCCACAGATGAGGCAAAA
TTAGACGAAGAAGCTGCTAAATACCATCAAGAAGATTGGGGTTTTTTAGCACAAGAAAAGG
GGCGTTAACATCTAAAGATGAATTGGTTGAATGCTTTAAAAACCTCACTATTGCTTTACAGA
ACGCAGGAATCAAGCTTCCTAGTAACAATGCCAAATCTCCTTCTGCTCCGCCTCTTCCCCCTG
CTTATGCTCCTTCTGTTGTGGCTGGTCTCGATCCCCCTCCAGGGCCCCCTCCACTGTCTGAGA
ACATGTCTCCGCTGCAAAAGGCATTGAGACAGGCACAGCGACTTGGTGAGGTTGTCTCTGAT
TTTTCTCTTGCTTTTCCTGTCTTTGAAAATAACAACCAGCGTTATTATGAATCACTGCCTTTTA
AACAACTGAAAGAGTTAAAGATTGCTTGCTCACAATACGGTCCTACCGCTCCATTCACCATT
GCTATGATAGAAAATTTGGGTACTCAAGCTTTACCTCCAAATGATTGGAAGCAGACAGCTAG
GGCATGTCTCTCAGGGGGAGATTATTTATTATGGAAATCTGAATTTTTTGAACAATGTGCTCG
TATAGCTGATGTTAACCGACAGCAAGGTATACAGACCTCCTATGAAATGTTGATTGGTGAAG
GCCCTTACCAGGCTACTGATACTCAACTTAATTTCTTACCTGGTGCATATGCACAAATATCAA
ATGCGGCTCGGCAGGCATGGAAAAACTTCCTAGCTCCAGTACTAAGACAGAGGATCTTTCAA
AAGTCCGGCAGGGACCTGATGAGCCTTACCAGGACTTCGTGGCACGACTTTTAGATACTATA
GGTAAGATAATGTCAGATGAAAAGGCTGGGATGGTACTGGCAAAACAATTGGCTTTTGAAA
ACGCTAACTCTGCTTGTCAAGCTGCTTTAAGACCTTATCGAAAAAAGGGAGATCTGTCTGAT
TTTATTCGCATTTGTGCTGACATTGGACCCTCCTACATGCAAGGCATTGCTATGGCAGCAGCA
TTACAAGGAAAAAGCATAAAAGAGGTACTTTTCCAGCAGCAAGCCCGGAACAAGAAAGGAC
TTCAAAAGTCAGGTAATTTGGGTTGCTTTGTTTGTGGTCAGCCTGGCCATCGGGCTGCAGTGT
GCCCTCAAAAACAACAAAGCCCTGTTAACACTCCTAATTTGTGCCCACGCTGTAAAAAAAGG
AAAGCATTGGGCGCGGGATTGCCGTTCCAAAACGGATGTTCAAGGTAATCCTTTGCCCCGGT

TTCGGGAAACTGGGTGAGGGCCAGCCCTGGCCCCGAAACAATGTTATGGGGCAACACTGCA
GGTTCCAAAAGGACCATTGCAGACCTCTGTCGAGCCACAAGAGGCAGCGCGGGATTGGACC
TCTGTGCCACCTCCTACACAGTATTAACTCCCGAGATGGGGGTCCAAACCCTTGCCACAGGA
GTGTTTGGGCCTTTACCTCCAGGGACAGCTGGACTGCTTTTAGGGCGCAGCAGTGCGTCTTT
AAAAGGAATACTTATTCATCCTGGTGTGATTGACTCTGATTATACAGGAGAGATAAAAATAT
TAGCCTCCGCTCCTAACAAAATTATTGTAATCAATGCAGGACAGCGTATAGCTCAACTTCTTT
TAGTTCCATTAGTCATACAGGGAAAAACAATTAACCGAGACCGTCAAGATAAAGGTTTCGG
GTCCTCTGACGCCTATTGGGTGCAAAATGTTACCGAGGCACGACCAGAACTTGAGCTACGCA
TTAATGGTAAGCTTTTCCGAGGAGTGCTTGATACAGGGGCCGATATTAGTGTTATTTCTGATA
AATATTGGCCTACTACATGGCCAAAACAGATGGCTATTTCCACTCTCCAGGGTATTGGCCAA
ACTACCAATCCAGAACAGAGTTCATCCCTTCTTACTTGGAAAGATAAAGATGGACATACAGG
CCAATTTAAACCTTATATTCTGCCCTATCTTCCAGTTAATCTATGGGGGCGTGATATATTGAG
CAAAATGGGTGTTTATTTATATAGTCCTTCACCCACTGTGACAGATTTGATGTTAGATCAGGG
CTTACTTCCAAATCAAGGTTTAGGTAAACAACATCAAGGCATCATTTTGCCCCTTGATTTAAA
AATCTAATCAAGATCGAAAAGGCTTGGGGTGTTTTCCTAGGGACCTCTGATTCTCCTGTGAC
ACATGCCGATCCTATTGATTGGAAATCTGAGGAACCGGTATGGGTCGATCAGTGGCCCCTAA
CACAGGAAAAACTTTCTGCCGCACAACAGCTGGTGCAGGAACAGCTGAGACTTGGGCATAT
TGAACCCTCTACCTCTGCTTGGAATTCCCAATTTTTGTTATTAAAAAGAAGTCTGGGAAATGG
AGATTGCTACAAGATCTTCGTAAGGTAAATGAAACAATGATGCATATGGGAGCCCTACAAC
CTGGGTTGCCCACTCCTTCTGCTATACCTGATAAATCCTATATCATTGTTATAGATTTAAAAG
ATTGTTTTTACACTATTCCTCTTGCACCTCAAGATTGCAAAAGATTTGCTTTCAGTTTACCCTC
TGTTAATTTTAAAGAGCCTATGCAACGCTATCAATGGAGAGTTCTCCCGCAAGGAATGACTA
ATAGCCCTACGCTGTGCCAAAAATTTGTTGCTACAGCAATAGCTCCGGTTCGTCAACGTTTTC
CTCAGCTATATTTGGTTCATTATATGGATGATATATTACTAGCTCATGCTGACGAACATCTAT
TGTATCAAGCTTTTCGATTCTAAAACAACATTTAAGCCTTAATGGTCTTGTTATTGCTGATGA
AAAATTCAGACTCATTTTCCTTATAATTATTTGGGTTTCTCCTTATATCCTCGTGTTTATAATA
CCCAATTAGTACAATTACAGACTGACCATTTAAAAACTCTAAATGACTTTCAAAAACTTTTA
GGAGACATTAATTGGATACGTCCTTATTTAAAATTACCCACTTATACCTTGCAGCCATTATTT
GACATCCTTAAAGGTGACTCTGATCCTGCGTCACCCCGAACACTTTCTTTAGAAGGACGAAC
TGCTTTACAATCAATAGAAGAAGCTATTAGACAACAACAGATTACTTATTGTGATTACCAAC
GATCATGGGGTTTGTATATACTTCCTACCCCCCGAGCACCCACAGGGGTTCTCTATCAAGAT
AAACCTTTGCGATGGATATATTTGTCTGCTACTCCAACTAAACATCTGCTCCCTTACTATGAA
CTTGTTGCAAAATTGTAGCAAAGGGACGTCACGAGGCCATCCAATATTTTGGTATGGAACCC
CCTTCATTTGTGTTCCTTATGCTTTAGAACAACAAGATTGGCTTTTTCAATTTTCAGATAATTG
GTCTATAGCTTTTGCAAATTACCCGGGACGGATTACTCATCATTACCCTTCTGATAAATTGTT
ACAATTTGCTAGCTCTCATGCCTTTATTTTTCCAAAAATAGTTCGCCGACAACCTATTCCCGA
AGCGACACTTATATTTACAGATGGATCTTCTAATGGAACTGCAGCTTTAATCATTAACCATC
AAACCTATTACGCACAAACCAGTTTTTCTTCTGCTCAAGTTGTGGAATTATTTGCAGTCCACC
AAGCGTTGCTAACTGTACCTACTTCCTTCAATTTATTTACAGACAGCTCCTATGTGGTCGGTG
CCTTACAGATGATTGAAACTGTTCCAATTATCGGCACCACCTCTCCTGAAGTTCTTAACTTAT
TTACATTGATTCAACAGGTTCTCCATTGCCGCCAACACCCCTGTTTCTTTGGACATATTCGTG
CACACTCCACCCTTCCTGGTGCCCTCGTACAAGGCAATCACACTGCGGACGTTCTTACTAAA
CAAGTGTTTTTCCAATCAGCTATTGATGCAGCCCGAAAATCCCATGATTTACATCACCAAAA
TAGTCAGTCTTTACGCTTGCAATTTAAAATTTCCCGTGAAGCTGCACGGCAAATTGTTAAATC
TTGCTCTACTTGTCCTCAATTCTTTGTTCTCCCTCAATATGGTGTCAACCTCGAGGTTTACGCC
CTAATCACCTCTGGCAAACAGATGTTACTCACATTCCTCAATTTGGGCGTCTTAAATATGTTC
ATGTTTCTATTGACACTTTTTCCAATTTTCTCATGGCTTCCCTTCACACTGGAGAATCAACAC

GTCACTGTATTCAACATTTGCTGTTTTGCTTTTCTACTTCAGGAATCCCACAAACCCTTAAAA
CAGATAATGGACCTGGTTATACTAGCCGTTCTTTTCAACGTTTTTGTCTTTCTTTCCAAATTCA
TCATAAAACAGGAATTCCTTATAATCCACAGGGACAAGGTATTGTGGAACGAGCCCATCAA
CGCCTTAAACATCAATTATTAAAACAAAAAAAGGGGAATGAACTGTATAGCCCCTCACCGC
ATAACGCCTTAAACCATGCTCTTTATGTTTTAAATTTTTTAACTTTAGACGCAGAAGGCAATT
CAGCAGCCCAGCGTTTTTGGGGAGAACGATCCTCATGCAAAAAACCACTTGTGCGATGGAA
GGATCCACTTACCAATCTGTGGTATGGGCCAGACCCTGTACTAATATGGGGACGAGGGCATG
TTTGTGTTTTTCCACAGGATGCCGAAGCGCCGCGCTGGATTCCGGAAAGGCTGGTACGCGCA
GCAGAGGAACTCCCTGACACATCAAATGCAATGCATGACACTGAGTGAGCCCACGAGTGAG
CTGCCTACCCAGAGGCAAATTGAGGCGCTGATGCGTTATGCTTGGAATGAGGCTCATGTACA
ACCTCCAGTGACACCTACTAATATACTGATCATGTTATTATTATTGTTACAGCGGATACAAA
ACGGGGCAGCTGCGGCTTTTTGGGCATACATTCCTGATCCGCCTATGATTCAATCCTTAGGAT
GGGATAAAGAAACAGTACCTGTATATGTTAATGATACAAGTCTTTTAGGAGGAAAATCAGA
TATTCACATTTCTCCTCAGCAAGCCAATATCTCCTTTTATGGTCTTACTACTCAATACCCTATG
TGCTTTTCTTATCAATCACAGCATCCTCATTGTATACAGGTGTCAGCTGATATATCCTATCCT
CGAGTGACTATTTCAGGCATTGATGAAAAAACCGGAAAGAGATCGTACCGTGACGGAACCG
GACCCCTCGACATTCCGTTTTGTGACAAACATTTAAGCATCGGCATAGGAATAGACACTCCT
TGGACTTTATGTCGAGCACGAATTGCATCGGTGTATAACATCAACAATGCCAATACCACCCT
TTTATGGGACTGGGCACCTGGAGGAACACCTGATTTCTCCGAATATCGAGGACAGCATCCAC
CCATTCTTTCTGTAAACACTGCTCCTATATTTCAAACTGAACTGTGGAAACTTTTGGCTGCTT
TTGGTCATGGTAATAGCCTATATTTACAGCCCAATATTAGTGGGAGTAAATATGGTGATGTG
GGAGTTACAGGATTTTTATATCCCCGAGCTTGTGTTCCTTACCCATTCATGTTGATACAAGGC
CATATGGAAATAACACTGTCATTGAATATTTATCATTTAAATTGTTCTAATTGCATACTTACT
AATTGCATTAGAGGTGTAGCCAAAGGAGAACAAGTTATAATAGTAAAACAACCTGCTTTTGT
AATGTTACCTGTTGAAATAACTGAAGAATGGTATGATGAAACTGCTTTAGAATTGTTACAAC
GCATTAATACGGCTCTTAGCCGTCCTAAAAGAGGTCTGAGCCTGATTATTCTGGGTATAGTG
TCTTTAATCACCCTTATAGCAACTGCTGTTACTGCTTCTGTATCTTTAGCACAATCCATTCAA
GCTGCTCATACTGTAGATTCCTTGTCATATAATGTTACTAAAGTAATGGAACTCAAGAAGA
TATAGATAAAAAATAGAAGATAGATTATCAGCTTTATATGATGTAGTTAGAGTTCTAGGAGA
ACAAGTTCAGAGCATTAATTTTCGCATGAAAATTCAATGCCATGCTAATTATAAATGGATTT
GTGTTACAAAAAGCCTTACAATACTTCTGACTTTCCGTGGGATAAGGTGAAAAACATCTGCA
AGGAATTTGGTTTAATACTAATGTTTCTTTAGATCTTTTACAATTGCATAATGAAATTCTTGA
CATCGAAAATTCTCCAAAAGCTACTTTGAATATAGCTGATACCGTCGATAATTTTTTACAAA
ATTTATTTTCTAACTTTCCTAGCCTTCATTCACTGTGGCGAAGTATAATTGCTATGGGCGCGG
TTCTGACTGTTGTGCTTATCATAATTTGTTTAGCTCCTTGCCTTATTCGTAGCATTGTTAAAGA
ATTTCTACATATGAGAGTTTTAATACATAAAAACATGTTGCAACACCAACATCTTATGGAGC
TTTTAAAAAATAAAGAGAGGGGAGCTGCGGGGGACGACCCGTGAAGGGTTAAGTCTTGGGA
GCTCCCTGGCAGGTATGCCAGGCCCTAGGACACGTGCCTAAGCTCCCTGTCCCGCCACCCTC
AAGAGTTTTTATAACCCTTAAGGCTCCAAGATGTTTGGTTTCGGCAACATTTCATAGAAGAT
AGATTATCTTATTGTGTATATTTCATAGAAGATAGATATTCTGATTGTGTTCTGTATACAATG
GTAAGGGTCTGGTGATTGTATCCTGAGATTAAAAAACAACCTTGTGAGTGCCTTAAGTCACG
TACTTTACCCTATATATACTGCAGCACAATAAAGCAAGGTATCAGCCATTTTGGGGCTGATC
CTCTCAACCCCATCTTTTGTCTATCTCTTATTTTCTTAGCGGGGACGCTCCGTTCTCTCCCTGT
GCAGGTGCGACTCTTGCTTGTGCTGGCCGCGGCATTGGATGAGAATTGTTTTTAATTGGTCAT
TTGTTTATACATTGTTGATTGCTCTTCGGATGAAAATTGAAAAGCGTGCCGTCACTCTAGTAC
CACCTAAAAGCAGAGATCTGGAGTGGTGAGCAGGGAGGTGCTGGTGGAGGAGTGATGCTGT
TTCCACACCAGGTTCCCGGAATCTGAAATCATGCCTAATCCAAAATGACTCTTTCAATCACA

AACAATGAATTGTTTGTCAGGAAAAGGAAATAGTCTAAACTAGGGTAAGTGTGCCGGGAAT
ATAAAAAGACATACAGTTTAGTAAATAGTTTTACTGTTTTGTGTGCTGCATGTTGAAGTCTAC
ATATTGACTAAGCCTCTGGAAACTTTCTCTCCATTACTGTGTTCTCGTGCGTACTAATATCCC
TCCCTTTTGATTTAGGTAAAATGAAATGGGAGAAATTTGGGAAAATTAATTAAAATTAACCC
ATTACATTTCTCTCTGACAGGTAATAAGTATGCCATTGGTTCCACGATGCACTTTCTAGTCAT
TGTGGATCAGGGCAAGGTGAATTGAGTTTTGTGTCTTCTGACAAATTTTGTCTTCTGGAGAAT
AAATAACACTATTAAATTGGGGCTTGGTATTACATTGTTAAATAGTTTCAGATGTTTTGAAAT
ATATCCAGAGACCTTTGTCACACACGGCCATCTCAAGGAGGAAATGAGAGAAAATTGGCAA
ACGTTCTGGAGGTGACATAGCAGACAGCTTGGCTGCCTTCATCGACGACTCCGCAGTGAGCA
CAAATAGAAGAGCATTGTGCATCCGTCAGTGCTTGTGAACTTTAACAGTGGAGTGAGGACA
GTTTCAAAAGTGATGAGTTTGATCTCAGTGTTGTTTGCTCTTATTAAATCTTCCAAACTTCAA
TCTTACATTCCTCCCATCTTAATTTTCCCAAATTTCTCCCATTTCATTTCACCTAAATCAAAAG
GGAGGGATATTAGTACGCATGAGAACACAGTCTAATTGAAATTCATTTGGAGGTTCACTTCA
TTCTATGTAGAACTTCATTATTACTCTATTTGAAATGTAACAGATGCTGTTATAAGGGGTCTG
TCTTGTATCTCACTCTAAGAAGCTTTGGAAATAAAGAAATAAAAGGTTATTCAGTACAGTGG
TCCCATCTCATGGACCCTAAATACAGTATCTTCTCAGAGTAGGCATACACTTTAGTAAGTGG
CATCTTTCCTACAAGTATTCTTACTTTGCTGCTAAGTCATTTCAATCATGTTCAACTCTGTGTG
ACCTCATAGATGGCAGCCCACCAGGCTCCCCTGTCCCTGGGATTCTCCAGGCAACAACACTG
GAGTGGGTTGCTATTTCCTTCTCCAATGCATGAAAGTGAAAAGTGAAAGTGAAGTCACTCAG
TTGTGTCCGACTCTTCATGACACCATGGACTGCAGCCTACTAGGCTCCTCCATCCATGTGATT
TTCCAGGCAAGAGTACTGGAGTGGGGTGCCATTGCCTTCTCTGATTCTTACTTTGATGGGACC
CTAAAACAACAGAAACCTACTTTGGTTGAATACATAATAGAGTCATCCTCTTTCAGATAAAC
TTATTTCTCGATGCATTTGTTGAAAAATATTTGAGTACTTACTCTATGTCAGACATTGTTGTA
AACTACAGAAAACATTATTAGATACCTTATTGTGCTGAACACTGACAAGGATCCAGGATCTT
TTTCTTTTCAGGTGGTGATTCTAAATTTTGTTTCTATGGAATGAAACCTTTTGATGAAAGTGA
AAAAAGAAAACCTTAAGTTAGTAAATGATGAAGACACAAGTTGAATTCTGTGGATACTTTGC
CACCACTGCACACAAACAGACCCTGTAAGGGCCTATCAATCTCCTGAAGAAAACAGGCCAA
ACAAGTGACTTAAAAATAATACAATATTTACAGGAGGAATAGAAGGAAGATACCAGAGTGA
GCATCTTGCTGTTGCCGTTATAAATGCATAATTGACTCTTAAAATTCTAGTAACTAACATATA
TAACACATGGAAATACCTCCAAAACATAATAACTGTACTGTGTGCTCTAAGGCTTGTTGTTG
TTATTGCTCAGTTGCTCAGTTGTGTCTGACTCTTTGTGATCCCATGAACTGTAGCATGCCAGG
CTTCCCTGTCCTTCACTATCTCCTGAATTTGCTCAAACTCATGTCCATGAGTTTGAGACGATG
ATACCATTCAACCATGCCATCCTTTGTCGCCTCCTTATCCTCCTGTCCTCAATCTTTCTAAGCA
TCAGGGTTTTTTTTTTTGCAATGAGTCAGCTCTTTGTATCAGGTGGCCCAAGTATTGGAGCTT
CAGCATCAGCATCAGTCCTTCCAGTGAGTATTCAGGGTTGATAACCTTTAGGATTGACTGGT
TTGATCTCCTTGCTATTCAAGAAACTCTCAAGAATCTTCTCCAGCACTGCAGTTGAAAAGCAT
CAATTCTTTGGTGCTCAGCCTTCTTTATGGTCCAACTCTCACATCCATACATGACTACTGGAA
AACCATAGTTTTGACTAGATGGACCTTTGTCAGAAAAGTGATGTCTCTGCTTTTTAGTACCCT
GGCTAGATTTGTCATAGCTTTTCTTCCAAGAAGCAAGCACCTTTTAATTTCATGGTTGCGCTC
CACAGTGTTTTTGGAGCCCAAGAAAATAAAGTCTGTCAATGTTTCCATTTTTCCCCATCTATT
TGCCATGAAGTGATGGGACTGGATGCTATGATCTTAGCTTTTGAATGTTGAGTTTTAAGCCA
GGTTTTTCTCTCTCCTATTTTGCCTTCATCAAGAGGCACTTTAATTTCTCCTCAGTTCCTGCCA
TTAGGGTGGTGTCCTCTGCATATCTGAGGTTATTGATATTTCTCCTGACACTCTTGATTCCAG
CTTGTGCTTCATCTACTCTGAATATAAGTTAAATAAGTAGGGTGACAATATACAGTCTTGAT
GTACTCATTTCCCAATTTGGGACCAGTCCATTGTTCCATGTCTGGTTCTAACTGTTGCTTCTTG
ACTGACCTACAGGTTTCTCAGGAGGCAAGTAAGATGGTCTAGTATTCCCATCTCTTTCAGAA
TTTTCCAGTTTGTTGTAACCCACATAGTCAGGGATTTAGTGTAATCAATGAAGCAAATGTAA

ATGATTTTCTGGAATTCCTTTGCTTTTTCTATGATTCAATAGATGTTGGCAATTTGATCTCTGG
TTCCTCTGCATTGTCTAAATACAACTTGTACATTTGGAAGTTCTCGGTTCATATACTGTTGAA
GTCTAGCTTGAAGGATTTTGAGCATTACCTCGCTAGCATGTGAAATGAGTACAACTGTGTGG
TAGTTTGAACATTCTTTGGCATTGCCTTTCTTTGGGGTTGGAATGAAAACTGACCTTTTCCAG
TCCTGTGACCACTGCTGAGTTTTCCAAATTTGCTGGCATATTGAGTGCAGCACTTTAACAGCA
TCGTCTTTTAGGATTTTAAATAGTTCAACTAGAATTGCATCACCTCCGCTAGTTTTGTTCATA
GTGATGCTTTCTAAGGCCCACTTGACTTCACACTGCAGGATGTCTGGCTCTAGGTGAGTGAC
CATACCGTCATGGTTTTCTGGGTCATTAAGACCTTTTTTGTATAGTTCTTCTGTGTATTCTTAC
CACTTCTTCTTAATCTCTTTTGCTTCTGTTAGGCCCTTCCTGTTTCTCTCCTTTATTATGCCCAT
CTTTGCATGAAATATTCCCTTGACATCTCTAATTTTCTGGAAGAGATCTCTAGTCTTTCCTAG
TCTATTGTTTTTCTCTATTTATTTTCATTGTTCACTTAAGAAGGCTTTCTAATATCCCCTTTGG
AGAGAGAAGCTTTGAAACATCTGGAGTTCCAAAGCTATTCTTTGGAATTCTCCATCAACTGT
GTATATTTTTCCCTTTCTCCTTTACCTCTCACTCCTCTTCTCGTCTCAGCTATTTATCTCAGCTA
TCTTATTTCAGACAACCTTTTGCCTTCTTGCATTTCTTTTTCTGGGGGGATAGTTTTGGTCACT
GCCTATGTATAGTGTTATGAATCTCTGTCCATAGTTCTTTAGGCACTCCATCTATCAGATCTA
ATTCCTTGAATCTATTCATCATCTCCACTGTAAAATCATAAGGGATTTGATTTAGGTCATACC
CAAATGGCCTAGTGTTTTTTGTTACTTTATTCAATTTAAGCCTGAATTTTGTGATGAGAAACT
GATGTCCTGAGTCACAGTCAGCTCCAGGTCTTGTTTTCACTAACTATATACAACTTTTCCATC
TTCAGCTACAAATGATATAATCAATCTGATTTTGGTATTGACCATCTGGTGATGTCCACATGT
AGATTCATCTCTTGTGTTGCTGGAAGAGGTGTTTGCTATGATCAGTGCATTGTCTTGGCAAAA
CTCTGTTAGCCTTTGTCCTGCTGCTTTTTTGTACTCCAAGGCCAGACTTGCCTGTTACTCCAGG
TATCTCTTGACTTTCTACTTTTGCATTCCAGTCCTCCATGATGAAAAGGACATCTTTTGTTCGT
GTTAGTTCTAGAAGGCCTTGTAGGTCTTTATAGCTTCTTTGGCATAAGTGGTTGGGGCGTAGA
CTTGGATTACTGTGATATTGAATGGTTTGCCTTGGAAACAAACGAAGATCATTCTGTCACTTT
TGAGTCTGCACCTAATTACTGCATTTCAGACTCTGTTGTTGACTCTGATATCTACTCCATTTCT
TCTAAGGAATTCTTGCCCACAGTAGATATAATGGTCACCTGAATTAATTTTGCCATTCCCATT
CATTTTAGCTTAGCTTCCTAAATTTACTTCTGATGGAAGTGTTTTTGTCTGCAGTGGGGACAA
ATGTAATCTAAATTGTAGTTGGGTCTCTTGGTGGAAAGACTCTAGTTTGCATGGAAACATCT
AGTTTTTATGAGCCCAGTCTTTAAGAGCTGCTTGGTCAAAAGTTACATCCAAGCAGACACGT
AGACAGGGAATTCCAATGCATTACCTTGAGCTGTGATAAGGCCAGTGCTCAGAGATACCAG
CTCTGTTCTTTGATAAAAGGCGTCTGTTTACAGACCTTTGCTATTATGTGGAGTCTGCACTTC
TTGCTGGGTTGTGAACTCCATGAAGGCTGGGTTTCGTTTGCAACTTAGTATCAGCAATATCCA
GTGCTGGATACATAGTAAGCACTTAATAAACAGATAGTACCTATTTATTCAAAAATAGGTGT
AAATCTTTATAACCCAAAAGGTTCACCTTCCAATGAGGTCTACATTAGGATCTTAAGAATTTT
TAGTCATGTAAAACAATTTAAAATCTTTTATCTCCATTTCTTATGCTTAGCACTTAAAAAAAA
TATCCAACTTGACATAAATCTCCACCATAAAGTAATACATAGATGTGTGCAGTTTTGTGAGT
GAGAATTCTCAGGAAAGCAGTTTCATGGAATATTACCATTACCAGTACCACCCTCACCTTTC
TCAGCACGTCTGTTTTATTTTATTTTTTAACTCTCTCTCTCTCTGTGTAGGGTGGATCATCTAT
TAAAATTATAAAATTTAACAGAAATATTCTGTTTCATGCTACGCTAAGTATGCAAATTCATA
GACACTGATAATTTCTCAATGAACCAAATCCGTGACGCTCACTCATCTCACCTAATGCCATG
AAGACCTTATCGTTTTTCCCATTCTACGTGCAGAGCTTAGATATGTTTGCTTCTGCGTTCCGT
CTCCTAGGTGTCTACAGTATATCTACCAAGTGTTTAGATAAAGCCAGAGAAAAATTCAGTCG
TTCCACTCTGGGTCCAATGGGCATTTTTAAGATTTGCTAACAGGGAAAGTACAGGTTTTGATT
TCTCCACTTCTAACTTCACGGGAAACTGTAGCATAGTAGATGTTCTTTGTTAGCCTAATCACA
CATACCGCCAAAACCCTCCCTTGAGGTGGATGTAATGGCCACAGCAAGTGGAGAGATACAG
ACATCACTCTCTGTTTTCTTTCTCAATTCATTGGTCAGAGTTGCGTCCTGACCTCATTGAGAG
TTTGTCTCCTGTTATCCAAACTTCTTTGTTTCACATACTATAGTTTAGTTATAGCAGTTCAGCA

AAATGCTATGTTTTACCGGAAGAAGTCAGGAAAACCAGGATTAGGCAAGGAAAAAAAGCAA
ATGCTAATATCAGGGGGCAAACAGAAAGGAAGCTTACCATAATGATCTCCTCCCTCTTCTCT
GCCTCATTAATATTCCTCCTGTGTATGAAGGATGCCATTTTGTTTTCTTCCTCACGTCTCCCAC
GCAGAACTCTGTACATAGTAACAGCAGGAGCATTTGTGAAGCCGAAATCCTGTAGAATAAA
TAAAAGCAATTGAAACCATCTGAATGGGCTTTTGTGAGAAGAAAACTTGTTTAATAATAATT
TACCATGGCAGAGCAGCAGCTATGAAGCTTAGCTTTCTCCCTCTGGTTTAAGGCTAAAAGTC
ACCTTTCAGCAGGTCTTCCAATGTTCACATTTGTTCACAACAGAAGCTGCAGACATTGGAAG
TGAGAATAGTTCTTGGCACAGTCTTAAACCATGATTCACTAATTTTTTTTTTTTTTTTAACAA
GTGAGGACTCTGAGGCCCAAAGAAGTGAAGTGAGCTTTCACGTCTGGGCCTAGCCTTTGGAT
TGCCCTTTCTGCAGTTTTTCTAGGCTCCTCCTTCCATTCAACACCAACATCTATTTTCTTATGG
TTTCAAACTATAACCTAGCTGTTGAAGAGACCCAAATCTTTTCACCACCCCAATCTCTTCCCT
GATTATTAGACTCATCCATTTATGTGCCTACTGCTCAGGTTCTAGACATGGATGCATTACTCG
TGAGTTGAAAGTGTCTAACCTCTTTCAACACATTATCTTGTTACTGTGCTTTGATTCATGAGG
TGCATTTTATGCTCAAAATTGTAAACAAATACGATTGAGTTTTGTTTCCTACTCTGCTTGAGT
ATCTTCTAAAGTTCAGTAAAACATGCATATATATGTACTCTGCAAACACTTTTAAACTAACA
GGCTGGATTAAATTATAATAGCTAATTATAACCTACTGGTATGTTCAGGGCACTCATAATAA
TTATGAAAAATGCCTAGTATTTAATGAGAGGTACTGGGATTCACTGGTTGAGATACTCTCTG
TAGCTGGATTGAAAAGATTTGAAACCTGGAATCTGCCACTTTCCAGCTCTGTGAGCTTGGGG
AACATATTAAACCTCTTTGTATCTCTGCTTTGTCTGTTAAATAAGATTAATATAAGTACTGCC
TTTATGGACCAGTTGCTCAAAATAAGCAAGTGACTATGTAAAATATGCAAGTGCCTTGCACA
TAAGTTCTGAAGAAGGGTTAACTACCATTATTTAATTCTCACAATAGTTTTAAATAGGCCTTC
ATATAGGTACGAGTAAACATGCTCATAGAGATAAAGATATTTCCCCGGGTCCTGCAGTTAGT
GAGAATGCTAGATAGAGCTATCCTGTCCCACCCCAGTCAGTTCAGTCACTCAGTCATGTCCG
ACTGTTTGCGACCCCATGAATTGCAGCACGCCAAGCCTCCCTGTCTATCACCAACTCCTGGA
GTTCACTCAAACTCACGTCCATCAAGTTAGTGATGCCATCCAGCCATCCCATCCTCTGTTGTC
CTCTTCTCCTCCTGCCCCAACCCCTCCCTGCATCAGAGTCTTTTCCAATGAGTCAACTCTTTGC
ATGAGGTGGCCAAAGTATTGGAGTTTCAGCTTTAGCGTCAGTCCTTCCAAAGAACACCCAGG
ACTGATCTCCTTCAGAATGGACTGGTTGGATCTCCTTGCAGTCCAAGGGACTCTCAAGAGTC
TTCTCCAACACCACAGTTCAAAAGCATCAATTCTTTGGTGCTCAGCGTTCTTCACAGTCCAAC
TCTCACATCCATACACGACTACTGGAAAAACCATAGCCTTGACTAGATGGACCTTTGTTGGC
AAAGTAATGTCTCTGCTTTTCAATATGCTATCTAGGTTGGTCATAACTTTCCTTACAAAGAGT
AAGCGTTTTTTAATTTCATGGCTGCAGTCACCATCTGCAGTGAGTTTGGAGCCCCAAAAAAT
AAAGTCTGACACTGTTTCTCCATCTATTTCCCATGAAGTGATGGGACCGGATGCCATGATCTT
TGTTTTCTGAATGTTGAGCTTTAAGCCAACTTTTTCACTCTCCTCTTTCACTTTCATCAAGAGA
CTCTTTAGTTCCTCTTCACTTTCTGCCATAAGGGTGGTATCATCTGCATATCTGAGGTGATTG
ATGTTTCTCCTGGCAATCTTAATTCCAGCTTGTGCTTCTTCCAGCCCAGCGTTTCTTATGATGT
ACTCTGCATAGAAGTTAAATAAGCAGGGTGACAATATACAGCCTTGACGTACTCCTTTTCCT
ATTTGAAACCAATCTGTTGTTCCATGTCCAGTTCTAACTGTTGCTTCCTGACCTGCATATAGG
TTTCTCAAGAAGCAGGTCAAGTGGTCTGGTATTCCCATCTCTTTCAGAATTTCCACAGTTTAT
TGTGATCCACACAGTCAAAGGCTTTGGCATAGTCAATAAAGCAGAAATAGATGTTTCTCTGG
AACTCTCTTGCTTTTTCCATGATCCAGTGGATGTTGGCAATTTGATCTCTGGTTCCTATGCCTT
TTCTAAAACCAGCTTGGACATCTGGAAGTTCACGGTTCACGTACTGCTGTAGTCTGGCTTGG
AGAATTTTAAGCATTACTTTACTAGCATGTGAGATGAGTGCAATTGTGCAGTAGTTTGAGCA
TTCTTTGGCATTGCCTTTCTTTGGCATTGGAATGAAAACTGACCTTTTCCAGTCCTGTGTTCAC
TGCTGAGTTTTCCAAATTTGCTGGCATATTGAGTGAAGCAGTTTCATAGCATCATCTTTCAGG
ATTTGAAATAGCTCCACTGGAATTCCATCACCTCCACTAGCTTTTTTTGTAGTGAGGCTTTCT
AAGGCCCACTTGACTTCACATACCAGGATGTCTGGCTCTAGGTGAGTGATCACACCATCGTG

ATTATCTTGGTTGTGAAGATCTTTTTTGTACAGTTCTTCTGTGTATTCTTGCCACCTCTTCTTA
ATACCTTCTGCTTCTGTTAGGTCCAGACCATTTCTGTCCTTTATTGAGCCTATCTTTGCATGAA
ATGTTCCCTTGGTATCTTTAATTTTCTTGAAGAGATCTCTAGTCTTTCCCATTCTGTTCCTTTA
CTCTATTTCTTTGCATTGATCACTGAGGAAGGCTTTCTTATCTCCTCTTGTTATTCTTTGGAAC
TCTGCATTCAGATGCTTATATCTTTCCTTTCCTCCTTTGCTTTTTGCTTCTCCATTCTTTCTTTT
TATATTGTTGCTCTGAGTCTAAAACCTAGAATTCATTTTGTGTTTAAACCCCTCTACTTAGCT
CTACTAACCAAGTCTTGGCTGTTTATACTACCTTCTAAAATCCCTGAGTGACTCTATTGCATT
CAAGAGAAGTTTCTGAGAGAATGAATTCCTAGGTATTCAGGCAAAACCCTCAGCCTGGTATA
TTATGCATTACATTAGGGCTTCATGATGTCATTTGTGCCCACCTCTCCAGTGTCCTTTCTTTTC
CAAAAGTCGTACATTGTACCAAAGCCTTACCAAAGTGTATTACCCTGTTGTACAATTTCTTAG
GCATCTGTAGTTTAGGAGCATGCCTCTAATCCCTTTGCTGTTTCCTCCTTCCTTTCCTGGACCC
TGTGTACTATACGCAGTAAGCTATCACTTCTTCCACCAGCAGTCCTTTCACTTTCTTACATGA
CCCACCGTTTTTCTTCCCATCCAGGTGTAGGAGTCACAGTAAATCAATGTTATAATTGTCTGT
TTACAAAACTGTCTTCCTAGGGATGCTGTGGGCTACTTGCATGAAAATACCAGGTTTTTGTTC
TGTTGATTCCCTCATTTCATCCCAATTATTAGTCCAGGCTAAGCATGCTTAAGGAATTTATTT
TATTTTACAGTTTCAGTGGCTACAGGTTAATGAAACATTTAGCAGTGTAAATGGAAGAATAA
AAGTATAAACAAATGAGAAGCAAAGTTCATGATTCAAACTATTATAGAAAAGTAGCTTCAA
AAGTAATCTAATATTTTTTAAAAATGCAAAAAGCTTGATAGGGGAAATGATCCAATATGAAA
ACCAGTAAAAATCATTGCAAAAAGAATGGAGCTAGGATTTGTGTATCTTCTGGGTTGTGATC
CAGTGGTGAAGGAGCCAGACTAGAAGAAGTTGGTTCAGGTTGCAGGTGTGACTGCTTGGCA
AGTGCTTGCCATGTAGGCAAGAGGTTGAGGACAAGATAAGATTAGGTGAGGACATATACTA
TCATGGGTAAGCCAAGTTCAAAGGCTTACGTAGTTTTAAATTCTTCCTTCTTTTGGAAAATAA
GGACTACTAAGTGAGTGCTGGAAACTACAGAGTATGAAGTATTATCTCATAGCACTAGGCTC
AATTTTAATAGTTGTTTAAATCAAACAAGATTTAACACACTAGATCTCAAAATATTGACATG
CATGAGAGTGACTAAGAATGCTTGATGCTTAGGCCATGCTTAGGCTATTCTCTTGCTCTCTGA
ACATCCCTTGCTCAACCACTCCCTGCCCACCTATGCCCTACTCACATCCTACCTACCTTCCTT
GTTAATCACTGAATCTAATCAATAAGTGCCTACAAACTTGTCCCTGGCCCCAACTAGGGATT
CTTCCAATCTTTCTCTTCCTTTCCTTTGCTTCTTTTTTATTTTTATTTTTTTGGCTTTACCATATT
GTGTTAGTTTCTGCTGTACAAGGAACTGAATCAGTTATATGCATACATATATCCCCTCCCTTT
TGGACTGTCCTCTGCCCCACCCCCATCCCACCCATCTAGGTCATCACAGTCCAGGAGCTGAA
TAAAATAGATAGCTAGTGGGCACTGCTGTCCTAATCTTCTGATCAGAACATCTCCACCAAGA
TAGCTTATCAAAGGGGAATGAGGGATGCACTCCTCTATTGGTTTCTCTGGTAACTGATGACT
CGACCTGATGTCAACTGCTAACTTCCCTCTCCACATCCTCTGGCCCCCTGCCAGCAGACTGCT
ACCATGTCTTGCCCATTGTCTGCCCCACATGGTGGAGTGTCAATTCAGGAGCTTGCTTCTGAC
ATGTAAGATCCCCCATTCATTACACCACTGATGTGTCTGCCACTGACTTTGAGCTCTTTATTC
AGTCTTATGGCTGGGCAAGCACAGAGGGTGCAGTCCAAGTGTAGCCGTAATGCCTGCTTCTA
AGCAATTCATTGTTATATGTCATATTGTTGTTATAATTTTATAACTATCTTCATCATCATTGTC
AGTTACTCTTATTTTTTACATTTGTACCCTGATCTTCCCATCTTGCTGATTATCTGACATTTTA
ATCCCTTCTAAATACACACTTTGTCTTCTTAATCTCTTCTAAATTATGAAAATTCACCTACAA
CTATCTTGATAACACTTTACTGGGAGGGTTATTAGTTTCCTAGTGATATTTAGCTTTTCTTTCG
TGCTCCTCTTTCCATGGGATTTCCCAGGCAAGAACACTGGAGTAGGGTTGCCATTTCCTTCTC
CAGGGGGTCTTCCCAGCCCAGGGATCAAACTCACATCTCCTGCTTGGCAGGCAGGTTCTTCA
CAGCTGAGCTACCAGGGAAGCCATGGTTTTTACAGATATAATACATGTATAAGAAGAGTATA
TTTTCAACATAGTGGCTAAGACTTGGTATTCAGACTTAAATTTTCTTCTTATAACCCTTTCTTC
TTTCTAGCTGTGAAACAGGGTTATAAGACTCTTTGGTGTTTCAGGGTTCTTCAGCTCATGATC
TGCCTCTCCTGTGCCCTGAATCATGTTCTTCATCTCAGTGGGATATTTCGCTCCATACTTGAA
ACAGTAGCTTTAGCCTCAGAATCTTTGTCTAGTATCATTTTGCATACCCCATAATAATCCCTC

CTGTTCCTCATCACTGTGTCATCTACTAATGTTCCTTCTGTGAAGTTCTATTTGTTGTTGTTGT
TTTAGTTGCTAAGTTGTGTCCAGCTCTTTGCAATCCCATGGACTGTATGCCTGCCAGGCTCCT
CTGTCCATTGTATTTCCCAGGCAAGCGTATTGGAGTGGGTTGCCGTTTCCTTTTCCACGGGAT
CTTCCCTGCTCAGGGGCTGAAACCACATCTTCTGCATTGGCAGGTGGATTCTTTACTGTTGAG
CCACCAGGGAAGCCTGATGTTCTATTTACTCTTCTCCTAAATTCTGGGAAATTATATAACCAA
ATGCATCAGAACCACCTGATAACACAGGCATGAGGAGTCAAACTTTTTAGCTGCCTTATTAC
TGTCCTTGCCCTTAAGCCAGAGACTACCAAGAAGACACCTGTAGTTGAAAAAGTTGAGTTCA
TTACCTATTGCAAAGAGGAAAAAGTGTACCATGGGGTCAGAAGGCCTTAAAAAGAACCTCT
TATAAAATCTGAACTTCGGTTGAATGATCTGGGGAGAGAGTTTAAGGAAGTAGAGCATTACT
CTGAATTGAATGTTGTCAGGAAGCAGGGTAATTTCACAATCTGGTGCTTTAAAAGGTCTTAT
TTAGGAAGAGGGAAGATAAGCCTGCCTTGGTAATGCAGCAGCAGTGACCCAGATTAGCTGG
GAAAGGGGGACATTTGATGCCTGTGGCTTAGACAGGGTTTATAAGAGGTCTGTATTCAGACA
TAATTACAGAGCGTTTTGTTTTGTCTTGATTCTTCATGATCATATAGTGTCCTTGCCTAAAGCT
GATGTTCTGTGAAATTGCTTCTGTTCAACATGAGGATACCACAGCCAGGTGTGAGAGCCAGG
GCAGTGTCCAGAAACACTGTGATAGCACCGGCCCAGGTCCTGGATGTCAGGGGCTGTTTTCT
CTCTCATGAAAAAATTCTTTACTACTTCAGTAATTAAACTTATTTCCATGAAATCCACTTCCC
TCTACTTTGGTGATAAGATTCTAACAGTTCTTTGTAATTTCCTCCCTAAGCCCTATGGTATGC
TCAACCCATGGTGTATTTTAATACAAAATTTCATTTCTCTTAAGCTAGCAATATGATGATGAT
CAATAAGGACCACAAAAGTAGCTAGATCTATGTATAATAACTACAAAGGGCACAGATTGTT
TAGGGAACTGATTATTTTTTGTATTTATACCATAAACACTAATTTTTTGTGTAAATATAAACC
ATTGGCATATAGGAAATAAATGGAATAAAATTTTAATTTTATTTAGTGGAAGTTACTTGTGA
GGACTTTAAAGGGTCTTTCCACTTACTATATAGAAAAGAGTTTAATAATCAGAGACTGGAAT
TTTGGAGTTGGGAATTATGATGAATCAAAATATCTCTGGAGATATATACTTGGCTAAACATA
TAAATGTGGTTAAAACTTTACTACTTAGACATTCTAGATGCCTACTAAAAAAAGTGAGATAA
TCTGTTTCTTTTACATAATGGACTGTCATTATCCTAAGTTTTACTTAAAGAACACCTAATTAA
GTGATTTAAGTTCATGATTGAATAATCTTTAAGTAATGTATGGTTTTAAATGAGTAGAAACT
GGCAAATGTCCCATGCTAATAATTTGCTTTGTAACTTGTTACAGATATGTAGAGGAAGTGTTT
TGGGTGGAAGGCTCTGAAGAGAGAAGGAGATAAGGTTATATAAATCAGGCCTATTCTATAA
GTCACACATGAAAAAATATCTTCCACAATGTAATATTTAATGTATATGTAATATTGCTAAAT
ATAAGAATTTTTTTGACTTATATGATCTTTAAACATGAGTTATTTAATAGGCTCAAGATTACC
AATTTCATATGTAAGATTAAGTTTCTTCATCTTTGAGTTTTTTGATTGGTTTTAGAATAGAAT
AAAAGCAATAAAGAAGAAATAGTTACTTATTTTCCAACTCTGCTTTACTTATCATACGGTCTT
ATTAGTAAGGATAAGATGTTTTGATTGAACTTCATAAAATTTGGCCTTTATTTACCCTTGAAG
ATATGATCTAAACCAAGAAAGATGCTCATTTTTCATTGTCTCTTTACGGTTTACTCTTTTGTAT
GATTCTATTTCATCTTTCCTGTTTTCAAGTAAAATGTGTAGCTATGTTTTATGTGAACCAGAA
ATAATAATTTACAACAGGAAATGGAAGTTAAAAAGTTAAAATTCAAGTTAAATATTTAACTT
TTAATTAGACAATAAGGCTTAAATCATTTTAAAGACTATAGATACTTGTCATGGGCATAGAG
GTGGGTCTGTTCACTCAGTACAATCGAAGGCAGCACTTTGGATCCTTAAATCTGAATGGTTC
AATAATTAAACTACAGAAGATTCCTGATAACTGATTTAATTCTTTGTTAACAGTAATAAGTG
AGAATACAATAAATATCCTTGGCTACTTGGGAACTTGGCCTATTCTTGGAGTCTATTTTATGC
GGGGCCAAATTAGTATAATGTGCTTTTGGATATGTGTGTTCTTTAATATTACTGTCTAGAAGT
TTACTACAGATGAACTTATCTAACTGCTTTTTGTCAAAAGAAGTATTTTGAGTGCCCATTGAA
GCAGTAGGTACCTATCTGAAATGAAAAGGATTCAAGCCACTCAATTTTCCTTCCTGAAATAT
CCCTGCACAAGGTGTGCTTCTGCAAATTCTTAACACATCACAGATGGTCTCGAAACTGTTGTT
GCGACTGATGCCAGAATAAAAAGGTAGCTTTCTTATACTTGTTTACATATATTCCTTTCTTCT
TGGAGCATATCAGGCTTGGAGGCAAACAGTGGCAATGGTTTTCAACTGCAAAACCTATCCAG
ATGATTTCTTCCAGAGTCTCCCTTTTCCCCACTGTCATTAGGCTTAGATTCTCCTCTTTTCCAT

CCTATCCTTTTTGAACGGTCATAATGCATTTACATTTTATACATCTACTGGCCAAACGAGAAA
GATTACATGTTCAACAAAAAGCAGGCTCATATAGTCATTTTTTTCCATTGTTTCTTTCCAACA
AGGTCCAGTTATTGACTCTAGACTGAGTAAACCAAAGGATCGACGTTTCCTGCTTACTCTTCC
ATGGCAGTTTGCAGTGGACCTCTCCATCTCTTGATCTTTGTTCCTCACCCTCATCCCCACATCT
CTTCCCCAACTCTCATGAGACTGAGCATGGCCCTAAGTCCAGCAAACTCAGCCTGGAATGTG
AAGTGGGGAAAGTGAGAAGTTAGGAGATTAAATACTTCCTAATAAAGCTCAACATTCAGAA
AACAAAGATCATGGCATCCGGTCCAATCACTTCATGGGAAATAGATGGGGAAACAGTGTCA
GACTTTATTTTTTCGGGCTTCAAAATCACTGCAGATGGTGACTGCAGCCATGAAATTAAAAG
ACTCTTACTCCTTGGAAGGAATGTTATGACCAACCTAGATAGCATATTCAAAAGCAGAGACG
TTACTTTGCCAACTAAGGTCCGTCTAGTCAAGGCTATGGTTTTACCAGTAGTCATGTTCGGAT
GTAAGAGCTGGACCCATAAAGAAGGCCAAGTGCCGAGGAATTGATGCTTTTGAACAGTGGT
GCTGGAAGACTCTTGAGAGTCCCTTGGACAGCAAGGAGATCAAACCAGTCAATCCTAAAGT
AAATCAACCCTGAATACTCAATGGAAGGCCTGATGCTGAAGCTCCAATGCTTTGGCCACCTG
ACGTGAACAGCTGACTCATTGGAAAAGCCCCTGAGCTGAGAAAGATTGAGGACAGGAGGAG
AAGGGGGCGACAGAGGATGAGATGGTGAGATGGCATCATTGACTCAATGGACTTGAGTTTG
AGCAAATTCTGGGAGATAGTGAAGGACAGGGAAGCCTGGCTTGCTGCAGTTCATGGGGTCG
CAAAGAGTTGGACATAACTGAGTGACTGAACAATACTTTTCCTCCACTTCCAGTTTCAACTG
AACTCAGGATTTAATTGATGGAAAAAGCTATTAGCTTTTCCTGAAATGTAACCAGTCATTTTT
CTATTATTTAAGGTATCTAAACAGACACTATGGAGAAGGCAGTGGCACCCCATTCCAGTACT
CTTGCCTGGAAAATCCCATGGATAGAAGAGTCTGGTAGGCTGTGGTCCATGGGGTCGCTAAG
AGTCGGACAACTGAGTGATATCACTTTCACTTTTCATTTTCATGCATTGGAGAAGGAAAGGA
CAGCCCACTCCAGTGTTGTGCCTGGAGAATGTCAGGGATGGGGGAGCCAGGTGGGCTGACG
TCTATGGAGTTTCACAGAGTCGGACACTACTGAAGTGACTTAGCAGCAAACAGACACTAAG
AACTGTTTTATTGACCTGTTGAGACAAAATGACTCATAGTTAATCATAAATTCCTTTAATATT
GTTCAGTTCAGTTGCTCAGTGGTGTCCGATTCTTTGCGACCCCATGAATTGCAAAAGGCCAG
GCCTCCCTGTCCATCACCAACTCCTGGAGTTCACTCAGACTCATGTCCATCGAGTCGGTGATG
CCATCCAGCCATCTCATCCTCTGTCGTCCCCTTTTCCTCCTGCAATCCCTCCCAGCATCAGGG
TCTTTTCCAATGAGTCAACTCTTCACATGACGTGGCCAAAGTACTGGAGTTTCAGCTTTAGCA
TCATTCCTTCCAAAGGAATTCTTTCCAAAGGAACACCGAGGACTGATCTCCTTTAGAATGGA
CTGGTTGGATCTCCTTGCAGTCCAAGGGACACTCAAGAGTCTTCTCCAACACCACAGTTCAA
AAGCATCAATTCTTCAGTGCTCAGCTTTCTTCACAGTCCAACTCTCACATCTGTACATGACCA
CAGGAAGAACCATAGCCTTGACTAGATGGACCTTTGTTGGCAAAGTAATGTCTCTGCTTTTA
AATATGCTATCTAGGTTGGTTATAACTTTTCTTCCAAGGAGTAAGTGTCTTTTAATTTCATGG
CTGCAGTCACCATCTGCAGTGATTTTGGAGCCCCCCAAAATAAAGTCTGATACTGTTTCCACT
GTTTCCCCACCTATTTCCCATGAAGTGATGGGACCAGATGCCATGATCTTCGTTTTCTGAATG
TTGAGCTTTAAGCCAACTTTTTCACTCTCCTCTTTCACTTTCATTAAGAGGCTTTAGTTCCTCT
TCACTTTCTGCCATAAAGGTGGTGTCATCTGCATATCTGAGGTGATTGATATTTCTCCCAGCA
ATCTTGATTTCAGCTTGTGATTCCTCCAGCCCAGCATTTCTCATGATGTACTCTGCATATAAG
TTAAATAGCTGGGTGACAATATACAGGCTTTATGCACTCCTTTTCCTATTTGGAACCAGTCTG
TTGTTCCATGTCCAGTTTTAACTGTTGCTTCCTGACCTGCATACAGATTTCTCAAGAGGCAGG
TCAGGTGGTCTGGTATTCCCATCTCTCTCAGAATTTTCCACAGTTTATTGTGATCCACACAGT
CGAAGGCTTTGGCATAGTCAATAAAGCAGAAATAGATGTTTTTCTGGAACTCTCTTGCTTTTT
CCATGATCCAGTGGATGTTGGCAATTTGATCTCTGGTTCCTCTGCCTTTTCTAAAACCAGCTT
GAACATCTGGAAGTTCACGGTTCACATATTGCTAAAGCCTGGCTTGGAGAATTTTGAGCATT
ACTTTACTAATATGTGAGATGAGTGCAATGTGTGGTAGTTTGTACATTCTTTGGCAATGCCTT
TCTTTGGGATTGGAATGAAAACTGACCGTATATATGCACTAATTCCACATATATGCATTAAT
ATATGATATTTGTTTTTCCCTTTCTGACTTACTTCACTCTGTATTACAGTCTCTAGGTCCATCC

ACATCTATAAATGACCCAATTCCATTCTTTTTTATGGCTGAGTAATATTCCATTGTATATCTG
TACCACATCTTCTTTATCCATTTCTCTGTTGATGGGCATTTAAGTTGCTTCCATGCCCTGGCTG
TTGTAAATAGTGTTGCAATGAATATTGTGGTACATGTGTCTTTTGGAATTATGGTTTTGTTTG
GGTATATGTCCAGAAGTGGGATTGCTGGGTCATATGGTAGATTTATTCCTAGTATTTTGAGA
AATTTCTCTACTGTTCTCCATAGTGGCTCTATCAATCTACAATCTCATCAACAATGCAAGAGG
GGTCCCCTTTCTCCACACCCTCTATGACATTTATCATTTACAAATTTTGCAATGACGGCCAAT
CTGATTGGTATGAGGTCGTACCTCGTGGTAGTTTTGATTTGTATTTCTCTAATATTTAGTGAT
GTTGAGCATCTTTTCATGTGCCTCTTGAGCATATGTATATCTTCTTTGGATAAATATCTGTCTG
CTTAGGACTTCTGCCTCCAGCTGCTTTTTTTTTAAGGTTGTTTGTATTTTTTATATTGAGTTGT
ATGAGCTGCTTTAATGGGAACACATGTACACCCATGGTGGATTCATGTTGATGTATGGCAAA
ACCAATGCAGTATTGTAAAGTAAAATTAAAAAAAATTAGGTCCCATCTGCTTATTTTTGTTTT
TATTTTCATTTCTCTAGGAGGTGGATCAAAAAGGATCTTGCTGCAATTTGTGGCAAAGAGTA
GTCTGCCTATGATTTTCTCTAAGAGTTGTATAGTCTGGCCTTATCTTCAGGTCTTTAATCCATT
TTGACTTTATTTTTATGTATGTTTATTTTTATCACTTTATTTATTTATTTATTGTATCATTATTA
TTTATGTAGTTTATTTTTAGGAAGTGTTCTAATTTCATTATTTTTTCATGTAGCTATCCAGTTT
TCTCAGCATGACATATTGAAGAGGCTGTCTATTCTCCATTCTACCTTCTTGCCTCCTTTGTCA
AAGGCAACCACAGGTGTGTGGGTTTATATCTTGGCTTTATATCCAGTTCTGTTGACTTAAAAA
GTTATTTCTAACATCACATTTCTGCTGCTTAGCTAAAATGAGGAAGTTGGTCCAGATGATCTA
TAAGGGATGTTCAAATACTAGGTTTATTTGATCATCTATTGTGTGAATCTATGCTTCTGTTAC
CTTCTTTTTTAGTATATACTATTGCATTGAAGTTTCCCAAATATTTTTTTAAGTATTGACAAAT
GACCATAATTGCAACAGGAGAAATTATTCTAGATTTACAGAGTAGGTGACTGCAAGCTAGTA
AGCAACCGGCTATAATATCTTGGTCAAATTCTAAACAAAGAATTACAATTTCTGTGTTGATTT
TCTGATTGTAGAATTATATAGCAAATGTTTGTAAAGATTTCAATAAAATTTTCAGGCTAGAC
AGAAATGATACAAGTAGAATGGTTGCTGAAATTTCTACACAAATCTCCTACTAGAAAAATGA
ACTTAACAACTATTCATGCCCACAAATACCTTCACAAGAAGCTCAGGATTCCATGTGATAGT
ACCTGGGTCAAGGAAGTACTATATTTTGAAGAGATTTTAAAAGATACACAATGGATAGCCTC
AAACTTTTTAAAGCAGGAAATAAAACTTTATCCTGAGAAGATAGGTTTCCAGGGAAATACTT
AGGAAAATGAAGATGCCACAGCAAAAAAACCAAGAGAGCGAAGGCAAGAGTCACAAGATA
GGAAAAAAGTAAAGGACTGCTTAACACAGCTCAATTCAATTACAATCTTGTTTTTAAAATGT
ATGTAGATATCAAATGAATGTTTGTTAGTTTTTTAAAAGACCACATGGTATTTTAAAACTTAA
AGAAAATAGACAATTGCCTTATTTTCTCCACCCTGTCCCATTCCCCATGTTAAATTTTAGAAT
AATGAACTATTTTTAGATGACATACTTAAAGTGTTCTTGATGTTTTTTTTTTTTAAAGAAAAA
CACAAAAATCTGTTTTTTAAATACAATTTCTTCCTGTAGAAGAGAAGGATCTAACTTTTATGT
ATTAAATACAATACAGCTTGATCCATCACATCTATATACATATTTCTCATCTTCTACCTAATA
ATGTAGTTATTTTTCTAATATTATTGACTGTGTAAATCATTATCATAAATTTTCTAAGCAGCA
GAGCCAAGAAATAAACAATGAGTGCTTTCTTTCTGGTGGAATTTATGTTTTCCTGGTGATAAT
TGCATCCTTAAATCCTCTGTCCTCCTGGTCCAATATTAACTGGTTTTTGTTTTTTCCCCCTTGA
CTTGTGACATAATTCTTACTGCGAAACTTCCCTTCCGCTGTTCCTGGAAATTCCCTATGCCTC
TCATTTGTTGTATTAGCTATTTCCTTAATTCCCTGTCTTCTTTTACTTCCTTGTTTCAGTAAAAT
TTATATTCTGAGATTTCTTGGGCTCCTTAAAAAACAGTAAAAGAGAAGTAAAATTTAGGACT
GTGTGTATATTAAAATGTCTATATCCTCTTTCTAATTTTTGGACATTTTTGAAGAGATTGTTAT
TTGACTTGTGAATCCCCAGTGCTGCTGTCTGTTGTCTCTTTGATTTGTATGTGACATTACCCTC
AAGCCCCCGAGAGGTTTTTAAGAATTTCTCTTTATTCTTGGTACCTGTGATGGGAAAACAGG
CAGCTAGGTCATAGCCTGTGGCCCTTTATATCCACTCCGCTGTAGATGGGTGACTCTGCACTC
TGGGGGTTTAATGTCCTTTGCTTCTGGAAAATATTCATCATTCTATCCTATTTCCGCTTTGTCT
GGTTGTGTTTTCCCTGGAAACCCCACTGTGTGGTTGTTGGCTCTATCCTATAGCTAGCTGCTC
TAATGCTGCTCCCGTGGCTCCTCTGTACTCAGATATCCCAAACGCCTGCTCCACTGTGTTACT

TCTACTATCACGGTTTTCTTTTTTCAGATTGCTGCCTTCTATTTATGTGGATTTTAGGATTTCA
TGGTTTTCTTTTAAAAGTGATTCTTTCCTATTTATTTTTTAAAATTATGATTAGAATTTTTACA
TGTAATTTTTAGATTAATTTTTAACTCTTTGGCTGTGCTGGGTCTTCATTTCTGCGCAGGCTTT
TCTCCAGTTGTGGTGAGTGCAGACTACTCTCTCTTTGTGGCCGGTTCTTTTGTTGCAGGGGCG
CATAGGCTCTAGGTGTGTGGGGTTCAGTAGTTAGGGCTCCCAGGCTCTAGAGCACAGGCTTG
GTAGTTGTGATCCGCAGGCATAGTTGCTCCACGGCATGTGGTTATCTTCCTGGACCAGGGAT
TGAATCATGTCTTCTGCGTTGGCTGGTGGATTCTTTACCACTTAGCCACCAGGAAAGCCCCAT
GGTTTTTTGTTTTACTAGTTTCATGACACAAAATTGTCTCTTAGTTGTCTAATGATAGTAATC
ACAAAAATATTTTGTGTTTTTTATTCCCTTTCCTTGTATTCAACCTTAATGTTTACTGTATTTA
ACTTTTTTCATGCAAGTCAGAAGTATTCCTCAAATGTCTAGTGATTATCCATTTATATTTAAG
ATTGAGGCAAAACGAATACAGATTTCATTCTAGAGTTGCACTGTCCAATATGGTAGCCACTA
GCCCCAAGTGACTATTGAGAACTTGAAACATAGTTAATCCAGATTGTGTTCTAAGAGTAAAG
TAGAAACCAGACTTTTAACACTTTGTGCCCCTGCAAAAAAATCTCATCAATAATTTATGTTG
GTTTAAATGACAGTATTTATGATGTTTTCAGTTCAGTTCAGTCGCTCAGTCGTGTCCAACTCT
TTGCGACCCCATGAATCGCAGCACGCCAGGCCTCCCTGTCCATCACCAACTCCCAGAGTTCA
CTCAGACTCACATCCATCGAGTCAGTGATGCCATCCAGCCATCTCATCCTCTGTTGTCCCCTT
GTTCTTCTGCCCCCAATCCCTCCCAAAATCAAAGTCTTTCCCAATGAGTCAACTCTTCGCATG
AGGTGGCCAAAGTACTGGAGTTTCAGCTTTAGCATCATTCCTTCCAAAGAAATCCCAGGGTT
GATCTCCTTCAGAACGGACTGGTTGGATCTCCTTGCAGTCCAAGGGACTCTCAAGAGTCTTC
TCCAACACCACAGTTCAAAAGCATCAATTCTGTGCTCAGCCTTCTTTACAGTCCAACTCTCAC
ATCCATACATGACTACTGGAAAACCATAGCCTTGACTAGACGGACCTTAGTTGGCAAAGTAA
TGTCTCTGCTTTTCAATATGCTATCTAGGTTGCTCATAACTTTTCTTCCAAGGAATATGATGTT
GTAGTAAGTAATATATAATATTAAAAGTAATATTGCCTATTTACTTACATTTTCAGTGTGGCT
ACTGGATATTTTAAAGGGATATTTAGCATGAGGCTTCTGTCATATTTGCACCAGACAGTACT
ACTCTGATTGAGCAGAAGGTCCTGACCATTTCACTGTGCTCCCTGACCTCCTCCCCACTTCAG
CTAATAAGTAGTGTCAAGTGTTTTCTCTTGTGCCAGTTTCCTTAGAAAGGAATCCTATAATAT
TTTGCTTGATAGAGTTCTGGAATTGGTAGGGGAAGAGAAATCAGTGTGTGGGCTTAAACTTA
TTTAGACATGGATCTCCCTCATACCCCCAGCTGGGCTTCCTTGTCTTAGGTCCAGAAATCTCT
TACTCCATGTCTCAAAAGAGGAAACACCCGATCTTCTGACAGATGAGGAGGAAGGAGAGTG
AGCAGGCTGTGTAAAGTATATGGAGAGAAGATGTAGGTTTAGGACTTCCTGAGATATTCAAT
CAAGTTAAGACTCTGCTTTCCATGCACCCCATGGTTCTATGTGGACTTATACAGTAGATTCTT
TTTGCTTCTGCTCTAGGTGTACGTGTTTAGAATCTGACATGCAGGATGTTTGAACAATTACCA
TTAGTCCATTATGCTTTCTATATGTTGACTGGTTTCTTGTCTTCACTCATCTAAATCTTTGAGC
TGAACATGTGTGTTCCTATTTTTATAAATTCTACCTACAACTTTCATTTTAATGAGATTTCAG
ATGTAAGTGAAAATGTATTCCACCTGCCATGTTTAACCTGAGGTCATAATTTTAAATCTTAAT
ATCTTGTTATTCCCTTCTATATTTTTTGTCAGATTATATTCAGGTACTTAAGTCTCCTATAACC
TGCTCTAATATGTCCATCTCTCTGTAGATAGGAACCTTTATGGCAAGCCAGGTTTAGCTTGTG
CACATGCCTTTTTCCAACCATACCAATTACATTTCTAAATGATTTACATGATTTATCTAACTTT
ATTTTCCACGAATCCCACTTCCTTCTACTTTTTTACATTTAACTCATCTTCTAGGGAAGATTAA
GGATGTCAGAGTTCTATGAACATATTCTTAGAAATTCAGTCCCCACTGATCTCTTTCTTAGCT
AAACTTCTATCATGTGTGTGTTTCCCACAATTTTTAGTTTTTTATACTATGATAAATTATACTG
TGAGGTATTAATCCACATGGTTTTATTCTGCCCACTTCATTCTCTTTTCTAACTGCAAGATAT
GAAGCTGCAAATTGAGGGAAAATTTTTAGTGGATACAAGGAACTGACATTTGGTTACTATTC
AGTCTTACTAAAGACTAGGAAAATAGTTGTGCCCTAAGCATGGATAACGGAGAAGGCAATG
GCACCCCACTCCAGTACTCTTGCCTGGAAAATCCCATGGACGGAGGAGCCTGATAGGCTGCA
GTCCATGGGGTCACTGAGGTTCAGACATGACTGAGAGACTTCACTTTCACTTTTCACTTTCAT
GCACTGGAGAAGGAAATGGCAACCCACTCCAGTGTTCTTGCCTGGAGAATCCCAGGGACGG

CAGAGCCTGGTGGGCTGCTGTCTCTGGGGTGGCACAGAGTCGGACACGACTGAAGTGACTT
AGCAGCAGCAAGCATGGATAAAAGGGAAACACAGACTTTAGACATGACCGAATCCCAGAG
AGCTGCTGTGATCCGTTCTGCAGCAGACGCACTCCTAAAGGCAAGTTTTCTTCTCCCTTTCAA
CTCTCAAGATGGAGGACCTCAGAACACAAGTGTTATCAGACAGAACAAGCCTCAGAAAACA
CTGACTCAGAGACTACGGCAGTGGAGTATCCGGGGCTGTCCGTGGTACATAGGCTGCTGTTT
AGAGGCAGATTCATGGAGCCCCTTGGACCCTGACACTGAGCTCAAGGCCCCTGAAGGTTATG
CAAGTTAGTTGAGAAAAAGAAAGGTACCACCTGCAAAGTGCTCGAGGCCAGAACAAGGGA
AAGTGATAGCCACAAATTTAGAGGGCATGCTCTGTGTTCTCATCTCCTTCTCTGAAAATAGC
CTGTACTGAGGAAAATGGAACAAGAGTTTTAAAGTTGAAATGACTGAGGGACAGCAAAAAT
GAAATTTCTTAAAAAGTGACTAAATATGCTGTATCAGACAGAATGGAGGTTTGAGATAAAA
CGAAATTTAGTGCAACAAAATTTTGTTTTTGCACACCTGAGTCTGTGATTGTAAAACTCATTT
CCATTTCAGTAATATACAGCATACAGCCATTCCCAAAGTTTCTTTCTCACAAAAAACAAATG
AAAAAGAAAACCTTTTCCAGGTAACCATGATTATTTTTCTTTGTTTTAGATTAATTTTATATTT
CACATTCCCTTTGTCAGGTTAAACAGTTTTGTTTCCCAGAGTGATAGAAACTTGGACTTAATT
TTTATTTTGAACTTTGATTTTGAAAGCTTAACATGTAATATTTTCTCCCTTTAAAGAATTATAC
CCACCTAGGGGACAGGAGATGGTCTGGATTTTGAGTTGATTGTATATACATACACATGTATA
TGCAATTTAAGCAAATAATTGTATTATCAAGATGGTATGACAGCCCAATACTTAAAAATGGC
TGCTTTAGAGAAGAGCTAGAATTTGGGAAGCTGAAGGTTATTTCCTAATATTTATGATTTGA
CAAGTAAAGTTCCCCCATATTCTGATCTACTTTTCTTGGAGCATTCTCTTACTAGCAGACAAA
AGTGTCCTAAGTTACACAACCATAGCTTGGCATTCTTTGACCTTGAGCAGTTTTCTTGACCTC
TCAGTCCTTTTGCCTCTCAGTCCTTTGGTACTTTTCAGTTCAGTGCAGTCACTCAGTCGTGTCC
GACTCTTTGAGACCCCATGGACTGCATGGTACTTGTAATGTCAGGAAACCAACAATTCTTAT
TTCAAATTATGGTTATGAAGATTAATTTAGATAATACATATAGTCCTTAGAAAGGTGTTTGCC
TGTAGTAAGTGCCCTCGATGTTAGCCGTTTGTATTACATGACACACTTTGGGCTTTTCAAAAA
AATGTGCTTCTGATAAGTTTTATTGCCGGGGTCCAGTCCCGGTGGATCCAGGGTAATTCAAA
GGTGGGGACGGAATCGGCGTCCTGGAAAAAACTTATTTAATTACAGATATAGAGAGAGATT
GGAAACAGATAGTGTAGTAGGAGAATTAGTGGAGAAAAGAGGCTGAATAACTGGTTTACAT
GGAATACCAATCACCACCTACGTAGGCCACAGGCGTCCTTCCATTCTCCCGAAGGACAGGAG
GCACTGAGGCCTCCCCGGTCCGATCTCAGAAGCTCAGGCAGAATTAGCAGGCTTGGTGAGTA
CCCACATTTCAGATGGGAATTCAGCCAGGAAAGCAGGGAGCAAGAAAGAAACAACATGGG
GGAATCAGTCTTTCCAGAATTTGATCCAATTTCTTTATTTTTCAGGTTTGTTTACATACCTTTT
TGTTATACATAGGGATGAATACAGAGTCACGTGGGGGTCAGCAGACCTGACCCTTGTCACAA
TCAGGTGCTTCATATAAAATTATACAAAGGTCTTATGAGTTTCATCATCTTCTGGCCATGAGG
TCTGCTGACATTTTATGGCCCTTTCTGATACCAGTCAGTTAACCAGAAAACTTATTTTTCCAG
GGGTGATTTTTTCTTAAATCAGGCGCCACCCTCCAAATAAAGTTGCATTCCTATAGGGTGAG
GGTGTAGTGAGTTACAATCAAGAAAGGAATTTACTTAACCTAAGGTTTAACATGATTCATCT
TAAAGGTTAATACTTATTTCTCCTATATGCTAGTTATATTCATTATAAGGGCAGGAATATGGA
GATTTAGCAGCAAATATTGGCTCAACAAATGTAAACCCTTCACTAATGCTCCCCTTAAGATC
TATTTTGTCTTAAGATAAAGTTACATTTTTGCAAAGCAAGGACACAGTGATTTATAACAAAG
TACAGTGGTCTATTACAAAAGAGAAAATTCATTAACTCAAAAAGTCTAGTATTGCTAACATC
AAAAAACTACTATATTTCCTTTTCTATATTCCAAATACATTGATTAATATATTCCCAGGTGCC
TAAGGATATGGAAGCCTGATGGCAATCATTGACTCATCAATGAAAAAAGCCCTATGCTAATA
CTCCAAACTCTCTGTGCTGTTTATGGTTGAGAGGTTGTCACACAAGCTAGTCTGTCAGCAGA
GAGGTTTGACCTGAGACATCCTTGTCACACTCAGGGCAGGGAATTAGCAGTAATTATTGGCA
CGACAAATGAAGAAAAAACCCTTCACCGATATAATTCCTAATCAACCCACTAATACTATACT
AATGATCTTCTAATTTCTCAAAAGAGTCTGTATTTAGAAAGTTTTAAAACATCCCGTGCCTCT
CACAGTTGGGAGGCTGTAAACAATCACATGTGGCTGGACGAGCCTGATCAGGCAGGCCAGA

GAACCTTCAGAGTTCCTAAGTTGAAACACTCTTGTCACGCCCAGGAATTTTTATTAACTTGGA
GCTGCAAGTTAACTCCTTCTCCAAGAGAAATGTTTATGGGGGAGAGCTCCCCGTAAAGTACT
CTGGTTTTGGGGGTAGATGCTGGGGAACAGGGTGTATCCTGAGGCTTGATCATACCTTTGTG
TATGCCAAGCTTCCTTCCTCTTGATCTTTGGCATGGGCAGAGTTCCTCATGCTGGCTCCCAAC
ATTTTATCATTTCACATATTAAACACCAAGTGGTTTTCCAAGGAATAAAACATGGTGATGTG
ATTATTTTAAAAGTTAGTCATCACACAACAAGAATTTGTCCACTGCCCTCCATAAGATTCTAG
AGATCAGTTAAGTTCAGTTCAGTCGCTCAGTCGTGTCCGACTCTTTGCGACCCCATGAATCGC
AGCACGCCAAGTTCAATGACTTTTAAAAGCACTCACCGTATGCATACGTATATCCCCTCTTTT
TTTTTTTTGATATCCTTCCCATTTAGGTCACCACAGAAAACTTGAGTATAGAGTTCCCTGTGC
TGTACAGTAGGGCCTCATTACTTATCTATTTTATACATGATATTTGGAGCTCCTTTCTATCTGC
TAGATTGGATGCTGCCAGATTCAAAATGATTTTTGCTCAAATAAGCTCAATATTTAAAAAAT
ATAAAAGTACTCATCAAAAATAGCTTTGGTCACAGATAGTCTAAAGGAGGACAGGACATTA
GACACCATTTAGGTGGTATTTTCAATTAGACTGAAATAATTATTTGGCCTGTAATCTCATTTT
ACAAGGTTTCCCATATGCCTGAATGTTTATTCGTGGTTTTAAACATGGAAGCTCTGTGAATTT
CAGGGCAAGAAGAGTCCAATCCCTGTCTCCAAGGACTTTGGGAGTTACCTATTGGATAGAGG
AGAGCAAGACTCTGCTTTTTAATTTTATTTTTTCGACACGAAAAAACATTTTGTAATGGGGTA
TAGCCAACGAACAATGTTGTGAAATTTCAGGTGAGCAGTGAAGGGACTCTGCCATATATATA
CATGTATCCACTCTCCCTGGAGAAGGAAATGGCAACCCACTCCAGTGTTTTTACCTGGAGAA
TCCCATGGATAGAGGAGCCTGACAGGCTACAGTCCATGGGGTCACAAAGAGTCAAACACGA
CTGAATGACTGAGCACATCCATTCTTCGCCAAACCCCACTCCCATCCAGGCTAGCACATAAC
ATTGAACACAAGCTTCATGCGCTATACAATAGGTTTTTGTTGGTTATCCATTTTAAACACAGC
AGTGTGTACATGACCTTCCCAAAGTCCTTAGCTATCCCTTCCCCCCAGCAACTGCAAATTCAT
TTTCTAAGTCTGTGAAGAATCTGCTGCTTATTACCTAAATTCAGTTGAGCAACAGAGAAGAG
GGAAGGAGTCCAGGACACACTGCCAGACAGAGGCCAGAGGGAGGGTTACACAGAGAAAGA
ACGTGCTGCCCTTATACTGCCTCTGGGTCTGGTAAGGTTAGTACAGCTTAGAATTGAATATG
ATAAGAAAGCATTATAACTGGTTGGCACCAGTAGTGTAGATTTTAAAATGAAAGACCTGAG
ATTGTGAAGAAGAAAAACTTTTTTCACAGAGATAGCGTCTTTATTACCATTATCAATGATAC
CAGTTATTACAACTAGTTGTTTCAATGAGTGCATATATTTAATCTTATTTGAACACCATGGCA
TTACAAGATAAATATTGTTATCATCCTCATCTTTTAGAGGAGGAAACTGAAAAACAGAGAAG
TCAAGCAACAGGTCCAAAACTATTCGGGATACTCACTATCAACCCAACCACTTGTCTCCACA
ATCTGCAATCTTACCCACTCACTCTCTGTCCCAAAGTTCCTCTGCAGAACCCAGACTATCAAT
GTGTGTCCCAGAGCACATGAAAAAAGAAAAGACCCAACCAACCCTCAAGGATCCTGTCCTG
TCTGGCCAGAGCCAATTGATCAACAGACATCAAGCCAAGCAAGCTTTTACAAGAGAAACCG
CCTTTGTGGACAATTGCTAAATTTGGGCTTCACAAGAAGTCAATAGTTATCAAGTTATTTGCT
TGCCAGACCCAGAGCAGTCTGAATTCCCTTTGTTCCTGCAGCCCAAAGGACTACCCCTTTCCC
CATCTCCAGTCTCTCTCTTTCTTTCCTATACTCTATCATTCATTTTTCCTGTTTACAAAGATAA
AAAATATAGGTCACAAAAGGTTATGTGTGTGTGCTAAGTGGCTTCACTAGTGTCTGACTCTG
TGCAGCCCCATGCACTGTAGCCTGCCAGGCTCCTCTGTCCGTGGGATTCTCCGGGCAAGAGT
ACTGGAGTGGGTTGCCATTTTCTACTCCAGGGAATCTTTCCAACCCAGGAATTGAACCCCTG
TCTCTTATGTCTCCTGCATTGACAGGCAGGTTCTTTACCACGAGTGCCACTTGGGAAGCCCTA
CCAAAGATTGTAAGATAATTTAAGTTGTAGGCTTAACCTTTCAAGTCAGATATACCACCTAA
ATACTCCCTTTTGCCTTTTCTTAGAAATTTTTACTTGTCCTTCTAATTTAATTCAGCACTTCCC
CAACTGTCTTTATGAAACACTGTTTCAGTAGATGTTAATAAAGATATCCTAGGGTGACACAT
GGCCAAATAAGTTTGGGAAATAAGGCATACCAGGACCTTTTGTTGGAGCTGTGTTATGAGAA
TAGTAAGACTGAGAAATCATGAAGTAAGGAAACTGTTGAATATGACTTTTAATTTAAATATT
CTGGGTCCTCTTATGTACTAGGCTCAGTTTTGGTTTTGTTTTTTTTTTTTTTGTATTGGAGTAT
GAAAAATGTTTTAAAGTGAGAGGAACAAAAGAGATGAGAGGAAAATCCATATTTCTAAAAA

GTCAACTTCTATGACATAGTGCTATAATATAGACTATTCATATTTTAAGAATGCTTTATGGTA
AAAATAATTGAACCAAGAAAGTTTTATTTCAAAATATTTTTCCTCTGTGCTTTCAAATGGAAA
TAAATTCTCCCCATGTTTATTACTTTTTAAAAATACAACTGCATTATTGTTTCTGTCTTATTAT
GAGTTAGCATTTATACAACCTCAGGGTTCTTATTTCCTTAGCCTTGAATATAAGTGTTTGCTT
TCTCTAACCCCTAACATTCCCGGTAAGATGATATGCTTTACCTGTAGTAAGAGACATTTGTTG
TTGTTCAGTTGCTAAGTCGTGTCTGACTCTGCAACCCCATGAACTGCAGCATGCCAGGCCTTC
CTGTCCTTCACTATCTACCTGAGTTTGCCCAAACCCGTCTATTGAATCAGTGATGCCATCCAA
CCATCTCATCCTCTGTCATCCCCTTCTCCTCCTGCCCTCAATCTTTCCCAGAATCAGGGGTTTT
TCCAGTGAATTGGCTCTTTGTATCAGGTGGCCAAAGTACTGGAGCTTCAGCTTTAGCATCAG
TCCTTCCAATTAATATTCAGGGTTGATACCCTTTACGATTGACTAGGTTGATCTCTTTGCTGT
CCAAGGGACTCTCAAGAGTCTTCTCTAACACCACAGTTTGAAAGCATCAGTTCACTGGCACT
CAGCCTTCTTTATGGTCCAACTCTCACATCCATACCTGACTACTGAAAAAACCATAACTTTGA
CTACAGGGACCTATGTTGGTAAAGCAATATTCCTGCATTTTAATACACTGTCTAAGTTTGTCA
CAGGTTTTTAATCCAAGGAGCAAGCATCTTTTAATTTCATGGCTGCAGTCACCGTCTGGAAT
GATTTTGGAGTCCAATAAAATAAAATCTATCACTGTTTCCACTTTTTCCTCATCAATTTGATG
TGATGTGACCAAATGCCATGATCTTAGTTTATAAAAAGTTGAGTTTTAAGCCAGCTTTTTCAC
TCACCTTTTCCACATTCATCAAGAGGCTCTTTAGTTCCTCTTCACTTTCTGCCATTAGAGTGGT
ATCATCTGCATATCTGAGGCTGTTGATATTCTCCTGGCAGTCTTGATTCAAGCTTGTGATTTA
TCCAGCCCAGCATTTCTCATGATGTACTGTGCATATATGTTAAATAAGCAGGGTGACAATAT
ACAGCCTTGACGTACTCCTTTCCCAATTTTGAACCAGTTCATTGTTCCATGTCCAGTACTAAC
TGTTGTTTCTTGTCCTGCATACAGGTTTCTCAGGAGGCAGGTAGGGTGGTCAGGTATTCCCAT
CCTTTTAAGAATTTTCCACACTTTGTTGTCATCCACACAGTCAAAGACTTTAGTCATAATGAA
GCTGATGTGTTTTTTTTTTTTTAATTCCCTTACTTTTTCTGTGATTCATCAGATGTTTGCAATTT
GATCTCTGGTTCCTCTGCCTCTTCTAAACCCAGCTTGTACATCTGGAAGTTCTTTTTTCATACT
GCTGAAGCCGAGCTTGAAGGATTTTGAGCATAATCTTGCTAGCATGTGAAAAGATGCAATTA
TACAGTAATTTGAACATTCTTTGGCATTGCCTTTCTTTGGAACTGGAATAAAAACTGAACTTT
TCCAGTCCTGTGGTCACTGCTGAGTTCTGCAGATTTGCTGGCATATTGAGTGCAGCACTTTAA
TAGCATCATCCTTTAGGATTTGAAATACTTCAGCTGGAATGCTATCACCTTCTCTAGCTTTGT
TTGTAGTGATTCTTCTTAAGGCCCACTCAACTTCACACTCTAGGATGTATGGCTCTAGGTGAG
TGACCACACCATTGTAATTACCTGGGTCATTAGAAACGTTTTCGTACAGTTCTTCTGTATATT
CTTTCCACCTCTTCTTAATCTCTTCTGCTTCTGTTAGGTCCTTGCTGTTTCTATCCTTTGTGCCC
ATCTGTTCATGAAATGTTCCCTTGGATCTGGTAAGAGACACAGAAATATACTAATATATTTTT
AAAATTTATATTCCTTATGCAACAGGAAATCTGTATCTTAAACACTTGCCCCTTGATACTTAG
TCTAATATTCATTTTGAAAATCACTGAAATCCTTCCAAAGATATATATATATACACATATATA
TCTTATAAACTTAAATAAATTTAAATGAGCTTTCTAAAATCTATACAGGCACCAAATAGTAA
CATAACAAAACACTTGTAAAAGGCATCCAGTATGGTTTCTTTTCTCAATATTAAGTAAAAA
TTTTTGTGCTTTGAAGCAGAATTTTTAGTTCTCGTGCAATAGAAGTTGTAACTTTTGGCAGTT
TAAATAAAAATTTATTTTAAATTCTAAGGTGAAACTACATATTGTTCCCTAACAGTGCCTAG
GAAAAGTAGACACTCAATTAATTTGAAATTAATTTCTTTAATCTCATATGTTTTTTGCCACAG
CAGCTTTTCATATTTTCTTCATTGGAGCCACTATCTTCTGGCAGAAACTGCCATATCTTATGA
AAGATGGAGAAATATTCTCTGCCTTTATGTTAGACAACGTGTTCTTTGTGTTAGTCAGTACTT
GGTAACTGGCCTCTGGACTCATGCTTGAAGAAGAAGGTTCAAGGTTAGGTTCTCCTTGAAGC
TTCCCCTTCTAAAAGCTACCCTGTCTGTAGGTTTCCCACGGTACCATAAACCTTCCATTAGAC
CATCTATTGTATGATTTCATAATAATTTTTCTATGTGTCTGCCATTCCATGAAGCTAGAGTTTT
ATTTTCATAATTTCTGGACCCAGGAATATCTGGTGGCAAGGAGGAATAGTTTCTTCAGTAAT
TCTGTGAACCAATGAGTTTTTAACTATAATTTGTATATGAAAATAAATAATTCTGAAACATC
AGAGTAGCATAGCACAATGCATCTGCTGAGAGATTTATACTGACACAAGGTAAGGATTTAG

ATAAGGGCAAAAACTGCCTTTCATGATCATGCATCCTTATGTCTTCTGACTAATCTCTGATAT
TACTTGTCACCTGGATGCATAAACCTAAGGTGATACATTTCATGCAAATGCTGGAAATCTTA
GCATTTATCTAATTTTTCTAATTTTCTCTGTCAGTTGTCTCCTCTATAGGAATGTAGGCTAACC
ATACCTAATTACAGAATTTTCCATAGATTTAAATAATAACATGGAAGTATTTAGCCTGAAAA
CTGATCCATAATAATATGTCAACAAATACTTGTCCTCCCCCTTATCCTTCCCTAGAACTTTTC
AAGTTGTCTTTATAAGGAGGAATAAATAGTATTGTTTTTATATTTAATAATTTTATTTAGATA
ATTTTAAAAGCATATTTAAAATAAAATGTAAAAAGGCAAGAGCACTGGAACCAGAATAATT
TAGAGAAAACTAACCTCATTCTAGTAACAGGGTACAGTATATGGGAATCAAAGACGTATAA
ACTTTTCCAATTACCCAGGAAAGTAATATTAAATTGACTAGCACTTCTGAAAGTATAATGAC
ATACAAACAATATTAAAGTAATACACTAGCAAATATCAGGCCAGATCCTGGGAGAAGATAT
GTGAAAAGAGGTGAAATATACAAAATAAAAATAAACAAAAAGAAACATCAGGAGAAAAAG
AGAGCTATATTTTAGCTGAGAGGCTTTGTTTTTTTTAACTACTCTTAGGGCAATGTAAGATGA
GCATTTCCTCAGATCCCTGAAACTTTATCTGTCAAATGATCAAGATTGATGATTCTTAAATTT
TTTTCACATTTTGTAATATAAAGTCACGAGCAAGAGAGAAAAAATGTGTCATACATGGATGT
GGGTGAAAGGTCTTTGAACTAAGAGTCACAGCTATTTATAACTGAGTAAATGTGGATGTCTC
GTGAGCCCAGAGCCTTGTGGTTGTCTACTCTAGAATCTTTGTGTGTAACTGTCCCCTGTGTCT
AAGCTACATCTCTAGAAGATGAGCTCCTTAAGGTAAGTGCTGCTAAACTATCCAAGCCAATT
TGGGCACACACTGTTACTGGGTTATCTTGGTAAATGTTGGTAGTTTTCAATAAATAACAGTG
GGGAGAGCTACAGATCTTTCTCAAAACAGGTACAAAGAAAGCCCTCGCAGTACTGGCTTCTA
ACAGAAGCAGGGAAGTAGCAGTTAAGTAGCAAGGTTCCCCAACCTGTTCCATGGGATTTGT
GAAGTACCCACTCTCTGTCCTTTTAAGTGTCCCAGTAGTTCTTGCCTGGAAGTTTCATCAGGG
GCACACACAACATGCTAACTATAGAATTCAGCTAACTATCCAATCCCAGTCAGGCCACCAGG
AATGCCCTGAAATCAAGAGCTAGACAATGAACTTGGTGGGTTTTTATAGCCTGTGATATAAG
GATAGTTAGCCAGTAATTTTTCTGGTCAGTTCATAAATCTTTGAAGCCATTAATTTCACCAGT
GAAAACCTCTCTGTTATATTTATTGTGAAAATTTCCAGAGTTTAGGAGAAAAGACAACTAAA
ATACATATTTTGATTATATGCTAAGTAAACAGTTCTAGAATGAATATTTTCAACTAGGTTAGC
TGTCCACTTAGATTAAAAGTATTCAATACTTCATCATTGCATTTTATTTGCAAAGTTTTAAAG
TACATTACAAAAAATCTCACTGGAAGGATCAGGCTTTCTTCTTCCTGATGACAGGATTCTAA
GAACACAGTGGACACATAGCAGATTTTCATTAAGTATGCGTGTGGGTGTGTGTATGAGGATA
CATGTCATCAACTTTAATCCTTAGAGAATTAATGCCTTAAGTACTATCAAGAAAATCATATTT
TTTTAATCTAATTTAGCATATACTGTAATGGCTTGATTTGCTTTTAGAATATATTTATCTTAGT
TTCTTCATTCTTATGCGTTGCATATATAATAGCATAGTCACCAAACATTGCTAATCTTACATG
GCAAAAGAATGTCATAACTAGAATAAACTTGAGCTTGTCCATTTTGCAAAGCCCTGCCCAAG
GCTGCTATCATCTCCAGCTTTCCACAAACTAGAATATTCACTGATGCCTTGTTTCTTATCCTG
AAGGCCAGTGTGCAATGTTGTTATGTGGTTCCTGTGAAGACTATAAAGTCCTACACAGAGAC
AAAGGATGAAAACAGTCAAGGGAATTGCTATAAGAAAATAAGTAGGGATATCTCTGGAGAG
GGAGTTGCTTCTGTGTTCCTTTAAGCATCACAGAGTAATTGTTTCTTTTTTAAGGAAAGAACC
AGGAGAAGAGAAGCTCATTTTCAAGCTAGAGAGCTTGGCAAACACAAGCTGCAGGGTTTAC
TTTGGTTTAGGAATCCCACTGCCTCACTGGAGTGTCAATCAAAATTCCACAGCCATTCTTGGA
TACTTGGAGGTGGGGCTGAGTGATGTACAGCAGTGATTTGACCCAGCTCTTGAGGCAGACAC
AACAAGAGTCATCAGTGTTAATTTCGTTGTCCTCCAACCAGCGTTCTGAAAGTGCGTTGACA
CTGGTGGCCTTGCACTTTTTCATCGGACTTCCAACTGGTAATTACACCTATATTATCACTGTG
AAGTTATCTAACCTAACAAAGTAGGACTCAAAACCAGTGCCTGTAATACTAAAGTCTCTTAC
TTTGATCTGATTTTTGCATTGTTCTTGTCCAGTTTAAGTTGTATGTGCATTTTTCCTCTTGTCCT
AATTCATAGAGTATTCGAGATAGTATAGATATTAAGGAAGCCTGTCCCAAGCTTTTAGATAA
GAAATTGTGTCTAAAAGAAAACAGAAGGAGTTGGTCAGGTTTTGTAATTTTGCTATGATGTT
ATCAGTAATAACTAAATATTAGATAAGAGGACATTCAAAAATATGTGAAATTGTCTAAACCT

TCCAACACATCTATGCTTGTATACATGCTGCAATATTGCTAGTAGAGCAATATATTAAAAGA
TCAAAAGCTATCTTTTATTCTAAAGAAAATACATGCCCAGGAAGACTTAAATTATAGATGGC
CACTTTTACCTGAAGAGGCAATTATAATGAATATTATTGTATAGGTTTGGGACTCTCATATTA
AGATTGTAAGGCAGAGCAGTTGTTCTTTTCAGTCTTGGAGAAAGAAGAATCTATCCTCCCCC
ATGGACAGGGAAGCCTGGTGTGCTGCAGTCCATGGGGTAGCAAAGAGTCGGACATGACTTG
GACACTGAACACCACCAACCAGCAGAGGTCACAGGCACTTTGTACTGGGACATTCCTGGAA
TGGATCTAGTCCATTAAGGGTTACTGTGACTTGCTAGACAGGTGACTTTGGAATTTTATTTTA
TACTCAAGTTAGGGAAACTCAGTCATCATACTGTATACCTGACTTGGGCTACTGAATCTTTA
ATGCAATAGCTAACAGGATATAAGCAGTAACATTATAAAATTTGCCATTAAATTAAAGCCAA
TTTTCTCTTTGCAAATCATTGTCACGAGGTTTCTCATGGCAAGAACAAACTACATAAAATGA
CAACGGGAGGCAGAGAAAACCAGGTATAAGAATTAGGTAAAATTTTCTAGATTCCAGACTT
AATCATTTTATTGGTCACATGTAATCATTTCTATTAATGACATTTTATTTCAGCCTATATTATG
ATACAGTGTTTTGTAAGGACAGACAGCATTGGCCTAATTTTATTTCATTGTGATTATCTTAAA
TAGCTGGAAACTGTAAATTAAAATTGGATGCAACTGTTTGCCTCAGTGAGTACTGTGTCAAG
ACAGCAGACTCAAGGAAGTGTTTAACATAGAAAAGGACAGGAAATGTTAAAAGATTATTCA
AGGGACAATTCACAACTTGGCTTGCATCTTAGATGGATACAATTCTAATTGACATTTTGGTA
GTTAATATTGTTACATAAGTATTCCAGACAATCCATAATTAAATATTTTGAATTAAACTCACA
AATAACTGCAGACTTACAATTATGTATGTTACCTAAATGTAAATGATTACAAAATTAAAAAA
ACTGAGATGCATGCTTTATAGTAATGCTACTAAACAGATATATTTAATATATATATAAAGTT
AAGGACTTTATGAATGCCAGCATTATGTAAATCACTTTGGAGGGTTGGATTATGTGTTGAAT
CTTGTTTATTCACTCAACACACATTTGTTTGCCCACAACAAACTAGGTATTGATCATTTCCCG
TTCCTAATTTCATCTACTTAACACTTTGGGTAATCAATTAATTCTCTAGTATGAACATCATTA
TTTAATAAATTAAATCTATCAATACAGGGGACGTTGTATAAAGGTCACTGCTCTACTGGATA
ATTGGGGAAACTTCCACTGAGATTGGTACCACAGTTTCTCAGATTTTTCCCTTAAGGATGATG
AAAGATCAGTGGACTCCTAAGTATCTCAAGTCTGTGGAGTCTTAGGTTGCTTAGAAACCCGG
TGACTATGGGCTTGCTATAGCAACCCAACTTATCAGTGAATGCAAAACTAACTTACCTGGCA
TTTTGCCAAATCTCCATATTTTTCTAGTATGAATATTCCGATCACATGACTGTTTATTATTCAC
ACAATCAAGCTAAATACTACTACAGTTTACCAAATTTTGAAAAGAATAACTTACAATAGCTA
GCAAATATAAGAAAATTAAGCTTAAGCTCACTATGTAATTTTAGTTACTTGAAATTTGTTATG
TCACCTAGTTATTATCCCATTGCCTACATCTGATTATATATATGGAAGATTTGCACTAAATTA
GAAGCATGGGAAAGTTAGGCTAGTACAGTGTATAATCTATGTGATAATAAAAAGGCTCTATC
AATGACTTCTTTAATATTTATAAATCCGTAAATTTATCCATACATTTATATGAGGTGCATTTT
AATGATATTCTTATTTTACATTATAAACCCCCTTTTCACATATAAATATATTTCTTTAAAATTA
TTTACCACATTCTCTTTAAAATCTTAAGCCTTCAGTAATATGCTGTGTAATCTGTATACACAC
ATGTATTAGAAAATTAATTTATTTGAAAAGCACATTTTTCCAATAATGGAAAAGATGGGAAT
GTAATCTCATCGTCAGTCTAAGTATTTGCTCTTTGTAGTATATTTTTAACTATTTGCCCCCTTT
GATTTAAGGCATGTGTTGCGATTTTAATAGTATCTTCAGTTTATCAGTTTTTACATTGTTGTTC
AACAATAGCGATCTCTGTACCTCTTCATATGTAGGAACTGTATTTTTCTTATCTCCTGTTGATT
TCGACTCTCATGTTTATCACAGCCAATAGCACAGGTAGACCCAGCAGCCTCTCCTGAGACTA
GAGTCCTATAAGGTTTTAGTTACTGTCCTTTTCTCCTGGGTTTTGGCATTTATGCATTTTGTTG
ATATTTAATAAGCGTTTGTCATATTAAAAATTCTCTTGAGAATAAAAGAGTCAGTGTGACTT
AGGTTTTTATGGGATAAGGATGCATTTGCTGCTATTTTCAATTTTTCTTTACACTTTATTTCTA
CAGAATTGTGCAATTGATAAAATTTTTGACCCCTCTAGTATATCAGTTTAACTGGGTAGTGCC
GATGTTAATGATGATACATCTCATCTCACAGATGGAAACAAGTAGGAAGACAAAAATTGGG
CTATTTTAGTTAAATTGTCATCAGAACAGAAAAGCCAGTTCTCAGAGGTCCTTGTTTAAGGG
TCCAATGATTGGGCTACCCTGGCACCCTCAACAGAGGTGCATTTGTGCAATCGGATATTTTTT
AACAAAATAAGTGAACAGTCCAGTACAAATGGACTAAATAACCCAAAGGATCAAACTATT

AGCAAGGGATCACACGTTAGCTTATAAATAGATAGTAGATTGGCATGTAAAATTAGATGGT
AGAGTATAATACTCAGGAGACTTAGAATTTTTGATTGGCAAAAAAACCAATACAAGGAATG
ATCTATATTTAATCATTATTTTTGGCCTATGATTGAATTATTTTCATAAATTTTTGAAATCTTC
ACTTTAATCAATATGTCTGTTGCTGGCAGTGAAGATATAAACATAATTGGCACATTTGACCA
TTTAAAATATTTGAGAAAAGCTTATCGTGTAGTGGTATGTTGTTTGTCCTTGTACTGCAGTTA
CTCAATACATATCTGTGGGACAATAGAAGTAAGCCCTCCTTGAGAATAAATATTTTTCAGTG
TGGAAGAACCTGACTGTTTAATAATTCAGATAACCAGTCCTTTTAAATTCTGTGTTGGATATC
TTGGAAAAGTAAATATATTCTGCCAAAGTATTAACATACCCATGTCTCTTTAATGTATGTTGT
GATACTTGTAGTATAGGTTCTTCAGTATTTATTTGATGAGTATCTGAATACCTATTATGTGTA
AGGAACTATTTTAAAATCTGGCATATAAAAGTAAATGTTCCCAACACAGTTCTCAGTGAATA
TATTATAGAGAGTTCCACTGTGACCTCTCCCTTGGAAGTCATAAATGAGTATATAATGAACT
GAGCTTTTTTGTTATACTTTGTATGAGTAGGACACTAAAGCATTCTGGAGATATAAAGATGA
ATAAATATAGTCACGTGGCTTTTTGTATGTGCTTAATATTAAGATCTCTGCTTGTTGCACATG
GTATACAAAACCATGTTGTAGGGACTGTTTATTTCCTTTTTTTTAGGGACCTATTGATTAAAC
CACATTTCTTGATGATAGTTAACGAGGAGAAATTTGGTTAGATGTTTAGTTATTTAAAATGTT
TGTCATCTACAATTCACAATTTACATAGAATCTCAGGATATGAGTTTGAGTGTTTTCAACTCA
AACCATTGTATCAATTCAGGGATTTAGGGTACACCTTAACTAAAAAAACTTCAGAGTTAGCAC
TCTGTAGGCATAATTAGATCAAACTGTTGGTTCTTTTGAACTTATTTGCTCTTGCCTGTTATTT
GGGTGTTGGCTTTCACACTAGTTTCCTTGAAGTTCTAAGATGGTTCCTGGCAGCATTCGTACA
TGTTTCCTTATTACAAATAAATAGGAAGATGGGGAGAGATAATAGTGAAAAGATCATTCCCA
CCCATACTGAACAAAAGAGCAAAGCTTTTGTGAGTCCTGAGATGTATGTTTTTATAACACTG
AAGTGCTTATAACGCTTTCAGTTCAGTTCAGTTCAGTCGCTCAGTCGTGTCCGACTCTGCGAC
CCCATGAATCACAGCACGCCAGGCCTCCCTGTCCATCACCATCTCCCGGAGTTCACTCAGAC
TCGCGTCCATCGAGTCCGTGATGCCATCCAGCCATCTCATCCGCTGTTGTCCCCTTCTCCTCC
TGTCCCCAATCCCTCCCAGCATCAGAGTATAATGCTTTAACTTATAACAATTAAATAGGGAT
TTAATAAAAAAAAATATTAAATAGAACATTGACATTTTTAGAGGCAATATCATGTTCAGTTC
AGTCACTCAGTCATGTCCAACTCTTTGTGACCCCATGGAAATATCATGTTAGTACCATGACA
ATACATGTTTTATAAAACAAAATATGGAGGAAACTTTTGAATCTTTTCTTCTGTATTCAGTGT
TGTGAGTCATCTCCGAAAACAAAGTCAGTTGCTCATGGTTTCTAGAACATCAGTTACTTCTA
ACTACATAGGATATACTAATGATATGCAGAGTACATCATGAGAAACGCTGGACTGGAAGAA
ACACAAGCTGGAATCAAGATTGCCGGCAGAAATATCAATAACCTCAGATAGACACCACCCT
TATGGCAGAAAGTGAAGAGGAACTAAAAAGCCTCTTGATGAAAGTAAAAGAAGAGAGTGA
AAAAGTTGGCTTAAAGCTCAACATTCAGAAAACGAAGATCATGTCATCTAGTCCCATCACTC
CATGGGAAATAGATGGAGAAACAGTGGAAACAGTGTCAGACTTTATTTTTTGGGGCTCCAA
AATCACTGCAGATGGTGACTGCAGCCATGAAATTAAAAGACGCTTACTCCTTGGAAGAAAA
GTTATGACCAACCTAGATAGCATATTCAAAAGCAGAGACATTACTTTGCCGACTAAGGTCCG
TCTAGTCAAGGCTATGGTTTTTCCAGTAGTCATGTATGGATGTGGGAGTTGGACTGTGAAGA
AGGTTGAGCGCAGAAGAATTGATGCTTTTGAACTGTGGTGTTGGAGAAGACTCTTGAGAGTC
CCTTGGACTACAAGGAGATCCAACCAGTCCATTCTGAAGGAGATCAACCCTGGGATTTCTTT
GGAAGAAATGATGTTAAACCTGAAACTCCAGTACTTTGGCCACGTCATGTGAAGAGTTGACT
CACTGGAAAAGACTTTGATTTGGGGAGGGATTGGGGCAGGAGAAGAAGGGGACGACTGAG
GATGAGATGGCTGGATGGCATCACTGACTCGATGGACGCGAGTCTGAGTGAACTCTGGGAG
ATGGTGACGGACAGGGAGGCCTGGCATGTTGTGATTCATGGGGTCGCAAAGAGTCGGACAC
GACTGAGCGACTGAACTGAACTGAATGATCTGTTAGGCTAAGATAAGTTCTTGAAGAAACAT
TATGATCTTCCTTTCCTCTGGGAAATTCTTAAACATGTGGAAATATCCCTTAAAGTTTAGATT
TAATCAAAAGGCTGAATTTAGTAATATTTAATCATTATGTTTCATCTTTAAAACTGAAGCCAG
TTCCCTAAAACATATCTCAACACATATGCATTGGAATTTTTAAAAAACTATGTTGTCTATTTT

CAATTTTAATTTTAATCACTTGATATTGGATGAAGATTATCTGAAAGGATGATATTTTCAATG
GTGGAATACTTTATCACAAAAAGAAAAGGTTTCCCTGTAATTAGTTTGCACCAAAACTTACA
AAGAAAATCACTACAGTATTTTTTTCCCTAACCTACGTGACATGGCAAATGAGCCAGAGAAA
TTTCAGTAAATAGCACAAATTTAGTTTAATCTTTTATTTTGTTTTAATCTTTATTTTGTTTCAG
AGCTTAAAGAATTTAAATGTTTTCTACTATACAGGATGGTAAGGTGTGTGTGCATAATCGTG
TCTGACTCTTTGCGACCCCATGGACTGTAGCCTGCCAGGCTCCTCTGTCCATGGAATTTCCCA
GGCAGGAAAACTGGAGTGGGTTGCCATTTCCTACTCCAACAGGATGGTAATATGAGATTAA
AAGAATAAATGGCAGAATATGGTTCAGTCATAACCAAAATATTTTTGGTAATATATGGAAAT
CTGTGATTGTGTACTGAACTACCACTGGAGGGTGGATGTTTTTCACAGCAAAACTACCAGTT
AAGTGGGTAATGTGAACATATCAATAAAAGGTGTGAAGGGACAAATGAATTTATGGTTTCT
GGTAATTTAGGAATAGCTGTGATTTTATAATTGTAGTAAGTTAATTTGCATGCAGTTCCAAAT
TTACATACCTAAAACCACACTGAATTTTGAAAAACTATTAGATAAAAAATGAGATGTCAAAT
AAAAATCAACTAGGATAGATAAACGCAAGGTAACCAAGGAAGAAAAAAAAGAAAATAATG
AAAACTGGGAAGCTTGTGGAGGAGAGAAATCTATCCTGAAACATAAAAATAGTATTGGGGG
ACAGGCTTCTTTATAACATGGGAAATATTTAGTGGCTTTTAATTTTGAGTTGCATGAAATGGT
ATGGTTTCCATGGGCTTATTTCACCTCCAAATATTACTTTAATGTAGTCCTCTTTTCTACAACC
TACTGTGATTATCCAGTTTTCCCTGAGGGGTTGCAGAAAATGAGAGGAAAAACAGGCCTGCA
ACAGTCTTGGAACCTGTCCAGAAAATGTTTTTAGTCCTTGGACTACTTGAAGGACTGTGCTCC
AAACCTTGTATACACAGATTGGAAAACAATGATAATCCCAGTGTATTCACTCTGAATAGGAA
TTTTCTGTGTGTCATTAACAAAAGGAATATTACAGAATACACTGAAACACACTGGGATACCA
TAGGATCTTGATTAACATTTTTACCTTTATCCATGAACAGGAGGTATTGTGGAGTAGCAGAG
TTTGCTTACTAAATACCACAACTGAATGTCCTGAAAGCCAGGAAAACAGTTGTTAGAACAAT
CTCTGAGGCTACATGTATCAAGCAGTGCAGAACTAGCAGGGCCAGTAGAGTCATGTGAGGG
TGACATTCCACAGCAATTCAGATTCCGTAACTTGCTGTTTACTTAAATCACAACCTCCTAACA
AAGTGAAGTTCGGGGATCTGTTAAGAGGATGCACGATTTGAGTCCTTGGTTGCCTTTTGGAG
AACACTGAGGATGACAGACACTTTCATAAAGACAAGGCTGATGTTACCTAAACCAACTTAA
GAATTGGAAGATTTCCTTCTACTTAACCTAAGGTAAATTCCTTTATCCAAGCCATTGTCAACG
CCCCCTAATGTGAAGCAAACTTTAATGTGGTTACTAACACGTGAGTGCTAAAATGAAGTTTA
ATAGGTAAGTTGAACTTCTCTTTTCCTTAAAATTTACTAAATTTTGTGCAGATACTAAAGAGA
AAGTGTTACTATACTCCTGAGTAGTTGTCATTTGCTTCATTTAAGCAGCTAACCAAATGTGTC
ACGGAGAAACATGCAGGACAATTGCAGGGTTCTGCTAATTTAGAATTCTGAATTTTTATGAA
ATCTACCTTATGCATTAAACAGTACTAGTCTTCCATTTTTATTCTGTTTAGGTTTTCATGTTTA
TGATTTACAGAATACAGTCATGAAAATAAGATATATAGAAAGAATTTTAAAGATTTATAACA
TTTCCAGGAAAATATTTGCTTCCAGAAGTTTTATTACATAGATAGATATACACTCTATATTTG
GGTGTGCATATATGTTACAAGAATTGGTTTGTTTTTACAAGCTATTATATGAGGCTTTGTTCA
AAATGAAAAATACAATTAGAGTAAGATGTTCCAACTATGAATACTTATGGATTCTTCGAAAT
CTTAATACATTTCAAGTTCTTCGAAGAAGTGTATTTGAGAAGTAGCACTCTAATCCAAACTCT
TTGGCCTCTTGAAAGATAGAAAACTCAGGCTTGACCACAAGCAGATCTGCTCCTTAAGTCTA
TGAGAACAAATCTGGGGAAATGATGATTTGATTTGATTGTGATGGCCTTCCCTGGTGGCTCA
GATGGTAAAGAATCTGCCTGCAATTCTGGAGACCTGGGTTGGGGAGATCCCCTGGAGAAGG
GAATGGCCACCCACTTCAGAATTCTTGCCTGGAGAATCCCATGGTGGAGGAGTCTGGCAGGC
TACAGTCTAGGGGTCACAAAGAGTTAGACATGACTGAGCGACTAACATTTTCACTTTAGGGT
TTGATAGACTGTTTGATACTGCTGTGCCACTCGAATGCAAGGTTTGTACCAAATGTTTTTTAA
AAGTGTCAGGGGAGAAGGATCAAACTTTTCCATTTCAAGGAAACAGAAAAAGAAAGGCATT
AGGAATGTATTATTTTCTATGTGGTGATTTCTAAAATTATTATTACATGACATGCTTCTTAGA
AAGTATTTTCTCAGCTTTAAAAAGGAATTTGATTAAGCTTCCCTGTCCCTGGAAACTGTGTGT
GTGCGTGTGCCCCCATGTGTGTATGCATGTGTATGAGTTTAACTCTTTCAGGAATCTGTCACC

CAAGTGTTGCATATCTCCAAATTTAACAGATTATCAGTTGGCACAGACAACAAAATTGTTAA
AAAGACGGACTAGAGTATAGTTGTCTCGTTTTTAGGAAATGGAACAGAATTACTGTTCTCAT
TATCCATCTGATTTCTTTCCTGGTTTTAAACATTCATCATGCTCTTCAAGTTTTTTTTTTTTTC
TGCTGGTAGTTAAAAAGAGTGAAAAACATCCTTTAGCATTTTAAAGCATATCATTTTGATCA
AGCCATCAACCAATGTGAATGTGTAAATTGATTCTCTAACTCTTCTTAAAATTATTTTATTTC
CTTTTTAAGACCCTACAGGGACTATTTATAACGTAGATGGCATGCCTCAAAAGAAAACTTTG
TTGTACCCTCAATTCAAAGAATTTAGTATTTCTGCCCCTTGAAATTACAAATTAATTGCTCAC
ATAATTTTTTCAATCTTTTTTGACATTGTTTAACTAACTAAATCTATATTTTGTTATTGATTAT
TGATTTTCCCCCTACTGTTTCATAACACCAAGCTTTGAAGCCAAGCTATCCTCTTACTTGAAG
ATTTCTGTTGTTCCCTCCTAATTATTTCCCTTCCACTGTATGAACACAAATTGTACTGACAACT
GTGCAAATCTTAACACTTTCTTAAGTTTCTTAGTATTGTTTAAATGTTGACATTTCTCCACCTC
CTCAGAGAGTTAAGTCAGCTTTGCAAACAGTTCAAATAAAGATTAGATCTGCCAACATTTTT
AGGCCTGTTGAATTTACATATTTATATGGCTCTGCCCATATAAGATCTATCCAGATATAGAG
AGGTTCTCTCTTTATACACGGCTCCTACCATGGCTTCTAGGAAACACAGGGGAAAGTTTGCA
TCATGAATGCAGTAGGTTTGTCAATACTTTGATCGTGTGCTTTGATTCATTAGATGATGTAGC
TTAAGGCTAAACTTTAATGACCCAGATATTCTGCATTATCCTTAATGTTGTCCTTTGAAATTT
TCCTTTAAAAGCGGTATTAAAAACTTCCATTTTAATCTCTTAAGCTTTCACTGACATTATCAT
GTCTATGTCTTAATGCATGTACTAGTCTCTTTAAATTATGTACGTATGTATTTCATTCCGGTAT
GTGAAGCCCCACCTCATCATATGAAACAGATAGAACCTGTCATTAGTTTAATACTTCCATAA
TGACCAGGGCAGCATTTGGAAAAATAGATATCTATTTTTGTAGCACACATAATCATGTACAT
CATATGTACATAAATATGTGTGTGTGTAAATCTCCATGGTTAGTTATCCCAAGGATTAAAGT
GATAAATCAAATTTTGCCTTATGGTTTTACACTGGTTAACTAAAGTTTTAAGATACTTTACAG
TGTATCTGTGTAGTATTATGTGTTATAGGTCTTCAGAATGAATTTTATTTTTTGAGTTAATTTC
AAAATAATTCTGTTAACTGTTAATTACAGAATACTTTCACTGTTCATTCCTTGTTTATTGTACT
TAGATATCATTTTCTTATATTAGGCTATTTAAAATTGTTATTCAGATTGAACAAATGTGCTAA
AAAGAATATGCAAATTAGGTATTGATCAAGAACTCATATATATATATATATATATACACATA
CACATTTTTCTTTCAGAAAGACTATAGACTCTTAGTCAGTCTATATGGGTTTTTATTATTATG
GCACATAGTAGCTGTGTGACCTCAACTAGTTGTCTAAAAACATAGCAAGGTTGTATGATAGG
TCAATAAGATAACTTATGTGAGACACTTATTTCAACAACTGGCAGATAAGCCCTCAACACAT
GTTAACTCTGTTATTGCATCATCATCATAATTTATAGGTCTTTGCTTAACCATACAATCTAGA
ATTTTAATGCACATGATACTAAAATTCTTTTCATTTGCTGAAGTGTTAAAAAAAAAGAGGGT
CAATTAAAAGTTTTTATCTACTACATAGAATCTCTATAAAGCTTTACAAATCTGTCGTGTGAC
TTGTCAGCTTGAGGCTGTAGGTAGGTTCTGTAGTTACTCTCTTTGACAAGACAGGAAATGAG
AAATATGACTGTGGGCTGCTTATTCACAAAACCTATGATTGTTATTGTTATTAATAGGGCTGT
GGTCAGAACTTGGGCCATGCCATGCAATTCCTCTTCTACTATGCTTCCATTGTATGCTTCTCA
GATATGTTGCAATATTTATAATTACTCAATGTACTTCACTCTTCCTCTTTTACTTCACAAGTGT
TATATGCCATGAATTAGCTAATCTAGAAATATATTCTAAAGGATTTTTGAAACCATAGAAAT
GCTAGAATAAAGTTTATTATTTTTCCAATGTTTGGAGAAAGCATTTCAAAGCATGACATAAT
ACTTAAAAGGGAATTACTGTGAAAGTTAATGGCATAAAAATGGAAATTTTCAGTAATTTAAA
AATTCAATAAAGTTTAGTTAAAAACTTTTAAAAAAAATTGTCAAATGTCAGAAGACTGGTTAT
TTCCTTATTAGCTAATAAGGAAAAGATATCACAAATGTTTAAAAATATAAATGCTAATATGC
TTTAGACAATTGGTGAAACTTCACTCAAATATTTAACAATAAAATAAGTACTTATAAACGGA
GTTTAGTATGAGCCAGTGTGACCAATGTATGAAGATGGGCCTTTTCACACATTGCTGTATAC
ATGCATGAACAGAAATTGGGTAAAATTCCCCTTGCCAAAACAAGTACATACTTCTCACCCAG
AAATTTCACTTTCTGCCATTTACTAGTGTCTTAGCACAGTAAACAAAATTATATATGTACATA
AATTTTTAGCATTGCTTTTGTAAGATCAAAGAACAATTTGGTTAGCCAAAAAGTCTGTTCAG
GTTTTTCCGTTATGTGTGTATGTTCAGTCATGTCCAACTCTTTGCAACCCCATGGACTGTAGC

CTGCCAGGCTCCTCTGTCCATGGGATTCTCCATGCAAGAACACTGGATTGGATTGCTATTTCC
TCCTCCAGGGGACCTTTCTGACACAGGGATTGAATCTGTGTCTCCGATGTCTCCTATAGTGGC
AGGCAGATTCTTTACTCTGAGCCACCAGGGAAGAAAGGTGTAACAGAATGGAAAAAGAAAA
ATCAAAAGTTCTATTACATATGATATTGGTCATTTAAAAGAAAATGGTACATTTTTAGGACT
ATTTTTTCTAATAATTGAGAAAGAAAAAAGAGAGTTAACACTATCAATGATATGAAAATATC
TCCAAGTCCCTTGATATTAAAGAGACAAGTTGTAGAAAGTGTGATTCAGCATATGGTTTAGT
CCATATGTGTATGTGTGTGTGTGTATATGTTATAAACTTTTAAGTTTATACTTAGGAAATCAG
TAAAATGATGACTGTATGCTGTGTGTTTATAAGTTTATAACATACAGAAGTATATATATATA
AGTTTATATATATATATATATGTAAGTTTAAAATTAATAAAATTCCTAGGTGGTAGTGACAT
ACCTAATATTGGTCACTTCTGAGGAATAAAATTGATGCTTGAATGAGACTTCTCATTTTACAC
CTTTTTATAATATTCAATATTTTTTACTTGTCCATATTACCTTAATGAATTCCCTAAAATAAAA
ATTTGCTTAATAGAAAATGATCACCATACCAATTATTTATTAAACAAATACTATATATGTCA
AGAACTGCACTGAGTTCATCACATTACTACTCTCATCTTTGCAACAATTCTGAGAGGGAGCT
ATTTCCACTATGACTTACAGAGGTAAGGTAGTAGCAACAAGTCACACACTCTAGGAAGGGA
TGAACTCCAAATTCTGAGTTAGGTCCATTTACTTCCATAGCATATGCCCTTCATCTCAAAGCA
CTGTATATCTGTGGTATGAACAAACAGTATAATAACCTCTTATTTCTGGCCATTCTCATTTCT
TTCAAAAATATTATTGTGGTTATGACAATCATGTGCATCCAGTCATTCAACTGATTACCAGCT
ATGACCCTGCTCTGTGCTCCTGGGGCTTCAGCAGTGCAATAAACAGACAAGAGTCTCTCATT
CCATGCAGTCAACATTCTAGCAAATGCAGAGAAAGGACCAAAAAAAAAAAAAAAAGCCAAA
TGCTTGTCTGATGGTGCTGTGTGATCTCCAGTAATCAGAGCAGAAGGGAGAAAGGGACAAT
GACTTGAGGGGAAGGGAAGTGCTTCATTGCCTCACAGTCAGGCCCCTGTCAAGTGCTGCACA
CAATCCACCTCAGTTAGTCCTTATTATGATCTCTTAGTTTTCTCCATTTTTTGATTAAGAAAGT
AAGAACAAATTGTTAGTTAACTCCTAATCCCCAGTCAATATAGCTAGTGGTAGTACATTGCA
AGTCGCTGTAGTCTGTCTGATTCCAAATTCTAACTGATCTTCCAGGTAAAAAGTTTGCAAAG
CACTGTGCCTAAGCTTACTTAGCATACTGACAGTTTGAGCTTTCATTCTGCTACATCTGTTCT
AGTGGACTTACAGAAGGTAATGTATACGCTCATATAAAAATATCCCTTAAAAATACTGCACA
TACATAATCTTTTAAAAATGAAAACCCCAACATGCATTACTGAGAAATGGGTGCAGCAGTTT
CCTGCCTTGTTGTTCAAGGTGAACATTTTGCTCAAAATACAGACATATGATTGCAGCTTTTAC
TTGGCTCTGGGTTTCCTGTGTTTTCCTGTATCAGGAACTGTTGTGCACAGTATTAAAACAGTT
AACAGCTGCCTCTGGGAATGCAAATTTGATTCTTTGGGCTGCTCATGGGAAAGTGTGACATT
TTCCTCTCCAAACACAAAGAATTTAGATGTCATTAGACTACTGATTAAATAGTAATCCAAGT
AAATTCAGTGTCAGCTTAAGTATCAATTTTACAGGTATTTTTTCTGAAATATCTTACTCTAAC
AATGTTCATTTTTTATATAATTTAATTATCCTTTCAGTGCATTTAAGATAGGCTGTTTAAAAT
AGCATCTGAACTTCTGTGTTGGTGGGCTATTTCTGAATTGCCACACTGGAGGATATTTGTGTA
CATGTGTACTCGTGTATATTTGTGAGAATTAAGGCTGAGAAGGAAATGTTATCAAAGCAGCT
TAAATACAATAAAATAGATTTTCAATATCAAAATAATTATTAAACTAGCCTTTATATTACCTT
TTTATTAGTATAACAAAGATAAAAAAATGTCACAGTGATAGTGATAAGGGAATCATCTTTTT
AAATTATCTAAAATCTAAAATTTTTAAATTATTCTTTCTCAAAAGCATAAATCATAATGAGAT
TATACAAGGCCATTTCTACTTTATCCTAATTAAGTGATATTTGGAAAAACAATCCCAATATA
ACTAAAGACTTCTATAACATTGATTTTTAAATAAATTTGTCTATAAAAATATCCTGGAAAAA
GAAAGCAATGCTCAAGGTAAAATATTGCTTTGGGTTCCTTTCCAGTTGTATTGCACAGCTAG
CAAATGAGAGAATATAACACATGCCTAGAAGCAATAGCACAAACAGCCCAGTGAGGACTGG
TTTAAATATGATCTAATGAAGTAAATGTATTTCTGCCTGTTATTGTTATGCTGTTGTCTTTCAA
GGCTGAGTTAGAGGCATTTATTCATCTGGTCTGACAGATAACAGTTTAACCTGAAGATTTTT
ATTTTCCCTGTAGGATTCTGAGAATTTCTTTCTAAGAATGGGATTTTCTTTTTATTTTTAATTT
TTATTTATTTATTTGTTTAAGAATGGGATTTTAACATGTGGGTCTTTTCTCCCCAAAGCTTAA
GGTCTTCTTTTCTGATGAAATTTTAAAATAATGCCTTTCTTTTAAAAGTAACTTCATTATATAC

TTTTACTATTTAGGATGGCTTTTAATATGAAATTTTATTTAAATATTATTTAAAATTGTCTTTT
TATCTAAAGAGATCTAAGAGAAATACAATTTCTTGGTTTTCATAGAAGGATTTTGCTCTGCTC
ATGAAATTTCTGTAAGACTAACAGAAAATAATAAATAGTGAAGAGCTTCCAGTTAGAACAG
TAGGCTATTTCCTTTTTTAAAGTCTTTTTGTCATCCCTGTTGATTGACGAAGTCCAACTGTTTT
AAAAATCATTAGACTTGACACACTTTGAATAAATGAAGCATAGAAAGTACATTTTATTTAAA
CTTCGTGAAGTAGGTTTTTCAAGCAGGAAAGTGAAAGAAGCAGGTAATTCTTCCTAATTTAC
ATTTTCCACCCCCAGGCCCGGTTTATCATCTGGAATAATGACTGTATATGAAAGGTCGCTCA
GCAAGCTAGCTCCTTTTGTCTCTCTGGCCTCTGACTTACTTGGATGGGAAAATGGCCAACAG
AAGGAAAAAAAAAAAAGACAGCAGAGGATCTCAGATATAGGTATTAGGTCTGCACTACAATG
TAAAAACAGACCCTCAAGACCTATCACACCCCTCCCACACACTCTAAGTGGGAAGTGTGGG
GTTTCCTTTTTTTTTTTTCAAACTCCTTTTGGAACAAACACTCATTGGGATCTGACTGATGTTG
CCTTTCTCTTTTTTTTAAAGTATATTTAAACTCACACACTTTTTATAATGAGGATTTCAGTGTA
GGGTTTTCTGCAGGATACCATTTGGTCCTACTAAGAACTGACTGTTCAAATGTTGGAACATTT
GATCGGAAAATACTTCTTAGTCTTTTTAAAGGTAAGTCATACTGATTTTTTTTTTTTAATTTCT
GTCTCGTAAATGACAGGAGATGACACTATTATATACACACCCTTAAAGTTAAAGAACAGCA
GCAGTAAGCAGGAACTGTTTTTTGAAATCATTAGGCTTTTCCTAAAAGCTAGGTTTGTGCTGT
TTCCAAAGTTATCAACCTTACCTATTATGTTTAAGAAATATTAAAACTTTGTCTAATAATCAG
TGTCATGTTGCTTCTTGGTGTTTAAAACCTTATGTTTATGCATAAACAGTTTTTGTTAAAATG
ATGTACTATCCAATTTTGTTACTTCAGAACTTTTATTGTCTACTTAGGGTATTTGTGCATGGTT
TTAAATTATGAATGAAAATCAACATAAATAAAAATCAGTTTAGTAGGATTAGTTCTTAAAAG
TCACTTGACACTCTTATGCCAACATGCTTTCTAATATGCAATTCTTCAGTGCTTATTATTTTAG
AGAGTCTTTGGTTTATTTAATCTTCTATTTTTTAATGTCACAATTTTTTGGTGAACCTGACTTT
GTCTACATGAACAGCAGTGTATATTTTGGATATATTCCTCTTCAGCATTTAAAAAAAATTCAT
TCTCATTGAATTTTACTTGCTGCTGGGAATGGTAAATTAATTCCAAATCATTTTGATTTATAT
AACTGTATAAATTATATAAAACTTGGTAATAGAATTTTTTAAAAATTGAAGTTTTTGTGATTT
TAGGAAACAATACAAATAGCAAATTATTTTCTTGAATGACAATACTAATAAATGACAAATCC
TTCTGGAGAAATTAAAAGCTATAAATTAATTATAGTAAGTGAACTCATACATAACTGTAAAT
GGCATAAATTCACTACAGTAAATTTCAAACAGTTTCTAGAATTGTCAAGTAACATATTGAAA
AATGTTTCTAAAATCTTTGCATGTACTTCAGTTCTTTAGTGTGTAGTTTTAAATTAGTTAAAA
ACAAAGTGCTCTTAATATTAAATCAAAAAATAACCAAAATAATATTTTTCAAAAATTTTGTT
ATATAGAAGAGTCCAGTGGTCCACTGGATTTCATGAGAGAAAAAAATACACAGAAATTTGT
TTCATTTACAAGAATCATTACAAGAACAGAACAATAAATTTAATACACAAACATCAGATAG
AAGTTAAACTGAGAGGAATAAAACTATACTTAGGTACTGGACAGAATGCACTGAAAATTTT
GGAATTTATCTTGGGAGTTACAGTTGCTACCCTATAGGCAAAAATCTTGTGGATTCTTTCTTC
CTGAAAGAACTGATTCTGTAACTATTTTCTGATTTAATTACAAGGCATTTTTTAAATTCAGTG
GCCAAAATCATGGTTGAAAGTTTTGAGAAATTATTCTAACCTAGGAGTTGATTTTTCATTTGC
ATAATATTCTAAACCCCTGTATACCTAAGAATAAGCCATCTCATCAGATTTTATTGTAATCTA
AGAGTTTTAGAAATGATCATTTATTATTTATGAAAAGACACATTTACTAGAATTTTAGAAAA
TACTTGGCCAATTCACAATAATATTTTACTGTTGTTACAGTTCAATATATTTCATAATATTGC
AAATAAAATAACAGGTGCTATGATAGGAAATTTTATTCCTTCTCACTGAATTTTACTTGACAT
TTATAACCATATATAAGACTCTGATGCTGGGAGGGATTGGGGGCAGGAGGAGAAGGGGACA
ACAGAGGATGAGACGGCTGGATGGCATCACGGACTCGATGCACGTGAGTCTGAGTGAACTC
TGGGAGCTGGTGATGGACAGGGAGGCCTGGCATGCTGTGATTCATGGGGTCGCAAAGAGTC
AGACATGACTGAGCAACTGAACTGAACTGAATGATGTATATATACCCATTTGAGAGAAAAC
TGAGCCACAGAGAAACTATGTTAGCCTGCAAAGCAAACCTAAATTACTTGAATTCAAAAGC
CCAGAATAATAACTATTGAAAATCTTATAGTATCTTCCCATTCATAGGAAGAGTTGGGTTGG
GGAAAAAGAAACAATAGTGTTTCTATCAGGATTCAAAAATCAAGAATGGTTCTAGATAATTC

TATTTAAACTGATAAGACTCTGAGAGAATCATTTCAAATCTATAGTAATCTCTATAATCAAG
TACTCTAAAACCTACTCCAAAAGATTGGTGTGTTTCTAGAAGAACATTTAATGTGATAATGA
AGTCAATGTAATTATTGTTTTAATTATTTTAAGATTACCACAAAAATAAAAGTTGTTATATTT
GAAAGGCATAATTTAGTGAAGTGTGTTATATTATGATATCTATACATTTTCTATTATATATAT
TTACCTCCATATATTCTGATAGGATGGAAATGCCCATATACATTAAAAAGATTATGGTAACT
TCAATAAGAGAAGATGATGAGGATTTCTGAATCTAATTTAAACATTTACCACAGAGCCCTAG
GGTTCTGCGGGGATGACTCAGAGCCCAAATGAGATGTGACTAGAAGGGGAAAGGGCCACAG
TTGCTGCCCACTTTGATCAGAATAGATATTCTTTTAACTACCTTCAAATGTTGGGGTTGCATG
TAAAATAAGTACTAATCTAGTGACCTTATGTATTCACTTGATGTAACACAACCAGATTTAAA
TAACTGATTTAACCAGCTTGATATGAATCTGCATCTTACTCATGTCTAGGGAATCATGGCTTT
GAAAACCCACCTAGATATCATGTAAGGTAAGAGTGTGAAAAGGAAGTTAGTTTGGAAAGCT
AATTTTCATATTCAACCTGTCGTTGAATTAATCGCCCCCAAACTCCAAACCTGGGTTTTGTGG
GCAAATACTGTATTTAGTGAGGAAAAGCCGTGTATTAAAAGAATTACTTGTGTGTCCAGTAG
AAAACAGAGATGATCTGGTTTCCTATTCTGAAGACAAGCAGAAGGAAGGCTTCTGGATAGA
ATAGGAAATTTGGGATATTCACTCATGAGTCAGAAAATGTCATCCAGGTCCTGCATTCATTC
ATAGCCCAAGGATGTAATATGAAACTGTTTTAAAGTTTCATAGTTCATATCCCAGGAAACCA
TACCTCTTGGCCATGCCTTTTTCTGGGTGGAGTTTCCAAGGAAACACAGCTCTATACACACAT
GAAGATAATCACAAATTCTCTGGGAAGTGTCTCTATTATTCTGACCTACTTATGGGCACACTT
CTTAAAGCTCAGTCTCAGTTCCGACCACCAGGTTTCCAACACAAATGGAAAGGCATAACACT
CATAAGTTCATCTAGAAACACACTGTTGACTTGACAGCAAGAAAATAAACAGCCCACATGT
AGTTACATTCACAAACTCCACAAAAGAGTAGGATTTACAATCTGTGAACTGACAGGACACA
GTCAAGAAAGGTATGGGAATAGTGGGATCTTACAATGTCTTTATTTTCTTGTCTTTTTTCTTTT
AAGTCTCAAAAATAGGTTTTCTTTCCTGATTATATGTGAATTTTTTGTTTTCTAAATTGAAGT
ACTTTAAGCAAGCAGCTTACTCCTTGGAAGGAAAGTTATGACCAACCTAGATAGCATATTCA
AAAGCAGAGACATTACTTTGCCAATAAAGGTCCACCTAGTCAAGGCTATGGTTTTCCAGTGG
TCATGTATGGATGTGAGAGTTGGACTGTGAAGAAAGCTGAGCGCCAAAGAATTGATGCTTTT
GGACTGTGGTGTTGGAGAAGACTCTTGAGAGTCCCTTGGACTGCAAGGAGATCCAACCAGTC
CATTCTAAAGGAGATCAGTCCTGGGTGTTCATTGGAAAGTCTGATGCTAAAGCTGAAACTCT
AATACTTTGGCCATCTCATGCGAAGAGTTGACTCATTGGAAAAGACTCTGATGCTGGGAGGG
AATGCGGGCAGGAGGAGAAGGGGATGACAGAGGATGAGATGGCTGGATGGCATCACTGAC
TTGATGGACATGGGTTTGGGTGAACTCCAGGAGTTGGTGATGGGCAGGGAGGCCTGGCATG
CTGCGGTTCATGGGGTCACAAAGAGTCGGACATGACTGAGTAACTGAACTGAATTGGACTG
AAGCAAGCAGTAAATTATTTCTAATAAAGAAATAAGGGTAAAGAATAAAATTTAACCTAAA
ATTAGCAAAAAAAAAAATTTATTAGGAAAGAATCTGTATGACTAAGAAAACCAAGTGTGCA
AAATGCAAATCTTATCTGCTCTAAATAGCTACATTTGAGAGAATAGTGGAGACAAACTACAC
TTTATACAGCTGTTGTTGAGTACAGCATGCTGAGTAACCTGAACTTTTCTCATGTATTCAGTG
CATGTCCTATTTAATTTGTTGGTTCCTCCTTTGCTTCAGGTGAATAAAACTACGGTGGCATGT
ATGTTCGGTCGCTAAGTCCTGTCTGACTCTTTGCAACAACCCCATGTACTTACTGTAGCCCGC
TAGGCTCCTCTGTCCAATGGGATTTTTCCAGGCAAGAATATTGGAGTAGGTTGCCATTTCCTT
CTCCATGGAATCTTCATGACCCTGGGATTAAATCCACACCTCCTGCATTGGCAGGCAGATTC
TTTACTACTGCGCCCCTGGGAAGCACAAATTACAGCTGACCCTTGGACACAACAGGTTTGAA
CTTTGTCAGTGCACTATGGAGTATCTTTTCAATAAATACTACAGTACAACCTGATCCCATATA
TGCAGTTGGTTGGATACCACAGGTAAGCTGATGGTGTAGTTATATGTGGATTTTCCACTGCCT
GCATTGTCCATAACCCTCTGTGTTCAAGGGTCAGCCTGTATTTGTAATTTTGTAGTATGAAGC
ATAAGCTTGAAGTATGATTATAATAACAACACTTATGGTAACAAGGCACAGAAGCATGATA
AACCCAAGACCCTCAGTGGGTGCCTGAGCCTAGAGATAGCACCAAACCCCAAGTAATTCCC
CTTCATCCAAGATTTTACTTTCTACAGATTCAGTTAGCTTCAGTTGACCACTTTTGACTGTGG

ATAAGTGAAACCATGGAAAGCAAAGCCATTGATAAAGAAGGATTACATTTATATAAAATAT
CTTGCCTATTTTTAGAGAAAAAAAAAAACTTGCATCAAGATAACAAAATAAGCTGGGCAAAA
GTTCATAAAGTCAATCTCTTTCTGTCAAAAATATCAGTTTATGTATGCAAGAATGCGAGTCA
CACTACTATTTTGCTTAGACATCATCCAGACCAGTGTTATATTAGTAACTGTCTAAGCATGCA
ATTTAGCAGAATAAATCATTGACACTTATGTTTTCTGATGAACTTGACTCAACAGCACTGGG
CTGTGTGCACTATCTCACTTCAGTTTTAAGTTCAAAACAGACAGAGTCTAGGCAGTACCCAG
GTCTGCCAATAACTTGACATCTCTGGGCGCAGGGTCCTTGGATGGTATAATAATGCACTTTC
ATGTTTAGAGAGGAAGGTGCTAAAGAACACAGTGGTTCATCCAGCAGATTTTTGGGGACTTT
TTGCCCCTTGGGACTCTTCACTATAGTCCATGGGACTACTTAAATAAAGGATAATGATTCTG
GGAAAGGTCAGACAGATTCTCAACTAGGTGAAGGCACAGAATCTGGGAACAGACAAATAAT
ACACACCTATATCCATTTCAGAAATAAAGATTTATAATTTTAACATCATTTTATATTCTATCT
GACAGAACTGCAAAGGACATTAATATTGAATAATTTTGTGGCATTTATGTAGACCATATCTT
TCCAAAAGAGATGATTTGAGTTGTATTCCCGATGTTATATAAACAAAACTAGAATCTGACCC
TTGGGGTTTCCTATCCAATTTTTTAATAAATTGCAATCCACAGGTTCTACAGAGATGCTCCTC
AAGCATCCTAAACATGGGACAAGATAGTGCTAGGTGGGACACAGGAGACCTAGTTAGGAAT
CAGGGTCTCGGTGGTCCCACAAATGCTGTAATCTAAGTCACTTGGTTTGGTCTTCCATGTAAG
GATTTGAGTGAAGAATCAGTCTAGCTGCTTAAATGAAAAAAATGGGGGTCTCATCTTATAAT
CTTACTTTATACATGAAGAAATTGAGGAACAGAGCAATTAAATAGCTTGCCCATGGTCATCT
GGCAAGAAAGTGGCCAATCGCACTTTGGTTTTATCTCAAGTCTCCCACCCTTAATTACAGCA
GTTTGGAACCCTGCATTTTGATGTCTACCTCAAATATAACTTACAGATGAGAACTGGGGAAT
GGTCTATAAGATGGGCATTCAGTAAAATAGTACTTGATAAGCTCCCCTGAATATGGTCACTT
ACATACAGTACAGCACACTCGAATGCTATTCACAAGTAGAAGAGAGATTCTACTTGAGAAT
ATCTAGTCATTATTATCAAGTTTAAATAATTTATTCCACTGTATTATGACAATCCTAGCCAAA
GCCATAGGAGGGAGCATGTTATTCCTCATCCTCTGTGTTAAATCATTTCAGTCATGACCAATG
TGACCCTATGGACTGTAGCCCGCCAGGCTCCTCTGTCCATGGAATTCTCCAGTCTAGAATGG
AGTGGGTGGCCATTCCCTTTCTAGGGATCTTCCTGACCCAGGAAACTAACCTGTGTCTCTTAC
ATCTCCTGCACTGGCAGGCGAGTTCTACCCCCAAGCAATTAATAAAGGAAGGAAAGCTATG
CCAAACTTAGTGTACTGAAAAGCAAAGACATCACTTTCCTACAAAGGTCTGTATAGCCAAAG
CTATGGTTTTTATAGCAGTCATGTACAGATGTGACAGTTGAACCATAAAGAAGGCCGAATGC
CAACAAATTGATGCTTTCAAACTAGGGGCTGGAGAAGACTTTTGAGAGTCTCTTGGACTACA
AGGAGATCAAACCAGTCAATCCCAAAGGAAATCAACCCTCAATATTCATTGGAAGGACCGG
TTGAAGCTCCAATACTTTGGCCACCTGATGTGAAGAGCCGACTAATTAGAAAAGACCCTGAT
GCTGGGAAAGACTGAAGGCAACAGGAGAAAGGGGTAGCAGAGGATGAGATAGTTCTATAG
CATCATGGACTCAATGGATATGAATTTGAGCAAACTCCAGGAGATAGTGGAGGACAGTGGA
GCCTGGCGTGCTGCAGCCCATGGGTTGCTAAGAGTTGCTAATGACTTAGCAACTAAACAACA
ACAACTCAACTTGCTCAGAGACATAAAATTCCACGGGAAAAATAAACCACTTGTTTAGGCTT
GTGTGTTTTGTTTACATTGGGAAAAAACATGTGACATTAATGACTATTCAAACTTCTTAGCCA
CCAGGACCTCAGTGTGAATCAGTTTGTTCATCCTGATTTACAGTGGATCATCCTAGGTATTTG
GTATAAGAGAATGGAAATCCTGAAGAATGAAAAAAGTTTGTAAGATTTGCCTTTTTCCATTT
TGAGGGAAAGTCTTATTCCTTTCTTTTAATAACATTATTCTTAACATTTTCTTAGCTATGCAAT
TAATAATTTTAAAAAATTAAATGCTTACTCAAGTGGAAAGAAATACTAATAAAACCATCAGA
ATGGTACTGTAACCACAACCAAGCTATGGAGGGAAAAATCTGAAACCATAACGAAGTATGA
TTTTTACTATTATCCTTACAAGACTGACAAAATAGTGTGTGTCCCTCCAATCTGTTGCAATTT
TTGTCTTCCTTGGGCAAGGGTATTCTGAATTAGGGAGTATTTGTTTACAAGCTACAAAGCAC
ATTCTGTTTATCAATGTCAGGTCAACTCTAGTACAGATTTCCTACTGCAATCAAAACTGCTTA
AATAGTATGTTTTAGGTATTTGAATAATAAAGTCTTGTTACCATAGGATCTGCTTATTATGCT
AGGAATTTTTTCCAAAGATAAGACTAAGTGAAATACATATTGACACAATCGAGTTGATTTTA

ACAGAATTTTTAAGAATCAGTTTATTGTTGTTGTTTAGTCTCTAAGTGTGTCTGATTCTTTTGC
AACCTCATGAACTGTAGCCCACCAGGCTCCTCTGTCCATGGGATTTCCCAGGCAAAAATACC
GGAGTGGATTTCCACTTGCTTCTCCAGGGGATCGTCCCAACCCAGGGATTCAACCCACATCT
CCTGTATTGGCAGCCAGATTCTTTACCACTGAGTCACCAGGGAAGCCCTATGAATCAGCTCC
CTACAACGAAAGGAACACTATTGCTCTTGAATATGTTAAAATTTATGTATTTGAGGCTAGTA
AGAAAGCAATACCTATTACAGAATCTAGATATACTCTACCTTACATATTTTCTTCAAGTTCTG
TTTACCTTTAGATGATATAAAAATCAGTTATTAAAAAGAAGTGAAAATATTATGTAAAATAT
TTCAACATTTTCAACACAGTTGATAAGAAAGAATTCAAGCATGAACTCAATATCCATGAGAA
TGGAAATTTCTTTTAGCAGTGATTATAATTTTGGTGATATTAGAGAGTAGTGTAGTGTTAATT
TTCTGACAATGTATTTCCCTAATTTACTCATGTTTTTTGCTTTAACAGATAGGCTACCAGTGA
TTAGAAGAACTGACTCCTTCTAAGACTCATCAGATCATTTCCTGTAAAAACTGTATCTTTCTA
ATTGTGAAAAAGGTAAAGAAGACATTTGTTATCATTCTTGTTTAGAATTTAGATGGAATAGA
CTCCAGTCAACCCACATTCAGTTTGCATAACACCAGGTTCAGTTCAGTTCAGTCGCATCCGA
CTCTGTGACTCCATGAACCACAGTACGCCAGGCCTCCCTGTCCATCATCAACTCCCGGAGTC
TACCCAAACTCGCATCCATTGAATCAGTGATGCCATCCAACCATCTCATCCTCTGTCGTCCCC
TTCTCCTGCCCTCAATCTTTCCCAGCATTAGGGTCTTTTCAAATGAGCCAGCTCTTCCACATC
AGGTGGCCAAAGTATTGGAGTTTCAGCTTCAGAATTAGTCCTTCCAATGAACACCCAGGACT
GATCTCCTTTAGGATAGACTGGTTGGATCTCCCGGCAGTCCAAGGGACTCTAAAGAGTCTTC
TCCAACACCACAGTTCAAAAGCATCAATTCTTCAGTGCTCAGCTTTCTCTATAGTCCAACTCT
CACATCCATACATAACCACTGGAAAAACCATAGCCTTGACTAGATGGACCTTTGTTAACAAA
GTAATGTCTCTGCTTTTGAATATGCTATCTAGGTTGGTCATAACTTTCCTTCCAAGGAGTAAG
TGTCTTTTAATTTCATGACTGCAATCACCATCTGCAGTGGTTTTGAAGTCCAAAATATAAAGT
CAGTCACTGTTTCCACTATTTCTCAATCTATTTGCCATGAAGTGATGGGACCAAATGCCATGA
TCTTAGTTTTCCGAATATTGAGCTTTAAAGCAACTTTTTCACTCTCCTCTTTCACTTTCATCAA
GAGGCTTTTAGTTCTTCACTTTCTGCCATAAGGGTGGTGTTATCTGCATATCTGAGGTTACTG
ATATTTCTCCCGGCAATCTTGATTCTAGCTTGTACTTCTTTCAGCCCAGTGTTTCTCATGATGT
ACTCTGCATATAAGTTAAATAAGCAGGGTGACAATATACAGCCTTGATGTACTCCTTTTCCT
ATTTGGAACCAGTCTGTTGTTCCCCAGGTAGTACTGTATAAATGGCTGTGTTCAGGTGGAAA
AGCCAGATAGAGGGAAAAAATGTCCATAAGTGTCCCAGGCTGACAGGTAGGCAGAGGGCA
AGAGTACATATGCATATTCTCCATAGATAAGATTTCAGTATGTTAATTATCCACAGAAAAAA
AAAAAAGAGGAGGATTTTGAGGAATTGCTCTATGTGATGATATTTATTGTTTCTACCACTGG
TAGGTAGTCAAATTAAAAAGAAAATACAAGACCTAGAAAGTTTTCTTAAAAAAAAAACAAA
AAACGTCTGTTTATACCTGGCAAAATGATTTGTTTCAGTTGCGTGTTTTCCTTGTACTTTGATC
TTTTTGTACTGATATTTATGCTTCTGTTTTATGATCTCCTTCTAACGAAAGAACCAGACCTCTT
GGCAACCACCTTCATCAGACCTGGGAGTTTGAAAGAAGCAGGTGCTGAACCTCAGTTTTTCC
TTGAACAAGAAAAATGGGTTGCAATAGAAACTGTGGGCTCATCGCTGGTGCTGTCATTGGTG
CAGTCCTGGCTGTGTTTGGAGGGATTCTAATGCCAGTTGGAGACATGCTCATTGAGAAGACA
ATTAAAAAGGTACAAGTTGTATCAAGAATATTTCTTTTCATCCTGATTCAGTCTACCTTGATT
TGTGTTTGACTTAGGGTTTCTGTTTTATATTTCATTATAACCAGCAATTTAACACAAAGGTGA
TTTTGAACAACTGAAACTCTACATGACAGAGAAACAATCAAAGTATGATGTATACTCCATAT
GAATGTACTGTCATCTTAAGTCTAAATATTTTAATTTCTGATAAAAACTATTATAGGTATGCA
CATAAGTAGAAATAATTCCCTTTAATTATGTCCAAAGAGTATGCTGATATGCTTTTCATATGC
TTTTGAATGAGAAATGAGTGTTATTTTGTGAGTACTATTTATACTATAAATAGTACTATAAAT
AGTACTATAAGTGAGTACTATTTATAAAAGTAGTTGCACTTTGGTTACAGTTGATTAAAGTA
AAATTATATATAACTTAATACTGTGTTTAATATTGATTTAGGAATATTTGGTAAAACTGGTAA
AATTTATTTTTGTTTGGTAAACTGTTCTATATGTGTGTAATGTTAATAATATTATTCTTATAAA
CATTTCCTACTGATGAAGGTAGATAAGGGATTTTTACAAAATACTTTCAGAAATGTTATGCA

GAACATATCCCAGTATATATATGTATGTATATGTGTGTATATATATATGTGTGTGTGCATGTG
TATATATATATATGTATACAAACAAGTTCACTGTAGATTAACTAAAAATAAAGTGACTTAAT
ACATAGGCTTTGTACAACAAACTAGAATCTGTCTCTCATCCTAGTTCCTTCTAATTCAGCTTT
ATACGCACCAACTTTAATTGATAAAGAAAAGGAAGACTAGGGTAAGGAGATGTAGCAAATT
CAAATAACTTCAGGGTTTGGAGACAAGCCTTAAAAAAAGCTACCAAGAACAATATATAAAC
AAGACAATGACTACTGTGAAGTTAAAAGTGTGTGGCCTACTGAGAGGCATGTATGTTTATTT
TTAAAATATAATGCTGGCCAAATAAACTTATCTGAGTGCTGGCCTCAGCTGTCATCCTGGTTC
AGATGCCAGTGGTCTAGATGTCAGTAAGCACAATGTACGGAGGAGATCAACAAGAACTGAA
TCATACAATGGGCTAGACTACCAACTCATACTGGGGTTTCTGTTCCTATCAAGACCTTTATCT
GTCTCATCCATTCACCCATGTTTGACATCCTGCTCTTACTAGGAGTTTAAATAATTACAATTA
TTTTTCACTAAAGCATTGCCTGACTTAATATGAAGATTTAATTTCAAATCTAGTTACCCAGGA
CAAGAAGTTACTATTTTCCAAAAGCTTTTCGGTTCGTTTTGTTCAGCTAAATGCTTAGGCTGT
AAATTTAAGAATTTTTCTGACTGTACTATTATAGCTATTGTCTTAGCACAACAGATTAAGAAA
TAGGACATTTTTGGTACAGTTTTTACACTTGTATGCTATATTTGCTTAATAACCACACAGGCA
TTTCTATCTTTTCTCTTGCTAACTGACAGCAAAAATTTTAACTTCATGAATTTAACTGGAAAT
ATTATACATTAAATACCATCTCCTTCCTTGGAATTCCGGTTTTCTTGATATTATTTTAATATAG
CTCAATAGAGACAGACAGGAGATGGTGAGGTATGGAGTGTCTAACGTGTTAGAGAGAACTG
CTATTGTCCTGTATTCTTTCTGCTCTAGAAACTCACTTCCCTTCTGTATCTGTGGAGTGAGAA
CAGGATAAATTTGACACACAGACTATAACTTTTTTTAATCTTAAGAGCCACAATAATCTTCC
AATGTACAGCATCTTAGACTAAGATTCTTAGACTAATAATTTTTATTCATACTTGATAATCAG
TAGCAAACAGTGATCATCTATTGTTTCTTATTATTCAACAATATTATACATACAGAGTCTCTG
TGTCATGAGAGGCACTAACATCTATTTTAATTTTTAATATGTATCTCTAAGTTCATATGGCTA
ATTTTGAAATAAGAGTTTTGAGACATCAAAAATTTGTATAGAGAGAATCAATATTTATCTCA
ACCTGATTCGCACCACCGGGAGCCACTAGAACTCAAAAGCAATCTTGAGAAAGAAGAATGG
AACTGGAGCCATGACATATAGATCAATGGAACAAAATAGAAAAGTCAGCAAGAATATACAA
TGACAATCTCTTTAACAAGTGGTGAGTACAAAAGTACCTAAGCAATGTAACTGTGAAGGCCT
ACTGAGAGGCATGTATGTTTATTTTTAAAATATAATGCTGGCCAAATAAACTTATCTGAGTG
CTGGCCTCAGCTGTCATCCTGGTTCAGATGCCAGTGGTCTAGATGTCAGTAAGCACAATGTA
CGGAGGAGATCAACAAGAACTGAATCATACAATGGGCTAGACTACCAACTCATACTGGGGT
TTCTGTTCCTGTCAAGACCTTTATCTGTCTCATCCATTCACCCATGTTTGACATCCTGCTCTTA
CTAGGAGTTTAAATAATTACAATTATTTTCACTAAAAGCATTGCCTGACTTAATATGAAGATT
TAATTTCAAATCTAGTTACCCAGGACAAGAAGTTACTATTTTCCAAAGCTTTTCAGTTCGTTT
TTGTTCAGCTAAATGCTTAGGCTGTAAATTTAAGAATTTTTCTGACTGTACTATTATAGCTAT
TGTCTTAGCACAACAGATTAAGAAATAGGACATTTTGGTACAGTTTTTACACTTGTATGCTAT
ATTTGCTTAATAACCACACAGGCATTTCTATCTTCTCTTGCTAACTGACAGCAAAAATTTTAA
CTTCATGAATTTAACTGGAAATATTATACATTAAATACCATCTCCTTCCTTGGAATTCCCGGT
TTTCTTGATATTATTTTAATATAGCTCAATAGAGACAGACAGGAGATGGTGAGGTATGGAGT
GTCTAACGTGTTAGAGAACTGCTATTGTCCTGTATTCTTTCTGCTCTGAAACTCACTTCCCTTC
TGTATCTGTGGAGTGAGAACAGGATAAATTTGACACACAGACTATCTTGAGAAAATACAAT
AATCTTCCAATGTATCTTAGACTAAGATTATACTAATAATTTTTATTCATACTTGATAATCAG
TAGCAAACAGTGATCATCTATTGTTTCTTATTATTCAACAATATTATACATACAGAGTCTCTG
TGTCATGAGAGGCACTAACATCTATTTTAATTTTTAATATGTATCTCTAAGTTCATATGGCTA
ATTTTGAGGAAATAAGAGTTTTGAGACATCAAAATTTGTATAGAGGCTAAGAGATAATCAAT
ATTTATCTCAAGAAATATAAATTTAATTGGGAAAAGCATATTTAAAAAGCTAGAGACTTGTG
AAACTCTGTAATTGAATTCTAAAGCATACACTATTTAGAAAGAGAACTTTCATAGAGTATTC
AATAACTTCAGGAAGAAGGTGAGACTTGAGTTGGATTTTGAAGTATAAAATTTTGATCGCCC
AAAGAGAGTTCCAATAAAAGTGTCAAGGGACTGCTTAATGCCATAGAAAAGAGTTGTGGAA

ATGCAACTAAATATTGTGACGATAGAAGGAAAAATATTTTTTTAATGTGTCAAAACAAAGAA
ATAACAGCAAAGATTATTGGAAAGATAATAACATGGGGTAAAATTTAGGAAACTTTTGAGC
ATCAGGCAGAAGACTATTTAAGTAGTGACATGACAGGCAATAGTAGTGTATATTTACCTGTA
GTTGTTAGATATAACAAAATCTGCATTTAGAGACTGGTATCAAATATACTGAAAAAAGAAAT
GGCAATACTATATACTAGTAAGTTATATGCCACACATACTAATATATTATACTATATACTAA
ATAAATAAAGCATTTTTTAGTCCTACTAAGAGCTTCATATATACTAATTCATTTAATCTTCAC
AAATATCAATGAGAAGATACTACCATCACAAGACCTTAAATCAACCTGACAACTTACCCAGC
CAAAAGGAATATGAGTTAGTTTCTAAACCTAGGCAATCTTAAGGCAGAGATGAGATGATTTA
TACATTTTGCACTCTCTAATTCTACAATAGAAAGTGTTGGAAAGTGAGGAGACCAAAAACAG
GAAATGTTATGTAGGACATGACCACTTTCAAGGAGAATTTAACACTCACTTTTAAACAGATT
TTTGCACAGTTTGCATATAACAAAAGGTTCTCTGGTCTAGTGTGATGGAGTATGTAGGACCG
ATGGGTGATGGAATGCAATGGACAGAGGAACTAAATAAATAGGGGGAGAGAGGAGTAAAA
GTTTCAAACTACAAGATGTCCAGTTGTGTTCTTGATAGAAATATGCATACCTTAGAAGAATA
AAGCACAGAGGTTTTATAAGTGATTTGAAAAGAACATCCGTGTATATTTCAAAGGGAAGGTT
CAGAGATTTGTTCATTAGTATAGAAAATACAGATAAAACTAAACAATAGATATGTTTAAGGA
GAGTTTGAATATAGAGACGAAAGAGCAGAGAAATTACTATGGGATTTATGCATTCAGAGGT
AAGTAGGAAGATAACAGAAAAAAATATTTTATATGAAGCAATGAAAACAATAGACTGTAATT
TTTTTTAGAAGTTATTATGAAGTCAGTTTTTAAGGAAAAAGTAAAATTATCTCAGGCAGATG
AAAATTAAGGAAATAGTAGTTAGAATACAGTCACTAATAACTTTAAAATTATATTTTCAGTA
GTGCCAAGTAATAAAGTGGGATAAAATAAAGAAATAGAGTGTTTTCCCTTCTTATTTTTGAA
ATGAGGGTTACCTGCAATCTTTGGGTCATACTGTAACCCAGCTCAATATTCACAGACTAAGA
CATCATCTCTAAGACATCACTTCTCAGCCTTTTGGCTAAGATTACGTGGCTTAGATGGTAAAG
CATCTGCCTGCAATGCAGGAGACCTGGGTTCAATCCCTGGGTCAGGAAGATCCCCTGGAGAA
GGAAATGGCAACCCACTCCAGTACTCTTGCCTGGAAAATCCCATGGTCAGAGGAACTTGATA
AGTTACAGTCCATGGGTCACAAAGAGTTGGACACAACTAAACAACTTCACTTTCACTTTTCA
AGACATCATCAGAAGCCCTTCAATGGTCAGGAGCTGTGTTGTATAGTTAAGACAGCTTGCTT
TTGCGCTCAGATTTTCATATAGCGGTGTTGACCTTGCTTGTTTTAATTATGTAAAGCTGTTGT
GACTTTTTCTTCATTCAAATGAAACTGTTTCTTTTAAATTTAGTTTACAAAATTATTTTAAATA
TAAGATTAGCATATGTATCTGTTTGGCATACGTGTAGATGTCAAATCTTTGAACTATGTGTAA
GCACAGAGAGCTAAGTGTCTCGCCCCTGCCTCAAGGGCACTTAGGTTAATACTGCTATTTTA
CAAACATATATATATATATATATATATATATATGTTGTACTACCAGACAGTTTTTCTCTACAATT
AAAACTCATTGCATGTAACTTGCATTATTTAATGGACTAACAGCAAATTGGCAAAGTTATTA
CCTATATATAAGCACCATTAAAAAAATAAAGTGCTTAATTTAAAAAGATAAGAAAACCATCT
CATCATGTTCTCTAGAGTTGGGACTCTGATATTTTCTGGTTCTCAAAGATGACACCTGATACC
AATATCTGAAATGGCGTAAAACAGTCAATACATTTTACCATTATATTTCTTTATTCACTATTT
CCAAAGGAAATTTTGTAGGGTTTTTATGCATTATAGATAATTATGCATTATGTAGGGGTTTAT
GCATTATAGCTGAATGCTTTTATACCAGTGTTTCAGATCATGATTCCTAGTAGAATTGAGTGG
GACCTGCAGATTTGTGCAATTTTGCTAGATAGACTAAAAGTCAATGGCATGAGCTGCCATGA
ATGTGGAAAGCAGAAATTATTAAAGGACTTCAGAAAAAGGTCAAAGTTCTGGCACCACTCA
CAAAGGATAATTTATCACCTGACACTATAAAATATCACTTGATTAATATTGCTGAATATTAT
ATAATAAGCAATTTTAAATAATTTGAATTCACCTATGATATTAACCATGGAACATCAAGACA
GAAAATCAAGATATCTTTTTCAATTAAAAGACTAATGTTCAATCATACCATCATTCAATTAC
ACCATTTCCTTTCCCTATCCTTGAAAAGAATGATCTTCTTGTAGCCAAAAAAATGCTTTCAGAT
AAAATAAACATAAATGATGATTATTTTGACTTATTAAAATGACTATAATTTCAATATTTGATA
GTAAATTTTATACACATATATATTAATTTCATGGTATATGCTTAGACTATTTCTAGATATGAC
AAAATAATTTTTACCTTATTATAGTATCTAAAAGCTCAAAAAAGTGCCAGAGGTATATAGGT
TATTAAAGATAGTAGAACTTGCTTTAATTTAATAATAATTATGAGAACATGAAATAATCTAA

AATAAACTCTAAATTTAAATAGTAGTAAAACATGGATTAAACATGTATTTGCATATTAACTC
CAATAATAAAATCACTTTAAAATGTTTTAAGAAACAGCAGGTCAGCGTAATTTTCTTCCTCC
AGCCCAATTTAATATCAACATAGTATACTTTTCATGCCAAAGAAAAACTCTCCCATTTTAGGT
TCTTATGTAAACACTTGGAGGCTGATTTCCTGAGTTTCAGAAACATATTACATCTAATCAATT
TCACCACCTGTTATTCTGATGTTTAAGTGATGACTTGTTAATAACCTGAGACAAAAATCACTT
TTTGACTGTTAGCACAACTGGCACTGATATTCTAATCCTCTCTTTCTCTTTCTCTCAGCATGTA
TACACATAGGAGAAAATGTTTTGATCATAGGAAGACTGCTTGTAACTGGTTCTAAACAGAAC
AAATTGTGGTAGTGAGTGAAGAAGAAAAAGACTCTTTCAGTATGTTTCTGCTGTTTAGGAAA
GTTTTGATTTCACTGGATGGAGAATAAATATAGAAATGATTAAACAGCAGAAGTGTCACAG
GATTTTACATGAGCTATTGCCACATGACTGGTTCAGACAGGAATGCTATGAATGAAATAATT
AATATAATTATACCATATTCCACCTATATTGACTTTCAGAAATATGAATTTCATATCAGTCGA
CCTCATATTATTTCCAATATTATTATTATCCCTGTTTTACAAATAAGGAAGCTGAGGTACAGG
AAATCAAATAGCCTGCCCAAGTTTAAATGGTTAGCATATGATCAAACTGGGAATCAAACTCA
TAGCATCTATTTCATTGTTAACCACTTCACTCTAGGCTTCCCAGGAGGTTCAAACAGTAAAG
AATCTGCCTGTAATACATGAGACCCAGGATCAGGAAGACCCCCTGATCAGACCCTGATCAG
GATCCTGACCCCTAGATCAGGAAGATCCCCTGGAGAAGGGAATGGCAACCCACTCTTATATT
CTTCCCTGGAGAATTCCATAGACAAAAGATCCTGATGGGCTACAATCCATCCAGTCACAAAG
AGTCAGACATGACTGAGCGATTAACACTTTCACTTTTCACTCTAGGCTTTGAGTAAAGAAGC
CAATATACTTGGGTTGGTAGTAGGGTAAGGAAATCTACTCATAGATTAAAATCTGCTTTATA
TGATTCCATCTTTCAGTACTCTGTAAGAACCTTGAGAAATGGGACAATATTATTTTCTGCGAA
TGTATTCTTTATAGTTCATTCACAGGAACATATTTAGACTAAAGTTGCCCATCATGTGGATGA
ATATTGCTTTCCCATAACAGCAAAAATCAAGTGACCACAGGACTAGATAGAGGGTATTACA
GTCCCAGATATCTGCCTTCAAATTAATGTAGCAAAACAGAACTTTCAAAGTAAATGGCTTTC
AACTCAGCTGAAGACCATGCCACACTAGAGTCATCAAGCTGAAATCCAGGGCTAAATAGTC
TGAATGCTTAAAGTCAGGGATAGATACTGATGTATGTATCAGCCAAGGGAGAGATCTTCAAT
TATATTACTTTTTTTTTTGTACATATAATTTCATCTTTGTATTTCAAATTTACTGAAATGTCCA
CAAAATAGGTCAGACAGTCCAAAGTCAATTTAAAATTCATGGCCTTTTAAAGTCACAAAGAA
CTTAAGCTCCTTTTTAAGAGGACCTTATGATATATTTAGTCTTGATAGTCAAAGAGGTCTATA
CAAATTTCTAGTACCTAGGTAGTCTTAGGATCAGAAGAAATTCATTCCTATTCTGACTGATTC
AATATTTATCTCTGTCTCTGTACAAATTGTGGTTTTATAAAAACCAAAGAAGATTGGTGTGA
AATGCCGGATTCACCATTATCTACTACCAAATTATTGTGCCACATTGAATCAGACATCTGTTT
TTCTAGGACCCACTTCTACTACTAAAAAAAAAAAAAAAAAATGGATGGTGTTAGATGACCCTA
ACTCTAAAGCTAGAAGTTCAGTGCACTCATTTCAATTCTTTGTGTTTCAACTTCCTGATTTTA
AAAAAGTTATCTCCAGATTGACAAGTAAAACAAAGTAACTCATGGTCCTTAGAAGAAAGAC
ACAATATAGTTACAATGTTTTTTAATATAAAATGCACAAGTGTTAGACTCAGGGAGATTTCC
GTCCCACCAAGTACATTATTGAAATCTGTGTCTGGAGAGGTAATGATAAACAATTGATATTA
TACTAGTCCTTAAGAGTCTACCGTGTAAAAGTAATAATGTTCATGTGTATATGTTCATGTGTG
TTCTCCTCTCATGTCTCTTGAAATGGGTAAATGAAAATAGTTTGCAGCTTCCGCGTAGACTTC
AGCTTCAAAACCCTCATCATTTACAGTAAACACCTTTACATTTAGAATAGCCATAATACTTAT
CTATACATAATTTTGTCTTGATTTGGGTTTGCTATGGAATTAAGACTTTTAGGAGTAATAAAA
CAATCTTCAAGGACAATTGCTTGTTAAAAGTACTAACGTCTCACACTGCGTTAACAGTTGCG
ATGTACCCAGTGTAAAAGGATTGTGACAGCACAGGATTTTGTAATGGCATTTAGCTGATGCC
CTGTTCTCCTCTAAGGAATTCTTTTTCTTTCCATTAAGCAACAAGTCAAGTATCCTGAAGACT
GCTGATTCACTTTGACTTCTACCAACTAAGTGCAGAGGTGACAGGAACAAAAATAGTAACAC
GGTGTGAAAGAGGACTAGCATAAGGGTCAGAGATTAGGATCTTCATTGTGATTCTGCCACTA
GGCATTTGGCTCAGTGACCCTTGGGCCAGATACCTGGTTTCTCAGAGTGTCAGCATGCTCTCC
TAGTAAATGTTCACTGTCCTTTTCTGATCCATCATTCTATGAATTGATGCTTCCTGTTGAACTA

AACAAACAGGTGAGATTGTTCTTGTGGGTTTTTTCAGGCTTATTTTCTGTGAAATGCTTTCCT
TCTCTTCCTCTAGCAGTAAAAGGGTAAACTGGAGCTTTCCACCTGAGTATGTATTCCTTCCAA
GGTAGTTAATTGGAAACATTGCCTGCCACAAAGTCACCTTTGTCTCCATTACCTCCATAGTCT
TCTTGTTCAGAGAGTAAAAGCCTTGGAAGGAACCTGGGAGAGCATCTATGATAACTCCCTCA
CTTTACAGATAATAAAATTGACCTCAAAGGGTTTGGTGACTCTCTATTCTGTCTCAGCTCCTA
AACTATTAATAGATACTTATGTATCCATACTAATAATACCTAAACTAGTAGATACTTCTAATG
TTGGATTTTCTGATTTCACAAAAAGAAAAAAAAAAAACTTCTGTATTTCTTCTTTTATTTTTAA
AGTATTAACCTAATAATAATGAATTAGACTACTGTAATCTTGGAAACAGGTACTAATAGGTT
TCTGCAAATGTACTGAGAGGAAAGCCTCATTGATCAGGGTTTTGAATGGGAGAAATCAAAG
CTGTGTTTTTCTTAACAAATGTTAACCGATTTTCACCTGAAGTATCCAAATTCTGAAGAAAAT
GAGAAAACTTCTAATTCCACTAGATTTTTCTATTTTTCCGTTTTGCCTCTTCAGTCTGACTCAT
TGAGGGTACATTAGAAATTAATATTCAACCTGATAAGAACTTTTTGAAATGCTTTAATTAAT
CTTGCTTAATAAATTGTTACAAATTGTTGCAATATTGAAACAGGGTCACTCTAAAGCTTGCC
AATGGTTGGCCTTACAATCACCAGACATGTTTGCAATATTATACACTGATTTTCCTTGTAAAA
GGCTGACTGAAAGAGATTGCTACCATAATGTGTTGAAAACATTTCTGTTGCAAGCATGTGGC
AGGTTGCAAAGGTTTTCATGAGCAGGATACTTGAGCTTGATCTTTCACTGACTAACGAAAAA
GTTTCAACTCCCAACAGCTGCCCGTATTGGCTACAAAAAATAACCCAAACATTTTCTTTTCAT
AGGAAGTTGTCCTTGAAGATGGCACAATTGCTTTTAAAAATTGGGTTAAAACAGGCACAGAT
GTTTACAGACAGTTTTGGATATTTGATGTGCAGAATCCAGACGAAGTGGCAGTTAATAGCAG
CAAAATTAAAGTTAAGCAAAGAGGTCCTTACACTTACAGGTGAGTCCCCAAAATACGTGGC
ACTCTTTCCTTGAACACAGGTATTTCTGAAAAGCTTCCACTTGACAAATGTCACTGTATTGAA
ATGTACTTATTATTTTCTTACCATAAATATATATGGAAATATTTTGTTTCTGCCTACATAAAA
TCCTAATCTAAGGATTTAATTATTATTAAAGAACAGGTTTATTATGAATTTGTCAGCATCAGT
TAACTACCCTACATTTAACAAGTCACATGTGTTTTACACTTGTGGCATATATGTTTCAAAAGC
TCTGAAATACGCATCTAAATAGAAAAAATATATCAAAATTTTTAAAAATGAGTATTTTAAAA
TCTTAAAGTTGAAAAGTGTAGGTACATGAGTAGTTCTCAAAGTATGGCTCCTGGACCAGCAA
CATCAGCATCACCTGGGAACTAAGGCAGAAATGCAAATTCTCAGGCCCCACCCCTAGACCA
AGTGAATCAGAGACCCTTGGTTTGAGCCTGGTAATGAGTTTTATAGCTCAGCTGGTAGACTC
CGCCTGCAATGCACAAGACCTGGGTTCGATCCCTGGGTTGGGAAGATCCCCTGGAGAAGGG
AAAGGCTACCCACTCCAGTATTCTGGCCTGGAGAATTCCATGGACTGTATAGTCTACAGGGT
CGCAGAGAGTCGGACACGACTGAGCGACTTTCACTCACTCTAATGAGTTTTACAAATACTCT
GGGTGATTGCAATACATAGTCAGTTTTGCTAACCACAGCATTAACTTCTATGTAAGTAAAAT
ACCTGCTTAGGATTTCTATTCAAACTGTTTTCCAGGTCTTAAGAGTGTATTTATGCAGTAGCT
TCATTCTTATATTTTCTCCAGTACAAAGACTGAATAAAAAATACCTTTATCTACATGCCGTAA
CAGTTTAAAACATTTTGTATGGAATCTAAGATCCATCAGATGTAGGGAGAAATGATTAGCCA
GAACTTTTCATTTTGTAAATTTAAAAATCAGACTGACACCAGGTGAAACAGTTTGTCCACGA
TGACAAAATCCTGGAAACTGATAACTCCTAGTTATTGGTGTACATTTTCCCCCTGTCAAATTT
ATAGATTGAAGTGCTAAAAACACTAACCTTAGCTAGGTATTAGGACATTTATAGACCCCATA
TGGGAACAGAATTAAGTCCTGTTGTATCATTTGTTATATAAATATGATTTTTCAAGGATAATT
ACAACTAGTTATAGACAATAATCAAGTATTATCACAAAACTTCATGTTTGTAAAGATTTCTA
ATTCATAGTTTTACTTTCGTACTTGTTTTGTGTAGTCTATAATCATGTAATTTCTTTCCTCTCC
ATGCATTAATTTATTTCTATGTCAGCAACTCATGCTCAAAGATAATGAAGTAACAGTAGCTG
GGAGGGTGATGCCTGCTGCTGTAACATAATATATAAGCTGTAGAACACCATGGTACTATTCC
ATCCTAGTAATTATCCCCATTTCCTGTCTTCAGACATATGTCTATGACTATTCACAACTCGGT
CATAGAAGTCTTGGTTTTCATTGTTACAGAAAATAATCTTACTTTCAGAAATATTAAAAAAA
ATTTTTTTTCAGAGTCTTATACTTCTTATATCACTATCAAAAGCCAGATATAATAAGATATCT
GAATATCTTATTCCAAAAAACGTGTACTCTTAAAATCTAGAACTCACGTATGACCTAATATA

ATCTAAGTATAACTGACTAAACTTCTGTCATAGCTCAATTGTTAAAGAATCCGCCTGCAGTG
CCAGAGTCCGCCTGCAGTGCAGGAGACCTGGGTTCGATTCCTGAGTTGGGAAGATCCCCTAA
TGGCAACCCAATCTAGTATTCTTGCCTGGGAAACCCCATGGACAGAGGAGCCTGGTGGGCTA
CAGTCCATGAGGTCTCAAGAGCCAGACATGACTTCGTGACTAAGACACCAAACCATAACTG
ATTAAAAATCATACCCTGAGAGAACTGAATGCATGTGATAAGAGAAAAGAGGTGGTATTTA
AGATCTGTCTCATCACAGGTTTTGCTTAATTTTTTTGCTTGCTTGCTTGGGGGGGTGGGTATG
TTTTTACTTTTAAGGCTGTTCATTGTGGAATGTAAAAAACAAACAATATAGAATCCTGTCCCA
GTCCTGCTCTGATAGGTGATAAGATATATATCTTATTCTTGGAGGCTAAAACCTTCATTCTCT
AAGGGATAAAAATGGAGTGGATTAGATATAATTTGTTTACTTGGAGAAGTAATGAGGTGAG
GGTTGGAGAAGTTGGATTTTCAGCTAAAGTATGTAAACAAACTCGAATTTATATAACAATTC
TAAAGGAATCTCATGATGTTTTATGATGTTGACAAAGGAATCCTTACTGTCTACCTAGATATC
TTAAATAATGGGAAAAACAATGGTTACCTTTCAGAAAAAGTTAATAGTTTGAGAGTCTTCCA
TGTGTTGAGGTGAATTTACTTCCTATCAATTACACCCTGAATTCAGTTGAAATTGGAAGTGG
AAGAGGAGGTCCCTCCTCAACAGAACCACACAGGTTTATTTGCCTTTTAGCTGCTGAAGTGA
GCTTGATGAAACTTTGACAGGGTGTGACATGAGTCAAAACATGAAACTTCCTGGATTGAGAT
GTTAATTATGTATTCCATCTCTATGAAGACCTGGTAACTCCTTCAGAATATCCTCAGATCGAA
CATGAAAGTCAAGTCGCTCAGTCGTGTCCGACTCTCTGCAACCCCGTGGACTGTAGCCCACC
AGGCTCCTCCGTGGGATTCTCCAGGCAAGAATACTGGAGTGGGTTGCCATTTCCTTCTCCAG
GGGAATCTTCCCAACCCAGGGATCGAACCCAGGTCTCCCTCATTGCAGGCAGACGCTTTAAC
CTCTGAGCTACCAAATTTTTTAATTACTTCGCTTTGGCAAGAGTACATGGAAAACAAAATAT
GAAGCAATTACCTCCCTTTGACCTTATTAACCAATACTCTGTAAAACATGTATACATTCTAAT
TTTCAAACAAAAACTTGAGTACTTATAATCTTTTTAAAAGTATATTCTAAACACAGACCTTAA
AAGTATATTCTAAACACAGACCTCAGTTTGAAGGATTTTTTTCTGTTAGTGAGTGCCATCTG
CTGGTATAATGTAAGTACAACTAAGCAAACTACAATTTGAGTCACAATTATGGCTCAGCTGG
TAAAGAAGCCACCTGCAATGCGCGAGAGTCAGACACCAATGAGTGACTGTCAGTTACTTTCA
CTTATTTATATTGCTATTATTTCAAGTATATAAATATAAATGTTCCTGAAAATTAAAGTATTT
TAATTTAAATATGAATATGCAATATAATTTTAACATATAATTATCTGATATAAAAGAGTAAC
TGACTAGTAATAGATTCTCCAGGATGATGTCTCAGATATTTATATTCTTTGTATGTCTACAGC
ATTCCAAGGAGAATAAACAATAAAAAGCTTAATGAAATTGTATTAGAAATAATATATATTTC
CATATTTTTGTGAATTCTCTTTTTAAAGAATGTAAAAAATAACTTCTTCATTCAAAACTATTT
ACCAAGAACTTACCCATGTACCAGACACTGCTTGGGGATTCAAAATACTCATAATACCTATG
GCATAGGAACCTACATTTTTATTATACTTGATTGAAATCAGGTCAGGTTTTTCTGGTTGGTTT
TGTTTTTTCCATTTCAGTCCCATGTTGTTTCAATCTTTATCTTAGATAACAACACATAACTATT
AGAAGCTCATATGATAGAGACATCCGGATATATGATACAGCAGTGAATAATGTACACCTTTT
TCCATCTCAGTAAATATCTTGTATGTATAAAATATTTTTTTCCCATGATATTACCCTTCACTAC
ATTCCTAAAAATTAGTTGGTACTCTGCCTACAGATTAGAGCCAGGAGCTCAGAACTAGTAAC
TATTCCATACATGGGGTAGGGTTGGAGAGGAATTGATCTGTTATGTGGAAATATATTGTTAG
TTATCTTTAACTTTCATTAAAATAATGAAAATTATAGCCAAGCCAGTGATGGAAAGGCTATC
TACTTTGAACAGTCCTTCTAATATAAGAAAATGGGCTCATAATATTCCCCTGCTTCAGCTTCT
GTATTTCCCCCTCACCCTCAAGATGAAAGGTGAACATCTGAATGTAGAATGCAACCCTTTAG
TCTTTGAAATCTTGCTCATTTCAGTGGCTTTGTTTTCTAGCACTCCCCTTACTCAGACACTGTA
AACCAACCACACTGAAACGTGTTATTTCTAAGAGTATACCATGATCTTACTTATCCTATATGT
TCTAACATATTTTACCAAATTAAATTTTTATCTGCCACTCAAACCACTTATATGAAATTGTAT
CCTGTCCATTATCCCCACCATAACCATTCTAAGCCTGAGTTTGTACCTATTCTGTCTTCTCAC
AGAATCTGTATAACCTCCTTAATAACATGTATTCCTTCTCCTCTGGTATGTACATCTAAGACC
TTCCTTCTCTAATTTGCTACAGACTCAGCATCAGCTTGCTTATCCTTTTGGTCTACTTGTTTAG
CATAATATCTGACACAGTCTATGTACTTAAAATTATTTGTTGCATGAATGAATGACATTCATT

TGAAACAACGGTTTTGAGTTTTAGTTCCTCCTGTTTTTCAGAGTTCGTTATCTAGCCAAGGAA
AATATAACCCAGGACCCTGAGACCCACACGGTCTCTTTCCTGCAGCCCAATGGCGCCATCTT
TGAACCCTCGCTATCAGTTGGAACTGAGGATGACATGTTCACCATTCTCAACCTGGCTGTAG
CAGTGAGTAGACAAACCGCAAAATTACTGGTTTTGAAATCTTCTAAAATCCAACTGTAAATA
ACCTCACAATTAAAACTACAATTAGATATCTTTTTTTTCATTTTTATAAAAATGCTTTCTTACG
CTATTATTTTGAATTACGATCATTTGGAAAGATGACAAGATCATAGAATGATCAACTGAAGA
CTGATTAGAAGACCATTTAGAGTGTATCATTTTAAAAAGGATTTATGGGGCTTCCCTCGTGG
CTCAGTGATAAAGAATCCGCCTGATAAAGAATCCACCTGATAATGCAGGAGACATGAGTTC
AATCCCTGGTCCAGGAAGATCCCACATGCCTCAGAGCAGCTAAGCCTGCACACCACAACTAT
TGAGCATGTATGTGTTCTAGAGCTGCGGAGCTGCCACTACTGAGCCCAGATGCCTCAGCTAC
TGAAGTCTGCACACCCCAGAGCCCATGCTCTGCACCAAGAGAAGCCACCACCACGAGAAGC
CCACGCACCACAATCAGAGACTGGCCCTGCTCAAGGCAACTAGAGAGAAGTATGCCCAGCA
GTGAAGACTCAGCACAGCCAAAATAAATCACTCATGAAAAATATAAGCAAAATTTGTCAAA
AAGTAGGACTCAACTTGAAACTCTTCTTCCTGTGGCCCACACACCACAGAGAAATGTAATCA
TTAAGAGATGAAATAATATACTTTTAGTCCTGGCTAGATTCGGCAGTCGTTTTAAAAATAGC
TGGAAATTGTTTTATTTGGTTTAATTCTTGTATTTACATTTATTTGTTGTTATATTGCACTTGG
TGTCTTAAAGCATGATTTTTTTTTTTGCAGGCTGTACCACATCTCTATCCAAATTCATTTATTC
AAGGAATACTCAATTCACTTATCAAAAAGTCCAAATCTTCCATGTTTCAAAACAGAACTTTG
AAAGAACTATTGTGGGGCTATACGGATCCATTCTTGAATTTGGTTCCATATCCTGTTACCACT
ACAGTTGGTGTGTTTTATCCTGTGAGTAACAATTATAAATCTTGATACTGTTAGACTTTAACT
GGATATATATAATCAATCACACTGGCAATTCATAAGTTTATCATTCATTGTGTTAAACCCTAT
TTTGGATCTCGGAATACATACCCTAAGTTTTTAAAAATCTCTACCTCACATGGATTTAGCTTT
CCTAGTTGGGAAAATTTACTTATCTGAATTTAACAATTAAGTTTAAGATTAAGAAGTGGCCA
TTTAATCAAGTGTCCACTCCTAGATAAAGATGCAATTGTCTAAAATTAGGTTATAAGGTGCT
AAGAGTTTGAATACTGTTTTTGGTTTTGTTTCCTGTCTGAAAAACAAGTTTAAGCTGTATTTT
ATTTAGCTGTATTTTATTTTTGTTAATTGTATTCATCATATCATTCTTAAAAAATTGTTTCAGT
GTAAATTTAAAAGTCACCTTACATTGTACATAAGATTGATACATCAGCATGTTGTATTCTCTC
AGATTTAGTTGGTAAGAACTTCTGTGAGTGAGTGCAGTGCTCAGAGCTGATAAGAACTTCTA
TCTACAAAGTCATGAAATGGCAGTTTGGAGATAAAAAGTGAATGTGGTGATGTACTTAGTTA
TGGCAGAATGTTCTGAATTACAAATAGGACAAGGTGACTATTGCATGGAAAATTTTGCTGTG
ATTCCATTTGGCACTATATGAGATATTAATTTTATTTTGCAGTTCTTTGTTATTTTACTTTAGG
TGGTTTTTAGGGTTGCAAGTTCATATATCAATCAAATATGAATACAGAAAGCCTTAAGAGAG
ATAAATTCAGTTGCTATTATTTCATTCACATATTCTTATTTCTTTCCTTGGCTAATTCTGTTTTT
AATTTTGTACACTTCATCTTAGTAACTACTTAGAGATCATAACATTGAGACAATGTCTAGAA
ATTGGTCATTTTTCTCATAGGTTTTGTTCACTCTCCCCTCACTTCAATATACCAGGAGTAGTG
ATCAAAATGGCCTTTCTGGATTGAATAAAACAAAGAGTGCTAGAATTCGATCATGACAGCTT
TGGCATTTGTTACCTTACTTTGTCTTGGCATCTAGCACTCATTTTTAGTAACCTTTTGTATTCT
GTCTTTTTAAAATATTAAAGACCAATAATTTTGAAATGATTGCATGTATATTTAAAATCATAT
AAAATAATTATGAACAACCACTGAAAATGTGAGATAATTACATATTTAATAACTTGGAAATT
ATGGTTTGTTGTAAAAGTAATTTTTTCTTGCATCTCTTCAGTACAATAATACTGCGGATGGAA
TTTACAAAGTTTTCAATGGAAAGGACGACATAAGCAAAGTCGCCATAATTGACACATACAA
AGGCAGAAAGTAAGTATCCAGGCAAAGTGTGTGTCACTAGGATACTCTAAGGCAGGCAAGA
AACTTATTTACCGGCGGGTAAGGCACAGGCACAACCTGTGGGACGGAGCTGTTTATATCTCA
GCCCTAAAAACTCCCTAGGTCTACCAAGCATTAATCCTTCAGTTGTGATGACTGGTCACACT
AGTGCATTTTTGCTGAGTATCAGCCCCACTGGAGGGTTCACCTTTATGAAAAAGTTCTCTTAC
ATAAAGAAATCTAAAGGGACTACTTCTATCTCTGCTGTATAACTGGTATTATCATACACACA
ACAATGACATAAATGCAAATGAAAATGAGGAAAATCAATATATCAACTCATCATCTAGCAC

GTAGAAATACAGACATCTTACTGTTTCTTTTTTAATTGAGCATGTCAAAAAACATCATTCATT
CCAGGGAGATGATTAAATGCTGGGGTCCACCACCCCCACCCCCCCGCAGGATCCAGGGGAA
CCTGAAGGAAAAATGGCGTCAGTGATTGATTTAGAGAGAGATAAGGAAAGAATGTTGAAGA
TAAGAAAATAGAGGAGAGAAAGAGGCTGATATTCCTAGGTTTACATAGAAAGCCAATAAAA
CTCCCAGACAAGAAGTTTGTCCTGTTCACTGAGGCTACAGGTGCCCTCCTGGTCTCCTGAGG
GAGTGAAGACGCAGAACGTCTTCCCGTTCAGGTCTTAGAAACCCGAGCAGATAAATGAATG
CAGGGAGCCTCTATGCTCCAAGGGATCAGCCTGAAAAAGAGAGGGAGGGAGAGGGAGAGA
ATGATTGACGCGGGGAGACCAAGCTGCTTCAGTGAGCGAGGCCCAATAGCTTTATTTTTAAA
AGGTACTTTTATACCTTGTCTTATACATAGAGGGAAATGAAAGATGCAAAGTCATACAGAGT
CAGCCCAAACATTACATCTGTTTTGTCTTTATCTAAACCAGGATTTTTTCTGCAAACCTTTCC
CATAAACAATATTGTGTACATTATCTTCTGGCCTTGGAGGCCTGTGGACATTTTGTGACCCTC
TTTTGATAAAGGCTGCTCAACCAGAAAACTTATTTTCCCTTGAAATGTTTTTTCTTTATATTTC
TAATCTATGTCAGCCTCAGAAAGTATTAAACAAGTTACATTTCTCATGGAGCAAAGTGCAGT
GAGTTACAAGAAAGAACAAATTAGCTCAAATGTCTGATGTGGTTAATTTCAAGGCTACACTT
GTTTTTCTTACTTTCCAACTATGTTAACTAATGCACCCCCAGGTGCACAATGGATAAGAGATA
TGGGAACTTAGCAACAAGTATTGGCCCAATAATGAAATCCTACACCAGCACTACTCTAGTAA
CTTTTAACTCTTTAAAAGGCTCTATGTTTTAGGCTTTCTGTGCCTCTCACCGTTGGGAGGCTG
TAAATAATCATATGTATAGCTGCAAGAGTCTGGATATACCTGTCAAGCAAGCTAGAATGCTA
ACAGAGGGGGTTTGATTTGAAATATTCCTATCATGTCCAAGAGACTTATTAGCTATAGCCCT
AAGTTGATTTTCTCTAGAGAAAGGTGGTCAGGGATAGCCCCCTGTTAATGTCAGAGGAGTTG
GTGAAAGTCATGAAATATTAAAACAGACAGATTGTAGTTTTGGGGTAGATGCTCGAGAAAG
CTTAGGGAGCCTGTTGAGTCCTGAAGCCTTGCTTAACAGTTCTCTTCCACATGACCTTGTCAT
GGGTGGGATCTCCCATGGGTGGCTCCCGGCAATTAAATGAACCTATAAAGATGAATGTTCAA
TGAATCCATGTGAACATTTTCATGTCTTGTCCCTTAAAAATTTTCCTGAAATAATTTTCCATA
TTAATAATTCAGCTTTTTAAACCATTATCAATGCAAAAATGGAATTATTATTTCCTGTTCCAA
AAATGACATATTAAGCCTGAAAATAAAGTGTACACCTTCTATGAAGTAGCAGTAGAGTGAG
ATATGTTCACATACCCATGATTTCCTACTTCTCCGAAGTTCCTGCCTGTTTATGTGCAGTACA
CCTAATCTGTCATATATCTAAAATACAAAGAAGCCCAATAAATATCAGGTGGGGGGAAACTT
TTCTCCTCAAACTTGAAATTAGAGTTTTAAGATCAGTAGTTCCTGACAAAGAATAAAGTGAT
CAAAATCAAAAGAGATGAAATATTTTTATGATTAAGTAACCATTACTTCACACTGAGTTTGT
CCCTAAAAATTGCCTAAAACAGTAAAATACTCGCTCACGTAGACTTTTCATCTACAGAGTTC
ATAGTCCTTTATTGTTTTGAAAATTCTAATGGAGGAAAACAAACACTTCTAAATGTTCATTGT
AGGGAAATCATATCTCTGTATATTGAGAAAAGTGATAATTTCTTCCATTTCAATATTACCAG
AGTTTTTGAGGAGTTACTAAATGTTTCAAATTGGAGTAAGAATTTCATTTAAGATTATGATA
AGGTTAACTTAACTGTCTGAGCATGACTGACAGCTTGTTCTGTTTAAAAAAGAAGTAATAGC
TTGCCTTGACATCTTACTGGATGGTATGTTAGACTAACAAACCCATTTTTCCATCACCAAAAA
GTGGTTGAGTATACAGAGAAACTATCACTACTGTTGTTTTCATTATTTCCATATATAAATATA
CACACACATATACATTTTATGTAGATACCAGAATATAAATTAATAAACTTCATCCTACTTTAA
TGCCAGCTCATGCAAGAGTTGAGAGGAAAAGTGTATATTGATTTTCTACATGTTTCTAAATC
TCTATTAATCAAACATCTCTTTCCTCTTCCACATTCTACTCTTTTCTCTCATAATGAGTTTATA
TCAGTTAACCATTTAGTATCTTCCTCCTACTCAGCTGGACACTTCAACATAATATGAAATACT
CCACTTCTCCCATTGGAACAACAACAAAAAAACCTTTCTTGAAGTAGACATCTAAATGTCTG
ATATGTGTTTTTCAATTTTCTTTTTGCAGAATTTTATCTTTTCCAAGTTGTTCCTAACTACCAA
ATTTTGGCAGCTTTTATTTTATAATCCAGCTACTTAGTGTTATCAAGCACTTCCTTTATCTCAA
CAAGATGCCAGCTTCAAACAATACATAAACCAAATGACTTAGAACATTTCTTATACTTAGCA
CCTGTCACATCTACTCTAATCAATTATTATTTCTGCAGAAATACTGACTCATAATAGACAATG
TAATGTGAGAAGGTGCTCCTGAAATGAGACTGATCATTTTATTTTTCTTTTTTTAATTCCTAA

GGAATCTCTCCTATTGGTCAAGTTATTGTGACCTGATTAATGGTACAGGTAAGGACAATCTG
TTTTGCGGTAATATCACAGTGAAACCACCTTCTTTCTCCCACAAATCCTCCATCATATCTACA
ATGTTCTGGAGGCTGAGGATATGCATTTACTTATCTTTGTATCCCTACAACATAATTCAGAGT
CTCTATACTTATGCTGCTGCTGCTGCTAAGTCGCTTCAATCGCGTCCGACTCTGTGCGACCCC
ATAGATGGCAGCCCACCAGGCTCCCCCATCCCTGGGATTCTCCAGGCAAGAACACTGCAGTG
GGTTGCCATTTCCTTCTCCGATGCATGAAAGTGAAAAGTGAAAGTGAAGTCGCTCAGTCGTG
TCCAACTCTTAGAGACCCCAGGGACTGCAGCCCACCAGGCTCCTCCATCCATGGGATTCTCC
AGGCAAGAGCACCGGAGTGGAGTGCCACCACCCTCTCCACTATACTTATATATAGGCTTATT
AAATATAAGTCTAATGTGATGGTATTTCTGAAGATTAACCATTGAAGATAAGCCTTTACTAC
ATTCCTTGGGGAATGTTTGCTTCATAAAAGCTTTACTCAAGTGAACTCCCTTCCTGGCCACAG
AAGTAAGGGGGTAGAATATAAGTAGTGGTCACTGTTTGCATGTTTTGGGAATACTATATAAG
CTGTGACCAACCGAGACAGCATATTAAAAGCAGAGACATGACTTTGCTGACAAAGGTCCAT
CTAGTCAAACCTATGGTTTTTCCATTAGTCATGTATGAGTGTGAGAGTTGGACCATAAAGAA
AGCTGAGCGCAAAACTGATAATTTTGAACTGTGGTGTCAGAGTAGACTCTTTTTGAGGGTCC
CATGGACTGCAAGGAGATCAAACCAGTCAATCCTAAAGGAAATCAGTCCCGAATATTCATT
GGAAGGACTGATGCTGAAGATGAAACTCCAATACTTTGACCACCTGATGCAAAGAATTGCCT
CCTTAGAAAAAATCCTGATGCTGGGAAAGTTTGAAGGGAGGAGGAGAAGCGGACAGCTGAG
GATGAGATGGTTGGATGGCATCACCAACTCGATGGACACAAGTCTGAGCAAGCTCTGGGAG
TTGGTGATGGACAGGGAAGTCTGGCATGCTGCAGTCCATGGGATCACAAAGAGTCGGACAA
AACTGAACTGAATGGTTTAAAACTTAAAAGAATTAAAAAATCAAGATAAATACAAAAGTAA
ACCTTGTGTTCAAGTGGAGGTTTCAATATGAGGCAGAATCAGAGAAGTTCAAGGCTGAACA
CAGATTTTGAATGAAATTATTAAAATGAGAAAGAAAATAACAACAATAAAACACAGTTGCA
TCTGTACTAACCATGAGAAGTATATGCAAAAATAACATTGTGAATTAGAGCTGGGAAGAAA
GAAGGTACTAGGACTTTAGATTCAGAGAGTGATAGATCCAGTTTATTATTCTCCTTAAAGAA
AAGCAAAGTACACATATGCCTATTAGTTTAAGTATTAAAATTTTTAAAAGTAATAGGAATAA
AATATTTAAAATCAAAGGTGATCTTTCAGCTACTAAGAAGCAGAAGCAGAATATTATGTGTG
TAAAGTTGATAAGGCAACACTTTAGAAGAATGTAACACAATGAAGAAAAAAATGTTATACA
TAAAGTGCGTAAAAGAAAATAAGGAAACATAGAGAACGTTCATTTTCACAAAGGTATTGCT
GAGTTTCACAGGTTTAATTTTGTTTACTCTGTGTTAAAAAAAAAACACACACACAAACAGTA
AAACCACCGCTGAATTTGCTTTGCAACACTTTGAGTCATGGTGATGGGTGAAGAAATCCTTT
CCAAGTAGTTCATTTCTGCAAATAGTCAACTGTCCAAATACCACTAACTTTAATTTCATAAGC
ATACGCTTCAGTCCTTAAAATAGAAATATACTGTGCTTCTCCTAAATAGTAAACTATTGTTTA
TGGGCTTAAAATACATTTAAATCTTAGTCTAAAACAAAAAATAGAGCAGTTTTAAAACAGTA
ATAAGAGGATCCATTAATGACAGTAACAGATACATAGAATCAGGCATGAAATTCAAAGCAT
CTAAGAAGAAAATCACAGTAAAACAAACTAGGAGCAAAGGTTGATTGGATAGACTAGGGAC
TCTGATGGGAGATTCTCTTGGTCATAGTACTAAAAAAAGAGACTGCTATTGCTGCTGCTGCT
GCTGCTGCTGCTGCTGCTGCTGCTGCTGCTGCTGCTGCTGCTAAGTCGCTTCAGTCGTGTCCA
ACTCTGTGTGACCCCATAGACAGCAGCCCACCAGGCTCCCCCGTCCCTGGGATTCTCCAGGC
TAGAACACTGGAGTGGGTTGCCATTTCCTTCTCCAATGCATGAAATTGAAAAGTGAAAGTGT
TTGTTAGCCATCCGTATGTCTTCTTTGGAGAAATGTCTATTTAGTTCTTTGGCCCATTTTTTGA
TTGGGTCGTTTATTTTTCTGGAGTTGAGCTGCAGAAGTTGCTTGTATATTTTTGAGATTAGTT
GTTTGTCAGTTGCTTCATTTGCTATTATTTTCTCCCATTCAGAAGGCTGTCTTTTCACCTTGCT
TATATTTTCCTTTGTTGTGCAGAAGCTTTTAATTTTAATATATATGGAATTTAGGAAGATGGC
AATGACGACCCTGTATGCAAGACAGGGAAAGAGACACAGATGTGTATAACGGACTTTTTGG
ACTCAGAGGGAGAGGGAGAGGGTGGGATGATTTGGGAGAATGACATTCTAACATGTATACT
ATCATGTGAATTGAATCGCCAGTCTATGTCTGACGCAGGATGCAGCATGCTTGGGGCTGGTG
CATGGGGATGACCCAGAAAGATGTTATGGGGAGGGAGGTGGAGGGGGTTCATGTTTGGGAA

TGCATGAAAAAAAATAAATAAATAAATAAATAAAATAAATAAAATCTAAAAAAATAAAAAA
AAAAAAAAAAAAAAAGAAAAGTGAAAGTGAAGTCGCTCAGTTGTGTCCGACTCTTAGCGACC
CCATGGACTGCAGCCTACCAGGCTCCTCTGTCCATGGGATTTTCCAGGCAAGAGTACTGGAG
TGGGGTGCCACTGCCTTCTCCGAGAAAAAGAGACTAATGAAATGTAAATGGATCCAAGGTG
ATTTTTAAATCATGATTTAGAAGTACTTCCTCTACCATCTTCATTAAGTTTAAAGACATAGAA
TGAAAATATTTATTTGAATTTATTTTAAAAAGGGAGCCCTGCTGGCACCAGCTATGTTACAG
TCATGGCCCTTGGTAGTCACCGGTGTCATTCCGACATAATCTTCGATGATATTTCTGATGGTT
TTTAAAGACTGTCAATGTACTCTTTTCCTTTGATGGCTAAGGTGTTGGGACACCAGTTGTCGG
CAAGGCCCATTCGGGGACCTATTTGGGGAATTTAATCTTCACTATCCCATTCTCTCAATGTCT
CTATCAGGTTCACGGAGATCCTCTGTTATATTTCACCTATCACAAGAATGTATGTGAAATGTA
ATAAAATCACAAGGTCAGAGCAGTTACCAGAAAAAGAGTGTGGTATTCAGAACTGAAGGAT
AGGTCTTACTGTTTTGAACAAACATGTTCAGTGGTGGCTCATGTTTGGTGATATTCATTTGTG
GAGCAAATGGAGGAATTATCTAGCTATTTTTTCTTACATCCAGTGTGTACTATGGAGCACAA
CCATAGTAAAGATTTGCTAAGCTCAAAAAATGCCCTTTTTAGTTTGATGTGTATACTTTCCAA
CCATTTGTGTGATATTGTTTAGCAATTTGAAAAATATTGATCAGCCATAAGGATAGGTGAAT
GGATCACTATTAAAAGTACGAATAACATTTACAAAAATGAGTTATAAAGAAATCTCAAGATT
AAAAAATTGCTACTCTACTGATAGATGGAAGATTTAAAAACATAAAATCGCAGTTTTAACAA
CGTGATTTTGACATTTTAAAAAACTTTTATGGAGTGGAAAATTTAATATGAAACAGAAAGC
CAATGACTACATATAGTATACGGACTTATTAGATATGACAGCTGCTAGCCACATGTCACAAT
TCAGCAGCGCTTGAAATGCAGCTAGTACAAACTGAAATACTGTAATTATAAACACTGTATTT
TCAGATTTTTGTTAATTTTTATATGTTAAAATGATGGTATTTTTGATATATGGGGTTAAACAA
CATATACTTGATACATTTTTTTTTCTCATCTTTAAAATGTAGTTACTGGAGAATTTTAAATATA
CATTTTGCATTACATTTCTCTTAAGCATGCTGGAATCAAGATTTCTAGATTTGTCTAAGACAC
ACTCTTTCCATCTAATCCCTTGCCACTGATTTGGTTTTTAAACAGATGCAGCCTCATTTCCAC
CTTTTGTTGAGAAGACAAGGAAATTGCAATTTTTCTCCTCTGATATTTGCAGGTAAGAGAAA
GACAATGATGTATTTCTCCAAGTCAGAATTCTTTCTCTAAGTGATAAAACACAGACCAAGAA
ACTCATGCTTATTAATCATGTTATTTGAGAAAAATAAAATACTTTTAGGGGCTTTTTCCTGAT
TAAGAATAAATACAATGTGAAATTCATGATGTATTTATGCCCTGGAGAAGGTAATGGCAAAC
CACTCCAGTATTCTTGCCTAGAGAATACCATGGACAGAGGACCATGGTGGGCTACAGTCCAT
GGGGTTGCAAAGAGTTGGACACAACTGAGCCACCAATACACACACACACACACACACACAC
ACACACACAATGTATTTATGAGTCTTTAACATTTGATGTAGAAGGATGTTGTGTATAATGCA
AATCAGTTTCTTTTTGTTATTTTTAAGAAAGAACTACTTTTTTCATTCTTTTACTAAATTAGCA
GTAAGAGTGATAACAGTCAAGTCTAGAAAAGCATGCCCAGTCTTTGTAAAGAAGAGGATAC
CACAATCACAAAACTATGTTCATGTGCCCCACTGACAATACAGCATCTTTTCAAATTGACGG
GGGCCCAGTAGGTATCACACAAGTGTTGGCGCCTACACCTGTATCCTTGAACGCTCACCTGC
TTTCTTAACACTGTTTGGGAAAGCAAGCATAATATCTCTGAATCTTTTTCCTTATCTGTATAA
TTGAAAAGAACAGCATTTCTTCAAATAGTCATCTGGGGACTAGAAAATATTTAATCTATAAA
GGAAACTAACTGTGAGGAATGCCTTAATTGTAAATACTCCACAGTAATTCACAAGCAAAAAT
TATTCTCAGAAGCGTTTGAAAACAAAGATAAGAATAGCTCATACTCAAGTATTAAGGTCTAG
TGTGTCTTAAAGTTTTGCAAGTTTTTTTTTCCTAAAAGGTAAGGATTCTAATAAGATTATATC
TAATTTTTCTAATTTAAGGCAGCAATAGTATAAATTTAAATATAAGAGAAAACAAATCTCCA
CAACAAAGTTCAGGTTCCCAGAATGAAGCTCCTTCTTATCTGTGTTCAGGTCTATCTATGCTG
TGTTCGGAGCTGAACTGAATCTGAAAGGAATCCCTGTGTACAGATTTGTTCTTCCATCCTTGG
CTTTTGCATCTCCACTTCAAAATCCAGACAACCACTGTTTCTGCACAGAAAAAATCATCTCA
AAAAATTGTACCTTATATGGTGTGCTAGACATTAGCAAATGCAAAGAAGGTGAGTACAAGTT
CTCAGTGTCACAATCCTTGGCATAATTTGCATTATCCTTTAAAATGTAAACTTCACCCAGGAT
TCCTTTCCACCAGAAATATAAAGTGGGAAAGCAATAAGGGCAGAAAGTAAGAGTGATGGTA

GGATATTTTTGTTTTGATGTTTGTCTTGCTCTCAAGGTCTCAGTTGTTTTCATGTTGCAAAACT
TAGTTTTTATTAGGTATAATCATGCTTATTGATACTTCCTGTGCTACTAGGAAAATTTCTCTTT
AGGTCTTTTGCTCATTTTCAAATTGGTTTATTATTTTTTTTGAGTTGTATAAATTCTTGTATAT
TTTGGATGTTAATCACTTTTGGGATATGTGTTTTGGAAGTATTTTCTCCCACCCACAGGTAAC
ATTTTCATTTCTGACCATTTCTTTTGCTGTGTAGAATGGCTGTGCTCCTTTTAGATGTAGGGTA
AAATGAAAATAGTTTGCAGACTACTTTGAGCATCCCAACTTGTCCATCTGACATTTTTCTTCT
TCTACCCTAGAATCTGTAAATAAGTATATTTTAGGATGTCTCTGAAACAAAAATTGATTCAA
ACATGGCATCTGGAGTCTATCTTATTTTGCTTACCTTCTTCATGTTAGAGTGGGTGAGAACAA
AGAAAGAAAAGCCTCTTTAAATGAATTCTCGGTCCAAAGACATTTTACAGCCTGAAACATGA
AGGCATATGGTTGTAACTTTGTGTCCAGTAGCTAAGCTTTACATACTGAAAACACCTAAGGT
GAAAATAAACACCTAAGGGATGAACAACAGACATTAACTAAATAGTCAGTAGGATGACGTA
GAAGTTTACATTTTATTTTTGTTCAAAAGTGATCAGATACAATAATTTTCCCTCACTCTCATC
CCCTGATAAGATTTCAAAGCCAAATTTGTGTTTCTTTTTTTAAATTAAAAAGCTTTGTTTTCA
AGGAAATCTTTCCTAAGTGGTGTATGAAATGAGGAAAATGATTTACCCTAACTGTCTCTTAA
AACTTGCTTTCAGGAAAACCTGTGTACATTTCACTTCCTCATTTTCTACATGGAAGTCCTGAA
CTTGCAGAACCTATTGAAGGCTTAAGTCCAAATGAAGAAGAACATAGCACGTACCTAGATG
TTGAACCTGTAAGCAAATACTTATACTTTATTGATCAGATTTGTTTTTGTTTTGTTTTGTTTTG
TTTTGTTTTGTTTTAGTTTTTTATTTTTTAAATTTTAAAATCTTTAATTCTTACATGCGTTCCCA
AACATGACCCCCCCTCCCACCTCCCTCCCCATAACATCTCTCTGGGTCATCCCCATGCACCAG
CCCCAAGCATGCTGTATCCTGCGTCAGACATAGACTGGCGATTCAATTCTTACATGATAGTA
TACATGTTAGAATGCCATTCTCCCAAATCATCCCACCCTCTCCCTCTCCCTCTGAGTCCAAAA
GTCCGTTATACACATCTGTGTCTTTTTTCCTGTCTTGTGTACAGGGTCGTCATTGCCATCTTCC
TAAATTCCATATATATGTGTTAGTATACTGTATTGGTGTTTTTCTTTCTGGCTTACTTCACTCT
GCATAATCGGCTCCAGTTTCATCCATCTCATCAGAACTGATTCAAATGAATTCTTTTTAACGG
CTGAGTAATACTCCATTGTGTATATGTACCACAGCTTTCTTATCCATTCATCTGCTGATGGAC
ATCTAGGTTGTTTCCATGTCCTGGCTATTATAAACAGTGCTGCGATGAACATTGGGGTACAC
GTGTCTCTTTCCATTCTGGTTTCCTTGGTGTGTATGCCCAGCAGTGGGATCGCTGGGTCATAA
GGTAGTTCTATTTGCAATTTTTTAAGGAATCTCCACACTGTTCTCCATAGTGGCTGTACTAGT
TTGCATTCCCACTAACAGTGTAGGAGGGTTCCCTTTTCTCCACACCCTCTCCAGCATTTATTG
CTTGCAGATTTTTGGATTGCAGACATTCTGACTGGTGGGAAGTGGTACCTCATTGTGGTTTTG
ATTTGCATTTCTCTAATAATGAGTGATGTTAAAAATGAAGAAAAAATTAAATTATCTCCTTTC
TATCAATCACATTTAGTCACATTTATTGTACTACATTTGTAATAAATTACTTATTTTTACTTCG
AGGAAAATTACAGTGATTTCTTCCTGTTGTTGCTTATGAAATGTCAAGACAATTTTAGTTTTT
CAGCAAAGGTCAGGAATAATGAAAATCATTCTATTTGGATTAAAATGATTTTACATGTTACT
CTTTCAAATGTACTTTATACTTAACTGCAAATATTGCTTATATGAAGGGACCCTGTAGATTAT
GAGGGATTAAAATTTTTTATGGGTATACATACATTTGAGGTAAGATCAAATATATTTCTTAGT
GTCAAGAAAAATCTATTAAGTCACTATAGTTTAACAGTTTCAATTTTTCATTTATATTAATAT
TCCCCTTTTCAACAGATAAATGAAATATCAGGACTTTAGTAGTCATATTTTAGTTTAAATGAT
GACAGAAGTAACATTATAATTATACTGATGATGCATGGATTCTGACTGAGTACATATTTAAA
TTCCAGGATATATTTTGTCAAATGAGTTTTAGATTTCACTACTGTTGATGTCGTTACTAATTC
CCCAAAATCAATTCAAGTAAATGTCACAAATCTAACTAAACCTTGACATTAGGTTGGGCAAC
GATATATCTGCATACACACATGCATATACTGATGAAATATAATTATGGAAAGATACATGTGT
AATATTTAATATGTCTGATTAAGTCCAAATAATAAATAACAAATTTCACAGGTTCAAGTAGT
ACACTTTCACCTTTTAGGCTCTACATAACAAAAATAAGGTCTGGATATTATAAAAGAATGTT
TTTCAAATAAGTTGGTTATTAGTTTGTTCTCTTTTTAGATAACTGGATTTACTTTACGATTTGC
AAAACGGCTGCAGATCAACATACTGGTCAAGCCAGCAAGAAAAATTGAGTGAGTTTCTTAA
ACAAGTTTCATTTCGATTTTTCAAATTTTCTTGTAAGATGCCAGGTATATCTATCAAAGGAGA

TGGAAATAAAATTTCAACTTCTGTTCGGCTTAATGTCATTAAATAATTATTAAATACTTATAA
CTGTCATTTGCAATTTAGAATTACATCTAATGTGTAGTGAAATGGTACTTACCCATATTTTGG
AATGAGTATTTCTGGTAATTTCTCTCTTTGCTAACTGTCCAAATATTTTTATGACAATAGTTTC
AGCATTCATATTTTAGTTTCCTGGGTATAATTTGTTGAAAGGGAAAATACATACATAGAAAA
ATTCTAATGAAAAAACCTGTGAGTCCACCTTTCACTGCACGGGACACTGGTTTGATACCTGG
TACGGGAAGATCCCACATGCCTCTGGGAAACTAACCCCGGGCACCACAACTGAGCCTGTGCT
CTGGAGCTCTCGAGTCGCAACTACTGAGCCCAAATGCAGCAGCTACTGAAGCCTGCGCTCCT
GGAGCCCCTGCTCCACAACAAGAGAAGCCTCTCCAATGAGAAGCCCATGCCCAGCAACTAG
AGTAGCCGCTGCTTGCCGCAACTAGAGAAGGCCCATGTACGACAACGAAGACCCAGTGCAG
CCAAAAATAAATAAATGTTTTAAGAAATCTGTATTAAACAGATGTCTATGTAGAAAAGATAT
CTGAGAAACAGCAATATTAAAATTTAAATGCTTCATTACAGGGAAAACAAAGCAAGTGACT
TACCCAGGTTGTAAAAGCAACTAATTTCTGAACATTTATTTTCACTAGTAATTACTAATATCT
GTCATATGTGTATAGCATATTAGTATATATTTTCATACTTATTTTCAATGTTTATTACAGTGCA
TTAAAGAATCTGAAGCACAACTATATTGTCCCTATTCTTTGGCTTAATGAGGTTAGTATTTTT
ATCTATTAGTCACTAAAAACAGTAAGCTTCTTAATATACAGAAAAAGTTTTTGATGTTTGAA
AATTAAAATATATATACACACACACAGTATTTGAACTTGTCTTTCCATTGTATCTATGGGTGA
ATTTATATATTATTAATTATATAATTAATTATTTTTTAAAGCAAGTCATACAGAAGTATCAGG
ATTATGTTAGACTTAAGTCTAACTTTTATGAAAATAAAAATACTTCATAGTGCATTTATTAGA
TAATTTTTCTTGTCATAGTGCATGTGTTAGGAAAGAGTCATGAAAATATTAAATTCCAAATA
ACTCAATATGAGCCTTGTAGTGCTAATAAAACCACCCAAGCTTATAATGGGCAAATTAGAGT
AGATGTAGGCAGTCTGTCTAGTGCCTGACAAGATATTTTTAATATAAATCATGGAACCGACA
AACCACAGAATTAGGAAATGCAGTGCATAAGCCTTTAGAGGATAGAAACAGTTGAGCTTTG
CCATTTCCCTCTGTCACTAAGAAGCAACCTTTGGGAAACTGGAGTCTTACTGAAAGACTCCA
GTAAGACTGCTATACTGATCAATGAAATCAATATATACAATAGCACTAGATAACATGTTTAA
AGGGAGGAATATTATCTTTTTCTCTCTAAATATTCTACCTGGGCATTTACTGTCTTCTTTGAC
ATGCAAAGAGAATTCCAACAGGCTTGTCTTGCAGATAAAGATGACTGACACCTGTGTGCTGT
TTTGATAAAGTGATACACATAAGTGGAGAGTTTTGAGTATTCTGCTCTCAAATCTATTCAAAT
AACACTATTTATTAACTTGATTACAGACTGGTACCATTGGTGATGAGAAGGCGGAAATGTTC
AGAAAGCAAGTGACGGGGAAAATAAACCTCCTTGGCCTGGTGGAAATGGTCTTGCTCAGTG
TTGGTGTGGTGATGTTTATTGCTTTTATGATTTCATATTGTGCATGCAGATCAAAGAGAGTAA
ATTAAGTAAGTATATAAGAAACCATATGTGACTTCAGTAACATTAAATATTACCTGTTTTCAT
TTTATCAAAACAGTTATAAACTAGGCTTCAAATATTAATGAACATGTCTAGTCAGCTATCATT
TTTAATACATCCTTGAAACTTTGAGACACTTAATATAAGTTAAAATGAAGACTGAATGGGTG
CTTAGGCTTATCAGTATTAAACAGATCACTATATTTCATCTTTATTCTGGGAGGAAATAGGCT
AAGCTGTGAAGGTGTCACCTTCAAAAAGTTAGAATGCTGCACAAAAGCTATACATGTACTCA
ATTTTTATTTCTTAATTATTTCTAGATAGTTATTTTTAAAATCACAAATAATTTATCTGACTAG
GAAACATGTGAAACTGAAAATGGCAGCTATTTGGGGAGACAAATTTATAAAGGAAAAGAAA
ATAAAAACTAAAATTAGATAAGCTATAAAATGATGAGAATTAAAGAGTCAATAAATGGTAG
AGATAAAATCAGATAAAAATATGCTATTTTTAAATGCAAATAGTGTATTAAATGGGCTTCCC
TGGTGGCTCAGAGGTTAAAGCATCTGCCTGGAATGTGGGAGACCGAGGTTCGATCCCTGGGT
AGGGAAGATCCCCTGGAGAAAGAAATGGCAACCCACTCTAGTATTCTTGCCTGGAGAATCC
CGTGGAAGGAGGAGCCTGGTAGGCTACAGTCCACGGGATCACAAAGAGTCAGACACGACTG
AGCGACTTCACTCACTTAAGTGTATTAATATAAAAACAGAGCTGCTGCATATGGACATGCTG
TCCAAAATCATGACATGTACCCTAGATGTTCCATGTGAAAGCCCTGATGAATGATTATAATT
CAGGAGCTAGAATTAAATGAGTACAAGAAAAATAGAATAAAGATAATGTTGAACTCACTAA
AACCAAATAATTAGAGTGTAAAGGAAAAATCAGATAAATATGGACGGATGAGTTTTAGCAA
AACTGAAGAGGTGGTTCAGGCCTCTGTGTTTTAAATATTAGTACCCGTGTCCCCTTGTACATA

AAAGAGTGTATGCCAGACCCTATTTACTCTAAATGGAAGCCTTTCTTTAATAATCATAGGTG
GCATTGGTATGGCCTGAGGGGAGTGAATTCTCCACTAACTCATAGGTTCTATCTGCCGTTCCT
TCCCTGGGATATGAAAGAAAAAAAATGAAGTGAAAATCAATTGGTAGCTTCCTACTATTTTC
TACCTCATAAACTGTAGCTCACCAGGCTCCTCTGTCTATGGAATTCTCCAGGCAAGAATACT
GGAATGGGCTGCCATTCCCTACTCCAAAGGACCTTCCTGGCCCAGGGATGGAACCCGGGTCT
CCTGCATTGCAGGCAGATTCTTTACCATCTAAGCCACCAGGGAAAACCAGAAGAAGAAAAA
ATATATATATATATGTATATATGAAATCTAAATTAAAGCAAGGACTGATGCTGAAGCTCCTA
TACTTTGGGCCACCTGATGTGAAGAGCTGACTCACTGGAAAAGACTGAGGGAATGAAGAAA
AGAGGGTGATAGAGGATGAGATGGCTGGATGGCATCACTGATTCAATAGACATGAGTTTGA
GCAAACTCAGGGAGATAGTGAAACACTGGGTAGCCTGGCATGCTCCAGTTCATAGGATTGC
AAAGAGTTGGACATAATTTATCAAGTGAACAACGACAGCAGCAAATTGCTCTGAAGAAAAC
AATTCACAATTTTAAAACAAGTCTTCTTATTCCACTTTCAATCAGACTGCTGACAGTAAAATG
TCCTGCATAGAAATTCTGGAGCTGCCACTGGCAATTATTGACCTTAATGTGCTCAAATCATG
CTATTTTACATGAATTTCACAGCTAATCCCTAATAAAAATTTATGATTTTCCAGTAGCCATGG
ATCTATCAGCATCCATTCTGTTTTTTGAAGACATTACAGAAAACTAAATTTTAACAGTATAAA
ACTGTCTATTTTCAAAGTGTGAATCAGTTAAATTGAAGATTTAAAAAACAAATTGGCCTTTTT
GGACCAATTTGTGTGTGTGTGTTCGTGAAATTGAAAACAGACTGAGGAAGGCAACTCGATCT
TTTGTTTTGATGCTTTCTGCCTATATATATATTTCCTTAAAAGGAGACTGAACAGTTCTGCCTAC
AGTCTACTTGGACACAATATATTCAAAATGCTAATACATTCATGTTATAATAAAGGAAATAT
GGCATCACCTTTAAAACCTGTCATCAATCCAGTAACCAGTTATGTTTAAACACAGTAATTCA
TATGACCATTTATATAACAGACCAAAATATCACCTATTGAAAAGCCACGTTAAAACAAAAAC
TTTTTCTAGTGGGAAAATAAGCTTTTCAAAATGAACTATAGTAGTTATAAATTTTGGGGTATT
ATTCTCTATTAAAATTATATAACCTATTTCATCAATGCTTAATGAGCCCTGAATATCCGACAT
GTTGGGTCTCTATGTAGCTTGGGCTTCCCTGGTAGCTCAGCAGGTAAAGAATCCGCCTGCAA
TCAACCCTGGTTTGATTCCTGGGTCAGGAAGGTCCCCTGGATGAGGACATGGCAGTCCACTT
CAGTATTCTTGCCTGGAGAATCCCCATGGATAGAGGAGCCTGGAGGGCTAGAGTCTATGTGG
TCACAATGAGTGGGACACAGCTGAGCGATTCAGCAGAGCTATGCAGCTGTAAGTGAAAGAC
TGAAAACTTTCCAGAGTGTCTTTATCAGCAAGTACATTGGTTTGGCTTAAACTTCTGACTGTT
ATAAGGAAAGTAACTGCTCTTTATACAACAGTATGTCTTTTTTCTTTACAAATAGAGAAGAA
AAATTTTCTAGGTTGATTACTTGATTTACTCGATTTAGAAGAATCACAGATGTAGATTTCCTT
AAACAGGCCTCATGTCGAATACTGTAATTATGCCTTTGGGAAGATTTATAAACAACTGAAAA
ATAAGGAGACGCTGTGCAGAGGAAATAAAGTAAAGACGGGAGAGTGAAGGTGTGGATTTAT
TCAATAGATCACAAAAAGAATGAGCCAACAGTCTTTTCAAAAGTAGCAGGACATACACCAC
TAAATGGAGGATGGTCTGAAGGGACAGCAAGGAGTGGAAATGCTCATGCAGAGCAGATTTA
ATGAGTGACCCTCCTTATTTGACTGCGTGCAGCTCTTGAGAGCTGGGAAGGAGCCTTCCATG
AACCCGGGAATGGGAAAATAACTGCCTTATCCGTGAAGTACATCAGAGAAATTTTCTCTAGA
GCTGGAGAAATTGGGCTGATTTCTAGAAAATATAACATCTTTAATATATATAAAATTTGAGTCT
TAACTATTTTTCCATGAATTTCTGATATGTACCTTACCTTTCTTCTTTCCAGGGAGCAAAAGA
GTCTCTACATTTATGCACCAGCTATGTCAGGATCTTTCTTATTATCACCTTGCAAAAGTCAAG
ATTTTCATGCTTTATTTTTGCAAAACACACCTTATCTTACAGTTCAAGAACTGGTGACACTCC
CTCCCACCCGCCACCCATAAGCAGCAGCACATTTCAAAAGGATTATTAAGACATCATTAAAA
TCCACACTTCGAACAAAAAACCAGCACTTAAAAAAATTCAACATATTCACAATGGAATGGA
CTTCAGTTTCTACAGATGTGGCTTGAGCGTGACCCACTTATTTTCACTTGGTACTGATTCACC
GATTTATTCCCAATGCTAAATTCAGCAGCGAGATGGTCATTCTACACAAATCCTGGACCCTG
AACTAGCCTTCCCCATCAAAAGGAAAACATCACTACCCTCATCAGTGTCCCTGCAAAACACA
GACCCAGTACTATCAGGACATTGCCTCTGAAGAGCAAATATTTTGAAAGATATTTGAAAACA
TAAACTAAATGACTGGCCTATGAACCATTTATTATACATCTCTCAAGTTTTTTCCTCTATGGA

ATCCTTTGTACAGCATCAGCAAGGCATTATAACCATGGTATTTATAACAATATGTGCTTGTAT
TTTTGTCAATGGTTATGCATTTGGACGTGTTTGAGAACATTGCTATCTTCCATGTTCTACAGC
TCTTCTGGAAATCTGAGCAAATTTTCATCTTTCTACTCAGTTGCATACAACTTATGCTTGGCA
CCTCCAGAATTCTGTGCTAGTACTGAGAGATGTAAATGATAATACAGAAATTATTGTGTGGA
AGATTACTAGCTGTAGAACGTGCATTCATAGTTATTTGTTGTAAAATTTCTTGCTTTCATTGT
CACCAGAAAGACTGGTTTTGAACATTAAAAGACGTTCCTTAAAATCTTGTACTTTGTCTAGTT
CCTGATGTG

>NC_056057.1:C41968317-41861053_(CD36_GENE)

GCTGGAAGTCTCAGGATGTCAATGGCTTCACATGTCAGGATAACCTTAACGACAGACGAATG
GTTAAGACCCTGAGCCTCATTTCTGAGTTCTCAGCTGTGGAAATCATGTGTTTATTTTCTGCA
TCTCCTCCTACAACAGTCACTCTGAAGCCAGTTTTAAGATCCTACATCTGGGCAAGCAAGCT
TCTCTGAAAGTTGGTGAGCACAGTTATTTTTGTTTAGGGGTGTGGCTATAAATGTCTTTGTAA
TTGATGGACTGTCATCAAGTAAACATTAATACTTGTTATTTCAAACTCCTTAAGAAGCTAAAT
ACTAACTGGTGTAATAATTTAGTTTATGATATTGTTTAAACAGAGTTATAAGCTTTTAACTAA
ATGGACAGACTTTTTAAGACAAAGTTTTCATTTCCACTAGTCTCTCAACTGATCCTATGAAAT
TTCCATTTATACATTCTCATATCTTTTATTTTCAAAGTATCAGAAAACTTAACCCAAGTATTTT
TAAAAGTACAATTTTGGTGGAACTTTTTCTTCCTTAAGATTCTGAAGTTGATCTGTCATGTCC
TGTTCTTAAACTGATTTCTTATTTTATTAATTTTCGTATGTCTTGTTTTAAAAATGTCACTCTTT
TGAAAAGGATGCTCTCAAATTCCGAGCAGTTCAATTTTCTGCTTACAAGTTGAAAAAACATG
TTTTTAATGAGATTAGAAGTTGAATGACCTTTGTTCATGGGACCTAAAAATGTTTTTTGGTGT
TTTGTCAAGACTGACAAGTATATTTAAGAAATAATCTAAAAGACTCAATCCTCTTAACAAAC
TCTTAGAAGAAAAAAATATTTCCATAAATAAAATTACTGATCACTAGTGATCACAGAAATTA
TACTGACTGTTGGCACCAGATATTTGATGGAAGGGAATTTTGTTTCATGGCAAAGTTTTTGTT
CTATGGTGTGTTTCATACTATGGCTATATAAGACCAGGAAACAACAAAGAATGATAGAGGA
TGACTTATTCAGACAGGAAACTAAGGGTATGCTTAGAGACAAATAAGTATATAATGATATAC
TTCTTTAAAGACAACTGCTGATACCAAAAAAGCAGTTCCATCTGACTATGCTATGTTACAAA
GTCTGAGTATGAAATGAACAGGCTTCTTTACCTTTAAAAAAAGTCACCTTCCATAATTCTTTG
GAGAATGGTCTCTTGGAGCCGTGCTGAATTCAGGGGAAAGCACTCTGGACTAATACGTTTTA
AAGTATTAACTTCTGTTCACTTCCTATGCAAGAACTCAGGTAAGATCTGGATCAGATGAAGA
GAAACAAGCAATCTGTCCTGTTCTCAGGTATACCTGACTAAGTAAAGAAGGAGGGTTTGTGG
GACAATATATGAGACTCCTGCAATCCTGGCAAGGGGAAAGGAGAAATACTTTCAGAGGATG
TACAATACGAACTGTAATTATTTGGAATTTGATCCTTTTGTAAAGGACAATGACGACTTTGC
AATGAATTATTCTGAGAATTACAGAAAGAAAGCAAACACTTGAGAATAATAAAAAGATTTT
CACCTGGGGAAAAATGGTAAGCTCCCAAACTCAGAAGAATCATTGTTAGGCAAAAAAGGAA
AAACAAAGAAGGAAAAAAGGCTTTAAAATGCTAACAAACGGGTGGCAAAGACCGTATAAG
ATGGCAGAGTGGATCAATCAAATGAAAGAATATGTTTTTCTTGGAATGAAGAAATACATAAT
AATGTACTGCCCTTGAAGTGATTAGGACTAATTTTGCTTTTAGCATAGATTGTTCGTTGAGAT
CACTAGCTAAGGAATTTAATATTCTTAGTATTTACAAACTAAACTTCACAGTCGAATTTTTTA
AGGAAAAATTCACAAAATTTTAAAATGAAAAAGAAATGCTTTTGGATTGCGGGGGACGACC
CGTGAAGGGTTAAGTCTTGGGAGCTCCCTGGCAGGTATGCCAGGCCCTAGGACACGTGCCTA
AGCTCCCTGTCCCGCCACCCTCAAGAGTTTTTATAACCCTTAAGGCTCCAAGATGTTTGGTTT
CGGCAACATTTCATAGAAGATAGATTATCTTATTGTGTATATTTCATAGAAGATAGATATTCT
GATTGTGTTCTGTATACAATGGTAAGGGTCTGGTGATTGTATCCTGAGATTAAAAAACAACC
TTGTGAGTGCCTTAAGTCACGTACTTTACCCTATATATACTGCAGCACAATAAAGCAAGGTA
TCAGCCATTTTGGGGCTGATCCTCTCAACCCCATCTTTTGTCTATCTCTTATTTTCTTAGCGGG
GACGCTCCGTTCTCTCCCTGTGCAGGTGCGACTCTTGCTTGTGCTGGCCGCGGCAGGTGGCG

CCCAACGTGGGGCTCGAGCTCGACAGTTTTCCTCGCCACTACTCTTATTAATTGAAAAGAGT
GAGTATATGAGTAAACAAGTGAATTAAATTGAGGAGGAGTAGTAAGGTATATAGTTGAGAG
TATAAATATGGGACAGACGCATAGTCGCCAGTTGTTTGTGCATATGTTATCTGTAATGTTAA
AACATAGGGGAATTACTGTTTCTAAACCTAAATTAATCAATTTTCTTTCATTCATCGAGGAAG
TTTGCCCTTGGTTCCCCAGAGAAGGTACAGTAAATTTGGAGACATGGAAGAAGGTAGGGGA
ACAAATTCGGACTCATTATACTTTACATGGCCCTAAAAAAATCCCTGTCAAAACTTTATCCTT
TTGGACACTAATTCGTGACTGCCTGGACTTTGATAATGATGAATTAAAACGTTTAGGAAATT
TATTAAAACAGGAAGAAGATCCTCTCCATGTTCCTGATTCGGAACCCAGATATGCTGTTCCC
GAGGGGGTTAAAAGCGACCCTCCGTTTTCTAACTTATTGCATCCTTCAGATAATGATGATTTA
CTTTCATCCACAGATGAGGCAAAATTAGACGAAGAAGCTGCTAAATACCATCAAGAAGATT
GGGGTTTTTTAGCACAAGAAAAGGGGCGTTAACATCTAAAGATGAATTGGTTGAATGCTTTA
AAAACCTCACTATTGCTTTACAGAACGCAGGAATCAAGCTTCCTAGTAACAATGCCAAATCT
CCTTCTGCTCCGCCTCTTCCCCCTGCTTATGCTCCTTCTGTTGTGGCTGGTCTCGATCCCCCTC
CAGGGCCCCCTCCACTGTCTGAGAACATGTCTCCGCTGCAAAAGGCATTGAGACAGGCACA
GCGACTTGGTGAGGTTGTCTCTGATTTTTCTCTTGCTTTTCCTGTCTTTGAAAATAACAACCA
GCGTTATTATGAATCACTGCCTTTTAAACAACTGAAAGAGTTAAAGATTGCTTGCTCACAAT
ACGGTCCTACCGCTCCATTCACCATTGCTATGATAGAAAATTTGGGTACTCAAGCTTTACCTC
CAAATGATTGGAAGCAGACAGCTAGGGCATGTCTCTCAGGGGGAGATTATTTATTATGGAA
ATCTGAATTTTTTGAACAATGTGCTCGTATAGCTGATGTTAACCGACAGCAAGGTATACAGA
CCTCCTATGAAATGTTGATTGGTGAAGGCCCTTACCAGGCTACTGATACTCAACTTAATTTCT
TACCTGGTGCATATGCACAAATATCAAATGCGGCTCGGCAGGCATGGAAAAACTTCCTAGCT
CCAGTACTAAGACAGAGGATCTTTCAAAAGTCCGGCAGGGACCTGATGAGCCTTACCAGGA
CTTCGTGGCACGACTTTTAGATACTATAGGTAAGATAATGTCAGATGAAAAGGCTGGGATGG
TACTGGCAAAACAATTGGCTTTTGAAAACGCTAACTCTGCTTGTCAAGCTGCTTTAAGACCTT
ATCGAAAAAAGGGAGATCTGTCTGATTTTATTCGCATTTGTGCTGACATTGGACCCTCCTAC
ATGCAAGGCATTGCTATGGCAGCAGCATTACAAGGAAAAAGCATAAAAGAGGTACTTTTCC
AGCAGCAAGCCCGGAACAAGAAAGGACTTCAAAAGTCAGGTAATTTGGGTTGCTTTGTTTGT
GGTCAGCCTGGCCATCGGGCTGCAGTGTGCCCTCAAAAACAACAAAGCCCTGTTAACACTCC
TAATTTGTGCCCACGCTGTAAAAAAAGGAAAGCATTGGGCGCGGGATTGCCGTTCCAAAAC
GGATGTTCAAGGTAATCCTTTGCCCCGGTTTCGGGAAACTGGGTGAGGGCCAGCCCTGGCCC
CGAAACAATGTTATGGGGCAACACTGCAGGTTCCAAAAGGACCATTGCAGACCTCTGTCGA
GCCACAAGAGGCAGCGCGGGATTGGACCTCTGTGCCACCTCCTACACAGTATTAACTCCCGA
GATGGGGGTCCAAACCCTTGCCACAGGAGTGTTTGGGCCTTTACCTCCAGGGACAGCTGGAC
TGCTTTTAGGGCGCAGCAGTGCGTCTTTAAAAGGAATACTTATTCATCCTGGTGTGATTGACT
CTGATTATACAGGAGAGATAAAAATATTAGCCTCCGCTCCTAACAAAATTATTGTAATCAAT
GCAGGACAGCGTATAGCTCAACTTCTTTTAGTTCCATTAGTCATACAGGGAAAAACAATTAA
CCGAGACCGTCAAGATAAAGGTTTCGGGTCCTCTGACGCCTATTGGGTGCAAAATGTTACCG
AGGCACGACCAGAACTTGAGCTACGCATTAATGGTAAGCTTTTCCGAGGAGTGCTTGATACA
GGGGCCGATATTAGTGTTATTTCTGATAAATATTGGCCTACTACATGGCCAAAACAGATGGC
TATTTCCACTCTCCAGGGTATTGGCCAAACTACCAATCCAGAACAGAGTTCATCCCTTCTTAC
TTGGAAAGATAAAGATGGACATACAGGCCAATTTAAACCTTATATTCTGCCCTATCTTCCAG
TTAATCTATGGGGGCGTGATATATTGAGCAAAATGGGTGTTTATTTATATAGTCCTTCACCCA
CTGTGACAGATTTGATGTTAGATCAGGGCTTACTTCCAAATCAAGGTTTAGGTAAACAACAT
CAAGGCATCATTTTGCCCCTTGATTTAAAAATCTAATCAAGATCGAAAAGGCTTGGGGTGTT
TTCCTAGGGACCTCTGATTCTCCTGTGACACATGCCGATCCTATTGATTGGAAATCTGAGGA
ACCGGTATGGGTCGATCAGTGGCCCCTAACACAGGAAAAACTTTCTGCCGCACAACAGCTG
GTGCAGGAACAGCTGAGACTTGGGCATATTGAACCCTCTACCTCTGCTTGGAATTCCCAATT

TTTGTTATTAAAAAGAAGTCTGGGAAATGGAGATTGCTACAAGATCTTCGTAAGGTAAATGA
AACAATGATGCATATGGGAGCCCTACAACCTGGGTTGCCCACTCCTTCTGCTATACCTGATA
AATCCTATATCATTGTTATAGATTTAAAAGATTGTTTTTACACTATTCCTCTTGCACCTCAAG
ATTGCAAAAGATTTGCTTTCAGTTTACCCTCTGTTAATTTTAAAGAGCCTATGCAACGCTATC
AATGGAGAGTTCTCCCGCAAGGAATGACTAATAGCCCTACGCTGTGCCAAAAATTTGTTGCT
ACAGCAATAGCTCCGGTTCGTCAACGTTTTCCTCAGCTATATTTGGTTCATTATATGGATGAT
ATATTACTAGCTCATGCTGACGAACATCTATTGTATCAAGCTTTTCGATTCTAAAACAACATT
TAAGCCTTAATGGTCTTGTTATTGCTGATGAAAAATTCAGACTCATTTTCCTTATAATTATTT
GGGTTTCTCCTTATATCCTCGTGTTTATAATACCCAATTAGTACAATTACAGACTGACCATTT
AAAAACTCTAAATGACTTTCAAAAACTTTTAGGAGACATTAATTGGATACGTCCTTATTTAA
AATTACCCACTTATACCTTGCAGCCATTATTTGACATCCTTAAAGGTGACTCTGATCCTGCGT
CACCCCGAACACTTTCTTTAGAAGGACGAACTGCTTTACAATCAATAGAAGAAGCTATTAGA
CAACAACAGATTACTTATTGTGATTACCAACGATCATGGGGTTTGTATATACTTCCTACCCCC
CGAGCACCCACAGGGGTTCTCTATCAAGATAAACCTTTGCGATGGATATATTTGTCTGCTAC
TCCAACTAAACATCTGCTCCCTTACTATGAACTTGTTGCAAAATTGTAGCAAAGGGACGTCA
CGAGGCCATCCAATATTTTGGTATGGAACCCCCTTCATTTGTGTTCCTTATGCTTTAGAACAA
CAAGATTGGCTTTTTCAATTTTCAGATAATTGGTCTATAGCTTTTGCAAATTACCCGGGACGG
ATTACTCATCATTACCCTTCTGATAAATTGTTACAATTTGCTAGCTCTCATGCCTTTATTTTTC
CAAAAATAGTTCGCCGACAACCTATTCCCGAAGCGACACTTATATTTACAGATGGATCTTCT
AATGGAACTGCAGCTTTAATCATTAACCATCAAACCTATTACGCACAAACCAGTTTTTCTTCT
GCTCAAGTTGTGGAATTATTTGCAGTCCACCAAGCGTTGCTAACTGTACCTACTTCCTTCAAT
TTATTTACAGACAGCTCCTATGTGGTCGGTGCCTTACAGATGATTGAAACTGTTCCAATTATC
GGCACCACCTCTCCTGAAGTTCTTAACTTATTTACATTGATTCAACAGGTTCTCCATTGCCGC
CAACACCCCTGTTTCTTTGGACATATTCGTGCACACTCCACCCTTCCTGGTGCCCTCGTACAA
GGCAATCACACTGCGGACGTTCTTACTAAACAAGTGTTTTTCCAATCAGCTATTGATGCAGC
CCGAAAATCCCATGATTTACATCACCAAAATAGTCAGTCTTTACGCTTGCAATTTAAAATTTC
CCGTGAAGCTGCACGGCAAATTGTTAAATCTTGCTCTACTTGTCCTCAATTCTTTGTTCTCCC
TCAATATGGTGTCAACCTCGAGGTTTACGCCCTAATCACCTCTGGCAAACAGATGTTACTCA
CATTCCTCAATTTGGGCGTCTTAAATATGTTCATGTTTCTATTGACACTTTTTCCAATTTTCTC
ATGGCTTCCCTTCACACTGGAGAATCAACACGTCACTGTATTCAACATTTGCTGTTTTGCTTT
TCTACTTCAGGAATCCCACAAACCCTTAAAACAGATAATGGACCTGGTTATACTAGCCGTTC
TTTTCAACGTTTTTGTCTTTCTTTCCAAATTCATCATAAAACAGGAATTCCTTATAATCCACA
GGGACAAGGTATTGTGGAACGAGCCCATCAACGCCTTAAACATCAATTATTAAAACAAAAA
AAGGGGAATGAACTGTATAGCCCCTCACCGCATAACGCCTTAAACCATGCTCTTTATGTTTT
AAATTTTTTAACTTTAGACGCAGAAGGCAATTCAGCAGCCCAGCGTTTTTGGGGAGAACGAT
CCTCATGCAAAAAACCACTTGTGCGATGGAAGGATCCACTTACCAATCTGTGGTATGGGCCA
GACCCTGTACTAATATGGGGACGAGGGCATGTTTGTGTTTTTCCACAGGATGCCGAAGCGCC
GCGCTGGATTCCGGAAAGGCTGGTACGCGCAGCAGAGGAACTCCCTGACACATCAAATGCA
ATGCATGACACTGAGTGAGCCCACGAGTGAGCTGCCTACCCAGAGGCAAATTGAGGCGCTG
ATGCGTTATGCTTGGAATGAGGCTCATGTACAACCTCCAGTGACACCTACTAATATACTGAT
CATGTTATTATTATTGTTACAGCGGATACAAAACGGGGCAGCTGCGGCTTTTTGGGCATACA
TTCCTGATCCGCCTATGATTCAATCCTTAGGATGGGATAAAGAAACAGTACCTGTATATGTT
AATGATACAAGTCTTTTAGGAGGAAAATCAGATATTCACATTTCTCCTCAGCAAGCCAATAT
CTCCTTTTATGGTCTTACTACTCAATACCCTATGTGCTTTTCTTATCAATCACAGCATCCTCAT
TGTATACAGGTGTCAGCTGATATATCCTATCCTCGAGTGACTATTTCAGGCATTGATGAAAA
AACCGGAAAGAGATCGTACCGTGACGGAACCGGACCCCTCGACATTCCGTTTTGTGACAAA
CATTTAAGCATCGGCATAGGAATAGACACTCCTTGGACTTTATGTCGAGCACGAATTGCATC

GGTGTATAACATCAACAATGCCAATACCACCCTTTTATGGGACTGGGCACCTGGAGGAACAC
CTGATTTCTCCGAATATCGAGGACAGCATCCACCCATTCTTTCTGTAAACACTGCTCCTATAT
TTCAAACTGAACTGTGGAAACTTTTGGCTGCTTTTGGTCATGGTAATAGCCTATATTTACAGC
CCAATATTAGTGGGAGTAAATATGGTGATGTGGGAGTTACAGGATTTTTATATCCCCGAGCT
TGTGTTCCTTACCCATTCATGTTGATACAAGGCCATATGGAAATAACACTGTCATTGAATATT
TATCATTTAAATTGTTCTAATTGCATACTTACTAATTGCATTAGAGGTGTAGCCAAAGGAGA
ACAAGTTATAATAGTAAAACAACCTGCTTTTGTAATGTTACCTGTTGAAATAACTGAAGAAT
GGTATGATGAAACTGCTTTAGAATTGTTACAACGCATTAATACGGCTCTTAGCCGTCCTAAA
AGAGGTCTGAGCCTGATTATTCTGGGTATAGTGTCTTTAATCACCCTTATAGCAACTGCTGTT
ACTGCTTCTGTATCTTTAGCACAATCCATTCAAGCTGCTCATACTGTAGATTCCTTGTCATAT
AATGTTACTAAAGTAATGGGAACTCAAGAAGATATAGATAAAAAATAGAAGATAGATTATC
AGCTTTATATGATGTAGTTAGAGTTCTAGGAGAACAAGTTCAGAGCATTAATTTTCGCATGA
AAATTCAATGCCATGCTAATTATAAATGGATTTGTGTTACAAAAAGCCTTACAATACTTCTG
ACTTTCCGTGGGATAAGGTGAAAAACATCTGCAAGGAATTTGGTTTAATACTAATGTTTCTTT
AGATCTTTTACAATTGCATAATGAAATTCTTGACATCGAAAATTCTCCAAAAGCTACTTTGA
ATATAGCTGATACCGTCGATAATTTTTTACAAAATTTATTTTCTAACTTTCCTAGCCTTCATTC
ACTGTGGCGAAGTATAATTGCTATGGGCGCGGTTCTGACTGTTGTGCTTATCATAATTTGTTT
AGCTCCTTGCCTTATTCGTAGCATTGTTAAAGAATTTCTACATATGAGAGTTTTAATACATAA
AAACATGTTGCAACACCAACATCTTATGGAGCTTTTAAAAAATAAAGAGAGGGGAGCTGCG
GGGGACGACCCGTGAAGGGTTAAGTCTTGGGAGCTCCCTGGCAGGTATGCCAGGCCCTAGG
ACACGTGCCTAAGCTCCCTGTCCCGCCACCCTCAAGAGTTTTTATAACCCTTAAGGCTCCAA
GATGTTTGGTTTCGGCAACATTTCATAGAAGATAGATTATCTTATTGTGTATATTTCATAGAA
GATAGATATTCTGATTGTGTTCTGTATACAATGGTAAGGGTCTGGTGATTGTATCCTGAGATT
AAAAAACAACCTTGTGAGTGCCTTAAGTCACGTACTTTACCCTATATATACTGCAGCACAAT
AAAGCAAGGTATCAGCCATTTTGGGGCTGATCCTCTCAACCCCATCTTTTGTCTATCTCTTAT
TTTCTTAGCGGGGACGCTCCGTTCTCTCCCTGTGCAGGTGCGACTCTTGCTTGTGCTGGCCGC
GGCATTGGATGAGAATTGTTTTTAATTGGTCATTTGTTTATACATTGTTGATTGCTCTTCGGA
TGAAAATTGAAAAGCGTGCCGTCACTCTAGTACCACCTAAAAGCAGAGATCTGGAGTGGTG
AGCAGGGAGGTGCTGGTGGAGGAGTGATGCTGTTTCCACACCAGGTTCCCGGAATCTGAAA
TCATGCCTAATCCAAAATGACTCTTTCAATCACAAACAATGAATTGTTTGTCAGGAAAAGGA
AATAGTCTAAACTAGGGTAAGTGTGCCGGGAATATAAAAAGACATACAGTTTAGTAAATAG
TTTTACTGTTTTGTGTGCTGCATGTTGAAGTCTACATATTGACTAAGCCTCTGGAAACTTTCT
CTCCATTACTGTGTTCTCGTGCGTACTAATATCCCTCCCTTTTGATTTAGGTAAAATGAAATG
GGAGAAATTTGGGAAAATTAATTAAAATTAACCCATTACATTTCTCTCTGACAGGTAATAAG
TATGCCATTGGTTCCACGATGCACTTTCTAGTCATTGTGGATCAGGGCAAGGTGAATTGAGT
TTTGTGTCTTCTGACAAATTTTGTCTTCTGGAGAATAAATAACACTATTAAATTGGGGCTTGG
TATTACATTGTTAAATAGTTTCAGATGTTTTGAAATATATCCAGAGACCTTTGTCACACACGG
CCATCTCAAGGAGGAAATGAGAGAAAATTGGCAAACGTTCTGGAGGTGACATAGCAGACAG
CTTGGCTGCCTTCATCGACGACTCCGCAGTGAGCACAAATAGAAGAGCATTGTGCATCCGTC
AGTGCTTGTGAACTTTAACAGTGGAGTGAGGACAGTTTCAAAAGTGATGAGTTTGATCTCAG
TGTTGTTTGCTCTTATTAAATCTTCCAAACTTCAATCTTACATTCCTCCCATCTTAATTTTCCC
AAATTTCTCCCATTTCATTTCACCTAAATCAAAAGGGAGGGATATTAGTACGCATGAGAACA
CAGTCTAATTGAAATTCATTTGGAGGTTCACTTCATTCTATGTAGAACTTCATTATTACTCTA
TTTGAAATGTAACAGATGCTGTTATAAGGGGTCTGTCTTGTATCTCACTCTAAGAAGCTTTGG
AAATAAAGAAATAAAAGGTTATTCAGTACAGTGGTCCCATCTCATGGACCCTAAATACAGTA
TCTTCTCAGAGTAGGCATACACTTTAGTAAGTGGCATCTTTCCTACAAGTATTCTTACTTTGC
TGCTAAGTCATTTCAATCATGTTCAACTCTGTGTGACCTCATAGATGGCAGCCCACCAGGCTC

CCCTGTCCCTGGGATTCTCCAGGCAACAACACTGGAGTGGGTTGCTATTTCCTTCTCCAATGC
ATGAAAGTGAAAAGTGAAAGTGAAGTCACTCAGTTGTGTCCGACTCTTCATGACACCATGGA
CTGCAGCCTACTAGGCTCCTCCATCCATGTGATTTTCCAGGCAAGAGTACTGGAGTGGGGTG
CCATTGCCTTCTCTGATTCTTACTTTGATGGGACCCTAAAACAACAGAAACCTACTTTGGTTG
AATACATAATAGAGTCATCCTCTTTCAGATAAACTTATTTCTCGATGCATTTGTTGAAAAATA
TTTGAGTACTTACTCTATGTCAGACATTGTTGTAAACTACAGAAAACATTATTAGATACCTTA
TTGTGCTGAACACTGACAAGGATCCAGGATCTTTTTCTTTTCAGGTGGTGATTCTAAATTTTG
TTTCTATGGAATGAAACCTTTTGATGAAAGTGAAAAAAGAAAACCTTAAGTTAGTAAATGAT
GAAGACACAAGTTGAATTCTGTGGATACTTTGCCACCACTGCACACAAACAGACCCTGTAAG
GGCCTATCAATCTCCTGAAGAAAACAGGCCAAACAAGTGACTTAAAAATAATACAATATTT
ACAGGAGGAATAGAAGGAAGATACCAGAGTGAGCATCTTGCTGTTGCCGTTATAAATGCAT
AATTGACTCTTAAAATTCTAGTAACTAACATATATAACACATGGAAATACCTCCAAAACATA
ATAACTGTACTGTGTGCTCTAAGGCTTGTTGTTGTTATTGCTCAGTTGCTCAGTTGTGTCTGA
CTCTTTGTGATCCCATGAACTGTAGCATGCCAGGCTTCCCTGTCCTTCACTATCTCCTGAATT
TGCTCAAACTCATGTCCATGAGTTTGAGACGATGATACCATTCAACCATGCCATCCTTTGTCG
CCTCCTTATCCTCCTGTCCTCAATCTTTCTAAGCATCAGGGTTTTTTTTTTTGCAATGAGTCAG
CTCTTTGTATCAGGTGGCCCAAGTATTGGAGCTTCAGCATCAGCATCAGTCCTTCCAGTGAGT
ATTCAGGGTTGATAACCTTTAGGATTGACTGGTTTGATCTCCTTGCTATTCAAGAAACTCTCA
AGAATCTTCTCCAGCACTGCAGTTGAAAAGCATCAATTCTTTGGTGCTCAGCCTTCTTTATGG
TCCAACTCTCACATCCATACATGACTACTGGAAAACCATAGTTTTGACTAGATGGACCTTTGT
CAGAAAAGTGATGTCTCTGCTTTTTAGTACCCTGGCTAGATTTGTCATAGCTTTTCTTCCAAG
AAGCAAGCACCTTTTAATTTCATGGTTGCGCTCCACAGTGTTTTTGGAGCCCAAGAAAATAA
AGTCTGTCAATGTTTCCATTTTTCCCCATCTATTTGCCATGAAGTGATGGGACTGGATGCTAT
GATCTTAGCTTTTGAATGTTGAGTTTTAAGCCAGGTTTTTCTCTCTCCTATTTTGCCTTCATCA
AGAGGCACTTTAATTTCTCCTCAGTTCCTGCCATTAGGGTGGTGTCCTCTGCATATCTGAGGT
TATTGATATTTCTCCTGACACTCTTGATTCCAGCTTGTGCTTCATCTACTCTGAATATAAGTTA
AATAAGTAGGGTGACAATATACAGTCTTGATGTACTCATTTCCCAATTTGGGACCAGTCCAT
TGTTCCATGTCTGGTTCTAACTGTTGCTTCTTGACTGACCTACAGGTTTCTCAGGAGGCAAGT
AAGATGGTCTAGTATTCCCATCTCTTTCAGAATTTTCCAGTTTGTTGTAACCCACATAGTCAG
GGATTTAGTGTAATCAATGAAGCAAATGTAAATGATTTTCTGGAATTCCTTTGCTTTTTCTAT
GATTCAATAGATGTTGGCAATTTGATCTCTGGTTCCTCTGCATTGTCTAAATACAACTTGTAC
ATTTGGAAGTTCTCGGTTCATATACTGTTGAAGTCTAGCTTGAAGGATTTTGAGCATTACCTC
GCTAGCATGTGAAATGAGTACAACTGTGTGGTAGTTTGAACATTCTTTGGCATTGCCTTTCTT
TGGGGTTGGAATGAAAACTGACCTTTTCCAGTCCTGTGACCACTGCTGAGTTTTCCAAATTTG
CTGGCATATTGAGTGCAGCACTTTAACAGCATCGTCTTTTAGGATTTTAAATAGTTCAACTAG
AATTGCATCACCTCCGCTAGTTTTGTTCATAGTGATGCTTTCTAAGGCCCACTTGACTTCACA
CTGCAGGATGTCTGGCTCTAGGTGAGTGACCATACCGTCATGGTTTTCTGGGTCATTAAGAC
CTTTTTTGTATAGTTCTTCTGTGTATTCTTACCACTTCTTCTTAATCTCTTTTGCTTCTGTTAGG
CCCTTCCTGTTTCTCTCCTTTATTATGCCCATCTTTGCATGAAATATTCCCTTGACATCTCTAA
TTTTCTGGAAGAGATCTCTAGTCTTTCCTAGTCTATTGTTTTTCTCTATTTATTTTCATTGTTCA
CTTAAGAAGGCTTTCTAATATCCCCTTTGGAGAGAGAAGCTTTGAAACATCTGGAGTTCCAA
AGCTATTCTTTGGAATTCTCCATCAACTGTGTATATTTTTCCCTTTCTCCTTTACCTCTCACTC
CTCTTCTCGTCTCAGCTATTTATCTCAGCTATCTTATTTCAGACAACCTTTTGCCTTCTTGCAT
TTCTTTTTCTGGGGGGATAGTTTTGGTCACTGCCTATGTATAGTGTTATGAATCTCTGTCCAT
AGTTCTTTAGGCACTCCATCTATCAGATCTAATTCCTTGAATCTATTCATCATCTCCACTGTA
AAATCATAAGGGATTTGATTTAGGTCATACCCAAATGGCCTAGTGTTTTTTGTTACTTTATTC
AATTTAAGCCTGAATTTTGTGATGAGAAACTGATGTCCTGAGTCACAGTCAGCTCCAGGTCT

TGTTTTCACTAACTATATACAACTTTTCCATCTTCAGCTACAAATGATATAATCAATCTGATT
TTGGTATTGACCATCTGGTGATGTCCACATGTAGATTCATCTCTTGTGTTGCTGGAAGAGGTG
TTTGCTATGATCAGTGCATTGTCTTGGCAAAACTCTGTTAGCCTTTGTCCTGCTGCTTTTTTGT
ACTCCAAGGCCAGACTTGCCTGTTACTCCAGGTATCTCTTGACTTTCTACTTTTGCATTCCAG
TCCTCCATGATGAAAAGGACATCTTTTGTTCGTGTTAGTTCTAGAAGGCCTTGTAGGTCTTTA
TAGCTTCTTTGGCATAAGTGGTTGGGGCGTAGACTTGGATTACTGTGATATTGAATGGTTTGC
CTTGGAAACAAACGAAGATCATTCTGTCACTTTTGAGTCTGCACCTAATTACTGCATTTCAGA
CTCTGTTGTTGACTCTGATATCTACTCCATTTCTTCTAAGGAATTCTTGCCCACAGTAGATAT
AATGGTCACCTGAATTAATTTTGCCATTCCCATTCATTTTAGCTTAGCTTCCTAAATTTACTTC
TGATGGAAGTGTTTTTGTCTGCAGTGGGGACAAATGTAATCTAAATTGTAGTTGGGTCTCTTG
GTGGAAAGACTCTAGTTTGCATGGAAACATCTAGTTTTTATGAGCCCAGTCTTTAAGAGCTG
CTTGGTCAAAAGTTACATCCAAGCAGACACGTAGACAGGGAATTCCAATGCATTACCTTGAG
CTGTGATAAGGCCAGTGCTCAGAGATACCAGCTCTGTTCTTTGATAAAAGGCGTCTGTTTAC
AGACCTTTGCTATTATGTGGAGTCTGCACTTCTTGCTGGGTTGTGAACTCCATGAAGGCTGGG
TTTCGTTTGCAACTTAGTATCAGCAATATCCAGTGCTGGATACATAGTAAGCACTTAATAAA
CAGATAGTACCTATTTATTCAAAAATAGGTGTAAATCTTTATAACCCAAAAGGTTCACCTTC
CAATGAGGTCTACATTAGGATCTTAAGAATTTTTAGTCATGTAAAACAATTTAAAATCTTTTA
TCTCCATTTCTTATGCTTAGCACTTAAAAAAAATATCCAACTTGACATAAATCTCCACCATAA
AGTAATACATAGATGTGTGCAGTTTTGTGAGTGAGAATTCTCAGGAAAGCAGTTTCATGGAA
TATTACCATTACCAGTACCACCCTCACCTTTCTCAGCACGTCTGTTTTATTTTATTTTTTAACT
CTCTCTCTCTCTGTGTAGGGTGGATCATCTATTAAAATTATAAAATTTAACAGAAATATTCTG
TTTCATGCTACGCTAAGTATGCAAATTCATAGACACTGATAATTTCTCAATGAACCAAATCC
GTGACGCTCACTCATCTCACCTAATGCCATGAAGACCTTATCGTTTTTCCCATTCTACGTGCA
GAGCTTAGATATGTTTGCTTCTGCGTTCCGTCTCCTAGGTGTCTACAGTATATCTACCAAGTG
TTTAGATAAAGCCAGAGAAAAATTCAGTCGTTCCACTCTGGGTCCAATGGGCATTTTTAAGA
TTTGCTAACAGGGAAAGTACAGGTTTTGATTTCTCCACTTCTAACTTCACGGGAAACTGTAG
CATAGTAGATGTTCTTTGTTAGCCTAATCACACATACCGCCAAAACCCTCCCTTGAGGTGGA
TGTAATGGCCACAGCAAGTGGAGAGATACAGACATCACTCTCTGTTTTCTTTCTCAATTCATT
GGTCAGAGTTGCGTCCTGACCTCATTGAGAGTTTGTCTCCTGTTATCCAAACTTCTTTGTTTC
ACATACTATAGTTTAGTTATAGCAGTTCAGCAAAATGCTATGTTTTACCGGAAGAAGTCAGG
AAAACCAGGATTAGGCAAGGAAAAAAAGCAAATGCTAATATCAGGGGGCAAACAGAAAGG
AAGCTTACCATAATGATCTCCTCCCTCTTCTCTGCCTCATTAATATTCCTCCTGTGTATGAAG
GATGCCATTTTGTTTTCTTCCTCACGTCTCCCACGCAGAACTCTGTACATAGTAACAGCAGGA
GCATTTGTGAAGCCGAAATCCTGTAGAATAAATAAAAGCAATTGAAACCATCTGAATGGGC
TTTTGTGAGAAGAAAACTTGTTTAATAATAATTTACCATGGCAGAGCAGCAGCTATGAAGCT
TAGCTTTCTCCCTCTGGTTTAAGGCTAAAAGTCACCTTTCAGCAGGTCTTCCAATGTTCACAT
TTGTTCACAACAGAAGCTGCAGACATTGGAAGTGAGAATAGTTCTTGGCACAGTCTTAAACC
ATGATTCACTAATTTTTTTTTTTTTTTTTAACAAGTGAGGACTCTGAGGCCCAAAGAAGTGAA
GTGAGCTTTCACGTCTGGGCCTAGCCTTTGGATTGCCCTTTCTGCAGTTTTTCTAGGCTCCTCC
TTCCATTCAACACCAACATCTATTTTCTTATGGTTTCAAACTATAACCTAGCTGTTGAAGAGA
CCCAAATCTTTTCACCACCCCAATCTCTTCCCTGATTATTAGACTCATCCATTTATGTGCCTAC
TGCTCAGGTTCTAGACATGGATGCATTACTCGTGAGTTGAAAGTGTCTAACCTCTTTCAACAC
ATTATCTTGTTACTGTGCTTTGATTCATGAGGTGCATTTTATGCTCAAAATTGTAAACAAATA
CGATTGAGTTTTGTTTCCTACTCTGCTTGAGTATCTTCTAAAGTTCAGTAAAACATGCATATA
TATGTACTCTGCAAACACTTTTAAACTAACAGGCTGGATTAAATTATAATAGCTAATTATAA
CCTACTGGTATGTTCAGGGCACTCATAATAATTATGAAAAATGCCTAGTATTTAATGAGAGG
TACTGGGATTCACTGGTTGAGATACTCTCTGTAGCTGGATTGAAAAGATTTGAAACCTGGAA

TCTGCCACTTTCCAGCTCTGTGAGCTTGGGGAACATATTAAACCTCTTTGTATCTCTGCTTTG
TCTGTTAAATAAGATTAATATAAGTACTGCCTTTATGGACCAGTTGCTCAAAATAAGCAAGT
GACTATGTAAAATATGCAAGTGCCTTGCACATAAGTTCTGAAGAAGGGTTAACTACCATTAT
TTAATTCTCACAATAGTTTTAAATAGGCCTTCATATAGGTACGAGTAAACATGCTCATAGAG
ATAAAGATATTTCCCCGGGTCCTGCAGTTAGTGAGAATGCTAGATAGAGCTATCCTGTCCCA
CCCCAGTCAGTTCAGTCACTCAGTCATGTCCGACTGTTTGCGACCCCATGAATTGCAGCACG
CCAAGCCTCCCTGTCTATCACCAACTCCTGGAGTTCACTCAAACTCACGTCCATCAAGTTAGT
GATGCCATCCAGCCATCCCATCCTCTGTTGTCCTCTTCTCCTCCTGCCCCAACCCCTCCCTGC
ATCAGAGTCTTTTCCAATGAGTCAACTCTTTGCATGAGGTGGCCAAAGTATTGGAGTTTCAG
CTTTAGCGTCAGTCCTTCCAAAGAACACCCAGGACTGATCTCCTTCAGAATGGACTGGTTGG
ATCTCCTTGCAGTCCAAGGGACTCTCAAGAGTCTTCTCCAACACCACAGTTCAAAAGCATCA
ATTCTTTGGTGCTCAGCGTTCTTCACAGTCCAACTCTCACATCCATACACGACTACTGGAAAA
ACCATAGCCTTGACTAGATGGACCTTTGTTGGCAAAGTAATGTCTCTGCTTTTCAATATGCTA
TCTAGGTTGGTCATAACTTTCCTTACAAAGAGTAAGCGTTTTTTAATTTCATGGCTGCAGTCA
CCATCTGCAGTGAGTTTGGAGCCCCAAAAAATAAAGTCTGACACTGTTTCTCCATCTATTTCC
CATGAAGTGATGGGACCGGATGCCATGATCTTTGTTTTCTGAATGTTGAGCTTTAAGCCAAC
TTTTTCACTCTCCTCTTTCACTTTCATCAAGAGACTCTTTAGTTCCTCTTCACTTTCTGCCATA
AGGGTGGTATCATCTGCATATCTGAGGTGATTGATGTTTCTCCTGGCAATCTTAATTCCAGCT
TGTGCTTCTTCCAGCCCAGCGTTTCTTATGATGTACTCTGCATAGAAGTTAAATAAGCAGGGT
GACAATATACAGCCTTGACGTACTCCTTTTCCTATTTGAAACCAATCTGTTGTTCCATGTCCA
GTTCTAACTGTTGCTTCCTGACCTGCATATAGGTTTCTCAAGAAGCAGGTCAAGTGGTCTGGT
ATTCCCATCTCTTTCAGAATTTCCACAGTTTATTGTGATCCACACAGTCAAAGGCTTTGGCAT
AGTCAATAAAGCAGAAATAGATGTTTCTCTGGAACTCTCTTGCTTTTTCCATGATCCAGTGGA
TGTTGGCAATTTGATCTCTGGTTCCTATGCCTTTTCTAAAACCAGCTTGGACATCTGGAAGTT
CACGGTTCACGTACTGCTGTAGTCTGGCTTGGAGAATTTTAAGCATTACTTTACTAGCATGTG
AGATGAGTGCAATTGTGCAGTAGTTTGAGCATTCTTTGGCATTGCCTTTCTTTGGCATTGGAA
TGAAAACTGACCTTTTCCAGTCCTGTGTTCACTGCTGAGTTTTCCAAATTTGCTGGCATATTG
AGTGAAGCAGTTTCATAGCATCATCTTTCAGGATTTGAAATAGCTCCACTGGAATTCCATCA
CCTCCACTAGCTTTTTTTGTAGTGAGGCTTTCTAAGGCCCACTTGACTTCACATACCAGGATG
TCTGGCTCTAGGTGAGTGATCACACCATCGTGATTATCTTGGTTGTGAAGATCTTTTTTGTAC
AGTTCTTCTGTGTATTCTTGCCACCTCTTCTTAATACCTTCTGCTTCTGTTAGGTCCAGACCAT
TTCTGTCCTTTATTGAGCCTATCTTTGCATGAAATGTTCCCTTGGTATCTTTAATTTTCTTGAA
GAGATCTCTAGTCTTTCCCATTCTGTTCCTTTACTCTATTTCTTTGCATTGATCACTGAGGAAG
GCTTTCTTATCTCCTCTTGTTATTCTTTGGAACTCTGCATTCAGATGCTTATATCTTTCCTTTCC
TCCTTTGCTTTTTGCTTCTCCATTCTTTCTTTTTATATTGTTGCTCTGAGTCTAAAACCTAGAAT
TCATTTTGTGTTTAAACCCCTCTACTTAGCTCTACTAACCAAGTCTTGGCTGTTTATACTACCT
TCTAAAATCCCTGAGTGACTCTATTGCATTCAAGAGAAGTTTCTGAGAGAATGAATTCCTAG
GTATTCAGGCAAAACCCTCAGCCTGGTATATTATGCATTACATTAGGGCTTCATGATGTCATT
TGTGCCCACCTCTCCAGTGTCCTTTCTTTTCCAAAAGTCGTACATTGTACCAAAGCCTTACCA
AAGTGTATTACCCTGTTGTACAATTTCTTAGGCATCTGTAGTTTAGGAGCATGCCTCTAATCC
CTTTGCTGTTTCCTCCTTCCTTTCCTGGACCCTGTGTACTATACGCAGTAAGCTATCACTTCTT
CCACCAGCAGTCCTTTCACTTTCTTACATGACCCACCGTTTTTCTTCCCATCCAGGTGTAGGA
GTCACAGTAAATCAATGTTATAATTGTCTGTTTACAAAACTGTCTTCCTAGGGATGCTGTGGG
CTACTTGCATGAAAATACCAGGTTTTTGTTCTGTTGATTCCCTCATTTCATCCCAATTATTAGT
CCAGGCTAAGCATGCTTAAGGAATTTATTTTATTTTACAGTTTCAGTGGCTACAGGTTAATGA
AACATTTAGCAGTGTAAATGGAAGAATAAAAGTATAAACAAATGAGAAGCAAAGTTCATGA
TTCAAACTATTATAGAAAAGTAGCTTCAAAAGTAATCTAATATTTTTTAAAAATGCAAAAAG

CTTGATAGGGGAAATGATCCAATATGAAAACCAGTAAAAATCATTGCAAAAAGAATGGAGC
TAGGATTTGTGTATCTTCTGGGTTGTGATCCAGTGGTGAAGGAGCCAGACTAGAAGAAGTTG
GTTCAGGTTGCAGGTGTGACTGCTTGGCAAGTGCTTGCCATGTAGGCAAGAGGTTGAGGACA
AGATAAGATTAGGTGAGGACATATACTATCATGGGTAAGCCAAGTTCAAAGGCTTACGTAG
TTTTAAATTCTTCCTTCTTTTGGAAAATAAGGACTACTAAGTGAGTGCTGGAAACTACAGAG
TATGAAGTATTATCTCATAGCACTAGGCTCAATTTTAATAGTTGTTTAAATCAAACAAGATTT
AACACACTAGATCTCAAAATATTGACATGCATGAGAGTGACTAAGAATGCTTGATGCTTAGG
CCATGCTTAGGCTATTCTCTTGCTCTCTGAACATCCCTTGCTCAACCACTCCCTGCCCACCTA
TGCCCTACTCACATCCTACCTACCTTCCTTGTTAATCACTGAATCTAATCAATAAGTGCCTAC
AAACTTGTCCCTGGCCCCAACTAGGGATTCTTCCAATCTTTCTCTTCCTTTCCTTTGCTTCTTT
TTTATTTTTATTTTTTTGGCTTTACCATATTGTGTTAGTTTCTGCTGTACAAGGAACTGAATCA
GTTATATGCATACATATATCCCCTCCCTTTTGGACTGTCCTCTGCCCCACCCCCATCCCACCC
ATCTAGGTCATCACAGTCCAGGAGCTGAATAAAATAGATAGCTAGTGGGCACTGCTGTCCTA
ATCTTCTGATCAGAACATCTCCACCAAGATAGCTTATCAAAGGGGAATGAGGGATGCACTCC
TCTATTGGTTTCTCTGGTAACTGATGACTCGACCTGATGTCAACTGCTAACTTCCCTCTCCAC
ATCCTCTGGCCCCCTGCCAGCAGACTGCTACCATGTCTTGCCCATTGTCTGCCCCACATGGTG
GAGTGTCAATTCAGGAGCTTGCTTCTGACATGTAAGATCCCCCATTCATTACACCACTGATGT
GTCTGCCACTGACTTTGAGCTCTTTATTCAGTCTTATGGCTGGGCAAGCACAGAGGGTGCAG
TCCAAGTGTAGCCGTAATGCCTGCTTCTAAGCAATTCATTGTTATATGTCATATTGTTGTTAT
AATTTTATAACTATCTTCATCATCATTGTCAGTTACTCTTATTTTTTACATTTGTACCCTGATC
TTCCCATCTTGCTGATTATCTGACATTTTAATCCCTTCTAAATACACACTTTGTCTTCTTAATC
TCTTCTAAATTATGAAAATTCACCTACAACTATCTTGATAACACTTTACTGGGAGGGTTATTA
GTTTCCTAGTGATATTTAGCTTTTCTTTCGTGCTCCTCTTTCCATGGGATTTCCCAGGCAAGAA
CACTGGAGTAGGGTTGCCATTTCCTTCTCCAGGGGGTCTTCCCAGCCCAGGGATCAAACTCA
CATCTCCTGCTTGGCAGGCAGGTTCTTCACAGCTGAGCTACCAGGGAAGCCATGGTTTTTAC
AGATATAATACATGTATAAGAAGAGTATATTTTCAACATAGTGGCTAAGACTTGGTATTCAG
ACTTAAATTTTCTTCTTATAACCCTTTCTTCTTTCTAGCTGTGAAACAGGGTTATAAGACTCTT
TGGTGTTTCAGGGTTCTTCAGCTCATGATCTGCCTCTCCTGTGCCCTGAATCATGTTCTTCATC
TCAGTGGGATATTTCGCTCCATACTTGAAACAGTAGCTTTAGCCTCAGAATCTTTGTCTAGTA
TCATTTTGCATACCCCATAATAATCCCTCCTGTTCCTCATCACTGTGTCATCTACTAATGTTCC
TTCTGTGAAGTTCTATTTGTTGTTGTTGTTTTAGTTGCTAAGTTGTGTCCAGCTCTTTGCAATC
CCATGGACTGTATGCCTGCCAGGCTCCTCTGTCCATTGTATTTCCCAGGCAAGCGTATTGGAG
TGGGTTGCCGTTTCCTTTTCCACGGGATCTTCCCTGCTCAGGGGCTGAAACCACATCTTCTGC
ATTGGCAGGTGGATTCTTTACTGTTGAGCCACCAGGGAAGCCTGATGTTCTATTTACTCTTCT
CCTAAATTCTGGGAAATTATATAACCAAATGCATCAGAACCACCTGATAACACAGGCATGA
GGAGTCAAACTTTTTAGCTGCCTTATTACTGTCCTTGCCCTTAAGCCAGAGACTACCAAGAA
GACACCTGTAGTTGAAAAAGTTGAGTTCATTACCTATTGCAAAGAGGAAAAAGTGTACCATG
GGGTCAGAAGGCCTTAAAAAGAACCTCTTATAAAATCTGAACTTCGGTTGAATGATCTGGGG
AGAGAGTTTAAGGAAGTAGAGCATTACTCTGAATTGAATGTTGTCAGGAAGCAGGGTAATTT
CACAATCTGGTGCTTTAAAAGGTCTTATTTAGGAAGAGGGAAGATAAGCCTGCCTTGGTAAT
GCAGCAGCAGTGACCCAGATTAGCTGGGAAAGGGGGACATTTGATGCCTGTGGCTTAGACA
GGGTTTATAAGAGGTCTGTATTCAGACATAATTACAGAGCGTTTTGTTTTGTCTTGATTCTTC
ATGATCATATAGTGTCCTTGCCTAAAGCTGATGTTCTGTGAAATTGCTTCTGTTCAACATGAG
GATACCACAGCCAGGTGTGAGAGCCAGGGCAGTGTCCAGAAACACTGTGATAGCACCGGCC
CAGGTCCTGGATGTCAGGGGCTGTTTCTCTCTCATGAAAAAATTCTTTACTACTTCAGTAAT
TAAACTTATTTCCATGAAATCCACTTCCCTCTACTTTGGTGATAAGATTCTAACAGTTCTTTG
TAATTTCCTCCCTAAGCCCTATGGTATGCTCAACCCATGGTGTATTTTAATACAAAATTTCAT

TTCTCTTAAGCTAGCAATATGATGATGATCAATAAGGACCACAAAAGTAGCTAGATCTATGT
ATAATAACTACAAAGGGCACAGATTGTTTAGGGAACTGATTATTTTTTGTATTTATACCATA
AACACTAATTTTTTGTGTAAATATAAACCATTGGCATATAGGAAATAAATGGAATAAAATTT
TAATTTTATTTAGTGGAAGTTACTTGTGAGGACTTTAAAGGGTCTTTCCACTTACTATATAGA
AAAGAGTTTAATAATCAGAGACTGGAATTTTGGAGTTGGGAATTATGATGAATCAAAATATC
TCTGGAGATATATACTTGGCTAAACATATAAATGTGGTTAAAACTTTACTACTTAGACATTCT
AGATGCCTACTAAAAAAAGTGAGATAATCTGTTTCTTTTACATAATGGACTGTCATTATCCTA
AGTTTTACTTAAAGAACACCTAATTAAGTGATTTAAGTTCATGATTGAATAATCTTTAAGTAA
TGTATGGTTTTAAATGAGTAGAAACTGGCAAATGTCCCATGCTAATAATTTGCTTTGTAACTT
GTTACAGATATGTAGAGGAAGTGTTTTGGGTGGAAGGCTCTGAAGAGAGAAGGAGATAAGG
TTATATAAATCAGGCCTATTCTATAAGTCACACATGAAAAAATATCTTCCACAATGTAATAT
TTAATGTATATGTAATATTGCTAAATATAAGAATTTTTTTGACTTATATGATCTTTAAACATG
AGTTATTTAATAGGCTCAAGATTACCAATTTCATATGTAAGATTAAGTTTCTTCATCTTTGAG
TTTTTTGATTGGTTTTAGAATAGAATAAAAGCAATAAAGAAGAAATAGTTACTTATTTTCCA
ACTCTGCTTTACTTATCATACGGTCTTATTAGTAAGGATAAGATGTTTTGATTGAACTTCATA
AAATTTGGCCTTTATTTACCCTTGAAGATATGATCTAAACCAAGAAAGATGCTCATTTTTCAT
TGTCTCTTTACGGTTTACTCTTTTGTATGATTCTATTTCATCTTTCCTGTTTTCAAGTAAAATG
TGTAGCTATGTTTTATGTGAACCAGAAATAATAATTTACAACAGGAAATGGAAGTTAAAAAG
TTAAAATTCAAGTTAAATATTTAACTTTTAATTAGACAATAAGGCTTAAATCATTTTAAAGAC
TATAGATACTTGTCATGGGCATAGAGGTGGGTCTGTTCACTCAGTACAATCGAAGGCAGCAC
TTTGGATCCTTAAATCTGAATGGTTCAATAATTAAACTACAGAAGATTCCTGATAACTGATTT
AATTCTTTGTTAACAGTAATAAGTGAGAATACAATAAATATCCTTGGCTACTTGGGAACTTG
GCCTATTCTTGGAGTCTATTTTATGCGGGGCCAAATTAGTATAATGTGCTTTTGGATATGTGT
GTTCTTTAATATTACTGTCTAGAAGTTTACTACAGATGAACTTATCTAACTGCTTTTTGTCAA
AAGAAGTATTTTGAGTGCCCATTGAAGCAGTAGGTACCTATCTGAAATGAAAAGGATTCAA
GCCACTCAATTTTCCTTCCTGAAATATCCCTGCACAAGGTGTGCTTCTGCAAATTCTTAACAC
ATCACAGATGGTCTCGAAACTGTTGTTGCGACTGATGCCAGAATAAAAAGGTAGCTTTCTTA
TACTTGTTTACATATATTCCTTTCTTCTTGGAGCATATCAGGCTTGGAGGCAAACAGTGGCAA
TGGTTTTCAACTGCAAAACCTATCCAGATGATTTCTTCCAGAGTCTCCCTTTTCCCCACTGTC
ATTAGGCTTAGATTCTCCTCTTTTCCATCCTATCCTTTTTGAACGGTCATAATGCATTTACATT
TTATACATCTACTGGCCAAACGAGAAAGATTACATGTTCAACAAAAAGCAGGCTCATATAGT
CATTTTTTTCCATTGTTTCTTTCCAACAAGGTCCAGTTATTGACTCTAGACTGAGTAAACCAA
AGGATCGACGTTTCCTGCTTACTCTTCCATGGCAGTTTGCAGTGGACCTCTCCATCTCTTGAT
CTTTGTTCCTCACCCTCATCCCCACATCTCTTCCCCAACTCTCATGAGACTGAGCATGGCCCT
AAGTCCAGCAAACTCAGCCTGGAATGTGAAGTGGGGAAAGTGAGAAGTTAGGAGATTAAAT
ACTTCCTAATAAAGCTCAACATTCAGAAAACAAAGATCATGGCATCCGGTCCAATCACTTCA
TGGGAAATAGATGGGGAAACAGTGTCAGACTTTATTTTTTCGGGCTTCAAAATCACTGCAGA
TGGTGACTGCAGCCATGAAATTAAAAGACTCTTACTCCTTGGAAGGAATGTTATGACCAACC
TAGATAGCATATTCAAAAGCAGAGACGTTACTTTGCCAACTAAGGTCCGTCTAGTCAAGGCT
ATGGTTTTACCAGTAGTCATGTTCGGATGTAAGAGCTGGACCCATAAAGAAGGCCAAGTGCC
GAGGAATTGATGCTTTTGAACAGTGGTGCTGGAAGACTCTTGAGAGTCCCTTGGACAGCAAG
GAGATCAAACCAGTCAATCCTAAAGTAAATCAACCCTGAATACTCAATGGAAGGCCTGATG
CTGAAGCTCCAATGCTTTGGCCACCTGACGTGAACAGCTGACTCATTGGAAAAGCCCCTGAG
CTGAGAAAGATTGAGGACAGGAGGAGAAGGGGGCGACAGAGGATGAGATGGTGAGATGGC
ATCATTGACTCAATGGACTTGAGTTTGAGCAAATTCTGGGAGATAGTGAAGGACAGGGAAG
CCTGGCTTGCTGCAGTTCATGGGGTCGCAAAGAGTTGGACATAACTGAGTGACTGAACAATA
CTTTTCCTCCACTTCCAGTTTCAACTGAACTCAGGATTTAATTGATGGAAAAAGCTATTAGCT

TTTCCTGAAATGTAACCAGTCATTTTTCTATTATTTAAGGTATCTAAACAGACACTATGGAGA
AGGCAGTGGCACCCCATTCCAGTACTCTTGCCTGGAAAATCCCATGGATAGAAGAGTCTGGT
AGGCTGTGGTCCATGGGGTCGCTAAGAGTCGGACAACTGAGTGATATCACTTTCACTTTTCA
TTTTCATGCATTGGAGAAGGAAAGGACAGCCCACTCCAGTGTTGTGCCTGGAGAATGTCAGG
GATGGGGGAGCCAGGTGGGCTGACGTCTATGGAGTTTCACAGAGTCGGACACTACTGAAGT
GACTTAGCAGCAAACAGACACTAAGAACTGTTTTATTGACCTGTTGAGACAAAATGACTCAT
AGTTAATCATAAATTCCTTTAATATTGTTCAGTTCAGTTGCTCAGTGGTGTCCGATTCTTTGC
GACCCCATGAATTGCAAAAGGCCAGGCCTCCCTGTCCATCACCAACTCCTGGAGTTCACTCA
GACTCATGTCCATCGAGTCGGTGATGCCATCCAGCCATCTCATCCTCTGTCGTCCCCTTTTCC
TCCTGCAATCCCTCCCAGCATCAGGGTCTTTTCCAATGAGTCAACTCTTCACATGACGTGGCC
AAAGTACTGGAGTTTCAGCTTTAGCATCATTCCTTCCAAAGGAATTCTTTCCAAAGGAACAC
CGAGGACTGATCTCCTTTAGAATGGACTGGTTGGATCTCCTTGCAGTCCAAGGGACACTCAA
GAGTCTTCTCCAACACCACAGTTCAAAAGCATCAATTCTTCAGTGCTCAGCTTTCTTCACAGT
CCAACTCTCACATCTGTACATGACCACAGGAAGAACCATAGCCTTGACTAGATGGACCTTTG
TTGGCAAAGTAATGTCTCTGCTTTTAAATATGCTATCTAGGTTGGTTATAACTTTTCTTCCAA
GGAGTAAGTGTCTTTTAATTTCATGGCTGCAGTCACCATCTGCAGTGATTTTGGAGCCCCCCA
AAATAAAGTCTGATACTGTTTCCACTGTTTCCCCACCTATTTCCCATGAAGTGATGGGACCAG
ATGCCATGATCTTCGTTTTCTGAATGTTGAGCTTTAAGCCAACTTTTTCACTCTCCTCTTTCAC
TTTCATTAAGAGGCTTTAGTTCCTCTTCACTTTCTGCCATAAAGGTGGTGTCATCTGCATATC
TGAGGTGATTGATATTTCTCCCAGCAATCTTGATTTCAGCTTGTGATTCCTCCAGCCCAGCAT
TTCTCATGATGTACTCTGCATATAAGTTAAATAGCTGGGTGACAATATACAGGCTTTATGCA
CTCCTTTTCCTATTTGGAACCAGTCTGTTGTTCCATGTCCAGTTTTAACTGTTGCTTCCTGACC
TGCATACAGATTTCTCAAGAGGCAGGTCAGGTGGTCTGGTATTCCCATCTCTCTCAGAATTTT
CCACAGTTTATTGTGATCCACACAGTCGAAGGCTTTGGCATAGTCAATAAAGCAGAAATAGA
TGTTTTTCTGGAACTCTCTTGCTTTTTCCATGATCCAGTGGATGTTGGCAATTTGATCTCTGGT
TCCTCTGCCTTTTCTAAAACCAGCTTGAACATCTGGAAGTTCACGGTTCACATATTGCTAAAG
CCTGGCTTGGAGAATTTTGAGCATTACTTTACTAATATGTGAGATGAGTGCAATGTGTGGTA
GTTTGTACATTCTTTGGCAATGCCTTTCTTTGGGATTGGAATGAAAACTGACCGTATATATGC
ACTAATTCCACATATATGCATTAATATATGATATTTGTTTTTCCCTTTCTGACTTACTTCACTC
TGTATTACAGTCTCTAGGTCCATCCACATCTATAAATGACCCAATTCCATTCTTTTTTATGGC
TGAGTAATATTCCATTGTATATCTGTACCACATCTTCTTTATCCATTTCTCTGTTGATGGGCAT
TTAAGTTGCTTCCATGCCCTGGCTGTTGTAAATAGTGTTGCAATGAATATTGTGGTACATGTG
TCTTTTGGAATTATGGTTTTGTTTGGGTATATGTCCAGAAGTGGGATTGCTGGGTCATATGGT
AGATTTATTCCTAGTATTTTGAGAAATTTCTCTACTGTTCTCCATAGTGGCTCTATCAATCTAC
AATCTCATCAACAATGCAAGAGGGGTCCCCTTTCTCCACACCCTCTATGACATTTATCATTTA
CAAATTTTGCAATGACGGCCAATCTGATTGGTATGAGGTCGTACCTCGTGGTAGTTTTGATTT
GTATTTCTCTAATATTTAGTGATGTTGAGCATCTTTTCATGTGCCTCTTGAGCATATGTATATC
TTCTTTGGATAAATATCTGTCTGCTTAGGACTTCTGCCTCCAGCTGCTTTTTTTTTAAGGTTGT
TTGTATTTTTTATATTGAGTTGTATGAGCTGCTTTAATGGGAACACATGTACACCCATGGTGG
ATTCATGTTGATGTATGGCAAAACCAATGCAGTATTGTAAAGTAAAATTAAAAAAAAATTAGG
TCCCATCTGCTTATTTTTGTTTTTATTTTCATTTCTCTAGGAGGTGGATCAAAAAGGATCTTGC
TGCAATTTGTGGCAAAGAGTAGTCTGCCTATGATTTTCTCTAAGAGTTGTATAGTCTGGCCTT
ATCTTCAGGTCTTTAATCCATTTTGACTTTATTTTTATGTATGTTTATTTTTATCACTTTATTTA
TTTATTTATTGTATCATTATTATTTATGTAGTTTATTTTTAGGAAGTGTTCTAATTTCATTATTT
TTTCATGTAGCTATCCAGTTTTCTCAGCATGACATATTGAAGAGGCTGTCTATTCTCCATTCT
ACCTTCTTGCCTCCTTTGTCAAAGGCAACCACAGGTGTGTGGGTTTATATCTTGGCTTTATAT
CCAGTTCTGTTGACTTAAAAAGTTATTTCTAACATCACATTTCTGCTGCTTAGCTAAAATGAG

GAAGTTGGTCCAGATGATCTATAAGGGATGTTCAAATACTAGGTTTATTTGATCATCTATTGT
GTGAATCTATGCTTCTGTTACCTTCTTTTTTAGTATATACTATTGCATTGAAGTTTCCCAAATA
TTTTTTTAAGTATTGACAAATGACCATAATTGCAACAGGAGAAATTATTCTAGATTTACAGA
GTAGGTGACTGCAAGCTAGTAAGCAACCGGCTATAATATCTTGGTCAAATTCTAAACAAAGA
ATTACAATTTCTGTGTTGATTTTCTGATTGTAGAATTATATAGCAAATGTTTGTAAAGATTTC
AATAAAATTTTCAGGCTAGACAGAAATGATACAAGTAGAATGGTTGCTGAAATTTCTACACA
AATCTCCTACTAGAAAAATGAACTTAACAACTATTCATGCCCACAAATACCTTCACAAGAAG
CTCAGGATTCCATGTGATAGTACCTGGGTCAAGGAAGTACTATATTTTGAAGAGATTTTAAA
AGATACACAATGGATAGCCTCAAACTTTTTAAAGCAGGAAATAAAACTTTATCCTGAGAAG
ATAGGTTTCCAGGGAAATACTTAGGAAAATGAAGATGCCACAGCAAAAAAACCAAGAGAGC
GAAGGCAAGAGTCACAAGATAGGAAAAAAGTAAAGGACTGCTTAACACAGCTCAATTCAAT
TACAATCTTGTTTTTAAAATGTATGTAGATATCAAATGAATGTTTGTTAGTTTTTTAAAAGAC
CACATGGTATTTTAAAACTTAAAGAAAATAGACAATTGCCTTATTTTCTCCACCCTGTCCCAT
TCCCCATGTTAAATTTTAGAATAATGAACTATTTTTAGATGACATACTTAAAGTGTTCTTGAT
GTTTTTTTTTTTTAAAGAAAAACACAAAAATCTGTTTTTTAAATACAATTTCTTCCTGTAGAA
GAGAAGGATCTAACTTTTATGTATTAAATACAATACAGCTTGATCCATCACATCTATATACA
TATTTCTCATCTTCTACCTAATAATGTAGTTATTTTTCTAATATTATTGACTGTGTAAATCATT
ATCATAAATTTTCTAAGCAGCAGAGCCAAGAAATAAACAATGAGTGCTTTCTTTCTGGTGGA
ATTTATGTTTTCCTGGTGATAATTGCATCCTTAAATCCTCTGTCCTCCTGGTCCAATATTAACT
GGTTTTTGTTTTTTCCCCCTTGACTTGTGACATAATTCTTACTGCGAAACTTCCCTTCCGCTGT
TCCTGGAAATTCCCTATGCCTCTCATTTGTTGTATTAGCTATTTCCTTAATTCCCTGTCTTCTT
TTACTTCCTTGTTTCAGTAAAATTTATATTCTGAGATTTCTTGGGCTCCTTAAAAAACAGTAA
AAGAGAAGTAAAATTTAGGACTGTGTGTATATTAAAATGTCTATATCCTCTTTCTAATTTTTG
GACATTTTTGAAGAGATTGTTATTTGACTTGTGAATCCCCAGTGCTGCTGTCTGTTGTCTCTTT
GATTTGTATGTGACATTACCCTCAAGCCCCCGAGAGGTTTTTAAGAATTTCTCTTTATTCTTG
GTACCTGTGATGGGAAAACAGGCAGCTAGGTCATAGCCTGTGGCCCTTTATATCCACTCCGC
TGTAGATGGGTGACTCTGCACTCTGGGGGTTTAATGTCCTTTGCTTCTGGAAAATATTCATCA
TTCTATCCTATTTCCGCTTTGTCTGGTTGTGTTTTCCCTGGAAACCCCACTGTGTGGTTGTTGG
CTCTATCCTATAGCTAGCTGCTCTAATGCTGCTCCCGTGGCTCCTCTGTACTCAGATATCCCA
AACGCCTGCTCCACTGTGTTACTTCTACTATCACGGTTTTCTTTTTTCAGATTGCTGCCTTCTA
TTTATGTGGATTTTAGGATTTCATGGTTTTCTTTTAAAAGTGATTCTTTCCTATTTATTTTTTA
AAATTATGATTAGAATTTTTACATGTAATTTTTAGATTAATTTTTAACTCTTTGGCTGTGCTGG
GTCTTCATTTCTGCGCAGGCTTTTCTCCAGTTGTGGTGAGTGCAGACTACTCTCTCTTTGTGG
CCGGTTCTTTTGTTGCAGGGGCGCATAGGCTCTAGGTGTGTGGGGTTCAGTAGTTAGGGCTC
CCAGGCTCTAGAGCACAGGCTTGGTAGTTGTGATCCGCAGGCATAGTTGCTCCACGGCATGT
GGTTATCTTCCTGGACCAGGGATTGAATCATGTCTTCTGCGTTGGCTGGTGGATTCTTTACCA
CTTAGCCACCAGGAAAGCCCCATGGTTTTTTGTTTTACTAGTTTCATGACACAAAATTGTCTC
TTAGTTGTCTAATGATAGTAATCACAAAAATATTTTGTGTTTTTTATTCCCTTTCCTTGTATTC
AACCTTAATGTTTACTGTATTTAACTTTTTTCATGCAAGTCAGAAGTATTCCTCAAATGTCTA
GTGATTATCCATTTATATTTAAGATTGAGGCAAAACGAATACAGATTTCATTCTAGAGTTGC
ACTGTCCAATATGGTAGCCACTAGCCCCAAGTGACTATTGAGAACTTGAAACATAGTTAATC
CAGATTGTGTTCTAAGAGTAAAGTAGAAACCAGACTTTTAACACTTTGTGCCCCTGCAAAAA
AATCTCATCAATAATTTATGTTGGTTTAAATGACAGTATTTATGATGTTTTCAGTTCAGTTCA
GTCGCTCAGTCGTGTCCAACTCTTTGCGACCCCATGAATCGCAGCACGCCAGGCCTCCCTGT
CCATCACCAACTCCCAGAGTTCACTCAGACTCACATCCATCGAGTCAGTGATGCCATCCAGC
CATCTCATCCTCTGTTGTCCCCTTGTTCTTCTGCCCCCAATCCCTCCCAAAATCAAAGTCTTTC
CCAATGAGTCAACTCTTCGCATGAGGTGGCCAAAGTACTGGAGTTTCAGCTTTAGCATCATT

CCTTCCAAAGAAATCCCAGGGTTGATCTCCTTCAGAACGGACTGGTTGGATCTCCTTGCAGT
CCAAGGGACTCTCAAGAGTCTTCTCCAACACCACAGTTCAAAAGCATCAATTCTGTGCTCAG
CCTTCTTTACAGTCCAACTCTCACATCCATACATGACTACTGGAAAACCATAGCCTTGACTAG
ACGGACCTTAGTTGGCAAAGTAATGTCTCTGCTTTTCAATATGCTATCTAGGTTGCTCATAAC
TTTTCTTCCAAGGAATATGATGTTGTAGTAAGTAATATATAATATTAAAAGTAATATTGCCTA
TTTACTTACATTTTCAGTGTGGCTACTGGATATTTTAAAGGGATATTTAGCATGAGGCTTCTG
TCATATTTGCACCAGACAGTACTACTCTGATTGAGCAGAAGGTCCTGACCATTTCACTGTGCT
CCCTGACCTCCTCCCCACTTCAGCTAATAAGTAGTGTCAAGTGTTTTCTCTTGTGCCAGTTTC
CTTAGAAAGGAATCCTATAATATTTTGCTTGATAGAGTTCTGGAATTGGTAGGGGAAGAGAA
ATCAGTGTGTGGGCTTAAACTTATTTAGACATGGATCTCCCTCATACCCCCAGCTGGGCTTCC
TTGTCTTAGGTCCAGAAATCTCTTACTCCATGTCTCAAAAGAGGAAACACCCGATCTTCTGA
CAGATGAGGAGGAAGGAGAGTGAGCAGGCTGTGTAAAGTATATGGAGAGAAGATGTAGGT
TTAGGACTTCCTGAGATATTCAATCAAGTTAAGACTCTGCTTTCCATGCACCCCATGGTTCTA
TGTGGACTTATACAGTAGATTCTTTTTGCTTCTGCTCTAGGTGTACGTGTTTAGAATCTGACA
TGCAGGATGTTTGAACAATTACCATTAGTCCATTATGCTTTCTATATGTTGACTGGTTTCTTG
TCTTCACTCATCTAAATCTTTGAGCTGAACATGTGTGTTCCTATTTTTATAAATTCTACCTACA
ACTTTCATTTTAATGAGATTTCAGATGTAAGTGAAAATGTATTCCACCTGCCATGTTTAACCT
GAGGTCATAATTTTAAATCTTAATATCTTGTTATTCCCTTCTATATTTTTTGTCAGATTATATT
CAGGTACTTAAGTCTCCTATAACCTGCTCTAATATGTCCATCTCTCTGTAGATAGGAACCTTT
ATGGCAAGCCAGGTTTAGCTTGTGCACATGCCTTTTTCCAACCATACCAATTACATTTCTAAA
TGATTTACATGATTTATCTAACTTTATTTTCCACGAATCCCACTTCCTTCTACTTTTTTACATT
TAACTCATCTTCTAGGGAAGATTAAGGATGTCAGAGTTCTATGAACATATTCTTAGAAATTC
AGTCCCCACTGATCTCTTTCTTAGCTAAACTTCTATCATGTGTGTGTTTCCCACAATTTTTAGT
TTTTTATACTATGATAAATTATACTGTGAGGTATTAATCCACATGGTTTTATTCTGCCCACTTC
ATTCTCTTTTCTAACTGCAAGATATGAAGCTGCAAATTGAGGGAAAATTTTTAGTGGATACA
AGGAACTGACATTTGGTTACTATTCAGTCTTACTAAAGACTAGGAAAATAGTTGTGCCCTAA
GCATGGATAACGGAGAAGGCAATGGCACCCCACTCCAGTACTCTTGCCTGGAAAATCCCAT
GGACGGAGGAGCCTGATAGGCTGCAGTCCATGGGGTCACTGAGGTTCAGACATGACTGAGA
GACTTCACTTTCACTTTTCACTTTCATGCACTGGAGAAGGAAATGGCAACCCACTCCAGTGTT
CTTGCCTGGAGAATCCCAGGGACGGCAGAGCCTGGTGGGCTGCTGTCTCTGGGGTGGCACA
GAGTCGGACACGACTGAAGTGACTTAGCAGCAGCAAGCATGGATAAAAGGGAAACACAGA
CTTTAGACATGACCGAATCCCAGAGAGCTGCTGTGATCCGTTCTGCAGCAGACGCACTCCTA
AAGGCAAGTTTTCTTCTCCCTTTCAACTCTCAAGATGGAGGACCTCAGAACACAAGTGTTAT
CAGACAGAACAAGCCTCAGAAAACACTGACTCAGAGACTACGGCAGTGGAGTATCCGGGGC
TGTCCGTGGTACATAGGCTGCTGTTTAGAGGCAGATTCATGGAGCCCCTTGGACCCTGACAC
TGAGCTCAAGGCCCCTGAAGGTTATGCAAGTTAGTTGAGAAAAAGAAAGGTACCACCTGCA
AAGTGCTCGAGGCCAGAACAAGGGAAAGTGATAGCCACAAATTTAGAGGGCATGCTCTGTG
TTCTCATCTCCTTCTCTGAAAATAGCCTGTACTGAGGAAAATGGAACAAGAGTTTTAAAGTT
GAAATGACTGAGGGACAGCAAAAATGAAATTTCTTAAAAAGTGACTAAATATGCTGTATCA
GACAGAATGGAGGTTTGAGATAAAACGAAATTTAGTGCAACAAAATTTTGTTTTTGCACACC
TGAGTCTGTGATTGTAAAACTCATTTCCATTTCAGTAATATACAGCATACAGCCATTCCCAAA
GTTTCTTTCTCACAAAAAACAAATGAAAAAGAAAACCTTTTCCAGGTAACCATGATTATTTT
TCTTTGTTTTAGATTAATTTTATATTTCACATTCCCTTTGTCAGGTTAAACAGTTTTGTTTCCC
AGAGTGATAGAAACTTGGACTTAATTTTTATTTTGAACTTTGATTTTGAAAGCTTAACATGTA
ATATTTTCTCCCTTTAAAGAATTATACCCACCTAGGGGACAGGAGATGGTCTGGATTTTGAG
TTGATTGTATATACATACACATGTATATGCAATTTAAGCAAATAATTGTATTATCAAGATGGT
ATGACAGCCCAATACTTAAAAATGGCTGCTTTAGAGAAGAGCTAGAATTTGGGAAGCTGAA

GGTTATTTCCTAATATTTATGATTTGACAAGTAAAGTTCCCCCATATTCTGATCTACTTTTCTT
GGAGCATTCTCTTACTAGCAGACAAAAGTGTCCTAAGTTACACAACCATAGCTTGGCATTCT
TTGACCTTGAGCAGTTTTCTTGACCTCTCAGTCCTTTTGCCTCTCAGTCCTTTGGTACTTTTCA
GTTCAGTGCAGTCACTCAGTCGTGTCCGACTCTTTGAGACCCCATGGACTGCATGGTACTTGT
AATGTCAGGAAACCAACAATTCTTATTTCAAATTATGGTTATGAAGATTAATTTAGATAATA
CATATAGTCCTTAGAAAGGTGTTTGCCTGTAGTAAGTGCCCTCGATGTTAGCCGTTTGTATTA
CATGACACACTTTGGGCTTTTCAAAAAAATGTGCTTCTGATAAGTTTTATTGCCGGGGTCCAG
TCCCGGTGGATCCAGGGTAATTCAAAGGTGGGGACGGAATCGGCGTCCTGGAAAAAACTTA
TTTAATTACAGATATAGAGAGAGATTGGAAACAGATAGTGTAGTAGGAGAATTAGTGGAGA
AAAGAGGCTGAATAACTGGTTTACATGGAATACCAATCACCACCTACGTAGGCCACAGGCG
TCCTTCCATTCTCCCGAAGGACAGGAGGCACTGAGGCCTCCCCGGTCCGATCTCAGAAGCTC
AGGCAGAATTAGCAGGCTTGGTGAGTACCCACATTTCAGATGGGAATTCAGCCAGGAAAGC
AGGGAGCAAGAAAGAAACAACATGGGGGAATCAGTCTTTCCAGAATTTGATCCAATTTCTTT
ATTTTTCAGGTTTGTTTACATACCTTTTTGTTATACATAGGGATGAATACAGAGTCACGTGGG
GGTCAGCAGACCTGACCCTTGTCACAATCAGGTGCTTCATATAAAATTATACAAAGGTCTTA
TGAGTTTCATCATCTTCTGGCCATGAGGTCTGCTGACATTTTATGGCCCTTTCTGATACCAGT
CAGTTAACCAGAAAACTTATTTTTCCAGGGGTGATTTTTTCTTAAATCAGGCGCCACCCTCCA
AATAAAGTTGCATTCCTATAGGGTGAGGGTGTAGTGAGTTACAATCAAGAAAGGAATTTACT
TAACCTAAGGTTTAACATGATTCATCTTAAAGGTTAATACTTATTTCTCCTATATGCTAGTTA
TATTCATTATAAGGGCAGGAATATGGAGATTTAGCAGCAAATATTGGCTCAACAAATGTAAA
CCCTTCACTAATGCTCCCCTTAAGATCTATTTTGTCTTAAGATAAAGTTACATTTTTGCAAAG
CAAGGACACAGTGATTTATAACAAAGTACAGTGGTCTATTACAAAAGAGAAAATTCATTAA
CTCAAAAAGTCTAGTATTGCTAACATCAAAAAACTACTATATTTCCTTTTCTATATTCCAAAT
ACATTGATTAATATATTCCCAGGTGCCTAAGGATATGGAAGCCTGATGGCAATCATTGACTC
ATCAATGAAAAAAGCCCTATGCTAATACTCCAAACTCTCTGTGCTGTTTATGGTTGAGAGGT
TGTCACACAAGCTAGTCTGTCAGCAGAGAGGTTTGACCTGAGACATCCTTGTCACACTCAGG
GCAGGGAATTAGCAGTAATTATTGGCACGACAAATGAAGAAAAAACCCTTCACCGATATAA
TTCCTAATCAACCCACTAATACTATACTAATGATCTTCTAATTTCTCAAAAGAGTCTGTATTT
AGAAAGTTTTAAAACATCCCGTGCCTCTCACAGTTGGGAGGCTGTAAACAATCACATGTGGC
TGGACGAGCCTGATCAGGCAGGCCAGAGAACCTTCAGAGTTCCTAAGTTGAAACACTCTTGT
CACGCCCAGGAATTTTTATTAACTTGGAGCTGCAAGTTAACTCCTTCTCCAAGAGAAATGTTT
ATGGGGGAGAGCTCCCCGTAAAGTACTCTGGTTTTGGGGGTAGATGCTGGGGAACAGGGTG
TATCCTGAGGCTTGATCATACCTTTGTGTATGCCAAGCTTCCTTCCTCTTGATCTTTGGCATG
GGCAGAGTTCCTCATGCTGGCTCCCAACATTTTATCATTTCACATATTAAACACCAAGTGGTT
TTCCAAGGAATAAAACATGGTGATGTGATTATTTTAAAAGTTAGTCATCACACAACAAGAAT
TTGTCCACTGCCCTCCATAAGATTCTAGAGATCAGTTAAGTTCAGTTCAGTCGCTCAGTCGTG
TCCGACTCTTTGCGACCCCATGAATCGCAGCACGCCAAGTTCAATGACTTTTAAAAGCACTC
ACCGTATGCATACGTATATCCCCTCTTTTTTTTTTTTGATATCCTTCCCATTTAGGTCACCACA
GAAAACTTGAGTATAGAGTTCCCTGTGCTGTACAGTAGGGCCTCATTACTTATCTATTTTATA
CATGATATTTGGAGCTCCTTTCTATCTGCTAGATTGGATGCTGCCAGATTCAAAATGATTTTT
GCTCAAATAAGCTCAATATTTAAAAAATATAAAAGTACTCATCAAAAATAGCTTTGGTCACA
GATAGTCTAAAGGAGGACAGGACATTAGACACCATTTAGGTGGTATTTTCAATTAGACTGAA
ATAATTATTTGGCCTGTAATCTCATTTTACAAGGTTTCCCATATGCCTGAATGTTTATTCGTG
GTTTTAAACATGGAAGCTCTGTGAATTTCAGGGCAAGAAGAGTCCAATCCCTGTCTCCAAGG
ACTTTGGGAGTTACCTATTGGATAGAGGAGAGCAAGACTCTGCTTTTTAATTTTATTTTTTCG
ACACGAAAAAACATTTTGTAATGGGGTATAGCCAACGAACAATGTTGTGAAATTTCAGGTG
AGCAGTGAAGGGACTCTGCCATATATATACATGTATCCACTCTCCCTGGAGAAGGAAATGGC

AACCCACTCCAGTGTTTTTACCTGGAGAATCCCATGGATAGAGGAGCCTGACAGGCTACAGT
CCATGGGGTCACAAAGAGTCAAACACGACTGAATGACTGAGCACATCCATTCTTCGCCAAA
CCCCACTCCCATCCAGGCTAGCACATAACATTGAACACAAGCTTCATGCGCTATACAATAGG
TTTTTGTTGGTTATCCATTTTAAACACAGCAGTGTGTACATGACCTTCCCAAAGTCCTTAGCT
ATCCCTTCCCCCCAGCAACTGCAAATTCATTTTCTAAGTCTGTGAAGAATCTGCTGCTTATTA
CCTAAATTCAGTTGAGCAACAGAGAAGAGGGAAGGAGTCCAGGACACACTGCCAGACAGA
GGCCAGAGGGAGGGTTACACAGAGAAAGAACGTGCTGCCCTTATACTGCCTCTGGGTCTGG
TAAGGTTAGTACAGCTTAGAATTGAATATGATAAGAAAGCATTATAACTGGTTGGCACCAGT
AGTGTAGATTTTAAAATGAAAGACCTGAGATTGTGAAGAAGAAAAACTTTTTTCACAGAGAT
AGCGTCTTTATTACCATTATCAATGATACCAGTTATTACAACTAGTTGTTTCAATGAGTGCAT
ATATTTAATCTTATTTGAACACCATGGCATTACAAGATAAATATTGTTATCATCCTCATCTTT
TAGAGGAGGAAACTGAAAAACAGAGAAGTCAAGCAACAGGTCCAAAACTATTCGGGATACT
CACTATCAACCCAACCACTTGTCTCCACAATCTGCAATCTTACCCACTCACTCTCTGTCCCAA
AGTTCCTCTGCAGAACCCAGACTATCAATGTGTGTCCCAGAGCACATGAAAAAAGAAAAGA
CCCAACCAACCCTCAAGGATCCTGTCCTGTCTGGCCAGAGCCAATTGATCAACAGACATCAA
GCCAAGCAAGCTTTTACAAGAGAAACCGCCTTTGTGGACAATTGCTAAATTTGGGCTTCACA
AGAAGTCAATAGTTATCAAGTTATTTGCTTGCCAGACCCAGAGCAGTCTGAATTCCCTTTGTT
CCTGCAGCCCAAAGGACTACCCCTTTCCCCATCTCCAGTCTCTCTCTTTCTTTCCTATACTCTA
TCATTCATTTTTCCTGTTTACAAAGATAAAAAAATATAGGTCACAAAAGGTTATGTGTGTGTGC
TAAGTGGCTTCACTAGTGTCTGACTCTGTGCAGCCCCATGCACTGTAGCCTGCCAGGCTCCTC
TGTCCGTGGGATTCTCCGGGCAAGAGTACTGGAGTGGGTTGCCATTTTCTACTCCAGGGAAT
CTTTCCAACCCAGGAATTGAACCCCTGTCTCTTATGTCTCCTGCATTGACAGGCAGGTTCTTT
ACCACGAGTGCCACTTGGGAAGCCCTACCAAAGATTGTAAGATAATTTAAGTTGTAGGCTTA
ACCTTTCAAGTCAGATATACCACCTAAATACTCCCTTTTGCCTTTTCTTAGAAATTTTTACTTG
TCCTTCTAATTTAATTCAGCACTTCCCCAACTGTCTTTATGAAACACTGTTTCAGTAGATGTT
AATAAAGATATCCTAGGGTGACACATGGCCAAATAAGTTTGGGAAATAAGGCATACCAGGA
CCTTTTGTTGGAGCTGTGTTATGAGAATAGTAAGACTGAGAAATCATGAAGTAAGGAAACTG
TTGAATATGACTTTTAATTTAAATATTCTGGGTCCTCTTATGTACTAGGCTCAGTTTTGGTTTT
GTTTTTTTTTTTTTTGTATTGGAGTATGAAAAATGTTTTAAAGTGAGAGGAACAAAAGAGAT
GAGAGGAAAATCCATATTTCTAAAAAGTCAACTTCTATGACATAGTGCTATAATATAGACTA
TTCATATTTTAAGAATGCTTTATGGTAAAAATAATTGAACCAAGAAAGTTTTATTTCAAAAT
ATTTTTCCTCTGTGCTTTCAAATGGAAATAAATTCTCCCCATGTTTATTACTTTTTAAAAATAC
AACTGCATTATTGTTTCTGTCTTATTATGAGTTAGCATTTATACAACCTCAGGGTTCTTATTTC
CTTAGCCTTGAATATAAGTGTTTGCTTTCTCTAACCCCTAACATTCCCGGTAAGATGATATGC
TTTACCTGTAGTAAGAGACATTTGTTGTTGTTCAGTTGCTAAGTCGTGTCTGACTCTGCAACC
CCATGAACTGCAGCATGCCAGGCCTTCCTGTCCTTCACTATCTACCTGAGTTTGCCCAAACCC
GTCTATTGAATCAGTGATGCCATCCAACCATCTCATCCTCTGTCATCCCCTTCTCCTCCTGCC
CTCAATCTTTCCCAGAATCAGGGGTTTTTCCAGTGAATTGGCTCTTTGTATCAGGTGGCCAAA
GTACTGGAGCTTCAGCTTTAGCATCAGTCCTTCCAATTAATATTCAGGGTTGATACCCTTTAC
GATTGACTAGGTTGATCTCTTTGCTGTCCAAGGGACTCTCAAGAGTCTTCTCTAACACCACAG
TTTGAAAGCATCAGTTCACTGGCACTCAGCCTTCTTTATGGTCCAACTCTCACATCCATACCT
GACTACTGAAAAAACCATAACTTTGACTACAGGGACCTATGTTGGTAAAGCAATATTCCTGC
ATTTTAATACACTGTCTAAGTTTGTCACAGGTTTTTAATCCAAGGAGCAAGCATCTTTTAATT
TCATGGCTGCAGTCACCGTCTGGAATGATTTTGGAGTCCAATAAAATAAAATCTATCACTGT
TTCCACTTTTTCCTCATCAATTTGATGTGATGTGACCAAATGCCATGATCTTAGTTTATAAAA
AGTTGAGTTTTAAGCCAGCTTTTTCACTCACCTTTTCCACATTCATCAAGAGGCTCTTTAGTT
CCTCTTCACTTTCTGCCATTAGAGTGGTATCATCTGCATATCTGAGGCTGTTGATATTCTCCT

GGCAGTCTTGATTCAAGCTTGTGATTTATCCAGCCCAGCATTTCTCATGATGTACTGTGCATA
TATGTTAAATAAGCAGGGTGACAATATACAGCCTTGACGTACTCCTTTCCCAATTTTGAACC
AGTTCATTGTTCCATGTCCAGTACTAACTGTTGTTTCTTGTCCTGCATACAGGTTTCTCAGGA
GGCAGGTAGGGTGGTCAGGTATTCCCATCCTTTTAAGAATTTTCCACACTTTGTTGTCATCCA
CACAGTCAAAGACTTTAGTCATAATGAAGCTGATGTGTTTTTTTTTTTTTAATTCCCTTACTTT
TTCTGTGATTCATCAGATGTTTGCAATTTGATCTCTGGTTCCTCTGCCTCTTCTAAACCCAGCT
TGTACATCTGGAAGTTCTTTTTTCATACTGCTGAAGCCGAGCTTGAAGGATTTTGAGCATAAT
CTTGCTAGCATGTGAAAAGATGCAATTATACAGTAATTTGAACATTCTTTGGCATTGCCTTTC
TTTGGAACTGGAATAAAAACTGAACTTTTCCAGTCCTGTGGTCACTGCTGAGTTCTGCAGATT
TGCTGGCATATTGAGTGCAGCACTTTAATAGCATCATCCTTTAGGATTTGAAATACTTCAGCT
GGAATGCTATCACCTTCTCTAGCTTTGTTTGTAGTGATTCTTCTTAAGGCCCACTCAACTTCA
CACTCTAGGATGTATGGCTCTAGGTGAGTGACCACACCATTGTAATTACCTGGGTCATTAGA
AACGTTTTCGTACAGTTCTTCTGTATATTCTTTCCACCTCTTCTTAATCTCTTCTGCTTCTGTTA
GGTCCTTGCTGTTTCTATCCTTTGTGCCCATCTGTTCATGAAATGTTCCCTTGGATCTGGTAAG
AGACACAGAAATATACTAATATATTTTTAAAATTTATATTCCTTATGCAACAGGAAATCTGT
ATCTTAAACACTTGCCCCTTGATACTTAGTCTAATATTCATTTTGAAAATCACTGAAATCCTT
CCAAAGATATATATATATACACATATATATCTTATAAACTTAAATAAATTTAAATGAGCTTTC
TAAAATCTATACAGGCACCAAATAGTAACATAACAAAACACTTGTAAAAGGCATCCAGTAT
GGTTTTCTTTTCTCAATATTAAGTAAAAATTTTTGTGCTTTGAAGCAGAATTTTTAGTTCTCGT
GCAATAGAAGTTGTAACTTTTGGCAGTTTAAATAAAAATTTATTTTAAATTCTAAGGTGAAA
CTACATATTGTTCCCTAACAGTGCCTAGGAAAAGTAGACACTCAATTAATTTGAAATTAATT
TCTTTAATCTCATATGTTTTTTGCCACAGCAGCTTTTCATATTTTCTTCATTGGAGCCACTATC
TTCTGGCAGAAACTGCCATATCTTATGAAAGATGGAGAAATATTCTCTGCCTTTATGTTAGA
CAACGTGTTCTTTGTGTTAGTCAGTACTTGGTAACTGGCCTCTGGACTCATGCTTGAAGAAGA
AGGTTCAAGGTTAGGTTCTCCTTGAAGCTTCCCCTTCTAAAAGCTACCCTGTCTGTAGGTTTC
CCACGGTACCATAAACCTTCCATTAGACCATCTATTGTATGATTTCATAATAATTTTTCTATG
TGTCTGCCATTCCATGAAGCTAGAGTTTTATTTTCATAATTTCTGGACCCAGGAATATCTGGT
GGCAAGGAGGAATAGTTTCTTCAGTAATTCTGTGAACCAATGAGTTTTTAACTATAATTTGT
ATATGAAAATAAATAATTCTGAAACATCAGAGTAGCATAGCACAATGCATCTGCTGAGAGA
TTTATACTGACACAAGGTAAGGATTTAGATAAGGGCAAAAACTGCCTTTCATGATCATGCAT
CCTTATGTCTTCTGACTAATCTCTGATATTACTTGTCACCTGGATGCATAAACCTAAGGTGAT
ACATTTCATGCAAATGCTGGAAATCTTAGCATTTATCTAATTTTTCTAATTTCTCTGTCAGTT
GTCTCCTCTATAGGAATGTAGGCTAACCATACCTAATTACAGAATTTTCCATAGATTTAAATA
ATAACATGGAAGTATTTAGCCTGAAAACTGATCCATAATAATATGTCAACAAATACTTGTCC
TCCCCCTTATCCTTCCCTAGAACTTTTCAAGTTGTCTTTATAAGGAGGAATAAATAGTATTGT
TTTTATATTTAATAATTTTATTTAGATAATTTTAAAAGCATATTTAAAATAAAATGTAAAAAG
GCAAGAGCACTGGAACCAGAATAATTTAGAGAAAACTAACCTCATTCTAGTAACAGGGTAC
AGTATATGGGAATCAAAGACGTATAAACTTTTCCAATTACCCAGGAAAGTAATATTAAATTG
ACTAGCACTTCTGAAAGTATAATGACATACAAACAATATTAAAGTAATACACTAGCAAATAT
CAGGCCAGATCCTGGGAGAAGATATGTGAAAAGAGGTGAAATATACAAAATAAAAATAAA
CAAAAAGAAACATCAGGAGAAAAAGAGAGCTATATTTTAGCTGAGAGGCTTTGTTTTTTTTA
ACTACTCTTAGGGCAATGTAAGATGAGCATTTCCTCAGATCCCTGAAACTTTATCTGTCAAAT
GATCAAGATTGATGATTCTTAAATTTTTTTCACATTTTGTAATATAAAGTCACGAGCAAGAG
AGAAAAAATGTGTCATACATGGATGTGGGTGAAAGGTCTTTGAACTAAGAGTCACAGCTATT
TATAACTGAGTAAATGTGGATGTCTCGTGAGCCCAGAGCCTTGTGGTTGTCTACTCTAGAAT
CTTTGTGTGTAACTGTCCCCTGTGTCTAAGCTACATCTCTAGAAGATGAGCTCCTTAAGGTAA
GTGCTGCTAAACTATCCAAGCCAATTTGGGCACACACTGTTACTGGGTTATCTTGGTAAATG

TTGGTAGTTTTCAATAAATAACAGTGGGGAGAGCTACAGATCTTTCTCAAAACAGGTACAAA
GAAAGCCCTCGCAGTACTGGCTTCTAACAGAAGCAGGGAAGTAGCAGTTAAGTAGCAAGGT
TCCCCAACCTGTTCCATGGGATTTGTGAAGTACCCACTCTCTGTCCTTTTAAGTGTCCCAGTA
GTTCTTGCCTGGAAGTTTCATCAGGGGCACACACAACATGCTAACTATAGAATTCAGCTAAC
TATCCAATCCCAGTCAGGCCACCAGGAATGCCCTGAAATCAAGAGCTAGACAATGAACTTG
GTGGGTTTTTATAGCCTGTGATATAAGGATAGTTAGCCAGTAATTTTTCTGGTCAGTTCATAA
ATCTTTGAAGCCATTAATTTCACCAGTGAAAACCTCTCTGTTATATTTATTGTGAAAATTTCC
AGAGTTTAGGAGAAAAGACAACTAAAATACATATTTTGATTATATGCTAAGTAAACAGTTCT
AGAATGAATATTTTCAACTAGGTTAGCTGTCCACTTAGATTAAAAGTATTCAATACTTCATCA
TTGCATTTTATTTGCAAAGTTTTAAAGTACATTACAAAAAATCTCACTGGAAGGATCAGGCT
TTCTTCTTCCTGATGACAGGATTCTAAGAACACAGTGGACACATAGCAGATTTTCATTAAGT
ATGCGTGTGGGTGTGTGTATGAGGATACATGTCATCAACTTTAATCCTTAGAGAATTAATGC
CTTAAGTACTATCAAGAAAATCATATTTTTTAATCTAATTTAGCATATACTGTAATGGCTTG
ATTTGCTTTTAGAATATATTTATCTTAGTTTCTTCATTCTTATGCGTTGCATATATAATAGCAT
AGTCACCAAACATTGCTAATCTTACATGGCAAAAGAATGTCATAACTAGAATAAACTTGAGC
TTGTCCATTTTGCAAAGCCCTGCCCAAGGCTGCTATCATCTCCAGCTTTCCACAAACTAGAAT
ATTCACTGATGCCTTGTTTCTTATCCTGAAGGCCAGTGTGCAATGTTGTTATGTGGTTCCTGT
GAAGACTATAAAGTCCTACACAGAGACAAAGGATGAAAACAGTCAAGGGAATTGCTATAAG
AAAATAAGTAGGGATATCTCTGGAGAGGGAGTTGCTTCTGTGTTCCTTTAAGCATCACAGAG
TAATTGTTTCTTTTTTAAGGAAAGAACCAGGAGAAGAGAAGCTCATTTTCAAGCTAGAGAGC
TTGGCAAACACAAGCTGCAGGGTTTACTTTGGTTTAGGAATCCCACTGCCTCACTGGAGTGT
CAATCAAAATTCCACAGCCATTCTTGGATACTTGGAGGTGGGGCTGAGTGATGTACAGCAGT
GATTTGACCCAGCTCTTGAGGCAGACACAACAAGAGTCATCAGTGTTAATTTCGTTGTCCTC
CAACCAGCGTTCTGAAAGTGCGTTGACACTGGTGGCCTTGCACTTTTTCATCGGACTTCCAAC
TGGTAATTACACCTATATTATCACTGTGAAGTTATCTAACCTAACAAAGTAGGACTCAAAAC
CAGTGCCTGTAATACTAAAGTCTCTTACTTTGATCTGATTTTTGCATTGTTCTTGTCCAGTTTA
AGTTGTATGTGCATTTTTCCTCTTGTCCTAATTCATAGAGTATTCGAGATAGTATAGATATTA
AGGAAGCCTGTCCCAAGCTTTTAGATAAGAAATTGTGTCTAAAAGAAAACAGAAGGAGTTG
GTCAGGTTTTGTAATTTTGCTATGATGTTATCAGTAATAACTAAATATTAGATAAGAGGACA
TTCAAAAATATGTGAAATTGTCTAAACCTTCCAACACATCTATGCTTGTATACATGCTGCAAT
ATTGCTAGTAGAGCAATATATTAAAAGATCAAAAGCTATCTTTTATTCTAAAGAAAATACAT
GCCCAGGAAGACTTAAATTATAGATGGCCACTTTTACCTGAAGAGGCAATTATAATGAATAT
TATTGTATAGGTTTGGGACTCTCATATTAAGATTGTAAGGCAGAGCAGTTGTTCTTTTCAGTC
TTGGAGAAAGAAGAATCTATCCTCCCCCATGGACAGGGAAGCCTGGTGTGCTGCAGTCCATG
GGGTAGCAAAGAGTCGGACATGACTTGGACACTGAACACCACCAACCAGCAGAGGTCACAG
GCACTTTGTACTGGGACATTCCTGGAATGGATCTAGTCCATTAAGGGTTACTGTGACTTGCTA
GACAGGTGACTTTGGAATTTTATTTTATACTCAAGTTAGGGAAACTCAGTCATCATACTGTAT
ACCTGACTTGGGCTACTGAATCTTTAATGCAATAGCTAACAGGATATAAGCAGTAACATTAT
AAAATTTGCCATTAAATTAAAGCCAATTTTCTCTTTGCAAATCATTGTCACGAGGTTTCTCAT
GGCAAGAACAAACTACATAAAATGACAACGGGAGGCAGAGAAAACCAGGTATAAGAATTA
GGTAAAATTTTCTAGATTCCAGACTTAATCATTTTATTGGTCACATGTAATCATTTCTATTAA
TGACATTTTATTTCAGCCTATATTATGATACAGTGTTTTGTAAGGACAGACAGCATTGGCCTA
ATTTTATTTCATTGTGATTATCTTAAATAGCTGGAAACTGTAAATTAAAATTGGATGCAACTG
TTTGCCTCAGTGAGTACTGTGTCAAGACAGCAGACTCAAGGAAGTGTTTAACATAGAAAAG
GACAGGAAATGTTAAAAGATTATTCAAGGGACAATTCACAACTTGGCTTGCATCTTAGATGG
ATACAATTCTAATTGACATTTTGGTAGTTAATATTGTTACATAAGTATTCCAGACAATCCATA
ATTAAATATTTTGAATTAAACTCACAAATAACTGCAGACTTACAATTATGTATGTTACCTAA

ATGTAAATGATTACAAAATTAAAAAAACTGAGATGCATGCTTTATAGTAATGCTACTAAACA
GATATATTTAATATATATATAAAGTTAAGGACTTTATGAATGCCAGCATTATGTAAATCACTT
TGGAGGGTTGGATTATGTGTTGAATCTTGTTTATTCACTCAACACACATTTGTTTGCCCACAA
CAAACTAGGTATTGATCATTTCCCGTTCCTAATTTCATCTACTTAACACTTTGGGTAATCAAT
TAATTCTCTAGTATGAACATCATTATTTAATAAATTAAATCTATCAATACAGGGGACGTTGTA
TAAAGGTCACTGCTCTACTGGATAATTGGGGAAACTTCCACTGAGATTGGTACCACAGTTTC
TCAGATTTTTCCCTTAAGGATGATGAAAGATCAGTGGACTCCTAAGTATCTCAAGTCTGTGG
AGTCTTAGGTTGCTTAGAAACCCGGTGACTATGGGCTTGCTATAGCAACCCAACTTATCAGT
GAATGCAAAACTAACTTACCTGGCATTTTGCCAAATCTCCATATTTTTCTAGTATGAATATTC
CGATCACATGACTGTTTATTATTCACACAATCAAGCTAAATACTACTACAGTTTACCAAATTT
TGAAAAGAATAACTTACAATAGCTAGCAAATATAAGAAAATTAAGCTTAAGCTCACTATGT
AATTTTAGTTACTTGAAATTTGTTATGTCACCTAGTTATTATCCCATTGCCTACATCTGATTAT
ATATATGGAAGATTTGCACTAAATTAGAAGCATGGGAAAGTTAGGCTAGTACAGTGTATAAT
CTATGTGATAATAAAAAGGCTCTATCAATGACTTCTTTAATATTTATAAATCCGTAAATTTAT
CCATACATTTATATGAGGTGCATTTTAATGATATTCTTATTTTACATTATAAACCCCCTTTTCA
CATATAAATATATTTCTTTAAAATTATTTACCACATTCTCTTTAAAATCTTAAGCCTTCAGTA
ATATGCTGTGTAATCTGTATACACACATGTATTAGAAAATTAATTTATTTGAAAAGCACATTT
TTCCAATAATGGAAAAGATGGGAATGTAATCTCATCGTCAGTCTAAGTATTTGCTCTTTGTA
GTATATTTTTAACTATTTGCCCCCTTTGATTTAAGGCATGTGTTGCGATTTTAATAGTATCTTC
AGTTTATCAGTTTTTACATTGTTGTTCAACAATAGCGATCTCTGTACCTCTTCATATGTAGGA
ACTGTATTTTTCTTATCTCCTGTTGATTTCGACTCTCATGTTTATCACAGCCAATAGCACAGGT
AGACCCAGCAGCCTCTCCTGAGACTAGAGTCCTATAAGGTTTTAGTTACTGTCCTTTTCTCCT
GGGTTTTGGCATTTATGCATTTTGTTGATATTTAATAAGCGTTTGTCATATTAAAAATTCTCTT
GAGAATAAAAGAGTCAGTGTGACTTAGGTTTTTATGGGATAAGGATGCATTTGCTGCTATTT
TCAATTTTTCTTTACACTTTATTTCTACAGAATTGTGCAATTGATAAAATTTTTGACCCCTCTA
GTATATCAGTTTAACTGGGTAGTGCCGATGTTAATGATGATACATCTCATCTCACAGATGGA
AACAAGTAGGAAGACAAAAATTGGGCTATTTTAGTTAAATTGTCATCAGAACAGAAAAGCC
AGTTCTCAGAGGTCCTTGTTTAAGGGTCCAATGATTGGGCTACCCTGGCACCCTCAACAGAG
GTGCATTTGTGCAATCGGATATTTTTTAACAAAATAAGTGAACAGTCCAGTACAAATGGACT
AAATAACCCAAAGGGATCAAACTATTAGCAAGGGATCACACGTTAGCTTATAAATAGATAG
TAGATTGGCATGTAAAATTAGATGGTAGAGTATAATACTCAGGAGACTTAGAATTTTTGATT
GGCAAAAAAACCAATACAAGGAATGATCTATATTTAATCATTATTTTTGGCCTATGATTGAA
TTATTTTCATAAATTTTTGAAATCTTCACTTTAATCAATATGTCTGTTGCTGGCAGTGAAGAT
ATAAACATAATTGGCACATTTGACCATTTAAAATATTTGAGAAAAGCTTATCGTGTAGTGGT
ATGTTGTTTGTCCTTGTACTGCAGTTACTCAATACATATCTGTGGGACAATAGAAGTAAGCCC
TCCTTGAGAATAAATATTTTTCAGTGTGGAAGAACCTGACTGTTTAATAATTCAGATAACCA
GTCCTTTTAAATTCTGTGTTGGATATCTTGGAAAAGTAAATATATTCTGCCAAAGTATTAACA
TACCCATGTCTCTTTAATGTATGTTGTGATACTTGTAGTATAGGTTCTTCAGTATTTATTTGAT
GAGTATCTGAATACCTATTATGTGTAAGGAACTATTTTAAAATCTGGCATATAAAAGTAAAT
GTTCCCAACACAGTTCTCAGTGAATATATTATAGAGAGTTCCACTGTGACCTCTCCCTTGGAA
GTCATAAATGAGTATATAATGAACTGAGCTTTTTTGTTATACTTTGTATGAGTAGGACACTAA
AGCATTCTGGAGATATAAAGATGAATAAATATAGTCACGTGGCTTTTTGTATGTGCTTAATA
TTAAGATCTCTGCTTGTTGCACATGGTATACAAAACCATGTTGTAGGGACTGTTTATTTCCTT
TTTTTTAGGGACCTATTGATTAAACCACATTTCTTGATGATAGTTAACGAGGAGAAATTTGGT
TAGATGTTTAGTTATTTAAAATGTTTGTCATCTACAATTCACAATTTACATAGAATCTCAGGA
TATGAGTTTGAGTGTTTTCAACTCAAACCATTGTATCAATTCAGGGATTTAGGGTACACCTTA
ACTAAAAAACTTCAGAGTTAGCACTCTGTAGGCATAATTAGATCAAACTGTTGGTTCTTTTG

AACTTATTTGCTCTTGCCTGTTATTTGGGTGTTGGCTTTCACACTAGTTTCCTTGAAGTTCTAA
GATGGTTCCTGGCAGCATTCGTACATGTTTCCTTATTACAAATAAATAGGAAGATGGGGAGA
GATAATAGTGAAAAGATCATTCCCACCCATACTGAACAAAAGAGCAAAGCTTTTGTGAGTCC
TGAGATGTATGTTTTTATAACACTGAAGTGCTTATAACGCTTTCAGTTCAGTTCAGTTCAGTC
GCTCAGTCGTGTCCGACTCTGCGACCCCATGAATCACAGCACGCCAGGCCTCCCTGTCCATC
ACCATCTCCCGGAGTTCACTCAGACTCGCGTCCATCGAGTCCGTGATGCCATCCAGCCATCT
CATCCGCTGTTGTCCCCTTCTCCTCCTGTCCCCAATCCCTCCCAGCATCAGAGTATAATGCTT
TAACTTATAACAATTAAATAGGGATTTAATAAAAAAAAATATTAAATAGAACATTGACATTT
TTAGAGGCAATATCATGTTCAGTTCAGTCACTCAGTCATGTCCAACTCTTTGTGACCCCATGG
AAATATCATGTTAGTACCATGACAATACATGTTTTATAAAACAAAATATGGAGGAAACTTTT
GAATCTTTTCTTCTGTATTCAGTGTTGTGAGTCATCTCCGAAAACAAAGTCAGTTGCTCATGG
TTTCTAGAACATCAGTTACTTCTAACTACATAGGATATACTAATGATATGCAGAGTACATCA
TGAGAAACGCTGGACTGGAAGAAACACAAGCTGGAATCAAGATTGCCGGCAGAAATATCAA
TAACCTCAGATAGACACCACCCTTATGGCAGAAAGTGAAGAGGAACTAAAAAGCCTCTTGA
TGAAAGTAAAAGAAGAGAGTGAAAAAGTTGGCTTAAAGCTCAACATTCAGAAAACGAAGAT
CATGTCATCTAGTCCCATCACTCCATGGGAAATAGATGGAGAAACAGTGGAAACAGTGTCA
GACTTTATTTTTTGGGGCTCCAAAATCACTGCAGATGGTGACTGCAGCCATGAAATTAAAAG
ACGCTTACTCCTTGGAAGAAAAGTTATGACCAACCTAGATAGCATATTCAAAAGCAGAGAC
ATTACTTTGCCGACTAAGGTCCGTCTAGTCAAGGCTATGGTTTTTCCAGTAGTCATGTATGGA
TGTGGGAGTTGGACTGTGAAGAAGGTTGAGCGCAGAAGAATTGATGCTTTTGAACTGTGGTG
TTGGAGAAGACTCTTGAGAGTCCCTTGGACTACAAGGAGATCCAACCAGTCCATTCTGAAGG
AGATCAACCCTGGGATTTCTTTGGAAGAAATGATGTTAAACCTGAAACTCCAGTACTTTGGC
CACGTCATGTGAAGAGTTGACTCACTGGAAAAGACTTTGATTTGGGGAGGGATTGGGGCAG
GAGAAGAAGGGGACGACTGAGGATGAGATGGCTGGATGGCATCACTGACTCGATGGACGCG
AGTCTGAGTGAACTCTGGGAGATGGTGACGGACAGGGAGGCCTGGCATGTTGTGATTCATG
GGGTCGCAAAGAGTCGGACACGACTGAGCGACTGAACTGAACTGAATGATCTGTTAGGCTA
AGATAAGTTCTTGAAGAAACATTATGATCTTCCTTTCCTCTGGGAAATTCTTAAACATGTGGA
AATATCCCTTAAAGTTTAGATTTAATCAAAAGGCTGAATTTAGTAATATTTAATCATTATGTT
TCATCTTTAAAACTGAAGCCAGTTCCCTAAAACATATCTCAACACATATGCATTGGAATTTTT
AAAAAACTATGTTGTCTATTTTCAATTTTAATTTTAATCACTTGATATTGGATGAAGATTATC
TGAAAGGATGATATTTTCAATGGTGGAATACTTTATCACAAAAAGAAAAGGTTTCCCTGTAA
TTAGTTTGCACCAAAACTTACAAAGAAAATCACTACAGTATTTTTTTCCCTAACCTACGTGAC
ATGGCAAATGAGCCAGAGAAATTTCAGTAAATAGCACAAATTTAGTTTAATCTTTTATTTTG
TTTTAATCTTTATTTTGTTTCAGAGCTTAAAGAATTTAAATGTTTTCTACTATACAGGATGGT
AAGGTGTGTGTGCATAATCGTGTCTGACTCTTTGCGACCCCATGGACTGTAGCCTGCCAGGC
TCCTCTGTCCATGGAATTTCCCAGGCAGGAAAACTGGAGTGGGTTGCCATTTCCTACTCCAA
CAGGATGGTAATATGAGATTAAAAGAATAAATGGCAGAATATGGTTCAGTCATAACCAAAA
TATTTTTGGTAATATATGGAAATCTGTGATTGTGTACTGAACTACCACTGGAGGGTGGATGTT
TTTCACAGCAAAACTACCAGTTAAGTGGGTAATGTGAACATATCAATAAAAGGTGTGAAGG
GACAAATGAATTTATGGTTTCTGGTAATTTAGGAATAGCTGTGATTTTATAATTGTAGTAAGT
TAATTTGCATGCAGTTCCAAATTTACATACCTAAAACCACACTGAATTTTGAAAAACTATTA
GATAAAAAATGAGATGTCAAATAAAAATCAACTAGGATAGATAAACGCAAGGTAACCAAG
GAAGAAAAAAAAGAAAATAATGAAAACTGGGAAGCTTGTGGAGGAGAGAAATCTATCCTG
AAACATAAAAATAGTATTGGGGGACAGGCTTCTTTATAACATGGGAAATATTTAGTGGCTTT
TAATTTTGAGTTGCATGAAATGGTATGGTTTCCATGGGCTTATTTCACCTCCAAATATTACTT
TAATGTAGTCCTCTTTTCTACAACCTACTGTGATTATCCAGTTTTCCCTGAGGGGTTGCAGAA
AATGAGAGGAAAAACAGGCCTGCAACAGTCTTGGAACCTGTCCAGAAAATGTTTTTAGTCCT

TGGACTACTTGAAGGACTGTGCTCCAAACCTTGTATACACAGATTGGAAAACAATGATAATC
CCAGTGTATTCACTCTGAATAGGAATTTTCTGTGTGTCATTAACAAAAGGAATATTACAGAA
TACACTGAAACACACTGGGATACCATAGGATCTTGATTAACATTTTTACCTTTATCCATGAAC
AGGAGGTATTGTGGAGTAGCAGAGTTTGCTTACTAAATACCACAACTGAATGTCCTGAAAGC
CAGGAAAACAGTTGTTAGAACAATCTCTGAGGCTACATGTATCAAGCAGTGCAGAACTAGC
AGGGCCAGTAGAGTCATGTGAGGGTGACATTCCACAGCAATTCAGATTCCGTAACTTGCTGT
TTACTTAAATCACAACCTCCTAACAAAGTGAAGTTCGGGGATCTGTTAAGAGGATGCACGAT
TTGAGTCCTTGGTTGCCTTTTGGAGAACACTGAGGATGACAGACACTTTCATAAAGACAAGG
CTGATGTTACCTAAACCAACTTAAGAATTGGAAGATTTCCTTCTACTTAACCTAAGGTAAATT
CCTTTATCCAAGCCATTGTCAACGCCCCCTAATGTGAAGCAAACTTTAATGTGGTTACTAAC
ACGTGAGTGCTAAAATGAAGTTTAATAGGTAAGTTGAACTTCTCTTTTCCTTAAAATTTACTA
AATTTTGTGCAGATACTAAAGAGAAAGTGTTACTATACTCCTGAGTAGTTGTCATTTGCTTCA
TTTAAGCAGCTAACCAAATGTGTCACGGAGAAACATGCAGGACAATTGCAGGGTTCTGCTA
ATTTAGAATTCTGAATTTTTATGAAATCTACCTTATGCATTAAACAGTACTAGTCTTCCATTT
TTATTCTGTTTAGGTTTTCATGTTTATGATTTACAGAATACAGTCATGAAAATAAGATATATA
GAAAGAATTTTAAAGATTTATAACATTTCCAGGAAAATATTTGCTTCCAGAAGTTTTATTAC
ATAGATAGATATACACTCTATATTTGGGTGTGCATATATGTTACAAGAATTGGTTTGTTTTTA
CAAGCTATTATATGAGGCTTTGTTCAAAATGAAAAATACAATTAGAGTAAGATGTTCCAACT
ATGAATACTTATGGATTCTTCGAAATCTTAATACATTTCAAGTTCTTCGAAGAAGTGTATTTG
AGAAGTAGCACTCTAATCCAAACTCTTTGGCCTCTTGAAAGATAGAAAACTCAGGCTTGACC
ACAAGCAGATCTGCTCCTTAAGTCTATGAGAACAAATCTGGGGAAATGATGATTTGATTTGA
TTGTGATGGCCTTCCCTGGTGGCTCAGATGGTAAAGAATCTGCCTGCAATTCTGGAGACCTG
GGTTGGGGAGATCCCCTGGAGAAGGGAATGGCCACCCACTTCAGAATTCTTGCCTGGAGAA
TCCCATGGTGGAGGAGTCTGGCAGGCTACAGTCTAGGGGTCACAAAGAGTTAGACATGACT
GAGCGACTAACATTTTCACTTTAGGGTTTGATAGACTGTTTGATACTGCTGTGCCACTCGAAT
GCAAGGTTTGTACCAAATGTTTTTTAAAAGTGTCAGGGGAGAAGGATCAAACTTTTCCATTT
CAAGGAAACAGAAAAAGAAAGGCATTAGGAATGTATTATTTTCTATGTGGTGATTTCTAAAA
TTATTATTACATGACATGCTTCTTAGAAAGTATTTTCTCAGCTTTAAAAAGGAATTTGATTAA
GCTTCCCTGTCCCTGGAAACTGTGTGTGTGCGTGTGCCCCCATGTGTGTATGCATGTGTATGA
GTTTAACTCTTTCAGGAATCTGTCACCCAAGTGTTGCATATCTCCAAATTTAACAGATTATCA
GTTGGCACAGACAACAAAATTGTTAAAAAGACGGACTAGAGTATAGTTGTCTCGTTTTTAGG
AAATGGAACAGAATTACTGTTCTCATTATCCATCTGATTTCTTTCCTGGTTTTAAACATTCAT
CATGCTCTTCAAGTTTTTTTTTTTTTTCTGCTGGTAGTTAAAAAGAGTGAAAAACATCCTTTA
GCATTTTAAAGCATATCATTTTGATCAAGCCATCAACCAATGTGAATGTGTAAATTGATTCTC
TAACTCTTCTTAAAATTATTTTATTTCCTTTTTAAGACCCTACAGGGACTATTTATAACGTAG
ATGGCATGCCTCAAAAGAAAACTTTGTTGTACCCTCAATTCAAAGAATTTAGTATTTCTGCCC
CTTGAAATTACAAATTAATTGCTCACATAATTTTTTCAATCTTTTTTGACATTGTTTAACTAAC
TAAATCTATATTTTGTTATTGATTATTGATTTTCCCCCTACTGTTTCATAACACCAAGCTTTGA
AGCCAAGCTATCCTCTTACTTGAAGATTTCTGTTGTTCCCTCCTAATTATTTCCCTTCCACTGT
ATGAACACAAATTGTACTGACAACTGTGCAAATCTTAACACTTTCTTAAGTTTCTTAGTATTG
TTTAAATGTTGACATTTCTCCACCTCCTCAGAGAGTTAAGTCAGCTTTGCAAACAGTTCAAAT
AAAGATTAGATCTGCCAACATTTTTAGGCCTGTTGAATTTACATATTTATATGGCTCTGCCCA
TATAAGATCTATCCAGATATAGAGAGGTTCTCTCTTTATACACGGCTCCTACCATGGCTTCTA
GGAAACACAGGGGAAAGTTTGCATCATGAATGCAGTAGGTTTGTCAATACTTTGATCGTGTG
CTTTGATTCATTAGATGATGTAGCTTAAGGCTAAACTTTAATGACCCAGATATTCTGCATTAT
CCTTAATGTTGTCCTTTGAAATTTTCCTTTAAAAGCGGTATTAAAAACTTCCATTTTAATCTCT
TAAGCTTTCACTGACATTATCATGTCTATGTCTTAATGCATGTACTAGTCTCTTTAAATTATGT

ACGTATGTATTTCATTCCGGTATGTGAAGCCCCACCTCATCATATGAAACAGATAGAACCTG
TCATTAGTTTAATACTTCCATAATGACCAGGGCAGCATTTGGAAAAATAGATATCTATTTTTG
TAGCACACATAATCATGTACATCATATGTACATAAATATGTGTGTGTGTAAATCTCCATGGTT
AGTTATCCCAAGGATTAAAGTGATAAATCAAATTTTGCCTTATGGTTTTACACTGGTTAACTA
AAGTTTTAAGATACTTTACAGTGTATCTGTGTAGTATTATGTGTTATAGGTCTTCAGAATGAA
TTTTATTTTTTGAGTTAATTTCAAAATAATTCTGTTAACTGTTAATTACAGAATACTTTCACTG
TTCATTCCTTGTTTATTGTACTTAGATATCATTTTCTTATATTAGGCTATTTAAAATTGTTATT
CAGATTGAACAAATGTGCTAAAAAGAATATGCAAATTAGGTATTGATCAAGAACTCATATAT
ATATATATATATATACACATACACATTTTTCTTTCAGAAAGACTATAGACTCTTAGTCAGTCT
ATATGGGTTTTTATTATTATGGCACATAGTAGCTGTGTGACCTCAACTAGTTGTCTAAAAACA
TAGCAAGGTTGTATGATAGGTCAATAAGATAACTTATGTGAGACACTTATTTCAACAACTGG
CAGATAAGCCCTCAACACATGTTAACTCTGTTATTGCATCATCATCATAATTTATAGGTCTTT
GCTTAACCATACAATCTAGAATTTTAATGCACATGATACTAAAATTCTTTTCATTTGCTGAAG
TGTTAAAAAAAAAGAGGGTCAATTAAAAGTTTTTATCTACTACATAGAATCTCTATAAAGCT
TTACAAATCTGTCGTGTGACTTGTCAGCTTGAGGCTGTAGGTAGGTTCTGTAGTTACTCTCTT
TGACAAGACAGGAAATGAGAAATATGACTGTGGGCTGCTTATTCACAAAACCTATGATTGTT
ATTGTTATTAATAGGGCTGTGGTCAGAACTTGGGCCATGCCATGCAATTCCTCTTCTACTATG
CTTCCATTGTATGCTTCTCAGATATGTTGCAATATTTATAATTACTCAATGTACTTCACTCTTC
CTCTTTTACTTCACAAGTGTTATATGCCATGAATTAGCTAATCTAGAAATATATTCTAAAGGA
TTTTTGAAACCATAGAAATGCTAGAATAAAGTTTATTATTTTTCCAATGTTTGGAGAAAGCAT
TTCAAAGCATGACATAATACTTAAAAGGGAATTACTGTGAAAGTTAATGGCATAAAAATGG
AAATTTTCAGTAATTTAAAAATTCAATAAAGTTTAGTTAAAAACTTTTAAAAAAAATTGTCAA
ATGTCAGAAGACTGGTTATTTCCTTATTAGCTAATAAGGAAAAGATATCACAAATGTTTAAA
AATATAAATGCTAATATGCTTTAGACAATTGGTGAAACTTCACTCAAATATTTAACAATAAA
ATAAGTACTTATAAACGGAGTTTAGTATGAGCCAGTGTGACCAATGTATGAAGATGGGCCTT
TTCACACATTGCTGTATACATGCATGAACAGAAATTGGGTAAAATTCCCCTTGCCAAAACAA
GTACATACTTCTCACCCAGAAATTTCACTTTCTGCCATTTACTAGTGTCTTAGCACAGTAAAC
AAAATTATATATGTACATAAATTTTTAGCATTGCTTTTGTAAGATCAAAGAACAATTTGGTTA
GCCAAAAAGTCTGTTCAGGTTTTTCCGTTATGTGTGTATGTTCAGTCATGTCCAACTCTTTGC
AACCCCATGGACTGTAGCCTGCCAGGCTCCTCTGTCCATGGGATTCTCCATGCAAGAACACT
GGATTGGATTGCTATTTCCTCCTCCAGGGGACCTTTCTGACACAGGGATTGAATCTGTGTCTC
CGATGTCTCCTATAGTGGCAGGCAGATTCTTTACTCTGAGCCACCAGGGAAGAAAGGTGTAA
CAGAATGGAAAAAGAAAAATCAAAAGTTCTATTACATATGATATTGGTCATTTAAAAGAAA
ATGGTACATTTTTAGGACTATTTTTTCTAATAATTGAGAAAGAAAAAAGAGAGTTAACACTA
TCAATGATATGAAAATATCTCCAAGTCCCTTGATATTAAAGAGACAAGTTGTAGAAAGTGTG
ATTCAGCATATGGTTTAGTCCATATGTGTATGTGTGTGTGTGTATATGTTATAAACTTTTAAG
TTTATACTTAGGAAATCAGTAAAATGATGACTGTATGCTGTGTGTTTATAAGTTTATAACATA
CAGAAGTATATATATATATAAGTTTATATATATATATATATGTAAGTTTAAAATTAATAAAATTC
CTAGGTGGTAGTGACATACCTAATATTGGTCACTTCTGAGGAATAAAATTGATGCTTGAATG
AGACTTCTCATTTTACACCTTTTTATAATATTCAATATTTTTTACTTGTCCATATTACCTTAAT
GAATTCCCTAAAATAAAAATTTGCTTAATAGAAAATGATCACCATACCAATTATTTATTAAA
CAAATACTATATATGTCAAGAACTGCACTGAGTTCATCACATTACTACTCTCATCTTTGCAAC
AATTCTGAGAGGGAGCTATTTCCACTATGACTTACAGAGGTAAGGTAGTAGCAACAAGTCAC
ACACTCTAGGAAGGGATGAACTCCAAATTCTGAGTTAGGTCCATTTACTTCCATAGCATATG
CCCTTCATCTCAAAGCACTGTATATCTGTGGTATGAACAAACAGTATAATAACCTCTTATTTC
TGGCCATTCTCATTTCTTTCAAAAATATTATTGTGGTTATGACAATCATGTGCATCCAGTCAT
TCAACTGATTACCAGCTATGACCCTGCTCTGTGCTCCTGGGGCTTCAGCAGTGCAATAAACA

GACAAGAGTCTCTCATTCCATGCAGTCAACATTCTAGCAAATGCAGAGAAAGGACCAAAAA
AAAAAAAAAAGCCAAATGCTTGTCTGATGGTGCTGTGTGATCTCCAGTAATCAGAGCAGAA
GGGAGAAAGGGACAATGACTTGAGGGGAAGGGAAGTGCTTCATTGCCTCACAGTCAGGCCC
CTGTCAAGTGCTGCACACAATCCACCTCAGTTAGTCCTTATTATGATCTCTTAGTTTTCTCCA
TTTTTTGATTAAGAAAGTAAGAACAAATTGTTAGTTAACTCCTAATCCCCAGTCAATATAGCT
AGTGGTAGTACATTGCAAGTCGCTGTAGTCTGTCTGATTCCAAATTCTAACTGATCTTCCAGG
TAAAAAGTTTGCAAAGCACTGTGCCTAAGCTTACTTAGCATACTGACAGTTTGAGCTTTCATT
CTGCTACATCTGTTCTAGTGGACTTACAGAAGGTAATGTATACGCTCATATAAAAATATCCC
TTAAAAATACTGCACATACATAATCTTTTAAAAATGAAAACCCCAACATGCATTACTGAGAA
ATGGGTGCAGCAGTTTCCTGCCTTGTTGTTCAAGGTGAACATTTTGCTCAAAATACAGACAT
ATGATTGCAGCTTTTACTTGGCTCTGGGTTTCCTGTGTTTTCCTGTATCAGGAACTGTTGTGC
ACAGTATTAAAACAGTTAACAGCTGCCTCTGGGAATGCAAATTTGATTCTTTGGGCTGCTCA
TGGGAAAGTGTGACATTTTCCTCTCCAAACACAAAGAATTTAGATGTCATTAGACTACTGAT
TAAATAGTAATCCAAGTAAATTCAGTGTCAGCTTAAGTATCAATTTTACAGGTATTTTTTCTG
AAATATCTTACTCTAACAATGTTCATTTTTTATATAATTTAATTATCCTTTCAGTGCATTTAAG
ATAGGCTGTTTAAAATAGCATCTGAACTTCTGTGTTGGTGGGCTATTTCTGAATTGCCACACT
GGAGGATATTTGTGTACATGTGTACTCGTGTATATTTGTGAGAATTAAGGCTGAGAAGGAAA
TGTTATCAAAGCAGCTTAAATACAATAAAATAGATTTTCAATATCAAAATAATTATTAAACT
AGCCTTTATATTACCTTTTTATTAGTATAACAAAGATAAAAAAAATGTCACAGTGATAGTGAT
AAGGGAATCATCTTTTTAAATTATCTAAAATCTAAAATTTTTAAATTATTCTTTCTCAAAAGC
ATAAATCATAATGAGATTATACAAGGCCATTTCTACTTTATCCTAATTAAGTGATATTTGGAA
AAACAATCCCAATATAACTAAAGACTTCTATAACATTGATTTTTAAATAAATTTGTCTATAA
AAATATCCTGGAAAAAGAAAGCAATGCTCAAGGTAAAATATTGCTTTGGGTTCCTTTCCAGT
TGTATTGCACAGCTAGCAAATGAGAGAATATAACACATGCCTAGAAGCAATAGCACAAACA
GCCCAGTGAGGACTGGTTTAAATATGATCTAATGAAGTAAATGTATTTCTGCCTGTTATTGTT
ATGCTGTTGTCTTTCAAGGCTGAGTTAGAGGCATTTATTCATCTGGTCTGACAGATAACAGTT
TAACCTGAAGATTTTTATTTTCCCTGTAGGATTCTGAGAATTTCTTTCTAAGAATGGGATTTT
CTTTTTATTTTTAATTTTTATTTATTTATTTGTTTAAGAATGGGATTTTAACATGTGGGTCTTTT
CTCCCCAAAGCTTAAGGTCTTCTTTTCTGATGAAATTTTAAAATAATGCCTTTCTTTTAAAAG
TAACTTCATTATATACTTTTACTATTTAGGATGGCTTTTAATATGAAATTTTATTTAAATATTA
TTTAAAATTGTCTTTTTATCTAAAGAGATCTAAGAGAAATACAATTTCTTGGTTTTCATAGAA
GGATTTTGCTCTGCTCATGAAATTTCTGTAAGACTAACAGAAAATAATAAATAGTGAAGAGC
TTCCAGTTAGAACAGTAGGCTATTTCCTTTTTTAAAGTCTTTTTGTCATCCCTGTTGATTGACG
AAGTCCAACTGTTTTAAAAATCATTAGACTTGACACACTTTGAATAAATGAAGCATAGAAAG
TACATTTTATTTAAACTTCGTGAAGTAGGTTTTTCAAGCAGGAAAGTGAAAGAAGCAGGTAA
TTCTTCCTAATTTACATTTTCCACCCCCAGGCCCGGTTTATCATCTGGAATAATGACTGTATA
TGAAAGGTCGCTCAGCAAGCTAGCTCCTTTTGTCTCTCTGGCCTCTGACTTACTTGGATGGGA
AAATGGCCAACAGAAGGAAAAAAAAAAAAGACAGCAGAGGATCTCAGATATAGGTATTAGG
TCTGCACTACAATGTAAAAACAGACCCTCAAGACCTATCACACCCCTCCCACACACTCTAAG
TGGGAAGTGTGGGGTTTCCTTTTTTTTTTTTCAAACTCCTTTTGGAACAAACACTCATTGGGA
TCTGACTGATGTTGCCTTTCTCTTTTTTTTAAAGTATATTTAAACTCACACACTTTTTATAATG
AGGATTTCAGTGTAGGGTTTTCTGCAGGATACCATTTGGTCCTACTAAGAACTGACTGTTCA
AATGTTGGAACATTTGATCGGAAAATACTTCTTAGTCTTTTTAAAGGTAAGTCATACTGATTT
TTTTTTTTTAATTTCTGTCTCGTAAATGACAGGAGATGACACTATTATATACACACCCTTAAA
GTTAAAGAACAGCAGCAGTAAGCAGGAACTGTTTTTTGAAATCATTAGGCTTTTCCTAAAAG
CTAGGTTTGTGCTGTTTCCAAAGTTATCAACCTTACCTATTATGTTTAAGAAATATTAAAACT
TTGTCTAATAATCAGTGTCATGTTGCTTCTTGGTGTTTAAAACCTTATGTTTATGCATAAACA

GTTTTTGTTAAAATGATGTACTATCCAATTTTGTTACTTCAGAACTTTTATTGTCTACTTAGGG
TATTTGTGCATGGTTTTAAATTATGAATGAAAATCAACATAAATAAAAATCAGTTTAGTAGG
ATTAGTTCTTAAAAGTCACTTGACACTCTTATGCCAACATGCTTTCTAATATGCAATTCTTCA
GTGCTTATTATTTTAGAGAGTCTTTGGTTTATTTAATCTTCTATTTTTTAATGTCACAATTTTTT
GGTGAACCTGACTTTGTCTACATGAACAGCAGTGTATATTTTGGATATATTCCTCTTCAGCAT
TTAAAAAAAATTCATTCTCATTGAATTTTACTTGCTGCTGGGAATGGTAAATTAATTCCAAAT
CATTTTGATTTATATAACTGTATAAATTATATAAAACTTGGTAATAGAATTTTTTAAAAATTG
AAGTTTTTGTGATTTTAGGAAACAATACAAATAGCAAATTATTTTCTTGAATGACAATACTA
ATAAATGACAAATCCTTCTGGAGAAATTAAAAGCTATAAATTAATTATAGTAAGTGAACTCA
TACATAACTGTAAATGGCATAAATTCACTACAGTAAATTTCAAACAGTTTCTAGAATTGTCA
AGTAACATATTGAAAAATGTTTCTAAAATCTTTGCATGTACTTCAGTTCTTTAGTGTGTAGTT
TTAAATTAGTTAAAAACAAAGTGCTCTTAATATTAAATCAAAAAATAACCAAAATAATATTT
TTCAAAAATTTTGTTATATAGAAGAGTCCAGTGGTCCACTGGATTTCATGAGAGAAAAAAAT
ACACAGAAATTTGTTTCATTTACAAGAATCATTACAAGAACAGAACAATAAATTTAATACAC
AAACATCAGATAGAAGTTAAACTGAGAGGAATAAAACTATACTTAGGTACTGGACAGAATG
CACTGAAAATTTTGGAATTTATCTTGGGAGTTACAGTTGCTACCCTATAGGCAAAAATCTTGT
GGATTCTTTCTTCCTGAAAGAACTGATTCTGTAACTATTTTCTGATTTAATTACAAGGCATTT
TTTAAATTCAGTGGCCAAAATCATGGTTGAAAGTTTTGAGAAATTATTCTAACCTAGGAGTT
GATTTTTCATTTGCATAATATTCTAAACCCCTGTATACCTAAGAATAAGCCATCTCATCAGAT
TTTATTGTAATCTAAGAGTTTTAGAAATGATCATTTATTATTTATGAAAAGACACATTTACTA
GAATTTTAGAAAATACTTGGCCAATTCACAATAATATTTTACTGTTGTTACAGTTCAATATAT
TTCATAATATTGCAAATAAAATAACAGGTGCTATGATAGGAAATTTTATTCCTTCTCACTGA
ATTTTACTTGACATTTATAACCATATATAAGACTCTGATGCTGGGAGGGATTGGGGGCAGGA
GGAGAAGGGGACAACAGAGGATGAGACGGCTGGATGGCATCACGGACTCGATGCACGTGA
GTCTGAGTGAACTCTGGGAGCTGGTGATGGACAGGGAGGCCTGGCATGCTGTGATTCATGG
GGTCGCAAAGAGTCAGACATGACTGAGCAACTGAACTGAACTGAATGATGTATATATACCC
ATTTGAGAGAAAACTGAGCCACAGAGAAACTATGTTAGCCTGCAAAGCAAACCTAAATTAC
TTGAATTCAAAAGCCCAGAATAATAACTATTGAAAATCTTATAGTATCTTCCCATTCATAGG
AAGAGTTGGGTTGGGGAAAAAGAAACAATAGTGTTTCTATCAGGATTCAAAAATCAAGAAT
GGTTCTAGATAATTCTATTTAAACTGATAAGACTCTGAGAGAATCATTTCAAATCTATAGTA
ATCTCTATAATCAAGTACTCTAAAACCTACTCCAAAAGATTGGTGTGTTTCTAGAAGAACAT
TTAATGTGATAATGAAGTCAATGTAATTATTGTTTTAATTATTTTAAGATTACCACAAAAATA
AAAGTTGTTATATTTGAAAGGCATAATTTAGTGAAGTGTGTTATATTATGATATCTATACATT
TTCTATTATATATATTTACCTCCATATATTCTGATAGGATGGAAATGCCCATATACATTAAAA
AGATTATGGTAACTTCAATAAGAGAAGATGATGAGGATTTCTGAATCTAATTTAAACATTTA
CCACAGAGCCCTAGGGTTCTGCGGGGATGACTCAGAGCCCAAATGAGATGTGACTAGAAGG
GGAAAGGGCCACAGTTGCTGCCCACTTTGATCAGAATAGATATTCTTTTAACTACCTTCAAA
TGTTGGGGTTGCATGTAAAATAAGTACTAATCTAGTGACCTTATGTATTCACTTGATGTAACA
CAACCAGATTTAAATAACTGATTTAACCAGCTTGATATGAATCTGCATCTTACTCATGTCTAG
GGAATCATGGCTTTGAAAACCCACCTAGATATCATGTAAGGTAAGAGTGTGAAAAGGAAGT
TAGTTTGGAAAGCTAATTTTCATATTCAACCTGTCGTTGAATTAATCGCCCCCAAACTCCAAA
CCTGGGTTTTGTGGGCAAATACTGTATTTAGTGAGGAAAAGCCGTGTATTAAAAGAATTACT
TGTGTGTCCAGTAGAAAACAGAGATGATCTGGTTTCCTATTCTGAAGACAAGCAGAAGGAA
GGCTTCTGGATAGAATAGGAAATTTGGGATATTCACTCATGAGTCAGAAAATGTCATCCAGG
TCCTGCATTCATTCATAGCCCAAGGATGTAATATGAAACTGTTTTAAAGTTTCATAGTTCATA
TCCCAGGAAACCATACCTCTTGGCCATGCCTTTTTCTGGGTGGAGTTTCCAAGGAAACACAG
CTCTATACACACATGAAGATAATCACAAATTCTCTGGGAAGTGTCTCTATTATTCTGACCTAC

TTATGGGCACACTTCTTAAAGCTCAGTCTCAGTTCCGACCACCAGGTTTCCAACACAAATGG
AAAGGCATAACACTCATAAGTTCATCTAGAAACACACTGTTGACTTGACAGCAAGAAAATA
AACAGCCCACATGTAGTTACATTCACAAACTCCACAAAAGAGTAGGATTTACAATCTGTGAA
CTGACAGGACACAGTCAAGAAAGGTATGGGAATAGTGGGATCTTACAATGTCTTTATTTTCT
TGTCTTTTTTCTTTTAAGTCTCAAAAATAGGTTTTCTTTCCTGATTATATGTGAATTTTTTGTTT
TCTAAATTGAAGTACTTTAAGCAAGCAGCTTACTCCTTGGAAGGAAAGTTATGACCAACCTA
GATAGCATATTCAAAAGCAGAGACATTACTTTGCCAATAAAGGTCCACCTAGTCAAGGCTAT
GGTTTTCCAGTGGTCATGTATGGATGTGAGAGTTGGACTGTGAAGAAAGCTGAGCGCCAAA
GAATTGATGCTTTTGGACTGTGGTGTTGGAGAAGACTCTTGAGAGTCCCTTGGACTGCAAGG
AGATCCAACCAGTCCATTCTAAAGGAGATCAGTCCTGGGTGTTCATTGGAAAGTCTGATGCT
AAAGCTGAAACTCTAATACTTTGGCCATCTCATGCGAAGAGTTGACTCATTGGAAAAGACTC
TGATGCTGGGAGGGAATGCGGGCAGGAGGAGAAGGGGATGACAGAGGATGAGATGGCTGG
ATGGCATCACTGACTTGATGGACATGGGTTTGGGTGAACTCCAGGAGTTGGTGATGGGCAGG
GAGGCCTGGCATGCTGCGGTTCATGGGGTCACAAAGAGTCGGACATGACTGAGTAACTGAA
CTGAATTGGACTGAAGCAAGCAGTAAATTATTTCTAATAAAGAAATAAGGGTAAAGAATAA
AATTTAACCTAAAATTAGCAAAAAAAAAAAATTTATTAGGAAAGAATCTGTATGACTAAGAA
AACCAAGTGTGCAAAATGCAAATCTTATCTGCTCTAAATAGCTACATTTGAGAGAATAGTGG
AGACAAACTACACTTTATACAGCTGTTGTTGAGTACAGCATGCTGAGTAACCTGAACTTTTC
TCATGTATTCAGTGCATGTCCTATTTAATTTGTTGGTTCCTCCTTTGCTTCAGGTGAATAAAA
CTACGGTGGCATGTATGTTCGGTCGCTAAGTCCTGTCTGACTCTTTGCAACAACCCCATGTAC
TTACTGTAGCCCGCTAGGCTCCTCTGTCCAATGGGATTTTTCCAGGCAAGAATATTGGAGTA
GGTTGCCATTTCCTTCTCCATGGAATCTTCATGACCCTGGGATTAAATCCACACCTCCTGCAT
TGGCAGGCAGATTCTTTACTACTGCGCCCCTGGGAAGCACAAATTACAGCTGACCCTTGGAC
ACAACAGGTTTGAACTTTGTCAGTGCACTATGGAGTATCTTTTCAATAAATACTACAGTACA
ACCTGATCCCATATATGCAGTTGGTTGGATACCACAGGTAAGCTGATGGTGTAGTTATATGT
GGATTTTCCACTGCCTGCATTGTCCATAACCCTCTGTGTTCAAGGGTCAGCCTGTATTTGTAA
TTTTGTAGTATGAAGCATAAGCTTGAAGTATGATTATAATAACAACACTTATGGTAACAAGG
CACAGAAGCATGATAAACCCAAGACCCTCAGTGGGTGCCTGAGCCTAGAGATAGCACCAAA
CCCCAAGTAATTCCCCTTCATCCAAGATTTTACTTTCTACAGATTCAGTTAGCTTCAGTTGAC
CACTTTTGACTGTGGATAAGTGAAACCATGGAAAGCAAAGCCATTGATAAAGAAGGATTAC
ATTTATATAAAATATCTTGCCTATTTTTAGAGAAAAAAAAAACTTGCATCAAGATAACAAAA
TAAGCTGGGCAAAAGTTCATAAAGTCAATCTCTTTCTGTCAAAAATATCAGTTTATGTATGC
AAGAATGCGAGTCACACTACTATTTTGCTTAGACATCATCCAGACCAGTGTTATATTAGTAA
CTGTCTAAGCATGCAATTTAGCAGAATAAATCATTGACACTTATGTTTTCTGATGAACTTGAC
TCAACAGCACTGGGCTGTGTGCACTATCTCACTTCAGTTTTAAGTTCAAAACAGACAGAGTC
TAGGCAGTACCCAGGTCTGCCAATAACTTGACATCTCTGGGCGCAGGGTCCTTGGATGGTAT
AATAATGCACTTTCATGTTTAGAGAGGAAGGTGCTAAAGAACACAGTGGTTCATCCAGCAG
ATTTTTGGGGACTTTTTGCCCCTTGGGACTCTTCACTATAGTCCATGGGACTACTTAAATAAA
GGATAATGATTCTGGGAAAGGTCAGACAGATTCTCAACTAGGTGAAGGCACAGAATCTGGG
AACAGACAAATAATACACACCTATATCCATTTCAGAAATAAAGATTTATAATTTTAACATCA
TTTTATATTCTATCTGACAGAACTGCAAAGGACATTAATATTGAATAATTTTGTGGCATTTAT
GTAGACCATATCTTTCCAAAAGAGATGATTTGAGTTGTATTCCCGATGTTATATAAACAAAA
CTAGAATCTGACCCTTGGGGTTTCCTATCCAATTTTTTAATAAATTGCAATCCACAGGTTCTA
CAGAGATGCTCCTCAAGCATCCTAAACATGGGACAAGATAGTGCTAGGTGGGACACAGGAG
ACCTAGTTAGGAATCAGGGTCTCGGTGGTCCCACAAATGCTGTAATCTAAGTCACTTGGTTT
GGTCTTCCATGTAAGGATTTGAGTGAAGAATCAGTCTAGCTGCTTAAATGAAAAAAATGGGG
GTCTCATCTTATAATCTTACTTTATACATGAAGAAATTGAGGAACAGAGCAATTAAATAGCT

TGCCCATGGTCATCTGGCAAGAAAGTGGCCAATCGCACTTTGGTTTTATCTCAAGTCTCCCAC
CCTTAATTACAGCAGTTTGGAACCCTGCATTTTGATGTCTACCTCAAATATAACTTACAGATG
AGAACTGGGGAATGGTCTATAAGATGGGCATTCAGTAAAATAGTACTTGATAAGCTCCCCTG
AATATGGTCACTTACATACAGTACAGCACACTCGAATGCTATTCACAAGTAGAAGAGAGATT
CTACTTGAGAATATCTAGTCATTATTATCAAGTTTAAATAATTTATTCCACTGTATTATGACA
ATCCTAGCCAAAGCCATAGGAGGGAGCATGTTATTCCTCATCCTCTGTGTTAAATCATTTCA
GTCATGACCAATGTGACCCTATGGACTGTAGCCCGCCAGGCTCCTCTGTCCATGGAATTCTC
CAGTCTAGAATGGAGTGGGTGGCCATTCCCTTTCTAGGGATCTTCCTGACCCAGGAAACTAA
CCTGTGTCTCTTACATCTCCTGCACTGGCAGGCGAGTTCTACCCCCAAGCAATTAATAAAGG
AAGGAAAGCTATGCCAAACTTAGTGTACTGAAAAGCAAAGACATCACTTTCCTACAAAGGT
CTGTATAGCCAAAGCTATGGTTTTTATAGCAGTCATGTACAGATGTGACAGTTGAACCATAA
AGAAGGCCGAATGCCAACAAATTGATGCTTTCAAACTAGGGGCTGGAGAAGACTTTTGAGA
GTCTCTTGGACTACAAGGAGATCAAACCAGTCAATCCCAAAGGAAATCAACCCTCAATATTC
ATTGGAAGGACCGGTTGAAGCTCCAATACTTTGGCCACCTGATGTGAAGAGCCGACTAATTA
GAAAAGACCCTGATGCTGGGAAAGACTGAAGGCAACAGGAGAAAGGGGTAGCAGAGGATG
AGATAGTTCTATAGCATCATGGACTCAATGGATATGAATTTGAGCAAACTCCAGGAGATAGT
GGAGGACAGTGGAGCCTGGCGTGCTGCAGCCCATGGGTTGCTAAGAGTTGCTAATGACTTA
GCAACTAAACAACAACAACTCAACTTGCTCAGAGACATAAAATTCCACGGGAAAAATAAAC
CACTTGTTTAGGCTTGTGTGTTTTGTTTACATTGGGAAAAAACATGTGACATTAATGACTATT
CAAACTTCTTAGCCACCAGGACCTCAGTGTGAATCAGTTTGTTCATCCTGATTTACAGTGGAT
CATCCTAGGTATTTGGTATAAGAGAATGGAAATCCTGAAGAATGAAAAAAGTTTGTAAGATT
TGCCTTTTTCCATTTTGAGGGAAAGTCTTATTCCTTTCTTTTAATAACATTATTCTTAACATTT
TCTTAGCTATGCAATTAATAATTTTAAAAAATTAAATGCTTACTCAAGTGGAAAGAAATACT
AATAAAACCATCAGAATGGTACTGTAACCACAACCAAGCTATGGAGGGAAAAATCTGAAAC
CATAACGAAGTATGATTTTTACTATTATCCTTACAAGACTGACAAAATAGTGTGTGTCCCTCC
AATCTGTTGCAATTTTTGTCTTCCTTGGGCAAGGGTATTCTGAATTAGGGAGTATTTGTTTAC
AAGCTACAAAGCACATTCTGTTTATCAATGTCAGGTCAACTCTAGTACAGATTTCCTACTGC
AATCAAAACTGCTTAAATAGTATGTTTTAGGTATTTGAATAATAAAGTCTTGTTACCATAGG
ATCTGCTTATTATGCTAGGAATTTTTTCCAAAGATAAGACTAAGTGAAATACATATTGACAC
AATCGAGTTGATTTTAACAGAATTTTTAAGAATCAGTTTATTGTTGTTGTTTAGTCTCTAAGT
GTGTCTGATTCTTTTGCAACCTCATGAACTGTAGCCCACCAGGCTCCTCTGTCCATGGGATTT
CCCAGGCAAAAATACCGGAGTGGATTTCCACTTGCTTCTCCAGGGGATCGTCCCAACCCAGG
GATTCAACCCACATCTCCTGTATTGGCAGCCAGATTCTTTACCACTGAGTCACCAGGGAAGC
CCTATGAATCAGCTCCCTACAACGAAAGGAACACTATTGCTCTTGAATATGTTAAAATTTAT
GTATTTGAGGCTAGTAAGAAAGCAATACCTATTACAGAATCTAGATATACTCTACCTTACAT
ATTTTCTTCAAGTTCTGTTTACCTTTAGATGATATAAAAATCAGTTATTAAAAAGAAGTGAAA
ATATTATGTAAAATATTTCAACATTTTCAACACAGTTGATAAGAAAGAATTCAAGCATGAAC
TCAATATCCATGAGAATGGAAATTTCTTTTAGCAGTGATTATAATTTTGGTGATATTAGAGA
GTAGTGTAGTGTTAATTTTCTGACAATGTATTTCCCTAATTTACTCATGTTTTTTGCTTTAACA
GATAGGCTACCAGTGATTAGAAGAACTGACTCCTTCTAAGACTCATCAGATCATTTCCTGTA
AAAACTGTATCTTTCTAATTGTGAAAAAGGTAAAGAAGACATTTGTTATCATTCTTGTTTAGA
ATTTAGATGGAATAGACTCCAGTCAACCCACATTCAGTTTGCATAACACCAGGTTCAGTTCA
GTTCAGTCGCATCCGACTCTGTGACTCCATGAACCACAGTACGCCAGGCCTCCCTGTCCATC
ATCAACTCCCGGAGTCTACCCAAACTCGCATCCATTGAATCAGTGATGCCATCCAACCATCT
CATCCTCTGTCGTCCCCTTCTCCTGCCCTCAATCTTTCCCAGCATTAGGGTCTTTTCAAATGAG
CCAGCTCTTCCACATCAGGTGGCCAAAGTATTGGAGTTTCAGCTTCAGAATTAGTCCTTCCA
ATGAACACCCAGGACTGATCTCCTTTAGGATAGACTGGTTGGATCTCCCGGCAGTCCAAGGG

ACTCTAAAGAGTCTTCTCCAACACCACAGTTCAAAAGCATCAATTCTTCAGTGCTCAGCTTTC
TCTATAGTCCAACTCTCACATCCATACATAACCACTGGAAAAACCATAGCCTTGACTAGATG
GACCTTTGTTAACAAAGTAATGTCTCTGCTTTTGAATATGCTATCTAGGTTGGTCATAACTTT
CCTTCCAAGGAGTAAGTGTCTTTTAATTTCATGACTGCAATCACCATCTGCAGTGGTTTTGAA
GTCCAAAATATAAAGTCAGTCACTGTTTCCACTATTTCTCAATCTATTTGCCATGAAGTGATG
GGACCAAATGCCATGATCTTAGTTTTCCGAATATTGAGCTTTAAAGCAACTTTTTCACTCTCC
TCTTTCACTTTCATCAAGAGGCTTTTAGTTCTTCACTTTCTGCCATAAGGGTGGTGTTATCTGC
ATATCTGAGGTTACTGATATTTCTCCCGGCAATCTTGATTCTAGCTTGTACTTCTTTCAGCCC
AGTGTTTCTCATGATGTACTCTGCATATAAGTTAAATAAGCAGGGTGACAATATACAGCCTT
GATGTACTCCTTTTCCTATTTGGAACCAGTCTGTTGTTCCCCAGGTAGTACTGTATAAATGGC
TGTGTTCAGGTGGAAAAGCCAGATAGAGGGAAAAAATGTCCATAAGTGTCCCAGGCTGACA
GGTAGGCAGAGGGCAAGAGTACATATGCATATTCTCCATAGATAAGATTTCAGTATGTTAAT
TATCCACAGAAAAAAAAAAAAAGAGGAGGATTTTGAGGAATTGCTCTATGTGATGATATTTAT
TGTTTCTACCACTGGTAGGTAGTCAAATTAAAAAGAAAATACAAGACCTAGAAAGTTTTCTT
AAAAAAAAAACAAAAAACGTCTGTTTATACCTGGCAAAATGATTTGTTTCAGTTGCGTGTTT
TCCTTGTACTTTGATCTTTTTGTACTGATATTTATGCTTCTGTTTTATGATCTCCTTCTAACGA
AAGAACCAGACCTCTTGGCAACCACCTTCATCAGACCTGGGAGTTTGAAAGAAGCAGGTGC
TGAACCTCAGTTTTTCCTTGAACAAGAAAAATGGGTTGCAATAGAAACTGTGGGCTCATCGC
TGGTGCTGTCATTGGTGCAGTCCTGGCTGTGTTTGGAGGGATTCTAATGCCAGTTGGAGACA
TGCTCATTGAGAAGACAATTAAAAAGGTACAAGTTGTATCAAGAATATTTCTTTTCATCCTG
ATTCAGTCTACCTTGATTTGTGTTTGACTTAGGGTTTCTGTTTTATATTTCATTATAACCAGCA
ATTTAACACAAAGGTGATTTTGAACAACTGAAACTCTACATGACAGAGAAACAATCAAAGT
ATGATGTATACTCCATATGAATGTACTGTCATCTTAAGTCTAAATATTTTAATTTCTGATAAA
AACTATTATAGGTATGCACATAAGTAGAAATAATTCCCTTTAATTATGTCCAAAGAGTATGC
TGATATGCTTTTCATATGCTTTTGAATGAGAAATGAGTGTTATTTTGTGAGTACTATTTATAC
TATAAATAGTACTATAAATAGTACTATAAGTGAGTACTATTTATAAAAGTAGTTGCACTTTG
GTTACAGTTGATTAAAGTAAAATTATATATAACTTAATACTGTGTTTAATATTGATTTAGGAA
TATTTGGTAAAACTGGTAAAATTTATTTTTGTTTGGTAAACTGTTCTATATGTGTGTAATGTT
AATAATATTATTCTTATAAACATTTCCTACTGATGAAGGTAGATAAGGGATTTTTACAAAAT
ACTTTCAGAAATGTTATGCAGAACATATCCCAGTATATATATGTATGTATATGTGTGTATATA
TATATGTGTGTGTGCATGTGTATATATATATATGTATACAAACAAGTTCACTGTAGATTAACT
AAAAATAAAGTGACTTAATACATAGGCTTTGTACAACAAACTAGAATCTGTCTCTCATCCTA
GTTCCTTCTAATTCAGCTTTATACGCACCAACTTTAATTGATAAAGAAAAGGAAGACTAGGG
TAAGGAGATGTAGCAAATTCAAATAACTTCAGGGTTTGGAGACAAGCCTTAAAAAAAGCTA
CCAAGAACAATATATAAACAAGACAATGACTACTGTGAAGTTAAAAGTGTGTGGCCTACTG
AGAGGCATGTATGTTTATTTTTAAAATATAATGCTGGCCAAATAAACTTATCTGAGTGCTGG
CCTCAGCTGTCATCCTGGTTCAGATGCCAGTGGTCTAGATGTCAGTAAGCACAATGTACGGA
GGAGATCAACAAGAACTGAATCATACAATGGGCTAGACTACCAACTCATACTGGGGTTTCTG
TTCCTATCAAGACCTTTATCTGTCTCATCCATTCACCCATGTTTGACATCCTGCTCTTACTAGG
AGTTTAAATAATTACAATTATTTTTCACTAAAGCATTGCCTGACTTAATATGAAGATTTAATT
TCAAATCTAGTTACCCAGGACAAGAAGTTACTATTTTCCAAAAGCTTTTCGGTTCGTTTTGTT
CAGCTAAATGCTTAGGCTGTAAATTTAAGAATTTTTCTGACTGTACTATTATAGCTATTGTCT
TAGCACAACAGATTAAGAAATAGGACATTTTTGGTACAGTTTTTACACTTGTATGCTATATTT
GCTTAATAACCACACAGGCATTTCTATCTTTTCTCTTGCTAACTGACAGCAAAAATTTTAACT
TCATGAATTTAACTGGAAATATTATACATTAAATACCATCTCCTTCCTTGGAATTCCGGTTTT
CTTGATATTATTTTAATATAGCTCAATAGAGACAGACAGGAGATGGTGAGGTATGGAGTGTC
TAACGTGTTAGAGAGAACTGCTATTGTCCTGTATTCTTTCTGCTCTAGAAACTCACTTCCCTT

CTGTATCTGTGGAGTGAGAACAGGATAAATTTGACACACAGACTATAACTTTTTTTAATCTT
AAGAGCCACAATAATCTTCCAATGTACAGCATCTTAGACTAAGATTCTTAGACTAATAATTT
TTATTCATACTTGATAATCAGTAGCAAACAGTGATCATCTATTGTTTCTTATTATTCAACAAT
ATTATACATACAGAGTCTCTGTGTCATGAGAGGCACTAACATCTATTTTAATTTTTAATATGT
ATCTCTAAGTTCATATGGCTAATTTTGAAATAAGAGTTTTGAGACATCAAAAATTTGTATAG
AGAGAATCAATATTTATCTCAACCTGATTCGCACCACCGGGAGCCACTAGAACTCAAAAGCA
ATCTTGAGAAAGAAGAATGGAACTGGAGCCATGACATATAGATCAATGGAACAAAATAGAA
AAGTCAGCAAGAATATACAATGACAATCTCTTTAACAAGTGGTGAGTACAAAAGTACCTAA
GCAATGTAACTGTGAAGGCCTACTGAGAGGCATGTATGTTTATTTTTAAAATATAATGCTGG
CCAAATAAACTTATCTGAGTGCTGGCCTCAGCTGTCATCCTGGTTCAGATGCCAGTGGTCTA
GATGTCAGTAAGCACAATGTACGGAGGAGATCAACAAGAACTGAATCATACAATGGGCTAG
ACTACCAACTCATACTGGGGTTTCTGTTCCTGTCAAGACCTTTATCTGTCTCATCCATTCACC
CATGTTTGACATCCTGCTCTTACTAGGAGTTTAAATAATTACAATTATTTTCACTAAAAGCAT
TGCCTGACTTAATATGAAGATTTAATTTCAAATCTAGTTACCCAGGACAAGAAGTTACTATTT
TCCAAAGCTTTTCAGTTCGTTTTTGTTCAGCTAAATGCTTAGGCTGTAAATTTAAGAATTTTT
CTGACTGTACTATTATAGCTATTGTCTTAGCACAACAGATTAAGAAATAGGACATTTTGGTA
CAGTTTTTACACTTGTATGCTATATTTGCTTAATAACCACACAGGCATTTCTATCTTCTCTTGC
TAACTGACAGCAAAAATTTTAACTTCATGAATTTAACTGGAAATATTATACATTAAATACCA
TCTCCTTCCTTGGAATTCCCGGTTTTCTTGATATTATTTTAATATAGCTCAATAGAGACAGAC
AGGAGATGGTGAGGTATGGAGTGTCTAACGTGTTAGAGAACTGCTATTGTCCTGTATTCTTT
CTGCTCTGAAACTCACTTCCCTTCTGTATCTGTGGAGTGAGAACAGGATAAATTTGACACAC
AGACTATCTTGAGAAAATACAATAATCTTCCAATGTATCTTAGACTAAGATTATACTAATAA
TTTTTATTCATACTTGATAATCAGTAGCAAACAGTGATCATCTATTGTTTCTTATTATTCAAC
AATATTATACATACAGAGTCTCTGTGTCATGAGAGGCACTAACATCTATTTTAATTTTTAATA
TGTATCTCTAAGTTCATATGGCTAATTTTGAGGAAATAAGAGTTTTGAGACATCAAAATTTGT
ATAGAGGCTAAGAGATAATCAATATTTATCTCAAGAAATATAAATTTAATTGGGAAAAGCAT
ATTTAAAAAGCTAGAGACTTGTGAAACTCTGTAATTGAATTCTAAAGCATACACTATTTAGA
AAGAGAACTTTCATAGAGTATTCAATAACTTCAGGAAGAAGGTGAGACTTGAGTTGGATTTT
GAAGTATAAAATTTTGATCGCCCAAAGAGAGTTCCAATAAAAGTGTCAAGGGACTGCTTAAT
GCCATAGAAAAGAGTTGTGGAAATGCAACTAAATATTGTGACGATAGAAGGAAAAATATTT
TTTTAATGTGTCAAAACAAAGAAATAACAGCAAAGATTATTGGAAAGATAATAACATGGGG
TAAAATTTAGGAAACTTTTGAGCATCAGGCAGAAGACTATTTAAGTAGTGACATGACAGGC
AATAGTAGTGTATATTTACCTGTAGTTGTTAGATATAACAAAATCTGCATTTAGAGACTGGT
ATCAAATATACTGAAAAAAGAAATGGCAATACTATATACTAGTAAGTTATATGCCACACATA
CTAATATATTATACTATATACTAAATAAATAAAGCATTTTTTAGTCCTACTAAGAGCTTCATA
TATACTAATTCATTTAATCTTCACAAATATCAATGAGAAGATACTACCATCACAAGACCTTA
AATCAACCTGACAACTTACCCAGCCAAAAGGAATATGAGTTAGTTTCTAAACCTAGGCAATC
TTAAGGCAGAGATGAGATGATTTATACATTTTGCACTCTCTAATTCTACAATAGAAAGTGTT
GGAAAGTGAGGAGACCAAAAACAGGAAATGTTATGTAGGACATGACCACTTTCAAGGAGAA
TTTAACACTCACTTTTAAACAGATTTTTGCACAGTTTGCATATAACAAAAGGTTCTCTGGTCT
AGTGTGATGGAGTATGTAGGACCGATGGGTGATGGAATGCAATGGACAGAGGAACTAAATA
AATAGGGGGAGAGAGGAGTAAAAGTTTCAAACTACAAGATGTCCAGTTGTGTTCTTGATAG
AAATATGCATACCTTAGAAGAATAAAGCACAGAGGTTTTATAAGTGATTTGAAAAGAACAT
CCGTGTATATTTCAAAGGGAAGGTTCAGAGATTTGTTCATTAGTATAGAAAATACAGATAAA
ACTAAACAATAGATATGTTTAAGGAGAGTTTGAATATAGAGACGAAAGAGCAGAGAAATTA
CTATGGGATTTATGCATTCAGAGGTAAGTAGGAAGATAACAGAAAAAATATTTTATATGAA
GCAATGAAAACAATAGACTGTAATTTTTTTTAGAAGTTATTATGAAGTCAGTTTTTAAGGAA

AAAGTAAAATTATCTCAGGCAGATGAAAATTAAGGAAATAGTAGTTAGAATACAGTCACTA
ATAACTTTAAAATTATATTTTCAGTAGTGCCAAGTAATAAAGTGGGATAAAATAAAGAAATA
GAGTGTTTTCCCTTCTTATTTTTGAAATGAGGGTTACCTGCAATCTTTGGGTCATACTGTAAC
CCAGCTCAATATTCACAGACTAAGACATCATCTCTAAGACATCACTTCTCAGCCTTTTGGCTA
AGATTACGTGGCTTAGATGGTAAAGCATCTGCCTGCAATGCAGGAGACCTGGGTTCAATCCC
TGGGTCAGGAAGATCCCCTGGAGAAGGAAATGGCAACCCACTCCAGTACTCTTGCCTGGAA
AATCCCATGGTCAGAGGAACTTGATAAGTTACAGTCCATGGGTCACAAAGAGTTGGACACA
ACTAAACAACTTCACTTTCACTTTTCAAGACATCATCAGAAGCCCTTCAATGGTCAGGAGCT
GTGTTGTATAGTTAAGACAGCTTGCTTTTGCGCTCAGATTTTCATATAGCGGTGTTGACCTTG
CTTGTTTTAATTATGTAAAGCTGTTGTGACTTTTTCTTCATTCAAATGAAACTGTTTCTTTTAA
ATTTAGTTTACAAAATTATTTTAAATATAAGATTAGCATATGTATCTGTTTGGCATACGTGTA
GATGTCAAATCTTTGAACTATGTGTAAGCACAGAGAGCTAAGTGTCTCGCCCCTGCCTCAAG
GGCACTTAGGTTAATACTGCTATTTTACAAACATATATATATATATATATATATATATGTTGT
ACTACCAGACAGTTTTTCTCTACAATTAAAACTCATTGCATGTAACTTGCATTATTTAATGGA
CTAACAGCAAATTGGCAAAGTTATTACCTATATATAAGCACCATTAAAAAAATAAAGTGCTT
AATTTAAAAAGATAAGAAAACCATCTCATCATGTTCTCTAGAGTTGGGACTCTGATATTTTCT
GGTTCTCAAAGATGACACCTGATACCAATATCTGAAATGGCGTAAAACAGTCAATACATTTT
ACCATTATATTTCTTTATTCACTATTTCCAAAGGAAATTTTGTAGGGTTTTTATGCATTATAG
ATAATTATGCATTATGTAGGGGTTTATGCATTATAGCTGAATGCTTTTATACCAGTGTTTCAG
ATCATGATTCCTAGTAGAATTGAGTGGGACCTGCAGATTTGTGCAATTTTGCTAGATAGACT
AAAAGTCAATGGCATGAGCTGCCATGAATGTGGAAAGCAGAAATTATTAAAGGACTTCAGA
AAAAGGTCAAAGTTCTGGCACCACTCACAAAGGATAATTTATCACCTGACACTATAAAATAT
CACTTGATTAATATTGCTGAATATTATATAATAAGCAATTTTAAATAATTTGAATTCACCTAT
GATATTAACCATGGAACATCAAGACAGAAAATCAAGATATCTTTTTCAATTAAAAGACTAAT
GTTCAATCATACCATCATTCAATTACACCATTTCCTTTCCCTATCCTTGAAAAGAATGATCTT
CTTGTAGCCAAAAAATGCTTTCAGATAAAATAAACATAAATGATGATTATTTTGACTTATTA
AAATGACTATAATTTCAATATTTGATAGTAAATTTTATACACATATATATTAATTTCATGGTA
TATGCTTAGACTATTTCTAGATATGACAAAATAATTTTTACCTTATTATAGTATCTAAAAGCT
CAAAAAAGTGCCAGAGGTATATAGGTTATTAAAGATAGTAGAACTTGCTTTAATTTAATAAT
AATTATGAGAACATGAAATAATCTAAAATAAACTCTAAATTTAAATAGTAGTAAAACATGG
ATTAAACATGTATTTGCATATTAACTCCAATAATAAAATCACTTTAAAATGTTTTAAGAAAC
AGCAGGTCAGCGTAATTTTCTTCCTCCAGCCCAATTTAATATCAACATAGTATACTTTTCATG
CCAAAGAAAAACTCTCCCATTTTAGGTTCTTATGTAAACACTTGGAGGCTGATTTCCTGAGTT
TCAGAAACATATTACATCTAATCAATTTCACCACCTGTTATTCTGATGTTTAAGTGATGACTT
GTTAATAACCTGAGACAAAAATCACTTTTTGACTGTTAGCACAACTGGCACTGATATTCTAA
TCCTCTCTTTCTCTTTCTCTCAGCATGTATACACATAGGAGAAAATGTTTTGATCATAGGAAG
ACTGCTTGTAACTGGTTCTAAACAGAACAAATTGTGGTAGTGAGTGAAGAAGAAAAAGACT
CTTTCAGTATGTTTCTGCTGTTTAGGAAAGTTTTGATTTCACTGGATGGAGAATAAATATAGA
AATGATTAAACAGCAGAAGTGTCACAGGATTTTACATGAGCTATTGCCACATGACTGGTTCA
GACAGGAATGCTATGAATGAAATAATTAATATAATTATACCATATTCCACCTATATTGACTT
TCAGAAATATGAATTTCATATCAGTCGACCTCATATTATTTCCAATATTATTATTATCCCTGT
TTTACAAATAAGGAAGCTGAGGTACAGGAAATCAAATAGCCTGCCCAAGTTTAAATGGTTA
GCATATGATCAAACTGGGAATCAAACTCATAGCATCTATTTCATTGTTAACCACTTCACTCTA
GGCTTCCCAGGAGGTTCAAACAGTAAAGAATCTGCCTGTAATACATGAGACCCAGGATCAG
GAAGACCCCCTGATCAGACCCTGATCAGGATCCTGACCCCTAGATCAGGAAGATCCCCTGGA
GAAGGGAATGGCAACCCACTCTTATATTCTTCCCTGGAGAATTCCATAGACAAAAGATCCTG
ATGGGCTACAATCCATCCAGTCACAAAGAGTCAGACATGACTGAGCGATTAACACTTTCACT

TTTCACTCTAGGCTTTGAGTAAAGAAGCCAATATACTTGGGTTGGTAGTAGGGTAAGGAAAT
CTACTCATAGATTAAAATCTGCTTTATATGATTCCATCTTTCAGTACTCTGTAAGAACCTTGA
GAAATGGGACAATATTATTTTCTGCGAATGTATTCTTTATAGTTCATTCACAGGAACATATTT
AGACTAAAGTTGCCCATCATGTGGATGAATATTGCTTTCCCATAACAGCAAAAATCAAGTGA
CCACAGGACTAGATAGAGGGTATTACAGTCCCAGATATCTGCCTTCAAATTAATGTAGCAAA
ACAGAACTTTCAAAGTAAATGGCTTTCAACTCAGCTGAAGACCATGCCACACTAGAGTCATC
AAGCTGAAATCCAGGGCTAAATAGTCTGAATGCTTAAAGTCAGGGATAGATACTGATGTAT
GTATCAGCCAAGGGAGAGATCTTCAATTATATTACTTTTTTTTTTGTACATATAATTTCATCTT
TGTATTTCAAATTTACTGAAATGTCCACAAAATAGGTCAGACAGTCCAAAGTCAATTTAAAA
TTCATGGCCTTTTAAAGTCACAAAGAACTTAAGCTCCTTTTTAAGAGGACCTTATGATATATT
TAGTCTTGATAGTCAAAGAGGTCTATACAAATTTCTAGTACCTAGGTAGTCTTAGGATCAGA
AGAAATTCATTCCTATTCTGACTGATTCAATATTTATCTCTGTCTCTGTACAAATTGTGGTTTT
ATAAAAACCAAAGAAGATTGGTGTGAAATGCCGGATTCACCATTATCTACTACCAAATTATT
GTGCCACATTGAATCAGACATCTGTTTTTCTAGGACCCACTTCTACTACTAAAAAAAAAAAA
AAAAATGGATGGTGTTAGATGACCCTAACTCTAAAGCTAGAAGTTCAGTGCACTCATTTCAA
TTCTTTGTGTTTCAACTTCCTGATTTTAAAAAAGTTATCTCCAGATTGACAAGTAAAACAAAG
TAACTCATGGTCCTTAGAAGAAAGACACAATATAGTTACAATGTTTTTTAATATAAAATGCA
CAAGTGTTAGACTCAGGGAGATTTCCGTCCCACCAAGTACATTATTGAAATCTGTGTCTGGA
GAGGTAATGATAAACAATTGATATTATACTAGTCCTTAAGAGTCTACCGTGTAAAAGTAATA
ATGTTCATGTGTATATGTTCATGTGTGTTCTCCTCTCATGTCTCTTGAAATGGGTAAATGAAA
ATAGTTTGCAGCTTCCGCGTAGACTTCAGCTTCAAAACCCTCATCATTTACAGTAAACACCTT
TACATTTAGAATAGCCATAATACTTATCTATACATAATTTTGTCTTGATTTGGGTTTGCTATG
GAATTAAGACTTTTAGGAGTAATAAAACAATCTTCAAGGACAATTGCTTGTTAAAAGTACTA
ACGTCTCACACTGCGTTAACAGTTGCGATGTACCCAGTGTAAAAGGATTGTGACAGCACAGG
ATTTTGTAATGGCATTTAGCTGATGCCCTGTTCTCCTCTAAGGAATTCTTTTTCTTTCCATTAA
GCAACAAGTCAAGTATCCTGAAGACTGCTGATTCACTTTGACTTCTACCAACTAAGTGCAGA
GGTGACAGGAACAAAAATAGTAACACGGTGTGAAAGAGGACTAGCATAAGGGTCAGAGAT
TAGGATCTTCATTGTGATTCTGCCACTAGGCATTTGGCTCAGTGACCCTTGGGCCAGATACCT
GGTTTCTCAGAGTGTCAGCATGCTCTCCTAGTAAATGTTCACTGTCCTTTTCTGATCCATCAT
TCTATGAATTGATGCTTCCTGTTGAACTAAACAAACAGGTGAGATTGTTCTTGTGGGTTTTTT
CAGGCTTATTTTCTGTGAAATGCTTTCCTTCTCTTCCTCTAGCAGTAAAAGGGTAAACTGGAG
CTTTCCACCTGAGTATGTATTCCTTCCAAGGTAGTTAATTGGAAACATTGCCTGCCACAAAGT
CACCTTTGTCTCCATTACCTCCATAGTCTTCTTGTTCAGAGAGTAAAAGCCTTGGAAGGAACC
TGGGAGAGCATCTATGATAACTCCCTCACTTTACAGATAATAAAATTGACCTCAAAGGGTTT
GGTGACTCTCTATTCTGTCTCAGCTCCTAAACTATTAATAGATACTTATGTATCCATACTAAT
AATACCTAAACTAGTAGATACTTCTAATGTTGGATTTTCTGATTTCACAAAAAGAAAAAAAA
AAACTTCTGTATTTCTTCTTTTATTTTTAAAGTATTAACCTAATAATAATGAATTAGACTACT
GTAATCTTGGAAACAGGTACTAATAGGTTTCTGCAAATGTACTGAGAGGAAAGCCTCATTGA
TCAGGGTTTTGAATGGGAGAAATCAAAGCTGTGTTTTTCTTAACAAATGTTAACCGATTTTCA
CCTGAAGTATCCAAATTCTGAAGAAAATGAGAAAACTTCTAATTCCACTAGATTTTTCTATTT
TTCCGTTTTGCCTCTTCAGTCTGACTCATTGAGGGTACATTAGAAATTAATATTCAACCTGAT
AAGAACTTTTTGAAATGCTTTAATTAATCTTGCTTAATAAATTGTTACAAATTGTTGCAATAT
TGAAACAGGGTCACTCTAAAGCTTGCCAATGGTTGGCCTTACAATCACCAGACATGTTTGCA
ATATTATACACTGATTTTCCTTGTAAAAGGCTGACTGAAAGAGATTGCTACCATAATGTGTT
GAAAACATTTCTGTTGCAAGCATGTGGCAGGTTGCAAAGGTTTTCATGAGCAGGATACTTGA
GCTTGATCTTTCACTGACTAACGAAAAAGTTTCAACTCCCAACAGCTGCCCGTATTGGCTAC
AAAAAATAACCCAAACATTTTCTTTTCATAGGAAGTTGTCCTTGAAGATGGCACAATTGCTT

TTAAAAATTGGGTTAAAACAGGCACAGATGTTTACAGACAGTTTTGGATATTTGATGTGCAG
AATCCAGACGAAGTGGCAGTTAATAGCAGCAAAATTAAAGTTAAGCAAAGAGGTCCTTACA
CTTACAGGTGAGTCCCCAAAATACGTGGCACTCTTTCCTTGAACACAGGTATTTCTGAAAAG
CTTCCACTTGACAAATGTCACTGTATTGAAATGTACTTATTATTTTCTTACCATAAATATATA
TGGAAATATTTTGTTTCTGCCTACATAAAATCCTAATCTAAGGATTTAATTATTATTAAAGAA
CAGGTTTATTATGAATTTGTCAGCATCAGTTAACTACCCTACATTTAACAAGTCACATGTGTT
TTACACTTGTGGCATATATGTTTCAAAAGCTCTGAAATACGCATCTAAATAGAAAAAATATA
TCAAAATTTTTAAAAATGAGTATTTTAAAATCTTAAAGTTGAAAAGTGTAGGTACATGAGTA
GTTCTCAAAGTATGGCTCCTGGACCAGCAACATCAGCATCACCTGGGAACTAAGGCAGAAA
TGCAAATTCTCAGGCCCCACCCCTAGACCAAGTGAATCAGAGACCCTTGGTTTGAGCCTGGT
AATGAGTTTTATAGCTCAGCTGGTAGACTCCGCCTGCAATGCACAAGACCTGGGTTCGATCC
CTGGGTTGGGAAGATCCCCTGGAGAAGGGAAAGGCTACCCACTCCAGTATTCTGGCCTGGA
GAATTCCATGGACTGTATAGTCTACAGGGTCGCAGAGAGTCGGACACGACTGAGCGACTTTC
ACTCACTCTAATGAGTTTTACAAATACTCTGGGTGATTGCAATACATAGTCAGTTTTGCTAAC
CACAGCATTAACTTCTATGTAAGTAAAATACCTGCTTAGGATTTCTATTCAAACTGTTTTCCA
GGTCTTAAGAGTGTATTTATGCAGTAGCTTCATTCTTATATTTTCTCCAGTACAAAGACTGAA
TAAAAAATACCTTTATCTACATGCCGTAACAGTTTAAAACATTTTGTATGGAATCTAAGATC
CATCAGATGTAGGGAGAAATGATTAGCCAGAACTTTTCATTTTGTAAATTTAAAAATCAGAC
TGACACCAGGTGAAACAGTTTGTCCACGATGACAAAATCCTGGAAACTGATAACTCCTAGTT
ATTGGTGTACATTTTCCCCCTGTCAAATTTATAGATTGAAGTGCTAAAAACACTAACCTTAGC
TAGGTATTAGGACATTTATAGACCCCATATGGGAACAGAATTAAGTCCTGTTGTATCATTTG
TTATATAAATATGATTTTTCAAGGATAATTACAACTAGTTATAGACAATAATCAAGTATTATC
ACAAAACTTCATGTTTGTAAAGATTTCTAATTCATAGTTTTACTTTCGTACTTGTTTTGTGTAG
TCTATAATCATGTAATTTCTTTCCTCTCCATGCATTAATTTATTTCTATGTCAGCAACTCATGC
TCAAAGATAATGAAGTAACAGTAGCTGGGAGGGTGATGCCTGCTGCTGTAACATAATATAT
AAGCTGTAGAACACCATGGTACTATTCCATCCTAGTAATTATCCCCATTTCCTGTCTTCAGAC
ATATGTCTATGACTATTCACAACTCGGTCATAGAAGTCTTGGTTTTCATTGTTACAGAAAATA
ATCTTACTTTCAGAAATATTAAAAAAAAATTTTTTTTCAGAGTCTTATACTTCTTATATCACTAT
CAAAAGCCAGATATAATAAGATATCTGAATATCTTATTCCAAAAAACGTGTACTCTTAAAAT
CTAGAACTCACGTATGACCTAATATAATCTAAGTATAACTGACTAAACTTCTGTCATAGCTC
AATTGTTAAAGAATCCGCCTGCAGTGCCAGAGTCCGCCTGCAGTGCAGGAGACCTGGGTTCG
ATTCCTGAGTTGGGAAGATCCCCTAATGGCAACCCAATCTAGTATTCTTGCCTGGGAAACCC
CATGGACAGAGGAGCCTGGTGGGCTACAGTCCATGAGGTCTCAAGAGCCAGACATGACTTC
GTGACTAAGACACCAAACCATAACTGATTAAAAATCATACCCTGAGAGAACTGAATGCATG
TGATAAGAGAAAAGAGGTGGTATTTAAGATCTGTCTCATCACAGGTTTTGCTTAATTTTTTTG
CTTGCTTGCTTGGGGGGGTGGGTATGTTTTTACTTTTAAGGCTGTTCATTGTGGAATGTAAAA
AACAAACAATATAGAATCCTGTCCCAGTCCTGCTCTGATAGGTGATAAGATATATATCTTAT
TCTTGGAGGCTAAAACCTTCATTCTCTAAGGGATAAAAATGGAGTGGATTAGATATAATTTG
TTTACTTGGAGAAGTAATGAGGTGAGGGTTGGAGAAGTTGGATTTTCAGCTAAAGTATGTAA
ACAAACTCGAATTTATATAACAATTCTAAAGGAATCTCATGATGTTTTATGATGTTGACAAA
GGAATCCTTACTGTCTACCTAGATATCTTAAATAATGGGAAAAACAATGGTTACCTTTCAGA
AAAAGTTAATAGTTTGAGAGTCTTCCATGTGTTGAGGTGAATTTACTTCCTATCAATTACACC
CTGAATTCAGTTGAAATTGGAAGTGGAAGAGGAGGTCCCTCCTCAACAGAACCACACAGGT
TTATTTGCCTTTTAGCTGCTGAAGTGAGCTTGATGAAACTTTGACAGGGTGTGACATGAGTC
AAAACATGAAACTTCCTGGATTGAGATGTTAATTATGTATTCCATCTCTATGAAGACCTGGT
AACTCCTTCAGAATATCCTCAGATCGAACATGAAAGTCAAGTCGCTCAGTCGTGTCCGACTC
TCTGCAACCCCGTGGACTGTAGCCCACCAGGCTCCTCCGTGGGATTCTCCAGGCAAGAATAC

TGGAGTGGGTTGCCATTTCCTTCTCCAGGGGAATCTTCCCAACCCAGGGATCGAACCCAGGT
CTCCCTCATTGCAGGCAGACGCTTTAACCTCTGAGCTACCAAATTTTTTAATTACTTCGCTTT
GGCAAGAGTACATGGAAAACAAAATATGAAGCAATTACCTCCCTTTGACCTTATTAACCAAT
ACTCTGTAAAACATGTATACATTCTAATTTTCAAACAAAAACTTGAGTACTTATAATCTTTTT
AAAAGTATATTCTAAACACAGACCTTAAAAGTATATTCTAAACACAGACCTCAGTTTGAAGG
ATTTTTTTTCTGTTAGTGAGTGCCATCTGCTGGTATAATGTAAGTACAACTAAGCAAACTACA
ATTTGAGTCACAATTATGGCTCAGCTGGTAAAGAAGCCACCTGCAATGCGCGAGAGTCAGA
CACCAATGAGTGACTGTCAGTTACTTTCACTTATTTATATTGCTATTATTTCAAGTATATAAA
TATAAATGTTCCTGAAAATTAAAGTATTTTAATTTAAATATGAATATGCAATATAATTTTAAC
ATATAATTATCTGATATAAAAGAGTAACTGACTAGTAATAGATTCTCCAGGATGATGTCTCA
GATATTTATATTCTTTGTATGTCTACAGCATTCCAAGGAGAATAAACAATAAAAAGCTTAAT
GAAATTGTATTAGAAATAATATATATTTCCATATTTTTGTGAATTCTCTTTTTAAAGAATGTA
AAAAATAACTTCTTCATTCAAAACTATTTACCAAGAACTTACCCATGTACCAGACACTGCTT
GGGGATTCAAAATACTCATAATACCTATGGCATAGGAACCTACATTTTTATTATACTTGATTG
AAATCAGGTCAGGTTTTTCTGGTTGGTTTTGTTTTTTCCATTTCAGTCCCATGTTGTTTCAATC
TTTATCTTAGATAACAACACATAACTATTAGAAGCTCATATGATAGAGACATCCGGATATAT
GATACAGCAGTGAATAATGTACACCTTTTTCCATCTCAGTAAATATCTTGTATGTATAAAATA
TTTTTTTCCCATGATATTACCCTTCACTACATTCCTAAAAATTAGTTGGTACTCTGCCTACAG
ATTAGAGCCAGGAGCTCAGAACTAGTAACTATTCCATACATGGGGTAGGGTTGGAGAGGAA
TTGATCTGTTATGTGGAAATATATTGTTAGTTATCTTTAACTTTCATTAAAATAATGAAAATT
ATAGCCAAGCCAGTGATGGAAAGGCTATCTACTTTGAACAGTCCTTCTAATATAAGAAAATG
GGCTCATAATATTCCCCTGCTTCAGCTTCTGTATTTCCCCCTCACCCTCAAGATGAAAGGTGA
ACATCTGAATGTAGAATGCAACCCTTTAGTCTTTGAAATCTTGCTCATTTCAGTGGCTTTGTT
TTCTAGCACTCCCCTTACTCAGACACTGTAAACCAACCACACTGAAACGTGTTATTTCTAAG
AGTATACCATGATCTTACTTATCCTATATGTTCTAACATATTTTACCAAATTAAATTTTTATCT
GCCACTCAAACCACTTATATGAAATTGTATCCTGTCCATTATCCCCACCATAACCATTCTAAG
CCTGAGTTTGTACCTATTCTGTCTTCTCACAGAATCTGTATAACCTCCTTAATAACATGTATT
CCTTCTCCTCTGGTATGTACATCTAAGACCTTCCTTCTCTAATTTGCTACAGACTCAGCATCA
GCTTGCTTATCCTTTTGGTCTACTTGTTTAGCATAATATCTGACACAGTCTATGTACTTAAAA
TTATTTGTTGCATGAATGAATGACATTCATTTGAAACAACGGTTTTGAGTTTTAGTTCCTCCT
GTTTTTCAGAGTTCGTTATCTAGCCAAGGAAAATATAACCCAGGACCCTGAGACCCACACGG
TCTCTTTCCTGCAGCCCAATGGCGCCATCTTTGAACCCTCGCTATCAGTTGGAACTGAGGATG
ACATGTTCACCATTCTCAACCTGGCTGTAGCAGTGAGTAGACAAACCGCAAAATTACTGGTT
TTGAAATCTTCTAAAATCCAACTGTAAATAACCTCACAATTAAAACTACAATTAGATATCTTT
TTTTTCATTTTTATAAAAATGCTTTCTTACGCTATTATTTTGAATTACGATCATTTGGAAAGAT
GACAAGATCATAGAATGATCAACTGAAGACTGATTAGAAGACCATTTAGAGTGTATCATTTT
AAAAAGGATTTATGGGGCTTCCCTCGTGGCTCAGTGATAAAGAATCCGCCTGATAAAGAATC
CACCTGATAATGCAGGAGACATGAGTTCAATCCCTGGTCCAGGAAGATCCCACATGCCTCAG
AGCAGCTAAGCCTGCACACCACAACTATTGAGCATGTATGTGTTCTAGAGCTGCGGAGCTGC
CACTACTGAGCCCAGATGCCTCAGCTACTGAAGTCTGCACACCCCAGAGCCCATGCTCTGCA
CCAAGAGAAGCCACCACCACGAGAAGCCCACGCACCACAATCAGAGACTGGCCCTGCTCAA
GGCAACTAGAGAGAAGTATGCCCAGCAGTGAAGACTCAGCACAGCCAAAATAAATCACTCA
TGAAAAATATAAGCAAAATTTGTCAAAAAGTAGGACTCAACTTGAAACTCTTCTTCCTGTGG
CCCACACACCACAGAGAAATGTAATCATTAAGAGATGAAATAATATACTTTTAGTCCTGGCT
AGATTCGGCAGTCGTTTTAAAAATAGCTGGAAATTGTTTTATTTGGTTTAATTCTTGTATTTA
CATTTATTTGTTGTTATATTGCACTTGGTGTCTTAAAGCATGATTTTTTTTTTTGCAGGCTGTA
CCACATCTCTATCCAAATTCATTTATTCAAGGAATACTCAATTCACTTATCAAAAAGTCCAAA

TCTTCCATGTTTCAAAACAGAACTTTGAAAGAACTATTGTGGGGCTATACGGATCCATTCTTG
AATTTGGTTCCATATCCTGTTACCACTACAGTTGGTGTGTTTTATCCTGTGAGTAACAATTAT
AAATCTTGATACTGTTAGACTTTAACTGGATATATATAATCAATCACACTGGCAATTCATAA
GTTTATCATTCATTGTGTTAAACCCTATTTTGGATCTCGGAATACATACCCTAAGTTTTTAAA
AATCTCTACCTCACATGGATTTAGCTTTCCTAGTTGGGAAAATTTACTTATCTGAATTTAACA
ATTAAGTTTAAGATTAAGAAGTGGCCATTTAATCAAGTGTCCACTCCTAGATAAAGATGCAA
TTGTCTAAAATTAGGTTATAAGGTGCTAAGAGTTTGAATACTGTTTTTGGTTTTGTTTCCTGT
CTGAAAAACAAGTTTAAGCTGTATTTTATTTAGCTGTATTTTATTTTTGTTAATTGTATTCATC
ATATCATTCTTAAAAAATTGTTTCAGTGTAAATTTAAAAGTCACCTTACATTGTACATAAGAT
TGATACATCAGCATGTTGTATTCTCTCAGATTTAGTTGGTAAGAACTTCTGTGAGTGAGTGCA
GTGCTCAGAGCTGATAAGAACTTCTATCTACAAAGTCATGAAATGGCAGTTTGGAGATAAAA
AGTGAATGTGGTGATGTACTTAGTTATGGCAGAATGTTCTGAATTACAAATAGGACAAGGTG
ACTATTGCATGGAAAATTTTGCTGTGATTCCATTTGGCACTATATGAGATATTAATTTTATTT
TGCAGTTCTTTGTTATTTTACTTTAGGTGGTTTTTAGGGTTGCAAGTTCATATATCAATCAAAT
ATGAATACAGAAAGCCTTAAGAGAGATAAATTCAGTTGCTATTATTTCATTCACATATTCTT
ATTTCTTTCCTTGGCTAATTCTGTTTTTAATTTTGTACACTTCATCTTAGTAACTACTTAGAGA
TCATAACATTGAGACAATGTCTAGAAATTGGTCATTTTTCTCATAGGTTTTGTTCACTCTCCC
CTCACTTCAATATACCAGGAGTAGTGATCAAAATGGCCTTTCTGGATTGAATAAAACAAAGA
GTGCTAGAATTCGATCATGACAGCTTTGGCATTTGTTACCTTACTTTGTCTTGGCATCTAGCA
CTCATTTTTAGTAACCTTTTGTATTCTGTCTTTTTAAAATATTAAAGACCAATAATTTTGAAAT
GATTGCATGTATATTTAAAATCATATAAAATAATTATGAACAACCACTGAAAATGTGAGATA
ATTACATATTTAATAACTTGGAAATTATGGTTTGTTGTAAAAGTAATTTTTTCTTGCATCTCTT
CAGTACAATAATACTGCGGATGGAATTTACAAAGTTTTCAATGGAAAGGACGACATAAGCA
AAGTCGCCATAATTGACACATACAAAGGCAGAAAGTAAGTATCCAGGCAAAGTGTGTGTCA
CTAGGATACTCTAAGGCAGGCAAGAAACTTATTTACCGGCGGGTAAGGCACAGGCACAACC
TGTGGGACGGAGCTGTTTATATCTCAGCCCTAAAAACTCCCTAGGTCTACCAAGCATTAATC
CTTCAGTTGTGATGACTGGTCACACTAGTGCATTTTTGCTGAGTATCAGCCCCACTGGAGGGT
TCACCTTTATGAAAAAGTTCTCTTACATAAAGAAATCTAAAGGGACTACTTCTATCTCTGCTG
TATAACTGGTATTATCATACACACAACAATGACATAAATGCAAATGAAAATGAGGAAAATC
AATATATCAACTCATCATCTAGCACGTAGAAATACAGACATCTTACTGTTTCTTTTTTAATTG
AGCATGTCAAAAAACATCATTCATTCCAGGGAGATGATTAAATGCTGGGGTCCACCACCCCC
ACCCCCCCGCAGGATCCAGGGGAACCTGAAGGAAAAATGGCGTCAGTGATTGATTTAGAGA
GAGATAAGGAAAGAATGTTGAAGATAAGAAAATAGAGGAGAGAAAGAGGCTGATATTCCT
AGGTTTACATAGAAAGCCAATAAAACTCCCAGACAAGAAGTTTGTCCTGTTCACTGAGGCTA
CAGGTGCCCTCCTGGTCTCCTGAGGGAGTGAAGACGCAGAACGTCTTCCCGTTCAGGTCTTA
GAAACCCGAGCAGATAAATGAATGCAGGGAGCCTCTATGCTCCAAGGGATCAGCCTGAAAA
AGAGAGGGAGGGAGAGGGAGAGAATGATTGACGCGGGGAGACCAAGCTGCTTCAGTGAGC
GAGGCCCAATAGCTTTATTTTTAAAAGGTACTTTTATACCTTGTCTTATACATAGAGGGAAAT
GAAAGATGCAAAGTCATACAGAGTCAGCCCAAACATTACATCTGTTTTGTCTTTATCTAAAC
CAGGATTTTTTCTGCAAACCTTTCCCATAAACAATATTGTGTACATTATCTTCTGGCCTTGGA
GGCCTGTGGACATTTTGTGACCCTCTTTTGATAAAGGCTGCTCAACCAGAAAACTTATTTTCC
CTTGAAATGTTTTTTCTTTATATTTCTAATCTATGTCAGCCTCAGAAAGTATTAAACAAGTTA
CATTTCTCATGGAGCAAAGTGCAGTGAGTTACAAGAAAGAACAAATTAGCTCAAATGTCTG
ATGTGGTTAATTTCAAGGCTACACTTGTTTTTCTTACTTTCCAACTATGTTAACTAATGCACC
CCCAGGTGCACAATGGATAAGAGATATGGGAACTTAGCAACAAGTATTGGCCCAATAATGA
AATCCTACACCAGCACTACTCTAGTAACTTTTAACTCTTTAAAAGGCTCTATGTTTTAGGCTT
TCTGTGCCTCTCACCGTTGGGAGGCTGTAAATAATCATATGTATAGCTGCAAGAGTCTGGAT

ATACCTGTCAAGCAAGCTAGAATGCTAACAGAGGGGGTTTGATTTGAAATATTCCTATCATG
TCCAAGAGACTTATTAGCTATAGCCCTAAGTTGATTTTCTCTAGAGAAAGGTGGTCAGGGAT
AGCCCCCTGTTAATGTCAGAGGAGTTGGTGAAAGTCATGAAATATTAAAACAGACAGATTGT
AGTTTTGGGGTAGATGCTCGAGAAAGCTTAGGGAGCCTGTTGAGTCCTGAAGCCTTGCTTAA
CAGTTCTCTTCCACATGACCTTGTCATGGGTGGGATCTCCCATGGGTGGCTCCCGGCAATTAA
ATGAACCTATAAAGATGAATGTTCAATGAATCCATGTGAACATTTTCATGTCTTGTCCCTTAA
AAATTTTCCTGAAATAATTTTCCATATTAATAATTCAGCTTTTTAAACCATTATCAATGCAAA
AATGGAATTATTATTTCCTGTTCCAAAAATGACATATTAAGCCTGAAAATAAAGTGTACACC
TTCTATGAAGTAGCAGTAGAGTGAGATATGTTCACATACCCATGATTTCCTACTTCTCCGAA
GTTCCTGCCTGTTTATGTGCAGTACACCTAATCTGTCATATATCTAAAATACAAAGAAGCCC
AATAAATATCAGGTGGGGGGAAACTTTTCTCCTCAAACTTGAAATTAGAGTTTTAAGATCAG
TAGTTCCTGACAAAGAATAAAGTGATCAAAATCAAAAGAGATGAAATATTTTTATGATTAAG
TAACCATTACTTCACACTGAGTTTGTCCCTAAAAATTGCCTAAAACAGTAAAATACTCGCTC
ACGTAGACTTTTCATCTACAGAGTTCATAGTCCTTTATTGTTTTGAAAATTCTAATGGAGGAA
AACAAACACTTCTAAATGTTCATTGTAGGGAAATCATATCTCTGTATATTGAGAAAAGTGAT
AATTTCTTCCATTTCAATATTACCAGAGTTTTTGAGGAGTTACTAAATGTTTCAAATTGGAGT
AAGAATTTCATTTAAGATTATGATAAGGTTAACTTAACTGTCTGAGCATGACTGACAGCTTG
TTCTGTTTAAAAAAGAAGTAATAGCTTGCCTTGACATCTTACTGGATGGTATGTTAGACTAA
CAAACCCATTTTTCCATCACCAAAAAGTGGTTGAGTATACAGAGAAACTATCACTACTGTTG
TTTTCATTATTTCCATATATAAATATACACACACATATACATTTTATGTAGATACCAGAATAT
AAATTAATAAACTTCATCCTACTTTAATGCCAGCTCATGCAAGAGTTGAGAGGAAAAGTGTA
TATTGATTTTCTACATGTTTCTAAATCTCTATTAATCAAACATCTCTTTCCTCTTCCACATTCT
ACTCTTTTCTCTCATAATGAGTTTATATCAGTTAACCATTTAGTATCTTCCTCCTACTCAGCTG
GACACTTCAACATAATATGAAATACTCCACTTCTCCCATTGGAACAACAACAAAAAAACCTT
TCTTGAAGTAGACATCTAAATGTCTGATATGTGTTTTTCAATTTTCTTTTTGCAGAATTTTATC
TTTTCCAAGTTGTTCCTAACTACCAAATTTTGGCAGCTTTTATTTTATAATCCAGCTACTTAGT
GTTATCAAGCACTTCCTTTATCTCAACAAGATGCCAGCTTCAAACAATACATAAACCAAATG
ACTTAGAACATTTCTTATACTTAGCACCTGTCACATCTACTCTAATCAATTATTATTTCTGCA
GAAATACTGACTCATAATAGACAATGTAATGTGAGAAGGTGCTCCTGAAATGAGACTGATC
ATTTTATTTTTCTTTTTTTAATTCCTAAGGAATCTCTCCTATTGGTCAAGTTATTGTGACCTGA
TTAATGGTACAGGTAAGGACAATCTGTTTTGCGGTAATATCACAGTGAAACCACCTTCTTTCT
CCCACAAATCCTCCATCATATCTACAATGTTCTGGAGGCTGAGGATATGCATTTACTTATCTT
TGTATCCCTACAACATAATTCAGAGTCTCTATACTTATGCTGCTGCTGCTGCTAAGTCGCTTC
AATCGCGTCCGACTCTGTGCGACCCCATAGATGGCAGCCCACCAGGCTCCCCCATCCCTGGG
ATTCTCCAGGCAAGAACACTGCAGTGGGTTGCCATTTCCTTCTCCGATGCATGAAAGTGAAA
AGTGAAAGTGAAGTCGCTCAGTCGTGTCCAACTCTTAGAGACCCCAGGGACTGCAGCCCACC
AGGCTCCTCCATCCATGGGATTCTCCAGGCAAGAGCACCGGAGTGGAGTGCCACCACCCTCT
CCACTATACTTATATATAGGCTTATTAAATATAAGTCTAATGTGATGGTATTTCTGAAGATTA
ACCATTGAAGATAAGCCTTTACTACATTCCTTGGGGAATGTTTGCTTCATAAAAGCTTTACTC
AAGTGAACTCCCTTCCTGGCCACAGAAGTAAGGGGGTAGAATATAAGTAGTGGTCACTGTTT
GCATGTTTTGGGAATACTATATAAGCTGTGACCAACCGAGACAGCATATTAAAAGCAGAGA
CATGACTTTGCTGACAAAGGTCCATCTAGTCAAACCTATGGTTTTTCCATTAGTCATGTATGA
GTGTGAGAGTTGGACCATAAAGAAAGCTGAGCGCAAAACTGATAATTTTGAACTGTGGTGT
CAGAGTAGACTCTTTTTGAGGGTCCCATGGACTGCAAGGAGATCAAACCAGTCAATCCTAAA
GGAAATCAGTCCCGAATATTCATTGGAAGGACTGATGCTGAAGATGAAACTCCAATACTTTG
ACCACCTGATGCAAAGAATTGCCTCCTTAGAAAAAATCCTGATGCTGGGAAAGTTTGAAGG
GAGGAGGAGAAGCGGACAGCTGAGGATGAGATGGTTGGATGGCATCACCAACTCGATGGAC

ACAAGTCTGAGCAAGCTCTGGGAGTTGGTGATGGACAGGGAAGTCTGGCATGCTGCAGTCC
ATGGGATCACAAAGAGTCGGACAAAACTGAACTGAATGGTTTAAAACTTAAAAGAATTAAA
AAATCAAGATAAATACAAAAGTAAACCTTGTGTTCAAGTGGAGGTTTCAATATGAGGCAGA
ATCAGAGAAGTTCAAGGCTGAACACAGATTTTGAATGAAATTATTAAAATGAGAAAGAAAA
TAACAACAATAAAACACAGTTGCATCTGTACTAACCATGAGAAGTATATGCAAAAATAACA
TTGTGAATTAGAGCTGGGAAGAAAGAAGGTACTAGGACTTTAGATTCAGAGAGTGATAGAT
CCAGTTTATTATTCTCCTTAAAGAAAAGCAAAGTACACATATGCCTATTAGTTTAAGTATTAA
AATTTTTAAAAGTAATAGGAATAAAATATTTAAAATCAAAGGTGATCTTTCAGCTACTAAGA
AGCAGAAGCAGAATATTATGTGTGTAAAGTTGATAAGGCAACACTTTAGAAGAATGTAACA
CAATGAAGAAAAAAATGTTATACATAAAGTGCGTAAAAGAAAATAAGGAAACATAGAGAA
CGTTCATTTTCACAAAGGTATTGCTGAGTTTCACAGGTTTAATTTTGTTTACTCTGTGTTAAA
AAAAAAACACACACACAAACAGTAAAACCACCGCTGAATTTGCTTTGCAACACTTTGAGTC
ATGGTGATGGGTGAAGAAATCCTTTCCAAGTAGTTCATTTCTGCAAATAGTCAACTGTCCAA
ATACCACTAACTTTAATTTCATAAGCATACGCTTCAGTCCTTAAAATAGAAATATACTGTGCT
TCTCCTAAATAGTAAACTATTGTTTATGGGCTTAAAATACATTTAAATCTTAGTCTAAAACAA
AAAATAGAGCAGTTTTAAAACAGTAATAAGAGGATCCATTAATGACAGTAACAGATACATA
GAATCAGGCATGAAATTCAAAGCATCTAAGAAGAAAATCACAGTAAAACAAACTAGGAGCA
AAGGTTGATTGGATAGACTAGGGACTCTGATGGGAGATTCTCTTGGTCATAGTACTAAAAAA
AGAGACTGCTATTGCTGCTGCTGCTGCTGCTGCTGCTGCTGCTGCTGCTGCTGCTGCTGCTGC
TGCTAAGTCGCTTCAGTCGTGTCCAACTCTGTGTGACCCCATAGACAGCAGCCCACCAGGCT
CCCCCGTCCCTGGGATTCTCCAGGCTAGAACACTGGAGTGGGTTGCCATTTCCTTCTCCAATG
CATGAAATTGAAAAGTGAAAGTGTTTGTTAGCCATCCGTATGTCTTCTTTGGAGAAATGTCT
ATTTAGTTCTTTGGCCCATTTTTTGATTGGGTCGTTTATTTTTCTGGAGTTGAGCTGCAGAAGT
TGCTTGTATATTTTTGAGATTAGTTGTTTGTCAGTTGCTTCATTTGCTATTATTTTCTCCCATTC
AGAAGGCTGTCTTTTCACCTTGCTTATATTTTCCTTTGTTGTGCAGAAGCTTTTAATTTTAATA
TATATGGAATTTAGGAAGATGGCAATGACGACCCTGTATGCAAGACAGGGAAAGAGACACA
GATGTGTATAACGGACTTTTTGGACTCAGAGGGAGAGGGAGAGGGTGGGATGATTTGGGAG
AATGACATTCTAACATGTATACTATCATGTGAATTGAATCGCCAGTCTATGTCTGACGCAGG
ATGCAGCATGCTTGGGGCTGGTGCATGGGGATGACCCAGAAAGATGTTATGGGGAGGGAGG
TGGAGGGGGTTCATGTTTGGGAATGCATGAAAAAAATAAATAAATAAATAAAATAAA
TAAAATCTAAAAAAATAAAAAAAAAAAAAAAAAAAAAGAAAAGTGAAAGTGAAGTCGCTCA
GTTGTGTCCGACTCTTAGCGACCCCATGGACTGCAGCCTACCAGGCTCCTCTGTCCATGGGA
TTTTCCAGGCAAGAGTACTGGAGTGGGGTGCCACTGCCTTCTCCGAGAAAAAGAGACTAATG
AAATGTAAATGGATCCAAGGTGATTTTTAAATCATGATTTAGAAGTACTTCCTCTACCATCTT
CATTAAGTTTAAAGACATAGAATGAAAATATTTATTTGAATTTATTTTAAAAAGGGAGCCCT
GCTGGCACCAGCTATGTTACAGTCATGGCCCTTGGTAGTCACCGGTGTCATTCCGACATAAT
CTTCGATGATATTTCTGATGGTTTTTAAAGACTGTCAATGTACTCTTTTCCTTTGATGGCTAA
GGTGTTGGGACACCAGTTGTCGGCAAGGCCCATTCGGGGACCTATTTGGGGAATTTAATCTT
CACTATCCCATTCTCTCAATGTCTCTATCAGGTTCACGGAGATCCTCTGTTATATTTCACCTAT
CACAAGAATGTATGTGAAATGTAATAAAATCACAAGGTCAGAGCAGTTACCAGAAAAAGAG
TGTGGTATTCAGAACTGAAGGATAGGTCTTACTGTTTTGAACAAACATGTTCAGTGGTGGCT
CATGTTTGGTGATATTCATTTGTGGAGCAAATGGAGGAATTATCTAGCTATTTTTTCTTACAT
CCAGTGTGTACTATGGAGCACAACCATAGTAAAGATTTGCTAAGCTCAAAAAATGCCCTTTT
TAGTTTGATGTGTATACTTTCCAACCATTTGTGTGATATTGTTTAGCAATTTGAAAAATATTG
ATCAGCCATAAGGATAGGTGAATGGATCACTATTAAAAGTACGAATAACATTTACAAAAAT
GAGTTATAAAGAAATCTCAAGATTAAAAAATTGCTACTCTACTGATAGATGGAAGATTTAAA
AACATAAAATCGCAGTTTTAACAACGTGATTTTGACATTTTAAAAAACTTTTATGGAGTGGG

AAAATTTAATATGAAACAGAAAGCCAATGACTACATATAGTATACGGACTTATTAGATATGA
CAGCTGCTAGCCACATGTCACAATTCAGCAGCGCTTGAAATGCAGCTAGTACAAACTGAAAT
ACTGTAATTATAAACACTGTATTTTCAGATTTTTGTTAATTTTTATATGTTAAAATGATGGTA
TTTTTGATATATGGGGTTAAACAACATATACTTGATACATTTTTTTTTCTCATCTTTAAAATGT
AGTTACTGGAGAATTTTAAATATACATTTTGCATTACATTTCTCTTAAGCATGCTGGAATCAA
GATTTCTAGATTTGTCTAAGACACACTCTTTCCATCTAATCCCTTGCCACTGATTTGGTTTTTA
AACAGATGCAGCCTCATTTCCACCTTTTGTTGAGAAGACAAGGAAATTGCAATTTTTCTCCTC
TGATATTTGCAGGTAAGAGAAAGACAATGATGTATTTCTCCAAGTCAGAATTCTTTCTCTAA
GTGATAAAACACAGACCAAGAAACTCATGCTTATTAATCATGTTATTTGAGAAAAATAAAAT
ACTTTTAGGGGCTTTTTCCTGATTAAGAATAAATACAATGTGAAATTCATGATGTATTTATGC
CCTGGAGAAGGTAATGGCAAACCACTCCAGTATTCTTGCCTAGAGAATACCATGGACAGAG
GACCATGGTGGGCTACAGTCCATGGGGTTGCAAAGAGTTGGACACAACTGAGCCACCAATA
CACACACACACACACACACACACACACACACACAATGTATTTATGAGTCTTTAACATTTGATGT
AGAAGGATGTTGTGTATAATGCAAATCAGTTTCTTTTTGTTATTTTTAAGAAAGAACTACTTT
TTTCATTCTTTTACTAAATTAGCAGTAAGAGTGATAACAGTCAAGTCTAGAAAAGCATGCCC
AGTCTTTGTAAAGAAGAGGATACCACAATCACAAAACTATGTTCATGTGCCCCACTGACAAT
ACAGCATCTTTTCAAATTGACGGGGGCCCAGTAGGTATCACACAAGTGTTGGCGCCTACACC
TGTATCCTTGAACGCTCACCTGCTTTCTTAACACTGTTTGGGAAAGCAAGCATAATATCTCTG
AATCTTTTTCCTTATCTGTATAATTGAAAAGAACAGCATTTCTTCAAATAGTCATCTGGGGAC
TAGAAAATATTTAATCTATAAAGGAAACTAACTGTGAGGAATGCCTTAATTGTAAATACTCC
ACAGTAATTCACAAGCAAAAATTATTCTCAGAAGCGTTTGAAAACAAAGATAAGAATAGCT
CATACTCAAGTATTAAGGTCTAGTGTGTCTTAAAGTTTTGCAAGTTTTTTTTTCCTAAAAGGT
AAGGATTCTAATAAGATTATATCTAATTTTTCTAATTTAAGGCAGCAATAGTATAAATTTAA
ATATAAGAGAAAACAAATCTCCACAACAAAGTTCAGGTTCCCAGAATGAAGCTCCTTCTTAT
CTGTGTTCAGGTCTATCTATGCTGTGTTCGGAGCTGAACTGAATCTGAAAGGAATCCCTGTGT
ACAGATTTGTTCTTCCATCCTTGGCTTTTGCATCTCCACTTCAAAATCCAGACAACCACTGTT
TCTGCACAGAAAAAAATCATCTCAAAAAAATTGTACCTTATATGGTGTGCTAGACATTAGCAAA
TGCAAAGAAGGTGAGTACAAGTTCTCAGTGTCACAATCCTTGGCATAATTTGCATTATCCTTT
AAAATGTAAACTTCACCCAGGATTCCTTTCCACCAGAAATATAAAGTGGGAAAGCAATAAG
GGCAGAAAGTAAGAGTGATGGTAGGATATTTTTGTTTTGATGTTTGTCTTGCTCTCAAGGTCT
CAGTTGTTTTCATGTTGCAAAACTTAGTTTTTATTAGGTATAATCATGCTTATTGATACTTCCT
GTGCTACTAGGAAAATTTCTCTTTAGGTCTTTTGCTCATTTTCAAATTGGTTTATTATTTTTTT
TGAGTTGTATAAATTCTTGTATATTTTGGATGTTAATCACTTTTGGGATATGTGTTTTGGAAG
TATTTTCTCCCACCCACAGGTAACATTTTCATTTCTGACCATTTCTTTTGCTGTGTAGAATGGC
TGTGCTCCTTTTAGATGTAGGGTAAAATGAAAATAGTTTGCAGACTACTTTGAGCATCCCAA
CTTGTCCATCTGACATTTTTCTTCTTCTACCCTAGAATCTGTAAATAAGTATATTTTAGGATGT
CTCTGAAACAAAAATTGATTCAAACATGGCATCTGGAGTCTATCTTATTTTGCTTACCTTCTT
CATGTTAGAGTGGGTGAGAACAAAGAAAGAAAAGCCTCTTTAAATGAATTCTCGGTCCAAA
GACATTTTACAGCCTGAAACATGAAGGCATATGGTTGTAACTTTGTGTCCAGTAGCTAAGCT
TTACATACTGAAAACACCTAAGGTGAAAATAAACACCTAAGGGATGAACAACAGACATTAA
CTAAATAGTCAGTAGGATGACGTAGAAGTTTACATTTTATTTTTGTTCAAAAGTGATCAGAT
ACAATAATTTTCCCTCACTCTCATCCCCTGATAAGATTTCAAAGCCAAATTTGTGTTTCTTTTT
TTAAATTAAAAAGCTTTGTTTTCAAGGAAATCTTTCCTAAGTGGTGTATGAAATGAGGAAAA
TGATTTACCCTAACTGTCTCTTAAAACTTGCTTTCAGGAAAACCTGTGTACATTTCACTTCCT
CATTTTCTACATGGAAGTCCTGAACTTGCAGAACCTATTGAAGGCTTAAGTCCAAATGAAGA
AGAACATAGCACGTACCTAGATGTTGAACCTGTAAGCAAATACTTATACTTTATTGATCAGA
TTTGTTTTTGTTTTGTTTTGTTTTGTTTTGTTTTGTTTTAGTTTTTTATTTTTTAAATTTTAAAAT

CTTTAATTCTTACATGCGTTCCCAAACATGACCCCCCCTCCCACCTCCCTCCCCATAACATCT
CTCTGGGTCATCCCCATGCACCAGCCCCAAGCATGCTGTATCCTGCGTCAGACATAGACTGG
CGATTCAATTCTTACATGATAGTATACATGTTAGAATGCCATTCTCCCAAATCATCCCACCCT
CTCCCTCTCCCTCTGAGTCCAAAAGTCCGTTATACACATCTGTGTCTTTTTTCCTGTCTTGTGT
ACAGGGTCGTCATTGCCATCTTCCTAAATTCCATATATATGTGTTAGTATACTGTATTGGTGT
TTTTCTTTCTGGCTTACTTCACTCTGCATAATCGGCTCCAGTTTCATCCATCTCATCAGAACTG
ATTCAAATGAATTCTTTTTAACGGCTGAGTAATACTCCATTGTGTATATGTACCACAGCTTTC
TTATCCATTCATCTGCTGATGGACATCTAGGTTGTTTCCATGTCCTGGCTATTATAAACAGTG
CTGCGATGAACATTGGGGTACACGTGTCTCTTTCCATTCTGGTTTCCTTGGTGTGTATGCCCA
GCAGTGGGATCGCTGGGTCATAAGGTAGTTCTATTTGCAATTTTTTAAGGAATCTCCACACT
GTTCTCCATAGTGGCTGTACTAGTTTGCATTCCCACTAACAGTGTAGGAGGGTTCCCTTTTCT
CCACACCCTCTCCAGCATTTATTGCTTGCAGATTTTTGGATTGCAGACATTCTGACTGGTGGG
AAGTGGTACCTCATTGTGGTTTTGATTTGCATTTCTCTAATAATGAGTGATGTTAAAAATGAA
GAAAAAATTAAATTATCTCCTTTCTATCAATCACATTTAGTCACATTTATTGTACTACATTTG
TAATAAATTACTTATTTTTACTTCGAGGAAAATTACAGTGATTTCTTCCTGTTGTTGCTTATG
AAATGTCAAGACAATTTTAGTTTTTCAGCAAAGGTCAGGAATAATGAAAATCATTCTATTTG
GATTAAAATGATTTTACATGTTACTCTTTCAAATGTACTTTATACTTAACTGCAAATATTGCT
TATATGAAGGGACCCTGTAGATTATGAGGGATTAAAATTTTTTATGGGTATACATACATTTG
AGGTAAGATCAAATATATTTCTTAGTGTCAAGAAAAATCTATTAAGTCACTATAGTTTAACA
GTTTCAATTTTTCATTTATATTAATATTCCCCTTTTCAACAGATAAATGAAATATCAGGACTT
TAGTAGTCATATTTTAGTTTAAATGATGACAGAAGTAACATTATAATTATACTGATGATGCA
TGGATTCTGACTGAGTACATATTTAAATTCCAGGATATATTTTGTCAAATGAGTTTTAGATTT
CACTACTGTTGATGTCGTTACTAATTCCCCAAAATCAATTCAAGTAAATGTCACAAATCTAA
CTAAACCTTGACATTAGGTTGGGCAACGATATATCTGCATACACACATGCATATACTGATGA
AATATAATTATGGAAAGATACATGTGTAATATTTAATATGTCTGATTAAGTCCAAATAATAA
ATAACAAATTTCACAGGTTCAAGTAGTACACTTTCACCTTTTAGGCTCTACATAACAAAAAT
AAGGTCTGGATATTATAAAAGAATGTTTTTCAAATAAGTTGGTTATTAGTTTGTTCTCTTTTT
AGATAACTGGATTTACTTTACGATTTGCAAAACGGCTGCAGATCAACATACTGGTCAAGCCA
GCAAGAAAAATTGAGTGAGTTTCTTAAACAAGTTTCATTTCGATTTTTCAAATTTTCTTGTAA
GATGCCAGGTATATCTATCAAAGGAGATGGAAATAAAATTTCAACTTCTGTTCGGCTTAATG
TCATTAAATAATTATTAAATACTTATAACTGTCATTTGCAATTTAGAATTACATCTAATGTGT
AGTGAAATGGTACTTACCCATATTTTGGAATGAGTATTTCTGGTAATTTCTCTCTTTGCTAAC
TGTCCAAATATTTTTATGACAATAGTTTCAGCATTCATATTTTAGTTTCCTGGGTATAATTTGT
TGAAAGGGAAAATACATACATAGAAAAATTCTAATGAAAAAACCTGTGAGTCCACCTTTCA
CTGCACGGGACACTGGTTTGATACCTGGTACGGGAAGATCCCACATGCCTCTGGGAAACTAA
CCCCGGGCACCACAACTGAGCCTGTGCTCTGGAGCTCTCGAGTCGCAACTACTGAGCCCAAA
TGCAGCAGCTACTGAAGCCTGCGCTCCTGGAGCCCCTGCTCCACAACAAGAGAAGCCTCTCC
AATGAGAAGCCCATGCCCAGCAACTAGAGTAGCCGCTGCTTGCCGCAACTAGAGAAGGCCC
ATGTACGACAACGAAGACCCAGTGCAGCCAAAAATAAATAAATGTTTTAAGAAATCTGTAT
TAAACAGATGTCTATGTAGAAAAGATATCTGAGAAACAGCAATATTAAAATTTAAATGCTTC
ATTACAGGGAAAACAAAGCAAGTGACTTACCCAGGTTGTAAAAGCAACTAATTTCTGAACA
TTTATTTTCACTAGTAATTACTAATATCTGTCATATGTGTATAGCATATTAGTATATATTTTCA
TACTTATTTTCAATGTTTATTACAGTGCATTAAAGAATCTGAAGCACAACTATATTGTCCCTA
TTCTTTGGCTTAATGAGGTTAGTATTTTTATCTATTAGTCACTAAAAACAGTAAGCTTCTTAA
TATACAGAAAAAGTTTTTGATGTTTGAAAATTAAAATATATATACACACACACAGTATTTGA
ACTTGTCTTTCCATTGTATCTATGGGTGAATTTATATATTATTAATTATATAATTAATTATTTT
TTAAAGCAAGTCATACAGAAGTATCAGGATTATGTTAGACTTAAGTCTAACTTTTATGAAAA

TAAAAATACTTCATAGTGCATTTATTAGATAATTTTTCTTGTCATAGTGCATGTGTTAGGAAA
GAGTCATGAAAATATTAAATTCCAAATAACTCAATATGAGCCTTGTAGTGCTAATAAAACCA
CCCAAGCTTATAATGGGCAAATTAGAGTAGATGTAGGCAGTCTGTCTAGTGCCTGACAAGAT
ATTTTTAATATAAATCATGGAACCGACAAACCACAGAATTAGGAAATGCAGTGCATAAGCCT
TTAGAGGATAGAAACAGTTGAGCTTTGCCATTTCCCTCTGTCACTAAGAAGCAACCTTTGGG
AAACTGGAGTCTTACTGAAAGACTCCAGTAAGACTGCTATACTGATCAATGAAATCAATATA
TACAATAGCACTAGATAACATGTTTAAAGGGAGGAATATTATCTTTTTCTCTCTAAATATTCT
ACCTGGGCATTTACTGTCTTCTTTGACATGCAAAGAGAATTCCAACAGGCTTGTCTTGCAGAT
AAAGATGACTGACACCTGTGTGCTGTTTTGATAAAGTGATACACATAAGTGGAGAGTTTTGA
GTATTCTGCTCTCAAATCTATTCAAATAACACTATTTATTAACTTGATTACAGACTGGTACCA
TTGGTGATGAGAAGGCGGAAATGTTCAGAAAGCAAGTGACGGGGAAAATAAACCTCCTTGG
CCTGGTGGAAATGGTCTTGCTCAGTGTTGGTGTGGTGATGTTTATTGCTTTTATGATTTCATA
TTGTGCATGCAGATCAAAGAGAGTAAATTAAGTAAGTATATAAGAAACCATATGTGACTTCA
GTAACATTAAATATTACCTGTTTTCATTTTATCAAAACAGTTATAAACTAGGCTTCAAATATT
AATGAACATGTCTAGTCAGCTATCATTTTTAATACATCCTTGAAACTTTGAGACACTTAATAT
AAGTTAAAATGAAGACTGAATGGGTGCTTAGGCTTATCAGTATTAAACAGATCACTATATTT
CATCTTTATTCTGGGAGGAAATAGGCTAAGCTGTGAAGGTGTCACCTTCAAAAAGTTAGAAT
GCTGCACAAAAGCTATACATGTACTCAATTTTTATTTCTTAATTATTTCTAGATAGTTATTTTT
AAAATCACAAATAATTTATCTGACTAGGAAACATGTGAAACTGAAAATGGCAGCTATTTGG
GGAGACAAATTTATAAAGGAAAAGAAAATAAAAACTAAAATTAGATAAGCTATAAAATGAT
GAGAATTAAAGAGTCAATAAATGGTAGAGATAAAATCAGATAAAAATATGCTATTTTTAAA
TGCAAATAGTGTATTAAATGGGCTTCCCTGGTGGCTCAGAGGTTAAAGCATCTGCCTGGAAT
GTGGGAGACCGAGGTTCGATCCCTGGGTAGGGAAGATCCCCTGGAGAAAGAAATGGCAACC
CACTCTAGTATTCTTGCCTGGAGAATCCCGTGGAAGGAGGAGCCTGGTAGGCTACAGTCCAC
GGGATCACAAAGAGTCAGACACGACTGAGCGACTTCACTCACTTAAGTGTATTAATATAAA
AACAGAGCTGCTGCATATGGACATGCTGTCCAAAATCATGACATGTACCCTAGATGTTCCAT
GTGAAAGCCCTGATGAATGATTATAATTCAGGAGCTAGAATTAAATGAGTACAAGAAAAAT
AGAATAAAGATAATGTTGAACTCACTAAAACCAAATAATTAGAGTGTAAAGGAAAAATCAG
ATAAATATGGACGGATGAGTTTTAGCAAAACTGAAGAGGTGGTTCAGGCCTCTGTGTTTTAA
ATATTAGTACCCGTGTCCCCTTGTACATAAAAGAGTGTATGCCAGACCCTATTTACTCTAAAT
GGAAGCCTTTCTTTAATAATCATAGGTGGCATTGGTATGGCCTGAGGGGAGTGAATTCTCCA
CTAACTCATAGGTTCTATCTGCCGTTCCTTCCCTGGGATATGAAAGAAAAAAAATGAAGTGA
AAATCAATTGGTAGCTTCCTACTATTTTCTACCTCATAAACTGTAGCTCACCAGGCTCCTCTG
TCTATGGAATTCTCCAGGCAAGAATACTGGAATGGGCTGCCATTCCCTACTCCAAAGGACCT
TCCTGGCCCAGGGATGGAACCCGGGTCTCCTGCATTGCAGGCAGATTCTTTACCATCTAAGC
CACCAGGGAAAACCAGAAGAAGAAAAAATATATATATATATGTATATATGAAATCTAAATT
AAAGCAAGGACTGATGCTGAAGCTCCTATACTTTGGGCCACCTGATGTGAAGAGCTGACTCA
CTGGAAAAGACTGAGGGAATGAAGAAAAGAGGGTGATAGAGGATGAGATGGCTGGATGGC
ATCACTGATTCAATAGACATGAGTTTGAGCAAACTCAGGGAGATAGTGAAACACTGGGTAG
CCTGGCATGCTCCAGTTCATAGGATTGCAAAGAGTTGGACATAATTTATCAAGTGAACAACG
ACAGCAGCAAATTGCTCTGAAGAAAACAATTCACAATTTTAAAACAAGTCTTCTTATTCCAC
TTTCAATCAGACTGCTGACAGTAAAATGTCCTGCATAGAAATTCTGGAGCTGCCACTGGCAA
TTATTGACCTTAATGTGCTCAAATCATGCTATTTTACATGAATTTCACAGCTAATCCCTAATA
AAAATTTATGATTTTCCAGTAGCCATGGATCTATCAGCATCCATTCTGTTTTTTGAAGACATT
ACAGAAAACTAAATTTTAACAGTATAAAACTGTCTATTTTCAAAGTGTGAATCAGTTAAATT
GAAGATTTAAAAAACAAATTGGCCTTTTTGGACCAATTTGTGTGTGTGTGTTCGTGAAATTG
AAAACAGACTGAGGAAGGCAACTCGATCTTTTGTTTTGATGCTTTCTGCCTATATATATATTTCC

TTAAAAGGAGACTGAACAGTTCTGCCTACAGTCTACTTGGACACAATATATTCAAAATGCTA
ATACATTCATGTTATAATAAAGGAAATATGGCATCACCTTTAAAACCTGTCATCAATCCAGT
AACCAGTTATGTTTAAACACAGTAATTCATATGACCATTTATATAACAGACCAAAATATCAC
CTATTGAAAAGCCACGTTAAAACAAAAACTTTTTCTAGTGGGAAAATAAGCTTTTCAAAATG
AACTATAGTAGTTATAAATTTTGGGGTATTATTCTCTATTAAAATTATATAACCTATTTCATC
AATGCTTAATGAGCCCTGAATATCCGACATGTTGGGTCTCTATGTAGCTTGGGCTTCCCTGGT
AGCTCAGCAGGTAAAGAATCCGCCTGCAATCAACCCTGGTTTGATTCCTGGGTCAGGAAGGT
CCCCTGGATGAGGACATGGCAGTCCACTTCAGTATTCTTGCCTGGAGAATCCCCATGGATAG
AGGAGCCTGGAGGGCTAGAGTCTATGTGGTCACAATGAGTGGGACACAGCTGAGCGATTCA
GCAGAGCTATGCAGCTGTAAGTGAAAGACTGAAAACTTTCCAGAGTGTCTTTATCAGCAAGT
ACATTGGTTTGGCTTAAACTTCTGACTGTTATAAGGAAAGTAACTGCTCTTTATACAACAGTA
TGTCTTTTTTCTTTACAAATAGAGAAGAAAAATTTTCTAGGTTGATTACTTGATTTACTCGAT
TTAGAAGAATCACAGATGTAGATTTCCTTAAACAGGCCTCATGTCGAATACTGTAATTATGC
CTTTGGGAAGATTTATAAACAACTGAAAAATAAGGAGACGCTGTGCAGAGGAAATAAAGTA
AAGACGGGAGAGTGAAGGTGTGGATTTATTCAATAGATCACAAAAAGAATGAGCCAACAGT
CTTTTCAAAAGTAGCAGGACATACACCACTAAATGGAGGATGGTCTGAAGGGACAGCAAGG
AGTGGAAATGCTCATGCAGAGCAGATTTAATGAGTGACCCTCCTTATTTGACTGCGTGCAGC
TCTTGAGAGCTGGGAAGGAGCCTTCCATGAACCCGGGAATGGGAAAATAACTGCCTTATCC
GTGAAGTACATCAGAGAAATTTTCTCTAGAGCTGGAGAAATTGGGCTGATTTCTAGAAAATA
TAACATCTTTAATATATAAAATTTGAGTCTTAACTATTTTTCCATGAATTTCTGATATGTACCT
TACCTTTCTTCTTTCCAGGGAGCAAAAGAGTCTCTACATTTATGCACCAGCTATGTCAGGATC
TTTCTTATTATCACCTTGCAAAAGTCAAGATTTTCATGCTTTATTTTTGCAAAACACACCTTAT
CTTACAGTTCAAGAACTGGTGACACTCCCTCCCACCCGCCACCCATAAGCAGCAGCACATTT
CAAAAGGATTATTAAGACATCATTAAAATCCACACTTCGAACAAAAAACCAGCACTTAAAA
AAATTCAACATATTCACAATGGAATGGACTTCAGTTTCTACAGATGTGGCTTGAGCGTGACC
CACTTATTTTCACTTGGTACTGATTCACCGATTTATTCCCAATGCTAAATTCAGCAGCGAGAT
GGTCATTCTACACAAATCCTGGACCCTGAACTAGCCTTCCCCATCAAAAGGAAAACATCACT
ACCCTCATCAGTGTCCCTGCAAAACACAGACCCAGTACTATCAGGACATTGCCTCTGAAGAG
CAAATATTTTGAAAGATATTTGAAAACATAAACTAAATGACTGGCCTATGAACCATTTATTA
TACATCTCTCAAGTTTTTTCCTCTATGGAATCCTTTGTACAGCATCAGCAAGGCATTATAACC
ATGGTATTTATAACAATATGTGCTTGTATTTTTGTCAATGGTTATGCATTTGGACGTGTTTGA
GAACATTGCTATCTTCCATGTTCTACAGCTCTTCTGGAAATCTGAGCAAATTTTCATCTTTCT
ACTCAGTTGCATACAACTTATGCTTGGCACCTCCAGAATTCTGTGCTAGTACTGAGAGATGT
AAATGATAATACAGAAATTATTGTGTGGAAGATTACTAGCTGTAGAACGTGCATTCATAGTT
ATTTGTTGTAAAATTTCTTGCTTTCATTGTCACCAGAAAGACTGGTTTTGAACATTAAAAGAC
GTTCCTTAAAA

>NC_056057.1:C41968317-41861053_(*CD36*_GENE)

GCTGGAAGTCTCAGGATGTCAATGGCTTCACATGTCAGGATAACCTTAACGACAGAC
GAATGGTTAAGACCCTGAGCCTCATTTCTGAGTTCTCAGCTGTGGAAATCATGTGTT
TATTTTCTGCATCTCCTCCTACAACAGTCACTCTGAAGCCAGTTTTAAGATCCTACAT
CTGGGCAAGCAAGCTTCTCTGAAAGTTGGTGAGCACAGTTATTTTTGTTTAGGGGTG
TGGCTATAAATGTCTTTGTAATTGATGGACTGTCATCAAGTAAACATTAATACTTGTT
ATTTCAAACTCCTTAAGAAGCTAAATACTAACTGGTGTAATAATTTAGTTTATGATA
TTGTTTAAACAGAGTTATAAGCTTTTAACTAAATGGACAGACTTTTTAAGACAAAGT
TTTCATTTCCACTAGTCTCTCAACTGATCCTATGAAATTTCCATTTATACATTCTCATA
TCTTTTATTTTCAAAGTATCAGAAAACTTAACCCAAGTATTTTTAAAAGTACAATTTT
GGTGGAACTTTTTCTTCCTTAAGATTCTGAAGTTGATCTGTCATGTCCTGTTCTTAAA
CTGATTTCTTATTTTATTAATTTTCGTATGTCTTGTTTTAAAAATGTCACTCTTTTGAA
AAGGATGCTCTCAAATTCCGAGCAGTTCAATTTTCTGCTTACAAGTTGAAAAAACAT
GTTTTTAATGAGATTAGAAGTTGAATGACCTTTGTTCATGGGACCTAAAAATGTTTTT
TGGTGTTTTGTCAAGACTGACAAGTATATTTAAGAAATAATCTAAAAGACTCAATCC
TCTTAACAAACTCTTAGAAGAAAAAAATATTTCCATAAATAAAATTACTGATCACTA
GTGATCACAGAAATTATACTGACTGTTGGCACCAGATATTTGATGGAAGGGAATTTT
GTTTCATGGCAAAGTTTTTGTTCTATGGTGTGTTTCATACTATGGCTATATAAGACCA
GGAAACAACAAAGAATGATAGAGGATGACTTATTCAGACAGGAAACTAAGGGTATG
CTTAGAGACAAATAAGTATATAATGATATACTTCTTTAAAGACAACTGCTGATACCA
AAAAAGCAGTTCCATCTGACTATGCTATGTTACAAAGTCTGAGTATGAAATGAACAG
GCTTCTTTACCTTTAAAAAAAGTCACCTTCCATAATTCTTTGGAGAATGGTCTCTTGG
AGCCGTGCTGAATTCAGGGGAAAGCACTCTGGACTAATACGTTTTAAAGTATTAACT
TCTGTTCACTTCCTATGCAAGAACTCAGGTAAGATCTGGATCAGATGAAGAGAAACA
AGCAATCTGTCCTGTTCTCAGGTATACCTGACTAAGTAAAGAAGGAGGGTTTGTGGG
ACAATATATGAGACTCCTGCAATCCTGGCAAGGGGAAAGGAGAAATACTTTCAGAG
GATGTACAATACGAACTGTAATTATTTGGAATTTGATCCTTTTGTAAAGGACAATGA
CGACTTTGCAATGAATTATTCTGAGAATTACAGAAAGAAAGCAAACACTTGAGAAT
AATAAAAAGATTTTCACCTGGGGAAAAATGGTAAGCTCCCAAACTCAGAAGAATCA
TTGTTAGGCAAAAAAGGAAAAACAAAGAAGGAAAAAAGGCTTTAAAATGCTAACA
AACGGGTGGCAAAGACCGTATAAGATGGCAGAGTGGATCAATCAAATGAAAGAAT
ATGTTTTTCTTGGAATGAAGAAATACATAATAATGTACTGCCCTTGAAGTGATTAGG
ACTAATTTTGCTTTTAGCATAGATTGTTCGTTGAGATCACTAGCTAAGGAATTTAATA
TTCTTAGTATTTACAAACTAAACTTCACAGTCGAATTTTTTAAGGAAAAATTCACAA
AATTTTAAAATGAAAAAGAAATGCTTTTGGATTGCGGGGGACGACCCGTGAAGGGT
TAAGTCTTGGGAGCTCCCTGGCAGGTATGCCAGGCCCTAGGACACGTGCCTAAGCTC
CCTGTCCCGCCACCCTCAAGAGTTTTTATAACCCTTAAGGCTCCAAGATGTTTGGTTT
CGGCAACATTTCATAGAAGATAGATTATCTTATTGTGTATATTTCATAGAAGATAGA
TATTCTGATTGTGTTCTGTATACAATGGTAAGGGTCTGGTGATTGTATCCTGAGATTA
AAAAACAACCTTGTGAGTGCCTTAAGTCACGTACTTTACCCTATATATACTGCAGCA
CAATAAAGCAAGGTATCAGCCATTTTGGGGCTGATCCTCTCAACCCCATCTTTTGTCT
ATCTCTTATTTTCTTAGCGGGGACGCTCCGTTCTCTCCCTGTGCAGGTGCGACTCTTG
CTTGTGCTGGCCGCGGCAGGTGGCGCCCAACGTGGGGCTCGAGCTCGACAGTTTTCC

TCGCCACTACTCTTATTAATTGAAAAGAGTGAGTATATGAGTAAACAAGTGAATTAA
ATTGAGGAGGAGTAGTAAGGTATATAGTTGAGAGTATAAATATGGGACAGACGCAT
AGTCGCCAGTTGTTTGTGCATATGTTATCTGTAATGTTAAAACATAGGGGAATTACT
GTTTCTAAACCTAAATTAATCAATTTTCTTTCATTCATCGAGGAAGTTTGCCCTTGGT
TCCCCAGAGAAGGTACAGTAAATTTGGAGACATGGAAGAAGGTAGGGGAACAAATT
CGGACTCATTATACTTTACATGGCCCTAAAAAAATCCCTGTCAAAACTTTATCCTTTT
GGACACTAATTCGTGACTGCCTGGACTTTGATAATGATGAATTAAAACGTTTAGGAA
ATTTATTAAAACAGGAAGAAGATCCTCTCCATGTTCCTGATTCGGAACCCAGATATG
CTGTTCCCGAGGGGGTTAAAAGCGACCCTCCGTTTTCTAACTTATTGCATCCTTCAGA
TAATGATGATTTACTTTCATCCACAGATGAGGCAAAATTAGACGAAGAAGCTGCTAA
ATACCATCAAGAAGATTGGGGTTTTTTAGCACAAGAAAAGGGGCGTTAACATCTAA
AGATGAATTGGTTGAATGCTTTAAAAACCTCACTATTGCTTTACAGAACGCAGGAAT
CAAGCTTCCTAGTAACAATGCCAAATCTCCTTCTGCTCCGCCTCTTCCCCCTGCTTAT
GCTCCTTCTGTTGTGGCTGGTCTCGATCCCCCTCCAGGGCCCCCTCCACTGTCTGAGA
ACATGTCTCCGCTGCAAAAGGCATTGAGACAGGCACAGCGACTTGGTGAGGTTGTCT
CTGATTTTTCTCTTGCTTTTCCTGTCTTTGAAAATAACAACCAGCGTTATTATGAATC
ACTGCCTTTTAAACAACTGAAAGAGTTAAAGATTGCTTGCTCACAATACGGTCCTAC
CGCTCCATTCACCATTGCTATGATAGAAAATTTGGGTACTCAAGCTTTACCTCCAAA
TGATTGGAAGCAGACAGCTAGGGCATGTCTCTCAGGGGGAGATTATTTATTATGGAA
ATCTGAATTTTTTGAACAATGTGCTCGTATAGCTGATGTTAACCGACAGCAAGGTAT
ACAGACCTCCTATGAAATGTTGATTGGTGAAGGCCCTTACCAGGCTACTGATACTCA
ACTTAATTTCTTACCTGGTGCATATGCACAAATATCAAATGCGGCTCGGCAGGCATG
GAAAAACTTCCTAGCTCCAGTACTAAGACAGAGGATCTTTCAAAAGTCCGGCAGGG
ACCTGATGAGCCTTACCAGGACTTCGTGGCACGACTTTTAGATACTATAGGTAAGAT
AATGTCAGATGAAAAGGCTGGGATGGTACTGGCAAAACAATTGGCTTTTGAAAACG
CTAACTCTGCTTGTCAAGCTGCTTTAAGACCTTATCGAAAAAAGGGAGATCTGTCTG
ATTTTATTCGCATTTGTGCTGACATTGGACCCTCCTACATGCAAGGCATTGCTATGGC
AGCAGCATTACAAGGAAAAAGCATAAAAGAGGTACTTTTCCAGCAGCAAGCCCGGA
ACAAGAAAGGACTTCAAAAGTCAGGTAATTTGGGTTGCTTTGTTTGTGGTCAGCCTG
GCCATCGGGCTGCAGTGTGCCCTCAAAAACAACAAAGCCCTGTTAACACTCCTAATT
TGTGCCCACGCTGTAAAAAAAGGAAAGCATTGGGCGCGGGATTGCCGTTCCAAAAC
GGATGTTCAAGGTAATCCTTTGCCCCGGTTTCGGGAAACTGGGTGAGGGCCAGCCCT
GGCCCCGAAACAATGTTATGGGGCAACACTGCAGGTTCCAAAAGGACCATTGCAGA
CCTCTGTCGAGCCACAAGAGGCAGCGCGGGATTGGACCTCTGTGCCACCTCCTACAC
AGTATTAACTCCCGAGATGGGGGTCCAAACCCTTGCCACAGGAGTGTTTGGGCCTTT
ACCTCCAGGGACAGCTGGACTGCTTTTAGGGCGCAGCAGTGCGTCTTTAAAAGGAAT
ACTTATTCATCCTGGTGTGATTGACTCTGATTATACAGGAGAGATAAAAATATTAGC
CTCCGCTCCTAACAAAATTATTGTAATCAATGCAGGACAGCGTATAGCTCAACTTCT
TTTAGTTCCATTAGTCATACAGGGAAAAACAATTAACCGAGACCGTCAAGATAAAG
GTTTCGGGTCCTCTGACGCCTATTGGGTGCAAAATGTTACCGAGGCACGACCAGAAC
TTGAGCTACGCATTAATGGTAAGCTTTTCCGAGGAGTGCTTGATACAGGGGCCGATA
TTAGTGTTATTTCTGATAAATATTGGCCTACTACATGGCCAAAACAGATGGCTATTTC
CACTCTCCAGGGTATTGGCCAAACTACCAATCCAGAACAGAGTTCATCCCTTCTTAC

TTGGAAAGATAAAGATGGACATACAGGCCAATTTAAACCTTATATTCTGCCCTATCT
TCCAGTTAATCTATGGGGGCGTGATATATTGAGCAAAATGGGTGTTTATTTATATAG
TCCTTCACCCACTGTGACAGATTTGATGTTAGATCAGGGCTTACTTCCAAATCAAGG
TTTAGGTAAACAACATCAAGGCATCATTTTGCCCCTTGATTTAAAAATCTAATCAAG
ATCGAAAAGGCTTGGGGTGTTTTCCTAGGGACCTCTGATTCTCCTGTGACACATGCC
GATCCTATTGATTGGAAATCTGAGGAACCGGTATGGGTCGATCAGTGGCCCCTAACA
CAGGAAAAACTTTCTGCCGCACAACAGCTGGTGCAGGAACAGCTGAGACTTGGGCA
TATTGAACCCTCTACCTCTGCTTGGAATTCCCAATTTTTGTTATTAAAAAGAAGTCTG
GGAAATGGAGATTGCTACAAGATCTTCGTAAGGTAAATGAAACAATGATGCATATG
GGAGCCCTACAACCTGGGTTGCCCACTCCTTCTGCTATACCTGATAAATCCTATATC
ATTGTTATAGATTTAAAAGATTGTTTTTACACTATTCCTCTTGCACCTCAAGATTGCA
AAAGATTTGCTTTCAGTTTACCCTCTGTTAATTTTAAAGAGCCTATGCAACGCTATCA
ATGGAGAGTTCTCCCGCAAGGAATGACTAATAGCCCTACGCTGTGCCAAAAATTTGT
TGCTACAGCAATAGCTCCGGTTCGTCAACGTTTTCCTCAGCTATATTTGGTTCATTAT
ATGGATGATATATTACTAGCTCATGCTGACGAACATCTATTGTATCAAGCTTTTCGAT
TCTAAAACAACATTTAAGCCTTAATGGTCTTGTTATTGCTGATGAAAAATTCAGACT
CATTTTCCTTATAATTATTTGGGTTTCTCCTTATATCCTCGTGTTTATAATACCCAATT
AGTACAATTACAGACTGACCATTTAAAAACTCTAAATGACTTTCAAAAACTTTTAGG
AGACATTAATTGGATACGTCCTTATTTAAAATTACCCACTTATACCTTGCAGCCATTA
TTTGACATCCTTAAAGGTGACTCTGATCCTGCGTCACCCCGAACACTTTCTTTAGAAG
GACGAACTGCTTTACAATCAATAGAAGAAGCTATTAGACAACAACAGATTACTTATT
GTGATTACCAACGATCATGGGGTTTGTATATACTTCCTACCCCCCGAGCACCCACAG
GGGTTCTCTATCAAGATAAACCTTTGCGATGGATATATTTGTCTGCTACTCCAACTAA
ACATCTGCTCCCTTACTATGAACTTGTTGCAAAATTGTAGCAAAGGGACGTCACGAG
GCCATCCAATATTTTGGTATGGAACCCCCTTCATTTGTGTTCCTTATGCTTTAGAACA
ACAAGATTGGCTTTTTCAATTTTCAGATAATTGGTCTATAGCTTTTGCAAATTACCCG
GGACGGATTACTCATCATTACCCTTCTGATAAATTGTTACAATTTGCTAGCTCTCATG
CCTTTATTTTTCCAAAAATAGTTCGCCGACAACCTATTCCCGAAGCGACACTTATATT
TACAGATGGATCTTCTAATGGAACTGCAGCTTTAATCATTAACCATCAAACCTATTA
CGCACAAACCAGTTTTTCTTCTGCTCAAGTTGTGGAATTATTTGCAGTCCACCAAGC
GTTGCTAACTGTACCTACTTCCTTCAATTTATTTACAGACAGCTCCTATGTGGTCGGT
GCCTTACAGATGATTGAAACTGTTCCAATTATCGGCACCACCTCTCCTGAAGTTCTTA
ACTTATTTACATTGATTCAACAGGTTCTCCATTGCCGCCAACACCCCTGTTTCTTTGG
ACATATTCGTGCACACTCCACCCTTCCTGGTGCCCTCGTACAAGGCAATCACACTGC
GGACGTTCTTACTAAACAAGTGTTTTTCCAATCAGCTATTGATGCAGCCCGAAAATC
CCATGATTTACATCACCAAAATAGTCAGTCTTTACGCTTGCAATTTAAAATTTCCCGT
GAAGCTGCACGGCAAATTGTTAAATCTTGCTCTACTTGTCCTCAATTCTTTGTTCTCC
CTCAATATGGTGTCAACCTCGAGGTTTACGCCCTAATCACCTCTGGCAAACAGATGT
TACTCACATTCCTCAATTTGGGCGTCTTAAATATGTTCATGTTTCTATTGACACTTTTT
CCAATTTTCTCATGGCTTCCCTTCACACTGGAGAATCAACACGTCACTGTATTCAACA
TTTGCTGTTTTGCTTTTCTACTTCAGGAATCCCACAAACCCTTAAAACAGATAATGGA
CCTGGTTATACTAGCCGTTCTTTTCAACGTTTTTGTCTTTCTTTCCAAATTCATCATAA
AACAGGAATTCCTTATAATCCACAGGGACAAGGTATTGTGGAACGAGCCCATCAAC

GCCTTAAACATCAATTATTAAAACAAAAAAAGGGGAATGAACTGTATAGCCCCTCA
CCGCATAACGCCTTAAACCATGCTCTTTATGTTTTAAATTTTTTAACTTTAGACGCAG
AAGGCAATTCAGCAGCCCAGCGTTTTTGGGGAGAACGATCCTCATGCAAAAAACCA
CTTGTGCGATGGAAGGATCCACTTACCAATCTGTGGTATGGGCCAGACCCTGTACTA
ATATGGGGACGAGGGCATGTTTGTGTTTTTCCACAGGATGCCGAAGCGCCGCGCTGG
ATTCCGGAAAGGCTGGTACGCGCAGCAGAGGAACTCCCTGACACATCAAATGCAAT
GCATGACACTGAGTGAGCCCACGAGTGAGCTGCCTACCCAGAGGCAAATTGAGGCG
CTGATGCGTTATGCTTGGAATGAGGCTCATGTACAACCTCCAGTGACACCTACTAAT
ATACTGATCATGTTATTATTATTGTTACAGCGGATACAAAACGGGGCAGCTGCGGCT
TTTTGGGCATACATTCCTGATCCGCCTATGATTCAATCCTTAGGATGGGATAAAGAA
ACAGTACCTGTATATGTTAATGATACAAGTCTTTTAGGAGGAAAATCAGATATTCAC
ATTTCTCCTCAGCAAGCCAATATCTCCTTTTATGGTCTTACTACTCAATACCCTATGT
GCTTTTCTTATCAATCACAGCATCCTCATTGTATACAGGTGTCAGCTGATATATCCTA
TCCTCGAGTGACTATTTCAGGCATTGATGAAAAAACCGGAAAGAGATCGTACCGTG
ACGGAACCGGACCCCTCGACATTCCGTTTTGTGACAAACATTTAAGCATCGGCATAG
GAATAGACACTCCTTGGACTTTATGTCGAGCACGAATTGCATCGGTGTATAACATCA
ACAATGCCAATACCACCCTTTTATGGGACTGGGCACCTGGAGGAACACCTGATTTCT
CCGAATATCGAGGACAGCATCCACCCATTCTTTCTGTAAACACTGCTCCTATATTTCA
AACTGAACTGTGGAAACTTTTGGCTGCTTTTGGTCATGGTAATAGCCTATATTTACA
GCCCAATATTAGTGGGAGTAAATATGGTGATGTGGGAGTTACAGGATTTTTATATCC
CCGAGCTTGTGTTCCTTACCCATTCATGTTGATACAAGGCCATATGGAAATAACACT
GTCATTGAATATTTATCATTTAAATTGTTCTAATTGCATACTTACTAATTGCATTAGA
GGTGTAGCCAAAGGAGAACAAGTTATAATAGTAAAACAACCTGCTTTTGTAATGTTA
CCTGTTGAAATAACTGAAGAATGGTATGATGAAACTGCTTTAGAATTGTTACAACGC
ATTAATACGGCTCTTAGCCGTCCTAAAAGAGGTCTGAGCCTGATTATTCTGGGTATA
GTGTCTTTAATCACCCTTATAGCAACTGCTGTTACTGCTTCTGTATCTTTAGCACAAT
CCATTCAAGCTGCTCATACTGTAGATTCCTTGTCATATAATGTTACTAAAGTAATGG
GAACTCAAGAAGATATAGATAAAAAATAGAAGATAGATTATCAGCTTTATATGATG
TAGTTAGAGTTCTAGGAGAACAAGTTCAGAGCATTAATTTTCGCATGAAAATTCAAT
GCCATGCTAATTATAAATGGATTTGTGTTACAAAAAGCCTTACAATACTTCTGACTTT
CCGTGGGATAAGGTGAAAAACATCTGCAAGGAATTTGGTTTAATACTAATGTTTCTT
TAGATCTTTTACAATTGCATAATGAAATTCTTGACATCGAAAATTCTCCAAAAGCTA
CTTTGAATATAGCTGATACCGTCGATAATTTTTTACAAAATTTATTTTCTAACTTTCC
TAGCCTTCATTCACTGTGGCGAAGTATAATTGCTATGGGCGCGGTTCTGACTGTTGT
GCTTATCATAATTTGTTTAGCTCCTTGCCTTATTCGTAGCATTGTTAAAGAATTTCTA
CATATGAGAGTTTTAATACATAAAAACATGTTGCAACACCAACATCTTATGGAGCTT
TTAAAAAATAAAGAGAGGGGAGCTGCGGGGGACGACCCGTGAAGGGTTAAGTCTTG
GGAGCTCCCTGGCAGGTATGCCAGGCCCTAGGACACGTGCCTAAGCTCCCTGTCCCG
CCACCCTCAAGAGTTTTTATAACCCTTAAGGCTCCAAGATGTTTGGTTTCGGCAACA
TTTCATAGAAGATAGATTATCTTATTGTGTATATTTCATAGAAGATAGATATTCTGAT
TGTGTTCTGTATACAATGGTAAGGGTCTGGTGATTGTATCCTGAGATTAAAAAACAA
CCTTGTGAGTGCCTTAAGTCACGTACTTTACCCTATATATACTGCAGCACAATAAAG
CAAGGTATCAGCCATTTTGGGGCTGATCCTCTCAACCCCATCTTTTGTCTATCTCTTA

TTTTCTTAGCGGGGACGCTCCGTTCTCTCCCTGTGCAGGTGCGACTCTTGCTTGTGCT
GGCCGCGGCATTGGATGAGAATTGTTTTTAATTGGTCATTTGTTTATACATTGTTGAT
TGCTCTTCGGATGAAAATTGAAAAGCGTGCCGTCACTCTAGTACCACCTAAAAGCAG
AGATCTGGAGTGGTGAGCAGGGAGGTGCTGGTGGAGGAGTGATGCTGTTTCCACAC
CAGGTTCCCGGAATCTGAAATCATGCCTAATCCAAAATGACTCTTTCAATCACAAAC
AATGAATTGTTTGTCAGGAAAAGGAAATAGTCTAAACTAGGGTAAGTGTGCCGGGA
ATATAAAAAGACATACAGTTTAGTAAATAGTTTTACTGTTTTGTGTGCTGCATGTTG
AAGTCTACATATTGACTAAGCCTCTGGAAACTTTCTCTCCATTACTGTGTTCTCGTGC
GTACTAATATCCCTCCCTTTTGATTTAGGTAAAATGAAATGGGAGAAATTTGGGAAA
ATTAATTAAAATTAACCCATTACATTTCTCTCTGACAGGTAATAAGTATGCCATTGGT
TCCACGATGCACTTTCTAGTCATTGTGGATCAGGGCAAGGTGAATTGAGTTTTGTGT
CTTCTGACAAATTTTGTCTTCTGGAGAATAAATAACACTATTAAATTGGGGCTTGGT
ATTACATTGTTAAATAGTTTCAGATGTTTTGAAATATATCCAGAGACCTTTGTCACAC
ACGGCCATCTCAAGGAGGAAATGAGAGAAAATTGGCAAACGTTCTGGAGGTGACAT
AGCAGACAGCTTGGCTGCCTTCATCGACGACTCCGCAGTGAGCACAAATAGAAGAG
CATTGTGCATCCGTCAGTGCTTGTGAACTTTAACAGTGGAGTGAGGACAGTTTCAAA
AGTGATGAGTTTGATCTCAGTGTTGTTTGCTCTTATTAAATCTTCCAAACTTCAATCT
TACATTCCTCCCATCTTAATTTTCCCAAATTTCTCCCATTTCATTTCACCTAAATCAAA
AGGGAGGGATATTAGTACGCATGAGAACACAGTCTAATTGAAATTCATTTGGAGGT
TCACTTCATTCTATGTAGAACTTCATTATTACTCTATTTGAAATGTAACAGATGCTGT
TATAAGGGGTCTGTCTTGTATCTCACTCTAAGAAGCTTTGGAAATAAAGAAATAAAA
GGTTATTCAGTACAGTGGTCCCATCTCATGGACCCTAAATACAGTATCTTCTCAGAG
TAGGCATACACTTTAGTAAGTGGCATCTTTCCTACAAGTATTCTTACTTTGCTGCTAA
GTCATTTCAATCATGTTCAACTCTGTGTGACCTCATAGATGGCAGCCCACCAGGCTC
CCCTGTCCCTGGGATTCTCCAGGCAACAACACTGGAGTGGGTTGCTATTTCCTTCTCC
AATGCATGAAAGTGAAAAGTGAAAGTGAAGTCACTCAGTTGTGTCCGACTCTTCATG
ACACCATGGACTGCAGCCTACTAGGCTCCTCCATCCATGTGATTTTCCAGGCAAGAG
TACTGGAGTGGGGTGCCATTGCCTTCTCTGATTCTTACTTTGATGGGACCCTAAAAC
AACAGAAACCTACTTTGGTTGAATACATAATAGAGTCATCCTCTTTCAGATAAACTT
ATTTCTCGATGCATTTGTTGAAAAATATTTGAGTACTTACTCTATGTCAGACATTGTT
GTAAACTACAGAAAACATTATTAGATACCTTATTGTGCTGAACACTGACAAGGATCC
AGGATCTTTTTCTTTTCAGGTGGTGATTCTAAATTTTGTTTCTATGGAATGAAACCTT
TTGATGAAAGTGAAAAAAGAAAACCTTAAGTTAGTAAATGATGAAGACACAAGTTG
AATTCTGTGGATACTTTGCCACCACTGCACACAAACAGACCCTGTAAGGGCCTATCA
ATCTCCTGAAGAAAACAGGCCAAACAAGTGACTTAAAAATAATACAATATTTACAG
GAGGAATAGAAGGAAGATACCAGAGTGAGCATCTTGCTGTTGCCGTTATAAATGCA
TAATTGACTCTTAAAATTCTAGTAACTAACATATATAACACATGGAAATACCTCCAA
AACATAATAACTGTACTGTGTGCTCTAAGGCTTGTTGTTGTTATTGCTCAGTTGCTCA
GTTGTGTCTGACTCTTTGTGATCCCATGAACTGTAGCATGCCAGGCTTCCCTGTCCTT
CACTATCTCCTGAATTTGCTCAAACTCATGTCCATGAGTTTGAGACGATGATACCATT
CAACCATGCCATCCTTTGTCGCCTCCTTATCCTCCTGTCCTCAATCTTTCTAAGCATC
AGGGTTTTTTTTTTTGCAATGAGTCAGCTCTTTGTATCAGGTGGCCCAAGTATTGGAG
CTTCAGCATCAGCATCAGTCCTTCCAGTGAGTATTCAGGGTTGATAACCTTTAGGAT

TGACTGGTTTGATCTCCTTGCTATTCAAGAAACTCTCAAGAATCTTCTCCAGCACTGC
AGTTGAAAAGCATCAATTCTTTGGTGCTCAGCCTTCTTTATGGTCCAACTCTCACATC
CATACATGACTACTGGAAAACCATAGTTTTGACTAGATGGACCTTTGTCAGAAAAGT
GATGTCTCTGCTTTTTAGTACCCTGGCTAGATTTGTCATAGCTTTTCTTCCAAGAAGC
AAGCACCTTTTAATTTCATGGTTGCGCTCCACAGTGTTTTTGGAGCCCAAGAAAATA
AAGTCTGTCAATGTTTCCATTTTTCCCCATCTATTTGCCATGAAGTGATGGGACTGGA
TGCTATGATCTTAGCTTTTGAATGTTGAGTTTTAAGCCAGGTTTTTCTCTCTCCTATTT
TGCCTTCATCAAGAGGCACTTTAATTTCTCCTCAGTTCCTGCCATTAGGGTGGTGTCC
TCTGCATATCTGAGGTTATTGATATTTCTCCTGACACTCTTGATTCCAGCTTGTGCTT
CATCTACTCTGAATATAAGTTAAATAAGTAGGGTGACAATATACAGTCTTGATGTAC
TCATTTCCCAATTTGGGACCAGTCCATTGTTCCATGTCTGGTTCTAACTGTTGCTTCTT
GACTGACCTACAGGTTTCTCAGGAGGCAAGTAAGATGGTCTAGTATTCCCATCTCTT
TCAGAATTTTCCAGTTTGTTGTAACCCACATAGTCAGGGATTTAGTGTAATCAATGA
AGCAAATGTAAATGATTTTCTGGAATTCCTTTGCTTTTTCTATGATTCAATAGATGTT
GGCAATTTGATCTCTGGTTCCTCTGCATTGTCTAAATACAACTTGTACATTTGGAAGT
TCTCGGTTCATATACTGTTGAAGTCTAGCTTGAAGGATTTTGAGCATTACCTCGCTAG
CATGTGAAATGAGTACAACTGTGTGGTAGTTTGAACATTCTTTGGCATTGCCTTTCTT
TGGGGTTGGAATGAAAACTGACCTTTTCCAGTCCTGTGACCACTGCTGAGTTTTCCA
AATTTGCTGGCATATTGAGTGCAGCACTTTAACAGCATCGTCTTTTAGGATTTTAAAT
AGTTCAACTAGAATTGCATCACCTCCGCTAGTTTTGTTCATAGTGATGCTTTCTAAGG
CCCACTTGACTTCACACTGCAGGATGTCTGGCTCTAGGTGAGTGACCATACCGTCAT
GGTTTTCTGGGTCATTAAGACCTTTTTTGTATAGTTCTTCTGTGTATTCTTACCACTTC
TTCTTAATCTCTTTTGCTTCTGTTAGGCCCTTCCTGTTTCTCTCCTTTATTATGCCCATC
TTTGCATGAAATATTCCCTTGACATCTCTAATTTTCTGGAAGAGATCTCTAGTCTTTC
CTAGTCTATTGTTTTTCTCTATTTATTTTCATTGTTCACTTAAGAAGGCTTTCTAATAT
CCCCTTTGGAGAGAGAAGCTTTGAAACATCTGGAGTTCCAAAGCTATTCTTTGGAAT
TCTCCATCAACTGTGTATATTTTTCCCTTTCTCCTTTACCTCTCACTCCTCTTCTCGTCT
CAGCTATTTATCTCAGCTATCTTATTTCAGACAACCTTTTGCCTTCTTGCATTTCTTTT
TCTGGGGGGATAGTTTTGGTCACTGCCTATGTATAGTGTTATGAATCTCTGTCCATAG
TTCTTTAGGCACTCCATCTATCAGATCTAATTCCTTGAATCTATTCATCATCTCCACT
GTAAAATCATAAGGGATTTGATTTAGGTCATACCCAAATGGCCTAGTGTTTTTTGTT
ACTTTATTCAATTTAAGCCTGAATTTTGTGATGAGAAACTGATGTCCTGAGTCACAG
TCAGCTCCAGGTCTTGTTTTCACTAACTATATACAACTTTTCCATCTTCAGCTACAAA
TGATATAATCAATCTGATTTTGGTATTGACCATCTGGTGATGTCCACATGTAGATTCA
TCTCTTGTGTTGCTGGAAGAGGTGTTTGCTATGATCAGTGCATTGTCTTGGCAAAACT
CTGTTAGCCTTTGTCCTGCTGCTTTTTTGTACTCCAAGGCCAGACTTGCCTGTTACTC
CAGGTATCTCTTGACTTTCTACTTTTGCATTCCAGTCCTCCATGATGAAAAGGACATC
TTTTGTTCGTGTTAGTTCTAGAAGGCCTTGTAGGTCTTTATAGCTTCTTTGGCATAAG
TGGTTGGGGCGTAGACTTGGATTACTGTGATATTGAATGGTTTGCCTTGGAAACAAA
CGAAGATCATTCTGTCACTTTTGAGTCTGCACCTAATTACTGCATTTCAGACTCTGTT
GTTGACTCTGATATCTACTCCATTTCTTCTAAGGAATTCTTGCCCACAGTAGATATAA
TGGTCACCTGAATTAATTTTGCCATTCCCATTCATTTTAGCTTAGCTTCCTAAATTTA
CTTCTGATGGAAGTGTTTTTGTCTGCAGTGGGGACAAATGTAATCTAAATTGTAGTT

GGGTCTCTTGGTGGAAAGACTCTAGTTTGCATGGAAACATCTAGTTTTTATGAGCCC
AGTCTTTAAGAGCTGCTTGGTCAAAAGTTACATCCAAGCAGACACGTAGACAGGGA
ATTCCAATGCATTACCTTGAGCTGTGATAAGGCCAGTGCTCAGAGATACCAGCTCTG
TTCTTTGATAAAAGGCGTCTGTTTACAGACCTTTGCTATTATGTGGAGTCTGCACTTC
TTGCTGGGTTGTGAACTCCATGAAGGCTGGGTTTCGTTTGCAACTTAGTATCAGCAA
TATCCAGTGCTGGATACATAGTAAGCACTTAATAAACAGATAGTACCTATTTATTCA
AAAATAGGTGTAAATCTTTATAACCCAAAAGGTTCACCTTCCAATGAGGTCTACATT
AGGATCTTAAGAATTTTTAGTCATGTAAAACAATTTAAAATCTTTTATCTCCATTTCT
TATGCTTAGCACTTAAAAAAAATATCCAACTTGACATAAATCTCCACCATAAAGTAA
TACATAGATGTGTGCAGTTTTGTGAGTGAGAATTCTCAGGAAAGCAGTTTCATGGAA
TATTACCATTACCAGTACCACCCTCACCTTTCTCAGCACGTCTGTTTTATTTTATTTTT
TAACTCTCTCTCTCTCTGTGTAGGGTGGATCATCTATTAAAATTATAAAATTTAACAG
AAATATTCTGTTTCATGCTACGCTAAGTATGCAAATTCATAGACACTGATAATTTCTC
AATGAACCAAATCCGTGACGCTCACTCATCTCACCTAATGCCATGAAGACCTTATCG
TTTTTCCCATTCTACGTGCAGAGCTTAGATATGTTTGCTTCTGCGTTCCGTCTCCTAG
GTGTCTACAGTATATCTACCAAGTGTTTAGATAAAGCCAGAGAAAAATTCAGTCGTT
CCACTCTGGGTCCAATGGGCATTTTTAAGATTTGCTAACAGGGAAAGTACAGGTTTT
GATTTCTCCACTTCTAACTTCACGGGAAACTGTAGCATAGTAGATGTTCTTTGTTAGC
CTAATCACACATACCGCCAAAACCCTCCCTTGAGGTGGATGTAATGGCCACAGCAA
GTGGAGAGATACAGACATCACTCTCTGTTTTCTTTCTCAATTCATTGGTCAGAGTTGC
GTCCTGACCTCATTGAGAGTTTGTCTCCTGTTATCCAAACTTCTTTGTTTCACATACT
ATAGTTTAGTTATAGCAGTTCAGCAAAATGCTATGTTTTACCGGAAGAAGTCAGGAA
AACCAGGATTAGGCAAGGAAAAAAAGCAAATGCTAATATCAGGGGGCAAACAGAA
AGGAAGCTTACCATAATGATCTCCTCCCTCTTCTCTGCCTCATTAATATTCCTCCTGT
GTATGAAGGATGCCATTTTGTTTTCTTCCTCACGTCTCCCACGCAGAACTCTGTACAT
AGTAACAGCAGGAGCATTTGTGAAGCCGAAATCCTGTAGAATAAATAAAAGCAATT
GAAACCATCTGAATGGGCTTTTGTGAGAAGAAAACTTGTTTAATAATAATTTACCAT
GGCAGAGCAGCAGCTATGAAGCTTAGCTTTCTCCCTCTGGTTTAAGGCTAAAAGTCA
CCTTTCAGCAGGTCTTCCAATGTTCACATTTGTTCACAACAGAAGCTGCAGACATTG
GAAGTGAGAATAGTTCTTGGCACAGTCTTAAACCATGATTCACTAATTTTTTTTTTTT
TTTTTAACAAGTGAGGACTCTGAGGCCCAAAGAAGTGAAGTGAGCTTTCACGTCTGG
GCCTAGCCTTTGGATTGCCCTTTCTGCAGTTTTTCTAGGCTCCTCCTTCCATTCAACA
CCAACATCTATTTTCTTATGGTTTCAAACTATAACCTAGCTGTTGAAGAGACCCAAA
TCTTTTCACCACCCCAATCTCTTCCCTGATTATTAGACTCATCCATTTATGTGCCTACT
GCTCAGGTTCTAGACATGGATGCATTACTCGTGAGTTGAAAGTGTCTAACCTCTTTC
AACACATTATCTTGTTACTGTGCTTTGATTCATGAGGTGCATTTTATGCTCAAAATTG
TAAACAAATACGATTGAGTTTTGTTTCCTACTCTGCTTGAGTATCTTCTAAAGTTCAG
TAAAACATGCATATATATGTACTCTGCAAACACTTTTAAACTAACAGGCTGGATTAA
ATTATAATAGCTAATTATAACCTACTGGTATGTTCAGGGCACTCATAATAATTATGA
AAAATGCCTAGTATTTAATGAGAGGTACTGGGATTCACTGGTTGAGATACTCTCTGT
AGCTGGATTGAAAAGATTTGAAACCTGGAATCTGCCACTTTCCAGCTCTGTGAGCTT
GGGGAACATATTAAACCTCTTTGTATCTCTGCTTTGTCTGTTAAATAAGATTAATATA
AGTACTGCCTTTATGGACCAGTTGCTCAAAATAAGCAAGTGACTATGTAAAATATGC

AAGTGCCTTGCACATAAGTTCTGAAGAAGGGTTAACTACCATTATTTAATTCTCACA
ATAGTTTTAAATAGGCCTTCATATAGGTACGAGTAAACATGCTCATAGAGATAAAGA
TATTTCCCCGGGTCCTGCAGTTAGTGAGAATGCTAGATAGAGCTATCCTGTCCCACC
CCAGTCAGTTCAGTCACTCAGTCATGTCCGACTGTTTGCGACCCCATGAATTGCAGC
ACGCCAAGCCTCCCTGTCTATCACCAACTCCTGGAGTTCACTCAAACTCACGTCCAT
CAAGTTAGTGATGCCATCCAGCCATCCCATCCTCTGTTGTCCTCTTCTCCTCCTGCCC
CAACCCCTCCCTGCATCAGAGTCTTTTCCAATGAGTCAACTCTTTGCATGAGGTGGC
CAAAGTATTGGAGTTTCAGCTTTAGCGTCAGTCCTTCCAAAGAACACCCAGGACTGA
TCTCCTTCAGAATGGACTGGTTGGATCTCCTTGCAGTCCAAGGGACTCTCAAGAGTC
TTCTCCAACACCACAGTTCAAAAGCATCAATTCTTTGGTGCTCAGCGTTCTTCACAGT
CCAACTCTCACATCCATACACGACTACTGGAAAAACCATAGCCTTGACTAGATGGAC
CTTTGTTGGCAAAGTAATGTCTCTGCTTTTCAATATGCTATCTAGGTTGGTCATAACT
TTCCTTACAAAGAGTAAGCGTTTTTTAATTTCATGGCTGCAGTCACCATCTGCAGTGA
GTTTGGAGCCCCAAAAAATAAAGTCTGACACTGTTTCTCCATCTATTTCCCATGAAG
TGATGGGACCGGATGCCATGATCTTTGTTTTCTGAATGTTGAGCTTTAAGCCAACTTT
TTCACTCTCCTCTTTCACTTTCATCAAGAGACTCTTTAGTTCCTCTTCACTTTCTGCCA
TAAGGGTGGTATCATCTGCATATCTGAGGTGATTGATGTTTCTCCTGGCAATCTTAAT
TCCAGCTTGTGCTTCTTCCAGCCCAGCGTTTCTTATGATGTACTCTGCATAGAAGTTA
AATAAGCAGGGTGACAATATACAGCCTTGACGTACTCCTTTTCCTATTTGAAACCAA
TCTGTTGTTCCATGTCCAGTTCTAACTGTTGCTTCCTGACCTGCATATAGGTTTCTCA
AGAAGCAGGTCAAGTGGTCTGGTATTCCCATCTCTTTCAGAATTTCCACAGTTTATTG
TGATCCACACAGTCAAAGGCTTTGGCATAGTCAATAAAGCAGAAATAGATGTTTCTC
TGGAACTCTCTTGCTTTTTCCATGATCCAGTGGATGTTGGCAATTTGATCTCTGGTTC
CTATGCCTTTTCTAAAACCAGCTTGGACATCTGGAAGTTCACGGTTCACGTACTGCT
GTAGTCTGGCTTGGAGAATTTTAAGCATTACTTTACTAGCATGTGAGATGAGTGCAA
TTGTGCAGTAGTTTGAGCATTCTTTGGCATTGCCTTTCTTTGGCATTGGAATGAAAAC
TGACCTTTTCCAGTCCTGTGTTCACTGCTGAGTTTTCCAAATTTGCTGGCATATTGAG
TGAAGCAGTTTCATAGCATCATCTTTCAGGATTTGAAATAGCTCCACTGGAATTCCA
TCACCTCCACTAGCTTTTTTTGTAGTGAGGCTTTCTAAGGCCCACTTGACTTCACATA
CCAGGATGTCTGGCTCTAGGTGAGTGATCACACCATCGTGATTATCTTGGTTGTGAA
GATCTTTTTTGTACAGTTCTTCTGTGTATTCTTGCCACCTCTTCTTAATACCTTCTGCT
TCTGTTAGGTCCAGACCATTTCTGTCCTTTATTGAGCCTATCTTTGCATGAAATGTTC
CCTTGGTATCTTTAATTTTCTTGAAGAGATCTCTAGTCTTTCCCATTCTGTTCCTTTAC
TCTATTTCTTTGCATTGATCACTGAGGAAGGCTTTCTTATCTCCTCTTGTTATTCTTTG
GAACTCTGCATTCAGATGCTTATATCTTTCCTTTCCTCCTTTGCTTTTTGCTTCTCCAT
TCTTTCTTTTTATATTGTTGCTCTGAGTCTAAAACCTAGAATTCATTTTGTGTTTAAAC
CCCTCTACTTAGCTCTACTAACCAAGTCTTGGCTGTTTATACTACCTTCTAAAATCCC
TGAGTGACTCTATTGCATTCAAGAGAAGTTTCTGAGAGAATGAATTCCTAGGTATTC
AGGCAAAACCCTCAGCCTGGTATATTATGCATTACATTAGGGCTTCATGATGTCATT
TGTGCCCACCTCTCCAGTGTCCTTTCTTTTCCAAAAGTCGTACATTGTACCAAAGCCT
TACCAAAGTGTATTACCCTGTTGTACAATTTCTTAGGCATCTGTAGTTTAGGAGCATG
CCTCTAATCCCTTTGCTGTTTCCTCCTTCCTTTCCTGGACCCTGTGTACTATACGCAGT
AAGCTATCACTTCTTCCACCAGCAGTCCTTTCACTTTCTTACATGACCCACCGTTTTT

CTTCCCATCCAGGTGTAGGAGTCACAGTAAATCAATGTTATAATTGTCTGTTTACAA
AACTGTCTTCCTAGGGATGCTGTGGCTACTTGCATGAAAATACCAGGTTTTTGTTCT
GTTGATTCCCTCATTTCATCCCAATTATTAGTCCAGGCTAAGCATGCTTAAGGAATTT
ATTTTATTTTACAGTTTCAGTGGCTACAGGTTAATGAAACATTTAGCAGTGTAAATG
GAAGAATAAAAGTATAAACAAATGAGAAGCAAAGTTCATGATTCAAACTATTATAG
AAAAGTAGCTTCAAAAGTAATCTAATATTTTTTAAAAAATGCAAAAAGCTTGATAGGG
GAAATGATCCAATATGAAAACCAGTAAAAATCATTGCAAAAAGAATGGAGCTAGGA
TTTGTGTATCTTCTGGGTTGTGATCCAGTGGTGAAGGAGCCAGACTAGAAGAAGTTG
GTTCAGGTTGCAGGTGTGACTGCTTGGCAAGTGCTTGCCATGTAGGCAAGAGGTTGA
GGACAAGATAAGATTAGGTGAGGACATATACTATCATGGGTAAGCCAAGTTCAAAG
GCTTACGTAGTTTTAAATTCTTCCTTCTTTTGGAAAATAAGGACTACTAAGTGAGTGC
TGGAAACTACAGAGTATGAAGTATTATCTCATAGCACTAGGCTCAATTTTAATAGTT
GTTTAAATCAAACAAGATTTAACACACTAGATCTCAAAATATTGACATGCATGAGAG
TGACTAAGAATGCTTGATGCTTAGGCCATGCTTAGGCTATTCTCTTGCTCTCTGAACA
TCCCTTGCTCAACCACTCCCTGCCCACCTATGCCCTACTCACATCCTACCTACCTTCC
TTGTTAATCACTGAATCTAATCAATAAGTGCCTACAAACTTGTCCCTGGCCCCAACT
AGGGATTCTTCCAATCTTTCTCTTCCTTTCCTTTGCTTCTTTTTTATTTTTATTTTTTTG
GCTTTACCATATTGTGTTAGTTTCTGCTGTACAAGGAACTGAATCAGTTATATGCATA
CATATATCCCCTCCCTTTTGGACTGTCCTCTGCCCCACCCCCATCCCACCCATCTAGG
TCATCACAGTCCAGGAGCTGAATAAAATAGATAGCTAGTGGGCACTGCTGTCCTAAT
CTTCTGATCAGAACATCTCCACCAAGATAGCTTATCAAAGGGGAATGAGGGATGCA
CTCCTCTATTGGTTTCTCTGGTAACTGATGACTCGACCTGATGTCAACTGCTAACTTC
CCTCTCCACATCCTCTGGCCCCCTGCCAGCAGACTGCTACCATGTCTTGCCCATTGTC
TGCCCCACATGGTGGAGTGTCAATTCAGGAGCTTGCTTCTGACATGTAAGATCCCCC
ATTCATTACACCACTGATGTGTCTGCCACTGACTTTGAGCTCTTTATTCAGTCTTATG
GCTGGGCAAGCACAGAGGGTGCAGTCCAAGTGTAGCCGTAATGCCTGCTTCTAAGC
AATTCATTGTTATATGTCATATTGTTGTTATAATTTTATAACTATCTTCATCATCATTG
TCAGTTACTCTTATTTTTTACATTTGTACCCTGATCTTCCCATCTTGCTGATTATCTGA
CATTTTAATCCCTTCTAAATACACACTTTGTCTTCTTAATCTCTTCTAAATTATGAAA
ATTCACCTACAACTATCTTGATAACACTTTACTGGGAGGGTTATTAGTTTCCTAGTGA
TATTTAGCTTTTCTTTCGTGCTCCTCTTTCCATGGGATTTCCCAGGCAAGAACACTGG
AGTAGGGTTGCCATTTCCTTCTCCAGGGGGTCTTCCCAGCCCAGGGATCAAACTCAC
ATCTCCTGCTTGGCAGGCAGGTTCTTCACAGCTGAGCTACCAGGGAAGCCATGGTTT
TTACAGATATAATACATGTATAAGAAGAGTATATTTTCAACATAGTGGCTAAGACTT
GGTATTCAGACTTAAATTTTCTTCTTATAACCCTTTCTTCTTTCTAGCTGTGAAACAG
GGTTATAAGACTCTTTGGTGTTTCAGGGTTCTTCAGCTCATGATCTGCCTCTCCTGTG
CCCTGAATCATGTTCTTCATCTCAGTGGGATATTTCGCTCCATACTTGAAACAGTAGC
TTTAGCCTCAGAATCTTTGTCTAGTATCATTTTGCATACCCCATAATAATCCCTCCTG
TTCCTCATCACTGTGTCATCTACTAATGTTCCTTCTGTGAAGTTCTATTTGTTGTTGTT
GTTTTAGTTGCTAAGTTGTGTCCAGCTCTTTGCAATCCCATGGACTGTATGCCTGCCA
GGCTCCTCTGTCCATTGTATTTCCCAGGCAAGCGTATTGGAGTGGGTTGCCGTTTCCT
TTTCCACGGGATCTTCCCTGCTCAGGGGCTGAAACCACATCTTCTGCATTGGCAGGT
GGATTCTTTACTGTTGAGCCACCAGGGAAGCCTGATGTTCTATTTACTCTTCTCCTAA

ATTCTGGGAAATTATATAACCAAATGCATCAGAACCACCTGATAACACAGGCATGA
GGAGTCAAACTTTTTAGCTGCCTTATTACTGTCCTTGCCCTTAAGCCAGAGACTACCA
AGAAGACACCTGTAGTTGAAAAAGTTGAGTTCATTACCTATTGCAAAGAGGAAAAA
GTGTACCATGGGGTCAGAAGGCCTTAAAAAGAACCTCTTATAAAATCTGAACTTCGG
TTGAATGATCTGGGGAGAGAGTTTAAGGAAGTAGAGCATTACTCTGAATTGAATGTT
GTCAGGAAGCAGGGTAATTTCACAATCTGGTGCTTTAAAAGGTCTTATTTAGGAAGA
GGGAAGATAAGCCTGCCTTGGTAATGCAGCAGCAGTGACCCAGATTAGCTGGGAAA
GGGGGACATTTGATGCCTGTGGCTTAGACAGGGTTTATAAGAGGTCTGTATTCAGAC
ATAATTACAGAGCGTTTTGTTTTGTCTTGATTCTTCATGATCATATAGTGTCCTTGCC
TAAAGCTGATGTTCTGTGAAATTGCTTCTGTTCAACATGAGGATACCACAGCCAGGT
GTGAGAGCCAGGGCAGTGTCCAGAAACACTGTGATAGCACCGGCCCAGGTCCTGGA
TGTCAGGGGCTGTTTTCTCTCTCATGAAAAAATTCTTTACTACTTCAGTAATTAAACT
TATTTCCATGAAATCCACTTCCCTCTACTTTGGTGATAAGATTCTAACAGTTCTTTGT
AATTTCCTCCCTAAGCCCTATGGTATGCTCAACCCATGGTGTATTTTAATACAAAATT
TCATTTCTCTTAAGCTAGCAATATGATGATGATCAATAAGGACCACAAAAGTAGCTA
GATCTATGTATAATAACTACAAAGGGCACAGATTGTTTAGGGAACTGATTATTTTTT
GTATTTATACCATAAACACTAATTTTTTGTGTAAATATAAACCATTGGCATATAGGA
AATAAATGGAATAAAATTTTAATTTTATTTAGTGGAAGTTACTTGTGAGGACTTTAA
AGGGTCTTTCCACTTACTATATAGAAAAGAGTTTAATAATCAGAGACTGGAATTTTG
GAGTTGGGAATTATGATGAATCAAAATATCTCTGGAGATATATACTTGGCTAAACAT
ATAAATGTGGTTAAAACTTTACTACTTAGACATTCTAGATGCCTACTAAAAAAAGTG
AGATAATCTGTTTCTTTTACATAATGGACTGTCATTATCCTAAGTTTTACTTAAAGAA
CACCTAATTAAGTGATTTAAGTTCATGATTGAATAATCTTTAAGTAATGTATGGTTTT
AAATGAGTAGAAACTGGCAAATGTCCCATGCTAATAATTTGCTTTGTAACTTGTTAC
AGATATGTAGAGGAAGTGTTTTGGGTGGAAGGCTCTGAAGAGAGAAGGAGATAAGG
TTATATAAATCAGGCCTATTCTATAAGTCACACATGAAAAAATATCTTCCACAATGT
AATATTTAATGTATATGTAATATTGCTAAATATAAGAATTTTTTTGACTTATATGATC
TTTAAACATGAGTTATTTAATAGGCTCAAGATTACCAATTTCATATGTAAGATTAAG
TTTCTTCATCTTTGAGTTTTTTGATTGGTTTTAGAATAGAATAAAAGCAATAAAGAAG
AAATAGTTACTTATTTTCCAACTCTGCTTTACTTATCATACGGTCTTATTAGTAAGGA
TAAGATGTTTTGATTGAACTTCATAAAATTTGGCCTTTATTTACCCTTGAAGATATGA
TCTAAACCAAGAAAGATGCTCATTTTTCATTGTCTCTTTACGGTTTACTCTTTTGTAT
GATTCTATTTCATCTTTCCTGTTTTCAAGTAAAATGTGTAGCTATGTTTTATGTGAAC
CAGAAATAATAATTTACAACAGGAAATGGAAGTTAAAAAGTTAAAATTCAAGTTAA
ATATTTAACTTTTAATTAGACAATAAGGCTTAAATCATTTTAAAGACTATAGATACTT
GTCATGGGCATAGAGGTGGGTCTGTTCACTCAGTACAATCGAAGGCAGCACTTTGGA
TCCTTAAATCTGAATGGTTCAATAATTAAACTACAGAAGATTCCTGATAACTGATTT
AATTCTTTGTTAACAGTAATAAGTGAGAATACAATAAATATCCTTGGCTACTTGGGA
ACTTGGCCTATTCTTGGAGTCTATTTTATGCGGGGCCAAATTAGTATAATGTGCTTTT
GGATATGTGTGTTCTTTAATATTACTGTCTAGAAGTTTACTACAGATGAACTTATCTA
ACTGCTTTTTGTCAAAAGAAGTATTTTGAGTGCCCATTGAAGCAGTAGGTACCTATC
TGAAATGAAAAGGATTCAAGCCACTCAATTTTCCTTCCTGAAATATCCCTGCACAAG
GTGTGCTTCTGCAAATTCTTAACACATCACAGATGGTCTCGAAACTGTTGTTGCGAC

TGATGCCAGAATAAAAAGGTAGCTTTCTTATACTTGTTTACATATATTCCTTTCTTCT
TGGAGCATATCAGGCTTGGAGGCAAACAGTGGCAATGGTTTTCAACTGCAAAACCT
ATCCAGATGATTTCTTCCAGAGTCTCCCTTTTCCCCACTGTCATTAGGCTTAGATTCT
CCTCTTTTCCATCCTATCCTTTTTGAACGGTCATAATGCATTTACATTTTATACATCTA
CTGGCCAAACGAGAAAGATTACATGTTCAACAAAAAGCAGGCTCATATAGTCATTTT
TTTCCATTGTTTCTTTCCAACAAGGTCCAGTTATTGACTCTAGACTGAGTAAACCAAA
GGATCGACGTTTCCTGCTTACTCTTCCATGGCAGTTTGCAGTGGACCTCTCCATCTCT
TGATCTTTGTTCCTCACCCTCATCCCCACATCTCTTCCCCAACTCTCATGAGACTGAG
CATGGCCCTAAGTCCAGCAAACTCAGCCTGGAATGTGAAGTGGGGAAAGTGAGAAG
TTAGGAGATTAAATACTTCCTAATAAAGCTCAACATTCAGAAAACAAAGATCATGG
CATCCGGTCCAATCACTTCATGGGAAATAGATGGGGAAACAGTGTCAGACTTTATTT
TTTCGGGCTTCAAAATCACTGCAGATGGTGACTGCAGCCATGAAATTAAAAGACTCT
TACTCCTTGGAAGGAATGTTATGACCAACCTAGATAGCATATTCAAAAGCAGAGAC
GTTACTTTGCCAACTAAGGTCCGTCTAGTCAAGGCTATGGTTTTACCAGTAGTCATGT
TCGGATGTAAGAGCTGGACCCATAAAGAAGGCCAAGTGCCGAGGAATTGATGCTTT
TGAACAGTGGTGCTGGAAGACTCTTGAGAGTCCCTTGGACAGCAAGGAGATCAAAC
CAGTCAATCCTAAAGTAAATCAACCCTGAATACTCAATGGAAGGCCTGATGCTGAA
GCTCCAATGCTTTGGCCACCTGACGTGAACAGCTGACTCATTGGAAAAGCCCCTGAG
CTGAGAAAGATTGAGGACAGGAGGAGAAGGGGGCGACAGAGGATGAGATGGTGAG
ATGGCATCATTGACTCAATGGACTTGAGTTTGAGCAAATTCTGGGAGATAGTGAAGG
ACAGGGAAGCCTGGCTTGCTGCAGTTCATGGGGTCGCAAAGAGTTGGACATAACTG
AGTGACTGAACAATACTTTTCCTCCACTTCCAGTTTCAACTGAACTCAGGATTTAATT
GATGGAAAAAGCTATTAGCTTTTCCTGAAATGTAACCAGTCATTTTTCTATTATTTAA
GGTATCTAAACAGACACTATGGAGAAGGCAGTGGCACCCCATTCCAGTACTCTTGCC
TGGAAAATCCCATGGATAGAAGAGTCTGGTAGGCTGTGGTCCATGGGGTCGCTAAG
AGTCGGACAACTGAGTGATATCACTTTCACTTTTCATTTTCATGCATTGGAGAAGGA
AAGGACAGCCCACTCCAGTGTTGTGCCTGGAGAATGTCAGGGATGGGGGAGCCAGG
TGGGCTGACGTCTATGGAGTTTCACAGAGTCGGACACTACTGAAGTGACTTAGCAGC
AAACAGACACTAAGAACTGTTTTATTGACCTGTTGAGACAAAATGACTCATAGTTAA
TCATAAATTCCTTTAATATTGTTCAGTTCAGTTGCTCAGTGGTGTCCGATTCTTTGCG
ACCCCATGAATTGCAAAAGGCCAGGCCTCCCTGTCCATCACCAACTCCTGGAGTTCA
CTCAGACTCATGTCCATCGAGTCGGTGATGCCATCCAGCCATCTCATCCTCTGTCGTC
CCCTTTTCCTCCTGCAATCCCTCCCAGCATCAGGGTCTTTTCCAATGAGTCAACTCTT
CACATGACGTGGCCAAAGTACTGGAGTTTCAGCTTTAGCATCATTCCTTCCAAAGGA
ATTCTTTCCAAAGGAACACCGAGGACTGATCTCCTTTAGAATGGACTGGTTGGATCT
CCTTGCAGTCCAAGGGACACTCAAGAGTCTTCTCCAACACCACAGTTCAAAAGCATC
AATTCTTCAGTGCTCAGCTTTCTTCACAGTCCAACTCTCACATCTGTACATGACCACA
GGAAGAACCATAGCCTTGACTAGATGGACCTTTGTTGGCAAAGTAATGTCTCTGCTT
TTAAATATGCTATCTAGGTTGGTTATAACTTTTCTTCCAAGGAGTAAGTGTCTTTTAA
TTTCATGGCTGCAGTCACCATCTGCAGTGATTTTGGAGCCCCCCAAAATAAAGTCTG
ATACTGTTTCCACTGTTTCCCCACCTATTTCCCATGAAGTGATGGGACCAGATGCCAT
GATCTTCGTTTTCTGAATGTTGAGCTTTAAGCCAACTTTTTCACTCTCCTCTTTCACTT
TCATTAAGAGGCTTTAGTTCCTCTTCACTTTCTGCCATAAAGGTGGTGTCATCTGCAT

ATCTGAGGTGATTGATATTTCTCCCAGCAATCTTGATTTCAGCTTGTGATTCCTCCAG
CCCAGCATTTCTCATGATGTACTCTGCATATAAGTTAAATAGCTGGGTGACAATATA
CAGGCTTTATGCACTCCTTTTCCTATTTGGAACCAGTCTGTTGTTCCATGTCCAGTTTT
AACTGTTGCTTCCTGACCTGCATACAGATTTCTCAAGAGGCAGGTCAGGTGGTCTGG
TATTCCCATCTCTCTCAGAATTTTCCACAGTTTATTGTGATCCACACAGTCGAAGGCT
TTGGCATAGTCAATAAAGCAGAAATAGATGTTTTTCTGGAACTCTCTTGCTTTTTCCA
TGATCCAGTGGATGTTGGCAATTTGATCTCTGGTTCCTCTGCCTTTTCTAAAACCAGC
TTGAACATCTGGAAGTTCACGGTTCACATATTGCTAAAGCCTGGCTTGGAGAATTTT
GAGCATTACTTTACTAATATGTGAGATGAGTGCAATGTGTGGTAGTTTGTACATTCTT
TGGCAATGCCTTTCTTTGGGATTGGAATGAAAACTGACCGTATATATGCACTAATTC
CACATATATGCATTAATATATGATATTTGTTTTTCCCTTTCTGACTTACTTCACTCTGT
ATTACAGTCTCTAGGTCCATCCACATCTATAAATGACCCAATTCCATTCTTTTTTATG
GCTGAGTAATATTCCATTGTATATCTGTACCACATCTTCTTTATCCATTTCTCTGTTGA
TGGGCATTTAAGTTGCTTCCATGCCCTGGCTGTTGTAAATAGTGTTGCAATGAATATT
GTGGTACATGTGTCTTTTGGAATTATGGTTTTGTTTGGGTATATGTCCAGAAGTGGGA
TTGCTGGGTCATATGGTAGATTTATTCCTAGTATTTTGAGAAATTTCTCTACTGTTCT
CCATAGTGGCTCTATCAATCTACAATCTCATCAACAATGCAAGAGGGGTCCCCTTTC
TCCACACCCTCTATGACATTTATCATTTACAAATTTTGCAATGACGGCCAATCTGATT
GGTATGAGGTCGTACCTCGTGGTAGTTTTGATTTGTATTTCTCTAATATTTAGTGATG
TTGAGCATCTTTTCATGTGCCTCTTGAGCATATGTATATCTTCTTTGGATAAATATCT
GTCTGCTTAGGACTTCTGCCTCCAGCTGCTTTTTTTTTAAGGTTGTTTGTATTTTTTAT
ATTGAGTTGTATGAGCTGCTTTAATGGGAACACATGTACACCCATGGTGGATTCATG
TTGATGTATGGCAAAACCAATGCAGTATTGTAAAGTAAAATTAAAAAAAATTAGGT
CCCATCTGCTTATTTTTGTTTTTATTTTCATTTCTCTAGGAGGTGGATCAAAAAGGAT
CTTGCTGCAATTTGTGGCAAAGAGTAGTCTGCCTATGATTTTCTCTAAGAGTTGTATA
GTCTGGCCTTATCTTCAGGTCTTTAATCCATTTTGACTTTATTTTTATGTATGTTTATT
TTTATCACTTTATTTATTTATTTATTGTATCATTATTATTTATGTAGTTTATTTTTAGG
AAGTGTTCTAATTTCATTATTTTTTCATGTAGCTATCCAGTTTTCTCAGCATGACATA
TTGAAGAGGCTGTCTATTCTCCATTCTACCTTCTTGCCTCCTTTGTCAAAGGCAACCA
CAGGTGTGTGGGTTTATATCTTGGCTTTATATCCAGTTCTGTTGACTTAAAAAGTTAT
TTCTAACATCACATTTCTGCTGCTTAGCTAAAATGAGGAAGTTGGTCCAGATGATCT
ATAAGGGATGTTCAAATACTAGGTTTATTTGATCATCTATTGTGTGAATCTATGCTTC
TGTTACCTTCTTTTTTAGTATATACTATTGCATTGAAGTTTCCCAAATATTTTTTTAAG
TATTGACAAATGACCATAATTGCAACAGGAGAAATTATTCTAGATTTACAGAGTAGG
TGACTGCAAGCTAGTAAGCAACCGGCTATAATATCTTGGTCAAATTCTAAACAAAGA
ATTACAATTTCTGTGTTGATTTTCTGATTGTAGAATTATATAGCAAATGTTTGTAAAG
ATTTCAATAAAATTTTCAGGCTAGACAGAAATGATACAAGTAGAATGGTTGCTGAA
ATTTCTACACAAATCTCCTACTAGAAAAATGAACTTAACAACTATTCATGCCCACAA
ATACCTTCACAAGAAGCTCAGGATTCCATGTGATAGTACCTGGGTCAAGGAAGTACT
ATATTTTGAAGAGATTTTAAAAGATACACAATGGATAGCCTCAAACTTTTTAAAGCA
GGAAATAAAACTTTATCCTGAGAAGATAGGTTTCCAGGGAAATACTTAGGAAAATG
AAGATGCCACAGCAAAAAAACCAAGAGAGCGAAGGCAAGAGTCACAAGATAGGAA
AAAAGTAAAGGACTGCTTAACACAGCTCAATTCAATTACAATCTTGTTTTTAAAATG

TATGTAGATATCAAATGAATGTTTGTTAGTTTTTTAAAAGACCACATGGTATTTTAAA
ACTTAAAGAAAATAGACAATTGCCTTATTTTCTCCACCCTGTCCCATTCCCCATGTTA
AATTTTAGAATAATGAACTATTTTTAGATGACATACTTAAAGTGTTCTTGATGTTTTT
TTTTTTTAAAGAAAAACACAAAAATCTGTTTTTTAAATACAATTTCTTCCTGTAGAAG
AGAAGGATCTAACTTTTATGTATTAAATACAATACAGCTTGATCCATCACATCTATA
TACATATTTCTCATCTTCTACCTAATAATGTAGTTATTTTTCTAATATTATTGACTGTG
TAAATCATTATCATAAATTTTCTAAGCAGCAGAGCCAAGAAATAAACAATGAGTGCT
TTCTTTCTGGTGGAATTTATGTTTTCCTGGTGATAATTGCATCCTTAAATCCTCTGTCC
TCCTGGTCCAATATTAACTGGTTTTTGTTTTTTCCCCCTTGACTTGTGACATAATTCTT
ACTGCGAAACTTCCCTTCCGCTGTTCCTGGAAATTCCCTATGCCTCTCATTTGTTGTA
TTAGCTATTTCCTTAATTCCCTGTCTTCTTTTACTTCCTTGTTTCAGTAAAATTTATAT
TCTGAGATTTCTTGGGCTCCTTAAAAAACAGTAAAAGAGAAGTAAAATTTAGGACTG
TGTGTATATTAAAATGTCTATATCCTCTTTCTAATTTTTGGACATTTTTGAAGAGATT
GTTATTTGACTTGTGAATCCCCAGTGCTGCTGTCTGTTGTCTCTTTGATTTGTATGTG
ACATTACCCTCAAGCCCCCGAGAGGTTTTTAAGAATTTCTCTTTATTCTTGGTACCTG
TGATGGGAAAACAGGCAGCTAGGTCATAGCCTGTGGCCCTTTATATCCACTCCGCTG
TAGATGGGTGACTCTGCACTCTGGGGGTTTAATGTCCTTTGCTTCTGGAAAATATTCA
TCATTCTATCCTATTTCCGCTTTGTCTGGTTGTGTTTTCCCTGGAAACCCCACTGTGTG
GTTGTTGGCTCTATCCTATAGCTAGCTGCTCTAATGCTGCTCCCGTGGCTCCTCTGTA
CTCAGATATCCCAAACGCCTGCTCCACTGTGTTACTTCTACTATCACGGTTTTCTTTT
TTCAGATTGCTGCCTTCTATTTATGTGGATTTTAGGATTTCATGGTTTTCTTTTAAAAG
TGATTCTTTCCTATTTATTTTTTAAAATTATGATTAGAATTTTTACATGTAATTTTTAG
ATTAATTTTTAACTCTTTGGCTGTGCTGGGTCTTCATTTCTGCGCAGGCTTTTCTCCAG
TTGTGGTGAGTGCAGACTACTCTCTCTTTGTGGCCGGTTCTTTTGTTGCAGGGGCGCA
TAGGCTCTAGGTGTGTGGGGTTCAGTAGTTAGGGCTCCCAGGCTCTAGAGCACAGGC
TTGGTAGTTGTGATCCGCAGGCATAGTTGCTCCACGGCATGTGGTTATCTTCCTGGA
CCAGGGATTGAATCATGTCTTCTGCGTTGGCTGGTGGATTCTTTACCACTTAGCCACC
AGGAAAGCCCCATGGTTTTTTGTTTTACTAGTTTCATGACACAAAATTGTCTCTTAGT
TGTCTAATGATAGTAATCACAAAAATATTTTGTGTTTTTTATTCCCTTTCCTTGTATTC
AACCTTAATGTTTACTGTATTTAACTTTTTTCATGCAAGTCAGAAGTATTCCTCAAAT
GTCTAGTGATTATCCATTTATATTTAAGATTGAGGCAAAACGAATACAGATTTCATT
CTAGAGTTGCACTGTCCAATATGGTAGCCACTAGCCCCAAGTGACTATTGAGAACTT
GAAACATAGTTAATCCAGATTGTGTTCTAAGAGTAAAGTAGAAACCAGACTTTTAAC
ACTTTGTGCCCCTGCAAAAAAATCTCATCAATAATTTATGTTGGTTTAAATGACAGT
ATTTATGATGTTTTCAGTTCAGTTCAGTCGCTCAGTCGTGTCCAACTCTTTGCGACCC
CATGAATCGCAGCACGCCAGGCCTCCCTGTCCATCACCAACTCCCAGAGTTCACTCA
GACTCACATCCATCGAGTCAGTGATGCCATCCAGCCATCTCATCCTCTGTTGTCCCCT
TGTTCTTCTGCCCCCAATCCCTCCCAAAATCAAAGTCTTTCCCAATGAGTCAACTCTT
CGCATGAGGTGGCCAAAGTACTGGAGTTTCAGCTTTAGCATCATTCCTTCCAAAGAA
ATCCCAGGGTTGATCTCCTTCAGAACGGACTGGTTGGATCTCCTTGCAGTCCAAGGG
ACTCTCAAGAGTCTTCTCCAACACCACAGTTCAAAAGCATCAATTCTGTGCTCAGCC
TTCTTTACAGTCCAACTCTCACATCCATACATGACTACTGGAAAACCATAGCCTTGA
CTAGACGGACCTTAGTTGGCAAAGTAATGTCTCTGCTTTTCAATATGCTATCTAGGTT

GCTCATAACTTTTCTTCCAAGGAATATGATGTTGTAGTAAGTAATATATAATATTAA
AAGTAATATTGCCTATTTACTTACATTTTCAGTGTGGCTACTGGATATTTTAAAGGGA
TATTTAGCATGAGGCTTCTGTCATATTTGCACCAGACAGTACTACTCTGATTGAGCA
GAAGGTCCTGACCATTTCACTGTGCTCCCTGACCTCCTCCCCACTTCAGCTAATAAGT
AGTGTCAAGTGTTTTCTCTTGTGCCAGTTTCCTTAGAAAGGAATCCTATAATATTTTG
CTTGATAGAGTTCTGGAATTGGTAGGGGAAGAGAAATCAGTGTGTGGGCTTAAACTT
ATTTAGACATGGATCTCCCTCATACCCCCAGCTGGGCTTCCTTGTCTTAGGTCCAGAA
ATCTCTTACTCCATGTCTCAAAAGAGGAAACACCCGATCTTCTGACAGATGAGGAGG
AAGGAGAGTGAGCAGGCTGTGTAAAGTATATGGAGAGAAGATGTAGGTTTAGGACT
TCCTGAGATATTCAATCAAGTTAAGACTCTGCTTTCCATGCACCCCATGGTTCTATGT
GGACTTATACAGTAGATTCTTTTTGCTTCTGCTCTAGGTGTACGTGTTTAGAATCTGA
CATGCAGGATGTTTGAACAATTACCATTAGTCCATTATGCTTTCTATATGTTGACTGG
TTTCTTGTCTTCACTCATCTAAATCTTTGAGCTGAACATGTGTGTTCCTATTTTTATAA
ATTCTACCTACAACTTTCATTTTAATGAGATTTCAGATGTAAGTGAAAATGTATTCCA
CCTGCCATGTTTAACCTGAGGTCATAATTTTAAATCTTAATATCTTGTTATTCCCTTCT
ATATTTTTTGTCAGATTATATTCAGGTACTTAAGTCTCCTATAACCTGCTCTAATATG
TCCATCTCTCTGTAGATAGGAACCTTTATGGCAAGCCAGGTTTAGCTTGTGCACATG
CCTTTTTCCAACCATACCAATTACATTTCTAAATGATTTACATGATTTATCTAACTTT
ATTTTCCACGAATCCCACTTCCTTCTACTTTTTTACATTTAACTCATCTTCTAGGGAA
GATTAAGGATGTCAGAGTTCTATGAACATATTCTTAGAAATTCAGTCCCCACTGATC
TCTTTCTTAGCTAAACTTCTATCATGTGTGTGTTTCCCACAATTTTTAGTTTTTTATAC
TATGATAAATTATACTGTGAGGTATTAATCCACATGGTTTTATTCTGCCCACTTCATT
CTCTTTTCTAACTGCAAGATATGAAGCTGCAAATTGAGGGAAAATTTTTAGTGGATA
CAAGGAACTGACATTTGGTTACTATTCAGTCTTACTAAAGACTAGGAAAATAGTTGT
GCCCTAAGCATGGATAACGGAGAAGGCAATGGCACCCCACTCCAGTACTCTTGCCT
GGAAAATCCCATGGACGGAGGAGCCTGATAGGCTGCAGTCCATGGGGTCACTGAGG
TTCAGACATGACTGAGAGACTTCACTTTCACTTTTCACTTTCATGCACTGGAGAAGG
AAATGGCAACCCACTCCAGTGTTCTTGCCTGGAGAATCCCAGGGACGGCAGAGCCT
GGTGGGCTGCTGTCTCTGGGGTGGCACAGAGTCGGACACGACTGAAGTGACTTAGC
AGCAGCAAGCATGGATAAAAGGGAAACACAGACTTTAGACATGACCGAATCCCAGA
GAGCTGCTGTGATCCGTTCTGCAGCAGACGCACTCCTAAAGGCAAGTTTTCTTCTCC
CTTTCAACTCTCAAGATGGAGGACCTCAGAACACAAGTGTTATCAGACAGAACAAG
CCTCAGAAAACACTGACTCAGAGACTACGGCAGTGGAGTATCCGGGGCTGTCCGTG
GTACATAGGCTGCTGTTTAGAGGCAGATTCATGGAGCCCCTTGGACCCTGACACTGA
GCTCAAGGCCCCTGAAGGTTATGCAAGTTAGTTGAGAAAAAGAAAGGTACCACCTG
CAAAGTGCTCGAGGCCAGAACAAGGGAAAGTGATAGCCACAAATTTAGAGGGCATG
CTCTGTGTTCTCATCTCCTTCTCTGAAAATAGCCTGTACTGAGGAAAATGGAACAAG
AGTTTTAAAGTTGAAATGACTGAGGGACAGCAAAAATGAAATTTCTTAAAAAGTGA
CTAAATATGCTGTATCAGACAGAATGGAGGTTTGAGATAAAACGAAATTTAGTGCA
ACAAAATTTTGTTTTTGCACACCTGAGTCTGTGATTGTAAAACTCATTTCCATTTCAG
TAATATACAGCATACAGCCATTCCCAAAGTTTCTTTCTCACAAAAAACAAATGAAAA
AGAAAACCTTTTCCAGGTAACCATGATTATTTTTCTTTGTTTTAGATTAATTTTATATT
TCACATTCCCTTTGTCAGGTTAAACAGTTTTGTTTCCCAGAGTGATAGAAACTTGGAC

TTAATTTTTATTTTGAACTTTGATTTTGAAAGCTTAACATGTAATATTTTCTCCCTTTA
AAGAATTATACCCACCTAGGGGACAGGAGATGGTCTGGATTTTGAGTTGATTGTATA
TACATACACATGTATATGCAATTTAAGCAAATAATTGTATTATCAAGATGGTATGAC
AGCCCAATACTTAAAAATGGCTGCTTTAGAGAAGAGCTAGAATTTGGGAAGCTGAA
GGTTATTTCCTAATATTTATGATTTGACAAGTAAAGTTCCCCCATATTCTGATCTACT
TTTCTTGGAGCATTCTCTTACTAGCAGACAAAAGTGTCCTAAGTTACACAACCATAG
CTTGGCATTCTTTGACCTTGAGCAGTTTTCTTGACCTCTCAGTCCTTTTGCCTCTCAGT
CCTTTGGTACTTTTCAGTTCAGTGCAGTCACTCAGTCGTGTCCGACTCTTTGAGACCC
CATGGACTGCATGGTACTTGTAATGTCAGGAAACCAACAATTCTTATTTCAAATTAT
GGTTATGAAGATTAATTTAGATAATACATATAGTCCTTAGAAAGGTGTTTGCCTGTA
GTAAGTGCCCTCGATGTTAGCCGTTTGTATTACATGACACACTTTGGGCTTTTCAAAA
AAATGTGCTTCTGATAAGTTTTATTGCCGGGGTCCAGTCCCGGTGGATCCAGGGTAA
TTCAAAGGTGGGGACGGAATCGGCGTCCTGGAAAAAACTTATTTAATTACAGATAT
AGAGAGAGATTGGAAACAGATAGTGTAGTAGGAGAATTAGTGGAGAAAAGAGGCT
GAATAACTGGTTTACATGGAATACCAATCACCACCTACGTAGGCCACAGGCGTCCTT
CCATTCTCCCGAAGGACAGGAGGCACTGAGGCCTCCCCGGTCCGATCTCAGAAGCT
CAGGCAGAATTAGCAGGCTTGGTGAGTACCCACATTTCAGATGGGAATTCAGCCAG
GAAAGCAGGGAGCAAGAAAGAAACAACATGGGGGAATCAGTCTTTCCAGAATTTGA
TCCAATTTCTTTATTTTTCAGGTTTGTTTACATACCTTTTTGTTATACATAGGGATGAA
TACAGAGTCACGTGGGGGTCAGCAGACCTGACCCTTGTCACAATCAGGTGCTTCATA
TAAAATTATACAAAGGTCTTATGAGTTTCATCATCTTCTGGCCATGAGGTCTGCTGA
CATTTTATGGCCCTTTCTGATACCAGTCAGTTAACCAGAAAACTTATTTTTCCAGGGG
TGATTTTTTCTTAAATCAGGCGCCACCCTCCAAATAAAGTTGCATTCCTATAGGGTG
AGGGTGTAGTGAGTTACAATCAAGAAAGGAATTTACTTAACCTAAGGTTTAACATG
ATTCATCTTAAAGGTTAATACTTATTTCTCCTATATGCTAGTTATATTCATTATAAGG
GCAGGAATATGGAGATTTAGCAGCAAATATTGGCTCAACAAATGTAAACCCTTCACT
AATGCTCCCCTTAAGATCTATTTTGTCTTAAGATAAAGTTACATTTTTGCAAAGCAAG
GACACAGTGATTTATAACAAAGTACAGTGGTCTATTACAAAAGAGAAAATTCATTA
ACTCAAAAAGTCTAGTATTGCTAACATCAAAAAACTACTATATTTCCTTTTCTATATT
CCAAATACATTGATTAATATATTCCCAGGTGCCTAAGGATATGGAAGCCTGATGGCA
ATCATTGACTCATCAATGAAAAAAGCCCTATGCTAATACTCCAAACTCTCTGTGCTG
TTTATGGTTGAGAGGTTGTCACACAAGCTAGTCTGTCAGCAGAGAGGTTTGACCTGA
GACATCCTTGTCACACTCAGGGCAGGGAATTAGCAGTAATTATTGGCACGACAAAT
GAAGAAAAAACCCTTCACCGATATAATTCCTAATCAACCCACTAATACTATACTAAT
GATCTTCTAATTTCTCAAAAGAGTCTGTATTTAGAAAGTTTTAAAACATCCCGTGCCT
CTCACAGTTGGGAGGCTGTAAACAATCACATGTGGCTGGACGAGCCTGATCAGGCA
GGCCAGAGAACCTTCAGAGTTCCTAAGTTGAAACACTCTTGTCACGCCCAGGAATTT
TTATTAACTTGGAGCTGCAAGTTAACTCCTTCTCCAAGAGAAATGTTTATGGGGGAG
AGCTCCCCGTAAAGTACTCTGGTTTTGGGGGTAGATGCTGGGGAACAGGGTGTATCC
TGAGGCTTGATCATACCTTTGTGTATGCCAAGCTTCCTTCCTCTTGATCTTTGGCATG
GGCAGAGTTCCTCATGCTGGCTCCCAACATTTTATCATTTCACATATTAAACACCAA
GTGGTTTTCCAAGGAATAAAACATGGTGATGTGATTATTTTAAAAGTTAGTCATCAC
ACAACAAGAATTTGTCCACTGCCCTCCATAAGATTCTAGAGATCAGTTAAGTTCAGT

TCAGTCGCTCAGTCGTGTCCGACTCTTTGCGACCCCATGAATCGCAGCACGCCAAGT
TCAATGACTTTTAAAAGCACTCACCGTATGCATACGTATATCCCCTCTTTTTTTTTTTT
GATATCCTTCCCATTTAGGTCACCACAGAAAACTTGAGTATAGAGTTCCCTGTGCTG
TACAGTAGGGCCTCATTACTTATCTATTTTATACATGATATTTGGAGCTCCTTTCTAT
CTGCTAGATTGGATGCTGCCAGATTCAAAATGATTTTTGCTCAAATAAGCTCAATAT
TTAAAAAATATAAAAGTACTCATCAAAAATAGCTTTGGTCACAGATAGTCTAAAGG
AGGACAGGACATTAGACACCATTTAGGTGGTATTTTCAATTAGACTGAAATAATTAT
TTGGCCTGTAATCTCATTTTACAAGGTTTCCCATATGCCTGAATGTTTATTCGTGGTT
TTAAACATGGAAGCTCTGTGAATTTCAGGGCAAGAAGAGTCCAATCCCTGTCTCCAA
GGACTTTGGGAGTTACCTATTGGATAGAGGAGAGCAAGACTCTGCTTTTTAATTTTA
TTTTTTCGACACGAAAAAACATTTTGTAATGGGGTATAGCCAACGAACAATGTTGTG
AAATTTCAGGTGAGCAGTGAAGGGACTCTGCCATATATATACATGTATCCACTCTCC
CTGGAGAAGGAAATGGCAACCCACTCCAGTGTTTTTACCTGGAGAATCCCATGGATA
GAGGAGCCTGACAGGCTACAGTCCATGGGGTCACAAAGAGTCAAACACGACTGAAT
GACTGAGCACATCCATTCTTCGCCAAACCCCACTCCCATCCAGGCTAGCACATAACA
TTGAACACAAGCTTCATGCGCTATACAATAGGTTTTTGTTGGTTATCCATTTTAAACA
CAGCAGTGTGTACATGACCTTCCCAAAGTCCTTAGCTATCCCTTCCCCCCAGCAACT
GCAAATTCATTTTCTAAGTCTGTGAAGAATCTGCTGCTTATTACCTAAATTCAGTTGA
GCAACAGAGAAGAGGGAAGGAGTCCAGGACACACTGCCAGACAGAGGCCAGAGGG
AGGGTTACACAGAGAAAGAACGTGCTGCCCTTATACTGCCTCTGGGTCTGGTAAGGT
TAGTACAGCTTAGAATTGAATATGATAAGAAAGCATTATAACTGGTTGGCACCAGTA
GTGTAGATTTTAAAATGAAAGACCTGAGATTGTGAAGAAGAAAAACTTTTTTCACAG
AGATAGCGTCTTTATTACCATTATCAATGATACCAGTTATTACAACTAGTTGTTTCAA
TGAGTGCATATATTTAATCTTATTTGAACACCATGGCATTACAAGATAAATATTGTT
ATCATCCTCATCTTTTAGAGGAGGAAACTGAAAAACAGAGAAGTCAAGCAACAGGT
CCAAAACTATTCGGGATACTCACTATCAACCCAACCACTTGTCTCCACAATCTGCAA
TCTTACCCACTCACTCTCTGTCCCAAAGTTCCTCTGCAGAACCCAGACTATCAATGTG
TGTCCCAGAGCACATGAAAAAAGAAAAGACCCAACCAACCCTCAAGGATCCTGTCC
TGTCTGGCCAGAGCCAATTGATCAACAGACATCAAGCCAAGCAAGCTTTTACAAGA
GAAACCGCCTTTGTGGACAATTGCTAAATTTGGGCTTCACAAGAAGTCAATAGTTAT
CAAGTTATTTGCTTGCCAGACCCAGAGCAGTCTGAATTCCCTTTGTTCCTGCAGCCCA
AAGGACTACCCCTTTCCCCATCTCCAGTCTCTCTCTTTCTTTCCTATACTCTATCATTC
ATTTTTCCTGTTTACAAAGATAAAAAATATAGGTCACAAAAGGTTATGTGTGTGTGC
TAAGTGGCTTCACTAGTGTCTGACTCTGTGCAGCCCCATGCACTGTAGCCTGCCAGG
CTCCTCTGTCCGTGGGATTCTCCGGGCAAGAGTACTGGAGTGGGTTGCCATTTTCTA
CTCCAGGGAATCTTTCCAACCCAGGAATTGAACCCCTGTCTCTTATGTCTCCTGCATT
GACAGGCAGGTTCTTTACCACGAGTGCCACTTGGGAAGCCCTACCAAAGATTGTAA
GATAATTTAAGTTGTAGGCTTAACCTTTCAAGTCAGATATACCACCTAAATACTCCC
TTTTGCCTTTTCTTAGAAATTTTTACTTGTCCTTCTAATTTAATTCAGCACTTCCCCAA
CTGTCTTTATGAAACACTGTTTCAGTAGATGTTAATAAAGATATCCTAGGGTGACAC
ATGGCCAAATAAGTTTGGGAAATAAGGCATACCAGGACCTTTTGTTGGAGCTGTGTT
ATGAGAATAGTAAGACTGAGAAATCATGAAGTAAGGAAACTGTTGAATATGACTTT
TAATTTAAATATTCTGGGTCCTCTTATGTACTAGGCTCAGTTTTGGTTTTGTTTTTTTT

TTTTTTGTATTGGAGTATGAAAAATGTTTTAAAGTGAGAGGAACAAAAGAGATGAG
AGGAAAATCCATATTTCTAAAAAGTCAACTTCTATGACATAGTGCTATAATATAGAC
TATTCATATTTTAAGAATGCTTTATGGTAAAAATAATTGAACCAAGAAAGTTTTATTT
CAAAATATTTTTCCTCTGTGCTTTCAAATGGAAATAAATTCTCCCCATGTTTATTACT
TTTTAAAAATACAACTGCATTATTGTTTCTGTCTTATTATGAGTTAGCATTTATACAA
CCTCAGGGTTCTTATTTCCTTAGCCTTGAATATAAGTGTTTGCTTTCTCTAACCCCTA
ACATTCCCGGTAAGATGATATGCTTTACCTGTAGTAAGAGACATTTGTTGTTGTTCA
GTTGCTAAGTCGTGTCTGACTCTGCAACCCCATGAACTGCAGCATGCCAGGCCTTCC
TGTCCTTCACTATCTACCTGAGTTTGCCCAAACCCGTCTATTGAATCAGTGATGCCAT
CCAACCATCTCATCCTCTGTCATCCCCTTCTCCTCCTGCCCTCAATCTTTCCCAGAAT
CAGGGGTTTTTCCAGTGAATTGGCTCTTTGTATCAGGTGGCCAAAGTACTGGAGCTT
CAGCTTTAGCATCAGTCCTTCCAATTAATATTCAGGGTTGATACCCTTTACGATTGAC
TAGGTTGATCTCTTTGCTGTCCAAGGGACTCTCAAGAGTCTTCTCTAACACCACAGTT
TGAAAGCATCAGTTCACTGGCACTCAGCCTTCTTTATGGTCCAACTCTCACATCCATA
CCTGACTACTGAAAAAACCATAACTTTGACTACAGGGACCTATGTTGGTAAAGCAAT
ATTCCTGCATTTTAATACACTGTCTAAGTTTGTCACAGGTTTTTAATCCAAGGAGCAA
GCATCTTTTAATTTCATGGCTGCAGTCACCGTCTGGAATGATTTTGGAGTCCAATAA
AATAAAATCTATCACTGTTTCCACTTTTTCCTCATCAATTTGATGTGATGTGACCAAA
TGCCATGATCTTAGTTTATAAAAAGTTGAGTTTTAAGCCAGCTTTTTCACTCACCTTT
TCCACATTCATCAAGAGGCTCTTTAGTTCCTCTTCACTTTCTGCCATTAGAGTGGTAT
CATCTGCATATCTGAGGCTGTTGATATTCTCCTGGCAGTCTTGATTCAAGCTTGTGAT
TTATCCAGCCCAGCATTTCTCATGATGTACTGTGCATATATGTTAAATAAGCAGGGT
GACAATATACAGCCTTGACGTACTCCTTTCCCAATTTTGAACCAGTTCATTGTTCCAT
GTCCAGTACTAACTGTTGTTTCTTGTCCTGCATACAGGTTTCTCAGGAGGCAGGTAG
GGTGGTCAGGTATTCCCATCCTTTTAAGAATTTTCCACACTTTGTTGTCATCCACACA
GTCAAAGACTTTAGTCATAATGAAGCTGATGTGTTTTTTTTTTTTTAATTCCCTTACTT
TTTCTGTGATTCATCAGATGTTTGCAATTTGATCTCTGGTTCCTCTGCCTCTTCTAAAC
CCAGCTTGTACATCTGGAAGTTCTTTTTTCATACTGCTGAAGCCGAGCTTGAAGGATT
TTGAGCATAATCTTGCTAGCATGTGAAAAGATGCAATTATACAGTAATTTGAACATT
CTTTGGCATTGCCTTTCTTTGGAACTGGAATAAAAACTGAACTTTTCCAGTCCTGTGG
TCACTGCTGAGTTCTGCAGATTTGCTGGCATATTGAGTGCAGCACTTTAATAGCATC
ATCCTTTAGGATTTGAAATACTTCAGCTGGAATGCTATCACCTTCTCTAGCTTTGTTT
GTAGTGATTCTTCTTAAGGCCCACTCAACTTCACACTCTAGGATGTATGGCTCTAGGT
GAGTGACCACACCATTGTAATTACCTGGGTCATTAGAAACGTTTTCGTACAGTTCTT
CTGTATATTCTTTCCACCTCTTCTTAATCTCTTCTGCTTCTGTTAGGTCCTTGCTGTTT
CTATCCTTTGTGCCCATCTGTTCATGAAATGTTCCCTTGGATCTGGTAAGAGACACAG
AAATATACTAATATATTTTTAAAATTTATATTCCTTATGCAACAGGAAATCTGTATCT
TAAACACTTGCCCCTTGATACTTAGTCTAATATTCATTTTGAAAATCACTGAAATCCT
TCCAAAGATATATATATATACACATATATATCTTATAAACTTAAATAAATTTAAATG
AGCTTTCTAAAATCTATACAGGCACCAAATAGTAACATAACAAAACACTTGTAAAA
GGCATCCAGTATGGTTTTCTTTTCTCAATATTAAGTAAAAATTTTTGTGCTTTGAAGC
AGAATTTTTAGTTCTCGTGCAATAGAAGTTGTAACTTTTGGCAGTTTAAATAAAAAT
TTATTTTAAATTCTAAGGTGAAACTACATATTGTTCCCTAACAGTGCCTAGGAAAAG

TAGACACTCAATTAATTTGAAATTAATTTCTTTAATCTCATATGTTTTTTGCCACAGC
AGCTTTTCATATTTTCTTCATTGGAGCCACTATCTTCTGGCAGAAACTGCCATATCTT
ATGAAAGATGGAGAAATATTCTCTGCCTTTATGTTAGACAACGTGTTCTTTGTGTTA
GTCAGTACTTGGTAACTGGCCTCTGGACTCATGCTTGAAGAAGAAGGTTCAAGGTTA
GGTTCTCCTTGAAGCTTCCCCTTCTAAAAGCTACCCTGTCTGTAGGTTTCCCACGGTA
CCATAAACCTTCCATTAGACCATCTATTGTATGATTTCATAATAATTTTTCTATGTGT
CTGCCATTCCATGAAGCTAGAGTTTTATTTTCATAATTTCTGGACCCAGGAATATCTG
GTGGCAAGGAGGAATAGTTTCTTCAGTAATTCTGTGAACCAATGAGTTTTTAACTAT
AATTTGTATATGAAAATAAATAATTCTGAAACATCAGAGTAGCATAGCACAATGCAT
CTGCTGAGAGATTTATACTGACACAAGGTAAGGATTTAGATAAGGGCAAAAACTGC
CTTTCATGATCATGCATCCTTATGTCTTCTGACTAATCTCTGATATTACTTGTCACCTG
GATGCATAAACCTAAGGTGATACATTTCATGCAAATGCTGGAAATCTTAGCATTTAT
CTAATTTTTCTAATTTTCTCTGTCAGTTGTCTCCTCTATAGGAATGTAGGCTAACCAT
ACCTAATTACAGAATTTTCCATAGATTTAAATAATAACATGGAAGTATTTAGCCTGA
AAACTGATCCATAATAATATGTCAACAAATACTTGTCCTCCCCCTTATCCTTCCCTAG
AACTTTTCAAGTTGTCTTTATAAGGAGGAATAAATAGTATTGTTTTTATATTTAATAA
TTTTATTTAGATAATTTTAAAAGCATATTTAAAATAAAATGTAAAAAGGCAAGAGCA
CTGGAACCAGAATAATTTAGAGAAAACTAACCTCATTCTAGTAACAGGGTACAGTA
TATGGGAATCAAAGACGTATAAACTTTTCCAATTACCCAGGAAAGTAATATTAAATT
GACTAGCACTTCTGAAAGTATAATGACATACAAACAATATTAAAGTAATACACTAG
CAAATATCAGGCCAGATCCTGGGAGAAGATATGTGAAAAGAGGTGAAATATACAAA
ATAAAAATAAACAAAAAGAAACATCAGGAGAAAAAGAGAGCTATATTTTAGCTGA
GAGGCTTTGTTTTTTTTAACTACTCTTAGGGCAATGTAAGATGAGCATTTCCTCAGAT
CCCTGAAACTTTATCTGTCAAATGATCAAGATTGATGATTCTTAAATTTTTTTCACAT
TTTGTAATATAAAGTCACGAGCAAGAGAGAAAAAAATGTGTCATACATGGATGTGGG
TGAAAGGTCTTTGAACTAAGAGTCACAGCTATTTATAACTGAGTAAATGTGGATGTC
TCGTGAGCCCAGAGCCTTGTGGTTGTCTACTCTAGAATCTTTGTGTGTAACTGTCCCC
TGTGTCTAAGCTACATCTCTAGAAGATGAGCTCCTTAAGGTAAGTGCTGCTAAACTA
TCCAAGCCAATTTGGGCACACACTGTTACTGGGTTATCTTGGTAAATGTTGGTAGTTT
TCAATAAATAACAGTGGGGAGAGCTACAGATCTTTCTCAAAACAGGTACAAAGAAA
GCCCTCGCAGTACTGGCTTCTAACAGAAGCAGGGAAGTAGCAGTTAAGTAGCAAGG
TTCCCCAACCTGTTCCATGGGATTTGTGAAGTACCCACTCTCTGTCCTTTTAAGTGTC
CCAGTAGTTCTTGCCTGGAAGTTTCATCAGGGGCACACACAACATGCTAACTATAGA
ATTCAGCTAACTATCCAATCCCAGTCAGGCCACCAGGAATGCCCTGAAATCAAGAG
CTAGACAATGAACTTGGTGGGTTTTTATAGCCTGTGATATAAGGATAGTTAGCCAGT
AATTTTTCTGGTCAGTTCATAAATCTTTGAAGCCATTAATTTCACCAGTGAAAACCTC
TCTGTTATATTTATTGTGAAAATTTCCAGAGTTTAGGAGAAAAGACAACTAAAATAC
ATATTTTGATTATATGCTAAGTAAACAGTTCTAGAATGAATATTTTCAACTAGGTTA
GCTGTCCACTTAGATTAAAAGTATTCAATACTTCATCATTGCATTTTATTTGCAAAGT
TTTAAAGTACATTACAAAAAATCTCACTGGAAGGATCAGGCTTTCTTCTTCCTGATG
ACAGGATTCTAAGAACACAGTGGACACATAGCAGATTTTCATTAAGTATGCGTGTGG
GTGTGTGTATGAGGATACATGTCATCAACTTTAATCCTTAGAGAATTAATGCCTTAA
GTACTATCAAGAAAATCATATTTTTTTAATCTAATTTAGCATATACTGTAATGGCTTG

ATTTGCTTTTAGAATATATTTATCTTAGTTTCTTCATTCTTATGCGTTGCATATATAAT
AGCATAGTCACCAAACATTGCTAATCTTACATGGCAAAAGAATGTCATAACTAGAAT
AAACTTGAGCTTGTCCATTTTGCAAAGCCCTGCCCAAGGCTGCTATCATCTCCAGCTT
TCCACAAACTAGAATATTCACTGATGCCTTGTTTCTTATCCTGAAGGCCAGTGTGCA
ATGTTGTTATGTGGTTCCTGTGAAGACTATAAAGTCCTACACAGAGACAAAGGATGA
AAACAGTCAAGGGAATTGCTATAAGAAAATAAGTAGGGATATCTCTGGAGAGGGAG
TTGCTTCTGTGTTCCTTTAAGCATCACAGAGTAATTGTTTCTTTTTTAAGGAAAGAAC
CAGGAGAAGAGAAGCTCATTTTCAAGCTAGAGAGCTTGGCAAACACAAGCTGCAGG
GTTTACTTTGGTTTAGGAATCCCACTGCCTCACTGGAGTGTCAATCAAAATTCCACA
GCCATTCTTGGATACTTGGAGGTGGGGCTGAGTGATGTACAGCAGTGATTTGACCCA
GCTCTTGAGGCAGACACAACAAGAGTCATCAGTGTTAATTTCGTTGTCCTCCAACCA
GCGTTCTGAAAGTGCGTTGACACTGGTGGCCTTGCACTTTTTCATCGGACTTCCAACT
GGTAATTACACCTATATTATCACTGTGAAGTTATCTAACCTAACAAAGTAGGACTCA
AAACCAGTGCCTGTAATACTAAAGTCTCTTACTTTGATCTGATTTTTGCATTGTTCTT
GTCCAGTTTAAGTTGTATGTGCATTTTTCCTCTTGTCCTAATTCATAGAGTATTCGAG
ATAGTATAGATATTAAGGAAGCCTGTCCCAAGCTTTTAGATAAGAAATTGTGTCTAA
AAGAAAACAGAAGGAGTTGGTCAGGTTTTGTAATTTTGCTATGATGTTATCAGTAAT
AACTAAATATTAGATAAGAGGACATTCAAAAATATGTGAAATTGTCTAAACCTTCCA
ACACATCTATGCTTGTATACATGCTGCAATATTGCTAGTAGAGCAATATATTAAAAG
ATCAAAAGCTATCTTTTATTCTAAAGAAAATACATGCCCAGGAAGACTTAAATTATA
GATGGCCACTTTTACCTGAAGAGGCAATTATAATGAATATTATTGTATAGGTTTGGG
ACTCTCATATTAAGATTGTAAGGCAGAGCAGTTGTTCTTTTCAGTCTTGGAGAAAGA
AGAATCTATCCTCCCCCATGGACAGGGAAGCCTGGTGTGCTGCAGTCCATGGGGTAG
CAAAGAGTCGGACATGACTTGGACACTGAACACCACCAACCAGCAGAGGTCACAGG
CACTTTGTACTGGGACATTCCTGGAATGGATCTAGTCCATTAAGGGTTACTGTGACT
TGCTAGACAGGTGACTTTGGAATTTTATTTTATACTCAAGTTAGGGAAACTCAGTCA
TCATACTGTATACCTGACTTGGGCTACTGAATCTTTAATGCAATAGCTAACAGGATA
TAAGCAGTAACATTATAAAATTTGCCATTAAATTAAAGCCAATTTTCTCTTTGCAAA
TCATTGTCACGAGGTTTCTCATGGCAAGAACAAACTACATAAAATGACAACGGGAG
GCAGAGAAAACCAGGTATAAGAATTAGGTAAAATTTTCTAGATTCCAGACTTAATC
ATTTTATTGGTCACATGTAATCATTTCTATTAATGACATTTTATTTCAGCCTATATTAT
GATACAGTGTTTTGTAAGGACAGACAGCATTGGCCTAATTTTATTTCATTGTGATTAT
CTTAAATAGCTGGAAACTGTAAATTAAAATTGGATGCAACTGTTTGCCTCAGTGAGT
ACTGTGTCAAGACAGCAGACTCAAGGAAGTGTTTAACATAGAAAAGGACAGGAAAT
GTTAAAAGATTATTCAAGGGACAATTCACAACTTGGCTTGCATCTTAGATGGATACA
ATTCTAATTGACATTTTGGTAGTTAATATTGTTACATAAGTATTCCAGACAATCCATA
ATTAAATATTTTGAATTAAACTCACAAATAACTGCAGACTTACAATTATGTATGTTA
CCTAAATGTAAATGATTACAAAATTAAAAAAACTGAGATGCATGCTTTATAGTAATG
CTACTAAACAGATATATTTAATATATATATAAAGTTAAGGACTTTATGAATGCCAGC
ATTATGTAAATCACTTTGGAGGGTTGGATTATGTGTTGAATCTTGTTTATTCACTCAA
CACACATTTGTTTGCCCACAACAAACTAGGTATTGATCATTTCCCGTTCCTAATTTCA
TCTACTTAACACTTTGGGTAATCAATTAATTCTCTAGTATGAACATCATTATTTAATA
AATTAAATCTATCAATACAGGGGACGTTGTATAAAGGTCACTGCTCTACTGGATAAT

TGGGGAAACTTCCACTGAGATTGGTACCACAGTTTCTCAGATTTTTCCCTTAAGGAT
GATGAAAGATCAGTGGACTCCTAAGTATCTCAAGTCTGTGGAGTCTTAGGTTGCTTA
GAAACCCGGTGACTATGGGCTTGCTATAGCAACCCAACTTATCAGTGAATGCAAAA
CTAACTTACCTGGCATTTTGCCAAATCTCCATATTTTTCTAGTATGAATATTCCGATC
ACATGACTGTTTATTATTCACACAATCAAGCTAAATACTACTACAGTTTACCAAATTT
TGAAAAGAATAACTTACAATAGCTAGCAAATATAAGAAAATTAAGCTTAAGCTCAC
TATGTAATTTTAGTTACTTGAAATTTGTTATGTCACCTAGTTATTATCCCATTGCCTA
CATCTGATTATATATATGGAAGATTTGCACTAAATTAGAAGCATGGGAAAGTTAGGC
TAGTACAGTGTATAATCTATGTGATAATAAAAAGGCTCTATCAATGACTTCTTTAAT
ATTTATAAATCCGTAAATTTATCCATACATTTATATGAGGTGCATTTTAATGATATTC
TTATTTTACATTATAAACCCCCTTTTCACATATAAATATATTTCTTTAAAATTATTTAC
CACATTCTCTTTAAAATCTTAAGCCTTCAGTAATATGCTGTGTAATCTGTATACACAC
ATGTATTAGAAAATTAATTTATTTGAAAAGCACATTTTTCCAATAATGGAAAAGATG
GGAATGTAATCTCATCGTCAGTCTAAGTATTTGCTCTTTGTAGTATATTTTTAACTAT
TTGCCCCCTTTGATTTAAGGCATGTGTTGCGATTTTAATAGTATCTTCAGTTTATCAG
TTTTTACATTGTTGTTCAACAATAGCGATCTCTGTACCTCTTCATATGTAGGAACTGT
ATTTTTCTTATCTCCTGTTGATTTCGACTCTCATGTTTATCACAGCCAATAGCACAGG
TAGACCCAGCAGCCTCTCCTGAGACTAGAGTCCTATAAGGTTTTAGTTACTGTCCTTT
TCTCCTGGGTTTTGGCATTTATGCATTTTGTTGATATTTAATAAGCGTTTGTCATATTA
AAAATTCTCTTGAGAATAAAAGAGTCAGTGTGACTTAGGTTTTTATGGGATAAGGAT
GCATTTGCTGCTATTTTCAATTTTTCTTTACACTTTATTTCTACAGAATTGTGCAATTG
ATAAAATTTTTGACCCCTCTAGTATATCAGTTTAACTGGGTAGTGCCGATGTTAATG
ATGATACATCTCATCTCACAGATGGAAACAAGTAGGAAGACAAAAATTGGGCTATT
TTAGTTAAATTGTCATCAGAACAGAAAAGCCAGTTCTCAGAGGTCCTTGTTTAAGGG
TCCAATGATTGGGCTACCCTGGCACCCTCAACAGAGGTGCATTTGTGCAATCGGATA
TTTTTTAACAAAATAAGTGAACAGTCCAGTACAAATGGACTAAATAACCCAAAGGG
ATCAAACTATTAGCAAGGGATCACACGTTAGCTTATAAATAGATAGTAGATTGGCAT
GTAAAATTAGATGGTAGAGTATAATACTCAGGAGACTTAGAATTTTTGATTGGCAAA
AAAACCAATACAAGGAATGATCTATATTTAATCATTATTTTTGGCCTATGATTGAAT
TATTTTCATAAATTTTTGAAATCTTCACTTTAATCAATATGTCTGTTGCTGGCAGTGA
AGATATAAACATAATTGGCACATTTGACCATTTAAAATATTTGAGAAAAGCTTATCG
TGTAGTGGTATGTTGTTTGTCCTTGTACTGCAGTTACTCAATACATATCTGTGGGACA
ATAGAAGTAAGCCCTCCTTGAGAATAAATATTTTTCAGTGTGGAAGAACCTGACTGT
TTAATAATTCAGATAACCAGTCCTTTTAAATTCTGTGTTGGATATCTTGGAAAAGTA
AATATATTCTGCCAAAGTATTAACATACCCATGTCTCTTTAATGTATGTTGTGATACT
TGTAGTATAGGTTCTTCAGTATTTATTTGATGAGTATCTGAATACCTATTATGTGTAA
GGAACTATTTTAAAATCTGGCATATAAAAGTAAATGTTCCCAACACAGTTCTCAGTG
AATATATTATAGAGAGTTCCACTGTGACCTCTCCCTTGGAAGTCATAAATGAGTATA
TAATGAACTGAGCTTTTTTGTTATACTTTGTATGAGTAGGACACTAAAGCATTCTGG
AGATATAAAGATGAATAAATATAGTCACGTGGCTTTTTGTATGTGCTTAATATTAAG
ATCTCTGCTTGTTGCACATGGTATACAAAACCATGTTGTAGGGACTGTTTATTTCCTT
TTTTTTAGGGACCTATTGATTAAACCACATTTCTTGATGATAGTTAACGAGGAGAAA
TTTGGTTAGATGTTTAGTTATTTAAAATGTTTGTCATCTACAATTCACAATTTACATA

GAATCTCAGGATATGAGTTTGAGTGTTTTCAACTCAAACCATTGTATCAATTCAGGG
ATTTAGGGTACACCTTAACTAAAAAACTTCAGAGTTAGCACTCTGTAGGCATAATTA
GATCAAACTGTTGGTTCTTTTGAACTTATTTGCTCTTGCCTGTTATTTGGGTGTTGGCT
TTCACACTAGTTTCCTTGAAGTTCTAAGATGGTTCCTGGCAGCATTCGTACATGTTTC
CTTATTACAAATAAATAGGAAGATGGGGAGAGATAATAGTGAAAAGATCATTCCCA
CCCATACTGAACAAAAGAGCAAAGCTTTTGTGAGTCCTGAGATGTATGTTTTTATAA
CACTGAAGTGCTTATAACGCTTTCAGTTCAGTTCAGTTCAGTCGCTCAGTCGTGTCCG
ACTCTGCGACCCCATGAATCACAGCACGCCAGGCCTCCCTGTCCATCACCATCTCCC
GGAGTTCACTCAGACTCGCGTCCATCGAGTCCGTGATGCCATCCAGCCATCTCATCC
GCTGTTGTCCCCTTCTCCTCCTGTCCCCAATCCCTCCCAGCATCAGAGTATAATGCTT
TAACTTATAACAATTAAATAGGGATTTAATAAAAAAAAATATTAAATAGAACATTG
ACATTTTTAGAGGCAATATCATGTTCAGTTCAGTCACTCAGTCATGTCCAACTCTTTG
TGACCCCATGGAAATATCATGTTAGTACCATGACAATACATGTTTTATAAAACAAAA
TATGGAGGAAACTTTTGAATCTTTTCTTCTGTATTCAGTGTTGTGAGTCATCTCCGAA
AACAAAGTCAGTTGCTCATGGTTTCTAGAACATCAGTTACTTCTAACTACATAGGAT
ATACTAATGATATGCAGAGTACATCATGAGAAACGCTGGACTGGAAGAAACACAAG
CTGGAATCAAGATTGCCGGCAGAAATATCAATAACCTCAGATAGACACCACCCTTAT
GGCAGAAAGTGAAGAGGAACTAAAAAGCCTCTTGATGAAAGTAAAAGAAGAGAGT
GAAAAAGTTGGCTTAAAGCTCAACATTCAGAAAACGAAGATCATGTCATCTAGTCC
CATCACTCCATGGGAAATAGATGGAGAAACAGTGGAAACAGTGTCAGACTTTATTTT
TTGGGGCTCCAAAATCACTGCAGATGGTGACTGCAGCCATGAAATTAAAAGACGCT
TACTCCTTGGAAGAAAAGTTATGACCAACCTAGATAGCATATTCAAAAGCAGAGAC
ATTACTTTGCCGACTAAGGTCCGTCTAGTCAAGGCTATGGTTTTTCCAGTAGTCATGT
ATGGATGTGGGAGTTGGACTGTGAAGAAGGTTGAGCGCAGAAGAATTGATGCTTTT
GAACTGTGGTGTTGGAGAAGACTCTTGAGAGTCCCTTGGACTACAAGGAGATCCAA
CCAGTCCATTCTGAAGGAGATCAACCCTGGGATTTCTTTGGAAGAAATGATGTTAAA
CCTGAAACTCCAGTACTTTGGCCACGTCATGTGAAGAGTTGACTCACTGGAAAAGAC
TTTGATTTGGGGAGGGATTGGGGCAGGAGAAGAAGGGGACGACTGAGGATGAGAT
GGCTGGATGGCATCACTGACTCGATGGACGCGAGTCTGAGTGAACTCTGGGAGATG
GTGACGGACAGGGAGGCCTGGCATGTTGTGATTCATGGGGTCGCAAAGAGTCGGAC
ACGACTGAGCGACTGAACTGAACTGAATGATCTGTTAGGCTAAGATAAGTTCTTGAA
GAAACATTATGATCTTCCTTTCCTCTGGGAAATTCTTAAACATGTGGAAATATCCCTT
AAAGTTTAGATTTAATCAAAAGGCTGAATTTAGTAATATTTAATCATTATGTTTCATC
TTTAAAACTGAAGCCAGTTCCCTAAAACATATCTCAACACATATGCATTGGAATTTT
TAAAAAACTATGTTGTCTATTTTCAATTTTAATTTTAATCACTTGATATTGGATGAAG
ATTATCTGAAAGGATGATATTTTCAATGGTGGAATACTTTATCACAAAAAGAAAAGG
TTTCCCTGTAATTAGTTTGCACCAAAACTTACAAAGAAAATCACTACAGTATTTTTTT
CCCTAACCTACGTGACATGGCAAATGAGCCAGAGAAATTTCAGTAAATAGCACAAA
TTTAGTTTAATCTTTTATTTTGTTTTAATCTTTATTTTGTTTCAGAGCTTAAAGAATTT
AAATGTTTTCTACTATACAGGATGGTAAGGTGTGTGTGCATAATCGTGTCTGACTCTT
TGCGACCCCATGGACTGTAGCCTGCCAGGCTCCTCTGTCCATGGAATTTCCCAGGCA
GGAAAACTGGAGTGGGTTGCCATTTCCTACTCCAACAGGATGGTAATATGAGATTAA
AAGAATAAATGGCAGAATATGGTTCAGTCATAACCAAAATATTTTTGGTAATATATG

GAAATCTGTGATTGTGTACTGAACTACCACTGGAGGGTGGATGTTTTTCACAGCAAA
ACTACCAGTTAAGTGGGTAATGTGAACATATCAATAAAAGGTGTGAAGGGACAAAT
GAATTTATGGTTTCTGGTAATTTAGGAATAGCTGTGATTTTATAATTGTAGTAAGTTA
ATTTGCATGCAGTTCCAAATTTACATACCTAAAACCACACTGAATTTTGAAAAACTA
TTAGATAAAAAATGAGATGTCAAATAAAAATCAACTAGGATAGATAAACGCAAGGT
AACCAAGGAAGAAAAAAAAGAAAATAATGAAAACTGGGAAGCTTGTGGAGGAGAG
AAATCTATCCTGAAACATAAAAATAGTATTGGGGGACAGGCTTCTTTATAACATGGG
AAATATTTAGTGGCTTTTAATTTTGAGTTGCATGAAATGGTATGGTTTCCATGGGCTT
ATTTCACCTCCAAATATTACTTTAATGTAGTCCTCTTTTCTACAACCTACTGTGATTA
TCCAGTTTTCCCTGAGGGGTTGCAGAAAATGAGAGGAAAAACAGGCCTGCAACAGT
CTTGGAACCTGTCCAGAAAATGTTTTTAGTCCTTGGACTACTTGAAGGACTGTGCTC
CAAACCTTGTATACACAGATTGGAAAACAATGATAATCCCAGTGTATTCACTCTGAA
TAGGAATTTTCTGTGTGTCATTAACAAAAGGAATATTACAGAATACACTGAAACACA
CTGGGATACCATAGGATCTTGATTAACATTTTTACCTTTATCCATGAACAGGAGGTA
TTGTGGAGTAGCAGAGTTTGCTTACTAAATACCACAACTGAATGTCCTGAAAGCCAG
GAAAACAGTTGTTAGAACAATCTCTGAGGCTACATGTATCAAGCAGTGCAGAACTA
GCAGGGCCAGTAGAGTCATGTGAGGGTGACATTCCACAGCAATTCAGATTCCGTAA
CTTGCTGTTTACTTAAATCACAACCTCCTAACAAAGTGAAGTTCGGGGATCTGTTAA
GAGGATGCACGATTTGAGTCCTTGGTTGCCTTTTGGAGAACACTGAGGATGACAGAC
ACTTTCATAAAGACAAGGCTGATGTTACCTAAACCAACTTAAGAATTGGAAGATTTC
CTTCTACTTAACCTAAGGTAAATTCCTTTATCCAAGCCATTGTCAACGCCCCCTAATG
TGAAGCAAACTTTAATGTGGTTACTAACACGTGAGTGCTAAAATGAAGTTTAATAGG
TAAGTTGAACTTCTCTTTTCCTTAAAATTTACTAAATTTTGTGCAGATACTAAAGAGA
AAGTGTTACTATACTCCTGAGTAGTTGTCATTTGCTTCATTTAAGCAGCTAACCAAAT
GTGTCACGGAGAAACATGCAGGACAATTGCAGGGTTCTGCTAATTTAGAATTCTGAA
TTTTTATGAAATCTACCTTATGCATTAAACAGTACTAGTCTTCCATTTTTATTCTGTTT
AGGTTTTCATGTTTATGATTTACAGAATACAGTCATGAAAATAAGATATATAGAAAG
AATTTTAAAGATTTATAACATTTCCAGGAAAATATTTGCTTCCAGAAGTTTTATTACA
TAGATAGATATACACTCTATATTTGGGTGTGCATATATGTTACAAGAATTGGTTTGTT
TTTACAAGCTATTATATGAGGCTTTGTTCAAAATGAAAAATACAATTAGAGTAAGAT
GTTCCAACTATGAATACTTATGGATTCTTCGAAATCTTAATACATTTCAAGTTCTTCG
AAGAAGTGTATTTGAGAAGTAGCACTCTAATCCAAACTCTTTGGCCTCTTGAAAGAT
AGAAAACTCAGGCTTGACCACAAGCAGATCTGCTCCTTAAGTCTATGAGAACAAAT
CTGGGGAAATGATGATTTGATTTGATTGTGATGGCCTTCCCTGGTGGCTCAGATGGT
AAAGAATCTGCCTGCAATTCTGGAGACCTGGGTTGGGGAGATCCCCTGGAGAAGGG
AATGGCCACCCACTTCAGAATTCTTGCCTGGAGAATCCCATGGTGGAGGAGTCTGGC
AGGCTACAGTCTAGGGGTCACAAAGAGTTAGACATGACTGAGCGACTAACATTTTC
ACTTTAGGGTTTGATAGACTGTTTGATACTGCTGTGCCACTCGAATGCAAGGTTTGT
ACCAAATGTTTTTTAAAAGTGTCAGGGGAGAAGGATCAAACTTTTCCATTTCAAGGA
AACAGAAAAAGAAAGGCATTAGGAATGTATTATTTTCTATGTGGTGATTTCTAAAAT
TATTATTACATGACATGCTTCTTAGAAAGTATTTTCTCAGCTTTAAAAAGGAATTTGA
TTAAGCTTCCCTGTCCCTGGAAACTGTGTGTGTGCGTGTGCCCCCATGTGTGTATGCA
TGTGTATGAGTTTAACTCTTTCAGGAATCTGTCACCCAAGTGTTGCATATCTCCAAAT

TTAACAGATTATCAGTTGGCACAGACAACAAAATTGTTAAAAAGACGGACTAGAGT
ATAGTTGTCTCGTTTTTAGGAAATGGAACAGAATTACTGTTCTCATTATCCATCTGAT
TTCTTTCCTGGTTTTAAACATTCATCATGCTCTTCAAGTTTTTTTTTTTTTTCTGCTGGT
AGTTAAAAAGAGTGAAAAACATCCTTTAGCATTTTAAAGCATATCATTTTGATCAAG
CCATCAACCAATGTGAATGTGTAAATTGATTCTCTAACTCTTCTTAAAATTATTTTAT
TTCCTTTTTAAGACCCTACAGGGACTATTTATAACGTAGATGGCATGCCTCAAAAGA
AAACTTTGTTGTACCCTCAATTCAAAGAATTTAGTATTTCTGCCCCTTGAAATTACAA
ATTAATTGCTCACATAATTTTTTCAATCTTTTTTGACATTGTTTAACTAACTAAATCTA
TATTTTGTTATTGATTATTGATTTTCCCCCTACTGTTTCATAACACCAAGCTTTGAAG
CCAAGCTATCCTCTTACTTGAAGATTTCTGTTGTTCCCTCCTAATTATTTCCCTTCCAC
TGTATGAACACAAATTGTACTGACAACTGTGCAAATCTTAACACTTTCTTAAGTTTCT
TAGTATTGTTTAAATGTTGACATTTCTCCACCTCCTCAGAGAGTTAAGTCAGCTTTGC
AAACAGTTCAAATAAAGATTAGATCTGCCAACATTTTTAGGCCTGTTGAATTTACAT
ATTTATATGGCTCTGCCCATATAAGATCTATCCAGATATAGAGAGGTTCTCTCTTTAT
ACACGGCTCCTACCATGGCTTCTAGGAAACACAGGGGAAAGTTTGCATCATGAATG
CAGTAGGTTTGTCAATACTTTGATCGTGTGCTTTGATTCATTAGATGATGTAGCTTAA
GGCTAAACTTTAATGACCCAGATATTCTGCATTATCCTTAATGTTGTCCTTTGAAATT
TTCCTTTAAAAGCGGTATTAAAAACTTCCATTTTAATCTCTTAAGCTTTCACTGACAT
TATCATGTCTATGTCTTAATGCATGTACTAGTCTCTTTAAATTATGTACGTATGTATT
TCATTCCGGTATGTGAAGCCCCACCTCATCATATGAAACAGATAGAACCTGTCATTA
GTTTAATACTTCCATAATGACCAGGGCAGCATTTGGAAAAATAGATATCTATTTTTG
TAGCACACATAATCATGTACATCATATGTACATAAATATGTGTGTGTGTAAATCTCC
ATGGTTAGTTATCCCAAGGATTAAAGTGATAAATCAAATTTTGCCTTATGGTTTTAC
ACTGGTTAACTAAAGTTTTAAGATACTTTACAGTGTATCTGTGTAGTATTATGTGTTA
TAGGTCTTCAGAATGAATTTTATTTTTTGAGTTAATTTCAAAATAATTCTGTTAACTG
TTAATTACAGAATACTTTCACTGTTCATTCCTTGTTTATTGTACTTAGATATCATTTTC
TTATATTAGGCTATTTAAAATTGTTATTCAGATTGAACAAATGTGCTAAAAAGAATA
TGCAAATTAGGTATTGATCAAGAACTCATATATATATATATATATATACACATACAC
ATTTTTCTTTCAGAAAGACTATAGACTCTTAGTCAGTCTATATGGGTTTTTATTATTA
TGGCACATAGTAGCTGTGTGACCTCAACTAGTTGTCTAAAAACATAGCAAGGTTGTA
TGATAGGTCAATAAGATAACTTATGTGAGACACTTATTTCAACAACTGGCAGATAAG
CCCTCAACACATGTTAACTCTGTTATTGCATCATCATCATAATTTATAGGTCTTTGCT
TAACCATACAATCTAGAATTTTAATGCACATGATACTAAAATTCTTTTCATTTGCTGA
AGTGTTAAAAAAAAAGAGGGTCAATTAAAAGTTTTTATCTACTACATAGAATCTCTA
TAAAGCTTTACAAATCTGTCGTGTGACTTGTCAGCTTGAGGCTGTAGGTAGGTTCTG
TAGTTACTCTCTTTGACAAGACAGGAAATGAGAAATATGACTGTGGGCTGCTTATTC
ACAAAACCTATGATTGTTATTGTTATTAATAGGGCTGTGGTCAGAACTTGGGCCATG
CCATGCAATTCCTCTTCTACTATGCTTCCATTGTATGCTTCTCAGATATGTTGCAATA
TTTATAATTACTCAATGTACTTCACTCTTCCTCTTTTACTTCACAAGTGTTATATGCCA
TGAATTAGCTAATCTAGAAATATATTCTAAAGGATTTTTGAAACCATAGAAATGCTA
GAATAAAGTTTATTATTTTTCCAATGTTTGGAGAAAGCATTTCAAAGCATGACATAA
TACTTAAAAGGGAATTACTGTGAAAGTTAATGGCATAAAAATGGAAATTTTCAGTA
ATTTAAAAATTCAATAAAGTTTAGTTAAAAACTTTTAAAAAAATTGTCAAATGTCAG

AAGACTGGTTATTTCCTTATTAGCTAATAAGGAAAAGATATCACAAATGTTTAAAAA
TATAAATGCTAATATGCTTTAGACAATTGGTGAAACTTCACTCAAATATTTAACAAT
AAAATAAGTACTTATAAACGGAGTTTAGTATGAGCCAGTGTGACCAATGTATGAAG
ATGGGCCTTTTCACACATTGCTGTATACATGCATGAACAGAAATTGGGTAAAATTCC
CCTTGCCAAAACAAGTACATACTTCTCACCCAGAAATTTCACTTTCTGCCATTTACTA
GTGTCTTAGCACAGTAAACAAAATTATATATGTACATAAATTTTTAGCATTGCTTTTG
TAAGATCAAAGAACAATTTGGTTAGCCAAAAAGTCTGTTCAGGTTTTTCCGTTATGT
GTGTATGTTCAGTCATGTCCAACTCTTTGCAACCCCATGGACTGTAGCCTGCCAGGC
TCCTCTGTCCATGGGATTCTCCATGCAAGAACACTGGATTGGATTGCTATTTCCTCCT
CCAGGGGACCTTTCTGACACAGGGATTGAATCTGTGTCTCCGATGTCTCCTATAGTG
GCAGGCAGATTCTTTACTCTGAGCCACCAGGGAAGAAAGGTGTAACAGAATGGAAA
AAGAAAAATCAAAAGTTCTATTACATATGATATTGGTCATTTAAAAGAAAATGGTAC
ATTTTTAGGACTATTTTTTCTAATAATTGAGAAAGAAAAAAGAGAGTTAACACTATC
AATGATATGAAAATATCTCCAAGTCCCTTGATATTAAAGAGACAAGTTGTAGAAAGT
GTGATTCAGCATATGGTTTAGTCCATATGTGTATGTGTGTGTGTGTATATGTTATAAA
CTTTTAAGTTTATACTTAGGAAATCAGTAAAATGATGACTGTATGCTGTGTGTTTATA
AGTTTATAACATACAGAAGTATATATATATATAAGTTTATATATATATATATATGTAAG
TTTAAAATTAATAAAATTCCTAGGTGGTAGTGACATACCTAATATTGGTCACTTCTG
AGGAATAAAATTGATGCTTGAATGAGACTTCTCATTTTACACCTTTTTATAATATTCA
ATATTTTTTACTTGTCCATATTACCTTAATGAATTCCCTAAAATAAAAATTTGCTTAA
TAGAAAATGATCACCATACCAATTATTTATTAAACAAATACTATATATGTCAAGAAC
TGCACTGAGTTCATCACATTACTACTCTCATCTTTGCAACAATTCTGAGAGGGAGCT
ATTTCCACTATGACTTACAGAGGTAAGGTAGTAGCAACAAGTCACACACTCTAGGA
AGGGATGAACTCCAAATTCTGAGTTAGGTCCATTTACTTCCATAGCATATGCCCTTC
ATCTCAAAGCACTGTATATCTGTGGTATGAACAAACAGTATAATAACCTCTTATTTC
TGGCCATTCTCATTTCTTTCAAAAATATTATTGTGGTTATGACAATCATGTGCATCCA
GTCATTCAACTGATTACCAGCTATGACCCTGCTCTGTGCTCCTGGGGCTTCAGCAGT
GCAATAAACAGACAAGAGTCTCTCATTCCATGCAGTCAACATTCTAGCAAATGCAG
AGAAAGGACCAAAAAAAAAAAAAAAAGCCAAATGCTTGTCTGATGGTGCTGTGTGAT
CTCCAGTAATCAGAGCAGAAGGGAGAAAGGGACAATGACTTGAGGGGAAGGGAAG
TGCTTCATTGCCTCACAGTCAGGCCCCTGTCAAGTGCTGCACACAATCCACCTCAGT
TAGTCCTTATTATGATCTCTTAGTTTTCTCCATTTTTTGATTAAGAAAGTAAGAACAA
ATTGTTAGTTAACTCCTAATCCCCAGTCAATATAGCTAGTGGTAGTACATTGCAAGT
CGCTGTAGTCTGTCTGATTCCAAATTCTAACTGATCTTCCAGGTAAAAAGTTTGCAA
AGCACTGTGCCTAAGCTTACTTAGCATACTGACAGTTTGAGCTTTCATTCTGCTACAT
CTGTTCTAGTGGACTTACAGAAGGTAATGTATACGCTCATATAAAAATATCCCTTAA
AAATACTGCACATACATAATCTTTTAAAAATGAAAACCCCAACATGCATTACTGAGA
AATGGGTGCAGCAGTTTCCTGCCTTGTTGTTCAAGGTGAACATTTTGCTCAAAATAC
AGACATATGATTGCAGCTTTTACTTGGCTCTGGGTTTCCTGTGTTTTCCTGTATCAGG
AACTGTTGTGCACAGTATTAAAACAGTTAACAGCTGCCTCTGGGAATGCAAATTTGA
TTCTTTGGGCTGCTCATGGGAAAGTGTGACATTTTCCTCTCCAAACACAAAGAATTT
AGATGTCATTAGACTACTGATTAAATAGTAATCCAAGTAAATTCAGTGTCAGCTTAA
GTATCAATTTTACAGGTATTTTTTCTGAAATATCTTACTCTAACAATGTTCATTTTTTA

TATAATTTAATTATCCTTTCAGTGCATTTAAGATAGGCTGTTTAAAATAGCATCTGAA
CTTCTGTGTTGGTGGGCTATTTCTGAATTGCCACACTGGAGGATATTTGTGTACATGT
GTACTCGTGTATATTTGTGAGAATTAAGGCTGAGAAGGAAATGTTATCAAAGCAGCT
TAAATACAATAAAATAGATTTTCAATATCAAAATAATTATTAAACTAGCCTTTATAT
TACCTTTTTATTAGTATAACAAAGATAAAAAAATGTCACAGTGATAGTGATAAGGGA
ATCATCTTTTTAAATTATCTAAAATCTAAAATTTTTAAATTATTCTTTCTCAAAAGCA
TAAATCATAATGAGATTATACAAGGCCATTTCTACTTTATCCTAATTAAGTGATATTT
GGAAAAACAATCCCAATATAACTAAAGACTTCTATAACATTGATTTTTAAATAAATT
TGTCTATAAAAATATCCTGGAAAAAGAAAGCAATGCTCAAGGTAAAATATTGCTTTG
GGTTCCTTTCCAGTTGTATTGCACAGCTAGCAAATGAGAGAATATAACACATGCCTA
GAAGCAATAGCACAAACAGCCCAGTGAGGACTGGTTTAAATATGATCTAATGAAGT
AAATGTATTTCTGCCTGTTATTGTTATGCTGTTGTCTTTCAAGGCTGAGTTAGAGGCA
TTTATTCATCTGGTCTGACAGATAACAGTTTAACCTGAAGATTTTTATTTTCCCTGTA
GGATTCTGAGAATTTCTTTCTAAGAATGGGATTTTCTTTTTATTTTTAATTTTTATTTA
TTTATTTGTTTAAGAATGGGATTTTAACATGTGGGTCTTTTCTCCCCAAAGCTTAAGG
TCTTCTTTTCTGATGAAATTTTAAAATAATGCCTTTCTTTTAAAAGTAACTTCATTAT
ATACTTTTACTATTTAGGATGGCTTTTAATATGAAATTTTATTTAAATATTATTTAAA
ATTGTCTTTTTATCTAAAGAGATCTAAGAGAAATACAATTTCTTGGTTTTCATAGAAG
GATTTTGCTCTGCTCATGAAATTTCTGTAAGACTAACAGAAAATAATAAATAGTGAA
GAGCTTCCAGTTAGAACAGTAGGCTATTTCCTTTTTTAAAGTCTTTTTGTCATCCCTG
TTGATTGACGAAGTCCAACTGTTTTAAAAATCATTAGACTTGACACACTTTGAATAA
ATGAAGCATAGAAAGTACATTTTATTTAAACTTCGTGAAGTAGGTTTTTCAAGCAGG
AAAGTGAAAGAAGCAGGTAATTCTTCCTAATTTACATTTTCCACCCCCAGGCCCGGT
TTATCATCTGGAATAATGACTGTATATGAAAGGTCGCTCAGCAAGCTAGCTCCTTTT
GTCTCTCTGGCCTCTGACTTACTTGGATGGGAAAATGGCCAACAGAAGGAAAAAAA
AAAAGACAGCAGAGGATCTCAGATATAGGTATTAGGTCTGCACTACAATGTAAAAA
CAGACCCTCAAGACCTATCACACCCCTCCCACACACTCTAAGTGGGAAGTGTGGGGT
TTCCTTTTTTTTTTTTCAAACTCCTTTTGGAACAAACACTCATTGGGATCTGACTGAT
GTTGCCTTTCTCTTTTTTTTAAAGTATATTTAAACTCACACACTTTTTATAATGAGGAT
TTCAGTGTAGGGTTTTCTGCAGGATACCATTTGGTCCTACTAAGAACTGACTGTTCA
AATGTTGGAACATTTGATCGGAAAATACTTCTTAGTCTTTTTAAAGGTAAGTCATAC
TGATTTTTTTTTTTTAATTTCTGTCTCGTAAATGACAGGAGATGACACTATTATATAC
ACACCCTTAAAGTTAAAGAACAGCAGCAGTAAGCAGGAACTGTTTTTTGAAATCATT
AGGCTTTTCCTAAAAGCTAGGTTTGTGCTGTTTCCAAAGTTATCAACCTTACCTATTA
TGTTTAAGAAATATTAAAACTTTGTCTAATAATCAGTGTCATGTTGCTTCTTGGTGTT
TAAAACCTTATGTTTATGCATAAACAGTTTTTGTTAAAATGATGTACTATCCAATTTT
GTTACTTCAGAACTTTTATTGTCTACTTAGGGTATTTGTGCATGGTTTTAAATTATGA
ATGAAAATCAACATAAATAAAAATCAGTTTAGTAGGATTAGTTCTTAAAAGTCACTT
GACACTCTTATGCCAACATGCTTTCTAATATGCAATTCTTCAGTGCTTATTATTTTAG
AGAGTCTTTGGTTTATTTAATCTTCTATTTTTTAATGTCACAATTTTTTGGTGAACCTG
ACTTTGTCTACATGAACAGCAGTGTATATTTTGGATATATTCCTCTTCAGCATTTAAA
AAAAATTCATTCTCATTGAATTTTACTTGCTGCTGGGAATGGTAAATTAATTCCAAAT
CATTTTGATTTATATAACTGTATAAATTATATAAAACTTGGTAATAGAATTTTTTAAA

AATTGAAGTTTTTGTGATTTTAGGAAACAATACAAATAGCAAATTATTTTCTTGAAT
GACAATACTAATAAATGACAAATCCTTCTGGAGAAATTAAAAGCTATAAATTAATTA
TAGTAAGTGAACTCATACATAACTGTAAATGGCATAAATTCACTACAGTAAATTTCA
AACAGTTTCTAGAATTGTCAAGTAACATATTGAAAAATGTTTCTAAAATCTTTGCAT
GTACTTCAGTTCTTTAGTGTGTAGTTTTAAATTAGTTAAAAACAAAGTGCTCTTAATA
TTAAATCAAAAAATAACCAAAATAATATTTTTCAAAAATTTTGTTATATAGAAGAGT
CCAGTGGTCCACTGGATTTCATGAGAGAAAAAAATACACAGAAATTTGTTTCATTTA
CAAGAATCATTACAAGAACAGAACAATAAATTTAATACACAAACATCAGATAGAAG
TTAAACTGAGAGGAATAAAACTATACTTAGGTACTGGACAGAATGCACTGAAAATT
TTGGAATTTATCTTGGGAGTTACAGTTGCTACCCTATAGGCAAAAATCTTGTGGATT
CTTTCTTCCTGAAAGAACTGATTCTGTAACTATTTTCTGATTTAATTACAAGGCATTT
TTTAAATTCAGTGGCCAAAATCATGGTTGAAAGTTTTGAGAAATTATTCTAACCTAG
GAGTTGATTTTTCATTTGCATAATATTCTAAACCCCTGTATACCTAAGAATAAGCCAT
CTCATCAGATTTTATTGTAATCTAAGAGTTTTAGAAATGATCATTTATTATTTATGAA
AAGACACATTTACTAGAATTTTAGAAAATACTTGGCCAATTCACAATAATATTTTAC
TGTTGTTACAGTTCAATATATTTCATAATATTGCAAATAAAATAACAGGTGCTATGA
TAGGAAATTTTATTCCTTCTCACTGAATTTTACTTGACATTTATAACCATATATAAGA
CTCTGATGCTGGGAGGGATTGGGGGCAGGAGGAGAAGGGGACAACAGAGGATGAG
ACGGCTGGATGGCATCACGGACTCGATGCACGTGAGTCTGAGTGAACTCTGGGAGC
TGGTGATGGACAGGGAGGCCTGGCATGCTGTGATTCATGGGGTCGCAAAGAGTCAG
ACATGACTGAGCAACTGAACTGAACTGAATGATGTATATATACCCATTTGAGAGAA
AACTGAGCCACAGAGAAACTATGTTAGCCTGCAAAGCAAACCTAAATTACTTGAAT
TCAAAAGCCCAGAATAATAACTATTGAAAATCTTATAGTATCTTCCCATTCATAGGA
AGAGTTGGGTTGGGGAAAAAGAAACAATAGTGTTTCTATCAGGATTCAAAAATCAA
GAATGGTTCTAGATAATTCTATTTAAACTGATAAGACTCTGAGAGAATCATTTCAAA
TCTATAGTAATCTCTATAATCAAGTACTCTAAAACCTACTCCAAAAGATTGGTGTGT
TTCTAGAAGAACATTTAATGTGATAATGAAGTCAATGTAATTATTGTTTTAATTATTT
TAAGATTACCACAAAAATAAAAGTTGTTATATTTGAAAGGCATAATTTAGTGAAGTG
TGTTATATTATGATATCTATACATTTTCTATTATATATATTTACCTCCATATATTCTGA
TAGGATGGAAATGCCCATATACATTAAAAAGATTATGGTAACTTCAATAAGAGAAG
ATGATGAGGATTTCTGAATCTAATTTAAACATTTACCACAGAGCCCTAGGGTTCTGC
GGGGATGACTCAGAGCCCAAATGAGATGTGACTAGAAGGGGAAAGGGCCACAGTT
GCTGCCCACTTTGATCAGAATAGATATTCTTTTAACTACCTTCAAATGTTGGGGTTGC
ATGTAAAATAAGTACTAATCTAGTGACCTTATGTATTCACTTGATGTAACACAACCA
GATTTAAATAACTGATTTAACCAGCTTGATATGAATCTGCATCTTACTCATGTCTAGG
GAATCATGGCTTTGAAAACCCACCTAGATATCATGTAAGGTAAGAGTGTGAAAAGG
AAGTTAGTTTGGAAAGCTAATTTTCATATTCAACCTGTCGTTGAATTAATCGCCCCCA
AACTCCAAACCTGGGTTTTGTGGGCAAATACTGTATTTAGTGAGGAAAAGCCGTGTA
TTAAAAGAATTACTTGTGTGTCCAGTAGAAAACAGAGATGATCTGGTTTCCTATTCT
GAAGACAAGCAGAAGGAAGGCTTCTGGATAGAATAGGAAATTTGGGATATTCACTC
ATGAGTCAGAAAATGTCATCCAGGTCCTGCATTCATTCATAGCCCAAGGATGTAATA
TGAAACTGTTTTAAAGTTTCATAGTTCATATCCCAGGAAACCATACCTCTTGGCCAT
GCCTTTTTCTGGGTGGAGTTTCCAAGGAAACACAGCTCTATACACACATGAAGATAA

TCACAAATTCTCTGGGAAGTGTCTCTATTATTCTGACCTACTTATGGGCACACTTCTT
AAAGCTCAGTCTCAGTTCCGACCACCAGGTTTCCAACACAAATGGAAAGGCATAAC
ACTCATAAGTTCATCTAGAAACACACTGTTGACTTGACAGCAAGAAAATAAACAGC
CCACATGTAGTTACATTCACAAACTCCACAAAAGAGTAGGATTTACAATCTGTGAAC
TGACAGGACACAGTCAAGAAAGGTATGGGAATAGTGGGATCTTACAATGTCTTTATT
TTCTTGTCTTTTTTCTTTTAAGTCTCAAAAATAGGTTTTCTTTCCTGATTATATGTGAA
TTTTTTGTTTTCTAAATTGAAGTACTTTAAGCAAGCAGCTTACTCCTTGGAAGGAAAG
TTATGACCAACCTAGATAGCATATTCAAAAGCAGAGACATTACTTTGCCAATAAAGG
TCCACCTAGTCAAGGCTATGGTTTTCCAGTGGTCATGTATGGATGTGAGAGTTGGAC
TGTGAAGAAAGCTGAGCGCCAAAGAATTGATGCTTTTGGACTGTGGTGTTGGAGAA
GACTCTTGAGAGTCCCTTGGACTGCAAGGAGATCCAACCAGTCCATTCTAAAGGAG
ATCAGTCCTGGGTGTTCATTGGAAAGTCTGATGCTAAAGCTGAAACTCTAATACTTT
GGCCATCTCATGCGAAGAGTTGACTCATTGGAAAAGACTCTGATGCTGGGAGGGAA
TGCGGGCAGGAGGAGAAGGGGATGACAGAGGATGAGATGGCTGGATGGCATCACT
GACTTGATGGACATGGGTTTGGGTGAACTCCAGGAGTTGGTGATGGGCAGGGAGGC
CTGGCATGCTGCGGTTCATGGGGTCACAAAGAGTCGGACATGACTGAGTAACTGAA
CTGAATTGGACTGAAGCAAGCAGTAAATTATTTCTAATAAAGAAATAAGGGTAAAG
AATAAAATTTAACCTAAAATTAGCAAAAAAAAAAATTTATTAGGAAAGAATCTGTA
TGACTAAGAAAACCAAGTGTGCAAAATGCAAATCTTATCTGCTCTAAATAGCTACAT
TTGAGAGAATAGTGGAGACAAACTACACTTTATACAGCTGTTGTTGAGTACAGCATG
CTGAGTAACCTGAACTTTTCTCATGTATTCAGTGCATGTCCTATTTAATTTGTTGGTT
CCTCCTTTGCTTCAGGTGAATAAAACTACGGTGGCATGTATGTTCGGTCGCTAAGTC
CTGTCTGACTCTTTGCAACAACCCCATGTACTTACTGTAGCCCGCTAGGCTCCTCTGT
CCAATGGGATTTTTCCAGGCAAGAATATTGGAGTAGGTTGCCATTTCCTTCTCCATG
GAATCTTCATGACCCTGGGATTAAATCCACACCTCCTGCATTGGCAGGCAGATTCTT
TACTACTGCGCCCCTGGGAAGCACAAATTACAGCTGACCCTTGGACACAACAGGTTT
GAACTTTGTCAGTGCACTATGGAGTATCTTTTCAATAAATACTACAGTACAACCTGA
TCCCATATATGCAGTTGGTTGGATACCACAGGTAAGCTGATGGTGTAGTTATATGTG
GATTTTCCACTGCCTGCATTGTCCATAACCCTCTGTGTTCAAGGGTCAGCCTGTATTT
GTAATTTTGTAGTATGAAGCATAAGCTTGAAGTATGATTATAATAACAACACTTATG
GTAACAAGGCACAGAAGCATGATAAACCCAAGACCCTCAGTGGGTGCCTGAGCCTA
GAGATAGCACCAAACCCCAAGTAATTCCCCTTCATCCAAGATTTTACTTTCTACAGA
TTCAGTTAGCTTCAGTTGACCACTTTTGACTGTGGATAAGTGAAACCATGGAAAGCA
AAGCCATTGATAAAGAAGGATTACATTTATATAAAATATCTTGCCTATTTTTAGAGA
AAAAAAAAACTTGCATCAAGATAACAAAATAAGCTGGGCAAAAGTTCATAAAGTCA
ATCTCTTTCTGTCAAAAATATCAGTTTATGTATGCAAGAATGCGAGTCACACTACTA
TTTTGCTTAGACATCATCCAGACCAGTGTTATATTAGTAACTGTCTAAGCATGCAATT
TAGCAGAATAAATCATTGACACTTATGTTTTCTGATGAACTTGACTCAACAGCACTG
GGCTGTGTGCACTATCTCACTTCAGTTTTAAGTTCAAAACAGACAGAGTCTAGGCAG
TACCCAGGTCTGCCAATAACTTGACATCTCTGGGCGCAGGGTCCTTGGATGGTATAA
TAATGCACTTTCATGTTTAGAGAGGAAGGTGCTAAAGAACACAGTGGTTCATCCAGC
AGATTTTTGGGGACTTTTTGCCCCTTGGGACTCTTCACTATAGTCCATGGGACTACTT
AAATAAAGGATAATGATTCTGGGAAAGGTCAGACAGATTCTCAACTAGGTGAAGGC

ACAGAATCTGGGAACAGACAAATAATACACACCTATATCCATTTCAGAAATAAAGA
TTTATAATTTTAACATCATTTTATATTCTATCTGACAGAACTGCAAAGGACATTAATA
TTGAATAATTTTGTGGCATTTATGTAGACCATATCTTTCCAAAAGAGATGATTTGAGT
TGTATTCCCGATGTTATATAAACAAAACTAGAATCTGACCCTTGGGGTTTCCTATCC
AATTTTTTAATAAATTGCAATCCACAGGTTCTACAGAGATGCTCCTCAAGCATCCTA
AACATGGGACAAGATAGTGCTAGGTGGGACACAGGAGACCTAGTTAGGAATCAGGG
TCTCGGTGGTCCCACAAATGCTGTAATCTAAGTCACTTGGTTTGGTCTTCCATGTAAG
GATTTGAGTGAAGAATCAGTCTAGCTGCTTAAATGAAAAAAATGGGGGTCTCATCTT
ATAATCTTACTTTATACATGAAGAAATTGAGGAACAGAGCAATTAAATAGCTTGCCC
ATGGTCATCTGGCAAGAAAGTGGCCAATCGCACTTTGGTTTTATCTCAAGTCTCCCA
CCCTTAATTACAGCAGTTTGGAACCCTGCATTTTGATGTCTACCTCAAATATAACTTA
CAGATGAGAACTGGGGAATGGTCTATAAGATGGGCATTCAGTAAAATAGTACTTGA
TAAGCTCCCCTGAATATGGTCACTTACATACAGTACAGCACACTCGAATGCTATTCA
CAAGTAGAAGAGAGATTCTACTTGAGAATATCTAGTCATTATTATCAAGTTTAAATA
ATTTATTCCACTGTATTATGACAATCCTAGCCAAAGCCATAGGAGGGAGCATGTTAT
TCCTCATCCTCTGTGTTAAATCATTTCAGTCATGACCAATGTGACCCTATGGACTGTA
GCCCGCCAGGCTCCTCTGTCCATGGAATTCTCCAGTCTAGAATGGAGTGGGTGGCCA
TTCCCTTTCTAGGGATCTTCCTGACCCAGGAAACTAACCTGTGTCTCTTACATCTCCT
GCACTGGCAGGCGAGTTCTACCCCCAAGCAATTAATAAAGGAAGGAAAGCTATGCC
AAACTTAGTGTACTGAAAAGCAAAGACATCACTTTCCTACAAAGGTCTGTATAGCCA
AAGCTATGGTTTTTATAGCAGTCATGTACAGATGTGACAGTTGAACCATAAAGAAGG
CCGAATGCCAACAAATTGATGCTTTCAAACTAGGGGCTGGAGAAGACTTTTGAGAG
TCTCTTGGACTACAAGGAGATCAAACCAGTCAATCCCAAAGGAAATCAACCCTCAA
TATTCATTGGAAGGACCGGTTGAAGCTCCAATACTTTGGCCACCTGATGTGAAGAGC
CGACTAATTAGAAAAGACCCTGATGCTGGGAAAGACTGAAGGCAACAGGAGAAAG
GGGTAGCAGAGGATGAGATAGTTCTATAGCATCATGGACTCAATGGATATGAATTT
GAGCAAACTCCAGGAGATAGTGGAGGACAGTGGAGCCTGGCGTGCTGCAGCCCATG
GGTTGCTAAGAGTTGCTAATGACTTAGCAACTAAACAACAACAACTCAACTTGCTCA
GAGACATAAAATTCCACGGGAAAAATAAACCACTTGTTTAGGCTTGTGTGTTTTGTT
TACATTGGGAAAAAACATGTGACATTAATGACTATTCAAACTTCTTAGCCACCAGGA
CCTCAGTGTGAATCAGTTTGTTCATCCTGATTTACAGTGGATCATCCTAGGTATTTGG
TATAAGAGAATGGAAATCCTGAAGAATGAAAAAAGTTTGTAAGATTTGCCTTTTTCC
ATTTTGAGGGAAAGTCTTATTCCTTTCTTTTAATAACATTATTCTTAACATTTTCTTAG
CTATGCAATTAATAATTTTAAAAAATTAAATGCTTACTCAAGTGGAAAGAAATACTA
ATAAAACCATCAGAATGGTACTGTAACCACAACCAAGCTATGGAGGGAAAAATCTG
AAACCATAACGAAGTATGATTTTTACTATTATCCTTACAAGACTGACAAAATAGTGT
GTGTCCCTCCAATCTGTTGCAATTTTTGTCTTCCTTGGGCAAGGGTATTCTGAATTAG
GGAGTATTTGTTTACAAGCTACAAAGCACATTCTGTTTATCAATGTCAGGTCAACTC
TAGTACAGATTTCCTACTGCAATCAAAACTGCTTAAATAGTATGTTTTAGGTATTTGA
ATAATAAAGTCTTGTTACCATAGGATCTGCTTATTATGCTAGGAATTTTTTCCAAAGA
TAAGACTAAGTGAAATACATATTGACACAATCGAGTTGATTTTAACAGAATTTTTAA
GAATCAGTTTATTGTTGTTGTTTAGTCTCTAAGTGTGTCTGATTCTTTTGCAACCTCAT
GAACTGTAGCCCACCAGGCTCCTCTGTCCATGGGATTTCCCAGGCAAAAATACCGGA

GTGGATTTCCACTTGCTTCTCCAGGGGATCGTCCCAACCCAGGGATTCAACCCACAT
CTCCTGTATTGGCAGCCAGATTCTTTACCACTGAGTCACCAGGGAAGCCCTATGAAT
CAGCTCCCTACAACGAAAGGAACACTATTGCTCTTGAATATGTTAAAATTTATGTAT
TTGAGGCTAGTAAGAAAGCAATACCTATTACAGAATCTAGATATACTCTACCTTACA
TATTTTCTTCAAGTTCTGTTTACCTTTAGATGATATAAAAATCAGTTATTAAAAAGAA
GTGAAAATATTATGTAAAATATTTCAACATTTTCAACACAGTTGATAAGAAAGAATT
CAAGCATGAACTCAATATCCATGAGAATGGAAATTTCTTTTAGCAGTGATTATAATT
TTGGTGATATTAGAGAGTAGTGTAGTGTTAATTTTCTGACAATGTATTTCCCTAATTT
ACTCATGTTTTTTGCTTTAACAGATAGGCTACCAGTGATTAGAAGAACTGACTCCTTC
TAAGACTCATCAGATCATTTCCTGTAAAAACTGTATCTTTCTAATTGTGAAAAAGGT
AAAGAAGACATTTGTTATCATTCTTGTTTAGAATTTAGATGGAATAGACTCCAGTCA
ACCCACATTCAGTTTGCATAACACCAGGTTCAGTTCAGTTCAGTCGCATCCGACTCT
GTGACTCCATGAACCACAGTACGCCAGGCCTCCCTGTCCATCATCAACTCCCGGAGT
CTACCCAAACTCGCATCCATTGAATCAGTGATGCCATCCAACCATCTCATCCTCTGT
CGTCCCCTTCTCCTGCCCTCAATCTTTCCCAGCATTAGGGTCTTTTCAAATGAGCCAG
CTCTTCCACATCAGGTGGCCAAAGTATTGGAGTTTCAGCTTCAGAATTAGTCCTTCC
AATGAACACCCAGGACTGATCTCCTTTAGGATAGACTGGTTGGATCTCCCGGCAGTC
CAAGGGACTCTAAAGAGTCTTCTCCAACACCACAGTTCAAAAGCATCAATTCTTCAG
TGCTCAGCTTTCTCTATAGTCCAACTCTCACATCCATACATAACCACTGGAAAAACC
ATAGCCTTGACTAGATGGACCTTTGTTAACAAAGTAATGTCTCTGCTTTTGAATATGC
TATCTAGGTTGGTCATAACTTTCCTTCCAAGGAGTAAGTGTCTTTTAATTTCATGACT
GCAATCACCATCTGCAGTGGTTTTGAAGTCCAAAATATAAAGTCAGTCACTGTTTCC
ACTATTTCTCAATCTATTTGCCATGAAGTGATGGGACCAAATGCCATGATCTTAGTTT
TCCGAATATTGAGCTTTAAAGCAACTTTTTCACTCTCCTCTTTCACTTTCATCAAGAG
GCTTTTAGTTCTTCACTTTCTGCCATAAGGGTGGTGTTATCTGCATATCTGAGGTTAC
TGATATTTCTCCCGGCAATCTTGATTCTAGCTTGTACTTCTTTCAGCCCAGTGTTTCTC
ATGATGTACTCTGCATATAAGTTAAATAAGCAGGGTGACAATATACAGCCTTGATGT
ACTCCTTTTCCTATTTGGAACCAGTCTGTTGTTCCCCAGGTAGTACTGTATAAATGGC
TGTGTTCAGGTGGAAAAGCCAGATAGAGGGAAAAAATGTCCATAAGTGTCCCAGGC
TGACAGGTAGGCAGAGGGCAAGAGTACATATGCATATTCTCCATAGATAAGATTTC
AGTATGTTAATTATCCACAGAAAAAAAAAAAAAGAGGAGGATTTTGAGGAATTGCTC
TATGTGATGATATTTATTGTTTCTACCACTGGTAGGTAGTCAAATTAAAAAGAAAAT
ACAAGACCTAGAAAGTTTTCTTAAAAAAAAAAACAAAAAACGTCTGTTTATACCTGG
CAAAATGATTTGTTTCAGTTGCGTGTTTTCCTTGTACTTTGATCTTTTTGTACTGATAT
TTATGCTTCTGTTTTATGATCTCCTTCTAACGAAAGAACCAGACCTCTTGGCAACCAC
CTTCATCAGACCTGGGAGTTTGAAAGAAGCAGGTGCTGAACCTCAGTTTTTCCTTGA
ACAAGAAAAATGGGTTGCAATAGAAACTGTGGGCTCATCGCTGGTGCTGTCATTGGT
GCAGTCCTGGCTGTGTTTGGAGGGATTCTAATGCCAGTTGGAGACATGCTCATTGAG
AAGACAATTAAAAAGGTACAAGTTGTATCAAGAATATTTCTTTTCATCCTGATTCAG
TCTACCTTGATTTGTGTTTGACTTAGGGTTTCTGTTTTATATTTCATTATAACCAGCAA
TTTAACACAAAGGTGATTTTGAACAACTGAAACTCTACATGACAGAGAAACAATCA
AAGTATGATGTATACTCCATATGAATGTACTGTCATCTTAAGTCTAAATATTTTAATT
TCTGATAAAAACTATTATAGGTATGCACATAAGTAGAAATAATTCCCTTTAATTATG

TCCAAAGAGTATGCTGATATGCTTTTCATATGCTTTTGAATGAGAAATGAGTGTTATT
TTGTGAGTACTATTTATACTATAAATAGTACTATAAATAGTACTATAAGTGAGTACT
ATTTATAAAAGTAGTTGCACTTTGGTTACAGTTGATTAAAGTAAAATTATATATAAC
TTAATACTGTGTTTAATATTGATTTAGGAATATTTGGTAAAACTGGTAAAATTTATTT
TTGTTTGGTAAACTGTTCTATATGTGTGTAATGTTAATAATATTATTCTTATAAACAT
TTCCTACTGATGAAGGTAGATAAGGGATTTTTACAAAATACTTTCAGAAATGTTATG
CAGAACATATCCCAGTATATATATGTATGTATATGTGTGTATATATATATGTGTGTGT
GCATGTGTATATATATATATGTATACAAACAAGTTCACTGTAGATTAACTAAAAATA
AAGTGACTTAATACATAGGCTTTGTACAACAAACTAGAATCTGTCTCTCATCCTAGT
TCCTTCTAATTCAGCTTTATACGCACCAACTTTAATTGATAAAGAAAAGGAAGACTA
GGGTAAGGAGATGTAGCAAATTCAAATAACTTCAGGGTTTGGAGACAAGCCTTAAA
AAAAGCTACCAAGAACAATATATAAACAAGACAATGACTACTGTGAAGTTAAAAGT
GTGTGGCCTACTGAGAGGCATGTATGTTTATTTTTAAAATATAATGCTGGCCAAATA
AACTTATCTGAGTGCTGGCCTCAGCTGTCATCCTGGTTCAGATGCCAGTGGTCTAGA
TGTCAGTAAGCACAATGTACGGAGGAGATCAACAAGAACTGAATCATACAATGGGC
TAGACTACCAACTCATACTGGGGTTTCTGTTCCTATCAAGACCTTTATCTGTCTCATC
CATTCACCCATGTTTGACATCCTGCTCTTACTAGGAGTTTAAATAATTACAATTATTT
TTCACTAAAGCATTGCCTGACTTAATATGAAGATTTAATTTCAAATCTAGTTACCCA
GGACAAGAAGTTACTATTTTCCAAAAGCTTTTCGGTTCGTTTTGTTCAGCTAAATGCT
TAGGCTGTAAATTTAAGAATTTTTCTGACTGTACTATTATAGCTATTGTCTTAGCACA
ACAGATTAAGAAATAGGACATTTTTGGTACAGTTTTTACACTTGTATGCTATATTTGC
TTAATAACCACACAGGCATTTCTATCTTTTCTCTTGCTAACTGACAGCAAAAATTTTA
ACTTCATGAATTTAACTGGAAATATTATACATTAAATACCATCTCCTTCCTTGGAATT
CCGGTTTTCTTGATATTATTTTAATATAGCTCAATAGAGACAGACAGGAGATGGTGA
GGTATGGAGTGTCTAACGTGTTAGAGAGAACTGCTATTGTCCTGTATTCTTTCTGCTC
TAGAAACTCACTTCCCTTCTGTATCTGTGGAGTGAGAACAGGATAAATTTGACACAC
AGACTATAACTTTTTTTAATCTTAAGAGCCACAATAATCTTCCAATGTACAGCATCTT
AGACTAAGATTCTTAGACTAATAATTTTTATTCATACTTGATAATCAGTAGCAAACA
GTGATCATCTATTGTTTCTTATTATTCAACAATATTATACATACAGAGTCTCTGTGTC
ATGAGAGGCACTAACATCTATTTTAATTTTTAATATGTATCTCTAAGTTCATATGGCT
AATTTTGAAATAAGAGTTTTGAGACATCAAAAATTTGTATAGAGAGAATCAATATTT
ATCTCAACCTGATTCGCACCACCGGGAGCCACTAGAACTCAAAAGCAATCTTGAGA
AAGAAGAATGGAACTGGAGCCATGACATATAGATCAATGGAACAAAATAGAAAAG
TCAGCAAGAATATACAATGACAATCTCTTTAACAAGTGGTGAGTACAAAAGTACCT
AAGCAATGTAACTGTGAAGGCCTACTGAGAGGCATGTATGTTTATTTTTAAAATATA
ATGCTGGCCAAATAAACTTATCTGAGTGCTGGCCTCAGCTGTCATCCTGGTTCAGAT
GCCAGTGGTCTAGATGTCAGTAAGCACAATGTACGGAGGAGATCAACAAGAACTGA
ATCATACAATGGGCTAGACTACCAACTCATACTGGGGTTTCTGTTCCTGTCAAGACC
TTTATCTGTCTCATCCATTCACCCATGTTTGACATCCTGCTCTTACTAGGAGTTTAAA
TAATTACAATTATTTTCACTAAAAGCATTGCCTGACTTAATATGAAGATTTAATTTCA
AATCTAGTTACCCAGGACAAGAAGTTACTATTTTCCAAAGCTTTTCAGTTCGTTTTTG
TTCAGCTAAATGCTTAGGCTGTAAATTTAAGAATTTTTCTGACTGTACTATTATAGCT
ATTGTCTTAGCACAACAGATTAAGAAATAGGACATTTTGGTACAGTTTTTACACTTG

TATGCTATATTTGCTTAATAACCACACAGGCATTTCTATCTTCTCTTGCTAACTGACA
GCAAAAATTTTAACTTCATGAATTTAACTGGAAATATTATACATTAAATACCATCTC
CTTCCTTGGAATTCCCGGTTTTCTTGATATTATTTTAATATAGCTCAATAGAGACAGA
CAGGAGATGGTGAGGTATGGAGTGTCTAACGTGTTAGAGAACTGCTATTGTCCTGTA
TTCTTTCTGCTCTGAAACTCACTTCCCTTCTGTATCTGTGGAGTGAGAACAGGATAAA
TTTGACACACAGACTATCTTGAGAAAATACAATAATCTTCCAATGTATCTTAGACTA
AGATTATACTAATAATTTTTATTCATACTTGATAATCAGTAGCAAACAGTGATCATCT
ATTGTTTCTTATTATTCAACAATATTATACATACAGAGTCTCTGTGTCATGAGAGGCA
CTAACATCTATTTTAATTTTTAATATGTATCTCTAAGTTCATATGGCTAATTTTGAGG
AAATAAGAGTTTTGAGACATCAAAATTTGTATAGAGGCTAAGAGATAATCAATATTT
ATCTCAAGAAATATAAATTTAATTGGGAAAAGCATATTTAAAAAGCTAGAGACTTGT
GAAACTCTGTAATTGAATTCTAAAGCATACACTATTTAGAAAGAGAACTTTCATAGA
GTATTCAATAACTTCAGGAAGAAGGTGAGACTTGAGTTGGATTTTGAAGTATAAAAT
TTTGATCGCCCAAAGAGAGTTCCAATAAAAGTGTCAAGGGACTGCTTAATGCCATAG
AAAAGAGTTGTGGAAATGCAACTAAATATTGTGACGATAGAAGGAAAAATATTTTT
TTAATGTGTCAAAACAAAGAAATAACAGCAAAGATTATTGGAAAGATAATAACATG
GGGTAAAATTTAGGAAACTTTTGAGCATCAGGCAGAAGACTATTTAAGTAGTGACA
TGACAGGCAATAGTAGTGTATATTTACCTGTAGTTGTTAGATATAACAAAATCTGCA
TTTAGAGACTGGTATCAAATATACTGAAAAAAGAAATGGCAATACTATATACTAGT
AAGTTATATGCCACACATACTAATATATTATACTATATACTAAATAAATAAAGCATT
TTTTAGTCCTACTAAGAGCTTCATATATACTAATTCATTTAATCTTCACAAATATCAA
TGAGAAGATACTACCATCACAAGACCTTAAATCAACCTGACAACTTACCCAGCCAA
AAGGAATATGAGTTAGTTTCTAAACCTAGGCAATCTTAAGGCAGAGATGAGATGAT
TTATACATTTTGCACTCTCTAATTCTACAATAGAAAGTGTTGGAAAGTGAGGAGACC
AAAAACAGGAAATGTTATGTAGGACATGACCACTTTCAAGGAGAATTTAACACTCA
CTTTTAAACAGATTTTTGCACAGTTTGCATATAACAAAAGGTTCTCTGGTCTAGTGTG
ATGGAGTATGTAGGACCGATGGGTGATGGAATGCAATGGACAGAGGAACTAAATAA
ATAGGGGGAGAGAGGAGTAAAAGTTTCAAACTACAAGATGTCCAGTTGTGTTCTTG
ATAGAAATATGCATACCTTAGAAGAATAAAGCACAGAGGTTTTATAAGTGATTTGA
AAAGAACATCCGTGTATATTTCAAAGGGAAGGTTCAGAGATTTGTTCATTAGTATAG
AAAATACAGATAAAACTAAACAATAGATATGTTTAAGGAGAGTTTGAATATAGAGA
CGAAAGAGCAGAGAAATTACTATGGGATTTATGCATTCAGAGGTAAGTAGGAAGAT
AACAGAAAAAATATTTTATATGAAGCAATGAAAACAATAGACTGTAATTTTTTTTAG
AAGTTATTATGAAGTCAGTTTTTAAGGAAAAAGTAAAATTATCTCAGGCAGATGAA
AATTAAGGAAATAGTAGTTAGAATACAGTCACTAATAACTTTAAAATTATATTTTCA
GTAGTGCCAAGTAATAAAGTGGGATAAAATAAAGAAATAGAGTGTTTTCCCTTCTTA
TTTTTGAAATGAGGGTTACCTGCAATCTTTGGGTCATACTGTAACCCAGCTCAATATT
CACAGACTAAGACATCATCTCTAAGACATCACTTCTCAGCCTTTTGGCTAAGATTAC
GTGGCTTAGATGGTAAAGCATCTGCCTGCAATGCAGGAGACCTGGGTTCAATCCCTG
GGTCAGGAAGATCCCCTGGAGAAGGAAATGGCAACCCACTCCAGTACTCTTGCCTG
GAAAATCCCATGGTCAGAGGAACTTGATAAGTTACAGTCCATGGGTCACAAAGAGT
TGGACACAACTAAACAACTTCACTTTCACTTTTCAAGACATCATCAGAAGCCCTTCA
ATGGTCAGGAGCTGTGTTGTATAGTTAAGACAGCTTGCTTTTGCGCTCAGATTTTCAT

ATAGCGGTGTTGACCTTGCTTGTTTTAATTATGTAAAGCTGTTGTGACTTTTTCTTCA
TTCAAATGAAACTGTTTCTTTTAAATTTAGTTTACAAAATTATTTTAAATATAAGATT
AGCATATGTATCTGTTTGGCATACGTGTAGATGTCAAATCTTTGAACTATGTGTAAG
CACAGAGAGCTAAGTGTCTCGCCCCTGCCTCAAGGGCACTTAGGTTAATACTGCTAT
TTTACAAACATATATATATATATATATATATATATGTTGTACTACCAGACAGTTTTTC
TCTACAATTAAAACTCATTGCATGTAACTTGCATTATTTAATGGACTAACAGCAAAT
TGGCAAAGTTATTACCTATATATAAGCACCATTAAAAAAATAAAGTGCTTAATTTAA
AAAGATAAGAAAACCATCTCATCATGTTCTCTAGAGTTGGGACTCTGATATTTTCTG
GTTCTCAAAGATGACACCTGATACCAATATCTGAAATGGCGTAAAACAGTCAATAC
ATTTTACCATTATATTTCTTTATTCACTATTTCCAAAGGAAATTTTGTAGGGTTTTTAT
GCATTATAGATAATTATGCATTATGTAGGGGTTTATGCATTATAGCTGAATGCTTTTA
TACCAGTGTTTCAGATCATGATTCCTAGTAGAATTGAGTGGGACCTGCAGATTTGTG
CAATTTTGCTAGATAGACTAAAAGTCAATGGCATGAGCTGCCATGAATGTGGAAAG
CAGAAATTATTAAAGGACTTCAGAAAAAGGTCAAAGTTCTGGCACCACTCACAAAG
GATAATTTATCACCTGACACTATAAAATATCACTTGATTAATATTGCTGAATATTATA
TAATAAGCAATTTTAAATAATTTGAATTCACCTATGATATTAACCATGGAACATCAA
GACAGAAAATCAAGATATCTTTTTCAATTAAAAGACTAATGTTCAATCATACCATCA
TTCAATTACACCATTTCCTTTCCCTATCCTTGAAAAGAATGATCTTCTTGTAGCCAAA
AAATGCTTTCAGATAAAATAAACATAAATGATGATTATTTTGACTTATTAAAATGAC
TATAATTTCAATATTTGATAGTAAATTTTATACACATATATATTAATTTCATGGTATA
TGCTTAGACTATTTCTAGATATGACAAAATAATTTTTACCTTATTATAGTATCTAAAA
GCTCAAAAAAGTGCCAGAGGTATATAGGTTATTAAAGATAGTAGAACTTGCTTTAAT
TTAATAATAATTATGAGAACATGAAATAATCTAAAATAAACTCTAAATTTAAATAGT
AGTAAAACATGGATTAAACATGTATTTGCATATTAACTCCAATAATAAAATCACTTT
AAAATGTTTTAAGAAACAGCAGGTCAGCGTAATTTTCTTCCTCCAGCCCAATTTAAT
ATCAACATAGTATACTTTTCATGCCAAAGAAAAACTCTCCCATTTTAGGTTCTTATGT
AAACACTTGGAGGCTGATTTCCTGAGTTTCAGAAACATATTACATCTAATCAATTTC
ACCACCTGTTATTCTGATGTTTAAGTGATGACTTGTTAATAACCTGAGACAAAAATC
ACTTTTTGACTGTTAGCACAACTGGCACTGATATTCTAATCCTCTCTTTCTCTTTCTCT
CAGCATGTATACACATAGGAGAAAATGTTTTGATCATAGGAAGACTGCTTGTAACTG
GTTCTAAACAGAACAAATTGTGGTAGTGAGTGAAGAAGAAAAAGACTCTTTCAGTA
TGTTTCTGCTGTTTAGGAAAGTTTTGATTTCACTGGATGGAGAATAAATATAGAAAT
GATTAAACAGCAGAAGTGTCACAGGATTTTACATGAGCTATTGCCACATGACTGGTT
CAGACAGGAATGCTATGAATGAAATAATTAATATAATTATACCATATTCCACCTATA
TTGACTTTCAGAAATATGAATTTCATATCAGTCGACCTCATATTATTTCCAATATTAT
TATTATCCCTGTTTTACAAATAAGGAAGCTGAGGTACAGGAAATCAAATAGCCTGCC
CAAGTTTAAATGGTTAGCATATGATCAAACTGGGAATCAAACTCATAGCATCTATTT
CATTGTTAACCACTTCACTCTAGGCTTCCCAGGAGGTTCAAACAGTAAAGAATCTGC
CTGTAATACATGAGACCCAGGATCAGGAAGACCCCCTGATCAGACCCTGATCAGGA
TCCTGACCCCTAGATCAGGAAGATCCCCTGGAGAAGGGAATGGCAACCCACTCTTA
TATTCTTCCCTGGAGAATTCCATAGACAAAAGATCCTGATGGGCTACAATCCATCCA
GTCACAAAGAGTCAGACATGACTGAGCGATTAACACTTTCACTTTTCACTCTAGGCT
TTGAGTAAAGAAGCCAATATACTTGGGTTGGTAGTAGGGTAAGGAAATCTACTCAT

AGATTAAAATCTGCTTTATATGATTCCATCTTTCAGTACTCTGTAAGAACCTTGAGAA
ATGGGACAATATTATTTTCTGCGAATGTATTCTTTATAGTTCATTCACAGGAACATAT
TTAGACTAAAGTTGCCCATCATGTGGATGAATATTGCTTTCCCATAACAGCAAAAAT
CAAGTGACCACAGGACTAGATAGAGGGTATTACAGTCCCAGATATCTGCCTTCAAAT
TAATGTAGCAAAACAGAACTTTCAAAGTAAATGGCTTTCAACTCAGCTGAAGACCAT
GCCACACTAGAGTCATCAAGCTGAAATCCAGGGCTAAATAGTCTGAATGCTTAAAG
TCAGGGATAGATACTGATGTATGTATCAGCCAAGGGAGAGATCTTCAATTATATTAC
TTTTTTTTTTGTACATATAATTTCATCTTTGTATTTCAAATTTACTGAAATGTCCACAA
AATAGGTCAGACAGTCCAAAGTCAATTTAAAATTCATGGCCTTTTAAAGTCACAAAG
AACTTAAGCTCCTTTTTAAGAGGACCTTATGATATATTTAGTCTTGATAGTCAAAGA
GGTCTATACAAATTTCTAGTACCTAGGTAGTCTTAGGATCAGAAGAAATTCATTCCT
ATTCTGACTGATTCAATATTTATCTCTGTCTCTGTACAAATTGTGGTTTTATAAAAAC
CAAAGAAGATTGGTGTGAAATGCCGGATTCACCATTATCTACTACCAAATTATTGTG
CCACATTGAATCAGACATCTGTTTTTCTAGGACCCACTTCTACTACTAAAAAAAAAA
AAAAAAATGGATGGTGTTAGATGACCCTAACTCTAAAGCTAGAAGTTCAGTGCACT
CATTTCAATTCTTTGTGTTTCAACTTCCTGATTTTAAAAAAGTTATCTCCAGATTGAC
AAGTAAAACAAAGTAACTCATGGTCCTTAGAAGAAAGACACAATATAGTTACAATG
TTTTTTAATATAAAATGCACAAGTGTTAGACTCAGGGAGATTTCCGTCCCACCAAGT
ACATTATTGAAATCTGTGTCTGGAGAGGTAATGATAAACAATTGATATTATACTAGT
CCTTAAGAGTCTACCGTGTAAAAGTAATAATGTTCATGTGTATATGTTCATGTGTGTT
CTCCTCTCATGTCTCTTGAAATGGGTAAATGAAAATAGTTTGCAGCTTCCGCGTAGA
CTTCAGCTTCAAAACCCTCATCATTTACAGTAAACACCTTTACATTTAGAATAGCCAT
AATACTTATCTATACATAATTTTGTCTTGATTTGGGTTTGCTATGGAATTAAGACTTT
TAGGAGTAATAAAACAATCTTCAAGGACAATTGCTTGTTAAAAGTACTAACGTCTCA
CACTGCGTTAACAGTTGCGATGTACCCAGTGTAAAAGGATTGTGACAGCACAGGATT
TTGTAATGGCATTTAGCTGATGCCCTGTTCTCCTCTAAGGAATTCTTTTTCTTTCCATT
AAGCAACAAGTCAAGTATCCTGAAGACTGCTGATTCACTTTGACTTCTACCAACTAA
GTGCAGAGGTGACAGGAACAAAAATAGTAACACGGTGTGAAAGAGGACTAGCATA
AGGGTCAGAGATTAGGATCTTCATTGTGATTCTGCCACTAGGCATTTGGCTCAGTGA
CCCTTGGGCCAGATACCTGGTTTCTCAGAGTGTCAGCATGCTCTCCTAGTAAATGTTC
ACTGTCCTTTTCTGATCCATCATTCTATGAATTGATGCTTCCTGTTGAACTAAACAAA
CAGGTGAGATTGTTCTTGTGGGTTTTTTCAGGCTTATTTTCTGTGAAATGCTTTCCTTC
TCTTCCTCTAGCAGTAAAAGGGTAAACTGGAGCTTTCCACCTGAGTATGTATTCCTTC
CAAGGTAGTTAATTGGAAACATTGCCTGCCACAAAGTCACCTTTGTCTCCATTACCT
CCATAGTCTTCTTGTTCAGAGAGTAAAAGCCTTGGAAGGAACCTGGGAGAGCATCTA
TGATAACTCCCTCACTTTACAGATAATAAAATTGACCTCAAAGGGTTTGGTGACTCT
CTATTCTGTCTCAGCTCCTAAACTATTAATAGATACTTATGTATCCATACTAATAATA
CCTAAACTAGTAGATACTTCTAATGTTGGATTTTCTGATTTCACAAAAAGAAAAAAA
AAAACTTCTGTATTTCTTCTTTTATTTTTAAAGTATTAACCTAATAATAATGAATTAG
ACTACTGTAATCTTGGAAACAGGTACTAATAGGTTTCTGCAAATGTACTGAGAGGAA
AGCCTCATTGATCAGGGTTTTGAATGGGAGAAATCAAAGCTGTGTTTTTCTTAACAA
ATGTTAACCGATTTTCACCTGAAGTATCCAAATTCTGAAGAAAATGAGAAAACTTCT
AATTCCACTAGATTTTTCTATTTTTCCGTTTTGCCTCTTCAGTCTGACTCATTGAGGGT

ACATTAGAAATTAATATTCAACCTGATAAGAACTTTTTGAAATGCTTTAATTAATCTT
GCTTAATAAATTGTTACAAATTGTTGCAATATTGAAACAGGGTCACTCTAAAGCTTG
CCAATGGTTGGCCTTACAATCACCAGACATGTTTGCAATATTATACACTGATTTTCCT
TGTAAAAGGCTGACTGAAAGAGATTGCTACCATAATGTGTTGAAAACATTTCTGTTG
CAAGCATGTGGCAGGTTGCAAAGGTTTTCATGAGCAGGATACTTGAGCTTGATCTTT
CACTGACTAACGAAAAAGTTTCAACTCCCAACAGCTGCCCGTATTGGCTACAAAAA
ATAACCCAAACATTTTCTTTTCATAGGAAGTTGTCCTTGAAGATGGCACAATTGCTTT
TAAAAATTGGGTTAAAACAGGCACAGATGTTTACAGACAGTTTTGGATATTTGATGT
GCAGAATCCAGACGAAGTGGCAGTTAATAGCAGCAAAATTAAAGTTAAGCAAAGAG
GTCCTTACACTTACAGGTGAGTCCCCAAAATACGTGGCACTCTTTCCTTGAACACAG
GTATTTCTGAAAAGCTTCCACTTGACAAATGTCACTGTATTGAAATGTACTTATTATT
TTCTTACCATAAATATATATGGAAATATTTTGTTTCTGCCTACATAAAATCCTAATCT
AAGGATTTAATTATTATTAAAGAACAGGTTTATTATGAATTTGTCAGCATCAGTTAA
CTACCCTACATTTAACAAGTCACATGTGTTTTACACTTGTGGCATATATGTTTCAAAA
GCTCTGAAATACGCATCTAAATAGAAAAAATATATCAAAATTTTTAAAAATGAGTAT
TTTAAAATCTTAAAGTTGAAAAGTGTAGGTACATGAGTAGTTCTCAAAGTATGGCTC
CTGGACCAGCAACATCAGCATCACCTGGGAACTAAGGCAGAAATGCAAATTCTCAG
GCCCCACCCCTAGACCAAGTGAATCAGAGACCCTTGGTTTGAGCCTGGTAATGAGTT
TTATAGCTCAGCTGGTAGACTCCGCCTGCAATGCACAAGACCTGGGTTCGATCCCTG
GGTTGGGAAGATCCCCTGGAGAAGGGAAAGGCTACCCACTCCAGTATTCTGGCCTG
GAGAATTCCATGGACTGTATAGTCTACAGGGTCGCAGAGAGTCGGACACGACTGAG
CGACTTTCACTCACTCTAATGAGTTTTACAAATACTCTGGGTGATTGCAATACATAGT
CAGTTTTGCTAACCACAGCATTAACTTCTATGTAAGTAAAATACCTGCTTAGGATTTC
TATTCAAACTGTTTTCCAGGTCTTAAGAGTGTATTTATGCAGTAGCTTCATTCTTATA
TTTTCTCCAGTACAAAGACTGAATAAAAAATACCTTTATCTACATGCCGTAACAGTT
TAAAACATTTTGTATGGAATCTAAGATCCATCAGATGTAGGGAGAAATGATTAGCCA
GAACTTTTCATTTTGTAAATTTAAAAATCAGACTGACACCAGGTGAAACAGTTTGTC
CACGATGACAAAATCCTGGAAACTGATAACTCCTAGTTATTGGTGTACATTTTCCCC
CTGTCAAATTTATAGATTGAAGTGCTAAAAACACTAACCTTAGCTAGGTATTAGGAC
ATTTATAGACCCCATATGGGAACAGAATTAAGTCCTGTTGTATCATTTGTTATATAA
ATATGATTTTTCAAGGATAATTACAACTAGTTATAGACAATAATCAAGTATTATCAC
AAAACTTCATGTTTGTAAAGATTTCTAATTCATAGTTTTACTTTCGTACTTGTTTTGTG
TAGTCTATAATCATGTAATTTCTTTCCTCTCCATGCATTAATTTATTTCTATGTCAGCA
ACTCATGCTCAAAGATAATGAAGTAACAGTAGCTGGGAGGGTGATGCCTGCTGCTG
TAACATAATATATAAGCTGTAGAACACCATGGTACTATTCCATCCTAGTAATTATCC
CCATTTCCTGTCTTCAGACATATGTCTATGACTATTCACAACTCGGTCATAGAAGTCT
TGGTTTTCATTGTTACAGAAAATAATCTTACTTTCAGAAATATTAAAAAAAATTTTTT
TTCAGAGTCTTATACTTCTTATATCACTATCAAAAGCCAGATATAATAAGATATCTG
AATATCTTATTCCAAAAAACGTGTACTCTTAAAATCTAGAACTCACGTATGACCTAA
TATAATCTAAGTATAACTGACTAAACTTCTGTCATAGCTCAATTGTTAAAGAATCCG
CCTGCAGTGCCAGAGTCCGCCTGCAGTGCAGGAGACCTGGGTTCGATTCCTGAGTTG
GGAAGATCCCCTAATGGCAACCCAATCTAGTATTCTTGCCTGGGAAACCCCATGGAC
AGAGGAGCCTGGTGGGCTACAGTCCATGAGGTCTCAAGAGCCAGACATGACTTCGT

GACTAAGACACCAAACCATAACTGATTAAAAATCATACCCTGAGAGAACTGAATGC
ATGTGATAAGAGAAAAGAGGTGGTATTTAAGATCTGTCTCATCACAGGTTTTGCTTA
ATTTTTTTGCTTGCTTGCTTGGGGGGGTGGGTATGTTTTTACTTTTAAGGCTGTTCATT
GTGGAATGTAAAAAACAAACAATATAGAATCCTGTCCCAGTCCTGCTCTGATAGGTG
ATAAGATATATATCTTATTCTTGGAGGCTAAAACCTTCATTCTCTAAGGGATAAAAA
TGGAGTGGATTAGATATAATTTGTTTACTTGGAGAAGTAATGAGGTGAGGGTTGGAG
AAGTTGGATTTTCAGCTAAAGTATGTAAACAAACTCGAATTTATATAACAATTCTAA
AGGAATCTCATGATGTTTTATGATGTTGACAAAGGAATCCTTACTGTCTACCTAGAT
ATCTTAAATAATGGGAAAAACAATGGTTACCTTTCAGAAAAAGTTAATAGTTTGAGA
GTCTTCCATGTGTTGAGGTGAATTTACTTCCTATCAATTACACCCTGAATTCAGTTGA
AATTGGAAGTGGAAGAGGAGGTCCCTCCTCAACAGAACCACACAGGTTTATTTGCCT
TTTAGCTGCTGAAGTGAGCTTGATGAAACTTTGACAGGGTGTGACATGAGTCAAAAC
ATGAAACTTCCTGGATTGAGATGTTAATTATGTATTCCATCTCTATGAAGACCTGGT
AACTCCTTCAGAATATCCTCAGATCGAACATGAAAGTCAAGTCGCTCAGTCGTGTCC
GACTCTCTGCAACCCCGTGGACTGTAGCCCACCAGGCTCCTCCGTGGGATTCTCCAG
GCAAGAATACTGGAGTGGGTTGCCATTTCCTTCTCCAGGGGAATCTTCCCAACCCAG
GGATCGAACCCAGGTCTCCCTCATTGCAGGCAGACGCTTTAACCTCTGAGCTACCAA
ATTTTTTAATTACTTCGCTTTGGCAAGAGTACATGGAAAACAAAATATGAAGCAATT
ACCTCCCTTTGACCTTATTAACCAATACTCTGTAAAACATGTATACATTCTAATTTTC
AAACAAAAACTTGAGTACTTATAATCTTTTTAAAAGTATATTCTAAACACAGACCTT
AAAAGTATATTCTAAACACAGACCTCAGTTTGAAGGATTTTTTTTCTGTTAGTGAGT
GCCATCTGCTGGTATAATGTAAGTACAACTAAGCAAACTACAATTTGAGTCACAATT
ATGGCTCAGCTGGTAAAGAAGCCACCTGCAATGCGCGAGAGTCAGACACCAATGAG
TGACTGTCAGTTACTTTCACTTATTTATATTGCTATTATTTCAAGTATATAAATATAA
ATGTTCCTGAAAATTAAAGTATTTTAATTTAAATATGAATATGCAATATAATTTTAAC
ATATAATTATCTGATATAAAAGAGTAACTGACTAGTAATAGATTCTCCAGGATGATG
TCTCAGATATTTATATTCTTTGTATGTCTACAGCATTCCAAGGAGAATAAACAATAA
AAAGCTTAATGAAATTGTATTAGAAATAATATATATTTCCATATTTTTGTGAATTCTC
TTTTTAAAGAATGTAAAAAATAACTTCTTCATTCAAAACTATTTACCAAGAACTTAC
CCATGTACCAGACACTGCTTGGGGATTCAAAATACTCATAATACCTATGGCATAGGA
ACCTACATTTTTATTATACTTGATTGAAATCAGGTCAGGTTTTTCTGGTTGGTTTTGTT
TTTTCCATTTCAGTCCCATGTTGTTTCAATCTTTATCTTAGATAACAACACATAACTA
TTAGAAGCTCATATGATAGAGACATCCGGATATATGATACAGCAGTGAATAATGTA
CACCTTTTTCCATCTCAGTAAATATCTTGTATGTATAAAATATTTTTTTCCCATGATAT
TACCCTTCACTACATTCCTAAAAATTAGTTGGTACTCTGCCTACAGATTAGAGCCAG
GAGCTCAGAACTAGTAACTATTCCATACATGGGGTAGGGTTGGAGAGGAATTGATC
TGTTATGTGGAAATATATTGTTAGTTATCTTTAACTTTCATTAAAATAATGAAAATTA
TAGCCAAGCCAGTGATGGAAAGGCTATCTACTTTGAACAGTCCTTCTAATATAAGAA
AATGGGCTCATAATATTCCCCTGCTTCAGCTTCTGTATTTCCCCCTCACCCTCAAGAT
GAAAGGTGAACATCTGAATGTAGAATGCAACCCTTTAGTCTTTGAAATCTTGCTCAT
TTCAGTGGCTTTGTTTTCTAGCACTCCCCTTACTCAGACACTGTAAACCAACCACACT
GAAACGTGTTATTTCTAAGAGTATACCATGATCTTACTTATCCTATATGTTCTAACAT
ATTTTACCAAATTAAATTTTTATCTGCCACTCAAACCACTTATATGAAATTGTATCCT

GTCCATTATCCCCACCATAACCATTCTAAGCCTGAGTTTGTACCTATTCTGTCTTCTC
ACAGAATCTGTATAACCTCCTTAATAACATGTATTCCTTCTCCTCTGGTATGTACATC
TAAGACCTTCCTTCTCTAATTTGCTACAGACTCAGCATCAGCTTGCTTATCCTTTTGG
TCTACTTGTTTAGCATAATATCTGACACAGTCTATGTACTTAAAATTATTTGTTGCAT
GAATGAATGACATTCATTTGAAACAACGGTTTTGAGTTTTAGTTCCTCCTGTTTTTCA
GAGTTCGTTATCTAGCCAAGGAAAATATAACCCAGGACCCTGAGACCCACACGGTC
TCTTTCCTGCAGCCCAATGGCGCCATCTTTGAACCCTCGCTATCAGTTGGAACTGAG
GATGACATGTTCACCATTCTCAACCTGGCTGTAGCAGTGAGTAGACAAACCGCAAA
ATTACTGGTTTTGAAATCTTCTAAAATCCAACTGTAAATAACCTCACAATTAAAACT
ACAATTAGATATCTTTTTTTTCATTTTTATAAAAATGCTTTCTTACGCTATTATTTTGA
ATTACGATCATTTGGAAAGATGACAAGATCATAGAATGATCAACTGAAGACTGATT
AGAAGACCATTTAGAGTGTATCATTTTAAAAAGGATTTATGGGGCTTCCCTCGTGGC
TCAGTGATAAAGAATCCGCCTGATAAAGAATCCACCTGATAATGCAGGAGACATGA
GTTCAATCCCTGGTCCAGGAAGATCCCACATGCCTCAGAGCAGCTAAGCCTGCACAC
CACAACTATTGAGCATGTATGTGTTCTAGAGCTGCGGAGCTGCCACTACTGAGCCCA
GATGCCTCAGCTACTGAAGTCTGCACACCCCAGAGCCCATGCTCTGCACCAAGAGA
AGCCACCACCACGAGAAGCCCACGCACCACAATCAGAGACTGGCCCTGCTCAAGGC
AACTAGAGAGAAGTATGCCCAGCAGTGAAGACTCAGCACAGCCAAAATAAATCACT
CATGAAAAATATAAGCAAAATTTGTCAAAAAGTAGGACTCAACTTGAAACTCTTCTT
CCTGTGGCCCACACACCACAGAGAAATGTAATCATTAAGAGATGAAATAATATACT
TTTAGTCCTGGCTAGATTCGGCAGTCGTTTTAAAAATAGCTGGAAATTGTTTTATTTG
GTTTAATTCTTGTATTTACATTTATTTGTTGTTATATTGCACTTGGTGTCTTAAAGCAT
GATTTTTTTTTTTGCAGGCTGTACCACATCTCTATCCAAATTCATTTATTCAAGGAAT
ACTCAATTCACTTATCAAAAAGTCCAAATCTTCCATGTTTCAAAACAGAACTTTGAA
AGAACTATTGTGGGGCTATACGGATCCATTCTTGAATTTGGTTCCATATCCTGTTACC
ACTACAGTTGGTGTGTTTTATCCTGTGAGTAACAATTATAAATCTTGATACTGTTAGA
CTTTAACTGGATATATATAATCAATCACACTGGCAATTCATAAGTTTATCATTCATTG
TGTTAAACCCTATTTTGGATCTCGGAATACATACCCTAAGTTTTTAAAAATCTCTACC
TCACATGGATTTAGCTTTCCTAGTTGGGAAAATTTACTTATCTGAATTTAACAATTAA
GTTTAAGATTAAGAAGTGGCCATTTAATCAAGTGTCCACTCCTAGATAAAGATGCAA
TTGTCTAAAATTAGGTTATAAGGTGCTAAGAGTTTGAATACTGTTTTTGGTTTTGTTT
CCTGTCTGAAAAACAAGTTTAAGCTGTATTTTATTTAGCTGTATTTTATTTTTGTTAA
TTGTATTCATCATATCATTCTTAAAAAATTGTTTCAGTGTAAATTTAAAAGTCACCTT
ACATTGTACATAAGATTGATACATCAGCATGTTGTATTCTCTCAGATTTAGTTGGTAA
GAACTTCTGTGAGTGAGTGCAGTGCTCAGAGCTGATAAGAACTTCTATCTACAAAGT
CATGAAATGGCAGTTTGGAGATAAAAAGTGAATGTGGTGATGTACTTAGTTATGGC
AGAATGTTCTGAATTACAAATAGGACAAGGTGACTATTGCATGGAAAATTTTGCTGT
GATTCCATTTGGCACTATATGAGATATTAATTTTATTTTGCAGTTCTTTGTTATTTTAC
TTTAGGTGGTTTTTAGGGTTGCAAGTTCATATATCAATCAAATATGAATACAGAAAG
CCTTAAGAGAGATAAATTCAGTTGCTATTATTTCATTCACATATTCTTATTTCTTTCCT
TGGCTAATTCTGTTTTTAATTTTGTACACTTCATCTTAGTAACTACTTAGAGATCATA
ACATTGAGACAATGTCTAGAAATTGGTCATTTTTCTCATAGGTTTTGTTCACTCTCCC
CTCACTTCAATATACCAGGAGTAGTGATCAAAATGGCCTTTCTGGATTGAATAAAAC

AAAGAGTGCTAGAATTCGATCATGACAGCTTTGGCATTTGTTACCTTACTTTGTCTTG
GCATCTAGCACTCATTTTTAGTAACCTTTTGTATTCTGTCTTTTTAAAATATTAAAGA
CCAATAATTTTGAAATGATTGCATGTATATTTAAAATCATATAAAATAATTATGAAC
AACCACTGAAAATGTGAGATAATTACATATTTAATAACTTGGAAATTATGGTTTGTT
GTAAAAGTAATTTTTTCTTGCATCTCTTCAGTACAATAATACTGCGGATGGAATTTAC
AAAGTTTTCAATGGAAAGGACGACATAAGCAAAGTCGCCATAATTGACACATACAA
AGGCAGAAAGTAAGTATCCAGGCAAAGTGTGTGTCACTAGGATACTCTAAGGCAGG
CAAGAAACTTATTTACCGGCGGGTAAGGCACAGGCACAACCTGTGGGACGGAGCTG
TTTATATCTCAGCCCTAAAAACTCCCTAGGTCTACCAAGCATTAATCCTTCAGTTGTG
ATGACTGGTCACACTAGTGCATTTTTGCTGAGTATCAGCCCCACTGGAGGGTTCACC
TTTATGAAAAAGTTCTCTTACATAAAGAAATCTAAAGGGACTACTTCTATCTCTGCT
GTATAACTGGTATTATCATACACACAACAATGACATAAATGCAAATGAAAATGAGG
AAAATCAATATATCAACTCATCATCTAGCACGTAGAAATACAGACATCTTACTGTTT
CTTTTTTAATTGAGCATGTCAAAAAACATCATTCATTCCAGGGAGATGATTAAATGC
TGGGGTCCACCACCCCCACCCCCCCGCAGGATCCAGGGGAACCTGAAGGAAAAATG
GCGTCAGTGATTGATTTAGAGAGAGATAAGGAAAGAATGTTGAAGATAAGAAAATA
GAGGAGAGAAAGAGGCTGATATTCCTAGGTTTACATAGAAAGCCAATAAAACTCCC
AGACAAGAAGTTTGTCCTGTTCACTGAGGCTACAGGTGCCCTCCTGGTCTCCTGAGG
GAGTGAAGACGCAGAACGTCTTCCCGTTCAGGTCTTAGAAACCCGAGCAGATAAAT
GAATGCAGGGAGCCTCTATGCTCCAAGGGATCAGCCTGAAAAAGAGAGGGAGGGA
GAGGGAGAGAATGATTGACGCGGGGAGACCAAGCTGCTTCAGTGAGCGAGGCCCA
ATAGCTTTATTTTTAAAAGGTACTTTTATACCTTGTCTTATACATAGAGGGAAATGAA
AGATGCAAAGTCATACAGAGTCAGCCCAAACATTACATCTGTTTTGTCTTTATCTAA
ACCAGGATTTTTTCTGCAAACCTTTCCCATAAACAATATTGTGTACATTATCTTCTGG
CCTTGGAGGCCTGTGGACATTTTGTGACCCTCTTTTGATAAAGGCTGCTCAACCAGA
AAACTTATTTTCCCTTGAAATGTTTTTTCTTTATATTTCTAATCTATGTCAGCCTCAGA
AAGTATTAAACAAGTTACATTTCTCATGGAGCAAAGTGCAGTGAGTTACAAGAAAG
AACAAATTAGCTCAAATGTCTGATGTGGTTAATTTCAAGGCTACACTTGTTTTTCTTA
CTTTCCAACTATGTTAACTAATGCACCCCCAGGTGCACAATGGATAAGAGATATGGG
AACTTAGCAACAAGTATTGGCCCAATAATGAAATCCTACACCAGCACTACTCTAGTA
ACTTTTAACTCTTTAAAAGGCTCTATGTTTTAGGCTTTCTGTGCCTCTCACCGTTGGG
AGGCTGTAAATAATCATATGTATAGCTGCAAGAGTCTGGATATACCTGTCAAGCAAG
CTAGAATGCTAACAGAGGGGGTTTGATTTGAAATATTCCTATCATGTCCAAGAGACT
TATTAGCTATAGCCCTAAGTTGATTTTCTCTAGAGAAAGGTGGTCAGGGATAGCCCC
CTGTTAATGTCAGAGGAGTTGGTGAAAGTCATGAAATATTAAAACAGACAGATTGT
AGTTTTGGGGTAGATGCTCGAGAAAGCTTAGGGAGCCTGTTGAGTCCTGAAGCCTTG
CTTAACAGTTCTCTTCCACATGACCTTGTCATGGGTGGGATCTCCCATGGGTGGCTCC
CGGCAATTAAATGAACCTATAAAGATGAATGTTCAATGAATCCATGTGAACATTTTC
ATGTCTTGTCCCTTAAAAATTTTCCTGAAATAATTTTCCATATTAATAATTCAGCTTT
TTAAACCATTATCAATGCAAAAATGGAATTATTATTTCCTGTTCCAAAAATGACATA
TTAAGCCTGAAAATAAAGTGTACACCTTCTATGAAGTAGCAGTAGAGTGAGATATGT
TCACATACCCATGATTTCCTACTTCTCCGAAGTTCCTGCCTGTTTATGTGCAGTACAC
CTAATCTGTCATATATCTAAAATACAAAGAAGCCCAATAAATATCAGGTGGGGGA

AACTTTTCTCCTCAAACTTGAAATTAGAGTTTTAAGATCAGTAGTTCCTGACAAAGA
ATAAAGTGATCAAAATCAAAAGAGATGAAATATTTTTATGATTAAGTAACCATTACT
TCACACTGAGTTTGTCCCTAAAAATTGCCTAAAACAGTAAAATACTCGCTCACGTAG
ACTTTTCATCTACAGAGTTCATAGTCCTTTATTGTTTTGAAAATTCTAATGGAGGAAA
ACAAACACTTCTAAATGTTCATTGTAGGGAAATCATATCTCTGTATATTGAGAAAAG
TGATAATTTCTTCCATTTCAATATTACCAGAGTTTTTGAGGAGTTACTAAATGTTTCA
AATTGGAGTAAGAATTTCATTTAAGATTATGATAAGGTTAACTTAACTGTCTGAGCA
TGACTGACAGCTTGTTCTGTTTAAAAAAGAAGTAATAGCTTGCCTTGACATCTTACT
GGATGGTATGTTAGACTAACAAACCCATTTTTCCATCACCAAAAAGTGGTTGAGTAT
ACAGAGAAACTATCACTACTGTTGTTTTCATTATTTCCATATATAAATATACACACAC
ATATACATTTTATGTAGATACCAGAATATAAATTAATAAACTTCATCCTACTTTAATG
CCAGCTCATGCAAGAGTTGAGAGGAAAAGTGTATATTGATTTTCTACATGTTTCTAA
ATCTCTATTAATCAAACATCTCTTTCCTCTTCCACATTCTACTCTTTTCTCTCATAATG
AGTTTATATCAGTTAACCATTTAGTATCTTCCTCCTACTCAGCTGGACACTTCAACAT
AATATGAAATACTCCACTTCTCCCATTGGAACAACAACAAAAAAACCTTTCTTGAAG
TAGACATCTAAATGTCTGATATGTGTTTTTCAATTTTCTTTTTGCAGAATTTTATCTTT
TCCAAGTTGTTCCTAACTACCAAATTTTGGCAGCTTTTATTTTATAATCCAGCTACTT
AGTGTTATCAAGCACTTCCTTTATCTCAACAAGATGCCAGCTTCAAACAATACATAA
ACCAAATGACTTAGAACATTTCTTATACTTAGCACCTGTCACATCTACTCTAATCAAT
TATTATTTCTGCAGAAATACTGACTCATAATAGACAATGTAATGTGAGAAGGTGCTC
CTGAAATGAGACTGATCATTTTATTTTTCTTTTTTTAATTCCTAAGGAATCTCTCCTAT
TGGTCAAGTTATTGTGACCTGATTAATGGTACAGGTAAGGACAATCTGTTTTGCGGT
AATATCACAGTGAAACCACCTTCTTTCTCCCACAAATCCTCCATCATATCTACAATGT
TCTGGAGGCTGAGGATATGCATTTACTTATCTTTGTATCCCTACAACATAATTCAGA
GTCTCTATACTTATGCTGCTGCTGCTGCTAAGTCGCTTCAATCGCGTCCGACTCTGTG
CGACCCCATAGATGGCAGCCCACCAGGCTCCCCCATCCCTGGGATTCTCCAGGCAAG
AACACTGCAGTGGGTTGCCATTTCCTTCTCCGATGCATGAAAGTGAAAAGTGAAAGT
GAAGTCGCTCAGTCGTGTCCAACTCTTAGAGACCCCAGGGACTGCAGCCCACCAGG
CTCCTCCATCCATGGGATTCTCCAGGCAAGAGCACCGGAGTGGAGTGCCACCACCCT
CTCCACTATACTTATATATAGGCTTATTAAATATAAGTCTAATGTGATGGTATTTCTG
AAGATTAACCATTGAAGATAAGCCTTTACTACATTCCTTGGGGAATGTTTGCTTCAT
AAAAGCTTTACTCAAGTGAACTCCCTTCCTGGCCACAGAAGTAAGGGGGTAGAATA
TAAGTAGTGGTCACTGTTTGCATGTTTTGGGAATACTATATAAGCTGTGACCAACCG
AGACAGCATATTAAAAGCAGAGACATGACTTTGCTGACAAAGGTCCATCTAGTCAA
ACCTATGGTTTTTCCATTAGTCATGTATGAGTGTGAGAGTTGGACCATAAAGAAAGC
TGAGCGCAAAACTGATAATTTTGAACTGTGGTGTCAGAGTAGACTCTTTTTGAGGGT
CCCATGGACTGCAAGGAGATCAAACCAGTCAATCCTAAAGGAAATCAGTCCCGAAT
ATTCATTGGAAGGACTGATGCTGAAGATGAAACTCCAATACTTTGACCACCTGATGC
AAAGAATTGCCTCCTTAGAAAAAATCCTGATGCTGGGAAAGTTTGAAGGGAGGAGG
AGAAGCGGACAGCTGAGGATGAGATGGTTGGATGGCATCACCAACTCGATGGACAC
AAGTCTGAGCAAGCTCTGGGAGTTGGTGATGGACAGGGAAGTCTGGCATGCTGCAG
TCCATGGGATCACAAAGAGTCGGACAAAACTGAACTGAATGGTTTAAAACTTAAAA
GAATTAAAAAATCAAGATAAATACAAAAGTAAACCTTGTGTTCAAGTGGAGGTTTC

AATATGAGGCAGAATCAGAGAAGTTCAAGGCTGAACACAGATTTTGAATGAAATTA
TTAAAATGAGAAAGAAAATAACAACAATAAAACACAGTTGCATCTGTACTAACCAT
GAGAAGTATATGCAAAAATAACATTGTGAATTAGAGCTGGGAAGAAAGAAGGTACT
AGGACTTTAGATTCAGAGAGTGATAGATCCAGTTTATTATTCTCCTTAAAGAAAAGC
AAAGTACACATATGCCTATTAGTTTAAGTATTAAAATTTTTAAAAGTAATAGGAATA
AAATATTTAAAATCAAAGGTGATCTTTCAGCTACTAAGAAGCAGAAGCAGAATATT
ATGTGTGTAAAGTTGATAAGGCAACACTTTAGAAGAATGTAACACAATGAAGAAAA
AAATGTTATACATAAAGTGCGTAAAAGAAAATAAGGAAACATAGAGAACGTTCATT
TTCACAAAGGTATTGCTGAGTTTCACAGGTTTAATTTTGTTTACTCTGTGTTAAAAAA
AAAACACACACACAAACAGTAAAACCACCGCTGAATTTGCTTTGCAACACTTTGAGT
CATGGTGATGGGTGAAGAAATCCTTTCCAAGTAGTTCATTTCTGCAAATAGTCAACT
GTCCAAATACCACTAACTTTAATTTCATAAGCATACGCTTCAGTCCTTAAAATAGAA
ATATACTGTGCTTCTCCTAAATAGTAAACTATTGTTTATGGGCTTAAAATACATTTAA
ATCTTAGTCTAAAACAAAAAATAGAGCAGTTTTAAAACAGTAATAAGAGGATCCAT
TAATGACAGTAACAGATACATAGAATCAGGCATGAAATTCAAAGCATCTAAGAAGA
AAATCACAGTAAAACAAACTAGGAGCAAAGGTTGATTGGATAGACTAGGGACTCTG
ATGGGAGATTCTCTTGGTCATAGTACTAAAAAAAGAGACTGCTATTGCTGCTGCTGC
TGCTGCTGCTGCTGCTGCTGCTGCTGCTGCTGCTGCTGCTGCTAAGTCGCTTCAGTCG
TGTCCAACTCTGTGTGACCCCATAGACAGCAGCCCACCAGGCTCCCCCGTCCCTGGG
ATTCTCCAGGCTAGAACACTGGAGTGGGTTGCCATTTCCTTCTCCAATGCATGAAAT
TGAAAAGTGAAAGTGTTTGTTAGCCATCCGTATGTCTTCTTTGGAGAAATGTCTATTT
AGTTCTTTGGCCCATTTTTTGATTGGGTCGTTTATTTTTCTGGAGTTGAGCTGCAGAA
GTTGCTTGTATATTTTTGAGATTAGTTGTTTGTCAGTTGCTTCATTTGCTATTATTTTC
TCCCATTCAGAAGGCTGTCTTTTCACCTTGCTTATATTTTCCTTTGTTGTGCAGAAGC
TTTTAATTTTAATATATATGGAATTTAGGAAGATGGCAATGACGACCCTGTATGCAA
GACAGGGAAAGAGACACAGATGTGTATAACGGACTTTTTGGACTCAGAGGGAGAGG
GAGAGGGTGGGATGATTTGGGAGAATGACATTCTAACATGTATACTATCATGTGAAT
TGAATCGCCAGTCTATGTCTGACGCAGGATGCAGCATGCTTGGGGCTGGTGCATGGG
GATGACCCAGAAAGATGTTATGGGGAGGGAGGTGGAGGGGGTTCATGTTTGGGAAT
GCATGAAAAAAATAAATAAATAAATAAATAAAATAAATAAAATCTAAAAAAAATAA
AAAAAAAAAAAAAAAAAAAGAAAAGTGAAAGTGAAGTCGCTCAGTTGTGTCCGACT
CTTAGCGACCCCATGGACTGCAGCCTACCAGGCTCCTCTGTCCATGGGATTTTCCAG
GCAAGAGTACTGGAGTGGGGTGCCACTGCCTTCTCCGAGAAAAAGAGACTAATGAA
ATGTAAATGGATCCAAGGTGATTTTTAAATCATGATTTAGAAGTACTTCCTCTACCA
TCTTCATTAAGTTTAAAGACATAGAATGAAAATATTTATTTGAATTTATTTTAAAAA
GGGAGCCCTGCTGGCACCAGCTATGTTACAGTCATGGCCCTTGGTAGTCACCGGTGT
CATTCCGACATAATCTTCGATGATATTTCTGATGGTTTTTAAAGACTGTCAATGTACT
CTTTTCCTTTGATGGCTAAGGTGTTGGGACACCAGTTGTCGGCAAGGCCCATTCGGG
GACCTATTTGGGGAATTTAATCTTCACTATCCCATTCTCTCAATGTCTCTATCAGGTT
CACGGAGATCCTCTGTTATATTTCACCTATCACAAGAATGTATGTGAAATGTAATAA
AATCACAAGGTCAGAGCAGTTACCAGAAAAAGAGTGTGGTATTCAGAACTGAAGGA
TAGGTCTTACTGTTTTGAACAAACATGTTCAGTGGTGGCTCATGTTTGGTGATATTCA
TTTGTGGAGCAAATGGAGGAATTATCTAGCTATTTTTTCTTACATCCAGTGTGTACTA

TGGAGCACAACCATAGTAAAGATTTGCTAAGCTCAAAAAATGCCCTTTTTAGTTTGA
TGTGTATACTTTCCAACCATTTGTGTGATATTGTTTAGCAATTTGAAAAATATTGATC
AGCCATAAGGATAGGTGAATGGATCACTATTAAAAGTACGAATAACATTTACAAAA
ATGAGTTATAAAGAAATCTCAAGATTAAAAAATTGCTACTCTACTGATAGATGGAA
GATTTAAAAACATAAAATCGCAGTTTTAACAACGTGATTTTGACATTTTAAAAAACT
TTTATGGAGTGGGAAAATTTAATATGAAACAGAAAGCCAATGACTACATATAGTAT
ACGGACTTATTAGATATGACAGCTGCTAGCCACATGTCACAATTCAGCAGCGCTTGA
AATGCAGCTAGTACAAACTGAAATACTGTAATTATAAACACTGTATTTTCAGATTTT
TGTTAATTTTTATATGTTAAAATGATGGTATTTTTGATATATGGGGTTAAACAACATA
TACTTGATACATTTTTTTTTCTCATCTTTAAAATGTAGTTACTGGAGAATTTTAAATA
TACATTTTGCATTACATTTCTCTTAAGCATGCTGGAATCAAGATTTCTAGATTTGTCT
AAGACACACTCTTTCCATCTAATCCCTTGCCACTGATTTGGTTTTTAAACAGATGCAG
CCTCATTTCCACCTTTTGTTGAGAAGACAAGGAAATTGCAATTTTTCTCCTCTGATAT
TTGCAGGTAAGAGAAAGACAATGATGTATTTCTCCAAGTCAGAATTCTTTCTCTAAG
TGATAAAACACAGACCAAGAAACTCATGCTTATTAATCATGTTATTTGAGAAAAATA
AAATACTTTTAGGGGCTTTTTCCTGATTAAGAATAAATACAATGTGAAATTCATGAT
GTATTTATGCCCTGGAGAAGGTAATGGCAAACCACTCCAGTATTCTTGCCTAGAGAA
TACCATGGACAGAGGACCATGGTGGGCTACAGTCCATGGGGTTGCAAAGAGTTGGA
CACAACTGAGCCACCAATACACACACACACACACACACACACACACACACAATGTA
TTTATGAGTCTTTAACATTTGATGTAGAAGGATGTTGTGTATAATGCAAATCAGTTTC
TTTTTGTTATTTTTAAGAAAGAACTACTTTTTTCATTCTTTTACTAAATTAGCAGTAA
GAGTGATAACAGTCAAGTCTAGAAAAGCATGCCCAGTCTTTGTAAAGAAGAGGATA
CCACAATCACAAAACTATGTTCATGTGCCCCACTGACAATACAGCATCTTTTCAAAT
TGACGGGGGCCCAGTAGGTATCACACAAGTGTTGGCGCCTACACCTGTATCCTTGAA
CGCTCACCTGCTTTCTTAACACTGTTTGGGAAAGCAAGCATAATATCTCTGAATCTTT
TTCCTTATCTGTATAATTGAAAAGAACAGCATTTCTTCAAATAGTCATCTGGGGACT
AGAAAATATTTAATCTATAAAGGAAACTAACTGTGAGGAATGCCTTAATTGTAAATA
CTCCACAGTAATTCACAAGCAAAAATTATTCTCAGAAGCGTTTGAAAACAAAGATA
AGAATAGCTCATACTCAAGTATTAAGGTCTAGTGTGTCTTAAAGTTTTGCAAGTTTTT
TTTTCCTAAAAGGTAAGGATTCTAATAAGATTATATCTAATTTTTCTAATTTAAGGCA
GCAATAGTATAAATTTAAATATAAGAGAAAACAAATCTCCACAACAAAGTTCAGGT
TCCCAGAATGAAGCTCCTTCTTATCTGTGTTCAGGTCTATCTATGCTGTGTTCGGAGC
TGAACTGAATCTGAAAGGAATCCCTGTGTACAGATTTGTTCTTCCATCCTTGGCTTTT
GCATCTCCACTTCAAAATCCAGACAACCACTGTTTCTGCACAGAAAAAATCATCTCA
AAAAATTGTACCTTATATGGTGTGCTAGACATTAGCAAATGCAAAGAAGGTGAGTA
CAAGTTCTCAGTGTCACAATCCTTGGCATAATTTGCATTATCCTTTAAAATGTAAACT
TCACCCAGGATTCCTTTCCACCAGAAATATAAAGTGGGAAAGCAATAAGGGCAGAA
AGTAAGAGTGATGGTAGGATATTTTTGTTTTGATGTTTGTCTTGCTCTCAAGGTCTCA
GTTGTTTTCATGTTGCAAAACTTAGTTTTTATTAGGTATAATCATGCTTATTGATACT
TCCTGTGCTACTAGGAAAATTTCTCTTTAGGTCTTTTGCTCATTTTCAAATTGGTTTAT
TATTTTTTTTGAGTTGTATAAATTCTTGTATATTTTGGATGTTAATCACTTTTGGGATA
TGTGTTTTGGAAGTATTTTCTCCCACCCACAGGTAACATTTTCATTTCTGACCATTTC
TTTTGCTGTGTAGAATGGCTGTGCTCCTTTTAGATGTAGGGTAAAATGAAAATAGTT

TGCAGACTACTTTGAGCATCCCAACTTGTCCATCTGACATTTTTCTTCTTCTACCCTA
GAATCTGTAAATAAGTATATTTTAGGATGTCTCTGAAACAAAAATTGATTCAAACAT
GGCATCTGGAGTCTATCTTATTTTGCTTACCTTCTTCATGTTAGAGTGGGTGAGAACA
AAGAAAGAAAAGCCTCTTTAAATGAATTCTCGGTCCAAAGACATTTTACAGCCTGAA
ACATGAAGGCATATGGTTGTAACTTTGTGTCCAGTAGCTAAGCTTTACATACTGAAA
ACACCTAAGGTGAAAATAAACACCTAAGGGATGAACAACAGACATTAACTAAATAG
TCAGTAGGATGACGTAGAAGTTTACATTTTATTTTTGTTCAAAAGTGATCAGATACA
ATAATTTTCCCTCACTCTCATCCCCTGATAAGATTTCAAAGCCAAATTTGTGTTTCTT
TTTTTAAATTAAAAAGCTTTGTTTTCAAGGAAATCTTTCCTAAGTGGTGTATGAAATG
AGGAAAATGATTTACCCTAACTGTCTCTTAAAACTTGCTTTCAGGAAAACCTGTGTA
CATTTCACTTCCTCATTTTCTACATGGAAGTCCTGAACTTGCAGAACCTATTGAAGGC
TTAAGTCCAAATGAAGAAGAACATAGCACGTACCTAGATGTTGAACCTGTAAGCAA
ATACTTATACTTTATTGATCAGATTTGTTTTTGTTTTGTTTTGTTTTGTTTTGTTTTGTT
TTAGTTTTTTATTTTTTAAATTTTAAAATCTTTAATTCTTACATGCGTTCCCAAACATG
ACCCCCCCTCCCACCTCCCTCCCCATAACATCTCTCTGGGTCATCCCCATGCACCAGC
CCCAAGCATGCTGTATCCTGCGTCAGACATAGACTGGCGATTCAATTCTTACATGAT
AGTATACATGTTAGAATGCCATTCTCCCAAATCATCCCACCCTCTCCCTCTCCCTCTG
AGTCCAAAAGTCCGTTATACACATCTGTGTCTTTTTTCCTGTCTTGTGTACAGGGTCG
TCATTGCCATCTTCCTAAATTCCATATATATGTGTTAGTATACTGTATTGGTGTTTTTC
TTTCTGGCTTACTTCACTCTGCATAATCGGCTCCAGTTTCATCCATCTCATCAGAACT
GATTCAAATGAATTCTTTTTAACGGCTGAGTAATACTCCATTGTGTATATGTACCACA
GCTTTCTTATCCATTCATCTGCTGATGGACATCTAGGTTGTTTCCATGTCCTGGCTAT
TATAAACAGTGCTGCGATGAACATTGGGGTACACGTGTCTCTTTCCATTCTGGTTTCC
TTGGTGTGTATGCCCAGCAGTGGGATCGCTGGGTCATAAGGTAGTTCTATTTGCAAT
TTTTTAAGGAATCTCCACACTGTTCTCCATAGTGGCTGTACTAGTTTGCATTCCCACT
AACAGTGTAGGAGGGTTCCCTTTTCTCCACACCCTCTCCAGCATTTATTGCTTGCAGA
TTTTTGGATTGCAGACATTCTGACTGGTGGGAAGTGGTACCTCATTGTGGTTTTGATT
TGCATTTCTCTAATAATGAGTGATGTTAAAAATGAAGAAAAAATTAAATTATCTCCT
TTCTATCAATCACATTTAGTCACATTTATTGTACTACATTTGTAATAAATTACTTATTT
TTACTTCGAGGAAAATTACAGTGATTTCTTCCTGTTGTTGCTTATGAAATGTCAAGAC
AATTTTAGTTTTTCAGCAAAGGTCAGGAATAATGAAAATCATTCTATTTGGATTAAA
ATGATTTTACATGTTACTCTTTCAAATGTACTTTATACTTAACTGCAAATATTGCTTA
TATGAAGGGACCCTGTAGATTATGAGGGATTAAAATTTTTTATGGGTATACATACAT
TTGAGGTAAGATCAAATATATTTCTTAGTGTCAAGAAAAATCTATTAAGTCACTATA
GTTTAACAGTTTCAATTTTTCATTTATATTAATATTCCCCTTTTCAACAGATAAATGA
AATATCAGGACTTTAGTAGTCATATTTTAGTTTAAATGATGACAGAAGTAACATTAT
AATTATACTGATGATGCATGGATTCTGACTGAGTACATATTTAAATTCCAGGATATA
TTTTGTCAAATGAGTTTTAGATTTCACTACTGTTGATGTCGTTACTAATTCCCCAAAA
TCAATTCAAGTAAATGTCACAAATCTAACTAAACCTTGACATTAGGTTGGGCAACGA
TATATCTGCATACACACATGCATATACTGATGAAATATAATTATGGAAAGATACATG
TGTAATATTTAATATGTCTGATTAAGTCCAAATAATAAATAACAAATTTCACAGGTT
CAAGTAGTACACTTTCACCTTTTAGGCTCTACATAACAAAAATAAGGTCTGGATATT
ATAAAAGAATGTTTTTCAAATAAGTTGGTTATTAGTTTGTTCTCTTTTTAGATAACTG

GATTTACTTTACGATTTGCAAAACGGCTGCAGATCAACATACTGGTCAAGCCAGCAA
GAAAAATTGAGTGAGTTTCTTAAACAAGTTTCATTTCGATTTTTCAAATTTTCTTGTA
AGATGCCAGGTATATCTATCAAAGGAGATGGAAATAAAATTTCAACTTCTGTTCGGC
TTAATGTCATTAAATAATTATTAAATACTTATAACTGTCATTTGCAATTTAGAATTAC
ATCTAATGTGTAGTGAAATGGTACTTACCCATATTTTGGAATGAGTATTTCTGGTAAT
TTCTCTCTTTGCTAACTGTCCAAATATTTTTATGACAATAGTTTCAGCATTCATATTTT
AGTTTCCTGGGTATAATTTGTTGAAAGGGAAAATACATACATAGAAAAATTCTAATG
AAAAAACCTGTGAGTCCACCTTTCACTGCACGGGACACTGGTTTGATACCTGGTACG
GGAAGATCCCACATGCCTCTGGGAAACTAACCCCGGGCACCACAACTGAGCCTGTG
CTCTGGAGCTCTCGAGTCGCAACTACTGAGCCCAAATGCAGCAGCTACTGAAGCCTG
CGCTCCTGGAGCCCCTGCTCCACAACAAGAGAAGCCTCTCCAATGAGAAGCCCATG
CCCAGCAACTAGAGTAGCCGCTGCTTGCCGCAACTAGAGAAGGCCCATGTACGACA
ACGAAGACCCAGTGCAGCCAAAAATAAATAAATGTTTTAAGAAATCTGTATTAAAC
AGATGTCTATGTAGAAAAGATATCTGAGAAACAGCAATATTAAAATTTAAATGCTTC
ATTACAGGGAAAACAAAGCAAGTGACTTACCCAGGTTGTAAAAGCAACTAATTTCT
GAACATTTATTTTCACTAGTAATTACTAATATCTGTCATATGTGTATAGCATATTAGT
ATATATTTTCATACTTATTTTCAATGTTTATTACAGTGCATTAAAGAATCTGAAGCAC
AACTATATTGTCCCTATTCTTTGGCTTAATGAGGTTAGTATTTTTATCTATTAGTCACT
AAAAACAGTAAGCTTCTTAATATACAGAAAAAGTTTTTGATGTTTGAAAATTAAAAT
ATATATACACACACACAGTATTTGAACTTGTCTTTCCATTGTATCTATGGGTGAATTT
ATATATTATTAATTATATAATTAATTATTTTTTAAAGCAAGTCATACAGAAGTATCAG
GATTATGTTAGACTTAAGTCTAACTTTTATGAAAATAAAAATACTTCATAGTGCATTT
ATTAGATAATTTTTCTTGTCATAGTGCATGTGTTAGGAAAGAGTCATGAAAATATTA
AATTCCAAATAACTCAATATGAGCCTTGTAGTGCTAATAAAACCACCCAAGCTTATA
ATGGGCAAATTAGAGTAGATGTAGGCAGTCTGTCTAGTGCCTGACAAGATATTTTTA
ATATAAATCATGGAACCGACAAACCACAGAATTAGGAAATGCAGTGCATAAGCCTT
TAGAGGATAGAAACAGTTGAGCTTTGCCATTTCCCTCTGTCACTAAGAAGCAACCTT
TGGGAAACTGGAGTCTTACTGAAAGACTCCAGTAAGACTGCTATACTGATCAATGA
AATCAATATATACAATAGCACTAGATAACATGTTTAAAGGGAGGAATATTATCTTTT
TCTCTCTAAATATTCTACCTGGGCATTTACTGTCTTCTTTGACATGCAAAGAGAATTC
CAACAGGCTTGTCTTGCAGATAAAGATGACTGACACCTGTGTGCTGTTTTGATAAAG
TGATACACATAAGTGGAGAGTTTTGAGTATTCTGCTCTCAAATCTATTCAAATAACA
CTATTTATTAACTTGATTACAGACTGGTACCATTGGTGATGAGAAGGCGGAAATGTT
CAGAAAGCAAGTGACGGGGAAAATAAACCTCCTTGGCCTGGTGGAAATGGTCTTGC
TCAGTGTTGGTGTGGTGATGTTTATTGCTTTTATGATTTCATATTGTGCATGCAGATC
AAAGAGAGTAAATTAAGTAAGTATATAAGAAACCATATGTGACTTCAGTAACATTA
AATATTACCTGTTTTCATTTTATCAAAACAGTTATAAACTAGGCTTCAAATATTAATG
AACATGTCTAGTCAGCTATCATTTTTAATACATCCTTGAAACTTTGAGACACTTAATA
TAAGTTAAAATGAAGACTGAATGGGTGCTTAGGCTTATCAGTATTAAACAGATCACT
ATATTTCATCTTTATTCTGGGAGGAAATAGGCTAAGCTGTGAAGGTGTCACCTTCAA
AAAGTTAGAATGCTGCACAAAAGCTATACATGTACTCAATTTTTATTTCTTAATTATT
TCTAGATAGTTATTTTTAAAATCACAAATAATTTATCTGACTAGGAAACATGTGAAA
CTGAAAATGGCAGCTATTTGGGGAGACAAATTTATAAAGGAAAAGAAAATAAAAAC

TAAAATTAGATAAGCTATAAAATGATGAGAATTAAAGAGTCAATAAATGGTAGAGA
TAAAATCAGATAAAAATATGCTATTTTTAAATGCAAATAGTGTATTAAATGGGCTTC
CCTGGTGGCTCAGAGGTTAAAGCATCTGCCTGGAATGTGGGAGACCGAGGTTCGAT
CCCTGGGTAGGGAAGATCCCCTGGAGAAAGAAATGGCAACCCACTCTAGTATTCTT
GCCTGGAGAATCCCGTGGAAGGAGGAGCCTGGTAGGCTACAGTCCACGGGATCACA
AAGAGTCAGACACGACTGAGCGACTTCACTCACTTAAGTGTATTAATATAAAAACA
GAGCTGCTGCATATGGACATGCTGTCCAAAATCATGACATGTACCCTAGATGTTCCA
TGTGAAAGCCCTGATGAATGATTATAATTCAGGAGCTAGAATTAAATGAGTACAAG
AAAAATAGAATAAAGATAATGTTGAACTCACTAAAACCAAATAATTAGAGTGTAAA
GGAAAAATCAGATAAATATGGACGGATGAGTTTTAGCAAAACTGAAGAGGTGGTTC
AGGCCTCTGTGTTTTAAATATTAGTACCCGTGTCCCCTTGTACATAAAAGAGTGTAT
GCCAGACCCTATTTACTCTAAATGGAAGCCTTTCTTTAATAATCATAGGTGGCATTG
GTATGGCCTGAGGGGAGTGAATTCTCCACTAACTCATAGGTTCTATCTGCCGTTCCTT
CCCTGGGATATGAAAGAAAAAAAATGAAGTGAAAATCAATTGGTAGCTTCCTACTA
TTTTCTACCTCATAAACTGTAGCTCACCAGGCTCCTCTGTCTATGGAATTCTCCAGGC
AAGAATACTGGAATGGGCTGCCATTCCCTACTCCAAAGGACCTTCCTGGCCCAGGG
ATGGAACCCGGGTCTCCTGCATTGCAGGCAGATTCTTTACCATCTAAGCCACCAGGG
AAAACCAGAAGAAGAAAAAATATATATATATATGTATATATGAAATCTAAATTAAA
GCAAGGACTGATGCTGAAGCTCCTATACTTTGGGCCACCTGATGTGAAGAGCTGACT
CACTGGAAAAGACTGAGGGAATGAAGAAAAGAGGGTGATAGAGGATGAGATGGCT
GGATGGCATCACTGATTCAATAGACATGAGTTTGAGCAAACTCAGGGAGATAGTGA
AACACTGGGTAGCCTGGCATGCTCCAGTTCATAGGATTGCAAAGAGTTGGACATAAT
TTATCAAGTGAACAACGACAGCAGCAAATTGCTCTGAAGAAAACAATTCACAATTTT
AAAACAAGTCTTCTTATTCCACTTTCAATCAGACTGCTGACAGTAAAATGTCCTGCA
TAGAAATTCTGGAGCTGCCACTGGCAATTATTGACCTTAATGTGCTCAAATCATGCT
ATTTTACATGAATTTCACAGCTAATCCCTAATAAAAATTTATGATTTTCCAGTAGCCA
TGGATCTATCAGCATCCATTCTGTTTTTTGAAGACATTACAGAAAACTAAATTTTAAC
AGTATAAAACTGTCTATTTTCAAAGTGTGAATCAGTTAAATTGAAGATTTAAAAAAC
AAATTGGCCTTTTTGGACCAATTTGTGTGTGTGTGTTCGTGAAATTGAAAACAGACT
GAGGAAGGCAACTCGATCTTTTGTTTTGATGCTTTCTGCCTATATATATTTCCTTAAA
AGGAGACTGAACAGTTCTGCCTACAGTCTACTTGGACACAATATATTCAAAATGCTA
ATACATTCATGTTATAATAAAGGAAATATGGCATCACCTTTAAAACCTGTCATCAAT
CCAGTAACCAGTTATGTTTAAACACAGTAATTCATATGACCATTTATATAACAGACC
AAAATATCACCTATTGAAAAGCCACGTTAAAACAAAAACTTTTTCTAGTGGGAAAAT
AAGCTTTTCAAAATGAACTATAGTAGTTATAAATTTTGGGGTATTATTCTCTATTAAA
ATTATATAACCTATTTCATCAATGCTTAATGAGCCCTGAATATCCGACATGTTGGGTC
TCTATGTAGCTTGGGCTTCCCTGGTAGCTCAGCAGGTAAAGAATCCGCCTGCAATCA
ACCCTGGTTTGATTCCTGGGTCAGGAAGGTCCCCTGGATGAGGACATGGCAGTCCAC
TTCAGTATTCTTGCCTGGAGAATCCCCATGGATAGAGGAGCCTGGAGGGCTAGAGTC
TATGTGGTCACAATGAGTGGGACACAGCTGAGCGATTCAGCAGAGCTATGCAGCTG
TAAGTGAAAGACTGAAAACTTTCCAGAGTGTCTTTATCAGCAAGTACATTGGTTTGG
CTTAAACTTCTGACTGTTATAAGGAAAGTAACTGCTCTTTATACAACAGTATGTCTTT
TTTCTTTACAAATAGAGAAGAAAAATTTTCTAGGTTGATTACTTGATTTACTCGATTT

AGAAGAATCACAGATGTAGATTTCCTTAAACAGGCCTCATGTCGAATACTGTAATTA
TGCCTTTGGGAAGATTTATAAACAACTGAAAAATAAGGAGACGCTGTGCAGAGGAA
ATAAAGTAAAGACGGGAGAGTGAAGGTGTGGATTTATTCAATAGATCACAAAAAGA
ATGAGCCAACAGTCTTTTCAAAAGTAGCAGGACATACACCACTAAATGGAGGATGG
TCTGAAGGGACAGCAAGGAGTGGAAATGCTCATGCAGAGCAGATTTAATGAGTGAC
CCTCCTTATTTGACTGCGTGCAGCTCTTGAGAGCTGGGAAGGAGCCTTCCATGAACC
CGGGAATGGGAAAATAACTGCCTTATCCGTGAAGTACATCAGAGAAATTTTCTCTAG
AGCTGGAGAAATTGGGCTGATTTCTAGAAAATATAACATCTTTAATATATAAAATTT
GAGTCTTAACTATTTTTCCATGAATTTCTGATATGTACCTTACCTTTCTTCTTTCCAGG
GAGCAAAAGAGTCTCTACATTTATGCACCAGCTATGTCAGGATCTTTCTTATTATCA
CCTTGCAAAAGTCAAGATTTTCATGCTTTATTTTTGCAAAACACACCTTATCTTACAG
TTCAAGAACTGGTGACACTCCCTCCCACCCGCCACCCATAAGCAGCAGCACATTTCA
AAAGGATTATTAAGACATCATTAAAATCCACACTTCGAACAAAAAACCAGCACTTA
AAAAAATTCAACATATTCACAATGGAATGGACTTCAGTTTCTACAGATGTGGCTTGA
GCGTGACCCACTTATTTTCACTTGGTACTGATTCACCGATTTATTCCCAATGCTAAAT
TCAGCAGCGAGATGGTCATTCTACACAAATCCTGGACCCTGAACTAGCCTTCCCCAT
CAAAAGGAAAACATCACTACCCTCATCAGTGTCCCTGCAAAACACAGACCCAGTAC
TATCAGGACATTGCCTCTGAAGAGCAAATATTTTGAAAGATATTTGAAAACATAAAC
TAAATGACTGGCCTATGAACCATTTATTATACATCTCTCAAGTTTTTTCCTCTATGGA
ATCCTTTGTACAGCATCAGCAAGGCATTATAACCATGGTATTTATAACAATATGTGC
TTGTATTTTTGTCAATGGTTATGCATTTGGACGTGTTTGAGAACATTGCTATCTTCCA
TGTTCTACAGCTCTTCTGGAAATCTGAGCAAATTTTCATCTTTCTACTCAGTTGCATA
CAACTTATGCTTGGCACCTCCAGAATTCTGTGCTAGTACTGAGAGATGTAAATGATA
ATACAGAAATTATTGTGTGGAAGATTACTAGCTGTAGAACGTGCATTCATAGTTATT
TGTTGTAAAATTTCTTGCTTTCATTGTCACCAGAAAGACTGGTTTTGAACATTAAAAG
ACGTTCCTTAAAA

ERV_13

TCTCCCCCTCTCCCTCCCCCATGCACTCCTCCACTCTCTTCTCTTCAAGTCTTTGGGTT
GGCATGCCCTCACCCTTCGAGGATGGAGTCTCCTGCTATCTTCTAAATAAAATAGAG
CTGTAACACTGATTTACCTGAGAGCTATAACACAGTTTGTCCAAGACCCGAGAGCTG
TGATGCACCGAGGGCTTTAATGTCTGTCGCTCCAAATCTTTGTTGTGATGAGACAAA
GAACCGAGGAACATACACTTGCGTGACATCTATGGTGCTGTGACTCGGATATAACCT
GGCTGAAACAACCTCCGCGTGGAAGAGGCCAGGCACAGCAGGAGCCCAACTCAGC
GAAGCTCCCGCAGTAGAGGCGGAAGGTGAAGAAAATCCAGCGCAGGGGAAGGCCC
ATCATGCTGGAAACCGGAACAACGGAAAACAAACTCAGCGGAAAGCTCACGTGGCC
CAGTCTCAGATCCCAGAAGACCTCTGGTTAAGGTAAGAGGTCCTCACTGGAGGGAC
ATGCCTAATGAAATCTTAATTCCTTTACAGTCTCTCGTTTTTTGTTCCCGCAAACCTC
CTGCGAACAGGCGGGCGGCAGGGGGCACAACTGAGGGACTCTGGAGAGGCTGCTCC
TCAGCATGTCTCAAAGGCACTATCTGCTGAGCCCCAGTAGCTGTTACACAAGCCAGT
GGGGGTTCTTCTGTCCTTTCTCTTCTCTGTGCCAAGGATCAGACCTATGAAATTGTGA
GCACCGGTCAGATATTTAGCAAATTTTCCAGCGGGCTATGAAGGGGATTCCTTGGCA
CGTTTTTCCCACTGCTTTTTCCTCCTGTCCTTCAGCTCTCTCCCAGGACTCAAGCTCG

GCCAAAACTAATGAAACTCAGGCCCTGATGTCTCATTGCAAAAATTCATTGAGAGAC
AAAGAGATAAGAGGTGGATTTGTTAGGATTCAGAGAGAAGCCACTCTTCAGGGTGT
GAACCATTGCCAAGGGCAAGGGCTGGGGCCACGGGATTAGGCCTGGCTAGGTTTTG
TGAAGGGGTGGAATTCATATGCTAATGAGTGGGAGGATCATCCCTACCATTGGGGA
ACCACCCACTCCTCCCTCTTTTGCCTTGGAGCTGTCCTGCCACCTCTGGGTGTGTCTG
TTGGCTTATAGATTGGGGATTAAGTACTTGAATTTCACTTTTCATCTTGGACCCAGTT
GGTTTTAATTGGTTTACATTATCCCCTTGTGCTATGTCATTCTTTCAAATGTTGTGCTC
TGCCCCTTTCCCTCCTGTTTCATGCTCTTTGCCTGAGCCCCATCCAGACCCACAAGGT
TGCCTCTACGATCTTCTGGAGAGACAACCAGAAAATAGATGGCCTTGGGAGCGAAA
TACTGTATAATATCCAACCCCCTAATCCTACCCAGTACTGGTCACACTGGAGATCTT
GGGAACGCCCAACTCCATCAGTCCAGGGGCCCCAGGAGGTCATCACGCCTTGACCT
GCCTAGGATCTGGTCCTCGAAATGTTGTCATGCCTTAACCTGCTCGGGGATCCGCTA
GTCCCAGGGGTCCCAGGAGGTCGTCACGCCTCGACCTGCCCAGGGATTTGATCTCTA
GGAGGCTGTCACGCCTCAGCCTGCCTGGGGATCTGCACCCTGACCTGGGGACGCCTG
GCTCTCAGGTTACAACAGTACTAGGTAGGATGAGCTTACATCATATCTATTCCCATC
CACGGTGGGCAAACTAGCAACGAGTTACAAACCCCTTAATTCTATCCAGCATTGGTC
ATGCTGGAGACCTTGGGGATGCCCAAACTCCAGTCCGGGGGTCCCAGGAGGTCATC
ACGCCTTGACCTGCCTAGGGATCTGGTCCTCGAAAAGTTATCACGCCTCAACCTGCT
CGGGGATCCGCCAGTCCCAGGGGTCCCAGGAGGTCGTCATGCCTCAACCTGCCCAG
GGACCCAATTCTCGGGAGGCCGTCATGCCCTGACCTGCCCGGGGATTCGATCTCTAG
GAGGCTGTCACGCCTCAGCCTGCCTGGGGATCTGCACCCCTGACCTGGGGATGCCTG
GCTCTCAGGTTGCAACAGTTCTGAGTAGGATAATCTTCAGAAAAACTCACCCCTAAG
GAAGTCCACCCATGGAAACAGAAGGAAGCCTATTACTTGTGGGACTGTGCCCAGTC
TCAGCAATAACTCAGGAGCCCGGTGAGAACCCCATTGCCTTTCTGGAAAGGCTGAA
AGAGGCACTCCAAAAGTTTACCAATCTGGACTTAGACTCTTACGAGGGACAGGTGA
TTTTAAAGGAAAAATTCCTGTCCCAATGTGCATCAGATATCAGAATTAAGTTACAAC
AACTACAACAGCAGGACCCTGCTGCCTCTTTAGATGAGATGGTCCAGACAGCCACC
AATACCTTTTATAACAGAGAACAGGAGAAGGAGGCCAAGGCCCAGGAGAAGGAGG
GAAAGAAAGAGACAAGCCATGCCCAGATGCTGGCTGCCCTCCAGAGAAGCCCTATT
GCAAACCCCGAGTCCTTGAAGGACAAGGCATGAGACAAATGCCTGATCTGTAGACA
GGCGGGGCATTGGGCCAAAGTGTCCAAACCATGACAAGTCTCCTAAAACAGCTTGC
CACAAATGGCATCAACTGGGACATTGGGCGGCACTCTGCCCTCGGGACCCAAGAGC
CTCAAGGTCAAGTGCCAAGCCTACCCTCACGATGGTTCAACAAGACTGAAGCGGCC
TGCTCCAGCCAGCCCACCTGTCACAGATAACCATCACGGGGCTGGAGCCAAGGGTG
CAACTGGATGTGGCAGGTAGGTCCGAGAATTTCTTGGTTGACACAGGGGCTGCCTAC
TCTGTCTTGATCTCCTACTCCGGACGCTTCTCCTCCCAAACCTGTACCATTTTGGGTG
CTACAGGAAAAGCAACTACTAAAAGATTCACCCGAGCACTTATTTGTTGCTGGGATG
GACAAATATTTTCCCACCAGTTTCTGGTGGTCCCTGAGTATCCTACTCCCTTATTGGG
AAGATATATATTCACTAAACTGGGGACCACCCTTGTGATGGGAAGTTTTTCAGCCCC
TAGAGCTCTACAGCTCCTGGTTACTACTGAGGAACCCATTACACTTTCAATAGAGAG
GGACCAAAAACCATAGGAAGACAAAATTAACCCCCAGGTGTGGGACCAGGGGATTC
CCAGACGAGCTTACCAAGCCGAAACAGTCATCATTGTCCTCCGAGATCCCACTCGGT
TTCCTAACCAGAAACAATACCCACTCAAAAGAGAGGCTCAGAAGGGACAGCCTTTA

ATAAATAAATTCCTTGCTTGTGGGCTATTGGTCCCCACCAGCTCGCCATGTAACACT
CCAATCCTCTCAGTAAAGAAAAAAGACGGAACCTGGTGAATGGTTCAAGATCTCCA
GATCGTAAATGAAGCTGTAGTCCCCCTCCGTCCCACAGTACCCAATCCCTATGTAAT
CTTGGGAGAAATCCCACCCAGTGCCAAGTGGTTTACAATCTTGGATCTCAAAGATGC
ATTTTTTGCATACCACTGGCTAAACAATCCCAATATCTTTTTGCCTTTGAGTGGGAGG
CCCCAGGAGAAAAACGCCAACAGATGACTTGGACAGTATTACCTTGGGGTTCAGAG
ATAGCACCCACCTGTTTGGACAGGCCCTTAGCCAGGATCTCCTAGATCTGGACCTGG
GACCTAATGGAAAAATATTACAATACGTAGATGACCTACTAATCTGCTCTCCAGATG
AGAAAAGTGCCCAACAACATGCAATTCAGGTTCTAAACTTCTTGGCAGAAAGGGGA
TATAAAGTCTCCCGTGCTAAGGCACAGATGGTCGAGACAAAGGTCATTTACCTGGG
AGTTCAGATTACACACGGGTCCAGGAGGCTGTCCTCTGATCGGGTACAAGGAATCCT
CCAGTTGCTCTCCCCCACGACTTGAAAACAATTGCGAGCTTTCCTGGGACTAACTGG
ATATTATAGAATCTGGATACCCAACTATGGTCTAATTGCCCAGCCCTTATATGAAAG
CTTAAAGGGGTGAGACGATTCAATCCCACTGATGTGGGGAACTCCTCAAAAGAAGG
CAGAGGCTACACTAAAACAGGCCTTAACTCAGGCACCTGCCTTGAGGTTGCCAGAC
CCAGAAAAAGCATTCCAACCTTATGTCCATGAAAGAGAGGGAATAGCTTTGGGAGT
GTTAACTCAAAGGTTGGGATCTGAGCCCCAGCCTGTAGCTTACTTATCCAAAAAGCT
TGATCCAACTACCTGAGGCTGGCCCCCCTGCCTTCAAAATCTTGCAGCTATTGCAAT
CATGATAGAAGATGCTTTAAAACTCTCCTTTGGGGGCAAACTAACTATTTTTACCAG
CCACCAAGTAAAACAACTCTTAAATGGAAGAGGCCATTTATGGATGTCTGGGTCGCT
AAGAGTTGGGCGTGACTGAGCGACTTTACTTTCACTTTTCACTTTCATGCATTGGAG
GAGGAAATGGCAACCCACTCCAGTATTCTTGCCTGGGGAATCCCAGGGACAGAGGA
ACCTAGTGGGCTGCCATCTATGGGGTCGCACAGAGTTGGACACGACTGAAGCGACT
TAGCAGCAGCAGCAGCAGATCAAAGAATCCTCAGATATCAAGTAATGCTGATGGAA
AATCCAGGCCTCACTATATCCCCTTGTGAGGGTCTTAACCCAGCCGCCCTCATGCCT
ACCCCTGAGGGCTCTCTCCCCTTTCACTCATGTCTAGAAACCTTGGACCACTGGACA
AAACCCTGAGAGGGATTGTCAGAAGATCCTCTGACCAATCCTGAGGAAATCTGGTA
CACTGATGGAAGCAGCTTTGTCTTGGATGGAAAAAGAAGAGCCAGGTATGCAGTAG
TCTCCAATTTTGAGACCATAGAGGCTAAGCCTCTGTCACCAGGTACTTCAGCCCAGT
TAGCTAATCTCATAGCCCTGACTCGAGCTTTAGAGCTGGGAAAAGGAAAAAGAATA
GCCATTTACACTGACTCCAAGTATGTCTGCCTGGTGCTACATGCACATGCTGCTATTT
GGAAAGAAAGGGGCCACTTGACCACCCGAGGGTCCCCAATCAAATATGGTGATCAG
ATTCTTTGACTCTTGGAGGCAGTCCATCTGCCCACTGAGGTTTCAGTCTCCCACCGTA
AAGGACATGAGGGAACCAAGCAGCCAATCAGGCAGCTAGGAGAGCAGCATTACAG
AACCATGACCTAACAGGGGTTGCCACCTTAGTTCCACAGACTAATTTGCCAGAAACT
CCTTCCTATACTGAAGGTGAGACTCTCAAAGCTAAGAGCGAGGGCTTCCAAGAAGA
TCATATGAGGTGGCTCCAAAAGGAGGGACTCCTTTTTCTGCCTGGGAACCTCCAATG
GAAGTTGGTTAACTCCTTACATGCCACTACTCATTTAGGAGAAAAGGCCCTCCAAAG
ATTACTAGAAAGGTCCTTCAGAGGAACAGGCTTCCAAACAACTATAAGACAGGTGG
TCTGTTGTCCCACTTGCCAATTAAACAACCCCCAAGGAGCTCGAAGACCCCAGCTGG
CCCAGCCCATCCAACGACATGGGGCCTACCCAGGAGAGGACTGGCAGATGGACTTC
ACCCAGATGCCAGTTTCTCAAGGGTATAAATACCTATTAGTCATGATAGATACATTC
ACAGGATGGATTGAAGCTTTCCCACCCAGACTGAGAAGGCTGAGGAGGTGATATAA

AAACTGCTCCATGAAATCATTCCAAGATTTGGTCTGCCCAGGTCATTACAAAGTGAC
AATGGGACATCATTTACTTCCAAGGTCACCCAAGGGGTCTCAAAAGCATTGGGCATT
ACTTATTATCTCCATTGTGCCTAGCCAATCAATTCTTAAAATCAGCTATAAAAAAGA
TAACCCAGGAGACCTCCCTGGGGTGGAAGGAGGCTTTACCGATAGCTCCTCCGCAC
CCGCATTGCCCCTAAGGAACAGGCTGGTCTTGGTCCTTATGAGATGCTATATGGGAG
ACCTTTTGTTTATGTCAATGACCTCTTCCTAGATCCAGAGGCTCAGACCCTCCAGTCT
TATACCATGGCCATTGGGCAATTCCAACAGGATATACAGTTGTGGGGTATGAACCAG
CACCCAAAAGATTATAAGGAGTCACCACTATATGCTCTGGGGACTCAAGTCCTAATT
AAAGTCTGGAAAGATGGGTCCCCAAAAGCTCAACTCCAGCCCACATGGAAGGGCCC
CTACCCTGTAATACTTTCTACCCCCACAGAAGTCAGGGTACCGGGACATGACTCCTG
GATTCACTACCATGAGTCAAGCCATGGAAGAAGACAGAAGAGGACAGTCAATACAC
CTGTGAGCCCCTCGGAGATCTCAGATACCTATTCAGGACTACCAATGAGTGCCATTC
TAATGAACACCCCCCAAATCTGGTTTCTGGGGATAAGATTTCTCAGGATAACTCTAA
ACAGCCAACATAGCTTGACAGAGATTGTACTCCAAAACAGACAAGAGATAGATCTT
CTGATCCCTGAACAAGGAGGGACTTGAGTCATCCTGGCGATGTGAATTTAAAACTGA
CTTTCATGTTTGGGAATGCATGTAAGAATTAAAGATTTTAAAATTTAAAAAATAATA
ATAAATTTAAAAAAAACTGACTAATGCCCCTACTAGTCCTTGTTACGCTACATTGAT
GATGCTTATGATTATTCCATGAACTGTCAATTGTCTAACCTGTCTTGTCTCTGCCTAG
GTCAACAAGCTACAACATGCAGTGCCAGTTCAACAAAGATATAAAACTACAGCCGA
CCATGAAAAATATCACACCCTTAGATGGACACCGCTATAAGCACTCTGAGGCTTGAG
ACTAACAAGAGGGGGAGGCCCAATACCCCTCACCGCCCCAGTTCAGCAGGAAGTAG
CCAGAAAGACCTCGACGCCCCTATTCCCAAAGAATTGGGCCTCCCATCTCTTGAGGG
GGGAATGTTAGGTAGGTAGAATAGGGAAAAGGAGTCCAAAATGGCGGTGGCTAAA
AGACAAGGAAGGGAAAAGCCCGCGAAAATAGAACAAAAGAAGGTCCGAGGACCAG
AGTGAAGACTTCAGGTAGAACAAACAGCACTCCTGGCTAAGCCCAGTTTGCACAGG
GCAGGCCCAGGTGGAGGAAAACATAAAAGGAGGAGCCAAAGCGCTTTCTCTCTCTC
TTTCTCTCCTGCATGCATGTGCTCTTTCCCTCTCTCTCTCTCCCCCTCTCCCTCCCCCA
TGCACTTCTCCACTCTCTTCTCTTCAAGTCTTTGGGTTGGCATGCCCTCACCCTTCGA
GGATGGATTCTCCTGCTATCTTCTAAATAAAATACAGCTGTAACACTGATGTACCTA
AGAGCTATAACACGGTTTGTCCAAGACCCGAGAGCTGTGATGCACCGAGGGCTTTA
ATGTCCGTCACTCCAAATCTTTGTCATGACGAGACAAAGAACCGAGGAACATACACT
CGGGTGACA
