# Supplementary material for: Association of a 7.9 kb Endogenous Retrovirus Insertion in Intron 1 of CD36 with Obesity and Fat Measurements in Sheep
Source: Mob DNA. 2025 Mar 14;16:12. doi: 10.1186/s13100-025-00349-w (PMC11908002; doi:10.1186/s13100-025-00349-w)
Supplement: Supplementary file 4 — Additional File 4 Supplementary Figures: Fig. S1. Sheep breeds scrutinized in the present study; Barki (B), Rahmani (R), Rahmani x Barki cross (RB), Awassi (A), Ossimi (O) and Romanov x Rahmani (V). Fig. S2. A) Points of consideration; 1. the sterna (sternum region), 2. the ribs (the rib cage) and 3. the lumbar vertebrae region which involves the spinous and transverse processes of spine ''behind the ribs'' (a: spinous process, b: flank and c: transverse process) are palpated to determine BCS. B) Diagram ''cross-section'' for assigning BCS in small ruminants. Fig. S3. A) The Rib-eye area (longissimus dorsi) in sheep. B) The muscle longissimus thoracis et lumborum (LL) in sheep. Fig. S4. A) Design principles for full-length LTR polymorphic primers. 1&2) LTR-primer produces a shorter band 1,056 bp. 3&4) Side-primer produces a band of 1,159 bp. B) After PCR, a band of 1,056 bp with Primer (1 and 2) and no band of 1,159 bp with Primer (1 and 3) indicates ERV+/+. While a band of 1159 bp without the 1,056 bp band suggests ERV-/-, while both bands mean ERV+/-. Fig. S5. Analysis of CD36 gene and its genomic regions and nearby genes in the reference genomes of sheep (A), goats (B), cattle (C), buffaloes (D), pigs (E), rabbit (F), chicken(G), zebrafish (H) and domestic ferret (I). Fig. S6. PCR Verification for the selected Ov-ERV-R13-CD36 in 24 individuals (n=12♂ and 12♀) of Rahmani x Barki crossbred Breed, M: DNA Ladder 5 kbp [file 13100_2025_349_MOESM4_ESM.pdf]

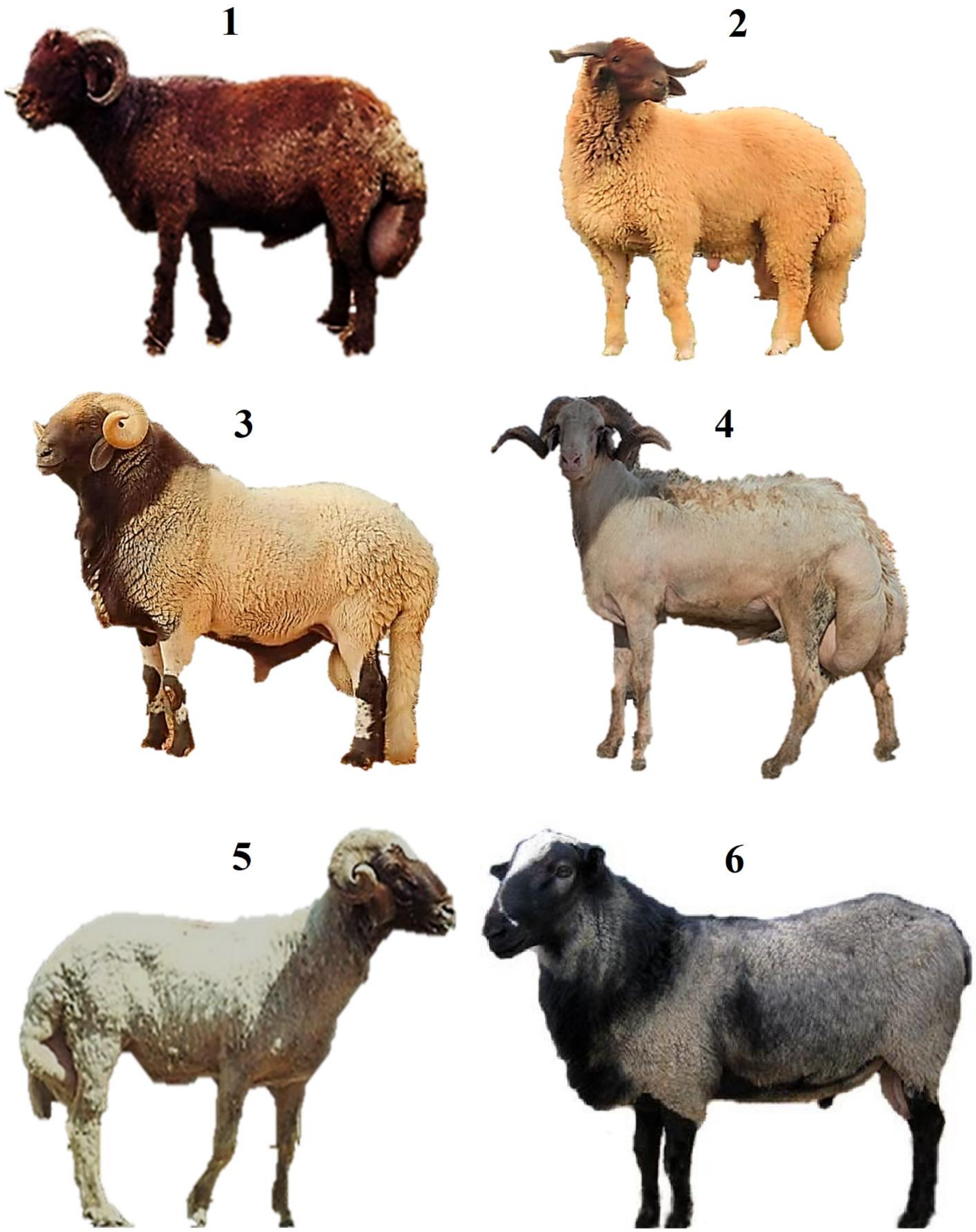

Fig. S1. Sheep breeds scrutinized in the present study; **1)** Rahmani, **2)** Barki, **3)** Rahmani *x* Barki cross, **4)** Ossimi, **5)** Awassi, and **6)** Romanov *x* Rahmani.

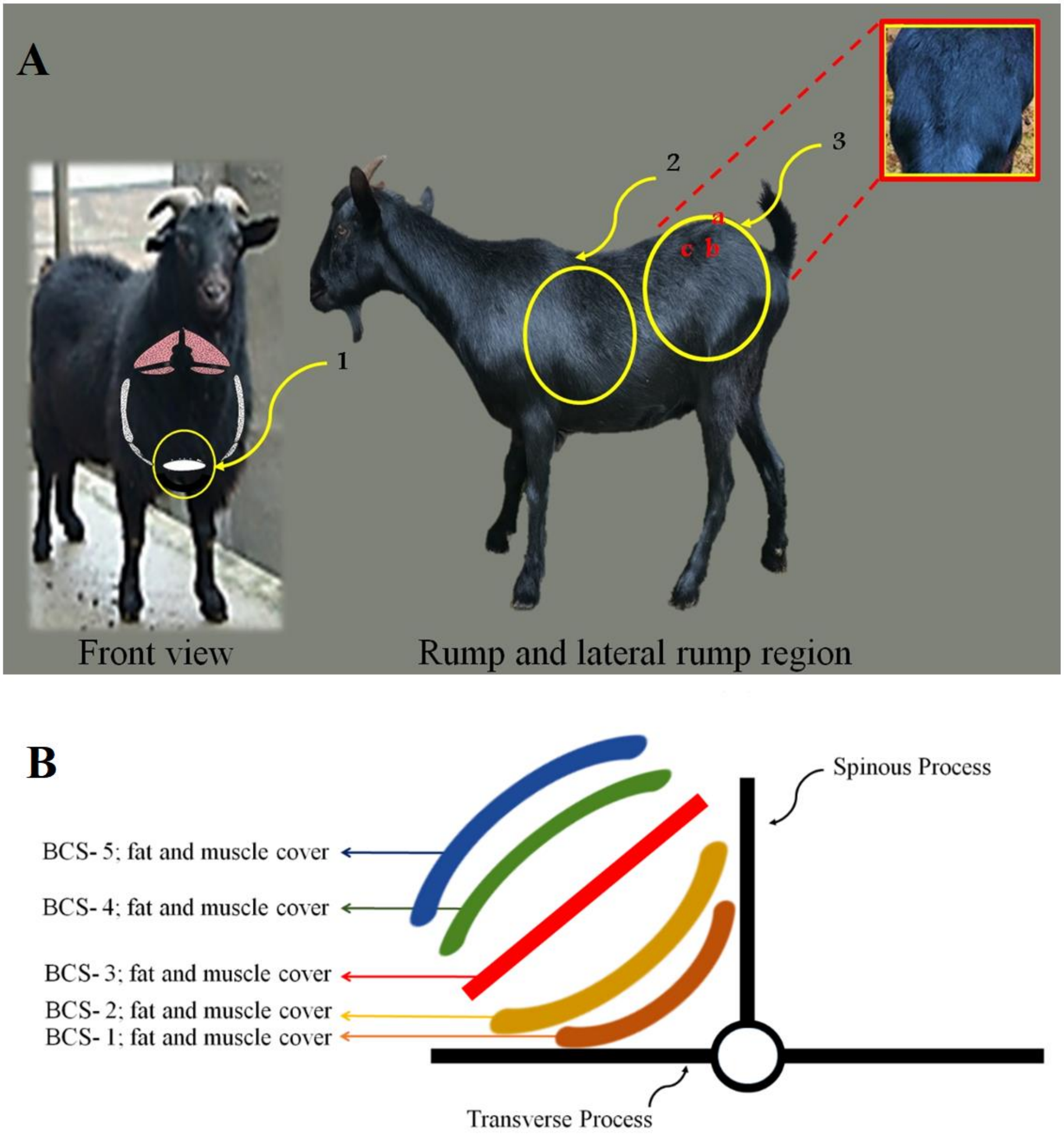

Fig. S2. A) Points of consideration; **1.** the sterna (sternum region), **2.** the ribs (the rib cage) and **3.** the lumbar vertebrae region which involves the spinous and transverse processes of spine "behind the ribs" (**a**: spinous process, **b**: flank and **c**: transverse process) are palpated to determine BCS. **B)** Diagram "cross-section" for assigning BCS in small ruminates.

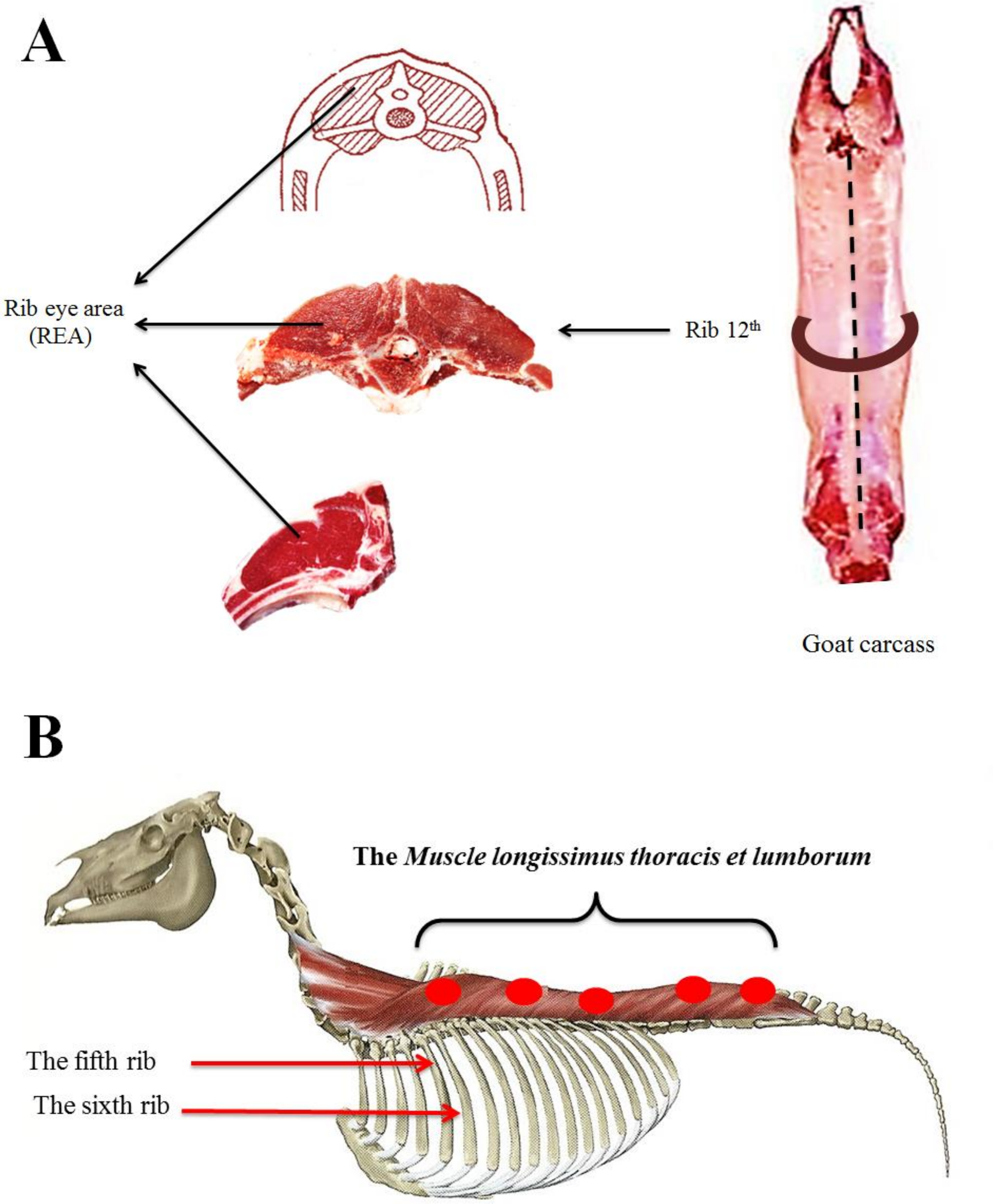

Fig. S3. A) The Rib-eye area (*longissimus dorsi*) in sheep. **B)** The muscle *longissimus thoracis et lumborum* (LL) in sheep.

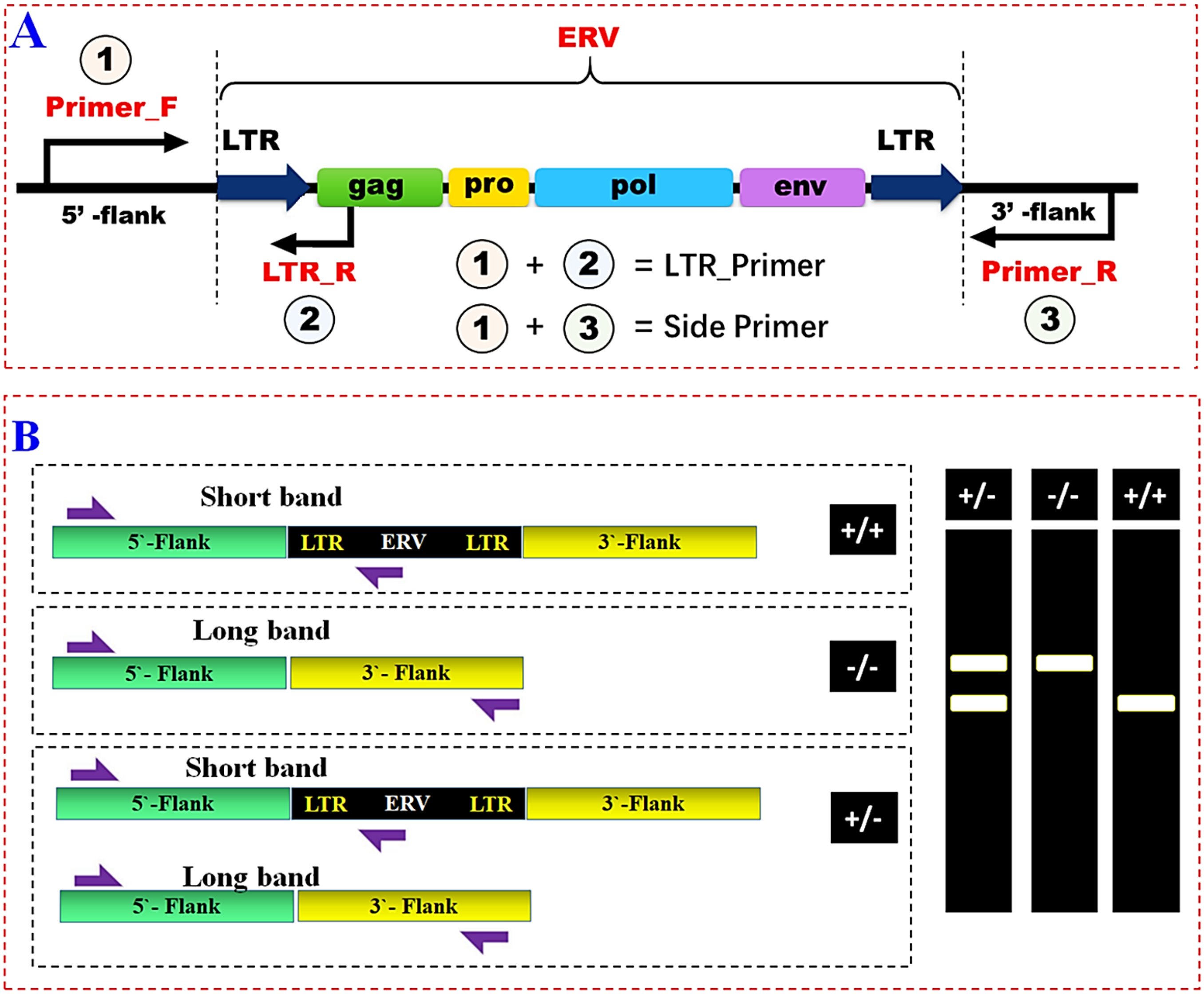

Fig. S4. **A)** Design principles for full-length LTR polymorphic primers. **1&2)** The LTR primer configuration produces a shorter PCR product of 1,056 bp, specifically designed to amplify a region that encompasses part of the gag gene, LTR and a segment of the flank in 3' to 5' direction, enhancing specificity for the full-length element. **3&4)** Side-primer produces a band of 1,159 bp. **B)** After PCR, a band of 1,056 bp with Primer (1 and 2) and no band of 1,159 bp with Primer (1 and 3) indicates ERV$^{+/+}$. While a band of 1,159 bp without the 1,056 bp band suggests ERV$^{-/-}$, while both bands mean ERV$^{+/-}$.

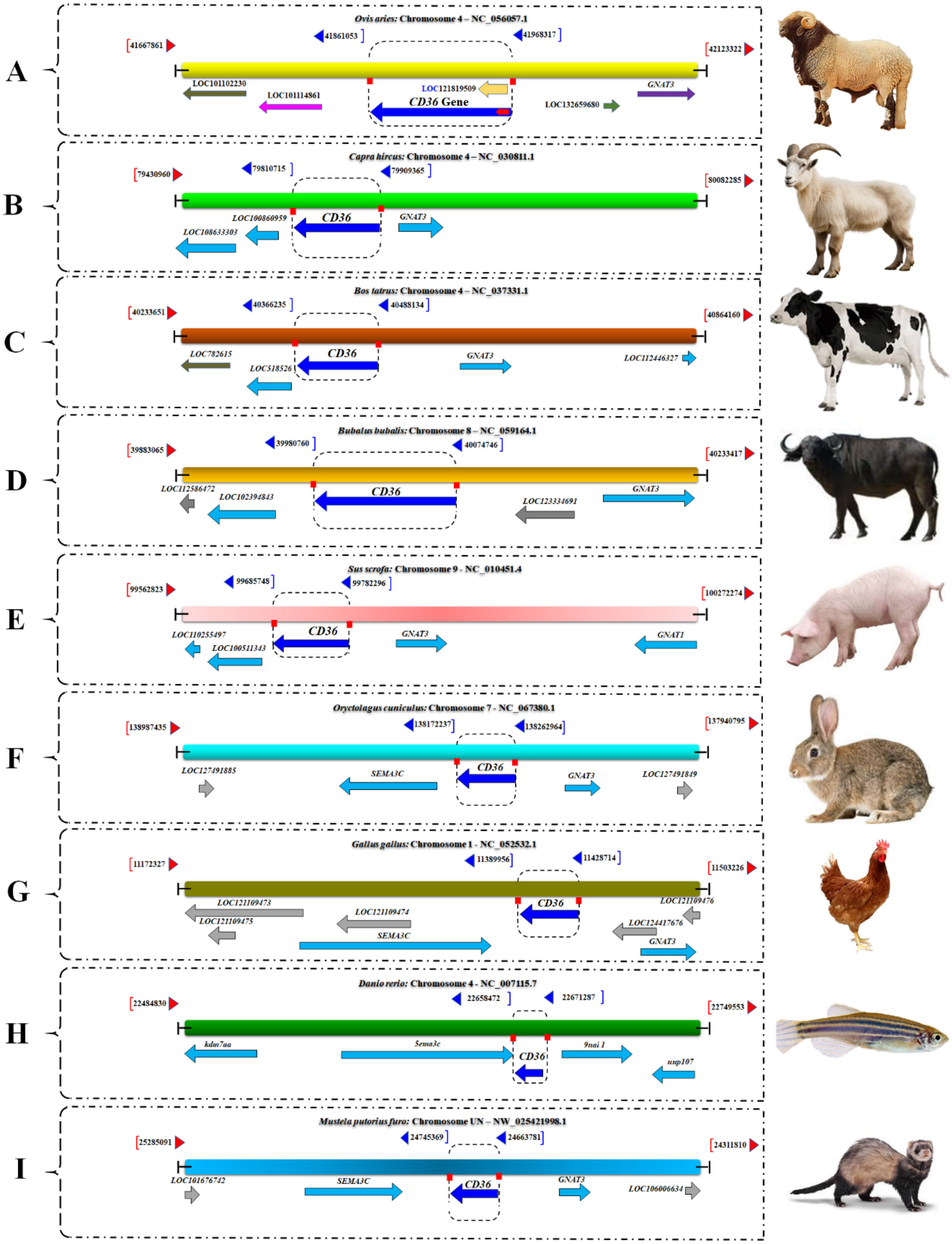

Fig. S5. Analysis of *CD36* gene and its genomic regions and nearby genes in the reference genomes of sheep (A), goats (B), cattle (C), buffaloes (D), pigs (E), rabbit (F), chicken(G), Zebrafish (H) and domestic ferret (I).

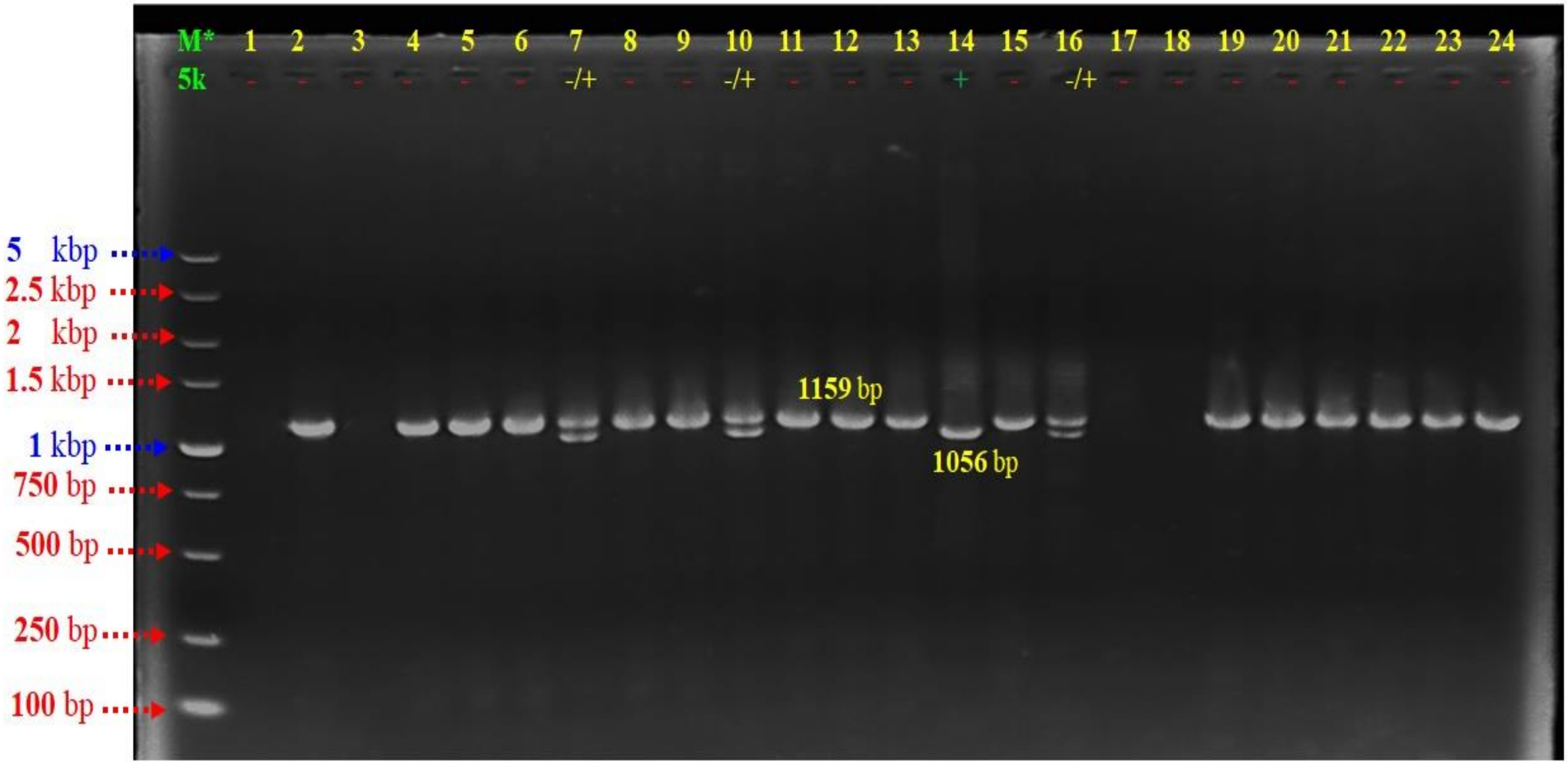

Fig. S6. PCR Verification for the selected Ov-ERV-R13-*CD36* in 24 individuals (n=12♂ and 12♀) of Rahmani *x* Barki crossbred Breed, M: DNA Ladder 5kbp.
